# Author Correction: Chiral phosphoric acid-catalyzed asymmetric epoxidation of alkenyl aza-heteroarenes using hydrogen peroxide

**DOI:** 10.1038/s41467-024-49937-z

**Published:** 2024-07-01

**Authors:** Hao-Chen Wen, Wei Chen, Meng Li, Chen Ma, Jian-Fei Wang, Aiping Fu, Shi-Qi Xu, Yi-Feng Zhou, Shao-Fei Ni, Bin Mao

**Affiliations:** 1 Collaborative Innovation Center of Yangtze River Delta Region Green Pharmaceuticals, Zhejiang University of Technology, Hangzhou, P.R. China; 2 Department of Chemistry and Key Laboratory for Preparation and Application of Ordered Structural Materials of Guangdong Province, Shantou University, Shantou, China; 3 College of Life Science, China Jiliang University, Hangzhou, P.R. China

**Keywords:** Asymmetric synthesis, Organocatalysis, Asymmetric catalysis

Correction to: *Nature Communications* 10.1038/s41467-024-49435-2, published online 20 June 2024

The original version of this Article contained an error in the CCDC number for compound 3ah in the Data availability statement, which incorrectly read ‘2165081 (3ah)’. The correct version states ‘2087973 (3ah)’ in place of ‘2165081 (3ah)’.

Furthermore, the original version of this Article omitted the Acknowledgements: Bin Mao acknowledges funding supported by Zhejiang University of Technology (KYY-HX-20220383).

This has been corrected in both the PDF and HTML versions of the Article.

