# Supplementary material for: Chiral phosphoric acid-catalyzed asymmetric epoxidation of alkenyl aza-heteroarenes using hydrogen peroxide
Source: Nat Commun. 2024 Jun 20;15:5277. doi: 10.1038/s41467-024-49435-2 (PMC11190242; doi:10.1038/s41467-024-49435-2)
Supplement: Supplementary file 1 — Supplementary Information [file 41467_2024_49435_MOESM1_ESM.pdf]

SUPPLEMENTARY INFORMATION

Chiral Phosphoric Acid-Catalyzed Asymmetric Epoxidation of Alkenyl Aza-Heteroarenes Using Hydrogen Peroxide

Hao-Chen Wen[1], Wei Chen[1], Meng Li[1], Chen Ma[2], Jian-Fei Wang[1], Ai-ping Fu[1], Shi-Qi Xu[1], Yi-Feng Zhou[3], Shao-Fei Ni[2*], Bin Mao[1,2*]

[1]*Collaborative Innovation Center of Yangtze River Delta Region Green Pharmaceuticals, Zhejiang University of Technology, Hangzhou 310014, P.R. China*

[2]*Department of Chemistry and Key Laboratory for Preparation and Application of Ordered Structural Materials of Guangdong Province, Shantou University, Shantou, Guangdong 515063, China*

[3]*College of Life Science, China Jiliang University, Hangzhou, Zhejiang 310018, P. R. China*

[*]**Corresponding author.** Email: sfni@stu.edu.cn (Shao-Fei Ni); maob@zjut.edu.cn (Bin Mao)

Supplementary Table of Contents

1. General Considerations....S3

2. Supplementary Methods....S4

2.1 Complete data for reaction optimizations....S4

2.2 Synthesis of alkenyl *N*-heteroarenes....S11

2.3. Enantioselective epoxidation of alkenyl aza-heteroarenes....S57

2.4. Scale-up reaction and synthetic applications of the chiral products....S125

3. Supplementary Discussion....S135

3.1 Mechanistic investigations....S135

3.2 Computational studies....S145

4. Supplementary Data....S147

4.1 X-Ray crystallographic data....S147

4.2. NMR spectroscopy for characterization....S153

5. Supplementary References....S351

1. General Considerations

■ ***Reactions & Reagents***

The majority of reactions were conducted in open air condition, with exceptions noted as applicable. Reagents, procured from commercial suppliers, were used directly without further purification, unless specified otherwise. Solvents such as toluene, tetrahydrofuran (THF), benzotrifluoride ($PhCF_3$), acetonitrile (MeCN), dichloromethane (DCM), ethyl acetate (EtOAc) were sourced from Energy Chemical (https://www.energy-chemical.com) and J&K Chemical (http://www.jkchemical.com) and were also used as received without additional purification. The chiral phosphoric acids (*R*)-**4j** and (*R*)-**4k** were synthesized according to documented literature procedures[1]. All other phosphoric acids were commercially obtained from Daicel Chiral Technologies (https://www.daicelchiraltech.cn).

■ ***Chromatography***

Analytical thin layer chromatography (TLC) was conducted on silica-coated glass plates (silicagel 60 F254, Huang Hai). Visualization was achieved under UV light at 254 nm or through staining with potassium permanganate ($KMnO_4$), cerium ammonium molybdenate (CAM), phosphomolybdic acid (PMA), iodine (I_2), or *p*-anisaldehyde. Flash column chromatography utilized silica gel 60 (Hai Yang, 200-300 mesh) and was performed without prior activation, except where noted.

■ ***Analytical instrumentation***

Melting points were determined using a Büchi B-540 capillary melting point apparatus. NMR spectroscopy including ^{1}H NMR, ^{13}C NMR, 2D NMR and ^{19}F NMR spectra were performed on Bruker 400 MHz or 600 MHz instruments. ^{13}C NMR spectra were broad band proton-decoupled. ^{1}H NMR Chemical shifts are reported in ppm relative to residual solvent signals ($CDCl_3$: 7.26 ppm; $(CD_3)_2SO$: 2.50 ppm). ^{13}C NMR chemical shifts are also in ppm relative to the solvent ($CDCl_3$: 77.2 ppm; $(CD_3)_2SO$: 39.5 ppm). Hexafluorobenzene (δ = –164.9 ppm) served as an external standard for ^{19}F NMR spectra. Coupling constants (J) are given in Hertz (Hz) and multiplicities are denoted as s (singlet), d (doublet), t (triplet), q (quartet), p (pentet), m (multiplet) or combinations thereof. High-resolution mass spectra (HRMS) were obtained on an Agilent 6210 TOF LC/MS using ESI (Electrospray Ionization) as the ion source. Optical rotations were measured with an AUTOPOL V automatic polarimeter. Enantioselectivities were determined by HPLC analysis on an Agilent 1100 system, using Daicel Chiralpak IA, IB, IC, ID, IE, IF, IG, IH, IJ, IM, OJ-H columns.

2. Supplementary Methods

2.1 Complete data for reaction optimizations

Comprehensive optimization studies for asymmetric epoxidation of tri-, tetra- and 1,1-disubstituted alkenyl aza-heteroarenes

Supplementary Table 1 Screening of chiral phosphoric acids[a]

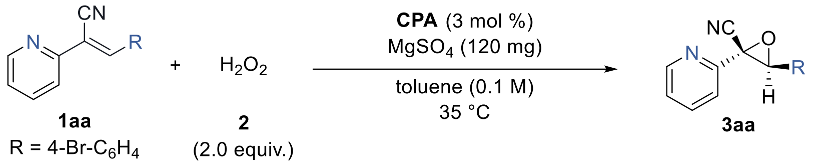

entry	CPA	yield (%)[b]	e.e. (%)[c]
1	(*S*)-**4a**	97	97
2	(*S*)-**4b**	77	93
3	(*S*)-**4c**	56	81
4	(*S*)-**4d**	89	95
5	(*S*)-**4e**	37	20
6	(*R*)-**4f**	57	67
7	(*R*)-**4g**	62	66

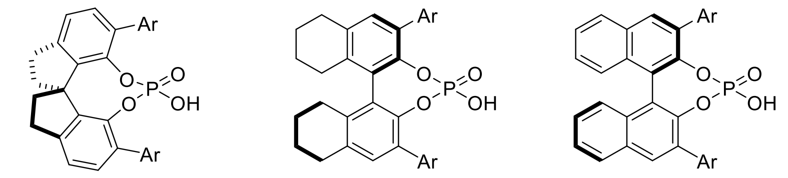

(*S*)-**4a**: Ar = 9-anthracenyl
(*S*)-**4b**: Ar = 1-napthyl
(*S*)-**4c**: Ar = 1-pyrenyl
(*S*)-**4d**: Ar = 2,4,6-$^{i}Pr_3C_6H_2$
(*S*)-**4e**: Ar = $SiPh_3$

(*R*)-**4f**: Ar = 9-anthracenyl

(*R*)-**4g**: Ar = 9-anthracenyl

[a]The standard reaction was executed with **1aa** (0.1 mmol) and **2** (0.2 mmol), in the presence of $MgSO_4$ (120 mg), and a chiral phosphoric acid (3 mol %). Reactions were conducted at 35 °C in toluene (1.0 mL), unless specified otherwise. [b]Isolated yields. [c]Enantiomeric excess (e.e.) values were ascertained through chiral HPLC analysis.

Supplementary Table 2 Screening of oxidants[a]

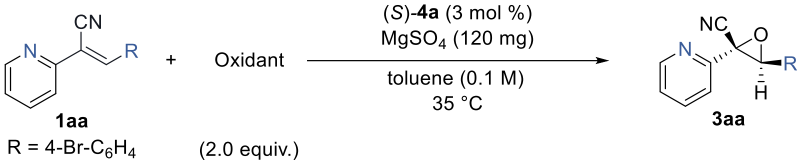

entry	oxidant	yield (%)[b]	e.e. (%)[c]
1	H_2O_2	97	97
2	TBHP	27	98
3	*m*-CPBA	81	97
4	CHP	16	97
5	DTBP	trace	95

[a]The standard reaction involved the use of **1aa** (0.1 mmol) and an oxidant (0.2 mmol), along with $MgSO_4$ (120 mg), and (*S*)-**4a** (3 mol %) as a catalyst. Reactions were conducted at 35 °C in toluene (1.0 mL), unless otherwise indicated. [b]Isolated yields. [c]Enantiomeric excess (e.e.) values were ascertained through chiral HPLC analysis. TBHP = *t*-butyl hydroperoxide; *m*-CPBA = 3-chloroperoxybenzoic acid; CHP = cumene hydroperoxide; DTBP = di-*t*-butyl peroxide.

Supplementary Table 3 Screening of additives[a]

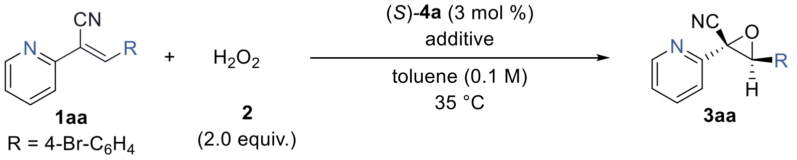

entry	additive	yield (%)[b]	e.e. (%)[c]
1	-	53	97
2	$MgSO_4$ (60 mg)	77	97
3	$MgSO_4$ (120 mg)	97	97
4	$NaSO_4$ (120 mg)	45	97
5	4 Å MS (120 mg)	trace	ND

[a]The standard reaction setup included **1aa** (0.1 mmol) and **2** (0.2 mmol), with the addition of various additives and (*S*)-**4a** (3 mol %) serving as a catalyst. Reactions were conducted at 35 °C in toluene (1.0 mL), except where noted differently. [b]Isolated yields. [c]Enantiomeric excess (e.e.) values were ascertained through chiral HPLC analysis. MS = molecular sieves; ND = not detected.

Supplementary Table 4 Screening of temperature[a]

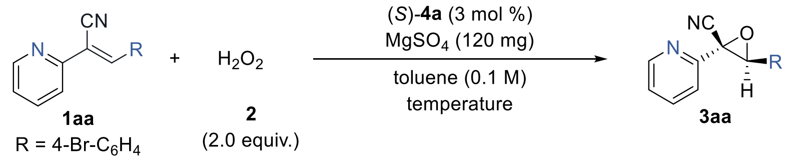

entry	temperature (°C)	yield (%)[b]	e.e. (%)[c]
1	−20	43	97
2	0	62	98
3	25	71	97
4	35	97	97
5	50	98	95

[a]The standard reaction involved the use of **1aa** (0.1 mmol) and **2** (0.2 mmol), along with $MgSO_4$ (120 mg), and (*S*)-**4a** (3 mol %) as a catalyst, in toluene (1.0 mL). Reactions were conducted at various temperatures, while maintaining all other conditions constant. [b]Isolated yields. [c]Enantiomeric excess (e.e.) values were determined using chiral HPLC analysis.

Supplementary Table 5 Screening of solvents and concentration adjustments[a]

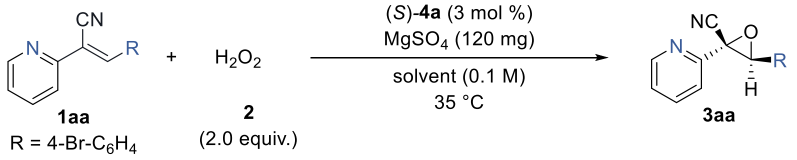

entry	solvent	yield (%)[b]	e.e. (%)[c]
1	$PhCF_3$	54	80
2	toluene	97	97
3	THF	33	82
4	EtOAc	39	92
5	CH_3CN	32	94
6[d]	CH_2Cl_2	97	97
7[e]	toluene	97	95

[a]In the standard reaction, **1aa** (0.1 mmol) and **2** (0.2 mmol) were used, along with $MgSO_4$ (120 mg), and (*S*)-**4a** (3 mol %) as a catalyst. Reactions were conducted at 35 °C for 5 h. Different solvents were tested while keeping all other conditions constant. [b]Isolated yields. [c]Enantiomeric excess (e.e.) values were determined using chiral HPLC analysis. [d]Extended reaction time of 18 h. [e]The reaction

concentration was adjusted to 0.2 M.

Supplementary Table 6 Screening of catalyst loadings[a]

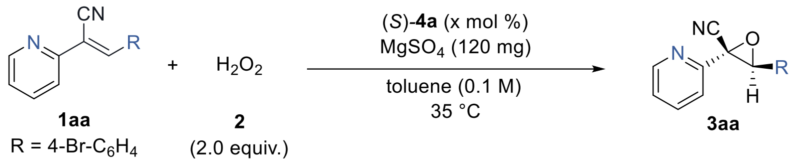

entry	catalyst loading (x mol %)	time (h)	yield (%)[b]	e.e. (%)[c]
1	10	2	95	99
2	5	3	95	98
3	3	5	93	97
4	1	12	62	80
5	0.5	18	54	65

[a]The standard reaction was carried out using **1aa** (0.1 mmol) and **2** (0.2 mmol), with $MgSO_4$ (120 mg), at 35 °C in toluene (1.0 mL) for a set duration. Various dosages of the catalyst (*S*)-**4a** were explored, while keeping all other reaction conditions constant. [b]Isolated products. [c]Enantiomeric excess (e.e.) values were determined using chiral HPLC analysis.

Comprehensive optimization studies for asymmetric epoxidation of mono-substituted and 1,2-disubstituted alkenyl azaarenes catalyzed by chiral phosphoric acid

Supplementary Table 7 Screening of chiral phosphoric acid catalysts[a]

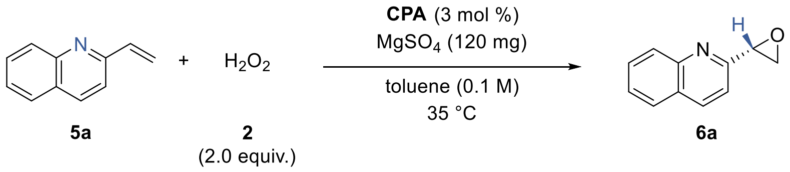

entry	CPA	yield (%)[b]	e.e. (%)[c]
1	(*S*)-**4a**	54	17
2	(*S*)-**4d**	75	69
3	(*R*)-**4h**	52	50
4	(*R*)-**4i**	43	55
5	(*R*)-**4j**	83	69
6	(*R*)-**4k**	75	62

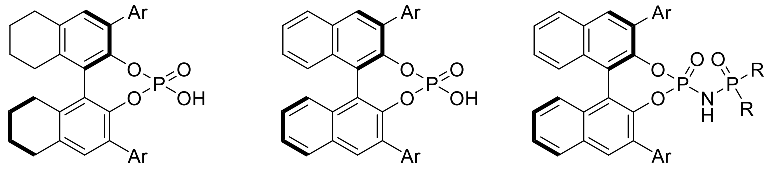

(*R*)-**4h**: Ar =2,4,6-$^{i}Pr_3C_6H_2$ (*R*)-**4i**: Ar =2,4,6-$^{i}Pr_3C_6H_2$ (*R*)-**4j**: Ar = 2,4,6-$^{i}Pr_3C_6H_2$ R = 4-CF_3-C_6H_4 (*R*)-**4k**: Ar = 2,4,6-$^{i}Pr_3C_6H_2$ R = C_6H_5

[a]The standard reaction was conducted with **5a** (0.1 mmol) and **2** (0.2 mmol), in the presence of $MgSO_4$ (120 mg), and a chiral phosphoric acid (3 mol %). Reactions were conducted at 35 °C in toluene (1.0 mL) for 72 h, unless specified otherwise. [b]Isolated yields. [c]Enantiomeric excess (e.e.) values were ascertained through chiral HPLC analysis.

Supplementary Table 8 Screening of additives and temperature[a]

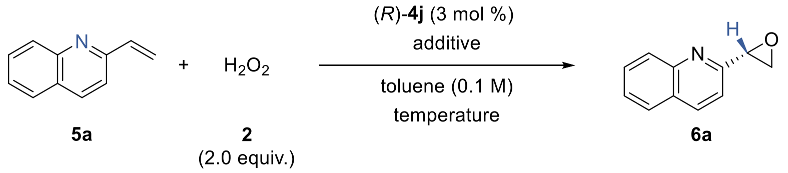

entry	additive	temperature (°C)	yield (%)[b]	e.e. (%)[c]
1	$MgSO_4$ (120 mg)	35	83	69
2	4 Å MS (120 mg)	35	trace	ND
3	-	35	81	77
4	-	25	81	87
5	-	0	52	85

[a]The standard reaction was conducted with **5a** (0.1 mmol) and **2** (0.2 mmol) with (*R*)-**4j** (3 mol %) in toluene (0.1 M), unless specified otherwise. [b]Isolated yields. [c]Enantiomeric excess (e.e.) values were ascertained through chiral HPLC analysis.

Supplementary Table 9 Screening of solvents and concentration adjustments[a]

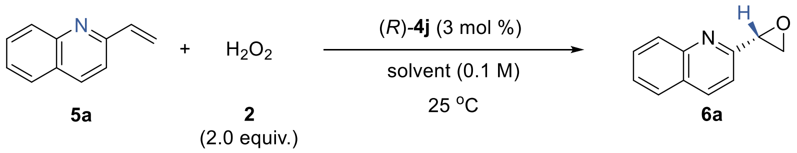

entry	solvent	yield (%)[b]	e.e. (%)[c]
1	$PhCF_3$	45	84
2	toluene	81	87
3	CH_2Cl_2	45	64
4	EtOAc	trace	ND
5	CH_3CN	trace	ND
6[d]	toluene	82	65

[a]Standard reaction conditions: conducted with **5a** (0.1 mmol) and **2** (0.2 mmol), employing (*R*)-**4j** (3 mol %) as the catalyst at 25 °C. Various solvents were tested under constant conditions. [b]Isolated yields. [c]Enantiomeric excess (e.e.) values were determined through chiral HPLC analysis. [d]The reaction concentration was adjusted to 0.2 M.

Supplementary Table 10 Screening of oxidants[a]

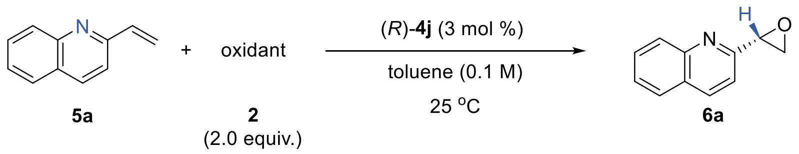

entry	oxidant	yield (%)[b]	e.e. (%)[c]
1	H_2O_2	81	87
2	TBHP	25	35
3	*m*-CPBA	trace	ND
4	CHP	45	17

[a]Standard reaction protocol: utilized **5a** (0.1 mmol) and various oxidants (0.2 mmol) with (*R*)-**4j** (3 mol %) as the catalyst. Reactions were carried out at 25 °C in toluene (0.1 M), except where specified otherwise. [b]Isolated yields. [c]Enantiomeric excess (e.e.) values were ascertained through chiral HPLC analysis. TBHP = *t*-butyl hydroperoxide; *m*-CPBA = 3-chloroperoxybenzoic acid; CHP = cumene hydroperoxide.

Supplementary Table 11 Screening of catalyst and hydrogen peroxide loadings[a]

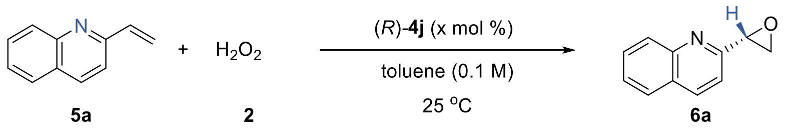

entry	catalyst loading (x mol %)	H_2O_2 (equiv.)	yield (%)[b]	e.e. (%)[c]
1	3	1.05	48	87
2	3	1.5	61	87
3	3	2.0	81	87
4	1	2.0	69	75
5	5	2.0	82	88

[a]Standard reaction conditions: executed with **5a** (0.1 mmol) at 25 °C in toluene (1.0 mL) for a predetermined duration. Various dosages of hydrogen peroxide **2** and the catalyst (*R*)-**4j** were tested, while all other reaction conditions were maintained constant. [b]Isolated yields. [c]Enantiomeric excess (e.e.) values were determined using chiral HPLC analysis.

2.2 Synthesis of alkenyl *N*-heteroarenes

2.2.1 General Procedures for the synthesis of trisubstituted alkenyl aza-heteroarenes

General Procedure A[2]:

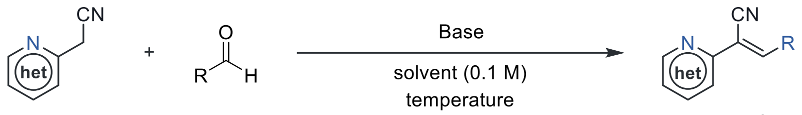

1aa-1gf
(R = alkyl, aryl)

In a round-bottom flask equipped with a magnetic stirrer, *N*-heterocyclic acetonitrile substrates (1.0 equiv.) and aldehyde (1.2 equiv.) were dissolved in methanol (0.1 M) or ethanol (0.1 M). A base (in stoichiometric or catalytic amount) was then added at room temperature. The reaction mixture was then stirred either at room temperature, heated to 50 °C, or brought to reflux, depending on the requirement for complete reaction of the starting materials. Upon completion, the formed precipitate was filtered and washed with cold methanol or ethanol, resulting in the desired product.

General Procedure B[3]:

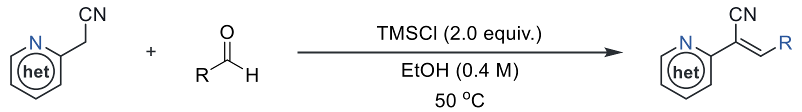

1aa-1gf
(R = alkyl, aryl)

In a reaction mixture, aldehyde (1.1 equiv.) and *N*-heterocyclic acetonitrile substrates (1.0 equiv.) were combined with TMSCl (2.0 equiv.) in ethanol (0.4 M). This solution was stirred at 50 °C for 3 hours. Following the reaction, the mixture was poured into deionized water (20.0 mL) to precipitate the product. The solid formed was then filtered and washed with cold isopropanol to yield the final product.

General Procedure C[4]:

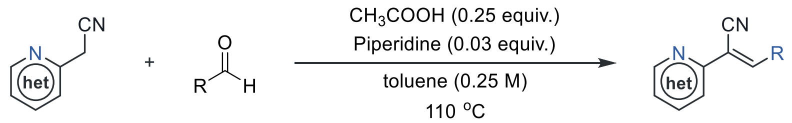

1aa-1gf
(R = alkyl, aryl)

In a flame-dried flask equipped with a magnetic stirring bar, a reaction mixture was prepared consisting of *N*-heterocyclic acetonitrile (1.0 equiv.), aldehyde (1.2 equiv.), acetic acid (0.25 equiv.), and piperidine (0.03 equiv.) in anhydrous toluene (0.25 M). This mixture was stirred at 110 °C until the reaction of the starting substrates was complete. Upon cooling to ambient temperature, the organic layer was washed with deionized water and subsequently extracted with ethyl acetate three times. The

combined organic phase was then dried over anhydrous Na_2SO_4. Afterward, the solvent was removed under reduced pressure. The resulting crude residue was purified using flash chromatography on silica gel to yield the desired product.

General Procedure D[5]:

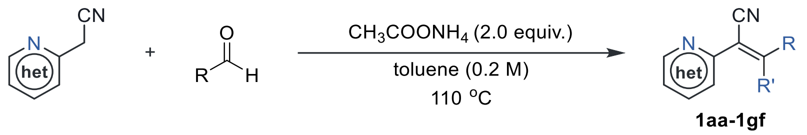

1aa-1gf
(R = alkyl, aryl)
(R' = H, alkyl)

In a flame-dried flask equipped with a magnetic stirring bar, a solution of *N*-heterocycle derived acetonitrile (1.0 equiv.) in anhydrous toluene (0.2 M) was prepared. To this solution, ammonium acetate (2.0 equiv.) and aldehydes or ketones (1.0 equiv.) were added. The reaction mixture was then heated under reflux conditions and monitored using thin-layer chromatography (TLC). Upon completion of the reaction, as indicated by TLC, the flask was cooled to ambient temperature. The mixture was subsequently washed with deionized water and extracted with ethyl acetate three times. The combined organic phase was dried over anhydrous Na_2SO_4, and the solvent was then removed under reduced pressure. The resulting crude residue underwent purification by flash chromatography on silica gel to yield the final product.

General Procedure E[6]:

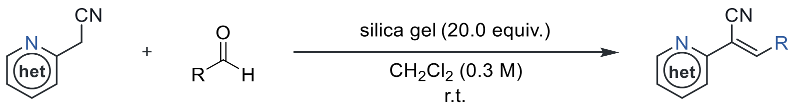

1aa-1gf
(R = alkyl, aryl)

In a flame-dried flask, *N*-heterocycle derived acetonitrile (1.0 equiv.) is dissolved in anhydrous dichloromethane. Subsequently, the corresponding aldehyde (1.2 equiv.) and silica gel (20.0 equiv.) are added to the solution. The reaction mixture is vigorously stirred at ambient temperature. The duration of the reaction varies based on the specific requirements of the substrate and the aldehyde but typically ranges from several hours to a few days. Upon completion, the reaction mixture is concentrated under reduced pressure. The crude product is then purified using flash column chromatography on silica gel to obtain the final product.

(*Z*)-3-(4-Bromophenyl)-2-(pyridin-2-yl)acrylonitrile (1aa)

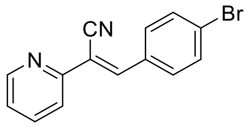

Compound **1aa** was synthesized following General Procedure A. Triethylamine (1.2 equiv.) was employed as the base, and ethanol was used as the solvent. The reaction proceeded at 50 °C, after which the product was purified by washing with cold ethanol to yield a white solid (0.56 g) in 83% yield.

^{1}H NMR (400 MHz, $CDCl_3$) δ 8.64 (ddd, *J* = 4.8, 1.8, 1.0 Hz, 1H), 8.44 (s, 1H), 7.86 (d, *J* = 8.5 Hz, 2H), 7.81 (td, *J* = 7.6, 1.7 Hz, 1H), 7.76 (dt, *J* = 7.9, 1.2 Hz, 1H), 7.61 (d, *J* = 8.6 Hz, 2H), 7.38 – 7.27 (m, 1H).

^{13}C NMR (100 MHz, $CDCl_3$) δ 150.8, 149.7, 143.9, 137.7, 132.4, 132.3, 131.4, 125.8, 123.9, 121.6, 117.7, 110.7.

Spectroscopic data are in accordance with the literature[7].

(*Z*)-3-(4-Bromophenyl)-2-(pyridin-4-yl)acrylonitrile (1ab)

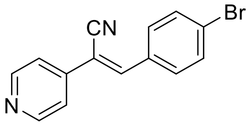

Compound **1ab** was synthesized following General Procedure A. Triethylamine (1.2 equiv.) was employed as the base, and ethanol was used as the solvent. The reaction proceeded at 50 °C, after which the product was purified by washing with cold ethanol to yield a white solid (0.60 g) in 76% yield.

^{1}H NMR (400 MHz, $CDCl_3$) δ 8.69 (d, *J* = 6.2 Hz, 2H), 7.79 (d, *J* = 8.5 Hz, 2H), 7.64 (s, 1H), 7.62 (d, *J* = 8.5 Hz, 2H), 7.54 (d, *J* = 6.3 Hz, 2H).

^{13}C NMR (100 MHz, $CDCl_3$) δ 150.3, 144.1, 142.1, 132.6, 131.7, 131.2, 126.5, 120.2, 116.7, 109.9.

Spectroscopic data are in accordance with the literature[8].

(*Z*)-3-(4-Bromophenyl)-2-(pyrazin-2-yl)acrylonitrile (1ac)

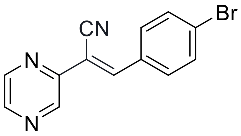

Compound **1ac** was synthesized following General Procedure A. Triethylamine (1.2 equiv.) was employed as the base, and ethanol was used as the solvent. The reaction proceeded at 50 °C, after which the product was purified by washing with cold ethanol to yield a white solid (0.96 g) in 67% yield.

m.p.: 155.5 – 158.2 °C

^{1}H NMR (600 MHz, $CDCl_3$) δ 9.02 (s, 1H), 8.59 (s, 2H), 8.39 (s, 1H), 7.86 (d, *J* = 8.5 Hz, 2H), 7.63 (d, *J* = 8.5 Hz, 2H).

^{13}C NMR (150 MHz, $CDCl_3$) δ 146.7, 145.5, 144.8, 144.2, 142.6, 132.6, 131.9, 131.6, 126.6, 116.6, 107.8.

HRMS (ESI) *m/z* calcd. for $C_{13}H_9BrN_3$ $[M+H]^+$: 285.9974; found: 285.9978.

(*E*)-3-(4-Bromophenyl)-2-(5-bromopyrimidin-2-yl)acrylonitrile (1ad)

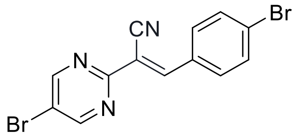

Compound **1ad** was synthesized following General Procedure A. Sodium methoxide (1.2 equiv.) was employed as the base, and ethanol was used as the solvent. The reaction proceeded at room temperature, after which the product was purified by washing with cold ethanol to yield a white solid (0.21 g) in 28% yield.

m.p.: 145.8 – 149.3 °C

^{1}H NMR (600 MHz, $CDCl_3$) δ 8.84 (s, 2H), 8.58 (s, 1H), 7.93 (d, *J* = 8.5 Hz, 1H), 7.64 (d, *J* = 8.5 Hz, 1H).

^{13}C NMR (150 MHz, $CDCl_3$) δ 159.4, 158.3, 148.2, 132.7, 132.0, 131.5, 127.1, 119.4, 116.7, 110.6.

HRMS (ESI) *m/z* calcd. for $C_{13}H_8Br_2N_3$ $[M+H]^+$: 363.9079; found: 363.9076.

(*Z*)-3-(4-Bromophenyl)-2-(6-chloropyridazin-3-yl)acrylonitrile (1ae)

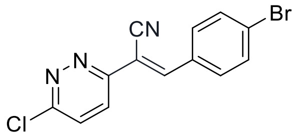

Compound **1ae** was synthesized following General Procedure A. Triethylamine (1.2 equiv.) was employed as the base, and methanol was used as the solvent. The reaction proceeded at room temperature, after which the product was purified by washing with cold methanol to yield a white solid (0.75 g) in 55% yield.

m.p.: 203.4 – 205.8 °C

^{1}H NMR (400 MHz, DMSO-d_6) δ 8.50 (s, 1H), 8.30 (d, *J* = 9.1 Hz, 1H), 8.07 (d, *J* = 9.1 Hz, 1H), 7.97 (d, *J* = 8.6 Hz, 2H), 7.81 (d, *J* = 8.6 Hz, 2H).

^{13}C NMR (100 MHz, DMSO-d_6) δ 156.0, 155.0, 147.0, 132.3, 132.0, 131.8, 129.6, 127.3, 125.6, 116.2, 108.4.

HRMS (ESI) *m/z* calcd. for $C_{13}H_7BrClN_3Na$ $[M+Na]^+$: 341.9404; found: 341.9403.

(*E*)-3-(4-Bromophenyl)-2-(4,6-dimethoxy-1,3,5-triazin-2-yl)acrylonitrile (1af)

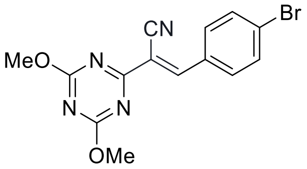

Compound **1af** was synthesized following General Procedure A. Triethylamine (1.2 equiv.) was employed as the base, and methanol was used as the solvent. The reaction proceeded at room temperature, after which the product was purified by washing with cold methanol to yield a white solid (0.14 g) in 74% yield.

m.p.: 175.7 – 187.5 °C

^{1}H NMR (400 MHz, $CDCl_3$) δ 8.69 (s, 1H), 7.95 (d, *J* = 7.7 Hz, 2H), 7.66 (d, *J* = 7.7 Hz, 2H), 4.13 (s, 6H).

^{13}C NMR (100 MHz, $CDCl_3$) δ 172.7, 171.6, 150.7, 132.8, 132.5, 131.0, 128.0, 115.9, 109.7, 55.8.

HRMS (ESI) *m/z* calcd. for $C_{14}H_{11}BrN_4O_2Na$ $[M+Na]^+$: 370.9938; found: 370.9938.

(*Z*)-3-(4-Bromophenyl)-2-(quinolin-2-yl)acrylonitrile (1ag)

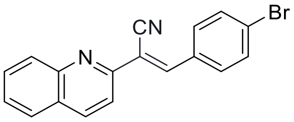

Compound **1ag** was synthesized in accordance with General Procedure B. The process resulted in the formation of a yellow solid (0.59 g) in 38% yield.

m.p.: 128.6 – 129.9 °C

^{1}H NMR (600 MHz, $CDCl_3$) δ 8.60 (s, 1H), 8.25 (d, *J* = 8.5 Hz, 1H), 8.12 (d, *J* = 8.5 Hz, 1H), 7.93 – 7.89 (m, 2H), 7.88 (d, *J* = 8.5 Hz, 1H), 7.83 (dd, *J* = 8.2, 1.4 Hz, 1H), 7.76 (ddd, *J* = 8.4, 6.8, 1.5 Hz, 1H), 7.64 – 7.60 (m, 2H), 7.57 (ddd, *J* = 8.0, 6.7, 1.1 Hz, 1H).

^{13}C NMR (150 MHz, $CDCl_3$) δ 150.6, 147.9, 145.3, 137.8, 132.5, 132.4, 131.6, 130.7, 129.6, 128.0, 127.8, 127.4, 126.0, 119.0, 117.9, 111.2.

HRMS (ESI) *m/z* calcd. for $C_{18}H_{12}BrN_2$ $[M+H]^+$: 335.0178; found: 335.0183.

(*Z*)-3-(3-Bromophenyl)-2-(isoquinolin-1-yl)acrylonitrile (1ah)

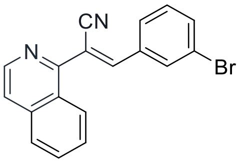

Compound **1ah** was synthesized in accordance with General Procedure B. The process resulted in the formation of a yellow solid (0.32 g) in 38% yield.

m.p.: 186.4 – 187.5 °C

^{1}H NMR (400 MHz, $CDCl_3$) δ 8.59 (d, *J* = 5.6 Hz, 1H), 8.39 (dd, *J* = 8.5, 1.1 Hz, 1H), 8.08 – 7.99 (m, 2H), 7.92 (d, *J* = 7.9 Hz, 1H), 7.81 – 7.64 (m, 4H), 7.63 (ddd, *J* = 8.0, 1.9, 1.0 Hz, 1H), 7.40 (t, *J* = 7.9 Hz, 1H).

^{13}C NMR (100 MHz, $CDCl_3$) δ 153.3, 148.7, 142.3, 137.2, 135.4, 134.2, 132.8, 130.9, 130.7, 128.4, 127.9, 127.7, 126.2, 125.5, 123.2, 122.1, 117.9, 111.5.

HRMS (ESI) *m/z* calcd. for $C_{18}H_{12}BrN_2$ $[M+H]^+$: 335.0178; found: 335.0189.

(*Z*)-3-(4-Bromophenyl)-2-(quinoxalin-2-yl)acrylonitrile (1ai)

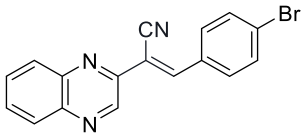

Compound **1ai** was synthesized following General Procedure A. Triethylamine (1.2 equiv.) was employed as the base, and methanol was used as the solvent. The reaction proceeded at room temperature, after which the product was purified by washing with cold methanol to yield a yellow solid (1.35 g) in 85% yield.

m.p.: 124.1 – 126.5 °C

^{1}H NMR (600 MHz, $CDCl_3$) δ 9.31 (s, 1H), 8.56 (s, 1H), 8.23 – 8.07 (m, 2H), 7.95 (d, *J* = 8.5 Hz, 2H), 7.90 – 7.75 (m, 2H), 7.67 (d, *J* = 8.5 Hz, 2H).

^{13}C NMR (150 MHz, $CDCl_3$) δ 146.6, 145.7, 142.8, 142.6, 141.8, 132.7, 132.0, 131.8, 131.3, 130.8, 129.6, 129.6, 126.8, 117.0, 108.5.

HRMS (ESI) *m/z* calcd. for $C_{17}H_{11}BrN_3$ $[M+H]^+$: 336.0131; found: 336.0133.

(*Z*)-3-(4-Bromophenyl)-2-(6,7-dimethoxyquinazolin-4-yl)acrylonitrile (1aj)

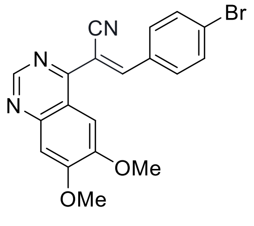

Compound **1aj** was synthesized following General Procedure A. Triethylamine (1.2 equiv.) was employed as the base, and methanol was used as the solvent. The reaction proceeded at room temperature, after which the product was purified by washing with cold methanol to yield a yellow solid (0.15 g) in 60% yield.

m.p.: 203.4 – 228.7 °C

^{1}H NMR (400 MHz, $CDCl_3$) δ 9.15 (s, 1H), 8.16 (s, 1H), 7.92 (d, J = 8.1 Hz, 2H), 7.75 (s, 1H), 7.67 (d, J = 8.1 Hz, 2H), 7.43 (s, 1H), 4.10 (s, 3H), 4.07 (s, 3H).

^{13}C NMR (100 MHz, $CDCl_3$) δ 157.1, 156.8, 152.9, 151.6, 151.2, 149.5, 132.7, 131.8, 131.7, 127.1, 118.2, 117.9, 108.5, 107.0, 102.1, 56.8, 56.6.

HRMS (ESI) *m/z* calcd. for $C_{19}H_{15}BrN_3O_2$ $[M+H]^+$: 396.0342; found: 396.0363.

(*Z*)-3-(4-Bromophenyl)-2-(7-((2-(trimethylsilyl)ethoxy)methyl)-7H-pyrrolo[2,3-*d*]pyrimidin-4-yl)acrylonitrile (1ak)

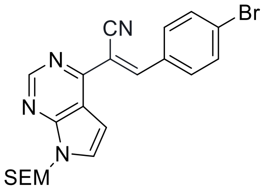

Compound **1ak** was synthesized following General Procedure A. Triethylamine (1.2 equiv.) was employed as the base, and methanol was used as the solvent. The reaction proceeded at room temperature, after which the product was purified by washing with cold methanol to yield a yellow solid (0.54 g) in 69% yield.

m.p.: 100.0 – 101.1 °C

^{1}H NMR (400 MHz, $CDCl_3$) δ 8.89 (s, 1H), 8.69 (s, 1H), 7.93 (d, J = 8.6 Hz, 2H), 7.65 (d, J = 8.6 Hz, 2H), 7.47 (d, J = 3.8 Hz, 1H), 7.33 (d, J = 3.8 Hz, 1H), 5.69 (s, 2H), 3.59 – 3.51 (m, 2H), 1.01 – 0.82 (m, 2H), –0.05 (s, 9H).

^{13}C NMR (100 MHz, $CDCl_3$) δ 153.2, 150.9, 149.7, 148.4, 132.6, 131.9, 131.9, 129.8, 126.8, 118.3, 115.8, 108.8, 101.4, 73.1, 66.9, 17.8, -1.3.

HRMS (ESI) *m/z* calcd. for $C_{21}H_{24}BrN_4O_2Si$ $[M+H]^+$: 455.0897; found: 455.0905.

(*E*)-3-(4-Bromophenyl)-2-(1-tosyl-1H-benzo[*d*]imidazol-2-yl)acrylonitrile (1al)

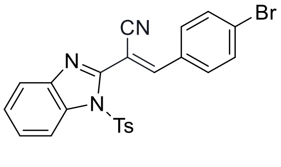

Compound **1al** was synthesized in accordance with General Procedure B. The process resulted in the formation of a yellow solid (0.77 g) in 35% yield.

m.p.: 139.3 – 142.7 °C

^{1}H NMR (400 MHz, $CDCl_3$) δ 8.03 (d, *J* = 7.5 Hz, 1H), 7.86 (d, *J* = 8.5 Hz, 2H), 7.81 (d, *J* = 8.5 Hz, 2H), 7.75 (d, *J* = 7.5 Hz, 1H), 7.67 (d, *J* = 8.5 Hz, 2H), 7.62 (s, 1H), 7.44 (dtd, *J* = 21.1, 7.4, 1.3 Hz, 2H), 7.27 (d, *J* = 7.5 Hz, 2H), 2.38 (s, 3H).

^{13}C NMR (100 MHz, $CDCl_3$) δ 151.2, 147.4, 146.7, 142.3, 134.5, 133.5, 132.7, 131.5, 131.3, 130.4, 127.4, 127.1, 126.6, 125.9, 121.1, 115.9, 114.7, 102.0, 21.9.

HRMS (ESI) *m/z* calcd. for $C_{23}H_{16}BrN_3O_2S$ $[M+H]^+$: 478.0219; found: 478.0228.

(*E*)-2-(Benzo[*d*]oxazol-2-yl)-3-(4-bromophenyl)acrylonitrile (1am)

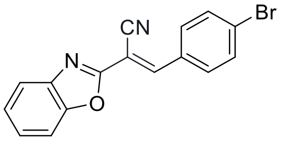

Compound **1am** was synthesized in accordance with General Procedure B. The process resulted in the formation of a yellow solid (1.30 g) in 88% yield.

m.p.: 137.1 – 139.0 °C

^{1}H NMR (600 MHz, $CDCl_3$) δ 8.25 (s, 1H), 7.92 (d, *J* = 8.5 Hz, 2H), 7.83 – 7.78 (m, 1H), 7.67 (d, *J* = 8.5 Hz, 2H), 7.61 - 7.56 (m, 1H), 7.47 - 7.37 (m, 2H).

^{13}C NMR (150 MHz, $CDCl_3$) δ 158.8, 150.9, 147.5, 141.7, 132.9, 131.9, 131.1, 127.7, 126.5, 125.5, 120.9, 114.9, 110.9, 100.2.

HRMS (ESI) *m/z* calcd. for $C_{16}H_{10}BrN_2O$ $[M+H]^+$: 324.9971; found: 324.9976.

(*E*)-2-(Benzo[*d*]thiazol-2-yl)-3-(4-bromophenyl)acrylonitrile (1an)

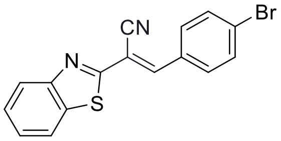

Compound **1an** was synthesized following General Procedure A. Triethylamine (1.2 equiv.) was employed as the base, and methanol was used as the solvent. The reaction proceeded at room temperature, after which the product was purified by washing with cold methanol to yield a yellow solid (0.84 g) in 98% yield.

m.p.: 115.6 – 125.4 °C

^{1}H NMR (400 MHz, DMSO-*d*$_6$) δ 8.40 (s, 1H), 8.18 (ddd, *J* = 8.0, 1.4, 0.6 Hz, 1H), 8.09 (ddd, *J* = 8.2, 1.3, 0.6 Hz, 1H), 8.05 – 7.98 (m, 2H), 7.84 – 7.79 (m, 2H), 7.59 (ddd, *J* = 8.1, 7.2, 1.4 Hz, 1H), 7.52 (ddd, *J* = 8.4, 7.2, 1.3 Hz, 1H).

^{13}C NMR (100 MHz, $CDCl_3$) δ 162.5, 153.7, 145.5, 135.2, 132.8, 131.7, 131.4, 127.2, 127.1, 126.3, 123.8, 121.9, 116.4, 106.3.

HRMS (ESI) *m/z* calcd. for $C_{16}H_{10}BrN_2S$ $[M+H]^+$: 340.9743; found: 340.9766.

(*E*)-3-(4-Bromophenyl)-2-(4-(4-fluorophenyl)thiazol-2-yl)acrylonitrile (1ao)

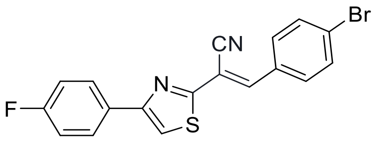

Compound **1ao** was synthesized in accordance with General Procedure B. The process resulted in the formation of a yellow solid (0.80 g) in 51% yield.

m.p.: 153.7 – 158.0 °C

^{1}H NMR (600 MHz, $CDCl_3$) δ 8.19 (s, 1H), 7.94 (dd, *J* = 8.6, 5.4 Hz, 2H), 7.85 (d, *J* = 8.5 Hz, 2H), 7.64 (d, *J* = 8.5 Hz, 2H), 7.51 (s, 1H), 7.14 (t, *J* = 8.6 Hz, 2H).

^{13}C NMR (150 MHz, $CDCl_3$) δ 164.0, 162.4, 162.3, 156.2, 143.0, 132.7, 131.6, 131.4, 130.1 (d, *J* = 3.3 Hz), 128.5 (d, *J* = 8.2 Hz), 126.5, 116.7, 116.1, 115.9, 114.1, 105.5.

^{19}F NMR (565 MHz, $CDCl_3$) δ −115.8.

HRMS (ESI) *m/z* calcd. for $C_{18}H_{11}BrFN_2S$ $[M+H]^+$: 384.9805; found: 384.9813.

(*E*)-3-(4-Bromophenyl)-2-(1-phenyl-1H-imidazol-2-yl)acrylonitrile (1ap)

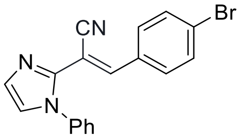

Compound **1ap** was synthesized following General Procedure A. Piperidine (0.2 equiv.) was employed as the base, and ethanol was used as the solvent. The reaction proceeded at room temperature, after which the product was purified by washing with cold methanol to yield a yellow solid (0.50 g) in 57% yield.

m.p.: 101.2 – 104.0 °C

^{1}H NMR (600 MHz, $CDCl_3$) δ 7.73 (s, 1H), 7.65 (d, *J* = 8.6 Hz, 2H), 7.54 (d, *J* = 8.6 Hz, 2H), 7.54 – 7.52 (m, 3H), 7.41 – 7.36 (m, 2H), 7.27 - 7.24 (m, 1H), 7.19 (d, *J* = 1.2 Hz, 1H).

^{13}C NMR (150 MHz, $CDCl_3$) δ 146.4, 141.4, 137.1, 132.4, 131.9, 131.1, 130.0, 129.7, 126.3, 125.9, 124.2, 115.4, 101.5.

HRMS (ESI) *m/z* calcd. for $C_{18}H_{13}BrN_3$ $[M+H]^+$: 350.0287; found: 350.0287.

(*E*)-3-(4-Bromophenyl)-2-(5-phenyloxazol-2-yl)acrylonitrile (1aq)

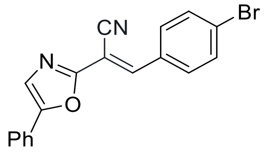

Compound **1aq** was synthesized following General Procedure A. Triethylamine (1.2 equiv.) was employed as the base, and methanol was used as the solvent. The reaction proceeded at room temperature, after which the product was purified by washing with cold methanol to yield a yellow solid (0.92 g) in 73% yield.

m.p.: 176.3 – 177.2 °C

^{1}H NMR (400 MHz, $CDCl_3$) δ 7.99 (s, 1H), 7.85 (d, *J* = 8.0 Hz, 2H), 7.69 (d, *J* = 7.5 Hz, 2H), 7.63 (d, *J* = 8.0 Hz, 2H), 7.50 – 7.41 (m, 3H), 7.41 – 7.34 (m, 1H).

^{13}C NMR (100 MHz, $CDCl_3$) δ 156.7, 152.8, 144.2, 132.7, 131.4, 131.3, 129.4, 129.2, 127.1, 126.9, 124.6, 124.2, 114.9, 99.9.

HRMS (ESI) *m/z* calcd. for $C_{18}H_{12}BrN_2O$ $[M+H]^+$: 351.0128; found: 351.0138.

(*E*)-3-(4-Bromophenyl)-2-(5-phenyloxazol-2-yl)acrylonitrile (1ar)

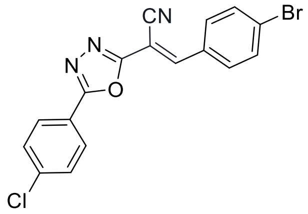

Compound **1ar** was synthesized following General Procedure A. Triethylamine (1.2 equiv.) was employed as the base, and methanol was used as the solvent. The reaction proceeded at room temperature, after which the product was purified by washing with cold methanol to yield a yellow solid (0.92 g) in 73% yield.

m.p.: 240.2 – 246.7 °C

^{1}H NMR (400 MHz, $CDCl_3$) δ 8.19 (s, 1H), 8.07 (d, *J* = 8.5 Hz, 2H), 7.90 (d, *J* = 8.5 Hz, 2H), 7.69 (d, *J* = 7.7 Hz, 2H), 7.53 (d, *J* = 7.7 Hz, 2H).

^{13}C NMR (100 MHz, $CDCl_3$) δ 164.7, 161.1, 148.0, 139.0, 133.0, 131.9, 130.7, 129.8, 128.6, 128.3, 121.7, 114.0, 96.3.

HRMS (ESI) *m/z* calcd. for $C_{17}H_{10}BrClN_3O$ $[M+H]^+$:385.9696 ; found: 385.9690.

(*E*)-3-(4-Bromophenyl)-2-(4,4-dimethyl-4,5-dihydrooxazol-2-yl)acrylonitrile (1as)

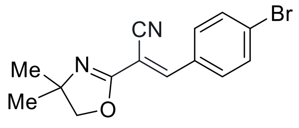

Compound **1as** was synthesized following General Procedure A. Triethylamine (1.2 equiv.) was employed as the base, and methanol was used as the solvent. The reaction proceeded at room temperature, after which the product was purified by washing with cold methanol to yield a white solid (0.30 g) in 90% yield.

m.p.: 84.2 – 86.6 °C

^{1}H NMR (400 MHz, DMSO-d_6) δ 8.04 (s, 1H), 7.90 (d, *J* = 8.5 Hz, 2H), 7.77 (d, *J* = 8.5 Hz, 2H), 4.14 (s, 2H), 1.28 (s, 6H).

^{13}C NMR (100 MHz, $CDCl_3$) δ 158.4, 148.6, 132.6, 131.7, 131.1, 127.2, 115.4, 101.3, 80.1, 68.6, 28.3.

HRMS (ESI) *m/z* calcd. for $C_{14}H_{14}BrN_2$ $[M+H]^+$: 305.0284; found: 305.0292.

(*E*)-2-(Benzo[*d*]thiazol-2-yl)-3-phenylacrylonitrile (1ba)

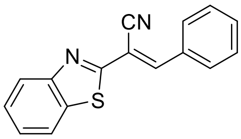

Compound **1ba** was synthesized following General Procedure A. Triethylamine (1.2 equiv.) was employed as the base, and ethanol was used as the solvent. The reaction proceeded at 50 °C, after which the product was purified by washing with cold ethanol to yield a white solid (0.38 g) in 45% yield.

^{1}H NMR (400 MHz, $CDCl_3$) δ 8.23 (s, 1H), 8.11 – 8.06 (m, 1H), 8.05 – 7.98 (m, 2H), 7.90 (dd, *J* = 8.0, 1.2 Hz, 1H), 7.59 – 7.47 (m, 4H), 7.43 (td, *J* = 7.7, 7.2, 1.2 Hz, 1H).

^{13}C NMR (100 MHz, $CDCl_3$) δ 162.9, 153.7, 147.1, 135.1, 132.5, 132.4, 130.5, 129.4, 127.1, 126.2, 123.7, 121.8, 116.6, 105.7.

Spectroscopic data are in accordance with the literature[9].

(*Z*)-2-(Pyridin-2-yl)-3-(4-((trifluoromethyl)thio)phenyl)acrylonitrile (1bb)

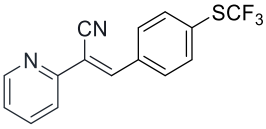

Compound **1bb** was synthesized following General Procedure A. Triethylamine (1.2 equiv.) was employed as the base, and methanol was used as the solvent. The reaction proceeded at 50 °C, after which the product was purified by washing with cold methanol to yield a yellow solid (0.43 g) in 28% yield.

m.p.: 149.8 − 150.0 °C

^{1}H NMR (400 MHz, $CDCl_3$) δ 8.65 (d, *J* = 4.6 Hz, 1H), 8.49 (s, 1H), 8.00 (d, *J* = 7.9 Hz, 2H), 7.79 (dt, *J* = 19.9, 8.1 Hz, 4H), 7.32 (t, *J* = 5.9 Hz, 1H).

^{13}C NMR (100 MHz, $CDCl_3$) δ 150.5, 149.9, 143.3, 137.7, 136.4, 135.6, 130.7, 129.5 (q, *J* = 308.6 Hz), 127.4, 124.1, 121.8, 117.4, 112.4.

^{19}F NMR (376 MHz, $CDCl_3$) δ −45.1.

HRMS (ESI) *m/z* calcd. for $C_{15}H_9F_3N_2NaS$ $[M+Na]^+$: 329.0331; found: 329.0329.

(*E*/*Z*)-3-(4-(Methylsulfonyl)phenyl)-2-(2-(methylthio)pyrimidin-4-yl)acrylonitrile (1bc)

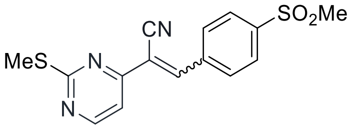

Compound **1bc** was synthesized following General Procedure A. Triethylamine (1.2 equiv.) was employed as the base, and methanol was used as the solvent. The reaction proceeded at 50 °C, after which the product was purified by flash column chromatography on silica gel (petroleum ether/EtOAc = 20/1) to yield a white solid (1.20 g) in 74% yield. Compound **1bc** was obtained as an inseparable *Z*/*E* isomer mixture, with a ratio of 6:1.

m.p.: 146.5 – 153.0 °C

^{1}H NMR (400 MHz, $CDCl_3$) δ 8.69 (*major*, s, 1H),8.66 (*major*, d, *J* = 5.2 Hz, 1H), 8.42 (*minor*, d, *J* = 5.0 Hz, 1H), 8.14 (*major*, d, *J* = 8.7 Hz, 2H), 8.07 (*major*, d, *J* = 8.7 Hz, 2H), 7.85 (*minor*, d, *J* = 8.0 Hz, 2H), 7.44 (*minor*, d, *J* = 8.0 Hz, 2H), 7.37 (*major*, d, *J* = 4.8 Hz, 1H), 6.93 (*minor*, d, *J* = 5.0 Hz, 1H), 6.05 (*minor*, s, 1H), 3.10 (*major*, s, 3H), 3.01 (*minor*, d, *J* = 2.0 Hz, 3H), 2.63 (*major*, s, 3H), 2.60 (*minor*, d, *J* = 1.9 Hz, 3H).

^{13}C NMR (100 MHz, $CDCl_3$, *carbons from the regioisomer are indicated in parentheses*) δ 173.4 (174.1), 159.2 (160.5), 157.1 (156.4), 145.6 (149.1), 143.0 (141.4), 137.5 (137.9), 131.0 (130.6), 128.3 (127.9), 116.0 (115.5), 113.3, 112.1, 44.5, 14.4.

HRMS (ESI) *m/z* calcd. for $C_{15}H_{14}N_2O_2S_2$ $[M+H]^+$: 332.0522; found: 332.0530.

(*E*)-2-(4,6-Dimethoxy-1,3,5-triazin-2-yl)-3-(4-(methoxymethoxy)phenyl)acrylonitrile (1bd)

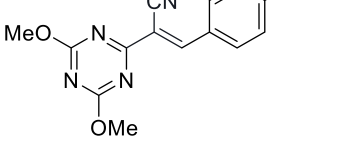

Compound **1bd** was synthesized following General Procedure A. Piperidine (0.2 equiv.) was employed as the base, and ethanol was used as the solvent. The reaction proceeded at 78 °C, after

which the product was purified by recrystallization from ethanol to yield a yellow solid (0.35 g) in 43% yield.

m.p.: 131.1 − 133.2 °C

^{1}H NMR (400 MHz, $CDCl_3$) δ 8.69 (s, 1H), 8.08 (d, *J* = 8.8 Hz, 2H), 7.15 (d, *J* = 8.9 Hz, 2H), 5.26 (s, 2H), 4.12 (s, 6H), 3.49 (s, 3H).

^{13}C NMR (100 MHz, $CDCl_3$) δ 172.7, 172.3, 161.3, 151.6, 133.7, 126.1, 116.9, 116.6, 106.3, 94.3, 56.5, 55.7.

HRMS (ESI) *m/z* calcd. for $C_{16}H_{17}N_4O_4$ $[M+H]^+$: 329.1244; found: 329.1242.

(*E*)-4-(2-Cyano-2-(4,6-dimethoxy-1,3,5-triazin-2-yl)vinyl)phenyl acetate (1be)

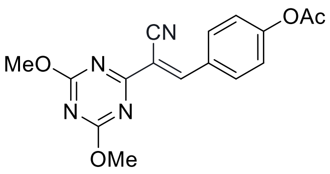

Compound **1be** was synthesized following General Procedure A. Piperidine (0.2 equiv.) was employed as the base, and ethanol was used as the solvent. The reaction proceeded at 78 °C, after which the product was purified by recrystallization from ethanol to yield a yellow solid (0.42 g) in 52% yield.

m.p.: 131.1 – 133.2 °C

^{1}H NMR (400 MHz, $CDCl_3$) δ 8.71 (s, 1H), 8.11 (d, *J* = 8.7 Hz, 2H), 7.26 (d, *J* = 8.7 Hz, 2H), 4.12 (s, 6H), 2.33 (s, 3H).

^{13}C NMR (100 MHz, $CDCl_3$) δ 172.7, 171.7, 168.8, 154.1, 150.8, 132.7, 129.8, 122.6, 116.0, 109.0, 55.7, 21.2.

HRMS (ESI) *m/z* calcd. for $C_{16}H_{14}N_4NaO_4$ $[M+Na]^+$: 349.0907; found: 349.0902.

(*E*)-2-(4,6-Dimethoxy-1,3,5-triazin-2-yl)-3-(4-nitrophenyl)acrylonitrile (1bf)

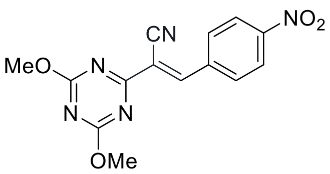

Compound **1bf** was synthesized following General Procedure A. Piperidine (0.2 equiv.) was employed as the base, and ethanol was used as the solvent. The reaction proceeded at 78 °C, after which the product was purified by recrystallization from ethanol to yield a yellow solid (0.36 g) in 46% yield.

m.p.: 245.3 − 245.9 °C

^{1}**H NMR** (600 MHz, $CDCl_3$) δ 8.78 (s, 1H), 8.35 (d, *J* = 8.8 Hz, 1H), 8.20 (d, *J* = 8.8 Hz, 2H), 4.14 (s, 6H).

^{13}**C NMR** (150 MHz, $CDCl_3$) δ 172.9, 170.8, 149.6, 148.8, 137.8, 131.7, 124.4, 115.2, 113.4, 55.9.

HRMS (ESI) *m/z* calcd. for $C_{14}H_{12}N_5O_4$ $[M+H]^+$: 314.0884; found: 314.0884.

(*Z*)-3-(3-Chlorophenyl)-2-(pyridin-2-yl)acrylonitrile (1bg)

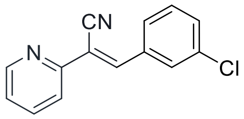

Compound **1bg** was synthesized following General Procedure A. Sodium methoxide (1.2 equiv.) was employed as the base, and methanol was used as the solvent. The reaction proceeded at room temperature, after which the product was purified by washing with cold methanol to yield a yellow solid (0.86 g) in 82% yield.

m.p.: 141.3 − 150.0 °C

^{1}**H NMR** (400 MHz, $CDCl_3$) δ 8.64 (ddd, *J* = 4.7, 1.7, 1.0 Hz, 1H), 8.42 (s, 1H), 7.90 (q, *J* = 2.8, 2.2 Hz, 2H), 7.80 (td, *J* = 7.9, 1.7 Hz, 1H), 7.76 (dt, *J* = 7.9, 1.2 Hz, 1H), 7.47 − 7.38 (m, 2H), 7.31 (ddd, *J* = 7.2, 4.7, 1.5 Hz, 1H).

^{13}**C NMR** (100 MHz, $CDCl_3$) δ 150.7, 149.9, 143.5, 137.6, 135.2, 135.1, 131.1, 130.4, 130.0, 127.8, 124.0, 121.6, 117.4, 111.8.

HRMS (ESI) *m/z* calcd. for $C_{14}H_{10}ClN_2$ $[M+H]^+$: 241.0533; found: 241.0521.

(*Z*)-3-(3-Chloro-2-fluorophenyl)-2-(6,7-dimethoxyquinazolin-4-yl)acrylonitrile (1bh)

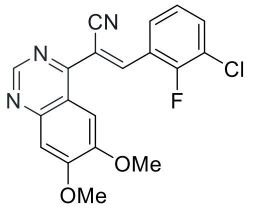

Compound **1bh** was synthesized following General Procedure A. Piperidine (0.2 equiv.) was employed as the base, and ethanol was used as the solvent. The reaction proceeded at 78 °C, after which the product was purified by recrystallization from ethanol to yield a yellow solid (0.46 g) in 50% yield.

m.p.: 198.9 − 200.2 °C

^{1}**H NMR** (400 MHz, $CDCl_3$) δ 9.16 (s, 1H), 8.33 (s, 1H), 8.28 (t, *J* = 7.2 Hz, 1H), 7.66 (s, 1H), 7.57 (t, *J* = 7.7 Hz, 1H), 7.39 (s, 1H), 7.29 (d, *J* = 8.2 Hz, 1H), 4.08 (s, 3H), 4.05 (s, 3H).

^{13}C NMR (100 MHz, $CDCl_3$) δ 158.2, 156.6 (d, J = 7.0 Hz), 155.6, 153.3, 151.3, 150.1, 143.0 (d, J = 6.6 Hz), 134.0, 127.2, 125.3 (d, J = 4.9 Hz), 123.0 (d, J = 11.5 Hz), 122.2 (d, J = 17.9 Hz), 118.2, 117.2, 112.1, 107.4, 101.9, 56.7, 56.5.

^{19}F NMR (376 MHz, $CDCl_3$) δ −118.1.

HRMS (ESI) *m/z* calcd. for $C_{19}H_{13}BrClFN_3NaO_2$ $[M+Na]^+$: 392.0573; found: 392.0591.

(*Z*)-3-(2,4-Difluorophenyl)-2-(pyridin-2-yl)acrylonitrile (1bi)

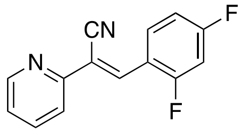

Compound **1bi** was synthesized following General Procedure A. Triethylamine (1.2 equiv.) was employed as the base, and methanol was used as the solvent. The reaction proceeded at room temperature, after which the product was purified by washing with cold methanol to yield a white solid (0.52 g) in 43% yield.

m.p.: 128.3 − 133.7 °C

^{1}H NMR (400 MHz, $CDCl_3$) δ 8.65 (ddd, J = 4.7, 1.8, 1.0 Hz, 1H), 8.62 (s, 1H), 8.36 (td, J = 8.6, 6.2 Hz, 1H), 7.85 − 7.71 (m, 2H), 7.30 (ddd, J = 7.3, 4.7, 1.3 Hz, 1H), 7.10 − 6.97 (m, 1H), 6.92 (ddd, J = 10.9, 8.6, 2.6 Hz, 1H).

^{13}C NMR (100 MHz, $CDCl_3$) δ 163.3 (dd, J = 12.2, 5.5 Hz), 163.3 (dd, J = 518.4, 12.3 Hz), 150.8, 149.9, 137.5, 135.7 (dd, J = 6.6, 1.9 Hz), 130.1 (dd, J = 10.1, 3.0 Hz), 123.9, 121.4, 118.4 (dd, J = 11.5, 4.0 Hz), 117.5, 112.4 (dd, J = 21.7, 3.7 Hz), 112.3 − 112.0 (m), 104.6 (t, J = 25.6 Hz).

^{19}F NMR (376 MHz, $CDCl_3$) δ −106.5 (d, J = 10.1 Hz), −111.5 (d, J = 10.1 Hz).

HRMS (ESI) *m/z* calcd. for $C_{14}H_9F_2N_2$ $[M+H]^+$: 243.07238; found: 243.0734.

(*Z*)-3-(Naphthalen-2-yl)-2-(pyridin-2-yl)acrylonitrile (1bj)

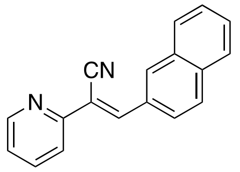

Compound **1bj** was synthesized following General Procedure A. Sodium methoxide (1.2 equiv.) was employed as the base, and methanol was used as the solvent. The reaction proceeded at room temperature, after which the product was purified by washing with cold methanol to yield a yellow solid (0.80 g) in 74% yield.

m.p.: 134.1 − 139.5 °C

^{1}H NMR (600 MHz, $CDCl_3$) δ 8.67 (dt, *J* = 4.7, 1.4 Hz, 1H), 8.64 (s, 1H), 8.40 − 8.34 (m, 1H), 8.20 (dd, *J* = 8.7, 1.9 Hz, 1H), 7.95 − 7.90 (m, 2H), 7.89 − 7.84 (m, 1H), 7.80 (d, *J* = 3.3 Hz, 2H), 7.62 − 7.48 (m, 2H), 7.29 (q, *J* = 4.4 Hz, 1H).
^{13}C NMR (150 MHz, $CDCl_3$) δ 151.4, 149.8, 145.3, 137.5, 134.6, 133.3, 131.8, 131.1, 129.1, 128.9, 128.1, 127.9, 126.9, 125.6, 123.6, 121.4, 118.1, 110.2.
HRMS (ESI) *m/z* calcd. for $C_{18}H_{13}N_2$ $[M+H]^+$: 257.1073; found: 257.1063.

(*Z*)-3-(Benzofuran-2-yl)-2-(5-(trifluoromethyl)pyridin-2-yl)acrylonitrile (1bk)

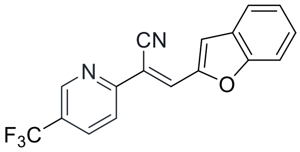

Compound **1bk** was synthesized following General Procedure A. Potassium hydroxide (1.2 equiv.) was employed as the base, and ethanol was used as the solvent. The reaction proceeded at room temperature, after which the product was purified by flash column chromatography on silica gel (petroleum ether/EtOAc = 10/1) a yellow solid (0.20 g) in 26% yield.
m.p.: 141.1 − 144.9 °C
^{1}H NMR (400 MHz, $CDCl_3$) δ 8.90 − 8.84 (m, 1H), 8.51 (s, 1H), 8.02 (ddd, *J* = 8.3, 2.3, 0.9 Hz, 1H), 7.89 (d, *J* = 8.3 Hz, 1H), 7.67 (dt, *J* = 7.8, 0.9 Hz, 1H), 7.60 (dq, *J* = 8.3, 0.9 Hz, 1H), 7.58 − 7.52 (m, 1H), 7.45 (ddd, *J* = 8.3, 7.2, 1.3 Hz, 1H), 7.31 (ddd, *J* = 7.9, 7.1, 0.9 Hz, 1H).
^{13}C NMR (100 MHz, $CDCl_3$) δ 156.2, 154.0, 151.0, 146.8 (d, *J* = 4.1 Hz), 134.9 (d, *J* = 3.5 Hz), 132.9, 128.1, 126.2 (q, *J* = 33.4 Hz), 124.8, 124.1, 122.6, 122.1, 121.0, 116.9, 115.2, 112.2, 108.0.
^{19}F NMR (376 MHz, $CDCl_3$) δ −65.5.
HRMS (ESI) *m/z* calcd. for $C_{17}H_{10}F_3N_2$ $[M+H]^+$: 315.0740; found: 315.0735.

(*Z*)-2-(Pyridin-2-yl)-3-(thiophen-2-yl)acrylonitrile (1bl)

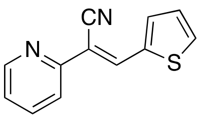

Compound **1bl** was synthesized following General Procedure A. Potassium hydroxide (1.2 equiv.) was employed as the base, and ethanol was used as the solvent. The reaction proceeded at room temperature, after which the product was purified by washing with cold ethanol to yield a yellow solid (0.37 g) in 69% yield.
m.p.: 117.2 − 118.5 °C

^{1}H NMR (400 MHz, $CDCl_3$) δ 8.60 (q, *J* = 1.8 Hz, 2H), 7.81 − 7.71 (m, 2H), 7.69 (dt, *J* = 7.9, 1.1 Hz, 1H), 7.60 (dt, *J* = 5.0, 1.1 Hz, 1H), 7.24 (ddd, *J* = 7.4, 4.7, 1.1 Hz, 1H), 7.17 (dd, *J* = 5.0, 3.8 Hz, 1H).

^{13}C NMR (100 MHz, $CDCl_3$) δ 150.9, 149.7, 137.9, 137.5, 137.3, 134.1, 131.5, 128.2, 123.3, 121.2, 118.0, 106.9.

HRMS (ESI) *m/z* calcd. for $C_{12}H_9N_2S$ $[M+H]^+$: 213.0481; found: 213.0474.

(*E*)-2-(5-Bromopyrimidin-2-yl)but-2-enenitrile (1ca)

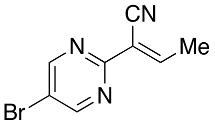

Compound **1ca** was synthesized in accordance with General Procedure E. The process resulted in the formation of a white solid (0.25 g) in 37% yield.

m.p.: 124.1 – 126.5 °C

^{1}H NMR (400 MHz, $CDCl_3$) δ 8.77 (s, 1H), 7.99 (q, *J* = 7.1 Hz, 1H), 2.30 (d, *J* = 7.2 Hz, 3H).

^{13}C NMR (100 MHz, $CDCl_3$) δ 158.3, 158.0, 152.4, 119.1, 116.9, 115.1, 18.0.

HRMS (ESI) *m/z* calcd. for $C_8H_6BrN_3Na$ $[M+H]^+$: 245.9643; found: 245.9637.

(*Z*)-4,4,4-Trifluoro-2-(pyridin-2-yl)but-2-enenitrile (1cb)

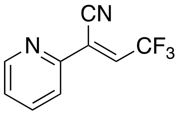

Compound **1cb** was synthesized following General Procedure A. Piperidine (0.2 equiv.) was employed as the base, and ethanol was used as the solvent. The reaction proceeded at 78 °C, after which the product was purified by flash column chromatography (petroleum ether/EtOAc = 10/1) to yield a yellow oil (0.52 g) in 73% yield.

^{1}H NMR (400 MHz, $CDCl_3$) δ 8.66 (d, *J* = 4.6 Hz, 1H), 7.91 − 7.81 (m, 2H), 7.69 (q, *J* = 7.5 Hz, 1H), 7.43 (ddd, *J* = 7.5, 4.6, 1.4 Hz, 1H).

^{13}C NMR (100 MHz, $CDCl_3$) δ 150.3, 147.5, 137.9, 131.4 (q, *J* = 36.3 Hz), 126.0, 122.9, 121.6 (q, *J* = 5.6 Hz), 121.6 (q, *J* = 271.0 Hz), 113.1.

^{19}F NMR (376 MHz, $CDCl_3$) δ −64.3.

HRMS (ESI) *m/z* calcd. for $C_9H_5F_3N_2Na[M+Na]^+$: 221.0297; found: 221.0300.

(*Z*)-4-Methyl-2-(pyridin-2-yl)pent-2-enenitrile (1cc)

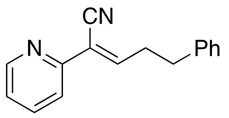

Compound **1cc** was synthesized following General Procedure A. Piperidine (0.2 equiv.) was employed as the base, and ethanol was used as the solvent. The reaction proceeded at 78 °C, after which the product was purified by flash column chromatography (petroleum ether/EtOAc = 20/1) to yield a yellow oil (0.70 g) in 87% yield.

^{1}H NMR (400 MHz, $CDCl_3$) δ 8.58 (d, *J* = 4.0 Hz, 1H), 7.73 (td, *J* = 7.7, 2.1 Hz, 1H), 7.68 (t, *J* = 7.5 Hz, 1H), 7.56 (d, *J* = 7.7 Hz, 1H), 7.33 (d, *J* = 7.5 Hz, 1H), 7.30 (d, *J* = 2.1 Hz, 1H), 7.27 − 7.18 (m, 4H), 3.21 − 2.67 (m, 4H).

^{13}C NMR (100 MHz, $CDCl_3$) δ 150.1, 149.9, 149.5, 139.9, 137.1, 128.4, 128.2, 126.2, 123.3, 120.7, 115.9, 115.9, 34.4, 33.5.

HRMS (ESI) *m/z* calcd. for $C_{16}H_{15}N_2$ $[M+H]^+$: 235.1230; found: 235.1236.

(*Z*)-5-Phenyl-2-(pyridin-2-yl)pent-2-enenitrile (1cd)

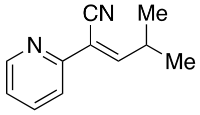

Compound **1cd** was synthesized following General Procedure A. Sodium methoxide (1.2 equiv.) was employed as the base, and methanol was used as the solvent. The reaction proceeded at 50 °C, after which the product was purified by flash column chromatography on silica gel (petroleum ether/EtOAc = 20/1) to yield a colorless oil (0.35 g) in 40% yield.

^{1}H NMR (400 MHz, $CDCl_3$) δ 8.58 (ddd, *J* = 4.7, 1.8, 0.9 Hz, 1H), 7.74 (td, *J* = 7.7, 1.8 Hz, 1H), 7.58 (d, *J* = 7.9 Hz, 1H), 7.49 (d, *J* = 10.4 Hz, 1H), 7.26 − 7.22 (m, 1H), 3.13 − 3.06 (m, 1H), 1.20 (s, 3H), 1.19 (s, 3H).

^{13}C NMR (100 MHz, $CDCl_3$) δ 157.7, 150.6, 149.7, 137.5, 123.5, 121.0, 116.3, 113.4, 31.9, 22.1.

HRMS (ESI) *m/z* calcd. for $C_{11}H_{12}N_2Na$ $[M+H]^+$: 195.0893; found: 195.0890.

(*Z*)-3-Cyclopropyl-2-(pyrazin-2-yl)acrylonitrile (1ce)

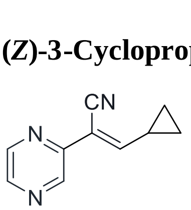

Compound **1ce** was synthesized following General Procedure A. Triethylamine (1.2 equiv.) was employed as the base, and methanol was used as the solvent. The reaction proceeded at room

temperature, after which the product was purified by washing with cold methanol to yield a white solid (0.42 g) in 49% yield.

m.p.: 116.6 − 144.9 °C

^{1}H NMR (400 MHz, $CDCl_3$) δ 8.79 (s, 1H), 8.53 − 8.39 (m, 2H), 7.06 (d, *J* = 11.1 Hz, 1H), 2.19 (dddd, *J* = 12.4, 11.2, 7.9, 4.4 Hz, 1H), 1.34 − 1.16 (m, 2H), 0.96 − 0.86 (m, 2H).

^{13}C NMR (100 MHz, $CDCl_3$) δ 158.8, 146.4, 144.0, 143.9, 141.5, 116.0, 109.1, 16.1, 10.6.

HRMS (ESI) *m/z* calcd. for $C_{10}H_{10}N_3$ $[M+H]^+$: 172.0869; found: 172.0868.

(*E*)-2-(Benzo[*d*]thiazol-2-yl)-3-cyclopentylacrylonitrile (1cf)

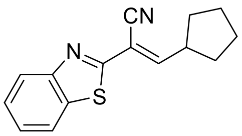

Compound **1cf** was synthesized following General Procedure A. Triethylamine (1.2 equiv.) was employed as the base, and ethanol was used as the solvent. The reaction proceeded at 50 °C, after which the product was purified by washing with cold ethanol to yield a white solid (0.33 g) in 61% yield.

^{1}H NMR (400 MHz, DMSO-*d_6*) δ 8.10 (d, *J* = 8.0 Hz, 1H), 8.03 (d, *J* = 8.1 Hz, 1H), 7.61 – 7.50 (m, 2H), 7.47 (t, *J* = 7.6 Hz, 1H), 3.07 (h, *J* = 8.3 Hz, 1H), 2.02 – 1.85 (m, 2H), 1.79 – 1.48 (m, 6H).

^{13}C NMR (100 MHz, DMSO-*d_6*) δ 161.8, 160.4, 152.6, 134.0, 126.9, 126.2, 123.1, 122.3, 114.5, 109.1, 43.0, 32.4, 25.4.

Spectroscopic data are in accordance with the literature[2].

Methyl (*E*)-5-(5-bromopyrimidin-2-yl)-5-cyanopent-4-enoate (1cg)

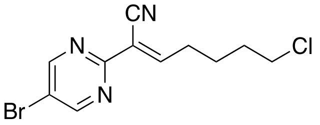

Compound **1cg** was synthesized following General Procedure B. To the reaction mixture, trimethyl chlorosilane (3.0 equiv.) and pyrrolidine (4.0 equiv.) were added dropwise while maintaining the temperature at 0 °C. Dichloromethane (0.2 M) served as the solvent. The reaction was allowed to proceed at room temperature. Following the completion of the reaction, as confirmed by TLC, the mixture was subjected to purification via flash column chromatography using a solvent system of petroleum ether/EtOAc in a 4/1 ratio. The process resulted in the formation of a white solid (0.28 g) in 64% yield.

m.p.: 41.3 − 43.8 °C

^{1}H NMR (400 MHz, $CDCl_3$) δ 8.78 (s, 2H), 7.90 (t, *J* = 8.0 Hz, 1H), 3.58 (t, *J* = 6.2 Hz, 2H), 2.70 (q, *J* = 7.5 Hz, 2H), 1.88 (q, *J* = 6.7, 6.0 Hz, 2H), 1.78 (p, *J* = 7.0 Hz, 2H).

^{13}C NMR (100 MHz, $CDCl_3$) δ 157.9, 157.8, 155.8, 119.1, 116.0, 114.8, 44.2, 31.7, 31.1, 25.2.

HRMS (ESI) *m/z* calcd. for $C_{11}H_{11}BrClN_3Na$ $[M+Na]^+$: 327.9717; found: 327.9721.

(*Z*)-2-(5-Bromopyridin-2-yl)-4-(1,3-dioxoisoindolin-2-yl)but-2-enenitrile (1ch)

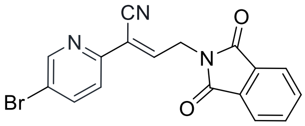

Compound **1ch** was synthesized in accordance with General Procedure C. The process resulted in the formation of a white solid (0.20 g) in 62% yield.

m.p.: 144.2 − 148.3 °C

^{1}H NMR (400 MHz, $CDCl_3$) δ 8.60 (s, 1H), 7.90 (s, 1H), 7.89 (s, 2H), 7.76 (d, *J* = 3.0 Hz, 2H), 7.63 − 7.57 (m, 1H), 7.53 (d, *J* = 8.4 Hz, 1H), 4.82 (dd, *J* = 6.6, 1.9 Hz, 2H).

^{13}C NMR (100 MHz, $CDCl_3$) δ 167.5, 151.1, 147.9, 143.5, 140.0, 134.5, 132.0, 123.8, 122.4, 121.4, 116.7, 114.8, 38.1.

HRMS (ESI) *m/z* calcd. for $C_{17}H_{11}BrN_3O_2$ $[M+H]^+$: 368.0029; found: 368.0046.

(*Z*)-4-(Benzyloxy)-2-(5-bromopyridin-2-yl)but-2-enenitrile (1ci)

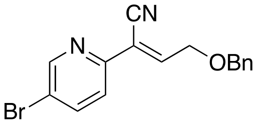

Compound **1ci** was synthesized following General Procedure A. K_2CO_3 (0.3 equiv.) was employed as the base, and toluene (0.1 M) was used as the solvent. The reaction proceeded at room temperature, after which the product was purified by washing with flash chromatography on silica gel to yield a white solid (4.13 g) in 61% yield.

m.p.: 75.3 − 81.8 °C

^{1}H NMR (400 MHz, $CDCl_3$) δ 8.65 (d, *J* = 2.4 Hz, 1H), 7.88 (dd, *J* = 8.5, 2.4 Hz, 1H), 7.73 (t, *J* = 6.3 Hz, 1H), 7.49 (d, *J* = 8.5 Hz, 1H), 7.40 − 7.35 (m, 4H), 7.31 (ddt , *J* = 8.5, 5.4, 3.2 Hz, 1H), 4.63 (s, 2H), 4.54 (d, *J* = 6.3 Hz, 2H).

^{13}C NMR (100 MHz, $CDCl_3$) δ 151.1, 148.3, 147.3, 139.9, 137.3, 128.7, 128.2, 128.1, 122.1, 121.2, 115.3, 115.2, 73.4, 68.6.

HRMS (ESI) *m/z* calcd. for $C_{16}H_{14}BrN_2O$ $[M+H]^+$: 329.0284; found: 329.0292.

(*Z*)-2-(5-Bromopyridin-2-yl)-4-((tert-butyldimethylsilyl)oxy)but-2-enenitrile (1cj)

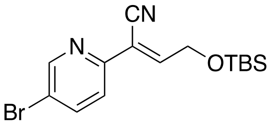

Compound **1cj** was synthesized in accordance with General Procedure C. The process resulted in the formation of a white solid (0.25 g) in 28% yield.

m.p.: 105.4 − 107.9 °C

^{1}H NMR (400 MHz, $CDCl_3$) δ 8.65 (dd, *J* = 2.4, 0.8 Hz, 1H), 7.88 (dd, *J* = 8.4, 2.4 Hz, 1H), 7.66 (t, *J* = 6.0 Hz, 1H), 7.51 (dd, *J* = 8.4, 0.8 Hz, 1H), 4.68 (d, *J* = 6.0 Hz, 2H), 0.94 (s, 9H), 0.14 (s, 6H).

^{13}C NMR (100 MHz, $CDCl_3$) δ 151.1, 150.4, 148.7, 139.9, 122.1, 121.0, 115.3, 113.5, 62.6, 29.8, 26.0, 18.5, −5.1.

HRMS (ESI) *m/z* calcd. for $C_{15}H_{22}BrN_2O_2Si$ $[M+H]^+$: 353.0679; found: 353.0686.

Methyl (*E*)-5-(5-bromopyrimidin-2-yl)-5-cyanopent-4-enoate (1ck)

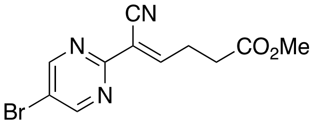

Compound **1ck** was synthesized in accordance with General Procedure C. The process resulted in the formation of a yellow solid (0.32 g) in 50% yield.

m.p.: 100.4 − 110.4 °C

^{1}H NMR (400 MHz, $CDCl_3$) δ 8.78 (s, 1H), 7.92 (t, *J* = 7.7 Hz, 2H), 3.72 (s, 2H), 2.96 (q, *J* = 7.1 Hz, 3H), 2.64 (t, *J* = 7.1 Hz, 3H).

^{13}C NMR (100 MHz, $CDCl_3$) δ 172.2, 158.2, 154.5, 119.5, 116.9, 114.9, 52.2, 32.3, 27.3.

HRMS (ESI) *m/z* calcd. for $C_{11}H_{10}BrN_3NaO_2$ $[M+Na]^+$: 317.9839; found: 317.9846.

(*Z*)-2-(5-Bromopyridin-2-yl)hept-2-en-6-ynenitrile (1cl)

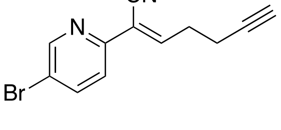

Compound **1cl** was synthesized in accordance with General Procedure C. The process resulted in the formation of a white solid (0.33 g) in 47% yield.

m.p.: 67.7 − 73.8 °C

^{1}H NMR (400 MHz, $CDCl_3$) δ 8.64 (d, *J* = 1.8 Hz, 1H), 7.88 (dd, *J* = 8.4, 2.4 Hz, 1H), 7.71 (t, *J* = 7.8 Hz, 1H), 7.49 (d, *J* = 8.1 Hz, 1H), 2.83 (q, *J* = 7.0 Hz, 2H), 2.49 (td, *J* = 6.9, 2.6 Hz, 2H), 2.06 (t, *J* = 2.6 Hz, 1H).

^{13}C NMR (100 MHz, $CDCl_3$) δ 151.0, 149.3, 148.7, 139.9, 122.1, 120.9, 116.1, 115.7, 82.0, 70.4, 31.0, 17.8.

HRMS (ESI) *m/z* calcd. for $C_{12}H_{10}BrN_2$ $[M+H]^+$: 261.0022; found: 261.0020.

(*Z*)-2-(5-Bromopyridin-2-yl)-5-(trimethylsilyl)pent-2-en-4-ynenitrile (1cm)

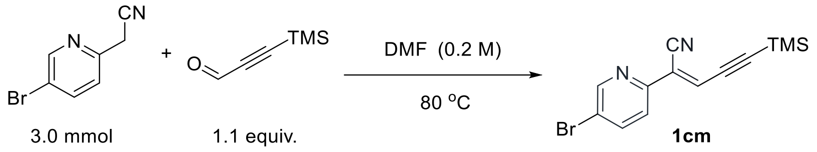

In a flame-dried flask under argon, 2-(5-bromopyridin-2-yl)acetonitrile (0.6 g, 3.0 mmol) and 3-trimethylsilylpropynal (0.44 g, 3.4 mmol) were dissolved in DMF (0.2 M) and stirred at 80 °C for 12 h. After completion, the reaction mixture was diluted with deionized water (60.0 mL) and extracted using ethyl acetate (50.0 mL × 3). The organic extracts were combined and dried over Na_2SO_4. After solvent removal under vacuum, the resulting residue was purified via flash chromatography (petroleum ether/EtOAc = 20/1) to obtain the product **1cm** as a yellow solid (0.24 g, 26% yield).

m.p.: 79.8 − 80.6°C

^{1}H NMR (400 MHz, $CDCl_3$) δ 8.62 (d, *J* = 2.4 Hz, 1H), 7.89 (dd, *J* = 8.4, 2.4 Hz, 1H), 7.52 (d, *J* = 8.3 Hz, 1H), 7.48 (s, 1H), 0.28 (s, 9H).

^{13}C NMR (100 MHz, $CDCl_3$) δ 151.3, 147.9, 140.0, 126.1, 122.8, 122.5, 121.9, 115.7, 113.7, 100.8, −0.3.

HRMS (ESI) *m/z* calcd. for $C_{13}H_{14}BrN_2Si$ $[M+H]^+$: 305.0104; found: 305.0105.

Ethyl (*E*)-3-(benzo[*d*]thiazol-2-yl)-3-cyanoacrylate (1cn)

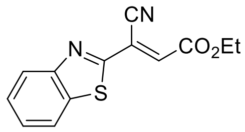

Compound **1cn** was synthesized following General Procedure A. Triethylamine (1.2 equiv.) was employed as the base, and ethanol was used as the solvent. The reaction proceeded at room temperature, after which the product was purified by washing with cold ethanol to yield a white solid (0.42 g) in 73% yield.

^{1}H NMR (400 MHz, $CDCl_3$) δ 8.12 (dt, *J* = 8.5, 0.8 Hz, 1H), 7.94 (ddd, *J* = 8.1, 1.4, 0.7 Hz, 1H), 7.61 – 7.44 (m, 3H), 4.40 (q, *J* = 7.1 Hz, 2H), 1.40 (t, *J* = 7.1 Hz, 3H).

^{13}C NMR (100 MHz, $CDCl_3$) δ 162.9, 153.7, 147.1, 135.1, 132.5, 132.4, 130.5, 129.4, 127.1, 126.2, 123.7, 121.8, 116.6, 105.7.

Spectroscopic data are in accordance with the literature[10].

(*Z*)-2-(5-Bromopyridin-2-yl)-4,4-dimethoxybut-2-enenitrile (1co)

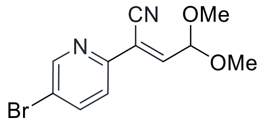

Compound **1co** was synthesized following General Procedure A. Sodium methoxide (1.2 equiv.) was employed as the base, and ethanol was used as the solvent. The reaction proceeded at 50 °C, after which the product was purified by washing with cold methanol to yield a yellow solid (0.32 g) in 22% yield.

m.p.: 93.4 − 95.8 °C

^{1}H NMR (400 MHz, $CDCl_3$) δ 8.65 (dd, *J* = 2.3, 0.8 Hz, 1H), 7.90 (dd, *J* = 8.4, 2.3 Hz, 1H), 7.53 (dd, *J* = 9.7, 7.7 Hz, 2H), 5.32 (d, *J* = 6.8 Hz, 1H), 3.47 (s, 6H).

^{13}C NMR (100 MHz, $CDCl_3$) δ 151.2, 148.1, 144.8, 139.9, 122.5, 121.7, 117.0, 115.0, 101.4, 54.1.

HRMS (ESI) *m/z* calcd. for $C_{11}H_{11}BrN_2NaO_2$ $[M+Na]^+$: 304.9896; found: 304.9888.

2-(Benzo[*d*]oxazol-2-yl)-2-cyclobutylideneacetonitrile (1da)

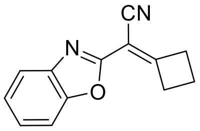

Compound **1da** was synthesized in accordance with General Procedure D. The process resulted in the formation of a yellow solid (0.86 g) in 68% yield.

m.p.: 170.2 − 170.7 °C

^{1}H NMR (400 MHz, $CDCl_3$) δ 7.79 − 7.70 (m, 1H), 7.57 − 7.48 (m, 1H), 7.40 − 7.30 (m, 2H), 3.51 − 3.42 (m, 2H), 3.29 − 3.18 (m, 2H), 2.31 (p, *J* = 7.9 Hz, 2H).

^{13}C NMR (100 MHz, $CDCl_3$) δ 178.8, 157.6, 150.4, 141.4, 125.8, 125.1, 120.5, 113.5, 110.7, 98.3, 35.1, 33.6, 17.1.

HRMS (ESI) *m/z* calcd. for $C_{13}H_{10}N_2NaO$ $[M+Na]^+$: 233.0685; found: 233.0681.

2-(Benzo[*d*]thiazol-2-yl)-2-cyclopentylideneacetonitrile (1db)

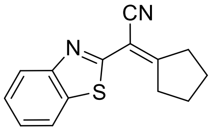

Compound **1db** was synthesized in accordance with General Procedure D. The process resulted in the formation of a white solid (0.49 g) in 34% yield.

m.p.: 209.7 − 210.6 °C

^{1}H NMR (600 MHz, $CDCl_3$) δ 8.07 (d, *J* = 8.2 Hz, 1H), 7.90 (d, *J* = 8.0 Hz, 1H), 7.51 (t, *J* = 7.7 Hz, 1H), 7.41 (t, *J* = 7.6 Hz, 1H), 3.08 (td, *J* = 7.2, 2.0 Hz, 2H), 2.94 (t, *J* = 7.3 Hz, 2H), 1.97 (p, *J* = 6.9 Hz, 2H), 1.90 (p, *J* = 6.9 Hz, 2H).

^{13}C NMR (150 MHz, $CDCl_3$) δ 175.8, 161.3, 153.5, 134.9, 126.7, 125.7, 123.7, 121.5, 116.8, 103.3, 37.8, 36.0, 27.2, 25.7.

HRMS (ESI) *m/z* calcd. for $C_{14}H_{13}N_2S$ $[M+H]^+$: 241.0794; found: 241.0801.

2-(Benzo[*d*]oxazol-2-yl)-2-cyclohexylideneacetonitrile (1dc)

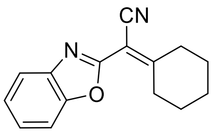

Compound **1dc** was synthesized in accordance with General Procedure D. The process resulted in the formation of a yellow solid (0.76 g) in 53% yield.

m.p.: 97.7 − 98.8 °C

^{1}H NMR (400 MHz, $CDCl_3$) δ 7.74 (d, *J* = 7.4 Hz, 1H), 7.65 − 7.52 (m, 1H), 7.46 − 7.31 (m, 2H), 3.16 (t, *J* = 6.2 Hz, 2H), 2.80 (t, *J* = 6.3 Hz, 2H), 2.07 − 1.58 (m, 6H).

^{13}C NMR (100 MHz, $CDCl_3$) δ 174.5, 157.6, 149.9, 141.3, 125.9, 125.0, 120.3, 115.5, 110.9, 97.7, 36.7, 32.5, 28.6, 28.2, 25.7.

HRMS (ESI) *m/z* calcd. for $C_{15}H_{15}N_2O$ $[M+H]^+$: 239.1179; found: 239.1176.

2-(Benzo[*d*]thiazol-2-yl)-2-(tetrahydro-4H-pyran-4-ylidene)acetonitrile (1dd)

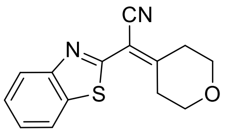

Compound **1dd** was synthesized in accordance with General Procedure D. The process resulted in the formation of a yellow solid (0.83 g) in 54% yield.

m.p.: 101.7 − 105.5 °C

^{1}H NMR (400 MHz, $CDCl_3$) δ 8.03 (d, *J* = 8.2 Hz, 1H), 7.91 (d, *J* = 8.1 Hz, 1H), 7.52 (t, *J* = 7.8 Hz, 1H), 7.43 (t, *J* = 7.8 Hz, 1H), 3.93 (t, *J* = 5.6 Hz, 2H), 3.84 (t, *J* = 5.7 Hz, 2H), 3.29 (t, *J* = 5.6 Hz, 2H), 2.90 (t, *J* = 5.6 Hz, 2H).

^{13}C NMR (100 MHz, $CDCl_3$) δ 164.1, 160.3, 153.4, 135.2, 126.8, 126.1, 123.7, 121.7, 116.6, 104.3, 68.5, 68.1, 36.7, 33.4.

HRMS (ESI) *m/z* calcd. for $C_{14}H_{13}N_2OS$ $[M+H]^+$: 257.0743; found: 257.0741.

2-(5-(4-Chlorophenyl)-1,3,4-oxadiazol-2-yl)-3-methylbut-2-enenitrile (1de)

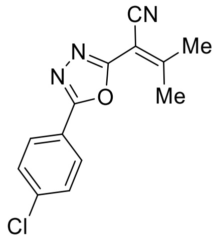

Compound **1de** was synthesized in accordance with General Procedure D. The process resulted in the formation of a yellow solid (0.20 g) in 54% yield.

m.p.: 121.1 − 125.9 °C

^{1}H NMR (400 MHz, $CDCl_3$) δ 8.03 (d, *J* = 8.7 Hz, 2H), 7.51 (d, *J* = 8.7 Hz, 2H), 2.55 (s, 3H), 2.45 (s, 3H).

^{13}C NMR (100 MHz, $CDCl_3$) δ 168.9, 163.5, 159.8, 138.7, 129.7, 128.5, 121.7, 114.6, 97.3, 26.9, 24.1.

HRMS (ESI) *m/z* calcd. for $C_{13}H_{11}ClN_3O$ $[M+H]^+$: 260.0585; found: 260.0584.

2-(3,5-Dichloropyridin-2-yl)acrylonitrile (1df)

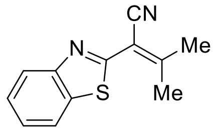

Compound **1df** was synthesized following General Procedure C. The process resulted in the formation of a white solid (0.24 g) in 52% yield.

^{1}H NMR (400 MHz, $CDCl_3$) δ 8.06 (dd, *J* = 8.2, 1.1 Hz, 1H), 7.91 (dd, *J* = 8.1, 1.1 Hz, 1H), 7.51 (tt, *J* = 7.2, 1.0 Hz, 1H), 7.43 (ddd, *J* = 8.1, 7.2, 1.1 Hz, 1H), 2.49 (s, 3H), 2.41 (s, 3H).

^{13}C NMR (100 MHz, $CDCl_3$) δ 163.4, 161.0, 153.4, 135.2, 126.7, 125.9, 123.7, 121.6, 117.2, 106.3, 27.0, 23.5.

Spectroscopic data are in accordance with the literature[11].

(*E/Z*)-3-(Benzo[*d*]thiazol-2-yl)-3-cyano-2-methylallyl benzoate (1dg)

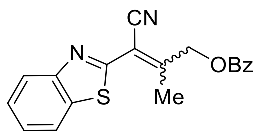

Compound **1dg** was synthesized in accordance with General Procedure D. The process resulted in the formation of a yellow solid (0.20 g) in 54% yield. Compound **1dg** was obtained as an inseparable *Z*/*E* isomer mixture, with a ratio of 3:1.

m.p.: 116.1 − 116.9 °C

^{1}H NMR (400 MHz, $CDCl_3$) δ 8.11 (*minor*, d, *J* = 6.4 Hz, 3H), 8.07 (*major*, d, *J* = 8.2 Hz, 3H), 7.94 (*minor*, d, *J* = 4.7 Hz, 1H), 7.92 (*major*, d, *J* = 5.0 Hz, 1H), 7.61 (*minor*, t, *J* = 7.6 Hz, 5H), 7.50 (*major*, dq, *J* = 18.5, 6.9 Hz, 5H), 5.36 (*minor*, s, 2H), 5.79 (*major*, s, 2H), 2.58 (*minor*, s, 3H), 2.48 (*major*, s, 3H).

^{13}C NMR (100 MHz, $CDCl_3$, *carbons from the regioisomer are indicated in parentheses*) δ 166.1 (160.0), 160.7, 159.9 (160.0), 158.7, 153.8 (153.4), 135.2 (135.3), 133.6 (133.7), 129.8 (130.0), 129.4 (129.2), 128.7, 126.9 (126.9), 126.2(126.4), 123.9 (124.0), 121.7 (121.7), 116.6 (115.8), 106.7 (108.2), 65.7 (67.4), 21.1 (19.1).

HRMS (ESI) *m/z* calcd. for $C_{19}H_{14}N_2NaO_2S$ $[M+Na]^+$: 357.0668; found: 357.0676.

Ethyl (2*E*,4*E*)-5-cyano-5-(4-(4-fluorophenyl)thiazol-2-yl)penta-2,4-dienoate (1fa)

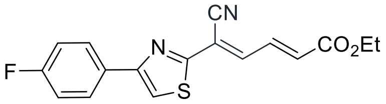

Compound **1fa** was synthesized following General Procedure A. Triethylamine (1.2 equiv.) was employed as the base, and ethanol was used as the solvent. The reaction proceeded at room temperature, after which the product was purified by flash column chromatography on silica gel (petroleum ether/EtOAc = 5/1) to yield a yellow solid (0.20 g) in 66% yield.

m.p.: 120.4 − 127.8 °C

^{1}H NMR (400 MHz, $CDCl_3$) δ 7.91 (td, *J* = 8.3, 5.8 Hz, 3H), 7.75 (ddd, *J* = 14.0, 12.1, 1.7 Hz, 1H), 7.57 (s, 1H), 7.14 (t, *J* = 8.6 Hz, 2H), 6.44 (d, *J* = 15.1 Hz, 1H), 4.30 (q, *J* = 7.0 Hz, 2H), 1.35 (t, *J* = 7.1 Hz, 3H).

^{13}C NMR (100 MHz, $CDCl_3$) δ 165.4, 163.3 (d, *J* = 249.0 Hz), 160.4, 156.9, 140.5, 137.5, 131.80, 129.8 (d, *J* = 3.3 Hz), 128.6 (d, *J* = 8.3 Hz), 116.1 (d, *J* = 21.7 Hz), 115.3, 114.5, 113.6, 61.5, 14.4.

^{19}F NMR (376 MHz, $CDCl_3$) δ −115.4.

HRMS (ESI) *m/z* calcd. for $C_{17}H_{14}FN_2O_2S$ $[M+H]^+$: 329.0755; found: 329.0762.

(2*E*,4*E*)-2-(4-(4-Methoxyphenyl)thiazol-2-yl)-6-oxo-6-phenylhexa-2,4-dienenitrile (1fb)

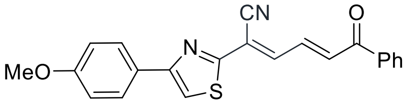

Compound **1fb** was synthesized in accordance with General Procedure D with the inclusion of additional acetic acid (0.1 equiv.). The process resulted in the formation of a yellow solid (0.30 g) in 67% yield.

m.p.: 124.2 − 129.8 °C

^{1}H NMR (400 MHz, $CDCl_3$) δ 8.07 (d, *J* = 12.0 Hz, 1H), 8.01 (d, *J* = 7.4 Hz, 2H), 7.93 − 7.84 (m, 3H), 7.63 (t, *J* = 7.4 Hz, 1H), 7.57 − 7.48 (m, 4H), 6.99 (d, *J* = 8.8 Hz, 2H), 3.87 (s, 3H).

^{13}C NMR (100 MHz, $CDCl_3$) δ 189.4, 160.4, 160.2, 157.9, 140.7, 137.4, 137.3, 134.4, 133.7, 129.0, 128.8, 128.1, 126.5, 114.7, 114.4, 114.3, 55.5.

HRMS (ESI) *m/z* calcd. for $C_{22}H_{17}N_2O_2S$ $[M+H]^+$: 373.1005; found: 373.1015.

(2*E*,4*E*,8*Z*)-2-(5-Bromopyrimidin-2-yl)undeca-2,4,8-trienenitrile (1fc)

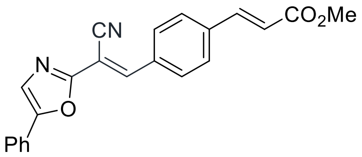

Compound **1fc** was synthesized in accordance with General Procedure D. The process resulted in the formation of a yellow solid (0.24 g) in 51% yield.

m.p.: 136.1 − 139.7 °C

^{1}H NMR (400 MHz, DMSO-*d$_6$*) δ 8.24 (d, *J* = 146.6 Hz, 1H), 8.10 (d, *J* = 8.0 Hz, 1H), 7.95 (d, *J* = 2.8 Hz, 2H), 7.89 (d, *J* = 7.9 Hz, 1H), 7.86 (s, 2H), 7.72 (d, *J* = 16.0 Hz, 1H), 7.54 (q, *J* = 7.4 Hz, 2H), 7.43 (dt, *J* = 16.8, 7.2 Hz, 2H), 6.80 (dd, *J* = 16.0, 12.1 Hz, 1H), 3.75 (s, 3H).

^{13}C NMR (100 MHz, DMSO-*d$_6$*) δ 166.5, 156.5, 152.0, 145.2, 143.1, 137.4, 134.0, 130.8, 130.4, 129.2, 129.1, 126.7, 125.0, 124.4, 120.2, 115.2, 99.3, 51.7.

HRMS (ESI) *m/z* calcd. for $C_{22}H_{17}N_2O_3$ $[M+H]^+$: 357.1234; found: 357.1250.

(2*Z*,4*E*)-5-Phenyl-2-(pyridin-2-yl)penta-2,4-dienenitrile (1fd)

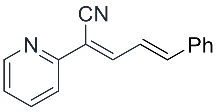

Compound **1fd** was synthesized following General Procedure A. Triethylamine (1.2 equiv.) was employed as the base, and methanol was used as the solvent. The reaction proceeded at 50 °C, after which the product was purified by flash column chromatography on silica gel (petroleum ether/EtOAc = 10/1) to yield a yellow solid (0.49 g) in 42% yield.

m.p.: 95.4 − 97.2 °C

^{1}H NMR (400 MHz, DMSO-d_6) δ 8.64 (d, J = 4.7 Hz, 1H), 8.26 (d, J = 11.1 Hz, 1H), 7.91 (td, J = 7.8, 1.8 Hz, 1H), 7.75 (d, J = 8.0 Hz, 1H), 7.65 (d, J = 7.2 Hz, 2H), 7.42 (tq, J = 8.4, 4.3, 3.8 Hz, 6H), 7.37 − 7.26 (m, 1H).

^{13}C NMR (100 MHz, DMSO-d_6) δ 150.6, 149.7, 145.1, 144.0, 137.6, 135.3, 130.0, 129.0, 127.7, 124.1, 123.6, 120.4, 116.3, 112.2.

HRMS (ESI) *m/z* calcd. for $C_{16}H_{13}N_2$ $[M+H]^+$: 233.1073; found: 233.1068.

(*Z*)-5-Methyl-2-(4-(trifluoromethyl)pyridin-2-yl)hexa-2,4-dienenitrile (1fe)

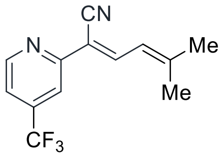

Compound **1fe** was synthesized following General Procedure A. Triethylamine (1.2 equiv.) was employed as the base, and methanol was used as the solvent. The reaction proceeded at 50 °C, after which the product was purified by flash column chromatography on silica gel (petroleum ether/EtOAc = 20/1) to yield a white solid (0.86 g) in 71% yield.

m.p.: 78.3 − 80.1 °C

^{1}H NMR (400 MHz, $CDCl_3$) δ 8.73 (d, J = 4.9 Hz, 1H), 8.40 (d, J = 12.1 Hz, 1H), 7.76 (s, 1H), 7.41 (d, J = 4.9 Hz, 1H), 6.58 (d, J = 12.2 Hz, 1H), 2.05 (d, J = 6.7 Hz, 6H).

^{13}C NMR (100 MHz, $CDCl_3$) δ 153.7, 152.8, 150.6, 143.3, 139.7 (q, J = 34.3 Hz), 123.1, 122.7 (q, J = 273.4 Hz), 118.3 (q, J = 3.5 Hz), 116.6, 116.5 (q, J = 3.7 Hz), 108.7, 27.3, 20.0.

^{19}F NMR (376 MHz, $CDCl_3$) δ −68.1.

HRMS (ESI) *m/z* calcd. for $C_{13}H_{12}F_3N_2$ $[M+H]^+$: 253.0947; found: 253.0944.

(2*E*,4*Z*)-2-(Benzo[*d*]thiazol-2-yl)-4-bromo-5-phenylpenta-2,4-dienenitrile (1ff)

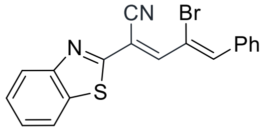

Compound **1ff** was synthesized in accordance with General Procedure D. The process resulted in the formation of a yellow solid (0.54 g) in 74% yield.

m.p.: 126.5 − 128.3 °C

^{1}H NMR (400 MHz, DMSO-d_6) δ 8.33 (s, 1H), 8.25 (s, 1H), 8.19 (d, J = 7.9 Hz, 1H), 8.08 (d, J = 7.7 Hz, 1H), 7.94 − 7.87 (m, 2H), 7.60 (t, J = 7.0 Hz, 1H), 7.55 − 7.48 (m, 4H).

^{13}C NMR (100 MHz, DMSO-d_6) δ 162.9, 152.9, 147.2, 145.3, 134.6, 134.2, 130.7, 130.3, 128.7, 127.3, 126.4, 123.2, 122.6, 115.1, 114.9, 106.5.

HRMS (ESI) *m/z* calcd. for $C_{18}H_{12}BrN_2S$ $[M+H]^+$: 366.9899; found: 366.9903.

(*E*)-2-(4-(4-Fluorophenyl)thiazol-2-yl)trideca-2,12-dienenitrile (1fg)

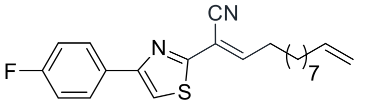

Compound **1fg** was synthesized in accordance with General Procedure D. The process resulted in the formation of a yellow oil (0.14 g) in 32% yield.

^{1}H NMR (400 MHz, $CDCl_3$) δ 7.94 − 7.85 (m, 2H), 7.47 (t, *J* = 8.0 Hz, 1H), 7.42 (s, 1H), 7.12 (t, *J* = 8.5 Hz, 2H), 5.80 (dt, *J* = 16.8, 8.4 Hz, 1H), 5.01 (s, 1H), 4.99 − 4.88 (m, 1H), 2.62 (q, *J* = 7.6 Hz, 2H), 2.04 (d, *J* = 6.7 Hz, 2H), 1.65 − 1.52 (m, 2H), 1.47 − 1.23 (m, 10H).

^{13}C NMR (100 MHz, $CDCl_3$) δ 163.1 (d, *J* = 248.2 Hz), 161.3, 155.7, 151.8, 139.3, 130.2 (d, *J* = 3.1 Hz), 128.4 (d, *J* = 8.2 Hz), 115.9 (d, *J* = 21.7 Hz), 115.2, 114.3, 113.0, 110.7, 33.9, 32.3, 29.4, 29.4, 29.4, 29.2, 29.0, 28.5.

^{19}F NMR (376 MHz, $CDCl_3$) δ −116.1.

HRMS (ESI) *m/z* calcd. for $C_{22}H_{26}FN_2S$ $[M+H]^+$: 369.1795; found: 369.1791.

(2*E*,4*E*,8*Z*)-2-(5-Bromopyrimidin-2-yl)undeca-2,4,8-trienenitrile (1fh)

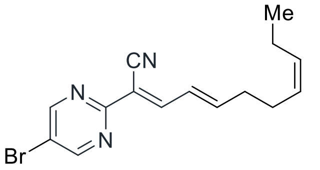

Compound **1fh** was synthesized following General Procedure A. Triethylamine (1.2 equiv.) was employed as the base, and methanol was used as the solvent. The reaction proceeded at 50 °C, after which the product was purified by flash column chromatography on silica gel (petroleum ether/EtOAc = 10/1) to yield a yellow oil (0.34 g) in 37% yield.

^{1}H NMR (400 MHz, $CDCl_3$) δ 8.75 (s, 2H), 8.15 (d, *J* = 11.5 Hz, 1H), 6.79 (dd, *J* = 15.0, 11.6 Hz, 1H), 6.54 (dt, *J* = 14.6, 7.0 Hz, 1H), 5.48 − 5.39 (m, 1H), 5.37 − 5.25 (m, 1H), 2.38 (q, *J* = 7.2 Hz, 2H), 2.24 (q, *J* = 7.1 Hz, 2H), 2.04 (p, *J* = 7.3, 6.9 Hz, 2H), 0.96 (t, *J* = 7.5 Hz, 3H).

^{13}C NMR (100 MHz, $CDCl_3$) δ 159.3, 158.1, 151.3, 150.4, 133.3, 127.7, 127.1, 118.7, 115.7, 110.7, 33.8, 26.1, 20.7, 14.4.

HRMS (ESI) *m/z* calcd. for $C_{15}H_{16}BrN_3Na$ $[M+Na]^+$: 340.0420; found: 340.0412.

(2*E*,8*Z*)-2-(Benzo[*d*]oxazol-2-yl)undeca-2,8-dienenitrile (1fi)

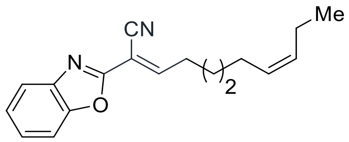

Compound **1fi** was synthesized in accordance with General Procedure D with the inclusion of additional acetic acid (0.1 equiv.). The process resulted in the formation of a yellow oil (0.34 g) in 38% yield.

^{1}H NMR (400 MHz, $CDCl_3$) δ 7.79 (d, *J* = 7.0 Hz, 1H), 7.69 (t, *J* = 8.0 Hz, 1H), 7.56 (d, *J* = 5.9 Hz, 1H), 7.45 − 7.34 (m, 2H), 5.43 (dt, *J* = 14.2, 7.3 Hz, 1H), 5.39 − 5.27 (m, 1H), 2.73 (q, *J* = 7.6 Hz, 2H), 2.09 (dq, *J* = 27.1, 7.4 Hz, 4H), 1.66 (q, *J* = 9.8, 8.5 Hz, 2H), 1.51 (p, *J* = 7.6 Hz, 2H), 0.98 (t, *J* = 7.6 Hz, 3H).

^{13}C NMR (100 MHz, $CDCl_3$) δ 157.6, 157.1, 150.7, 141.4, 132.6, 128.3, 126.3, 125.3, 120.8, 113.4, 110.8, 106.0, 32.4, 29.3, 27.8, 26.8, 20.7, 14.5.

HRMS (ESI) *m/z* calcd. for $C_{18}H_{20}N_2NaO$ $[M+H]^+$: 303.1468; found: 303.1474.

(*R*,*Z*)-3-(4-(Prop-1-en-2-yl)cyclohex-1-en-1-yl)-2-(pyridin-2-yl)acrylonitrile (1ga)

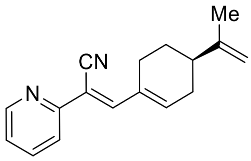

Compound **1ga** was synthesized following General Procedure A. Piperidine (0.03 equiv.) was employed as the base, and ethanol was used as the solvent. The reaction proceeded at 78 °C, after which the product was purified by flash column chromatography (petroleum ether/EtOAc = 20/1) to yield a yellow oil (0.38 g) in 60% yield.

^{1}H NMR (400 MHz, DMSO-*d*$_6$) δ 8.59 (d, *J* = 4.6 Hz, 1H), 7.93 (s, 1H), 7.88 (t, *J* = 8.0 Hz, 1H), 7.67 (d, *J* = 8.1 Hz, 1H), 7.36 (dd, *J* = 7.9, 4.6 Hz, 1H), 6.59 (d, *J* = 4.9 Hz, 1H), 4.73 (d, *J* = 8.7 Hz, 2H), 2.77 (d, *J* = 17.1 Hz, 1H), 2.65 − 2.51 (m, 1H), 2.37 (dt, *J* = 13.2, 6.8 Hz, 1H), 2.17 (d, *J* = 11.5 Hz, 2H), 1.90 (d, *J* = 12.8 Hz, 1H), 1.72 (s, 3H), 1.48 (tt, *J* = 11.0, 5.5 Hz, 1H).

^{13}C NMR (100 MHz, DMSO-*d*$_6$) δ 151.4, 149.5, 148.3, 148.0, 143.6, 137.7, 134.2, 123.3, 120.2, 117.7, 109.3, 106.2, 31.7, 26.6, 25.6, 23.5, 20.6.

HRMS (ESI) *m/z* calcd. for $C_{17}H_{19}N_2$ $[M+H]^+$: 251.1543; found: 251.1554.

(*E*)-3-((1*S*,5*R*)-6,6-Dimethylbicyclo[3.1.1]hept-2-en-3-yl)-2-(4-(4-methoxyphenyl)thiazol-2-yl)acrylonitrile (1gb)

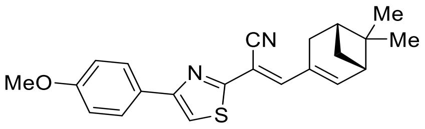

Compound **1gb** was synthesized following General Procedure A. Sodium methoxide (1.2 equiv.) was employed as the base, and methanol was used as the solvent. The reaction proceeded at room temperature, after which the product was purified by flash column chromatography on silica gel (petroleum ether/EtOAc = 50/1) to yield a colorless oil (0.45 g) in 22% yield.

^{1}H NMR (400 MHz, $CDCl_3$) δ 7.86 (d, *J* = 8.0 Hz, 2H), 7.79 (s, 1H), 7.35 (s, 1H), 6.95 (d, *J* = 6.9 Hz, 2H), 6.49 (s, 1H), 3.80 (s, 3H), 3.38 (d, *J* = 5.0 Hz, 1H), 2.57 (dp, *J* = 12.7, 3.3 Hz, 3H), 2.19 (s, 1H), 1.41 (s, 3H), 1.23 (d, *J* = 9.2 Hz, 1H), 0.88 (s, 3H).

^{13}C NMR (100 MHz, $CDCl_3$) δ 163.3, 160.0, 156.6, 145.5, 145.1, 140.6, 127.9, 127.0, 117.1, 114.3, 111.9, 101.3, 55.5, 42.4, 40.2, 38.1, 33.7, 31.5, 26.0, 21.4.

HRMS (ESI) *m/z* calcd. for $C_{22}H_{23}N_2OS$ $[M+H]^+$: 363.1526; found: 363.1524.

(*R*,*Z*)-5,9-Dimethyl-2-(pyridin-2-yl)deca-2,8-dienenitrile (1gc)

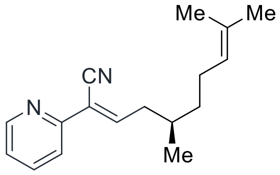

Compound **1gc** was synthesized in accordance with General Procedure D and extra acetic acid (0.1 equiv.) was added. The process resulted in the formation of a yellow oil (0.16 g) in 36% yield.

^{1}H NMR (400 MHz, $CDCl_3$) δ 8.61 (d, *J* = 4.8 Hz, 1H), 7.77 (t, *J* = 7.8 Hz, 1H), 7.71 (t, *J* = 8.0 Hz, 1H), 7.61 (d, *J* = 8.0 Hz, 1H), 7.27 (dd, *J* = 8.0, 4.1 Hz, 1H), 5.12 (s, 1H), 2.66 (dt, *J* = 13.8, 6.7 Hz, 1H), 2.53 (dt, *J* = 15.3, 8.1 Hz, 1H), 2.06 (qq, *J* = 14.5, 7.0 Hz, 2H), 1.83 (q, *J* = 6.8 Hz, 1H), 1.71 (s, 3H), 1.63 (s, 3H), 1.46 (dt, *J* = 13.5, 7.2 Hz, 1H), 1.32 (td, *J* = 14.7, 14.3, 7.6 Hz, 1H), 1.03 (d, *J* = 6.6 Hz, 3H).

^{13}C NMR (100 MHz, $CDCl_3$) δ 150.8, 150.6, 149.8, 137.4, 131.8, 124.3, 123.5, 120.9, 116.6, 116.3, 39.5, 36.9, 32.9, 25.9, 25.7, 19.8, 17.8.

HRMS (ESI) *m/z* calcd. for $C_{17}H_{22}N_2Na$ $[M+Na]^+$: 277.1675; found: 277.1679.

Ethyl (*E*)-2-(4-(2-(benzo[*d*]thiazol-2-yl)-2-cyanovinyl)-3-isobutoxyphenyl)-4-methylthiazole-5-carboxylate (1gd)

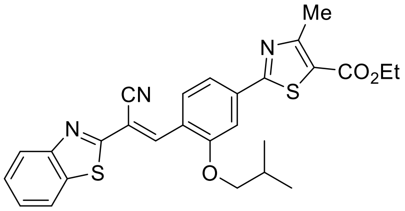

Compound **1gd** was synthesized following General Procedure A. Triethylamine (1.2 equiv.) was employed as the base, and methanol was used as the solvent. The reaction proceeded at room temperature, after which the product was purified by washing with cold methanol to yield a yellow solid (0.12 g) in 68% yield.

m.p.: 169.8 − 185.6 °C

^{1}H NMR (400 MHz, $CDCl_3$) δ 8.82 − 8.77 (m, 1H), 8.55 (s, 1H), 8.10 (dd, *J* = 8.6, 3.4 Hz, 2H), 7.88 (d, *J* = 8.0 Hz, 1H), 7.51 (t, *J* = 7.7 Hz, 1H), 7.42 (t, *J* = 7.6 Hz, 1H), 7.00 (d, *J* = 8.8 Hz, 1H), 4.34 (q, *J* = 7.1 Hz, 2H), 3.89 (d, *J* = 6.5 Hz, 2H), 2.77 (s, 3H), 2.22 (dp, *J* = 13.6, 6.8 Hz, 1H), 1.38 (t, *J* = 7.1 Hz, 3H), 1.11 (s, 3H), 1.10 (s, 3H).

^{13}C NMR (100 MHz, $CDCl_3$) δ 163.3, 160.0, 156.6, 145.5, 145.1, 140.6, 127.9, 127.0, 117.1, 114.3, 111.9, 101.3, 55.5, 42.4, 40.2, 38.1, 33.7, 31.5, 26.0, 21.4.

HRMS (ESI) *m/z* calcd. for $C_{27}H_{25}N_2NaO_3$ $[M+Na]^+$: 526.1230; found: 526.1226.

(3*R*,8*S*,9*S*,10*R*,13*R*,14*S*,17*R*)-10,13-Dimethyl-17-((*R*)-6-methylheptan-2-yl)2,3,4,7,8,9,10,11,12,13,14,15,16,17-tetradecahydro-1*H*-cyclopenta[*a*]phenanthren-3-yl(*Z*)-3-(5-bromopyridin-2-yl)-3-cyanoacrylate (1ge)

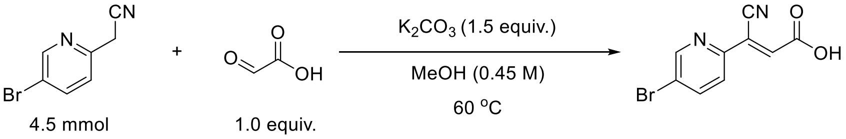

In a flame-dried 25 mL round-bottom flask equipped with a magnetic stirring bar, 2-oxoacetic acid (50 *wt*% in water, 3.0 mmol, 0.35 mL) was dissolved in methanol (10.0 mL). To this solution, potassium carbonate (K_2CO_3, 4.5 mmol, 0.62 g, 1.5 equiv.) and 2-(5-bromopyridin-2-yl)acetonitrile (3.0 mmol, 0.59 g) were added. The reaction mixture was then heated under reflux at 60 °C for 6 hours. After cooling to room temperature, the mixture was filtered, and the filter cake was washed with dichloromethane (5.0 mL × 3). The combined organic layers were acidified to pH = 4 by the slow addition of 1 N hydrochloric acid (20.0 mL). The resulting mixture was then concentrated to provide the crude (*Z*)-3-(5-bromopyridin-2-yl)-3-cyanoacrylic acid, which was used directly for subsequent reactions.

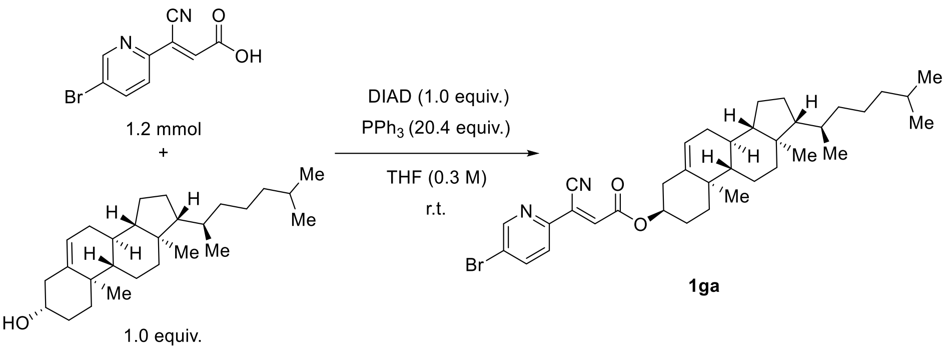

A solution of (*Z*)-3-(5-bromopyridin-2-yl)-3-cyanoacrylic acid (0.3 g, 1.2 mmol), triphenylphosphine (6.4 g, 20.4 mmol), and cholesterol (0.46 g, 1.2 mmol) in dry THF (8.0 mL) was prepared in a 25 mL flame-dried round-bottom flask. To this, diisopropyl azodicarboxylate (DIAD) (0.24 g, 1.2 mmol) in THF (4.0 mL) was added dropwise under an inert atmosphere. The mixture was stirred for 12 hours at room temperature, and then the solvent was evaporated under reduced pressure. The residue was dissolved in ethyl ether (10 mL), the precipitated solid was filtered off, and the filtrate was concentrated. Flash chromatography (petroleum ether/EtOAc = 10/1) of the concentrate afforded a colorless oil (0.45 g) in a 22% yield over two steps.

^{1}H NMR (600 MHz, $CDCl_3$) δ 8.69 (d, *J* = 2.3 Hz, 1H), 7.97 (dd, *J* = 8.4, 2.4 Hz, 1H), 7.76 (d, *J* = 8.4 Hz, 1H), 7.70 (s, 1H), 5.31 (dt, *J* = 4.8, 2.1 Hz, 1H), 5.23 (t, *J* = 3.0 Hz, 1H), 2.58 (dp, *J* = 15.7, 3.0 Hz, 1H), 2.34 (dt, *J* = 15.6, 2.7 Hz, 1H), 2.06 − 1.92 (m, 3H), 1.93 − 1.78 (m, 3H), 1.68 (dt, *J* = 13.4, 3.6 Hz, 1H), 1.61 − 1.55 (m, 3H), 1.52 (ddd, *J* = 13.3, 5.6, 2.8 Hz, 3H), 1.45 (ddd, *J* = 13.5, 10.1, 4.5 Hz, 2H), 1.34 (tdd, *J* = 12.1, 7.4, 2.4 Hz, 3H), 1.29 − 1.24 (m, 2H), 1.19 − 1.07 (m, 6H), 1.04 (s, 3H), 0.92 (d, *J* = 6.5 Hz, 3H), 0.86 (dd, *J* = 6.6, 2.7 Hz, 6H), 0.68 (s, 3H).

^{13}C NMR (150 MHz, $CDCl_3$) δ 163.1, 151.4, 147.6, 140.2, 137.9, 134.3, 124.2, 123.2, 123.1, 123.0, 114.8, 73.3, 56.9, 56.3, 49.9, 42.5, 39.9, 39.7, 37.1, 36.4, 36.3, 36.0, 33.5, 32.0, 32.0, 28.4, 28.2, 26.3, 24.4, 24.0, 23.0, 22.7, 20.9, 19.1, 18.9, 12.0.

HRMS (ESI) *m/z* calcd. for $C_{17}H_{22}N_2Na$ $[M+Na]^+$: 643.2870; found: 643.2901.

(3a*R*,3b*S*,6*S*,6*aS*,7*aR)*-2,2-Dimethyl-5-oxohexahydrofuro[2',3':4,5]furo[2,3-*d*][1,3]dioxol-6-yl 4-((*Z*)-2-(5-bromopyridin-2-yl)-2-cyanovinyl)benzoate (1gf)

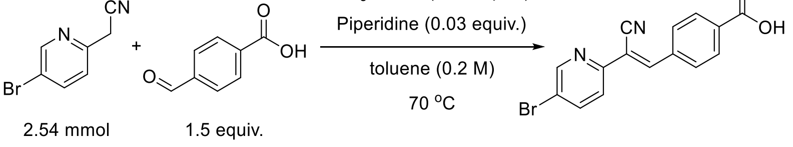

A mixture containing 2-(5-bromopyridin-2-yl)acetonitrile (0.5 g, 2.54 mmol, 1.0 equiv.), 4-formylbenzoic acid (0.57 g, 3.81 mmol, 1.5 equiv.), acetic acid (37.0 µL, 0.64 mmol, 0.25 equiv.), and piperidine (8.0 µL, 0.08 mmol, 0.03 equiv.) was prepared in anhydrous toluene (13.0 mL, 0.2 M)

in a flame-dried flask with stirring. The reaction was maintained at 70 °C until completion, followed by cooling and washing with 1 N HCl solution (10.0 mL) and extraction with ethyl acetate (10.0 mL × 3). The dried and concentrated organic phase provided the crude product.

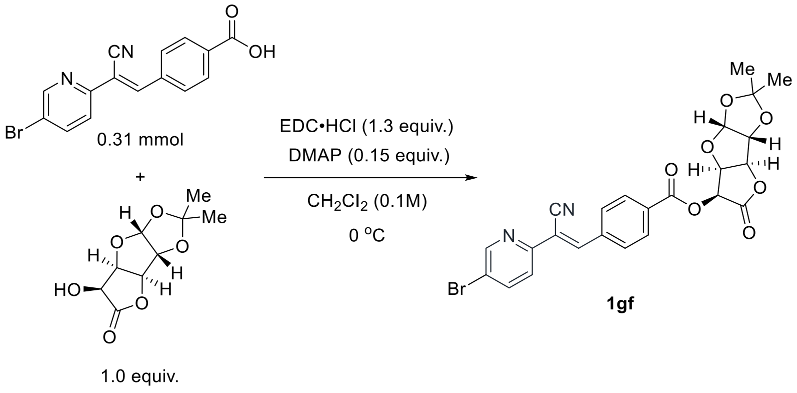

For the next reaction, (*Z*)-4-(2-(5-bromopyridin-2-yl)-2-cyanovinyl)benzoic acid (100.0 mg, 0.31 mmol, 1.0 equiv.) and derivative of D-glucuronic acid (70.0 mg, 0.31 mmol, 1.0 equiv.) were dissolved in CH_2Cl_2 (4.0 mL, 0.1 M). After cooling to 0 °C, 4-dimethylaminopyridine (DMAP) (6.0 mg, 0.05 mmol, 0.15 equiv.) and 1-ethyl-3-(3-dimethylaminopropyl) carbodiimide hydrochloride (EDC·HCl) (0.07 g, 0.4 mmol, 1.3 equiv.) were added in one portion. After stirring until completion, the reaction was quenched with water (5.0 mL) and extracted with dichloromethane (5.0 mL × 3). The dried and concentrated organic layer was purified by flash chromatography (petroleum ether/dichloromethane = 1/3) to yield a colorless oil (70.0 mg) in 42% yield.

^{1}H NMR (400 MHz, $CDCl_3$) δ 8.70 (s, 1H), 8.52 (s, 1H), 8.23 (d, *J* = 8.5 Hz, 2H), 8.05 (d, *J* = 8.5 Hz, 2H), 7.95 (dd, *J* = 8.4, 2.4 Hz, 1H), 7.68 (d, *J* = 8.4 Hz, 1H), 6.06 (d, *J* = 3.6 Hz, 1H), 5.75 (d, *J* = 4.4 Hz, 1H), 5.20 (dd, *J* = 4.4, 2.9 Hz, 1H), 4.97 (d, *J* = 2.9 Hz, 1H), 4.89 (d, *J* = 3.7 Hz, 1H), 1.52 (s, 3H), 1.36 (s, 3H).

^{13}C NMR (100 MHz, $CDCl_3$) δ 169.6, 164.6, 151.1, 149.1, 143.9, 140.1, 138.2, 131.0, 130.2, 130.0, 122.9, 113.8, 112.0, 107.2, 82.7, 82.4, 77.4, 77.3, 70.6, 27.1, 26.7.

HRMS (ESI) *m/z* calcd. for $C_{24}H_{20}BrN_2O_7$ $[M+H]^+$: 527.0448; found: 527.0467.

2.2.2 General Procedure F for synthesis of geminally disubstituted terminal alkenes

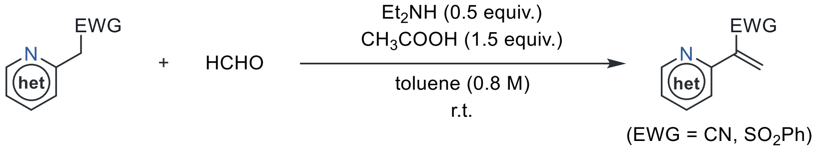

In a flame-dried, 25 mL round-bottomed flask equipped with a magnetic stirring bar, formaldehyde (0.35 mL, 37% aqueous solution, 2.0 equiv.) and acetic acid (1.5 equiv.) were combined, and toluene (0.8 M) was added to facilitate dissolution. Diethylamine (0.5 equiv.) was introduced to the solution, which was then stirred at ambient temperature for 1 hour. Subsequently, the corresponding *N*-heteroarenes (1.0 equiv.) were dissolved in toluene and added dropwise to the reaction mixture. Upon completion of the reaction, the solvent was removed under reduced pressure. The resulting crude residue was purified using flash chromatography on silica gel to yield the desired product.

2-(3,5-Dichloropyridin-2-yl)acrylonitrile (1ea)

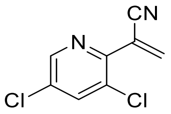

Compound **1ea** was synthesized in accordance with General Procedure F. The process resulted in the formation of a yellow oil (0.40 g) in 76% yield.

^{1}H NMR (400 MHz, $CDCl_3$) δ 8.52 (d, *J* = 2.1 Hz, 1H), 7.81 (d, *J* = 2.1 Hz, 1H), 6.75 (s, 1H), 6.56 (s, 1H).

^{13}C NMR (100 MHz, $CDCl_3$) δ 147.0, 146.6, 138.2, 138.1, 132.8, 130.4, 120.6, 116.6.

HRMS (ESI) *m/z* calcd. for $C_8H_5Cl_2N_2$ $[M+H]^+$: 198.9843; found: 198.9824.

Ethyl 2-(1-tosyl-1*H*-benzo[*d*]imidazol-2-yl)acrylate (1ec)

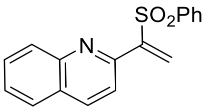

Compound **1ec** was synthesized in accordance with General Procedure F. The process resulted in the formation of a white solid (0.48 g) in 93% yield.

m.p.: 88.9 − 91.0 °C

^{1}H NMR (600 MHz, $CDCl_3$) δ 8.14 (d, *J* = 8.6 Hz, 1H), 7.95 (dd, *J* = 8.3, 1.1 Hz, 2H), 7.92 (d, *J* = 8.5 Hz, 1H), 7.88 (d, *J* = 8.6 Hz, 1H), 7.78 (d, *J* = 8.0 Hz, 1H), 7.68 (ddd, *J* = 8.4, 6.9, 1.3 Hz, 1H), 7.56 − 7.50 (m, 2H), 7.45 (t, *J* = 7.8 Hz, 2H), 6.93 (s, 1H), 6.71 (s, 1H).

^{13}C NMR (150 MHz, $CDCl_3$) δ 150.8, 150.5, 147.7, 140.0, 136.8, 133.5, 130.1, 129.6, 129.5, 129.0, 128.7, 127.7, 127.6, 127.5, 120.3.

HRMS (ESI) *m/z* calcd. for $C_{17}H_{14}NO_2S$ $[M+H]^+$: 296.0745; found: 296.0750.

2-Vinylquinoline (5a)

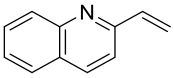

Compound **5a** was synthesized following General Procedure F. The process resulted in the formation of a yellow oil (2.3 g) in 36% yield.

^{1}H NMR (400 MHz, $CDCl_3$) δ 8.10 (d, J = 8.0 Hz, 1H), 8.05 (d, J = 8.0 Hz, 1H), 7.76 (d, J = 8.0 Hz, 1H), 7.70 – 7.66 (m, 1H), 7.59 (d, J = 8.0 Hz, 1H), 7.52 – 7.40 (m, 1H), 7.03 (dd, J = 16.0, 12.0 Hz, 1H), 6.26 (dd, J = 20.0, 0.8 Hz, 1H), 5.65 (dd, J = 8.0, 0.8 Hz, 1H).

^{13}C NMR (100 MHz, $CDCl_3$) δ 155.9, 147.6, 137.6, 136.7, 129.8, 129.1, 127.5, 126.5, 120.3, 118.3.

Spectroscopic data are in accordance with the literature[14].

7-Chloro-2-vinylquinoline (5b)

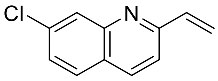

Compound **5b** was synthesized following General Procedure F. The process resulted in the formation of a white solid (2.1 g) in 65% yield.

^{1}H NMR (400 MHz, $CDCl_3$) δ 8.09 – 8.06 (m, 2H), 7.71 (d, J = 8.6 Hz, 1H), 7.58 (d, J = 8.5 Hz, 1H), 7.45 (dd, J = 8.7, 2.1 Hz, 1H), 7.00 (dd, J = 17.7, 10.9 Hz, 1H), 6.31 (dd, J = 17.6, 0.8 Hz, 1H), 5.69 (dd, J = 10.8, 0.8 Hz, 1H).

^{13}C NMR (100 MHz, $CDCl_3$) δ 157.0, 148.4, 137.5, 136.2, 135.4, 128.6, 128.3, 127.3, 125.8, 120.6, 118.7.

Spectroscopic data are in accordance with the literature[15].

2-Vinyl-1,5-naphthyridine (5e)

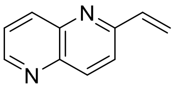

Compound **5e** was synthesized following General Procedure F. The process resulted in the formation of a yellow oil (0.48 g) in 60% yield.

^{1}H NMR (400 MHz, $CDCl_3$) δ 8.89 (dd, *J* = 4.1, 1.4 Hz, 1H), 8.33 (dd, *J* = 8.6, 2.6 Hz, 2H), 7.79 (d, *J* = 8.8 Hz, 1H), 7.59 (dd, *J* = 8.5, 4.2 Hz, 1H), 7.02 (dd, *J* = 17.7, 10.9 Hz, 1H), 6.36 – 6.26 (m, 1H), 5.69 (d, *J* = 10.9 Hz, 1H)。

^{13}C NMR (100 MHz, $CDCl_3$) δ 156.8, 150.8, 143.6, 143.5, 137.7, 137.3, 137.3, 124.6, 122.0, 121.2.

Spectroscopic data are in accordance with the literature[17].

1-Vinylisoquinoline (5f)

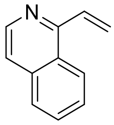

Compound **5f** was synthesized following General Procedure F. The process resulted in the formation of a colorless oil (0.44 g) in 73% yield.

^{1}H NMR (400 MHz, $CDCl_3$) δ 8.49 (d, J = 8.0 Hz, 1H), 8.21 (d, J = 8.0 Hz, 1H), 7.94 (d, J = 8.0 Hz, 1H), 7.79 – 7.73 (m, 1H), 7.64 – 7.61 (m, 1H), 7.58 (d, J = 8.0 Hz, 1H), 7.54 (d, J = 8.0 Hz, 1H), 6.49 (dd, J = 12.0, 4.0 Hz, 1H), 5.68 (dd, J = 12.0, 4.0 Hz, 1H).

^{13}C NMR (100 MHz, $CDCl_3$) δ 154.8, 142.3, 136.6, 132.2, 129.9, 127.2, 127.2, 126.4, 124.6, 121.7, 120.3.

Spectroscopic data are in accordance with the literature[14].

2-Vinylbenzo[*d*]oxazole (5m)

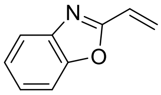

Compound **5m** was synthesized following General Procedure F. The process resulted in the formation of a yellow oil (0.33 g) in 42% yield.

^{1}H NMR (400 MHz, $CDCl_3$) δ 7.20 – 7.80 (m, 4H), 6.45 (dd, *J* = 1.2, 17.7 Hz, 1H), 6.74 (dd, *J* = 11.1, 17.7 Hz), 5.83 (dd, *J* = 1.2, 11.1 Hz, 1H).

^{13}C NMR (100 MHz, $CDCl_3$) δ 162.0, 150.3, 141.8, 125.4, 125.3, 124.5, 123.9, 120.1, 110.4.

Spectroscopic data are in accordance with the literature[21].

Ethyl 2-(1-tosyl-1*H*-benzo[*d*]imidazol-2-yl)acrylate (1eb)

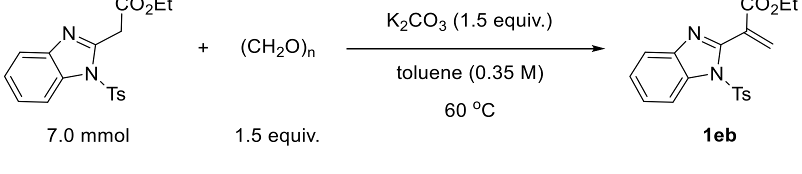

A flame-dried round-bottomed flask, equipped with a magnetic stirring bar, was charged with paraformaldehyde (0.95 g, 10.5 mmol, 1.5 equiv.), K_2CO_3 (1.45 g, 10.5 mmol, 1.5 equiv.), and *n*-Bu_4NI (0.13 g, 0.35 mmol, 0.05 equiv.) in a solution of the corresponding ester (2.51 g, 7.0 mmol, 1.0 equiv.) dissolved in 30.0 mL of toluene, all at ambient temperature. The reaction mixture was heated

to 60 °C and allowed to react for 12 hours. After cooling to ambient temperature, 15.0 mL of deionized water was added, and the resulting aqueous layer was extracted with ethyl acetate (20.0 mL × 3). The combined organic layers were dried over Na_2SO_4, concentrated under reduced pressure, and further purified using flash column chromatography on silica gel (petroleum ether/EtOAc = 5/1). This purification afforded Compound **1eb** as a yellow solid (1.21 g) in 46% yield.

m.p.: 178.4 − 179.6 °C

^{1}H NMR (400 MHz, $CDCl_3$) δ 7.91 (dd, *J* = 7.2, 1.4 Hz, 1H), 7.75 (d, *J* = 8.4 Hz, 2H), 7.72 (dd, *J* = 8.0, 1.3 Hz, 1H), 7.37 (pd, *J* = 7.4, 1.5 Hz, 2H), 7.24 (d, *J* = 8.4 Hz, 2H), 6.81 (s, 1H), 6.14 (s, 1H), 4.31 (q, *J* = 7.1 Hz, 2H), 2.35 (s, 3H), 1.29 (t, *J* = 7.1 Hz, 3H).

^{13}C NMR (100 MHz, $CDCl_3$) δ 164.5, 149.1, 146.2, 142.4, 134.9, 134.1, 132.9, 132.8, 130.2, 127.4, 125.8, 125.1, 120.8, 113.7, 61.9, 21.8, 14.2.

HRMS (ESI) *m/z* calcd. for $C_{19}H_{19}N_2O_4S$ $[M+H]^+$: 371.1060; found: 371.1063.

2.2.3 General Procedure G for synthesis of mono-substituted and 1,2-disubstituted alkenyl aza-heteroarenes

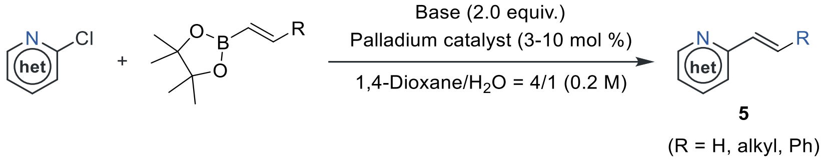

In a nitrogen-purged round-bottomed flask equipped with a magnetic stir bar, a mixture of 1-chlorinated heterocyclic compounds (1.0 equiv.), boronic ester (1.1 equiv.), and base (2.0 equiv.) is prepared in 1,4-dioxane/H_2O (4/1, 0.2 M). A palladium catalyst with ligand (3-10 mol %) is then added. The mixture is heated to 80 °C for 8 hours, and the progress of the reaction is monitored by Thin Layer Chromatography (TLC). Upon completion, the mixture is cooled to room temperature and diluted with water (10.0 mL). The mixture is then extracted with ethyl acetate (EtOAc) (20.0 mL × 4). The combined organic layers are washed with a saturated aqueous NH_4Cl solution (20.0 mL), dried over anhydrous Na_2SO_4, filtered, and concentrated under reduced pressure. Finally, the residue is purified using column chromatography to yield the desired alkene substrate.

2-(prop-1-en-2-yl)quinoline (1ed)

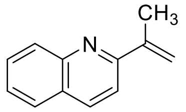

Compound **1ed** was synthesized following General Procedure G. The process resulted in the formation of a yellow oil (0.24 g) in 40% yield.

^{1}H NMR (400 MHz, $CDCl_3$) δ 8.12 − 7.48 (m, 6 H), 5.94 (s, 1 H), 5.50 (s, 1 H), 2.37 (s, 3 H).

^{13}C NMR (100 MHz, $CDCl_3$) δ 158.6, 147.9, 144.6, 136.2, 129.9, 129.6, 127.5, 127.4, 126.4, 118.4, 117.2, 20.8.

Spectroscopic data are in accordance with the literature[12].

2-(prop-1-en-2-yl)-1-tosyl-1H-benzo[d]imidazole (1ee)

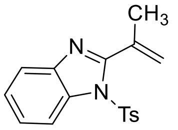

Compound **1ee** was synthesized following General Procedure G. The process resulted in the formation of a yellow oil (0.33 g) in 42% yield.

^{1}H NMR (400 MHz, $CDCl_3$) δ 7.91 (dd, *J* = 7.2, 1.4 Hz, 1H), 7.75 (d, *J* = 8.4 Hz, 2H), 7.72 (dd, *J* = 8.0, 1.3 Hz, 1H), 7.37 (pd, *J* = 7.4, 1.5 Hz, 2H), 7.24 (d, *J* = 8.2 Hz, 2H), 5.51 (s, 1H), 5.22 (t, *J* = 1.4 Hz, 1H), 2.33 (s, 3H), 2.17 (q, J = 0.7 Hz, 3H).

^{13}C NMR (100 MHz, $CDCl_3$) δ 145.1, 136.3, 135.7, 135.2, 129.9, 129.0, 126.9, 124.7, 124.0, 123.6, 123.5, 121.4, 113.8, 113.7, 23.1, 21.5

Spectroscopic data are in accordance with the literature[13].

2-Vinylquinoxaline (5c)

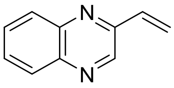

Compound **5c** was synthesized following General Procedure G. Palladium (II) acetate (4 mol %) and SPhos (8 mol %) were employed as catalysts and K_3PO_4 was utilized as base. The process resulted in the formation of a colorless oil (0.48 g) in 77% yield.

^{1}H NMR (400 MHz, $CDCl_3$) δ 8.98 (s, 1H), 8.07 – 8.03 (m, 2H), 7.77 – 7.68 (m, 2H), 7.02 (dd, J = 16.0, 12.0 Hz, 1H), 6.46 (dd, J = 16.0, 0.4 Hz, 1H), 5.77 (dd, J = 12.0, 0.4 Hz, 1H).

^{13}C NMR (100 MHz, $CDCl_3$) δ 150.5, 143.6, 142.2, 141.8, 134.9, 130.2, 129.5, 129.4, 129.2, 122.1.

Spectroscopic data are in accordance with the literature[14].

Ethyl 3-vinylquinoxaline-2-carboxylate (5d)

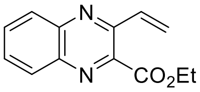

Compound **5d** was synthesized following General Procedure G. Tetrakis(triphenylphosphine) palladium ($Pd[P(C_6H_5)_3]_4$) (10 mol %) was utilized as the catalyst and Na_2CO_3 served as base. The process resulted in the formation of a colorless oil (0.75 g) in 45% yield.

^{1}H NMR (400 MHz, $CDCl_3$) δ 8.14 (ddd, *J* = 8.2, 1.6, 0.6 Hz, 1H), 8.11 – 8.06 (m, 1H), 7.81 (ddd, *J* = 8.4, 6.8, 1.6 Hz, 1H), 7.75 (ddd, *J* = 8.4, 6.8, 1.6 Hz, 1H), 7.45 (dd, *J* = 17.0, 10.8 Hz, 1H), 6.67 (dd, *J* = 17.0, 1.8 Hz, 1H), 5.74 (dd, *J* = 10.8, 1.8 Hz, 1H), 4.55 (q, *J* = 7.2 Hz, 2H), 1.48 (t, *J* = 7.1 Hz, 3H).

^{13}C NMR (100 MHz, $CDCl_3$) δ 165.8, 148.7, 143.8, 142.9, 140.5, 132.1, 131.9, 130.4, 129.8, 129.4, 123.7, 62.7, 14.4.

Spectroscopic data are in accordance with the literature[16].

7-((2-(Trimethylsilyl)ethoxy)methyl)-4-vinyl-7*H*-pyrrolo[2,3-*d*]pyrimidine (5g)

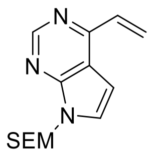

Compound **5g** was prepared following General Procedure G. Palladium (II) acetate (3 mol %) and SPhos (6 mol %) were employed as catalysts and K_3PO_4 was utilized as base. The process resulted in the formation of a yellow oil (1.81 g) in 78% yield.

^{1}H NMR (600 MHz, $CDCl_3$) δ 8.84 (s, 1H), 7.34 (d, *J* = 3.7 Hz, 1H), 7.10 (dd, *J* = 17.4, 10.8 Hz, 1H), 6.69 (d, *J* = 3.7 Hz, 1H), 6.63 (dd, *J* = 17.4, 1.5 Hz, 1H), 5.77 (dd, *J* = 10.9, 1.5 Hz, 1H), 5.63 (s, 2H), 3.53 – 3.48 (m, 2H), 0.94 – 0.85 (m, 2H), −0.09 (s, 9H).

^{13}C NMR (150 MHz, $CDCl_3$) δ 154.4, 152.6, 151.8, 133.5, 128.7, 123.3, 116.2, 100.2, 77.5, 77.2, 76.8, 72.9, 66.6, 17.8, −1.4.

HRMS (ESI) *m/z* calcd. for $C_{14}H_{22}N_3OSi$ $[M+H]^+$: 276.1527; found: 276.1542.

Methyl 6-vinylnicotinate (5h)

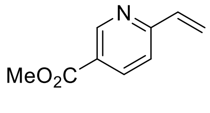

Compound **5h** was synthesized following General Procedure G. Palladium (II) acetate (4 mol %) and SPhos (8 mol %) were employed as catalysts and K_3PO_4 was utilized as base. The process resulted in the formation of a yellow oil (0.84 g) in 73% yield.

^{1}H NMR (400 MHz, $CDCl_3$) δ 9.15 (d, *J* = 2.2 Hz, 1H), 8.23 (dd, *J* = 8.2, 2.2 Hz, 1H), 7.39 (d, *J* = 8.2 Hz, 1H), 6.85 (dd, *J* = 17.4, 10.8 Hz, 1H), 6.33 (dd, *J* = 17.4, 1.2 Hz, 1H), 5.61 (dd, *J* = 10.8, 1.2 Hz, 1H), 3.93 (s, 3H).

^{13}**C NMR** (100 MHz, $CDCl_3$) δ 165.8, 159.3, 150.9, 137.8, 136.2, 124.6, 121.3, 121.2, 120.8, 52.5. Spectroscopic data are in accordance with the literature[18].

5-(Trifluoromethyl)-2-vinylpyridine (5i)

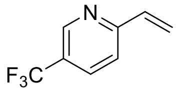

Compound **5i** was synthesized following General Procedure G. 1,1'-Bis(diphenylphosphino) ferrocene dichloropalladium (II) ($PdCl_2$(dppf)) (10 mol %) was utilized as the catalyst and CsF served as base. The process resulted in the formation of a yellow oil (0.33 g) in 31% yield.

^{1}**H NMR** (400 MHz, $CDCl_3$) δ 8.82 (br s, 1H), 7.87 (dd, J = 8.3, 2.3 Hz, 1H), 7.43 (d, J = 8.2 Hz, 1H), 6.86 (dd, J = 17.4, 10.8 Hz, 1H), 6.34 (d, J = 17.4 Hz, 1H), 5.63 (d, J = 10.8 Hz, 1H).

^{13}**C NMR** (100 MHz, $CDCl_3$) δ 159.1 (q, J = 1.5 Hz), 146.6 (q, J = 4.1 Hz), 135.9, 133.8 (q, J = 3.6 Hz), 125.2 (q, J = 33.0 Hz), 123.8 (q, J = 272.0 Hz), 121.3, 120.8.

^{19}**F NMR** (376 MHz, $CDCl_3$) δ – 62.33.

Spectroscopic data are in accordance with the literature[19].

5-Vinylpyrazine-2-carbonitrile (5j)

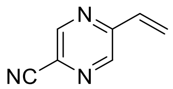

Compound **5j** was prepared following General Procedure G. 1,1'-Bis(diphenylphosphino) ferrocene dichloropalladium (II) ($PdCl_2$(dppf)) (10 mol %) was utilized as the catalyst and Cs_2CO_3 served as base. The process resulted in the formation of a colorless oil (0.22 g) in 44% yield.

^{1}**H NMR** (400 MHz, $CDCl_3$) δ 8.82 (d, J = 1.5 Hz, 1H), 8.67 (d, J = 1.5 Hz, 1H), 6.87 (dd, J = 17.4, 10.8 Hz, 1H), 6.55 (dd, J = 17.5, 0.9 Hz, 1H), 5.85 (dd, J = 10.9, 0.9 Hz, 1H).

^{13}**C NMR** (100 MHz, $CDCl_3$) δ 153.3, 147.8, 143.6, 132.4, 128.7, 125.5, 115.8.

HRMS (ESI) *m/z* calcd. for $C_7H_6N_3$ $[M+H]^+$: 132.0556; found: 132.0556.

Ethyl 2-vinylpyrimidine-5-carboxylate (5k)

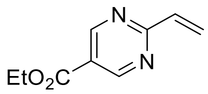

Compound **5k** was prepared following General Procedure G. 1,1'-Bis(diphenylphosphino) ferrocene-dichloropalladium(II) ($PdCl_2$(dppf)) (10 mol %) was used and $NaHCO_3$ served as base. The process resulted in the formation of a colorless oil (0.40 g) in 42% yield.

^{1}H NMR (600 MHz, $CDCl_3$) δ 9.22 (s, 2H), 6.95 (dd, *J* = 17.3, 10.5 Hz, 1H), 6.78 (dd, *J* = 17.2, 1.6 Hz, 1H), 5.88 (dd, *J* = 10.4, 1.6 Hz, 1H), 4.44 (q, *J* = 7.2 Hz, 2H), 1.42 (t, *J* = 7.2 Hz, 3H).
^{13}C NMR (150 MHz, $CDCl_3$) δ 167.0, 163.9, 160.7, 158.3, 136.0, 127.0, 122.0, 61.9, 14.4.
HRMS (ESI) *m/z* calcd. for $C_9H_{11}N_2O$ $[M+H]^+$: 179.0815; found: 179.0815.

2-(Methylthio)-4-vinylpyrimidine (5l)

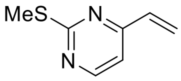

Compound **5l** was synthesized following General Procedure G. Palladium (II) acetate (4 mol %) and SPhos (8 mol %) were employed as catalysts and K_3PO_4 was utilized as base. The process resulted in the formation of a yellow oil (0.64 g) in 42% yield.
^{1}H NMR (400 MHz, $CDCl_3$) δ 3.39 (s, 3H), 5.89 (1H, d, *J* = 10.5 Hz), 6.65 (1H, d, *J* = 17.4 Hz), 6.86 (1H, dd, *J* = 10.5, 17.4 Hz), 7.49 (1H, d, *J* = 5.1 Hz), 8.85 (1H, d, *J* = 5.1 Hz).
^{13}C NMR (100 MHz, $CDCl_3$) δ 164.4, 159.0, 133.5, 127.5, 120.3, 39.1.
Spectroscopic data are in accordance with the literature[20].

5-(4-Fluorophenyl)-2-vinylthiazole-4-carbonitrile (5n)

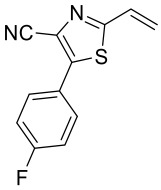

Compound **5n** was prepared following General Procedure G. Tetrakis(triphenylphosphine) palladium ($Pd[P(C_6H_5)_3]_4$) (5 mol %) was utilized as the catalyst and Cs_2CO_3 served as base. The process resulted in the formation of a white solid (0.63 g) in 65% yield.
m.p.: 62.7 – 64.5 °C
^{1}H NMR (400 MHz, $CDCl_3$) δ 8.13 (dd, *J* = 8.7, 5.4 Hz, 2H), 7.17 (t, *J* = 8.6 Hz, 2H), 6.91 (dd, *J* = 17.4, 10.9 Hz, 1H), 6.26 (d, *J* = 17.4 Hz, 1H), 5.77 (d, *J* = 10.9 Hz, 1H).
^{13}C NMR (100 MHz, $CDCl_3$) δ 169.9, 165.2, 162.7, 162.0, 130.3 (d, *J* = 8.7 Hz), 129.3, 128.4 (d, *J* = 3.3 Hz), 124.3, 116.3, 116.0, 113.6, 97.6.
^{19}F NMR (376 MHz, $CDCl_3$) δ −112.3.
HRMS (ESI) *m/z* calcd. for $C_{12}H_7FN_2S$ $[M+H]^+$: 231.0387; found: 231.0392.

(*E*)-2-(Hex-1-en-1-yl)quinoline (5o)

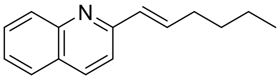

Compound **5o** was synthesized following General Procedure G. Palladium (II) acetate (4 mol %) and SPhos (8 mol %) were employed as catalysts and K_3PO_4 was utilized as base. The process resulted in the formation of a yellow oil (2.2 g) in 66% yield.

^{1}H NMR (400 MHz, $CDCl_3$) δ 8.06 (d, *J* = 8.7 Hz, 1H), 8.02 (dd, *J* = 8.4, 0.7 Hz, 1H), 7.75 (dd, *J* = 8.1, 1.2 Hz, 1H), 7.67 (ddd, *J* = 8.4, 6.9, 1.5 Hz, 1H), 7.52 (d, *J* = 8.6 Hz, 1H), 7.46 (ddd, *J* = 8.1, 6.9, 1.1 Hz, 1H), 6.83 (dt, *J* = 15.9, 6.7 Hz, 1H), 6.71 (dt, *J* = 15.9, 1.1 Hz, 1H), 2.38 – 2.30 (m, 2H), 1.59 – 1.50 (m, 2H), 1.42 (m, 2H), 0.95 (t, *J* = 7.3 Hz, 3H).

^{13}C NMR (100 MHz, $CDCl_3$) δ 156.5, 148.1, 138.0, 136.1, 131.0, 129.5, 129.1, 127.4, 127.1, 125.8, 118.7, 32.7, 31.0, 22.3, 13.9.

Spectroscopic data are in accordance with the literature[22].

(*E*)-2-(4-Chlorobut-1-en-1-yl)quinoxaline (5p)

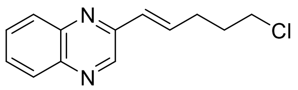

Compound **5p** was prepared following General Procedure G. Palladium (II) acetate (5 mol %) and PPh_3 (10 mol %) was utilized as the catalyst and CsF served as base. The process resulted in the formation of a yellow oil (0.38 g) in 58% yield.

^{1}H NMR (400 MHz, $CDCl_3$) δ 8.89 (s, 1H), 8.09 – 7.96 (m, 2H), 7.82 – 7.58 (m, 2H), 7.00 (dt, *J* = 15.9, 7.0 Hz, 1H), 6.75 (dt, *J* = 15.9, 1.3 Hz, 1H), 3.61 (t, *J* = 6.5 Hz, 2H), 2.57 – 2.49 (m, 2H), 2.04 (p, *J* = 6.6 Hz, 2H).

^{13}C NMR (100 MHz, $CDCl_3$) δ 150.5, 144.0, 142.3, 141.7, 138.2, 130.3, 129.3, 129.2, 129.0, 44.3, 31.5, 30.4.

HRMS (ESI) *m/z* calcd. for $C_{13}H_{14}ClN_2$ $[M+H]^+$: 233.0840; found: 233.0857.

(*E*)-2-(Prop-1-en-1-yl)benzo[*d*]oxazole (5q)

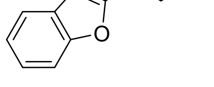

Compound **5q** was synthesized following General Procedure G. Palladium (II) acetate (4 mol %) and SPhos (8 mol %) were employed as catalysts and K_3PO_4 was utilized as base. The process resulted in the formation of a yellow oil (0.44 g) in 67% yield.

^{1}H NMR (400 MHz, $CDCl_3$) δ 7.70 – 7.65 (m, 1H), 7.50 – 7.45 (m, 1H), 7.32 – 7.27 (m, 2H), 7.09 – 6.99 (m, 1H), 6.49 – 6.43 (m, 1H), 2.02 (dd, J = 6.9, 1.6 Hz, 3H).

^{13}C NMR (100 MHz, $CDCl_3$) δ 162.4, 150.2, 141.8, 139.1, 124.7, 124.2, 119.7, 118.1, 110.1, 18.7.

Spectroscopic data are in accordance with the literature[23].

(*E*)-2-(5-Chloropent-1-en-1-yl)benzo[*d*]oxazole (5r)

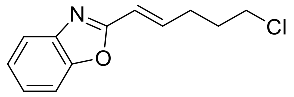

Compound **5r** was synthesized following General Procedure G. 1,1'-Bis(diphenylphosphino) ferrocene-dichloropalladium(II) ($PdCl_2$(dppf)) (5 mol %) and triphenylphosphine (10 mol %) were employed as catalysts and Ag_2CO_3 was utilized as base. The process resulted in the formation of a yellow oil (0.28 g) in 49% yield.

^{1}H NMR (400 MHz, $CDCl_3$) δ 7.63 – 7.70 (m, 1H), 7.49 – 7.55 (m, 1H) 7.21 – 7.29 (m, 2H), 6.94 (dt, J = 15.9, J = 7.1 Hz, 1H), 6.46 (dt, J = 15.9, J = 1.5, 1H), 3.55 (t, J = 6.4, 2H), 2.46 – 2.51 (m, 2H) 1.97 – 2.02 (m, 2H).

^{13}C NMR (100 MHz, $CDCl_3$) δ 162.4, 150.2, 141.8, 139.1, 124.7, 124.2, 119.7, 118.1, 110.1, 18.7.

Spectroscopic data are in accordance with the literature[24].

(*E*)-2-Styryl-1-tosyl-1*H*-benzo[d]imidazole (5s)

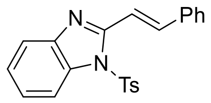

Compound **5s** was synthesized following General Procedure G. Palladium (II) acetate (4 mol %) and SPhos (8 mol %) were employed as catalysts and K_3PO_4 was utilized as base. The process resulted in the formation of a yellow oil (0.33 g) in 72% yield.

^{1}H NMR (400 MHz, $CDCl_3$) δ 8.09 − 8.07 (m, 1H), 7.93 (s, 2H), 7.78 (d, J = 8.4 Hz, 2H), 7.70 − 7.65 (m, 3H), 7.46 − 7.39 (m, 3H), 7.36 − 7.34 (m, 2H), 7.22 (d, J = 8.4 Hz, 2H), 2.33 (s, 3H).

^{13}C NMR (100 MHz, $CDCl_3$) δ 151.2, 145.9, 142.7, 139.8, 135.8, 135.3, 133.2, 130.2,129.6, 129.0, 127.8, 126.8, 125.3, 125.1, 119.9, 114.4, 114.0, 21.6.

Spectroscopic data are in accordance with the literature[25].

(*E*)-2-(4-Bromostyryl)pyridine (5t)

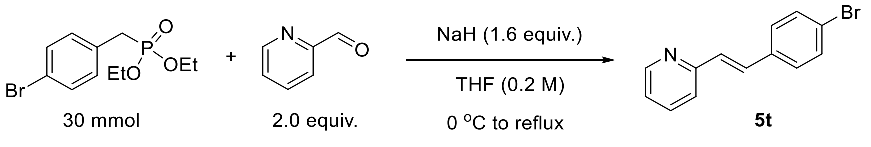

To a solution of diethyl (4-bromobenzyl)phosphonate (9.21 g, 30 mmol) in dry tetrahydrofuran (THF) (150 mL, 0.2 M) at 0 °C, sodium hydride (NaH, 60% *w/w* in oil) (1.92 g, 48 mmol, 1.6 equiv.) was added portionwise under a nitrogen (N_2) atmosphere. The mixture was stirred at 0 °C for 30 minutes. Subsequently, picolinaldehyde (6.42 g, 60 mmol, 2.0 equiv.) was added at the same temperature, and

the reaction mixture was then heated to reflux for 12 hours. After completion, the reaction was allowed to cool to room temperature and was quenched with water (30.0 mL). The resultant mixture was extracted four times with ethyl acetate (EtOAc) (30.0 mL × 4). The combined organic layers were washed with saturated aqueous sodium chloride (NaCl) solution (20.0 mL), dried over anhydrous sodium sulfate (Na_2SO_4), filtered, and the solvent was removed under reduced pressure. The crude product was purified by column chromatography using a petroleum ether/EtOAc mixture (10:1 as eluent), yielding compound **5t** as a white solid (7.8 g, 89% yield).

^{1}H NMR (400 MHz, $CDCl_3$) δ 8.60 (d, *J* = 4.5 Hz, 1H), 7.66 (t, *J* = 7.7 Hz, 1H), 7.57 (d, *J* = 16.1 Hz, 1H), 7.49 (d, *J* = 8.4 Hz, 2H), 7.43 (d, *J* = 8.5 Hz, 2H), 7.36 (d, *J* = 7.8 Hz, 1H), 7.19 – 7.10 (m, 2H).

^{13}C NMR (100 MHz, $CDCl_3$) δ 155.3, 149.8, 136.8, 135.7, 132.0, 131.6, 128.7, 128.6, 122.4, 122.4, 122.3.

Spectroscopic data are in accordance with the literature[26].

9-((3a*R*,4*R*,6*R*,6a*R*)-6-(((*tert*-Butyldimethylsilyl)oxy)methyl)-2,2-dimethyltetrahydrofuro[3,4-*d*][1,3]dioxol-4-yl)-6-vinyl-9*H*-purine (5u)

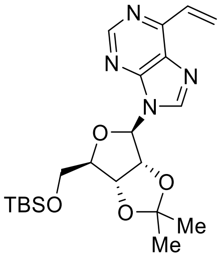

Compound **5u** was prepared following General Procedure G. Palladium (II) acetate (3 mol %) and SPhos (6 mol %) was utilized as the catalyst and K_3PO_4 served as base. The process resulted in the formation of a yellow oil (0.73 g) in 73% yield.

^{1}H NMR (600 MHz, $CDCl_3$) δ 8.91 (s, 1H), 8.29 (s, 1H), 7.36 – 7.22 (m, 1H), 7.00 (dd, *J* = 17.5, 1.6 Hz, 1H), 6.22 (d, *J* = 2.6 Hz, 1H), 5.93 (dd, *J* = 10.9, 1.7 Hz, 1H), 5.28 (dd, *J* = 6.1, 2.5 Hz, 1H), 4.93 (dd, *J* = 6.1, 2.4 Hz, 1H), 4.44 (td, *J* = 3.7, 2.3 Hz, 1H), 3.86 (dd, *J* = 11.2, 3.6 Hz, 1H), 3.75 (dd, *J* = 11.2, 4.0 Hz, 1H), 1.62 (s, 3H), 1.39 (s, 3H), 0.79 (s, 9H), −0.03 (d, *J* = 2.5 Hz, 6H).

^{13}C NMR (150 MHz, $CDCl_3$) δ 153.7, 152.6, 151.5, 143.3, 131.9, 131.8, 126.5, 114.3, 91.9, 87.6, 85.1, 81.6, 63.7, 27.3, 25.9, 25.5, 18.4, −5.4, −5.5.

HRMS (ESI) *m/z* calcd. for $C_{21}H_{33}N_4O_4Si$ $[M+H]^+$: 433.2266; found: 433.2282.

***N*-(5,6-Dimethoxypyrimidin-4-yl)-2-vinylbenzo[*d*]thiazole-5-sulfonamide (5v)**

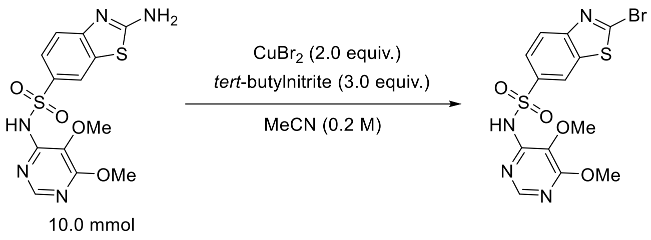

To a solution of 4-((2,3-dimethoxybenzyl)sulfonyl)aniline (10.0 mmol, 3.1 g, 1.0 equiv.) in acetonitrile (50.0 mL, 0.2 M), copper(II) bromide (20 mmol, 4.5 g, 2.0 equiv.) and *tert*-butylnitrite (30.0 mmol, 3.1 g, 3.0 equiv.) were added. The reaction mixture was stirred at 60 °C for 3 hours. Upon completion as indicated by TLC, the mixture was cooled to room temperature and diluted with deionized water (30.0 mL). The resulting mixture was extracted with ethyl acetate (3 × 30.0 mL). The combined organic layers were dried over anhydrous sodium sulfate and concentrated under reduced pressure. This intermediate was used directly in the subsequent step without further purification.

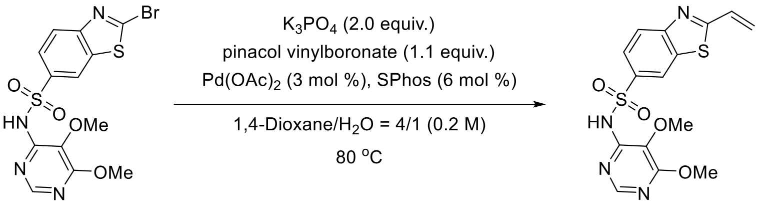

Under a nitrogen atmosphere, a round-bottom flask equipped with a magnetic stirring bar was charged with 2-bromo-*N*-(5,6-dimethoxypyrimidin-4-yl)benzo[*d*]thiazole-6-sulfonamide (0.69 g, 1.6 mmol, 1.0 equiv.), pinacol vinylboronate (0.27 g, 1.7 mmol, 1.1 equiv.), and K_3PO_4 (0.68 g, 3.2 mmol, 2.0 equiv.) in a 1,4-dioxane/H_2O mixture (4:1, *v*/*v*, 8 mL). Palladium(II) acetate (11 mg, 0.05 mmol, 3 mol %) and SPhos (40 mg, 0.1 mmol, 6 mol %) were added subsequently. The reaction mixture was heated at 80 °C for 8 hours, with the progress monitored by thin-layer chromatography (TLC). Upon completion, the reaction was cooled to room temperature and quenched with deionized water (10 mL). The aqueous mixture was extracted with ethyl acetate (4 × 20 mL). The combined organic extracts were washed with saturated aqueous NH_4Cl (20 mL), dried over anhydrous Na_2SO_4, filtered, and the solvent removed under reduced pressure. The crude product was purified by column chromatography (silica gel, eluting with a gradient of petroleum ether/ethyl acetate from 4/1 to 3/1) to afford 5**v** as a yellow solid (0.34 g, 57% yield over two steps).

m.p.: 148.0 – 153.6 °C

^{1}H NMR (400 MHz, $CDCl_3$) δ 8.77 − 8.68 (m, 1H), 8.19 – 8.14 (m, 2H), 8.06 (d, *J* = 8.1 Hz, 1H), 7.05 (dd, *J* = 17.5, 10.8 Hz, 1H), 6.29 (d, *J* = 17.5 Hz, 1H), 5.88 (d, *J* = 10.8 Hz, 1H), 3.97 (s, 3H), 3.87 (s, 3H).

^{13}C NMR (100 MHz, $CDCl_3$) δ 171.6, 161.0, 156.6, 151.0, 149.6, 136.3, 134.4, 131.0, 126.7, 126.0, 125.4, 123.5, 123.4, 60.7, 54.3.

HRMS (ESI) *m/z* calcd. for $C_{15}H_{15}N_4O_4S_2$ $[M+H]^+$: 379.0529; found: 379.0542.

2.3. Enantioselective epoxidation of alkenyl aza-heteroarenes

2.3.1 General Procedure for the asymmetric epoxidation of trisubstituted alkenyl aza-heteroarenes

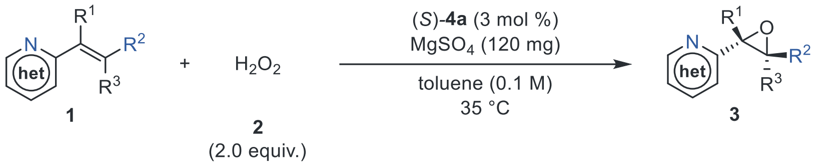

A mixture of alkenyl aza-heteroarenes (0.1 mmol), (*S*)-**4a** (2 mg, 0.03 mmol), and $MgSO_4$ (120 mg) was placed in a 4 mL vial with a magnetic stir bar. Freshly distilled toluene (1.0 mL) was added, followed by a dropwise addition of H_2O_2 (30% in H_2O *w*/*w*, 16 μL, 0.2 mmol). The mixture was stirred at 35 °C and its progress monitored via TLC. Once the reaction was deemed complete, the solution was filtered through a short celite pad. The combined organic layer was concentrated under reduced pressure and further purified via flash column chromatography on silica gel to isolate the desired products.

(2*R*,3*S*)-3-(4-Bromophenyl)-2-(pyridin-2-yl)oxirane-2-carbonitrile (3aa)

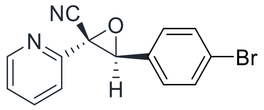

Compound **3aa** was synthesized according to the General Procedure and subsequently purified using flash column chromatography (petroleum ether/EtOAc = 10/1), resulting in a colorless oil with 93% yield (28 mg) on a 0.1 mmol scale.

^{1}H NMR (400 MHz, $CDCl_3$) δ 8.69 (dt, *J* = 4.7, 1.4 Hz, 1H), 7.83 (td, *J* = 7.8, 1.7 Hz, 1H), 7.70 – 7.55 (m, 1H), 7.59 (d, *J* = 8.5 Hz, 2H), 7.47 – 7.33 (m, 1H), 7.38 (d, *J* = 8.5 Hz, 2H), 4.56 (s, 1H).

^{13}C NMR (100 MHz, $CDCl_3$) δ 150.7, 150.2, 137.7, 132.0, 130.7, 128.3, 124.9, 124.3, 120.6, 115.3, 66.6, 58.2.

HRMS (ESI) *m/z* calcd. for $C_{14}H_{10}BrN_2O$ $[M+H]^+$: 300.9971; found: 300.9971.

$[\alpha]_D^{20}$ = −232.1 (*c* 1.0, $CHCl_3$)

The enantiomeric excess was determined by chiral HPLC analysis using a Daicel Chiralpak IA column, e.e. = 97% (*n*-hexane/ethanol = 70/30, flow rate = 1.0 mL/min, λ = 254 nm, T = 20 °C, t_r (major) = 8.556 min, t_r (minor) = 6.887 min).

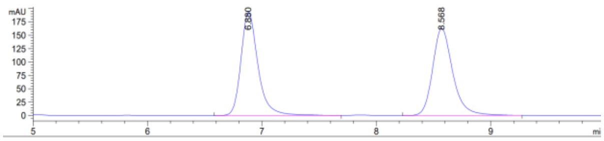

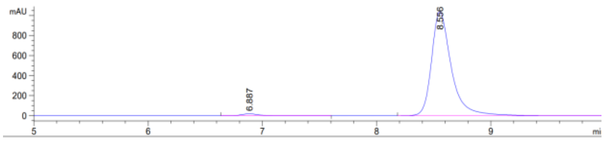

Signal 2: DAD1 B, Sig=254,4 Ref=off

Peak #	RetTime [min]	Type	Width [min]	Area [mAU*s]	Height [mAU]	Area %
1	6.880	BB	0.1574	2011.65601	193.27002	50.2104
2	8.568	BB	0.1869	1994.79700	162.80423	49.7896

Signal 2: DAD1 B, Sig=254,4 Ref=off

Peak #	RetTime [min]	Type	Width [min]	Area [mAU*s]	Height [mAU]	Area %
1	6.887	BB	0.1425	181.78635	19.19275	1.4364
2	8.556	BBA	0.1821	1.24741e4	1038.56750	98.5636

Supplementary Figure 1. HPLC spectra of compound **3aa**

(2*R*,3*S*)-3-(4-Bromophenyl)-2-(pyridin-4-yl)oxirane-2-carbonitrile (3ab)

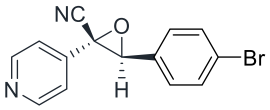

Compound **3ab** was synthesized following the General Procedure and subsequently purified using flash column chromatography (petroleum ether/EtOAc = 10/1), resulting in a white solid (27 mg) at 88% yield on a 0.1 mmol scale.

m.p.: 144.3 − 145.5 °C

^{1}H NMR (600 MHz, $CDCl_3$) δ 8.77 − 8.70 (m, 2H), 7.61 (d, *J* = 8.5 Hz, 2H), 7.46 − 7.41 (m, 2H), 7.35 (d, *J* = 8.5 Hz, 2H), 4.16 (s, 1H).

^{13}C NMR (150 MHz, $CDCl_3$) δ 150.8, 141.0, 132.2, 130.1, 128.2, 124.7, 119.6, 114.6, 68.5, 56.6.

HRMS (ESI) *m/z* calcd. for $C_{14}H_{10}BrN_2O$ $[M+H]^+$: 300.9971; found: 300.9977.

$[\alpha]_D^{20}$ = −110.4 (*c* 1.0, $CHCl_3$)

The enantiomeric excess was determined by chiral HPLC analysis using a Daicel Chiralpak IC column, e.e. = 34% (*n*-hexane/ethanol = 80/20, flow rate = 1.0 mL/min, λ = 254 nm, T = 20 °C, t_r (major) = 10.628 min, t_r (minor) = 9.587 min).

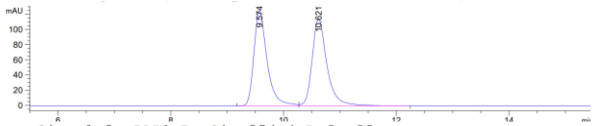

Signal 2: DAD1 B, Sig=254,4 Ref=off

Peak #	RetTime [min]	Type	Width [min]	Area [mAU*s]	Height [mAU]	Area %
1	9.574	BV	0.2469	2054.88013	125.56735	49.4597
2	10.621	VB	0.2736	2099.77197	114.67368	50.5403

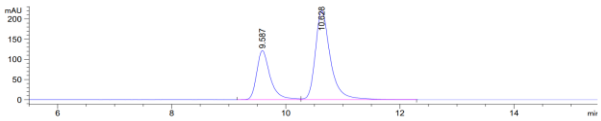

Signal 2: DAD1 B, Sig=254,4 Ref=off

Peak #	RetTime [min]	Type	Width [min]	Area [mAU*s]	Height [mAU]	Area %
1	9.587	BV	0.2456	1995.53113	121.51772	33.1257
2	10.628	VB	0.2755	4028.58057	220.09717	66.8743

Supplementary Figure 2. HPLC spectra of compound **3ab**

(2*R*,3*S*)-3-(4-Bromophenyl)-2-(pyridin-4-yl)oxirane-2-carbonitrile (3ac)

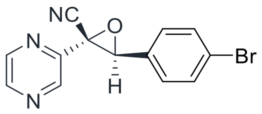

Compound **3ac** was prepared according to the General Procedure and subsequently purified using flash column chromatography (petroleum ether/EtOAc = 10/1), resulting in a yellow solid (19 mg) at 63% yield on a 0.1 mmol scale.

m.p.: 114.4 − 116.0 °C

^{1}H NMR (600 MHz, $CDCl_3$) δ 8.93 (s, 1H), 8.74 (s, 1H), 8.68 (s, 1H), 7.62 (d, *J* = 8.5 Hz, 2H), 7.39 (d, *J* = 8.5 Hz, 2H), 4.62 (s, 1H).

^{13}C NMR (150 MHz, $CDCl_3$) δ 146.7, 146.0, 144.5, 142.2, 132.1, 130.0, 128.2, 124.6, 114.2, 66.7, 56.6.

HRMS (ESI) *m/z* calcd. for $C_{13}H_9BrN_3O$ $[M+H]^+$: 301.9924; found: 301.9925.

$[\alpha]_D^{20}$ = −427.0 (*c* 1.0, $CHCl_3$)

The enantiomeric excess was determined by chiral HPLC analysis using a Daicel Chiralpak IB column, e.e. = 99% (*n*-hexane/ethanol = 70/30, flow rate = 1.0 mL/min, λ = 254 nm, T = 20 °C, t_r (major) = 7.274 min, t_r (minor) = 8.019 min).

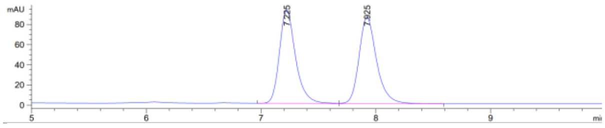

Signal 2: DAD1 B, Sig=254,4 Ref=off

Peak #	RetTime [min]	Type	Width [min]	Area [mAU*s]	Height [mAU]	Area %
1	7.225	BV	0.1479	908.83801	93.08624	49.9154
2	7.925	VB	0.1622	911.91833	85.66868	50.0846

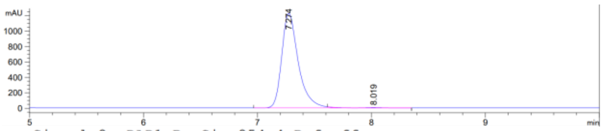

Signal 2: DAD1 B, Sig=254,4 Ref=off

Peak #	RetTime [min]	Type	Width [min]	Area [mAU*s]	Height [mAU]	Area %
1	7.274	BV R	0.1519	1.23982e4	1227.26746	99.5265
2	8.019	VB E	0.1796	58.98272	4.72752	0.4735

Supplementary Figure 3. HPLC spectra of compound **3ac**

(2*S*,3*S*)-3-(4-Bromophenyl)-2-(5-bromopyrimidin-2-yl)oxirane-2-carbonitrile (3ad)

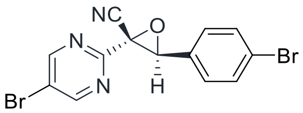

Compound **3ad** was synthesized following the General Procedure and subsequently purified using flash column chromatography (petroleum ether/EtOAc = 10/1), resulting in a white solid (34 mg) at 90% yield on a 0.1 mmol scale.

m.p.: 167.6 − 169.0 °C

^{1}H NMR (600 MHz, $CDCl_3$) δ 8.89 (s, 2H), 7.61 (d, *J* = 8.5 Hz, 2H), 7.39 (d, *J* = 8.5 Hz, 2H), 4.80 (s, 1H).

^{13}C NMR (150 MHz, $CDCl_3$) δ 158.9, 158.5, 132.2, 130.2, 128.5, 124.6, 121.5, 114.3, 66.2, 57.9.

HRMS (ESI) *m/z* calcd. for $C_{13}H_8Br_2N_3O$ $[M+H]^+$: 379.0929; found: 379.0924.

$[\alpha]_D^{20}$ = −176.0 (*c* 1.0, $CHCl_3$)

The enantiomeric excess was determined by chiral HPLC analysis using a Daicel Chiralpak IF column, e.e. = 99% (*n*-hexane/ethanol = 70/30, flow rate = 1.0 mL/min, λ = 254 nm, T = 20 °C, t_r (major) = 16.986 min, t_r (minor) = 11.495 min).

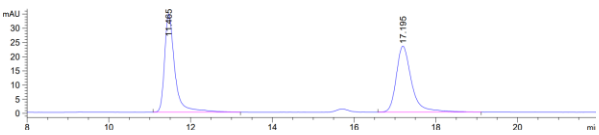

Signal 2: DAD1 B, Sig=254,4 Ref=off

Peak #	RetTime [min]	Type	Width [min]	Area [mAU*s]	Height [mAU]	Area %
1	11.465	BB	0.2586	601.63995	34.65165	49.9762
2	17.195	BB	0.3918	602.21252	23.28256	50.0238

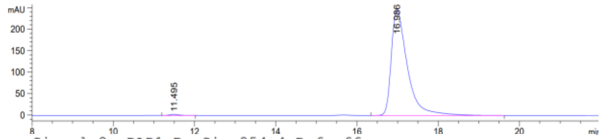

Signal 2: DAD1 B, Sig=254,4 Ref=off

Peak #	RetTime [min]	Type	Width [min]	Area [mAU*s]	Height [mAU]	Area %
1	11.495	BB	0.2346	38.93938	2.43464	0.5708
2	16.986	BB	0.4091	6783.42188	246.49344	99.4292

Supplementary Figure 4. HPLC spectra of compound **3ad**

(2*R*,3*S*)-3-(4-Bromophenyl)-2-(6-chloropyridazin-3-yl)oxirane-2-carbonitrile (3ae)

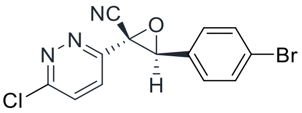

Compound **3ae** was synthesized following the General Procedure and subsequently purified using flash column chromatography (*n*-hexane/dichloromethane = 1/1), resulting in a white solid (33 mg) at 97% yield on a 0.1 mmol scale.

m.p.: 213.2 − 216.4 °C

^{1}H NMR (400 MHz, $CDCl_3$) δ 7.68 (d, *J* = 3.6 Hz, 2H), 7.63 (d, *J* = 8.5 Hz, 2H), 7.39 (d, *J* = 8.5 Hz, 2H), 4.67 (s, 1H).

^{13}C NMR (100 MHz, $CDCl_3$) δ 158.3, 153.8, 132.3, 129.8, 129.4, 128.4, 126.2, 124.9, 113.9, 66.6, 56.6.

HRMS (ESI) *m/z* calcd. for $C_{13}H_7BrN_3NaO$ $[M+Na]^+$: 357.9353; found: 357.9352.

$[\alpha]_D^{20}$ = −62.8 (*c* 1.0, $CHCl_3$)

The enantiomeric excess was determined by chiral HPLC analysis using a Daicel Chiralpak IA column, e.e. = 98% (*n*-hexane/ethanol = 70/30, flow rate = 1.0 mL/min, λ = 254 nm, T = 20 ºC, t_r (major) = 19.670 min, t_r (minor) = 16.385 min).

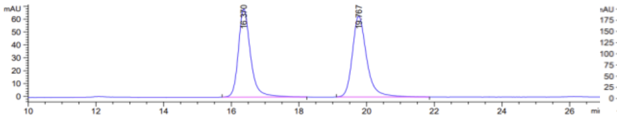

Signal 2: DAD1 B, Sig=254,4 Ref=off

Peak #	RetTime [min]	Type	Width [min]	Area [mAU*s]	Height [mAU]	Area %
1	16.370	BB	0.3778	1709.41614	68.33911	48.0932
2	19.767	BB	0.4433	1844.96790	62.70675	51.9068

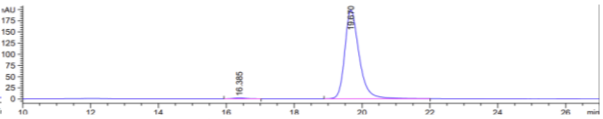

Signal 2: DAD1 B, Sig=254,4 Ref=off

Peak #	RetTime [min]	Type	Width [min]	Area [mAU*s]	Height [mAU]	Area %
1	16.385	BB	0.3198	38.14240	1.64913	0.6528
2	19.670	BB	0.4461	5804.41357	197.92393	99.3472

Supplementary Figure 5. HPLC spectra of compound **3ae**

(2*S*,3*S*)-3-(4-Bromophenyl)-2-(4,6-dimethoxy-1,3,5-triazin-2-yl)oxirane-2-carbonitrile (3af)

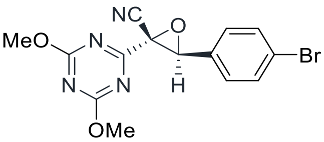

Compound **3af** was synthesized following the General Procedure and subsequently purified using flash column chromatography (petroleum ether/EtOAc = 10/1), resulting in a white solid (32 mg) at 89% yield on a 0.1 mmol scale.

m.p.: 165.3 − 179.7 °C

^{1}H NMR (400 MHz, $CDCl_3$) δ 7.60 (d, *J* = 8.2 Hz, 2H), 7.36 (d, *J* = 8.2 Hz, 2H), 4.76 (s, 1H), 4.11 (s, 6H).

^{13}C NMR (100 MHz, $CDCl_3$) δ 173.0, 172.1, 132.2, 129.8, 128.5, 124.7, 113.5, 65.6, 57.2, 56.1.

HRMS (ESI) *m/z* calcd. for $C_{14}H_{11}BrN_3NaO_3$ $[M+Na]^+$: 384.9907; found: 384.9908.

$[\alpha]_D^{20}$ = −19.7 (*c* 1.0, $CHCl_3$)

The enantiomeric excess was determined by chiral HPLC analysis using a Daicel Chiralpak IB column, e.e. = 98% (*n*-hexane/ethanol = 90/10, flow rate = 1.0 mL/min, λ = 250 nm, T = 20 °C, t_r (major) = 12.722 min, t_r (minor) = 13.943 min).

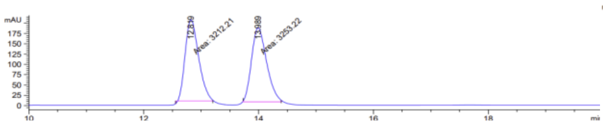

Signal 1: DAD1 A, Sig=250,4 Ref=off

Peak #	RetTime [min]	Type	Width [min]	Area [mAU*s]	Height [mAU]	Area %
1	12.819	MM	0.2723	3212.21289	196.60542	49.6829
2	13.989	MM	0.3028	3253.22217	179.08829	50.3171

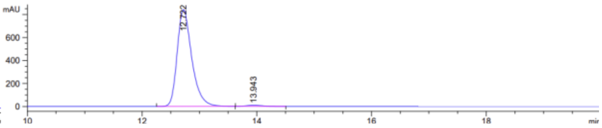

Signal 1: DAD1 A, Sig=250,4 Ref=off

Peak #	RetTime [min]	Type	Width [min]	Area [mAU*s]	Height [mAU]	Area %
1	12.722	BB	0.2676	1.48624e4	843.38495	98.9325
2	13.943	BB	0.2857	160.37120	8.67283	1.0675

Supplementary Figure 6. HPLC spectra of compound **3af**

(2*R*,3*S*)-3-(4-Bromophenyl)-2-(quinolin-2-yl)oxirane-2-carbonitrile (3ag)

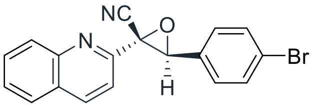

Compound **3ag** was synthesized following the General Procedure and subsequently purified using flash column chromatography (petroleum ether/EtOAc = 10/1), resulting in a white solid (32 mg) at 91% yield on a 0.1 mmol scale.

m.p.: 165.3 − 179.7 °C

^{1}H NMR (600 MHz, $CDCl_3$) δ 8.30 (d, *J* = 8.5 Hz, 1H), 8.17 (d, *J* = 8.5 Hz, 1H), 7.88 (dd, *J* = 8.2, 1.3 Hz, 1H), 7.80 (ddd, *J* = 8.4, 6.8, 1.4 Hz, 1H), 7.68 − 7.59 (m, 4H), 7.42 (d, *J* = 8.4 Hz, 2H), 4.62 (s, 1H).

^{13}C NMR (150 MHz, $CDCl_3$) δ 150.8, 147.9, 138.2, 132.1, 130.8, 130.8, 129.8, 128.5, 128.4, 128.0, 127.8, 124.3, 116.8, 115.3, 66.8, 58.9.

HRMS (ESI) *m/z* calcd. for $C_{19}H_{22}NaO_3$ $[M+Na]^+$: 351.0128; found: 351.0123.

$[\alpha]_D^{20}$ = −150.0 (*c* 1.0, $CHCl_3$)

The enantiomeric excess was determined by chiral HPLC analysis using a Daicel Chiralpak IJ column, e.e. = 98% (*n*-hexane/ethanol = 70/30, flow rate = 1.0 mL/min, λ = 254 nm, T = 20 °C, t_r (major) = 17.387 min, t_r (minor) = 12.648 min).

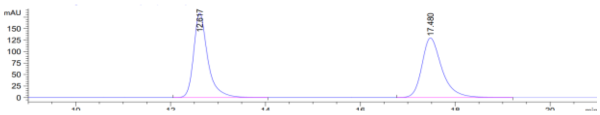

Signal 2: DAD1 B, Sig=254,4 Ref=off

Peak #	RetTime [min]	Type	Width [min]	Area [mAU*s]	Height [mAU]	Area %
1	12.617	BB	0.3084	3784.38086	183.63527	49.9827
2	17.480	BB	0.4375	3786.99463	129.39134	50.0173

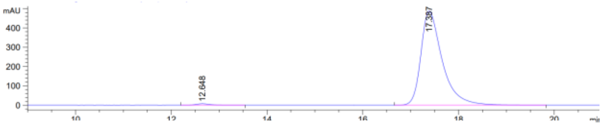

Signal 2: DAD1 B, Sig=254,4 Ref=off

Peak #	RetTime [min]	Type	Width [min]	Area [mAU*s]	Height [mAU]	Area %
1	12.648	BB	0.3098	149.72745	7.28501	0.9850
2	17.387	BB	0.4623	1.50505e4	487.35162	99.0150

Supplementary Figure 7. HPLC spectra of compound **3ag**

(2*R*,3*S*)-3-(3-Bromophenyl)-2-(isoquinolin-1-yl)oxirane-2-carbonitrile (3ah)

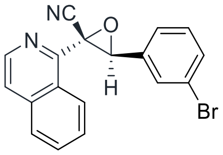

Compound **3ah** was synthesized following the General Procedure and subsequently purified using flash column chromatography (petroleum ether/EtOAc = 10/1), resulting in a white solid (31 mg) at 87% yield on a 0.1 mmol scale.

m.p.: 84.6 − 88.7 °C

^{1}H NMR (400 MHz, $CDCl_3$) δ 8.57 (d, *J* = 5.7 Hz, 1H), 8.52 − 8.41 (m, 1H), 7.95 (dd, *J* = 7.2, 2.4 Hz, 1H), 7.88 − 7.73 (m, 4H), 7.61 (ddd, *J* = 7.9, 6.6, 1.7 Hz, 2H), 7.40 (t, *J* = 7.9 Hz, 1H), 4.72 (s, 1H).

^{13}C NMR (100 MHz, $CDCl_3$) δ 149.7, 142.0, 136.9, 134.1, 133.1, 131.2, 130.5, 130.1, 129.0, 127.8, 126.6, 125.3, 124.3, 123.2, 123.0, 115.8, 63.4, 58.1.

HRMS (ESI) *m/z* calcd. for $C_{18}H_{11}BrN_2NaO$ $[M+Na]^+$: 372.9947; found: 372.9950.

$[\alpha]_D^{20}$ = −174.1 (*c* 1.0, $CHCl_3$)

The enantiomeric excess was determined by chiral HPLC analysis using a Daicel Chiralpak IJ column, e.e. = 94% (*n*-hexane/ethanol = 80/20, flow rate = 1.0 mL/min, λ = 254 nm, T = 20 °C, t_r (major) = 14.539 min, t_r (minor) = 12.182 min).

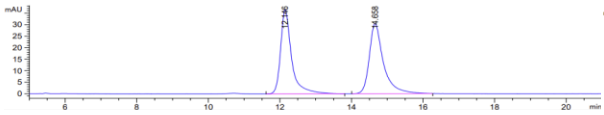

Signal 2: DAD1 B, Sig=254,4 Ref=off

Peak #	RetTime [min]	Type	Width [min]	Area [mAU*s]	Height [mAU]	Area %
1	12.146	BB	0.3173	786.48584	36.51925	48.5342
2	14.658	BB	0.4020	833.99164	30.40689	51.4658

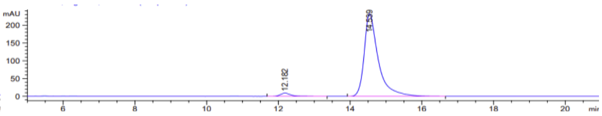

Signal 2: DAD1 B, Sig=254,4 Ref=off

Peak #	RetTime [min]	Type	Width [min]	Area [mAU*s]	Height [mAU]	Area %
1	12.182	BB	0.3180	196.99268	9.12023	3.0191
2	14.539	BB	0.3981	6327.93652	232.11490	96.9809

Supplementary Figure 8. HPLC spectra of compound **3ah**

(2*R*,3*S*)-3-(4-Bromophenyl)-2-(quinoxalin-2-yl)oxirane-2-carbonitrile (3ai)

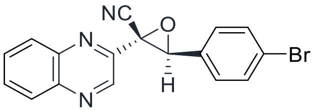

Compound **3ai** was synthesized following the General Procedure and subsequently purified using flash column chromatography (petroleum ether/EtOAc = 10/1), resulting in a colorless oil (19 mg) at 54% yield on a 0.1 mmol scale.

^{1}H NMR (600 MHz, $CDCl_3$) δ 9.04 (s, 1H), 8.18 (ddd, *J* = 7.0, 5.6, 3.3 Hz, 2H), 7.92 – 7.81 (m, 2H), 7.63 (d, *J* = 8.5 Hz, 2H), 7.42 (d, *J* = 8.5 Hz, 2H), 4.69 (s, 1H).

^{13}C NMR (150 MHz, $CDCl_3$) δ 145.7, 143.1, 141.5, 141.1, 132.2, 131.6, 131.5, 130.1, 129.7, 129.6, 128.4, 124.7, 114.4, 67.0, 57.6.

HRMS (ESI) *m/z* calcd. for $C_{17}H_{11}BrN_3O[M+H]^+$: 352.0080; found: 352.0082.

$[\alpha]_D^{20}$ = −54.0 (*c* 1.0, $CHCl_3$)

The enantiomeric excess was determined by chiral HPLC analysis using a Daicel Chiralpak IB column, e.e. = 93% (*n*-hexane/ethanol = 70/30, flow rate = 1.0 mL/min, λ = 254 nm, T = 20 °C, t_r (major) = 6.813 min, t_r (minor) = 8.149 min).

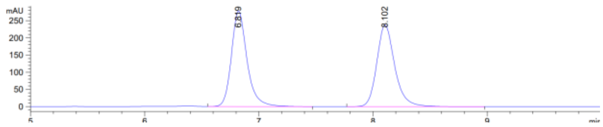

Signal 2: DAD1 B, Sig=254,4 Ref=off

Peak #	RetTime [min]	Type	Width [min]	Area [mAU*s]	Height [mAU]	Area %
1	6.819	BB	0.1453	2654.12939	278.32248	50.5133
2	8.102	BB	0.1678	2600.19092	237.41600	49.4867

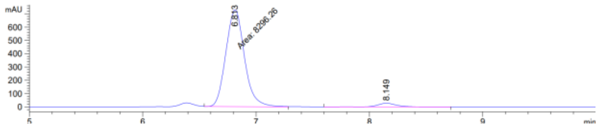

Signal 2: DAD1 B, Sig=254,4 Ref=off

Peak #	RetTime [min]	Type	Width [min]	Area [mAU*s]	Height [mAU]	Area %
1	6.813	MM	0.1898	8296.26465	728.67657	96.3808
2	8.149	VB R	0.1740	311.53690	27.11900	3.6192

Supplementary Figure 9. HPLC spectra of compound **3ai**

(2*R*,3*S*)-3-(4-Bromophenyl)-2-(6,7-dimethoxyquinazolin-4-yl)oxirane-2-carbonitrile (3aj)

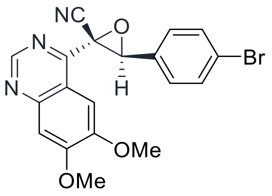

Compound **3aj** was synthesized following the General Procedure and subsequently purified using flash column chromatography (petroleum ether/EtOAc = 10/1), resulting in a white solid (37 mg) at 92% yield on a 0.1 mmol scale.

m.p.: 130.2 – 149.0 °C

^{1}H NMR (400 MHz, $CDCl_3$) δ 9.16 (s, 1H), 7.65 (d, *J* = 7.9 Hz, 2H), 7.64 (s, 1H), 7.48 (d, *J* = 8.0 Hz, 2H), 7.41 (s, 1H), 4.67 (s, 1H), 4.08 (d, *J* = 3.7 Hz, 6H).

^{13}C NMR (100 MHz, $CDCl_3$) δ 157.0, 154.4, 152.9, 151.7, 149.9, 132.3, 130.0, 128.4, 124.7, 118.5, 115.1, 107.4, 100.6, 64.1, 58.1, 56.7, 56.6.

HRMS (ESI) *m/z* calcd. for $C_{19}H_{15}BrN_3O_3$ $[M+H]^+$: 384.9907; found: 384.9908.

$[\alpha]_D^{20}$ = −16.5 (*c* 1.0, $CHCl_3$)

The enantiomeric excess was determined by chiral HPLC analysis using a Daicel Chiralpak IE column, e.e. = 95% (*n*-hexane/ethanol = 70/30, flow rate = 1.0 mL/min, λ = 254 nm, T = 20 °C, t_r (major) = 14.239 min, t_r (minor) = 15.893 min).

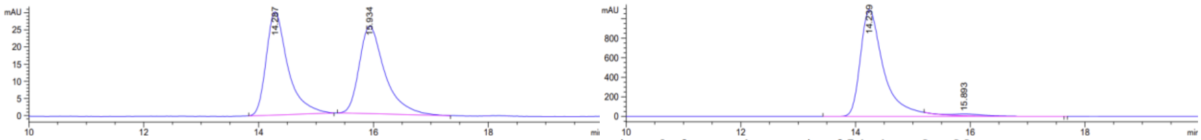

Signal 2: DAD1 B, Sig=254,4 Ref=off

Peak #	RetTime [min]	Type	Width [min]	Area [mAU*s]	Height [mAU]	Area %
1	14.287	BB	0.3873	787.07806	30.07933	49.8225
2	15.934	BB	0.4577	792.68500	25.56622	50.1775

Signal 2: DAD1 B, Sig=254,4 Ref=off

Peak #	RetTime [min]	Type	Width [min]	Area [mAU*s]	Height [mAU]	Area %
1	14.239	BV R	0.4275	3.12458e4	1086.49072	97.6309
2	15.893	VB E	0.5468	758.21405	19.03183	2.3691

Supplementary Figure 10. HPLC spectra of compound **3aj**

(2*R*,3*S*)-3-(4-Bromophenyl)-2-(7-((2-(trimethylsilyl)ethoxy)methyl)-7*H*-pyrrolo[2,3-*d*]pyrimidin-4-yl)oxirane-2-carbonitrile (3ak)

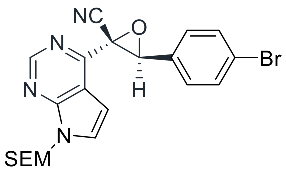

Compound **3ak** was synthesized following the General Procedure and subsequently purified using flash column chromatography (petroleum ether/EtOAc = 4/1), resulting in a white solid (47 mg) at 99% yield on a 0.1 mmol scale.

m.p.: 109.1 − 113.6 °C

^{1}H NMR (400 MHz, $CDCl_3$) δ 8.92 (s, 1H), 7.60 (d, *J* = 8.5 Hz, 2H), 7.46 (d, *J* = 3.7 Hz, 1H), 7.39 (d, *J* = 8.5 Hz, 2H), 6.94 (d, *J* = 3.7 Hz, 1H), 5.68 (s, 2H), 4.70 (s, 1H), 3.57 − 3.51 (m, 2H), 1.03 − 0.82 (m, 2H), −0.05 (s, 9H).

^{13}C NMR (100 MHz, $CDCl_3$) δ 152.7, 151.4, 150.5, 132.1, 130.4, 130.2, 128.4, 124.5, 115.0, 100.3, 73.1, 67.0, 66.2, 58.5, 17.8, −1.3.

HRMS (ESI) *m/z* calcd. for $C_{21}H_{24}BrN_4O_2Si$ $[M+H]^+$: 471.0846; found: 471.0840.

$[\alpha]_D^{20}$ = −94.7 (*c* 1.0, $CHCl_3$)

The enantiomeric excess was determined by chiral HPLC analysis using a Daicel Chiralpak IB column, e.e. = 99% (*n*-hexane/ethanol = 98/2 flow rate = 1.0 mL/min, λ = 210 nm, T = 20 °C, t_r (major) = 15.526 min, t_r (minor) = 13.110 min).

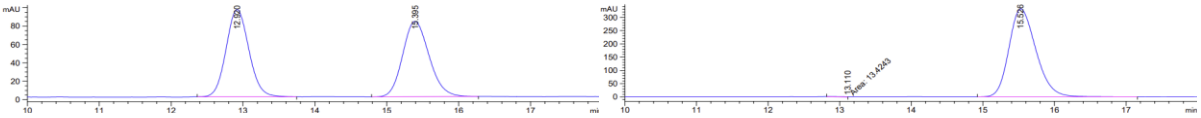

Signal 3: DAD1 C, Sig=210,4 Ref=off

Peak #	RetTime [min]	Type	Width [min]	Area [mAU*s]	Height [mAU]	Area %
1	12.920	BB	0.3429	2089.12280	94.86675	49.9237
2	15.395	BB	0.3963	2095.50977	81.95956	50.0763

Signal 3: DAD1 C, Sig=210,4 Ref=off

Peak #	RetTime [min]	Type	Width [min]	Area [mAU*s]	Height [mAU]	Area %
1	13.110	MM	0.2001	13.42432	1.11799	0.1546
2	15.526	BB	0.4053	8667.05078	331.22070	99.8454

Supplementary Figure 11. HPLC spectra of compound **3ak**

(2*S*,3*S*)-3-(4-Bromophenyl)-2-(1-tosyl-1*H*-benzo[*d*]imidazol-2-yl)oxirane-2-carbonitrile (3al)

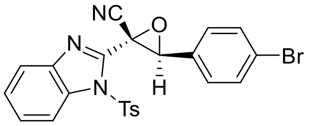

Compound **3al** was synthesized following the General Procedure and subsequently purified using flash column chromatography (petroleum ether/EtOAc = 10/1), resulting in a yellow oil (33 mg) at 67% yield on a 0.1 mmol scale.

^{1}H NMR (600 MHz, $CDCl_3$) δ 8.10 (d, *J* = 8.5 Hz, 2H), 7.94 (d, *J* = 8.4 Hz, 1H), 7.79 (d, *J* = 8.0 Hz, 1H), 7.65 (d, *J* = 8.4 Hz, 2H), 7.56 (d, *J* = 8.4 Hz, 2H), 7.49 (t, *J* = 7.6 Hz, 1H), 7.42 (t, *J* = 7.6 Hz, 1H), 7.32 (d, *J* = 8.5 Hz, 2H), 4.83 (s, 1H), 2.38 (s, 3H).

^{13}C NMR (150 MHz, $CDCl_3$) δ 147.0, 143.7, 141.4, 134.0, 132.6, 132.1, 130.4, 129.7, 128.7, 128.2, 127.3, 125.8, 124.6, 121.7, 114.2, 113.6, 64.9, 52.7, 21.9.

HRMS (ESI) *m/z* calcd. for $C_{23}H_{17}BrN_3O_3S$ $[M+H]^+$: 471.0846; found: 471.0840.

$[\alpha]_D^{20}$ = −109.2 (*c* 1.0, $CHCl_3$)

The enantiomeric excess was determined by chiral HPLC analysis using a Daicel Chiralpak IA column, e.e. = 98% (*n*-hexane/ethanol = 70/30, flow rate = 1.0 mL/min, λ = 254 nm, T = 20 °C, t_r (major) = 12.575 min, t_r (minor) = 9.164 min).

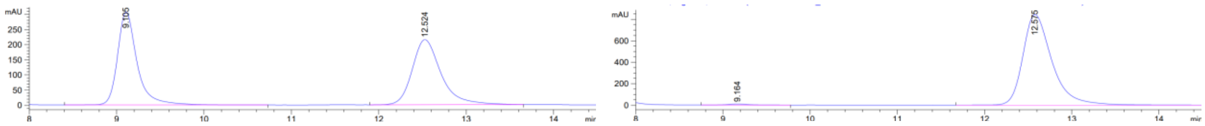

Signal 2: DAD1 B, Sig=254,4 Ref=off

Peak #	RetTime [min]	Type	Width [min]	Area [mAU*s]	Height [mAU]	Area %
1	9.105	BB	0.2313	4772.23340	307.16248	49.1149
2	12.524	BB	0.3460	4944.23633	215.24095	50.8851

Signal 2: DAD1 B, Sig=254,4 Ref=off

Peak #	RetTime [min]	Type	Width [min]	Area [mAU*s]	Height [mAU]	Area %
1	9.164	BB	0.2348	163.41582	10.20599	0.8260
2	12.575	BB	0.3509	1.96207e4	845.00122	99.1740

Supplementary Figure 12. HPLC spectra of compound **3al**

(2*R*,3*S*)-2-(Benzo[*d*]oxazol-2-yl)-3-(4-bromophenyl)oxirane-2-carbonitrile (3am)

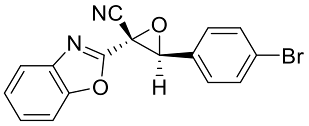

Compound **3am** was synthesized following the General Procedure and subsequently purified using flash column chromatography (petroleum ether/EtOAc = 10/1), resulting in a yellow oil (32 mg) at 94% yield on a 0.1 mmol scale.

^{1}H NMR (600 MHz, $CDCl_3$) δ 7.88 − 7.80 (m, 1H), 7.63 (d, *J* = 8.5 Hz, 2H), 7.62 − 7.59 (m, 1H), 7.46 (dtd, *J* = 19.3, 7.5, 1.3 Hz, 2H), 7.41 (d, *J* = 8.5 Hz, 2H), 4.97 (s, 1H).

^{13}C NMR (150 MHz, $CDCl_3$) δ 156.1, 151.2, 140.7, 132.4, 129.1, 128.4, 127.1, 125.8, 125.1, 121.2, 112.7, 111.3, 65.6, 51.9.

HRMS (ESI) *m/z* calcd. for $C_{16}H_{10}BrN_2O_2$ $[M+H]^+$: 340.9920; found: 340.9925.

$[\alpha]_D^{20}$ = −280.0 (*c* 1.0, $CHCl_3$)

The enantiomeric excess was determined by chiral HPLC analysis using a Daicel Chiralpak IA column, e.e. = 99% (*n*-hexane/ethanol = 70/30, flow rate = 1.0 mL/min, λ = 254 nm, T = 20 ºC, t_r (major) = 8.701 min, t_r (minor) = 7.528 min).

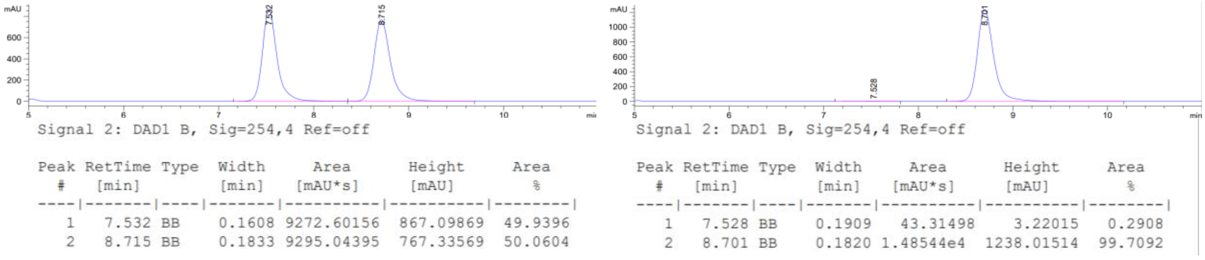

Peak #	RetTime [min]	Type	Width [min]	Area [mAU*s]	Height [mAU]	Area %
1	7.532	BB	0.1608	9272.60156	867.09869	49.9396
2	8.715	BB	0.1833	9295.04395	767.33569	50.0604

Peak #	RetTime [min]	Type	Width [min]	Area [mAU*s]	Height [mAU]	Area %
1	7.528	BB	0.1909	43.31498	3.22015	0.2908
2	8.701	BB	0.1820	1.48544e4	1238.01514	99.7092

Supplementary Figure 13. HPLC spectra of compound **3am**

(2*R*,3*S*)-2-(Benzo[*d*]thiazol-2-yl)-3-(4-bromophenyl)oxirane-2-carbonitrile (3an)

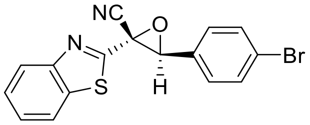

Compound **3an** was synthesized following the General Procedure and subsequently purified using flash column chromatography (petroleum ether/EtOAc = 3/1), resulting in a yellow solid (34 mg) at 96% yield on a 0.1 mmol scale.

m.p.: 132.6 − 136.2 °C

^{1}H NMR (400 MHz, $CDCl_3$) δ 8.13 (d, *J* = 8.2 Hz, 1H), 7.94 (d, *J* = 8.2 Hz, 1H), 7.62 (d, *J* = 8.2 Hz, 2H), 7.57 (d, *J* = 7.7 Hz, 1H), 7.50 (t, *J* = 7.7 Hz, 1H), 7.39 (d, *J* = 8.2 Hz, 2H), 4.65 (s, 1H).

^{13}C NMR (100 MHz, $CDCl_3$) δ 162.0, 153.2, 134.9, 132.3, 129.5, 128.3, 127.3, 126.8, 124.9, 124.2, 122.1, 113.7, 68.4, 56.2.

HRMS (ESI) *m/z* calcd. for $C_{16}H_{10}BrN_2OS$ $[M+H]^+$: 356.9697; found: 356.9692.

$[\alpha]_D^{20}$ = −30.2 (*c* 1.0, $CHCl_3$)

The enantiomeric excess was determined by chiral HPLC analysis using a Daicel Chiralpak IJ column, e.e. = 99% (*n*-hexane/ethanol = 70/30, flow rate = 1.0 mL/min, λ = 254 nm, T = 20 °C, t_r (major) = 16.498 min, t_r (minor) = 12.540 min).

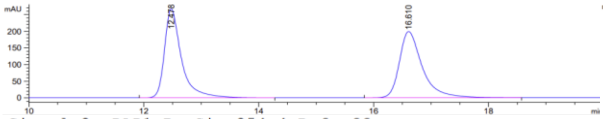

Signal 2: DAD1 B, Sig=254,4 Ref=off

Peak #	RetTime [min]	Type	Width [min]	Area [mAU*s]	Height [mAU]	Area %
1	12.478	BB	0.3038	5478.60059	266.56714	49.9321
2	16.610	BB	0.4169	5493.50635	198.47574	50.0679

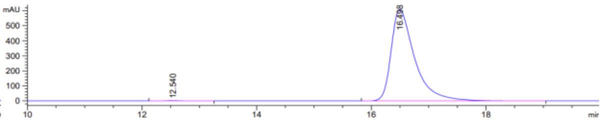

Signal 2: DAD1 B, Sig=254,4 Ref=off

Peak #	RetTime [min]	Type	Width [min]	Area [mAU*s]	Height [mAU]	Area %
1	12.540	BB	0.2707	40.70206	2.04055	0.2341
2	16.498	BB	0.4207	1.73439e4	608.23694	99.7659

Supplementary Figure 14. HPLC spectra of compound **3an**

(2*R*,3*S*)-3-(4-Bromophenyl)-2-(4-(4-fluorophenyl)thiazol-2-yl)oxirane-2-carbonitrile (3ao)

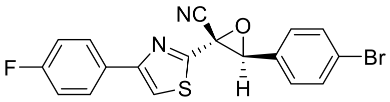

Compound **3ao** was synthesized following the General Procedure and subsequently purified using flash column chromatography (petroleum ether/EtOAc = 10/1), resulting in a yellow oil (38 mg) at 95% yield on a 0.1 mmol scale.

^{1}H NMR (600 MHz, $CDCl_3$) δ 7.91 (dd, *J* = 8.7, 5.4 Hz, 2H), 7.62 (d, *J* = 8.5 Hz, 2H), 7.54 (s, 1H), 7.39 (d, *J* = 8.5 Hz, 2H), 7.13 (t, *J* = 8.7 Hz, 2H), 4.67 (s, 1H).

^{13}C NMR (150 MHz, $CDCl_3$) δ 164.1, 162.4, 161.2, 156.2, 132.3, 129.7, 128.5 (d, *J* = 8.3 Hz), 128.3, 124.8, 116.0 (d, *J* = 21.6 Hz), 114.3, 114.0, 68.2, 55.8.

^{19}F NMR (565 MHz, $CDCl_3$) δ −115.5.

HRMS (ESI) *m/z* calcd. for $C_{18}H_{12}BrFN_2OS$ $[M+H]^+$: 340.9920; found: 340.9925.

$[\alpha]_D^{20}$ = −280.0 (*c* 1.0, $CHCl_3$)

The enantiomeric excess was determined by chiral HPLC analysis using a Daicel Chiralpak IA column, e.e. = 99% (*n*-hexane/ethanol = 90/10, flow rate = 1.0 mL/min, λ = 210 nm, T = 20 °C, t_r (major) = 16.182 min, t_r (minor) = 13.608 min).

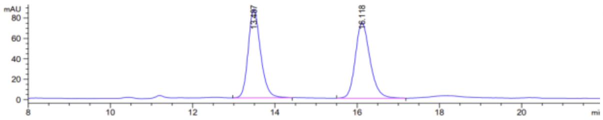

Signal 3: DAD1 C, Sig=210,4 Ref=off

Peak #	RetTime [min]	Type	Width [min]	Area [mAU*s]	Height [mAU]	Area %
1	13.487	BB	0.3204	1821.91211	86.98545	49.8465
2	16.118	BB	0.3764	1833.13574	74.15337	50.1535

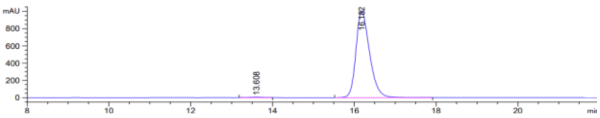

Signal 3: DAD1 C, Sig=210,4 Ref=off

Peak #	RetTime [min]	Type	Width [min]	Area [mAU*s]	Height [mAU]	Area %
1	13.608	BB	0.2770	80.42323	4.44672	0.3452
2	16.182	BB	0.3535	2.32143e4	1004.93158	99.6548

Supplementary Figure 15. HPLC spectra of compound **3ao**

(2*S*,3*S*)-3-(4-Bromophenyl)-2-(1-phenyl-1*H*-imidazol-2-yl)oxirane-2-carbonitrile (3ap)

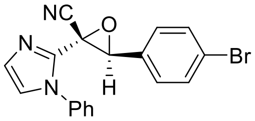

Compound **3ap** was synthesized following the General Procedure and subsequently purified using flash column chromatography (petroleum ether/EtOAc = 10/1), resulting in a yellow oil (34 mg) at 93% yield on a 0.1 mmol scale.

^{1}H NMR (600 MHz, $CDCl_3$) δ 7.60 (dd, *J* = 8.5, 6.5 Hz, 2H), 7.59 − 7.50 (m, 5H), 7.33 (d, *J* = 8.5 Hz, 2H), 7.23 (d, *J* = 14.3 Hz, 2H), 5.03 (s, 1H).

^{13}C NMR (150 MHz, $CDCl_3$) δ 138.1, 136.3, 132.0, 130.2, 130.1, 129.8, 129.5, 128.5, 125.7, 124.3, 124.3, 113.9, 63.7, 50.9.

HRMS (ESI) *m/z* calcd. for $C_{18}H_{13}BrN_2O$ $[M+H]^+$: 366.0237; found: 366.0238.

$[\alpha]_D^{20}$ = −290.9 (*c* 1.0, $CHCl_3$)

The enantiomeric excess was determined by chiral HPLC analysis using a Daicel Chiralpak IA column, e.e. = 67% (*n*-hexane/ethanol = 90/10, flow rate = 1.0 mL/min, λ = 254 nm, T = 20 °C, t_r (major) = 15.550 min, t_r (minor) = 12.650 min).

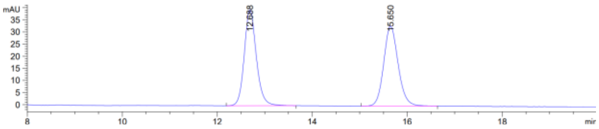

Signal 2: DAD1 B, Sig=254,4 Ref=off

Peak #	RetTime [min]	Type	Width [min]	Area [mAU*s]	Height [mAU]	Area %
1	12.688	BB	0.2678	698.97900	39.62671	50.1882
2	15.650	BB	0.3214	693.73724	32.98638	49.8118

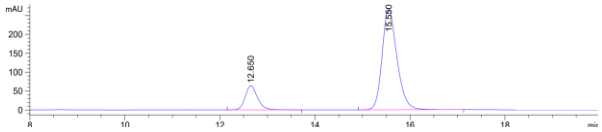

Signal 2: DAD1 B, Sig=254,4 Ref=off

Peak #	RetTime [min]	Type	Width [min]	Area [mAU*s]	Height [mAU]	Area %
1	12.650	BB	0.2709	1148.77112	64.16102	16.5605
2	15.550	BB	0.3316	5788.05762	266.29865	83.4395

Supplementary Figure 16. HPLC spectra of compound **3ap**

(2*R*,3*S*)-3-(4-Bromophenyl)-2-(5-phenyloxazol-2-yl)oxirane-2-carbonitrile (3aq)

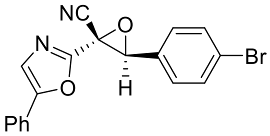

Compound **3aq** was synthesized following the General Procedure and subsequently purified using flash column chromatography (petroleum ether/EtOAc = 6/1), resulting in a white solid (33 mg) at 91% yield on a 0.1 mmol scale.

m.p.: 161.2 − 162.7 °C

^{1}H NMR (400 MHz, $CDCl_3$) δ 7.67 (d, *J* = 7.4 Hz, 2H), 7.62 (d, *J* = 8.0 Hz, 2H), 7.43 (dt, *J* = 21.4, 7.4 Hz, 6H), 4.93 (s, 1H).

^{13}C NMR (100 MHz, $CDCl_3$) δ 154.2, 153.5, 132.3, 129.7, 129.3, 129.3, 128.4, 126.7, 124.9, 124.8, 123.3, 112.9, 65.1, 51.5.

HRMS (ESI) *m/z* calcd. for $C_{16}H_{12}BrN_2O_2$ $[M+H]^+$: 367.0082; found: 367.0094.

$[\alpha]_D^{20}$ = −258.0 (*c* 1.0, $CHCl_3$)

The enantiomeric excess was determined by chiral HPLC analysis using a Daicel Chiralpak IA column, e.e. = 93% (*n*-hexane/ethanol = 90/10, flow rate = 1.0 mL/min, λ = 254 nm, T = 20 °C, t_r (major) = 15.223 min, t_r (minor) = 16.269 min).

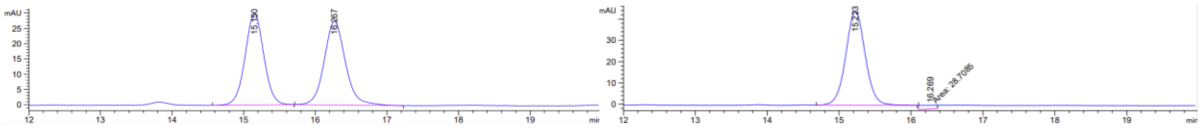

Signal 2: DAD1 B, Sig=254,4 Ref=off

Peak #	RetTime [min]	Type	Width [min]	Area [mAU*s]	Height [mAU]	Area %
1	15.150	BB	0.2780	545.06091	30.00195	49.1453
2	16.267	BB	0.3140	564.01880	27.41585	50.8547

Signal 2: DAD1 B, Sig=254,4 Ref=off

Peak #	RetTime [min]	Type	Width [min]	Area [mAU*s]	Height [mAU]	Area %
1	15.223	BB	0.2884	830.22949	44.35783	96.6577
2	16.269	MM	0.1954	28.70855	1.75898	3.3423

Supplementary Figure 17. HPLC spectra of compound **3aq**

(2*R*,3*S*)-3-(4-Bromophenyl)-2-(5-(4-chlorophenyl)-1,3,4-oxadiazol-2-yl)oxirane-2-carbonitrile (3ar)

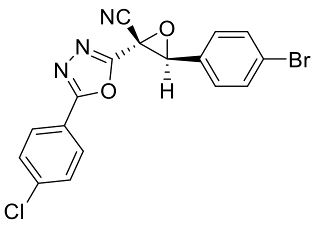

Compound **3ar** was synthesized following the General Procedure and subsequently purified using flash column chromatography (petroleum ether/EtOAc = 6/1), resulting in a white solid (35 mg) at 88% yield on a 0.1 mmol scale.

m.p.: 242.6 − 247.4 °C

^{1}H NMR (400 MHz, $CDCl_3$) δ 8.03 (d, *J* = 8.6 Hz, 2H), 7.65 (d, *J* = 8.5 Hz, 2H), 7.54 (d, *J* = 8.7 Hz, 2H), 7.41 (d, *J* = 8.4 Hz, 2H), 4.98 (s, 1H).

^{13}C NMR (100 MHz, $CDCl_3$) δ 165.9, 158.8, 139.5, 132.5, 129.9, 128.8, 128.5, 128.4, 125.4, 121.2, 112.0, 65.3, 49.4, 31.7, 22.8, 14.2.

HRMS (ESI) *m/z* calcd. for $C_{17}H_{10}BrClN_3O_2$ $[M+H]^+$:401.9645; found:401.9643.

$[\alpha]_D^{20}$ = −43.1 (*c* 1.0, $CHCl_3$)

The enantiomeric excess was determined by chiral HPLC analysis using a Daicel Chiralpak IJ column, e.e. = 97% (*n*-hexane/ethanol = 80/20, flow rate = 1.0 mL/min, λ = 254 nm, T = 20 °C, t_r (major) = 34.102 min, t_r (minor) = 27.987 min).

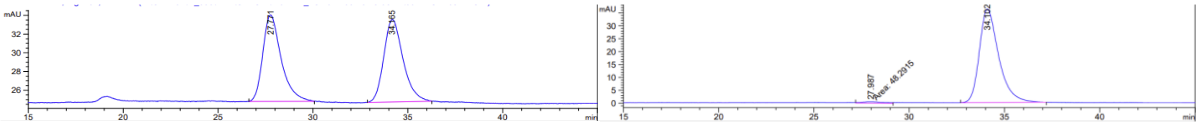

Signal 2: DAD1 B, Sig=254,4 Ref=off

Peak #	RetTime [min]	Type	Width [min]	Area [mAU*s]	Height [mAU]	Area %
1	27.771	BB	0.8120	621.48114	9.24860	49.9486
2	34.165	BB	0.8485	622.76086	8.79028	50.0514

Signal 2: DAD1 B, Sig=254,4 Ref=off

Peak #	RetTime [min]	Type	Width [min]	Area [mAU*s]	Height [mAU]	Area %
1	27.987	MM	1.4281	48.29150	5.63578e-1	1.8550
2	34.102	BB	0.9943	2555.00513	36.25180	98.1450

Supplementary Figure 18. HPLC spectra of compound **3ar**

(2*R*,3*S*)-3-(4-Bromophenyl)-2-(4,4-dimethyl-4,5-dihydrooxazol-2-yl)oxirane-2-carbonitrile (3as)

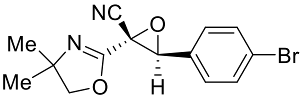

Compound **3as** was synthesized following the General Procedure and subsequently purified using flash column chromatography (petroleum ether/EtOAc = 10/1), resulting in a white solid (23 mg) at 82% yield on a 0.1 mmol scale.

m.p.: 85.2 − 88.7 °C

^{1}H NMR (400 MHz, $CDCl_3$) δ 7.58 (d, *J* = 8.5 Hz, 2H), 7.33 (d, *J* = 8.5 Hz, 2H), 4.58 (s, 1H), 4.21 − 4.02 (m, 2H), 1.38 (s, 3H), 1.36 (s, 3H).

^{13}C NMR (100 MHz, $CDCl_3$) δ 156.8, 132.2, 129.5, 128.5, 124.8, 113.2, 81.0, 69.0, 63.5, 51.5, 28.2, 28.2.

HRMS (ESI) *m/z* calcd. for $C_{14}H_{14}BrN_2O_2$ $[M+H]^+$:321.0239; found: 321.0277.

$[\alpha]_D^{20}$ = −20.2 (*c* 1.0, $CHCl_3$)

The enantiomeric excess was determined by chiral HPLC analysis using a Daicel Chiralpak IJ column, e.e. = 99% (*n*-hexane/ethanol = 90/10, flow rate = 1.0 mL/min, λ = 250 nm, T = 20 ºC, t_r (major) = 11.443 min, t_r (minor) = 12.096 min).

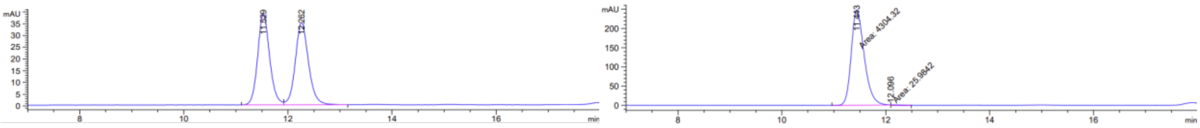

Signal 1: DAD1 A, Sig=250,4 Ref=off

Peak #	RetTime [min]	Type	Width [min]	Area [mAU*s]	Height [mAU]	Area %
1	11.529	BV	0.2451	621.33502	38.74083	50.1848
2	12.262	VB	0.2675	616.75879	35.01472	49.8152

Signal 1: DAD1 A, Sig=250,4 Ref=off

Peak #	RetTime [min]	Type	Width [min]	Area [mAU*s]	Height [mAU]	Area %
1	11.443	MM	0.2888	4304.31982	248.38832	99.3999
2	12.096	MM	0.2036	25.98425	1.59923	0.6001

Supplementary Figure 19. HPLC spectra of compound **3as**

(2*R*,3*S*)-2-(Benzo[*d*]thiazol-2-yl)-3-phenyloxirane-2-carbonitrile (3ba)

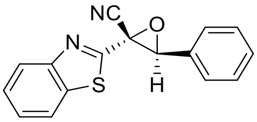

Compound **3ba** was synthesized following the General Procedure and subsequently purified using flash column chromatography (petroleum ether/EtOAc = 6/1), resulting in a white solid (27 mg) at 96% yield on a 0.1 mmol scale.

m.p.: 127.0 − 129.3 °C

^{1}H NMR (600 MHz, $CDCl_3$) δ 7.58 (d, *J* = 8.5 Hz, 2H), 7.33 (d, *J* = 8.5 Hz, 2H), 4.58 (s, 1H), 4.21 − 4.02 (m, 2H), 1.38 (s, 3H), 1.36 (s, 3H).

^{13}C NMR (150 MHz, $CDCl_3$) δ 156.8, 132.2, 129.5, 128.5, 124.8, 113.2, 81.0, 69.0, 63.5, 51.5, 28.2, 28.2.

HRMS (ESI) *m/z* calcd. for $C_{16}H_{11}N_2OS$ $[M+H]^+$: 279.0587; found: 279.0593.

$[\alpha]_D^{20}$ = −316.6 (*c* 1.0, $CHCl_3$)

The enantiomeric excess was determined by chiral HPLC analysis using a Daicel Chiralpak IB column, e.e. = 99% (*n*-hexane/ethanol = 80/20, flow rate = 1.0 mL/min, λ = 254 nm, T = 20 °C, t_r (major) = 5.812 min, t_r (minor) = 6.572 min).

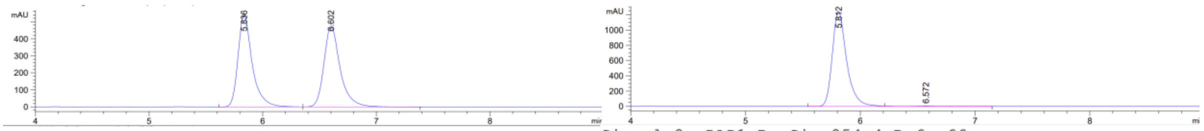

Signal 2: DAD1 B, Sig=254,4 Ref=off

Peak #	RetTime [min]	Type	Width [min]	Area [mAU*s]	Height [mAU]	Area %
1	5.836	BB	0.1311	4684.60449	540.64569	50.0069
2	6.602	BB	0.1490	4683.31836	475.23444	49.9931

Signal 2: DAD1 B, Sig=254,4 Ref=off

Peak #	RetTime [min]	Type	Width [min]	Area [mAU*s]	Height [mAU]	Area %
1	5.812	BV R	0.1311	1.08134e4	1248.05603	99.5766
2	6.572	VB E	0.1642	45.98225	4.05675	0.4234

Supplementary Figure 20. HPLC spectra of compound **3ba**

(2*R*,3*S*)-2-(Pyridin-2-yl)-3-(4-((trifluoromethyl)thio)phenyl)oxirane-2-carbonitrile (3bb)

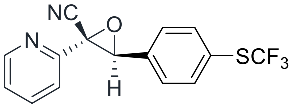

Compound **3bb** was synthesized following the General Procedure and subsequently purified using flash column chromatography (petroleum ether/EtOAc = 10/1), resulting in a colorless oil (31 mg) at 96% yield on a 0.1 mmol scale.

^{1}H NMR (400 MHz, $CDCl_3$) δ 8.70 (d, *J* = 4.7 Hz, 1H), 7.84 (t, *J* = 7.9 Hz, 1H), 7.75 (d, *J* = 8.0 Hz, 2H), 7.63 (d, *J* = 7.9 Hz, 1H), 7.57 (d, *J* = 8.0 Hz, 2H), 7.41 (t, *J* = 6.2 Hz, 1H), 4.65 (s, 1H).

^{13}C NMR (100 MHz, $CDCl_3$) δ 150.5, 150.3, 137.7, 136.5, 134.7, 129.5 (q, *J* = 308.4 Hz), 127.8, 126.4, 125.0, 120.8, 115.1, 66.3, 58.3.

^{19}F NMR (376 MHz, $CDCl_3$) δ −45.4.

HRMS (ESI) *m/z* calcd. for $C_{15}H_{10}F_3N_2OS$ $[M+H]^+$: 323.0460; found: 323.0457.

$[\alpha]_D^{20}$ = −218.0 (*c* 1.0, $CHCl_3$)

The enantiomeric excess was determined by chiral HPLC analysis using a Daicel Chiralpak IJ column, e.e. = 99% (*n*-hexane/ethanol = 80/20, flow rate = 1.0 mL/min, λ = 254 nm, T = 20 °C, t_r (major) = 8.455 min, t_r (minor) = 7.510 min).

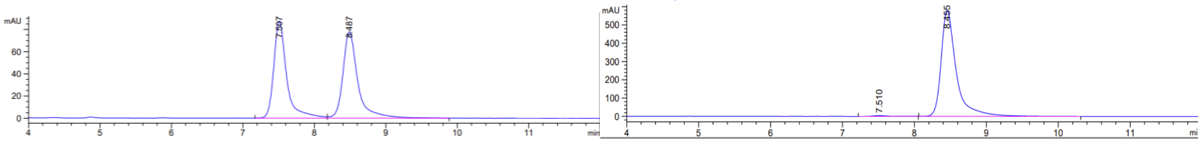

Signal 2: DAD1 B, Sig=254,4 Ref=off

Peak #	RetTime [min]	Type	Width [min]	Area [mAU*s]	Height [mAU]	Area %
1	7.507	BV	0.1869	1100.59875	87.38941	49.3327
2	8.487	VB	0.2153	1130.37280	77.90610	50.6673

Signal 2: DAD1 B, Sig=254,4 Ref=off

Peak #	RetTime [min]	Type	Width [min]	Area [mAU*s]	Height [mAU]	Area %
1	7.510	BB	0.1831	52.25182	4.32044	0.6128
2	8.455	BB	0.2163	8474.49805	580.53070	99.3872

Supplementary Figure 21. HPLC spectra of compound **3bb**

(2*R*,3*S*)-3-(4-(Methylsulfonyl)phenyl)-2-(2-(methylthio)pyrimidin-4-yl)oxirane-2-carbonitrile (3bc)

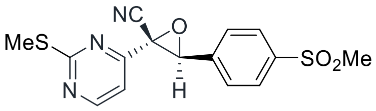

Compound **3bc** was synthesized following the General Procedure and subsequently purified using flash column chromatography (petroleum ether/EtOAc = 10/1), resulting in a colorless oil (26 mg) at 76% yield on a 0.1 mmol scale.

^{1}H NMR (400 MHz, $CDCl_3$) δ 8.66 (d, *J* = 5.0 Hz, 1H), 8.07 (d, *J* = 8.2 Hz, 2H), 7.71 (d, *J* = 8.2 Hz, 2H), 7.21 (d, *J* = 5.0 Hz, 1H), 4.66 (s, 1H), 3.10 (s, 3H), 2.60 (s, 3H).

^{13}C NMR (100 MHz, $CDCl_3$) δ 184.4, 174.4, 159.0, 142.4, 136.9, 128.1, 127.9, 113.7, 112.0, 66.0, 57.4, 44.6, 14.4.

HRMS (ESI) *m/z* calcd. for $C_{14}H_{13}N_2O_3S_2$ $[M+H]^+$:348.0471; found: 348.0474.

$[\alpha]_D^{20}$ = −127.0 (*c* 1.0, $CHCl_3$)

The enantiomeric excess was determined by chiral HPLC analysis using a Daicel Chiralpak IA column, e.e. = 90% (*n*-hexane/ethanol = 70/30, flow rate = 1.0 mL/min, λ = 254 nm, T = 20 °C, t_r (major) = 47.997 min, t_r (minor) = 58.486 min).

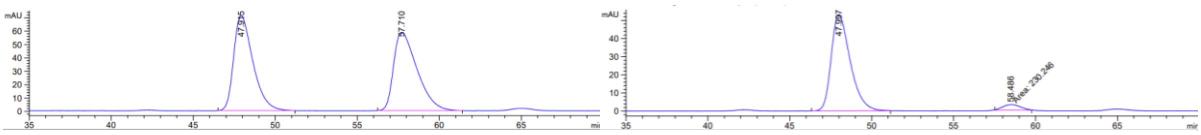

Signal 2: DAD1 B, Sig=254,4 Ref=off

Peak #	RetTime [min]	Type	Width [min]	Area [mAU*s]	Height [mAU]	Area %
1	47.915	BB	1.1594	5775.64014	71.12257	49.8822
2	57.710	BB	1.3203	5802.92480	58.54853	50.1178

Signal 2: DAD1 B, Sig=254,4 Ref=off

Peak #	RetTime [min]	Type	Width [min]	Area [mAU*s]	Height [mAU]	Area %
1	47.997	BB	1.1512	4287.81299	53.15156	94.9039
2	58.486	MM	1.2457	230.24565	3.08061	5.0961

Supplementary Figure 22. HPLC spectra of compound **3bc**

(2*S*,3*S*)-2-(4,6-Dimethoxy-1,3,5-triazin-2-yl)-3-(4-(methoxymethoxy)phenyl)oxirane-2-carbonitrile (3bd)

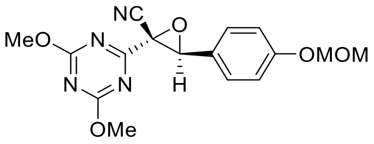

Compound **3bd** was synthesized following the General Procedure and subsequently purified using flash column chromatography (petroleum ether/EtOAc = 10/1), resulting in a colorless oil (32 mg) at 92% yield on a 0.1 mmol scale.

^{1}H NMR (400 MHz, DMSO-d_6) δ 7.46 (d, J = 7.9 Hz, 2H), 7.13 (d, J = 7.9 Hz, 2H), 5.25 (s, 2H), 4.91 (s, 1H), 4.02 (s, 6H), 3.39 (s, 3H).

^{13}C NMR (100 MHz, DMSO-d_6) δ 172.3, 171.6, 158.0, 128.3, 123.9, 116.1, 114.5, 93.7, 65.6, 57.1, 55.7, 55.7.

HRMS (ESI) *m/z* calcd. for $C_{16}H_{17}N_4O_5$ [M+H]$^+$: 345.1193; found: 345.1197.

$[\alpha]_D^{20}$ = −24.6 (c 1.0, $CHCl_3$)

The enantiomeric excess was determined by chiral HPLC analysis using a Daicel Chiralpak IJ column, e.e. = 99% (*n*-hexane/ethanol = 80/20, flow rate = 1.0 mL/min, λ = 254 nm, T = 20 ºC, t_r (major) = 12.308 min, t_r (minor) = 14.825 min).

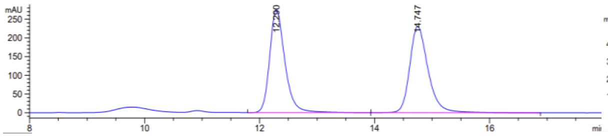

Signal 2: DAD1 B, Sig=254,4 Ref=off

Peak #	RetTime [min]	Type	Width [min]	Area [mAU*s]	Height [mAU]	Area %
1	12.290	BB	0.2802	5106.22217	275.50409	49.8932
2	14.747	BB	0.3356	5128.09033	230.43816	50.1068

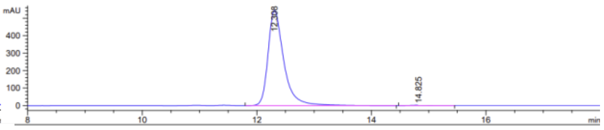

Signal 2: DAD1 B, Sig=254,4 Ref=off

Peak #	RetTime [min]	Type	Width [min]	Area [mAU*s]	Height [mAU]	Area %
1	12.308	BB	0.2878	1.04322e4	543.74207	99.5849
2	14.825	BB	0.2806	43.48927	2.14401	0.4151

Supplementary Figure 23. HPLC spectra of compound **3bd**

4-((2*S*,3*S*)-3-Cyano-3-(4,6-dimethoxy-1,3,5-triazin-2-yl)oxiran-2-yl)phenyl acetate (3be)

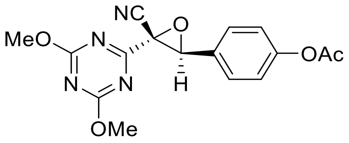

Compound **3be** was synthesized following the General Procedure and subsequently purified using flash column chromatography (petroleum ether/EtOAc = 10/1), resulting in a white solid (30 mg) at 88% yield on a 0.1 mmol scale.

m.p.: 170.1 – 170.8 °C

^{1}H NMR (400 MHz, $CDCl_3$) δ 7.50 (d, J = 8.6 Hz, 2H), 7.20 (d, J = 8.6 Hz, 2H), 4.78 (s, 1H), 4.11 (s, 6H), 2.31 (s, 3H).

^{13}C NMR (100 MHz, $CDCl_3$) δ 173.0, 172.3, 169.1, 152.2, 128.2, 122.2, 113.6, 65.8, 57.2, 56.1, 21.2.

HRMS (ESI) m/z calcd. for $C_{16}H_{14}N_4O_5$ $[M+Na]^+$: 365.0856; found: 365.0849.

$[\alpha]_D^{20}$ = −143.1 (c 1.0, $CHCl_3$)

The enantiomeric excess was determined by chiral HPLC analysis using a Daicel Chiralpak IA column, e.e. = 83% (n-hexane/ethanol = 70/30, flow rate = 1.0 mL/min, λ = 254 nm, T = 20 °C, t_r (major) = 15.250 min, t_r (minor) = 17.569 min).

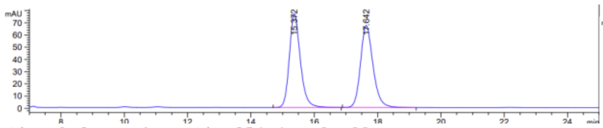

Signal 2: DAD1 B, Sig=254,4 Ref=off

Peak #	RetTime [min]	Type	Width [min]	Area [mAU*s]	Height [mAU]	Area %
1	15.372	BB	0.3687	1862.20398	76.83828	50.1637
2	17.642	BB	0.4208	1850.05188	67.28172	49.8363

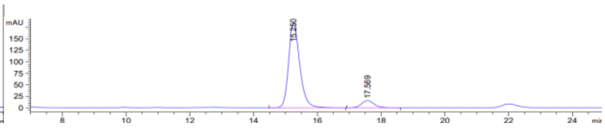

Signal 2: DAD1 B, Sig=254,4 Ref=off

Peak #	RetTime [min]	Type	Width [min]	Area [mAU*s]	Height [mAU]	Area %
1	15.250	BB	0.3682	4467.48145	184.70415	91.3075
2	17.569	BB	0.4117	425.30710	15.61906	8.6925

Supplementary Figure 24. HPLC spectra of compound **3be**

(2*S*,3*S*)-2-(4,6-Dimethoxy-1,3,5-triazin-2-yl)-3-(4-nitrophenyl)oxirane-2-carbonitrile (3bf)

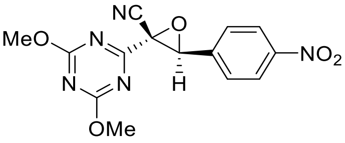

Compound **3bf** was synthesized following the General Procedure and subsequently purified using flash column chromatography (petroleum ether/EtOAc = 10/1), resulting in a white solid (30 mg) at 91% yield on a 0.1 mmol scale.

^{1}H NMR (600 MHz, $CDCl_3$) δ 8.33 (d, J = 8.7 Hz, 2H), 7.68 (d, J = 8.7 Hz, 2H), 4.93 (s, 1H), 4.12 (s, 6H).

^{13}C NMR (150 MHz, $CDCl_3$) δ 173.0, 171.6, 149.2, 137.7, 128.1, 124.1, 113.1, 64.6, 57.2, 56.2.

HRMS (ESI) m/z calcd. for $C_{14}H_{12}N_5O_5$ $[M+H]^+$: 330.0833; found: 330.0837.

$[\alpha]_D^{20}$ = −58.0 (c 1.0, $CHCl_3$)

The enantiomeric excess was determined by chiral HPLC analysis using a Daicel Chiralpak IF column, e.e. = 94% (n-hexane/ethanol = 70/30, flow rate = 1.0 mL/min, λ = 254 nm, T = 20 °C, t_r (major) = 19.121 min, t_r (minor) = 25.962 min).

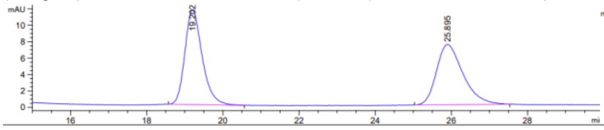

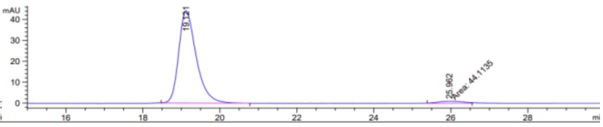

Signal 2: DAD1 B, Sig=254,4 Ref=off

Peak #	RetTime [min]	Type	Width [min]	Area [mAU*s]	Height [mAU]	Area %
1	19.202	BB	0.4698	364.74249	11.56959	50.7562
2	25.895	BB	0.6583	353.87363	7.34213	49.2438

Signal 2: DAD1 B, Sig=254,4 Ref=off

Peak #	RetTime [min]	Type	Width [min]	Area [mAU*s]	Height [mAU]	Area %
1	19.121	BB	0.4802	1393.52917	44.15956	96.9315
2	25.962	MM	0.7433	44.11349	9.89113e-1	3.0685

Supplementary Figure 25. HPLC spectra of compound **3bf**

(2*R*,3*S*)-3-(3-Chlorophenyl)-2-(pyridin-2-yl)oxirane-2-carbonitrile (3bg)

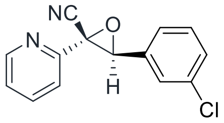

Compound **3bg** was synthesized following the General Procedure and subsequently purified using flash column chromatography (petroleum ether/EtOAc = 10/1), resulting in a white solid (23 mg) at 93% yield on a 0.1 mmol scale.

m.p.: 144.3 − 152.2 °C

^{1}H NMR (400 MHz, $CDCl_3$) δ 8.69 (d, *J* = 3.9 Hz, 1H), 7.83 (td, *J* = 7.9, 1.6 Hz, 1H), 7.62 (d, *J* = 7.9 Hz, 1H), 7.50 (s, 1H), 7.41 (m, 4H), 4.58 (s, 1H).

^{13}C NMR (100 MHz, $CDCl_3$) δ 150.4, 150.0, 137.4, 134.6, 133.5, 129.9, 126.7, 124.6, 124.5, 120.5, 114.8, 65.9, 57.9.

HRMS (ESI) *m/z* calcd. for $C_{14}H_{10}ClN_2O$ $[M+H]^+$: 257.0476; found: 257.0467.

$[\alpha]_D^{20}$ = −58.0 (*c* 1.0, $CHCl_3$)

The enantiomeric excess was determined by chiral HPLC analysis using a Daicel Chiralpak IC column, e.e. = 97% (*n*-hexane/ethanol = 90/10, flow rate = 1.0 mL/min, λ = 254 nm, T = 20 ºC, t_r (major) = 7.363 min, t_r (minor) = 8.491 min).

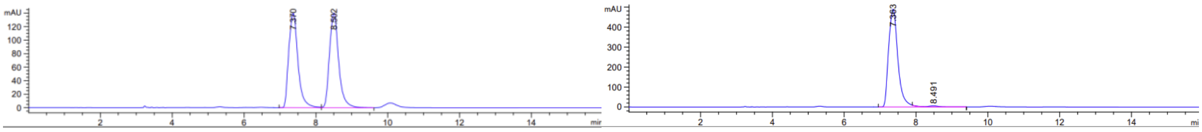

Signal 2: DAD1 B, Sig=254,4 Ref=off

Peak #	RetTime [min]	Type	Width [min]	Area [mAU*s]	Height [mAU]	Area %
1	7.370	BV	0.2840	2491.55615	139.76912	49.9076
2	8.502	VB	0.2799	2500.78271	140.30400	50.0924

Signal 2: DAD1 B, Sig=254,4 Ref=off

Peak #	RetTime [min]	Type	Width [min]	Area [mAU*s]	Height [mAU]	Area %
1	7.363	BV R	0.2862	8826.81250	489.88763	98.6460
2	8.491	VB E	0.3132	121.15920	5.76494	1.3540

Supplementary Figure 26. HPLC spectra of compound **3bg**

(2*R*,3*S*)-3-(3-Chloro-2-fluorophenyl)-2-(6,7-dimethoxyquinazolin-4-yl)oxirane-2-carbonitrile (3bh)

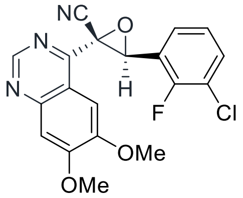

Compound **3bh** was synthesized following the General Procedure and subsequently purified using flash column chromatography (petroleum ether/EtOAc = 10/1), resulting in a white solid (24 mg) at 62% yield on a 0.1 mmol scale.

m.p.: 186.8 − 188.3 °C

^{1}H NMR (400 MHz, $CDCl_3$) δ 9.20 (s, 1H), 7.59 (s, 1H), 7.57 − 7.46 (m, 2H), 7.42 (s, 1H), 7.33 − 7.24 (m, 1H), 4.89 (s, 1H), 4.09 (s, 3H), 4.09 (s, 3H).

^{13}C NMR (100 MHz, $CDCl_3$) δ 157.3 (d, *J* = 250.9 Hz), 157.1, 153.9, 153.2, 151.9, 150.0, 132.5, 125.8 (d, *J* = 2.2 Hz), 125.3 (d, *J* = 4.7 Hz), 121.7 (d, *J* = 16.5 Hz), 120.9 (d, *J* = 12.6 Hz), 118.4, 114.9, 107.5, 100.5, 59.6 (d, *J* = 6.1 Hz), 57.2, 56.7, 56.6.

^{19}F NMR (376 MHz, $CDCl_3$) δ −123.3.

HRMS (ESI) *m/z* calcd. for $C_{19}H_{14}ClFN_3O_3$ $[M+H]^+$: 386.0702; found: 386.0698.

$[\alpha]_D^{20}$ = −68.0 (*c* 1.0, $CHCl_3$)

The enantiomeric excess was determined by chiral HPLC analysis using a Daicel Chiralpak IA column, e.e. = 88% (*n*-hexane/ethanol = 70/30, flow rate = 1.0 mL/min, λ = 254 nm, T = 20 °C, t_r (major) = 14.995 min, t_r (minor) = 12.050 min).

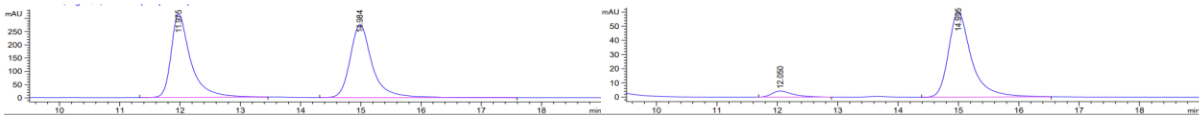

Signal 2: DAD1 B, Sig=254,4 Ref=off

Peak #	RetTime [min]	Type	Width [min]	Area [mAU*s]	Height [mAU]	Area %
1	11.975	BB	0.3296	7048.02393	314.29999	50.2346
2	14.984	BB	0.3802	6982.18311	275.02029	49.7654

Signal 2: DAD1 B, Sig=254,4 Ref=off

Peak #	RetTime [min]	Type	Width [min]	Area [mAU*s]	Height [mAU]	Area %
1	12.050	BB	0.3443	103.73347	4.31794	6.1533
2	14.995	BB	0.3953	1582.07983	60.06167	93.8467

Supplementary Figure 27. HPLC spectra of compound **3bh**

(2*R*,3*S*)-3-(2,4-Difluorophenyl)-2-(pyridin-2-yl)oxirane-2-carbonitrile (3bi)

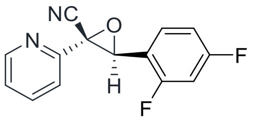

Compound **3bi** was synthesized following the General Procedure and subsequently purified using flash column chromatography (petroleum ether/EtOAc = 10/1), resulting in a colorless oil (24 mg) at 92% yield on a 0.1 mmol scale.

^{1}H NMR (400 MHz, $CDCl_3$) δ 8.70 (ddd, *J* = 4.8, 1.8, 0.9 Hz, 1H), 7.83 (td, *J* = 7.8, 1.8 Hz, 1H), 7.61 (dt, *J* = 7.8, 1.0 Hz, 1H), 7.50 (td, *J* = 8.5, 6.1 Hz, 1H), 7.40 (ddd, *J* = 7.6, 4.8, 1.1 Hz, 1H), 7.06 – 6.96 (m, 1H), 6.90 (ddd, *J* = 10.4, 8.6, 2.4 Hz, 1H), 4.78 (s, 1H).

^{13}C NMR (100 MHz, $CDCl_3$) δ 163.0 (dd, *J* = 434.3, 12.1 Hz), 163.0 (dd, *J* = 69.4, 12.1 Hz), 150.4, 150.4, 137.6, 128.5 (dd, *J* = 10.1, 4.4 Hz), 125.0, 120.7, 115.9 (dd, *J* = 12.8, 3.7 Hz), 115.2, 112.0 (dd, *J* = 21.9, 3.6 Hz), 104.3 (dd, *J* = 25.8, 24.1 Hz), 61.7 (d, *J* = 5.8 Hz), 58.0.

^{19}F NMR (376 MHz, $CDCl_3$) δ −110.0, −117.9.

HRMS (ESI) *m/z* calcd. for $C_{14}H_9F_2N_2O$ $[M+H]^+$: 259.0677; found: 259.0687.

$[\alpha]_D^{20}$ = −186.0 (*c* 1.0, $CHCl_3$)

The enantiomeric excess was determined by chiral HPLC analysis using a Daicel Chiralpak IA column, e.e. = 95% (*n*-hexane/ethanol = 80/20, flow rate = 1.0 mL/min, λ = 254 nm, T = 20 °C, t_r (major) = 6.599 min, t_r (minor) = 6.099 min).

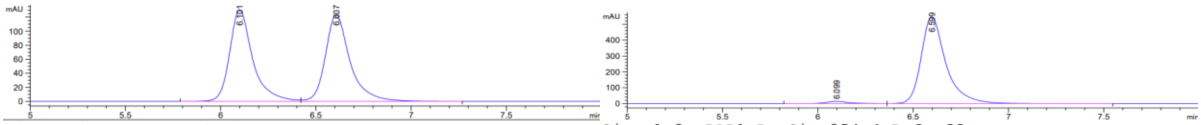

Signal 2: DAD1 B, Sig=254,4 Ref=off

Peak #	RetTime [min]	Type	Width [min]	Area [mAU*s]	Height [mAU]	Area %
1	6.101	BV	0.1261	1100.05566	130.80769	49.7926
2	6.607	VB	0.1329	1109.21826	123.35595	50.2074

Signal 2: DAD1 B, Sig=254,4 Ref=off

Peak #	RetTime [min]	Type	Width [min]	Area [mAU*s]	Height [mAU]	Area %
1	6.099	BV	0.1257	115.76114	13.82319	2.2894
2	6.599	VB	0.1332	4940.69238	547.97314	97.7106

Supplementary Figure 28. HPLC spectra of compound **3bi**

(2*R*,3*S*)-3-(naphthalen-2-yl)-2-(pyridin-2-yl)oxirane-2-carbonitrile (3bj)

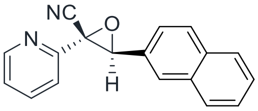

Compound **3bj** was synthesized following the General Procedure and subsequently purified using flash column chromatography (petroleum ether/EtOAc = 5/1), resulting in a white solid (25 mg) at 91% yield on a 0.1 mmol scale.

m.p.: 136.1 − 140.3 °C

^{1}H NMR (400 MHz, $CDCl_3$) δ 8.71 (dd, *J* = 4.8, 0.8 Hz, 1H), 8.01 (s, 1H), 7.93 (d, *J* = 8.6 Hz, 1H), 7.93 − 7.85 (m, 2H), 7.82 (td, *J* = 7.8, 1.7 Hz, 1H), 7.64 (d, *J* = 7.9 Hz, 1H), 7.59 (dd, *J* = 8.5, 1.8 Hz, 1H), 7.57 − 7.50 (m, 2H), 7.39 (ddd, *J* = 7.6, 4.8, 1.1 Hz, 1H), 4.75 (s, 1H).

^{13}C NMR (100 MHz, $CDCl_3$) δ 150.9, 149.9, 137.3, 133.8, 132.7, 128.8, 128.4, 128.1, 127.7, 126.8, 126.5, 126.5, 124.4, 123.1, 120.3, 115.2, 67.2, 58.2.

HRMS (ESI) *m/z* calcd. for $C_{18}H_{13}N_2O$ $[M+H]^+$: 273.1022; found: 273.1016.

$[\alpha]_D^{20}$ = −156.6 (*c* 1.0, $CHCl_3$)

The enantiomeric excess was determined by chiral HPLC analysis using a Daicel Chiralpak IC column, e.e. = 95% (*n*-hexane/ethanol = 80/20, flow rate = 1.0 mL/min, λ = 254 nm, T = 20 ºC, t_r (major) = 7.088 min, t_r (minor) = 8.417 min).

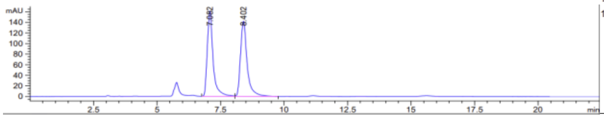

Signal 2: DAD1 B, Sig=254,4 Ref=off

Peak #	RetTime [min]	Type	Width [min]	Area [mAU*s]	Height [mAU]	Area %
1	7.082	BV	0.2445	2551.76343	161.39348	49.8744
2	8.402	VB	0.2770	2564.61255	141.83141	50.1256

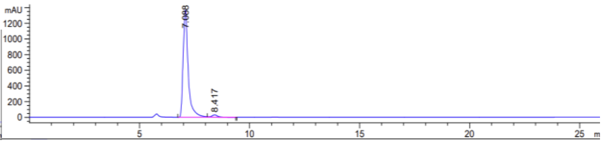

Signal 2: DAD1 B, Sig=254,4 Ref=off

Peak #	RetTime [min]	Type	Width [min]	Area [mAU*s]	Height [mAU]	Area %
1	7.088	BV R	0.2456	2.20443e4	1370.87732	97.6957
2	8.417	VB E	0.2717	519.94873	29.20392	2.3043

Supplementary Figure 29. HPLC spectra of compound **3bj**

(2*R*,3*S*)-3-(Benzofuran-2-yl)-2-(5-(trifluoromethyl)pyridin-2-yl)oxirane-2-carbonitrile (3bk)

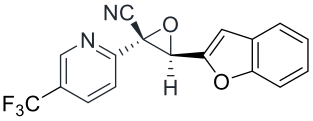

Compound **3bk** was synthesized following the General Procedure and subsequently purified using flash column chromatography (petroleum ether/EtOAc = 10/1), resulting in a yellow oil (21 mg) at 65% yield on a 0.1 mmol scale.

^{1}H NMR (400 MHz, $CDCl_3$) δ 9.01 – 8.91 (m, 1H), 8.10 (ddd, *J* = 8.3, 2.3, 0.8 Hz, 1H), 7.80 (dt, *J* = 8.3, 0.8 Hz, 1H), 7.65 (ddd, *J* = 7.7, 1.4, 0.7 Hz, 1H), 7.53 (dq, *J* = 8.4, 0.9 Hz, 1H), 7.38 (ddd, *J* = 8.4, 7.2, 1.4 Hz, 1H), 7.34 – 7.25 (m, 1H), 7.10 (t, *J* = 0.7 Hz, 1H), 4.76 (s, 1H).

^{13}C NMR (100 MHz, $CDCl_3$) δ 155.7, 154.1, 147.4 (d, *J* = 4.0 Hz), 135.1 (d, *J* = 3.6 Hz), 128.1 (q, *J* = 33.8 Hz), 127.5, 125.9, 124.5, 123.7, 122.0, 121.7, 120.7, 114.6, 111.8, 108.2, 61.6, 57.2.

^{19}F NMR (376 MHz, $CDCl_3$) δ −65.7.

HRMS (ESI) *m/z* calcd. for $C_{17}H_9F_3N_2NaO_2$ $[M+Na]^+$: 353.0508; found: 353.0498.

$[\alpha]_D^{20}$ = −212.0 (*c* 1.0, $CHCl_3$)

The enantiomeric excess was determined by chiral HPLC analysis using a Daicel Chiralpak IJ column, e.e. = 99% (*n*-hexane/ethanol = 80/20, flow rate = 1.0 mL/min, λ = 254 nm, T = 20 ºC, t_r (major) = 16.267 min, t_r (minor) = 18.180 min).

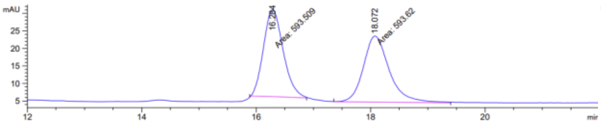

Signal 2: DAD1 B, Sig=254,4 Ref=off

Peak #	RetTime [min]	Type	Width [min]	Area [mAU*s]	Height [mAU]	Area %
1	16.284	MM	0.3977	593.50873	24.87033	49.9953
2	18.072	MM	0.5243	593.61969	18.86892	50.0047

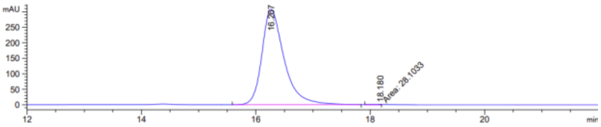

Signal 2: DAD1 B, Sig=254,4 Ref=off

Peak #	RetTime [min]	Type	Width [min]	Area [mAU*s]	Height [mAU]	Area %
1	16.267	BB	0.4017	8265.90527	307.39655	99.6612
2	18.180	MM	0.2587	28.10325	1.81068	0.3388

Supplementary Figure 30. HPLC spectra of compound **3bk**

(2*R*,3*R*)-2-(Pyridin-2-yl)-3-(thiophen-2-yl)oxirane-2-carbonitrile (3bl)

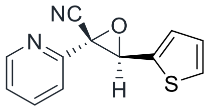

Compound **3bl** was synthesized following the General Procedure and subsequently purified using flash column chromatography (petroleum ether/EtOAc = 10/1), resulting in a colorless oil (12 mg) at 52% yield on a 0.1 mmol scale.

^{1}H NMR (400 MHz, $CDCl_3$) δ 8.69 (ddd, *J* = 4.8, 1.8, 0.9 Hz, 1H), 7.82 (td, *J* = 7.8, 1.8 Hz, 1H), 7.62 (dt, *J* = 7.9, 1.0 Hz, 1H), 7.43 (dd, *J* = 5.0, 1.2 Hz, 1H), 7.39 (ddd, *J* = 7.6, 4.8, 1.1 Hz, 1H), 7.36 (dt, *J* = 3.6, 1.0 Hz, 1H), 7.11 (dd, *J* = 5.1, 3.6 Hz, 1H), 4.78 (s, 1H).

^{13}C NMR (100 MHz, $CDCl_3$) δ 150.7, 150.3, 137.6, 134.6, 128.1, 127.6, 127.4, 124.9, 120.8, 115.6, 64.1, 59.0.

HRMS (ESI) *m/z* calcd. for $C_{12}H_8N_2NaOS$ $[M+Na]^+$: 251.0251; found: 251.0243.

$[\alpha]_D^{20}$ = −104.5 (*c* 1.0, $CHCl_3$)

The enantiomeric excess was determined by chiral HPLC analysis using a Daicel Chiralpak IC column, e.e. = 99% (*n*-hexane/ethanol = 90/10, flow rate = 1.0 mL/min, λ = 254 nm, T = 20 °C, t_r (major) = 10.271 min, t_r (minor) = 12.369 min).

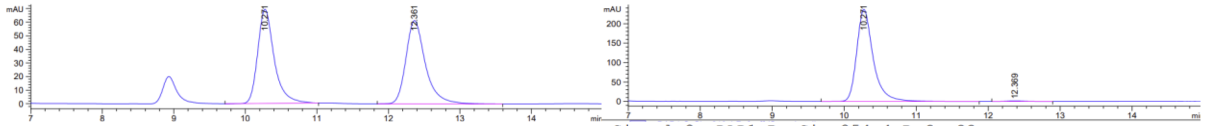

Signal 2: DAD1 B, Sig=254,4 Ref=off

Peak #	RetTime [min]	Type	Width [min]	Area [mAU*s]	Height [mAU]	Area %
1	10.271	BB	0.2411	1110.30713	69.24806	48.6614
2	12.361	BB	0.2868	1171.39392	61.32659	51.3386

Signal 2: DAD1 B, Sig=254,4 Ref=off

Peak #	RetTime [min]	Type	Width [min]	Area [mAU*s]	Height [mAU]	Area %
1	10.271	BB	0.2379	3771.38403	236.63229	99.2926
2	12.369	BB	0.2450	26.86806	1.50005	0.7074

Supplementary Figure 31. HPLC spectra of compound **3bl**

(2*S*,3*S*)-2-(5-Bromopyrimidin-2-yl)-3-methyloxirane-2-carbonitrile (3ca)

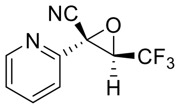

Compound **3ca** was synthesized following the General Procedure and subsequently purified using flash column chromatography (petroleum ether/EtOAc = 10/1), resulting in a yellow oil (21 mg) at 65% yield on a 0.1 mmol scale.

^{1}H NMR (400 MHz, $CDCl_3$) δ 8.83 (s, 2H), 3.89 (d, *J* = 5.0 Hz, 1H), 1.75 (d, *J* = 4.6 Hz, 3H).

^{13}C NMR (100 MHz, $CDCl_3$) δ 159.3, 158.7, 121.2, 115.2, 63.0, 55.4, 15.7.

HRMS (ESI) *m/z* calcd. for $C_8H_7BrN_3O$ $[M+H]^+$: 239.9767; found: 239.9777.

$[\alpha]_D^{20}$ = −78.8 (*c* 1.0, $CHCl_3$)

The enantiomeric excess was determined by chiral HPLC analysis using a Daicel Chiralpak IJ column, e.e. = 95% (*n*-hexane/ethanol = 90/10, flow rate = 1.0 mL/min, λ = 254 nm, T = 20 °C, t_r (major) = 20.778 min, t_r (minor) = 27.150 min).

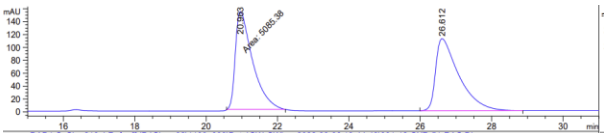

Signal 2: DAD1 B, Sig=254,4 Ref=off

Peak #	RetTime [min]	Type	Width [min]	Area [mAU*s]	Height [mAU]	Area %
1	20.963	MM	0.5593	5085.38428	151.54289	50.1708
2	26.612	BB	0.6456	5050.75439	111.67557	49.8292

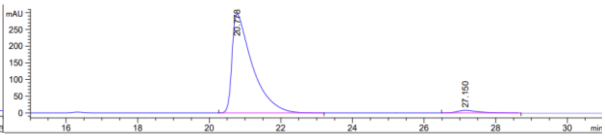

Signal 2: DAD1 B, Sig=254,4 Ref=off

Peak #	RetTime [min]	Type	Width [min]	Area [mAU*s]	Height [mAU]	Area %
1	20.778	BB	0.5611	1.16429e4	295.87622	97.5170
2	27.150	BB	0.5453	296.45880	7.59692	2.4830

Supplementary Figure 32. HPLC spectra of compound **3ca**

(2*R*,3*R*)-2-(Pyridin-2-yl)-3-(trifluoromethyl)oxirane-2-carbonitrile (3cb)

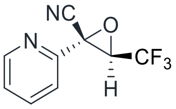

Compound **3cb** was synthesized following the General Procedure and subsequently purified using flash column chromatography (petroleum ether/EtOAc = 10/1), resulting in a yellow oil (24 mg) at 87% yield on a 0.1 mmol scale.

^{1}H NMR (400 MHz, $CDCl_3$) 8.67 (dt, *J* = 4.8, 0.9 Hz, 1H), 7.87 (td, *J* = 7.8, 1.7 Hz, 1H), 7.79 (dd, *J* = 7.9, 0.9 Hz, 1H), 7.44 (ddd, *J* = 7.6, 4.8, 1.1 Hz, 1H), 4.42 (q, *J* = 4.8 Hz, 1H).

^{13}C NMR (100 MHz, $CDCl_3$) δ 150.3, 147.7, 137.6 125.4, 122.1, 122.0, 119.2, 112.8, 59.8 (q, *J* = 42.6 Hz).

^{19}F NMR (376 MHz, $CDCl_3$) δ −73.4.

HRMS (ESI) *m/z* calcd. for $C_8H_7BrN_3NaO$ $[M+Na]^+$: 237.0246; found: 237.0246.

$[\alpha]_D^{20}$ = −8.1 (*c* 1.0, $CHCl_3$)

The enantiomeric excess was determined by chiral HPLC analysis using a Daicel Chiralpak IF column, e.e. = 98% (*n*-hexane/ethanol = 70/30, flow rate = 1.0 mL/min, λ = 250 nm, T = 20 °C, t_r (major) = 5.501 min, t_r (minor) = 5.115 min).

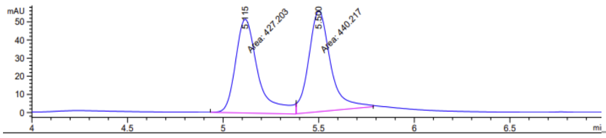

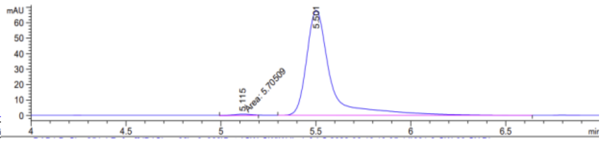

Signal 1: DAD1 A, Sig=250,4 Ref=off

Peak #	RetTime [min]	Type	Width [min]	Area [mAU*s]	Height [mAU]	Area %
1	5.115	MM	0.1377	427.20319	51.71160	49.2499
2	5.500	MM	0.1317	440.21677	55.69677	50.7501

Signal 1: DAD1 A, Sig=250,4 Ref=off

Peak #	RetTime [min]	Type	Width [min]	Area [mAU*s]	Height [mAU]	Area %
1	5.115	MM	0.1215	5.70509	7.82314e-1	0.9504
2	5.501	BB	0.1281	594.60962	67.94357	99.0496

Supplementary Figure 33. HPLC spectra of compound **3cb**

(2*R*,3*S*)-3-Phenethyl-2-(pyridin-2-yl)oxirane-2-carbonitrile (3cc)

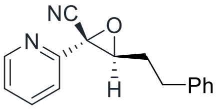

Compound **3cc** was synthesized following the General Procedure and subsequently purified using flash column chromatography (petroleum ether/EtOAc = 10/1), resulting in a colorless oil (21 mg) at 95% yield on a 0.1 mmol scale.

^{1}H NMR (400 MHz, $CDCl_3$) δ 8.64 (ddd, *J* = 4.8, 1.8, 1.1 Hz, 1H), 7.75 (td, *J* = 7.9, 1.8 Hz, 1H), 7.41 (dt, *J* = 7.9, 1.1 Hz, 1H), 7.37 – 7.27 (m, 3H), 7.25 – 7.18 (m, 3H), 2.95 (dt, *J* = 9.8, 7.5 Hz, 2H), 2.30 (tdd, *J* = 8.1, 6.5, 1.6 Hz, 2H).

^{13}C NMR (100 MHz, $CDCl_3$) δ 151.4, 149.8, 139.7, 137.2, 128.5, 128.3, 126.3, 124.2, 119.9, 115.8, 66.3, 55.2, 31.9, 31.8.

HRMS (ESI) *m/z* calcd. for $C_{16}H_{15}N_2O$ $[M+H]^+$: 251.1179; found: 251.1180.

$[\alpha]_D^{20}$ = −19.2 (*c* 1.0, $CHCl_3$)

The enantiomeric excess was determined by chiral HPLC analysis using a Daicel Chiralpak IC column, e.e. = 94% (*n*-hexane/ethanol = 90/10, flow rate = 1.0 mL/min, λ = 210 nm, T = 20 °C, t_r (major) = 14.800 min, t_r (minor) = 9.660 min).

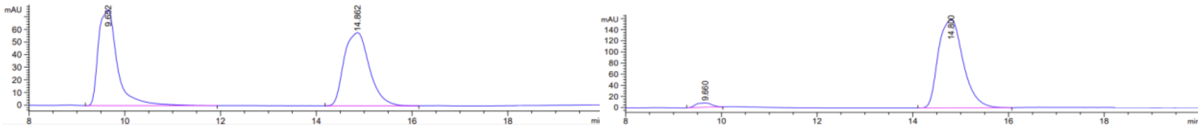

Signal 3: DAD1 C, Sig=210,4 Ref=off

Peak #	RetTime [min]	Type	Width [min]	Area [mAU*s]	Height [mAU]	Area %
1	9.652	BB	0.3709	2091.06934	76.03051	49.5366
2	14.862	BB	0.5884	2130.18921	58.01311	50.4634

Signal 3: DAD1 C, Sig=210,4 Ref=off

Peak #	RetTime [min]	Type	Width [min]	Area [mAU*s]	Height [mAU]	Area %
1	9.660	BB	0.3144	180.73483	7.97557	3.0773
2	14.800	BB	0.5682	5692.50439	158.80417	96.9227

Supplementary Figure 34. HPLC spectra of compound **3cc**

(2*R*,3*S*)-3-*iso*-Propyl-2-(pyridin-2-yl)oxirane-2-carbonitrile (3cd)

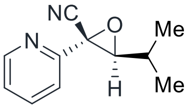

Compound **3cd** was synthesized following the General Procedure and subsequently purified using flash column chromatography (petroleum ether/EtOAc = 10/1), resulting in a yellow oil (18 mg) at 95% yield on a 0.1 mmol scale.

^{1}H NMR (400 MHz, $CDCl_3$) δ 8.66 (d, *J* = 4.2 Hz, 1H), 7.77 (td, *J* = 7.9, 1.6 Hz, 1H), 7.47 (d, *J* = 7.9 Hz, 1H), 7.34 (ddd, *J* = 7.7, 4.8, 1.1 Hz, 1H), 3.20 (d, *J* = 9.1 Hz, 1H), 2.06 – 1.90 (m, 1H), 1.24 (d, *J* = 6.7 Hz, 3H), 1.16 (d, *J* = 6.8 Hz, 3H).

^{13}C NMR (100 MHz, $CDCl_3$) δ 151.9, 150.1, 137.5, 124.5, 120.2, 116.3, 72.5, 55.5, 30.7, 19.7, 18.3.

HRMS (ESI) *m/z* calcd. for $C_{11}H_{12}N_2NaO$ $[M+Na]^+$: 211.0842; found: 211.0833.

$[\alpha]_D^{20}$ = −47.0 (*c* 1.0, $CHCl_3$)

The enantiomeric excess was determined by chiral HPLC analysis using a Daicel Chiralpak IC column, e.e. = 98% (*n*-hexane/ethanol = 90/10, flow rate = 1.0 mL/min, λ = 254nm, T = 20 ºC, t_r (major) = 7.669 min, t_r (minor) = 6.622 min).

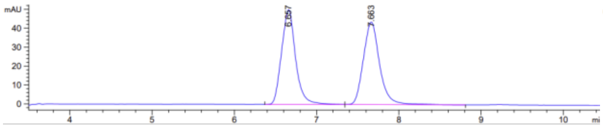

Signal 2: DAD1 B, Sig=254,4 Ref=off

Peak #	RetTime [min]	Type	Width [min]	Area [mAU*s]	Height [mAU]	Area %
1	6.657	BB	0.1843	594.51770	50.16508	49.7510
2	7.663	BB	0.2104	600.46875	43.66597	50.2490

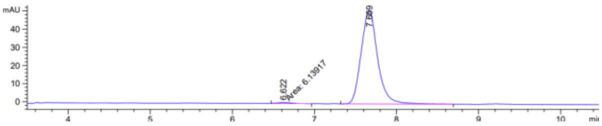

Signal 2: DAD1 B, Sig=254,4 Ref=off

Peak #	RetTime [min]	Type	Width [min]	Area [mAU*s]	Height [mAU]	Area %
1	6.622	MM	0.1912	6.13917	5.35233e-1	0.8477
2	7.669	BB	0.2141	718.03516	51.66844	99.1523

Supplementary Figure 35. HPLC spectra of compound **3cd**

(2*R*,3*S*)-3-Cyclopropyl-2-(pyrazin-2-yl)oxirane-2-carbonitrile (3ce)

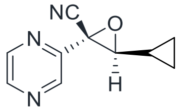

Compound **3ce** was synthesized following the General Procedure and subsequently purified using flash column chromatography (petroleum ether/EtOAc = 10/1), resulting in a yellow oil (12 mg) at 65% yield on a 0.1 mmol scale.

^{1}H NMR (400 MHz, $CDCl_3$) δ 8.72 (d, *J* = 1.5 Hz, 1H), 8.63 (d, *J* = 2.5 Hz, 1H), 8.59 (dd, *J* = 2.5, 1.5 Hz, 1H), 3.19 (d, *J* = 6.7 Hz, 1H), 1.25 (tdt, *J* = 8.2, 6.8, 4.9 Hz, 1H), 0.91 – 0.81 (m, 2H), 0.80 – 0.71 (m, 1H), 0.56 (dddd, *J* = 10.0, 5.3, 3.4, 1.8 Hz, 1H).

^{13}C NMR (100 MHz, $CDCl_3$) δ 147.6, 145.7, 144.2, 142.0, 115.4, 70.1, 53.9, 10.1, 3.5, 3.0.

HRMS (ESI) *m/z* calcd. for $C_{10}H_9N_3NaO$ $[M+Na]^+$: 210.0638; found: 210.0630.

$[\alpha]_D^{20}$ = −24.3 (*c* 1.0, $CHCl_3$)

The enantiomeric excess was determined by chiral HPLC analysis using a Daicel Chiralpak IA column, e.e. = 98% (*n*-hexane/ethanol = 70/30, flow rate = 1.0 mL/min, λ = 254 nm, T = 20 °C, t_r (major) = 14.230 min, t_r (minor) = 10.976 min).

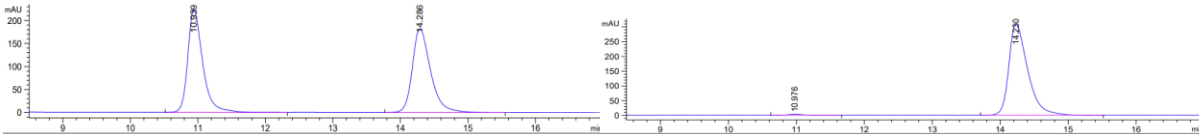

Signal 2: DAD1 B, Sig=254,4 Ref=off

Peak #	RetTime [min]	Type	Width [min]	Area [mAU*s]	Height [mAU]	Area %
1	10.939	BB	0.2308	3422.52612	223.38045	49.9761
2	14.286	BB	0.2851	3425.79663	182.41956	50.0239

Signal 2: DAD1 B, Sig=254,4 Ref=off

Peak #	RetTime [min]	Type	Width [min]	Area [mAU*s]	Height [mAU]	Area %
1	10.976	BB	0.2210	48.79036	3.32839	0.8114
2	14.230	BB	0.2872	5964.59717	311.75989	99.1886

Supplementary Figure 36. HPLC spectra of compound **3ce**

(2*R*,3*S*)-2-(Benzo[*d*]thiazol-2-yl)-3-cyclopentyloxirane-2-carbonitrile (3cf)

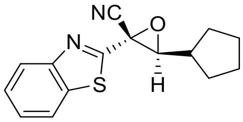

Compound **3cf** was synthesized following the General Procedure and subsequently purified using flash column chromatography (petroleum ether/EtOAc = 10/1), resulting in a colorless oil (25 mg) at 93% yield on a 0.1 mmol scale.

^{1}H NMR (400 MHz, $CDCl_3$) δ 8.11 (d, *J* = 8.2 Hz, 1H), 7.90 (d, *J* = 8.2 Hz, 1H), 7.55 (t, *J* = 7.7 Hz, 1H), 7.46 (t, *J* = 7.6 Hz, 1H), 3.43 (d, *J* = 8.7 Hz, 1H), 2.20 (h, *J* = 8.0 Hz, 1H), 2.08 (tq, *J* = 9.7, 5.1, 4.1 Hz, 2H), 1.71 (dddd, *J* = 17.3, 14.2, 10.5, 4.9 Hz, 5H), 1.50 (dt, *J* = 12.7, 7.6 Hz, 1H).

^{13}C NMR (100 MHz, $CDCl_3$) δ 163.2, 153.1, 134.7, 126.9, 126.3, 123.9, 121.9, 114.7, 73.2, 53.3, 40.8, 30.5, 28.4, 25.5, 25.4.

HRMS (ESI) *m/z* calcd. for $C_{15}H_{15}N_2OS$ $[M+H]^+$: 271.0900; found: 271.0901.

$[\alpha]_D^{20}$ = −24.3 (*c* 1.0, $CHCl_3$)

The enantiomeric excess was determined by chiral HPLC analysis using a Daicel Chiralpak IA column, e.e. = 95% (*n*-hexane/ethanol = 70/30, flow rate = 1.0 mL/min, λ = 254 nm, T = 20 °C, t_r (major) = 5.198 min, t_r (minor) = 4.743 min).

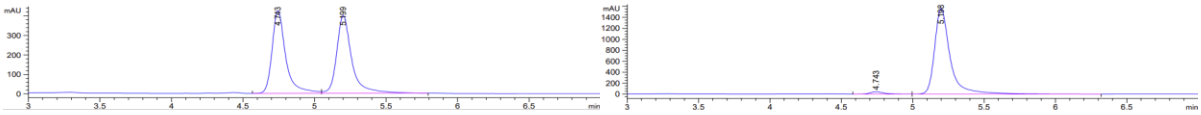

Signal 2: DAD1 B, Sig=254,4 Ref=off

Peak #	RetTime [min]	Type	Width [min]	Area [mAU*s]	Height [mAU]	Area %
1	4.743	BV	0.0975	2820.92798	424.39996	49.1205
2	5.199	VB	0.1076	2921.94507	398.45538	50.8795

Signal 2: DAD1 B, Sig=254,4 Ref=off

Peak #	RetTime [min]	Type	Width [min]	Area [mAU*s]	Height [mAU]	Area %
1	4.743	BV	0.1006	298.08481	44.22412	2.4519
2	5.198	VB	0.1128	1.18591e4	1557.73157	97.5481

Supplementary Figure 37. HPLC spectra of compound **3cf**

(2*S*,3*S*)-2-(5-Bromopyrimidin-2-yl)-3-(4-chlorobutyl)oxirane-2-carbonitrile (3cg)

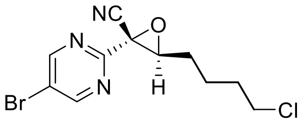

Compound **3cg** was synthesized following the General Procedure and subsequently purified using flash column chromatography (petroleum ether/EtOAc = 3/1), resulting in a white solid (30 mg) at 94% yield on a 0.1 mmol scale.

m.p.: 40.1 − 41.6 °C

^{1}H NMR (400 MHz, $CDCl_3$) δ 8.84 (s, 2H), 3.79 (t, *J* = 5.9 Hz, 1H), 3.59 (t, *J* = 6.2 Hz, 2H), 2.08 (dt, *J* = 14.0, 7.0 Hz, 1H), 2.00 (dd, *J* = 15.0, 7.8 Hz, 1H), 1.92 (dt, *J* = 12.4, 6.2 Hz, 2H), 1.84 − 1.76 (m, 2H).

^{13}C NMR (100 MHz, $CDCl_3$) δ 158.8, 158.5, 121.0, 114.9, 66.1, 54.6, 44.2, 31.7, 29.2, 23.0.

HRMS (ESI) *m/z* calcd. for $C_{11}H_{11}BrClN_3Na$ $[M+Na]^+$: 337.9666; found: 337.9674.

$[\alpha]_D^{20}$ = −101.7 (*c* 1.0, $CHCl_3$)

The enantiomeric excess was determined by chiral HPLC analysis using a Daicel Chiralpak IJ column, e.e. = 99% (*n*-hexane/ethanol = 90/10, flow rate = 1.0 mL/min, λ = 254 nm, T = 20 °C, t_r (major) = 33.377 min, t_r (minor) = 35.872 min).

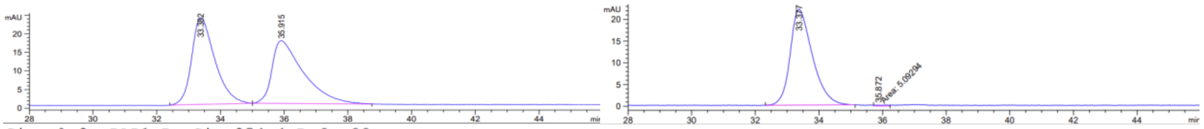

Signal 2: DAD1 B, Sig=254,4 Ref=off

Peak #	RetTime [min]	Type	Width [min]	Area [mAU*s]	Height [mAU]	Area %
1	33.382	BB	0.7200	1178.20007	23.29955	50.3616
2	35.915	BB	0.8433	1161.27881	16.86655	49.6384

Signal 2: DAD1 B, Sig=254,4 Ref=off

Peak #	RetTime [min]	Type	Width [min]	Area [mAU*s]	Height [mAU]	Area %
1	33.377	BB	0.6808	1070.89465	22.04333	99.5267
2	35.872	MM	0.4498	5.09294	1.88718e-1	0.4733

Supplementary Figure 38. HPLC spectra of compound **3cg**

(2*R*,3*S*)-2-(5-Bromopyridin-2-yl)-3-((1,3-dioxoisoindolin-2-yl)methyl)oxirane-2-carbonitrile (3ch)

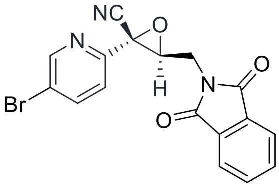

Compound **3ch** was synthesized following the General Procedure and subsequently purified using flash column chromatography (petroleum ether/EtOAc = 3/1), resulting in a white solid (34 mg) at 82% yield on a 0.1 mmol scale.

m.p.: 147.2 − 150.3 °C

^{1}H NMR (400 MHz, $CDCl_3$) δ 8.67 (s, 1H), 7.97 – 7.81 (m, 3H), 7.76 (dd, *J* = 5.6, 2.8 Hz, 2H), 7.41 (d, *J* = 8.4 Hz, 1H), 4.31 (ddd, *J* = 15.0, 5.6, 2.2 Hz, 1H), 4.19 (dd, *J* = 15.1, 4.3 Hz, 1H), 3.84 (t, *J* = 5.0 Hz, 1H).

^{13}C NMR (100 MHz, $CDCl_3$) δ 167.7, 151.4, 149.0, 140.2, 134.6, 134.4, 131.9, 123.8, 123.7, 122.3, 121.9, 115.0, 63.2, 54.7, 37.9.

HRMS (ESI) *m/z* calcd. for $C_{17}H_{10}N_3NaO$ $[M+Na]^+$: 405.9798; found: 405.9805.

$[\alpha]_D^{20}$ = −60.4 (*c* 1.0, $CHCl_3$)

The enantiomeric excess was determined by chiral HPLC analysis using a Daicel Chiralpak IJ column, e.e. = 87% (*n*-hexane/ethanol = 70/30 flow rate = 1.0 mL/min, λ = 254 nm, T = 20 ºC, t_r (major) = 18.117 min, t_r (minor) = 13.410 min).

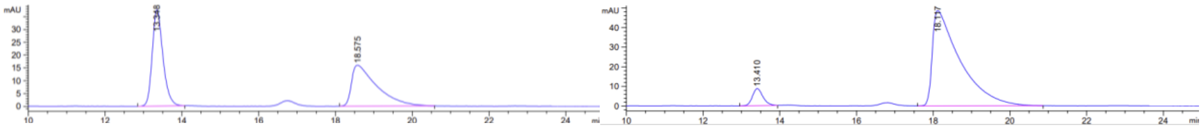

Signal 2: DAD1 B, Sig=254,4 Ref=off

Peak #	RetTime [min]	Type	Width [min]	Area [mAU*s]	Height [mAU]	Area %
1	13.348	BB	0.2938	727.57330	37.59568	49.9200
2	18.575	BB	0.5947	729.90515	15.98684	50.0800

Signal 2: DAD1 B, Sig=254,4 Ref=off

Peak #	RetTime [min]	Type	Width [min]	Area [mAU*s]	Height [mAU]	Area %
1	13.410	BB	0.2911	166.38995	8.70034	6.3797
2	18.117	BB	0.6692	2441.73682	48.51471	93.6203

Supplementary Figure 39. HPLC spectra of compound **3ch**

(2*R*,3*S*)-3-((Benzyloxy)methyl)-2-(5-bromopyridin-2-yl)oxirane-2-carbonitrile (3ci)

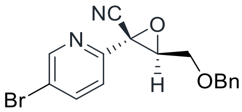

Compound **3ci** was synthesized following the General Procedure and subsequently purified using flash column chromatography (petroleum ether/EtOAc = 10/1), resulting in a white solid (29 mg) at 86% yield on a 0.1 mmol scale.

m.p.: 62.1 – 70.3 °C

^{1}H NMR (400 MHz, $CDCl_3$) δ 8.69 (d, *J* = 2.1 Hz, 1H), 7.90 (dd, *J* = 8.4, 2.3 Hz, 1H), 7.43 – 7.28 (m, 6H), 4.76 – 4.58 (m, 2H), 4.02 (dd, *J* = 11.7, 4.6 Hz, 1H), 3.91 (dd, *J* = 11.7, 5.5 Hz, 1H), 3.74 (t, *J* = 5.1 Hz, 1H).

^{13}C NMR (100 MHz, $CDCl_3$) δ 151.3, 149.5, 140.2, 137.2, 128.7, 128.2, 128.1, 122.1, 121.5, 115.2, 74.0, 68.6, 64.9, 53.4.

HRMS (ESI) *m/z* calcd. for $C_{16}H_{14}BrN_2O_2$ $[M+H]^+$: 345.0233; found: 345.0240.

$[\alpha]_D^{20}$ = −36.5 (*c* 1.0, $CHCl_3$)

The enantiomeric excess was determined by chiral HPLC analysis using a Daicel Chiralpak IA column, e.e. = 97% (*n*-hexane/ethanol = 70/30, flow rate = 1.0 mL/min, λ = 254 nm, T = 20 ºC, t_r (major) = 13.109 min, t_r (minor) = 16.598 min).

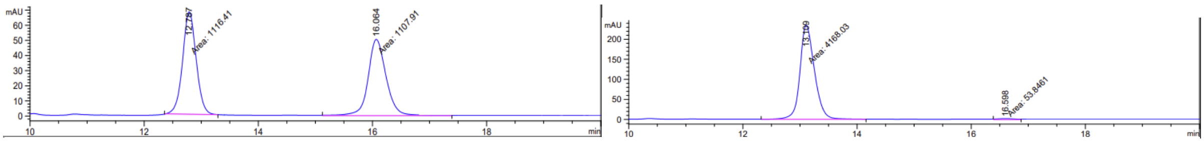

Signal 2: DAD1 B, Sig=254,4 Ref=off

Peak #	RetTime [min]	Type	Width [min]	Area [mAU*s]	Height [mAU]	Area %
1	12.787	MM	0.2754	1116.40527	67.55482	50.1909
2	16.064	MM	0.3667	1107.91418	50.35970	49.8091

Signal 2: DAD1 B, Sig=254,4 Ref=off

Peak #	RetTime [min]	Type	Width [min]	Area [mAU*s]	Height [mAU]	Area %
1	13.109	MM	0.2952	4168.02930	235.32846	98.7246
2	16.598	MM	0.3689	53.84605	2.43256	1.2754

Supplementary Figure 40. HPLC spectra of compound **3ci**

(2*R*,3*S*)-2-(5-Bromopyridin-2-yl)-3-(((tert-butyldimethylsilyl)oxy)methyl)oxirane-2-carbonitrile (3cj)

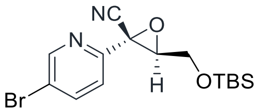

Compound **3cj** was synthesized following the General Procedure and subsequently purified using flash column chromatography (petroleum ether/EtOAc = 10/1), resulting in a colorless oil (31 mg) at 85% yield on a 0.1 mmol scale.

^{1}H NMR (400 MHz, $CDCl_3$) δ 8.69 (dd, *J* = 2.3, 0.7 Hz, 1H), 7.90 (dd, *J* = 8.4, 2.3 Hz, 1H), 7.36 (dd, *J* = 8.4, 0.7 Hz, 1H), 4.13 (dd, *J* = 12.1, 4.6 Hz, 1H), 4.03 (dd, *J* = 12.2, 5.3 Hz, 1H), 3.64 (dd, *J* = 5.3, 4.6 Hz, 1H), 0.91 (s, 9H), 0.13 (d, *J* = 2.5 Hz, 6H).

^{13}C NMR (100 MHz, $CDCl_3$) δ 151.3, 150.0, 140.2, 122.1, 121.5, 115.3, 66.6, 62.4, 53.7, 25.9, 18.4, −5.2, −5.2.

HRMS (ESI) *m/z* calcd. for $C_{15}H_{22}BrN_2O_2Si$ $[M+H]^+$: 369.0628; found: 369.0640.

$[\alpha]_D^{20}$ = −60.4 (*c* 1.0, $CHCl_3$)

The enantiomeric excess was determined by chiral HPLC analysis using a Daicel Chiralpak IA column, e.e. = 98% (*n*-hexane/ethanol = 98/2, flow rate = 1.0 mL/min, λ = 230 nm, T = 20 ºC, t_r (major) = 6.468 min, t_r (minor) = 7.427 min).

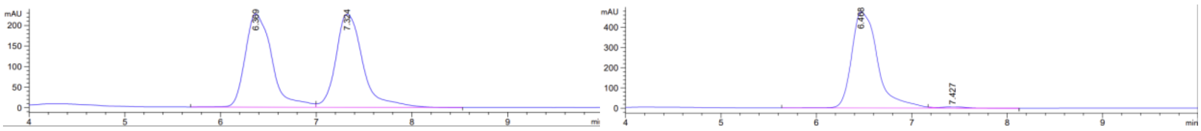

Signal 4: DAD1 D, Sig=230,4 Ref=off

Peak #	RetTime [min]	Type	Width [min]	Area [mAU*s]	Height [mAU]	Area %
1	6.369	BV	0.2730	4374.08984	226.80376	49.7021
2	7.324	VB	0.3005	4426.51660	226.03464	50.2979

Signal 4: DAD1 D, Sig=230,4 Ref=off

Peak #	RetTime [min]	Type	Width [min]	Area [mAU*s]	Height [mAU]	Area %
1	6.468	BV R	0.2956	9016.08496	474.74435	98.9035
2	7.427	VB E	0.2615	99.96176	5.90363	1.0965

Supplementary Figure 41. HPLC spectra of compound **3cj**

Methyl 3-((2*S*,3*S*)-3-(5-bromopyrimidin-2-yl)-3-cyanooxiran-2-yl)propanoate (3ck)

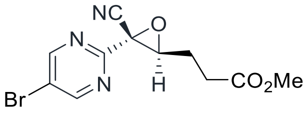

Compound **3ck** was synthesized following the General Procedure and subsequently purified using flash column chromatography (petroleum ether/EtOAc = 5/1), resulting in a white solid (23 mg) at 90% yield on a 0.1 mmol scale.

m.p.: 103.5 − 113.7 °C

^{1}H NMR (400 MHz, $CDCl_3$) δ 8.84 (s, 2H), 3.92 (t, *J* = 5.5 Hz, 1H), 3.71 (s, 3H), 2.67 (t, *J* = 6.8 Hz, 2H), 2.42 − 2.32 (m, 1H), 2.28 − 2.15 (m, 1H), 1.61 (s, 1H).

^{13}C NMR (100 MHz, $CDCl_3$) δ 172.4, 159.0, 158.8, 121.3, 115.0, 65.6, 55.4, 52.2, 30.0, 25.6.

HRMS (ESI) *m/z* calcd. for $C_{11}H_{10}BrN_3NaO_3$ $[M+Na]^+$: 333.9798; found: 335.9778.

$[\alpha]_D^{20}$ = −47.4 (*c* 1.0, $CHCl_3$)

The enantiomeric excess was determined by chiral HPLC analysis using a Daicel Chiralpak IB column, e.e. = 99% (*n*-hexane/ethanol = 90/10, flow rate = 1.0 mL/min, λ = 210 nm, T = 20 °C, t_r (major) = 16.766 min, t_r (minor) = 18.005 min).

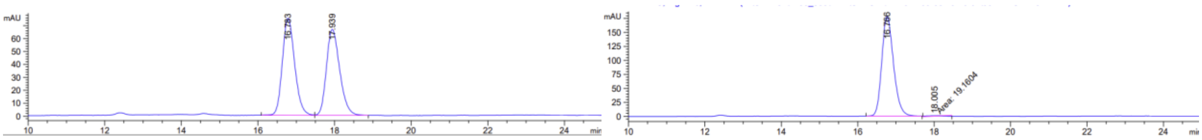

Signal 3: DAD1 C, Sig=210,4 Ref=off

Peak #	RetTime [min]	Type	Width [min]	Area [mAU*s]	Height [mAU]	Area %
1	16.783	BV	0.3444	1675.10535	75.04984	51.1204
2	17.939	VB	0.3710	1601.68140	66.49968	48.8796

Signal 3: DAD1 C, Sig=210,4 Ref=off

Peak #	RetTime [min]	Type	Width [min]	Area [mAU*s]	Height [mAU]	Area %
1	16.766	BB	0.3288	3794.20483	177.92491	99.4975
2	18.005	MM	0.4446	19.16041	7.18333e-1	0.5025

Supplementary Figure 42. HPLC spectra of compound **3ck**

(2*R*,3*S*)-2-(5-Bromopyridin-2-yl)-3-(but-3-yn-1-yl)oxirane-2-carbonitrile (3cl)

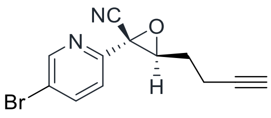

Compound **3cl** was synthesized following the General Procedure and subsequently purified using flash column chromatography (petroleum ether/EtOAc = 10/1), resulting in a yellow oil (30 mg) at 93% yield on a 0.1 mmol scale.

^{1}H NMR (400 MHz, $CDCl_3$) δ 8.70 (d, *J* = 2.3 Hz, 1H), 7.91 (dd, *J* = 8.4, 2.3 Hz, 1H), 7.36 (d, *J* = 8.4 Hz, 1H), 3.63 (dd, *J* = 6.6, 5.3 Hz, 1H), 2.54 (tq, *J* = 6.2, 2.0 Hz, 2H), 2.36 − 2.07 (m, 2H), 2.05 (t, *J* = 2.6 Hz, 1H).

^{13}**C NMR** (100 MHz, $CDCl_3$) δ 151.3, 150.1, 140.2, 122.0, 121.4, 115.7, 81.7, 70.5, 66.1, 55.1, 29.4, 15.3.

HRMS (ESI) *m/z* calcd. for $C_{12}H_{10}BrN_2O$ $[M+H]^+$: 276.9971; found: 276.9979.

$[\alpha]_D^{20}$ = −13.8 (*c* 1.0, $CHCl_3$)

The enantiomeric excess was determined by chiral HPLC analysis using a Daicel Chiralpak IB column, e.e. = 98% (*n*-hexane/ethanol = 99/1, flow rate = 1.0 mL/min, λ = 210 nm, T = 20 °C, t_r (major) = 10.877 min, t_r (minor) = 11.898 min).

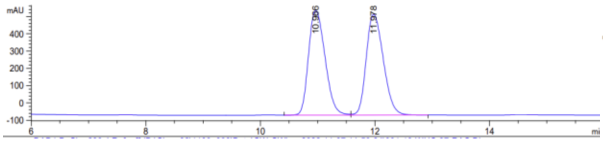

Signal 3: DAD1 C, Sig=210,4 Ref=off

Peak #	RetTime [min]	Type	Width [min]	Area [mAU*s]	Height [mAU]	Area %
1	10.966	BV	0.3180	1.21371e4	605.24579	50.1561
2	11.978	VB	0.3277	1.20616e4	582.20087	49.8439

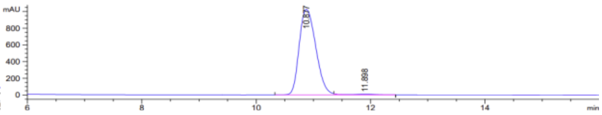

Signal 3: DAD1 C, Sig=210,4 Ref=off

Peak #	RetTime [min]	Type	Width [min]	Area [mAU*s]	Height [mAU]	Area %
1	10.877	BV R	0.3209	2.06403e4	1025.02197	98.9838
2	11.898	VB E	0.3505	211.90852	9.13811	1.0162

Supplementary Figure 43. HPLC spectra of compound **3cl**

(2*R*,3*S*)-2-(5-Bromopyridin-2-yl)-3-((trimethylsilyl)ethynyl)oxirane-2-carbonitrile (3cm)

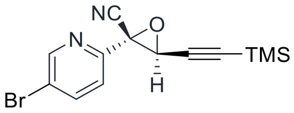

Compound **3cm** was synthesized following the General Procedure and subsequently purified using flash column chromatography (petroleum ether/EtOAc = 10/1), resulting in a yellow oil (11 mg) at 53% yield on a 0.1 mmol scale.

^{1}**H NMR** (400 MHz, DMSO-d_6) δ 8.82 (s, 1H), 8.21 (d, *J* = 8.5 Hz, 1H), 7.36 (d, *J* = 8.5 Hz, 1H), 4.54 (s, 1H), 0.22 (s, 9H).

^{13}**C NMR** (100 MHz, DMSO-d_6) δ 150.4, 148.4, 140.7, 122.0, 121.6, 114.9, 97.2, 94.2, 56.6, 53.5, −0.7.

HRMS (ESI) *m/z* calcd. for $C_{13}H_{13}BrN_2NaOSi$ $[M+H]^+$: 342.9873; found: 342.9881.

$[\alpha]_D^{20}$ = −13.8 (*c* 1.0, $CHCl_3$)

The enantiomeric excess was determined by chiral HPLC analysis using a Daicel Chiralpak OJ-H column, e.e. = 99% (*n*-hexane/ethanol = 99/1, flow rate = 1.0 mL/min, λ = 254 nm, T = 20 °C, t_r (major) = 11.469 min, t_r (minor) = 12.863 min).

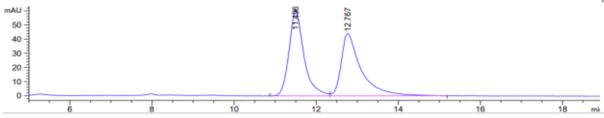

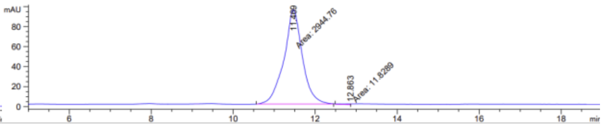

Signal 2: DAD1 B, Sig=254,4 Ref=off

Peak #	RetTime [min]	Type	Width [min]	Area [mAU*s]	Height [mAU]	Area %
1	11.496	BV	0.3710	1530.84790	60.53813	50.8639
2	12.767	VB	0.4800	1478.84583	44.00903	49.1361

Signal 2: DAD1 B, Sig=254,4 Ref=off

Peak #	RetTime [min]	Type	Width [min]	Area [mAU*s]	Height [mAU]	Area %
1	11.469	MM	0.5206	2944.75830	94.27325	99.5999
2	12.863	MM	0.2569	11.82889	7.67309e-1	0.4001

Supplementary Figure 44. HPLC spectra of compound **3cm**

Ethyl (2*R*,3*R*)-3-(Benzo[*d*]thiazol-2-yl)-3-cyanooxirane-2-carboxylate (3cn)

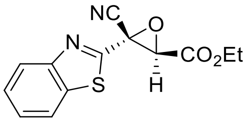

Compound **3cn** was synthesized following the General Procedure and subsequently purified using flash column chromatography (petroleum ether/EtOAc = 10/1), resulting in a yellow oil (26 mg) at 93% yield on a 0.1 mmol scale.

^{1}H NMR (400 MHz, $CDCl_3$) δ 8.12 (d, *J* = 8.2 Hz, 1H), 7.92 (d, *J* = 7.5 Hz, 1H), 7.57 (ddd, *J* = 8.3, 7.3, 1.3 Hz, 1H), 7.50 (ddd, *J* = 8.3, 7.3, 1.3 Hz, 1H), 4.53 – 4.34 (m, 2H), 4.24 (s, 1H), 1.40 (t, *J* = 7.1 Hz, 3H).

^{13}C NMR (100 MHz, $CDCl_3$) δ 163.0, 160.2, 153.1, 135.1, 127.4, 127.1, 124.4, 122.1, 112.7, 63.5, 61.6, 51.6, 14.2.

HRMS (ESI) *m/z* calcd. for $C_{13}H_{11}N_2O_3S$ $[M+H]^+$: 275.0485; found: 275.0490.

$[\alpha]_D^{20}$ = −206.6 (*c* 1.0, $CHCl_3$)

The enantiomeric excess was determined by chiral HPLC analysis using a Daicel Chiralpak IA column, e.e. = 99% (*n*-hexane/ethanol = 70/30, flow rate = 1.0 mL/min, λ = 254 nm, T = 20 ºC, t_r (major) = 7.492 min, t_r (minor) = 8.723 min).

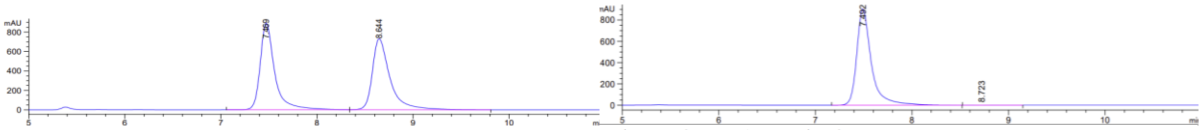

Signal 2: DAD1 B, Sig=254,4 Ref=off

Peak #	RetTime [min]	Type	Width [min]	Area [mAU*s]	Height [mAU]	Area %
1	7.469	BB	0.1568	9375.88477	890.58661	50.2165
2	8.644	BB	0.1885	9295.03809	730.01868	49.7835

Signal 2: DAD1 B, Sig=254,4 Ref=off

Peak #	RetTime [min]	Type	Width [min]	Area [mAU*s]	Height [mAU]	Area %
1	7.492	BB	0.1573	9550.67969	903.50623	99.6687
2	8.723	BB	0.1748	31.74820	2.87639	0.3313

Supplementary Figure 45. HPLC spectra of compound **3cn**

(2*R*,3*R*)-2-(5-Bromopyridin-2-yl)-3-(dimethoxymethyl)oxirane-2-carbonitrile (3co)

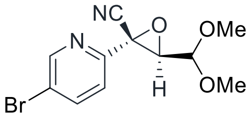

Compound **3co** was synthesized following the General Procedure and subsequently purified using flash column chromatography (petroleum ether/EtOAc = 10/1), resulting in a colorless oil (27 mg) at 90% yield on a 0.1 mmol scale.

^{1}H NMR (400 MHz, $CDCl_3$) δ 8.70 (dd, *J* = 2.3, 0.7 Hz, 1H), 7.91 (dd, *J* = 8.4, 2.3 Hz, 1H), 7.39 (dd, *J* = 8.4, 0.7 Hz, 1H), 4.55 (d, *J* = 6.0 Hz, 1H), 3.66 (d, *J* = 6.0 Hz, 1H), 3.55 (s, 3H), 3.53 (s, 3H).

^{13}C NMR (100 MHz, $CDCl_3$) δ 151.4, 149.2, 140.2, 122.3, 121.8, 115.1, 102.5, 65.1, 55.7, 54.9, 52.5.

HRMS (ESI) *m/z* calcd. for $C_{11}H_{11}BrN_2NaO_3$ $[M+Na]^+$: 320.9845; found: 320.9844.

$[\alpha]_D^{20}$ = −64.0 (*c* 1.0, $CHCl_3$)

The enantiomeric excess was determined by chiral HPLC analysis using a Daicel Chiralpak IJ column, e.e. = 99% (*n*-hexane/ethanol = 90/10, flow rate = 1.0 mL/min, λ = 210 nm, T = 20 °C, t_r (major) = 13.345 min, t_r (minor) = 12.473 min).

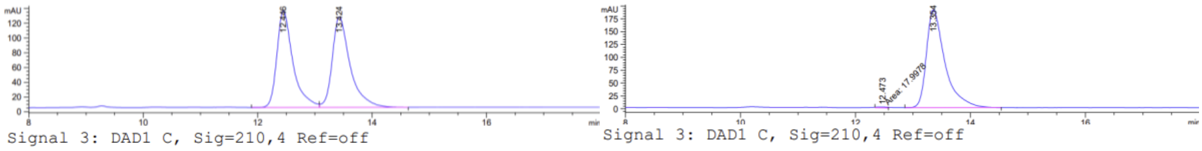

Signal 3: DAD1 C, Sig=210,4 Ref=off

Peak #	RetTime [min]	Type	Width [min]	Area [mAU*s]	Height [mAU]	Area %
1	12.446	BV	0.2925	2566.38501	131.01044	49.0351
2	13.424	VB	0.3237	2667.38477	122.68110	50.9649

Signal 3: DAD1 C, Sig=210,4 Ref=off

Peak #	RetTime [min]	Type	Width [min]	Area [mAU*s]	Height [mAU]	Area %
1	12.473	MM	0.2087	17.99782	1.43740	0.4229
2	13.354	BB	0.3342	4237.73584	190.03482	99.5771

Supplementary Figure 46. HPLC spectra of compound **3co**

(*R*)-2-(benzo[*d*]oxazol-2-yl)-1-oxaspiro[2.3]hexane-2-carbonitrile (3da)

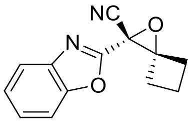

Compound **3da** was synthesized following the General Procedure and subsequently purified using flash column chromatography (petroleum ether/EtOAc = 10/1), resulting in a colorless oil (21 mg) at 95% yield on a 0.1 mmol scale.

^{1}H NMR (400 MHz, $CDCl_3$) δ 7.84 − 7.76 (m, 1H), 7.60 − 7.53 (m, 1H), 7.47 − 7.35 (m, 2H), 2.94 − 2.82 (m, 1H), 2.86 − 2.77 (m, 2H), 2.63 − 2.51 (m, 1H), 2.22 − 2.04 (m, 1H), 2.04 − 1.87 (m, 1H).

^{13}C NMR (100 MHz, $CDCl_3$) δ 156.5, 151.2, 140.7, 126.6, 125.5, 121.0, 114.2, 111.2, 74.0, 51.2, 30.2, 29.5, 12.3.

HRMS (ESI) *m/z* calcd. for $C_{13}H_{11}N_2O_2$ $[M+H]^+$: 227.0815; found: 227.0808.

$[\alpha]_D^{20}$ = −127.1 (*c* 1.0, $CHCl_3$)

The enantiomeric excess was determined by chiral HPLC analysis using a Daicel Chiralpak IA column, e.e. = 94% (*n*-hexane/ethanol = 80/20, flow rate = 1.0 mL/min, λ = 254 nm, T = 20 °C, t_r (major) = 6.357 min, t_r (minor) = 5.604 min).

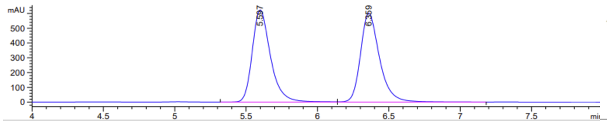

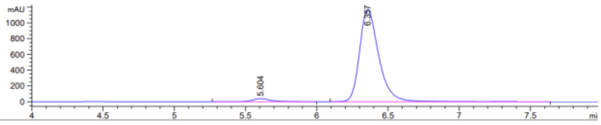

Signal 2: DAD1 B, Sig=254,4 Ref=off

Peak #	RetTime [min]	Type	Width [min]	Area [mAU*s]	Height [mAU]	Area %
1	5.597	BV	0.1357	5541.69238	623.36383	49.9196
2	6.359	VB	0.1419	5559.53809	601.13568	50.0804

Signal 2: DAD1 B, Sig=254,4 Ref=off

Peak #	RetTime [min]	Type	Width [min]	Area [mAU*s]	Height [mAU]	Area %
1	5.604	BB	0.1325	323.19934	37.50174	2.8686
2	6.357	BV R	0.1409	1.09435e4	1171.06885	97.1314

Supplementary Figure 47. HPLC spectra of compound **3da**

(*R*)-2-(Benzo[*d*]thiazol-2-yl)-1-oxaspiro[2.4]heptane-2-carbonitrile (3db)

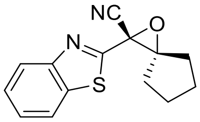

Compound **3db** was synthesized following the General Procedure and subsequently purified using flash column chromatography (petroleum ether/EtOAc = 10/1), resulting in a white solid (24 mg) at 94% yield on a 0.1 mmol scale.

m.p.: 186.5 − 187.4 °C

^{1}H NMR (600 MHz, $CDCl_3$) δ 8.11 (d, *J* = 8.2 Hz, 1H), 7.90 (d, *J* = 8.2 Hz, 1H), 7.55 (t, *J* = 7.6 Hz, 1H), 7.46 (t, *J* = 7.6 Hz, 1H), 2.43 (dt, *J* = 14.9, 7.4 Hz, 1H), 2.05 (dt, *J* = 14.7, 7.3 Hz, 1H), 1.94 (ddt, *J* = 29.9, 18.3, 6.6 Hz, 2H), 1.87 − 1.76 (m, 3H), 1.75 − 1.69 (m, 1H).

^{13}C NMR (150 MHz, $CDCl_3$) δ 162.9, 153.4, 134.9, 126.9, 126.4, 123.9, 121.9, 115.6, 80.5, 56.4, 32.9, 29.8, 25.5, 25.3.

HRMS (ESI) *m/z* calcd. for $C_{14}H_{13}N_2OS$ $[M+H]^+$: 257.0746; found: 257.0743.

$[\alpha]_D^{20}$ = −67.7 (*c* 1.0, $CHCl_3$)

The enantiomeric excess was determined by chiral HPLC analysis using a Daicel Chiralpak IA column, e.e. = 93% (*n*-hexane/ethanol = 80/20, flow rate = 1.0 mL/min, λ = 254 nm, T = 20 °C, t_r (major) = 5.832 min, t_r (minor) = 4.705 min).

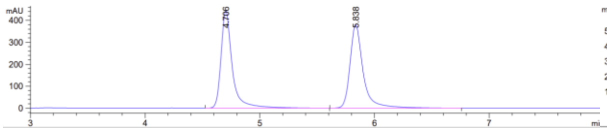

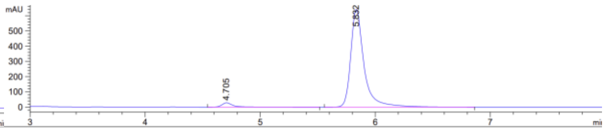

Signal 2: DAD1 B, Sig=254,4 Ref=off

Peak #	RetTime [min]	Type	Width [min]	Area [mAU*s]	Height [mAU]	Area %
1	4.706	BB	0.1014	3024.38501	444.49075	50.0251
2	5.838	BB	0.1190	3021.34912	379.07492	49.9749

Signal 2: DAD1 B, Sig=254,4 Ref=off

Peak #	RetTime [min]	Type	Width [min]	Area [mAU*s]	Height [mAU]	Area %
1	4.705	BB	0.1016	193.30682	28.33890	3.6160
2	5.832	BB	0.1197	5152.56348	641.52100	96.3840

Supplementary Figure 48. HPLC spectra of compound **3db**

(*R*)-2-(Benzo[*d*]oxazol-2-yl)-1-oxaspiro[2.5]octane-2-carbonitrile (3dc)

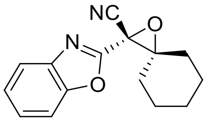

Compound **3dc** was synthesized following the General Procedure and subsequently purified using flash column chromatography (petroleum ether/EtOAc = 10/1), resulting in a yellow solid (24 mg) at 94% yield on a 0.1 mmol scale.

m.p.: 101.3 − 103.7 °C

^{1}H NMR (400 MHz, $CDCl_3$) δ 7.80 (d, *J* = 7.5 Hz, 1H), 7.60 (d, *J* = 7.5 Hz, 1H), 7.43 (p, *J* = 7.5 Hz, 2H), 2.05 (qd, *J* = 14.9, 13.4, 6.2 Hz, 2H), 1.91 (q, *J* = 6.2 Hz, 1H), 1.87 − 1.76 (m, 2H), 1.76 − 1.63 (m, 2H), 1.62 − 1.46 (m, 3H).

^{13}C NMR (100 MHz, $CDCl_3$) δ 156.3, 151.0, 140.6, 126.6, 125.5, 121.0, 114.8, 111.3, 73.7, 53.5, 32.9, 29.1, 25.0, 24.9, 24.9.

HRMS (ESI) *m/z* calcd. for $C_{15}H_{14}N_2NaO_2$ $[M+Na]^+$: 277.0947; found: 277.0945.

$[\alpha]_D^{20}$ = −56.9 (*c* 1.0, $CHCl_3$)

The enantiomeric excess was determined by chiral HPLC analysis using a Daicel Chiralpak IF column, e.e. = 70% (*n*-hexane/ethanol = 90/10, flow rate = 1.0 mL/min, λ = 254 nm, T = 20 °C, t_r (major) = 6.554 min, t_r (minor) = 5.957 min).

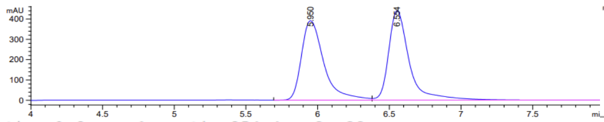

Signal 2: DAD1 B, Sig=254,4 Ref=off

Peak #	RetTime [min]	Type	Width [min]	Area [mAU*s]	Height [mAU]	Area %
1	5.950	BV	0.1647	4242.58887	390.71799	49.1056
2	6.554	VB	0.1506	4397.13232	439.81308	50.8944

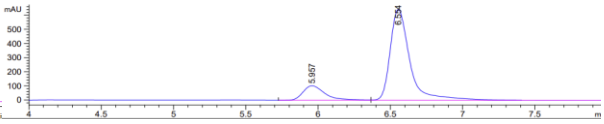

Signal 2: DAD1 B, Sig=254,4 Ref=off

Peak #	RetTime [min]	Type	Width [min]	Area [mAU*s]	Height [mAU]	Area %
1	5.957	BV	0.1626	1088.08496	101.86012	15.0933
2	6.554	VB	0.1450	6120.96973	643.26208	84.9067

Supplementary Figure 49. HPLC spectra of compound **3dc**

(*R*)-2-(benzo[*d*]thiazol-2-yl)-1,6-dioxaspiro[2.5]octane-2-carbonitrile (3dd)

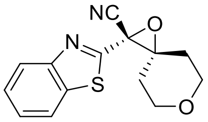

Compound **3dd** was synthesized following the General Procedure and subsequently purified using flash column chromatography (petroleum ether/EtOAc = 10/1), resulting in a white solid (26 mg) at 97% yield on a 0.1 mmol scale.

^{1}H NMR (400 MHz, $CDCl_3$) δ 8.12 (d, *J* = 8.3 Hz, 1H), 7.92 (d, *J* = 8.3 Hz, 1H), 7.57 (t, *J* = 7.8 Hz, 1H), 7.49 (t, *J* = 7.8 Hz, 1H), 4.06 − 3.89 (m, 2H), 3.78 (t, *J* = 5.6 Hz, 2H), 2.33 (dt, *J* = 12.8, 5.6 Hz, 1H), 2.20 − 2.02 (m, 1H), 1.81 (tq, *J* = 14.8, 9.3, 7.6 Hz, 2H).

^{13}C NMR (100 MHz, $CDCl_3$) δ 160.8, 153.3, 134.9, 127.2, 126.6, 124.1, 121.9, 115.2, 71.2, 66.3, 66.1, 57.3, 33.4, 29.7.

HRMS (ESI) *m/z* calcd. for $C_{14}H_{12}N_2NaO_2S$ $[M+Na]^+$: 295.0512; found: 295.0513.

$[\alpha]_D^{20}$ = −36.1 (*c* 1.0, $CHCl_3$)

The enantiomeric excess was determined by chiral HPLC analysis using a Daicel Chiralpak IJ column, e.e. = 80% (*n*-hexane/ethanol = 70/30, flow rate = 1.0 mL/min, λ = 254 nm, T = 20 °C, t_r (major) = 13.557 min, t_r (minor) = 9.540 min).

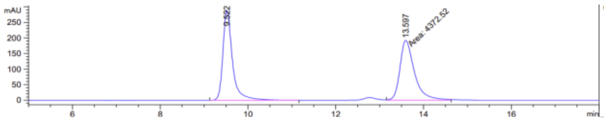

Signal 2: DAD1 B, Sig=254,4 Ref=off

Peak #	RetTime [min]	Type	Width [min]	Area [mAU*s]	Height [mAU]	Area %
1	9.522	BB	0.2258	4361.53418	289.36581	49.9371
2	13.597	MM	0.3798	4372.51807	191.89691	50.0629

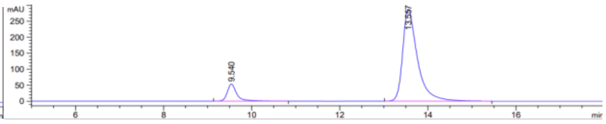

Signal 2: DAD1 B, Sig=254,4 Ref=off

Peak #	RetTime [min]	Type	Width [min]	Area [mAU*s]	Height [mAU]	Area %
1	9.540	BB	0.2225	796.27228	53.22982	10.6406
2	13.557	BB	0.3458	6687.05469	286.98978	89.3594

Supplementary Figure 50. HPLC spectra of compound **3dd**

(*R*)-2-(5-(4-Chlorophenyl)-1,3,4-oxadiazol-2-yl)-3,3-dimethyloxirane-2-carbonitrile (3de)

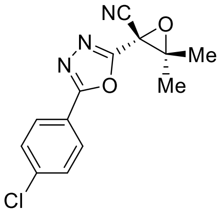

Compound **3de** was synthesized following the General Procedure and subsequently purified using flash column chromatography (petroleum ether/EtOAc = 10/1), resulting in a white solid (17 mg) at 72% yield on a 0.1 mmol scale.

^{1}H NMR (400 MHz, $CDCl_3$) δ 8.03 (d, *J* = 8.2 Hz, 2H), 7.53 (d, *J* = 8.2 Hz, 2H), 1.83 (s, 3H), 1.58 (s, 3H).

^{13}C NMR (100 MHz, $CDCl_3$) δ 165.6, 158.7, 139.2, 129.9, 128.7, 121.3, 114.1, 69.4, 51.3, 22.4, 19.5.

HRMS (ESI) *m/z* calcd. for $C_{13}H_{11}ClN_3O_2$ $[M+H]^+$: 276.0534; found: 276.0536.

$[\alpha]_D^{20}$ = −92.0 (*c* 1.0, $CHCl_3$)

The enantiomeric excess was determined by chiral HPLC analysis using a Daicel Chiralpak IA column, e.e. = 93% (*n*-hexane/ethanol = 80/20, flow rate = 1.0 mL/min, λ = 230 nm, T = 20 °C, t_r (major) = 9.873 min, t_r (minor) = 11.177 min).

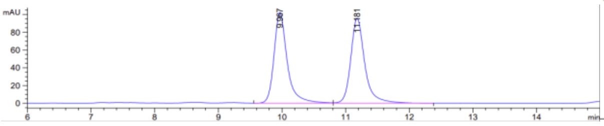

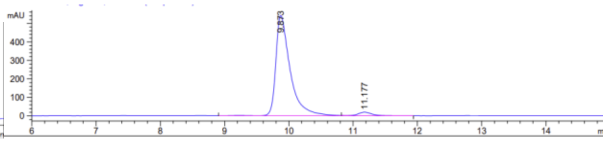

Signal 4: DAD1 D, Sig=230,4 Ref=off

Peak #	RetTime [min]	Type	Width [min]	Area [mAU*s]	Height [mAU]	Area %
1	9.967	BB	0.2163	232.31841	16.10348	50.6353
2	11.181	BB	0.2267	226.48923	14.95489	49.3647

Signal 4: DAD1 D, Sig=230,4 Ref=off

Peak #	RetTime [min]	Type	Width [min]	Area [mAU*s]	Height [mAU]	Area %
1	9.873	VV R	0.2267	8416.87500	541.58087	96.5322
2	11.177	VB E	0.2306	302.36630	19.75337	3.4678

Supplementary Figure 51. HPLC spectra of compound **3de**

(*R*)-2-(Benzo[*d*]thiazol-2-yl)-3,3-dimethyloxirane-2-carbonitrile (3df)

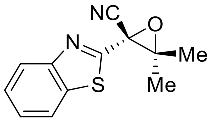

Compound **3df** was synthesized following the General Procedure and subsequently purified using flash column chromatography (petroleum ether/EtOAc = 10/1), resulting in a white solid (17 mg) at 74% yield on a 0.1 mmol scale.

^{1}H NMR (400 MHz, $CDCl_3$) δ 8.11 (d, *J* = 8.2 Hz, 1H), 7.92 (dd, *J* = 8.1, 1.2 Hz, 1H), 7.56 (t, *J* = 7.7 Hz, 1H), 7.48 (t, *J* = 7.6 Hz, 1H), 1.82 (s, 3H), 1.41 (s, 3H).

^{13}C NMR (100 MHz, $CDCl_3$) δ 162.1, 153.4, 134.9, 127.0, 126.4, 124.1, 121.9, 115.8, 69.5, 57.4, 22.9, 19.0.

HRMS (ESI) *m/z* calcd. for $C_{12}H_{11}N_2OS$ $[M+H]^+$: 231.0587; found: 231.0595.

$[\alpha]_D^{20}$ = −134.0 (*c* 1.0, $CHCl_3$)

The enantiomeric excess was determined by chiral HPLC analysis using a Daicel Chiralpak IF column, e.e. = 88% (*n*-hexane/ethanol = 90/10, flow rate = 1.0 mL/min, λ = 254 nm, T = 20 ºC, t_r (major) = 6.716 min, t_r (minor) = 5.981 min).

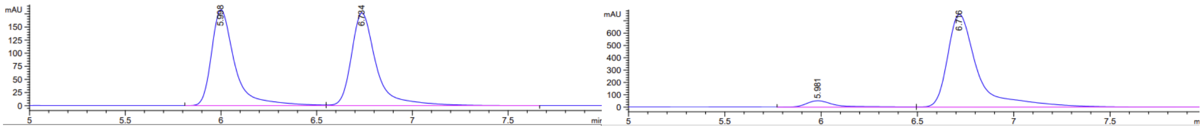

Signal 2: DAD1 B, Sig=254,4 Ref=off

Peak #	RetTime [min]	Type	Width [min]	Area [mAU*s]	Height [mAU]	Area %
1	5.998	BV	0.1267	1550.05164	183.19934	49.2003
2	6.734	VB	0.1355	1600.44153	177.00778	50.7997

Signal 2: DAD1 B, Sig=254,4 Ref=off

Peak #	RetTime [min]	Type	Width [min]	Area [mAU*s]	Height [mAU]	Area %
1	5.981	BV	0.1513	515.95209	51.33764	5.9142
2	6.716	VB	0.1633	8208.01172	752.32043	94.0858

Supplementary Figure 52. HPLC spectra of compound **3df**

((2*S*,3*R*)-3-(Benzo[*d*]thiazol-2-yl)-3-cyano-2-methyloxiran-2-yl)methyl benzoate (*trans*-3dg)

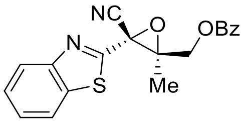

Compound *trans*-**3dg** was synthesized following the General Procedure and subsequently purified using flash column chromatography (petroleum ether/EtOAc = 10/1), resulting in a colorless oil (20 mg) at 58% yield on a 0.1 mmol scale.

^{1}H NMR (400 MHz, $CDCl_3$) δ 8.11 (d, *J* = 8.2 Hz, 1H), 7.94 (dd, *J* = 16.4, 7.9 Hz, 3H), 7.57 (q, *J* = 7.0 Hz, 2H), 7.46 (dt, *J* = 25.3, 7.9 Hz, 3H), 4.42 (d, *J* = 5.9 Hz, 2H), 1.96 (s, 3H).

^{13}C NMR (100 MHz, $CDCl_3$) δ 165.7, 160.0, 153.3, 135.0, 133.7, 129.9, 129.0, 128.7, 127.2, 126.8, 124.3, 122.0, 115.0, 69.1, 64.1, 56.9, 18.5.

HRMS (ESI) *m/z* calcd. for $C_{19}H_{14}N_2NaO_3S$ $[M+Na]^+$: 373.0617; found: 373.0628.

$[\alpha]_D^{20}$ = −66.9 (*c* 1.0, $CHCl_3$)

The enantiomeric excess was determined by chiral HPLC analysis using a Daicel Chiralpak IJ column, e.e. = 98% (*n*-hexane/ethanol = 70/30, flow rate = 1.0 mL/min, λ = 254 nm, T = 20 °C, t_r (major) = 15.820 min, t_r (minor) = 11.328 min).

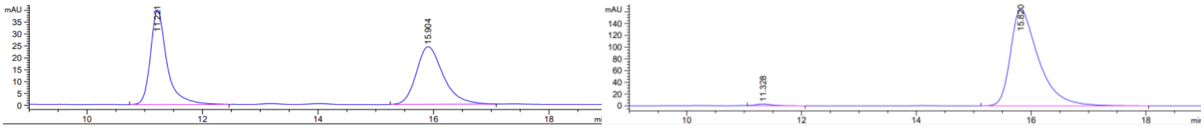

Signal 2: DAD1 B, Sig=254,4 Ref=off

Peak #	RetTime [min]	Type	Width [min]	Area [mAU*s]	Height [mAU]	Area %
1	11.221	BB	0.2925	786.15894	39.78254	50.0019
2	15.904	BB	0.4820	786.10022	24.26012	49.9981

Signal 2: DAD1 B, Sig=254,4 Ref=off

Peak #	RetTime [min]	Type	Width [min]	Area [mAU*s]	Height [mAU]	Area %
1	11.328	BB	0.2574	48.02189	2.78249	0.8549
2	15.820	BB	0.5042	5569.27344	164.00410	99.1451

Supplementary Figure 53. HPLC spectra of compound ***trans*-3dg**

((2*R*,3*R*)-3-(benzo[*d*]thiazol-2-yl)-3-cyano-2-methyloxiran-2-yl)methyl benzoate (*cis*-3dg)

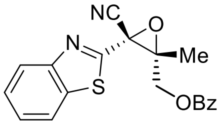

Compound *cis*-**3dg** was synthesized following the General Procedure and subsequently purified using flash column chromatography (petroleum ether/EtOAc = 10/1), resulting in a colorless oil (8 mg) at 25% yield on a 0.1 mmol scale.

^{1}H NMR (400 MHz, $CDCl_3$) δ 8.23 – 8.06 (m, 3H), 7.97 – 7.92 (m, 1H), 7.65 – 7.55 (m, 2H), 7.54 – 7.44 (m, 3H), 4.78 (q, *J* = 21.4 Hz, 2H), 1.53 (s, 3H).

^{13}C NMR (100 MHz, $CDCl_3$) δ 165.9, 160.8, 153.4, 135.0, 133.8, 130.1, 129.1, 128.7, 127.2, 126.7, 124.3, 121.9, 114.7, 69.1, 66.0, 56.0, 14.8.

HRMS (ESI) *m/z* calcd. for $C_{19}H_{15}N_2O_3S$ $[M+H]^+$: 351.0798; found: 351.0797.

$[\alpha]_D^{20}$ = −32.2 (*c* 1.0, $CHCl_3$)

The enantiomeric excess was determined by chiral HPLC analysis using a Daicel Chiralpak IJ column, e.e. = 25% (*n*-hexane/ethanol = 70/30, flow rate = 1.0 mL/min, λ = 254 nm, T = 20 ºC, t_r (major) = 15.764 min, t_r (minor) = 14.785 min).

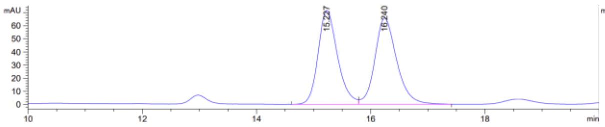

Signal 2: DAD1 B, Sig=254,4 Ref=off

Peak #	RetTime [min]	Type	Width [min]	Area [mAU*s]	Height [mAU]	Area %
1	15.227	BV	0.3594	1691.60901	71.65303	49.2674
2	16.240	VB	0.4010	1741.91919	65.77355	50.7326

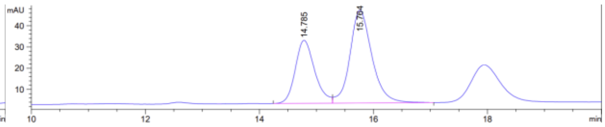

Signal 2: DAD1 B, Sig=254,4 Ref=off

Peak #	RetTime [min]	Type	Width [min]	Area [mAU*s]	Height [mAU]	Area %
1	14.785	BV	0.3581	703.44836	29.72114	37.5171
2	15.764	VB	0.4067	1171.55884	43.71496	62.4829

Supplementary Figure 54. HPLC spectra of compound ***cis*-3dg**

The absolute stereochemistry of stereoisomers was assigned by analogy and the relative stereochemistry was confirmed by ^{13}C NMR experiments.

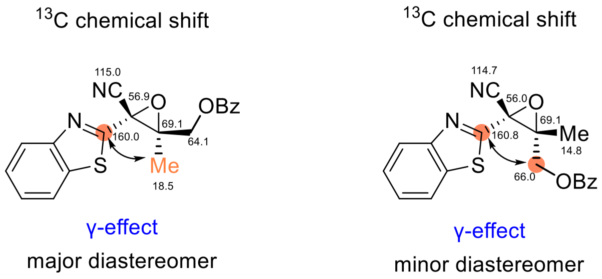

Supplementary Figure 55. Gammar effect of compound ***trans*-3dg** and ***cis*-3dg**

(*R*)-2-(3,5-Dichloropyridin-2-yl)oxirane-2-carbonitrile (3ea)

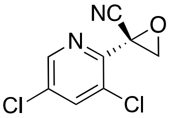

Compound **3ea** was synthesized following the General Procedure and subsequently purified using flash column chromatography (petroleum ether/EtOAc = 10/1), resulting in a colorless oil (19 mg) at 91% yield on a 0.1 mmol scale.

^{1}H NMR (400 MHz, $CDCl_3$) δ 8.48 (d, *J* = 2.1 Hz, 1H), 7.85 (d, *J* = 2.1 Hz, 1H), 3.64 (d, *J* = 5.7 Hz, 1H), 3.50 (d, *J* = 5.7 Hz, 1H).

^{13}C NMR (100 MHz, $CDCl_3$) δ 146.8, 145.7, 137.7, 134.1, 132.9, 115.9, 53.3, 48.2.

HRMS (ESI) *m/z* calcd. for $C_8H_4Cl_2N_2NaO$ $[M+Na]^+$: 236.9593 ; found: 236.9595.

$[\alpha]_D^{20}$ = −24.6 (*c* 1.0, $CHCl_3$)

The enantiomeric excess was determined by chiral HPLC analysis using a Daicel Chiralpak IF column, e.e. = 85% (n-hexane/ethanol = 90/10, flow rate = 1.0 mL/min, λ = 254 nm, T = 20 °C, t_r (major) = 15.762 min, t_r (minor) = 11.666 min).

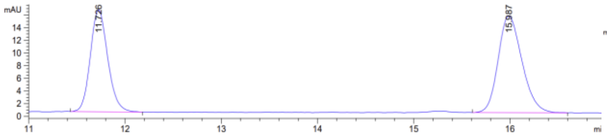

Signal 2: DAD1 B, Sig=254,4 Ref=off

Peak #	RetTime [min]	Type	Width [min]	Area [mAU*s]	Height [mAU]	Area %
1	11.726	MM	0.2157	209.81154	16.21113	50.3902
2	15.987	MM	0.2504	206.56226	13.74737	49.6098

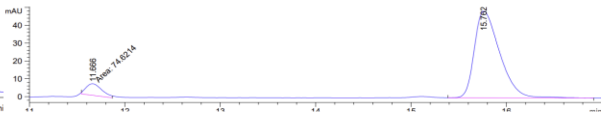

Signal 2: DAD1 B, Sig=254,4 Ref=off

Peak #	RetTime [min]	Type	Width [min]	Area [mAU*s]	Height [mAU]	Area %
1	11.666	MM	0.1917	74.62136	6.48887	7.6793
2	15.762	BB	0.2831	897.10504	48.64659	92.3207

Supplementary Figure 56. HPLC spectra of compound **3ea**

Ethyl (*S*)-2-(1-tosyl-1*H*-benzo[*d*]imidazol-2-yl)oxirane-2-carboxylate (3eb)

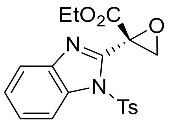

Compound **3eb** was synthesized following the General Procedure and subsequently purified using flash column chromatography (petroleum ether/EtOAc = 10/1), resulting in a colorless oil (25 mg) at 65% yield on a 0.1 mmol scale.

^{1}H NMR (600 MHz, $CDCl_3$) δ 8.10 (d, J = 8.3 Hz, 2H), 7.90 (d, J = 8.2 Hz, 1H), 7.72 (d, J = 7.9 Hz, 1H), 7.45 – 7.39 (m, 1H), 7.38 – 7.34 (m, 1H), 7.28 (d, J = 8.2 Hz, 2H), 4.42 (dq, J = 10.8, 7.1 Hz, 1H), 4.28 (dq, J = 10.8, 7.1 Hz, 1H), 3.68 (d, J = 6.6 Hz, 1H), 3.58 (d, J = 6.6 Hz, 1H), 2.37 (s, 3H), 1.30 (t, J = 7.1 Hz, 3H).

^{13}C NMR (150 MHz, $CDCl_3$) δ 167.5, 147.4, 146.4, 141.5, 134.5, 132.6, 130.1, 128.3, 126.3, 125.1, 121.2, 113.4, 62.9, 54.3, 53.6, 21.8, 14.2.

HRMS (ESI) m/z calcd. for $C_{19}H_{19}N_2O_5S$ $[M+H]^+$: 387.1009; found: 387.1009.

$[\alpha]_D^{20}$ = –31.2 (c 1.0, $CHCl_3$)

The enantiomeric excess was determined by chiral HPLC analysis using a Daicel Chiralpak IC column, e.e. = 85% (n-hexane/ethanol = 70/30, flow rate = 1.0 mL/min, λ = 254 nm, T = 20 °C, t_r (major) = 8.198 min, t_r (minor) = 9.175 min).

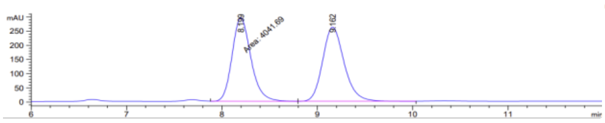

Signal 2: DAD1 B, Sig=254,4 Ref=off

Peak #	RetTime [min]	Type	Width [min]	Area [mAU*s]	Height [mAU]	Area %
1	8.199	MM	0.2289	4041.69214	294.25610	49.7285
2	9.162	BB	0.2405	4085.82324	261.22455	50.2715

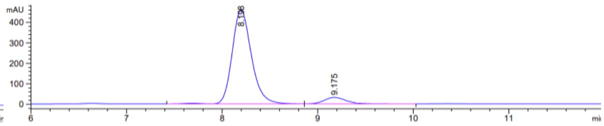

Signal 2: DAD1 B, Sig=254,4 Ref=off

Peak #	RetTime [min]	Type	Width [min]	Area [mAU*s]	Height [mAU]	Area %
1	8.198	VV R	0.2144	6421.62695	458.60797	92.6226
2	9.175	VB	0.2447	511.48669	31.96131	7.3774

Supplementary Figure 57. HPLC spectra of compound **3eb**

(*R*)-2-(2-(Phenylsulfonyl)oxiran-2-yl)quinoline (3ec)

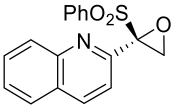

Compound **3ec** was synthesized following the General Procedure and subsequently purified using flash column chromatography (petroleum ether/EtOAc = 10/1), resulting in a yellow oil (22 mg) at 72% yield on a 0.1 mmol scale.

^{1}H NMR (600 MHz, $CDCl_3$) δ 8.16 (d, *J* = 8.4 Hz, 1H), 7.84 − 7.79 (m, 2H), 7.70 − 7.63 (m, 3H), 7.59 − 7.51 (m, 3H), 7.40 (t, *J* = 7.8 Hz, 2H), 4.74 (s, 2H).

^{13}C NMR (150 MHz, $CDCl_3$) δ 191.4, 150.9, 147.2, 137.6, 136.5, 134.1, 130.6, 130.6, 130.2, 129.4, 128.3, 128.0, 118.0, 71.0, 29.9.

HRMS (ESI) *m/z* calcd. for $C_{17}H_{14}NO_3S$ $[M+Na]^+$: 334.0514; found: 334.0522.

$[\alpha]_D^{20}$ = −89.3 (*c* 1.0, $CHCl_3$)

The enantiomeric excess was determined by chiral HPLC analysis using a Daicel Chiralpak IC column, e.e. = 75% (*n*-hexane/ethanol = 90/10, flow rate = 1.0 mL/min, λ = 254 nm, T = 20 °C, t_r (major) = 7.989 min, t_r (minor) = 9.235 min).

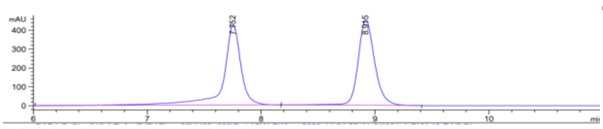

Signal 2: DAD1 B, Sig=254,4 Ref=off

Peak #	RetTime [min]	Type	Width [min]	Area [mAU*s]	Height [mAU]	Area %
1	7.752	BB	0.1550	4565.73389	426.03741	49.9926
2	8.915	BB	0.1535	4567.07813	453.40448	50.0074

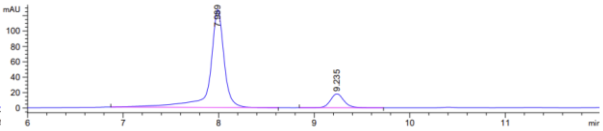

Signal 2: DAD1 B, Sig=254,4 Ref=off

Peak #	RetTime [min]	Type	Width [min]	Area [mAU*s]	Height [mAU]	Area %
1	7.989	BB	0.1500	1306.23340	127.03554	87.9578
2	9.235	BB	0.1511	178.83501	18.12877	12.0422

Supplementary Figure 58. HPLC spectra of compound **3ec**

Ethyl (*E*)-3-((2*S*,3*R*)-3-cyano-3-(4-(4-fluorophenyl)thiazol-2-yl)oxiran-2-yl)acrylate (3fa)

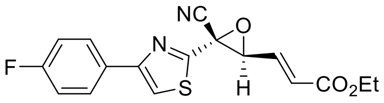

Compound **3fa** was synthesized following the General Procedure and subsequently purified using flash column chromatography (petroleum ether/EtOAc = 10/1), resulting in a white solid (28 mg) at 88% yield on a 0.1 mmol scale.

m.p.: 104.1 − 110.6 °C

^{1}H NMR (400 MHz, $CDCl_3$) δ 7.89 (dd, *J* = 8.9, 5.2 Hz, 2H), 7.53 (s, 1H), 7.13 (t, *J* = 8.7 Hz, 2H), 6.92 (dd, *J* = 15.7, 6.5 Hz, 1H), 6.42 (dd, *J* = 15.7, 0.8 Hz, 1H), 4.29 (dd, *J* = 7.2, 1.7 Hz, 1H), 4.27 – 4.24 (m, 2H), 1.34 (t, *J* = 7.2 Hz, 3H).

^{13}C NMR (100 MHz, $CDCl_3$) δ 164.5, 162.1, 160.4, 156.3, 136.0, 129.8, 129.6 (d, *J* = 3.3 Hz), 128.5 (d, *J* = 8.3 Hz), 116.1 (d, *J* = 21.8 Hz), 114.5, 113.8, 65.7, 61.5, 54.6, 14.3.

^{19}F NMR (376 MHz, $CDCl_3$) δ −115.5.

HRMS (ESI) *m/z* calcd. for $C_{17}H_{14}FN_2O_3S$ $[M+H]^+$: 345.0704; found: 345.0712.

$[\alpha]_D^{20}$ = −103.1 (*c* 1.0, $CHCl_3$)

The enantiomeric excess was determined by chiral HPLC analysis using a Daicel Chiralpak ID column, e.e. = 96% (*n*-hexane/ethanol = 70/30, flow rate = 1.0 mL/min, λ = 254 nm, T = 20 °C, t_r (major) = 8.258 min, t_r (minor) = 7.636 min).

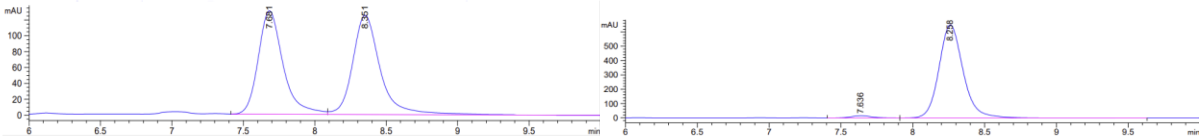

Signal 2: DAD1 B, Sig=254,4 Ref=off

Peak #	RetTime [min]	Type	Width [min]	Area [mAU*s]	Height [mAU]	Area %
1	7.681	BV	0.1830	1577.41248	130.52988	48.3373
2	8.351	VB	0.2010	1685.93311	125.25722	51.6627

Signal 2: DAD1 B, Sig=254,4 Ref=off

Peak #	RetTime [min]	Type	Width [min]	Area [mAU*s]	Height [mAU]	Area %
1	7.636	BV	0.1637	165.98767	15.65563	2.1320
2	8.258	VB	0.1784	7619.52490	651.82196	97.8680

Supplementary Figure 59. HPLC spectra of compound **3fa**

(2*R*,3*S*)-2-(4-(4-Methoxyphenyl)thiazol-2-yl)-3-((*E*)-3-oxo-3-phenylprop-1-en-1-yl)oxirane-2-carbonitrile (3fb)

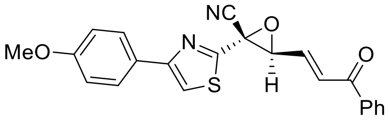

Compound **3fb** was synthesized following the General Procedure and subsequently purified using flash column chromatography (petroleum ether/EtOAc = 10/1), resulting in a yellow solid (20 mg) at 77% yield on a 0.1 mmol scale.

m.p.: 126.3 – 129.9 °C

^{1}H NMR (400 MHz, $CDCl_3$) δ 8.03 – 7.98 (m, 2H), 7.86 (d, *J* = 8.8 Hz, 2H), 7.62 (t, *J* = 7.4 Hz, 1H), 7.52 (t, *J* = 7.6 Hz, 2H), 7.48 – 7.41 (m, 2H), 7.03 (dd, *J* = 15.5, 5.8 Hz, 1H), 6.97 (d, *J* = 8.8 Hz, 2H), 4.41 (dd, *J* = 5.8, 0.9 Hz, 1H), 3.86 (s, 3H).

^{13}C NMR (100 MHz, $CDCl_3$) δ 188.8, 160.3, 160.0, 157.2, 136.9, 135.4, 133.8, 132.1, 129.0, 129.0, 128.0, 126.2, 114.4, 114.1, 113.2, 66.0, 55.5, 55.2.

HRMS (ESI) *m/z* calcd. for $C_{22}H_{16}N_2O_3S$ $[M+Na]^+$: 345.0704; found: 345.0712.

$[\alpha]_D^{20}$ = −30.8 (*c* 1.0, $CHCl_3$)

The enantiomeric excess was determined by chiral HPLC analysis using a Daicel Chiralpak IB column, e.e. = 81% (*n*-hexane/ethanol = 70/30, flow rate = 1.0 mL/min, λ = 254 nm, T = 20 °C, t_r (major) = 20.621 min, t_r (minor) = 14.718 min).

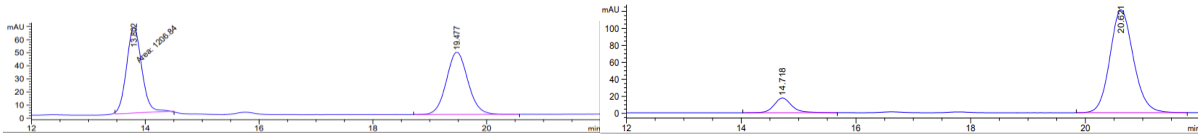

Signal 2: DAD1 B, Sig=254,4 Ref=off

Peak #	RetTime [min]	Type	Width [min]	Area [mAU*s]	Height [mAU]	Area %
1	13.802	MM	0.3073	1206.84412	65.45954	49.0753
2	19.477	BB	0.4071	1252.32507	47.58313	50.9247

Signal 2: DAD1 B, Sig=254,4 Ref=off

Peak #	RetTime [min]	Type	Width [min]	Area [mAU*s]	Height [mAU]	Area %
1	14.718	BB	0.3228	372.45596	17.32755	9.7679
2	20.621	BB	0.4357	3440.59351	121.01130	90.2321

Supplementary Figure 60. HPLC spectra of compound **3fb**

Methyl (*E*)-3-(4-((2*S*,3*R*)-3-cyano-3-(5-phenyloxazol-2-yl)oxiran-2-yl)phenyl)acrylate (3fc)

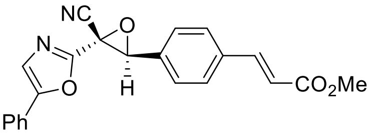

Compound **3fc** was synthesized following the General Procedure and subsequently purified using flash column chromatography (petroleum ether/EtOAc = 1/1), resulting in a yellow solid (33 mg) at 90% yield on a 0.1 mmol scale.

m.p.: 156.2 – 158.7 °C

^{1}H NMR (400 MHz, $CDCl_3$) δ 7.75 – 7.66 (m, 3H), 7.63 (d, *J* = 8.0 Hz, 2H), 7.55 (d, *J* = 8.1 Hz, 2H), 7.44 (ddt, *J* = 14.8, 8.6, 4.6 Hz, 4H), 6.50 (d, *J* = 16.1 Hz, 1H), 4.97 (s, 1H), 3.83 (s, 3H).

^{13}C NMR (100 MHz, $CDCl_3$) δ 167.2, 154.2, 153.6, 143.7, 136.5, 132.2, 129.8, 129.3, 128.6, 127.4, 126.7, 124.8, 123.3, 119.5, 113.0, 65.4, 52.0, 51.6.

HRMS (ESI) *m/z* calcd. for $C_{22}H_{17}N_2O_4Na$ $[M+H]^+$: 373.1183; found: 373.1175.

$[\alpha]_D^{20}$ = −61.0 (*c* 1.0, $CHCl_3$)

The enantiomeric excess was determined by chiral HPLC analysis using a Daicel Chiralpak IB column, e.e. = 98% (*n*-hexane/ethanol = 70/30, flow rate = 1.0 mL/min, λ = 280 nm, T = 20 °C, t_r (major) = 7.997 min, t_r (minor) = 12.511 min).

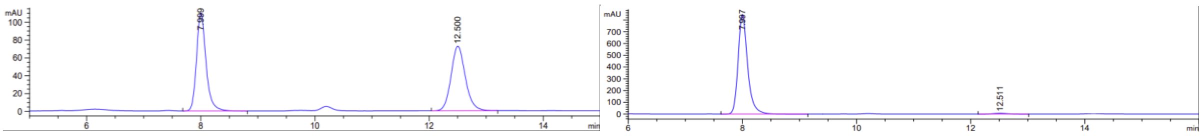

Signal 5: DAD1 E, Sig=280,4 Ref=off

Peak #	RetTime [min]	Type	Width [min]	Area [mAU*s]	Height [mAU]	Area %
1	7.999	BB	0.1771	1273.82239	110.02315	50.2375
2	12.500	BB	0.2654	1261.77649	72.37541	49.7625

Signal 5: DAD1 E, Sig=280,4 Ref=off

Peak #	RetTime [min]	Type	Width [min]	Area [mAU*s]	Height [mAU]	Area %
1	7.997	BB	0.1757	9844.37305	846.47131	98.7719
2	12.511	BB	0.2705	122.40146	6.98245	1.2281

Supplementary Figure 61. HPLC spectra of compound **3fc**

(2*R*,3*S*)-2-(Pyridin-2-yl)-3-((*E*)-styryl)oxirane-2-carbonitrile (3fd)

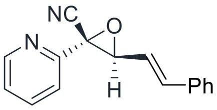

Compound **3fd** was synthesized following the General Procedure and subsequently purified using flash column chromatography (petroleum ether/EtOAc = 10/1), resulting in a colorless oil (20 mg) at 56% yield on a 0.1 mmol scale.

^{1}H NMR (400 MHz, $CDCl_3$) δ 8.68 (ddd, *J* = 4.8, 1.8, 1.0 Hz, 1H), 7.81 (td, *J* = 7.7, 1.8 Hz, 1H), 7.55 (d, *J* = 7.9 Hz, 1H), 7.51 – 7.45 (m, 2H), 7.40 – 7.32 (m, 4H), 7.03 (d, *J* = 15.9 Hz, 1H), 6.27 (dd, *J* = 15.9, 8.0 Hz, 1H), 4.13 (d, *J* = 7.9 Hz, 1H).

^{13}C NMR (100 MHz, $CDCl_3$) δ 151.3, 150.2, 139.8, 137.6, 135.3, 129.3, 129.0, 127.2, 124.7, 120.8, 120.4, 116.0, 67.2, 57.0.

HRMS (ESI) *m/z* calcd. for $C_{16}H_{13}N_2O$ $[M+H]^+$: 249.1022; found: 249.1030.

$[\alpha]_D^{20}$ = −48.4 (*c* 1.0, $CHCl_3$)

The enantiomeric excess was determined by chiral HPLC analysis using a Daicel Chiralpak IC column, e.e. = 98% (*n*-hexane/ethanol = 90/10, flow rate = 1.0 mL/min, λ = 254 nm, T = 20 °C, t_r (major) = 9.028 min, t_r (minor) = 10.365 min).

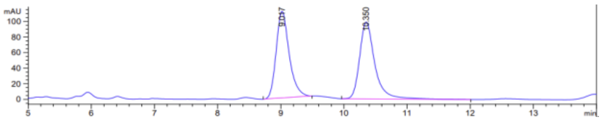

Signal 2: DAD1 B, Sig=254,4 Ref=off

Peak #	RetTime [min]	Type	Width [min]	Area [mAU*s]	Height [mAU]	Area %
1	9.017	BB	0.2145	1584.43152	111.02820	49.0176
2	10.350	BB	0.2510	1647.94287	98.58403	50.9824

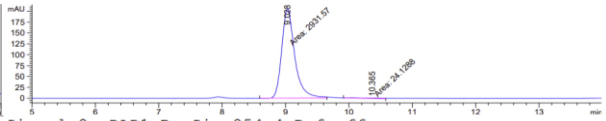

Signal 2: DAD1 B, Sig=254,4 Ref=off

Peak #	RetTime [min]	Type	Width [min]	Area [mAU*s]	Height [mAU]	Area %
1	9.028	MM	0.2387	2931.56934	204.64844	99.1837
2	10.365	MM	0.4457	24.12883	9.02240e-1	0.8163

Supplementary Figure 62. HPLC spectra of compound **3fd**

(2*R*,3*S*)-3-(2-Methylprop-1-en-1-yl)-2-(4-(trifluoromethyl)pyridin-2-yl)oxirane-2-carbonitrile (3fe)

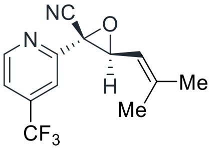

Compound **3fe** was synthesized following the General Procedure and subsequently purified using flash column chromatography (petroleum ether/EtOAc = 10/1), resulting in a yellow oil (20 mg) at 76% yield on a 0.1 mmol scale.

^{1}H NMR (400 MHz, DMSO-d_6) δ 8.10 (d, J = 8.3 Hz, 2H), 7.90 (d, J = 8.2 Hz, 1H), 7.72 (d, J = 7.9 Hz, 1H), 7.45 − 7.39 (m, 1H), 7.38 − 7.34 (m, 1H), 7.28 (d, J = 8.2 Hz, 2H), 4.42 (dq, J = 10.8, 7.1 Hz, 1H), 4.28 (dq, J = 10.8, 7.1 Hz, 1H), 3.68 (d, J = 6.6 Hz, 1H), 3.58 (d, J = 6.6 Hz, 1H), 2.37 (s, 3H), 1.30 (t, J = 7.1 Hz, 3H).

^{13}C NMR (100 MHz, DMSO-d_6) δ 151.7, 151.1, 147.8, 138.0 (q, J = 33.7 Hz), 122.7 (q, J = 273.4 Hz), 119.7 (d, J = 3.6 Hz), 118.3, 116.1 (d, J = 3.7 Hz), 115.0, 62.8, 70.0, 24.4, 19.3.

^{19}F NMR (376 MHz, DMSO-d_6) δ −65.6.

HRMS (ESI) *m/z* calcd. for $C_{13}H_{11}F_3N_2NaO$ $[M+Na]^+$: 291.0716; found: 291.0713.

$[\alpha]_D^{20}$ = −125.7 (*c* 1.0, $CHCl_3$)

The enantiomeric excess was determined by chiral HPLC analysis using a Daicel Chiralpak IA column, e.e. = 92% (*n*-hexane/ethanol = 80/20, flow rate = 1.0 mL/min, λ = 254 nm, T = 20 °C, t_r (major) = 4.473 min, t_r (minor) = 7.674 min).

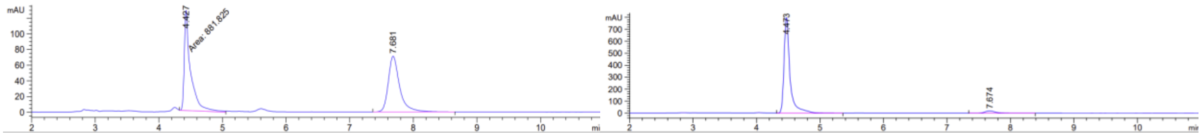

Signal 2: DAD1 B, Sig=254,4 Ref=off

Peak #	RetTime [min]	Type	Width [min]	Area [mAU*s]	Height [mAU]	Area %
1	4.427	MM	0.1148	881.82465	128.05328	50.0259
2	7.681	BB	0.1867	880.91010	71.01012	49.9741

Signal 2: DAD1 B, Sig=254,4 Ref=off

Peak #	RetTime [min]	Type	Width [min]	Area [mAU*s]	Height [mAU]	Area %
1	4.473	VB	0.0966	5239.70264	798.10016	95.7868
2	7.674	BB	0.1817	230.46933	18.97402	4.2132

Supplementary Figure 63. HPLC spectra of compound **3fe**

(2*R*,3*R*)-2-(benzo[*d*]thiazol-2-yl)-3-((*Z*)-1-bromo-2-phenylvinyl)oxirane-2-carbonitrile (3ff)

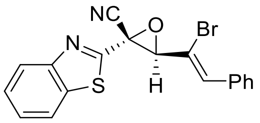

Compound **3ff** was synthesized following the General Procedure and subsequently purified using flash column chromatography (petroleum ether/EtOAc = 10/1), resulting in a white solid (35 mg) at 91% yield on a 0.1 mmol scale.

m.p.: 112.1 − 114.2 °C

^{1}H NMR (400 MHz, $CDCl_3$) δ 8.16 (d, J = 8.2 Hz, 1H), 7.94 (d, J = 8.2 Hz, 1H), 7.73 (d, J = 7.1 Hz, 2H), 7.59 (t, J = 7.8 Hz, 1H), 7.51 (t, J = 7.8 Hz, 1H), 7.47 − 7.37 (m, 4H), 4.49 (s, 1H).

^{13}C NMR (100 MHz, $CDCl_3$) δ 161.0, 153.2, 135.0, 133.5, 131.5, 129.5, 128.6, 127.3, 126.9, 124.3, 122.1, 113.2, 112.3, 69.3, 56.1.

HRMS (ESI) *m/z* calcd. for $C_{18}H_{12}BrN_2OS$ $[M+H]^+$: 382.9848; found: 382.9864.

$[\alpha]_D^{20}$ = −48.4 (*c* 1.0, $CHCl_3$)

The enantiomeric excess was determined by chiral HPLC analysis using a Daicel Chiralpak IJ column, e.e. = 99% (*n*-hexane/ethanol = 70/30, flow rate = 1.0 mL/min, λ = 250 nm, T = 20 ºC, t_r (major) = 14.117 min, t_r (minor) = 15.783 min).

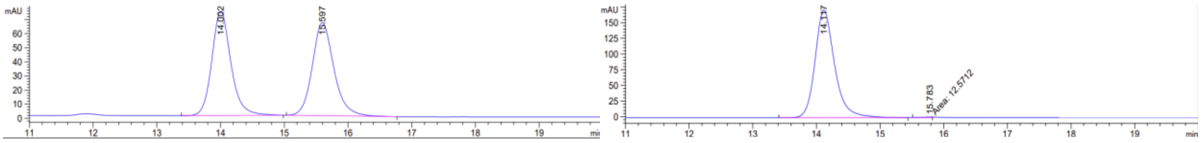

Signal 1: DAD1 A, Sig=250,4 Ref=off

Peak #	RetTime [min]	Type	Width [min]	Area [mAU*s]	Height [mAU]	Area %
1	14.002	BB	0.3153	1543.11963	73.98769	49.7501
2	15.597	BB	0.3576	1558.62012	65.96939	50.2499

Signal 1: DAD1 A, Sig=250,4 Ref=off

Peak #	RetTime [min]	Type	Width [min]	Area [mAU*s]	Height [mAU]	Area %
1	14.117	BB	0.3252	3672.88330	171.93700	99.6589
2	15.783	MM	0.2193	12.57119	9.55233e-1	0.3411

Supplementary Figure 64. HPLC spectra of compound **3ff**

(2*R*,3*S*)-3-(*dec*-9-En-1-yl)-2-(4-(4-fluorophenyl)thiazol-2-yl)oxirane-2-carbonitrile (3fg)

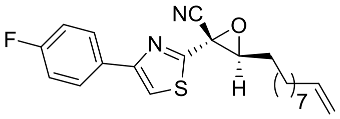

Compound **3fg** was synthesized following the General Procedure and subsequently purified using flash column chromatography (petroleum ether/EtOAc = 10/1), resulting in a colorless oil (37 mg) at 96% yield on a 0.1 mmol scale.

^{1}H NMR (400 MHz, $CDCl_3$) δ 7.94 – 7.86 (m, 2H), 7.47 (s, 1H), 7.12 (t, *J* = 8.1 Hz, 3H), 5.81 (d, *J* = 7.4 Hz, 1H), 5.06 – 4.89 (m, 2H), 3.58 (t, *J* = 5.6 Hz, 1H), 2.07 – 1.97 (m, 3H), 1.70 – 1.53 (m, 3H), 1.49 – 1.27 (m, 10H).

^{13}C NMR (100 MHz, $CDCl_3$) δ 164.4, 162.4, 155.9, 139.3, 129.8 (d, *J* = 2.8 Hz), 128.4 (d, *J* = 8.3 Hz), 116.0 (d, *J* = 21.8 Hz), 114.9, 114.3, 113.9, 69.2, 53.0, 34.0, 30.4, 29.5, 29.4, 29.3, 29.2, 29.0, 25.6.

^{19}F NMR (376 MHz, $CDCl_3$) δ −115.8.

HRMS (ESI) *m/z* calcd. for $C_{22}H_{26}FN_2OS$ $[M+H]^+$: 285.1744; found: 385.1754.

$[\alpha]_D^{20}$ = −48.4 (*c* 1.0, $CHCl_3$)

The enantiomeric excess was determined by chiral HPLC analysis using a Daicel Chiralpak IJ column, e.e. = 98% (*n*-hexane/ethanol = 90/10, flow rate = 1.0 mL/min, λ = 254 nm, T = 20 ºC, t_r (major) = 9.731 min, t_r (minor) = 8.289 min).

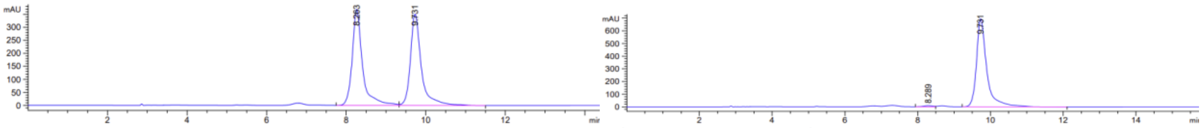

Signal 2: DAD1 B, Sig=254,4 Ref=off

Peak #	RetTime [min]	Type	Width [min]	Area [mAU*s]	Height [mAU]	Area %
1	8.263	BV	0.2645	6724.57422	369.14902	50.0442
2	9.731	VB	0.2813	6712.69922	350.67062	49.9558

Signal 2: DAD1 B, Sig=254,4 Ref=off

Peak #	RetTime [min]	Type	Width [min]	Area [mAU*s]	Height [mAU]	Area %
1	8.289	BV	0.2345	128.91434	8.15171	0.9358
2	9.731	BB	0.2872	1.36472e4	694.77747	99.0642

Supplementary Figure 65. HPLC spectra of compound **3fg**

(2*S*,3*S*)-2-(5-Bromopyrimidin-2-yl)-3-((1*E*,5*Z*)-octa-1,5-dien-1-yl)oxirane-2-carbonitrile (3fh)

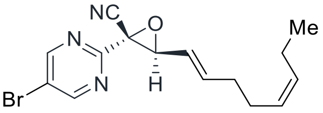

Compound **3fh** was synthesized following the General Procedure and subsequently purified using flash column chromatography (petroleum ether/EtOAc = 10/1), resulting in a colorless oil (26 mg) at 85% yield on a 0.1 mmol scale.

^{1}H NMR (400 MHz, $CDCl_3$) δ 8.84 (s, 2H), 6.26 (dt, *J* = 13.9, 6.5 Hz, 1H), 5.61 (dd, *J* = 15.6, 8.2 Hz, 1H), 5.43 (q, *J* = 8.7, 7.6 Hz, 1H), 5.32 (dt, *J* = 12.0, 7.2 Hz, 1H), 4.17 (d, *J* = 8.2 Hz, 1H), 2.23 (dt, *J* = 20.5, 7.2 Hz, 4H), 2.07 – 1.99 (m, 2H), 0.96 (t, *J* = 7.2 Hz, 3H).

^{13}C NMR (100 MHz, $CDCl_3$) δ 159.1, 158.8, 143.2, 133.0, 127.4, 122.0, 121.2, 115.1, 66.6, 56.4, 32.8, 26.3, 20.7, 14.4.

HRMS (ESI) *m/z* calcd. for $C_{15}H_{16}BrN_3ONa$ $[M+Na]^+$: 356.0369; found: 356.0379.

$[\alpha]_D^{20}$ = −36.3 (*c* 1.0, $CHCl_3$)

The enantiomeric excess was determined by chiral HPLC analysis using a Daicel Chiralpak IJ column, e.e. = 96% (*n*-hexane/ethanol = 70/30, flow rate = 1.0 mL/min, λ = 254 nm, T = 20 °C, t_r (major) = 13.799 min, t_r (minor) = 12.842 min).

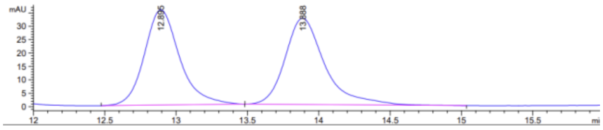

Signal 2: DAD1 B, Sig=254,4 Ref=off

Peak #	RetTime [min]	Type	Width [min]	Area [mAU*s]	Height [mAU]	Area %
1	12.895	BB	0.2603	610.28583	35.55317	49.4524
2	13.888	BB	0.2897	623.80048	32.23808	50.5476

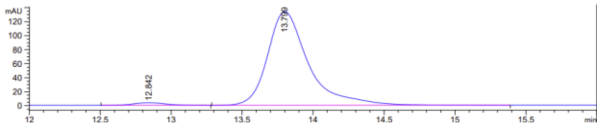

Signal 2: DAD1 B, Sig=254,4 Ref=off

Peak #	RetTime [min]	Type	Width [min]	Area [mAU*s]	Height [mAU]	Area %
1	12.842	BB	0.2474	60.60543	3.69422	2.2087
2	13.799	BB	0.2998	2683.38647	133.89679	97.7913

Supplementary Figure 66. HPLC spectra of compound **3fh**

(2*R*,3*S*)-2-(Benzo[*d*]oxazol-2-yl)-3-((*Z*)-oct-5-en-1-yl)oxirane-2-carbonitrile (3fi)

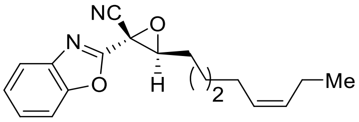

Compound **3fi** was synthesized following the General Procedure and subsequently purified using flash column chromatography (petroleum ether/EtOAc = 10/1), resulting in a colorless oil (25 mg) at 88% yield on a 0.1 mmol scale.

^{1}**H NMR** (400 MHz, $CDCl_3$) δ 7.79 (d, J = 7.0 Hz, 1H), 7.56 (d, J = 7.7 Hz, 1H), 7.42 (p, J = 7.7 Hz, 1H), 5.45 − 5.27 (m, 1H), 3.95 (t, J = 6.2 Hz, 1H), 2.05 (ddq, J = 31.7, 15.2, 7.3 Hz, 3H), 1.72 − 1.59 (m, 1H), 1.52 (p, J = 7.5 Hz, 1H), 0.96 (t, J = 7.6 Hz, 1H).

^{13}**C NMR** (100 MHz, $CDCl_3$) δ 156.9, 151.1, 140.7, 132.5, 128.3, 126.8, 125.6, 121.1, 113.7, 111.2, 66.1, 48.9, 30.0, 29.3, 26.9, 25.3, 20.7, 14.5.

HRMS (ESI) *m/z* calcd. for $C_{18}H_{21}N_2O_2$ $[M+H]^+$: 297.1598; found: 297.1594.

$[\alpha]_D^{20}$ = −39.3 (c 1.0, $CHCl_3$)

The enantiomeric excess was determined by chiral HPLC analysis using a Daicel Chiralpak IB column, e.e. = 96% (n-hexane/ethanol = 99/1, flow rate = 1.0 mL/min, λ = 254 nm, T = 20 °C, t_r (major) = 6.010 min, t_r (minor) = 6.716 min).

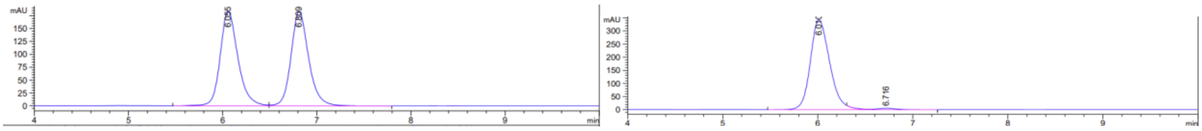

Signal 2: DAD1 B, Sig=254,4 Ref=off

Peak #	RetTime [min]	Type	Width [min]	Area [mAU*s]	Height [mAU]	Area %
1	6.055	BV	0.1991	2422.28589	184.64275	50.2168
2	6.809	VB	0.1984	2401.36621	183.82066	49.7832

Signal 2: DAD1 B, Sig=254,4 Ref=off

Peak #	RetTime [min]	Type	Width [min]	Area [mAU*s]	Height [mAU]	Area %
1	6.010	BV R	0.2190	4855.83398	343.39807	98.2509
2	6.716	VB E	0.2344	86.44305	5.35163	1.7491

Supplementary Figure 67. HPLC spectra of compound **3fi**

(2*R*,3*S*)-3-((*R*)-4-(Prop-1-en-2-yl)cyclohex-1-en-1-yl)-2-(pyridin-2-yl)oxirane-2-carbonitrile (3ga)

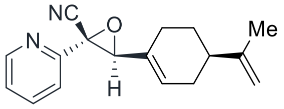

Compound **3ga** was synthesized following the General Procedure and subsequently purified using flash column chromatography (petroleum ether/EtOAc = 10/1), resulting in a yellow oil (16 mg) at 62% yield on a 0.1 mmol scale. The diastereomeric ratio of the resulting product was ascertained to exceed 20:1, as determined through crude ^{1}H NMR spectroscopic analysis.

^{1}**H NMR** (400 MHz, $CDCl_3$) δ 8.63 (d, J = 4.6 Hz, 1H), 7.76 (t, J = 7.9 Hz, 1H), 7.46 (d, J = 8.0 Hz, 1H), 7.32 (t, J = 6.2 Hz, 1H), 6.08 (s, 1H), 4.73 (d, J = 4.0 Hz, 2H), 3.81 (s, 1H), 2.23 (dt, J = 21.8, 9.8 Hz, 4H), 2.14 − 1.99 (m, 1H), 1.94 − 1.84 (m, 1H), 1.73 (s, 3H), 1.52 (qd, J = 11.2, 4.7 Hz, 1H).

^{13}**C NMR** (100 MHz, $CDCl_3$) δ 151.7, 150.0, 149.0, 137.5, 129.2, 128.7, 124.4, 119.9, 116.1, 109.3, 68.7, 56.3, 40.8, 30.4, 27.1, 25.4, 20.8.

HRMS (ESI) *m/z* calcd. for $C_{17}H_{19}N_2O$ $[M+Na]^+$: 267.1492; found: 267.1487.

$[\alpha]_D^{20}$ = −200.2 (c 1.0, $CHCl_3$)

(2*R*,3*S*)-3-((1*S*,5*R*)-6,6-Dimethylbicyclo[3.1.1]hept-2-en-3-yl)-2-(4-(4-methoxyphenyl) thiazol-2-yl)oxirane-2-carbonitrile (3gb)

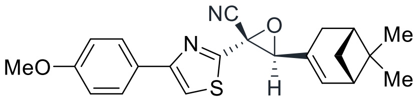

Compound **3gb** was synthesized following the General Procedure and subsequently purified using flash column chromatography (petroleum ether/EtOAc = 10/1), resulting in a colorless oil (29 mg) at 80% yield on a 0.1 mmol scale. The diastereomeric ratio of the resulting product was ascertained to exceed 20:1, as determined through crude ^{1}H NMR spectroscopic analysis.

^{1}H NMR (400 MHz, $CDCl_3$) δ 7.85 (d, *J* = 8.9 Hz, 2H), 7.39 (s, 1H), 6.95 (d, *J* = 8.8 Hz, 2H), 6.02 (s, 1H), 3.96 (s, 1H), 3.85 (s, 3H), 2.56 − 2.47 (m, 1H), 2.44 (td, *J* = 5.6, 2.1 Hz, 1H), 2.38 (d, *J* = 18.6 Hz, 1H), 2.17 (s, 1H), 1.35 (s, 2H), 1.31 (d, *J* = 8.8 Hz, 1H).

^{13}C NMR (100 MHz, $CDCl_3$) δ 162.1, 160.2, 156.8, 139.3, 128.0, 127.5, 126.5, 114.8, 114.3, 112.5, 69.5, 55.5, 53.8, 41.6, 40.9, 38.4, 31.8, 31.8, 25.8, 21.6.

HRMS (ESI) *m/z* calcd. for $C_{22}H_{22}N_2NaO_2S$ $[M+Na]^+$: 401.1294; found: 401.1307.

$[\alpha]_D^{20}$ = −50.4 (*c* 1.0, $CHCl_3$)

(2*R*,3*S*)-3-((*R*)-2,6-Dimethylhept-5-en-1-yl)-2-(pyridin-2-yl)oxirane-2-carbonitrile (3gc)

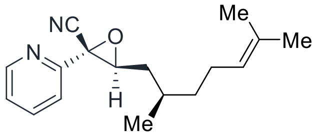

Compound **3gc** was synthesized following the General Procedure and subsequently purified using flash column chromatography (petroleum ether/EtOAc = 10/1), resulting in a yellow oil (26 mg) at 96% yield on a 0.1 mmol scale. The diastereomeric ratio of the resulting product was ascertained to exceed 20:1, as determined through crude ^{1}H NMR spectroscopic analysis.

^{1}H NMR (400 MHz, $CDCl_3$) δ 8.64 (ddd, *J* = 4.9, 1.8, 1.0 Hz, 1H), 7.76 (td, *J* = 7.7, 1.7 Hz, 1H), 7.46 (d, *J* = 7.8 Hz, 1H), 7.33 (ddd, *J* = 7.6, 4.8, 1.1 Hz, 1H), 5.10 − 5.03 (m, 1H), 3.48 (dd, *J* = 6.8, 4.7 Hz, 1H), 2.04 (ddd, *J* = 14.6, 9.1, 6.1 Hz, 3H), 1.92 − 1.71 (m, 2H), 1.67 (s, 3H), 1.59 (s, 3H), 1.05 (d, *J* = 6.5 Hz, 3H).

^{13}C NMR (100 MHz, $CDCl_3$) δ 151.9, 150.0, 137.5, 131.8, 124.5, 124.2, 120.1, 116.4, 66.5, 54.8, 37.3, 36.9, 31.0, 25.8, 25.5, 19.8, 17.8.

HRMS (ESI) *m/z* calcd. for $C_{17}H_{22}N_2NaO$ $[M+Na]^+$: 293.1624; found: 293.1614.

$[\alpha]_D^{20}$ = −76.8 (*c* 1.0, $CHCl_3$)

Ethyl 2-(4-((2*S*,3*R*)-3-(benzo[*d*]thiazol-2-yl)-3-cyanooxiran-2-yl)-3-isobutoxyphenyl)-4-methylthiazole-5-carboxylate (3gd)

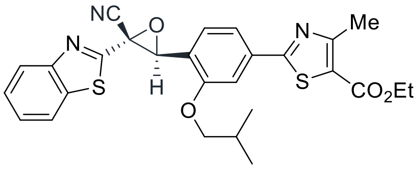

Compound **3gd** was synthesized following the General Procedure and subsequently purified using flash column chromatography (petroleum ether/EtOAc = 10/1), resulting in a yellow solid (34 mg) at 61% yield on a 0.1 mmol scale.

m.p.: 131.5 − 149.8 °C

^{1}H NMR (400 MHz, $CDCl_3$) δ 8.17 − 8.13 (m, 1H), 8.06 (dd, *J* = 8.6, 2.3 Hz, 1H), 8.00 − 7.93 (m, 2H), 7.58 (ddd, *J* = 8.3, 7.2, 1.3 Hz, 1H), 7.50 (ddd, *J* = 8.3, 7.2, 1.2 Hz, 1H), 7.00 (d, *J* = 8.7 Hz, 1H), 4.92 (s, 1H), 4.35 (q, *J* = 7.1 Hz, 2H), 3.92 − 3.81 (m, 2H), 2.78 (s, 3H), 2.05 (hept, *J* = 6.6 Hz, 1H), 1.39 (t, *J* = 7.1 Hz, 3H), 0.93 (d, *J* = 2.3 Hz, 3H), 0.92 (d, *J* = 2.2 Hz, 3H).

^{13}C NMR (100 MHz, $CDCl_3$) δ 169.0, 162.5, 162.1, 161.2, 160.0, 153.3, 135.0, 130.0, 127.1, 126.6, 126.1, 125.4, 124.3, 122.1, 121.4, 120.8, 114.0, 111.8, 75.1, 65.5, 61.3, 55.7, 28.2, 19.2, 19.2, 17.7, 14.5.

HRMS (ESI) *m/z* calcd. for $C_{22}H_{16}N_2O_3S$ $[M+Na]^+$: 345.0704; found: 345.0712.

$[\alpha]_D^{20}$ = −17.3 (*c* 1.0, $CHCl_3$)

The enantiomeric excess was determined by chiral HPLC analysis using a Daicel Chiralpak IJ column, e.e. = 86% (*n*-hexane/ethanol = 70/30, flow rate = 1.0 mL/min, λ = 254 nm, T = 20 °C, t_r (major) = 11.968 min, t_r (minor) = 20.895 min).

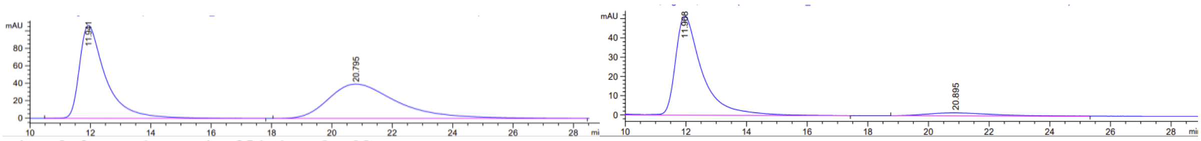

Signal 2: DAD1 B, Sig=254,4 Ref=off

Peak #	RetTime [min]	Type	Width [min]	Area [mAU*s]	Height [mAU]	Area %
1	11.941	BB	0.8949	6419.92920	106.55191	50.5592
2	20.795	BBA	2.4158	6277.91455	39.46772	49.4408

Signal 2: DAD1 B, Sig=254,4 Ref=off

Peak #	RetTime [min]	Type	Width [min]	Area [mAU*s]	Height [mAU]	Area %
1	11.968	BB	0.9000	3164.39795	51.55938	93.0292
2	20.895	BB	1.8275	237.11290	1.57581	6.9708

Supplementary Figure 68. HPLC spectra of compound **3gd**

(3*R*,8*S*,9*S*,10*R*,13*R*,14*S*,17*R*)-10,13-Dimethyl-17-((*R*)-6-methylheptan-2-yl)-2,3,4,7,8,9,10,11,12,13,14,15,16,17-tetradecahydro-1*H*-cyclopenta[*a*]phenanthren-3-yl(2*R*,3*R*)-3-(5-bromopyridin-2-yl)-3-cyanooxirane-2-carboxylate (3ge)

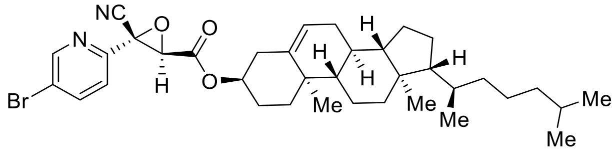

Compound **3ge** was synthesized following the General Procedure and subsequently purified using flash column chromatography (petroleum ether/EtOAc = 10/1), resulting in a colorless oil (56 mg) at 88% yield on a 0.1 mmol scale. The diastereomeric ratio of the resulting product was ascertained to exceed 20:1, as determined through crude ^{1}H NMR spectroscopic analysis.

^{1}H NMR (600 MHz, $CDCl_3$) δ 8.72 (d, *J* = 2.2 Hz, 1H), 7.95 (dd, *J* = 8.3, 2.3 Hz, 1H), 7.52 (d, *J* = 8.3 Hz, 1H), 5.34 (dt, *J* = 4.9, 2.1 Hz, 1H), 5.25 (p, *J* = 3.0 Hz, 1H), 4.13 (s, 1H), 2.57 (dp, *J* = 15.6, 3.1 Hz, 1H), 2.34 (dt, *J* = 15.6, 2.7 Hz, 1H), 2.01 (dt, *J* = 12.7, 3.5 Hz, 1H), 1.95 (ddt, *J* = 15.8, 10.6, 3.1 Hz, 2H), 1.87 − 1.78 (m, 2H), 1.68 (dt, *J* = 13.5, 3.5 Hz, 1H), 1.56 (s, 4H), 1.53 − 1.48 (m, 2H), 1.44 (ddd, *J* = 11.7, 7.4, 4.0 Hz, 3H), 1.34 (dt, *J* = 9.0, 2.5 Hz, 2H), 1.28 − 1.24 (m, 1H), 1.16 − 1.11 (m, 3H), 1.10 − 1.04 (m, 3H), 1.02 (s, 3H), 1.01 − 0.97 (m, 2H), 0.91 (d, *J* = 6.5 Hz, 3H), 0.87 (d, *J* = 2.7 Hz, 3H), 0.86 (d, *J* = 2.7 Hz, 3H), 0.68 (s, 3H).

^{13}C NMR (150 MHz, $CDCl_3$) δ 163.5, 151.6, 148.1, 140.3, 137.5, 123.2, 122.8, 122.6, 113.8, 74.6, 60.4, 56.9, 56.3, 53.2, 49.9, 42.5, 39.9, 39.7, 37.1, 36.3, 36.2, 35.9, 33.4, 32.0, 31.9, 28.4, 28.2, 26.2, 24.4, 24.0, 23.0, 22.7, 20.9, 19.0, 18.9, 12.0.

HRMS (ESI) *m/z* calcd. for $C_{36}H_{49}BrN_2NaO_3$ $[M+Na]^+$: 659.2819; found: 659.2867.

$[\alpha]_D^{20}$ = −42.4 (*c* 1.0, $CHCl_3$)

(3a*R*,3b*S*,6*S*,6*aS*,7*aR*)-2,2-dimethyl-5-oxohexahydrofuro[2',3':4,5]furo[2,3-*d*][1,3]dioxol-6-yl 4-((2*S*,3*R*)-3-(5-bromopyridin-2-yl)-3-cyanooxiran-2-yl)benzoate (3gf)

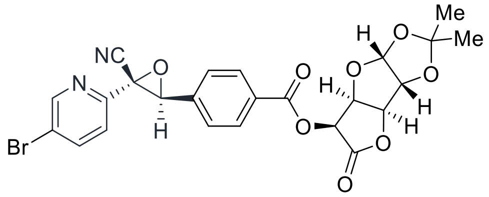

Compound **3gf** was synthesized following the General Procedure and subsequently purified using flash column chromatography (petroleum ether/EtOAc = 10/1), resulting in a colorless oil (53 mg) at 98% yield on a 0.1 mmol scale. The diastereomeric ratio of the resulting product was ascertained to exceed 20:1, as determined through crude ^{1}H NMR spectroscopic analysis.

^{1}H NMR (400 MHz, $CDCl_3$) δ 8.74 (dd, *J* = 2.3, 0.7 Hz, 1H), 8.22 (d, *J* = 8.5 Hz, 2H), 7.97 (dd, *J* = 8.4, 2.3 Hz, 1H), 7.60 (d, *J* = 8.4 Hz, 2H), 7.53 (dd, *J* = 8.4, 0.8 Hz, 1H), 6.05 (d, *J* = 3.6 Hz, 1H), 5.75 (d, *J* = 4.4 Hz, 1H), 5.19 (dd, *J* = 4.4, 2.9 Hz, 1H), 4.96 (d, *J* = 2.9 Hz, 1H), 4.87 (d, *J* = 3.6 Hz, 1H), 4.65 (s, 1H), 1.52 (s, 3H), 1.35 (s, 3H).

^{13}**C NMR** (100 MHz, $CDCl_3$) δ 169.6, 164.7, 151.5, 149.1, 140.3, 137.2, 130.8, 129.8, 127.0, 122.4, 122.1, 114.6, 113.7, 107.1, 82.7, 82.4, 77.3, 70.5, 66.4, 57.9, 27.0, 26.7.

HRMS (ESI) *m/z* calcd. for $C_{24}H_{19}BrN_2NaO$ $[M+Na]^+$: 565.0217; found: 565.0204.

$[\alpha]_D^{20}$ = −92.4 (*c* 1.0, $CHCl_3$)

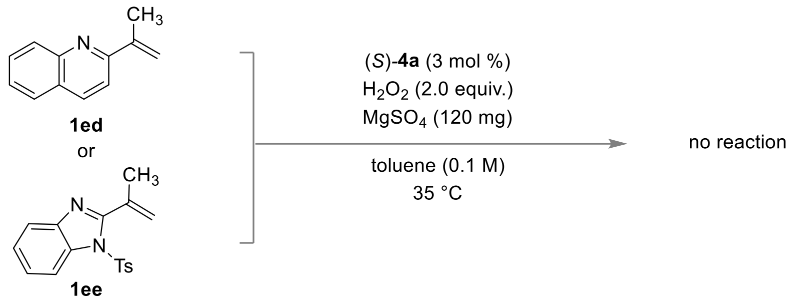

Supplementary Figure 69. Asymmetric epoxidation of methyl substituted alkenyl aza-heteroarenes

In a 4 mL vial equipped with a stirring bar, either substrate **1ed** or **1ee** (0.1 mmol, 1.0 equiv.) was added, along with (*S*)-**4a** (2 mg, 0.03 mmol, 0.03 equiv.) and $MgSO_4$ (120 mg). Freshly distilled toluene (1.0 mL) was then introduced into the vial. Subsequently, H_2O_2 (30% in H_2O *w*/*w*, 16 µL, 0.2 mmol, 2.0 equiv.) was added dropwise using microliter syringes. The reaction mixture was stirred at 35 °C. After 2 days, no desired product was detected in the reaction with substrates **1ed** or **1ee**.

4.2 General Procedure for the asymmetric epoxidation of mono-substituted and 1,2-disubstituted alkenyl aza-heteroarenes

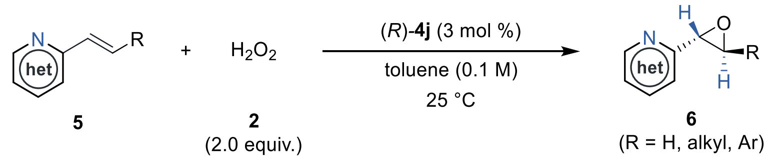

In a 4 mL vial equipped with a magnetic stir bar, a mixture of the corresponding alkenyl azaarenes (0.1 mmol, 1.0 equiv.) and (*R*)-**4j** (4 mg, 0.03 mmol, 0.03 equiv.) were placed. To this, freshly distilled toluene (1.0 mL) was added, followed by the dropwise addition of 30% aqueous H_2O_2 (30% in H_2O *w*/*w*, 16 µL, 0.2 mmol). The mixture was then stirred at 25 °C, with its progress monitored by TLC. Upon completion, the reaction mixture was filtered through a short celite pad. The filtrate was concentrated under reduced pressure and the product was purified using flash column chromatography on silica gel, resulting in the isolation of the desired epoxidation products.

(*S*)-2-(Oxiran-2-yl)quinoline (6a)

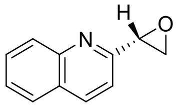

Compound **6a** was synthesized following the General Procedure and subsequently purified using flash column chromatography (petroleum ether/EtOAc = 10/1), resulting in a colorless oil (14 mg) at 81% yield on a 0.1 mmol scale.

^{1}H NMR (400 MHz, $CDCl_3$) δ 8.13 (d, *J* = 8.6 Hz, 1H), 8.07 (d, *J* = 8.5 Hz, 1H), 7.79 (dd, *J* = 8.1, 1.5 Hz, 1H), 7.71 (ddd, *J* = 8.5, 6.9, 1.5 Hz, 1H), 7.52 (ddd, *J* = 8.1, 6.9, 1.2 Hz, 1H), 7.27 (d, *J* = 8.6 Hz, 1H), 4.21 (dd, *J* = 4.3, 2.5 Hz, 1H), 3.26 (dd, *J* = 5.6, 4.2 Hz, 1H), 2.97 (dd, *J* = 5.5, 2.5 Hz, 1H).

^{13}C NMR (100 MHz, $CDCl_3$) δ 157.9, 147.7, 137.3, 130.0, 129.0, 128.0, 127.8, 126.7, 116.6, 53.5, 50.4.

HRMS (ESI) *m/z* calcd. for $C_{11}H_{10}NO$ $[M+H]^+$: 172.0757; found: 172.0766.

$[\alpha]_D^{20}$ = −13.9 (*c* 1.0, $CHCl_3$)

The enantiomeric excess was determined by chiral HPLC analysis using a Daicel Chiralpak ID column, e.e. = 87% (*n*-hexane/ethanol = 90/10, flow rate = 1.0 mL/min, λ = 250 nm, T = 20 °C, t_r (major) = 8.009 min, t_r (minor) = 7.398 min).

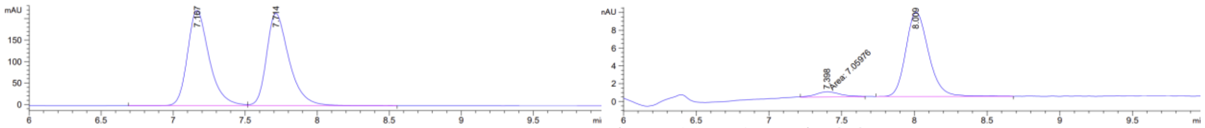

Signal 1: DAD1 A, Sig=250,4 Ref=off

Peak #	RetTime [min]	Type	Width [min]	Area [mAU*s]	Height [mAU]	Area %
1	7.167	BV	0.1582	2322.23389	221.66531	49.9096
2	7.714	VB	0.1644	2330.64502	215.14342	50.0904

Signal 1: DAD1 A, Sig=250,4 Ref=off

Peak #	RetTime [min]	Type	Width [min]	Area [mAU*s]	Height [mAU]	Area %
1	7.398	MM	0.1993	7.05976	5.90309e-1	6.3966
2	8.009	BB	0.1672	103.30720	9.47613	93.6034

Supplementary Figure 70. HPLC spectra of compound **6a**

(*S*)-7-Chloro-2-(oxiran-2-yl)quinoline (6b)

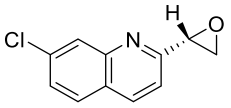

Compound **6b** was synthesized following the General Procedure and subsequently purified using flash column chromatography (petroleum ether/EtOAc = 10/1), resulting in a white solid (17 mg) at 85% yield on a 0.1 mmol scale.

m.p.: 113.4 − 121.3 °C

^{1}H NMR (600 MHz, $CDCl_3$) δ 8.11 (d, *J* = 8.6 Hz, 1H), 8.07 (d, *J* = 2.1 Hz, 1H), 7.72 (d, *J* = 8.7 Hz, 1H), 7.47 (dd, *J* = 8.7, 2.1 Hz, 1H), 7.27 (d, *J* = 8.5 Hz, 1H), 4.18 (dd, *J* = 4.2, 2.5 Hz, 1H), 3.26 (dd, *J* = 5.5, 4.2 Hz, 1H), 2.97 (dd, *J* = 5.5, 2.5 Hz, 1H).

^{13}C NMR (150 MHz, $CDCl_3$) δ 159.1, 148.1, 137.1, 135.8, 129.0, 128.2, 127.8, 126.3, 116.9, 53.4, 50.4.

HRMS (ESI) *m/z* calcd. for $C_{11}H_{19}ClNO$ $[M+H]^+$: 206.0367; found: 206.0384.

$[\alpha]_D^{20}$ = −14.9 (*c* 1.0, $CHCl_3$)

The enantiomeric excess was determined by chiral HPLC analysis using a Daicel Chiralpak IC column, e.e. = 96% (*n*-hexane/ethanol = 95/5, flow rate = 1.0 mL/min, λ = 230 nm, T = 20 °C, t_r (major) = 9.222 min, t_r (minor) = 8.508 min).

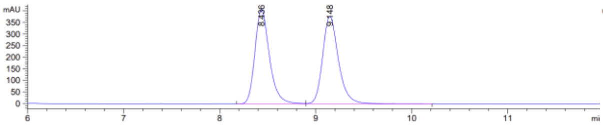

Signal 4: DAD1 D, Sig=230,4 Ref=off

Peak #	RetTime [min]	Type	Width [min]	Area [mAU*s]	Height [mAU]	Area %
1	8.436	BV	0.1607	4289.85938	407.83841	49.8207
2	9.148	VB	0.1735	4320.74316	377.53424	50.1793

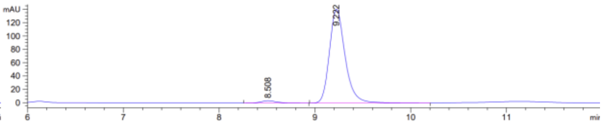

Signal 4: DAD1 D, Sig=230,4 Ref=off

Peak #	RetTime [min]	Type	Width [min]	Area [mAU*s]	Height [mAU]	Area %
1	8.508	BB	0.1623	35.98080	3.37669	2.2036
2	9.222	BB	0.1724	1596.83838	140.74402	97.7964

Supplementary Figure 71. HPLC spectra of compound **6b**

(*S*)-2-(Oxiran-2-yl)quinoxaline (6c)

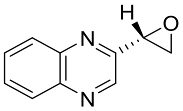

Compound **6c** was synthesized following the General Procedure and subsequently purified using flash column chromatography (petroleum ether/EtOAc = 10/1), resulting in a colorless oil (16 mg) at 91% yield on a 0.1 mmol scale.

^{1}H NMR (400 MHz, $CDCl_3$) δ 8.75 (s, 1H), 8.14 – 8.06 (m, 2H), 7.82 – 7.74 (m, 2H), 4.25 (dd, *J* = 4.2, 2.5 Hz, 1H), 3.35 (dd, *J* = 5.4, 4.2 Hz, 1H), 3.15 (dd, *J* = 5.4, 2.5 Hz, 1H).

^{13}C NMR (100 MHz, $CDCl_3$) δ 152.2, 142.5, 142.1, 141.8, 130.7, 130.2, 129.5, 129.2, 52.2, 50.5.

HRMS (ESI) *m/z* calcd. for $C_{10}H_9N_2O$ $[M+H]^+$: 173.0709; found: 173.0718.

$[\alpha]_D^{20}$ = −11.6 (*c* 1.0, $CHCl_3$)

The enantiomeric excess was determined by chiral HPLC analysis using a Daicel Chiralpak IA column, e.e. = 93% (*n*-hexane/ethanol = 70/30, flow rate = 1.0 mL/min, λ = 250 nm, T = 20 °C, t_r (major) = 8.896 min, t_r (minor) = 7.879 min).

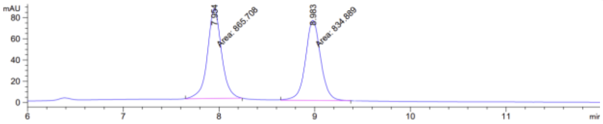

Signal 1: DAD1 A, Sig=250,4 Ref=off

Peak #	RetTime [min]	Type	Width [min]	Area [mAU*s]	Height [mAU]	Area %
1	7.954	MM	0.1706	865.70819	84.55813	50.9061
2	8.983	MM	0.1840	834.88934	75.61228	49.0939

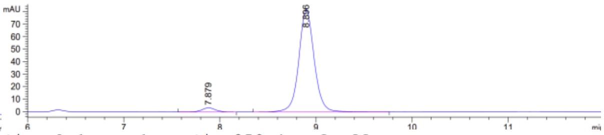

Signal 1: DAD1 A, Sig=250,4 Ref=off

Peak #	RetTime [min]	Type	Width [min]	Area [mAU*s]	Height [mAU]	Area %
1	7.879	BB	0.1492	32.12242	3.31016	3.4029
2	8.896	BB	0.1698	911.86090	81.94491	96.5971

Supplementary Figure 72. HPLC spectra of compound **6c**

Ethyl (*S*)−3−(oxiran−2−yl)quinoxaline−2−carboxylate (6d)

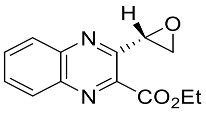

Compound **6d** was synthesized following the General Procedure and subsequently purified using flash column chromatography (petroleum ether/EtOAc = 10/1), resulting in a white solid (22 mg) at 91% yield on a 0.1 mmol scale.

m.p.: 107.4 − 110.3 °C

^{1}H NMR (400 MHz, $CDCl_3$) δ 8.21 (dd, *J* = 8.0, 1.9 Hz, 1H), 8.18 – 8.09 (m, 1H), 7.84 (dddd, *J* = 17.8, 8.4, 7.0, 1.6 Hz, 2H), 4.74 (dd, *J* = 4.1, 2.6 Hz, 1H), 4.58 (qq, *J* = 6.9, 3.7 Hz, 2H), 3.30 (dd, *J* = 6.1, 4.2 Hz, 1H), 3.26 (dd, *J* = 6.1, 2.6 Hz, 1H), 1.50 (t, *J* = 7.1 Hz, 3H).

^{13}C NMR (100 MHz, $CDCl_3$) δ 165.3, 151.0, 143.9, 142.6, 140.6, 132.4, 131.0, 130.0, 129.3, 62.9, 50.7, 49.8, 14.4.

HRMS (ESI) *m/z* calcd. for $C_{13}H_{13}N_2O_3$ $[M+H]^+$: 245.0921; found: 245.0918.

$[\alpha]_D^{20}$ = −104.4 (*c* 1.0, $CHCl_3$)

The enantiomeric excess was determined by chiral HPLC analysis using a Daicel Chiralpak IJ column, e.e. = 95% (*n*-hexane/ethanol = 90/10, flow rate = 1.0 mL/min, λ = 254 nm, T = 20 °C, t_r (major) = 18.951 min, t_r (minor) = 17.738 min).

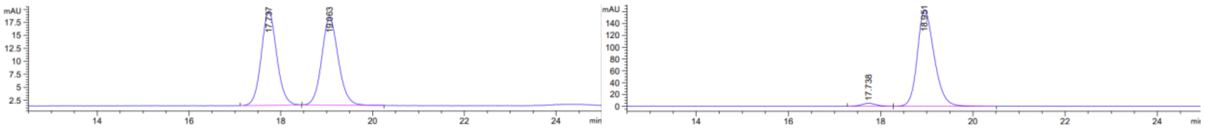

Signal 2: DAD1 B, Sig=254,4 Ref=off

Peak #	RetTime [min]	Type	Width [min]	Area [mAU*s]	Height [mAU]	Area %
1	17.737	BB	0.3592	424.44748	18.12268	49.9826
2	19.063	BB	0.3830	424.74298	16.79949	50.0174

Signal 2: DAD1 B, Sig=254,4 Ref=off

Peak #	RetTime [min]	Type	Width [min]	Area [mAU*s]	Height [mAU]	Area %
1	17.738	BB	0.3434	110.39172	4.92763	2.5969
2	18.951	BB	0.3857	4140.56885	163.38591	97.4031

Supplementary Figure 73. HPLC spectra of compound **6d**

(*S*)-2-(Oxiran-2-yl)-1,5-naphthyridine (6e)

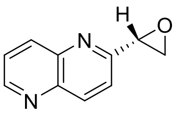

Compound **6e** was synthesized following the General Procedure and subsequently purified using flash column chromatography (petroleum ether/EtOAc = 10/1), resulting in a yellow oil (16 mg) at 95% yield on a 0.1 mmol scale.

^{1}H NMR (400 MHz, $CDCl_3$) δ 8.99 (dd, *J* = 4.2, 1.6 Hz, 1H), 8.47 – 8.39 (m, 2H), 7.68 (dd, *J* = 8.6, 4.2 Hz, 1H), 7.55 (d, *J* = 8.8 Hz, 1H), 4.26 (dd, *J* = 4.2, 2.5 Hz, 1H), 3.32 (dd, *J* = 5.5, 4.2 Hz, 1H), 3.03 (dd, *J* = 5.5, 2.5 Hz, 1H).

^{13}C NMR (100 MHz, $CDCl_3$) δ 158.9, 151.1, 143.7, 143.4, 138.5, 137.1, 124.8, 120.2, 53.3, 50.5.

HRMS (ESI) *m/z* calcd. for $C_{10}H_8N_2NaO$ $[M+Na]^+$: 195.0529; found: 195.0535.

$[\alpha]_D^{20}$ = −17.9 (*c* 1.0, $CHCl_3$)

The enantiomeric excess was determined by chiral HPLC analysis using a Daicel Chiralpak IC column, e.e. = 91% (*n*-hexane/ethanol = 90/10, flow rate = 1.0 mL/min, λ = 210 nm, T = 20 °C, t_r (major) = 18.613 min, t_r (minor) = 16.565 min).

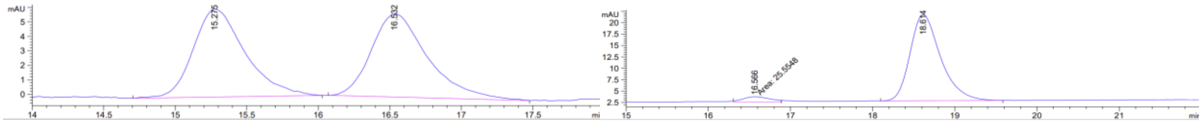

Signal 3: DAD1 C, Sig=210,4 Ref=off

Peak #	RetTime [min]	Type	Width [min]	Area [mAU*s]	Height [mAU]	Area %
1	15.275	BB	0.3622	152.52423	6.04866	49.3276
2	16.532	BB	0.3727	156.68259	5.73636	50.6724

Signal 3: DAD1 C, Sig=210,4 Ref=off

Peak #	RetTime [min]	Type	Width [min]	Area [mAU*s]	Height [mAU]	Area %
1	16.565	BB	0.3538	294.25189	12.63245	4.6820
2	18.613	BB	0.3883	5990.44385	232.70923	95.3180

Supplementary Figure 74. HPLC spectra of compound **6e**

(*S*)-1-(Oxiran-2-yl)isoquinoline (6f)

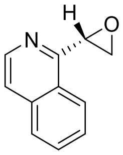

Compound **6f** was synthesized following the General Procedure and subsequently purified using flash column chromatography (petroleum ether/EtOAc = 10/1), resulting in a colorless oil (13 mg) at 75% yield on a 0.1 mmol scale.

^{1}H NMR (400 MHz, $CDCl_3$) δ 8.50 (d, *J* = 5.7 Hz, 1H), 8.40 (dd, *J* = 8.3, 1.1 Hz, 1H), 7.89 – 7.82 (m, 1H), 7.76 – 7.58 (m, 3H), 4.65 (dd, *J* = 4.1, 2.6 Hz, 1H), 3.35 (dd, *J* = 6.0, 2.6 Hz, 1H), 3.31 (dd, *J* = 6.0, 4.1 Hz, 1H).

^{13}C NMR (100 MHz, $CDCl_3$) δ 154.9, 142.2, 136.2, 130.3, 127.7, 127.6, 127.4, 124.2, 120.9, 51.0, 48.5.

HRMS (ESI) *m/z* calcd. for $C_{11}H_{10}NO$ $[M+H]^+$: 172.0757; found: 172.0761.

$[\alpha]_D^{20}$ = −20.8 (*c* 1.0, $CHCl_3$)

The enantiomeric excess was determined by chiral HPLC analysis using a Daicel Chiralpak IG column, e.e. = 89% (*n*-hexane/ethanol = 70/30, flow rate = 1.0 mL/min, λ = 250 nm, T = 20 °C, t_r (major) = 24.601 min, t_r (minor) = 28.568 min).

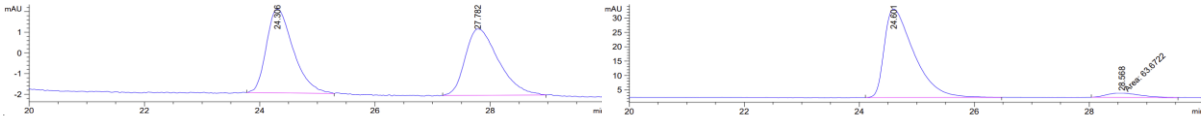

Signal 1: DAD1 A, Sig=250,4 Ref=off

Peak #	RetTime [min]	Type	Width [min]	Area [mAU*s]	Height [mAU]	Area %
1	24.306	BB	0.4531	131.08711	4.05592	50.4428
2	27.782	BB	0.4825	128.78571	3.20687	49.5572

Signal 1: DAD1 A, Sig=250,4 Ref=off

Peak #	RetTime [min]	Type	Width [min]	Area [mAU*s]	Height [mAU]	Area %
1	24.601	BB	0.5090	1094.41687	30.76720	94.5020
2	28.568	MM	0.6925	63.67223	1.53234	5.4980

Supplementary Figure 75. HPLC spectra of compound **6f**

(*S*)-4-(Oxiran-2-yl)-7-((2-(trimethylsilyl)ethoxy)methyl)-7*H*-pyrrolo[2,3-*d*]pyrimidine (6g)

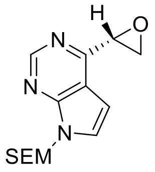

Compound **6g** was synthesized following the General Procedure and subsequently purified using flash column chromatography (petroleum ether/EtOAc = 10/1), resulting in a colorless oil (23 mg) at 81% yield on a 0.1 mmol scale.

^{1}H NMR (400 MHz, $CDCl_3$) δ 8.81 (s, 1H), 7.32 (d, *J* = 3.7 Hz, 1H), 6.74 (d, *J* = 3.7 Hz, 1H), 5.62 (s, 2H), 4.22 (dd, *J* = 4.2, 2.5 Hz, 1H), 3.54 – 3.43 (m, 2H), 3.27 (dd, *J* = 6.0, 4.3 Hz, 1H), 3.22 (dd, *J* = 6.0, 2.4 Hz, 1H), 0.91 – 0.81 (m, 2H), −0.09 (s, 9H).

^{13}C NMR (100 MHz, $CDCl_3$) δ 157.6, 152.1, 151.5, 128.8, 115.6, 100.4, 72.8, 66.7, 52.7, 49.6, 17.8, −1.4.

HRMS (ESI) *m/z* calcd. for $C_{14}H_{22}N_3O_2Si$ $[M+H]^+$: 292.1476; found: 292.1481.

$[\alpha]_D^{20}$ = −13.1 (*c* 1.0, $CHCl_3$)

The enantiomeric excess was determined by chiral HPLC analysis using a Daicel Chiralpak IE column, e.e. = 92% (*n*-hexane/ethanol =90/10, flow rate = 1.0 mL/min, λ = 254 nm, T = 20 ºC, t_r (major) = 12.863 min, t_r (minor) = 12.092 min).

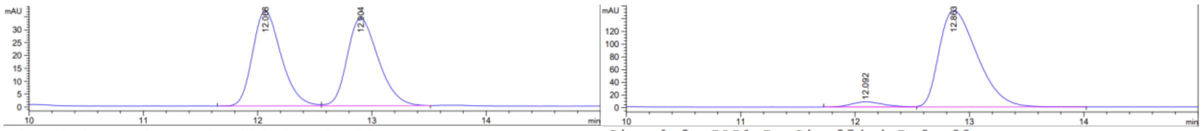

Signal 2: DAD1 B, Sig=254,4 Ref=off

Peak #	RetTime [min]	Type	Width [min]	Area [mAU*s]	Height [mAU]	Area %
1	12.068	BV	0.2664	639.09918	36.83306	49.8806
2	12.904	VB	0.2928	642.15820	33.93124	50.1194

Signal 2: DAD1 B, Sig=254,4 Ref=off

Peak #	RetTime [min]	Type	Width [min]	Area [mAU*s]	Height [mAU]	Area %
1	12.092	BV E	0.3037	156.34578	7.94104	4.2690
2	12.863	VB R	0.3598	3506.01196	151.61066	95.7310

Supplementary Figure 76. HPLC spectra of compound **6g**

Methyl (*S*)-6-(oxiran-2-yl)nicotinate (6h)

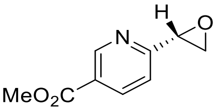

Compound **6h** was synthesized following the General Procedure and subsequently purified using flash column chromatography (petroleum ether/EtOAc = 10/1), resulting in a colorless oil (12 mg) at 77% yield on a 0.1 mmol scale.

^{1}H NMR (400 MHz, $CDCl_3$) δ 9.15 (d, J = 2.0 Hz, 1H), 8.28 (dd, J = 8.2, 2.1 Hz, 1H), 7.31 (d, J = 8.2 Hz, 1H), 4.07 (dd, J = 4.2, 2.5 Hz, 1H), 3.95 (s, 3H), 3.23 (dd, J = 5.7, 4.2 Hz, 1H), 2.93 (dd, J = 5.8, 2.4 Hz, 1H).

^{13}C NMR (100 MHz, $CDCl_3$) δ 165.6, 161.8, 150.7, 138.1, 125.6, 119.2, 52.7, 52.6, 50.9.

HRMS (ESI) *m/z* calcd. for $C_9H_{10}NO_3$ $[M+H]^+$: 180.0655; found: 180.0657.

$[\alpha]_D^{20}$ = −113.5 (c 1.0, $CHCl_3$)

The enantiomeric excess was determined by chiral HPLC analysis using a Daicel Chiralpak IA column, e.e. = 90% (n-hexane/ethanol = 90/10, flow rate = 1.0 mL/min, λ = 280 nm, T = 20 °C, t_r (major) = 19.681 min, t_r (minor) = 18.200 min).

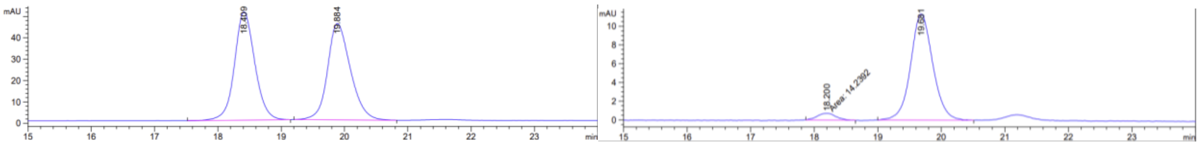

Signal 5: DAD1 E, Sig=280,4 Ref=off

Peak #	RetTime [min]	Type	Width [min]	Area [mAU*s]	Height [mAU]	Area %
1	18.409	BB	0.3406	1131.40735	50.65641	49.9771
2	19.884	BB	0.3848	1132.44287	45.12028	50.0229

Signal 5: DAD1 E, Sig=280,4 Ref=off

Peak #	RetTime [min]	Type	Width [min]	Area [mAU*s]	Height [mAU]	Area %
1	18.200	MM	0.3338	14.23915	7.11019e-1	4.9142
2	19.681	BB	0.3716	275.51614	11.33578	95.0858

Supplementary Figure 77. HPLC spectra of compound **6h**

(S)-4-(Oxiran-2-yl)-7-((2-(trimethylsilyl)ethoxy)methyl)-7H-pyrrolo[2,3-d]pyrimidine (6i)

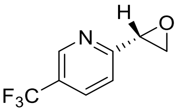

Compound **6i** was synthesized following the General Procedure and subsequently purified using flash column chromatography (petroleum ether/EtOAc = 10/1), resulting in a colorless oil (13 mg) at 73% yield on a 0.1 mmol scale.

^{1}H NMR (400 MHz, $CDCl_3$) δ 8.83 (s, 1H), 7.93 (d, J = 8.4 Hz, 1H), 7.37 (d, J = 8.3 Hz, 1H), 4.09 (d, J = 3.5 Hz, 1H), 3.23 (q, J = 4.0, 2.9 Hz, 1H), 3.00 – 2.86 (m, 1H).

^{13}C NMR (100 MHz, $CDCl_3$) δ 161.5, 146.5 (q, J = 4.1 Hz), 134.2 (d, J = 3.5 Hz), 126.1, 124.9, 119.2, 52.6, 50.9.

^{19}F NMR (376 MHz, $CDCl_3$) δ –65.6.

HRMS (ESI) *m/z* calcd. for $C_9H_{10}NO_3$ $[M+H]^+$: 190.0474; found: 190.0474.

$[\alpha]_D^{20}$ = −77.2 (*c* 1.0, $CHCl_3$)

The enantiomeric excess was determined by chiral HPLC analysis using a Daicel Chiralpak IJ column, e.e. = 94% (*n*-hexane/ethanol = 99/1, flow rate = 1.0 mL/min, λ = 210 nm, T = 20 °C, t_r (major) = 8.092 min, t_r (minor) = 7.332 min).

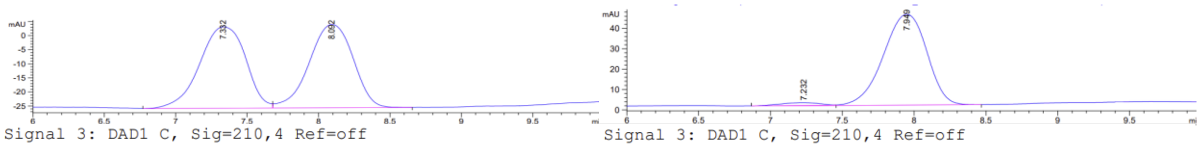

Signal 3: DAD1 C, Sig=210,4 Ref=off

Peak #	RetTime [min]	Type	Width [min]	Area [mAU*s]	Height [mAU]	Area %
1	7.332	BV	0.3614	647.42578	28.89619	50.6318
2	8.092	VB	0.3429	631.26782	29.59084	49.3682

Signal 3: DAD1 C, Sig=210,4 Ref=off

Peak #	RetTime [min]	Type	Width [min]	Area [mAU*s]	Height [mAU]	Area %
1	7.232	BV E	0.2475	27.58236	1.46624	2.8464
2	7.949	VB R	0.3318	941.43787	44.67569	97.1536

Supplementary Figure 78. HPLC spectra of compound **6i**

(*S*)-5-(Oxiran-2-yl)pyrazine-2-carbonitrile (6j)

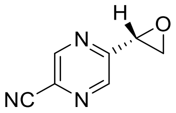

Compound **6j** was synthesized following the General Procedure and subsequently purified using flash column chromatography (petroleum ether/EtOAc = 10/1), resulting in a yellow solid (10 mg) at 82% yield on a 0.1 mmol scale.

m.p.: 66.1 – 70.4 °C

^{1}H NMR (600 MHz, $CDCl_3$) δ 8.84 (d, *J* = 1.5 Hz, 1H), 8.64 (d, *J* = 1.5 Hz, 1H), 4.12 (dd, *J* = 4.2, 2.4 Hz, 1H), 3.33 (dd, *J* = 5.6, 4.2 Hz, 1H), 3.05 (dd, *J* = 5.6, 2.4 Hz, 1H).

^{13}C NMR (150 MHz, $CDCl_3$) δ 156.2, 147.2, 142.7, 130.0, 115.3, 51.0, 50.9.

HRMS (ESI) *m/z* calcd. for $C_7H_6N_3O$ $[M+H]^+$: 148.0505; found: 148.0504.

$[\alpha]_D^{20}$ = −157.6 (*c* 1.0, $CHCl_3$)

The enantiomeric excess was determined by chiral HPLC analysis using a Daicel Chiralpak IF column, e.e. = 90% (*n*-hexane/ethanol = 70/30, flow rate = 1.0 mL/min, λ = 254 nm, T = 20 °C, t_r (major) = 21.730 min, t_r (minor) = 28.296 min).

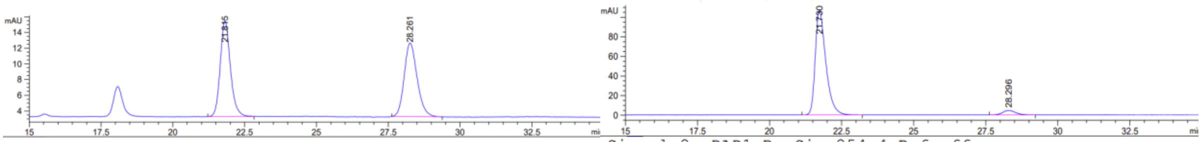

Signal 2: DAD1 B, Sig=254,4 Ref=off

Peak #	RetTime [min]	Type	Width [min]	Area [mAU*s]	Height [mAU]	Area %
1	21.815	BB	0.3752	300.66815	12.21277	50.6139
2	28.261	BB	0.4712	293.37418	9.37170	49.3861

Signal 2: DAD1 B, Sig=254,4 Ref=off

Peak #	RetTime [min]	Type	Width [min]	Area [mAU*s]	Height [mAU]	Area %
1	21.730	BB	0.3885	2722.49219	106.38975	95.0909
2	28.296	BB	0.4381	140.55104	4.57878	4.9091

Supplementary Figure 79. HPLC spectra of compound **6j**

Ethyl (*S*)-2-(oxiran-2-yl)pyrimidine-5-carboxylate (6k)

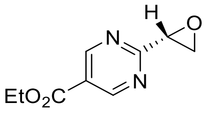

Compound **6k** was synthesized following the General Procedure and subsequently purified using flash column chromatography (petroleum ether/EtOAc = 10/1), resulting in a yellow solid (13 mg) at 84% yield on a 0.1 mmol scale.

m.p.: 66.1 – 70.4 °C

^{1}H NMR (600 MHz, $CDCl_3$) δ 9.22 (s, 2H), 4.43 (q, *J* = 7.2 Hz, 2H), 4.15 (dd, *J* = 4.1, 2.4 Hz, 1H), 3.26 (dd, *J* = 6.3, 2.4 Hz, 1H), 3.22 (dd, *J* = 6.4, 4.1 Hz, 1H), 1.41 (t, *J* = 7.1 Hz, 3H).

^{13}C NMR (150 MHz, $CDCl_3$) δ 169.3, 163.6, 158.6, 123.5, 62.1, 52.4, 49.8, 14.3.

HRMS (ESI) *m/z* calcd. for $C_9H_{11}N_2O_3$ $[M+H]^+$: 195.0764; found: 195.0771.

$[\alpha]_D^{20}$ = −86.5 (*c* 1.0, $CHCl_3$)

The enantiomeric excess was determined by chiral HPLC analysis using a Daicel Chiralpak IC column, e.e. = 86% (*n*-hexane/ethanol = 90/10, flow rate = 1.0 mL/min, λ = 230 nm, T = 20 °C, t_r (major) = 30.056 min, t_r (minor) = 25.358 min).

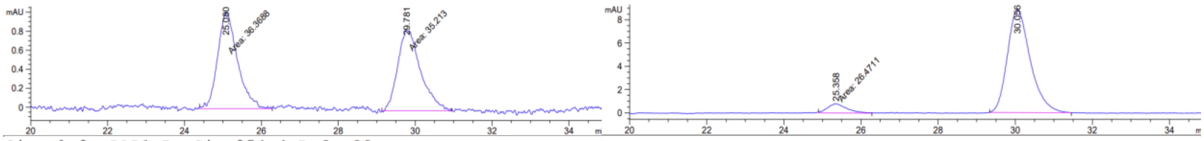

Signal 2: DAD1 B, Sig=254,4 Ref=off

Peak #	RetTime [min]	Type	Width [min]	Area [mAU*s]	Height [mAU]	Area %
1	25.080	MM	0.5951	36.36880	1.01863	50.8073
2	29.781	MM	0.6897	35.21297	8.50877e-1	49.1927

Signal 2: DAD1 B, Sig=254,4 Ref=off

Peak #	RetTime [min]	Type	Width [min]	Area [mAU*s]	Height [mAU]	Area %
1	25.358	MM	0.5631	26.47114	7.83559e-1	6.7551
2	30.056	BB	0.5532	365.39688	8.82218	93.2449

Supplementary Figure 80. HPLC spectra of compound **6k**

(*S*)-2-(Methylthio)-4-(oxiran-2-yl)pyrimidine (6l)

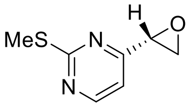

Compound **6l** was synthesized following the General Procedure and subsequently purified using flash column chromatography (petroleum ether/EtOAc = 10/1), resulting in a colorless oil (14 mg) at 85% yield on a 0.1 mmol scale.

^{1}H NMR (400 MHz, $CDCl_3$) δ 8.48 (d, *J* = 5.1 Hz, 1H), 6.86 (d, *J* = 5.1 Hz, 1H), 3.89 (dd, *J* = 4.1, 2.4 Hz, 1H), 3.19 (dd, *J* = 5.8, 4.3 Hz, 1H), 2.87 (dd, *J* = 5.8, 2.4 Hz, 1H), 2.58 (s, 3H).

^{13}C NMR (100 MHz, $CDCl_3$) δ 172.6, 166.6, 157.7, 111.2, 51.8, 50.3, 14.1.

HRMS (ESI) *m/z* calcd. for $C_7H_9N_2OS$ $[M+H]^+$: 169.0430; found: 169.0442.

[α]$_D^{20}$ = −29.7 (*c* 1.0, $CHCl_3$)

The enantiomeric excess was determined by chiral HPLC analysis using a Daicel Chiralpak IC column, e.e. = 94% (*n*-hexane/ethanol = 70/30, flow rate = 1.0 mL/min, λ = 250 nm, T = 20 ºC, t_r (major) = 8.110 min, t_r (minor) = 6.030 min).

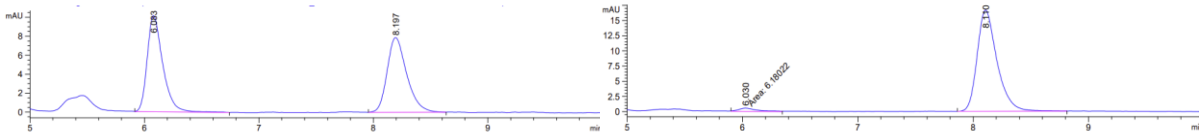

Signal 1: DAD1 A, Sig=250,4 Ref=off

Peak #	RetTime [min]	Type	Width [min]	Area [mAU*s]	Height [mAU]	Area %
1	6.083	VB	0.1422	95.87257	10.15075	50.6408
2	8.197	BB	0.1803	93.44626	7.88135	49.3592

Signal 1: DAD1 A, Sig=250,4 Ref=off

Peak #	RetTime [min]	Type	Width [min]	Area [mAU*s]	Height [mAU]	Area %
1	6.030	MM	0.1922	6.18022	5.35807e-1	3.0165
2	8.110	BB	0.1789	198.70276	16.68396	96.9835

Supplementary Figure 81. HPLC spectra of compound **6l**

(*R*)-2-(oxiran-2-yl)benzo[*d*]oxazole (6m)

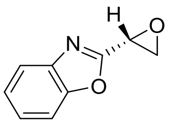

Compound **6m** was synthesized following the General Procedure and subsequently purified using flash column chromatography (petroleum ether/EtOAc = 10/1), resulting in a colorless oil (14 mg) at 88% yield on a 0.1 mmol scale.

^{1}H NMR (600 MHz, $CDCl_3$) δ 7.74 – 7.69 (m, 1H), 7.51 (dd, *J* = 7.5, 1.8 Hz, 1H), 7.40 – 7.32 (m, 2H), 4.16 (dd, *J* = 4.1, 2.5 Hz, 1H), 3.44 (dd, *J* = 5.7, 2.4 Hz, 1H), 3.28 (dd, *J* = 5.7, 4.1 Hz, 1H).

^{13}C NMR (150 MHz, $CDCl_3$) δ 161.9, 150.8, 141.1, 125.8, 124.9, 120.3, 111.0, 48.4, 45.9.

HRMS (ESI) *m/z* calcd. for $C_7H_8NO_2$ $[M+H]^+$: 162.0550; found: 162.0554.

[α]$_D^{20}$ = −117.9 (*c* 1.0, $CHCl_3$)

The enantiomeric excess was determined by chiral HPLC analysis using a Daicel Chiralpak IB column, e.e. = 96% (*n*-hexane/ethanol = 90/10, flow rate = 1.0 mL/min, λ = 250 nm, T = 20 ºC, t_r (major) = 6.014 min, t_r (minor) = 6.609 min).

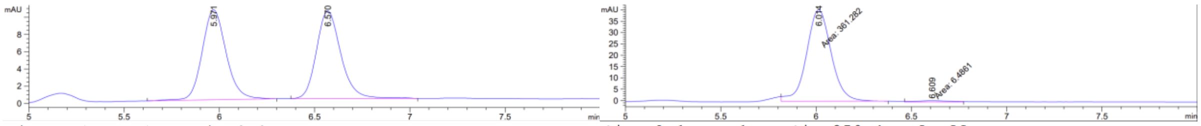

Signal 1: DAD1 A, Sig=250,4 Ref=off

Peak #	RetTime [min]	Type	Width [min]	Area [mAU*s]	Height [mAU]	Area %
1	5.971	BB	0.1338	91.08402	10.43658	50.2822
2	6.570	BB	0.1374	90.06174	10.16330	49.7178

Signal 1: DAD1 A, Sig=250,4 Ref=off

Peak #	RetTime [min]	Type	Width [min]	Area [mAU*s]	Height [mAU]	Area %
1	6.014	MM	0.1491	361.28217	40.37813	98.2364
2	6.609	MM	0.1874	6.48610	5.76794e-1	1.7636

Supplementary Figure 82. HPLC spectra of compound **6m**

(*R*)-2-(Oxiran-2-yl)-4,5-diphenyloxazole (6n)

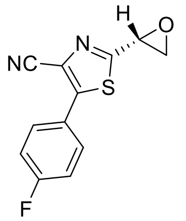

Compound **6n** was synthesized following the General Procedure and subsequently purified using flash column chromatography (petroleum ether/EtOAc = 10/1), resulting in a colorless oil (14 mg) at 88% yield on a 0.1 mmol scale.

The product was prepared according to the General Procedure and purified *via* flash column chromatography (petroleum ether/EtOAc = 10/1) to afford as a white solid (16 mg) in 73% yield on 0.1 mmol scale.

m.p.: 66.6 – 75.9 °C

^{1}H NMR (400 MHz, $CDCl_3$) δ 8.20 – 8.08 (m, 2H), 7.24 – 7.14 (m, 2H), 4.32 (dd, *J* = 4.0, 2.3 Hz, 1H), 3.32 (dd, *J* = 5.4, 4.0 Hz, 1H), 3.03 (dd, *J* = 5.4, 2.3 Hz, 1H).

^{13}C NMR (100 MHz, $CDCl_3$) δ 173.5, 165.4, 162.5 (d, *J* = 78.2 Hz), 130.3 (d, *J* = 8.7 Hz), 128.2 (d, *J* = 3.3 Hz), 116.3 (d, *J* = 22.0 Hz), 113.4, 98.6, 52.4, 50.6.

^{19}F NMR (376 MHz, $CDCl_3$) δ –112.0.

HRMS (ESI) *m/z* calcd. for $C_{12}H_8FN_2OS$ $[M+H]^+$: 247.0336; found: 247.0339.

$[\alpha]_D^{20}$ = −76.5 (*c* 1.0, $CHCl_3$)

The enantiomeric excess was determined by chiral HPLC analysis using a Daicel Chiralpak IF column, e.e. = 92% (*n*-hexane/ethanol = 70/30, flow rate = 1.0 mL/min, λ = 230 nm, T = 20 °C, t_r (major) = 9.869 min, t_r (minor) = 7.208 min).

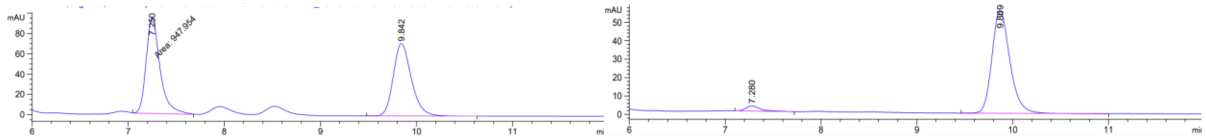

Signal 4: DAD1 D, Sig=230,4 Ref=off

Peak #	RetTime [min]	Type	Width [min]	Area [mAU*s]	Height [mAU]	Area %
1	7.250	MM	0.1675	947.95410	94.33677	50.4256
2	9.842	BB	0.2004	931.95160	71.33154	49.5744

Signal 4: DAD1 D, Sig=230,4 Ref=off

Peak #	RetTime [min]	Type	Width [min]	Area [mAU*s]	Height [mAU]	Area %
1	7.280	BB	0.1556	29.72870	2.85247	3.9255
2	9.869	BB	0.1973	727.59344	56.09359	96.0745

Supplementary Figure 83. HPLC spectra of compound **6n**

2-((2*S*,3*S*)-3-Butyloxiran-2-yl)quinoline (6o)

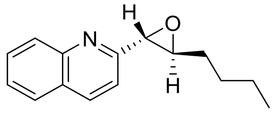

Compound **6o** was synthesized following the General Procedure and subsequently purified using flash column chromatography (petroleum ether/EtOAc = 10/1), resulting in a colorless oil (18 mg) at 82% yield on a 0.1 mmol scale.

^{1}H NMR (400 MHz, $CDCl_3$) δ 8.14 (d, J = 8.5 Hz, 1H), 8.08 (d, J = 8.5 Hz, 1H), 7.80 (d, J = 8.1 Hz, 1H), 7.71 (ddd, J = 8.4, 6.9, 1.5 Hz, 1H), 7.52 (ddd, J = 8.1, 6.9, 1.2 Hz, 1H), 7.27 (d, J = 8.1 Hz, 1H), 3.99 (d, J = 2.0 Hz, 1H), 3.13 (ddd, J = 6.7, 5.0, 2.0 Hz, 1H), 1.82 (dddd, J = 13.6, 8.3, 7.0, 4.8 Hz, 1H), 1.76 – 1.67 (m, 1H), 1.53 (dqd, J = 8.4, 7.1, 6.6, 4.3 Hz, 2H), 1.42 (h, J = 7.0 Hz, 2H), 0.94 (t, J = 7.2 Hz, 3H).

^{13}C NMR (100 MHz, $CDCl_3$) δ 158.3, 147.7, 137.2, 129.9, 129.0, 127.9, 127.8, 126.6, 116.9, 62.6, 59.6, 32.1, 28.2, 22.7, 14.1.

HRMS (ESI) *m/z* calcd. for $C_{15}H_{18}NO$ $[M+H]^+$: 228.1383; found: 228.1402.

$[\alpha]_D^{20}$ = −71.5 (c 1.0, $CHCl_3$)

The enantiomeric excess was determined by chiral HPLC analysis using a Daicel Chiralpak ID column, e.e. = 65% (n-hexane/ethanol = 70/30, flow rate = 1.0 mL/min, λ = 254 nm, T = 20 °C, t_r (major) = 5.470 min, t_r (minor) = 4.609 min).

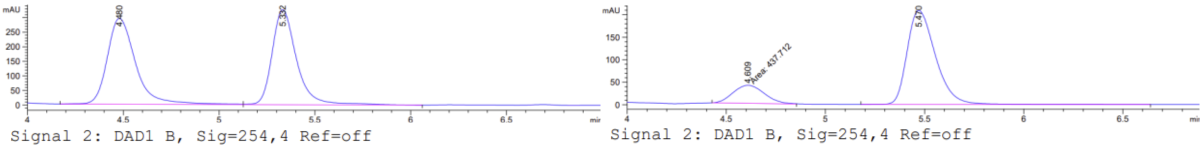

Signal 2: DAD1 B, Sig=254,4 Ref=off

Peak #	RetTime [min]	Type	Width [min]	Area [mAU*s]	Height [mAU]	Area %
1	4.480	BB	0.1534	218.47978	21.71287	50.3475
2	5.332	BB	0.1340	215.46346	24.63826	49.6525

Signal 2: DAD1 B, Sig=254,4 Ref=off

Peak #	RetTime [min]	Type	Width [min]	Area [mAU*s]	Height [mAU]	Area %
1	4.609	MM	0.1822	437.71161	40.03660	17.5128
2	5.470	BB	0.1532	2061.66479	208.86789	82.4872

Supplementary Figure 84. HPLC spectra of compound **6o**

2-((2*S*,3*S*)-3-(3-Chloropropyl)oxiran-2-yl)quinoxaline (6p)

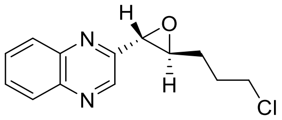

Compound **6p** was synthesized following the General Procedure and subsequently purified using flash column chromatography (petroleum ether/EtOAc = 10/1), resulting in a yellow oil (18 mg) at 71% yield on a 0.1 mmol scale.

^{1}H NMR (400 MHz, $CDCl_3$) δ 8.73 (s, 1H), 8.13 – 8.05 (m, 2H), 7.81 – 7.73 (m, 2H), 4.04 (d, J = 1.9 Hz, 1H), 3.71 – 3.61 (m, 2H), 3.37 – 3.31 (m, 1H), 2.06 (tq, J = 12.3, 6.1 Hz, 3H), 1.94 – 1.79 (m, 1H).

^{13}C NMR (100 MHz, $CDCl_3$) δ 152.0, 142.6, 142.3, 141.8, 130.6, 130.1, 129.5, 129.1, 61.6, 57.9, 44.4, 29.6, 29.0.

HRMS (ESI) *m/z* calcd. for $C_{13}H_{14}ClN_2O$ $[M+H]^+$: 249.0789; found: 249.0807.

$[\alpha]_D^{20}$ = −31.9 (*c* 1.0, $CHCl_3$)

The enantiomeric excess was determined by chiral HPLC analysis using a Daicel Chiralpak IC column, e.e. = 92% (*n*-hexane/ethanol =90/10, flow rate = 1.0 mL/min, λ = 250 nm, T = 20 ºC, t_r (major) = 9.317 min, t_r (minor) = 10.112 min).

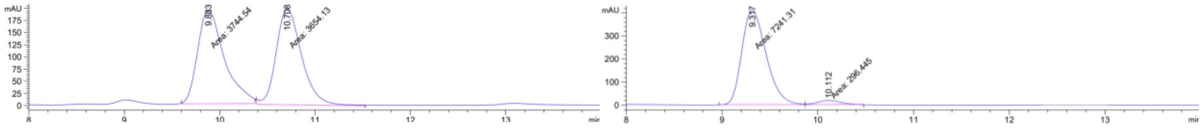

Signal 1: DAD1 A, Sig=250,4 Ref=off

Peak #	RetTime [min]	Type	Width [min]	Area [mAU*s]	Height [mAU]	Area %
1	9.883	MM	0.3239	3744.53906	192.70926	50.6110
2	10.708	MM	0.3092	3654.12988	196.99179	49.3890

Signal 1: DAD1 A, Sig=250,4 Ref=off

Peak #	RetTime [min]	Type	Width [min]	Area [mAU*s]	Height [mAU]	Area %
1	9.317	MM	0.2942	7241.30566	410.16983	96.0672
2	10.112	MM	0.2740	296.44531	18.03448	3.9328

Supplementary Figure 85. HPLC spectra of compound **6p**

2-((2*R*,3*S*)-3-Methyloxiran-2-yl)benzo[*d*]oxazole (6q)

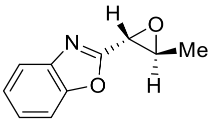

Compound **6q** was synthesized following the General Procedure and subsequently purified using flash column chromatography (petroleum ether/EtOAc = 10/1), resulting in a yellow oil (16 mg) at 92% yield on a 0.1 mmol scale.

^{1}H NMR (400 MHz, $CDCl_3$) δ 7.76 – 7.66 (m, 1H), 7.55 – 7.46 (m, 1H), 7.39 – 7.30 (m, 2H), 3.88 (d, *J* = 1.9 Hz, 1H), 3.68 (qd, *J* = 5.2, 2.0 Hz, 1H), 1.53 (d, *J* = 5.2 Hz, 3H).

^{13}C NMR (100 MHz, $CDCl_3$) δ 162.1, 150.8, 141.1, 125.7, 124.8, 120.3, 110.9, 56.6, 52.3, 17.5.

HRMS (ESI) *m/z* calcd. for $C_{10}H_{10}NO_2$ $[M+H]^+$: 176.0706; found: 176.0718.

$[\alpha]_D^{20}$ = −113.5 (*c* 1.0, $CHCl_3$)

The enantiomeric excess was determined by chiral HPLC analysis using a Daicel Chiralpak IF column, e.e. = 86% (*n*-hexane/ethanol = 95/5, flow rate = 1.0 mL/min, λ = 254 nm, T = 20 ºC, t_r (major) = 13.567 min, t_r (minor) = 14.413 min).

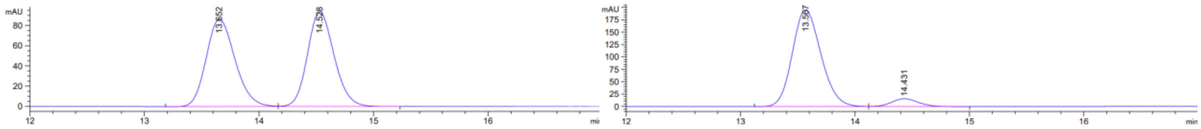

Signal 2: DAD1 B, Sig=254,4 Ref=off

Peak #	RetTime [min]	Type	Width [min]	Area [mAU*s]	Height [mAU]	Area %
1	13.652	BV	0.2719	1513.29504	86.58504	49.9294
2	14.528	VB	0.2505	1517.57214	93.92418	50.0706

Signal 2: DAD1 B, Sig=254,4 Ref=off

Peak #	RetTime [min]	Type	Width [min]	Area [mAU*s]	Height [mAU]	Area %
1	13.567	BV	0.2687	3398.04248	195.53755	92.9294
2	14.431	VB	0.2518	258.54224	15.89584	7.0706

Supplementary Figure 86. HPLC spectra of compound **6q**

2-((2*R*,3*S*)-3-(3-Chloropropyl)oxiran-2-yl)benzo[*d*]oxazole (6r)

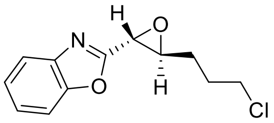

Compound **6r** was synthesized following the General Procedure and subsequently purified using flash column chromatography (petroleum ether/EtOAc = 10/1), resulting in a yellow oil (16 mg) at 87% yield on a 0.1 mmol scale.

^{1}H NMR (400 MHz, $CDCl_3$) δ 7.76 – 7.66 (m, 1H), 7.55 – 7.46 (m, 1H), 7.39 – 7.30 (m, 2H), 3.88 (d, *J* = 1.9 Hz, 1H), 3.68 (qd, *J* = 5.2, 2.0 Hz, 1H), 1.53 (d, *J* = 5.2 Hz, 3H).

^{13}C NMR (100 MHz, $CDCl_3$) δ 162.1, 150.8, 141.1, 125.7, 124.8, 120.3, 110.9, 56.6, 52.3, 17.5.

HRMS (ESI) *m/z* calcd. for $C_{12}H_{13}ClNO_2$ $[M+H]^+$: 238.0629; found: 238.0649.

$[\alpha]_D^{20}$ = −93.5 (*c* 1.0, $CHCl_3$)

The enantiomeric excess was determined by chiral HPLC analysis using a Daicel Chiralpak IG column, e.e. = 92% (*n*-hexane/ethanol = 70/30, flow rate = 1.0 mL/min, λ = 250 nm, T = 20 °C, t_r (major) = 20.908 min, t_r (minor) = 18.872 min).

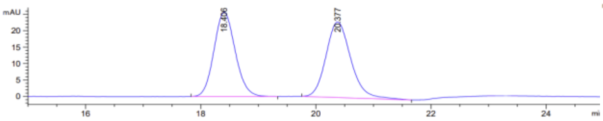

Signal 1: DAD1 A, Sig=250,4 Ref=off

Peak #	RetTime [min]	Type	Width [min]	Area [mAU*s]	Height [mAU]	Area %
1	18.407	BB	0.3952	864.97668	33.73066	49.9037
2	20.375	MM	0.4837	868.31512	29.91678	50.0963

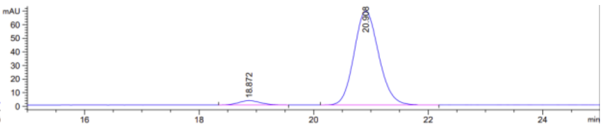

Signal 1: DAD1 A, Sig=250,4 Ref=off

Peak #	RetTime [min]	Type	Width [min]	Area [mAU*s]	Height [mAU]	Area %
1	18.872	BB	0.3613	83.27255	3.16133	3.8812
2	20.908	BB	0.4645	2062.24243	69.04249	96.1188

Supplementary Figure 87. HPLC spectra of compound **6r**

2-((2*S*,3*S*)-3-Phenyloxiran-2-yl)-1-tosyl-1*H*-benzo[*d*]imidazole (6s)

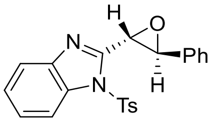

Compound **6s** was synthesized following the General Procedure and subsequently purified using flash column chromatography (petroleum ether/EtOAc = 10/1), resulting in a colorless oil (16 mg) at 87% yield on a 0.1 mmol scale.

^{1}H NMR (600 MHz, $CDCl_3$) δ 8.06 – 8.03 (m, 1H), 7.87 – 7.83 (m, 2H), 7.75 – 7.73 (m, 1H), 7.45 (d, *J* = 4.8 Hz, 4H), 7.43 – 7.40 (m, 2H), 7.37 (ddd, *J* = 8.5, 7.4, 1.2 Hz, 1H), 7.25 (d, *J* = 8.0 Hz, 2H), 4.82 (d, *J* = 1.9 Hz, 1H), 4.41 (d, *J* = 1.9 Hz, 1H), 2.38 (s, 3H).

^{13}C NMR (150 MHz, $CDCl_3$) δ 149.7, 146.5, 141.9, 135.4, 134.9, 133.2, 130.4, 129.2, 128.9, 127.3, 126.3, 125.9, 125.3, 121.0, 113.6, 60.6, 55.3, 21.9.

HRMS (ESI) *m/z* calcd. for $C_{22}H_{19}N_2O_3S$ $[M+H]^+$: 391.1111; found: 391.1119.

$[\alpha]_D^{20}$ = −49.0 (*c* 1.0, $CHCl_3$)

The enantiomeric excess was determined by chiral HPLC analysis using a Daicel Chiralpak IB column, e.e. = 89% (*n*-hexane/ethanol = 95/5, flow rate = 1.0 mL/min, λ = 250 nm, T = 20 °C, t_r (major) = 7.868 min, t_r (minor) = 10.049 min).

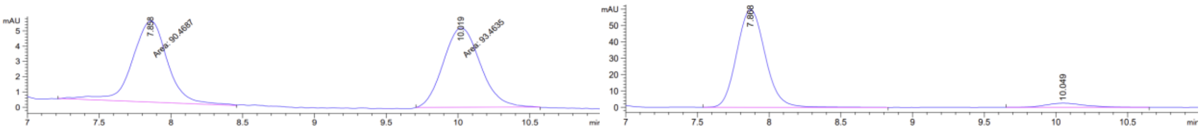

Signal 1: DAD1 A, Sig=250,4 Ref=off

Peak #	RetTime [min]	Type	Width [min]	Area [mAU*s]	Height [mAU]	Area %
1	7.858	MM	0.2813	105.19839	6.23297	49.6370
2	10.019	MM	0.3052	106.73717	5.82951	50.3630

Signal 1: DAD1 A, Sig=250,4 Ref=off

Peak #	RetTime [min]	Type	Width [min]	Area [mAU*s]	Height [mAU]	Area %
1	7.868	BB	0.2146	822.78070	59.76949	94.3264
2	10.049	BB	0.2834	49.48888	2.53918	5.6736

Supplementary Figure 88. HPLC spectra of compound **6s**

9-((3a*R*,4*R*,6*R*,6a*R*)-6-(((*tert*-Butyldimethylsilyl)oxy)methyl)-2,2-dimethyltetrahydrofuro[3,4-*d*][1,3]dioxol-4-yl)-6-((*S*)-oxiran-2-yl)-9*H*-purine (6u)

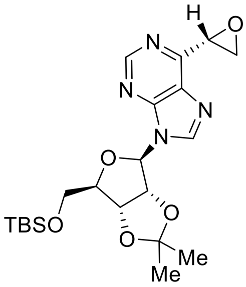

Compound **6u** was synthesized following the General Procedure and subsequently purified using flash column chromatography (petroleum ether/EtOAc = 10/1), resulting in a white solid (35 mg) at 85% yield on a 0.1 mmol scale. The diastereomeric ratio of the resulting product was ascertained to exceed 20:1, as determined through crude ^{1}H NMR spectroscopic analysis.

m.p.: 133.2 – 137.8 °C

^{1}H NMR (400 MHz, $CDCl_3$) δ 8.92 (s, 1H), 8.32 (s, 1H), 6.22 (d, *J* = 2.5 Hz, 1H), 5.24 (dd, *J* = 6.1, 2.5 Hz, 1H), 4.92 (dd, *J* = 6.1, 2.2 Hz, 1H), 4.53 (dd, *J* = 3.9, 2.5 Hz, 1H), 4.44 (q, *J* = 3.4 Hz, 1H), 3.86 (dd, *J* = 11.3, 3.5 Hz, 1H), 3.75 (dd, *J* = 11.3, 3.8 Hz, 1H), 3.54 (dd, *J* = 6.3, 2.4 Hz, 1H), 3.30 (dd, *J* = 6.3, 4.2 Hz, 1H), 1.62 (s, 3H), 1.38 (s, 3H), 0.79 (s, 9H), −0.02 (d, *J* = 3.0 Hz, 6H).

^{13}C NMR (100 MHz, $CDCl_3$) δ 156.2, 152.9, 151.0, 143.8, 133.5, 114.4, 91.9, 87.5, 85.1, 81.6, 63.7, 49.6, 49.2, 27.4, 26.0, 26.0, 25.5, 18.4, −5.3, −5.4.

HRMS (ESI) *m/z* calcd. for $C_{21}H_{33}N_4O_5Si$ $[M+H]^+$: 449.2215; found: 449.2226.

$[\alpha]_D^{20}$ = −26.5 (*c* 1.0, $CHCl_3$)

(*R*)-*N*-(5,6-Dimethoxypyrimidin-4-yl)-2-(oxiran-2-yl)benzo[*d*]thiazole-5-sulfonamide (6v)

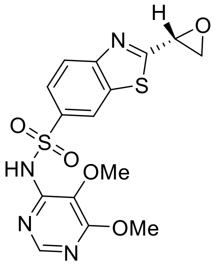

Compound **6v** was synthesized following the General Procedure and subsequently purified using flash column chromatography (petroleum ether/EtOAc = 10/1), resulting in a white solid (29 mg) at 74% yield on a 0.1 mmol scale.

m.p.: 111.3 – 118.4 °C

^{1}H NMR (400 MHz, DMSO-*d*$_6$) δ 11.36 (s, 1H), 8.83 (d, *J* = 1.8 Hz, 1H), 8.15 (d, *J* = 8.7 Hz, 1H), 8.10 – 8.05 (m, 1H), 4.54 (dd, *J* = 4.2, 2.4 Hz, 1H), 3.89 (s, 3H), 3.70 (s, 3H), 3.37 (dd, *J* = 5.4, 4.3 Hz, 1H), 3.14 (dd, *J* = 5.4, 2.4 Hz, 1H).

^{13}C NMR (100 MHz, DMSO-*d*$_6$) δ 175.0, 161.7, 155.2, 150.4, 150.2, 137.6, 133.8, 127.5, 125.2, 123.3, 79.2, 60.2, 54.1, 51.7, 50.2.

HRMS (ESI) *m/z* calcd. for $C_{15}H_{15}N_4O_5S_2$ $[M+H]^+$: 395.0478; found: 395.0489.

$[\alpha]_D^{20}$ = −33.1 (*c* 1.0, $CHCl_3$)

The enantiomeric excess was determined by chiral HPLC analysis using a Daicel Chiralpak IH column, e.e. = 97% (*n*-hexane/ethanol = 70/30, flow rate = 1.0 mL/min, λ = 250 nm, T = 20 ºC, t_r (major) = 32.980 min, t_r (minor) = 23.424 min).

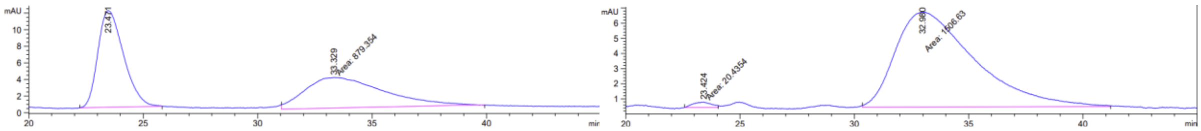

Signal 1: DAD1 A, Sig=250,4 Ref=off

Peak #	RetTime [min]	Type	Width [min]	Area [mAU*s]	Height [mAU]	Area %
1	23.471	BB	0.9153	904.91864	11.61046	50.7164
2	33.329	MM	3.9760	879.35443	3.68607	49.2836

Signal 1: DAD1 A, Sig=250,4 Ref=off

Peak #	RetTime [min]	Type	Width [min]	Area [mAU*s]	Height [mAU]	Area %
1	23.424	MM	0.9494	20.43536	3.58737e-1	1.3382
2	32.980	MM	4.0037	1506.62708	6.27183	98.6618

Supplementary Figure 89. HPLC spectra of compound **6v**

2.4. Scale-up reaction and synthetic applications of the chiral products

2.4.1 Gram-scale reaction

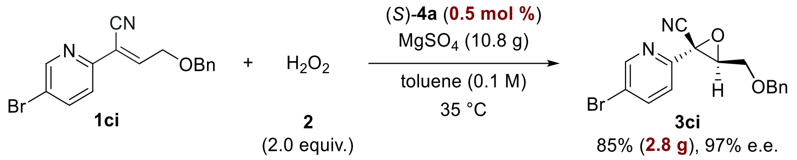

Acrylonitrile **1ci** (3.0 g, 9.1 mmol, 1.0 equiv.), (*S*)-**4a** (30 mg, 0.045 mmol, 0.05 equiv.), and $MgSO_4$ (10.9 g) were placed in a 250 mL vial, which was equipped with a stir bar. To this, freshly distilled toluene (91.0 mL) was added. Subsequently, H_2O_2 (30% in H_2O *w*/*w*, 1.45 mL, 18.2 mmol, 2.0 equiv.) was introduced dropwise using a syringe. The mixture was stirred at 35 ℃ and progress was monitored by TLC. After 7 hours, upon completion of the reaction, the mixture was filtered through a short celite pad. The filtrate was then concentrated under reduced pressure and the residue was purified by flash column chromatography on silica gel (petroleum ether/EtOAc = 20/1), yielding a white solid (2.8 g) in an 85% yield.

2.4.2 Product transformations

Methyl (2*S*,3*S*)-2-(5-bromopyridin-2-yl)-3-(((*tert*-butyldimethylsilyl)oxy)methyl)oxirane-2-carbimidate (7a)

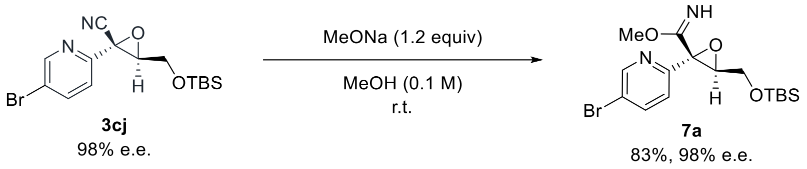

In a flame-dried 25 mL Schlenk flask equipped with a magnetic stir bar, a solution of **3cj** (37.0 mg, 0.1 mmol, 1.0 equiv.) in anhydrous methanol (1.0 mL, 0.1 M) was prepared. To this solution, sodium methoxide (5.5 M in MeOH, 22.0 μL, 0.12 mmol, 1.2 equiv.) was added dropwise at room temperature. The reaction mixture was then stirred for 12 hours at the same temperature. Upon completion, the reaction mixture was quenched with water (3.0 mL) in one portion and extracted with ethyl acetate (3.0 mL × 3). The combined organic layers were dried over anhydrous Na_2SO_4 and concentrated under reduced pressure. Finally, the residue was subjected to flash chromatography on silica gel (petroleum ether/EtOAc = 10/1), resulting in **7a** as a colorless oil (35.0 mg) in an 83% yield.

^{1}H NMR (400 MHz, $CDCl_3$) δ 8.64 (dd, *J* = 2.3, 0.7 Hz, 1H), 7.97 (s, 1H), 7.81 (dd, *J* = 8.4, 2.4 Hz, 1H), 7.28 (d, *J* = 8.2 Hz, 1H), 3.83 (s, 5H), 3.67 (dd, *J* = 10.8, 5.2 Hz, 1H), 0.89 (s, 9H), 0.07 (d, *J* = 4.3 Hz, 6H).

^{13}C NMR (100 MHz, $CDCl_3$) δ 167.1, 153.0, 150.8, 139.5, 123.6, 121.0, 64.4, 63.6, 61.7, 54.0, 25.9, 18.4, −5.1, −5.3.

HRMS (ESI) *m/z* calcd. for $C_{16}H_{25}BrN_2NaO_3Si$ $[M+Na]^+$: 423.0710; found: 423.0696.

$[\alpha]_D^{20}$ = −37.9 (*c* 1.0, $CHCl_3$)

The enantiomeric excess was determined by chiral HPLC analysis using a Daicel Chiralpak IC column, e.e. = 98% (*n*-hexane/ethanol = 90/10, flow rate = 1.0 mL/min, λ = 230 nm, T = 20 °C, t_r (major) = 4.632 min, t_r (minor) = 5.373 min).

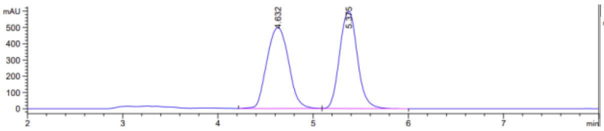

Signal 4: DAD1 D, Sig=230,4 Ref=off

Peak #	RetTime [min]	Type	Width [min]	Area [mAU*s]	Height [mAU]	Area %
1	4.632	BB	0.2648	7879.42334	497.34027	50.4914
2	5.375	BB	0.2031	7726.04297	596.34863	49.5086

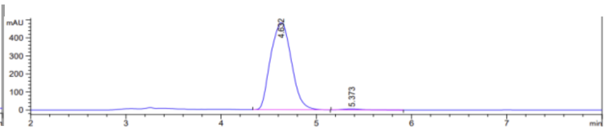

Signal 4: DAD1 D, Sig=230,4 Ref=off

Peak #	RetTime [min]	Type	Width [min]	Area [mAU*s]	Height [mAU]	Area %
1	4.632	BB	0.2484	7315.13965	483.67938	98.9369
2	5.373	BB	0.2248	78.60551	5.30770	1.0631

Supplementary Figure 90. HPLC spectra of compound **7a**

(2*S*,3*S*)-2-(5-Bromopyridin-2-yl)-3-(((*tert*-butyldimethylsilyl)oxy)methyl)oxirane-2-carbimidic acid (7b)

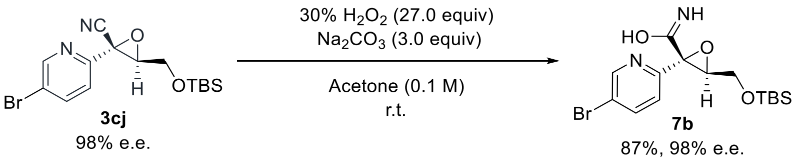

A solution of Compound **3cj** (37.0 mg, 0.1 mmol, 1.0 equiv.) was prepared in dry acetone (1.0 mL, 0.1 M) in a 4 mL sample vial. To this solution, a 1N aqueous solution of Na_2CO_3 (0.3 mL, 0.3 mmol, 3.0 equiv.) was added, followed by H_2O_2 (30% in H_2O *w*/*w*, 0.31 mL, 2.7 mmol, 27.0 equiv.). The mixture was stirred at room temperature for 12 hours, with the progress monitored by TLC. Upon completion, the solvent was evaporated under reduced pressure. The residue was taken up in ethyl acetate (8.0 mL) and washed with water. Additional extractions were performed with ethyl acetate (8.0 mL × 3). The combined organic layers were dried over anhydrous Na_2SO_4 and then concentrated under reduced pressure. The resulting residue was purified by column chromatography using petroleum ether/EtOAc (5:1) to yield **7b** as a colorless oil (34.0 mg), corresponding to an 87% yield.

^{1}H NMR (400 MHz, $CDCl_3$) δ 8.64 (d, *J* = 1.7 Hz, 1H), 7.84 (dd, *J* = 8.4, 2.4 Hz, 1H), 7.51 (d, *J* = 8.4 Hz, 1H), 7.31 (s, 1H), 6.16 (s, 1H), 4.02 (dd, *J* = 12.2, 3.9 Hz, 1H), 3.82 (dd, *J* = 12.1, 6.3 Hz, 1H), 3.58 (dd, *J* = 6.3, 3.9 Hz, 1H), 0.89 (s, 9H), 0.08 (d, *J* = 3.0 Hz, 6H).

^{13}C NMR (100 MHz, $CDCl_3$) δ 168.3, 153.1, 150.4, 139.9, 123.2, 120.9, 65.9, 62.7, 61.3, 26.0, 18.4, −5.1, −5.2.

HRMS (ESI) *m/z* calcd. for $C_{16}H_{25}BrN_2NaSi$ $[M+Na]^+$: 423.0710; found: 423.0696.

$[\alpha]_D^{20}$ = −45.7 (*c* 1.0, $CHCl_3$)

The enantiomeric excess was determined by chiral HPLC analysis using a Daicel Chiralpak IC column, e.e. = 98% (*n*-hexane/ethanol = 90/10, flow rate = 1.0 mL/min, λ = 230 nm, T = 20 °C, t_r (major) = 4.632 min, t_r (minor) = 5.373 min).

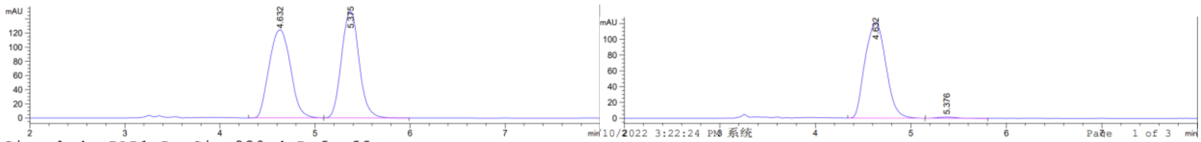

Signal 4: DAD1 D, Sig=230,4 Ref=off

Peak #	RetTime [min]	Type	Width [min]	Area [mAU*s]	Height [mAU]	Area %
1	4.632	BB	0.2648	7879.42334	497.34027	50.4914
2	5.375	BB	0.2031	7726.04297	596.34863	49.5086

Signal 4: DAD1 D, Sig=230,4 Ref=off

Peak #	RetTime [min]	Type	Width [min]	Area [mAU*s]	Height [mAU]	Area %
1	4.632	BB	0.2484	7315.13965	483.67938	98.9369
2	5.373	BB	0.2248	78.60551	5.30770	1.0631

Supplementary Figure 91. HPLC spectra of compound **7b**

((2*S*,3*R*)-2-(5-Bromopyridin-2-yl)-3-(dimethoxymethyl)oxiran-2-yl)(4-methoxyphenyl) methanone (7c)

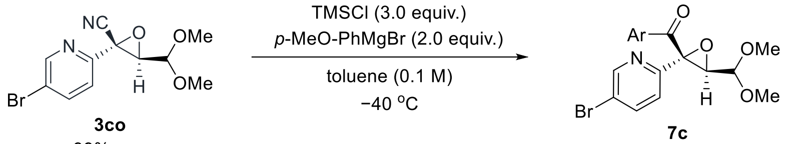

3co
99% e.e.

7c
87%, 98% e.e.
(Ar = 4-MeO-C_6H_4)

In a flame-dried 25 mL Schlenk flask equipped with a magnetic stir bar, a solution of **3co** (30.0 mg, 0.1 mmol, 1.0 equiv.) and TMSCl (27 µL, 0.3 mmol, 3.0 equiv.) in toluene (1.0 mL, 0.1 M) was prepared. Under a nitrogen atmosphere, 4-methoxyphenylmagnesium bromide (1.0 M in THF, 0.2 mL, 0.2 mmol, 2.0 equiv.) was added at −40 °C. The reaction mixture was stirred at this temperature for 3 hours. Completion of the reaction was confirmed by TLC. Subsequently, a saturated ammonium chloride aqueous solution (4.0 mL) was added slowly. The mixture was then extracted with ethyl acetate (4.0 mL × 3), and the combined organic layers were dried using anhydrous Na_2SO_4 and concentrated under reduced pressure. Purification of the residue by flash chromatography on silica gel (petroleum ether/EtOAc = 6/1) afforded **7c** as a colorless oil (35.0 mg) in an 87% yield.

^{1}H NMR (400 MHz, $CDCl_3$) δ 8.62 (d, *J* = 2.3 Hz, 1H), 7.86 (d, *J* = 8.9 Hz, 2H), 7.68 (dd, *J* = 8.4, 2.4 Hz, 1H), 7.13 (d, *J* = 8.3 Hz, 1H), 6.85 (d, *J* = 8.9 Hz, 2H), 4.40 (d, *J* = 7.2 Hz, 1H), 3.98 (d, *J* = 7.2 Hz, 1H), 3.81 (s, 3H), 3.47 (s, 3H), 3.21 (s, 3H).

^{13}C NMR (100 MHz, $CDCl_3$) δ 170.5, 162.2, 153.0, 151.1, 139.5, 130.1, 127.8, 124.9, 121.2, 113.9, 101.9, 66.3, 62.8, 55.5, 53.8.

HRMS (ESI) *m/z* calcd. for $C_{18}H_{18}BrNNaO_5$ $[M+Na]^+$: 430.0261; found: 430.0270.

$[\alpha]_D^{20}$ = −56.2 (*c* 1.0, $CHCl_3$)

The enantiomeric excess was determined by chiral HPLC analysis using a Daicel Chiralpak IC column, e.e. = 98% (*n*-hexane/ethanol = 90/10, flow rate = 1.0 mL/min, λ = 254 nm, T = 20 °C, t_r (major) = 16.961 min, t_r (minor) = 12.977 min).

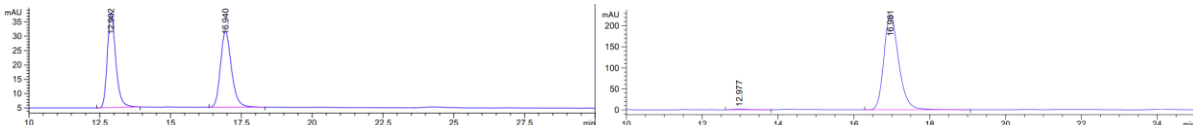

Signal 2: DAD1 B, Sig=254,4 Ref=off

Peak #	RetTime [min]	Type	Width [min]	Area [mAU*s]	Height [mAU]	Area %
1	12.902	BB	0.3171	688.71283	33.04556	49.1926
2	16.940	BB	0.4164	711.32135	26.39720	50.8074

Signal 2: DAD1 B, Sig=254,4 Ref=off

Peak #	RetTime [min]	Type	Width [min]	Area [mAU*s]	Height [mAU]	Area %
1	12.977	BB	0.3561	52.95634	2.15930	0.8558
2	16.961	BB	0.4161	6134.77002	226.41429	99.1442

Supplementary Figure 92. HPLC spectra of compound **7c**

(1*S*,5*S*)-1-(5-Bromopyridin-2-yl)-3,6-dioxabicyclo[3.1.0]hexan-2-one (7d)

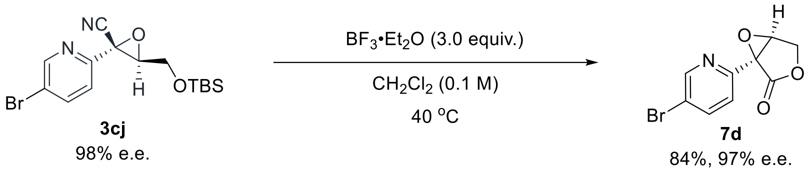

In a 4 mL sample vial, **3cj** (37.0 mg, 0.1 mmol, 1.0 equiv.) was dissolved in anhydrous dichloromethane (1.0 mL, 0.1 M) at room temperature. To this solution, boron trifluoride diethyl etherate (96.0 µL, 48% BF_3, 0.3 mmol, 3.0 equiv.) was added. The mixture was then stirred at 40 °C for 12 hours, with the progress of the reaction monitored by TLC. Upon completion, deionized water (2.0 mL) was added slowly and the solution was stirred at room temperature for an additional hour. The solution was extracted with ethyl acetate (3.0 mL × 3), and the combined organic layers were dried over anhydrous Na_2SO_4, then concentrated under reduced pressure. The resulting residue was purified using column chromatography (petroleum ether/EtOAc = 10/1), yielding **7d** as a white solid (21.0 mg) in an 84% yield.

m.p.: 123.1 − 126.9 °C

^{1}H NMR (400 MHz, $CDCl_3$) δ 8.70 (dd, *J* = 2.3, 0.8 Hz, 1H), 7.92 (dd, *J* = 8.4, 2.3 Hz, 1H), 7.84 (dd, *J* = 8.3, 0.8 Hz, 1H), 4.60 (d, *J* = 1.4 Hz, 1H), 4.55 (d, *J* = 11.4 Hz, 1H), 4.44 (dd, *J* = 11.5, 1.5 Hz, 1H).

^{13}C NMR (100 MHz, $CDCl_3$) δ 169.7, 151.3, 147.2, 139.6, 124.7, 121.8, 67.2, 62.8, 58.6.

HRMS (ESI) *m/z* calcd. for $C_9H_7BrNO_3$ $[M+H]^+$: 255.9604; found: 255.9626.

$[\alpha]_D^{20}$ = −49.5 (*c* 1.0, $CHCl_3$)

The enantiomeric excess was determined by chiral HPLC analysis using a Daicel Chiralpak IE column, e.e. = 97% (*n*-hexane/ethanol = 70/30, flow rate = 1.0 mL/min, λ = 254 nm, T = 20 °C, t_r (major) = 11.274 min, t_r (minor) = 10.321 min).

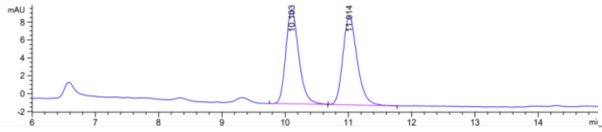

Signal 2: DAD1 B, Sig=254,4 Ref=off

Peak #	RetTime [min]	Type	Width [min]	Area [mAU*s]	Height [mAU]	Area %
1	10.103	BB	0.2183	149.64236	10.49628	49.1577
2	11.014	BB	0.2409	154.77057	9.87368	50.8423

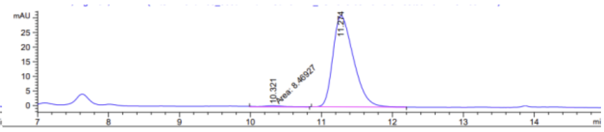

Signal 2: DAD1 B, Sig=254,4 Ref=off

Peak #	RetTime [min]	Type	Width [min]	Area [mAU*s]	Height [mAU]	Area %
1	10.321	MM	0.4084	8.46927	3.45657e-1	1.3323
2	11.274	BB	0.3068	627.21918	31.16813	98.6677

Supplementary Figure 93. HPLC spectra of compound **7d**

***tert*-Butyl (((2*S*,3*R*)-2-(5-bromopyridin-2-yl)-3-(dimethoxymethyl)oxiran-2-yl)methyl)carbamate (7e)**

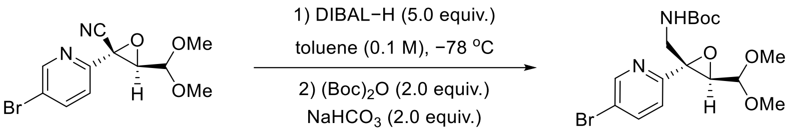

3co
99% e.e.

7e
63% 98% e.e.

In a flame-dried 25 mL Schlenk flask equipped with a magnetic stir bar, **3co** (30.0 mg, 0.1 mmol, 1.0 equiv.) was added and dissolved in toluene (1.0 mL, 0.1 M) under a nitrogen atmosphere. The reaction mixture was cooled to −78 °C, followed by the dropwise addition of diisobutylaluminium hydride (DIBAL-H) (0.5 mL, 1 M in hexane, 0.5 mmol, 5.0 equiv.) at the same temperature. The mixture was stirred for 3 hours at −78 °C, then quenched with methanol (5.0 mL) and gradually warmed to room temperature. A saturated Rochelle salt solution (10.0 mL) and ethyl acetate (5.0 mL) were added, and the mixture was stirred until both layers became clear. The aqueous phase was then extracted with ethyl acetate (5.0 mL × 3). The combined organic phases were dried over Na_2SO_4, filtered, and the crude product was obtained after solvent removal under reduced pressure.

In a separate 4 mL vial with a stir bar, the crude product was dissolved in methanol (1.0 mL, 0.1 M). Di-*tert*-butyl dicarbonate (44.0 mg, 0.2 mmol, 2.0 equiv.) and sodium bicarbonate (16.0 mg, 0.2 mmol, 2.0 equiv.) were added. The reaction mixture was stirred at room temperature for 24 hours, after which the solvents were removed under reduced pressure. The residue was purified using silica gel column chromatography to yield **7e** as a colorless oil (25.4 mg) with a 63% yield over the two steps.

^{1}H NMR (400 MHz, $CDCl_3$) δ 8.61 (d, *J* = 2.2 Hz, 1H), 7.80 (d, *J* = 8.5 Hz, 1H), 7.20 (dd, *J* = 8.4, 1.9 Hz, 1H), 5.57 (s, 1H), 4.55 (t, *J* = 6.1 Hz, 1H), 3.95 (dd, *J* = 14.5, 7.8 Hz, 1H), 3.79 (dd, *J* = 14.6, 4.4 Hz, 1H), 3.53 (s, 3H), 3.43 (s, 3H), 3.11 (d, *J* = 6.3 Hz, 1H), 1.42 (s, 9H).

^{13}C NMR (100 MHz, $CDCl_3$) δ 155.7, 150.0, 139.9, 121.0, 120.4, 101.8, 79.6, 65.5, 61.7, 54.8, 54.6, 41.5, 28.5.

HRMS (ESI) *m/z* calcd. for $C_{16}H_{23}BrN_2NaO_5$ $[M+Na]^+$: 425.0683; found: 425.0685.

$[\alpha]_D^{20}$ = −26.9 (*c* 1.0, $CHCl_3$)

The enantiomeric excess was determined by chiral HPLC analysis using a Daicel Chiralpak IA column, e.e. = 98% (*n*-hexane/ethanol = 90/10， flow rate = 1.0 mL/min, λ = 254 nm, T = 20 °C, t_r (major) = 13.878 min, t_r (minor) = 16.427 min).

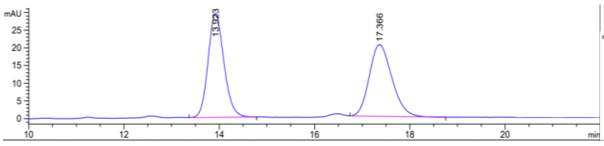

Signal 2: DAD1 B, Sig=254,4 Ref=off

Peak #	RetTime [min]	Type	Width [min]	Area [mAU*s]	Height [mAU]	Area %
1	13.923	BB	0.3535	677.83124	29.34550	50.3462
2	17.366	BB	0.5030	668.50916	20.25711	49.6538

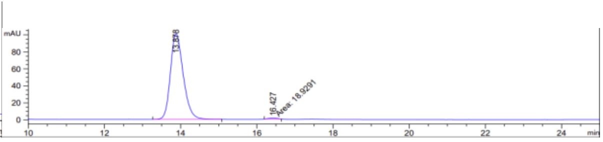

Signal 2: DAD1 B, Sig=254,4 Ref=off

Peak #	RetTime [min]	Type	Width [min]	Area [mAU*s]	Height [mAU]	Area %
1	13.878	BB	0.3576	2344.63257	100.72314	99.1991
2	16.427	MM	0.2754	18.92908	1.14540	0.8009

Supplementary Figure 94. HPLC spectra of compound **7e**

***tert*-Butyl ((2*S*,3*R*)-4-(benzyloxy)-2-(5-bromopyridin-2-yl)-3-chloro-2-hydroxybutyl) carbamate (7f)**

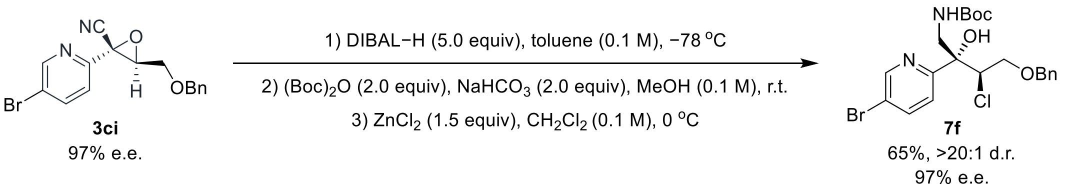

In a flame-dried 25 mL Schlenk flask containing a magnetic stir bar, **3ci** (34.0 mg, 0.1 mmol, 1.0 equiv.) was placed and dissolved in toluene (1.0 mL, 0.1 M) under a nitrogen atmosphere. The flask was then cooled to −78 °C, and diisobutylaluminium hydride (DIBAL-H) (0.5 mL, 1 M in hexane, 0.5 mmol, 5.0 equiv.) was added dropwise at this temperature. The reaction was stirred for 3 hours at −78 °C, after which it was quenched with methanol (5.0 mL) and gradually warmed to room temperature. A saturated Rochelle salt solution (10.0 mL) and ethyl acetate (5.0 mL) were added, and the mixture was stirred until clarity was achieved in both layers. The aqueous phase was then extracted three times with ethyl acetate (5.0 mL × 3). The combined organic phases were dried over Na_2SO_4, filtered, and the crude product was obtained after evaporation of the solvents under reduced pressure.

In a 4 mL vial with a stirring bar, this crude product was dissolved in methanol (1.0 mL, 0.1 M). Di-*tert*-butyl dicarbonate (44.0 mg, 0.2 mmol, 2.0 equiv.) and sodium bicarbonate (16.0 mg, 0.2 mmol, 2.0 equiv.) were added, and the mixture was stirred at room temperature for 24 hours. The mixture was then filtered to remove insoluble solids, and the solvents were removed under reduced pressure to yield a crude product.

Subsequently, in another 4 mL vial equipped with a stirring bar, the crude product was dissolved in dichloromethane (1.0 mL, 0.1 M). Zinc chloride (20.0 mg, 0.15 mmol, 1.5 equiv.) was added at 0 °C, and the mixture was stirred for 2 hours at the same temperature. The reaction mixture was filtered to

remove insoluble solids, and the solvent was concentrated under reduced pressure. The residue was purified by column chromatography (petroleum ether/EtOAc = 10/1) to yield **7f** as a colorless oil (31.0 mg) in 65% yield over three steps. The diastereomeric ratio of the resulting product was ascertained to exceed 20:1, as determined through crude ^{1}H NMR spectroscopic analysis.

^{1}H NMR (600 MHz, $CDCl_3$) δ 8.54 (d, J = 2.0 Hz, 1H), 7.77 (dd, J = 8.4, 2.2 Hz, 1H), 7.56 (d, J = 8.4 Hz, 1H), 7.32 − 7.27 (m, 3H), 7.14 (d, J = 6.7 Hz, 2H), 4.72 (s, 1H), 4.58 (t, J = 4.6 Hz, 1H), 4.45 − 4.30 (m, 2H), 3.81 (d, J = 6.1 Hz, 2H), 3.62 (d, J = 5.1 Hz, 2H), 1.31 (s, 9H).

^{13}C NMR (150 MHz, $CDCl_3$) δ 159.3, 157.0, 149.2, 139.4, 137.3, 128.5, 127.9, 127.8, 123.3, 119.8, 80.0, 79.1, 73.4, 71.3, 65.0, 48.3, 28.3.

HRMS (ESI) *m/z* calcd. for $C_{21}H_{27}BrClN_2O_4$ $[M+H]^+$: 485.0837; found: 485.0846.

$[\alpha]_D^{20}$ = −40.7 (c 1.0, $CHCl_3$)

The enantiomeric excess was determined by chiral HPLC analysis using a Daicel Chiralpak ID column, e.e. = 97% (*n*-hexane/*i*-propanol = 90/10, flow rate = 1.0 mL/min, λ = 230 nm, T = 20 °C, t_r (major) = 10.754 min, t_r (minor) = 15.584 min).

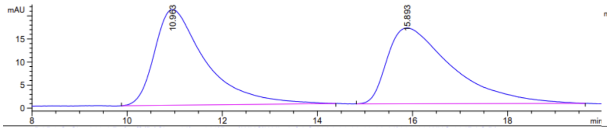

Signal 2: DAD1 B, Sig=254,4 Ref=off

Peak #	RetTime [min]	Type	Width [min]	Area [mAU*s]	Height [mAU]	Area %
1	10.963	BB	0.9824	1547.19910	20.73735	49.8438
2	15.893	BB	1.1204	1556.89661	16.41659	50.1562

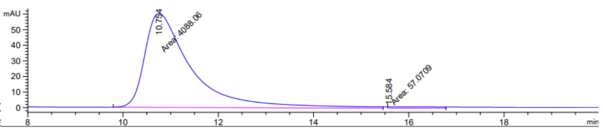

Signal 2: DAD1 B, Sig=254,4 Ref=off

Peak #	RetTime [min]	Type	Width [min]	Area [mAU*s]	Height [mAU]	Area %
1	10.754	MM	1.1308	4088.05713	60.25183	98.6232
2	15.584	MM	1.1195	57.07085	8.49683e-1	1.3768

Supplementary Figure 95. HPLC spectra of compound **7f**

(1*R*,2*R*)-3-(Benzyloxy)-1-(5-bromopyridin-2-yl)-2-chloro-1-cyanopropyl acetate (7g):

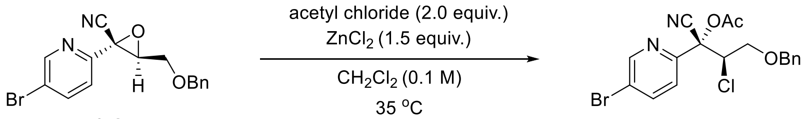

3ci 97% e.e.   **7g** 30%, 10:1 d.r. 97% e.e.

In a 4 mL sample vial, **3ci** (34.0 mg, 0.1 mmol, 1.0 equiv.) was dissolved in dichloromethane (1.0 mL, 0.1 M) at room temperature. Zinc chloride (20.0 mg, 0.15 mmol, 1.5 equiv.) and acetyl chloride (15.0 µL, 2.0 equiv.) were then added to the solution, which was stirred at 35 °C for 12 hours, with the reaction progress monitored by TLC. Upon completion, a saturated sodium bicarbonate aqueous solution (2.0 mL) was added, followed by extraction with dichloromethane (5.0 mL × 3). The combined organic layers were dried over Na_2SO_4 and the solvent was then removed under reduced pressure. The resulting residue was purified by column chromatography (petroleum ether/EtOAc = 20/1), yielding **7g** as a colorless oil (12.0 mg) in a 30% yield. The diastereomeric ratio of the resulting

product was ascertained to be 10:1, as determined through crude ^{1}H NMR spectroscopic analysis. Spectroscopic data collected only for major isomer.

^{1}H NMR (400 MHz, $CDCl_3$) δ 8.62 (d, *J* = 1.6 Hz, 1H), 7.91 (dd, *J* = 8.4, 2.3 Hz, 1H), 7.70 (d, *J* = 8.4 Hz, 1H), 7.45 – 7.29 (m, 5H), 4.72 (dd, *J* = 7.4, 3.1 Hz, 1H), 4.65 (d, *J* = 4.3 Hz, 2H), 4.17 (dd, *J* = 11.0, 3.1 Hz, 1H), 3.95 (dd, *J* = 11.0, 7.4 Hz, 1H), 2.09 (s, 3H).

^{13}C NMR (100 MHz, $CDCl_3$) δ 167.7, 151.1, 150.9, 139.6, 137.4, 128.6, 128.1, 127.9, 124.9, 121.8, 114.5, 73.7, 70.2, 61.9, 20.8.

HRMS (ESI) *m/z* calcd. for $C_{18}H_{17}BrClN_2O_3$ $[M+H]^+$: 423.0106; found: 423.0102.

$[\alpha]_D^{20}$ = −22.9 (*c* 1.0, $CHCl_3$)

The enantiomeric excess was determined by chiral HPLC analysis using a Daicel Chiralpak ID column, e.e. = 97% (*n*-hexane/ethanol = 98/2, flow rate = 1.0 mL/min, λ = 254 nm, T = 20 °C, t_r (major) = 14.492 min, t_r (minor) = 16.570 min).

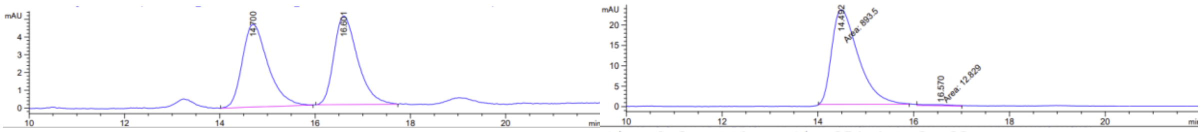

Signal 2: DAD1 B, Sig=254,4 Ref=off

Peak #	RetTime [min]	Type	Width [min]	Area [mAU*s]	Height [mAU]	Area %
1	14.700	BB	0.4944	175.10951	4.65984	50.3800
2	16.601	BB	0.4910	172.46762	4.99230	49.6200

Signal 2: DAD1 B, Sig=254,4 Ref=off

Peak #	RetTime [min]	Type	Width [min]	Area [mAU*s]	Height [mAU]	Area %
1	14.492	MM	0.6410	893.50000	23.23089	98.5845
2	16.570	MM	0.7220	12.82899	2.96158e-1	1.4155

Supplementary Figure 96. HPLC spectra of compound **7g**

(2*R*,3*R*)-2-Bromo-2-(5-bromopyridin-2-yl)-4-((tert-butyldimethylsilyl)oxy)-3-hydroxybutanenitrile (7h):

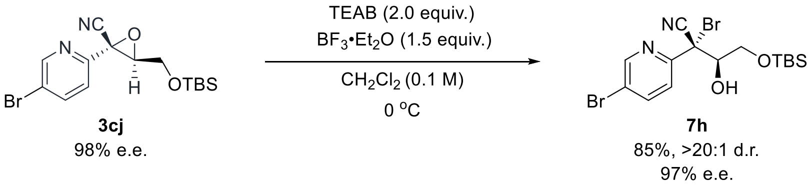

In a flame-dried 25 mL Schlenk flask equipped with a magnetic stir bar, **3cj** (37.0 mg, 0.1 mmol, 1.0 equiv.) and tetraethyl ammonium bromide (TEAB) (42.5 mg, 0.2 mmol, 2.0 equiv.) were added and dissolved in anhydrous dichloromethane (1.0 mL, 0.1 M) under a nitrogen atmosphere at 0 °C. Subsequently, boron trifluoride diethyl etherate (48.0 μL, 48% BF_3, 0.15 mmol, 1.5 equiv.) was introduced, and the solution was stirred at 0 °C for 1 hour, with the reaction progress monitored by TLC. Upon completion, the reaction was quenched with deionized water (5.0 mL) at the same temperature, followed by extraction with dichloromethane (5.0 mL × 3). The organic layers were combined, dried over anhydrous Na_2SO_4, and concentrated under reduced pressure. The residue was then purified by flash chromatography on silica gel (petroleum ether/EtOAc = 20/1), yielding **7h** as a

yellow oil (44.0 mg) in an 85% yield. The diastereomeric ratio of the resulting product was ascertained to exceed 20:1, as determined through crude ^{1}H NMR spectroscopic analysis.

^{1}H NMR (400 MHz, $CDCl_3$) δ 8.69 (d, J = 2.1 Hz, 1H), 7.94 (dd, J = 8.4, 2.3 Hz, 1H), 7.74 (d, J = 8.4 Hz, 1H), 4.66 (t, J = 4.7 Hz, 1H), 4.15 (dd, J = 4.7, 1.6 Hz, 2H), 3.83 (s, 1H), 0.91 (s, 9H), 0.12 (d, J = 2.4 Hz, 6H).

^{13}C NMR (100 MHz, $CDCl_3$) δ 153.3, 150.9, 140.3, 123.5, 121.7, 116.8, 75.3, 63.6, 49.3, 26.0, 18.5, −5.3, −5.4.

HRMS (ESI) *m/z* calcd. for $C_{15}H_{12}Br_2N_2O_2Si$ $[M+H]^+$: 448.9890; found: 448.9861.

$[\alpha]_D^{20}$ = −21.5 (c 1.0, $CHCl_3$)

The enantiomeric excess was determined by chiral HPLC analysis using a Daicel Chiralpak IM column, e.e. = 97% (*n*-hexane/ethanol = 98/2, flow rate = 1.0 mL/min, λ = 254 nm, T = 20 ºC, t_r (major) = 8.857 min, t_r (minor) = 8.430 min).

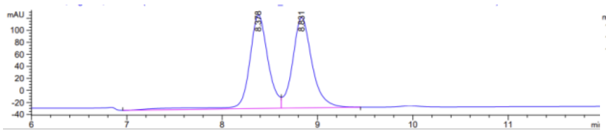

Signal 2: DAD1 B, Sig=254,4 Ref=off

Peak #	RetTime [min]	Type	Width [min]	Area [mAU*s]	Height [mAU]	Area %
1	8.378	BV	0.2080	2161.29346	155.61636	50.6594
2	8.831	VB	0.2094	2105.02905	150.31381	49.3406

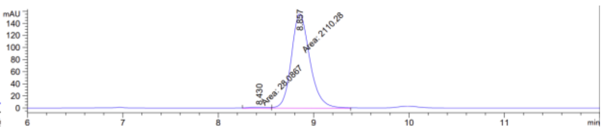

Signal 2: DAD1 B, Sig=254,4 Ref=off

Peak #	RetTime [min]	Type	Width [min]	Area [mAU*s]	Height [mAU]	Area %
1	8.430	MM	0.2429	28.08674	1.92740	1.3135
2	8.857	MM	0.2256	2110.27612	155.87469	98.6865

Supplementary Figure 97. HPLC spectra of compound **7h**

(2*R*,3*R*)-5-Amino-3-(5-bromopyridin-2-yl)-2-(dimethoxymethyl)-2,3-dihydrofuran-3-carbonitrile (7i)

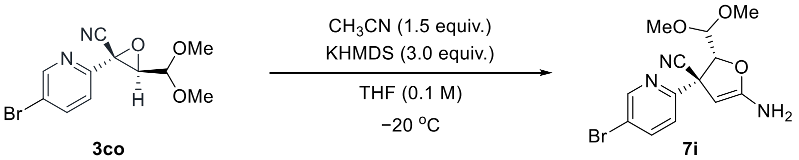

3co 99% e.e.

7i 76%, >20:1 d.r. 98% e.e.

In a flame-dried 25 mL Schlenk flask equipped with a magnetic stir bar, potassium bis(trimethylsilyl)amide (KHMDS) (0.3 mL, 1 M in THF, 0.3 mmol, 3.0 equiv.) was added under a nitrogen atmosphere and cooled to −20 °C. Acetonitrile (9.0 µL, 0.15 mmol, 1.5 equiv.) was then introduced and the mixture was allowed to react at −20 °C for 5 minutes. Separately, **3co** (30.0 mg, 0.1 mmol, 1.0 equiv.) was dissolved in anhydrous THF (1.0 mL, 0.1 M) and this solution was added dropwise to the flask, maintaining the temperature at −20 °C. The reaction mixture was stirred for 2 hours. Upon completion, a saturated ammonium chloride aqueous solution (5.0 mL) was added. The resultant mixture was extracted with ethyl acetate (5.0 mL × 3). The combined organic layers were dried over anhydrous Na_2SO_4 and concentrated under reduced pressure. Purification by flash

chromatography on silica gel (dichloromethane/methanol = 80/1) yielded **7i** as a yellow oil (26.0 mg) in a 76% yield. The diastereomeric ratio of the resulting product was ascertained to exceed 20:1, as determined through crude ^{1}H NMR spectroscopic analysis.

^{1}H NMR (400 MHz, $CDCl_3$) δ 8.61 (dd, *J* = 2.3, 0.7 Hz, 1H), 7.87 (dd, *J* = 8.4, 2.3 Hz, 1H), 7.35 (dd, *J* = 8.4, 0.7 Hz, 1H), 5.76 (s, 2H), 4.36 (s, 1H), 4.21 (d, *J* = 7.0 Hz, 1H), 3.47 (s, 3H), 3.45 (s, 3H), 3.34 (d, *J* = 7.0 Hz, 1H).

^{13}C NMR (100 MHz, $CDCl_3$) δ 157.5, 154.1, 150.1, 140.4, 122.0, 121.2, 118.2, 101.1, 64.6, 64.4, 61.9, 55.4, 54.1.

HRMS (ESI) *m/z* calcd. for $C_{13}H_{14}BrN_3NaO_3$ $[M+Na]^+$: 362.0111; found: 362.0106.

$[\alpha]_D^{20}$ = −33.1 (*c* 1.0, $CHCl_3$)

The enantiomeric excess was determined by chiral HPLC analysis using a Daicel Chiralpak IA column, e.e. = 98% (*n*-hexane/ethanol = 90/10, flow rate = 1.0 mL/min, λ = 254 nm, T = 20 °C, t_r (major) = 14.457 min, t_r (minor) = 12.551 min).

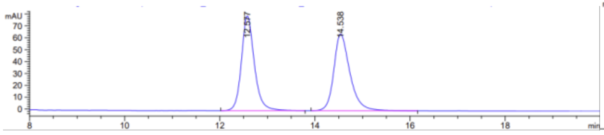

Signal 2: DAD1 B, Sig=254,4 Ref=off

Peak #	RetTime [min]	Type	Width [min]	Area [mAU*s]	Height [mAU]	Area %
1	12.577	BB	0.2849	1507.06653	79.59461	49.7659
2	14.538	BB	0.3532	1521.24744	64.48775	50.2341

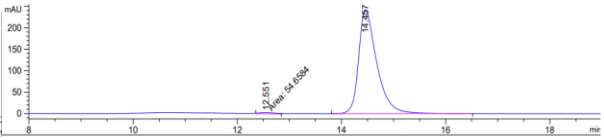

Signal 2: DAD1 B, Sig=254,4 Ref=off

Peak #	RetTime [min]	Type	Width [min]	Area [mAU*s]	Height [mAU]	Area %
1	12.551	MM	0.3577	54.65843	2.54680	0.9235
2	14.457	BB	0.3600	5863.90088	242.60065	99.0765

Supplementary Figure 98. HPLC spectra of compound **7i**

(*R*)-2-(5-Bromopyridin-2-yl)-2-(piperidin-1-yl)acetonitrile (7j):

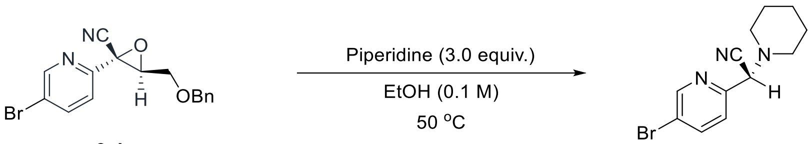

3ci
97% e.e.

7j
53%, 97% e.e.

In a 4 mL sample vial, **3ci** (34.0 mg, 0.1 mmol, 1.0 equiv.) was dissolved in ethanol (1.0 mL, 0.1 M) at room temperature. Piperidine (60.0 µL, 0.3 mmol, 3.0 equiv.) was then added, and the solution was stirred at 50 °C for 18 hours, with progress monitored by TLC. Upon completion, the solvent was evaporated under reduced pressure. The resulting residue was purified using column chromatography (petroleum ether/EtOAc = 20/1), yielding **7j** as a yellow oil (15.0 mg) in a 53% yield.

^{1}H NMR (600 MHz, $CDCl_3$) δ 8.68 (s, 1H), 7.86 (d, *J* = 8.4 Hz, 1H), 7.48 (d, *J* = 8.4 Hz, 1H), 4.86 (s, 1H), 2.64 – 2.44 (m, 4H), 1.64 (dq, *J* = 12.2, 6.5 Hz, 2H), 1.58 (dt, *J* = 12.2, 5.7 Hz, 2H), 1.51 – 1.46 (m, 2H).

^{13}C NMR (150 MHz, $CDCl_3$) δ 152.2, 150.7, 139.8, 123.8, 120.9, 114.8, 64.8, 51.4, 25.8, 23.8.

HRMS (ESI) *m/z* calcd. for $C_{12}H_{15}BrN_3$ $[M+H]^+$: 280.0444; found: 280.0456.

$[\alpha]_D^{20}$ = −25.8 (*c* 1.0, $CHCl_3$)

The enantiomeric excess was determined by chiral HPLC analysis using a Daicel Chiralpak IC column, e.e. = 97% (*n*-hexane/ethanol = 90/10, flow rate = 1.0 mL/min, λ = 254 nm, T = 20 °C, t_r (major) = 4.632 min, t_r (minor) = 5.379 min).

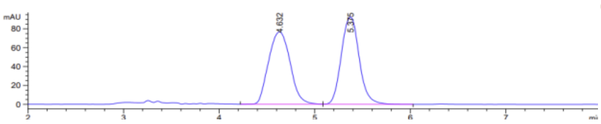

Signal 2: DAD1 B, Sig=254,4 Ref=off

Peak #	RetTime [min]	Type	Width [min]	Area [mAU*s]	Height [mAU]	Area %
1	4.632	BV	0.2628	1202.98792	76.78355	50.0452
2	5.375	VB	0.2033	1200.81396	92.56678	49.9548

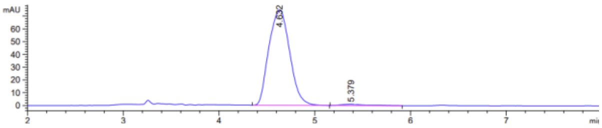

Signal 2: DAD1 B, Sig=254,4 Ref=off

Peak #	RetTime [min]	Type	Width [min]	Area [mAU*s]	Height [mAU]	Area %
1	4.632	BB	0.2480	1127.25220	74.72993	98.4981
2	5.379	BB	0.2354	17.18807	1.01597	1.5019

Supplementary Figure 99. HPLC spectra of compound **7j**

3. Supplementary Discussion

3.1 Mechanistic investigations

3.1.1 Control experiments

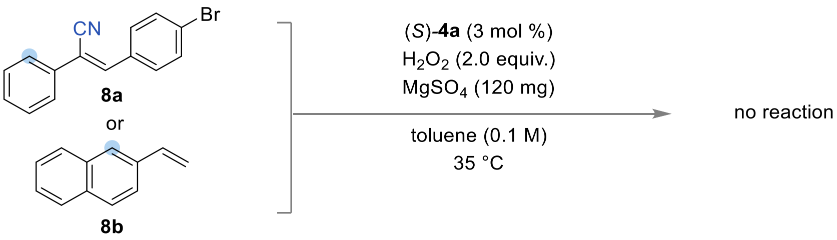

The substrates **8a**[27] and **8b**[28]were prepared following literature methods.

In a 4 mL vial equipped with a stirring bar, either substrate **8a**, **8b** (0.1 mmol, 1.0 equiv.) was added, along with (*S*)-**4a** (2 mg, 0.03 mmol, 0.03 equiv.) and $MgSO_4$ (120 mg). Freshly distilled toluene (1.0 mL) was then introduced into the vial. Subsequently, H_2O_2 (30% in H_2O *w/w*, 16 µL, 0.2 mmol, 2.0 equiv.) was added dropwise using microliter syringes. The reaction mixture was stirred at 35 °C. After 2 days, no desired product was detected in the reaction with substrates **8a**, **8b**.

3.1.2 ^{1}H NMR Study to investigate catalyst-substrate interactions in isomerization processes

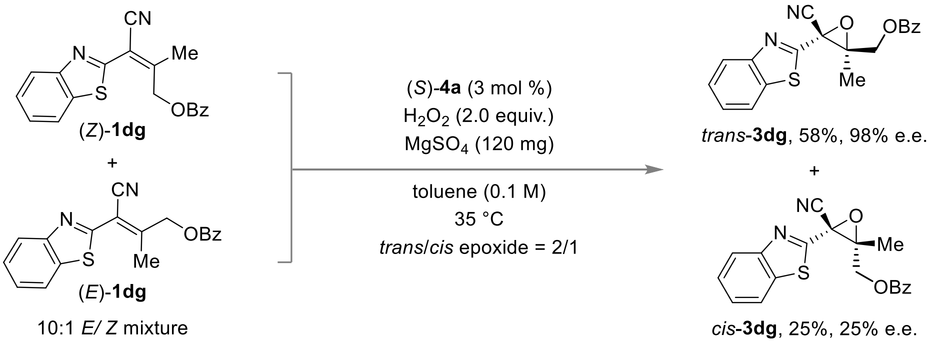

In a separate experiment, an *E/Z* mixture of **1dg** (0.1 mmol, 1.0 equiv.) was treated as follows: (*S*)-**4a** (2 mg, 0.03 mmol, 0.03 equiv.) and $MgSO_4$ (120 mg) were added to a 4 mL vial with a stirring bar. Freshly distilled toluene (1.0 mL) and H_2O_2 (30% in H_2O *w/w*, 16 µL, 0.2 mmol, 2.0 equiv.) were carefully added dropwise. The reaction mixture was stirred at 35 °C for 24 hours. Upon completion, the solution was filtered through a short celite pad, and the organic layer was concentrated under reduced pressure. The product was purified using flash column chromatography on silica gel (petroleum ether/EtOAc = 10/1), yielding *trans*-**3dg** as a colorless oil (20 mg) with a 58% yield, and *cis*-**3dg** as a colorless oil (8 mg) with a 25% yield.

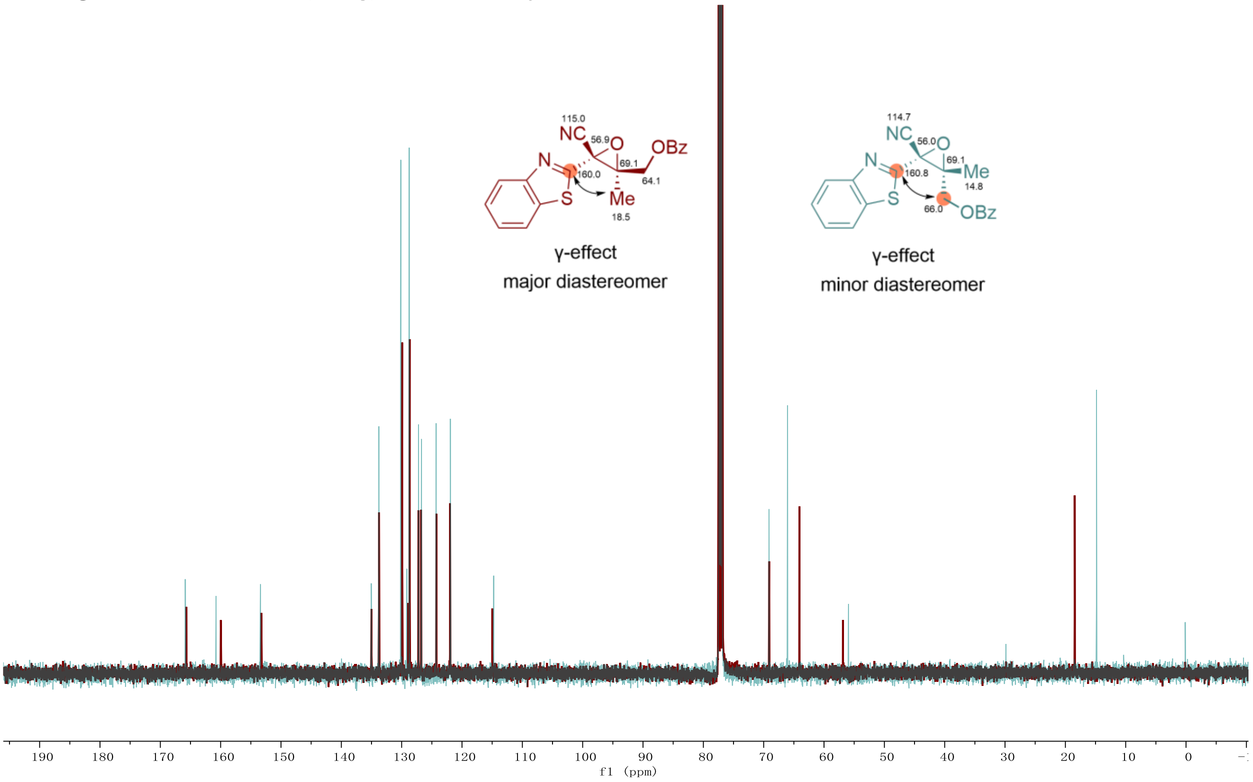

Supplementary Figure 100. ^{13}C NMR spectroscopy of *trans*-**3dg** and *cis*-**3dg**

An *E/Z* mixture (10/1) of **1dg** (0.1 mmol, 1.0 equiv.), (*S*)-**4a** (2 mg, 0.03 mmol), and $MgSO_4$ (120 mg) was placed in a 4 mL vial with a magnetic stir bar, and freshly distilled toluene (1.0 mL) was added. The mixture was stirred at 35 °C for 24 hours, followed by filtration through a short celite pad, concentration of the combined organic layer under reduced pressure. The ratio of resulted diastereoisomers was analyzed by ^{1}H NMR.

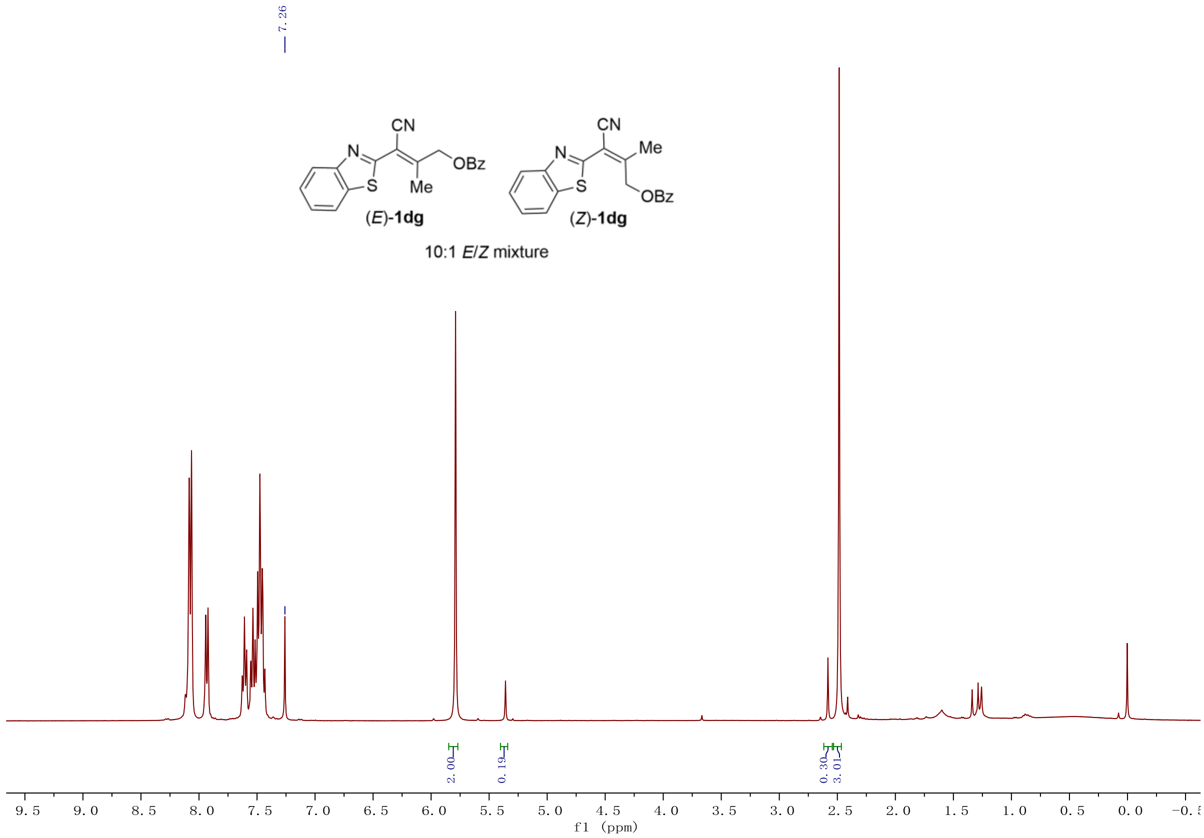

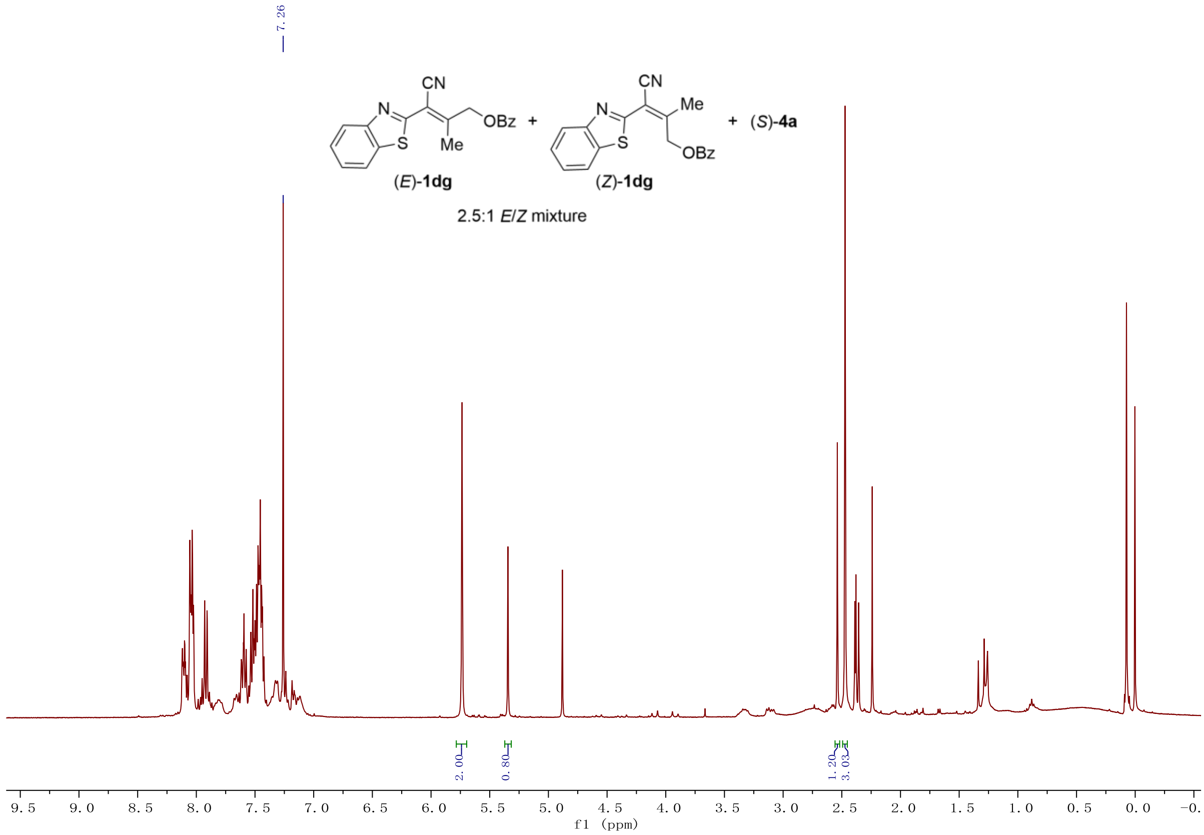

Supplementary Figure 101. Catalyst-substrate interactions in isomerization processes

3.1.3 Study of Nonlinear effects

In this segment of the study, a series of experiments were conducted to investigate the Nonlinear effects on the substrate by varying the ratios of two enantiomers, (*R*)-**4a** and (*S*)-**4a**.

Preparation of Enantiomer Mixtures:

Different mixtures of (*S*)-**4a** and (*R*)-**4a** were prepared in distinct ratios. Each enantiomer was initially dissolved in toluene solution (0.003 M) in separate small vials. Subsequently, these solutions were mixed in various volumes to create different profiles: Profile 1: (*S*)-**4a** 1.5 mL + (*R*)-**4a** 1.5 mL; Profile 2: (*S*)-**4a** 2.0 mL + (*R*)-**4a** 1.0 mL; Profile 3: (*S*)-**4a** 2.25 mL + (*R*)-**4a** 0.75 mL; Profile 4: (*S*)-**4a** 2.4 mL + (*R*)-**4a** 0.6 mL; Profile 5: (*S*)-**4a** 2.5 mL + (*R*)-**4a** 0.5 mL; Profile 6: (*S*)-**4a** 3.0 mL.

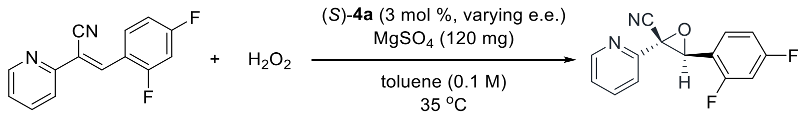

1bi 0.1 mmol; **2** 2.0 equiv.; **3bi**

In the experimental setup, a solution of the Compound **1bi** (0.1 mmol, 1.0 equiv.) and $MgSO_4$ (120 mg) was prepared in a 4 mL vial fitted with a stirring bar. To this solution, 1.0 mL of toluene solutions containing two enantiomers was added. The reaction was initiated by the gradual addition of H_2O_2 (30% in H_2O *w*/*w*, 16 µL, 0.2 mmol, 2.0 equiv.) using microliter syringes. The mixture was then stirred continuously at a temperature of 35 °C for a duration of 7 hours.

The enantiomeric excess of (*S*)-**4a** and (*R*)-**4a** was determined by chiral HPLC analysis using a Daicel Chiralpak IA column (*n*-hexane/ethanol = 70/30, flow rate = 1.0 mL/min, λ = 254 nm, T = 20 °C).

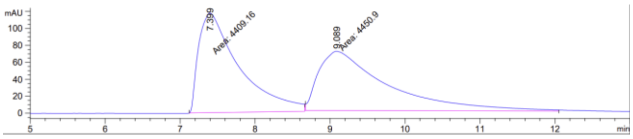

Signal 2: DAD1 B, Sig=254,4 Ref=off

Peak #	RetTime [min]	Type	Width [min]	Area [mAU*s]	Height [mAU]	Area %
1	7.399	MM	0.6251	4409.15820	117.56120	49.7644
2	9.089	MM	1.0618	4450.89941	69.86620	50.2356

Supplementary Figure 102. HPLC spectra of chiral phosphoric acid of profile 1

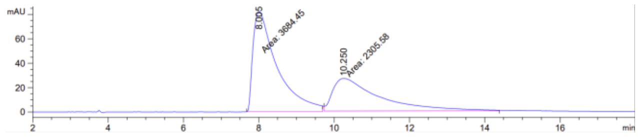

Signal 2: DAD1 B, Sig=254,4 Ref=off

Peak #	RetTime [min]	Type	Width [min]	Area [mAU*s]	Height [mAU]	Area %
1	8.005	MM	0.7470	3684.45117	82.20797	61.5097
2	10.250	MM	1.4394	2305.57886	26.69691	38.4903

Supplementary Figure 103. HPLC spectra of chiral phosphoric acid of profile 2

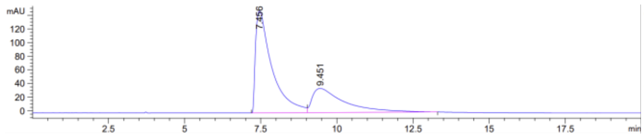

Signal 2: DAD1 B, Sig=254,4 Ref=off

Peak #	RetTime [min]	Type	Width [min]	Area [mAU*s]	Height [mAU]	Area %
1	7.456	BV	0.5347	5605.43994	149.08551	68.1779
2	9.451	VB	1.0176	2616.34058	35.34340	31.8221

Supplementary Figure 104. HPLC spectra of chiral phosphoric acid of profile 3

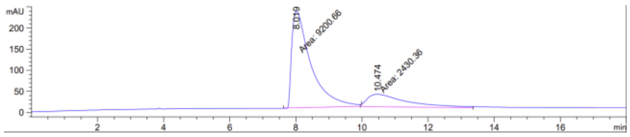

Signal 2: DAD1 B, Sig=254,4 Ref=off

Peak #	RetTime [min]	Type	Width [min]	Area [mAU*s]	Height [mAU]	Area %
1	8.019	MM	0.6734	9200.65527	227.73085	79.1045
2	10.474	MM	1.3763	2430.35596	29.43154	20.8955

Supplementary Figure 105. HPLC spectra of chiral phosphoric acid of profile 4

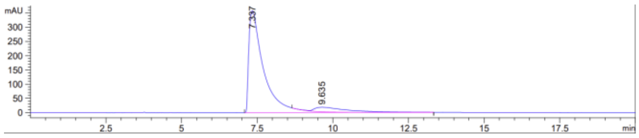

Signal 2: DAD1 B, Sig=254,4 Ref=off

Peak #	RetTime [min]	Type	Width [min]	Area [mAU*s]	Height [mAU]	Area %
1	7.337	BV R	0.4857	1.22092e4	358.18051	90.3631
2	9.635	VB E	1.0567	1302.07227	15.91113	9.6369

Supplementary Figure 106. HPLC spectra of chiral phosphoric acid of profile 5

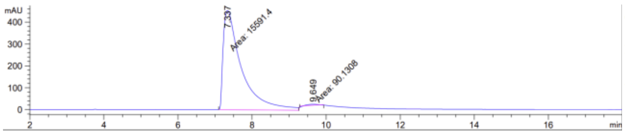

Signal 2: DAD1 B, Sig=254,4 Ref=off

Peak #	RetTime [min]	Type	Width [min]	Area [mAU*s]	Height [mAU]	Area %
1	7.337	MM	0.5723	1.55914e4	454.07150	99.4252
2	9.649	MM	0.2718	90.13083	4.39688	0.5748

Supplementary Figure 107. HPLC spectra of chiral phosphoric acid of profile 6

The enantiomeric excess of **3bi** was determined by chiral HPLC analysis using a Daicel Chiralpak IA column (*n*-hexane/ethanol = 90/10, flow rate = 1.0 mL/min, λ = 230 nm, T = 20 ^{o}C).

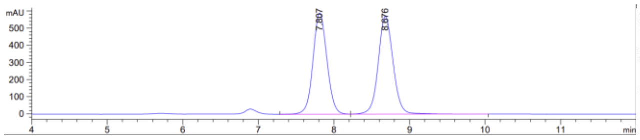

Signal 4: DAD1 D, Sig=230,4 Ref=off

Peak #	RetTime [min]	Type	Width [min]	Area [mAU*s]	Height [mAU]	Area %
1	7.807	BV	0.1994	7571.11572	591.13660	49.1355
2	8.676	VB	0.2123	7837.54053	570.57031	50.8645

Supplementary Figure 108. HPLC spectra of **3bi** of profile 1

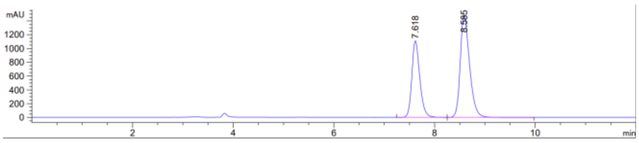

Signal 4: DAD1 D, Sig=230,4 Ref=off

Peak #	RetTime [min]	Type	Width [min]	Area [mAU*s]	Height [mAU]	Area %
1	7.618	BB	0.1662	1.21878e4	1108.90869	39.0066
2	8.585	BB	0.1935	1.90577e4	1487.62122	60.9934

Supplementary Figure 109. HPLC spectra of **3bi** of profile 2

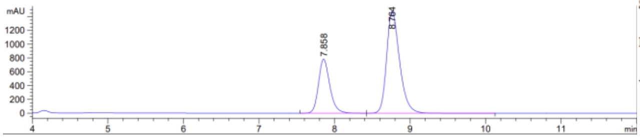

Signal 4: DAD1 D, Sig=230,4 Ref=off

Peak #	RetTime [min]	Type	Width [min]	Area [mAU*s]	Height [mAU]	Area %
1	7.858	BB	0.1593	8166.39258	785.23047	31.5693
2	8.764	BB	0.1839	1.77018e4	1476.39099	68.4307

Supplementary Figure 110. HPLC spectra of **3bi** of profile 3

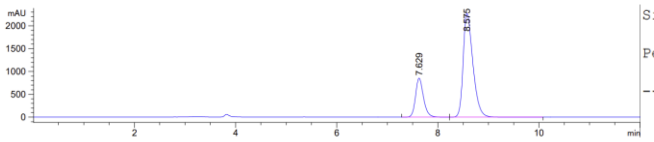


```
Signal 4: DAD1 D, Sig=230,4 Ref=off

Peak RetTime Type  Width     Area      Height     Area
  #   [min]        [min]   [mAU*s]     [mAU]        %
----|-------|----|-------|----------|----------|--------|
   1   7.629 BB    0.1679 9350.25293  852.99445  23.4841
   2   8.575 BB    0.2045 3.04649e4  2270.96582  76.5159
```

Supplementary Figure 111. HPLC spectra of **3bi** of profile 4

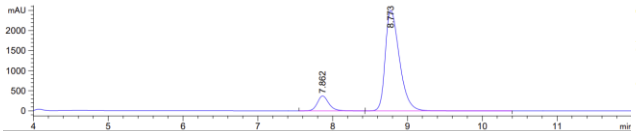


```
Signal 4: DAD1 D, Sig=230,4 Ref=off

Peak RetTime Type  Width     Area      Height     Area
  #   [min]        [min]   [mAU*s]     [mAU]        %
----|-------|----|-------|----------|----------|--------|
   1   7.862 BB    0.1609 3893.97705  369.71210  10.5929
   2   8.773 BB    0.2012 3.28661e4  2503.13184  89.4071
```

Supplementary Figure 112. HPLC spectra of **3bi** of profile 5

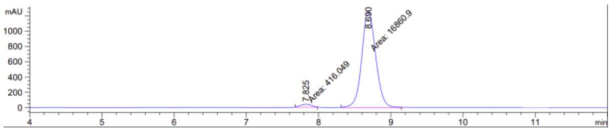


```
Signal 4: DAD1 D, Sig=230,4 Ref=off

Peak RetTime Type  Width     Area      Height     Area
  #   [min]        [min]   [mAU*s]     [mAU]        %
----|-------|----|-------|----------|----------|--------|
   1   7.825 MM    0.1755  416.04941   39.50227   2.4081
   2   8.690 MM    0.2236 1.68609e4  1256.88928  97.5919
```

Supplementary Figure 113. HPLC spectra of **3bi** of profile 6

Supplementary Table 12. Outcomes of Nonlinear effect experiments with varying e.e. of 3 mol % (*S*)-**4a**

entry	e.e. (%) of (*S*)-**4a**	e.e. (%) of product **3bi**
Profile 1	0	0
Profile 2	23	22
Profile 3	36	37
Profile 4	58	53
Profile 5	80	79
Profile 6	99	95

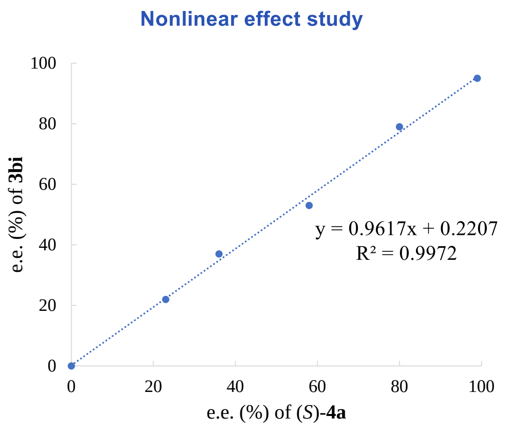

Supplementary Figure 114. Nonlinear effect curve for 3 mol % (*S*)-**4a** with varying enantiometric excess.

3.1.4 Reaction progress kinetic analysis

3.1.4.1 Calibration curves

The mechanistic study focused on the reaction between alkenyl aza-heteroarenes and hydrogen peroxide, forming epoxide with (*S*)-**4a** as the catalyst and *n*-dodecane as an internal standard. The elaboration of the calibration curves were established by preparing stock solutions of *n*-dodecane and substrate **1bi** in toluene, followed by systematic dilutions. These solutions were then analyzed using GC-FID. The GC conditions included an HP-5MS UI column (30 m × 250 μm × 0.25 μm), an injection temperature of 250 °C, and a temperature program starting at 50 °C (held for 2 min), ramping up to 300 °C at 15 °C/min, and maintaining 300 °C for 5 min. Retention times were recorded as 9.5 min for *n*-dodecane and 16.5 min for **1bi**.

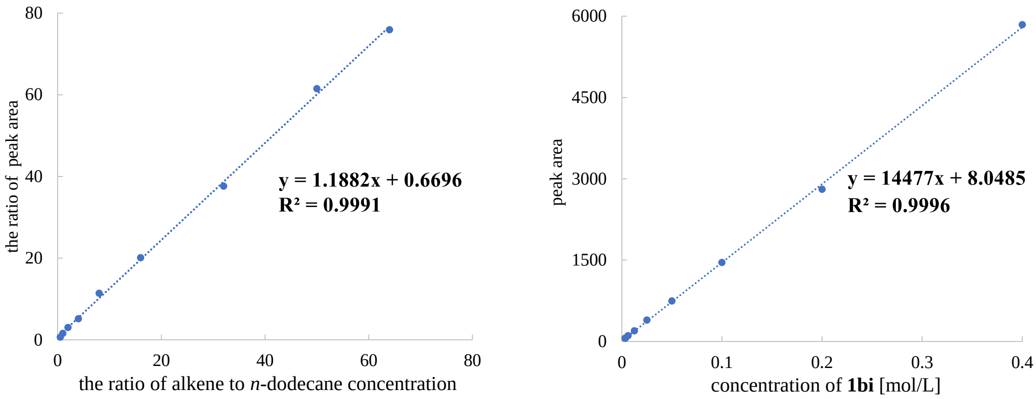

Supplementary Figure 115. Calibration curves for GC-FID analysis of standard solutions of *n*-dodecane and **1bi**

3.1.4.2 Determination of the order of the catalyst

Based on Bures' method[29], the reaction was determined to be first order dependence with respect to the catalyst concentration. The model reaction was performed with varying concentrations of the catalyst (*S*)-**4a** (3, 4, and 5 mol %) under otherwise identical conditions.

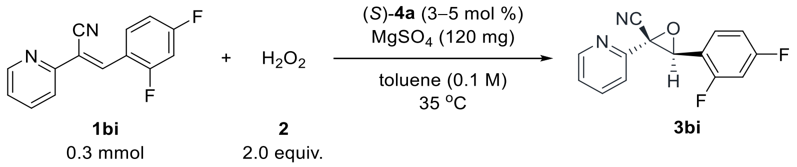

To ascertain the reaction profiles, a solution of **1bi** (0.3 mmol, 1.0 equiv.), (*S*)-**4a** in varying amounts (profile 1: 6.0 mg, 3 mol %; profile 2: 8.0 mg, 4 mol %; profile 3: 10.0 mg, 5 mol %), *n*-dodecane (5 μL, 0.03 mmol, 0.1 equiv.), and $MgSO_4$ (360 mg) was prepared in a 10 mL vial with a stirring bar. Freshly distilled toluene (1.0 mL) was added to this mixture. Subsequently, H_2O_2 (30% in H_2O *w*/*w*, 48 μL, 0.6 mmol, 2.0 equiv.) was added dropwise using microliter syringes. The reaction was stirred

at 35 °C, and sampling for GC-FID analysis commenced, involving dilution with 1 mol % Et_3N in EtOAc.

Supplementary Table 13. GC peak area ratios and calculated concentrations of *n*-dodecane and **1bi** for Profile **1**

t/min	peak area (**1bi**)/peak area (*n*-dodecane)	[**1bi**] (mmol/L)
0	0.807011	86.470387
30	0.851509	65.318381
60	0.990050	37.079136
90	1.072109	29.519807
120	1.183406	23.125455
150	1.370230	16.959025
180	1.471577	14.815889
210	1.635741	12.298416
240	1.886836	9.761459
270	2.127932	8.147662
300	2.332223	7.146541
330	2.637686	6.037336
360	3.020909	5.053356
390	3.332756	4.461624
420	3.800768	3.794750
450	4.205622	3.360273
480	4.652911	2.982945
510	5.038647	2.719586
540	5.451613	2.484728
570	5.657790	2.382027
600	5.915629	2.264951
630	7.688097	1.692955
660	8.259259	1.565551
690	8.523810	1.512819
720	13.064698	0.958605

Supplementary Table 14. GC peak area ratios and calculated concentrations of *n*-dodecane and **1bi** for Profile **2**

t/min	peak area (**1bi**)/peak area (*n*-dodecane)	[**1bi**] (mmol/L)
0	0.830161	74.00300192
30	0.910220	49.380833
60	1.097315	27.78015239
90	1.269117	19.8192775
120	1.515723	14.04287107
150	1.811358	10.40675974
180	2.215434	7.686465272
210	2.611412	6.119026775
240	3.103406	4.882065722
270	3.543389	4.134610605

300	4.411255	3.175599753
330	5.038647	2.719586003
360	5.983240	2.236131823
390	6.855460	1.920832491
420	8.090909	1.601065237
450	9.504202	1.34493896
480	10.750881	1.178620021
510	12.333333	1.018713276
540	13.771049	0.906922603
570	14.847861	0.838043564
600	16.667845	0.742706488
630	19.080321	0.645384818
660	22.255814	0.55044391
690	23.570025	0.518855009
720	26.247956	0.464533366

Supplementary Table 15. GC peak area ratios and calculated concentrations of *n*-dodecane and substrate **1bi** for Profile **3**

t/min	peak area (**1bi**)/peak area (*n*-dodecane)	[**1bi**] (mmol/L)
0	0.78986934	98.79492184
30	0.851166235	65.44168313
60	1.04248366	31.86516673
90	1.31000231	18.55396181
120	1.477086946	14.71478897
150	2.234152652	7.594503122
180	2.497726478	6.499550302
210	2.894080997	5.34147067
240	3.750593824	3.856547815
270	4.470459519	3.126135008
300	5.329113924	2.550051399
330	6.788161994	1.941959567
360	8.310986965	1.554953316
390	9.214504597	1.390536297
420	11.03369434	1.146458109
450	13.04494382	0.96013494
480	13.94768311	0.894858083
510	14.38461538	0.866349739
540	17.11594203	0.722470686
570	18.45525292	0.668066562
600	20.59827214	0.596226378
630	24.51020408	0.498393411
660	24.64102564	0.495673481
690	29.58103976	0.410979187
720	33.60207612	0.360798865

Supplementary Table 16. Calculated times multiplied by concentrations of (*S*)-**4a** for Profiles **1**, **2**, and **3**

t/min•[(*S*)-**4a**] (Profile **1**)	*t*/min•[(*S*)-**4a**] (Profile **2**)	*t*/min•[(*S*)-**4a**] (Profile **3**)
0	0	0
0.09	0.12	0.15
0.18	0.24	0.3
0.27	0.36	0.45
0.36	0.48	0.6
0.45	0.6	0.75
0.54	0.72	0.9
0.63	0.84	1.05
0.72	0.96	1.2
0.81	1.08	1.35
0.9	1.2	1.5
0.99	1.32	1.65
1.08	1.44	1.8
1.17	1.56	1.95
1.26	1.68	2.1
1.35	1.8	2.25
1.44	1.92	2.4
1.53	2.04	2.55
1.62	2.16	2.7
1.71	2.28	2.85
1.8	2.4	3
1.89	2.52	3.15
1.98	2.64	3.3
2.07	2.76	3.45
2.16	2.88	3.6

Supplementary Table 17. Calculated times multiplied by concentrations of (*S*)-**4a** for Profiles **1**, **2**, and **3**, including squared values.

t/min•[(*S*)-**4a**]^2 (Profile **1**)	*t*/min•[(*S*)-**4a**]^2 (Profile **2**)	*t*/min•[(*S*)-**4a**]^2 (Profile **3**)
0	0	0
0.00027	0.00048	0.00075
0.00054	0.00096	0.00150
0.00081	0.00144	0.00225
0.00108	0.00192	0.00300
0.00135	0.00240	0.00375
0.00162	0.00288	0.00450
0.00189	0.00336	0.00525
0.00216	0.00384	0.00600
0.00243	0.00432	0.00675
0.00270	0.00480	0.00750
0.00297	0.00528	0.00825
0.00324	0.00576	0.00900

0.00351	0.00624	0.00975
0.00378	0.00672	0.01050
0.00405	0.00720	0.01125
0.00432	0.00768	0.01200
0.00459	0.00816	0.01275
0.00486	0.00864	0.01350
0.00513	0.00912	0.01425
0.00540	0.00960	0.01500
0.00567	0.01008	0.01575
0.00594	0.01056	0.01650
0.00621	0.01104	0.01725
0.00648	0.01152	0.01800

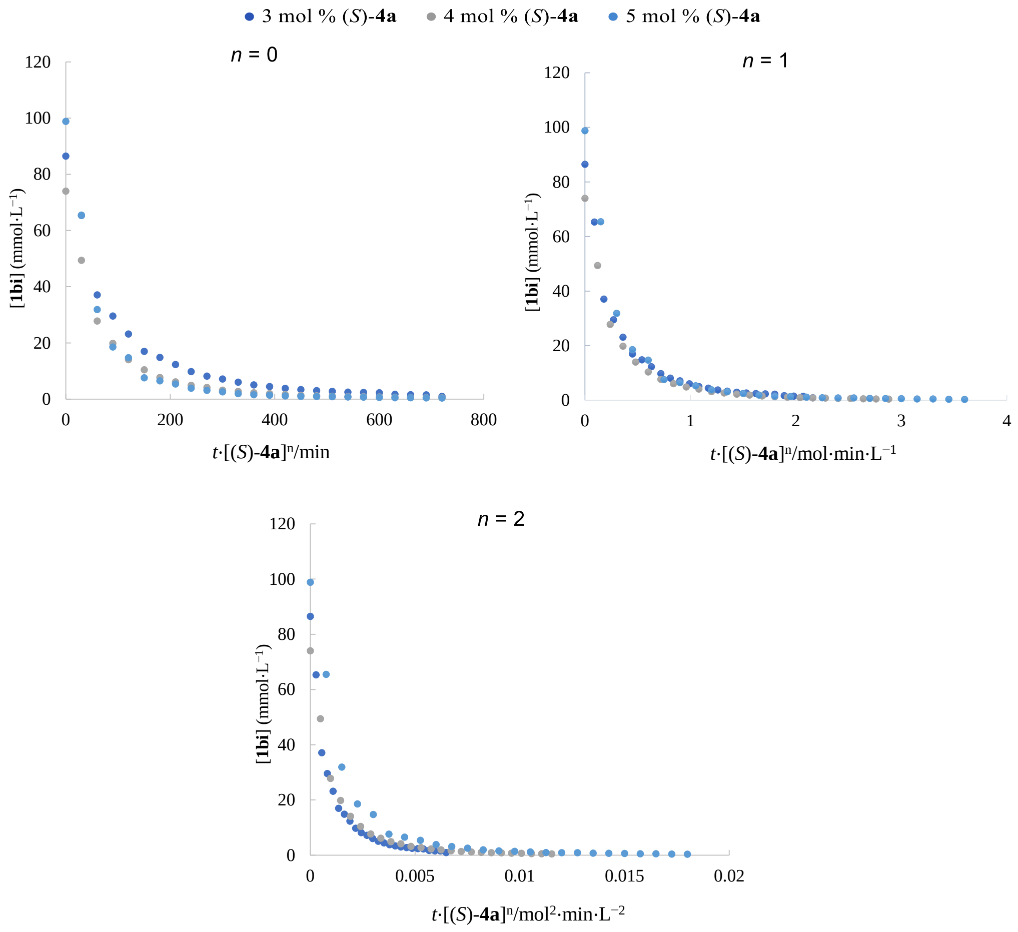

Supplementary Figure 116. The method of Burés revealed a first-order dependence on the catalyst concentration

3.2 Computational studies

All calculations were performed using Gaussian 16, Revision A.03 package[30]. All of the intermediates were optimized by the DFT with the B3LYP-D3(BJ) functional[31,32]. For geometry optimizations and frequency calculations, BS-I basis set system was employed. In BS-I, we employed LANL2DZ basis set for Br with effective core potentials, 6-31G(d) basis sets for H, C, O, N, and P. All the stationary

structures were characterized with no imaginary frequency and the transition state structures (TSs) were characterized with a single imaginary frequency. Intrinsic reaction coordinate (IRC) calculations were performed on the TSs. The solvent effect of toluene was evaluated through the SMD method[33], in which a better basis system BS-II was used. we employed SDD basis set for Br with effective core potentials, 6-311++G(d,p) basis sets for H, C, O, N, and P. All reported energies are free energies at a concentration of 1 M and a temperature of 298.15 K. All the 3D molecular structures of the species were generated by using the CYLview program[34]. The Non-Covalent Interactions (NCI) plot was prepared by Multiwfn 3.8[35].

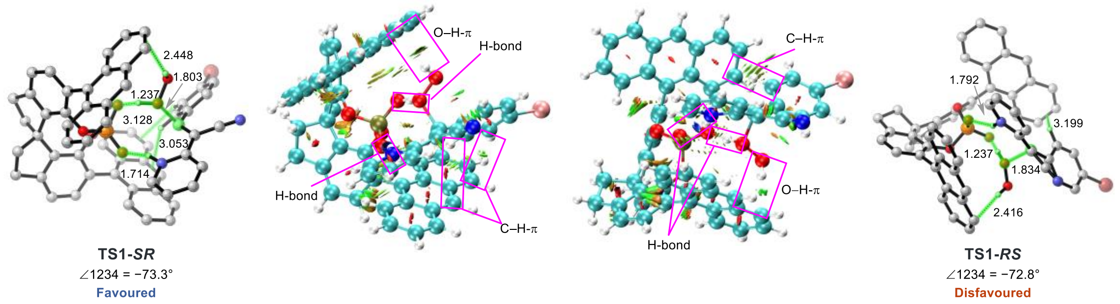

Supplementary Figure 117. The non-covalent interactions (NCI) plot for the calculated transition states leading to different enantioselectivity.

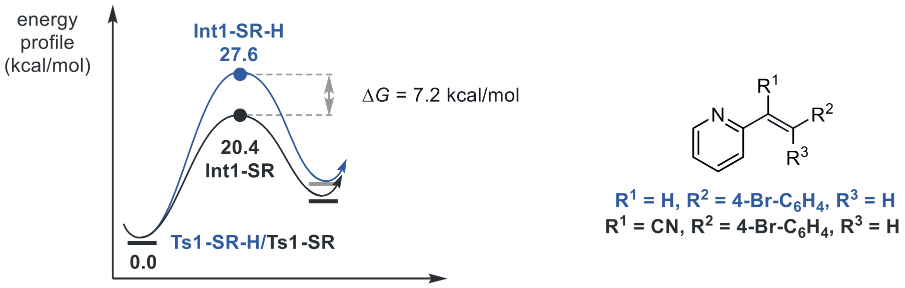

Supplementary Figure 118. DFT calculations focusing on the model **1aa** and its non-cyano variant to explore the impact of substituents on the reaction's energy barrier.

4. Supplementary Data

4.1 X-Ray crystallographic data

4.1.1 X-Ray crystallographic data of Compound 1ah

For the crystallographic analysis of Compound **1ah**, the Compound was first dissolved in ethyl acetate (EtOAc). To this solution, cold *n*-hexane was added, and the mixture was left for slow evaporation overnight, resulting in the formation of colorless needle-like crystals. A suitably sized crystal, measuring 0.48 mm × 0.13 mm × 0.11 mm, was carefully selected for X-ray diffraction analysis.

The crystallographic examination was carried out on a Bruker D8 Venture diffractometer, utilizing Mo radiation (l = 0.71073 Å). The data collection and cell determination were performed at a temperature of 296 K. The structural analysis was conducted using Olex2[36], where the crystal structure of Compound **1ah** was solved with the ShelXT[37] structure solution program applying Intrinsic Phasing. Further refinement was executed using the ShelX[38] refinement package through Least Squares minimization. The crystallographically solved structure of Compound **1ah** has been duly deposited at The Cambridge Crystallographic Data Centre, with the assigned CCDC number 2087974.

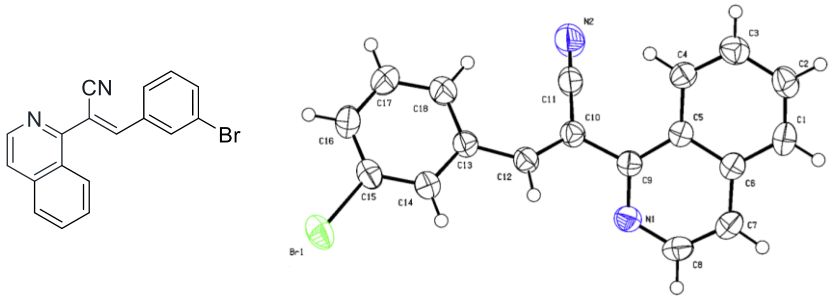

Supplementary Figure 119. ORTEP diagram of Compound **1ah**, showcasing the ellipsoids at the 50% contour probability level.

Crystal Data of 1ah

Chemical formula	$C_{18}H_{11}BrN_2$
Formula weight	335.20
Temperature	296 K
Wavelength	0.71073 Å
Crystal size	0.48 mm × 0.13 mm × 0.11 mm
Crystal habit	colourless needle
Crystal system	monoclinic
Space group	P 21/c
Unit cell dimensions	a = 13.3009(9) alpha = 90
	b = 3.9947 (3) beta = 92.934(3)
	c = 26.5269 (18) gamma = 90
Volum	1407.61 (17)
Z	4

4.1.2 X-Ray crystallographic data of Compound 3ah

For the crystallographic analysis of Compound **3ah**, the Compound was first dissolved in ethyl acetate (EtOAc). To this solution, cold *n*-hexane was added, and the mixture was left for slow evaporation overnight, resulting in the formation of colorless needle-like crystals. A suitably sized crystal, measuring 0.47 mm × 0.15 mm × 0.13 mm, was carefully selected for X-ray diffraction analysis.

The crystallographic examination was carried out on a Bruker D8 Venture diffractometer, utilizing Mo radiation (l = 0.71073 Å). The data collection and cell determination were performed at a temperature of 296 K. The structural analysis was conducted using Olex2[36], where the crystal structure of Compound **3ah** was solved with the ShelXT[37] structure solution

program applying Intrinsic Phasing. Further refinement was executed using the ShelX[38] refinement package through Least Squares minimization. The crystallographically solved structure of Compound **3ah** has been duly deposited at The Cambridge Crystallographic Data Centre, with the assigned CCDC number 2087973.

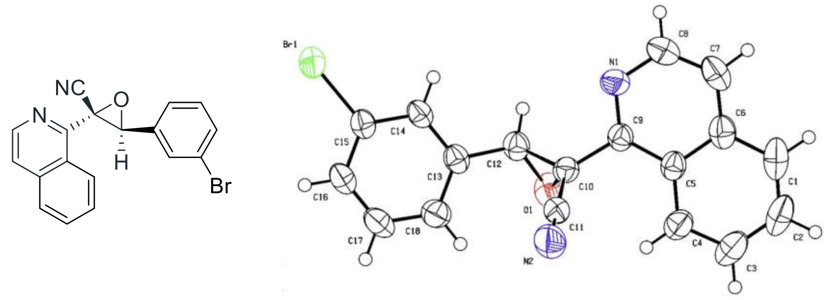

Supplementary Figure 120. ORTEP diagram of Compound **3ah**, showcasing the ellipsoids at the 50% contour probability level.

Crystal Data of 3ah

Chemical formula	$C_{18}H_{11}BrN_2O$
Formula weight	351.20
Temperature	296 K
Wavelength	0.71073 Å
Crystal size	0.47 mm × 0.15 mm × 0.13 mm
Crystal habit	colourless needle
Crystal system	monoclinic
Space group	P 1 21 1
Unit cell dimensions	a = 4.3119 (2) alpha = 90
	b = 9.5441 (4) beta = 91.496 (2)
	c = 18.3641 (8) gamma = 90
Volum	755.48 (6)
Z	2

4.1.3 X-Ray Crystallographic Data of Compound 6b

For the crystallographic analysis of Compound **6b**, the Compound was first dissolved in ethyl acetate (EtOAc). To this solution, cold *n*-hexane was added, and the mixture was left for slow evaporation overnight, resulting in the formation of colorless needle-like crystals. A suitably

sized crystal, measuring 0.06 mm × 0.05 mm × 0.06 mm, was carefully selected for X-ray diffraction analysis.

The crystallographic examination was carried out on a Bruker D8 Venture diffractometer, utilizing Mo radiation (l = 1.34139 Å). The data collection and cell determination were performed at a temperature of 250 K. The structural analysis was conducted using Olex2[36], where the crystal structure of Compound **6b** was solved with the ShelXT[37] structure solution program applying Intrinsic Phasing. Further refinement was executed using the ShelX[38] refinement package through Least Squares minimization. The crystallographically solved structure of Compound **6b** has been duly deposited at The Cambridge Crystallographic Data Centre, with the assigned CCDC number 2330838.

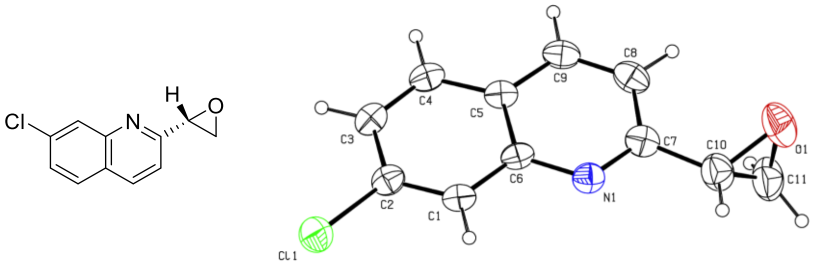

Supplementary Figure 121. ORTEP diagram of Compound **6b**, showcasing the ellipsoids at the 50% contour probability level.

Crystal Data of 6b

Chemical formula	$C_{11}H_8ClNO$
Formula weight	205.63
Temperature	250 K
Wavelength	1.34139 Å
Crystal size	0.06 mm × 0.05 mm × 0.06 mm
Crystal habit	colourless lamella
Crystal system	orthorhombic
Space group	P 1 21 1
Unit cell dimensions	a = 6.0275 (6) alpha = 90
	b = 14.3752 (16) beta = 100.088 (6)
	c = 11.0801 (12) gamma = 90
Volum	945.21 (18)
Z	4

4.1.4 X-Ray Crystallographic Data of Compound 7d

For the crystallographic analysis of Compound **7d**, the Compound was first dissolved in ethyl acetate (EtOAc). To this solution, cold *n*-hexane was added, and the mixture was left for slow evaporation overnight, resulting in the formation of colorless needle-like crystals. A suitably sized crystal, measuring 0.40 mm × 0.08 mm × 0.06 mm, was carefully selected for X-ray diffraction analysis.

The crystallographic examination was carried out on a Bruker D8 Venture diffractometer, utilizing Mo radiation (l = 0.71073 Å). The data collection and cell determination were performed at a temperature of 170 K. The structural analysis was conducted using Olex2[36], where the crystal structure of Compound **7d** was solved with the ShelXT[37] structure solution program applying Intrinsic Phasing. Further refinement was executed using the ShelX[38] refinement package through Least Squares minimization. The crystallographically solved structure of Compound **7d** has been duly deposited at The Cambridge Crystallographic Data Centre, with the assigned CCDC number 2299941.

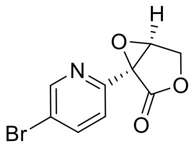

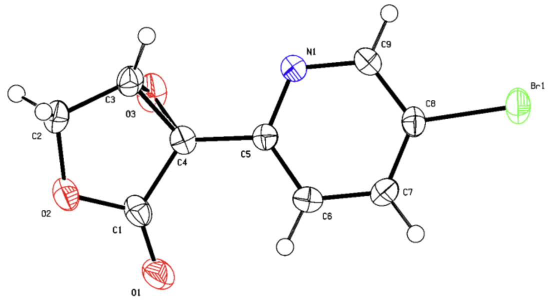

Supplementary Figure 122. ORTEP diagram of Compound **7d**, showcasing the ellipsoids at the 50% contour probability level.

Crystal Data of 7d

Chemical formula	$C_9H_6BrNO_3$
Formula weight	256.06
Temperature	170 K
Wavelength	0.71073 Å
Crystal size	0.40 mm × 0.08 mm × 0.06 mm
Crystal habit	colourless needle
Crystal system	orthorhombic
Space group	P 21 21 21
Unit cell dimensions	a = 4.1535 (1) alpha = 90

	b = 10.1419 (3) beta = 90
	c = 21.4969 (6) gamma = 90
Volum	905.54 (4)
Z	4

4.2. NMR spectroscopy for characterization

Supplementary Figure 123. ^{1}H NMR spectrum of compound **1ac** (600 MHz, $CDCl_3$)

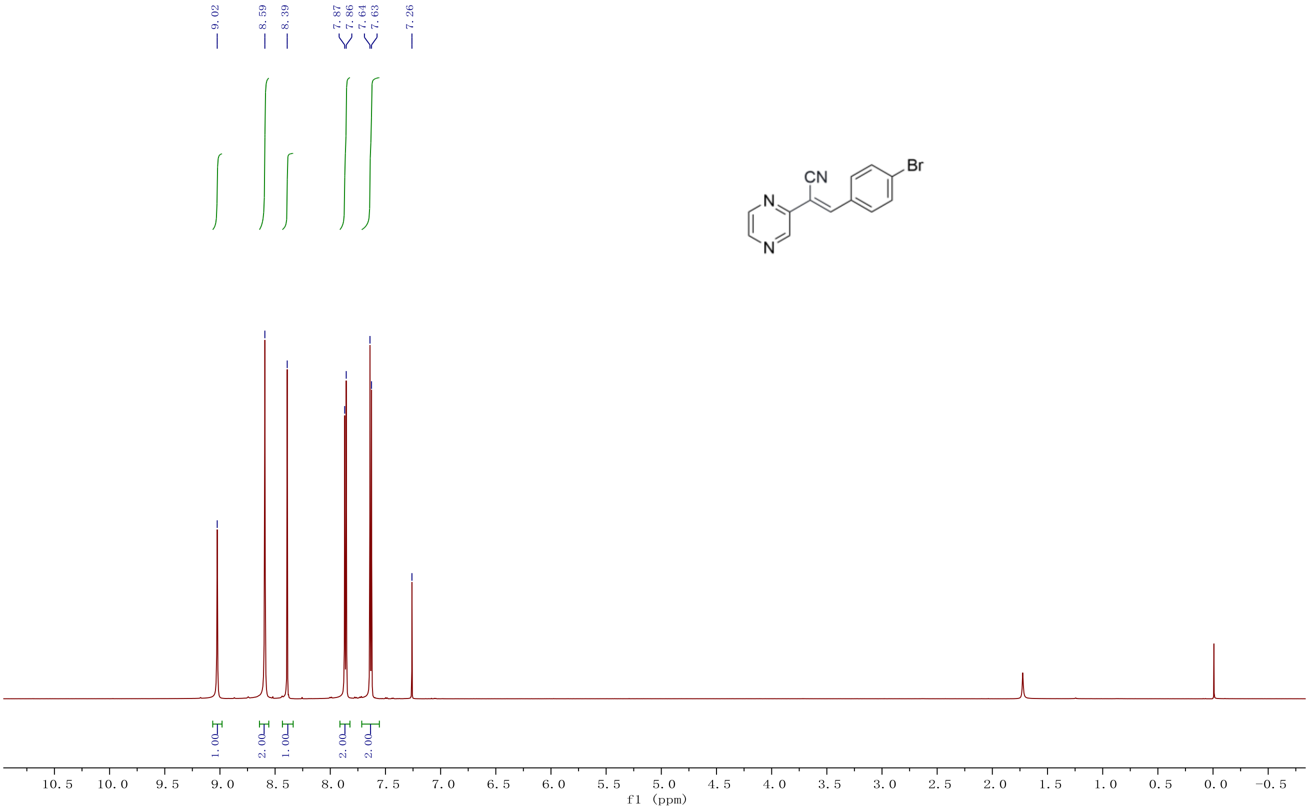

Supplementary Figure 124. ^{13}C NMR spectrum of compound **1ac** (150 MHz, $CDCl_3$)

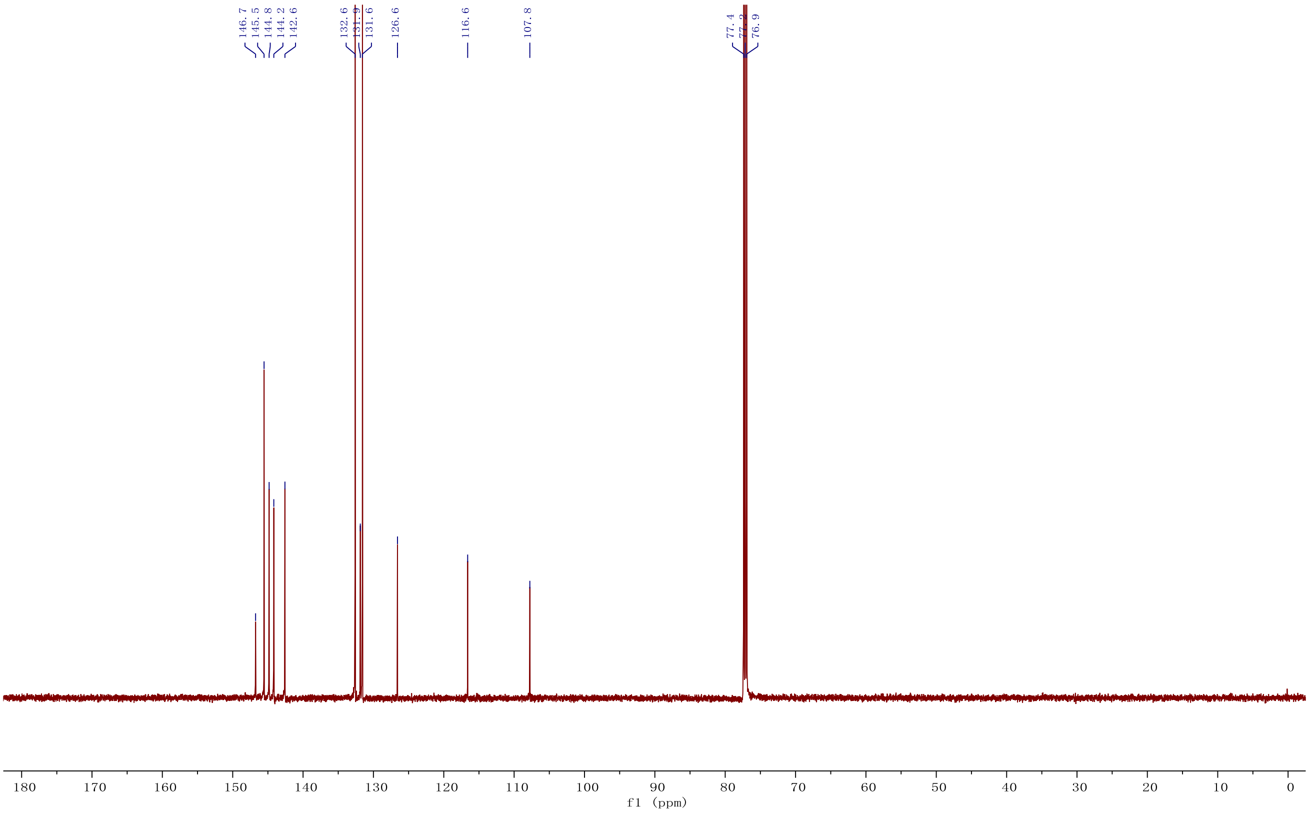

Supplementary Figure 125. ^{1}H NMR spectrum of compound **1ad** (600 MHz, $CDCl_3$)

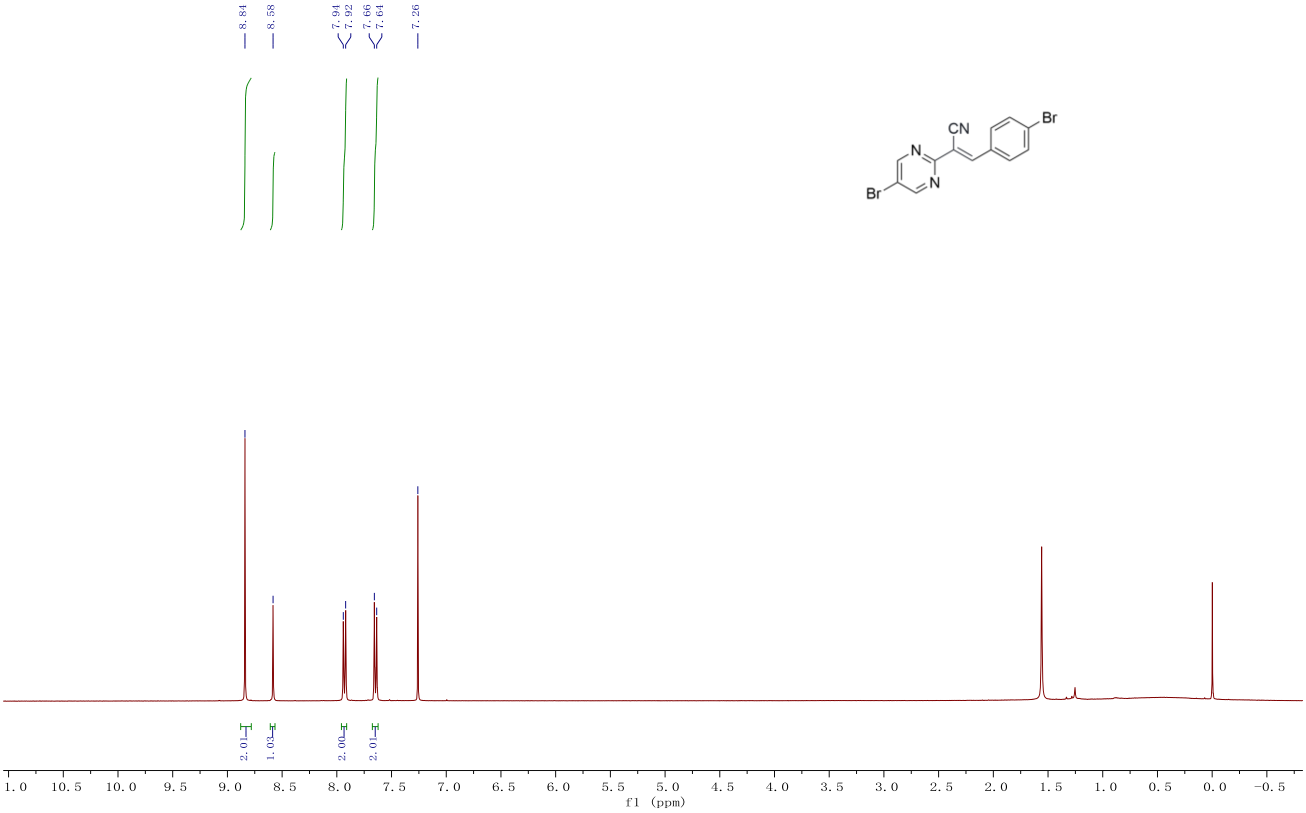

Supplementary Figure 126. ^{13}C NMR spectrum of compound **1ad** (150 MHz, $CDCl_3$)

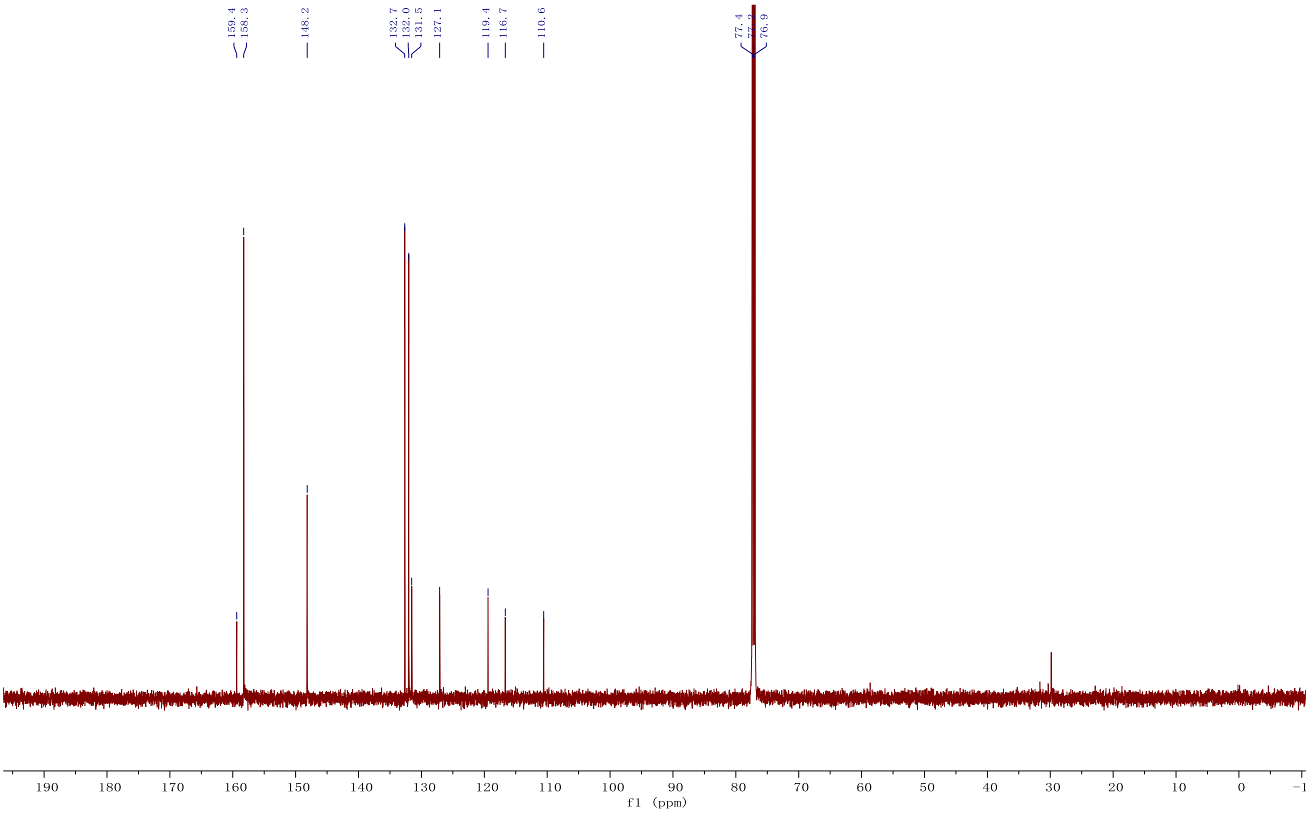

Supplementary Figure 127. ^{1}H NMR spectrum of compound **1ae** (400 MHz, DMSO-d_6)

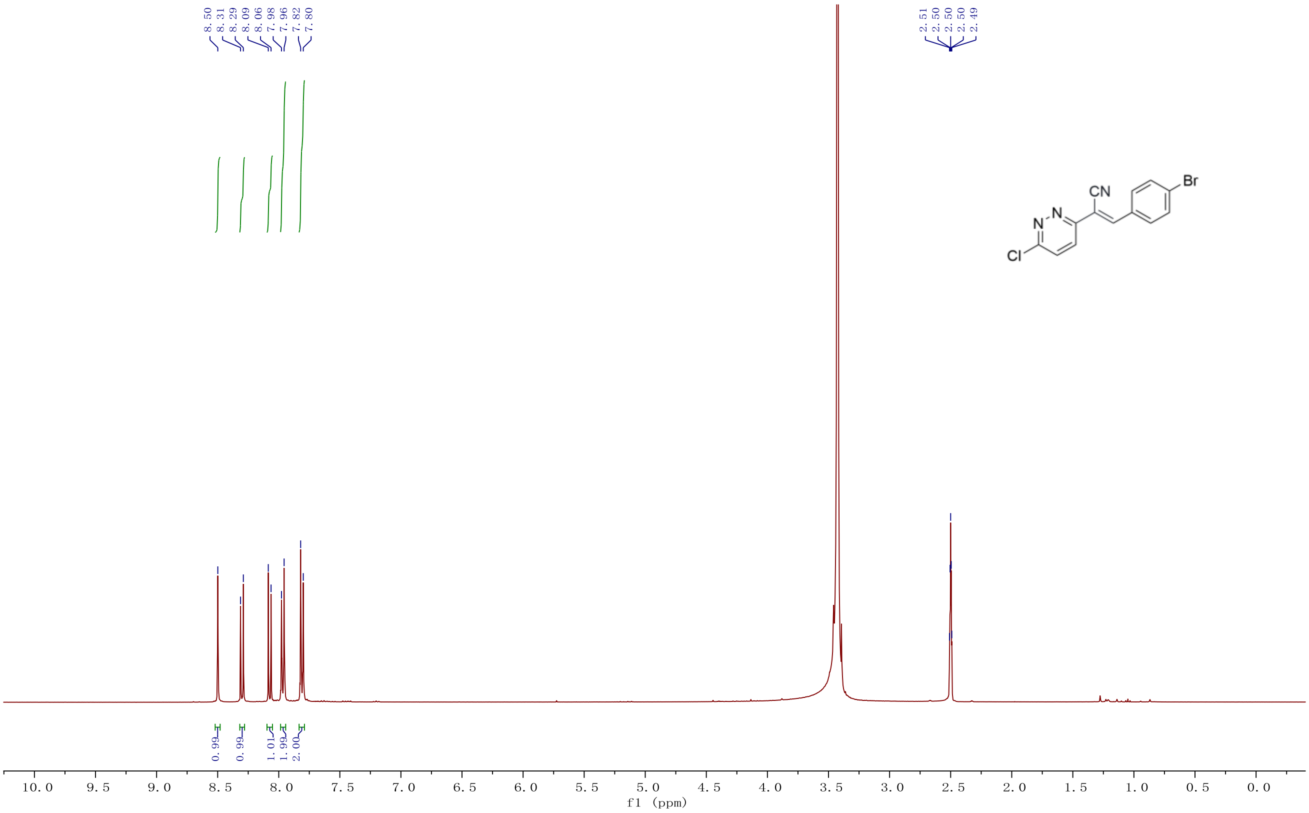

Supplementary Figure 128. ^{13}C NMR spectrum of compound **1ae** (100 MHz, DMSO-d_6)

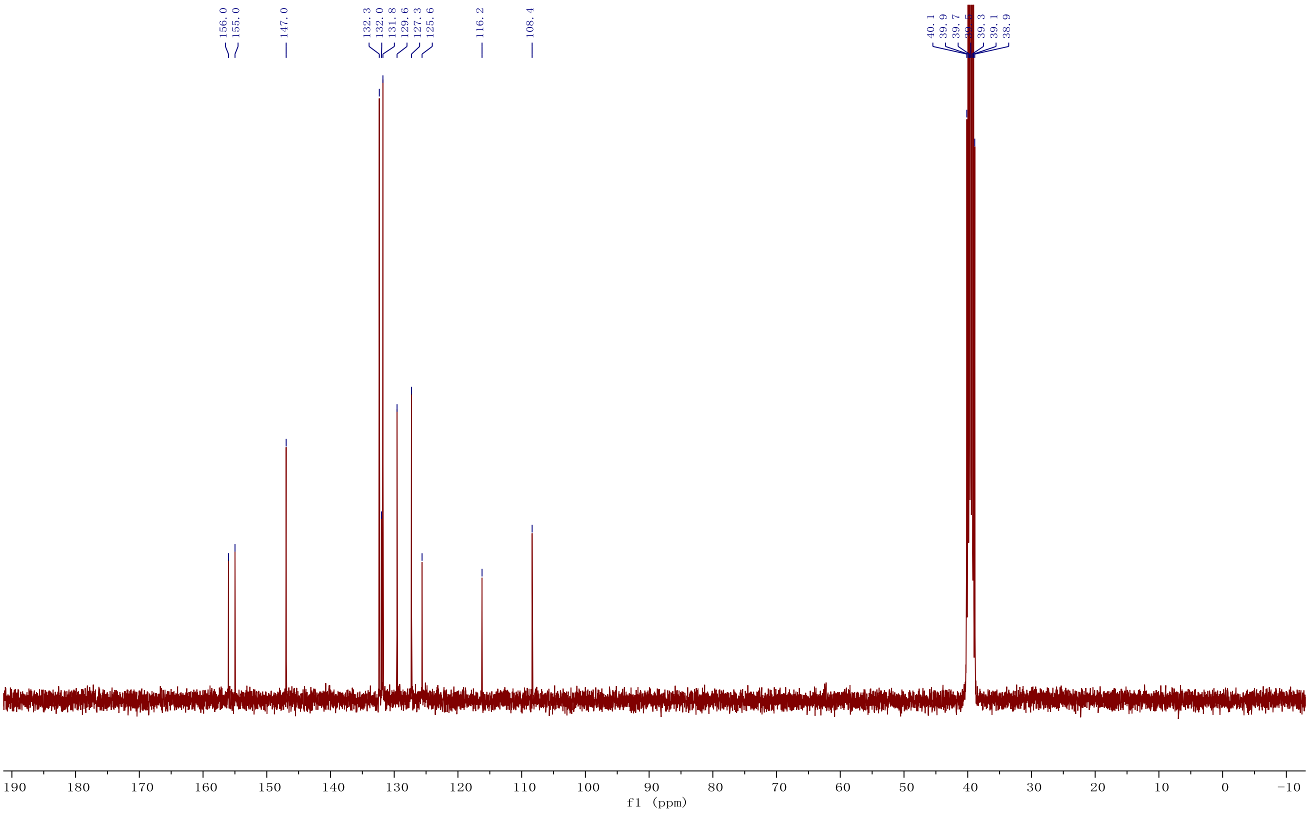

Supplementary Figure 129. ^{1}H NMR spectrum of compound **1af** (400 MHz, $CDCl_3$)

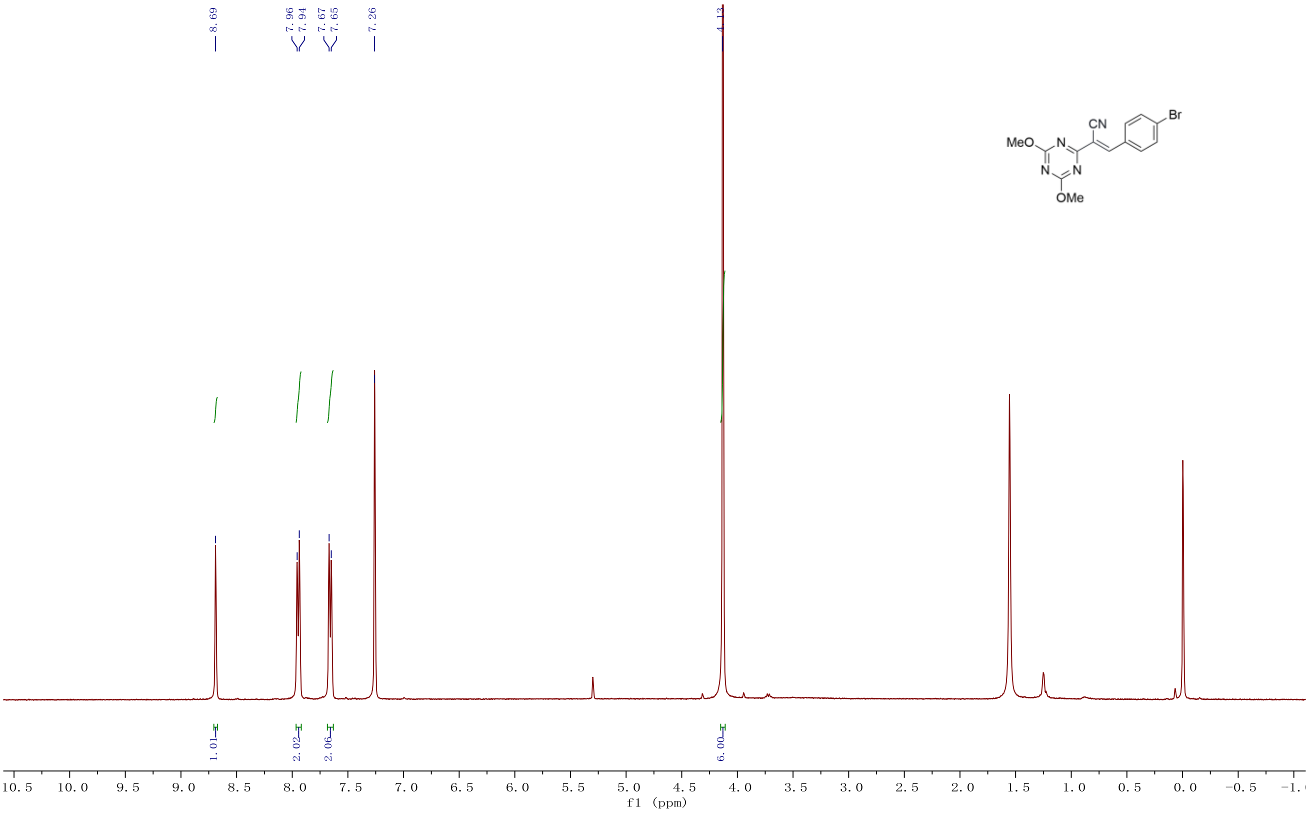

Supplementary Figure 130. ^{13}C NMR spectrum of compound **1af** (100 MHz, $CDCl_3$)

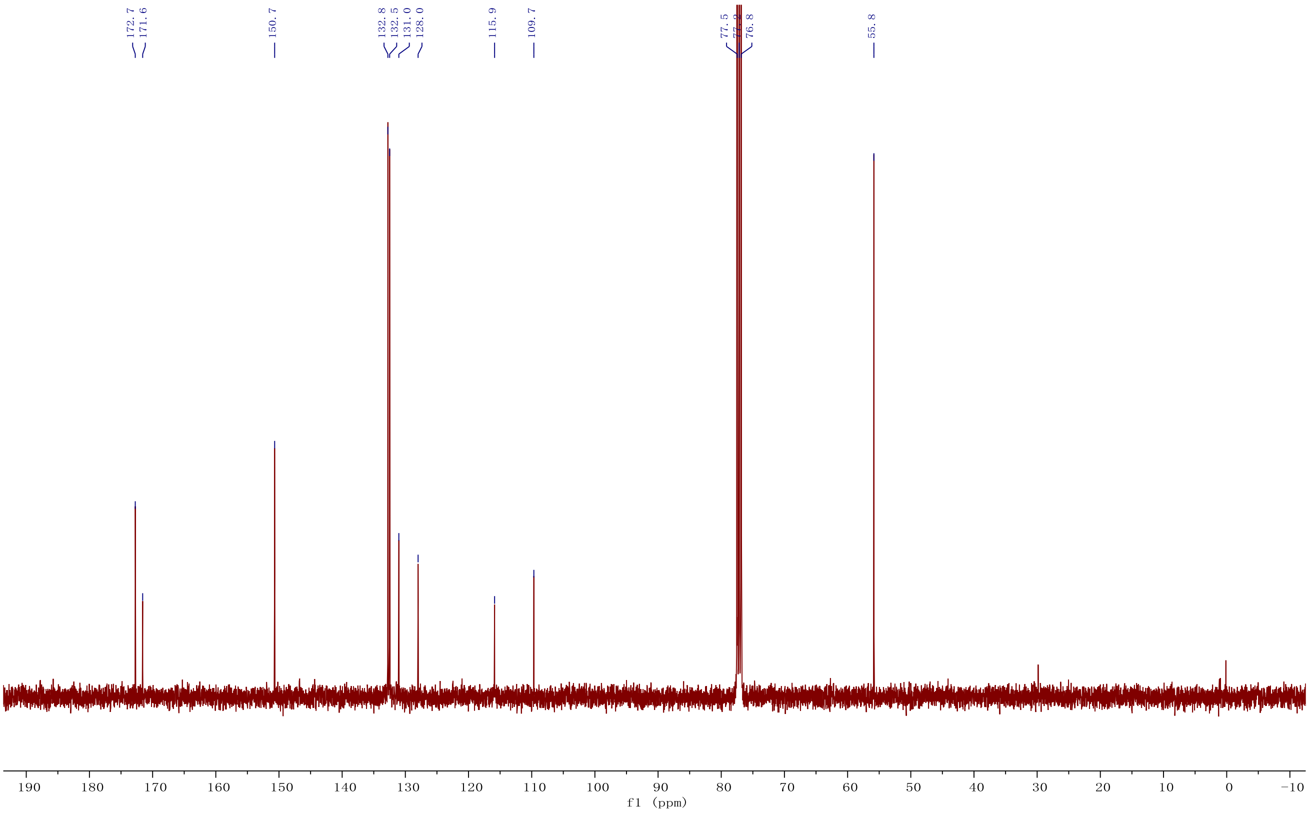

Supplementary Figure 131. ^{1}H NMR spectrum of compound **1ag** (600 MHz, $CDCl_3$)

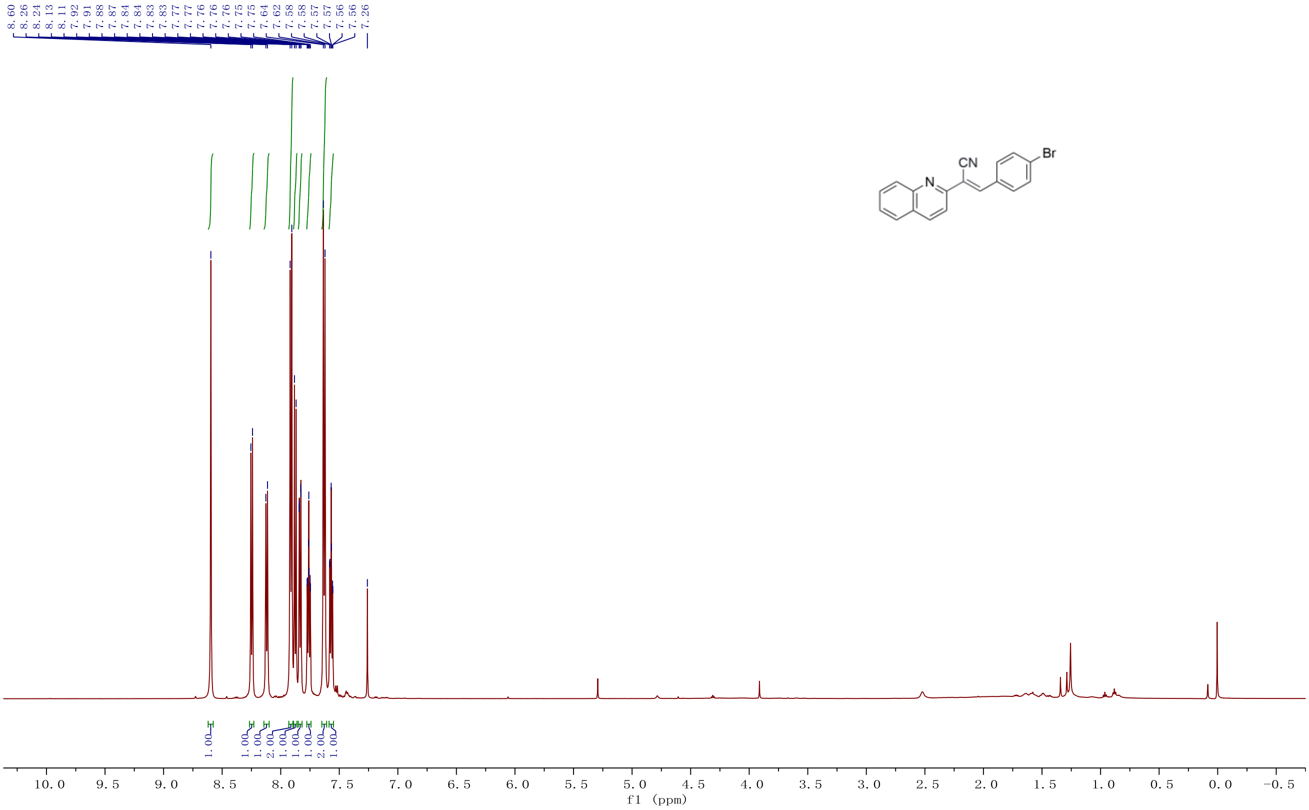

Supplementary Figure 132. ^{13}C NMR spectrum of compound **1ag** (150 MHz, $CDCl_3$)

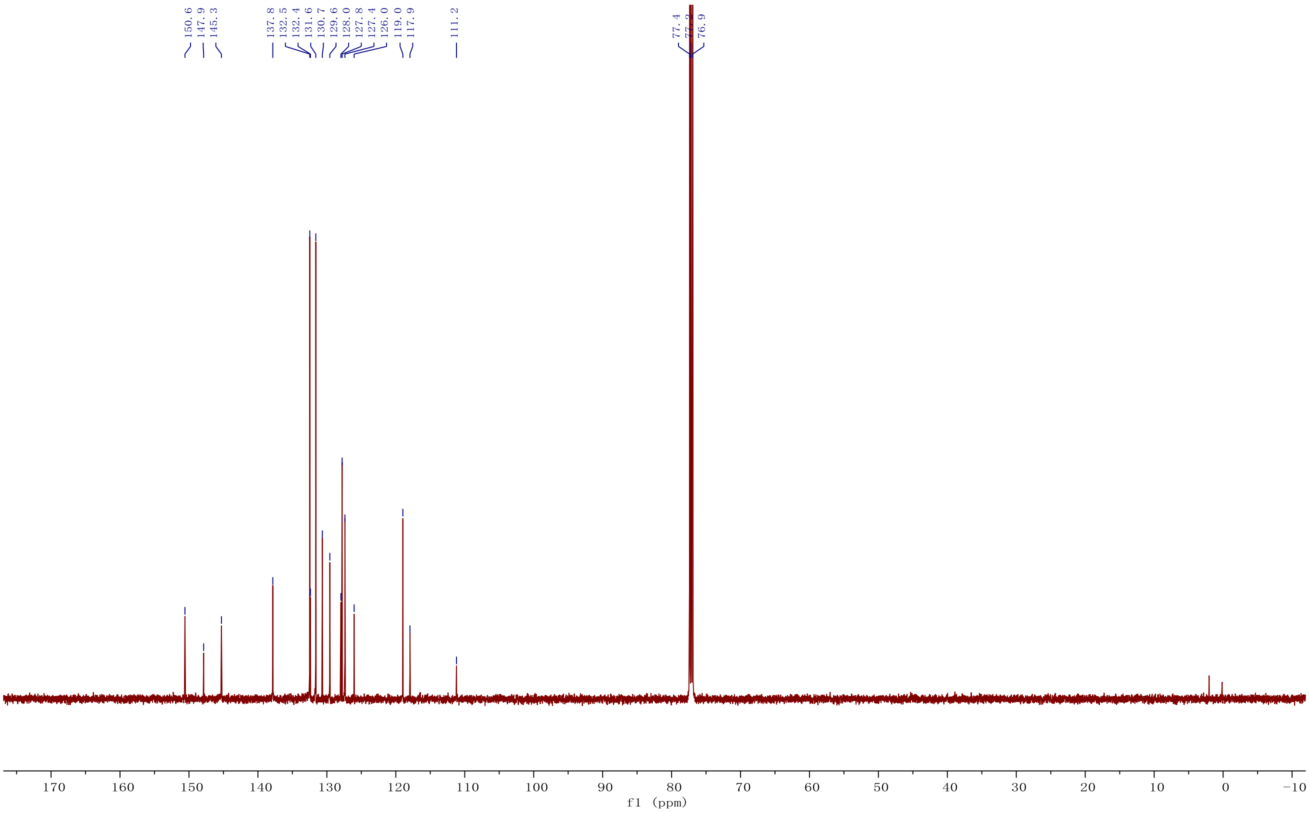

Supplementary Figure 133. ^{1}H NMR spectrum of compound **1ah** (400 MHz, $CDCl_3$)

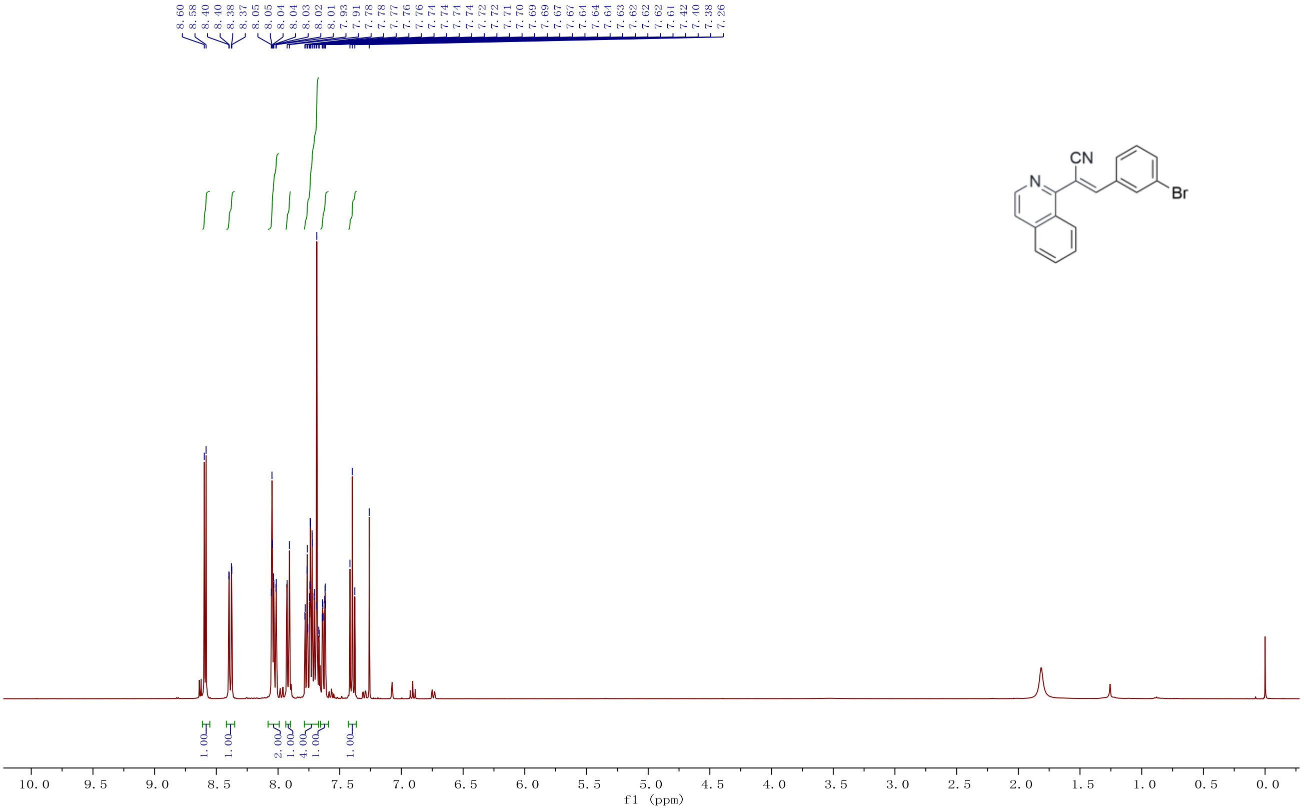

Supplementary Figure 134. ^{13}C NMR spectrum of compound **1ah** (100 MHz, $CDCl_3$)

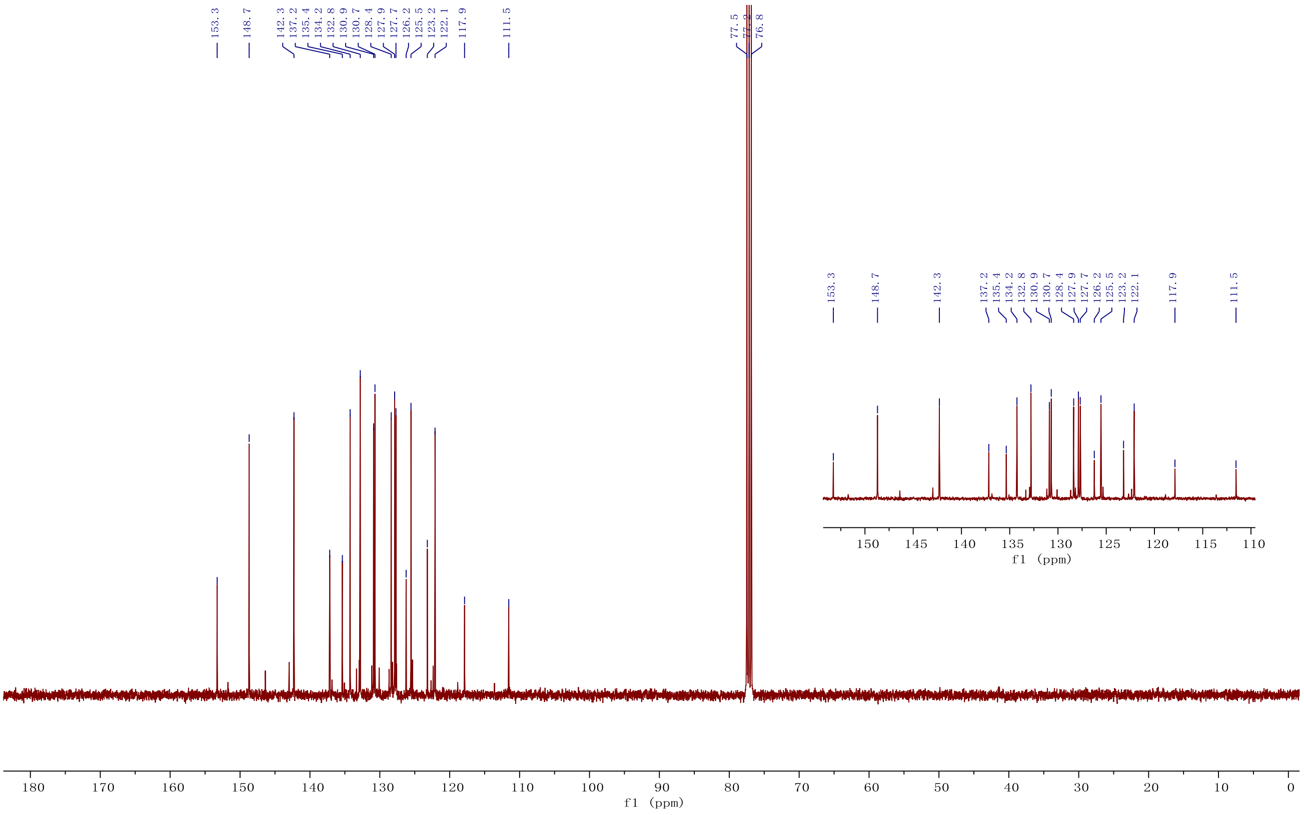

Supplementary Figure 135. ^{1}H NMR spectrum of compound **1ai** (600 MHz, $CDCl_3$)

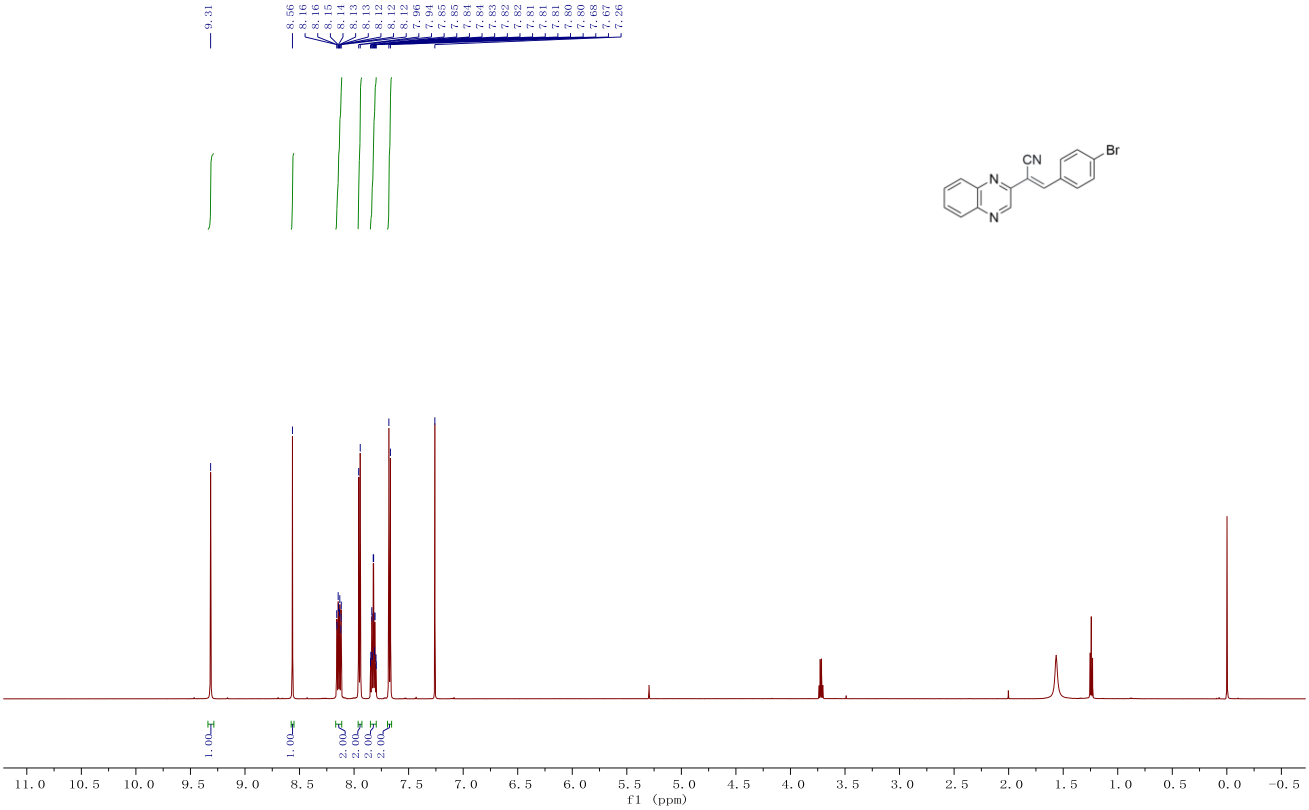

Supplementary Figure 136. ^{13}C NMR spectrum of compound **1ai** (150 MHz, $CDCl_3$)

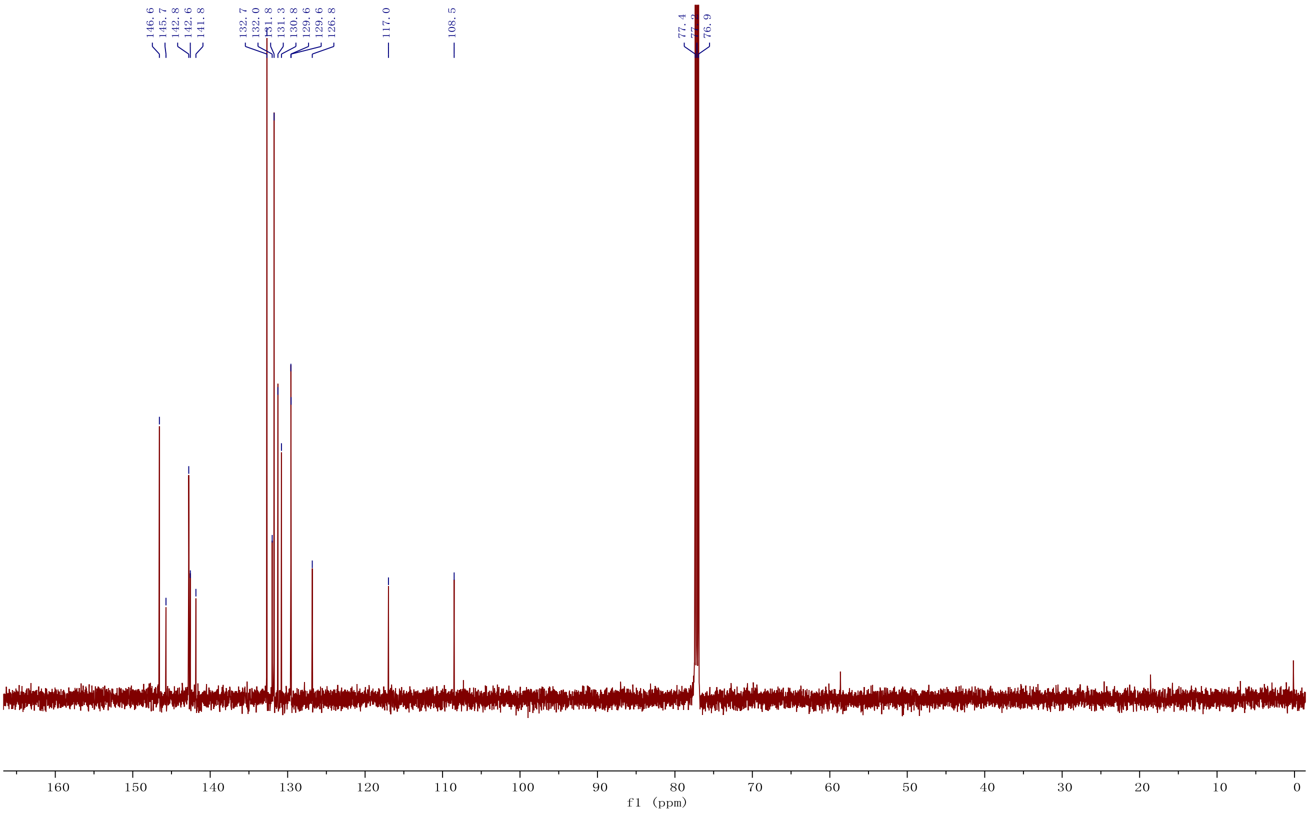

Supplementary Figure 137. ^{1}H NMR spectrum of compound **1aj** (400 MHz, $CDCl_3$)

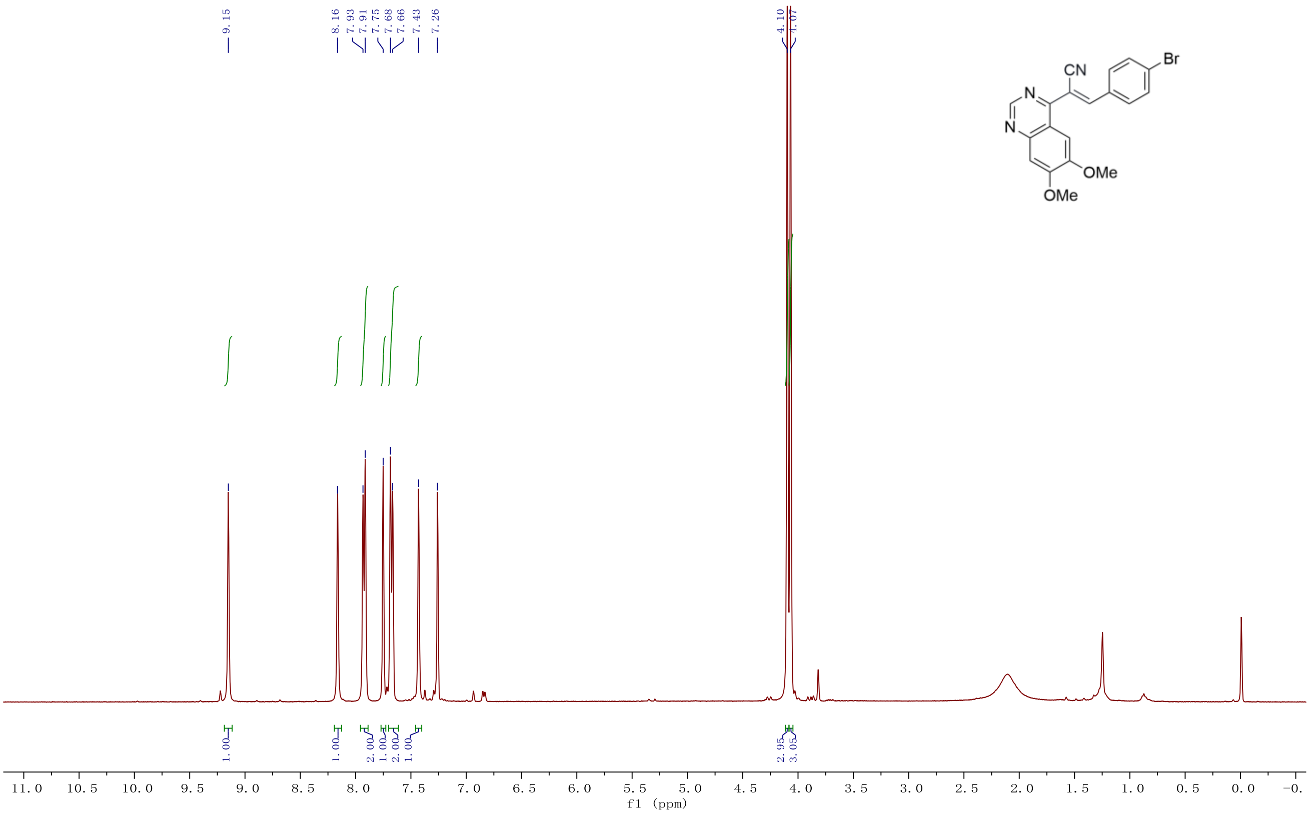

Supplementary Figure 138. ^{13}C NMR spectrum of compound **1aj** (100 MHz, $CDCl_3$)

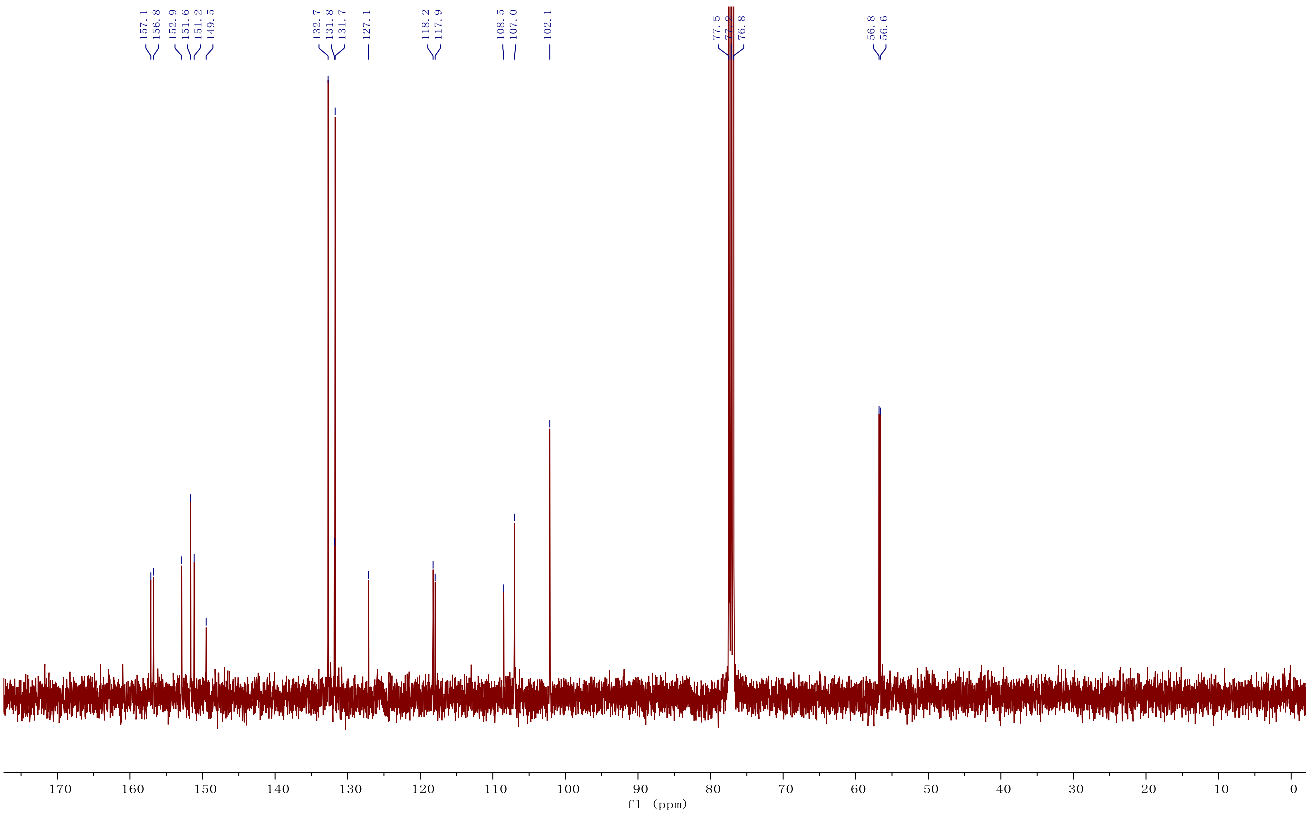

Supplementary Figure 139. ^{1}H NMR spectrum of compound **1ak** (400 MHz, $CDCl_3$)

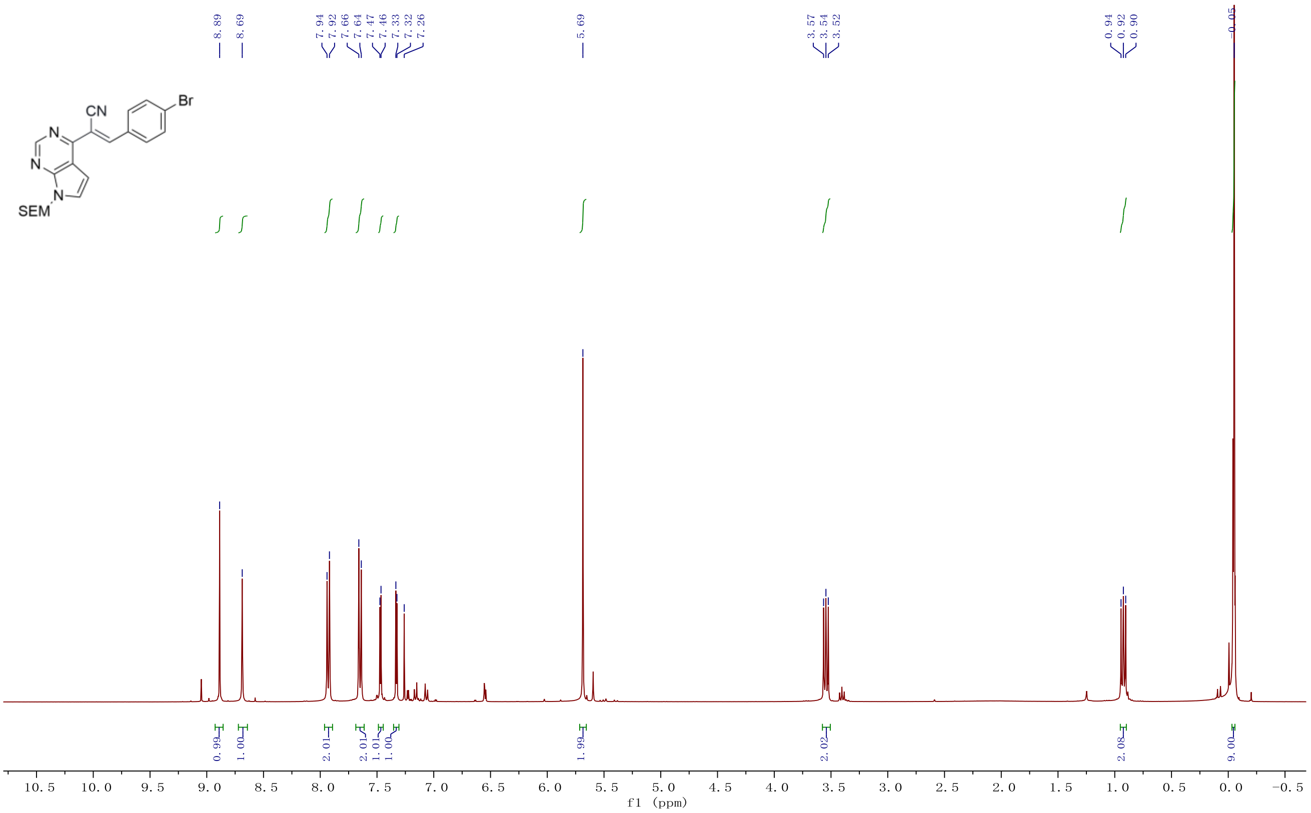

Supplementary Figure 140. ^{13}C NMR spectrum of compound **1ak** (100 MHz, $CDCl_3$)

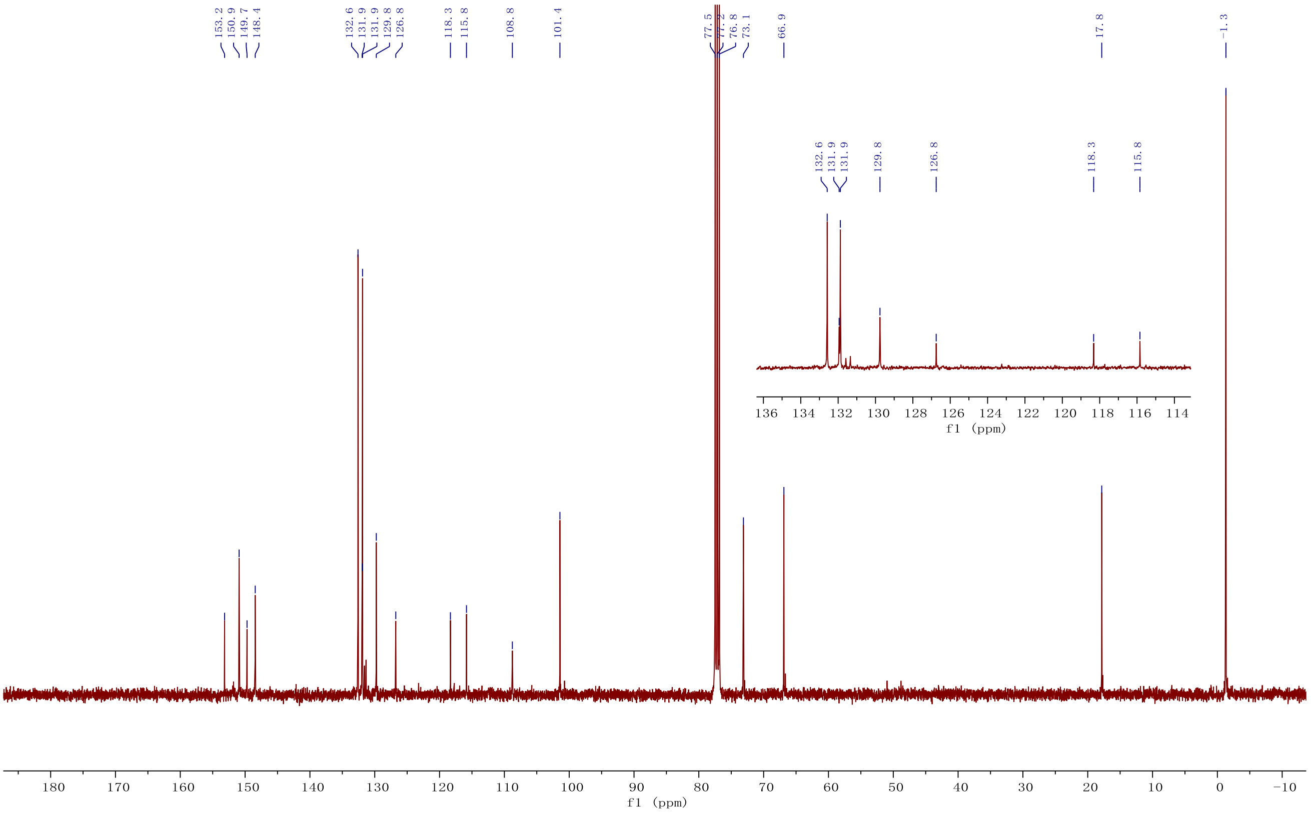

Supplementary Figure 141. ^{1}H NMR spectrum of compound **1al** (400 MHz, $CDCl_3$)

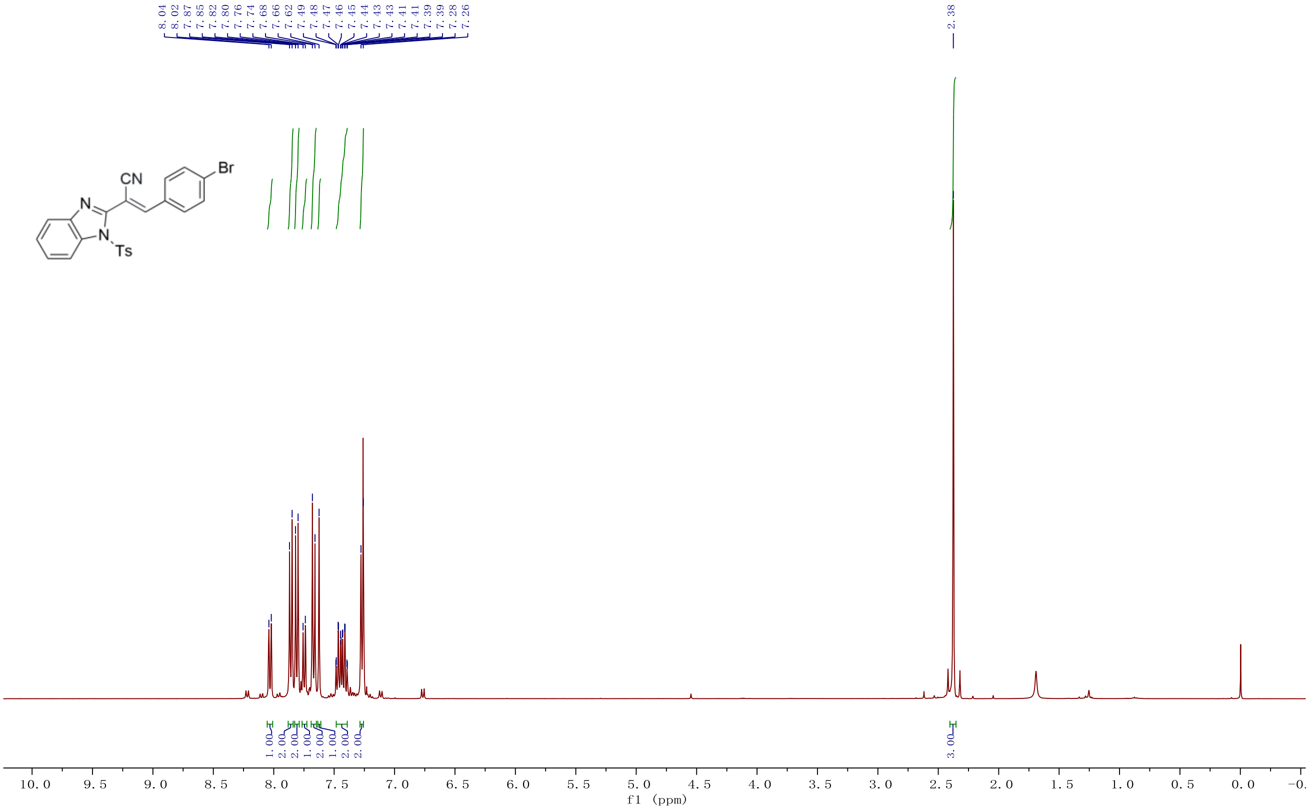

Supplementary Figure 142. ^{13}C NMR spectrum of compound **1al** (100 MHz, $CDCl_3$)

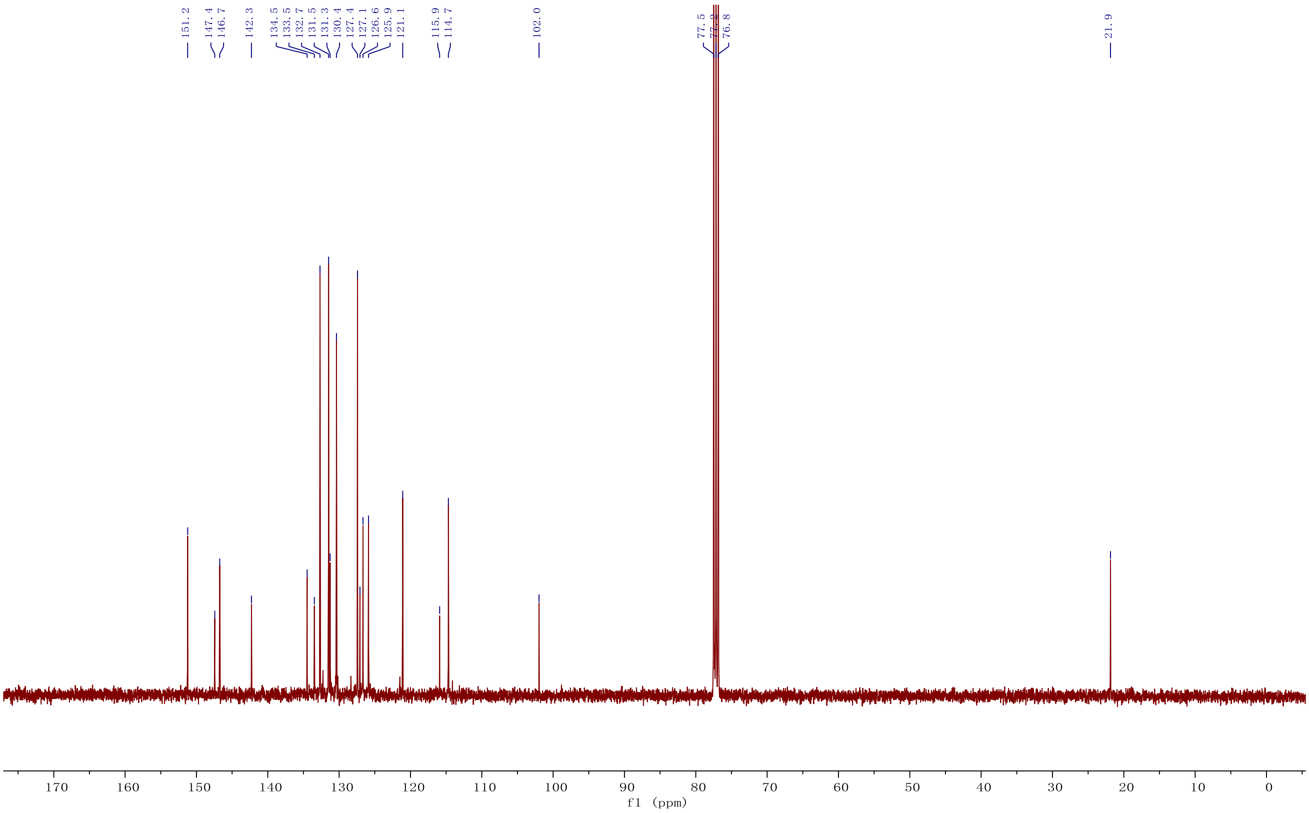

Supplementary Figure 143. ^{1}H NMR spectrum of compound **1am** (600 MHz, $CDCl_3$)

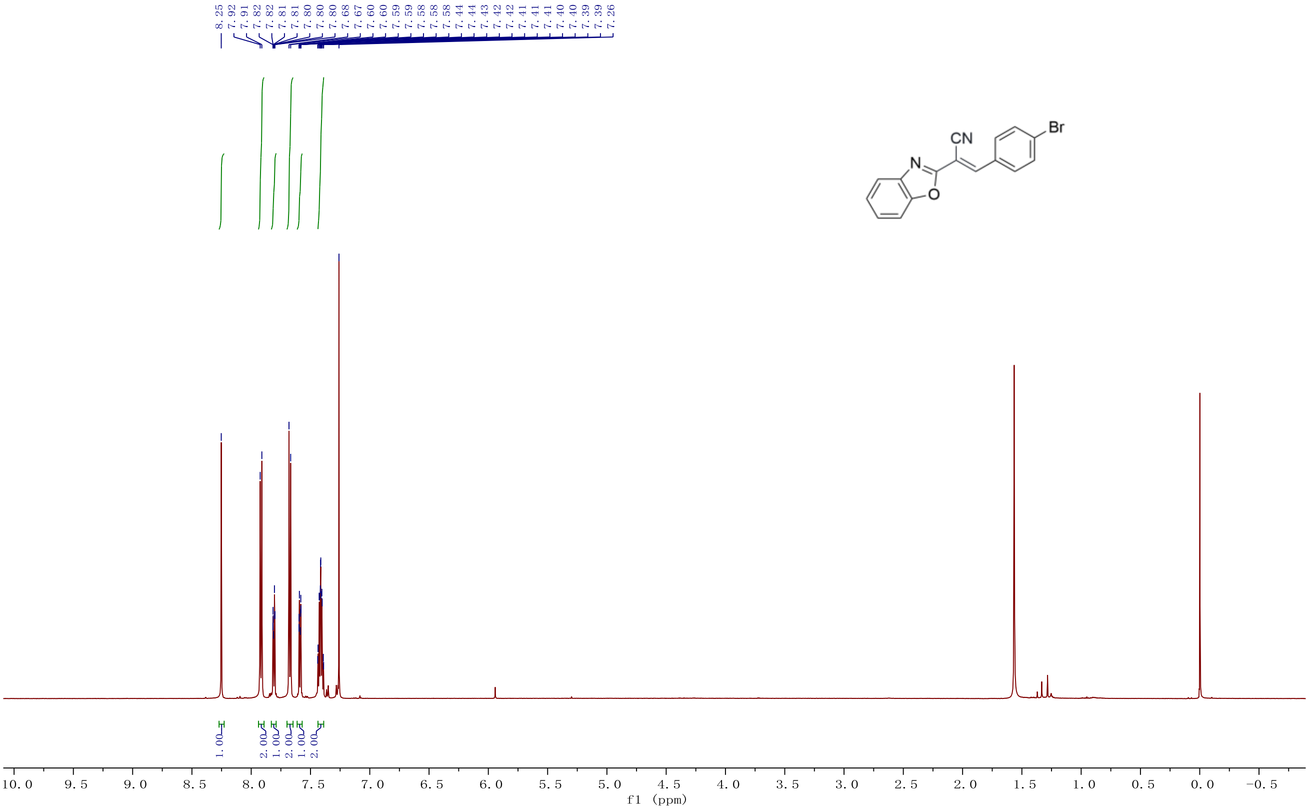

Supplementary Figure 144. ^{13}C NMR spectrum of compound **1am** (150 MHz, $CDCl_3$)

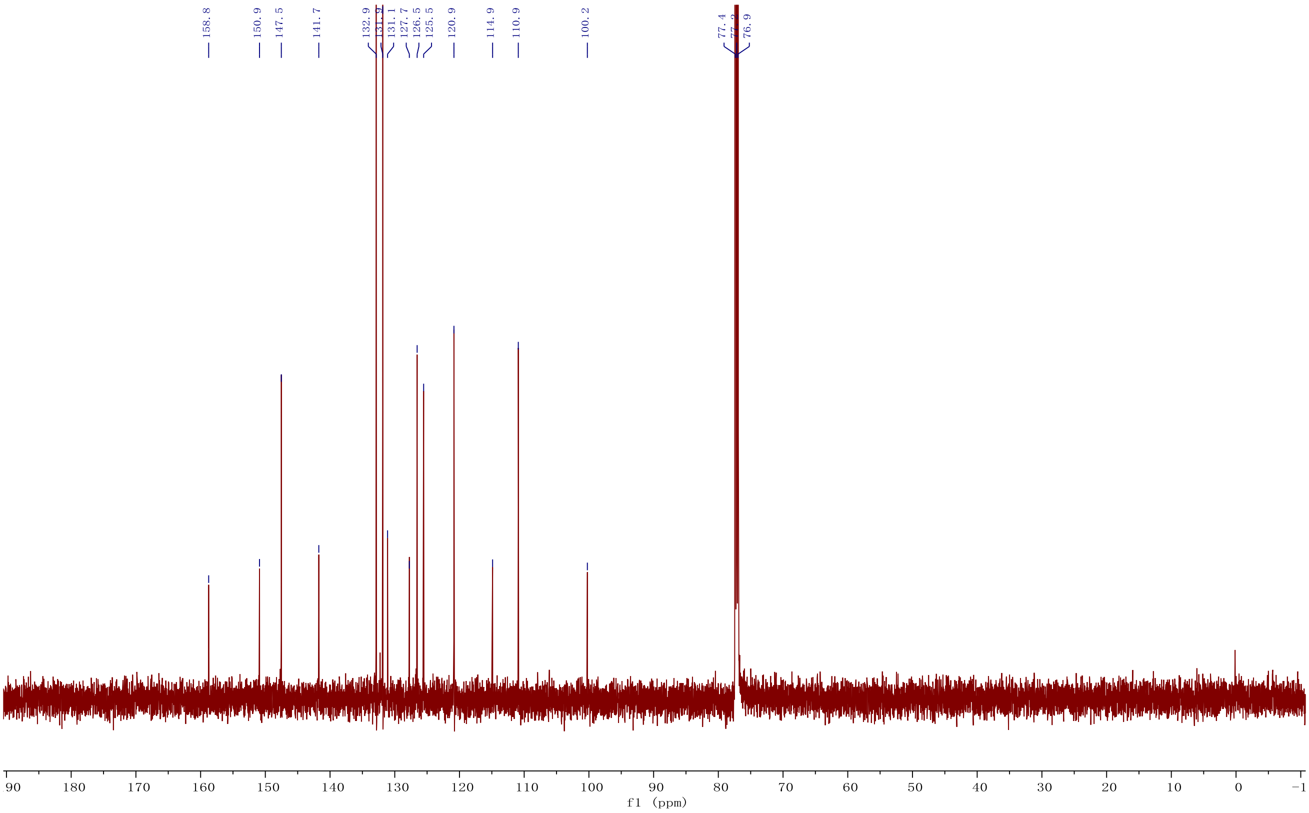

Supplementary Figure 145. ^{1}H NMR spectrum of compound **1an** (400 MHz, DMSO-d_6)

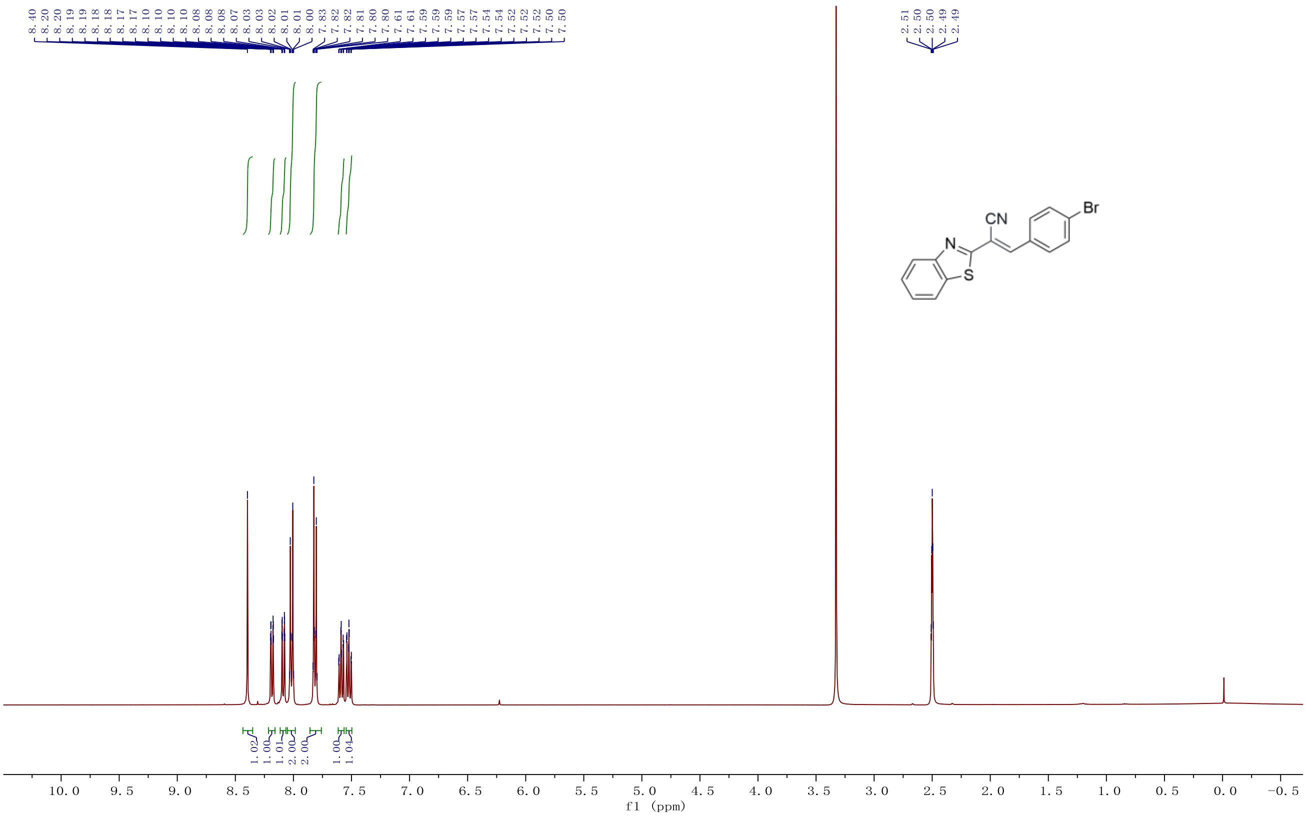

Supplementary Figure 146. ^{13}C NMR spectrum of compound **1an** (100 MHz, $CDCl_3$)

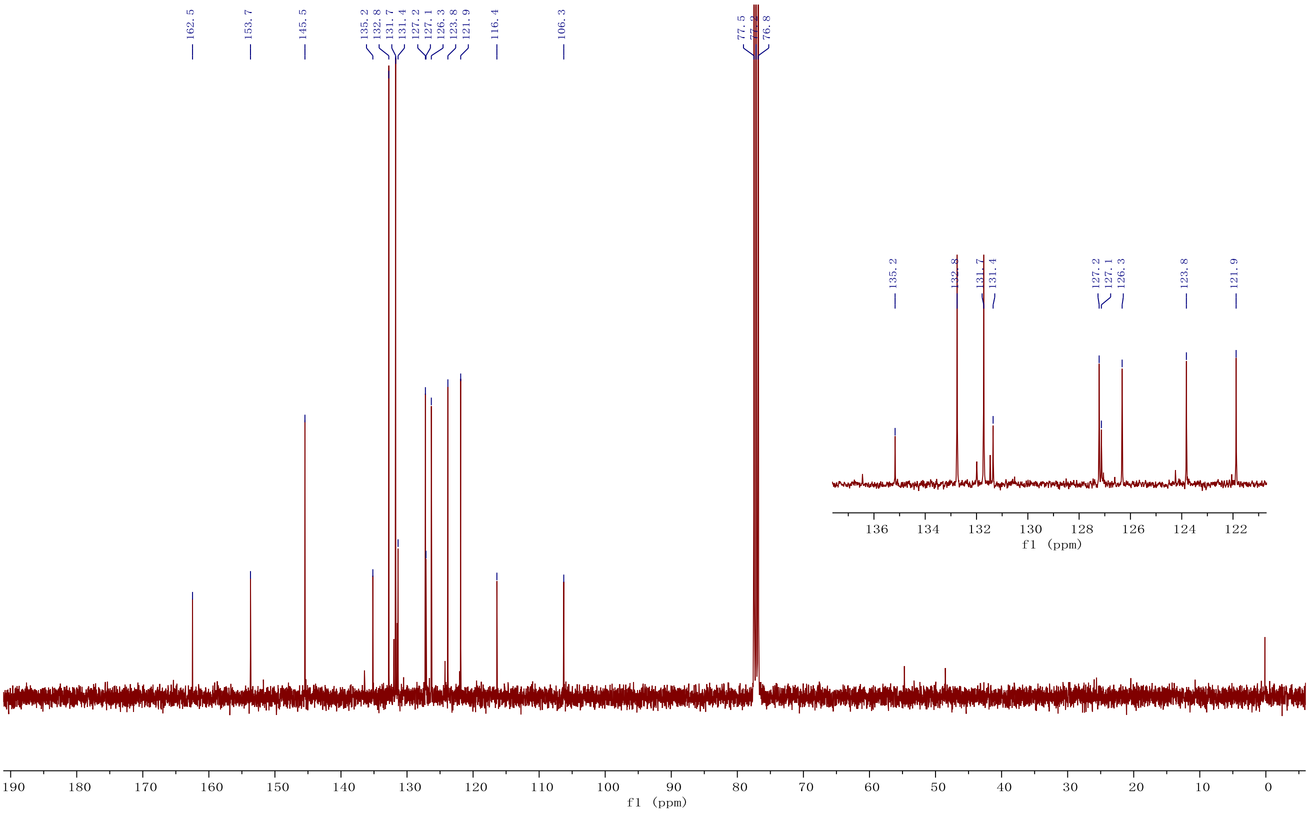

Supplementary Figure 147. ^{1}H NMR spectrum of compound **1ao** (600 MHz, $CDCl_3$)

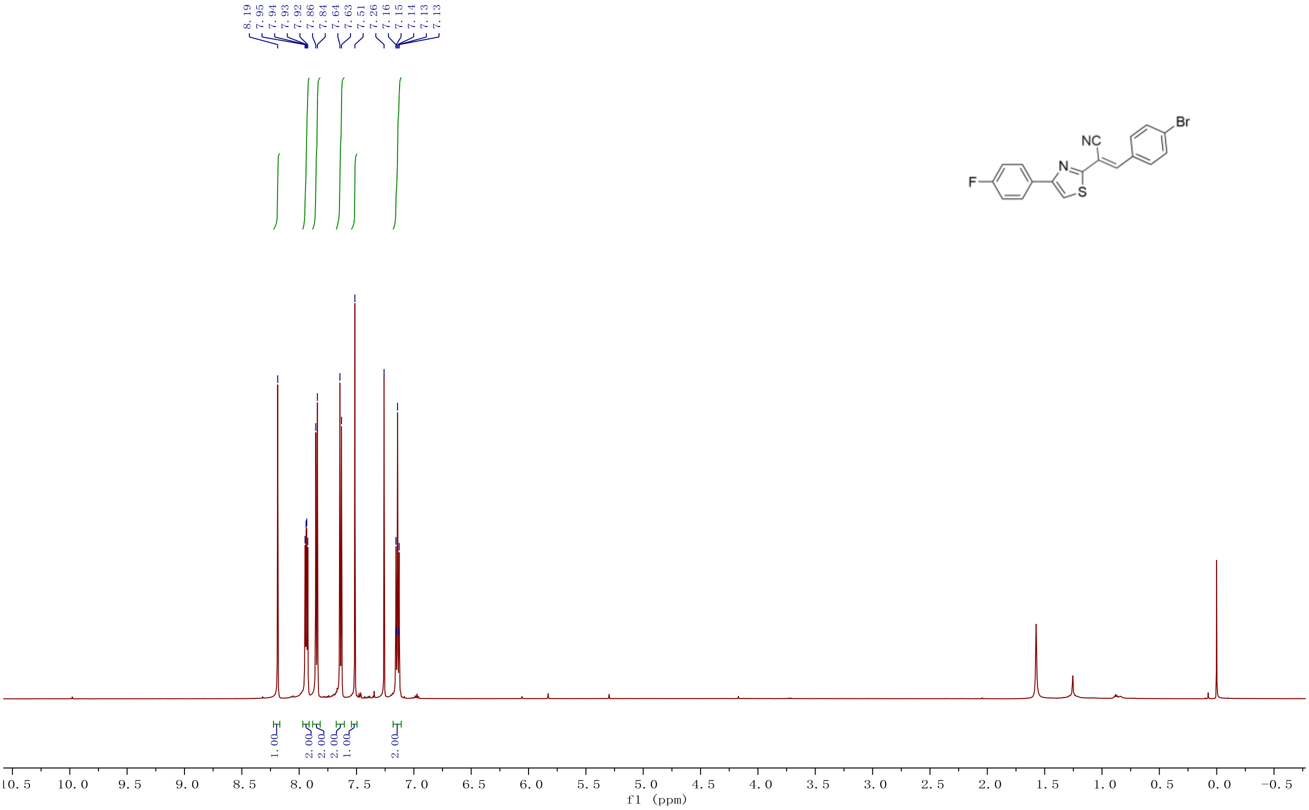

Supplementary Figure 148. ^{13}C NMR spectrum of compound **1ao** (150 MHz, $CDCl_3$)

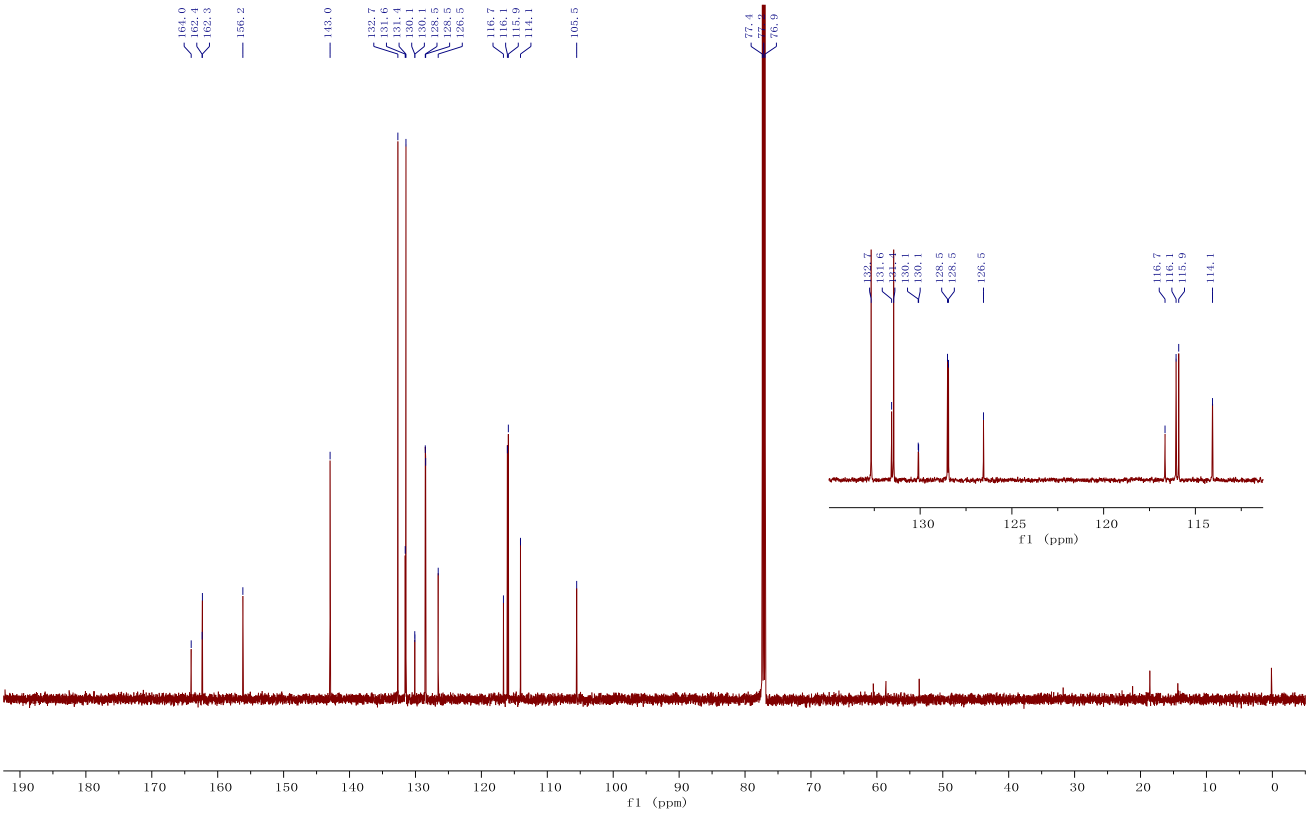

Supplementary Figure 149. ^{19}F NMR spectrum of compound **1ao** (565 MHz, $CDCl_3$)

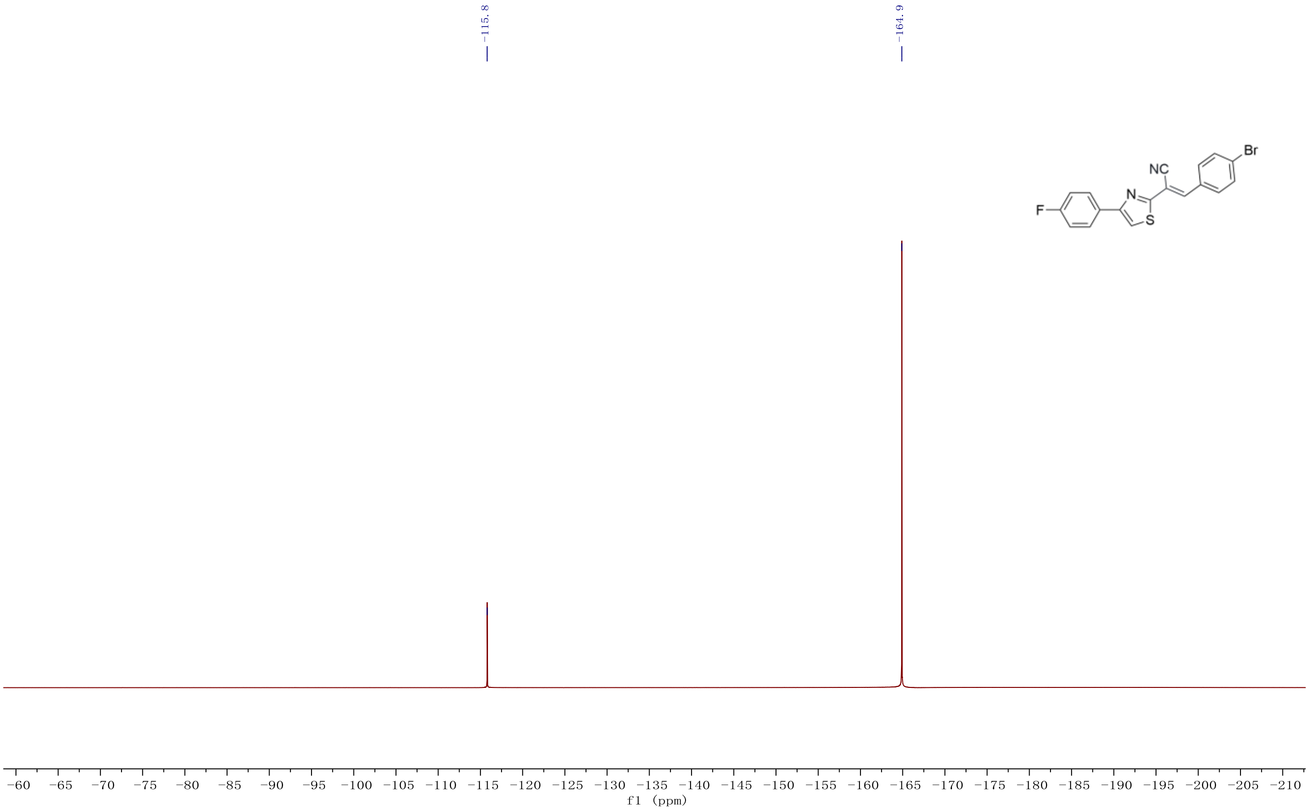

Supplementary Figure 150. ^{1}H NMR spectrum of compound **1ap** (600 MHz, $CDCl_3$)

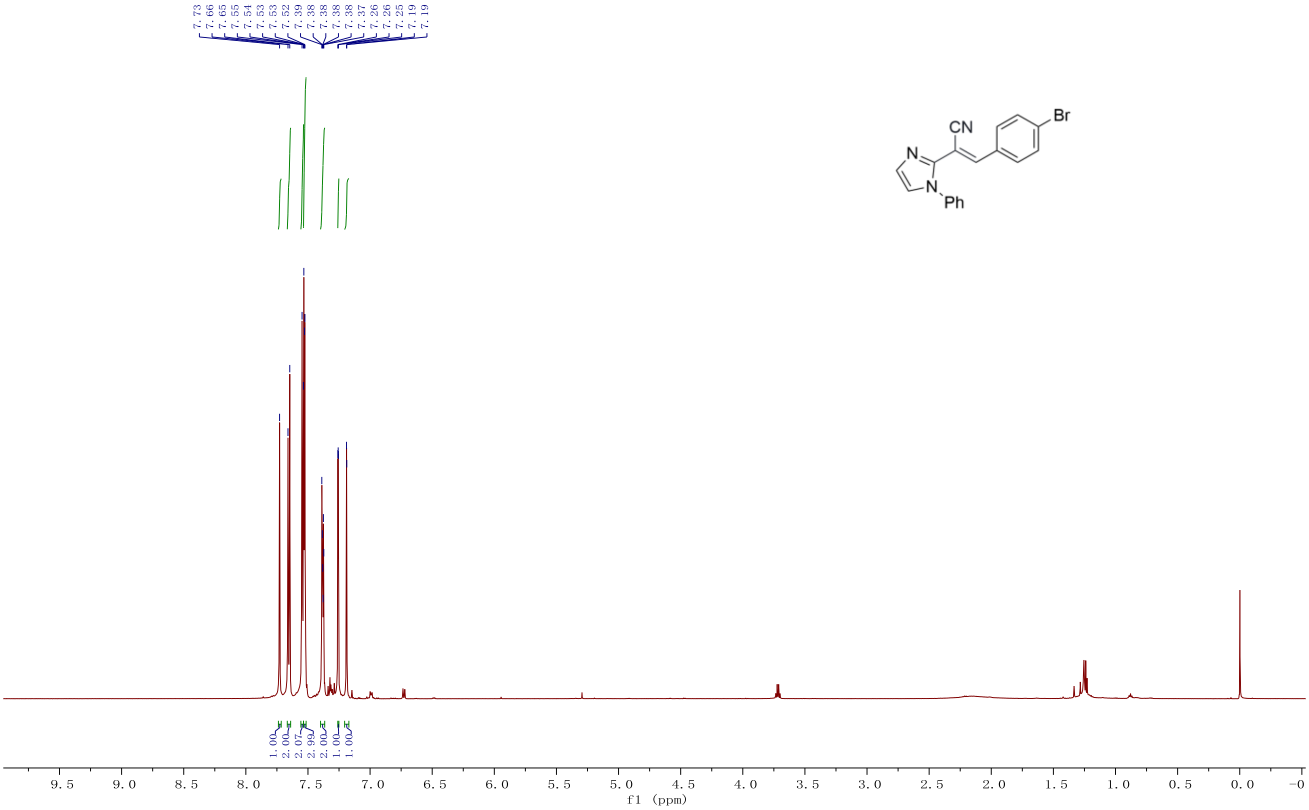

Supplementary Figure 151. ^{13}C NMR spectrum of compound **1ap** (150 MHz, $CDCl_3$)

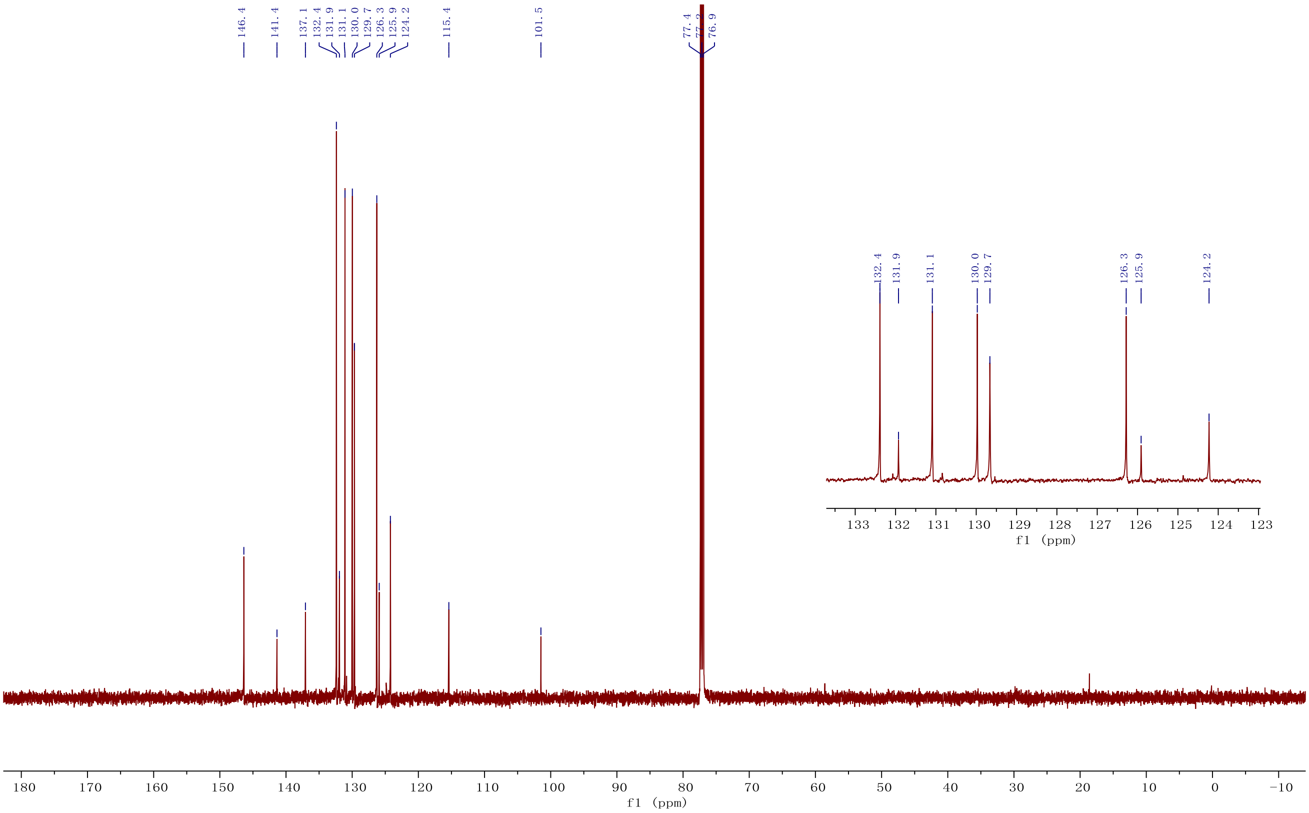

Supplementary Figure 152. ^{1}H NMR spectrum of compound **1aq** (400 MHz, $CDCl_3$)

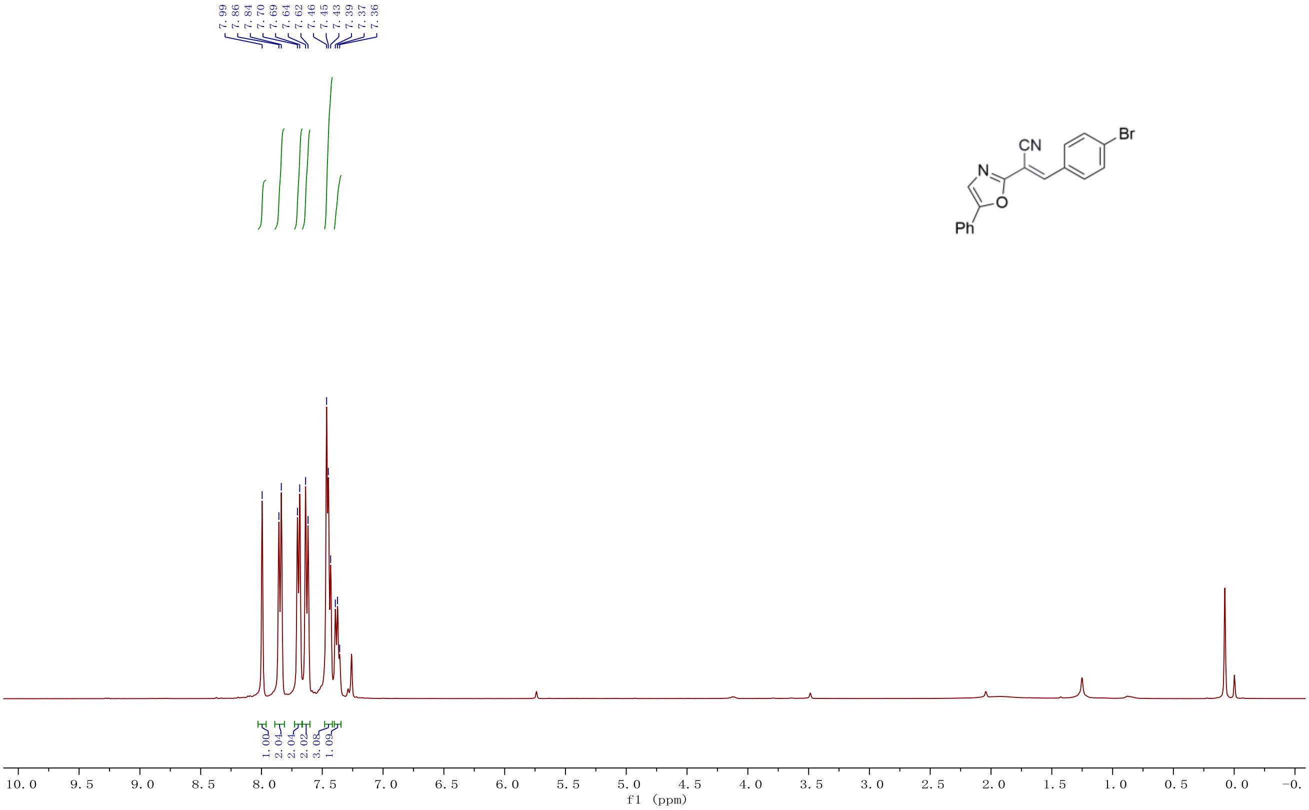

Supplementary Figure 153. ^{13}C NMR spectrum of compound **1aq** (100 MHz, $CDCl_3$)

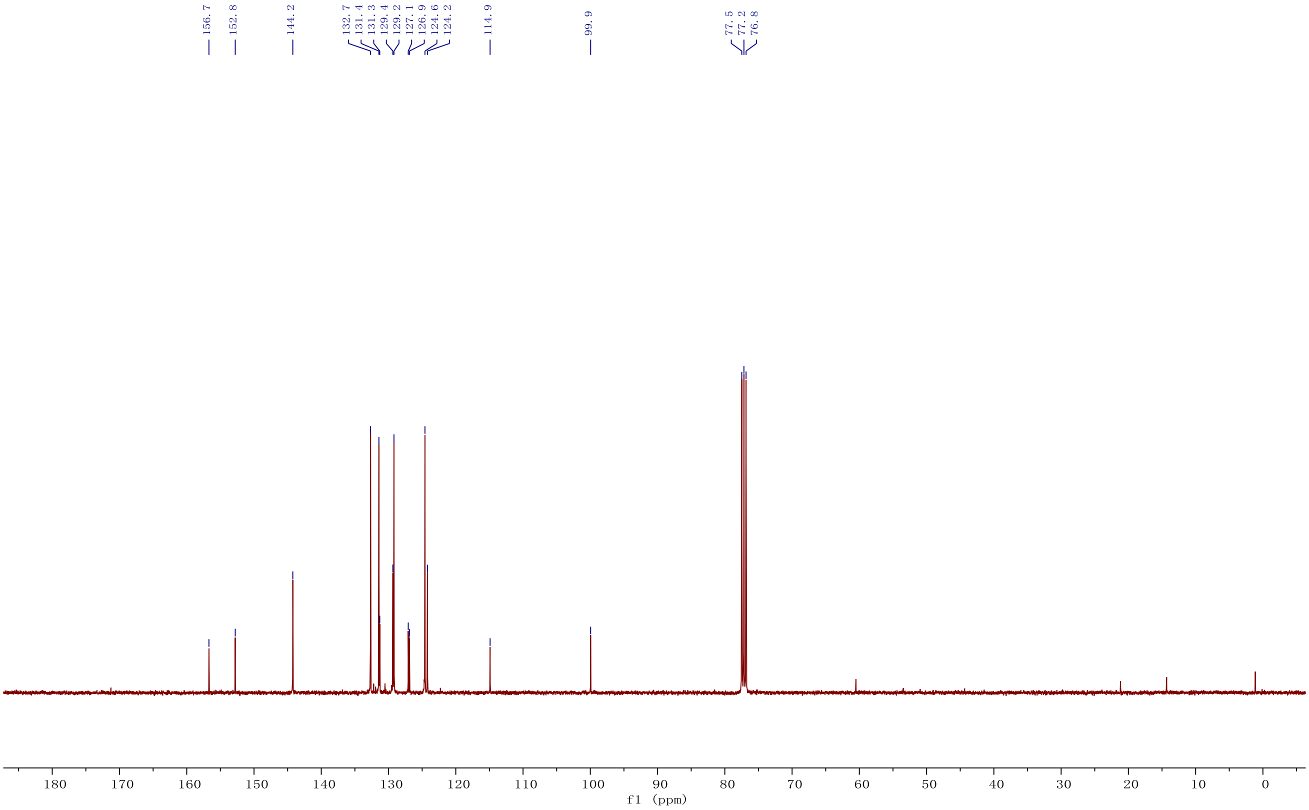

Supplementary Figure 154. ^{1}H NMR spectrum of compound **1ar** (400 MHz, $CDCl_3$)

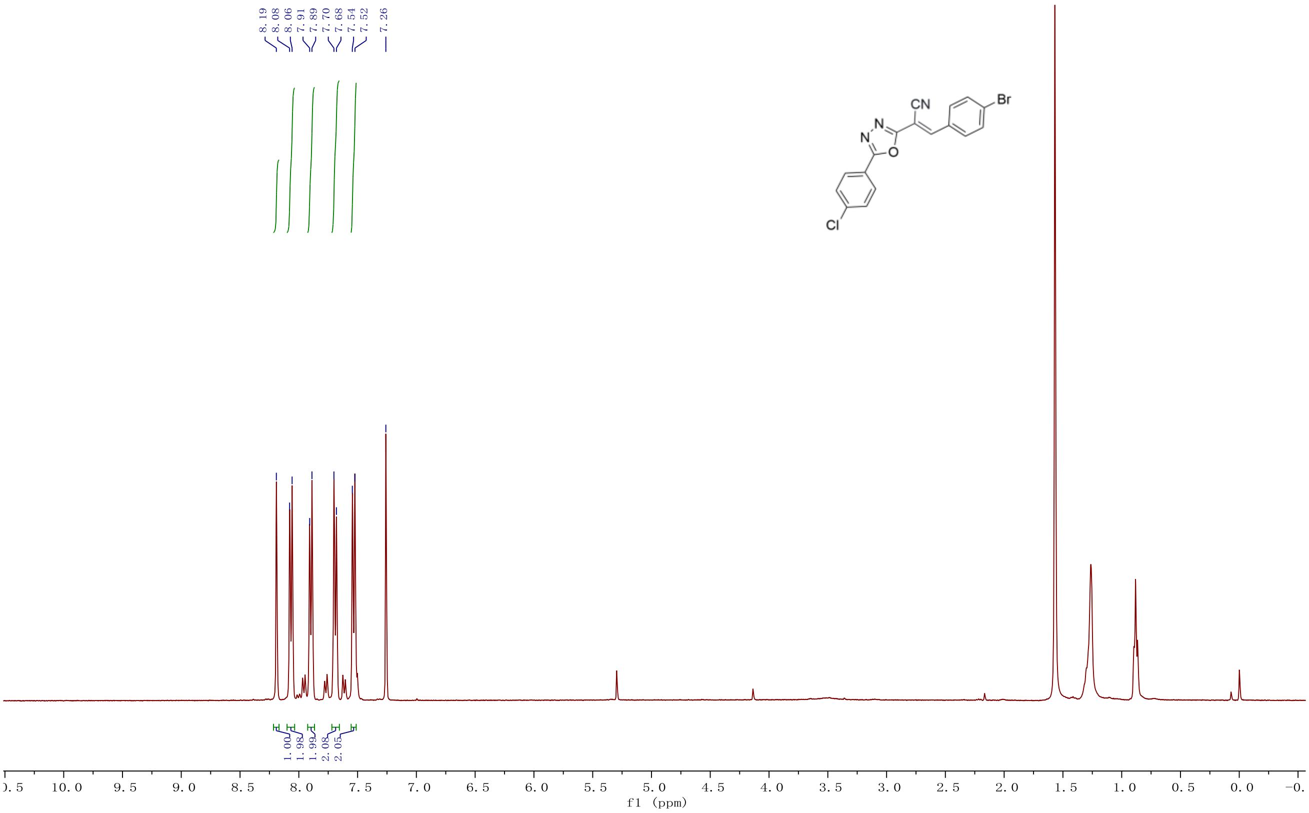

Supplementary Figure 155. ^{13}C NMR spectrum of compound **1ar** (100 MHz, $CDCl_3$)

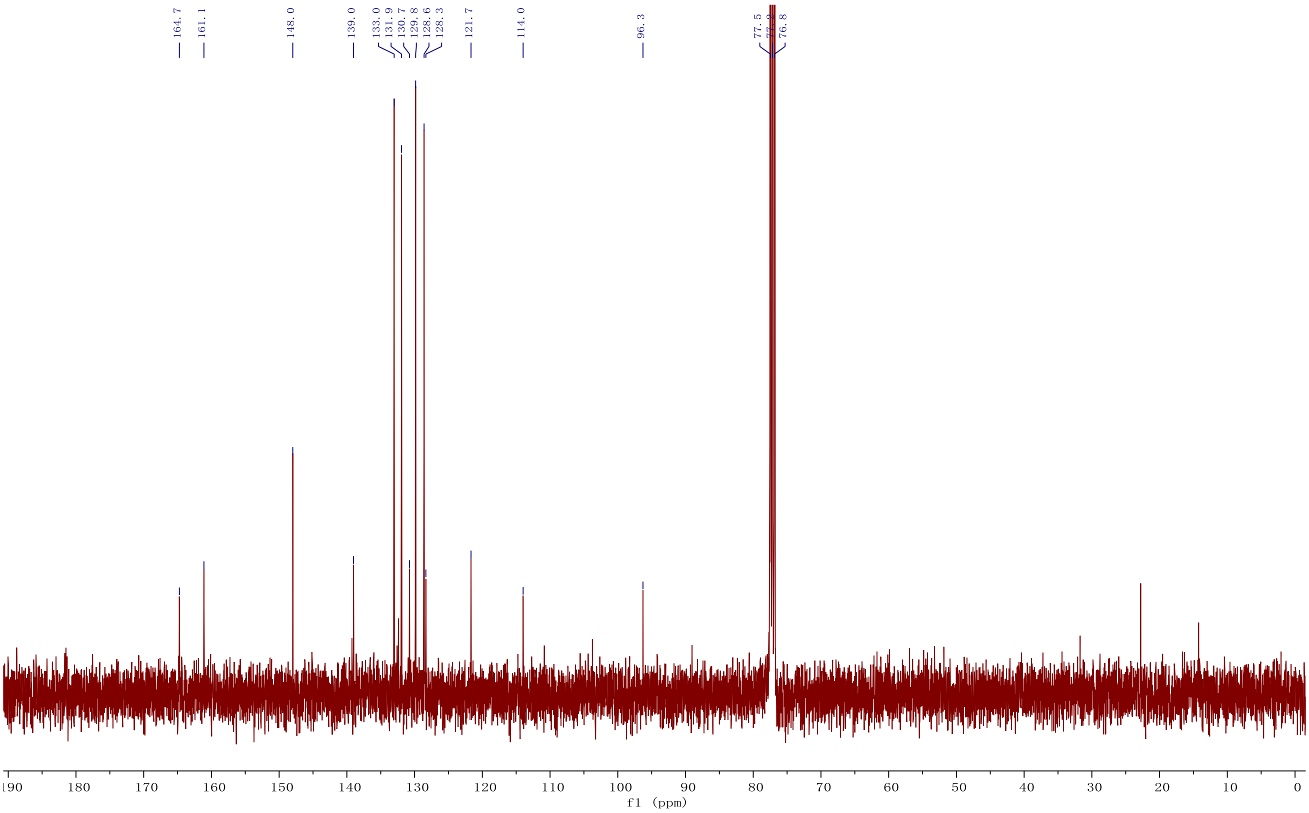

Supplementary Figure 156. ^{1}H NMR spectrum of compound **1as** (400 MHz, DMSO-d_6)

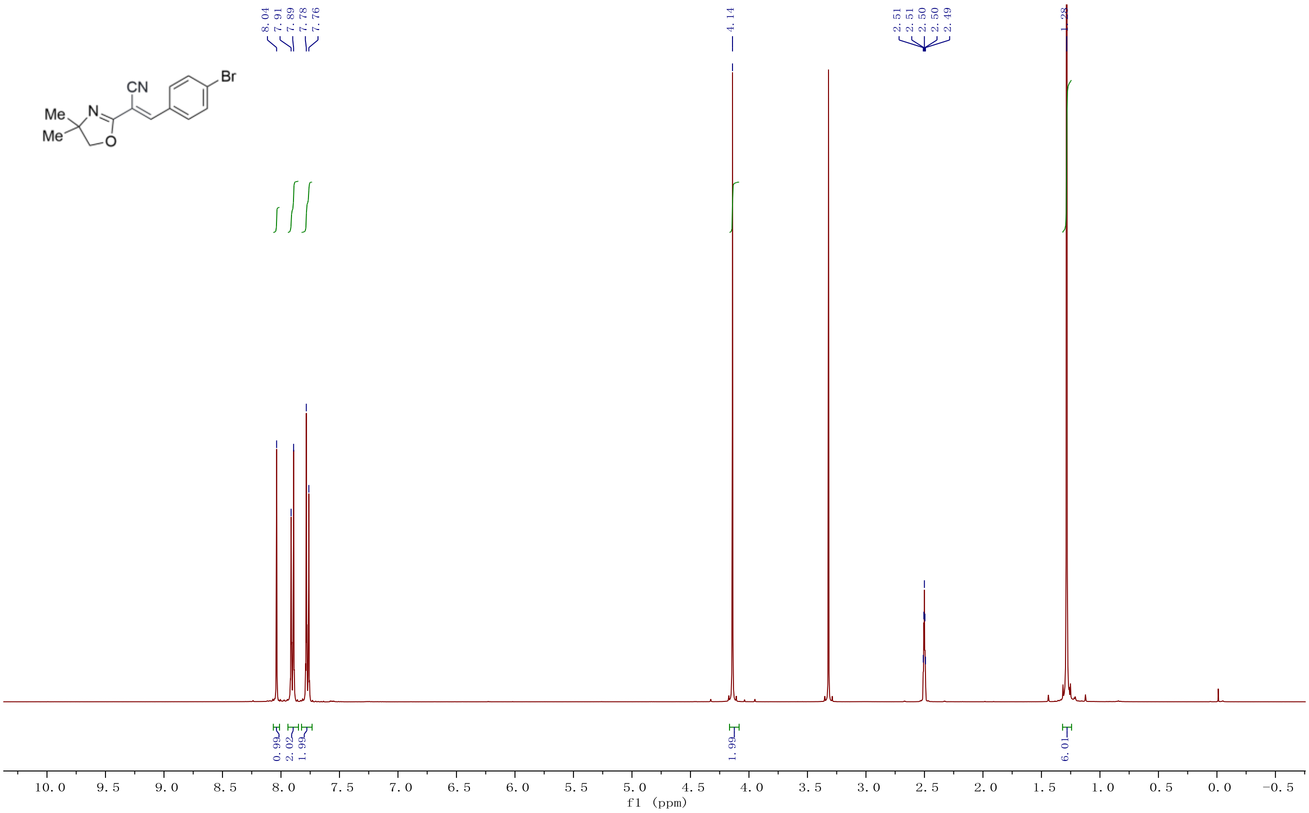

Supplementary Figure 157. ^{13}C NMR spectrum of compound **1as** (100 MHz, $CDCl_3$)

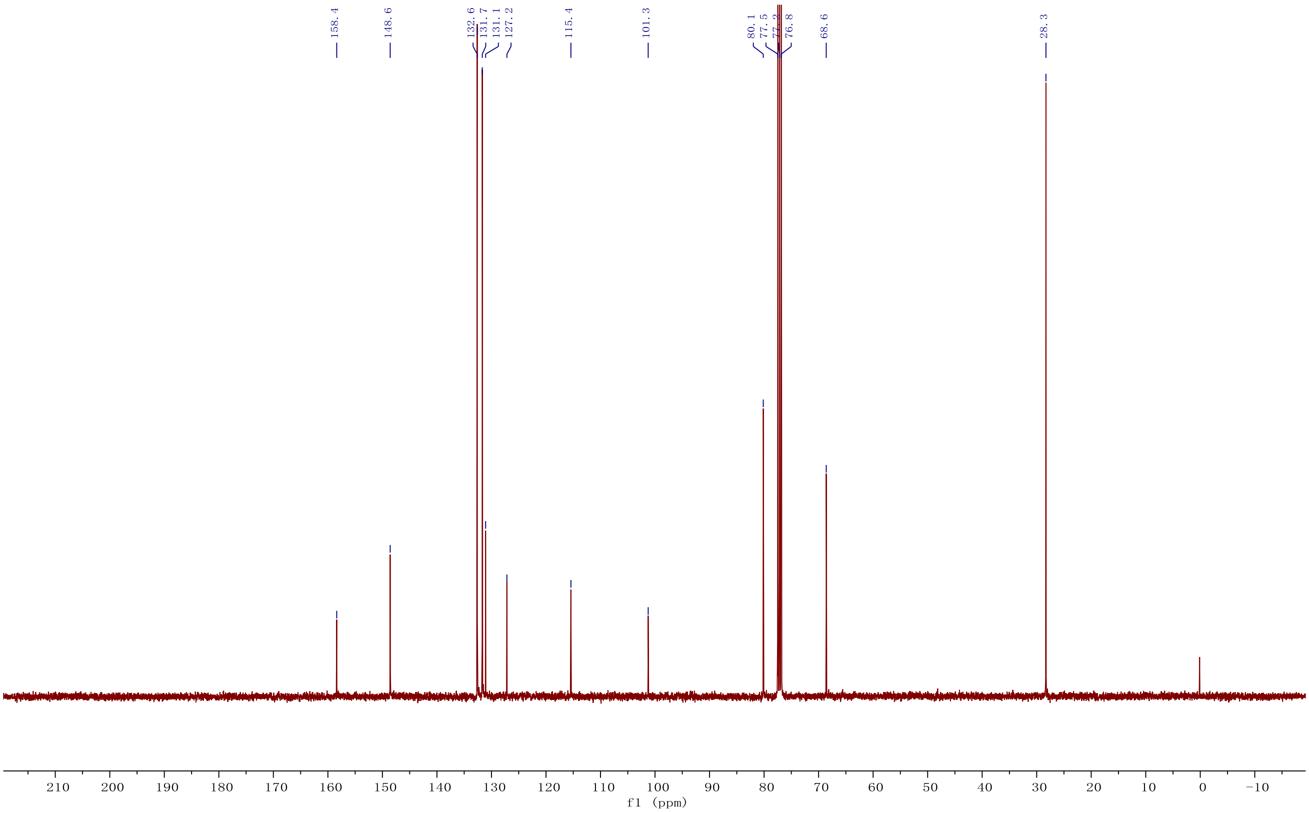

Supplementary Figure 158. ^{1}H NMR spectrum of compound **1bb** (400 MHz, $CDCl_3$)

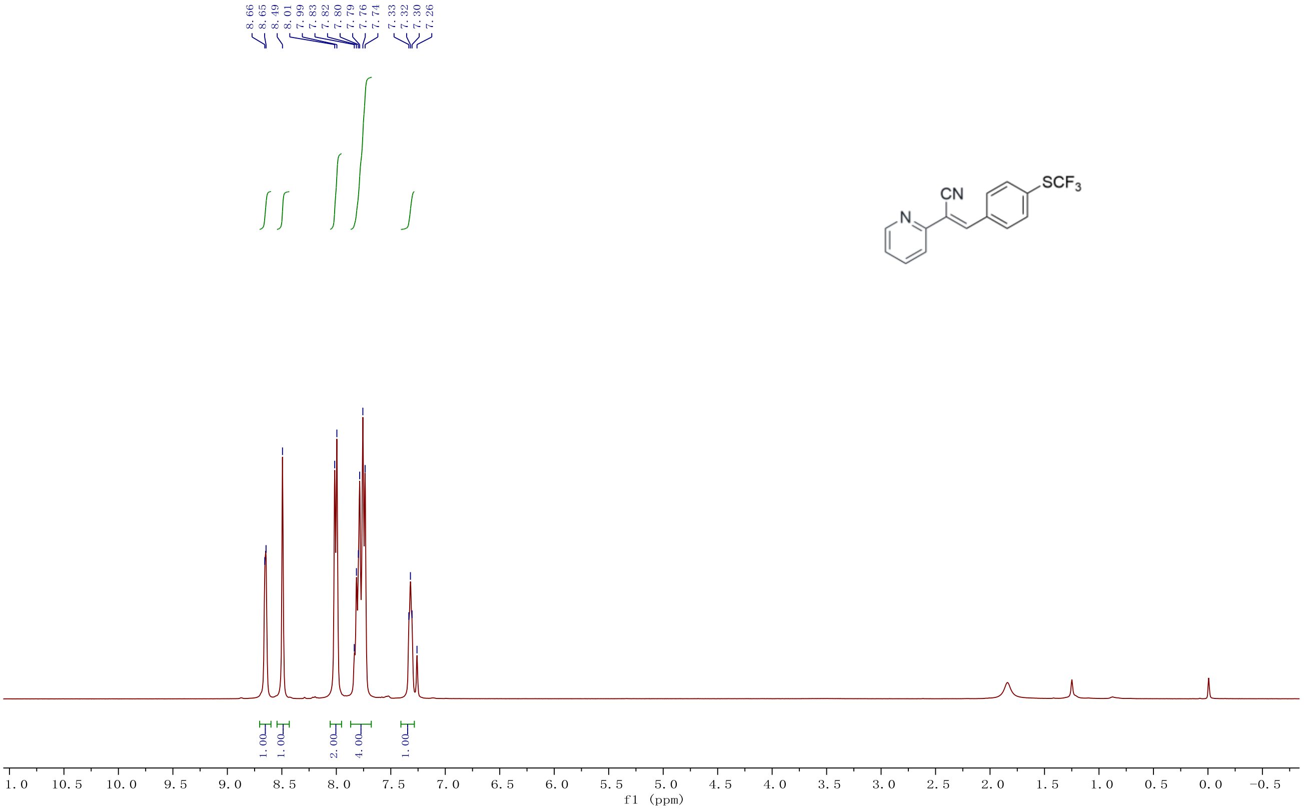

Supplementary Figure 159. ^{13}C NMR spectrum of compound **1bb** (100 MHz, $CDCl_3$)

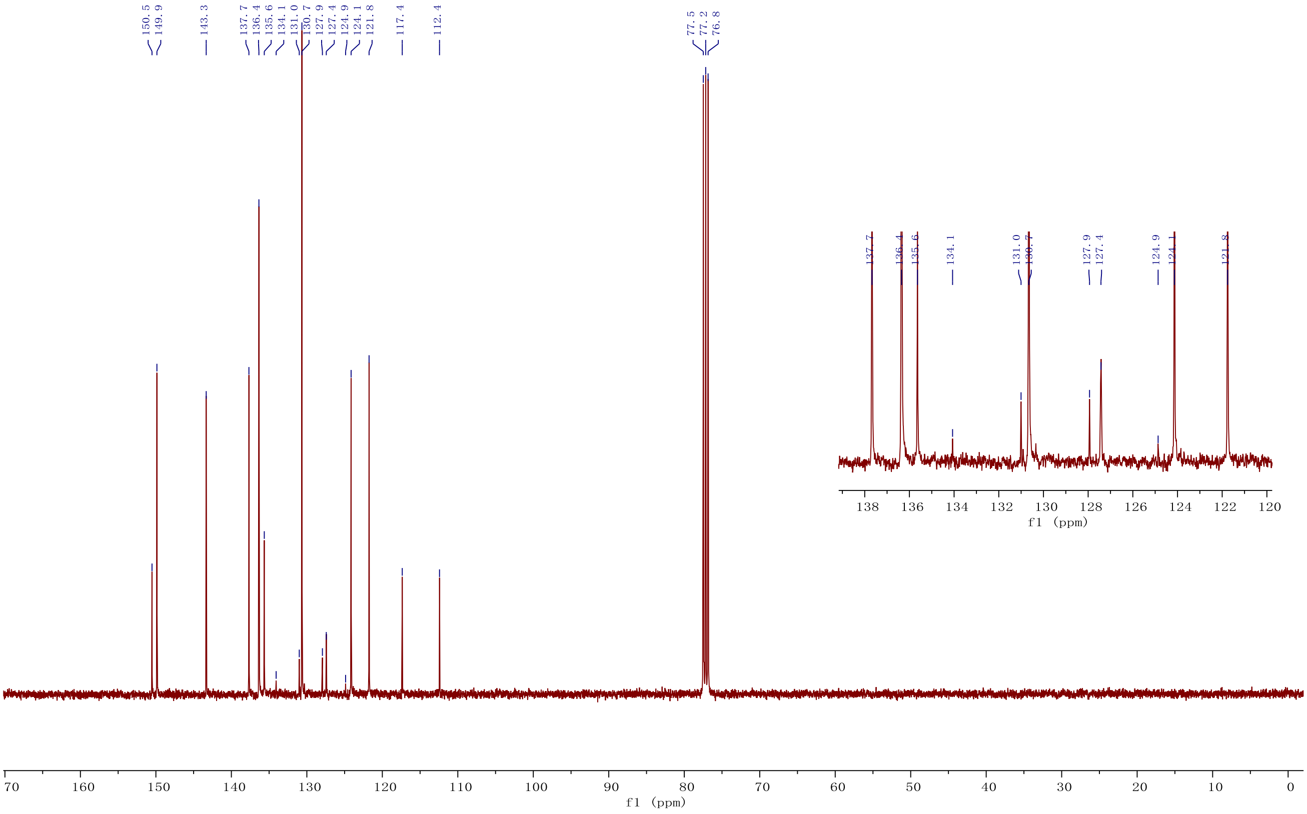

Supplementary Figure 160. ^{19}F NMR spectrum of compound **1bb** (376 MHz, $CDCl_3$)

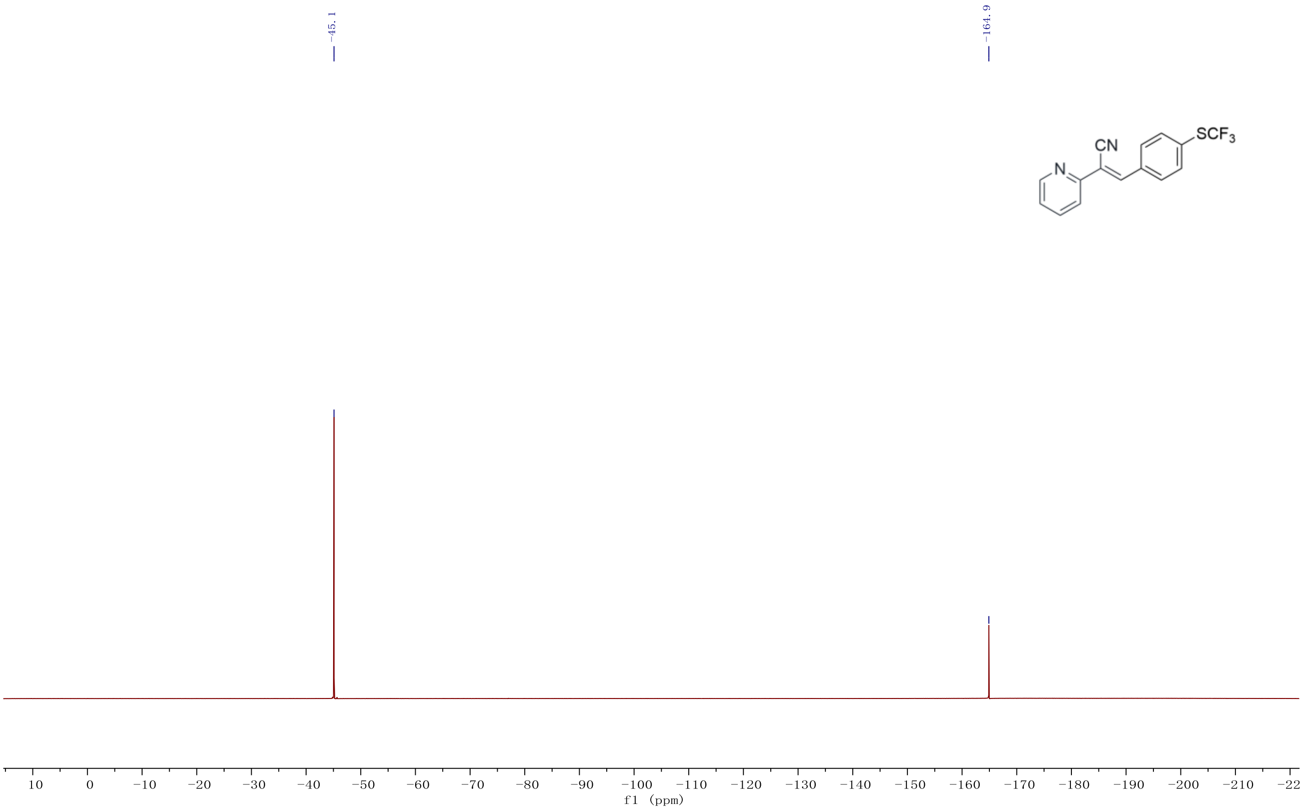

Supplementary Figure 161. ^{1}H NMR spectrum of compound **1bc** (400 MHz, $CDCl_3$)

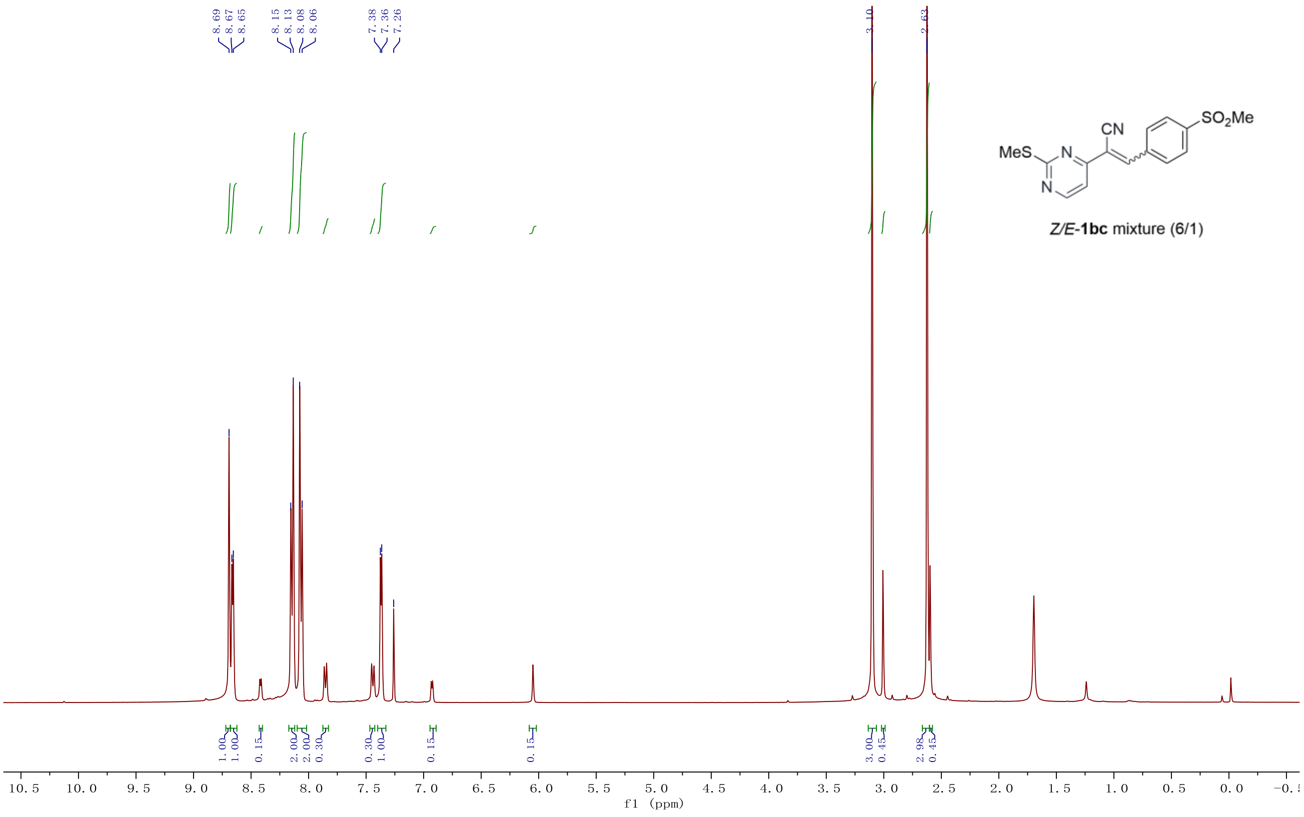

Supplementary Figure 162. ^{13}C NMR spectrum of compound **1bc** (100 MHz, $CDCl_3$)

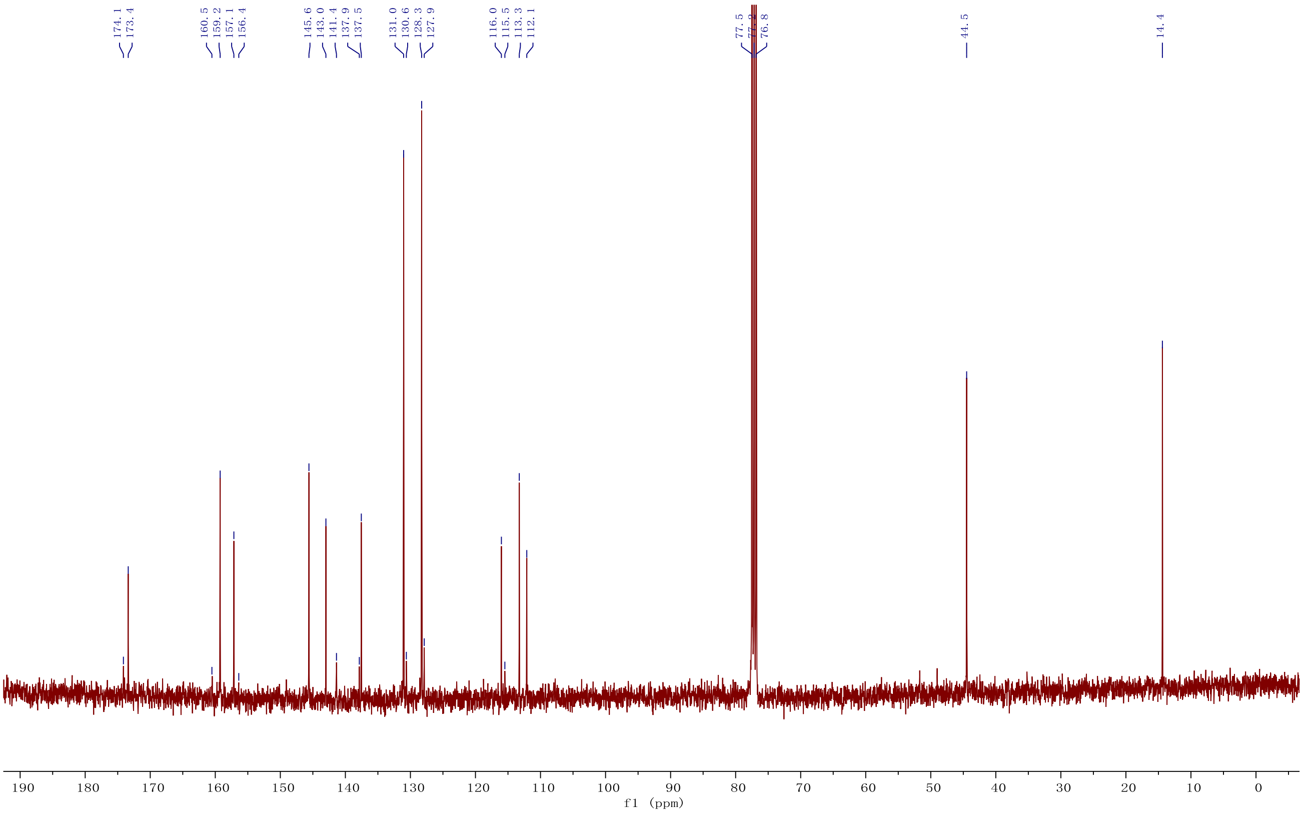

Supplementary Figure 163. ¹H NMR spectrum of compound **1bd** (400 MHz, $CDCl_3$)

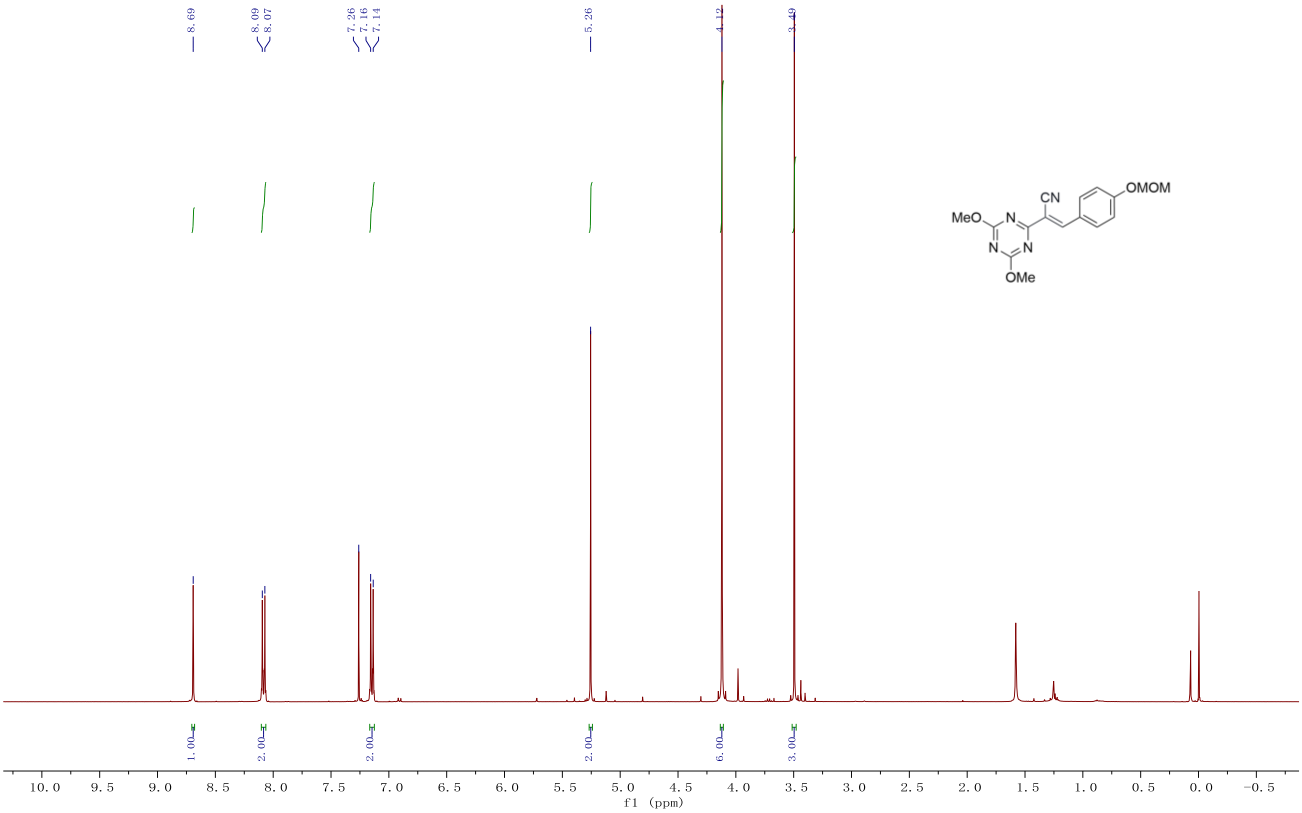

Supplementary Figure 164. ¹³C NMR spectrum of compound **1bd** (100 MHz, $CDCl_3$)

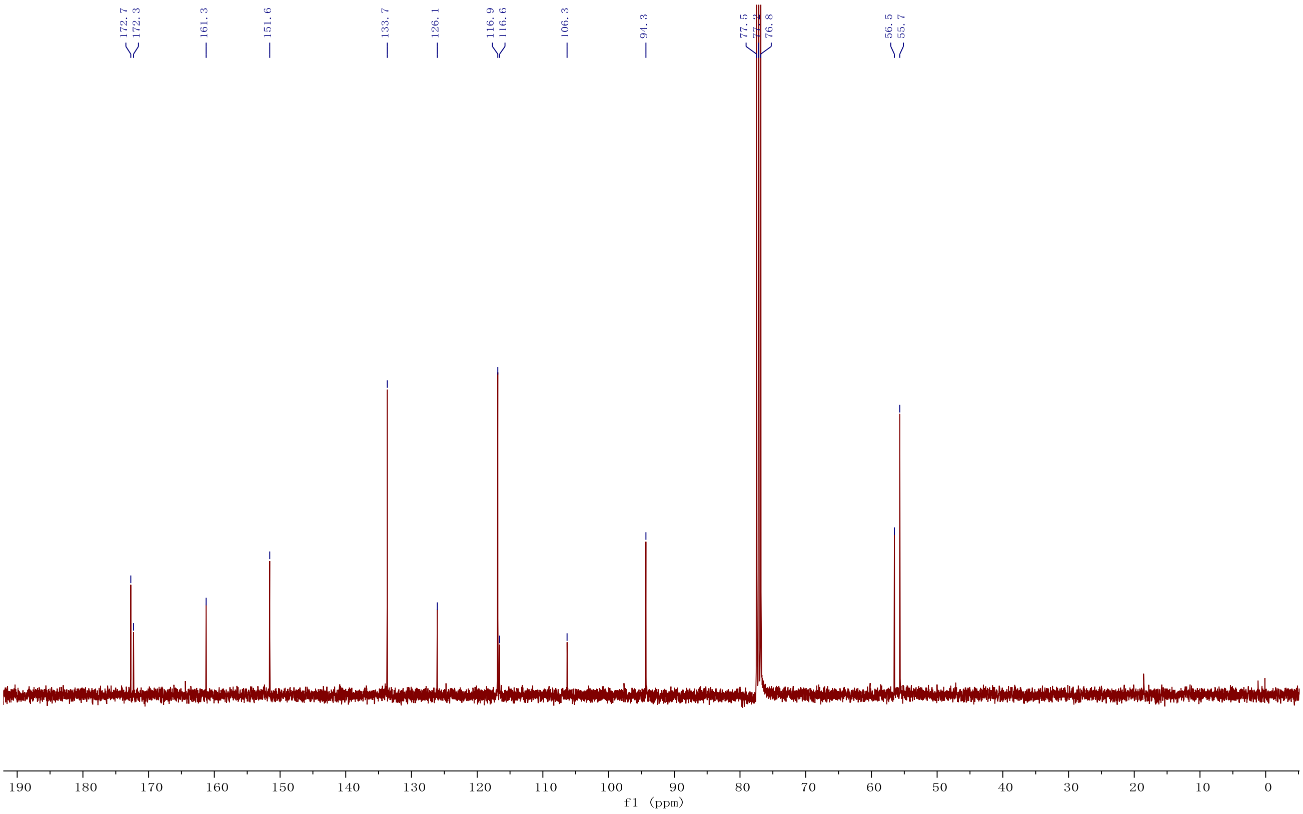

Supplementary Figure 165. ^{1}H NMR spectrum of compound **1be** (400 MHz, $CDCl_3$)

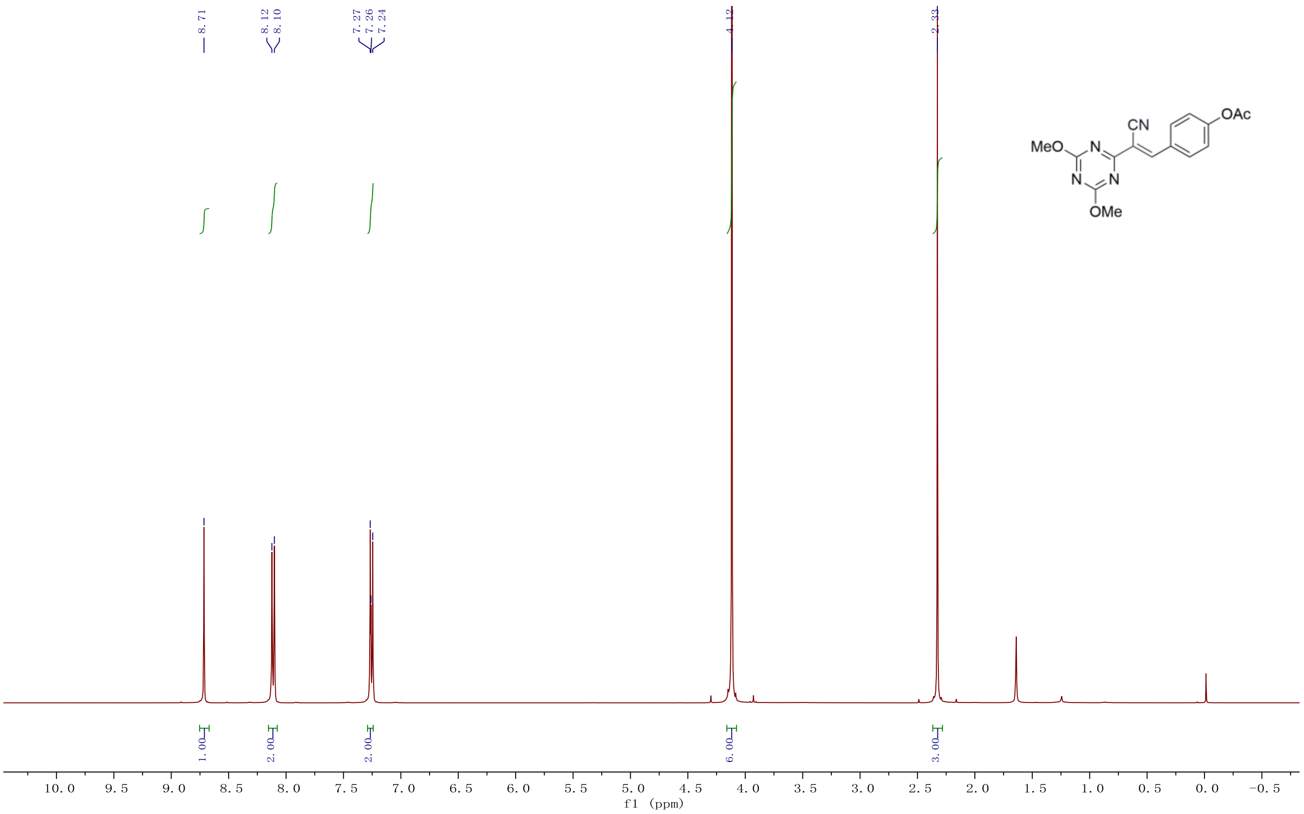

Supplementary Figure 166. ^{13}C NMR spectrum of compound **1be** (100 MHz, $CDCl_3$)

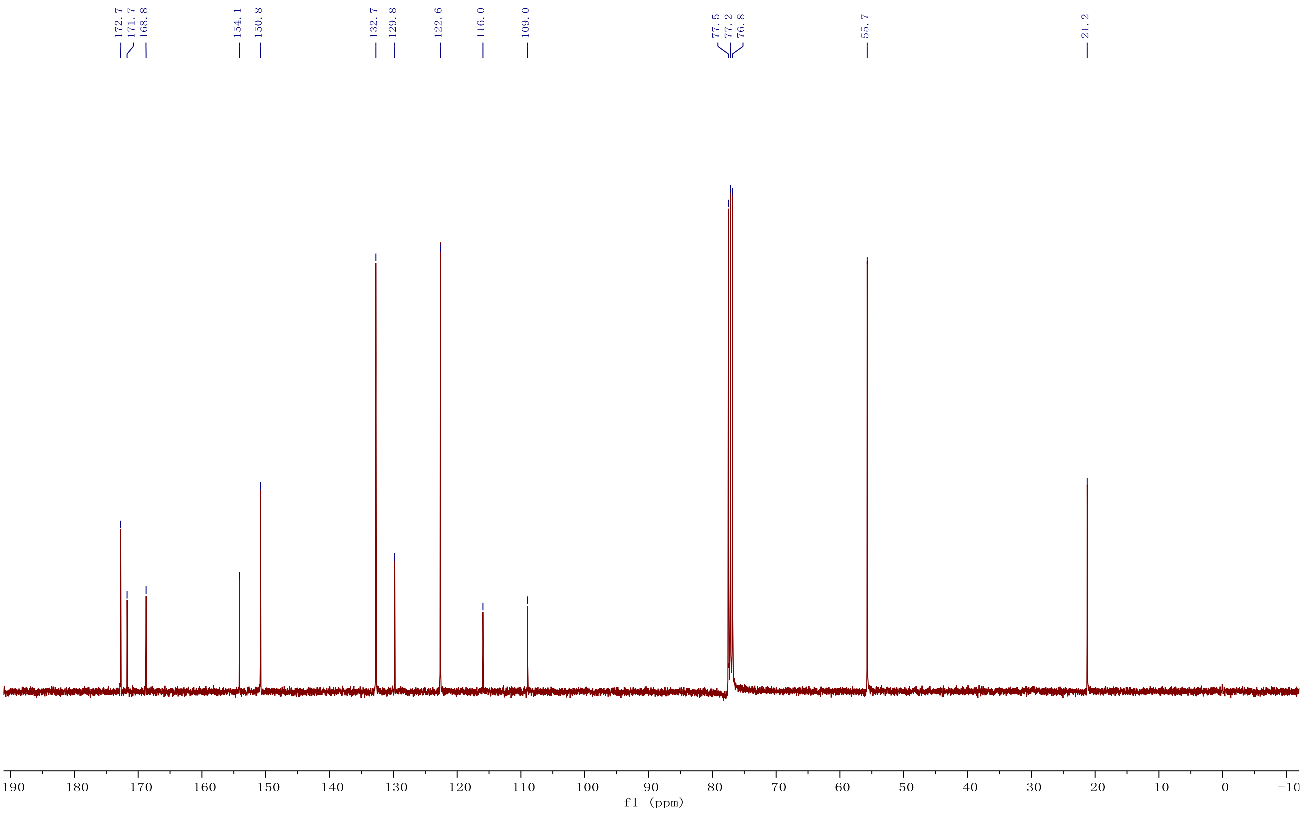

Supplementary Figure 167. ^{1}H NMR spectrum of compound **1bf** (600 MHz, $CDCl_3$)

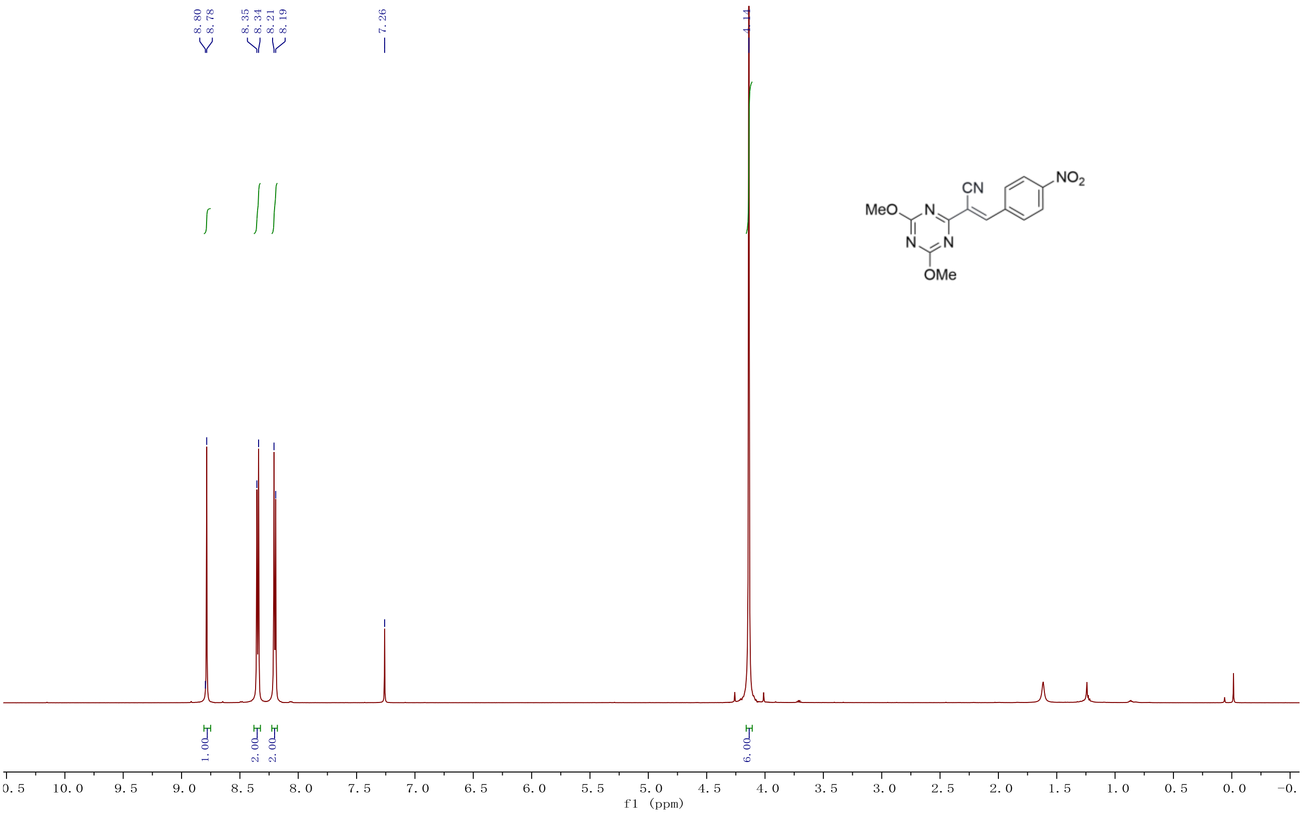

Supplementary Figure 168. ^{13}C NMR spectrum of compound **1bf** (150 MHz, $CDCl_3$)

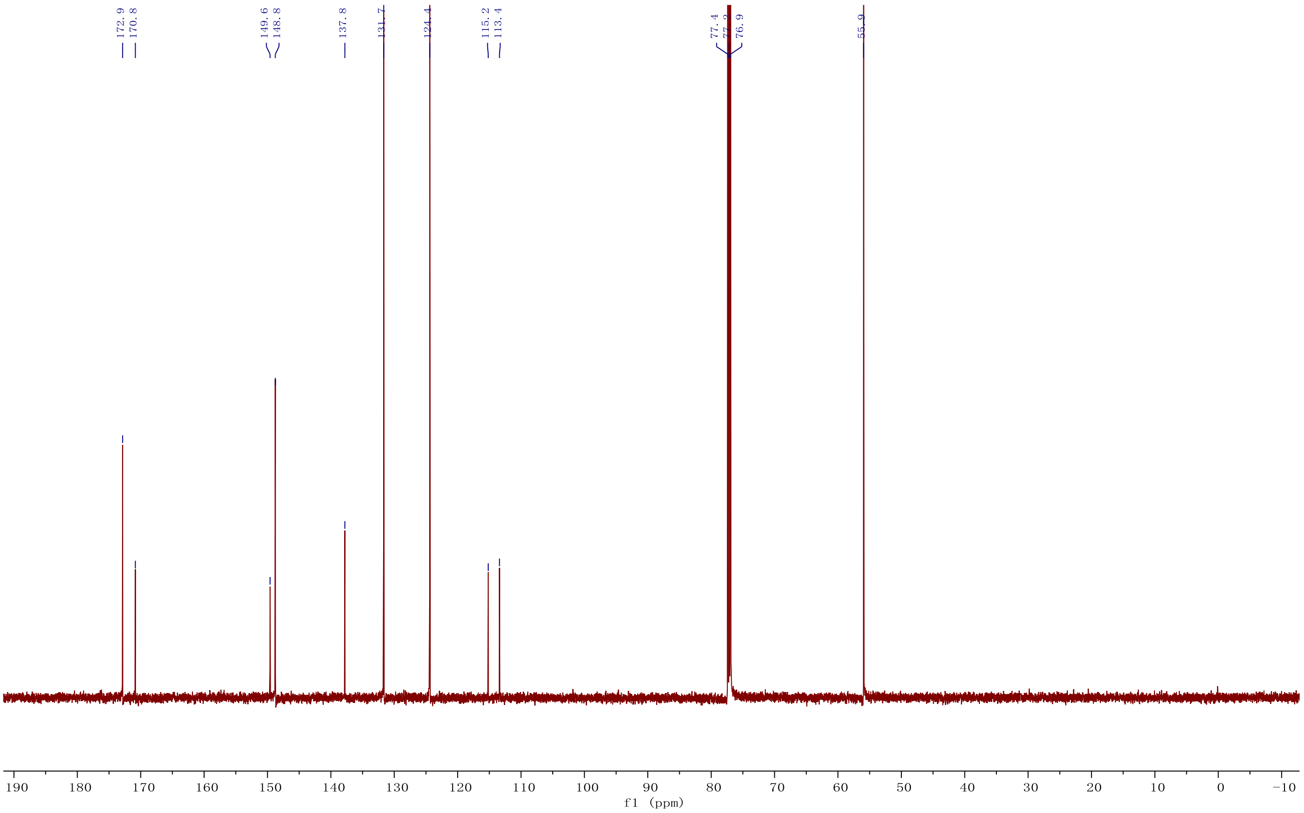

Supplementary Figure 169. ^{1}H NMR spectrum of compound **1bg** (400 MHz, $CDCl_3$)

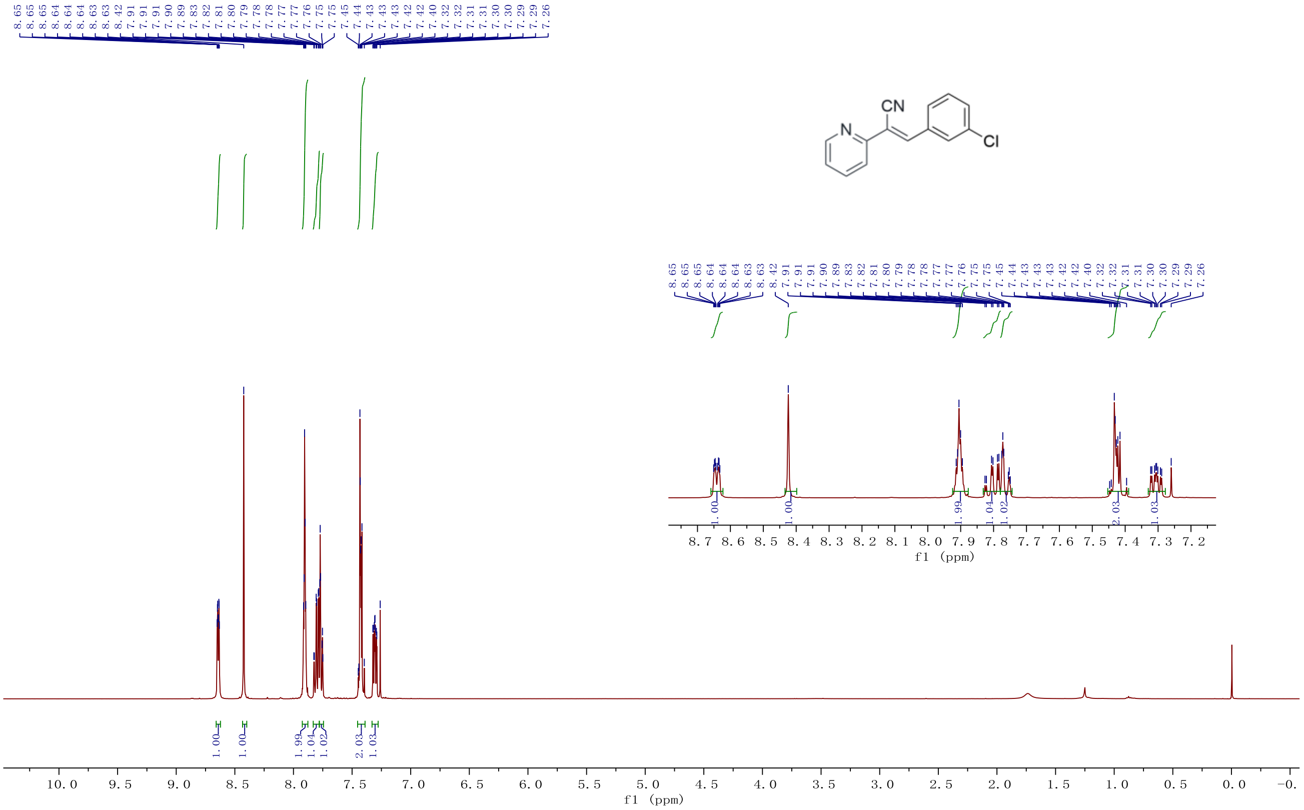

Supplementary Figure 170. ^{13}C NMR spectrum of compound **1bg** (100 MHz, $CDCl_3$)

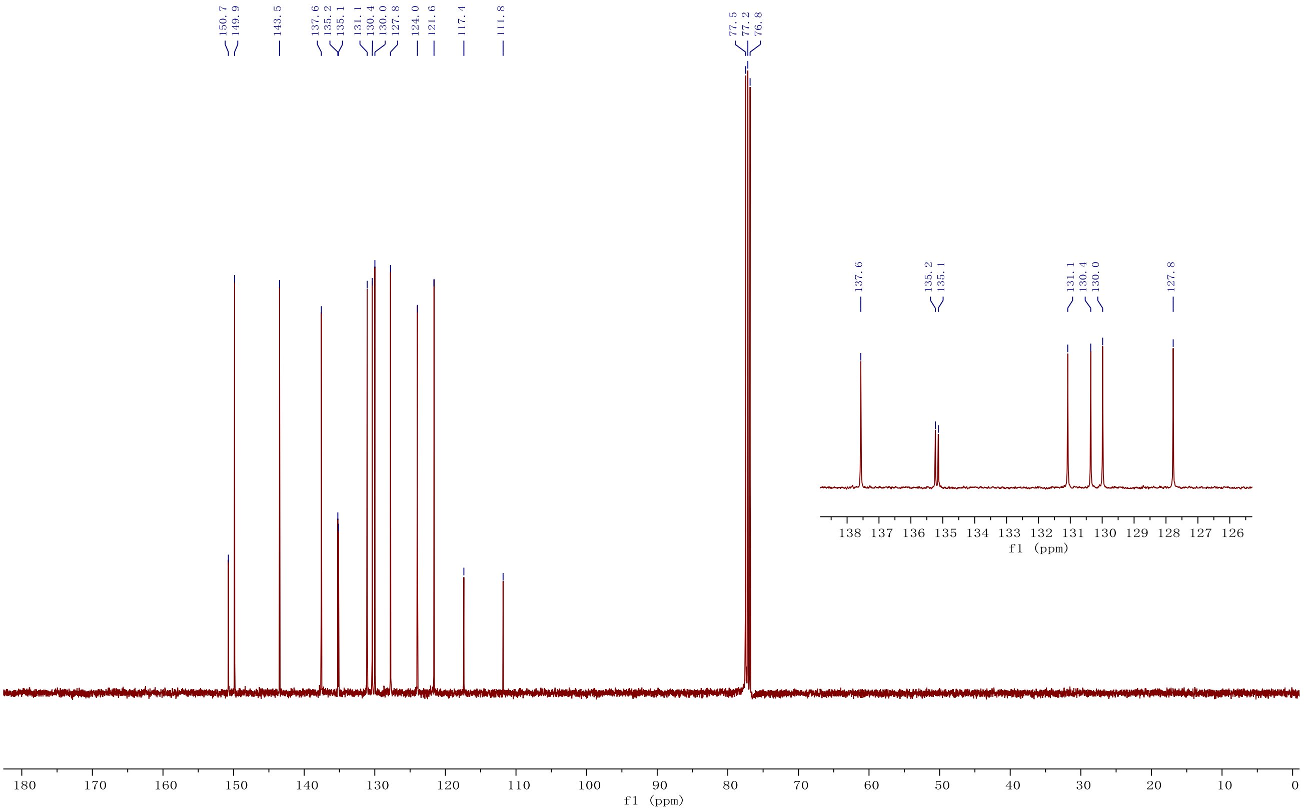

Supplementary Figure 171. ^{1}H NMR spectrum of compound **1bh** (400 MHz, $CDCl_3$)

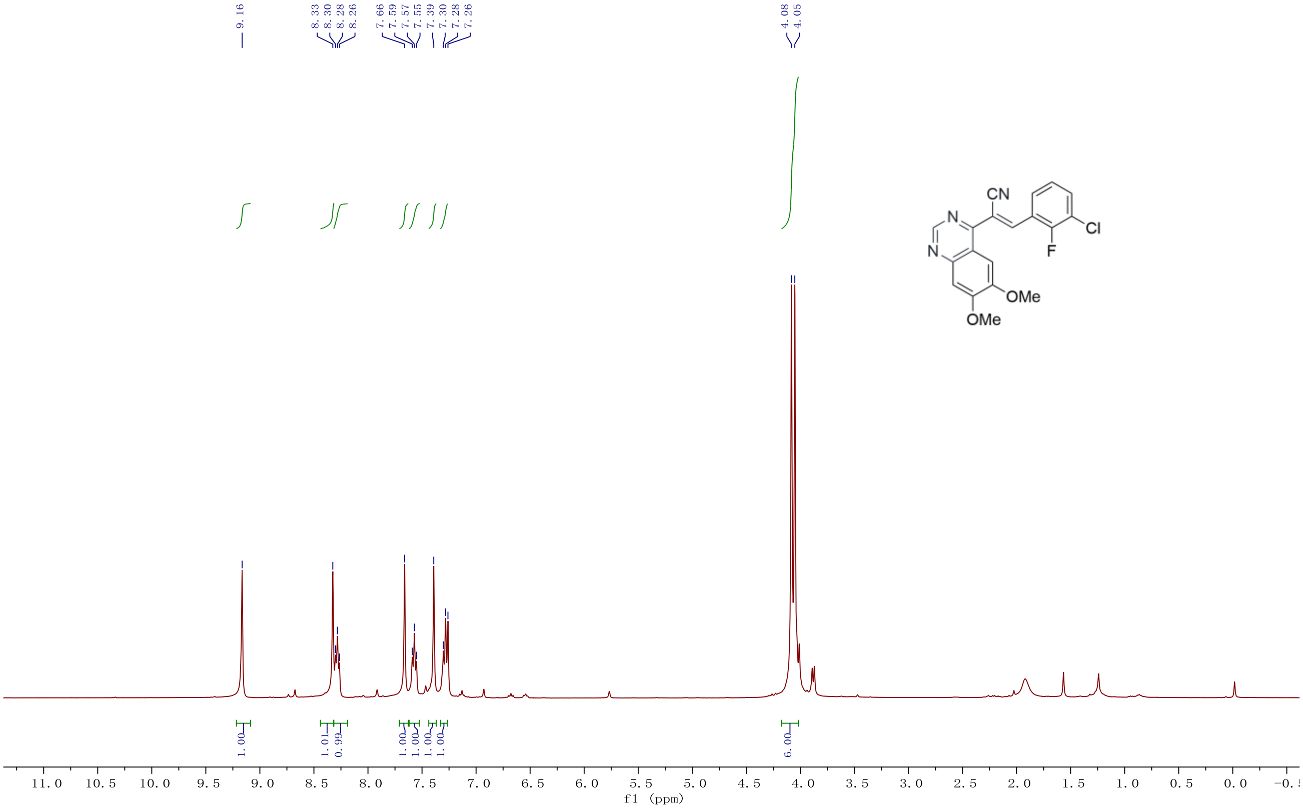

Supplementary Figure 172. ^{13}C NMR spectrum of compound **1bh** (100 MHz, $CDCl_3$)

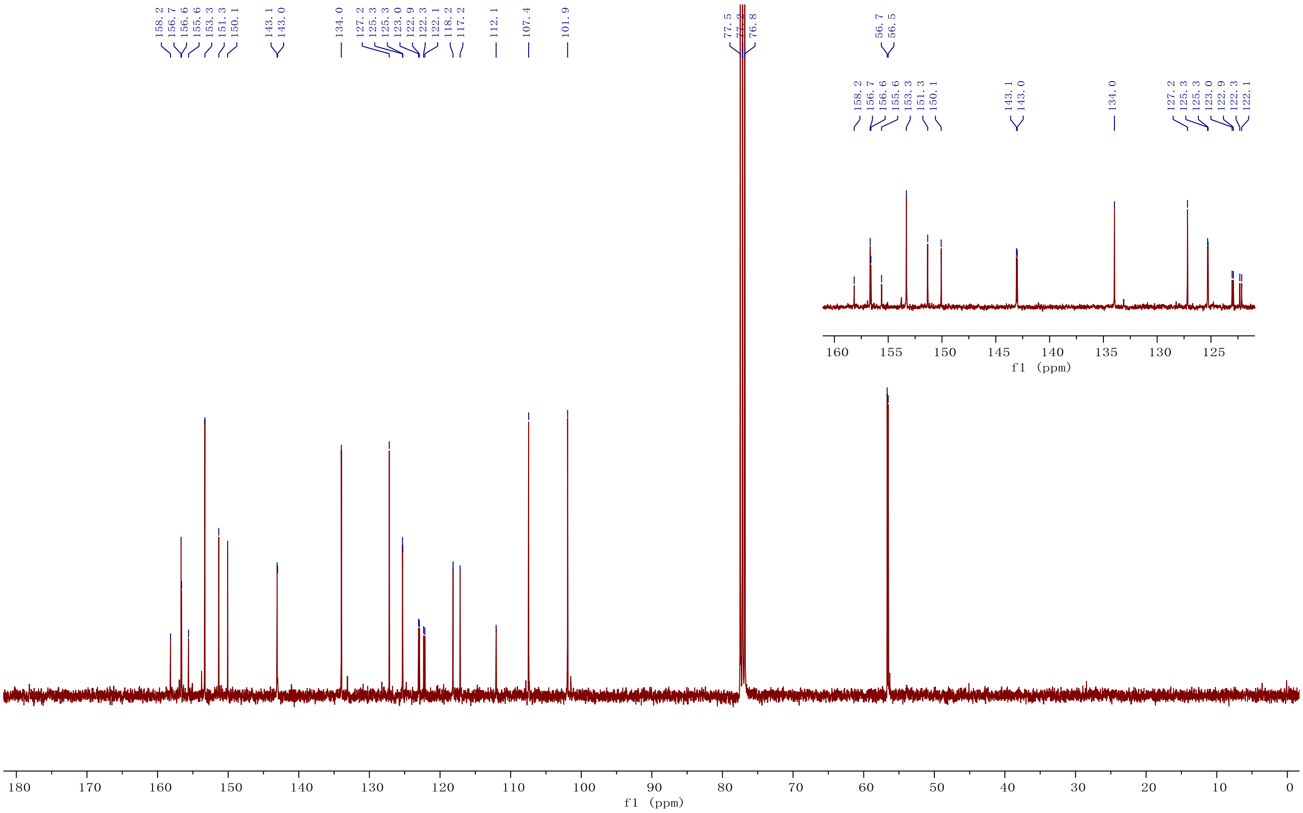

Supplementary Figure 173. ^{19}F NMR spectrum of compound **1bh** (376 MHz, $CDCl_3$)

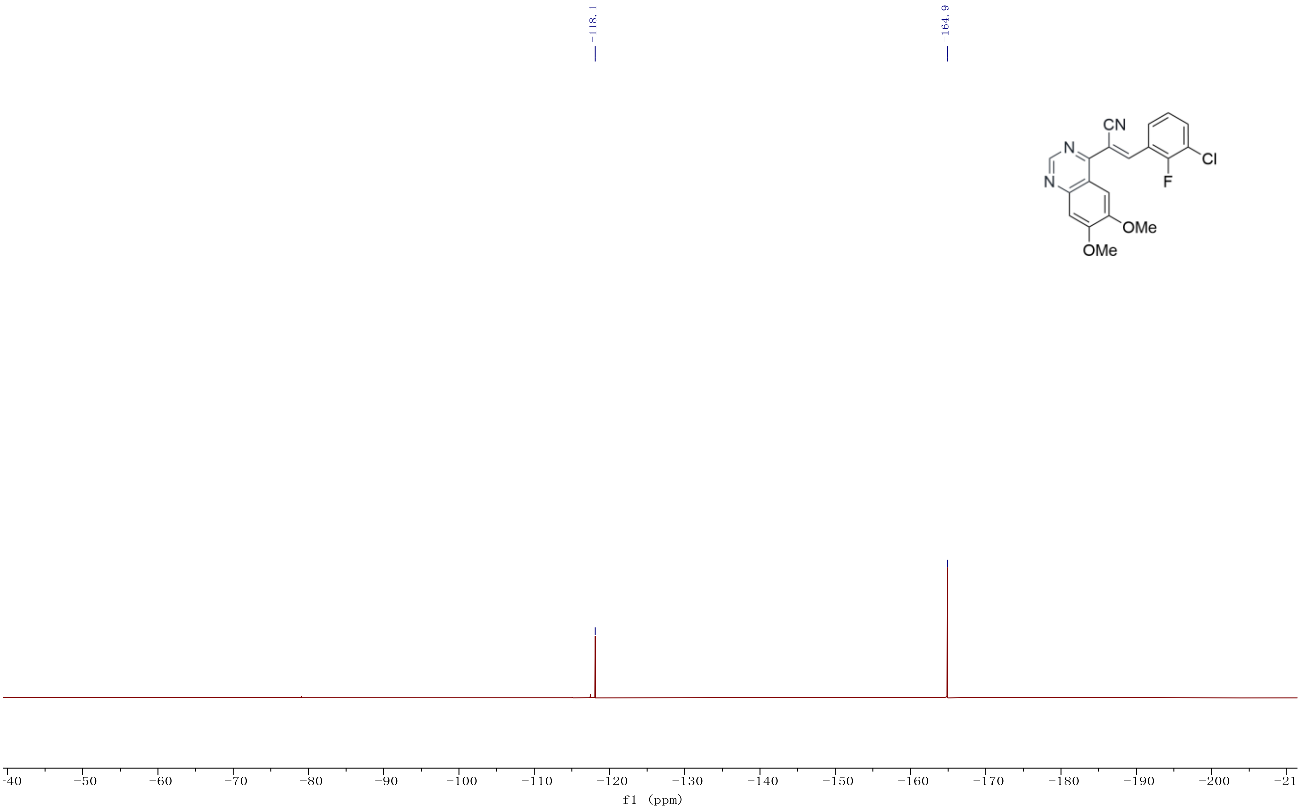

Supplementary Figure 174. ^{1}H NMR spectrum of compound **1bi** (400 MHz, $CDCl_3$)

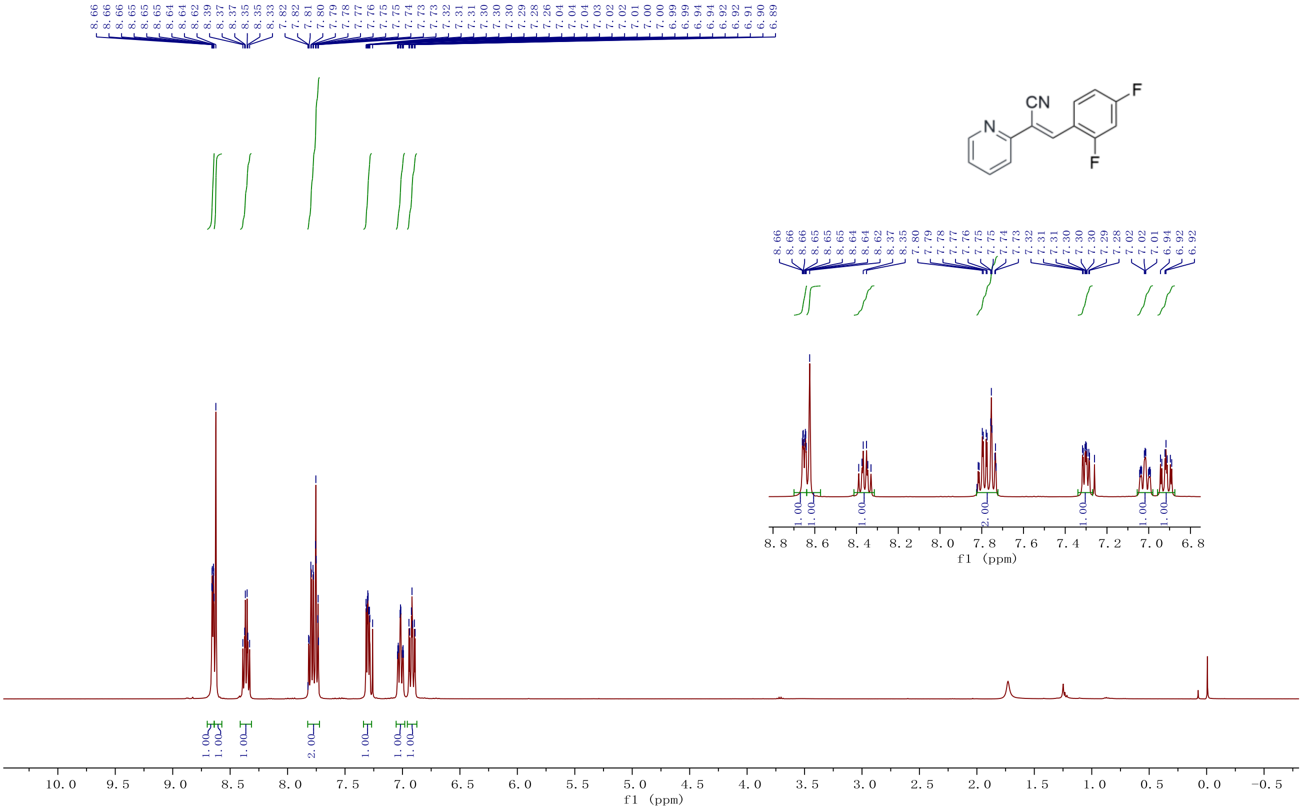

Supplementary Figure 175. ^{13}C NMR spectrum of compound **1bi** (100 MHz, $CDCl_3$)

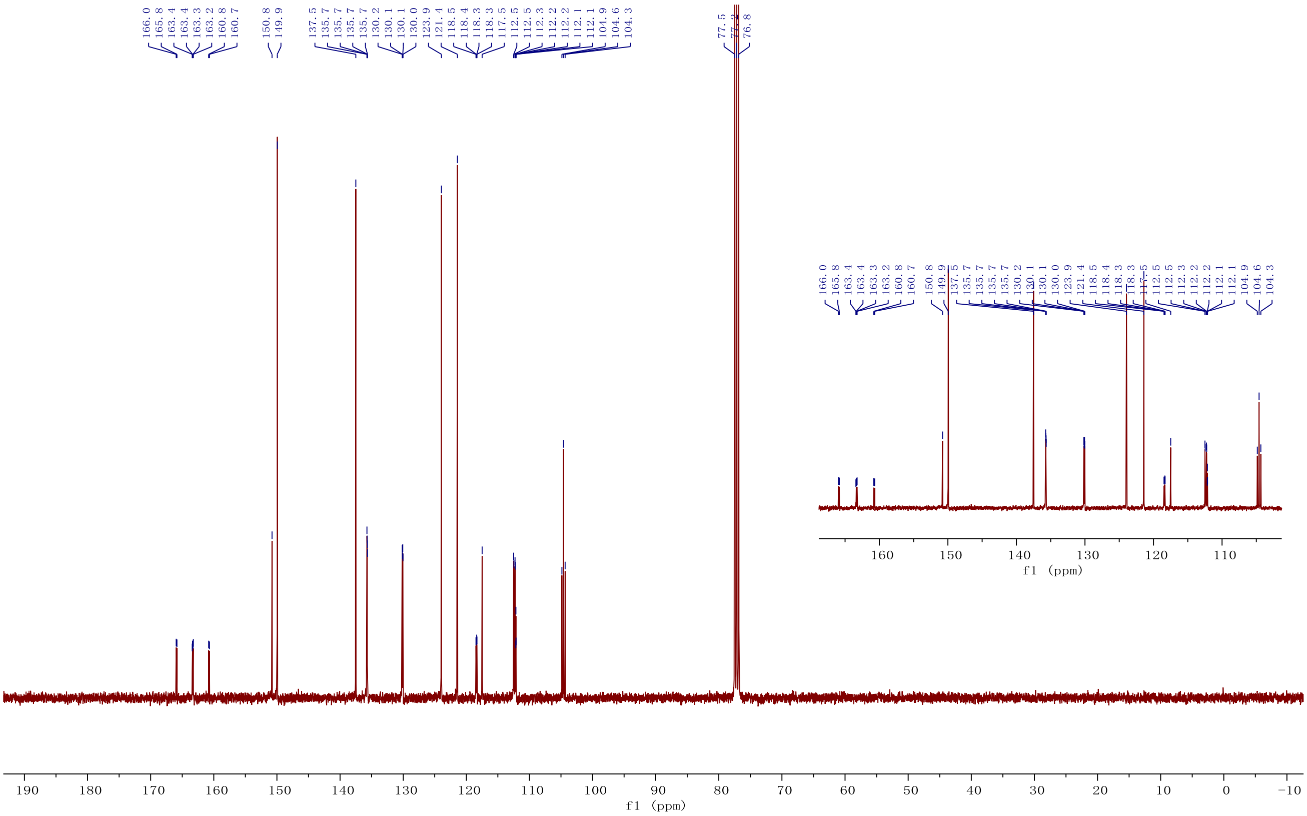

Supplementary Figure 176. ^{19}F NMR spectrum of compound **1bi** (376 MHz, $CDCl_3$)

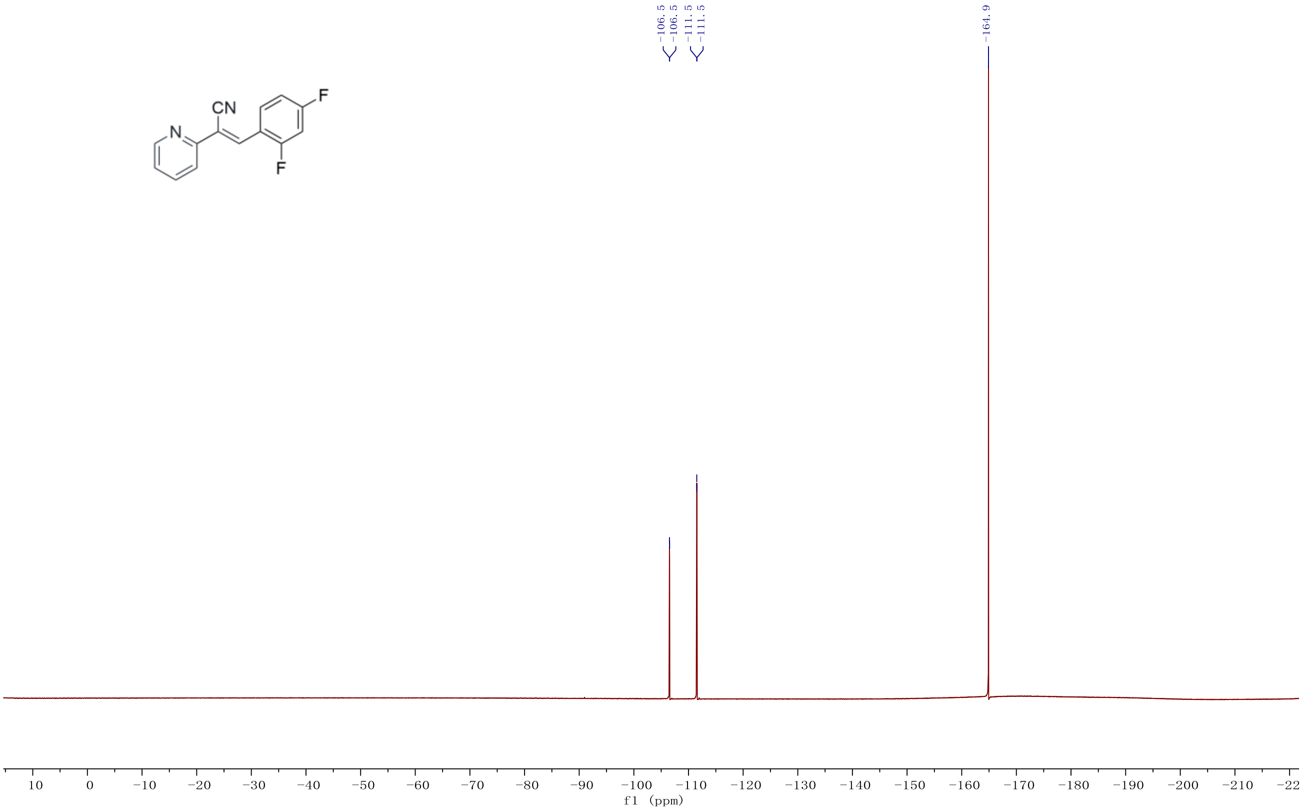

Supplementary Figure 177. ^{1}H NMR spectrum of compound **1bj** (600 MHz, $CDCl_3$)

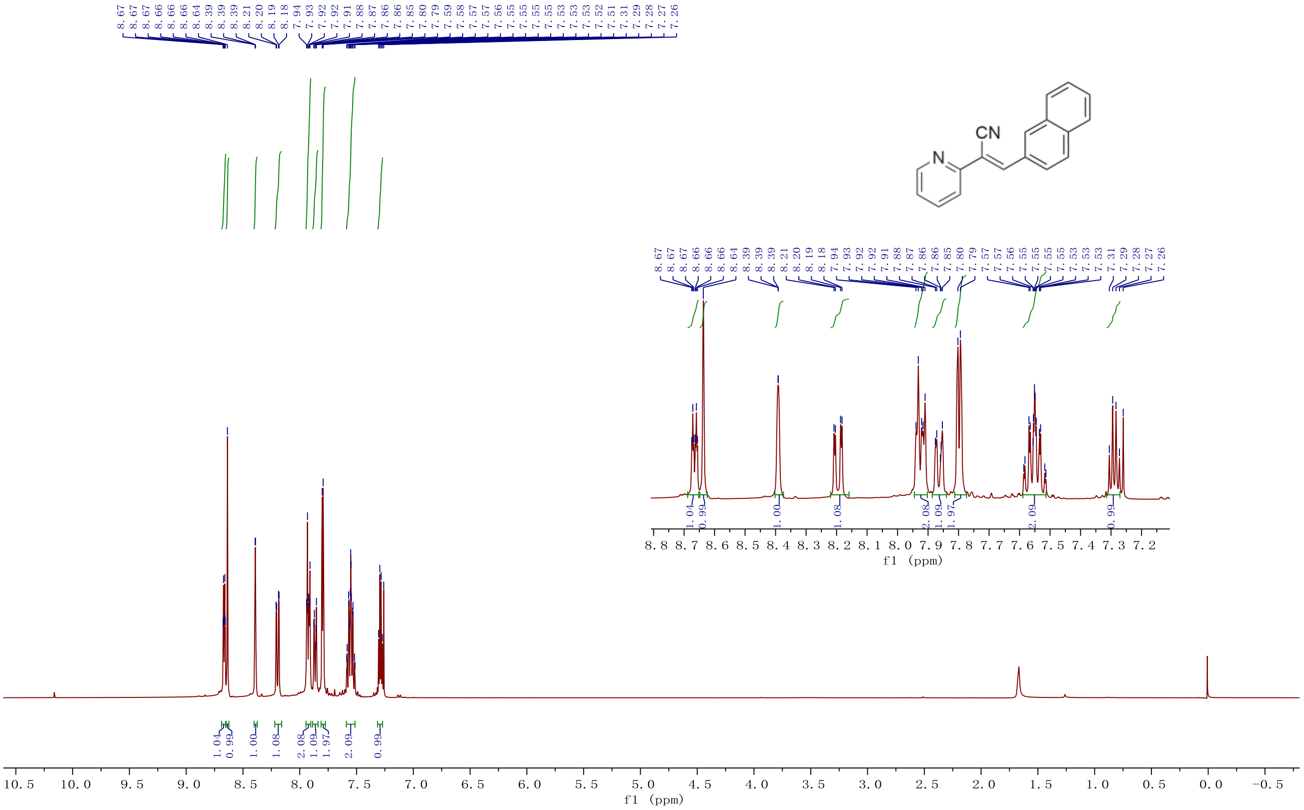

Supplementary Figure 178. ^{13}C NMR spectrum of compound **1bj** (150 MHz, $CDCl_3$)

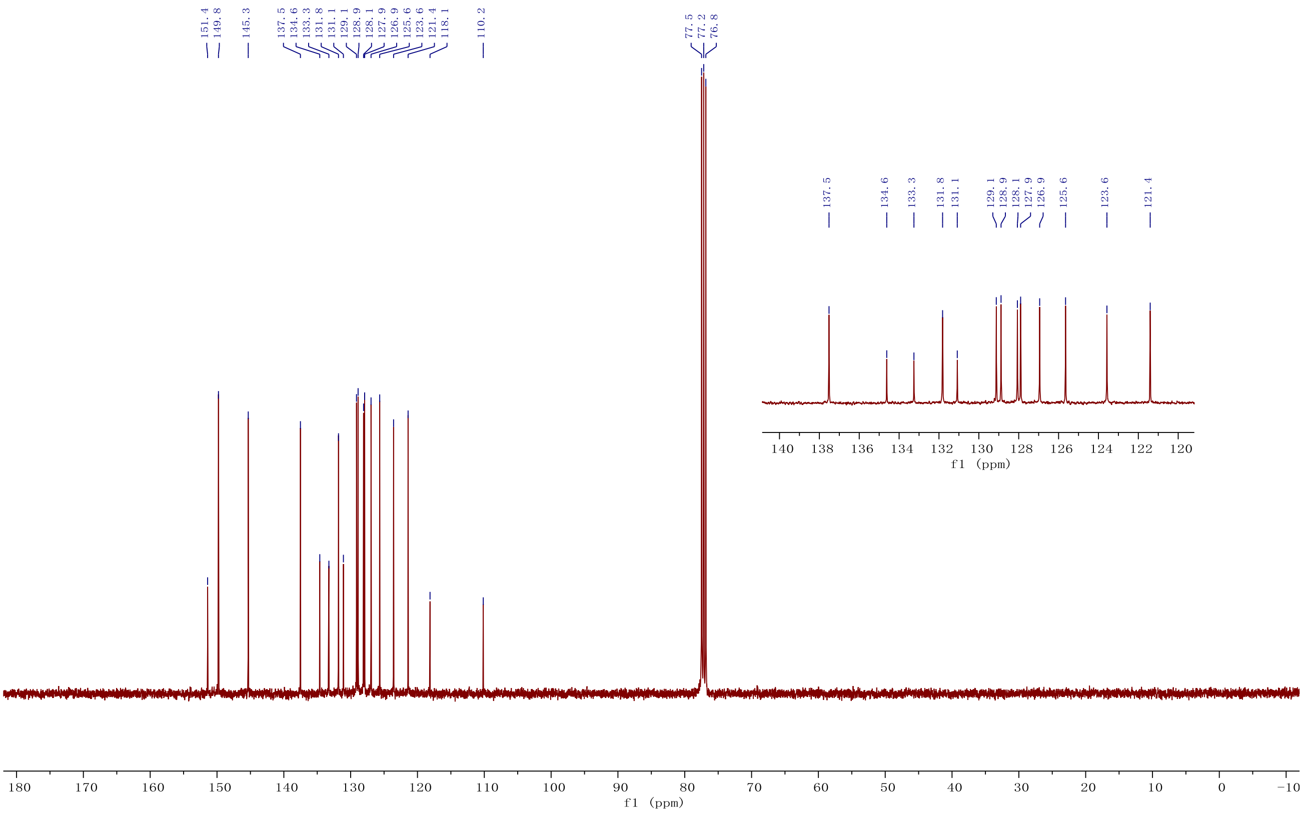

Supplementary Figure 179. ^{1}H NMR spectrum of compound **1bk** (400 MHz, $CDCl_3$)

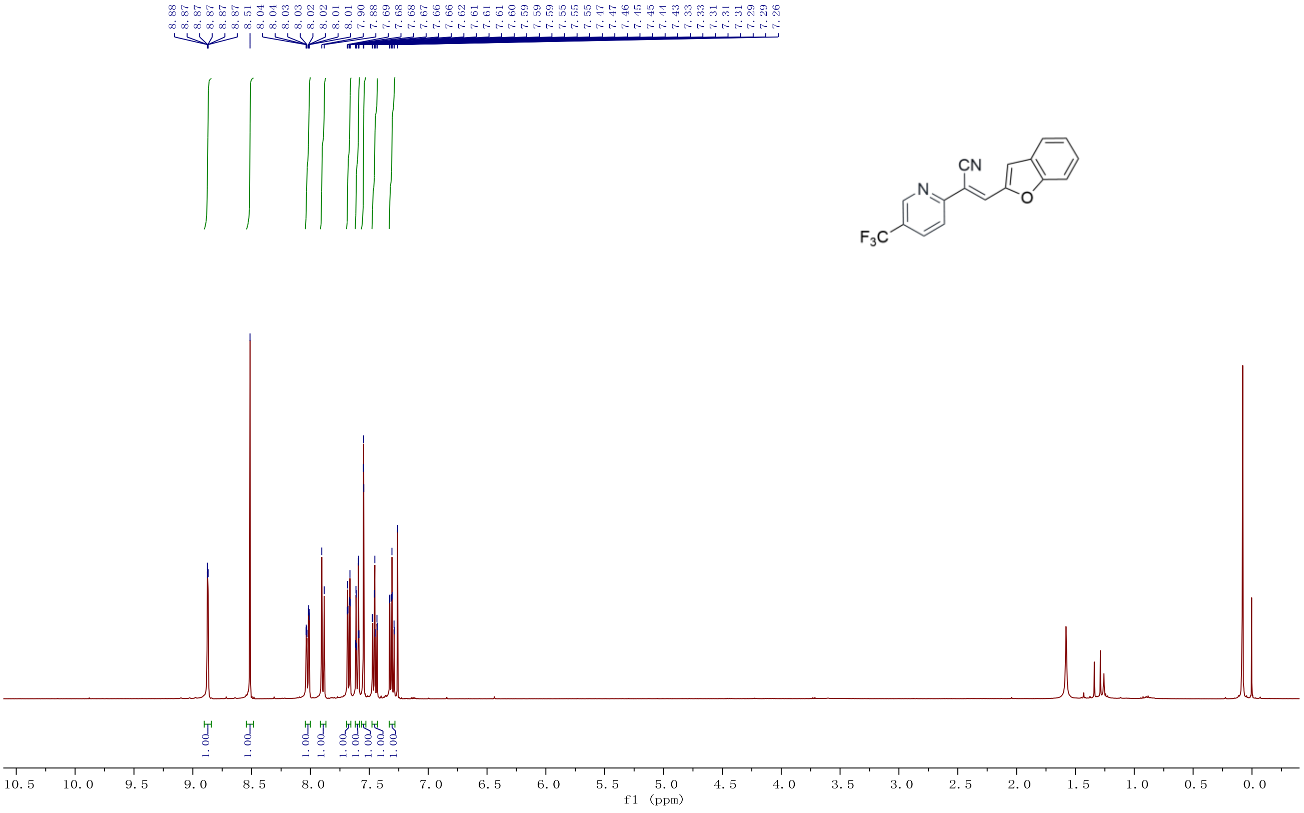

Supplementary Figure 180. ^{13}C NMR spectrum of compound **1bk** (100 MHz, $CDCl_3$)

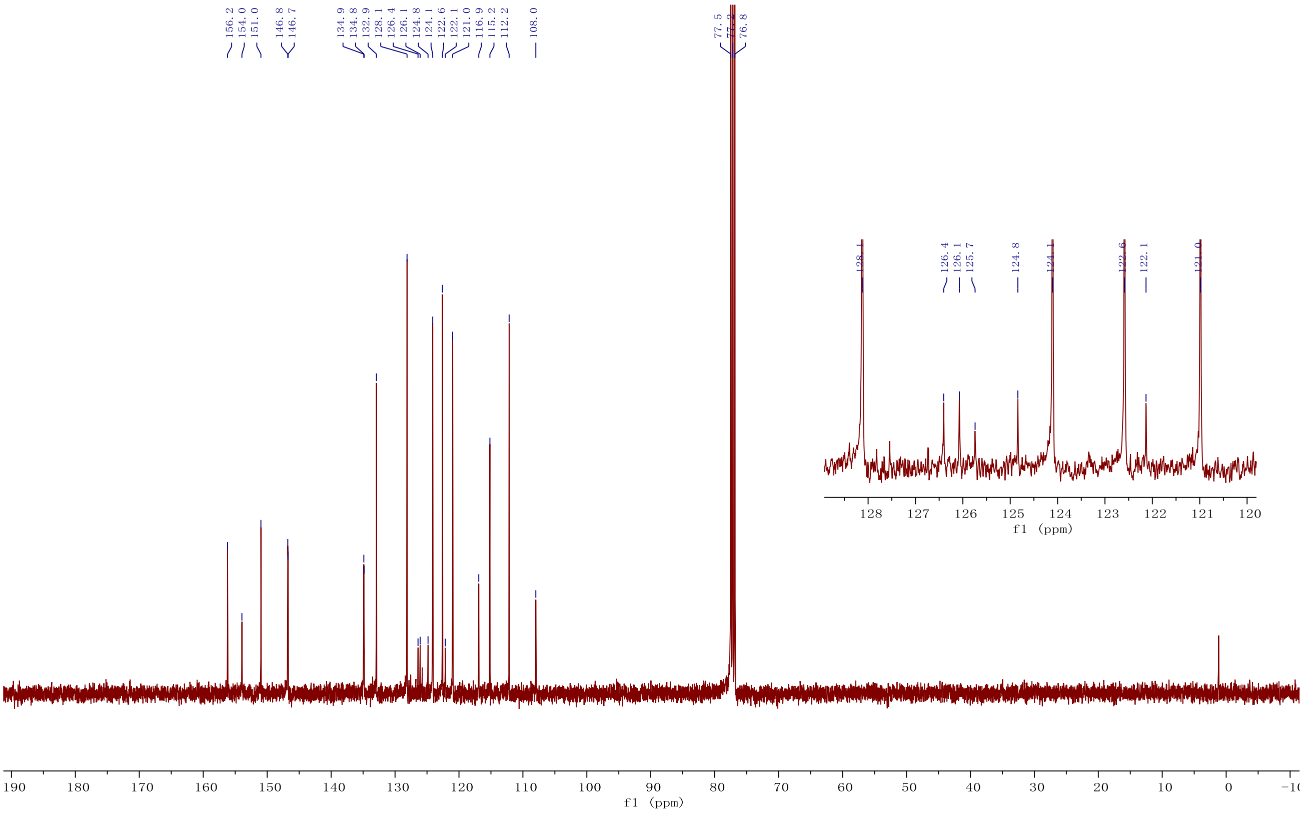

Supplementary Figure 181. ^{19}F NMR spectrum of compound **1bk** (376 MHz, $CDCl_3$)

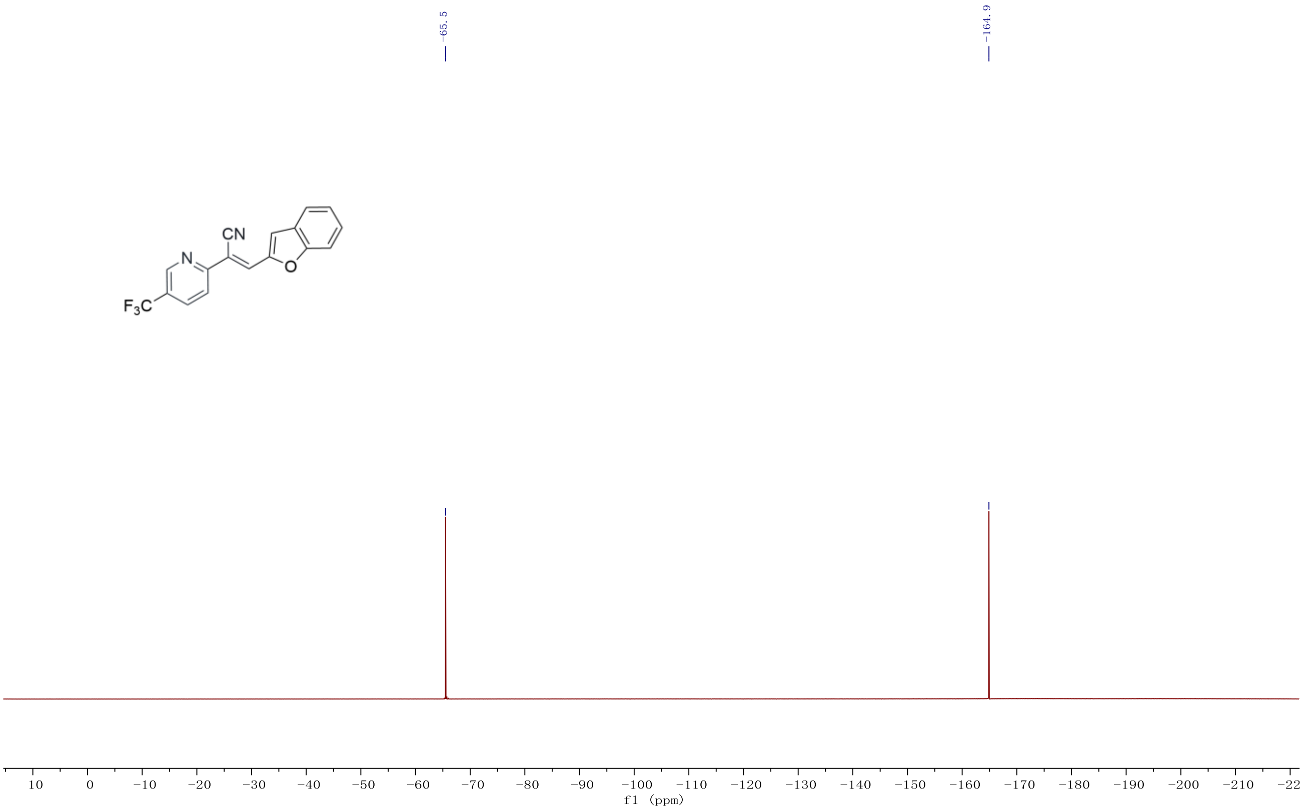

Supplementary Figure 182. ^{1}H NMR spectrum of compound **1bl** (400 MHz, $CDCl_3$)

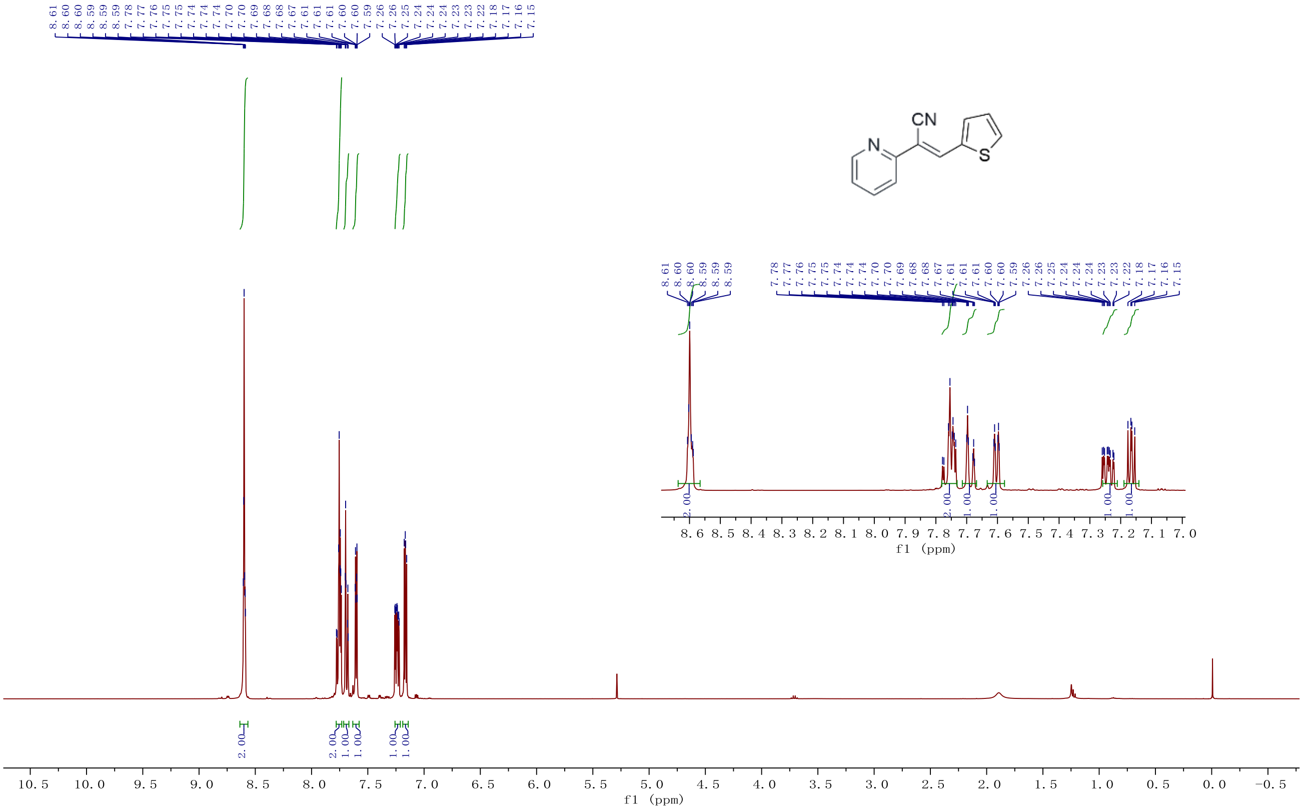

Supplementary Figure 183. ^{13}C NMR spectrum of compound **1bl** (100 MHz, $CDCl_3$)

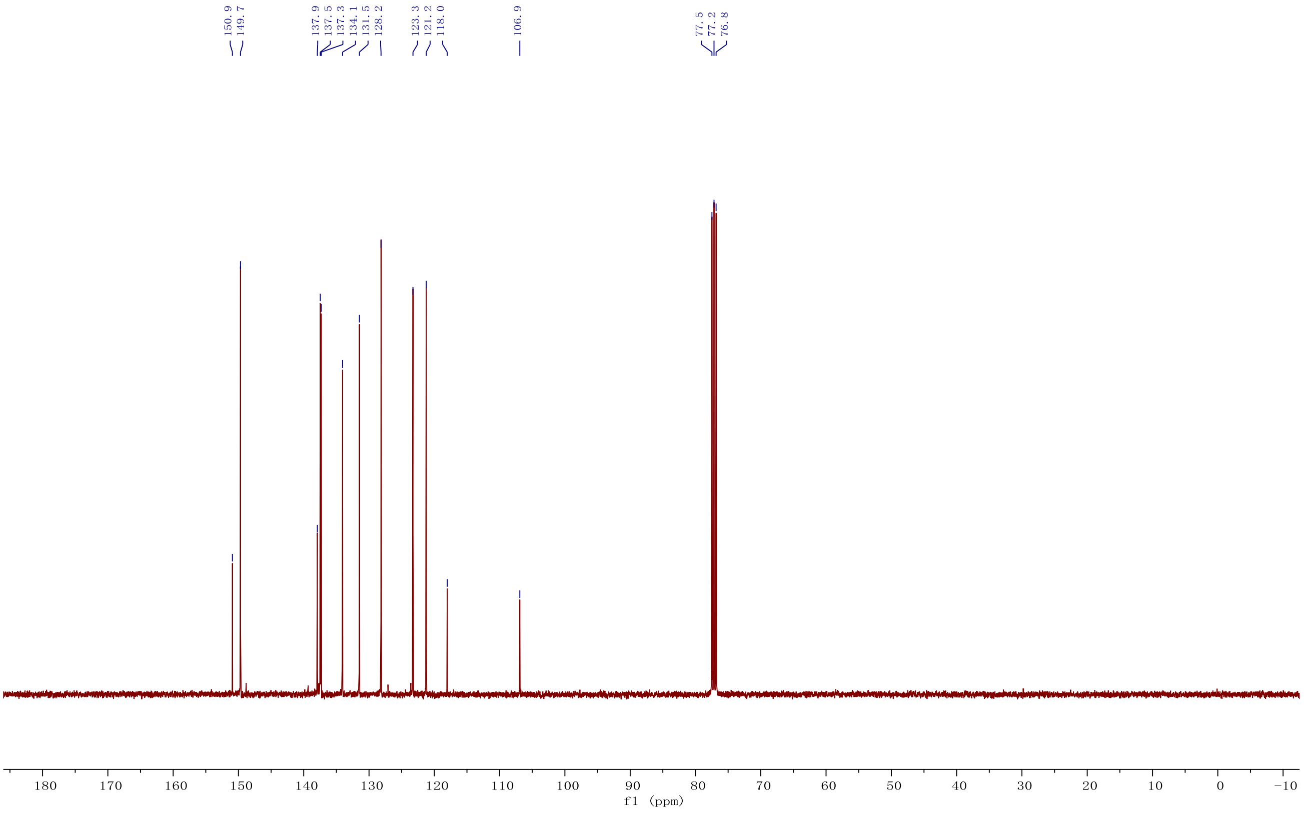

Supplementary Figure 184. ^{1}H NMR spectrum of compound **1ca** (400 MHz, $CDCl_3$)

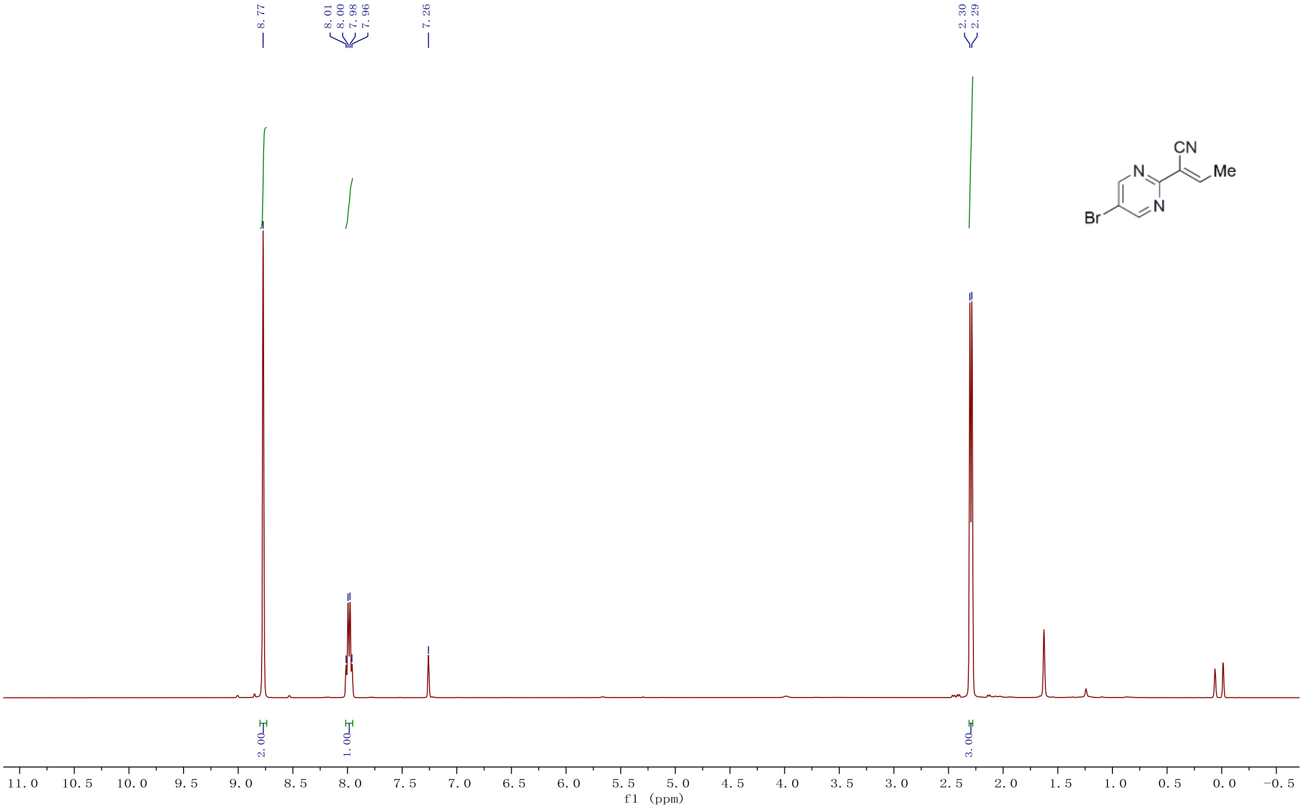

Supplementary Figure 185. ^{13}C NMR spectrum of compound **1ca** (100 MHz, $CDCl_3$)

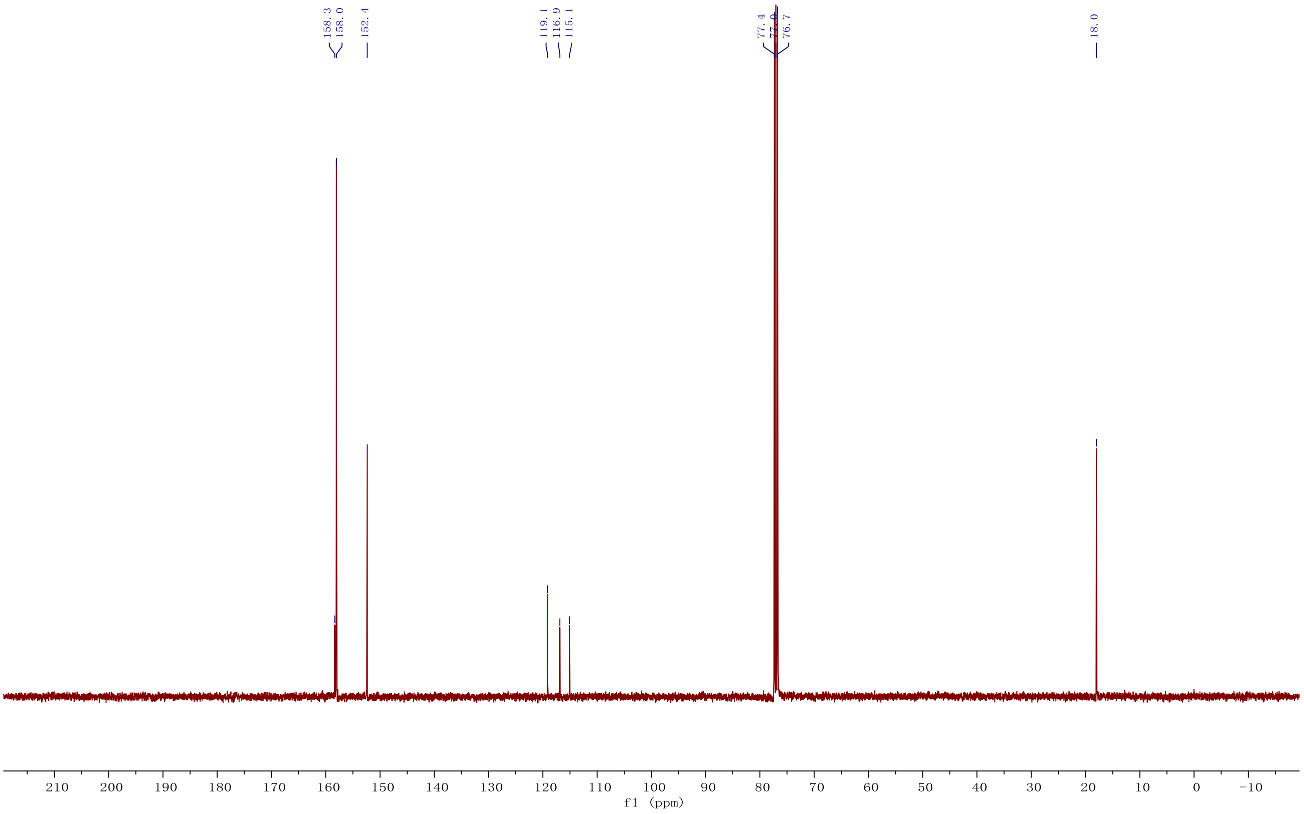

Supplementary Figure 186. ^{1}H NMR spectrum of compound **1cb** (400 MHz, $CDCl_3$)

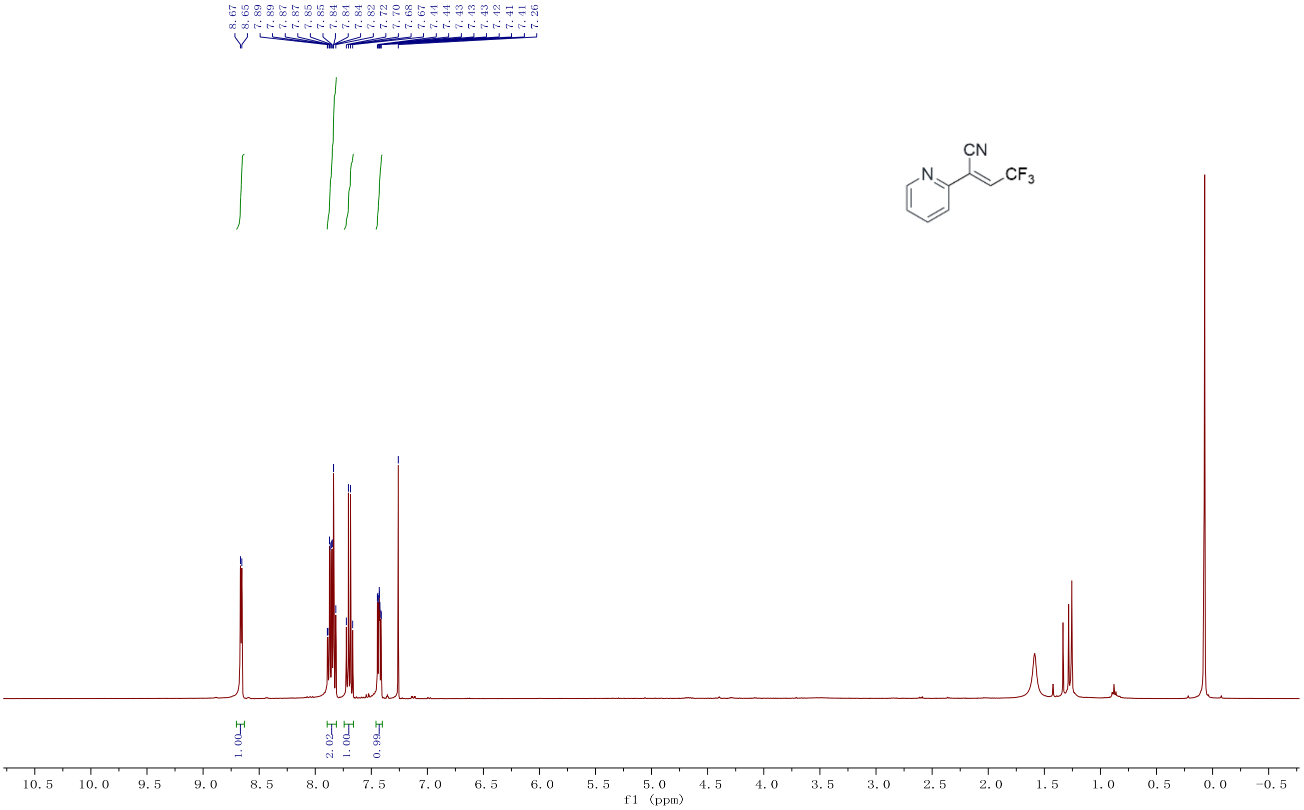

Supplementary Figure 187. ^{13}C NMR spectrum of compound **1cb** (100 MHz, $CDCl_3$)

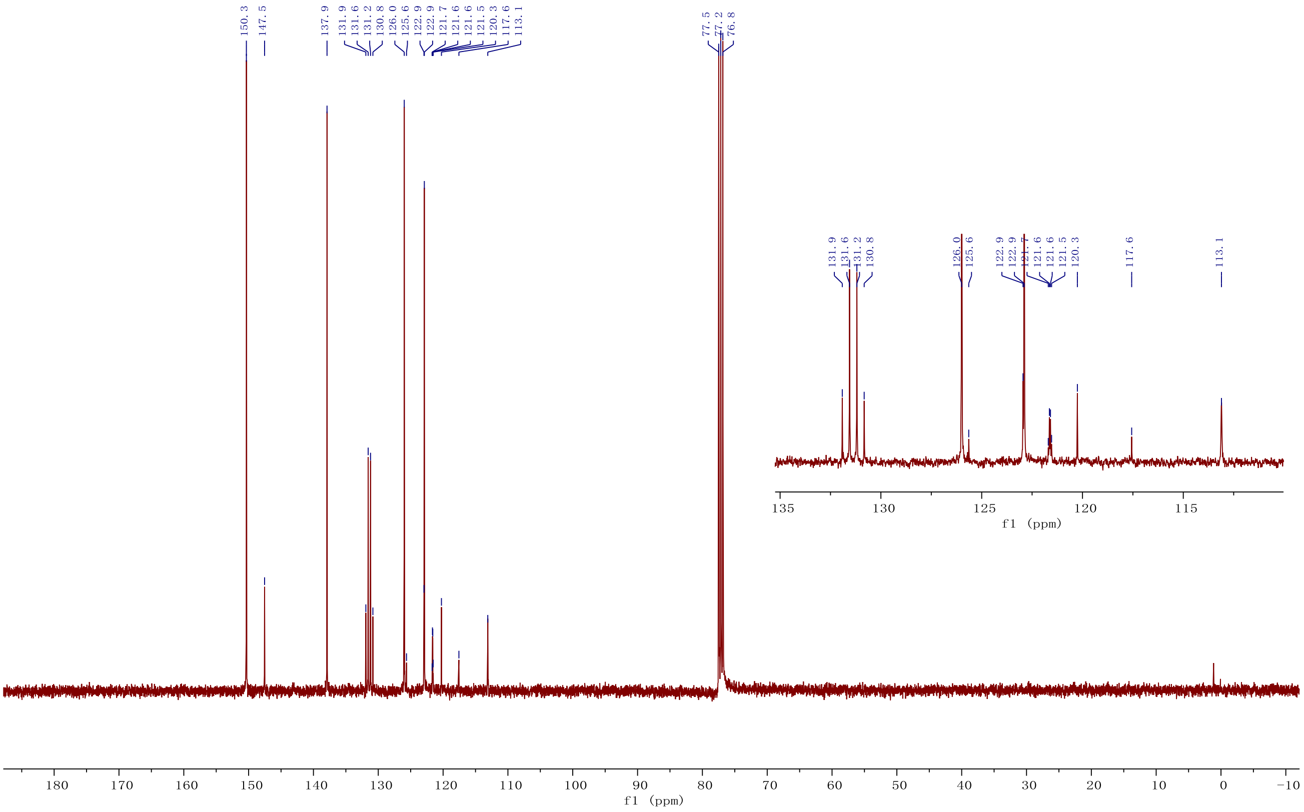

Supplementary Figure 188. ^{19}F NMR spectrum of compound **1cb** (376 MHz, $CDCl_3$)

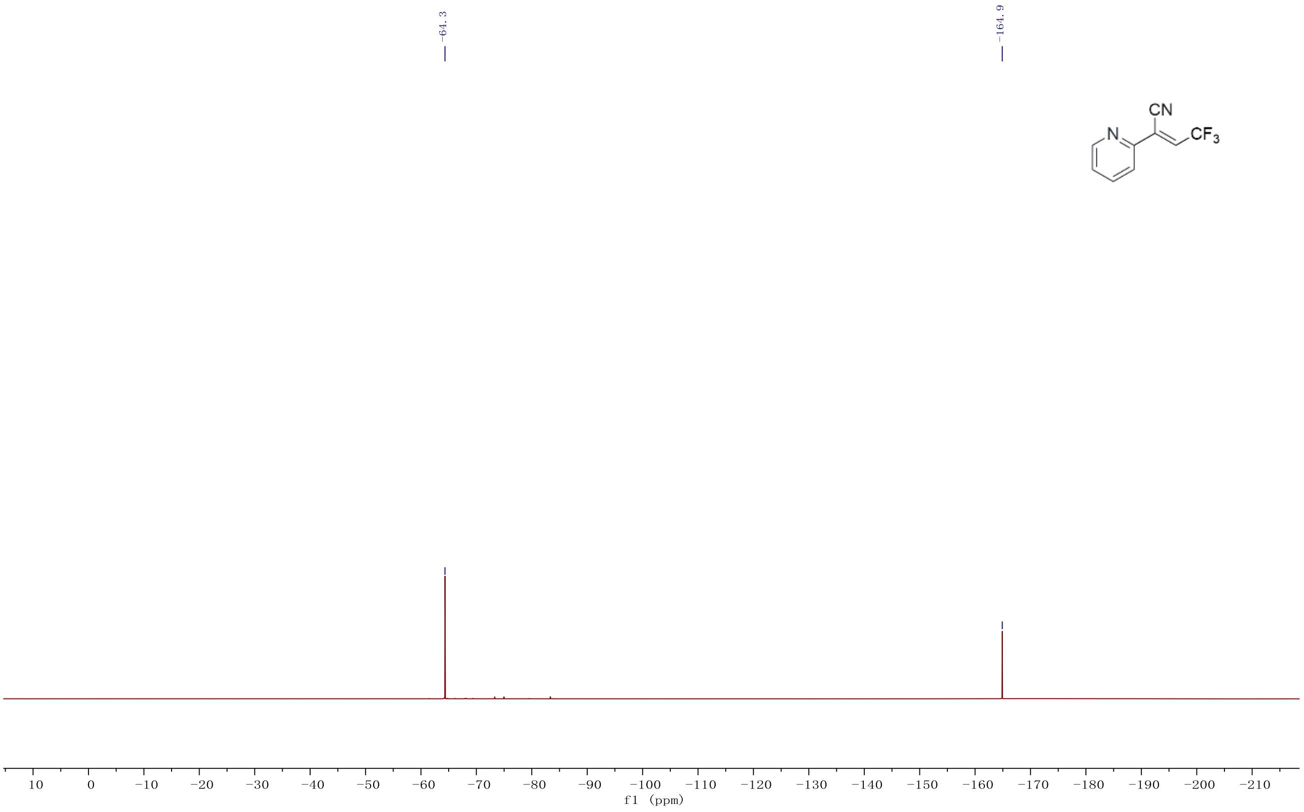

Supplementary Figure 189. ^{1}H NMR spectrum of compound **1cc** (400 MHz, $CDCl_3$)

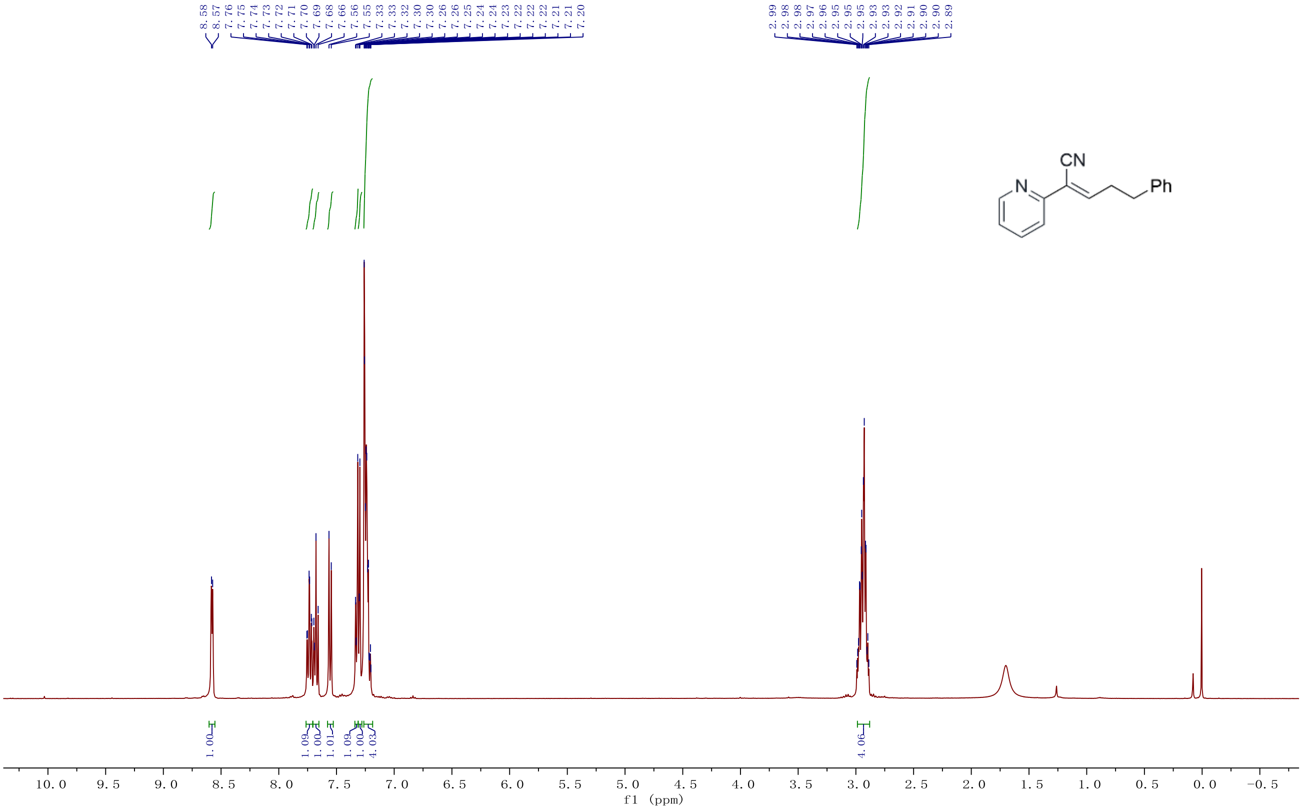

Supplementary Figure 190. ^{13}C NMR spectrum of compound **1cc** (100 MHz, $CDCl_3$)

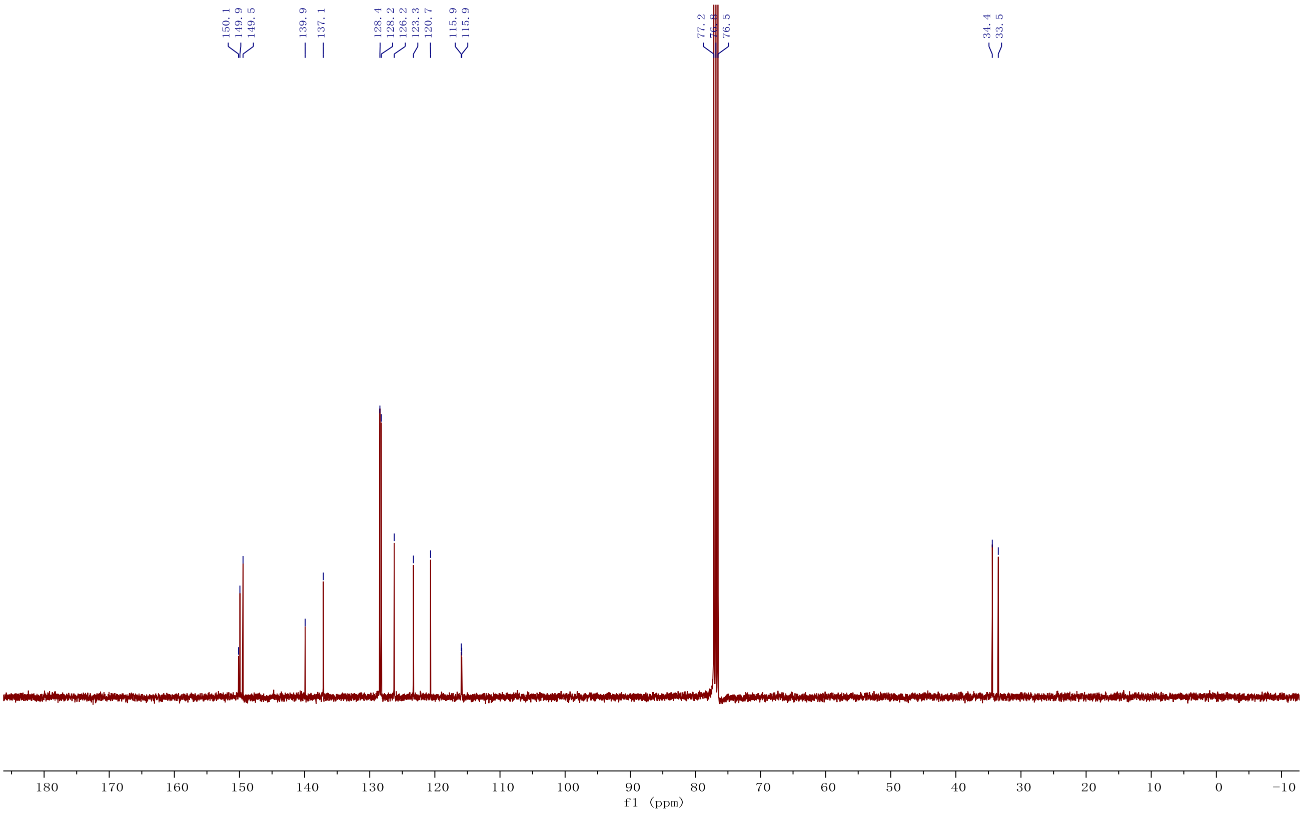

Supplementary Figure 191. ^{1}H NMR spectrum of compound **1cd** (400 MHz, $CDCl_3$)

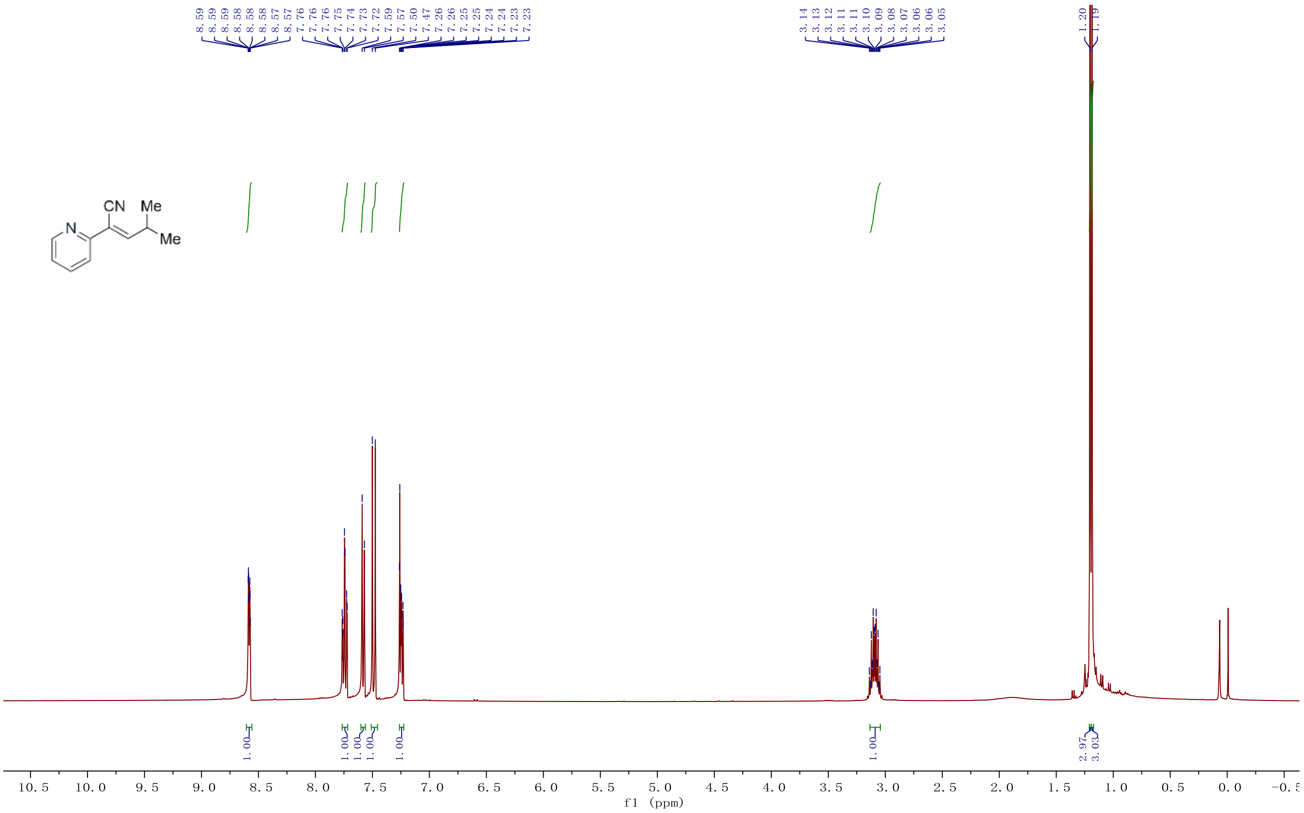

Supplementary Figure 192. ^{13}C NMR spectrum of compound **1cd** (100 MHz, $CDCl_3$)

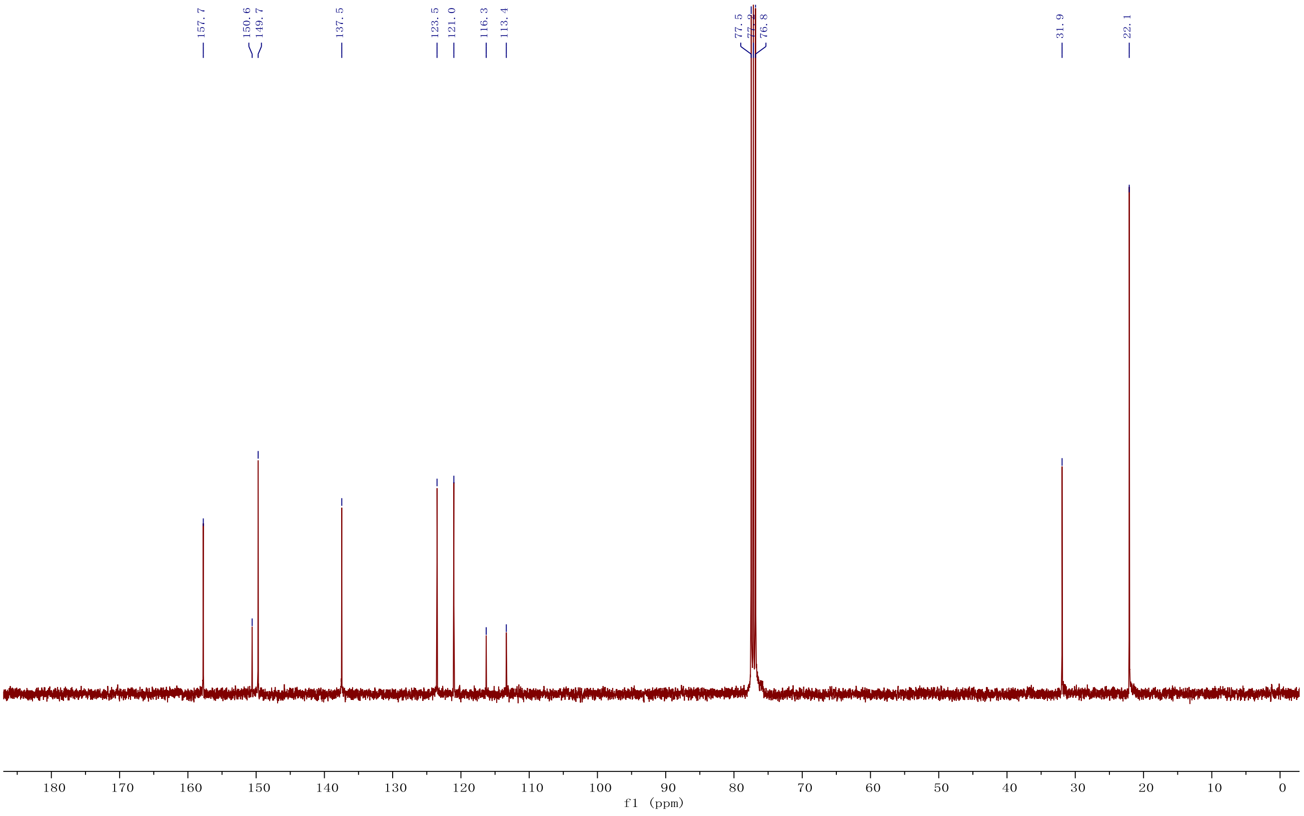

Supplementary Figure 193. ^{1}H NMR spectrum of compound **1ce** (400 MHz, $CDCl_3$)

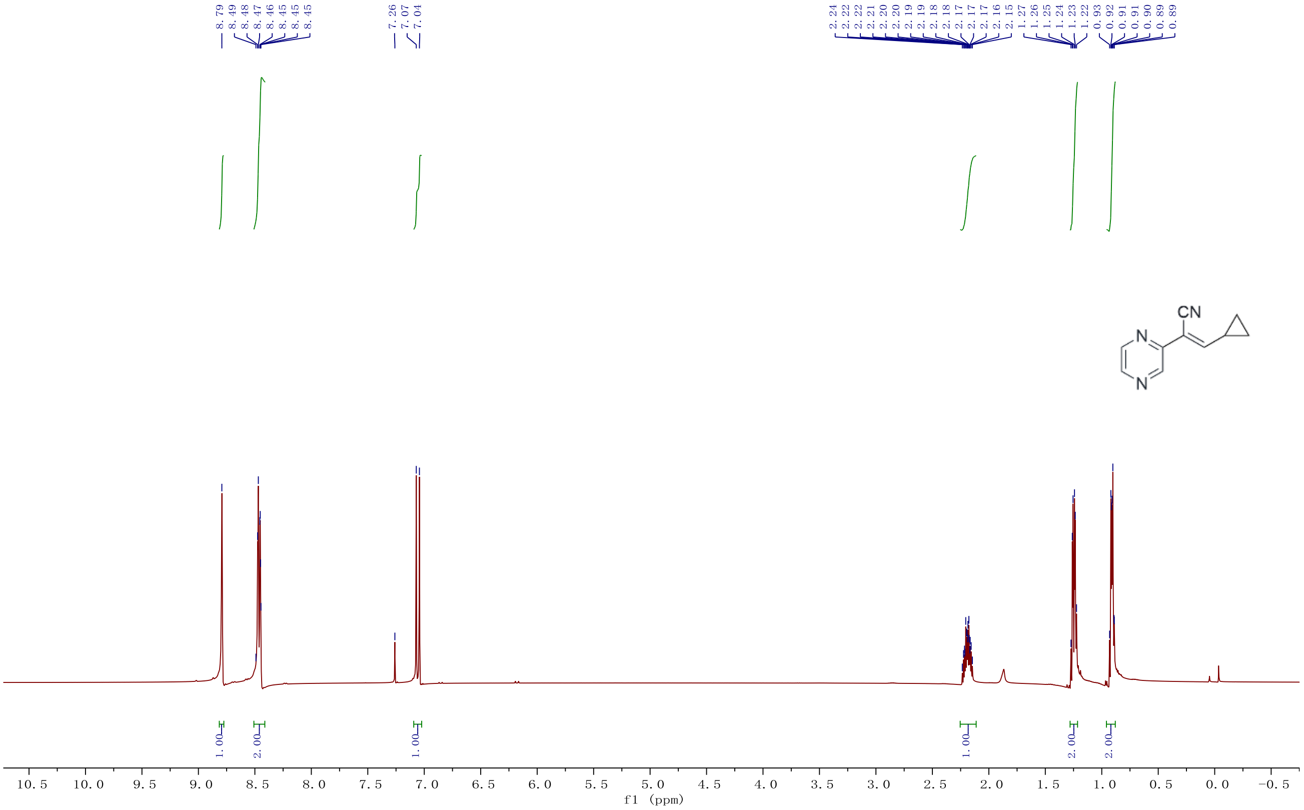

Supplementary Figure 194. ^{13}C NMR spectrum of compound **1ce** (100 MHz, $CDCl_3$)

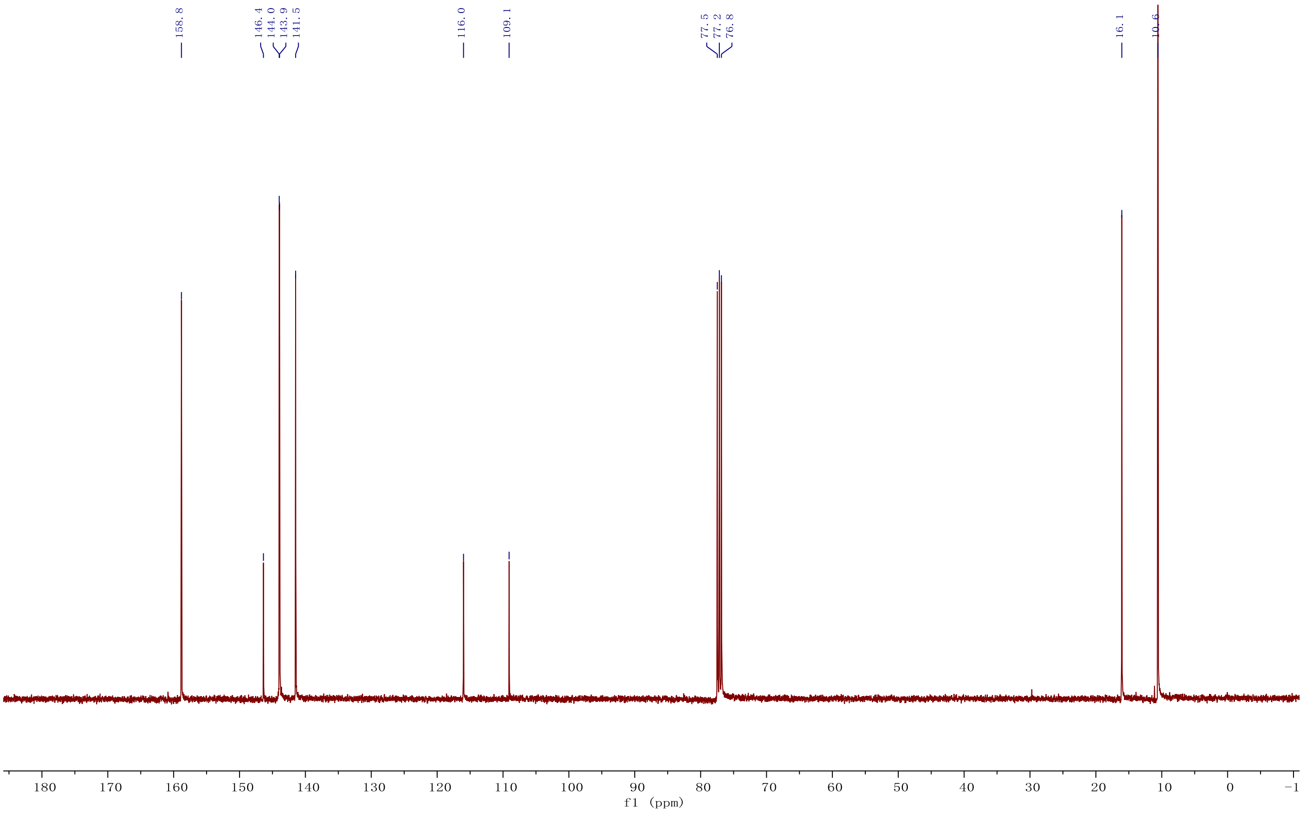

Supplementary Figure 195. ^{1}H NMR spectrum of compound **1cg** (400 MHz, $CDCl_3$)

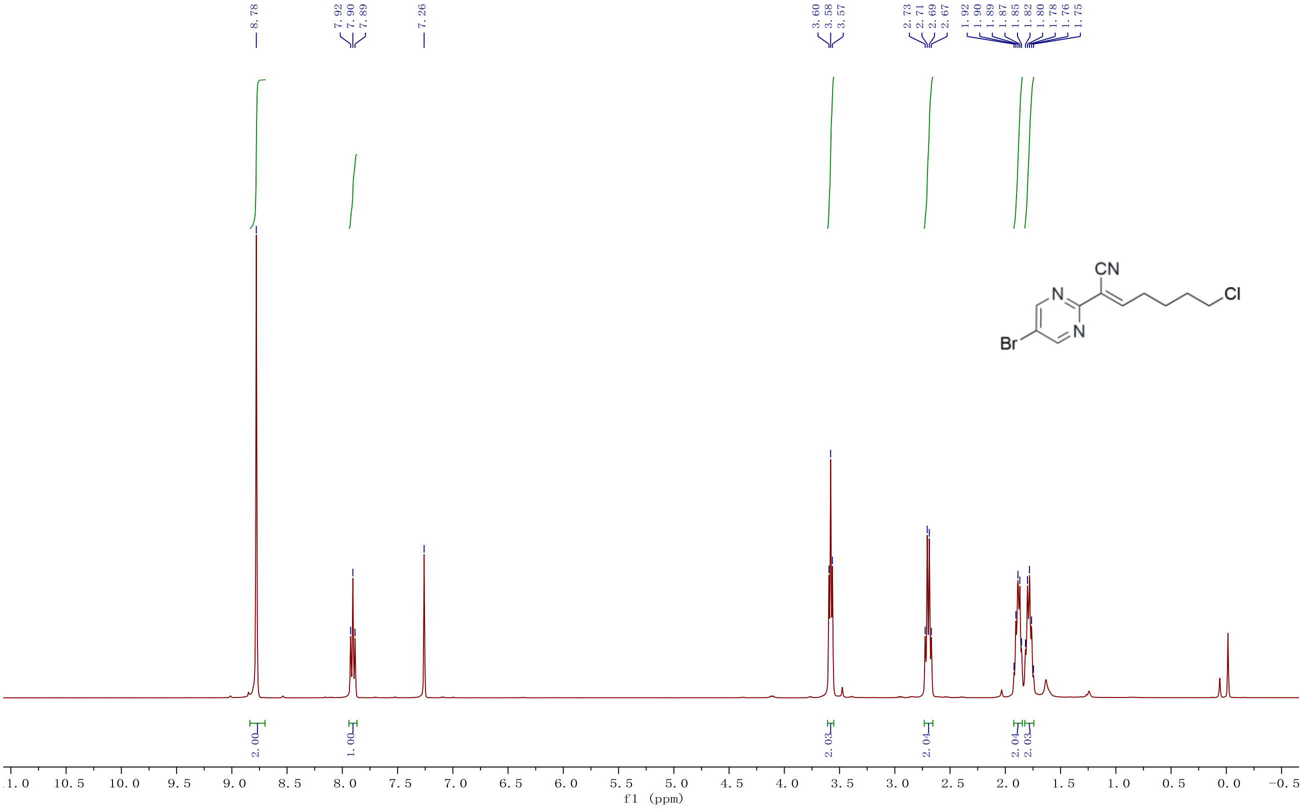

Supplementary Figure 196. ^{13}C NMR spectrum of compound **1cg** (100 MHz, $CDCl_3$)

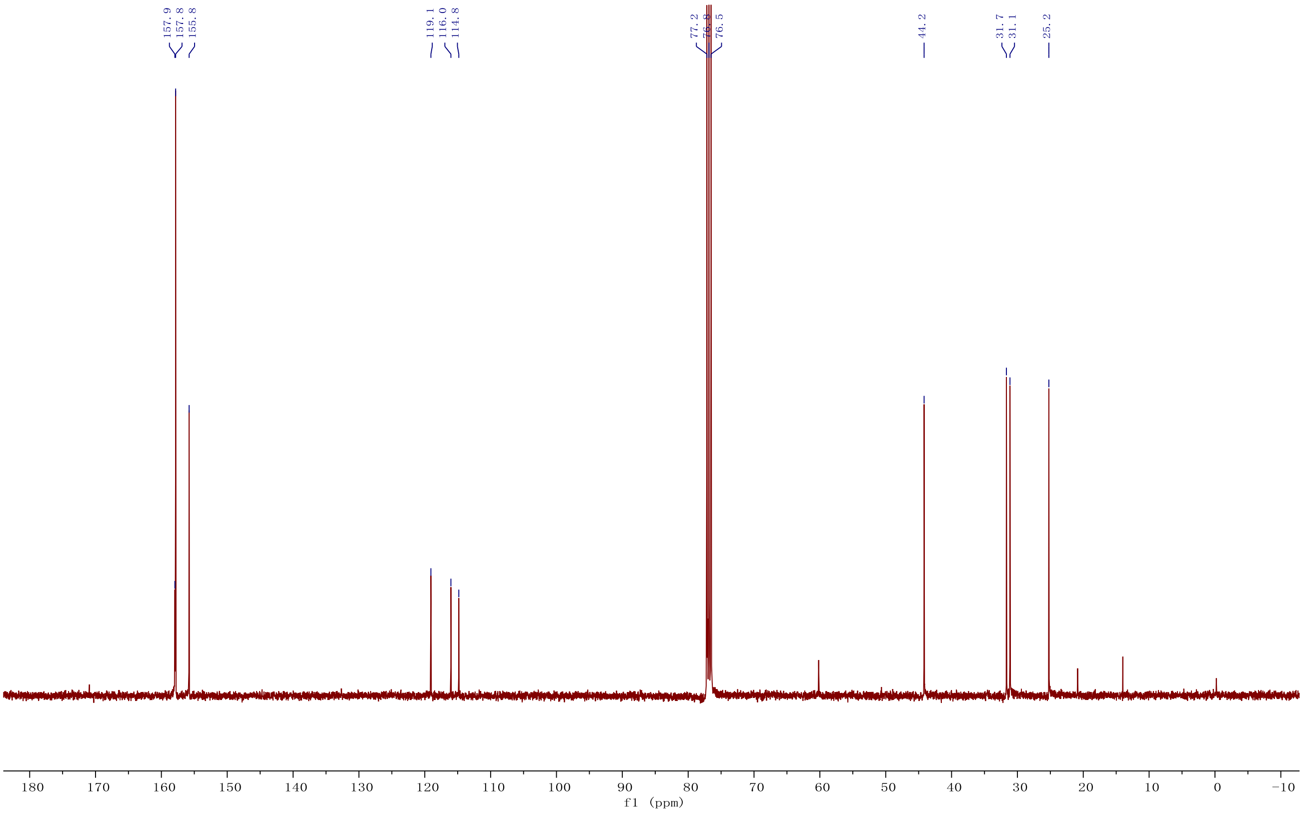

Supplementary Figure 197. ^{1}H NMR spectrum of compound **1ch** (400 MHz, $CDCl_3$)

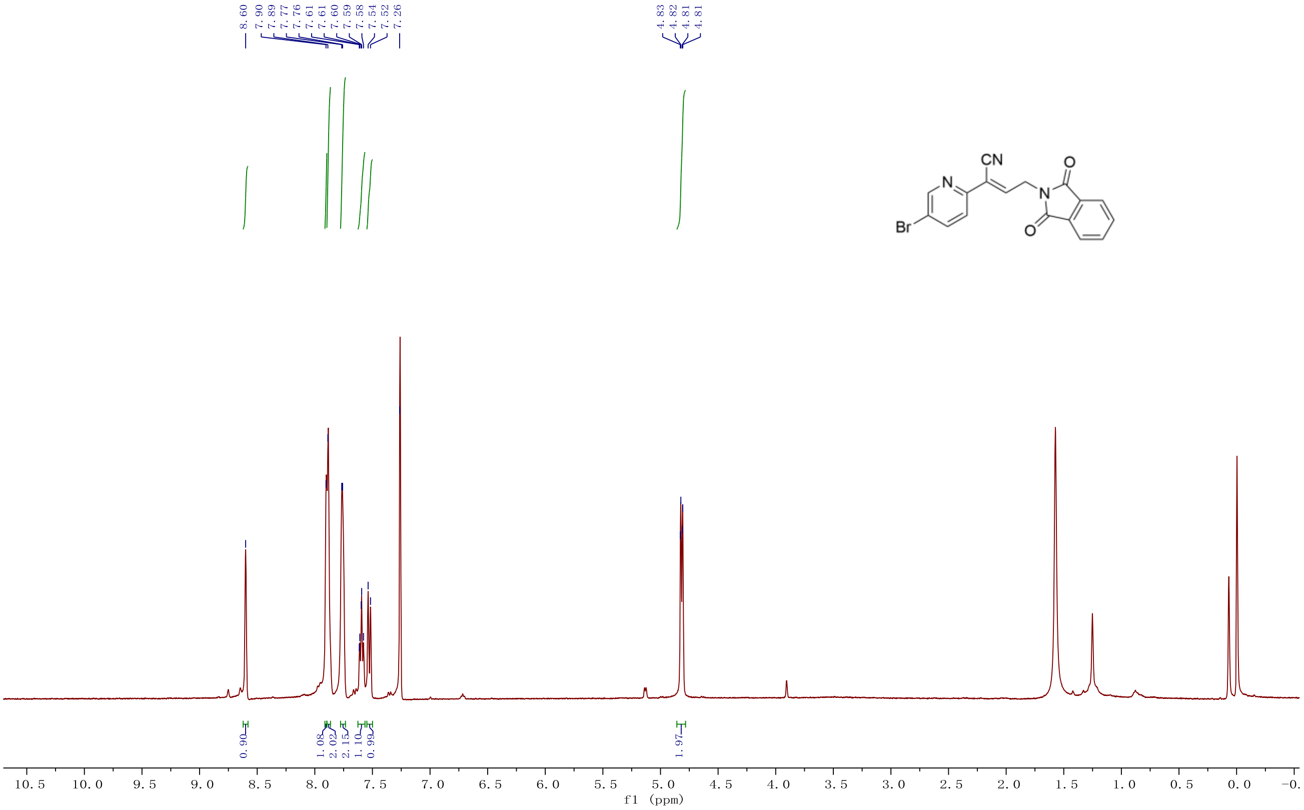

Supplementary Figure 198. ^{13}C NMR spectrum of compound **1ch** (100 MHz, $CDCl_3$)

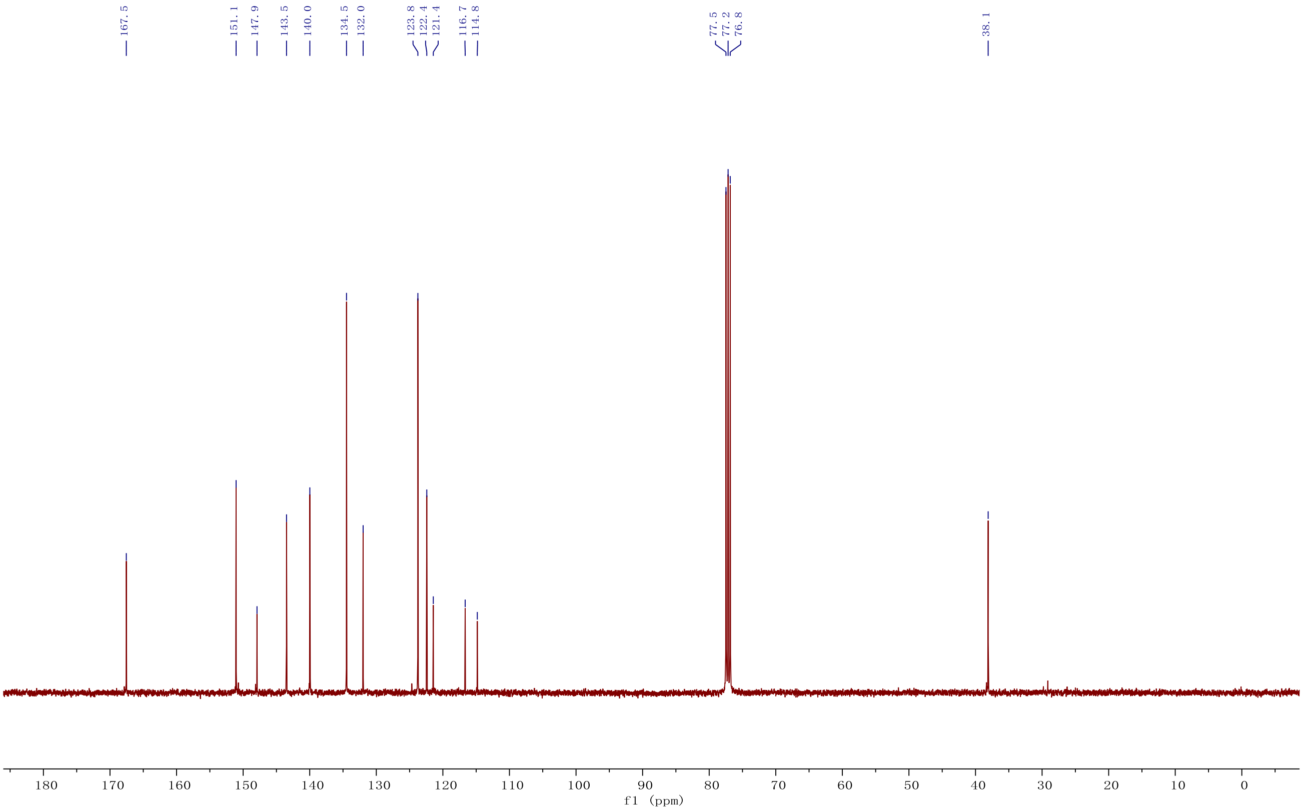

Supplementary Figure 199. ^{1}H NMR spectrum of compound **1ci** (400 MHz, $CDCl_3$)

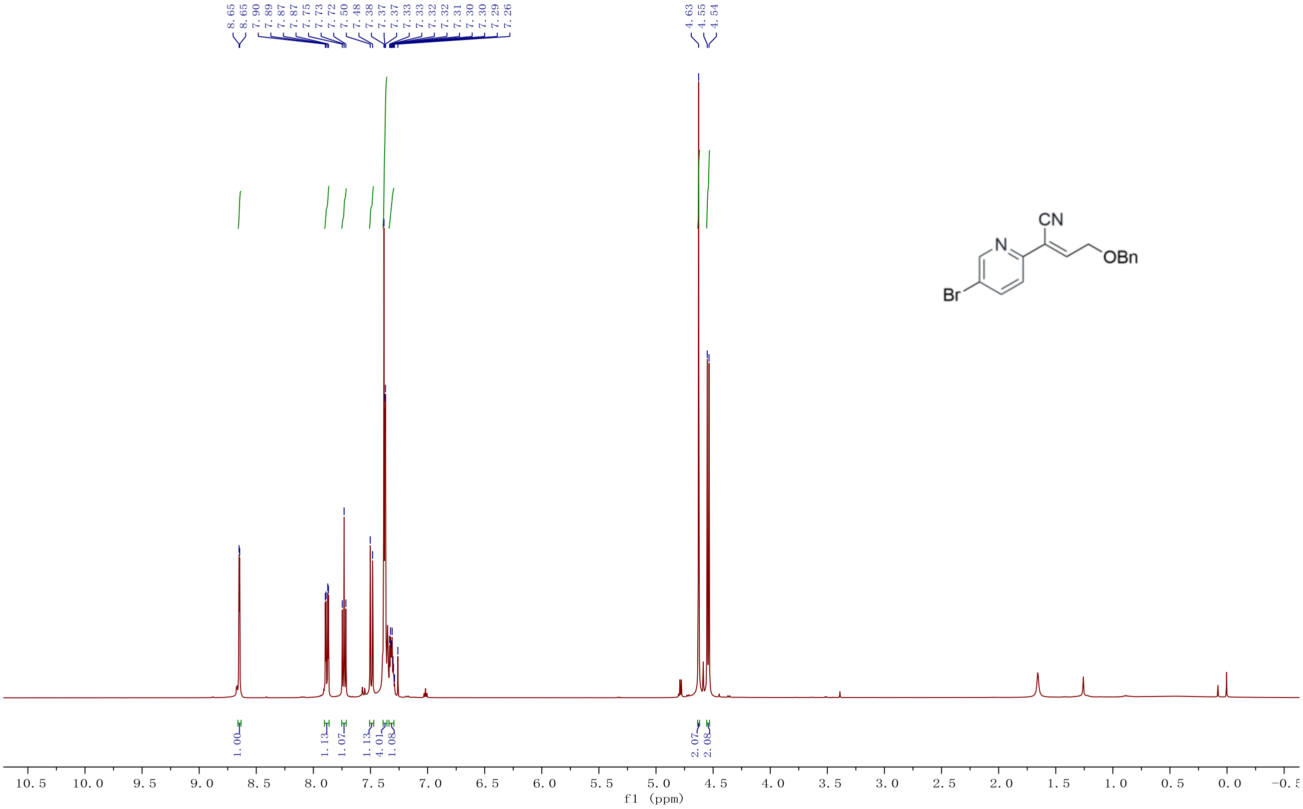

Supplementary Figure 200. ^{13}C NMR spectrum of compound **1ci** (100 MHz, $CDCl_3$)

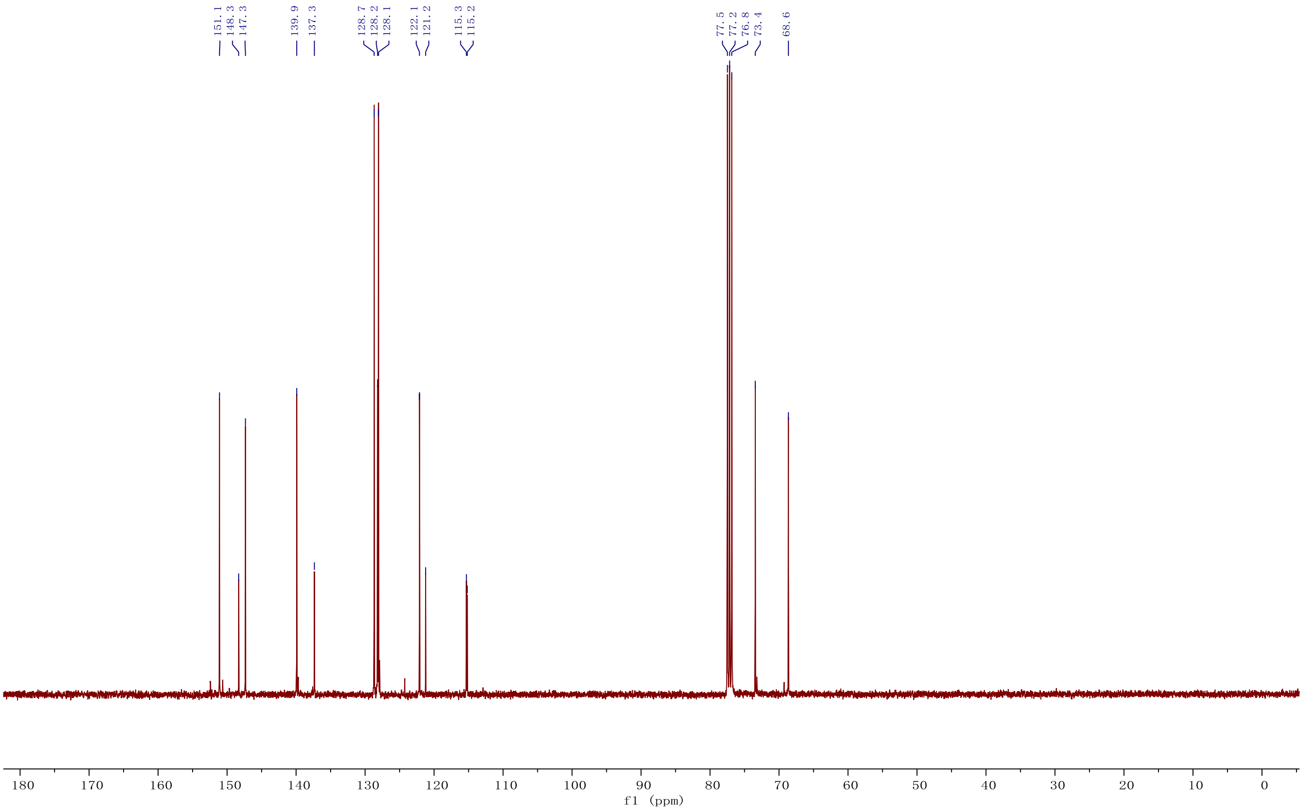

Supplementary Figure 201. ^{1}H NMR spectrum of compound **1cj** (400 MHz, $CDCl_3$)

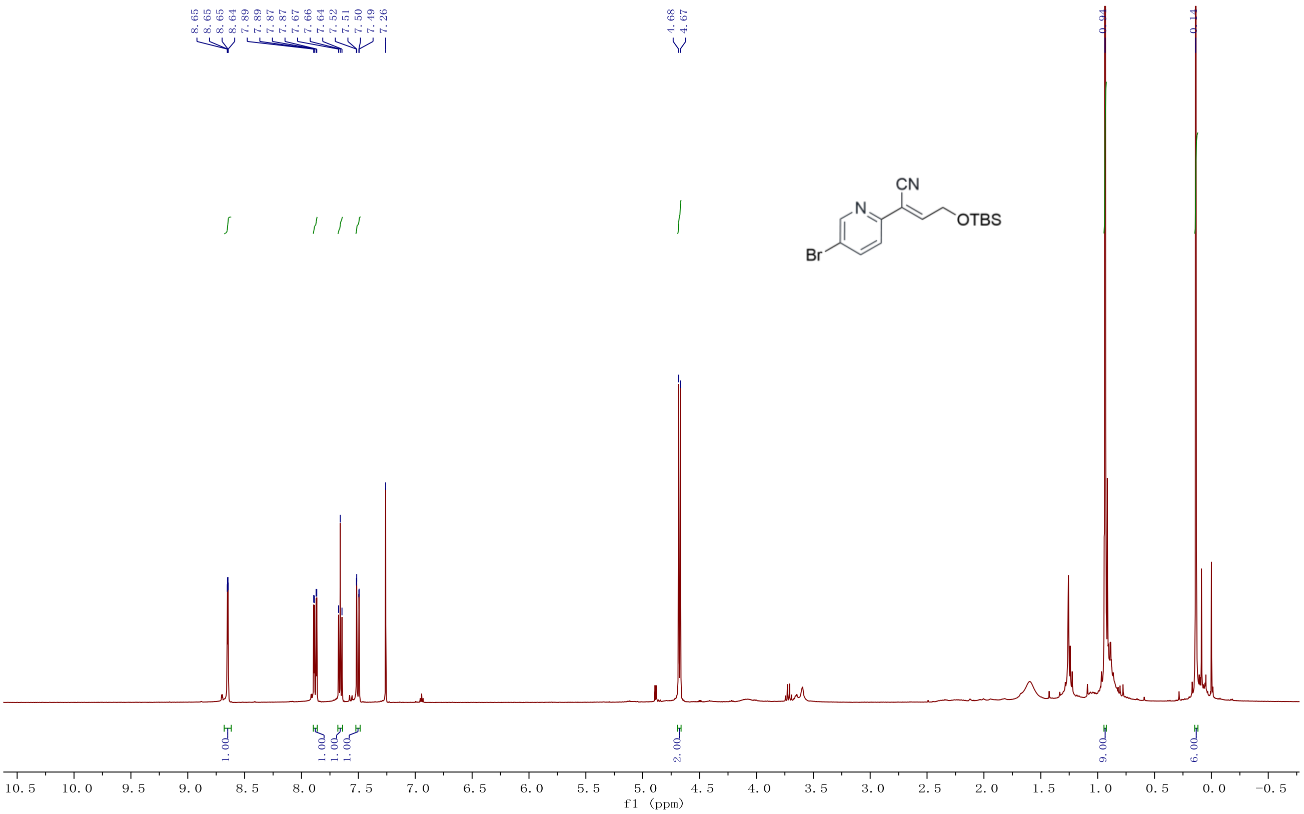

Supplementary Figure 202. ^{13}C NMR spectrum of compound **1cj** (100 MHz, $CDCl_3$)

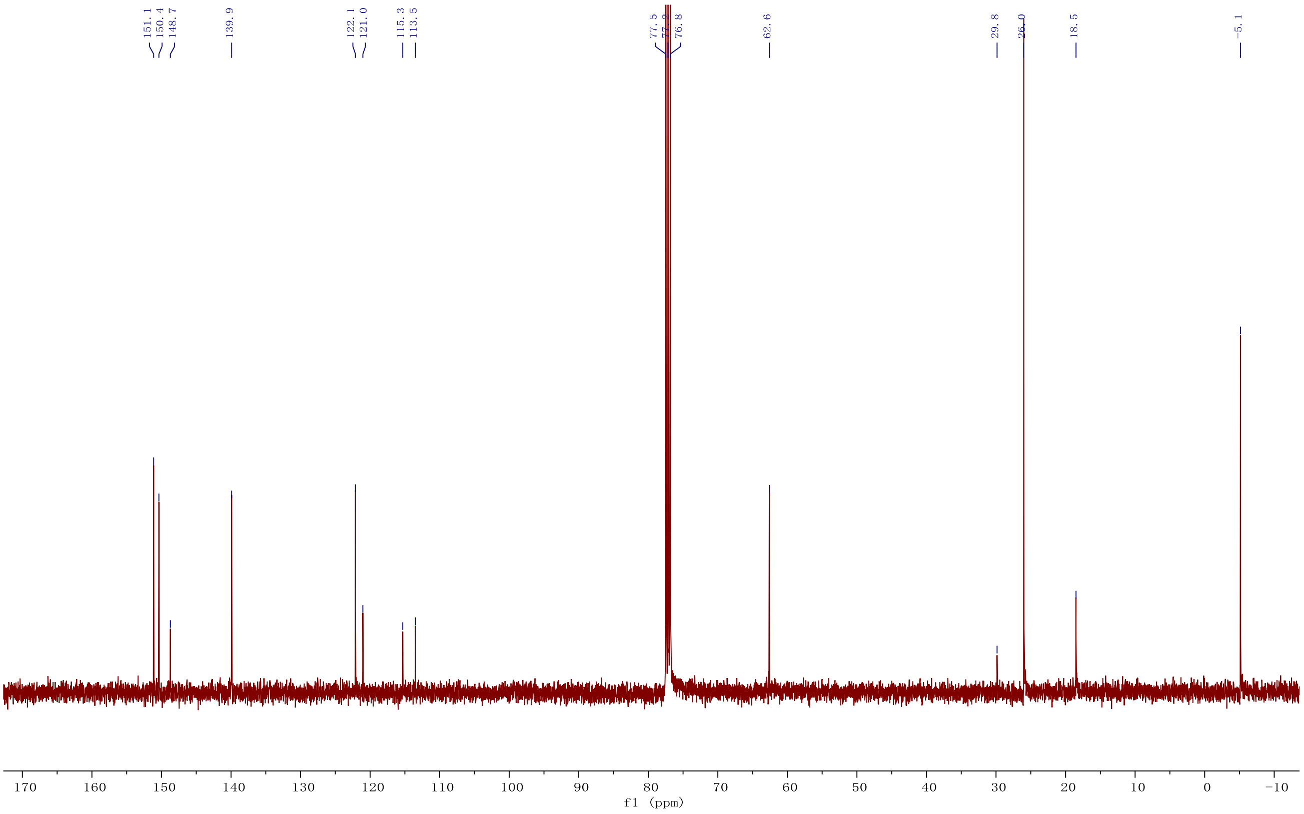

Supplementary Figure 203. ^{1}H NMR spectrum of compound **1ck** (400 MHz, $CDCl_3$)

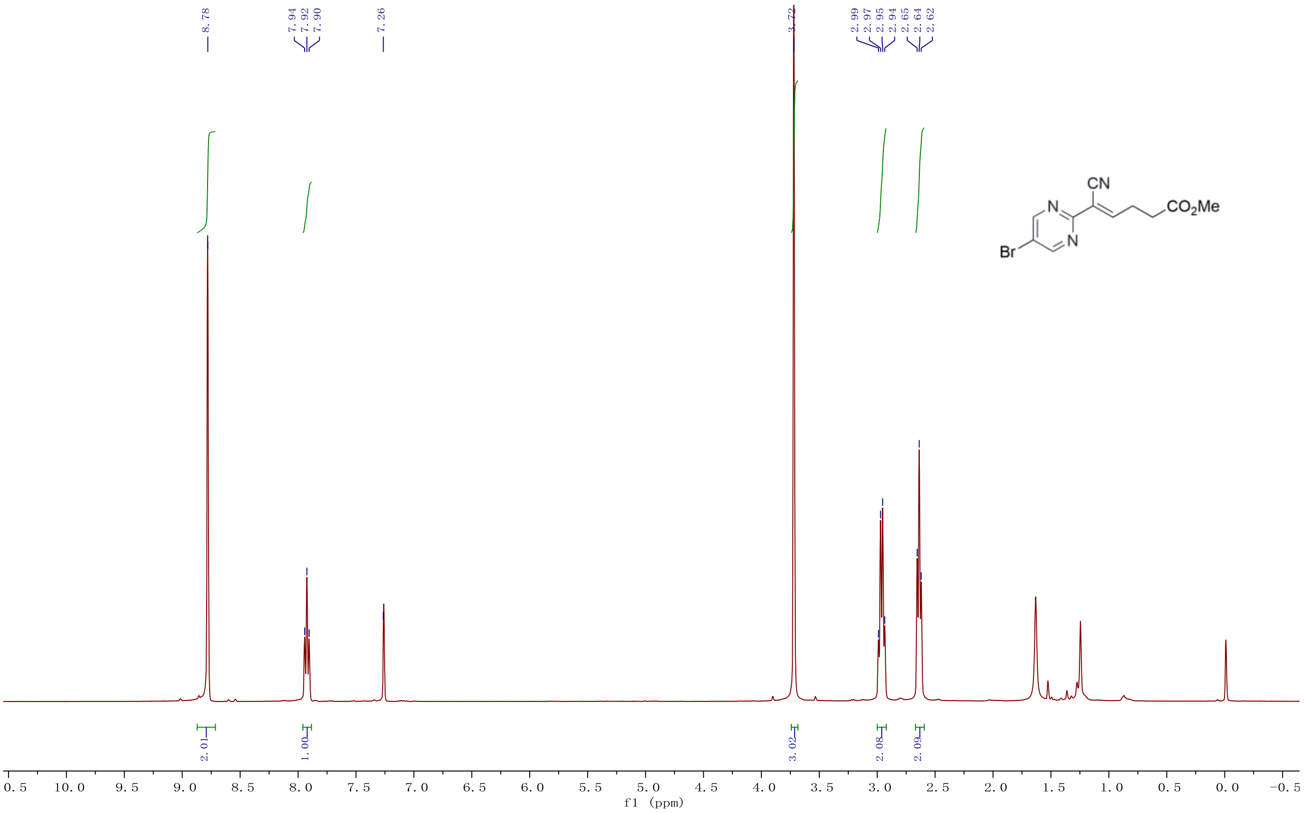

Supplementary Figure 204. ^{13}C NMR spectrum of compound **1ck** (100 MHz, $CDCl_3$)

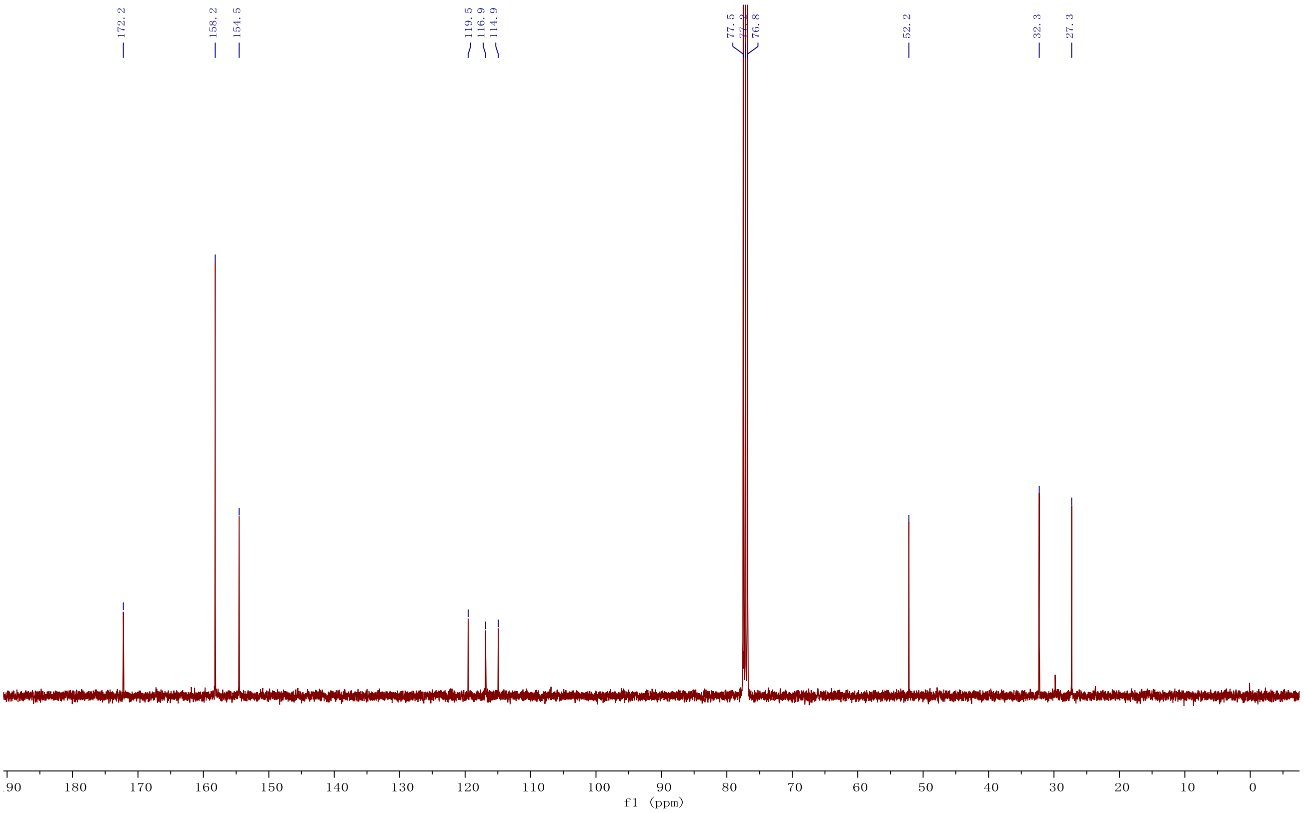

Supplementary Figure 205. ^{1}H NMR spectrum of compound **1cl** (400 MHz, $CDCl_3$)

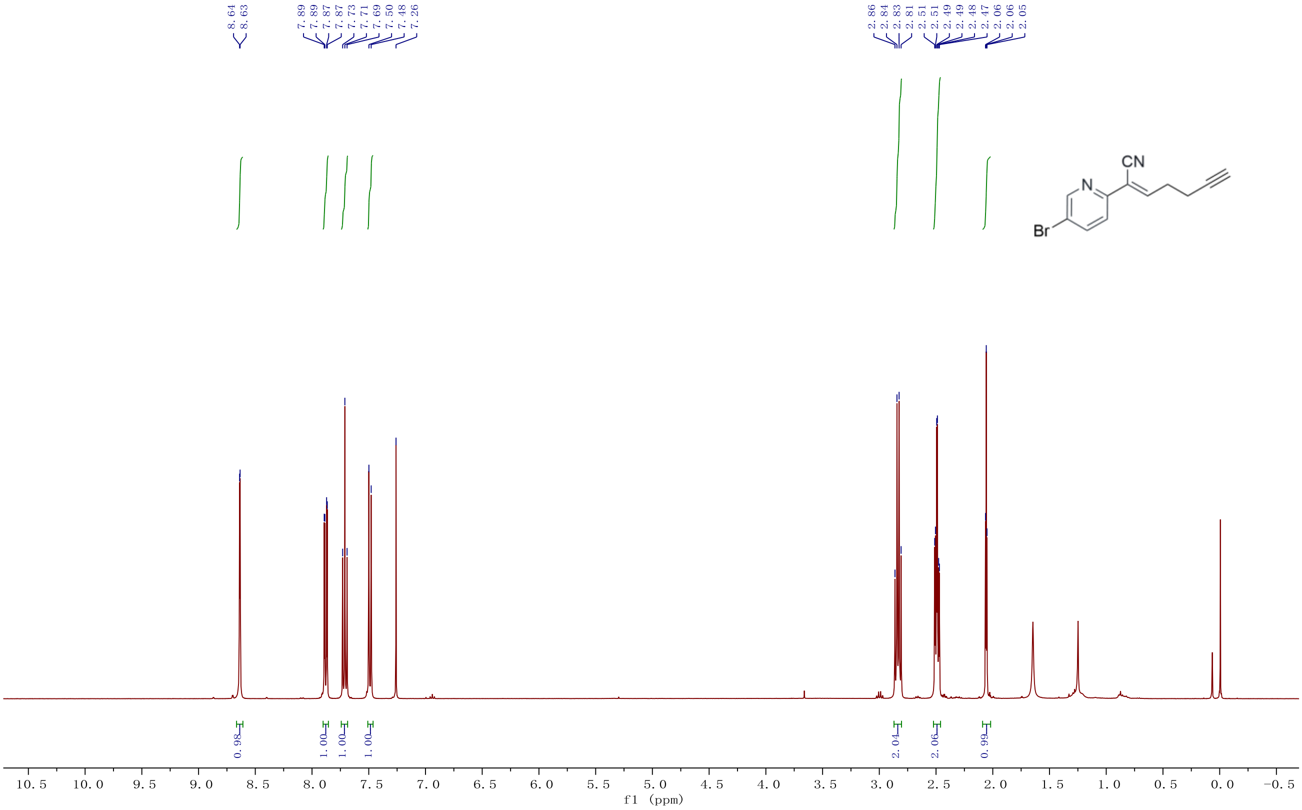

Supplementary Figure 206. ^{13}C NMR spectrum of compound **1cl** (100 MHz, $CDCl_3$)

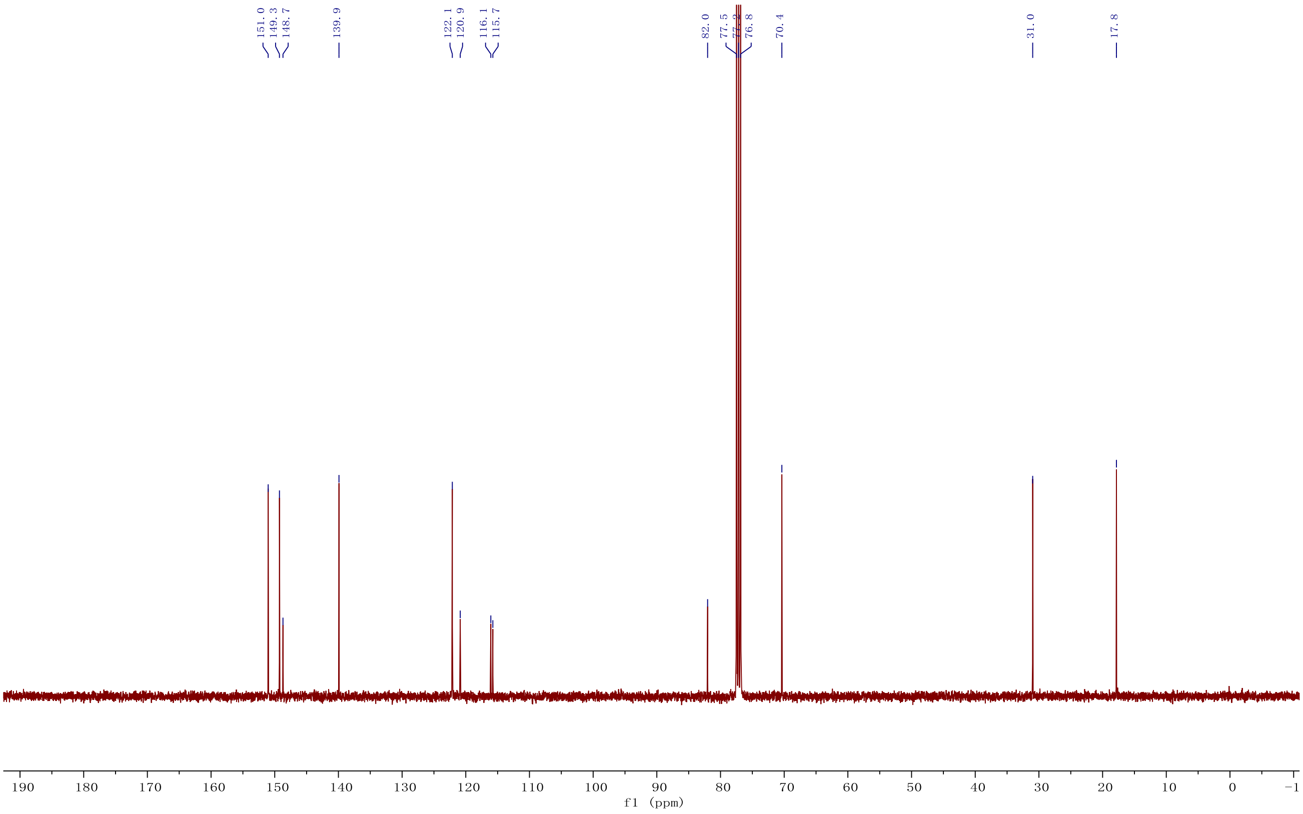

Supplementary Figure 207. ^{1}H NMR spectrum of compound **1cm** (400 MHz, $CDCl_3$)

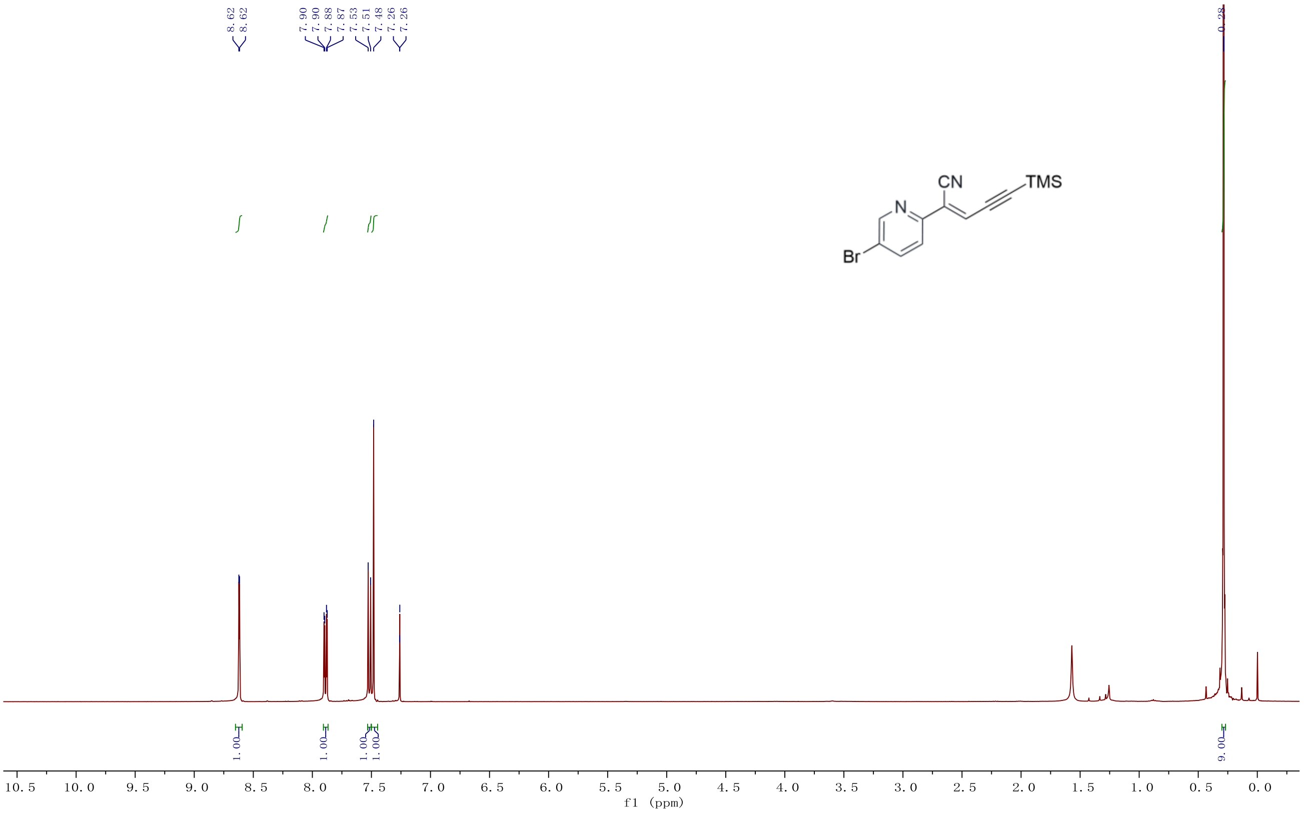

Supplementary Figure 208. ^{13}C NMR spectrum of compound **1cm** (100 MHz, $CDCl_3$)

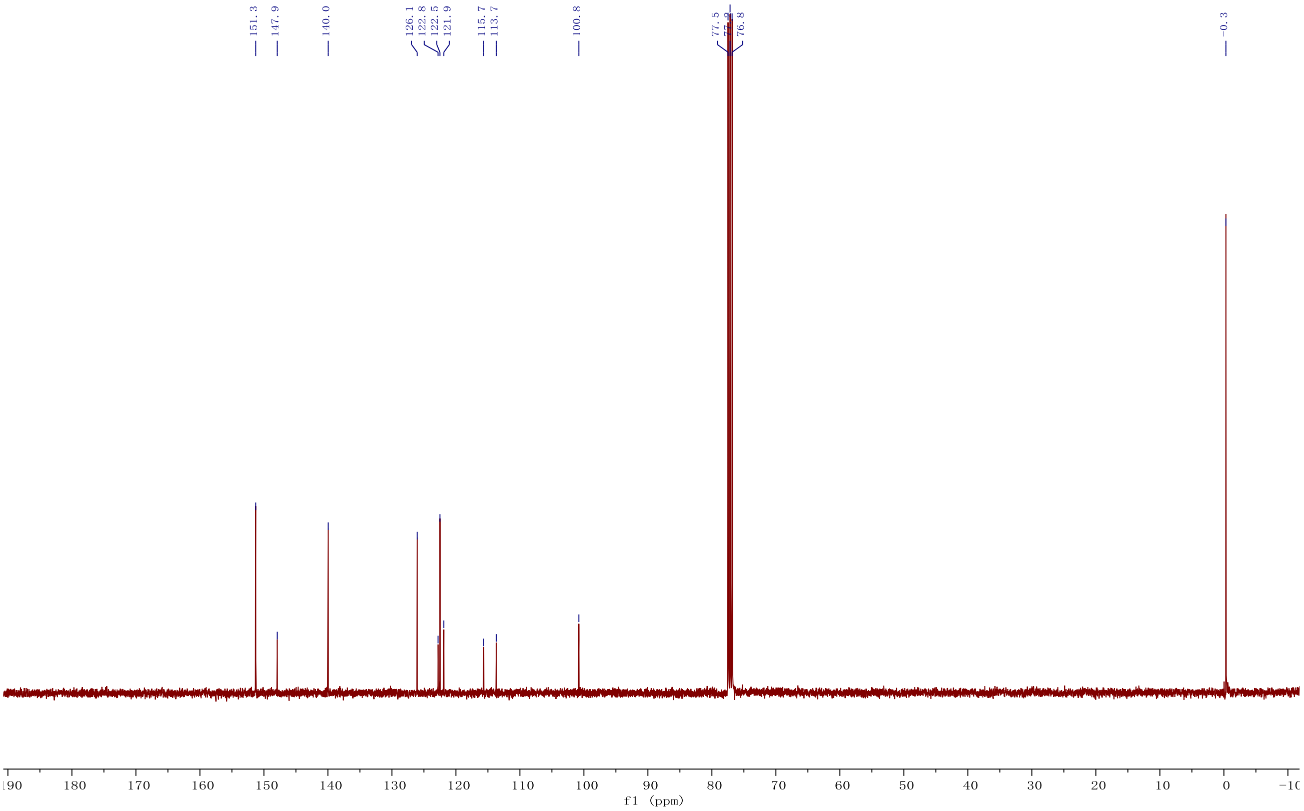

Supplementary Figure 209. ^{1}H NMR spectrum of compound **1co** (400 MHz, $CDCl_3$)

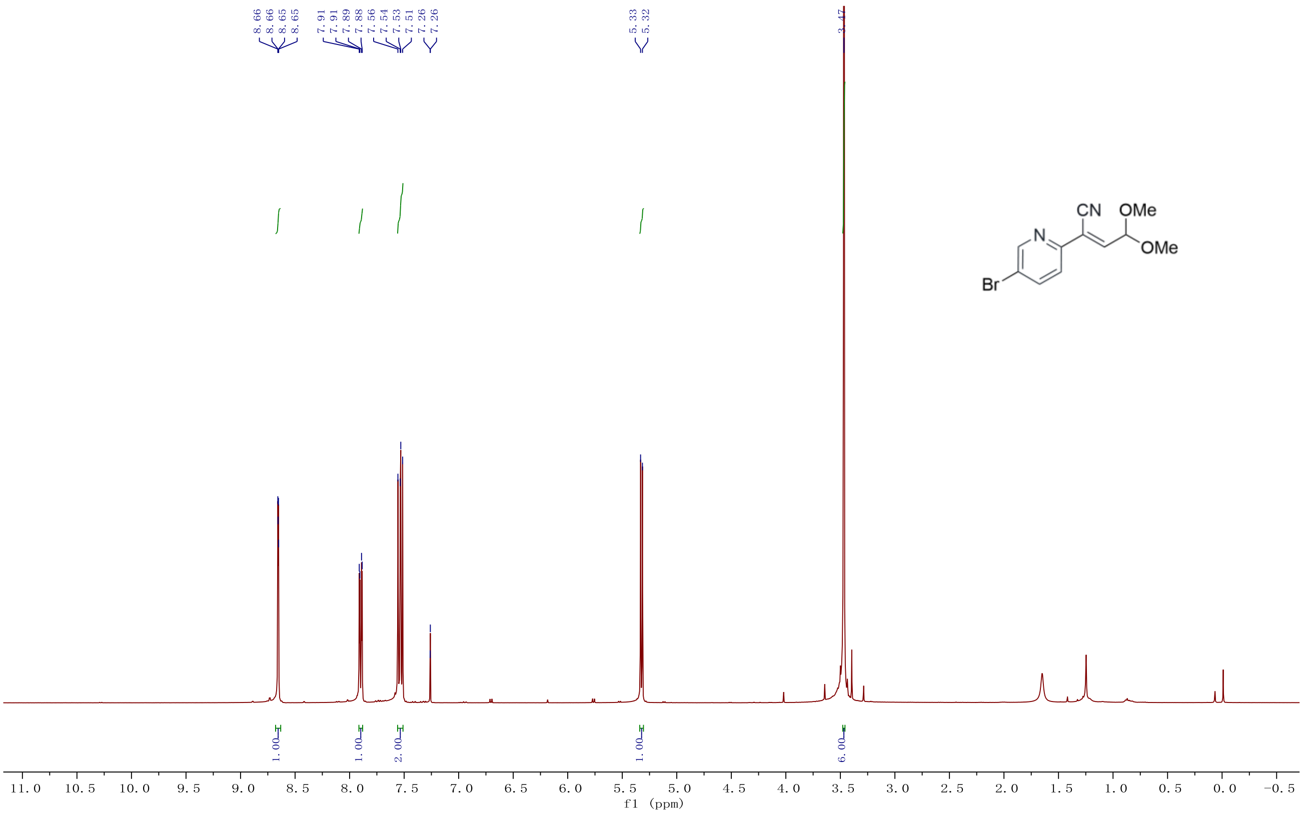

Supplementary Figure 210. ^{13}C NMR spectrum of compound **1co** (100 MHz, $CDCl_3$)

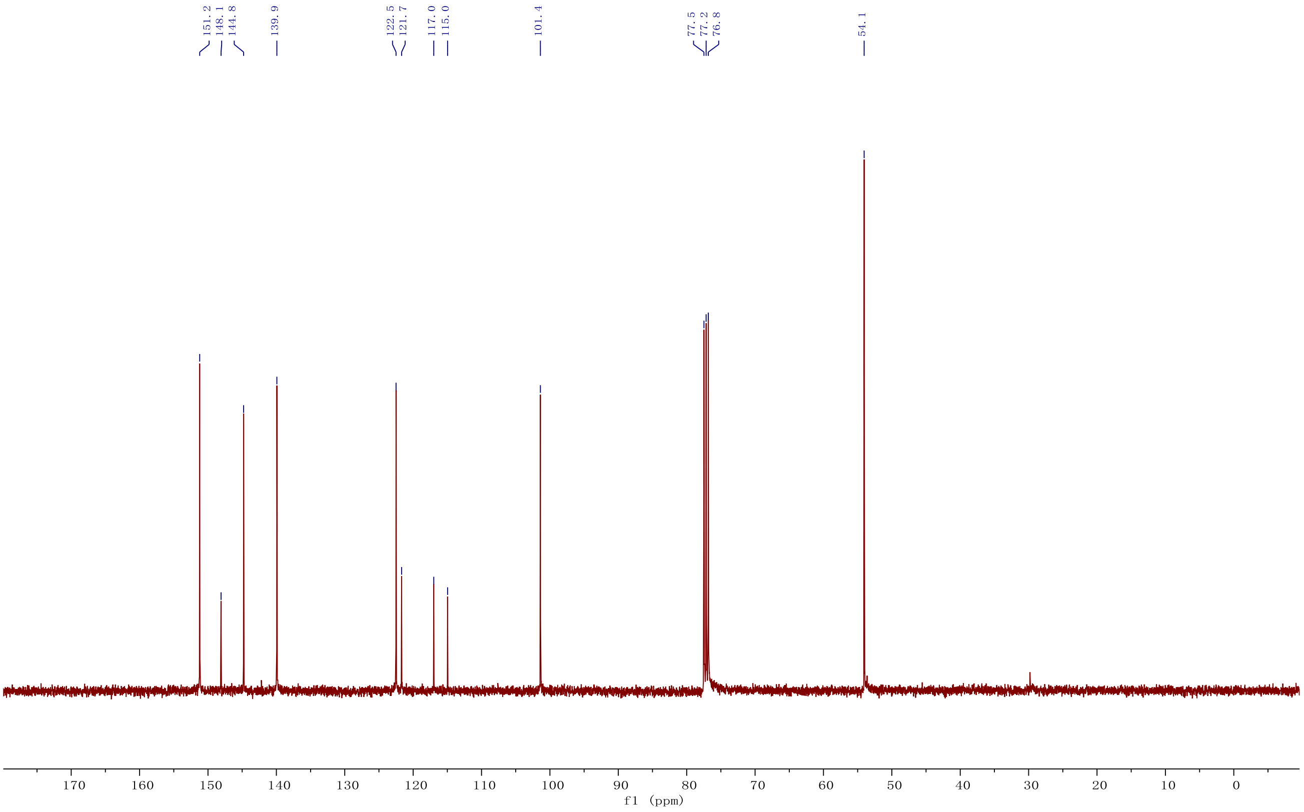

Supplementary Figure 211. ^{1}H NMR spectrum of compound **1da** (400 MHz, $CDCl_3$)

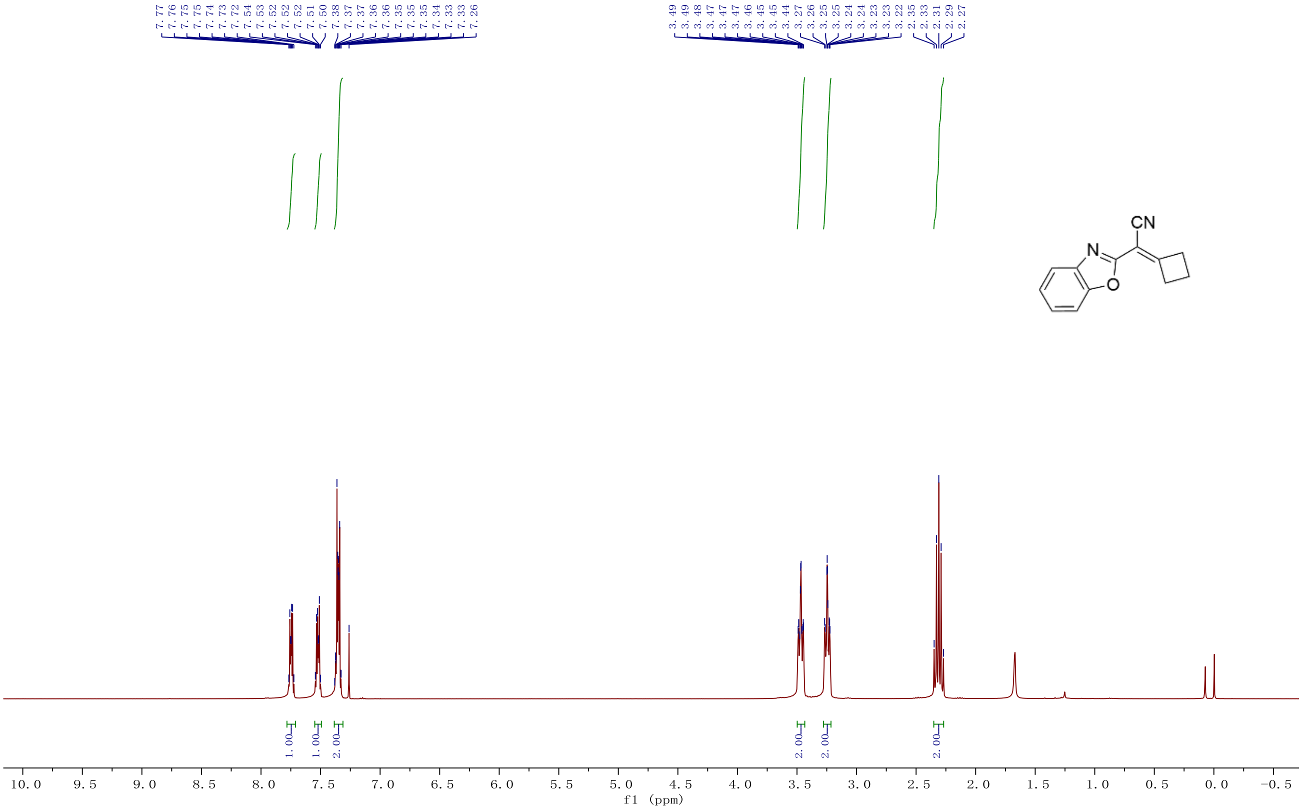

Supplementary Figure 212. ^{13}C NMR spectrum of compound **1da** (100 MHz, $CDCl_3$)

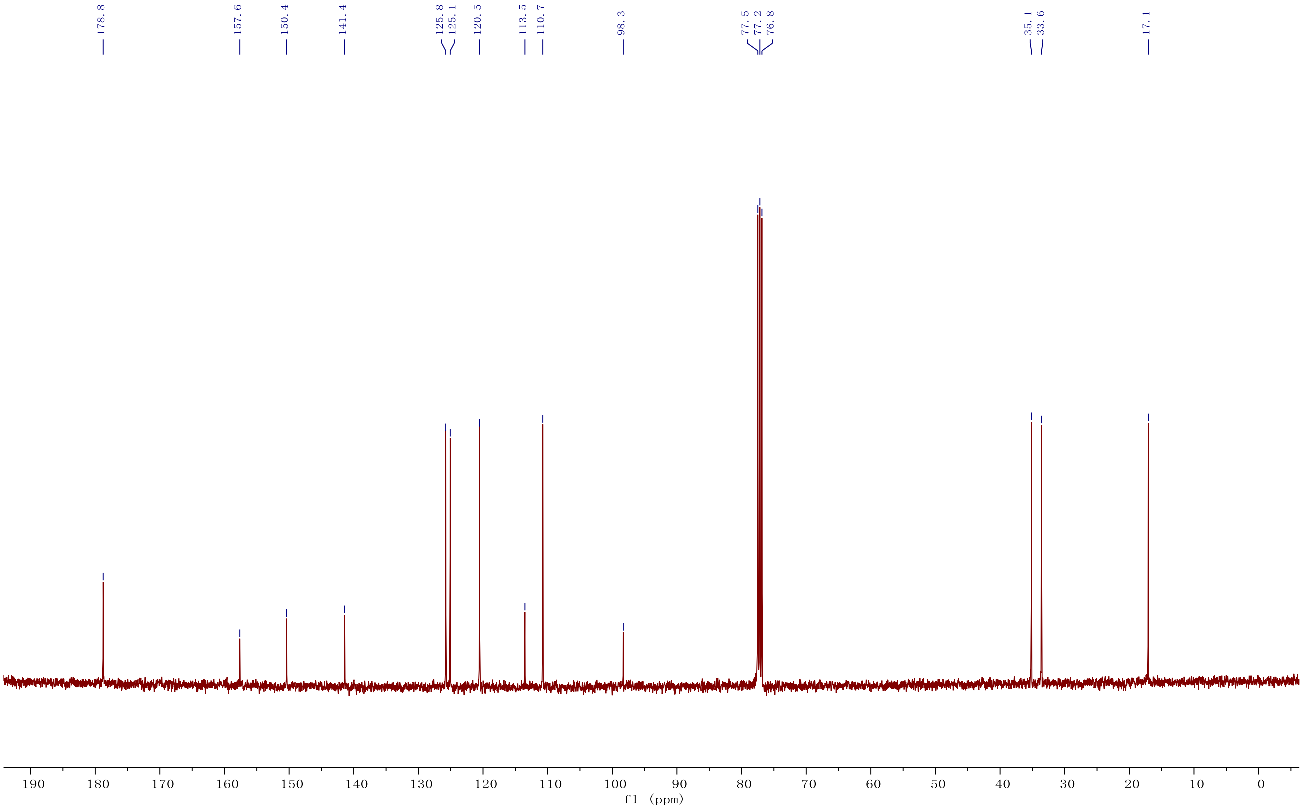

Supplementary Figure 213. ^{1}H NMR spectrum of compound **1db** (600 MHz, $CDCl_3$)

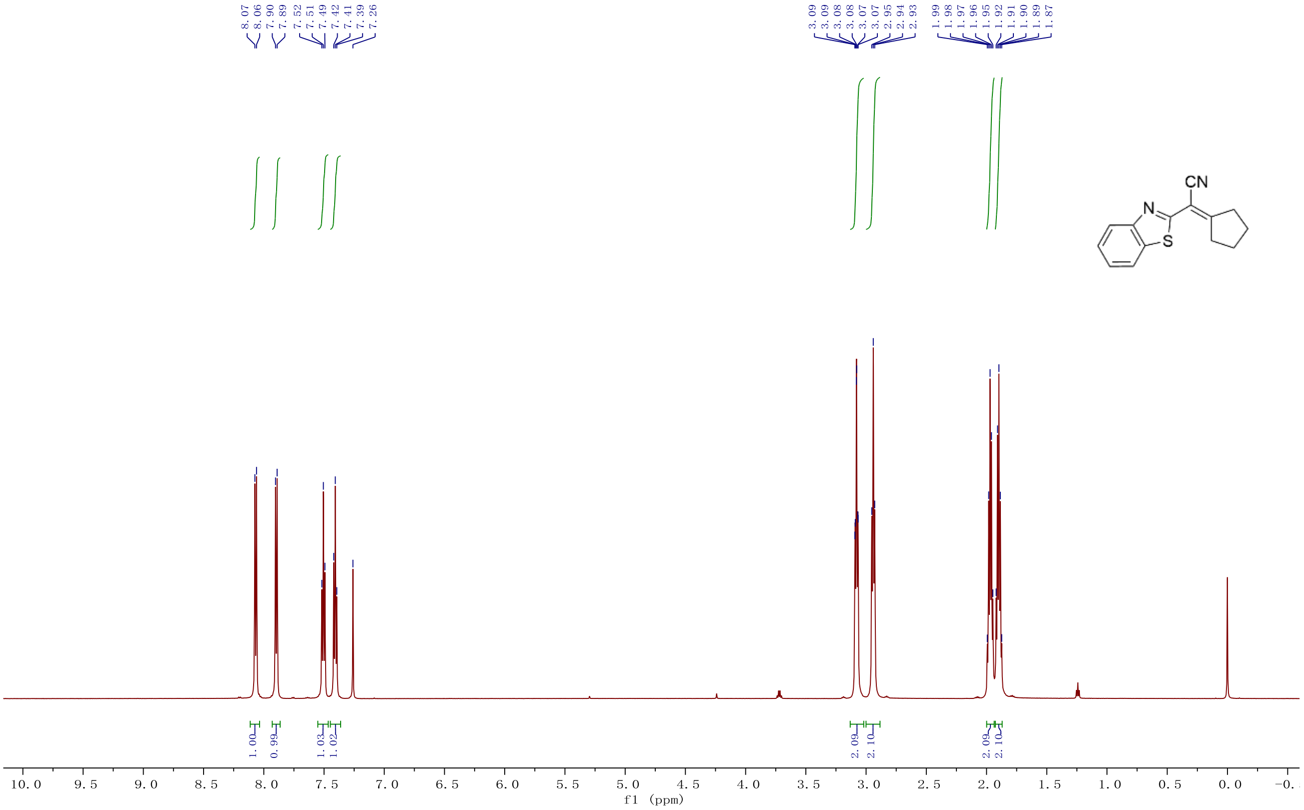

Supplementary Figure 214. ^{13}C NMR spectrum of compound **1db** (150 MHz, $CDCl_3$)

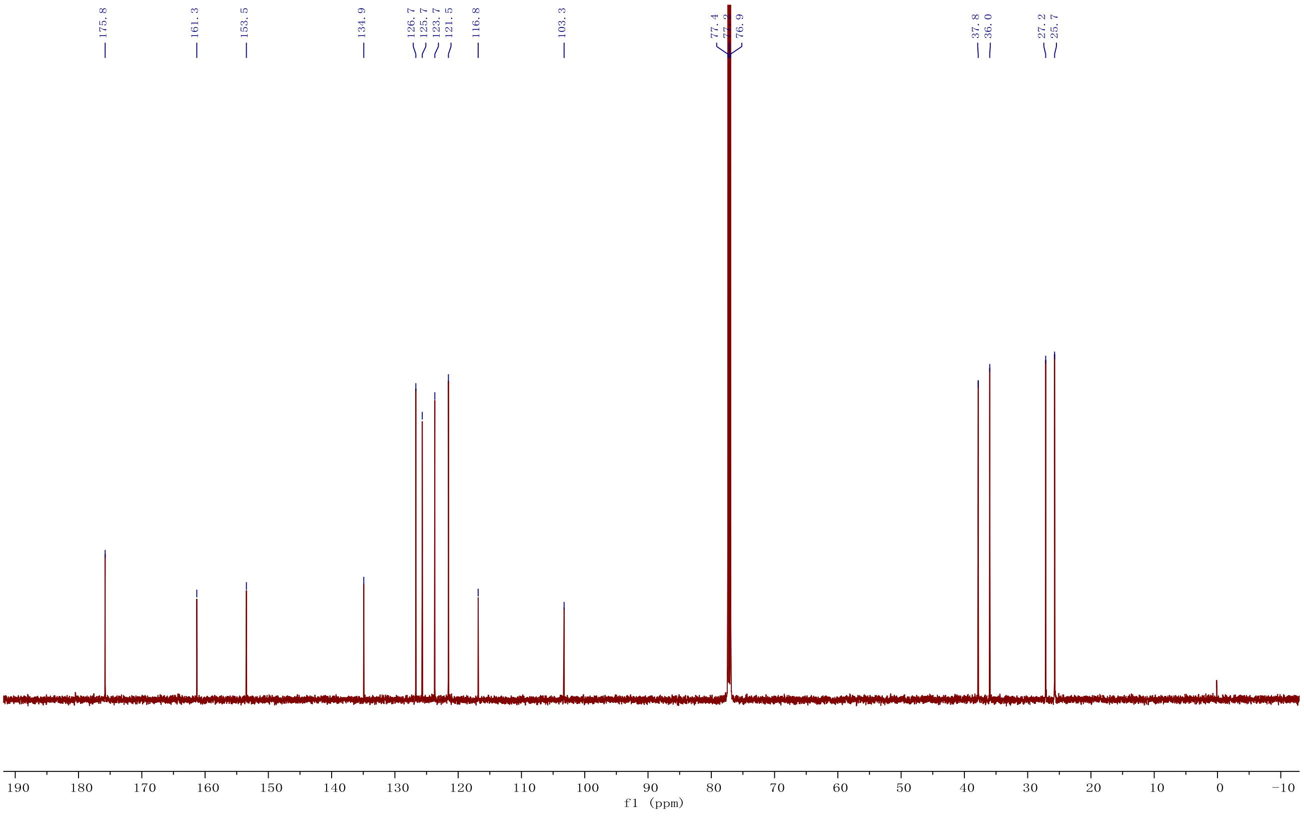

Supplementary Figure 215. ^{1}H NMR spectrum of compound **1dc** (400 MHz, $CDCl_3$)

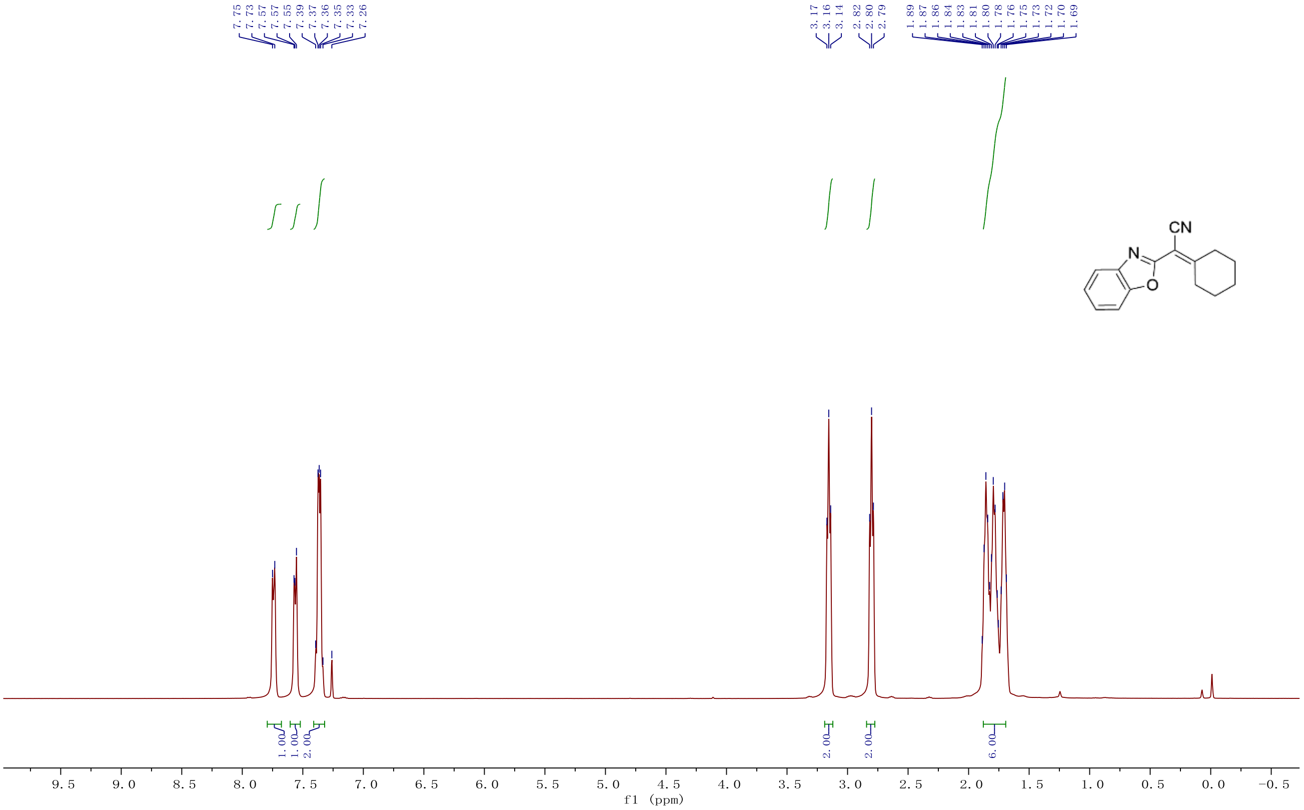

Supplementary Figure 216. ^{13}C NMR spectrum of compound **1dc** (100 MHz, $CDCl_3$)

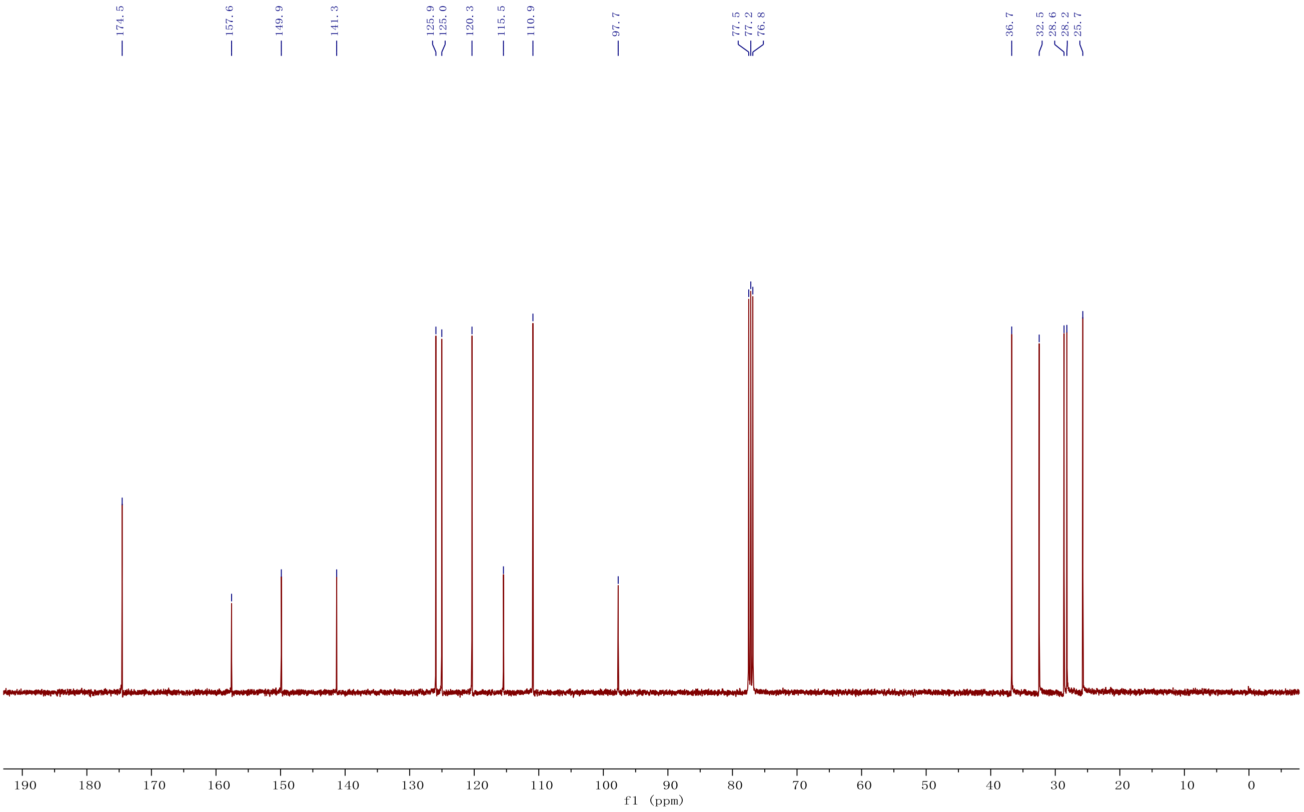

Supplementary Figure 217. ^{1}H NMR spectrum of compound **1dd** (400 MHz, $CDCl_3$)

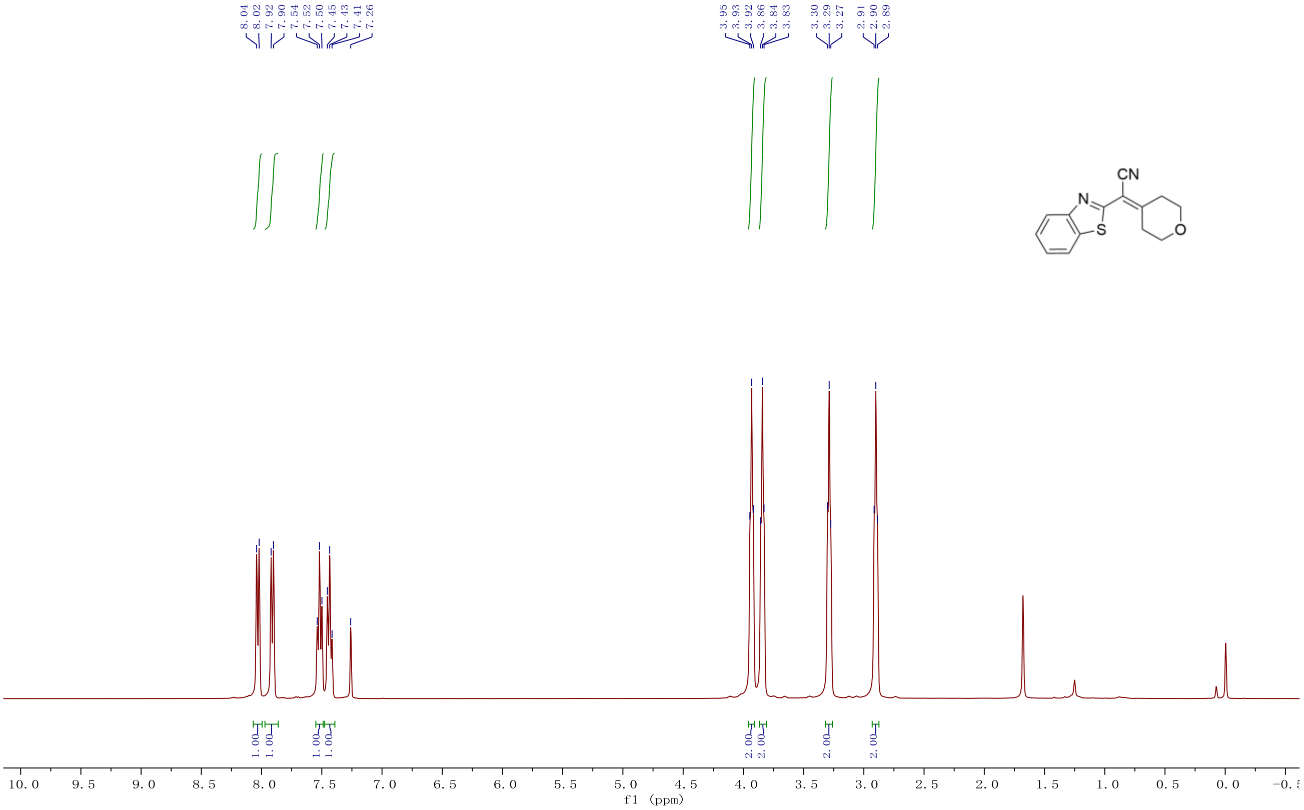

Supplementary Figure 218. ^{13}C NMR spectrum of compound **1dd** (100 MHz, $CDCl_3$)

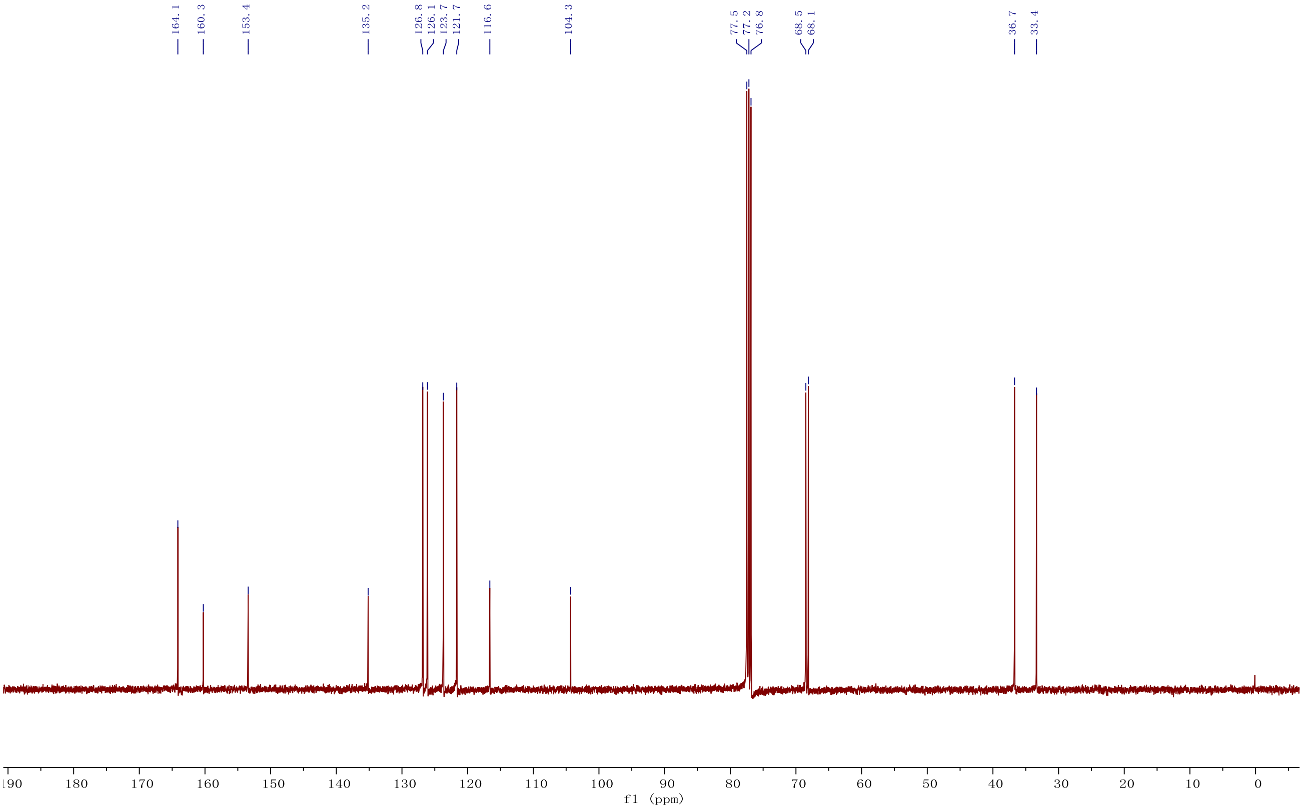

Supplementary Figure 219. ^{1}H NMR spectrum of compound **1de** (400 MHz, $CDCl_3$)

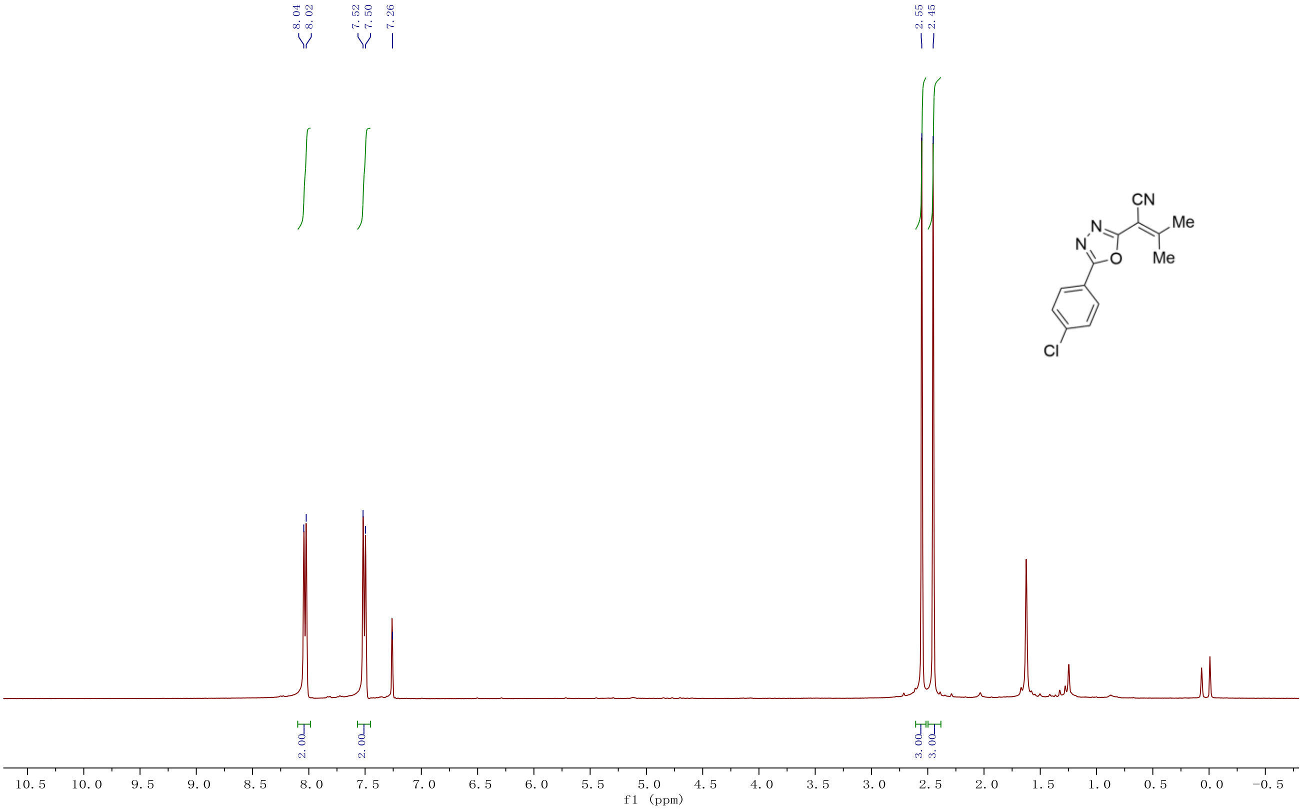

Supplementary Figure 220. ^{13}C NMR spectrum of compound **1de** (100 MHz, $CDCl_3$)

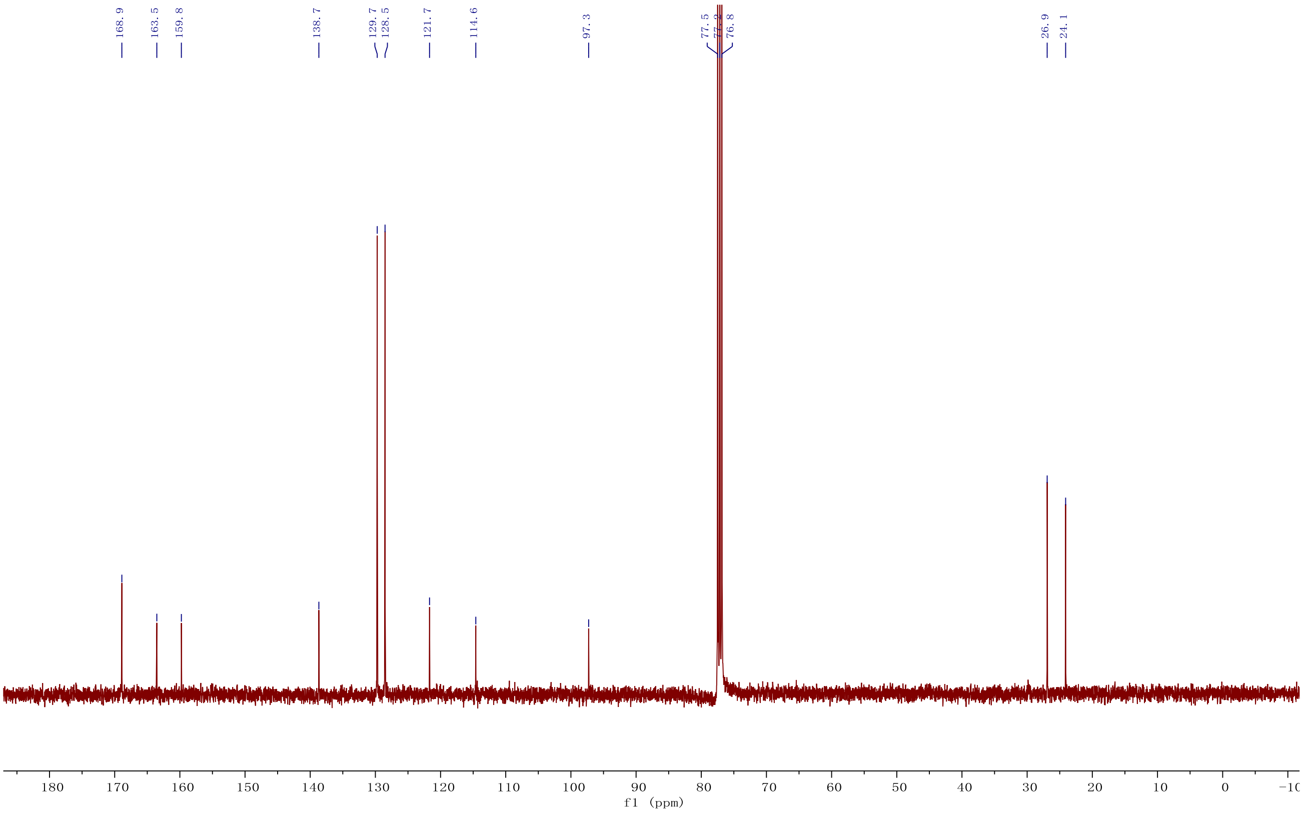

Supplementary Figure 221. ^{1}H NMR spectrum of compound **1dg** (400 MHz, $CDCl_3$)

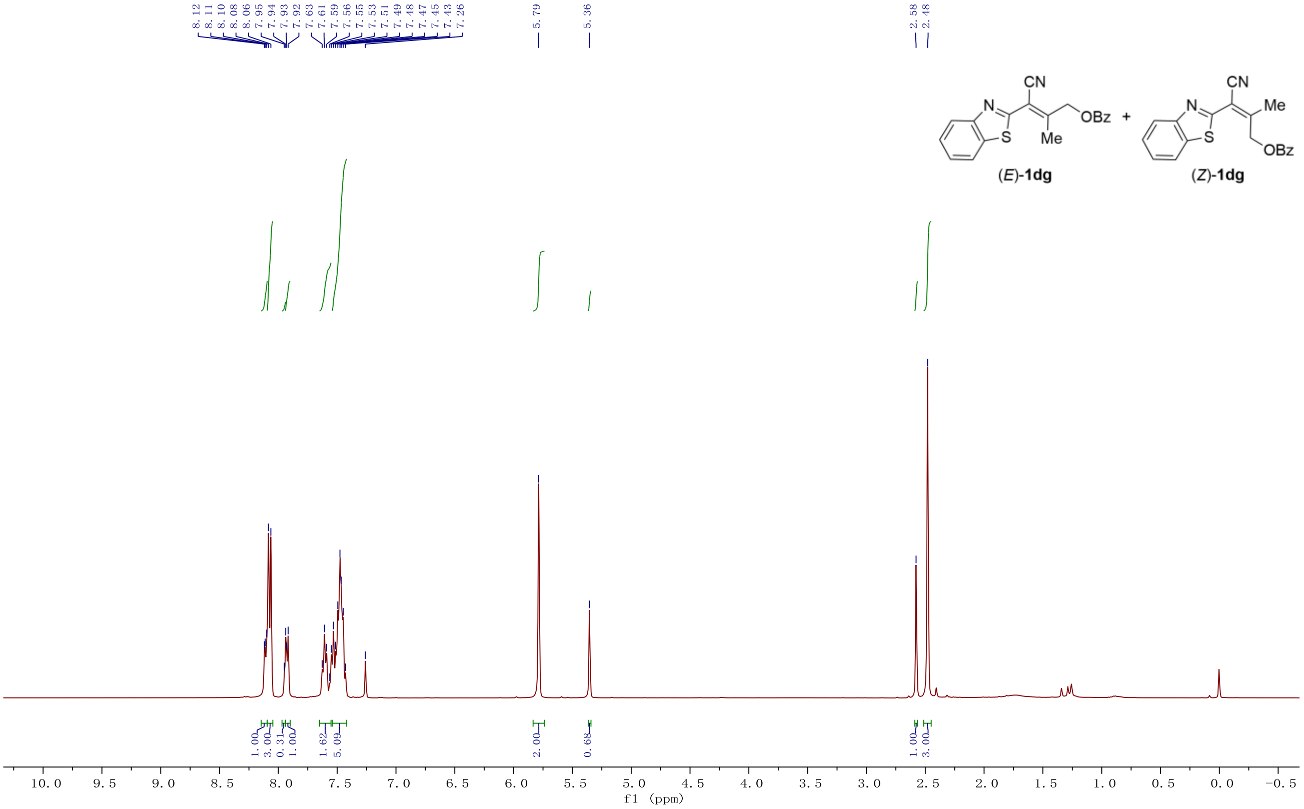

Supplementary Figure 222. ^{13}C NMR spectrum of compound **1dg** (100 MHz, $CDCl_3$)

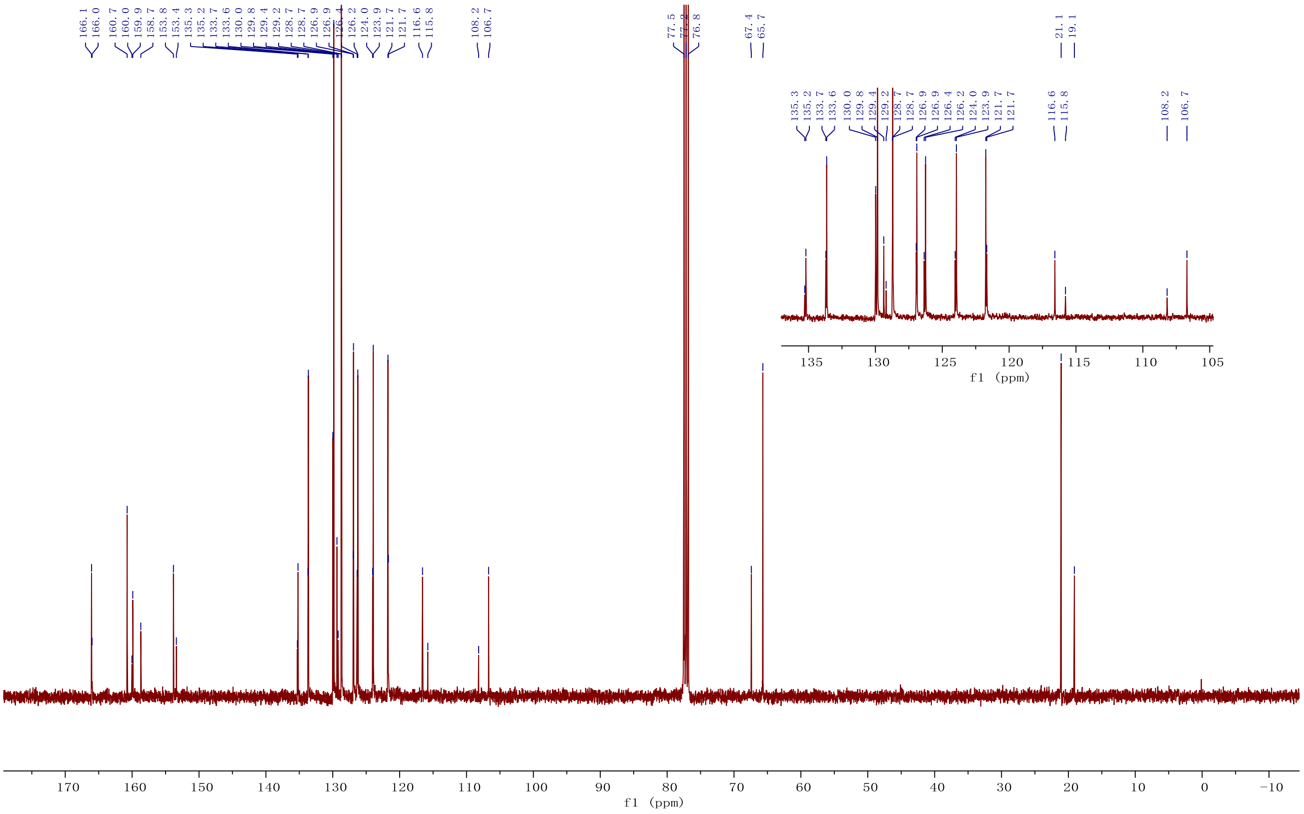

Supplementary Figure 223. ^{1}H NMR spectrum of compound **1fa** (400 MHz, $CDCl_3$)

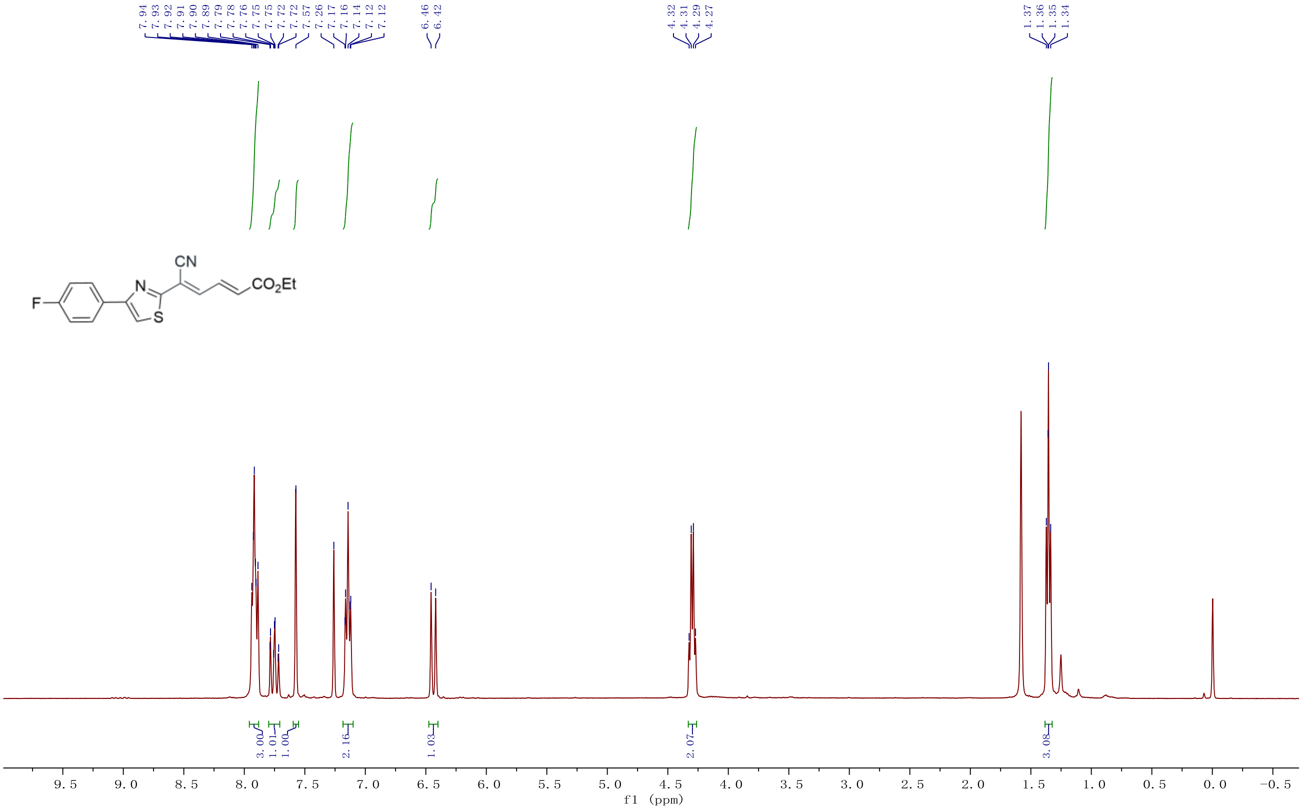

Supplementary Figure 224. ^{13}C NMR spectrum of compound **1fa** (100 MHz, $CDCl_3$)

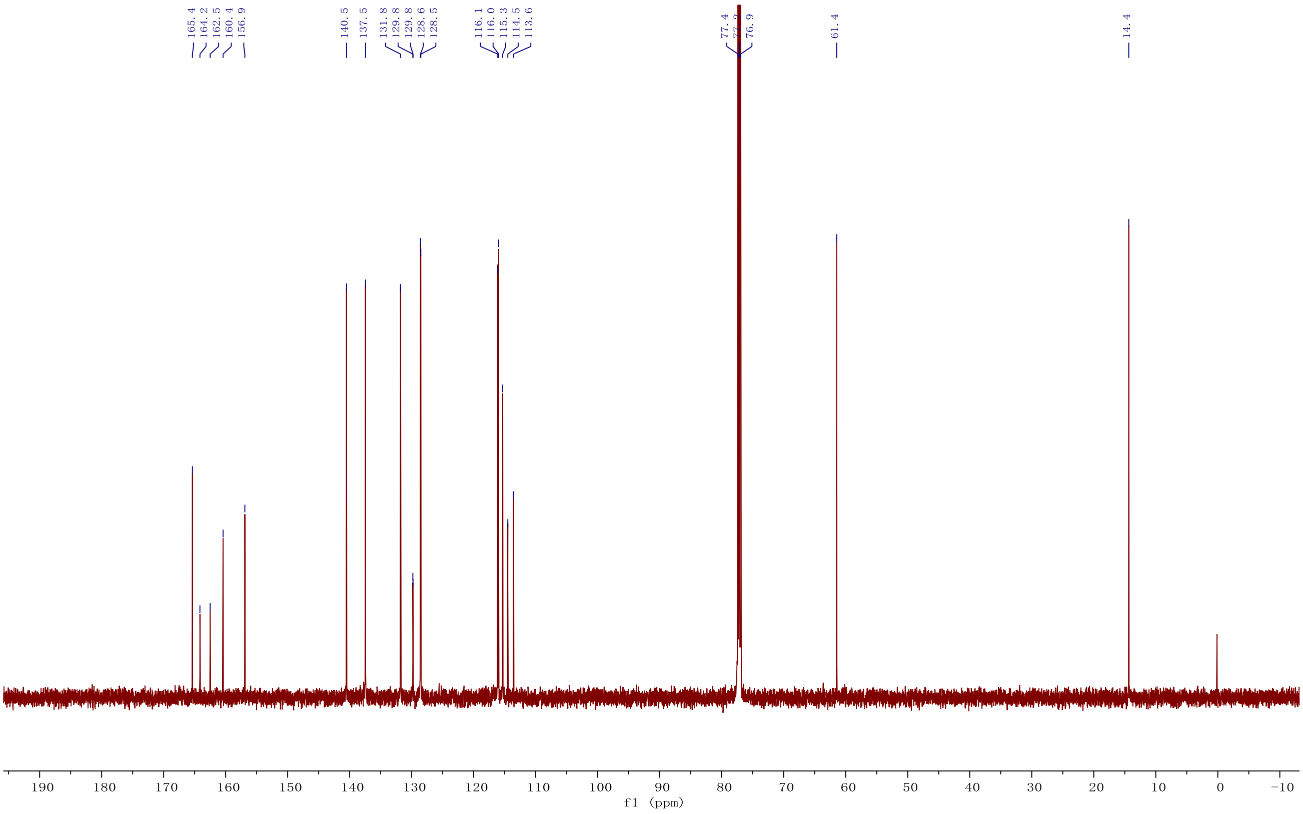

Supplementary Figure 225. ^{19}F NMR spectrum of compound **1fa** (376 MHz, $CDCl_3$)

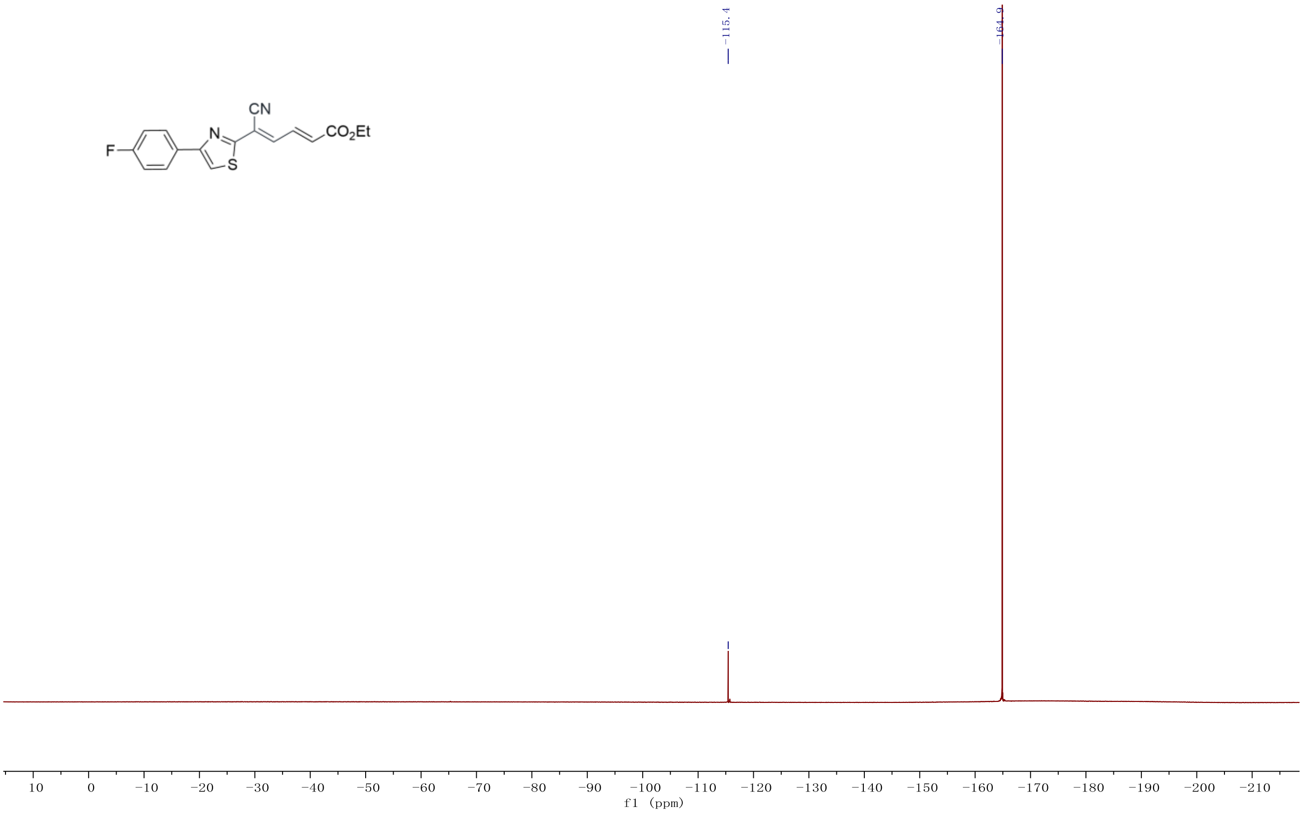

Supplementary Figure 226. ^{1}H NMR spectrum of compound **1fb** (400 MHz, $CDCl_3$)

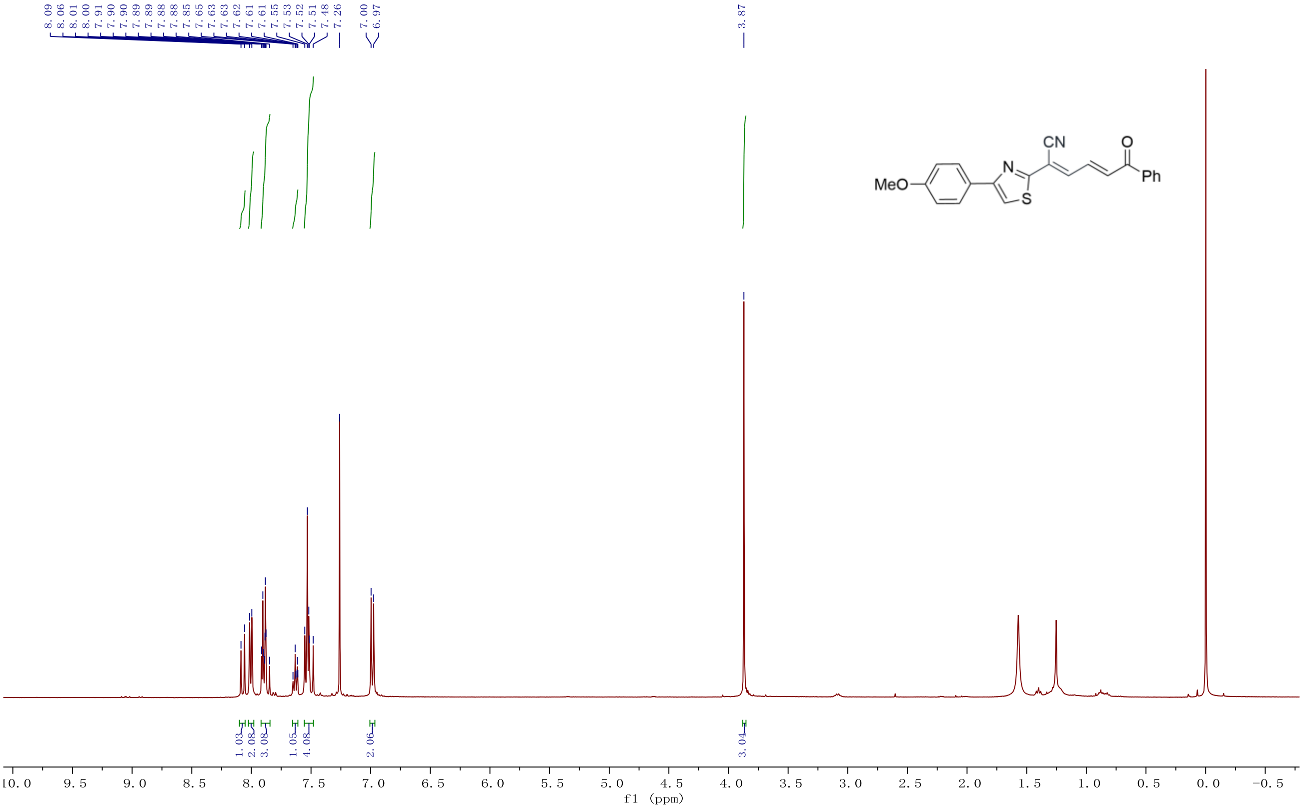

Supplementary Figure 227. ^{13}C NMR spectrum of compound **1fb** (100 MHz, $CDCl_3$)

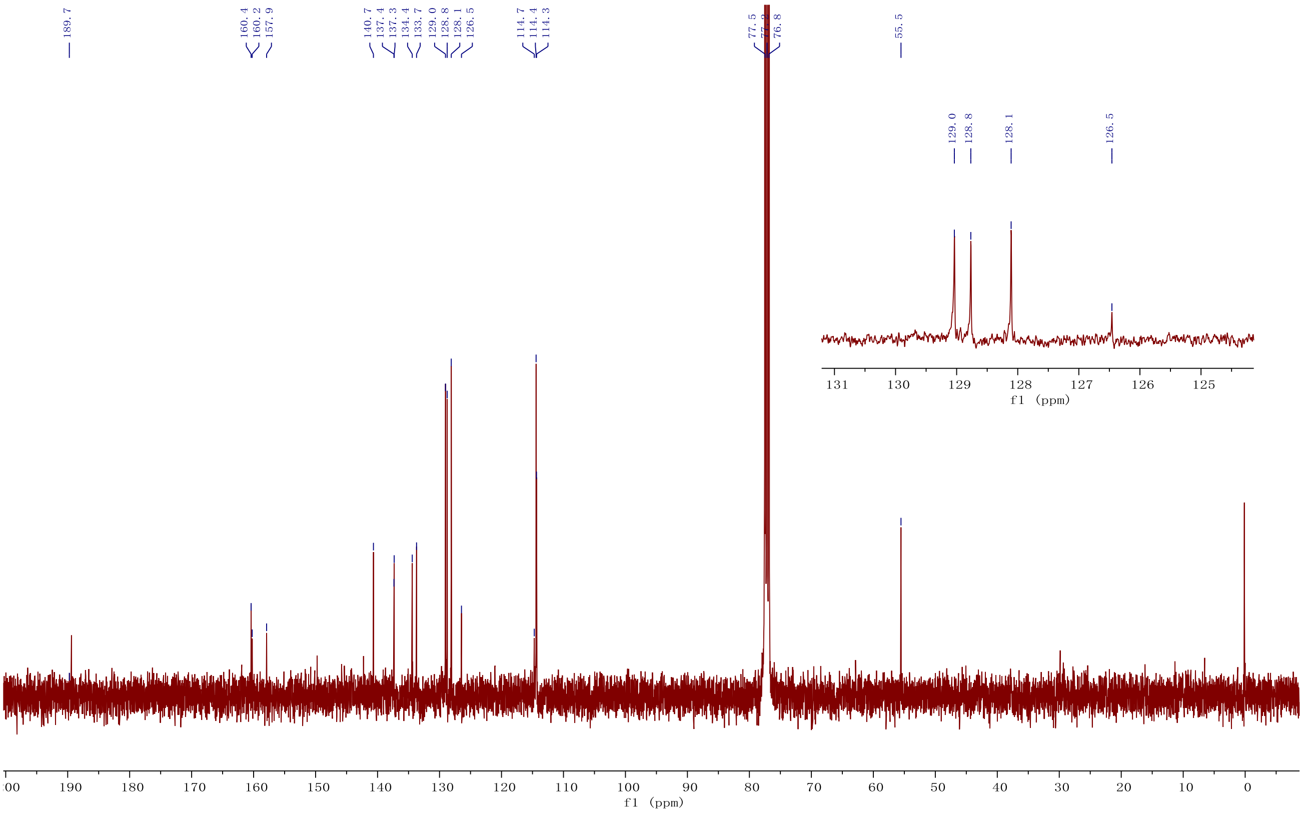

Supplementary Figure 228. ^{1}H NMR spectrum of compound **1fc** (400 MHz, DMSO-d_6)

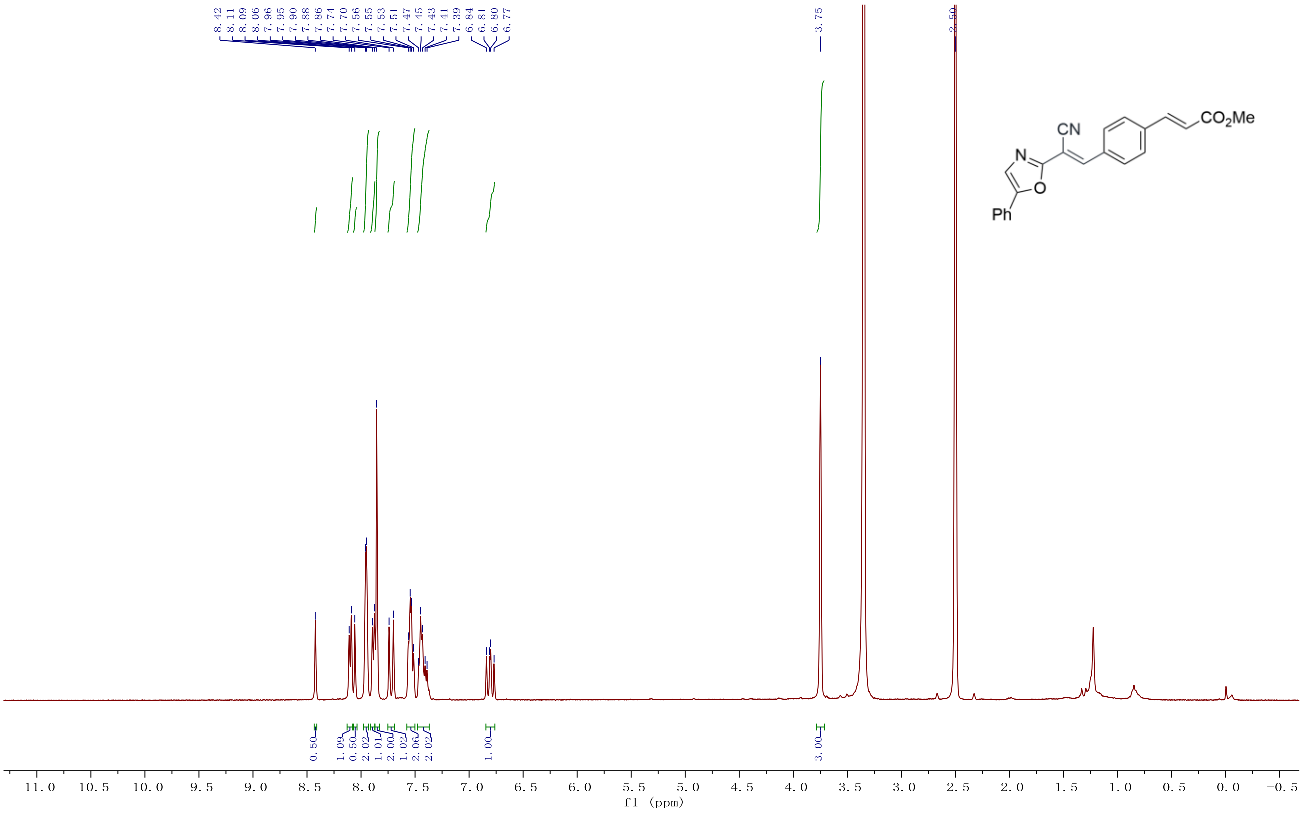

Supplementary Figure 229. ^{13}C NMR spectrum of compound **1fc** (100 MHz, DMSO-d_6)

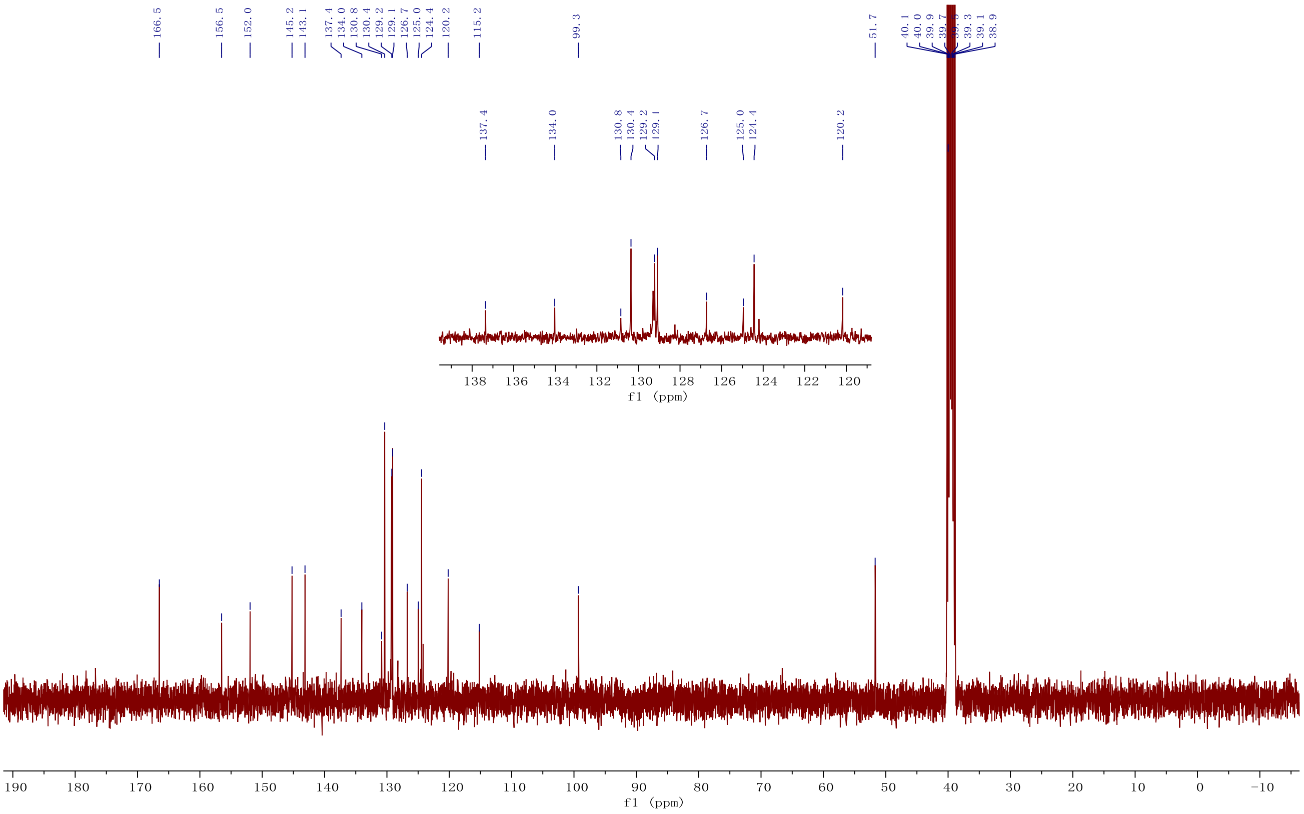

Supplementary Figure 230. ^{1}H NMR spectrum of compound **1fd** (400 MHz, DMSO-d_6)

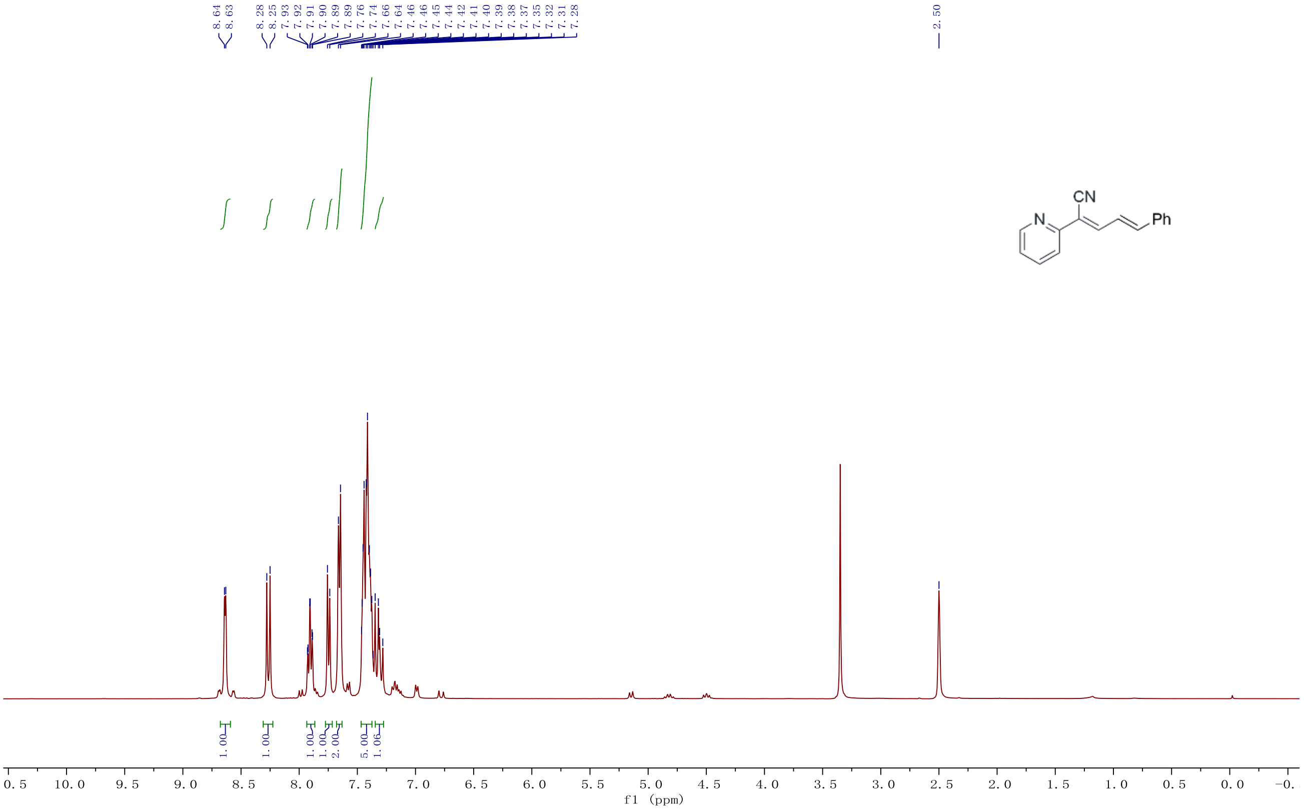

Supplementary Figure 231. ^{13}C NMR spectrum of compound **1fd** (100 MHz, DMSO-d_6)

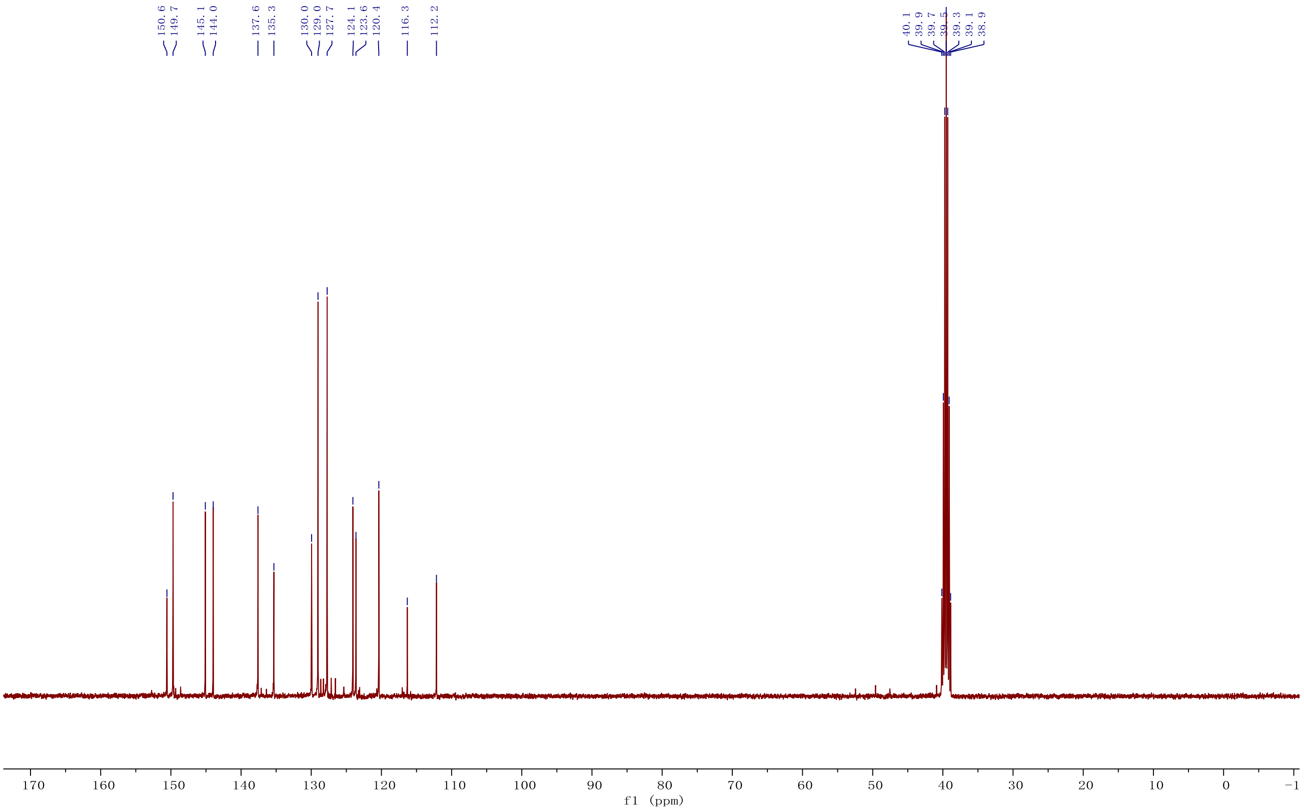

Supplementary Figure 232. ^{1}H NMR spectrum of compound **1fe** (400 MHz, $CDCl_3$)

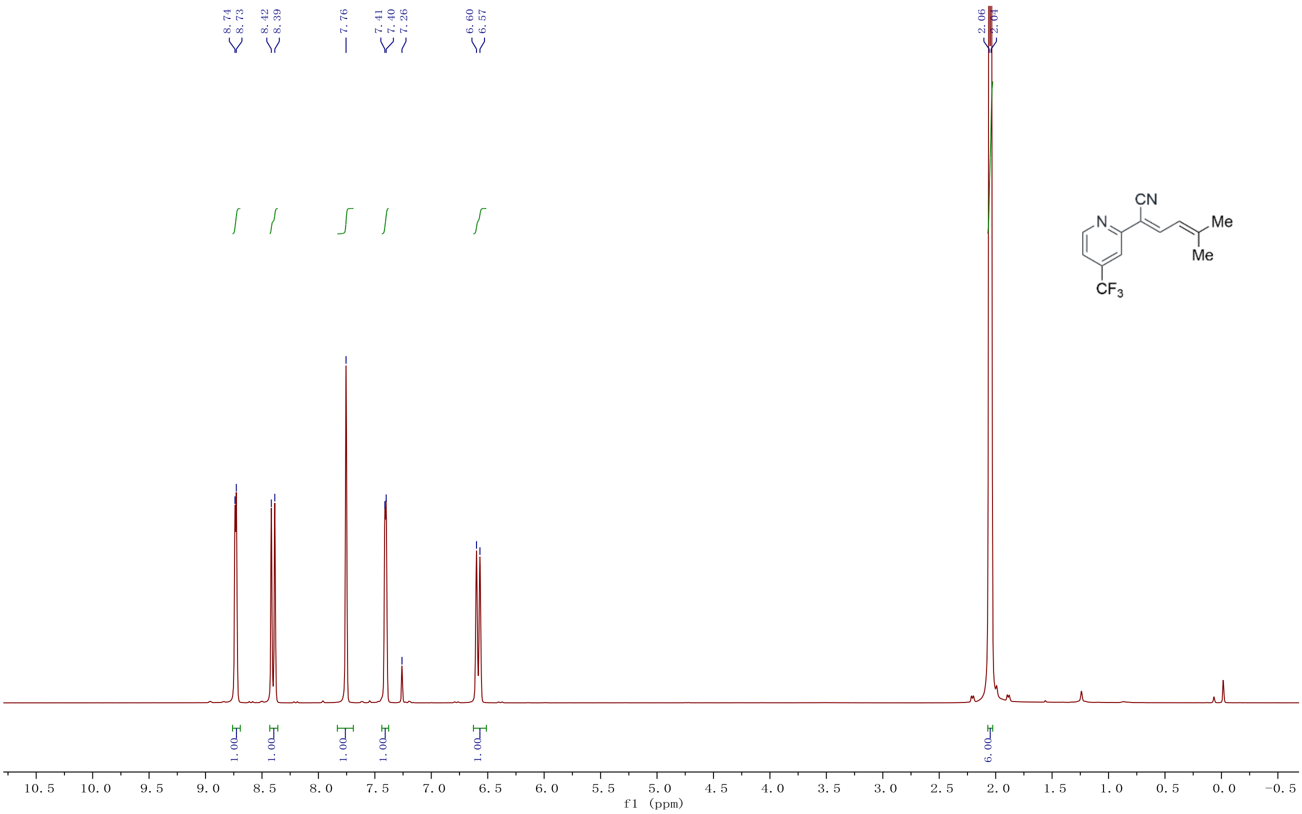

Supplementary Figure 233. ^{13}C NMR spectrum of compound **1fe** (100 MHz, $CDCl_3$)

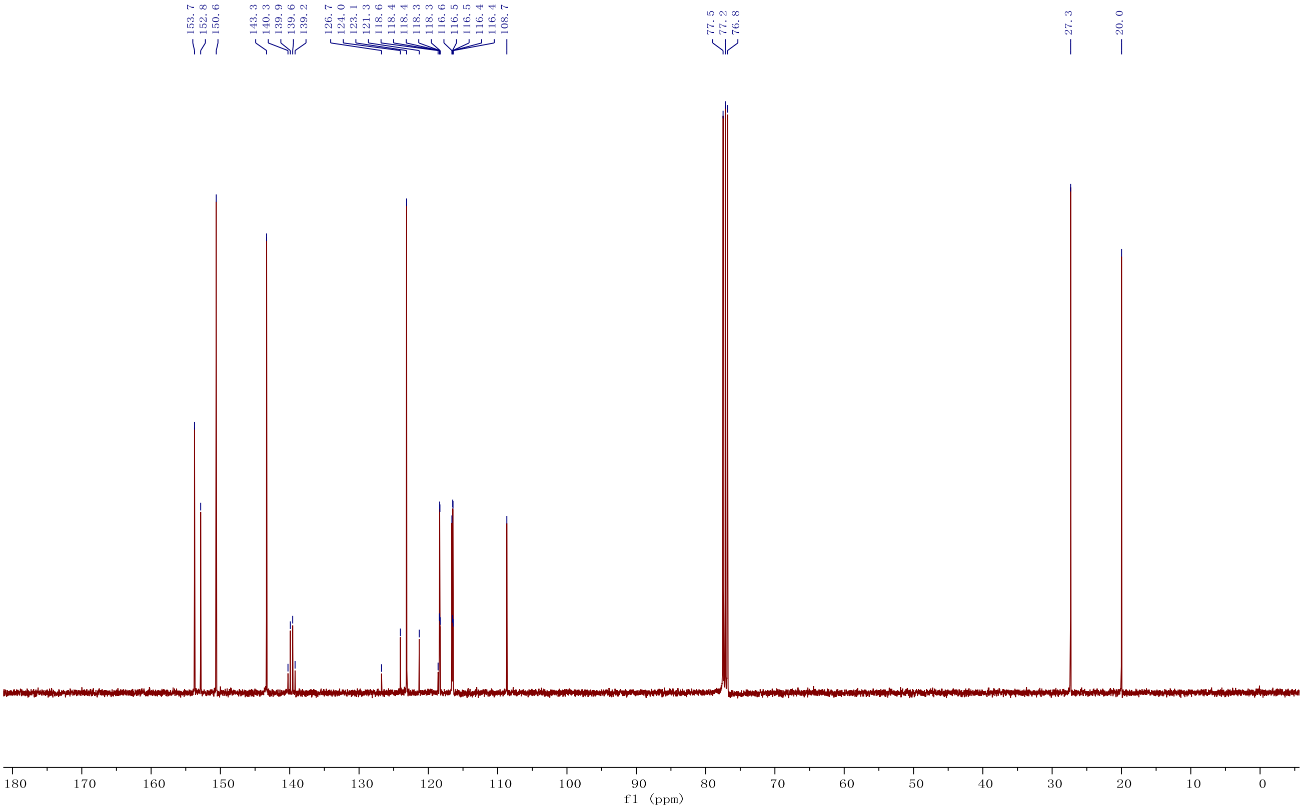

Supplementary Figure 234. ^{19}F NMR spectrum of compound **1fe** (376 MHz, $CDCl_3$)

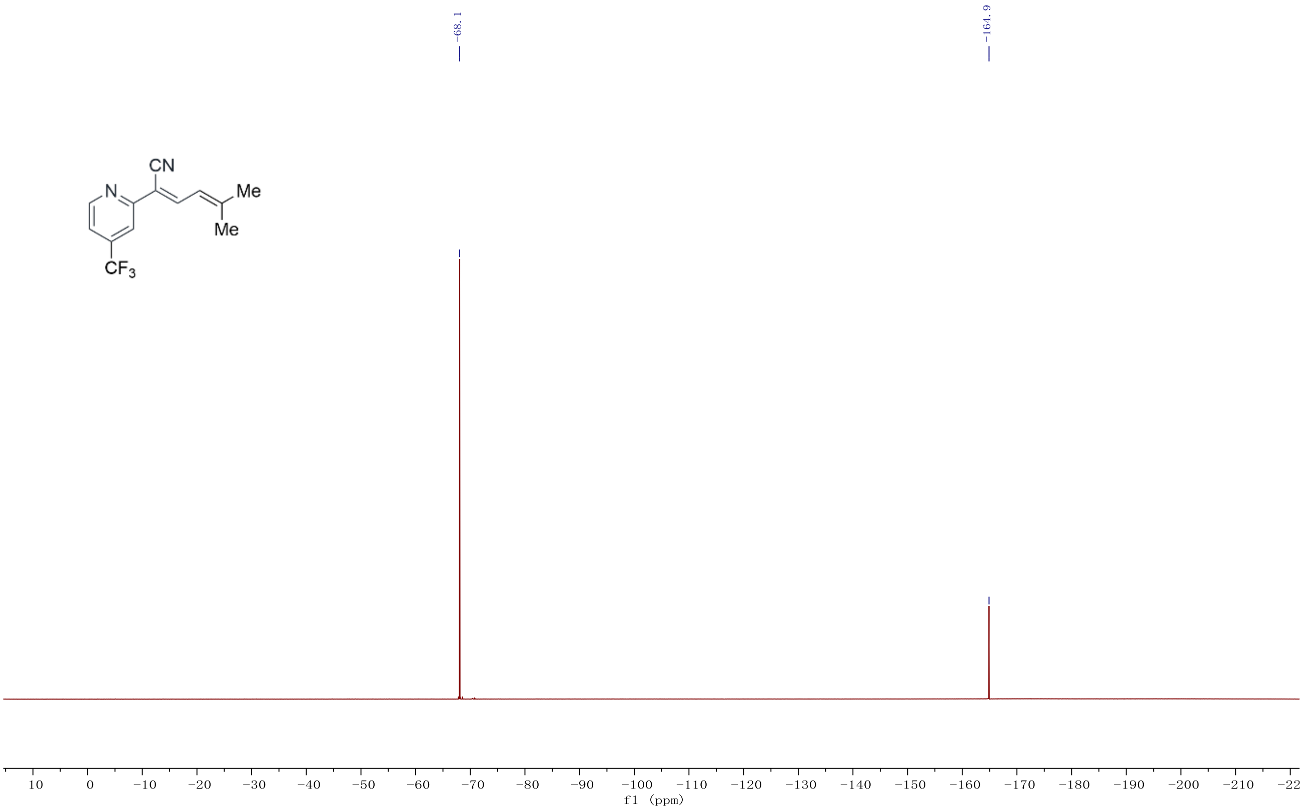

Supplementary Figure 235. ^{1}H NMR spectrum of compound **1ff** (400 MHz, DMSO-d_6)

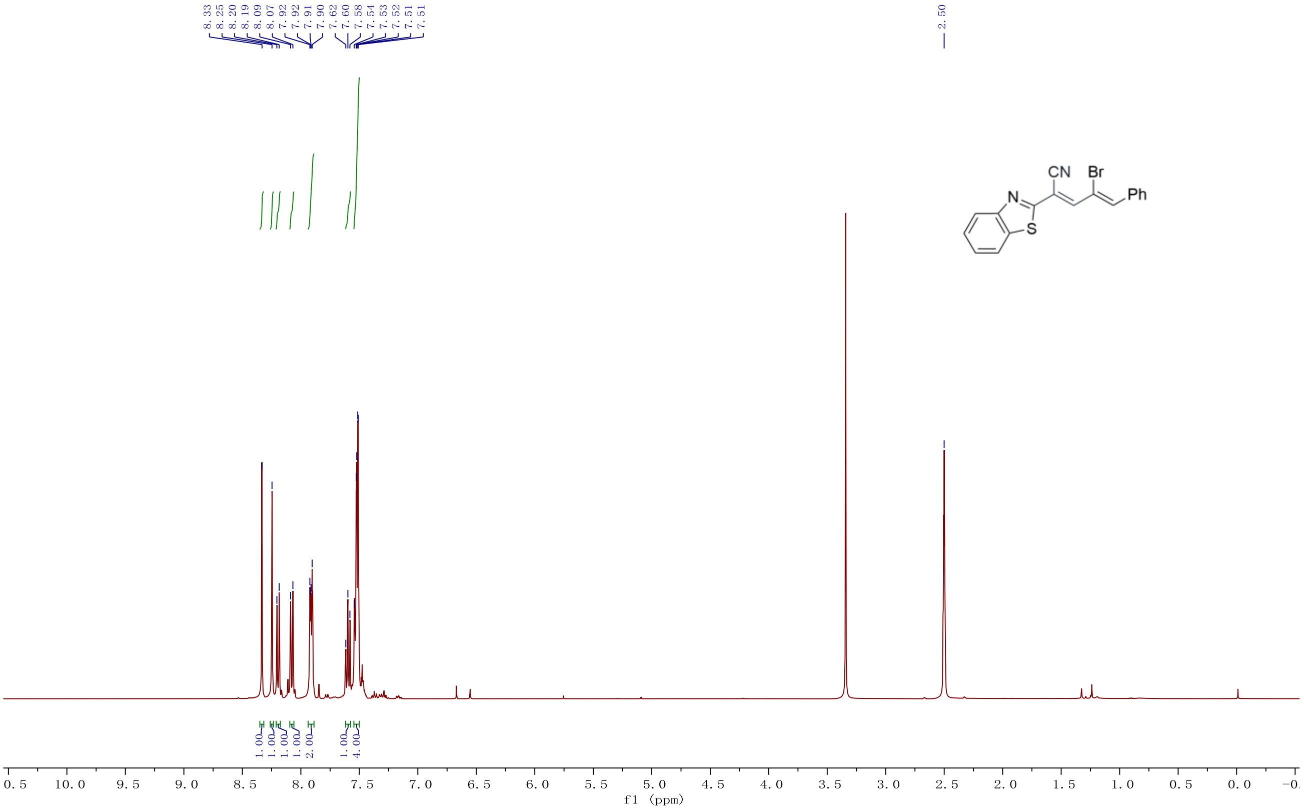

Supplementary Figure 236. ^{13}C NMR spectrum of compound **1ff** (100 MHz, DMSO-d_6)

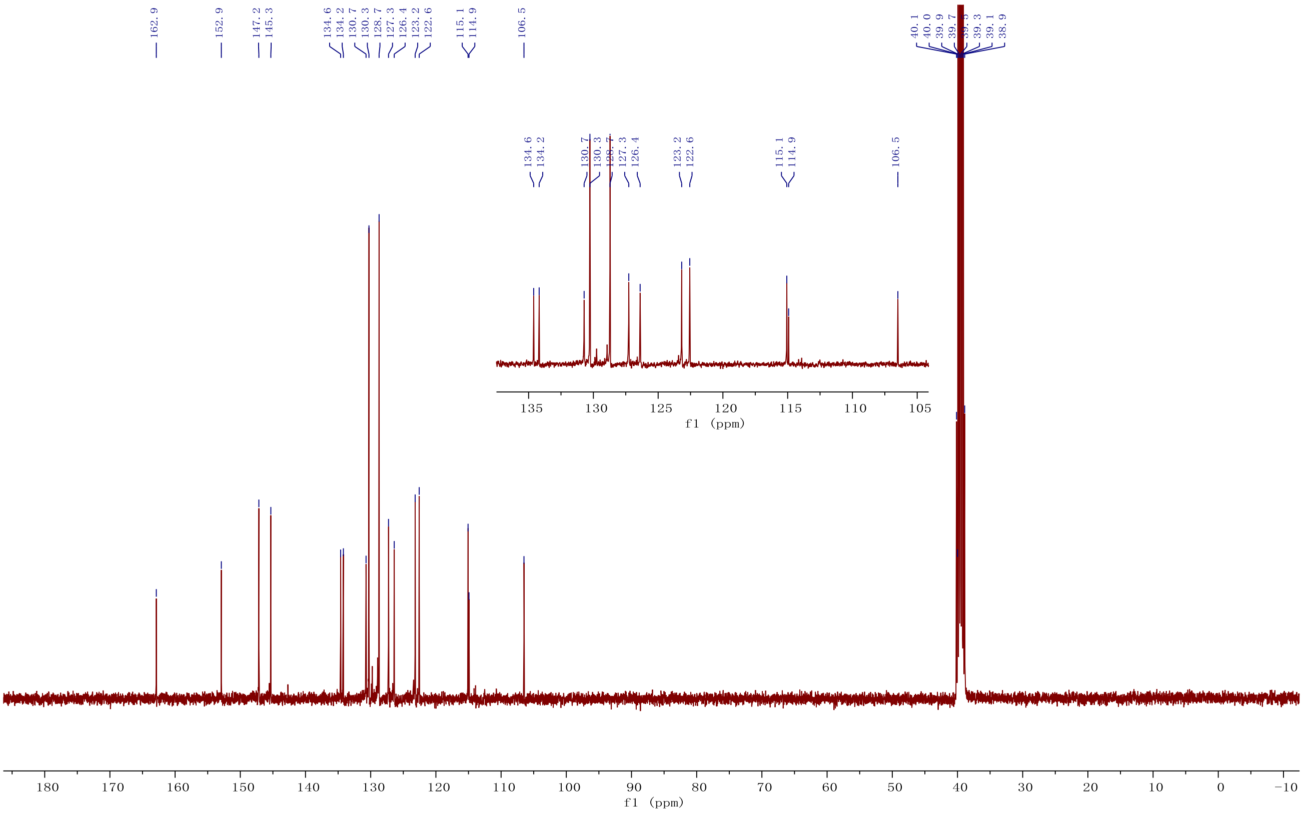

Supplementary Figure 237. ^{1}H NMR spectrum of compound **1fg** (400 MHz, $CDCl_3$)

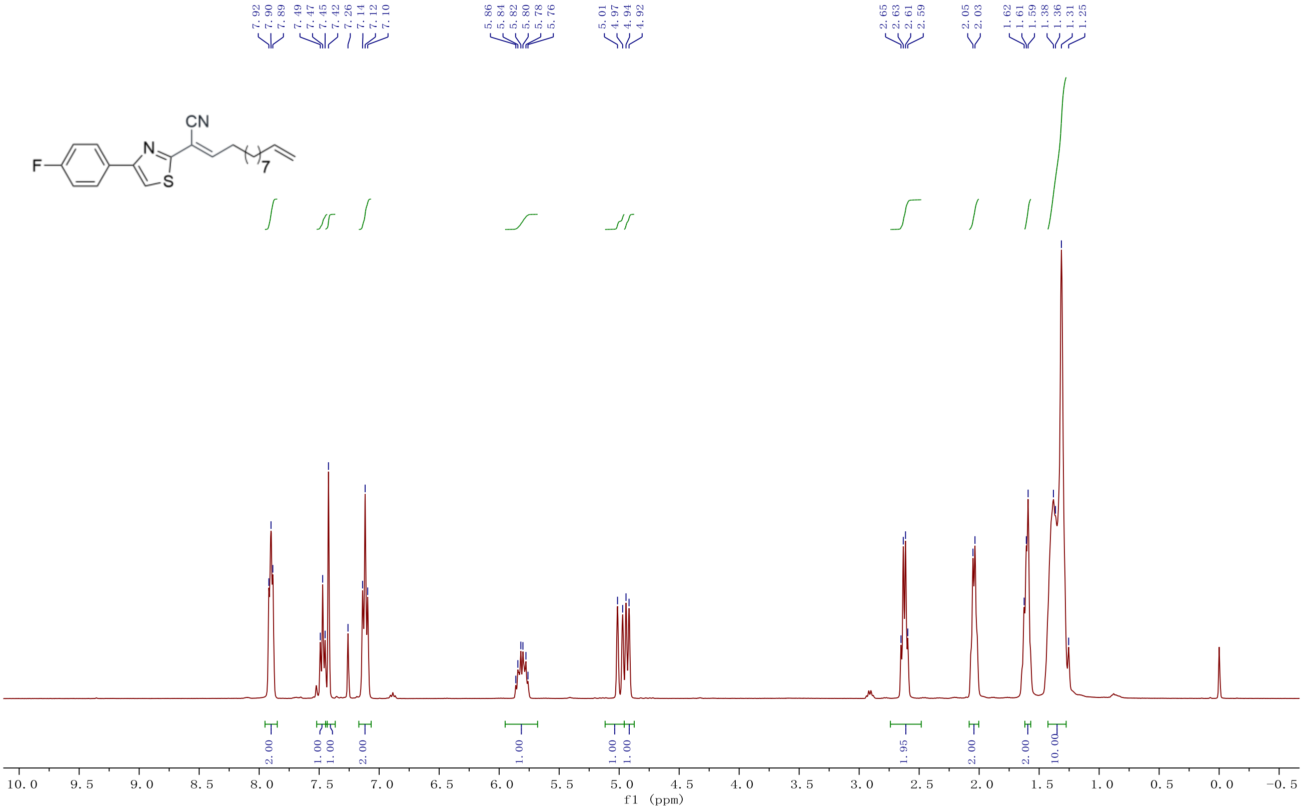

Supplementary Figure 238. ^{13}C NMR spectrum of compound **1fg** (100 MHz, $CDCl_3$)

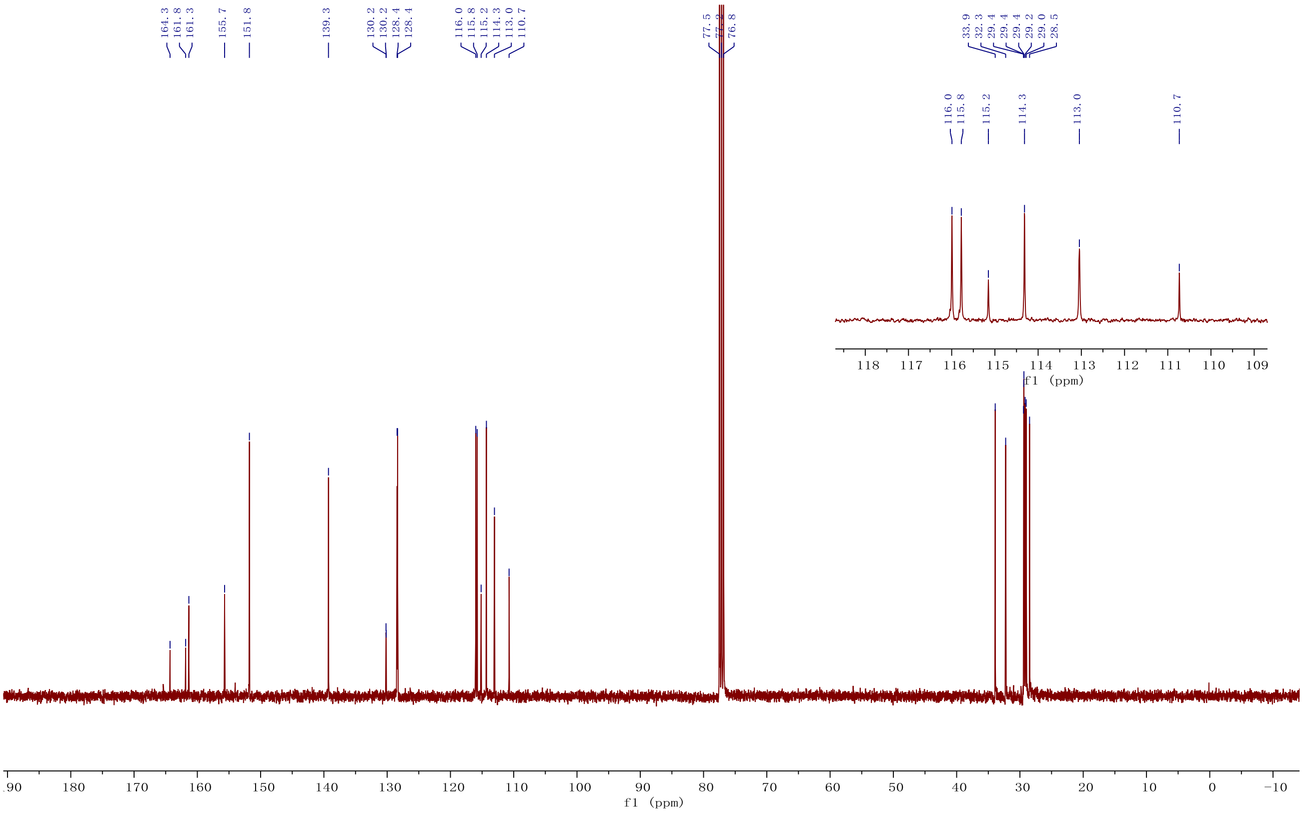

Supplementary Figure 239. ^{19}F NMR spectrum of compound **1fg** (376 MHz, $CDCl_3$)

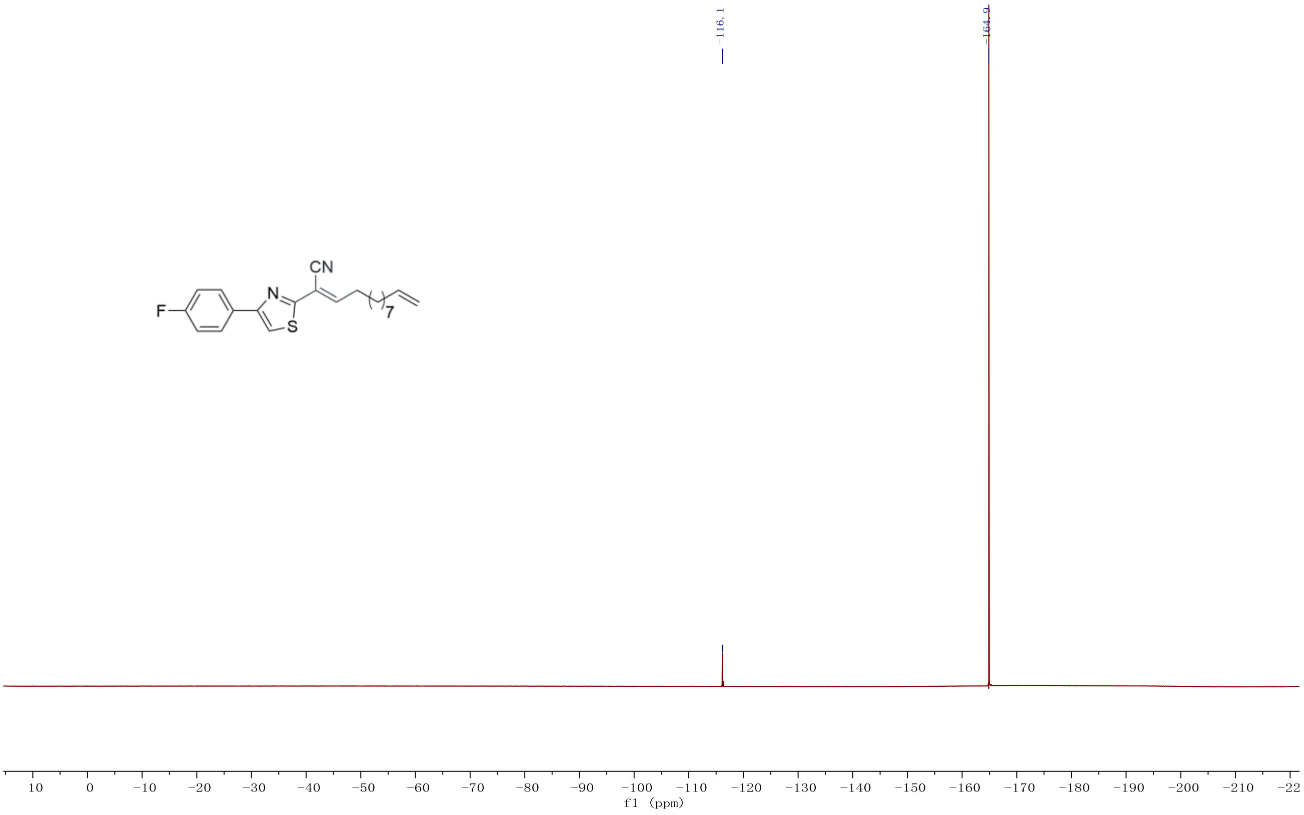

Supplementary Figure 240. ^{1}H NMR spectrum of compound **1fh** (400 MHz, $CDCl_3$)

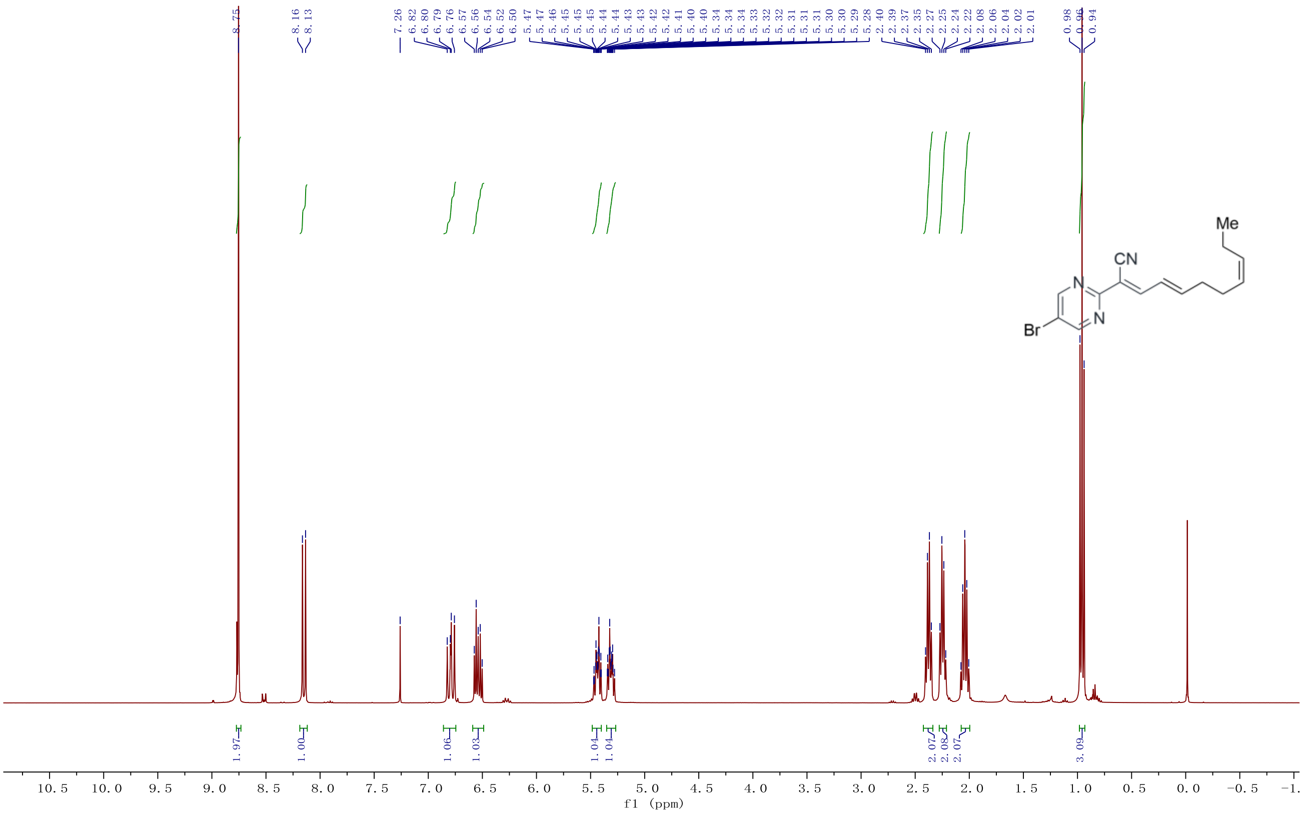

Supplementary Figure 241. ^{13}C NMR spectrum of compound **1fh** (100 MHz, $CDCl_3$)

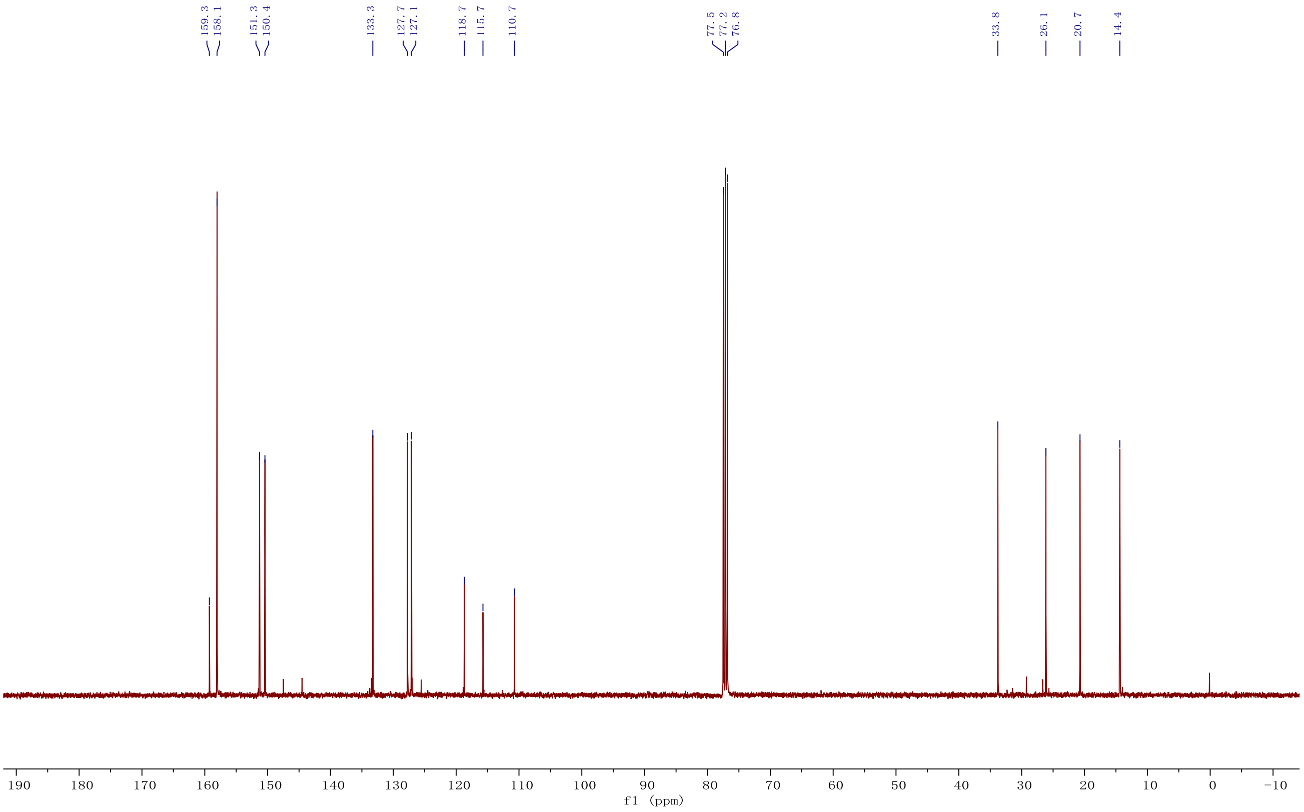

Supplementary Figure 242. ^{1}H NMR spectrum of compound **1fi** (400 MHz, $CDCl_3$)

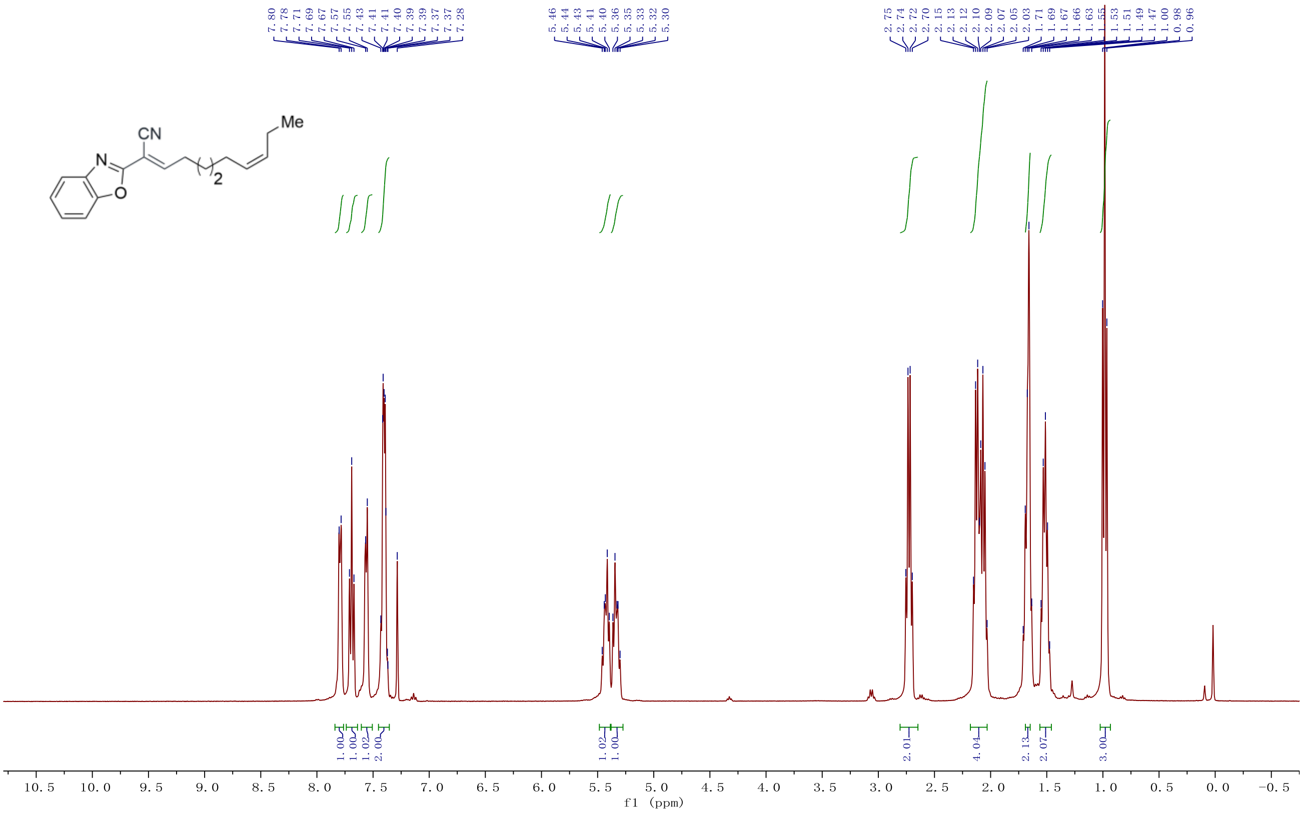

Supplementary Figure 243. ^{13}C NMR spectrum of compound **1fi** (100 MHz, $CDCl_3$)

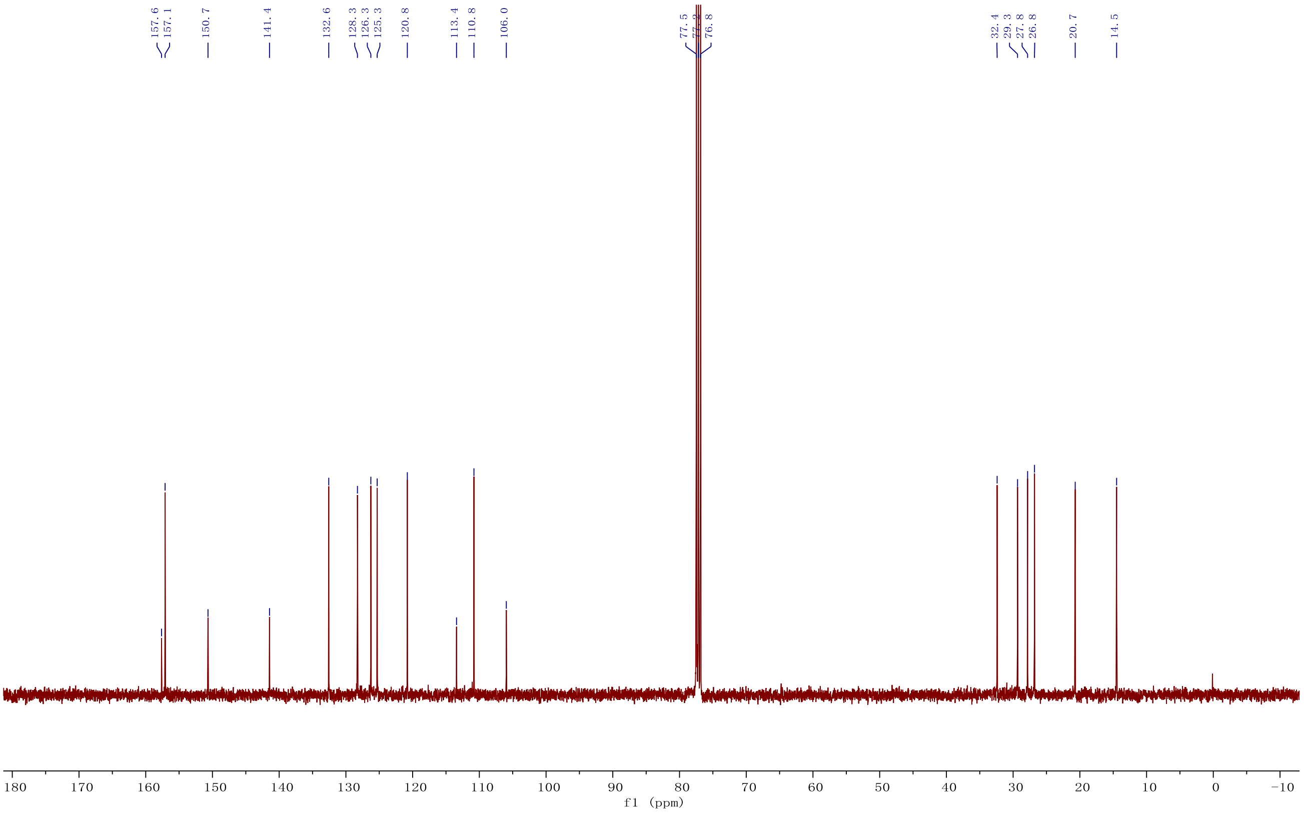

Supplementary Figure 244. ^{1}H NMR spectrum of compound **1ga** (400 MHz, DMSO-d_6)

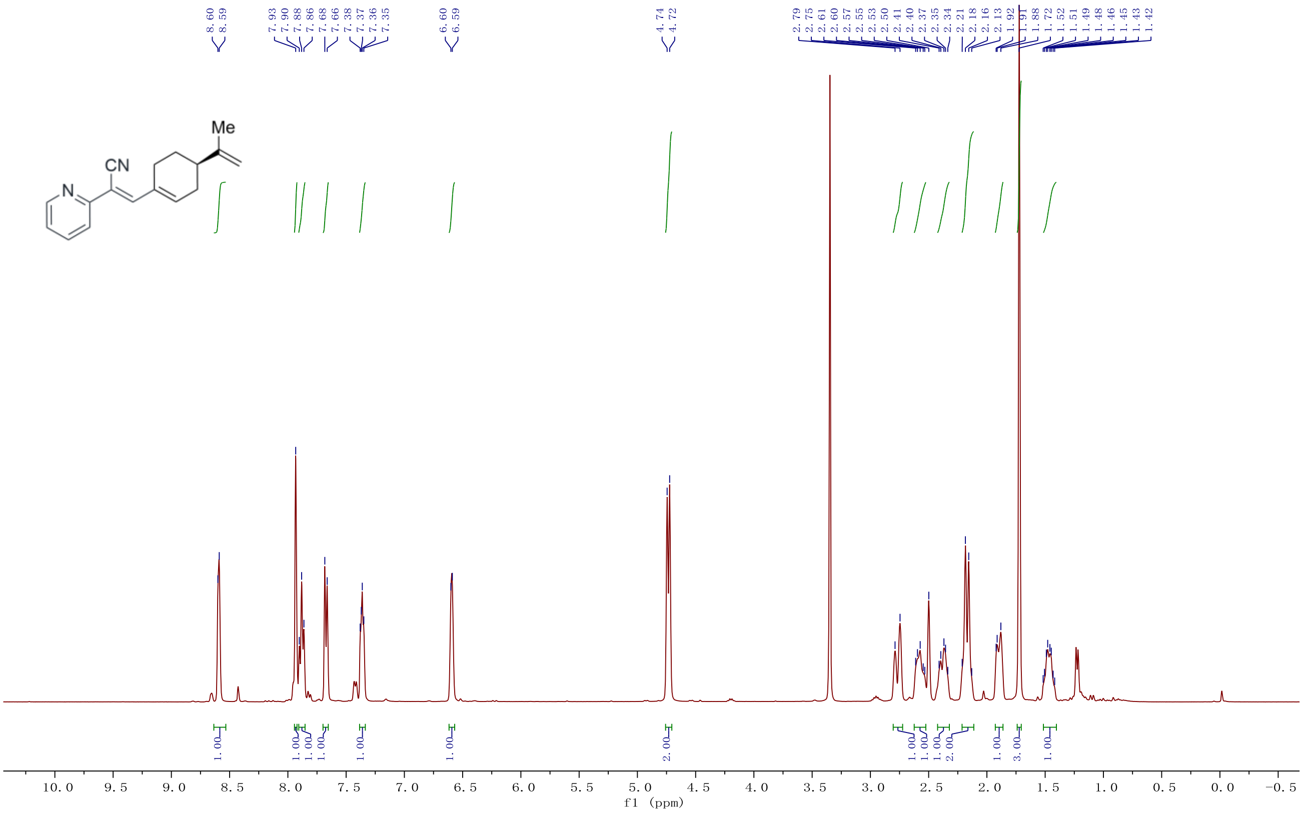

Supplementary Figure 245. ^{13}C NMR spectrum of compound **1ga** (100 MHz, DMSO-d_6)

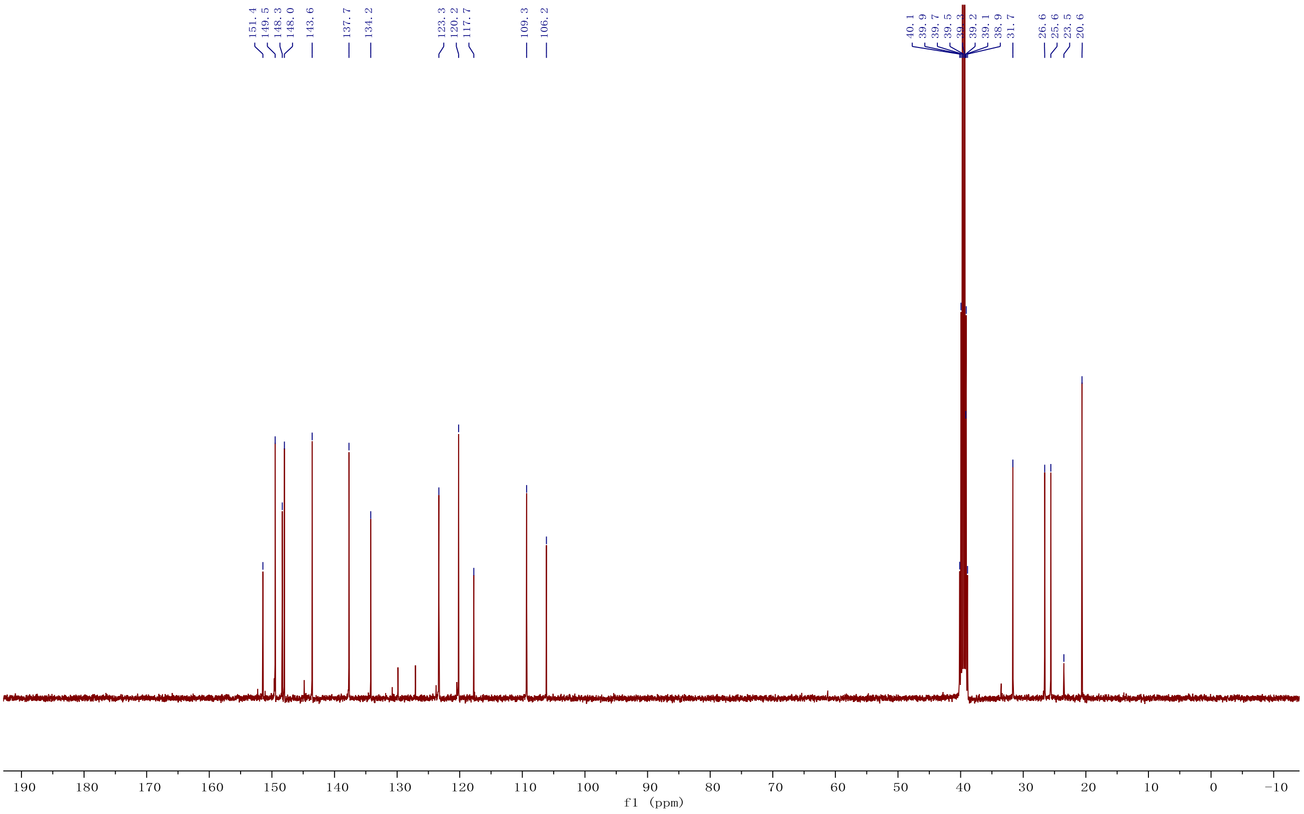

Supplementary Figure 246. ^{1}H NMR spectrum of compound **1gb** (400 MHz, $CDCl_3$)

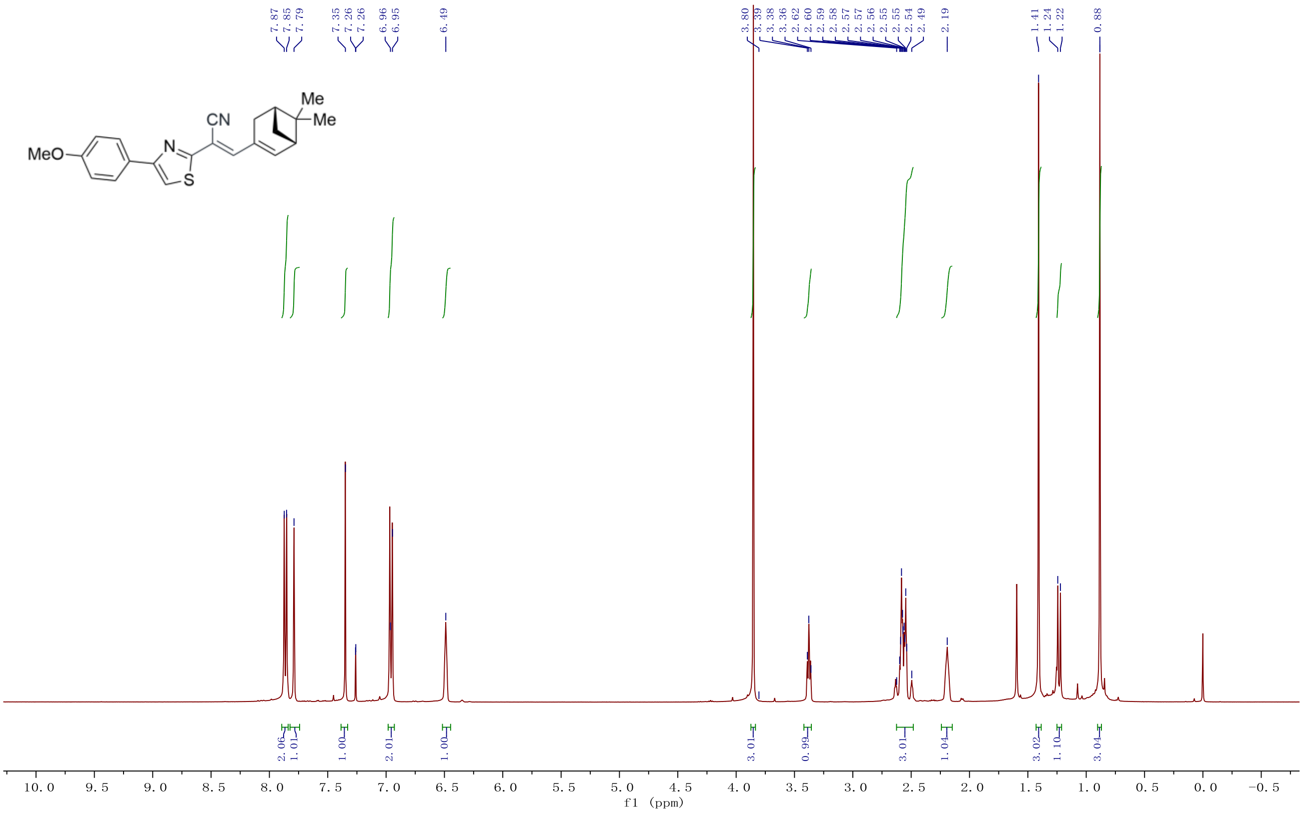

Supplementary Figure 247. ^{13}C NMR spectrum of compound **1gb** (100 MHz, $CDCl_3$)

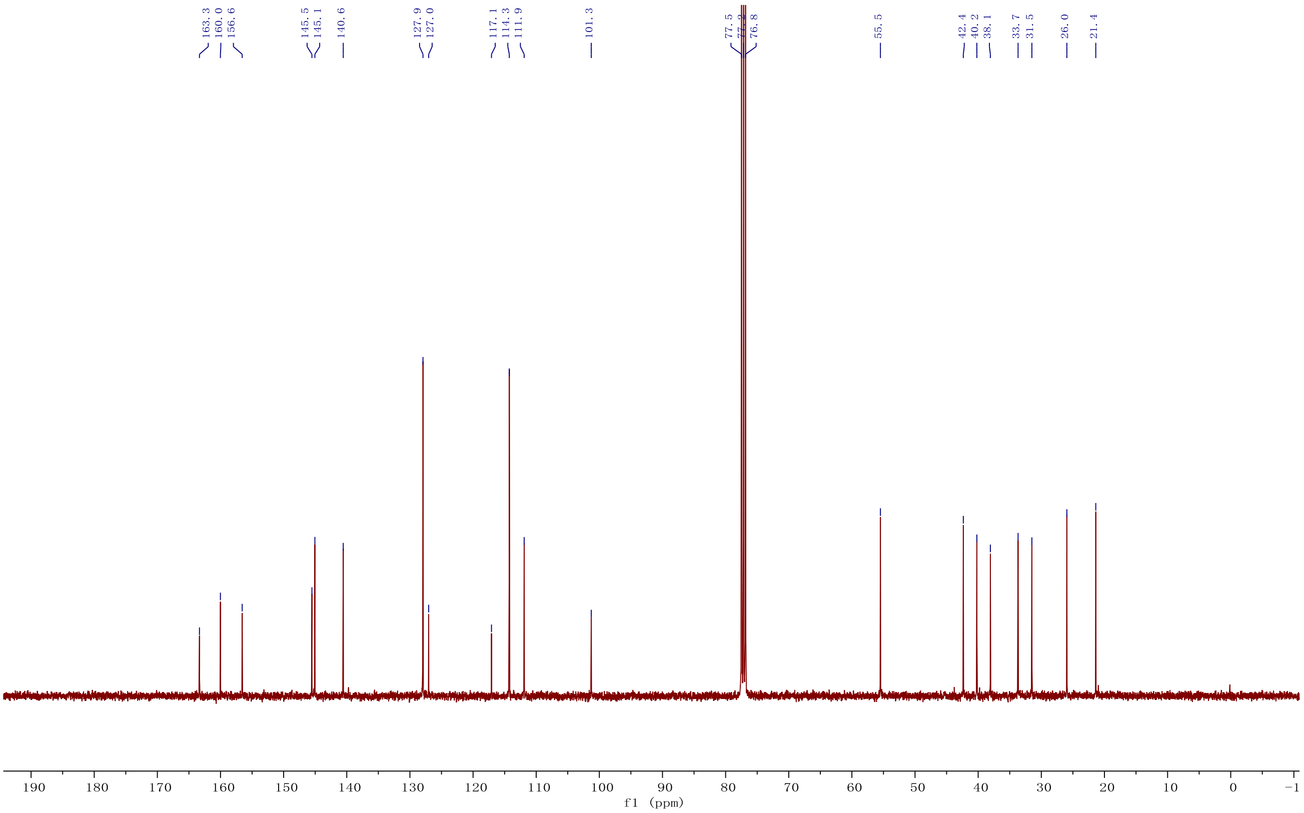

Supplementary Figure 248. ^{1}H NMR spectrum of compound **1gc** (400 MHz, $CDCl_3$)

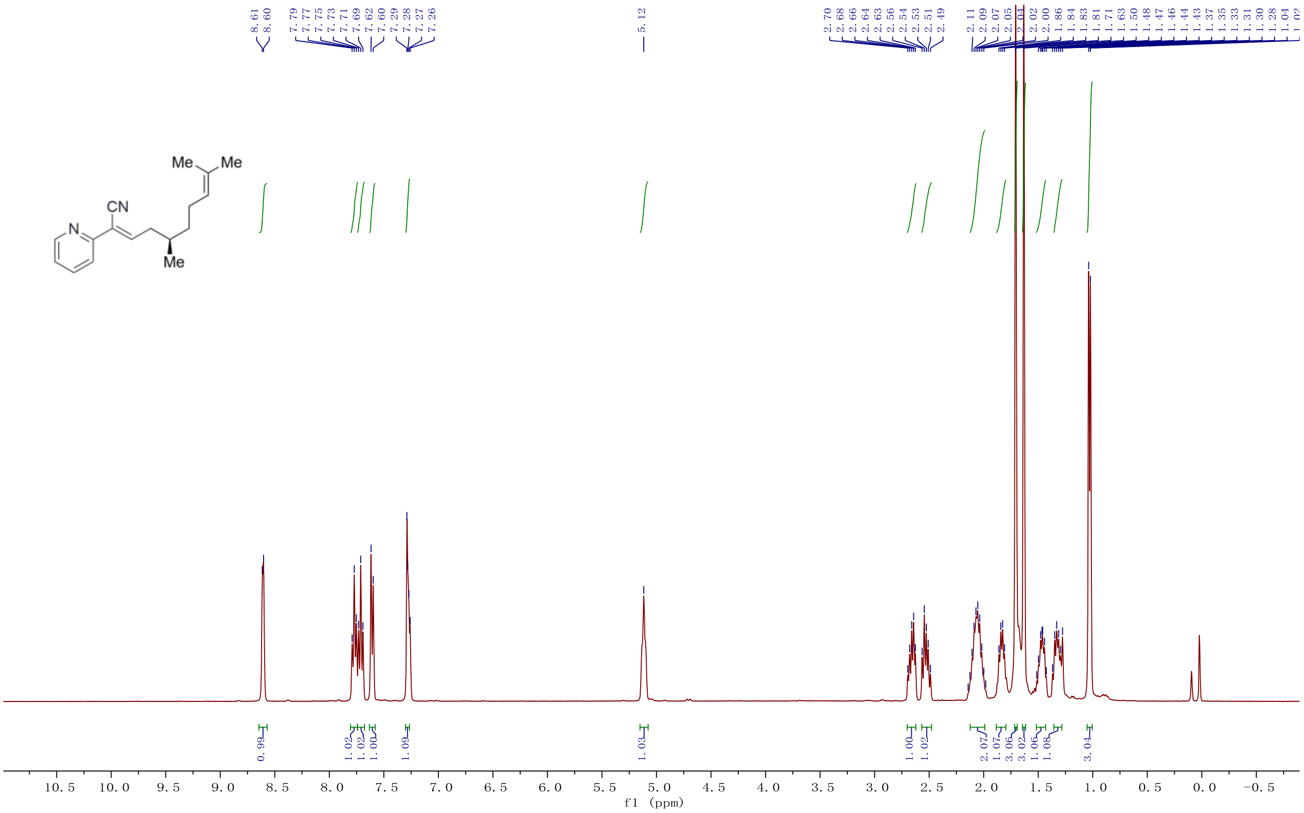

Supplementary Figure 249. ^{13}C NMR spectrum of compound **1gc** (100 MHz, $CDCl_3$)

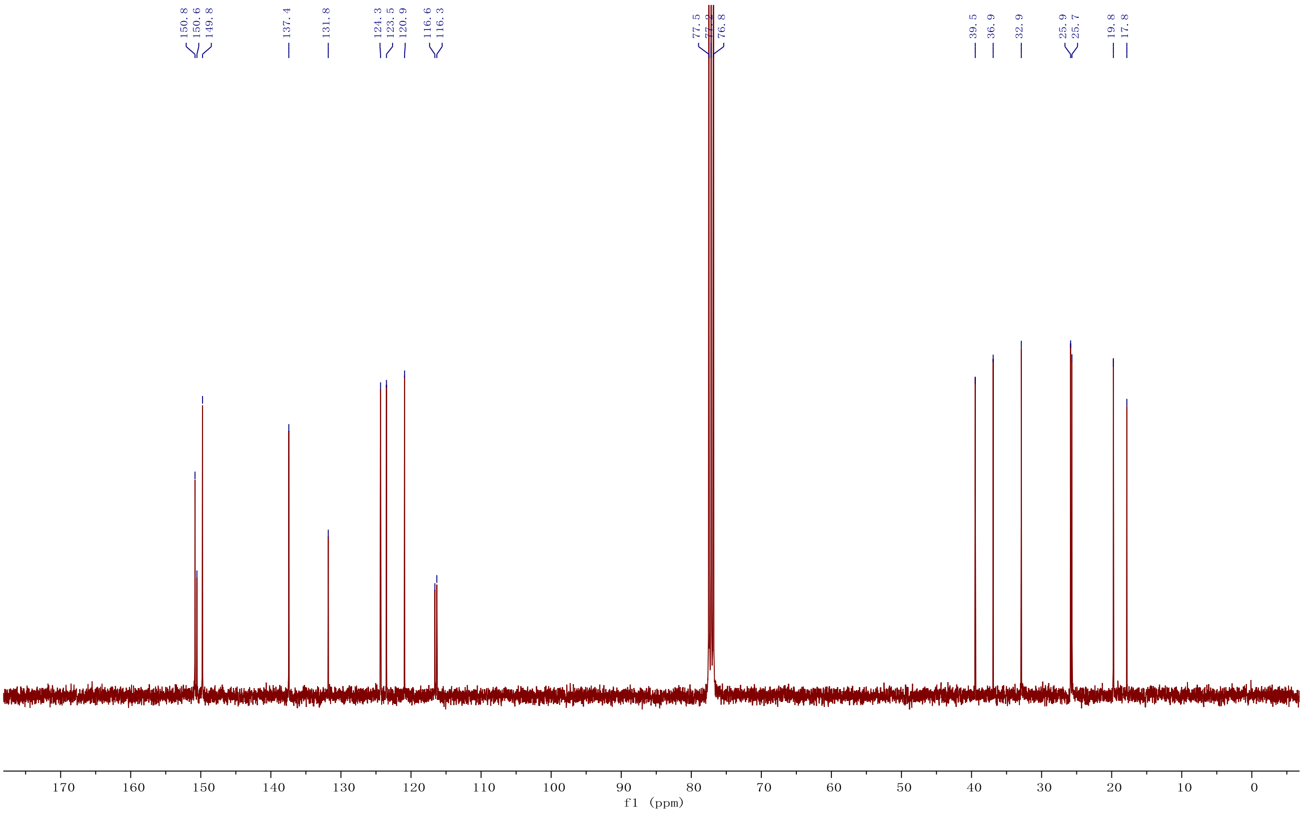

Supplementary Figure 250. ^{1}H NMR spectrum of compound **1gd** (400 MHz, $CDCl_3$)

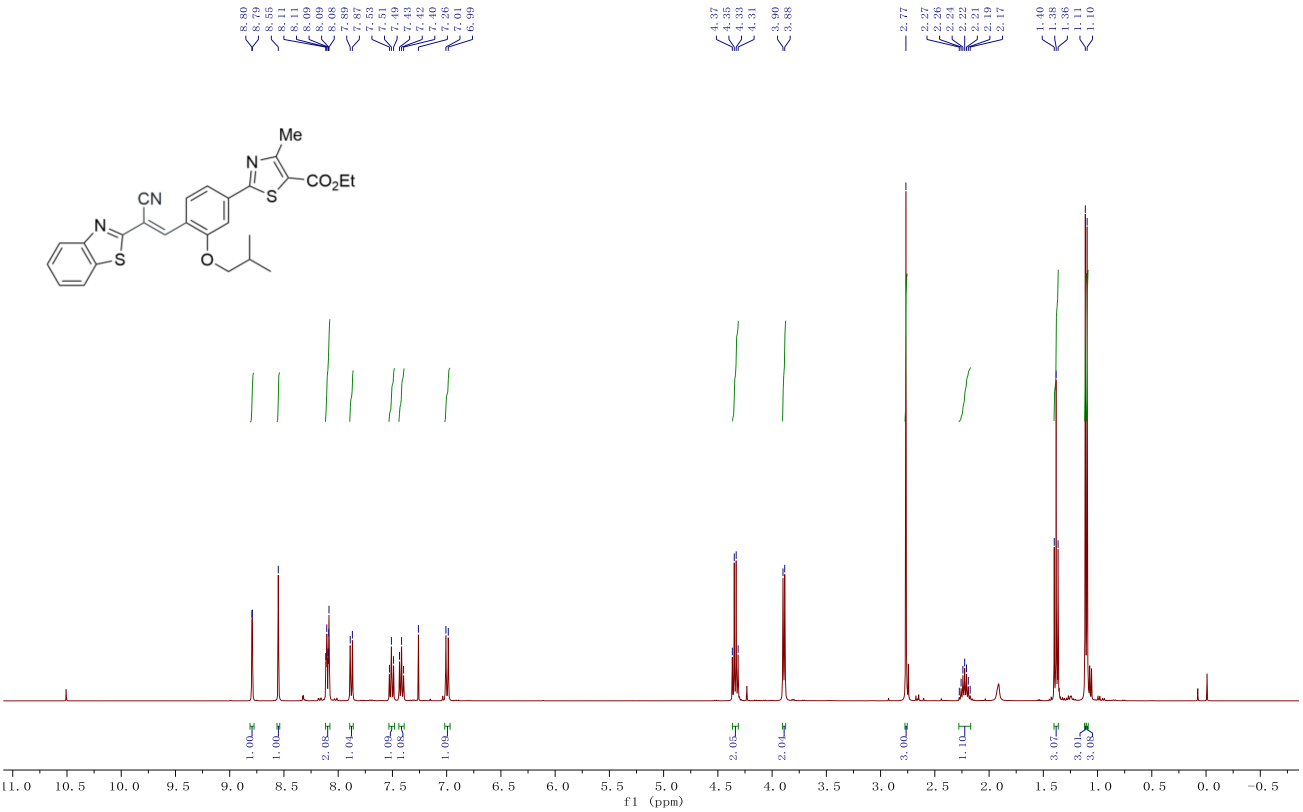

Supplementary Figure 251. ^{13}C NMR spectrum of compound **1gd** (100 MHz, $CDCl_3$)

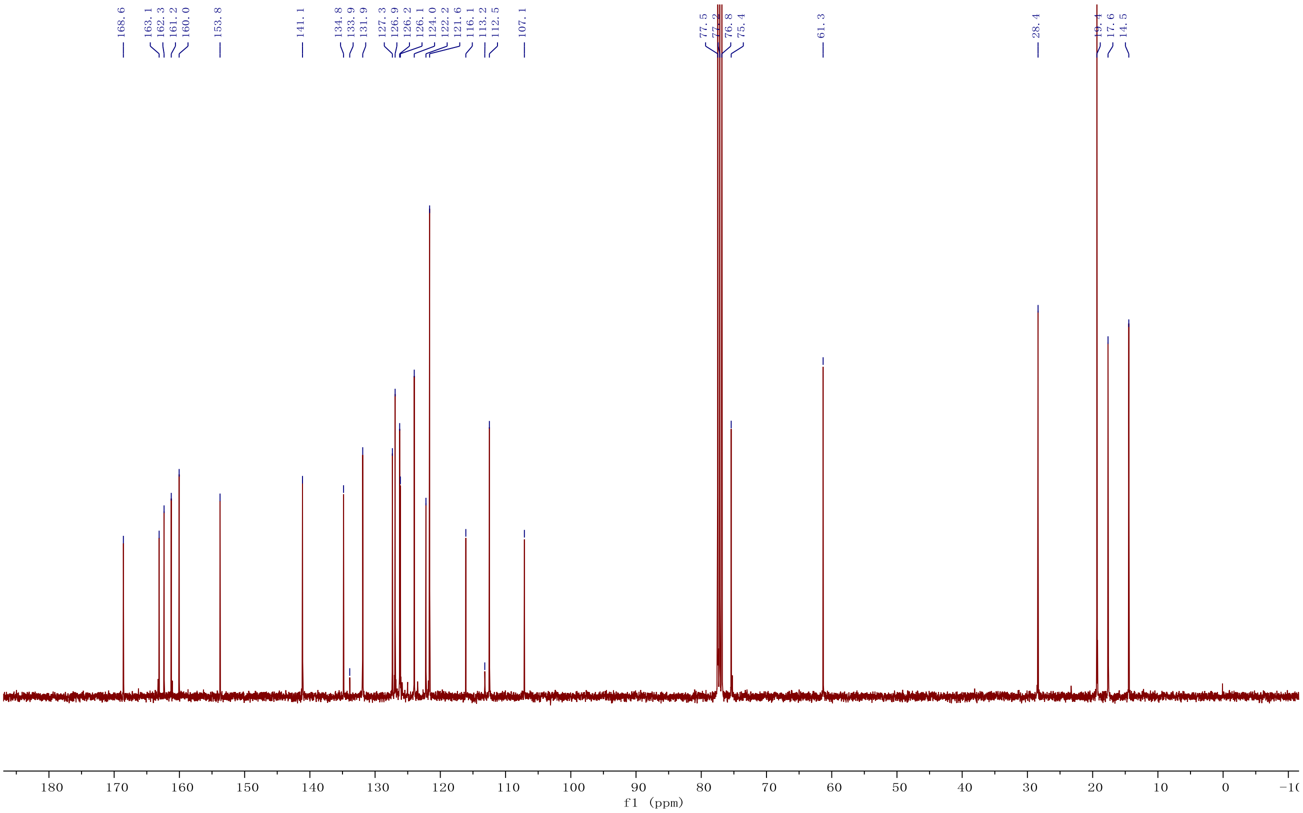

Supplementary Figure 252. ^{1}H NMR spectrum of compound **1ge** (600 MHz, $CDCl_3$)

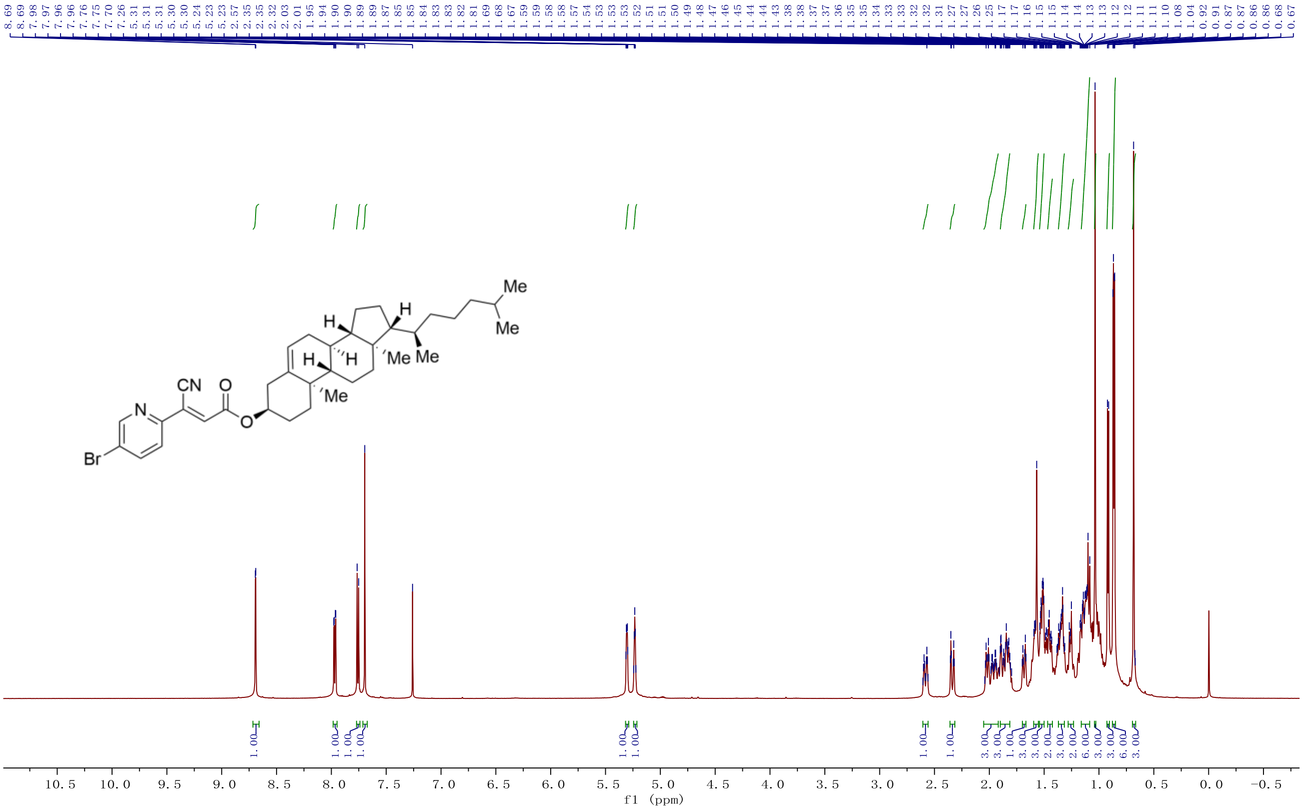

Supplementary Figure 253. ^{13}C NMR spectrum of compound **1ge** (150 MHz, $CDCl_3$)

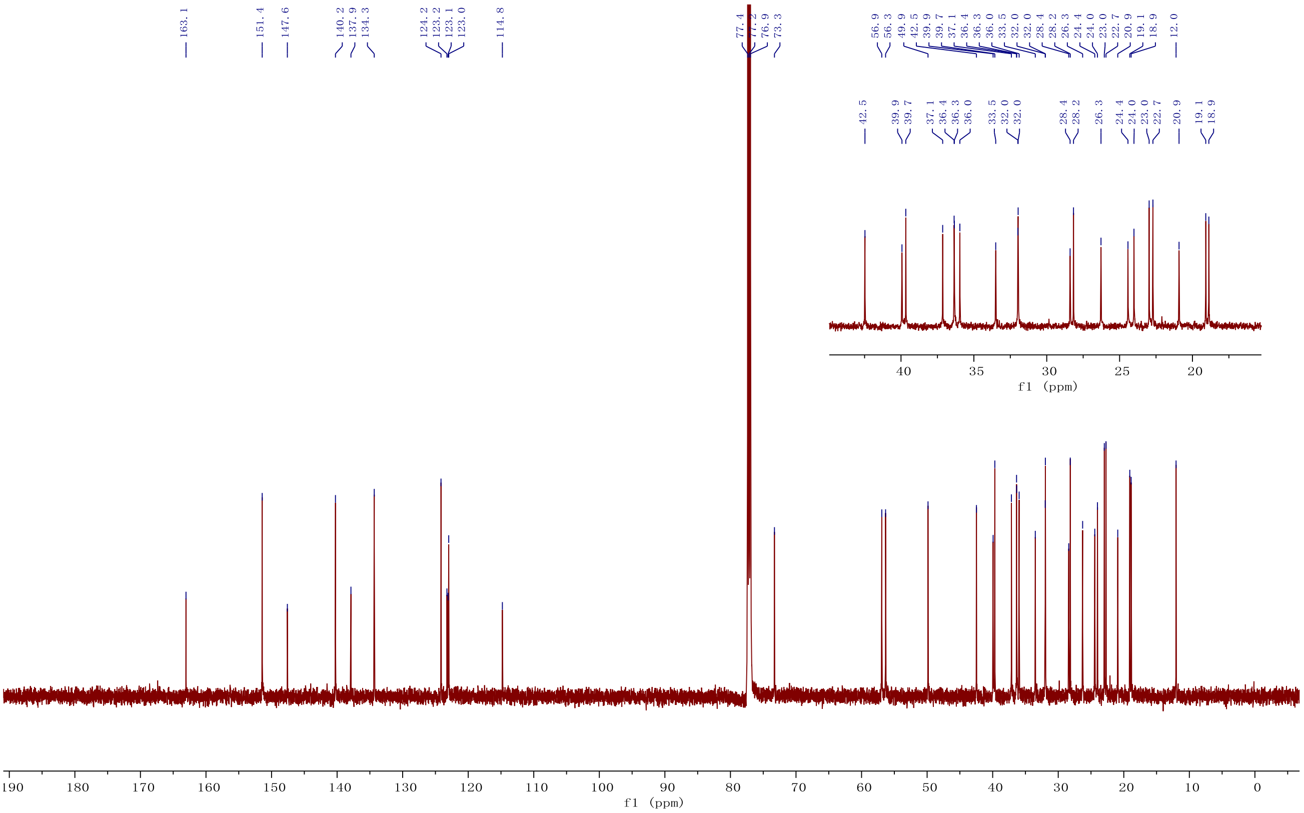

Supplementary Figure 254. ^{1}H NMR spectrum of compound **1gf** (400 MHz, $CDCl_3$)

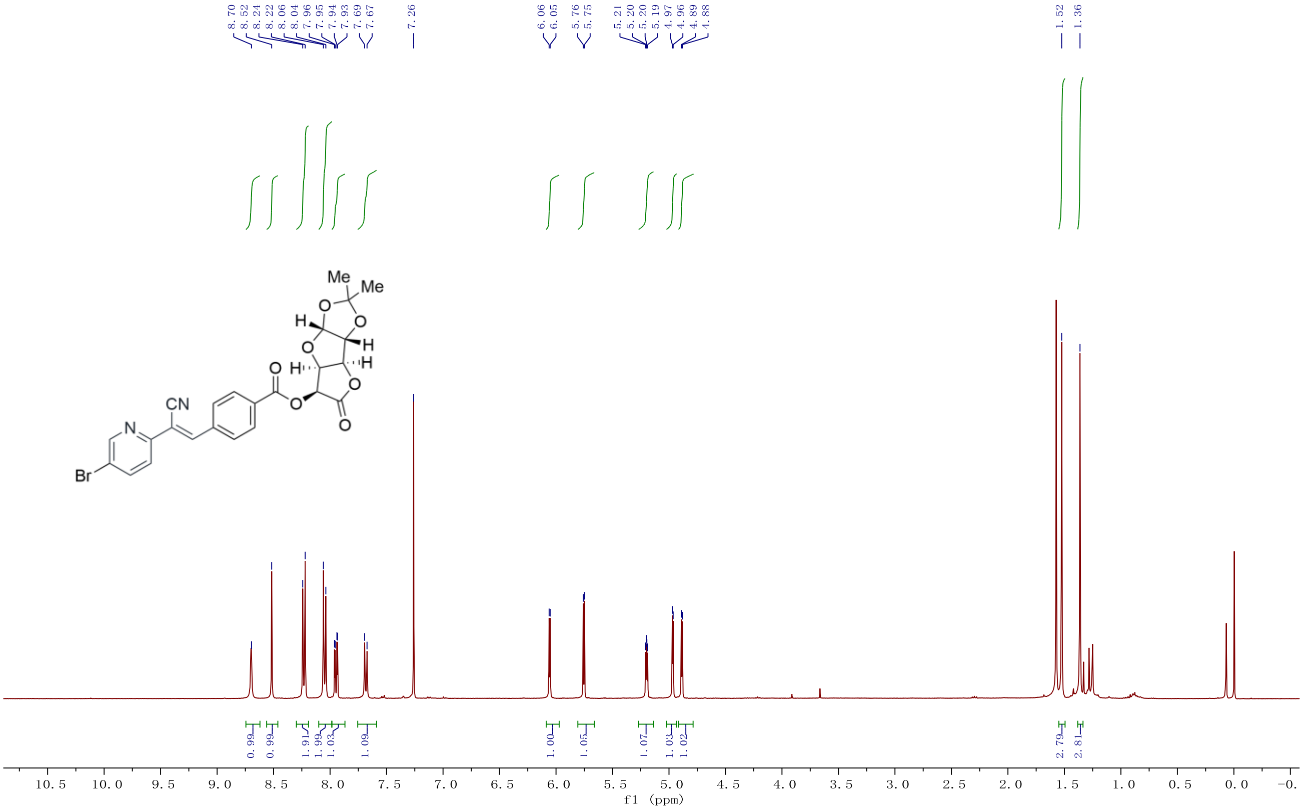

Supplementary Figure 255. ^{13}C NMR spectrum of compound **1gf** (100 MHz, $CDCl_3$)

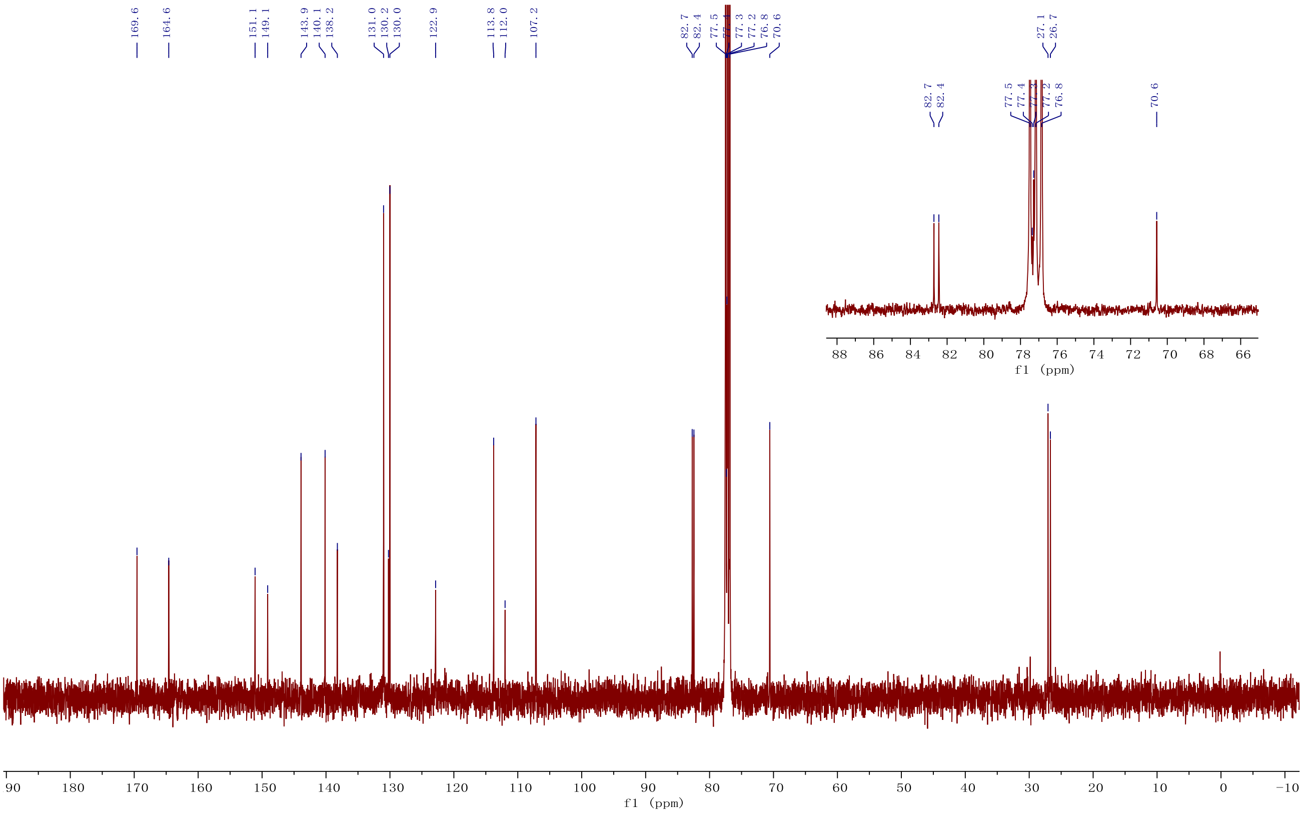

Supplementary Figure 256. ^{1}H NMR spectrum of compound **1ea** (400 MHz, $CDCl_3$)

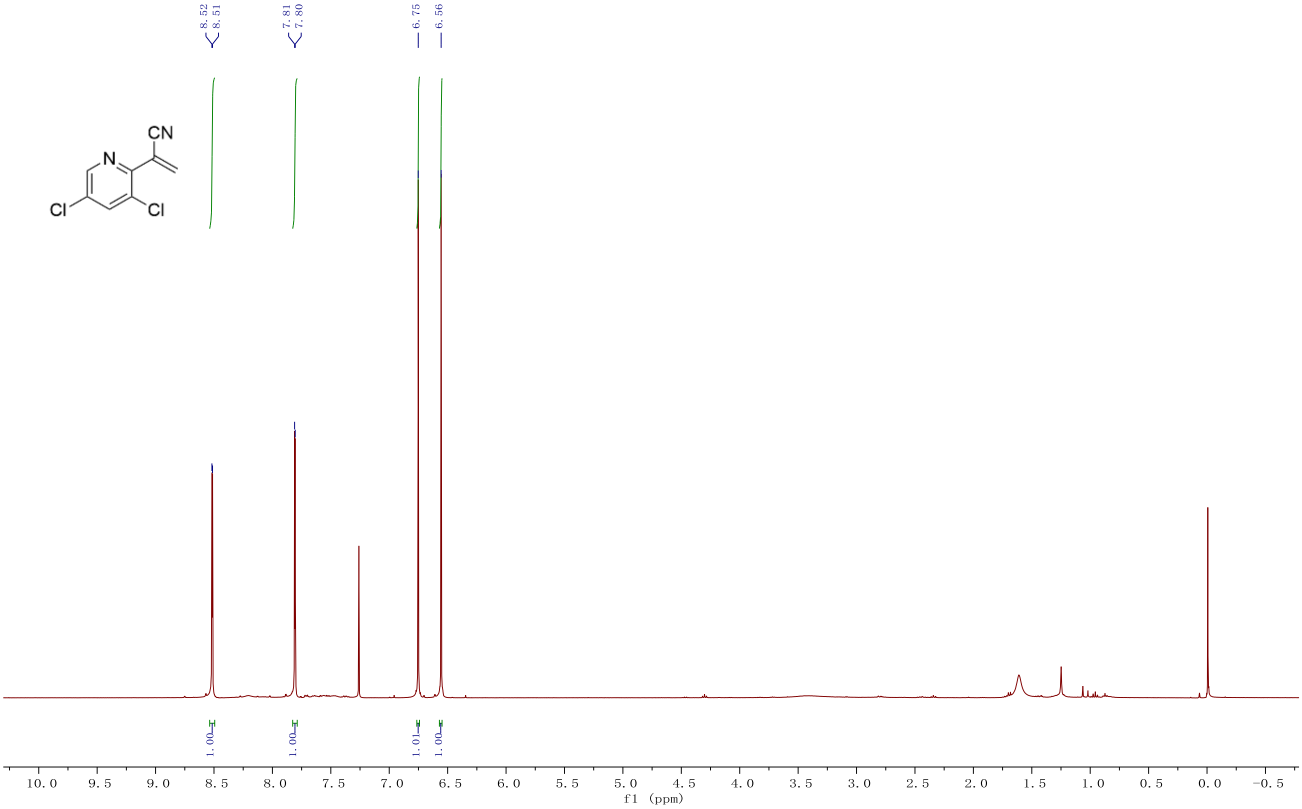

Supplementary Figure 257. ^{13}C NMR spectrum of compound **1ea** (100 MHz, $CDCl_3$)

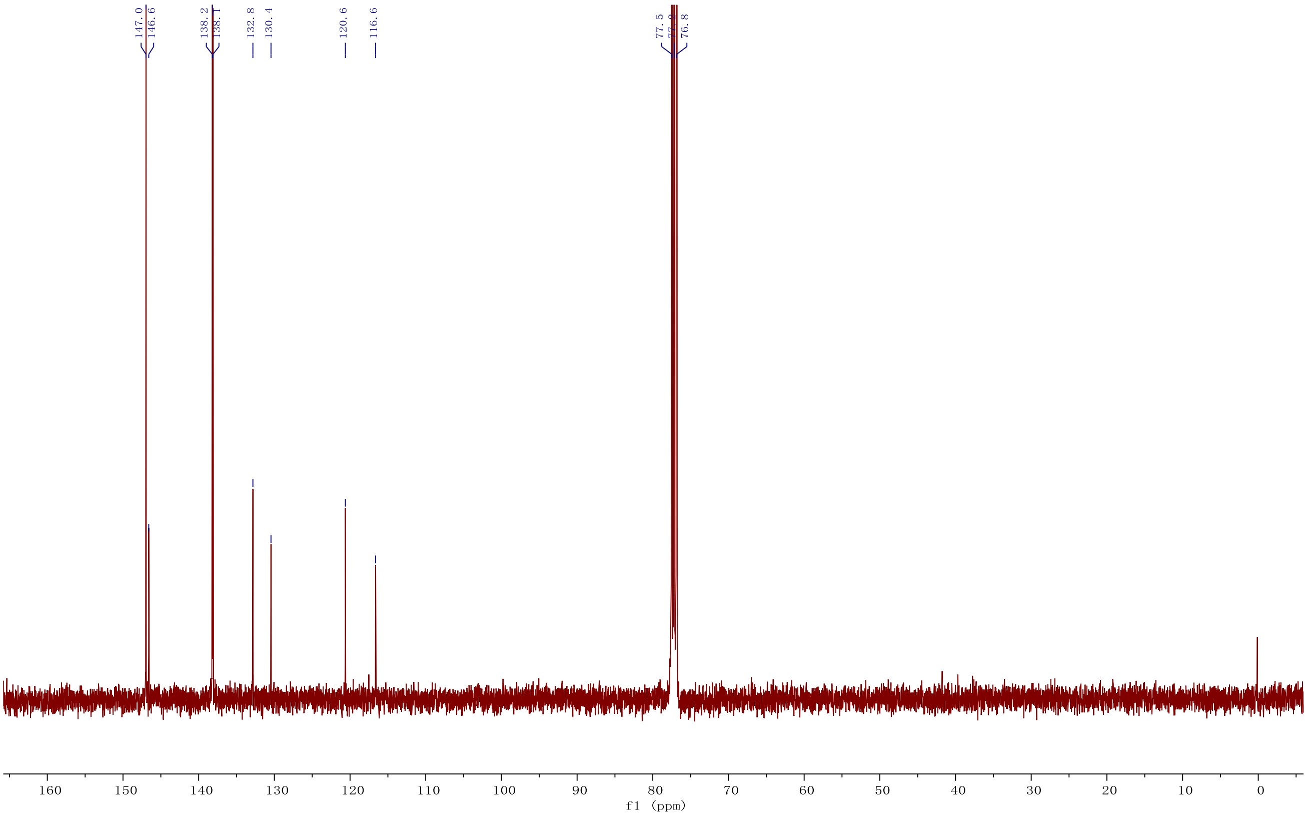

Supplementary Figure 258. ^{1}H NMR spectrum of compound **1ec** (600 MHz, $CDCl_3$)

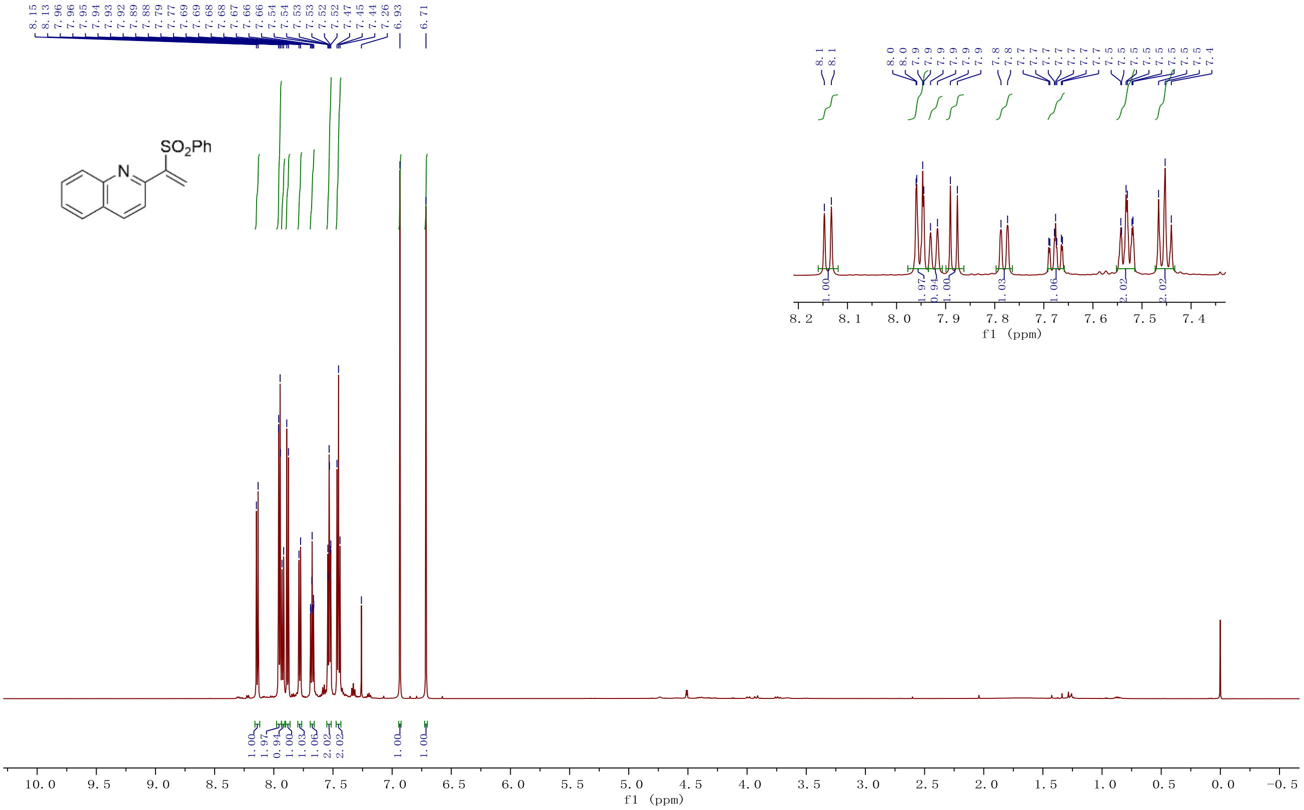

Supplementary Figure 259. ^{13}C NMR spectrum of compound **1ec** (150 MHz, $CDCl_3$)

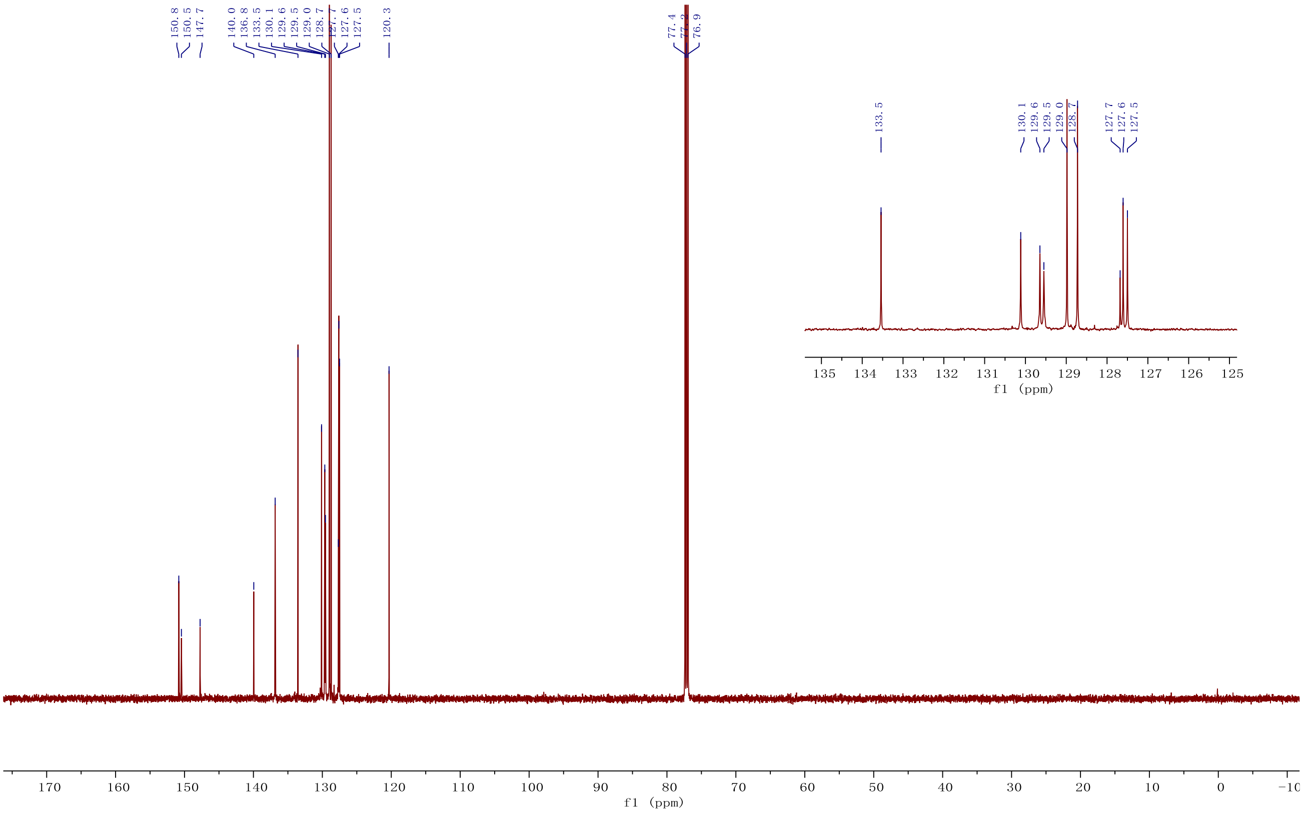

Supplementary Figure 260. ^{1}H NMR spectrum of compound **1eb** (400 MHz, $CDCl_3$)

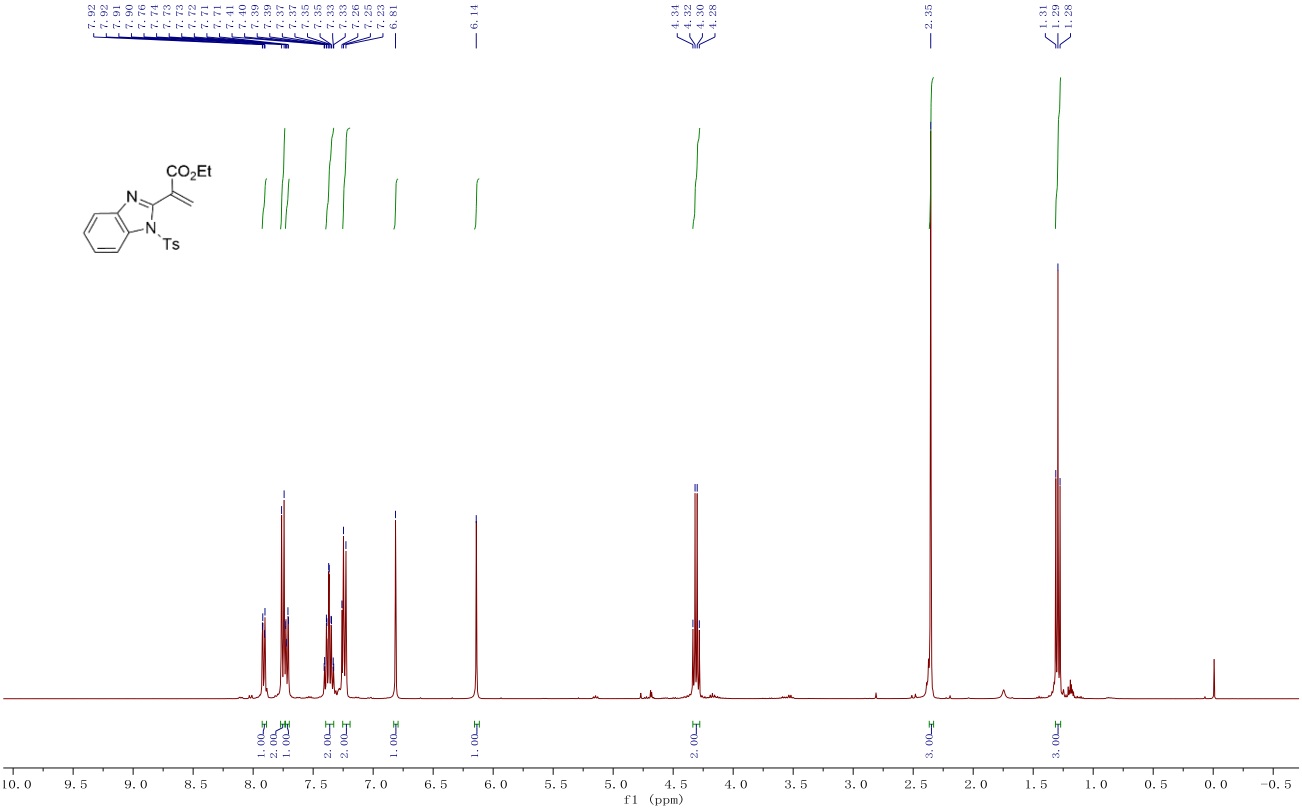

Supplementary Figure 261. ^{13}C NMR spectrum of compound **1eb** (100 MHz, $CDCl_3$)

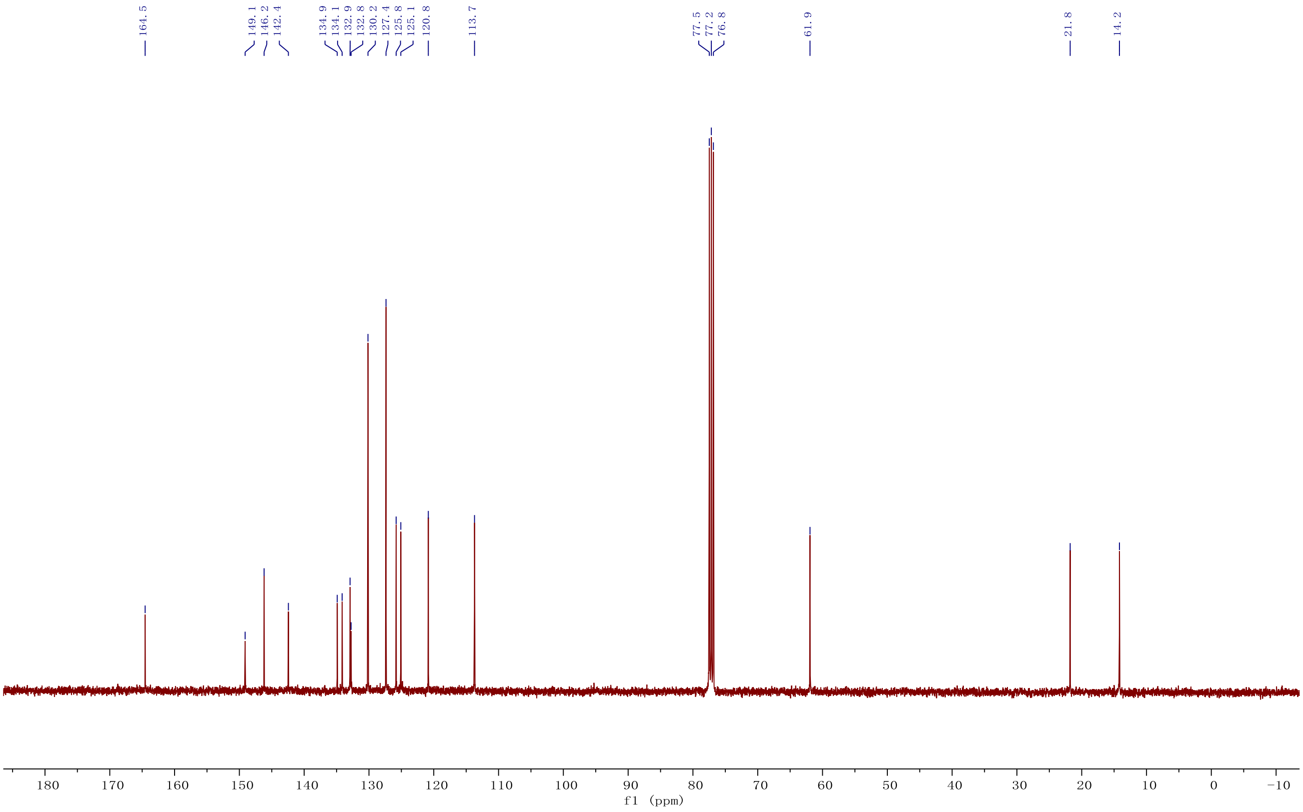

Supplementary Figure 262. ^{1}H NMR spectrum of compound **5g** (600 MHz, $CDCl_3$)

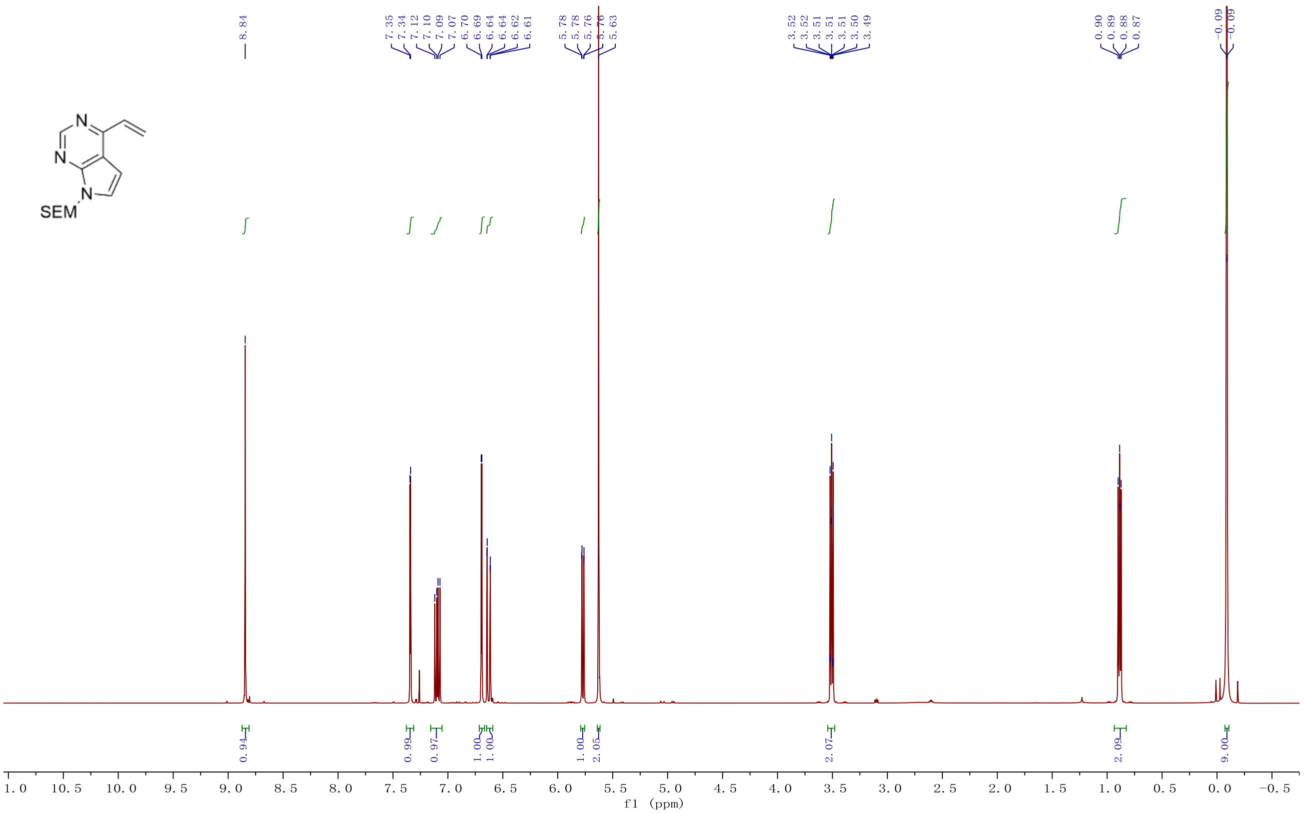

Supplementary Figure 263. ^{13}C NMR spectrum of compound **5g** (150 MHz, $CDCl_3$)

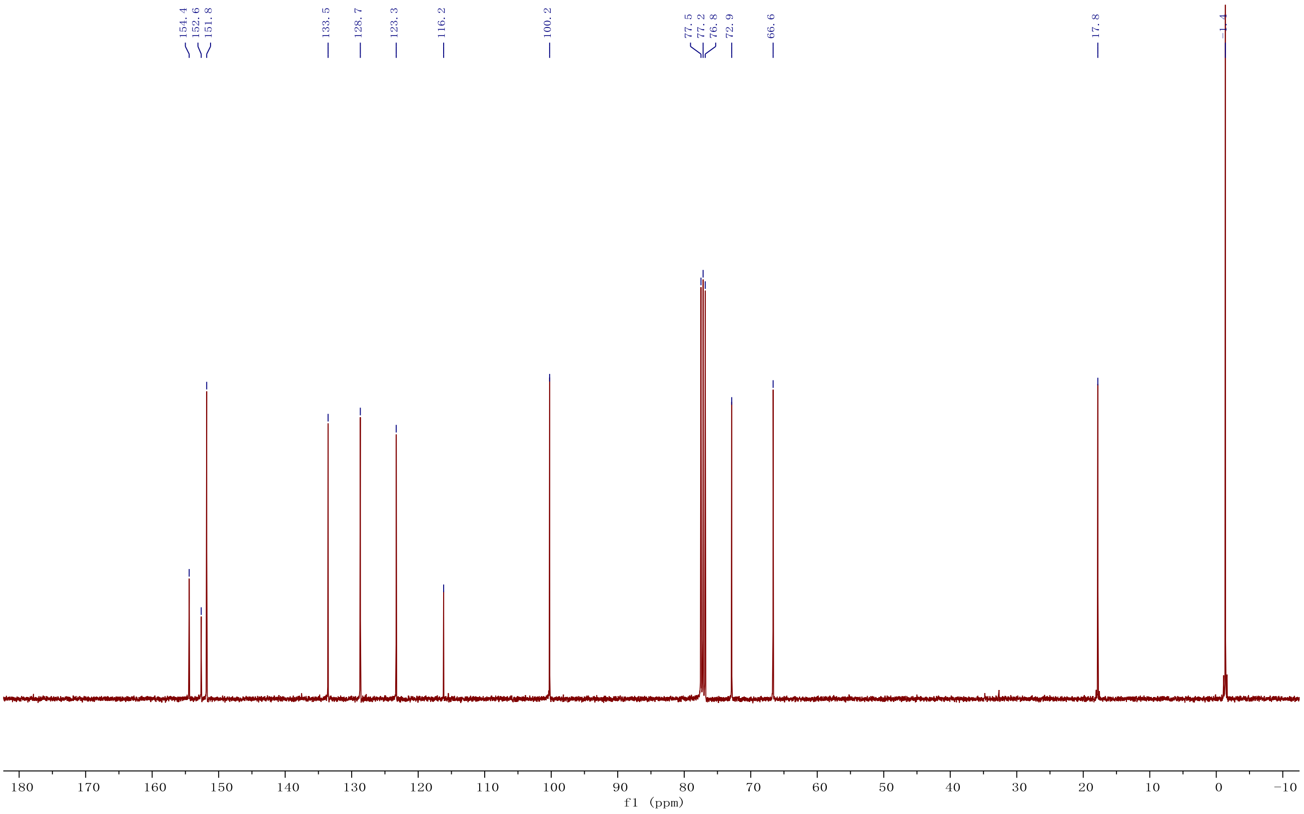

Supplementary Figure 264. ^{1}H NMR spectrum of compound **5j** (400 MHz, $CDCl_3$)

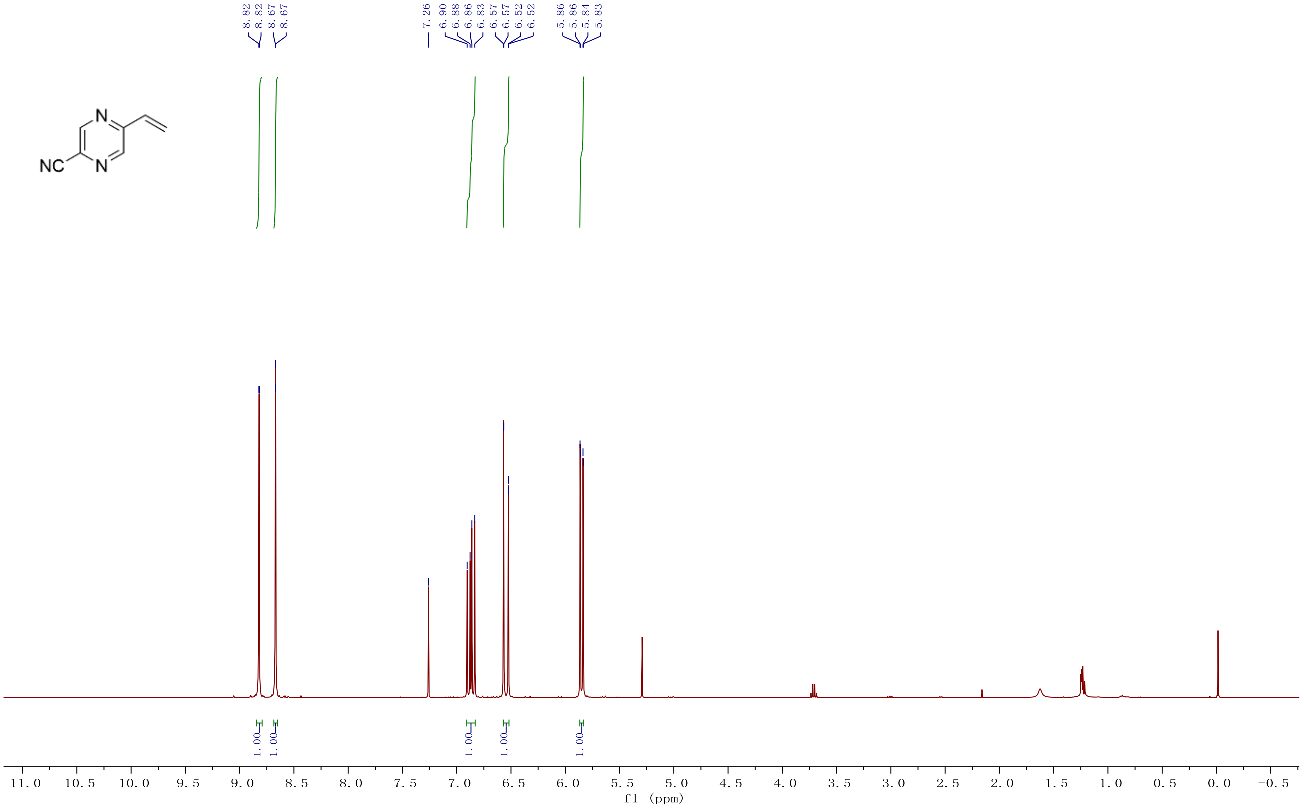

Supplementary Figure 265. ^{13}C NMR spectrum of compound **5j** (100 MHz, $CDCl_3$)

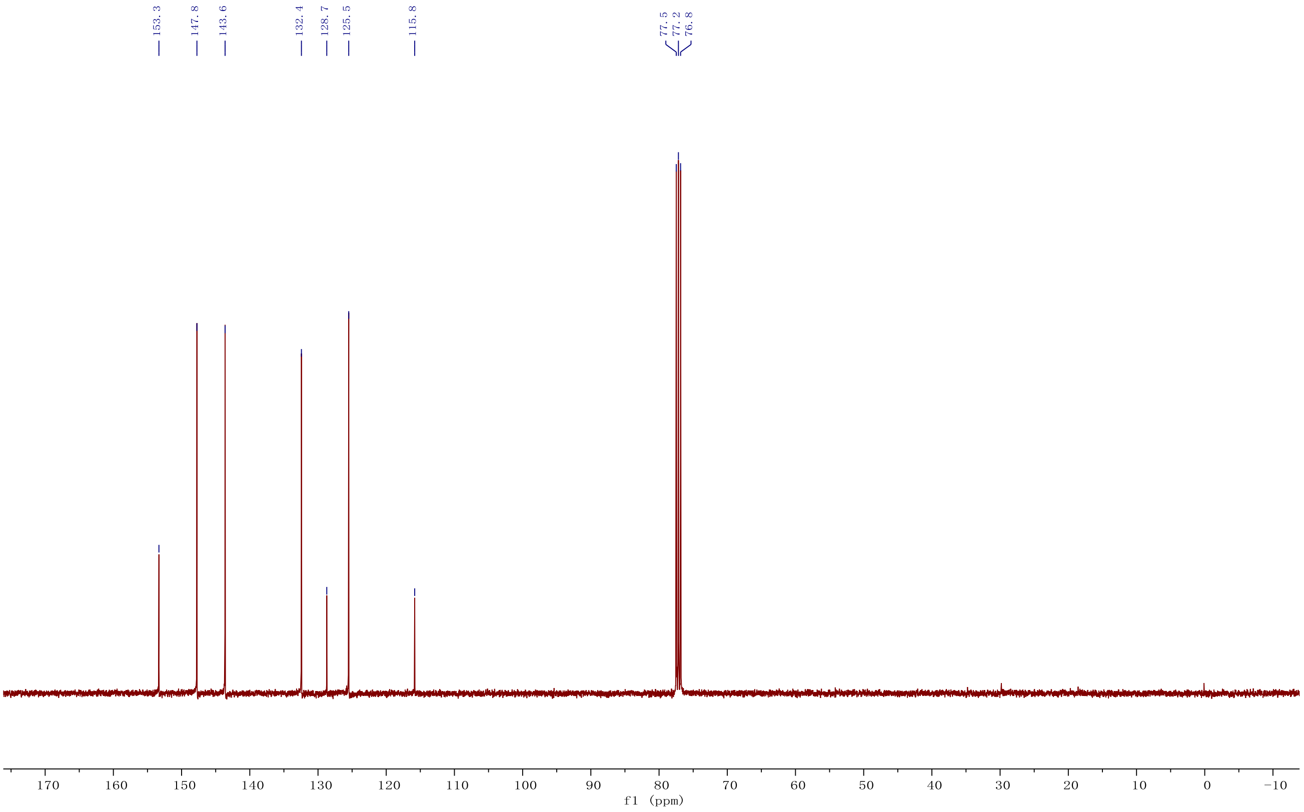

Supplementary Figure 266. ^{1}H NMR spectrum of compound **5k** (400 MHz, $CDCl_3$)

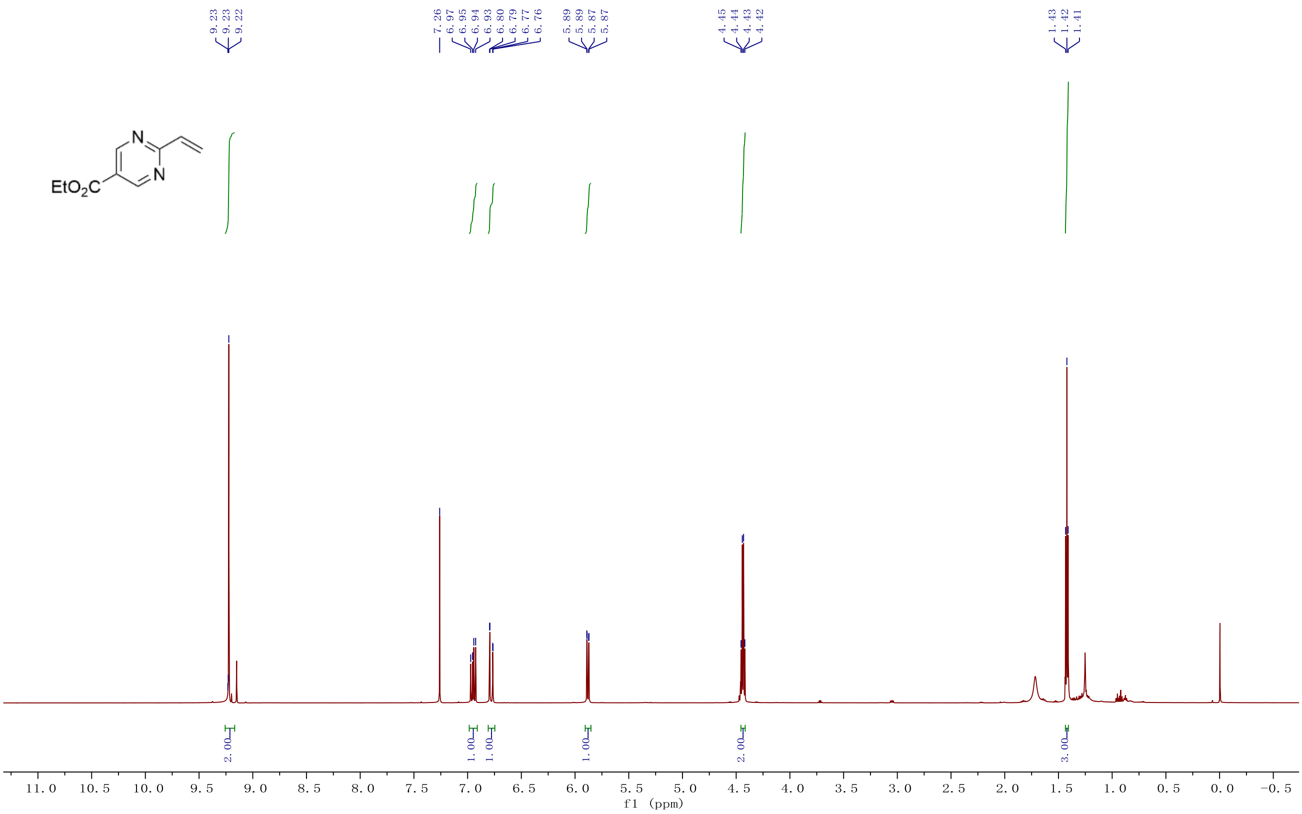

Supplementary Figure 267. ^{13}C NMR spectrum of compound **5k** (100 MHz, $CDCl_3$)

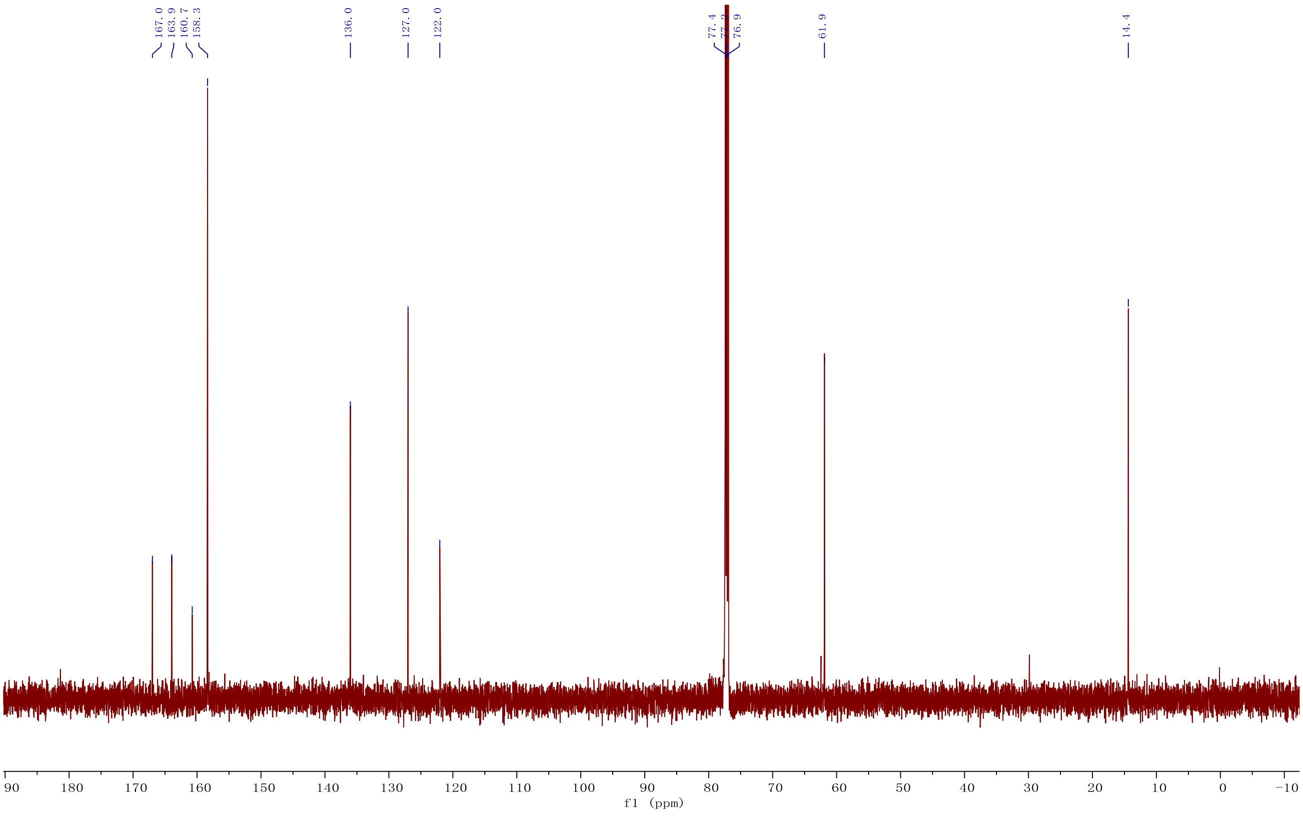

Supplementary Figure 268. ^{1}H NMR spectrum of compound **5n** (400 MHz, $CDCl_3$)

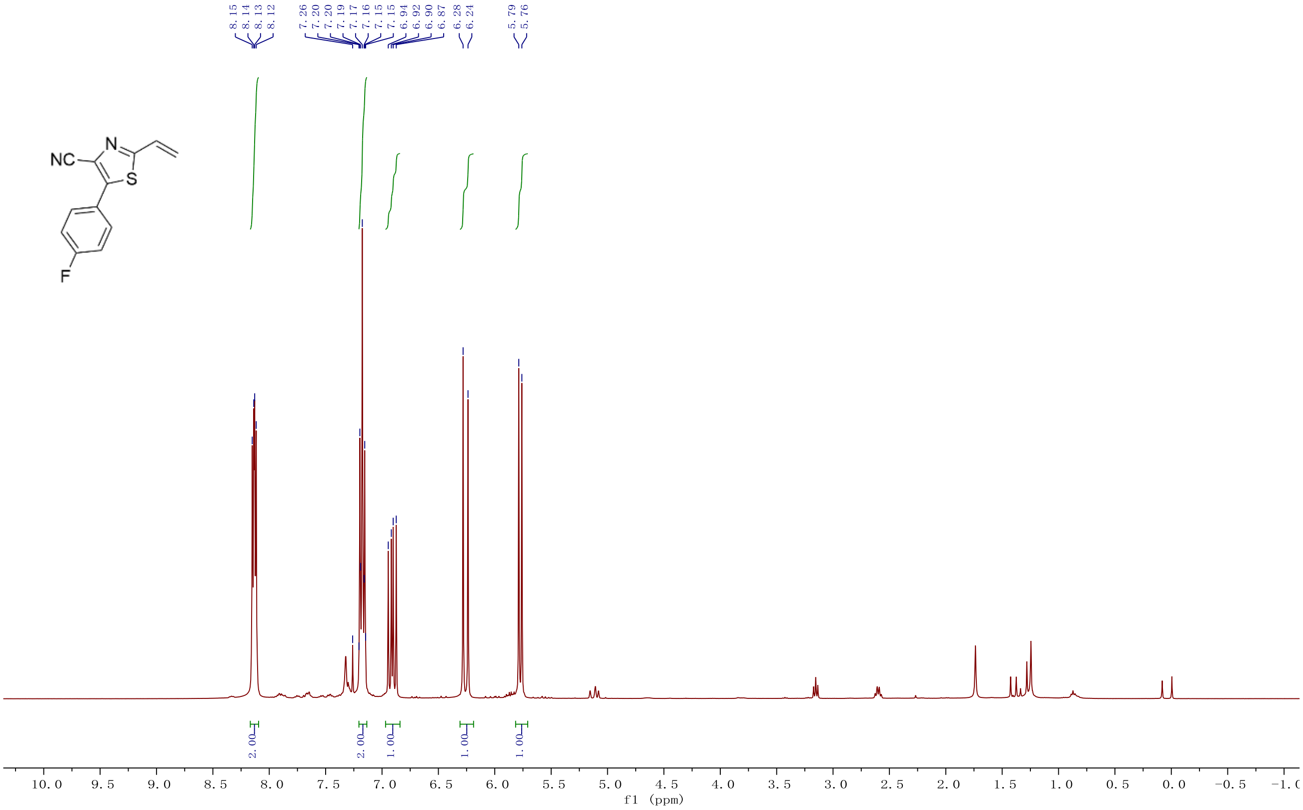

Supplementary Figure 269. ^{13}C NMR spectrum of compound **5n** (100 MHz, $CDCl_3$)

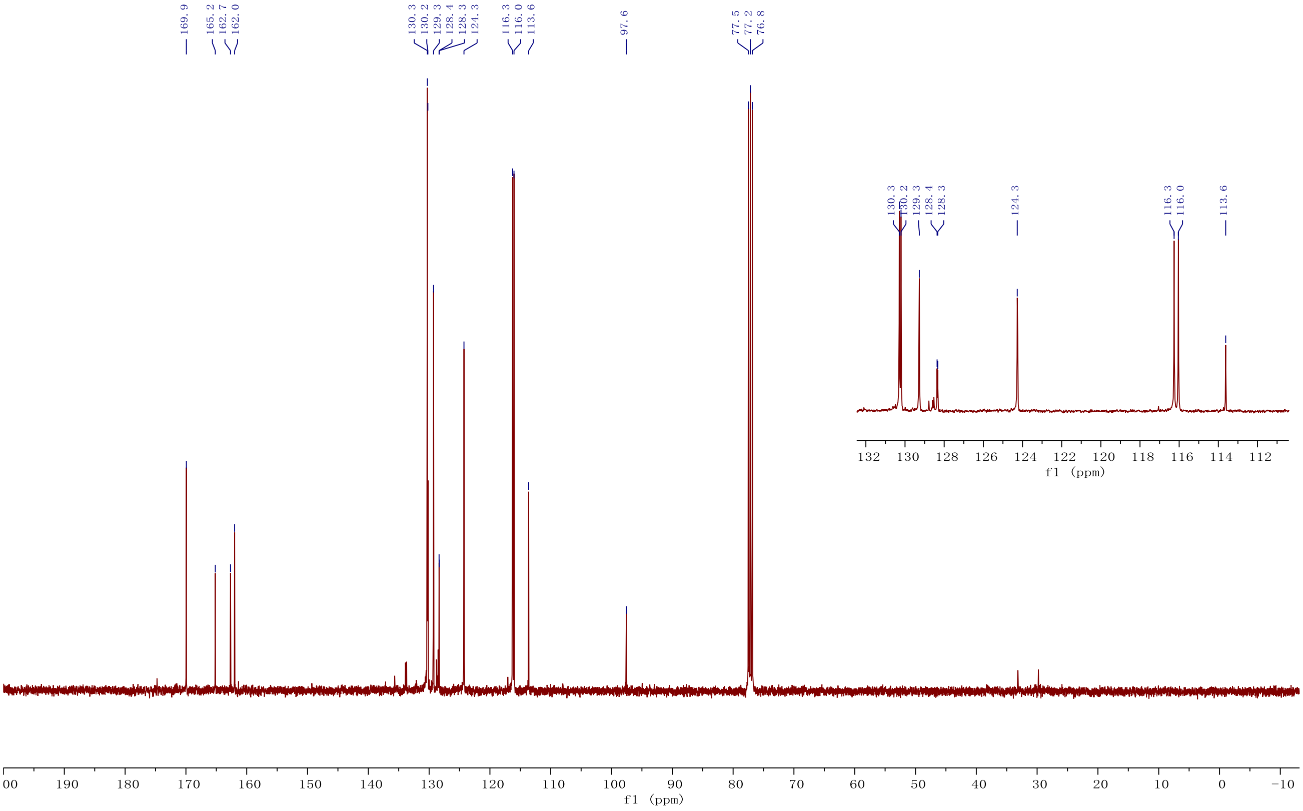

Supplementary Figure 270. ^{19}F NMR spectrum of compound **5n** (376 MHz, $CDCl_3$)

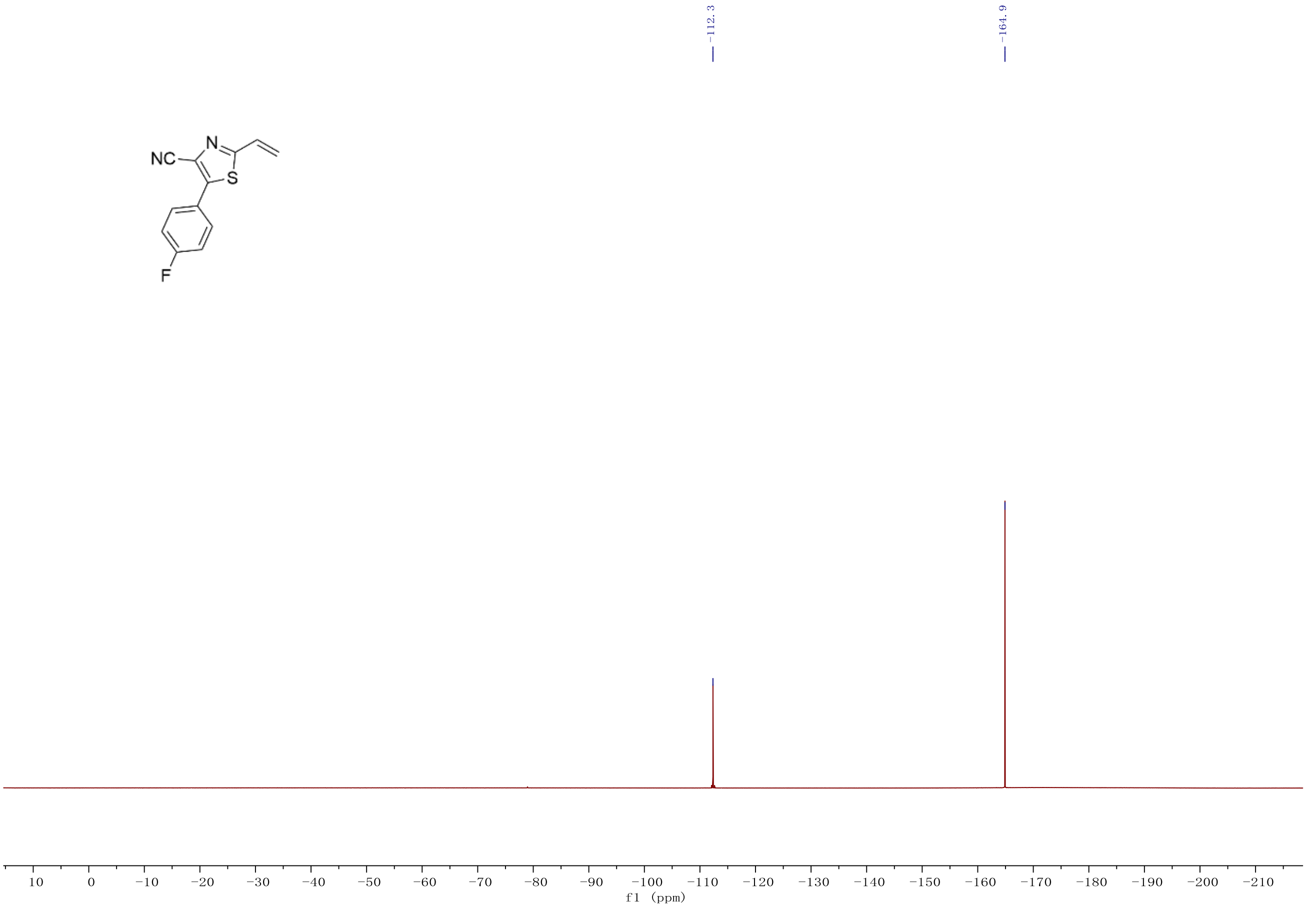

Supplementary Figure 271. ^{1}H NMR spectrum of compound **5p** (400 MHz, $CDCl_3$)

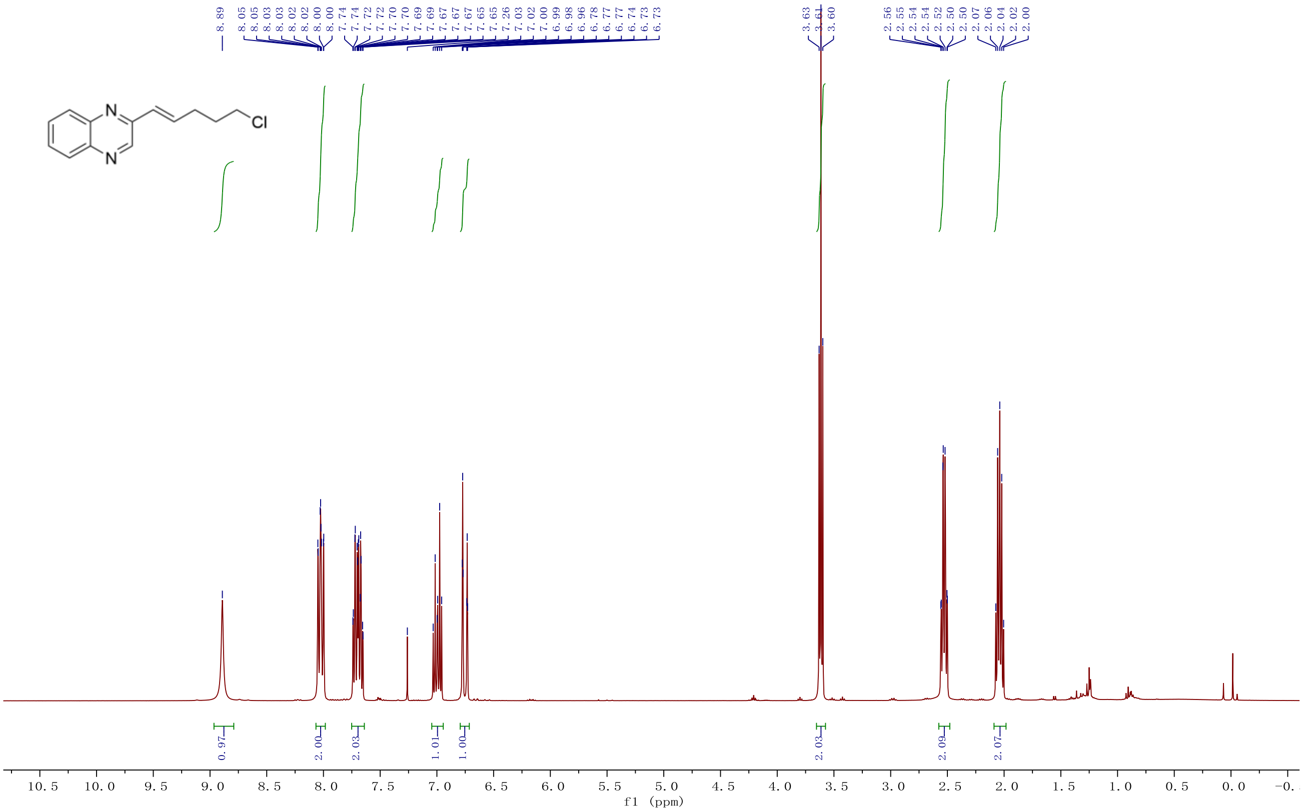

Supplementary Figure 272. ^{13}C NMR spectrum of compound **5p** (100 MHz, $CDCl_3$)

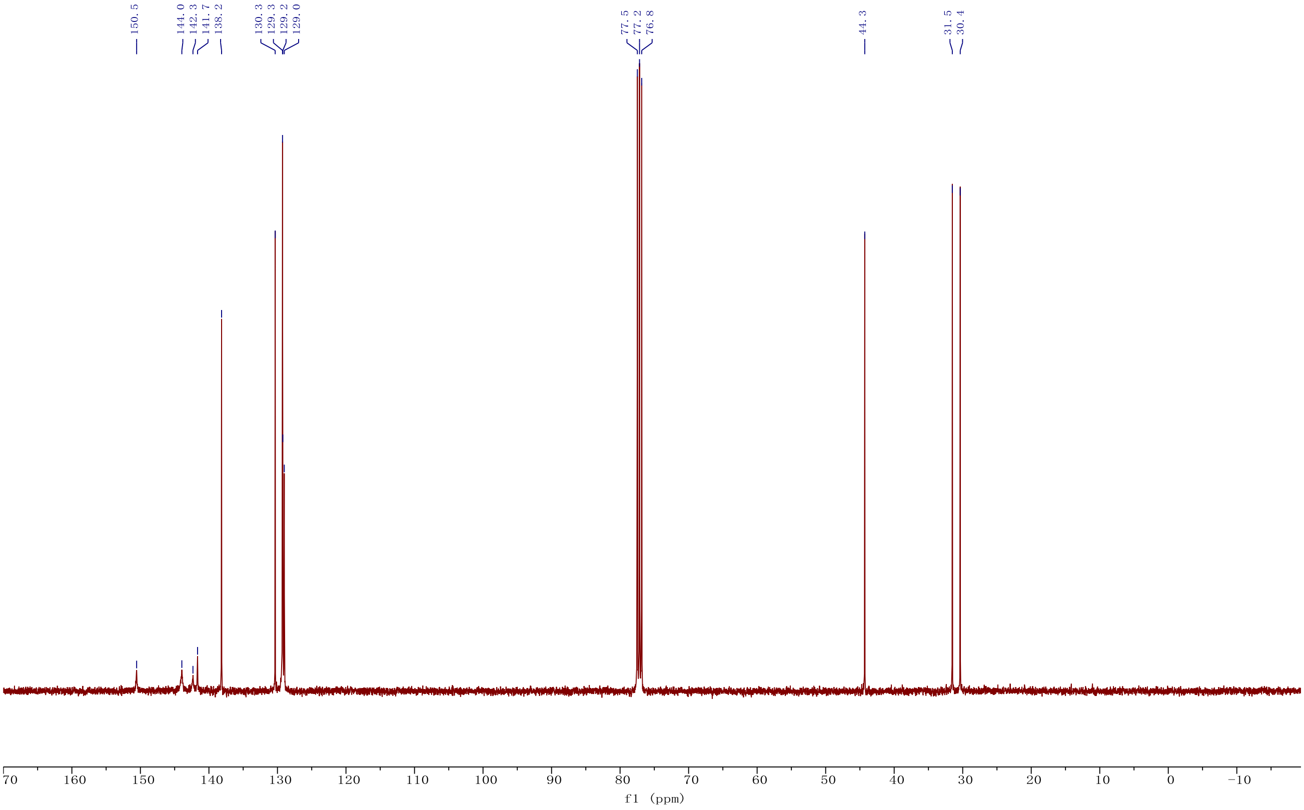

Supplementary Figure 273. ^{1}H NMR spectrum of compound **5u** (400 MHz, $CDCl_3$)

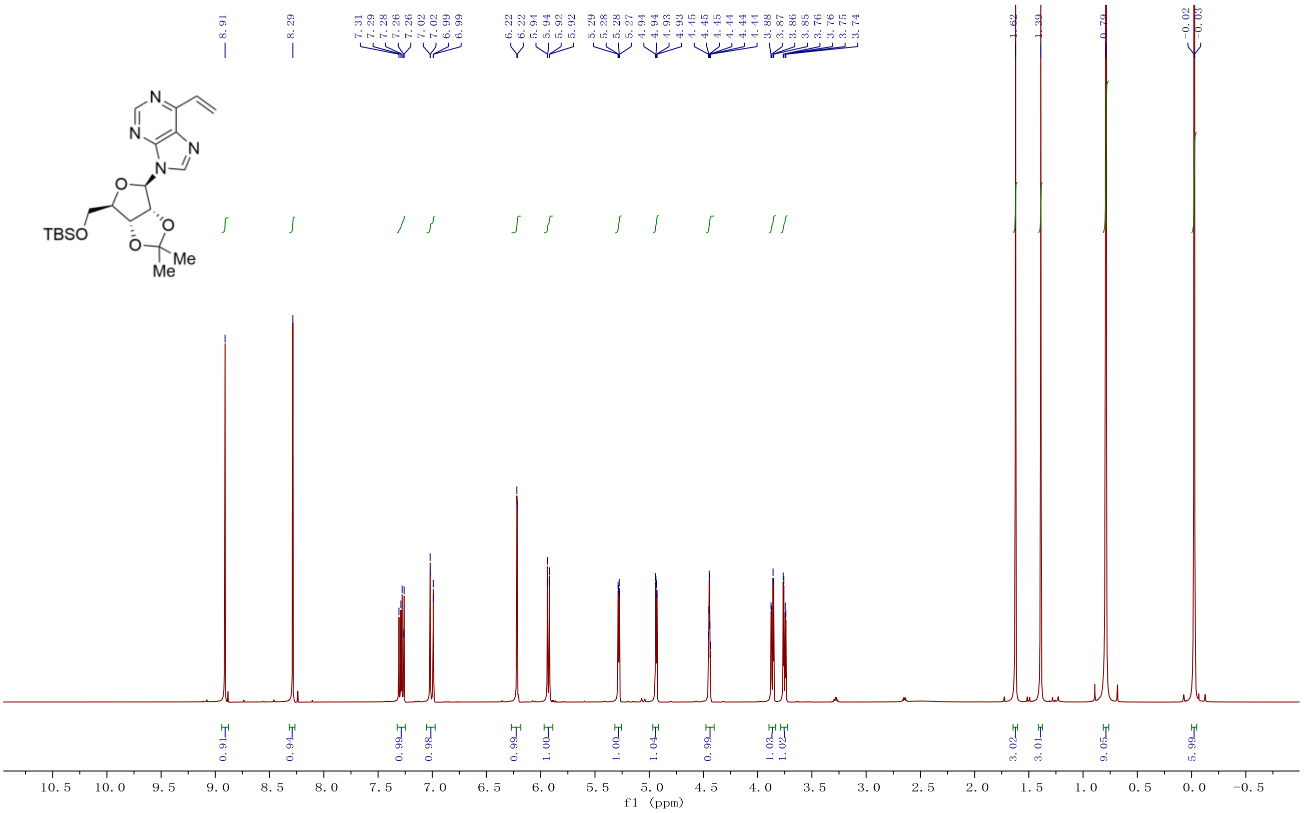

Supplementary Figure 274. ^{13}C NMR spectrum of compound **5u** (100 MHz, $CDCl_3$)

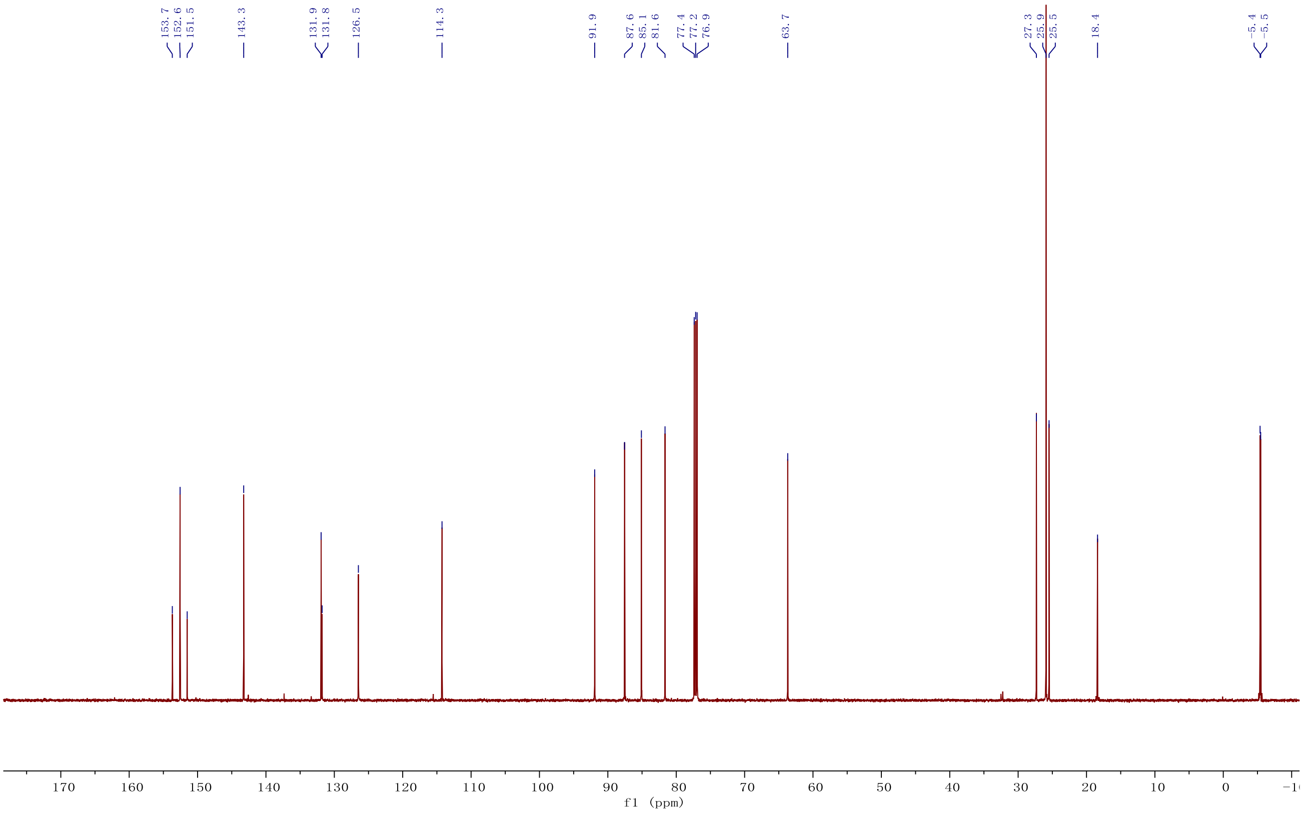

Supplementary Figure 275. ^{1}H NMR spectrum of compound **5v** (600 MHz, $CDCl_3$)

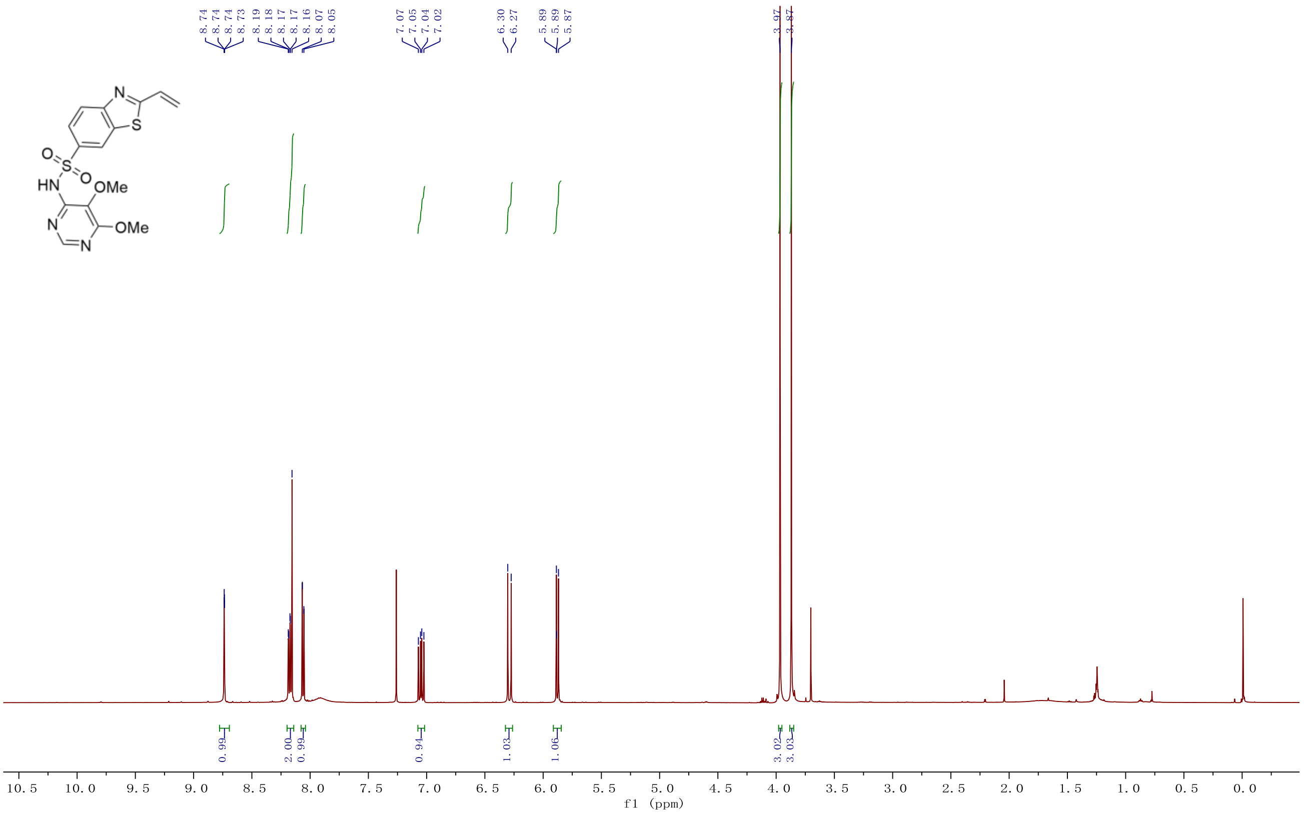

Supplementary Figure 276. ^{13}C NMR spectrum of compound **5v** (150 MHz, $CDCl_3$)

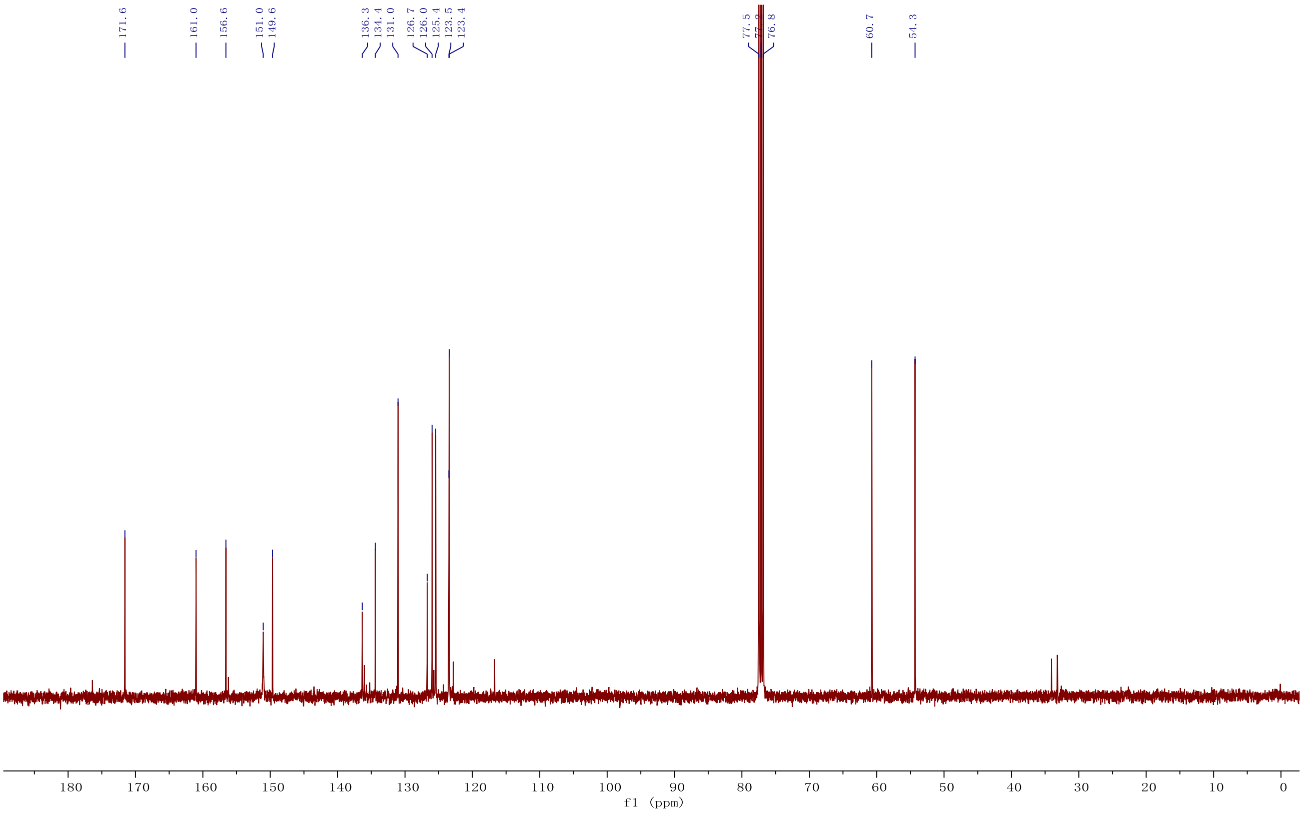

Supplementary Figure 277. ^{1}H NMR spectrum of compound **3aa** (400 MHz, $CDCl_3$)

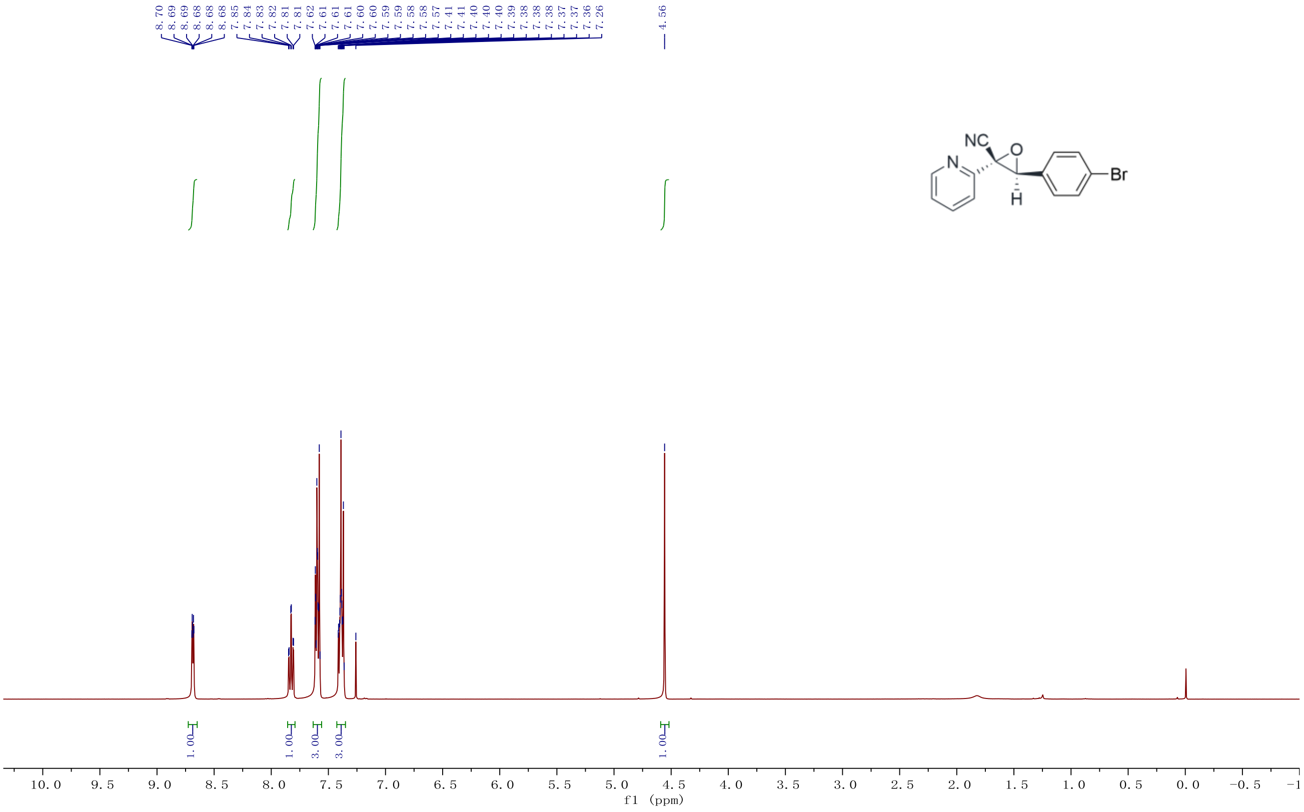

Supplementary Figure 278. ^{13}C NMR spectrum of compound **3aa** (100 MHz, $CDCl_3$)

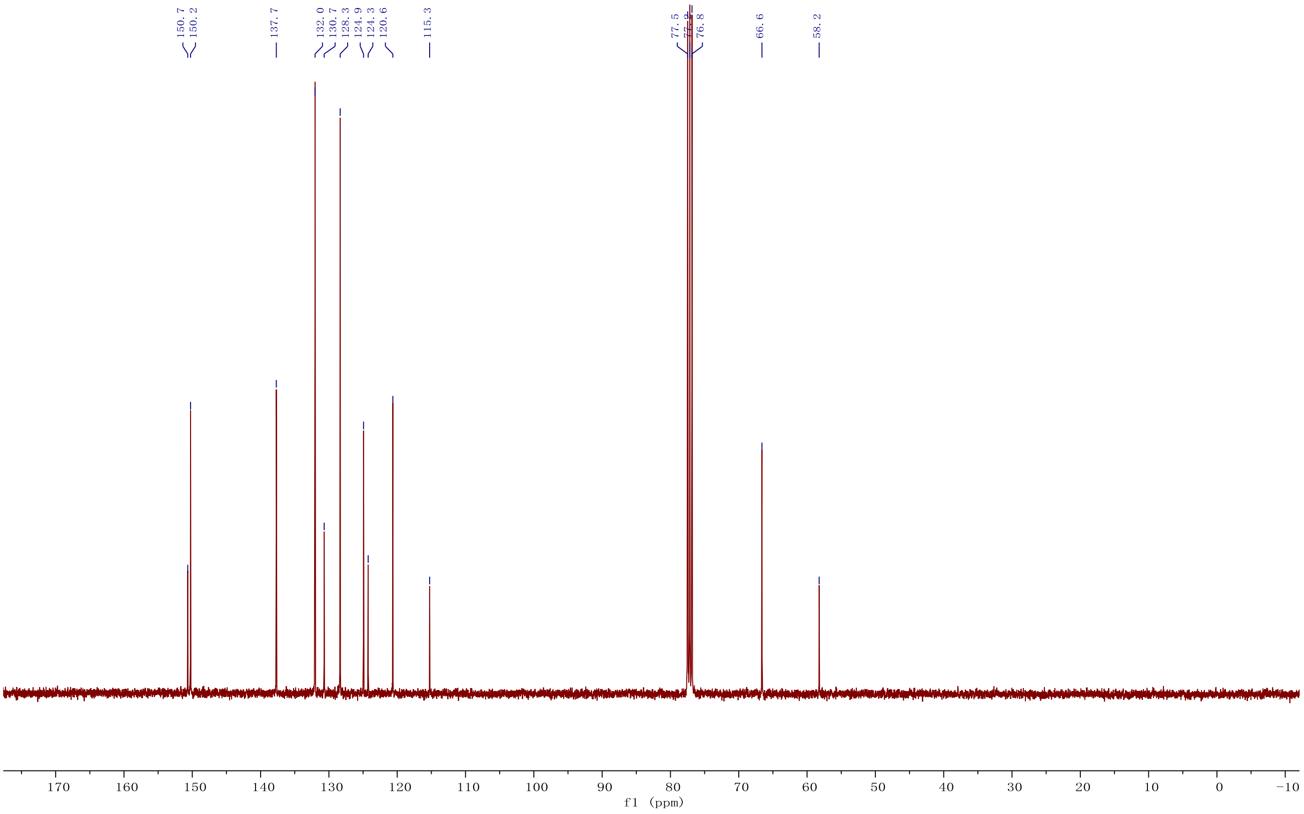

Supplementary Figure 279. ^{1}H NMR spectrum of compound **3ab** (600 MHz, $CDCl_3$)

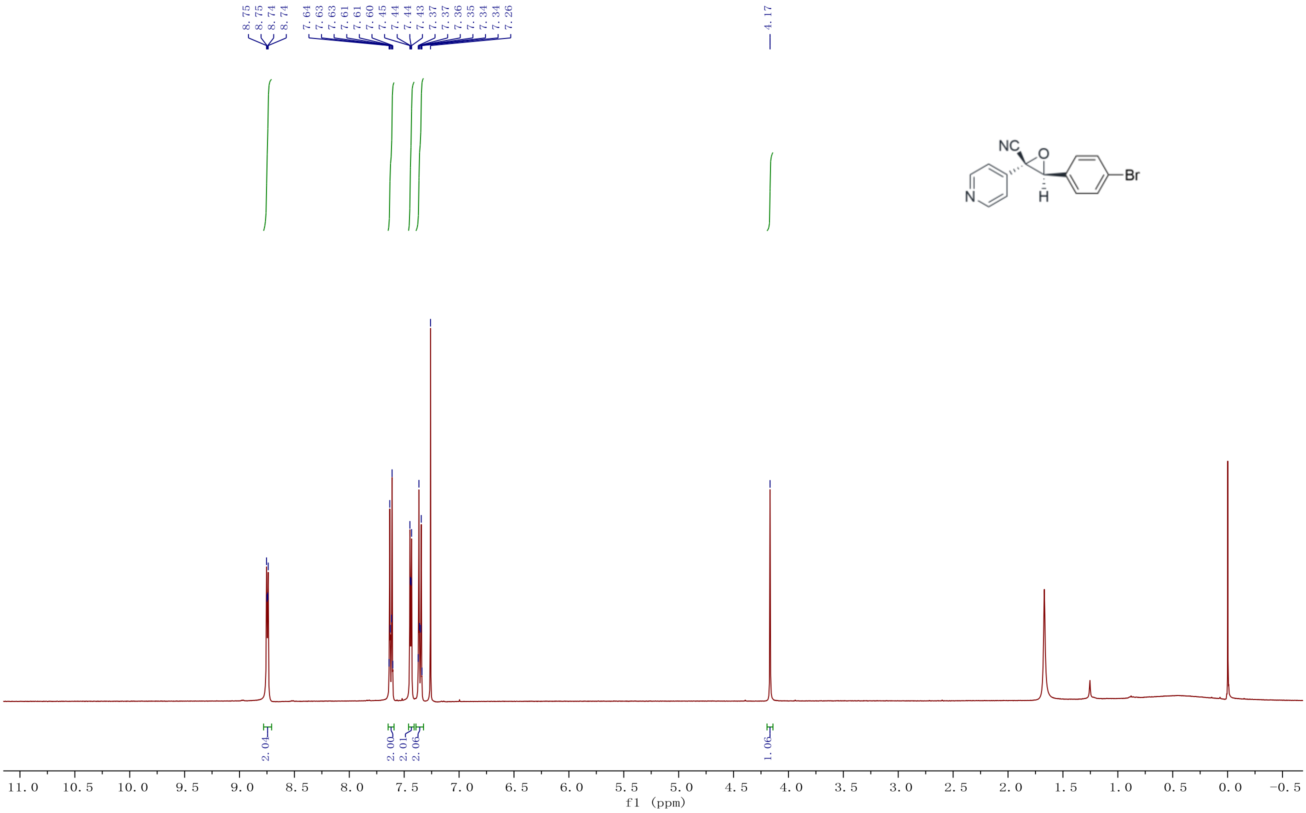

Supplementary Figure 280. ^{13}C NMR spectrum of compound **3ab** (150 MHz, $CDCl_3$)

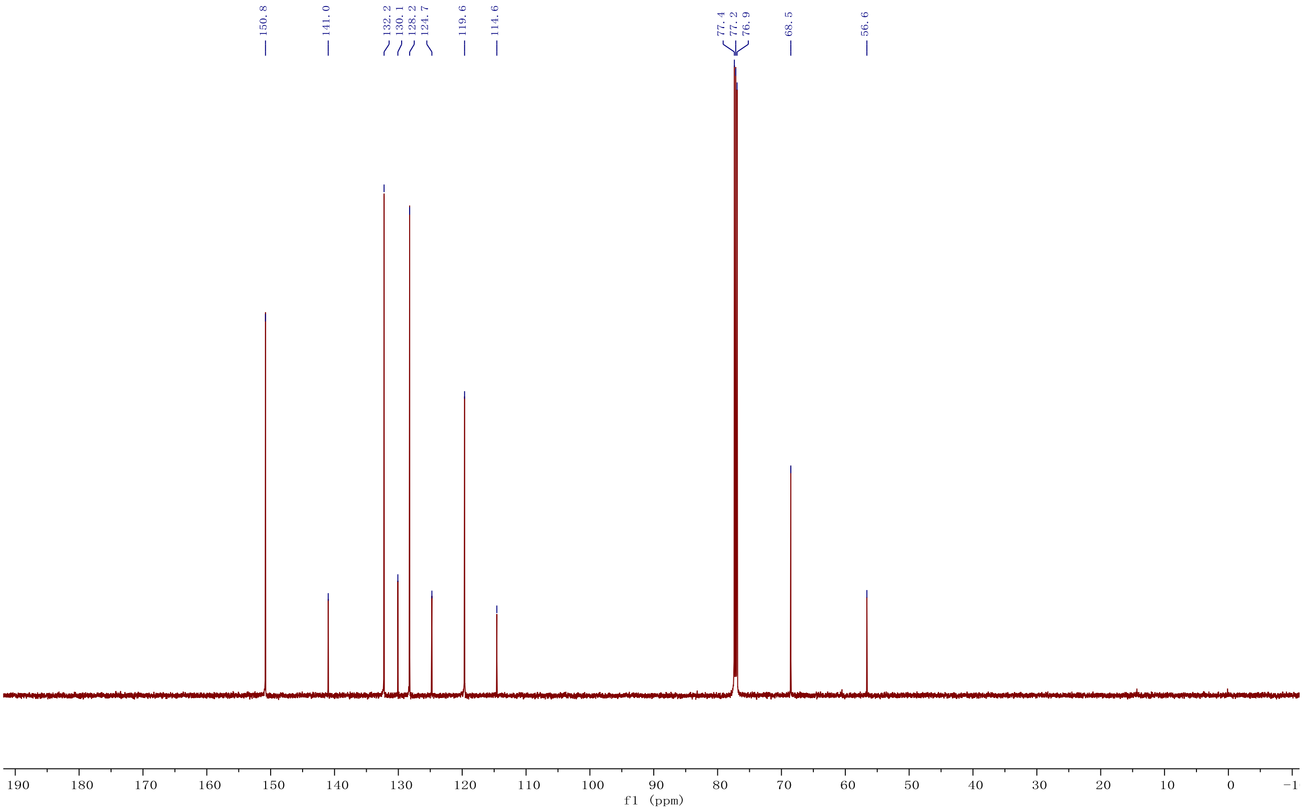

Supplementary Figure 281. ^{1}H NMR spectrum of compound **3ac** (600 MHz, $CDCl_3$)

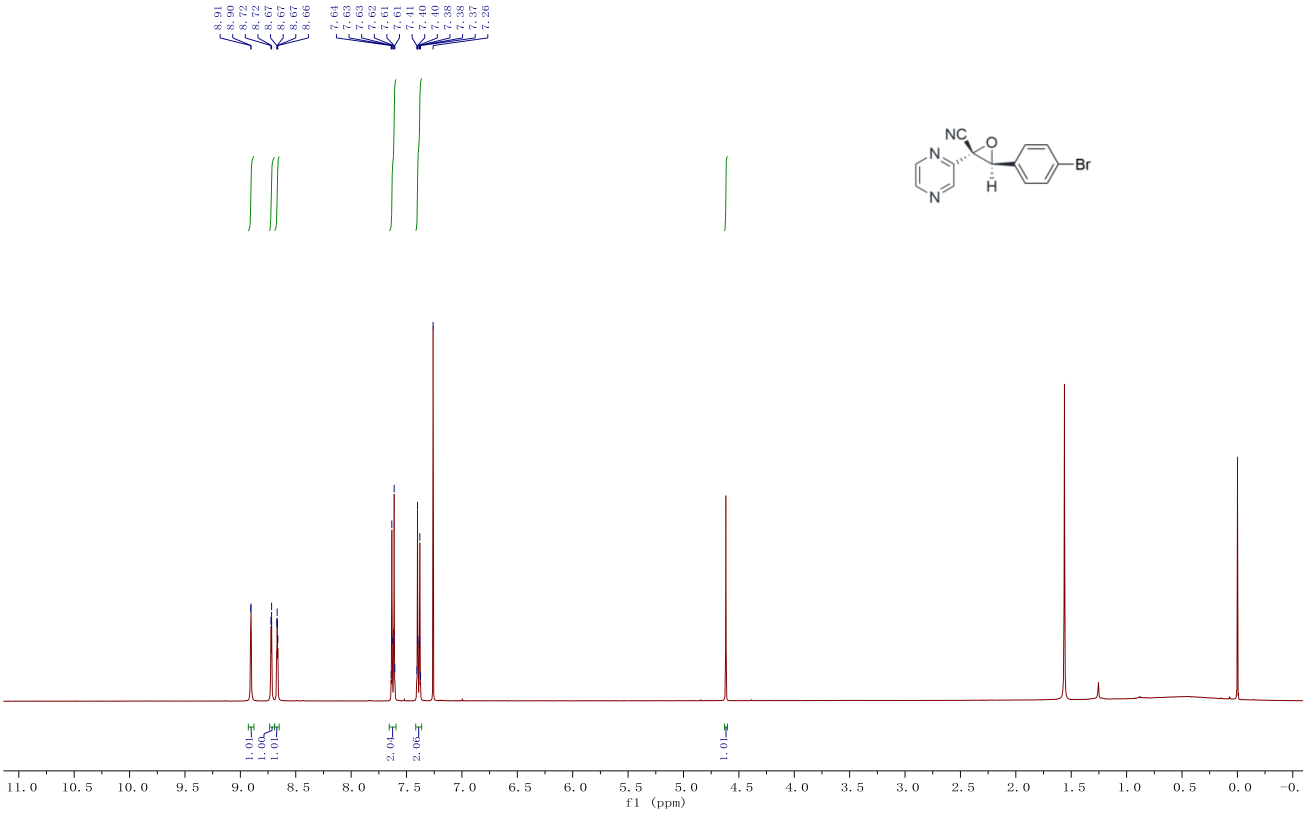

Supplementary Figure 282. ^{13}C NMR spectrum of compound **3ac** (150 MHz, $CDCl_3$)

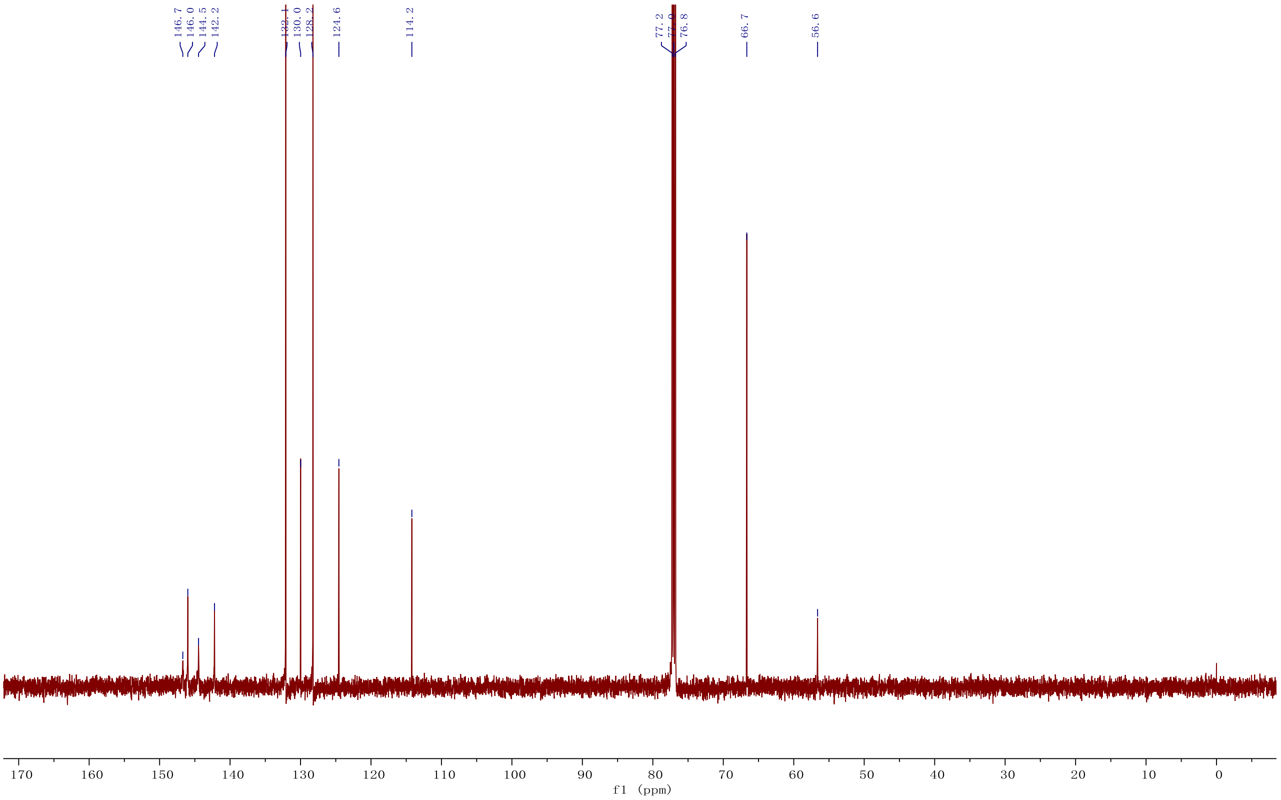

Supplementary Figure 283. ^{1}H NMR spectrum of compound **3ad** (600 MHz, $CDCl_3$)

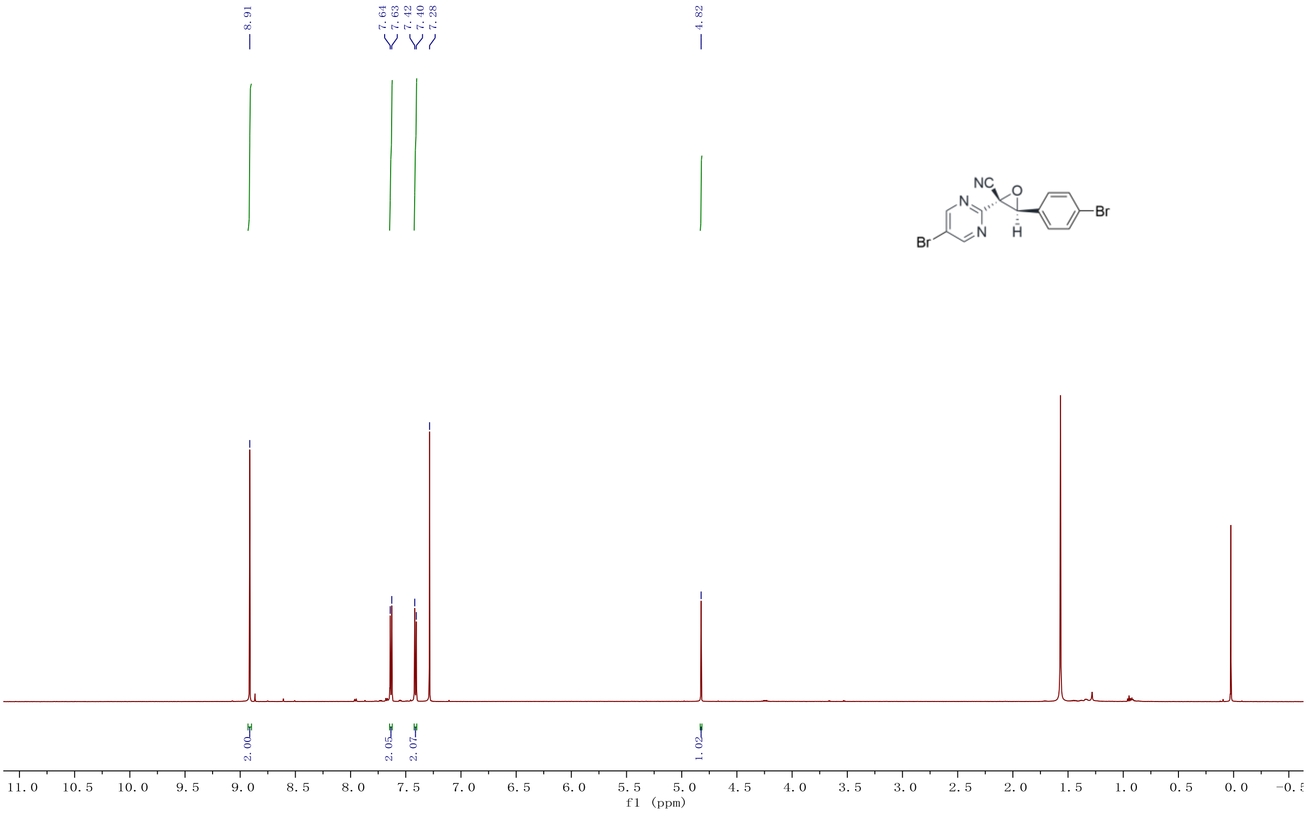

Supplementary Figure 284. ^{13}C NMR spectrum of compound **3ad** (150 MHz, $CDCl_3$)

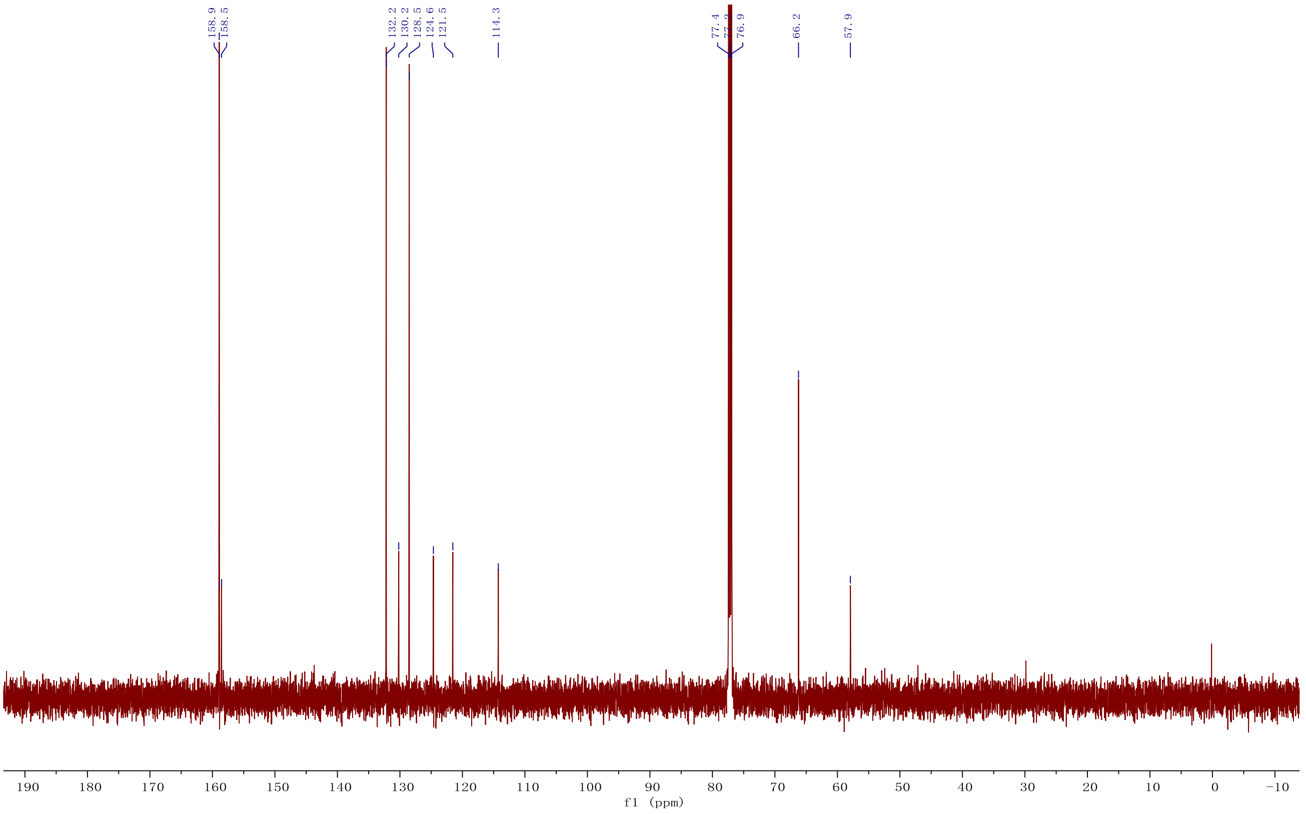

Supplementary Figure 285. ^{1}H NMR spectrum of compound **3ae** (400 MHz, $CDCl_3$)

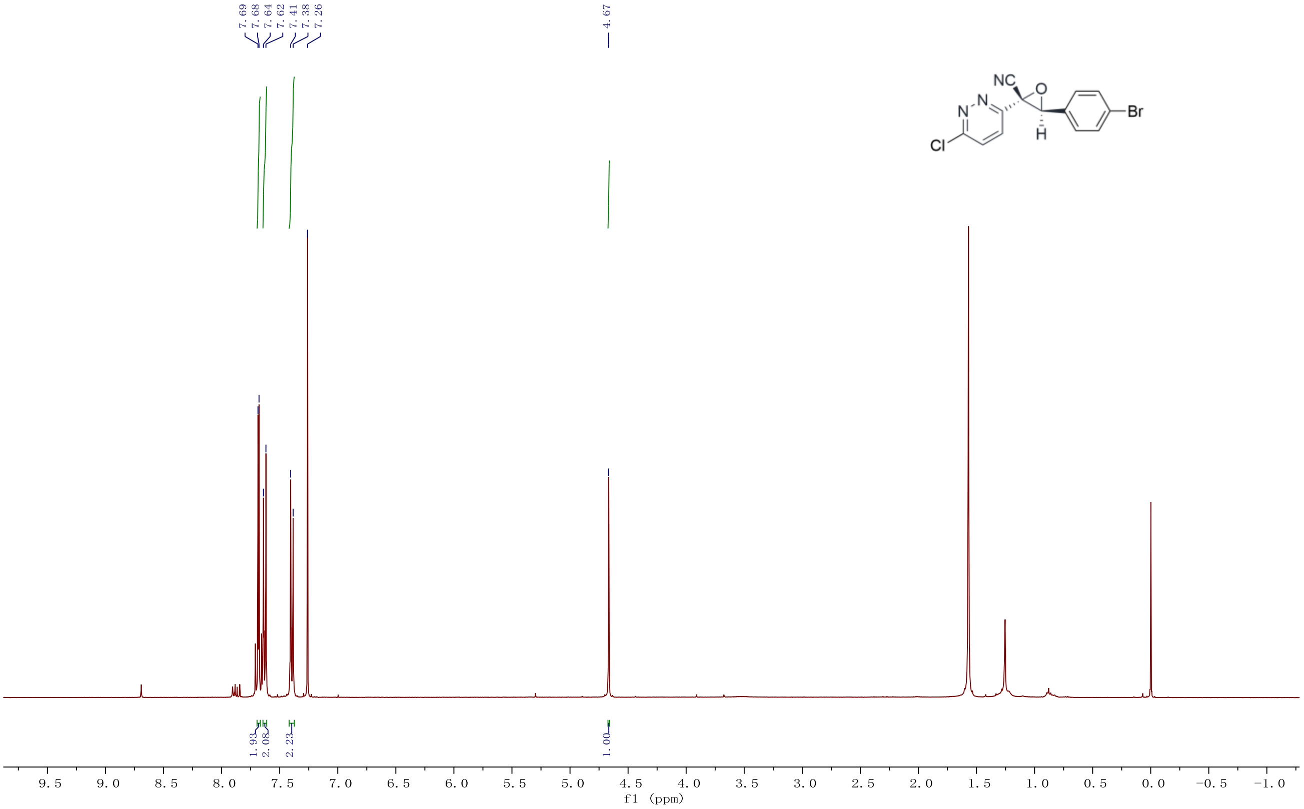

Supplementary Figure 286. ^{13}C NMR spectrum of compound **3ae** (100 MHz, $CDCl_3$)

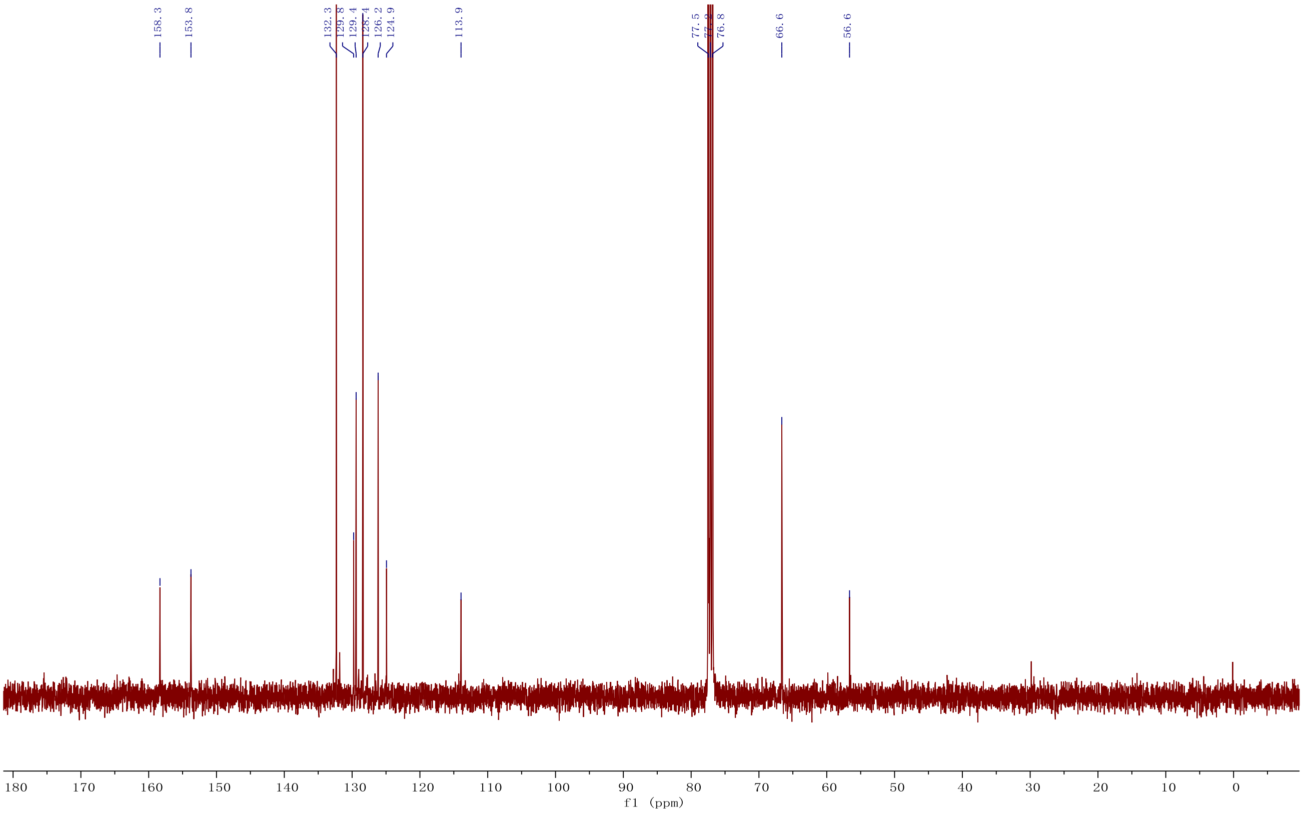

Supplementary Figure 287. ¹H NMR spectrum of compound **3af** (400 MHz, $CDCl_3$)

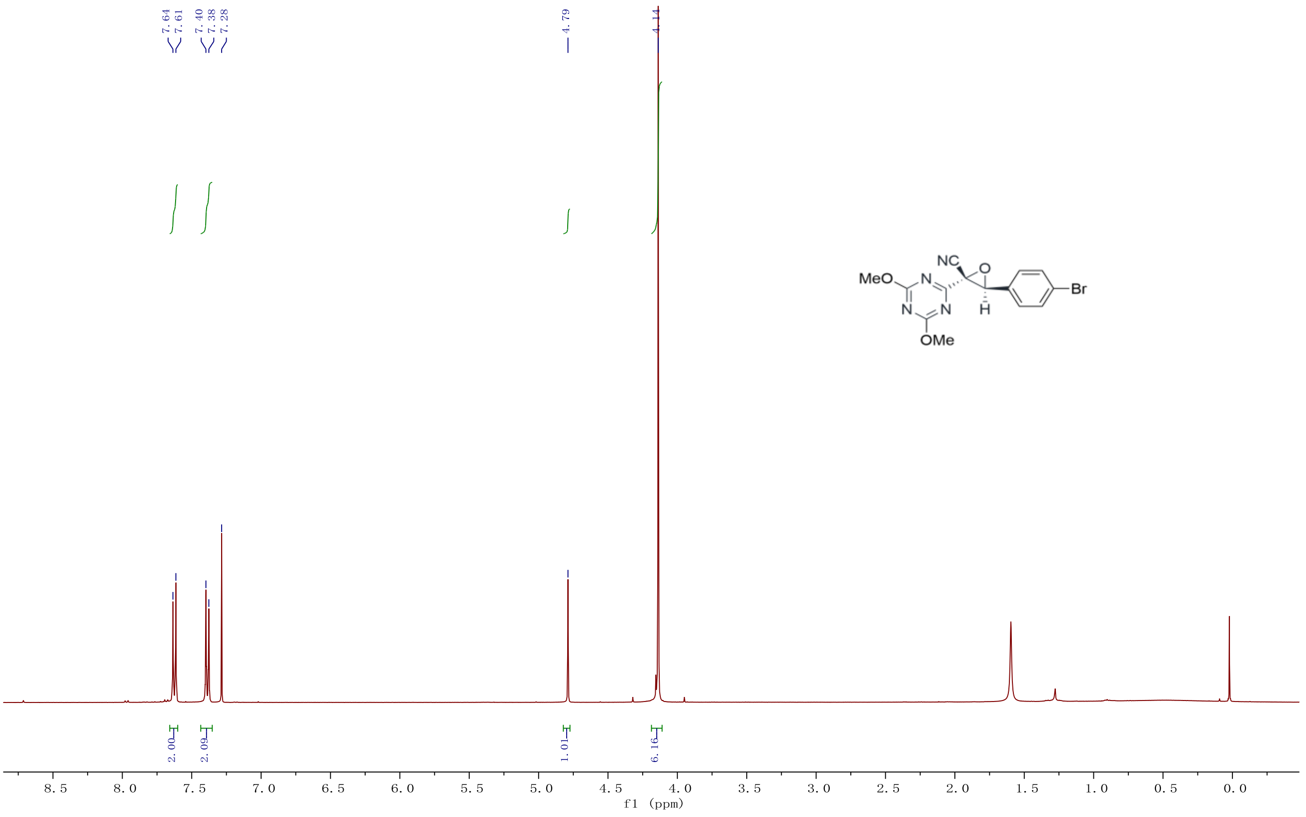

Supplementary Figure 288. ¹³C NMR spectrum of compound **3af** (100 MHz, $CDCl_3$)

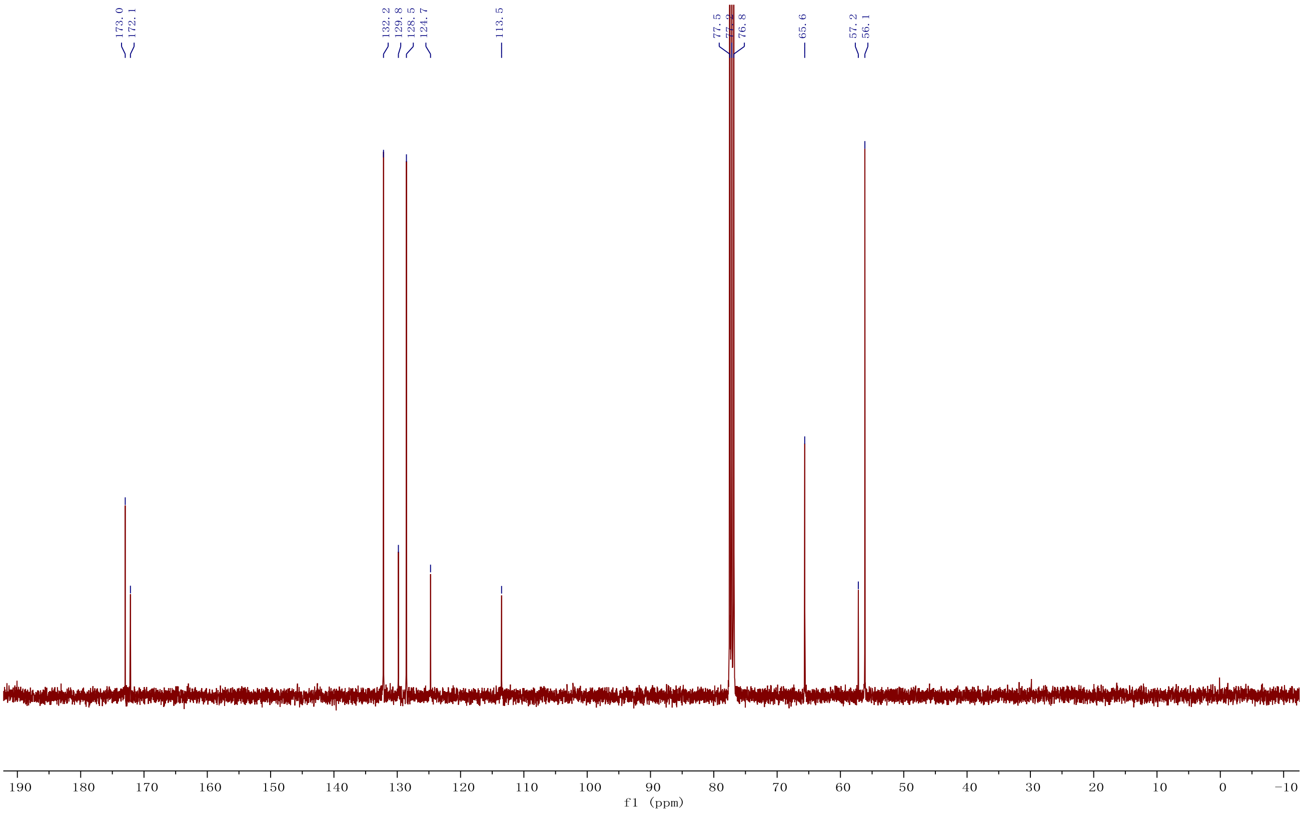

Supplementary Figure 289. ^{1}H NMR spectrum of compound **3ag** (600 MHz, $CDCl_3$)

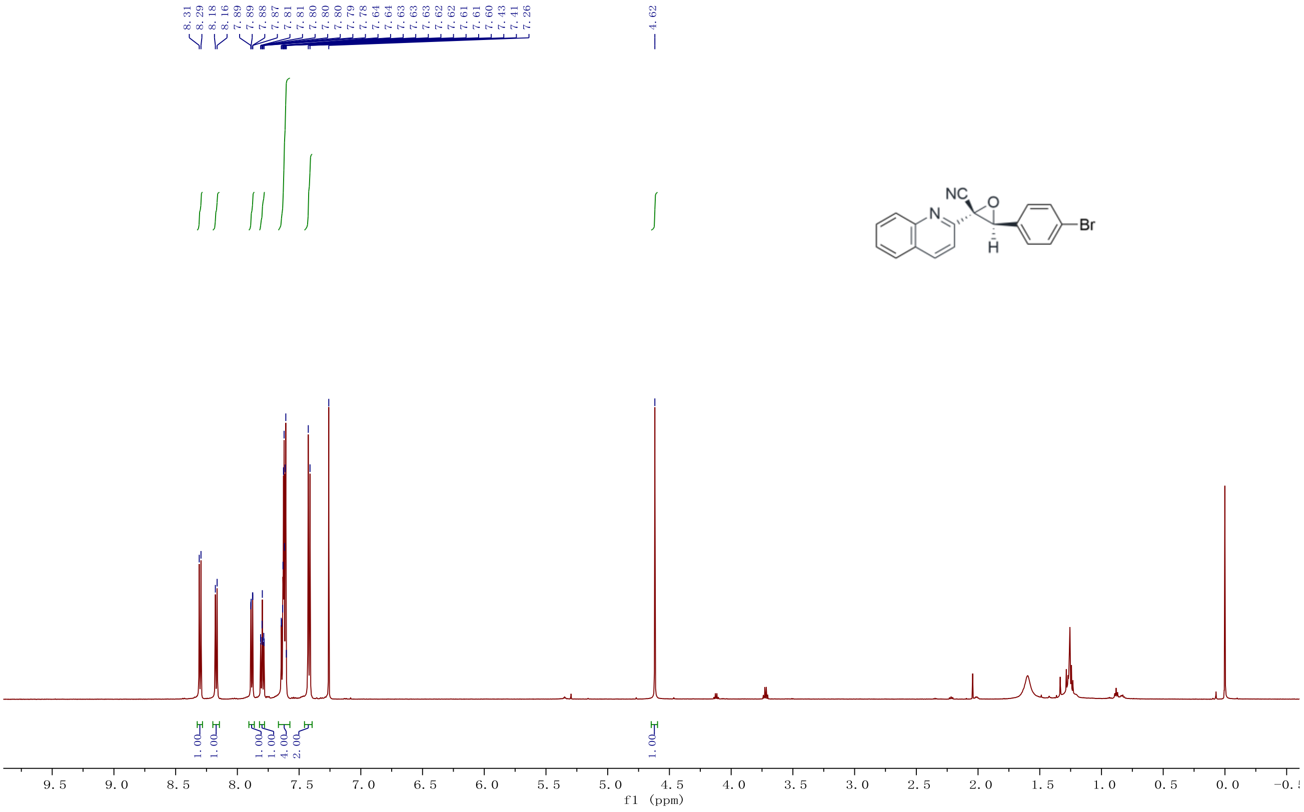

Supplementary Figure 290. ^{13}C NMR spectrum of compound **3ag** (150 MHz, $CDCl_3$)

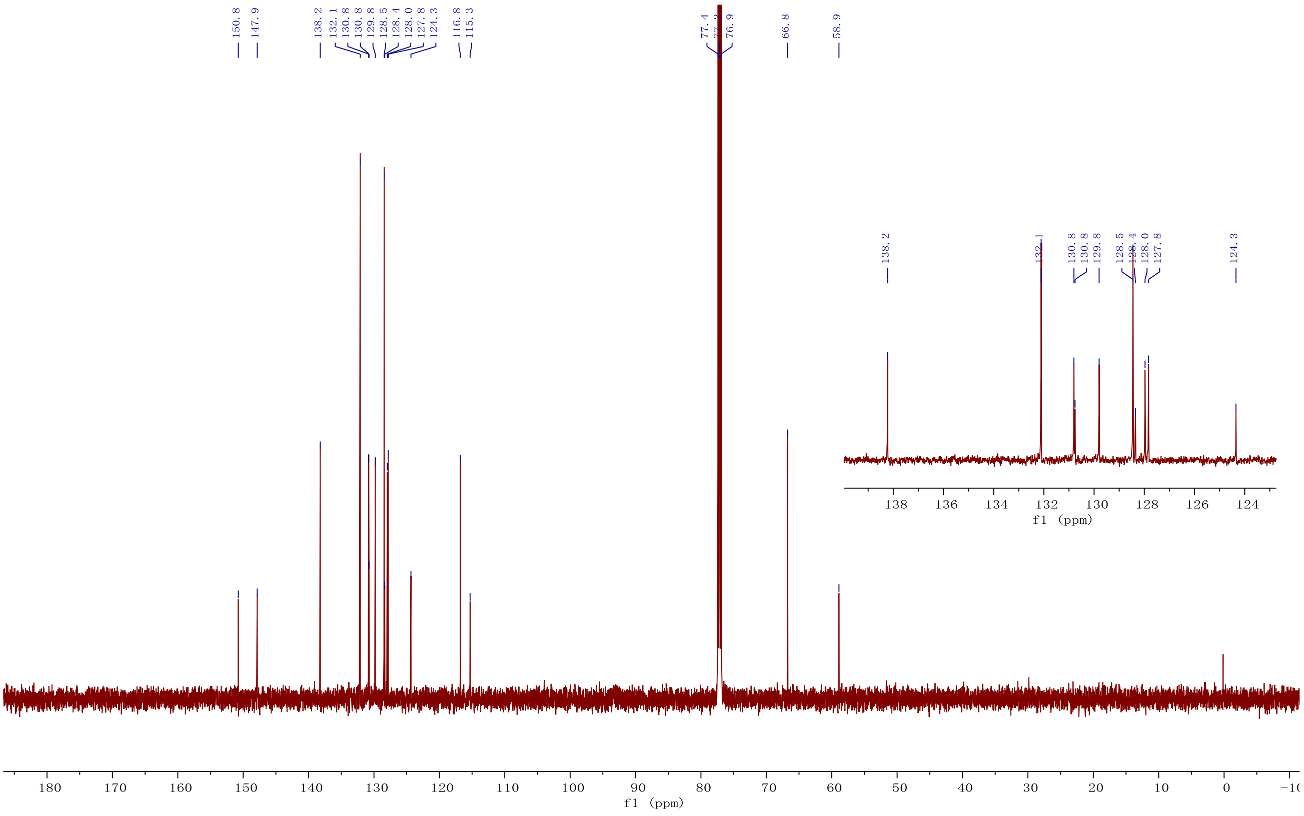

Supplementary Figure 291. ^{1}H NMR spectrum of compound **3ah** (400 MHz, $CDCl_3$)

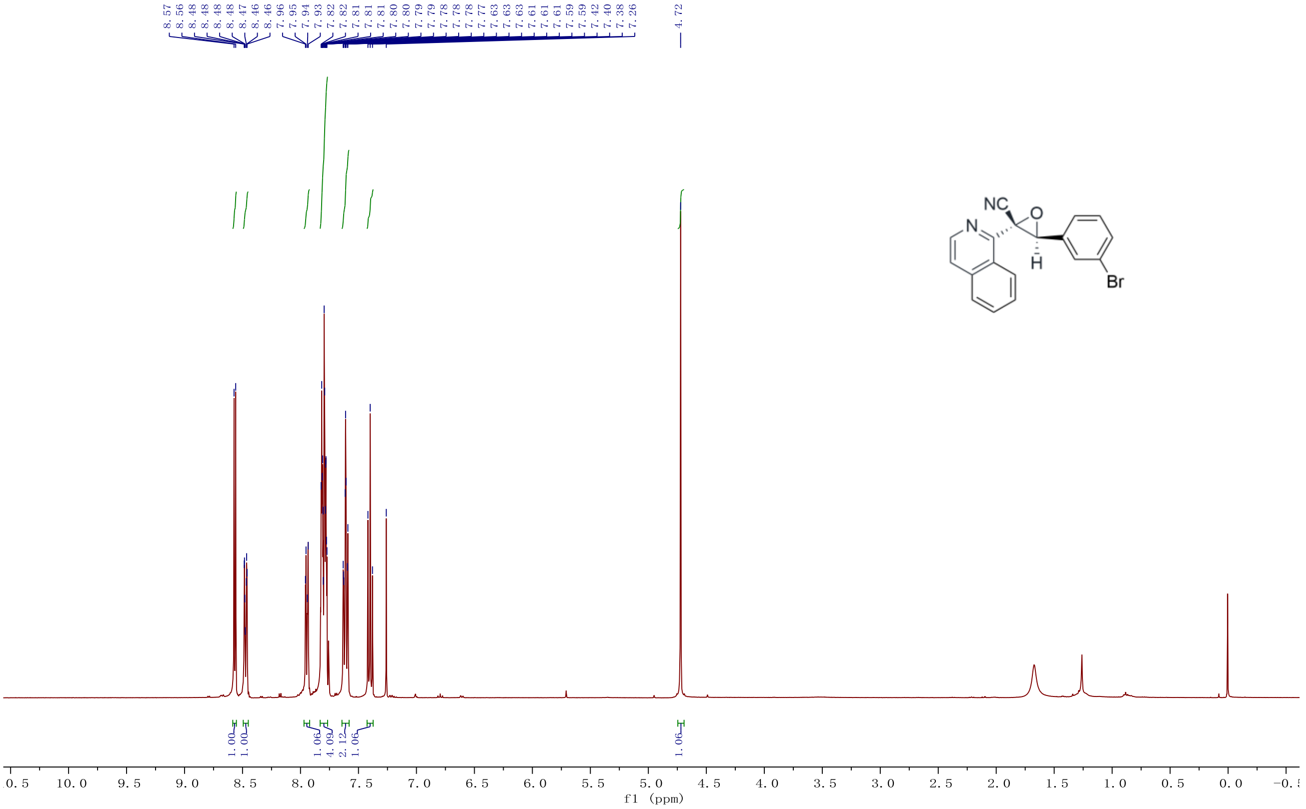

Supplementary Figure 292. ^{13}C NMR spectrum of compound **3ah** (100 MHz, $CDCl_3$)

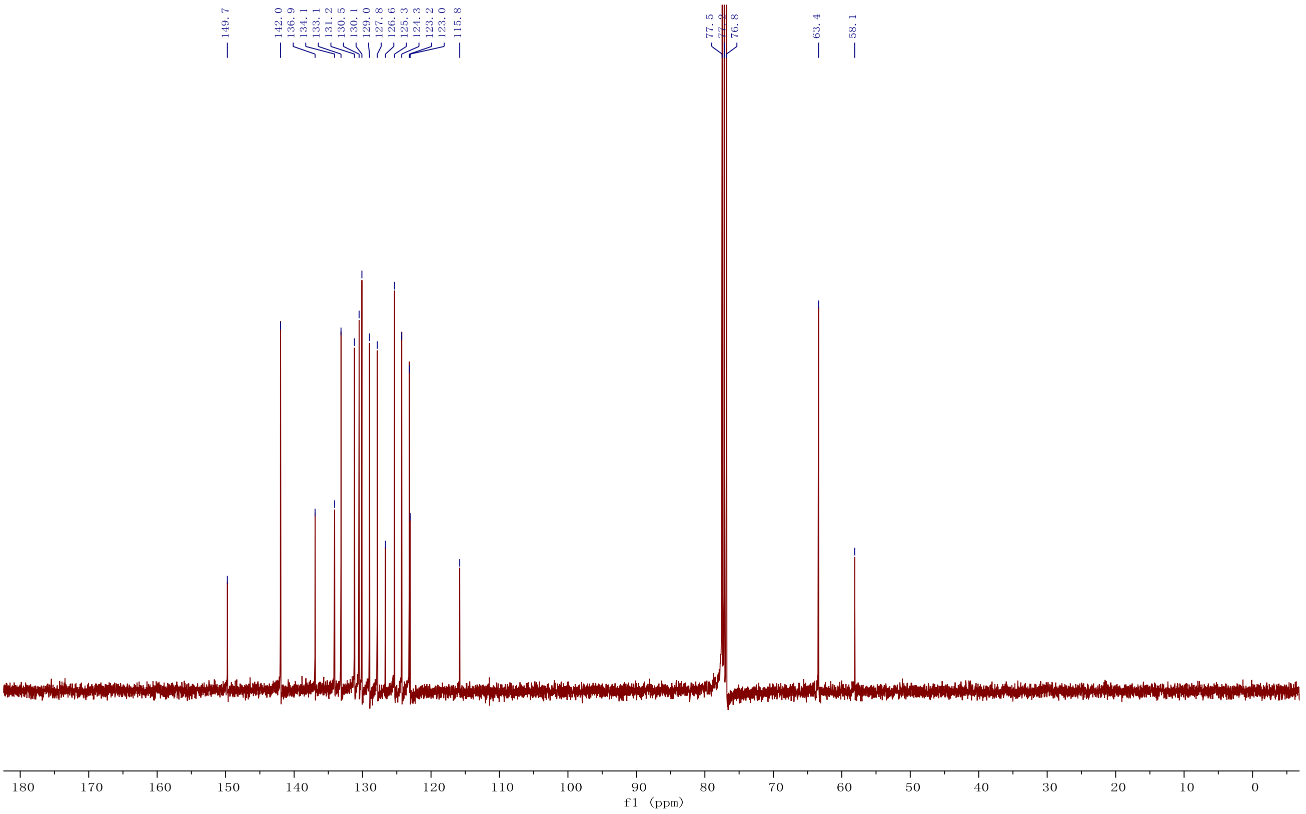

Supplementary Figure 293. ^{1}H NMR spectrum of compound **3ai** (600 MHz, $CDCl_3$)

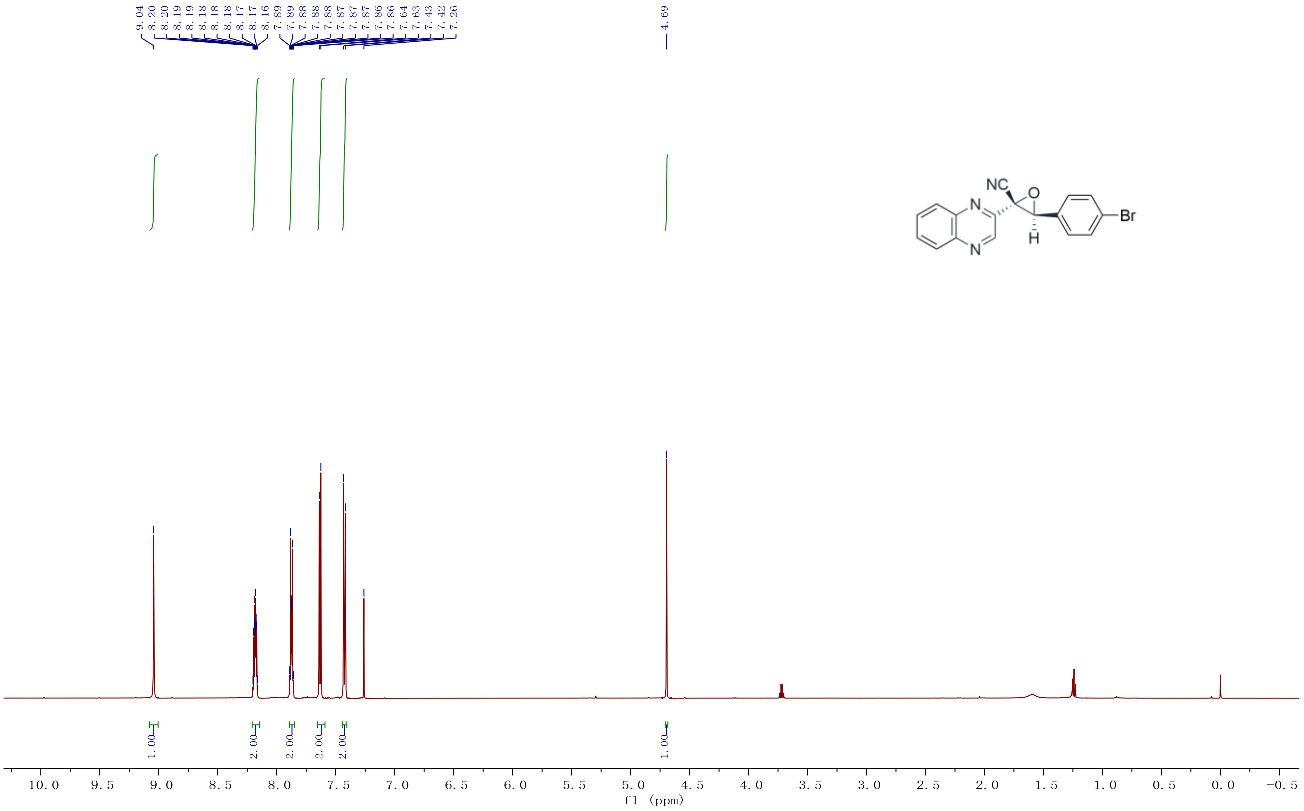

Supplementary Figure 294. ^{13}C NMR spectrum of compound **3ai** (150 MHz, $CDCl_3$)

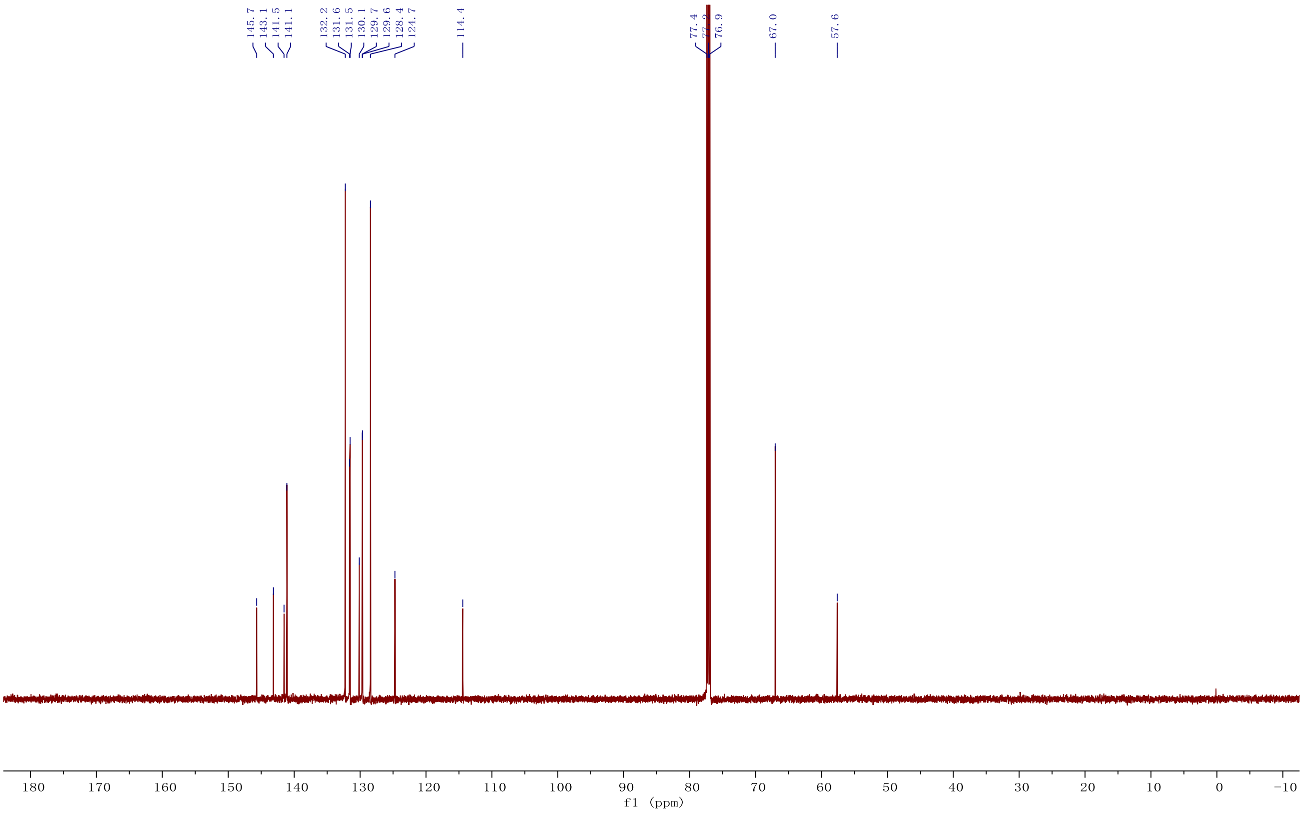

Supplementary Figure 295. ^{1}H NMR spectrum of compound **3aj** (400 MHz, $CDCl_3$)

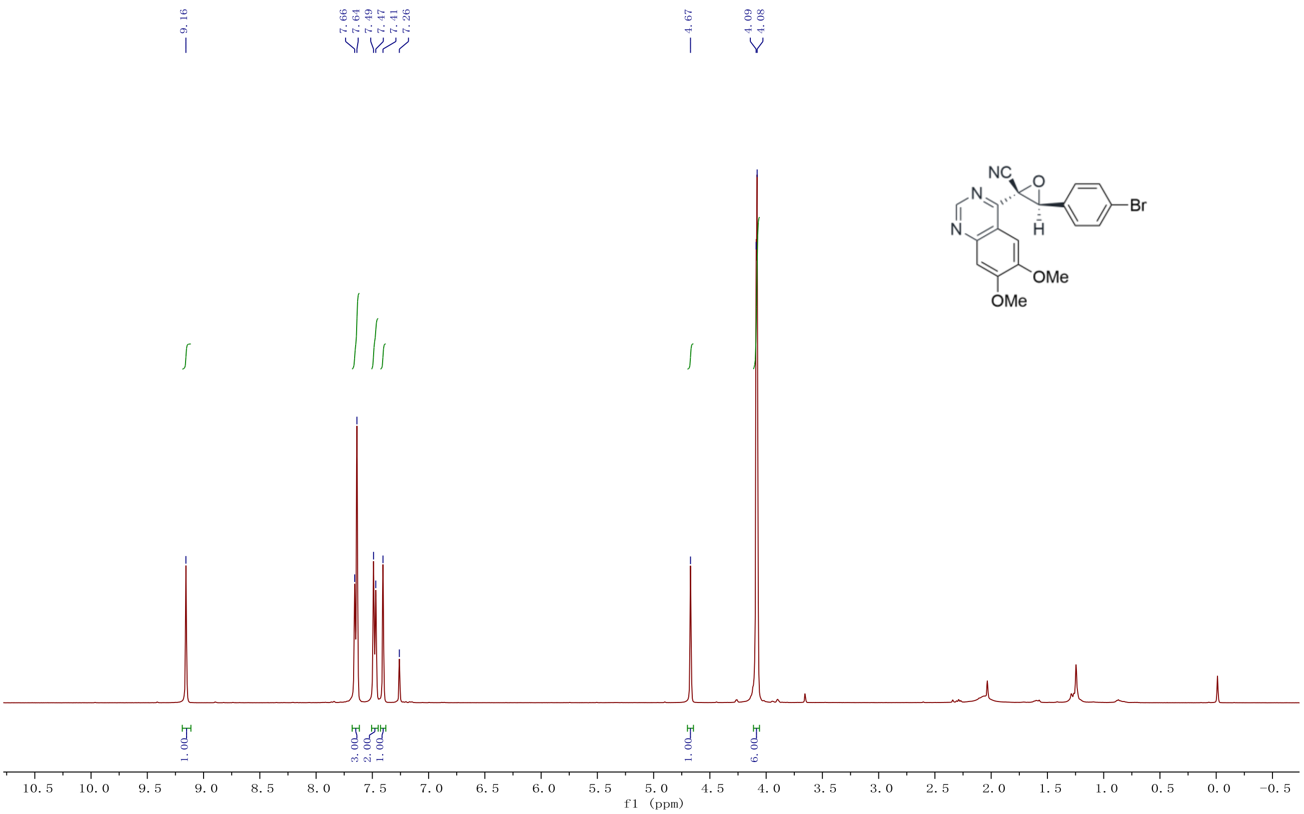

Supplementary Figure 296. ^{13}C NMR spectrum of compound **3aj** (100 MHz, $CDCl_3$)

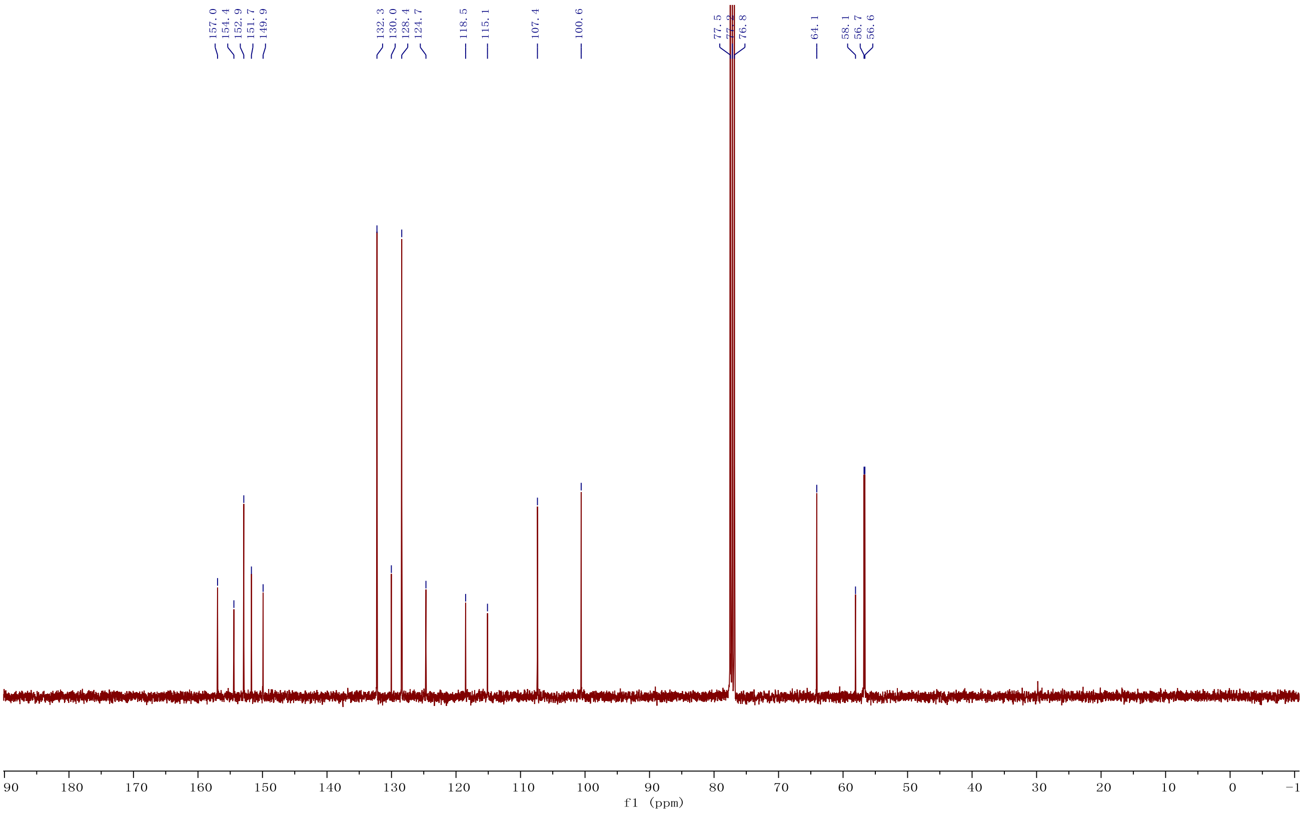

Supplementary Figure 297. ^{1}H NMR spectrum of compound **3ak** (400 MHz, $CDCl_3$)

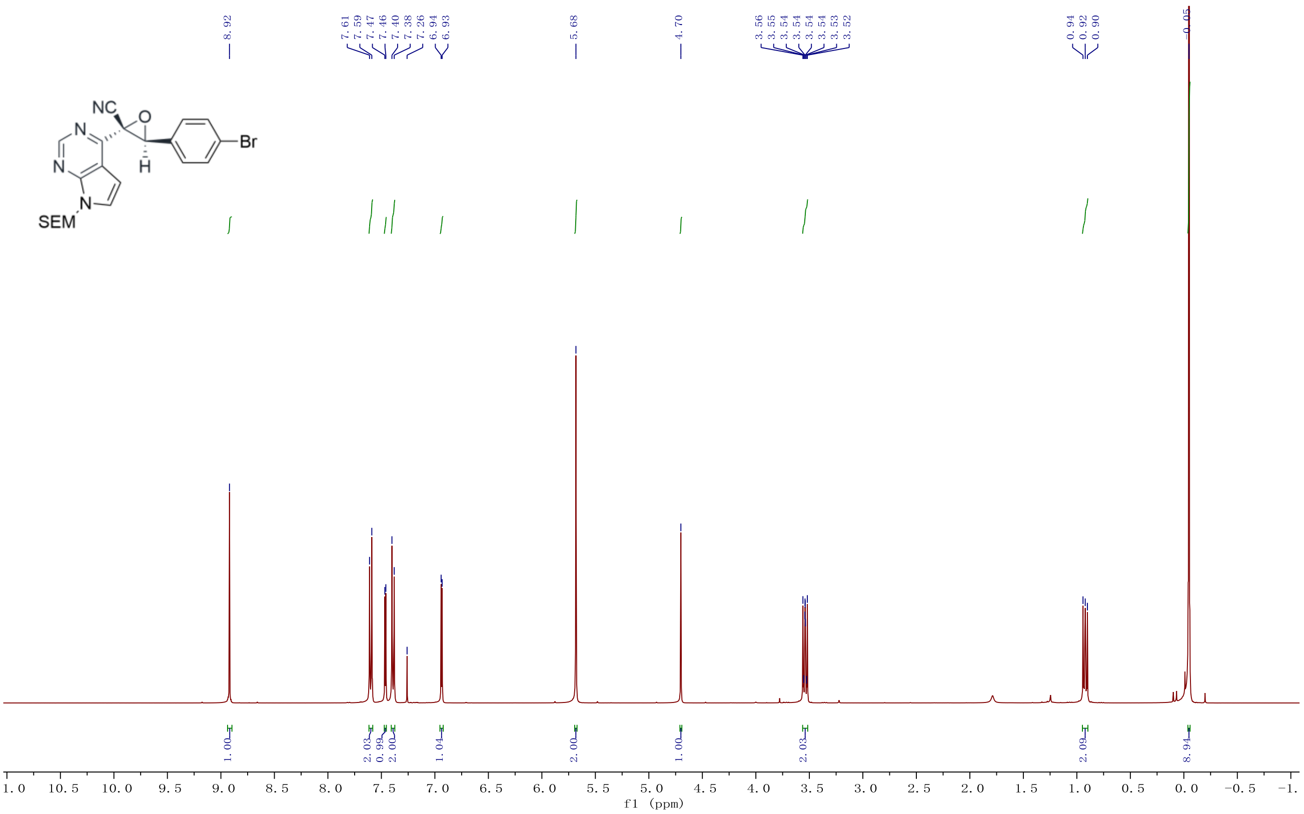

Supplementary Figure 298. ^{13}C NMR spectrum of compound **3ak** (100 MHz, $CDCl_3$)

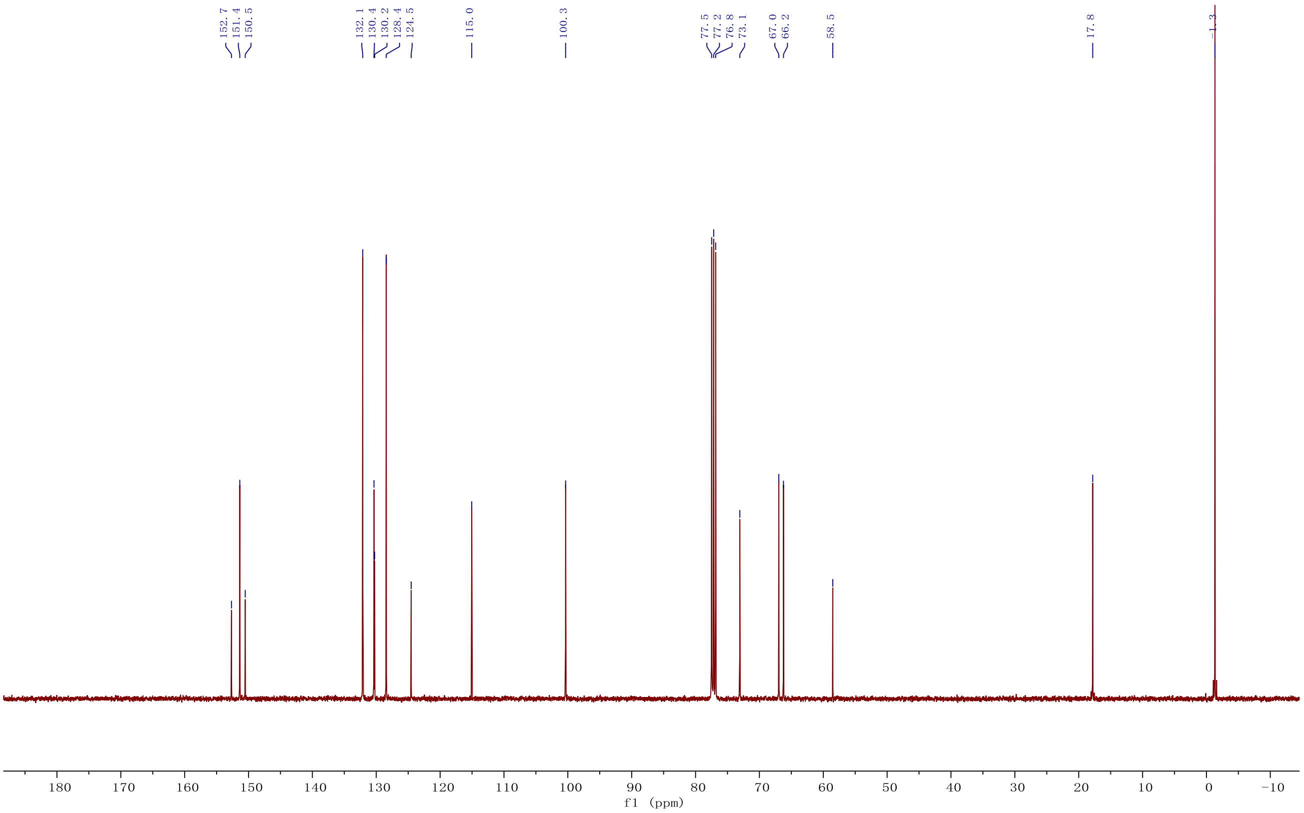

Supplementary Figure 299. ^{1}H NMR spectrum of compound **3al** (600 MHz, $CDCl_3$)

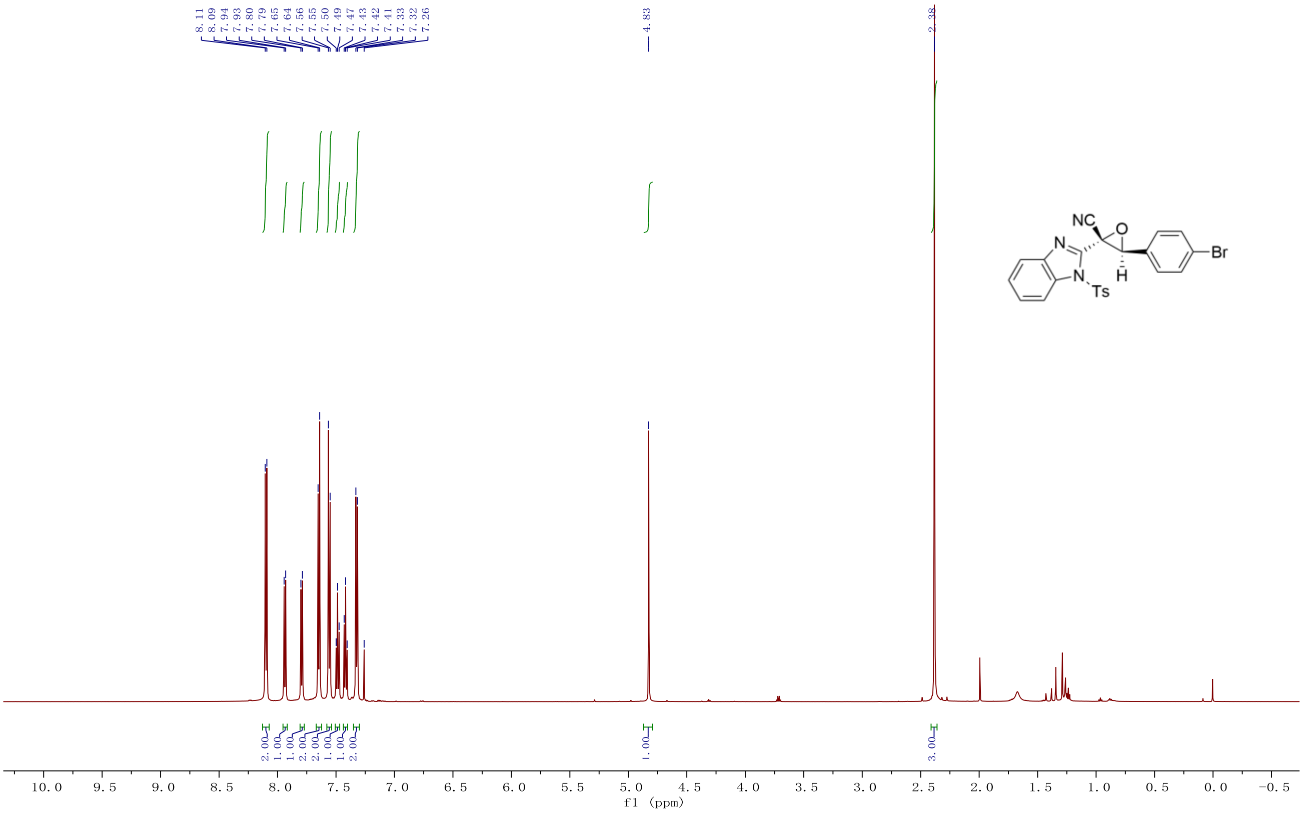

Supplementary Figure 300. ^{13}C NMR spectrum of compound **3al** (150 MHz, $CDCl_3$)

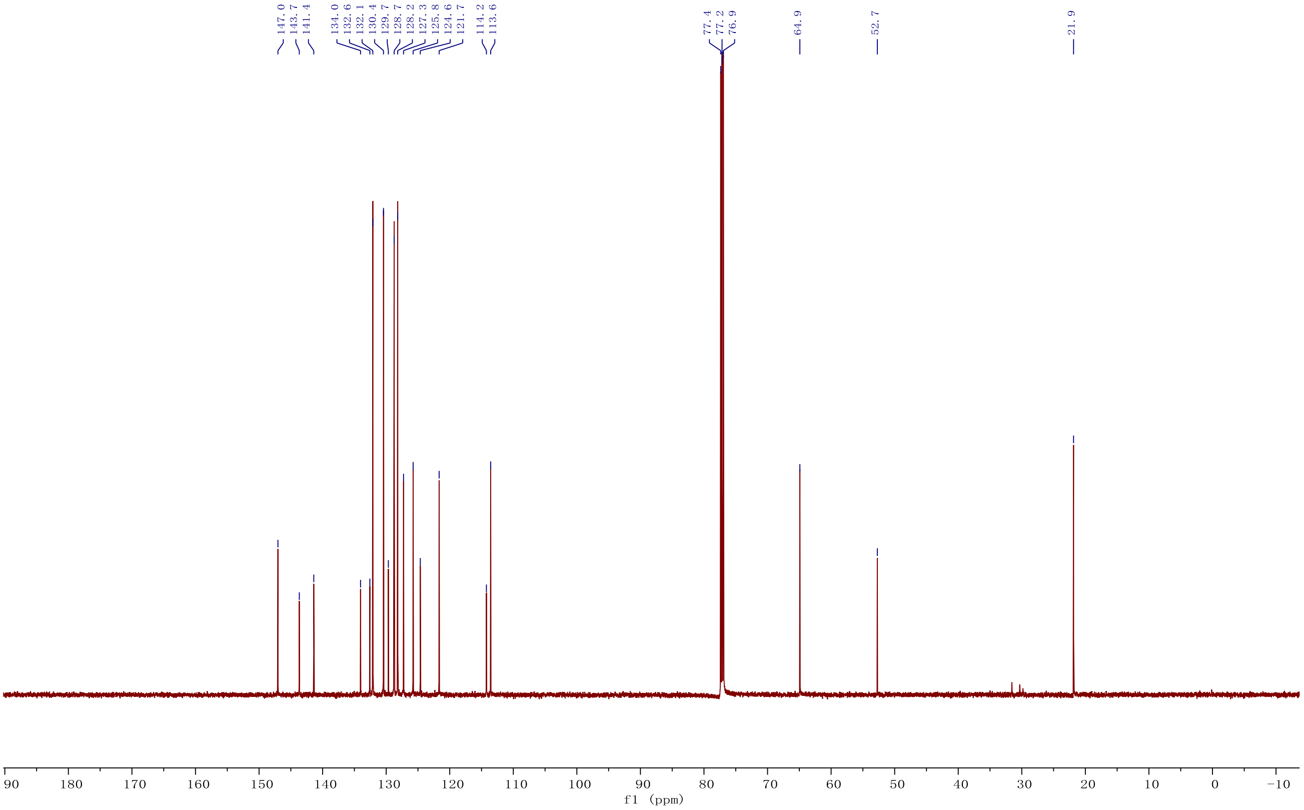

Supplementary Figure 301. ^{1}H NMR spectrum of compound **3am** (600 MHz, $CDCl_3$)

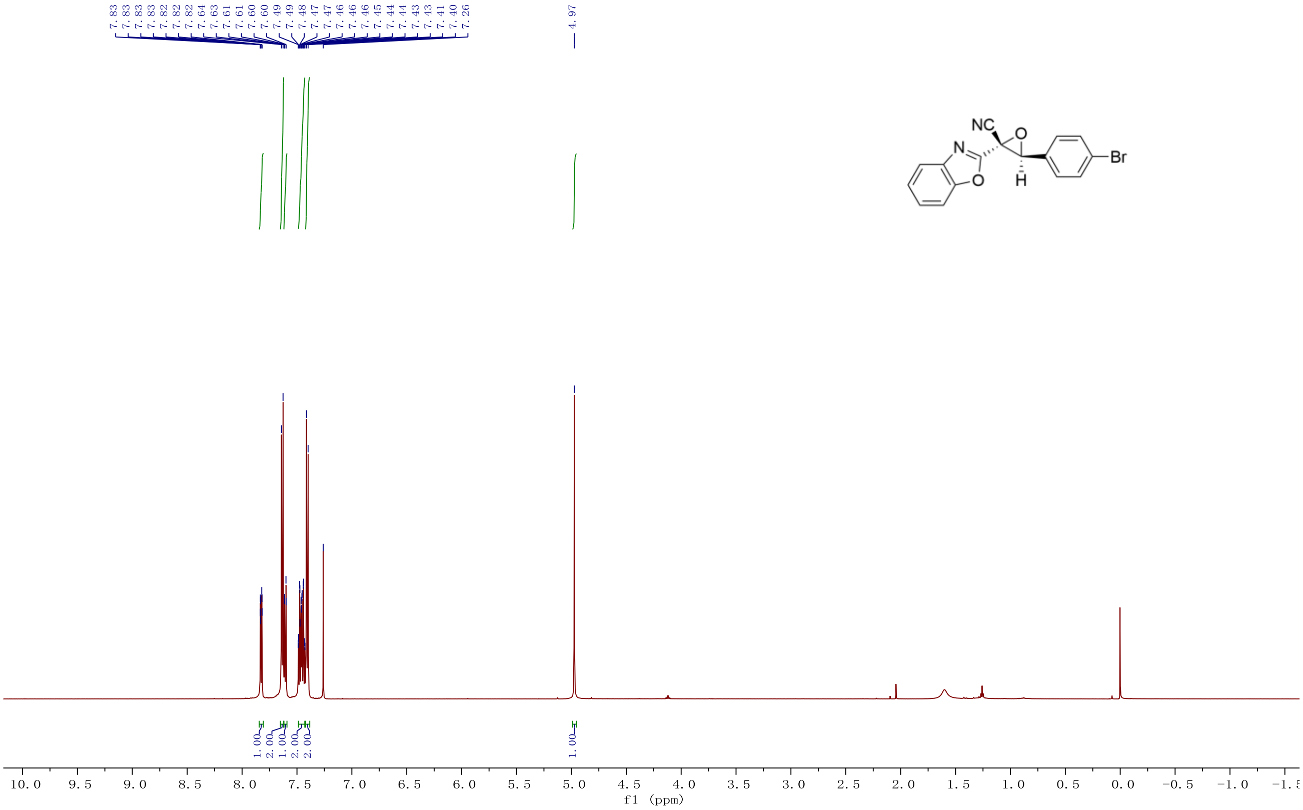

Supplementary Figure 302. ^{13}C NMR spectrum of compound **3am** (150 MHz, $CDCl_3$)

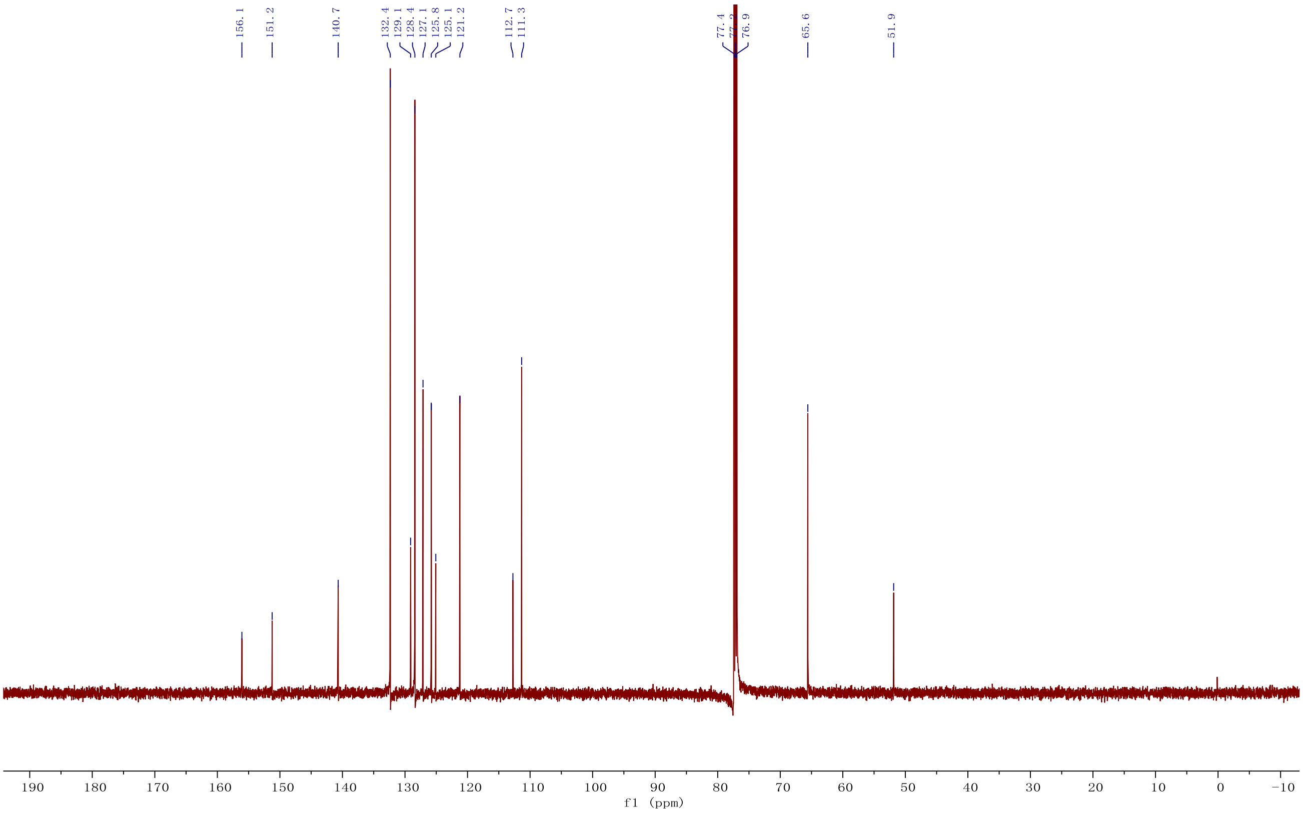

Supplementary Figure 303. ^{1}H NMR spectrum of compound **3an** (400 MHz, $CDCl_3$)

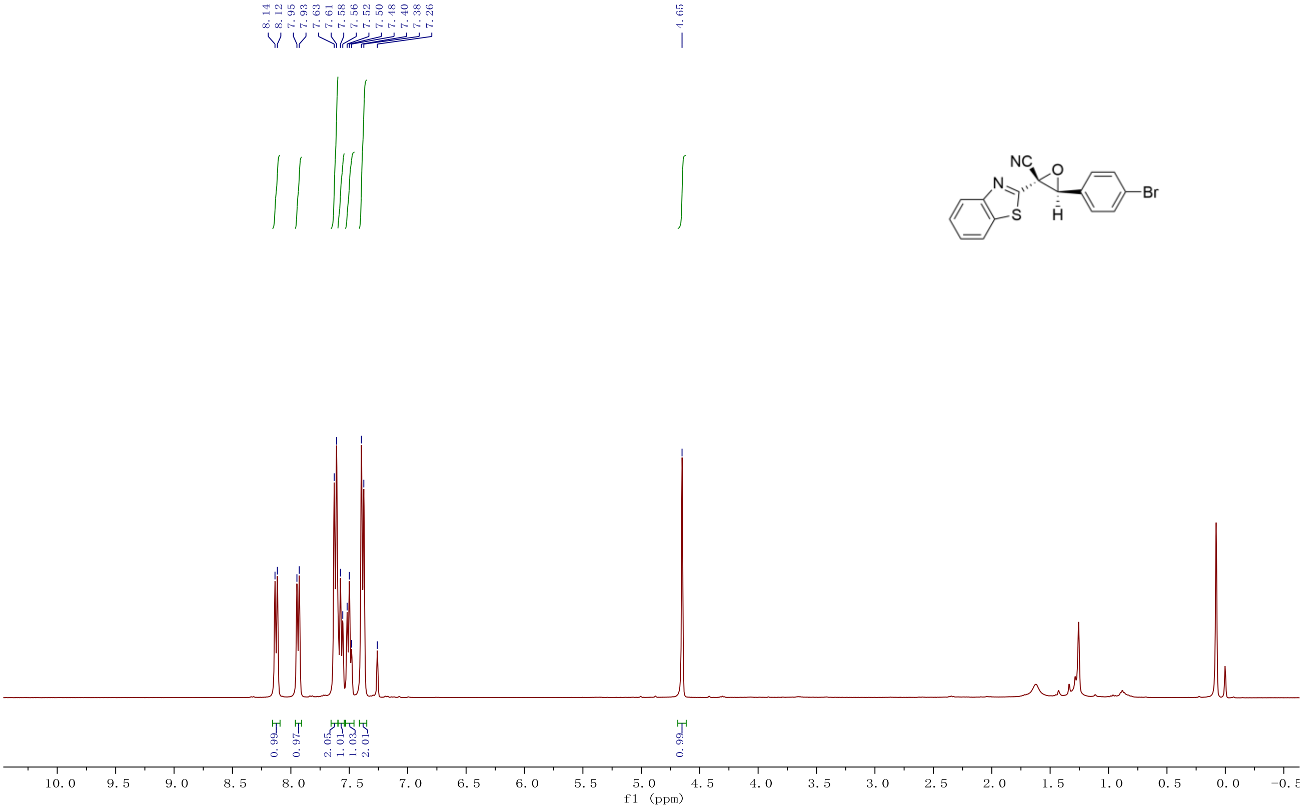

Supplementary Figure 304. ^{13}C NMR spectrum of compound **3an** (100 MHz, $CDCl_3$)

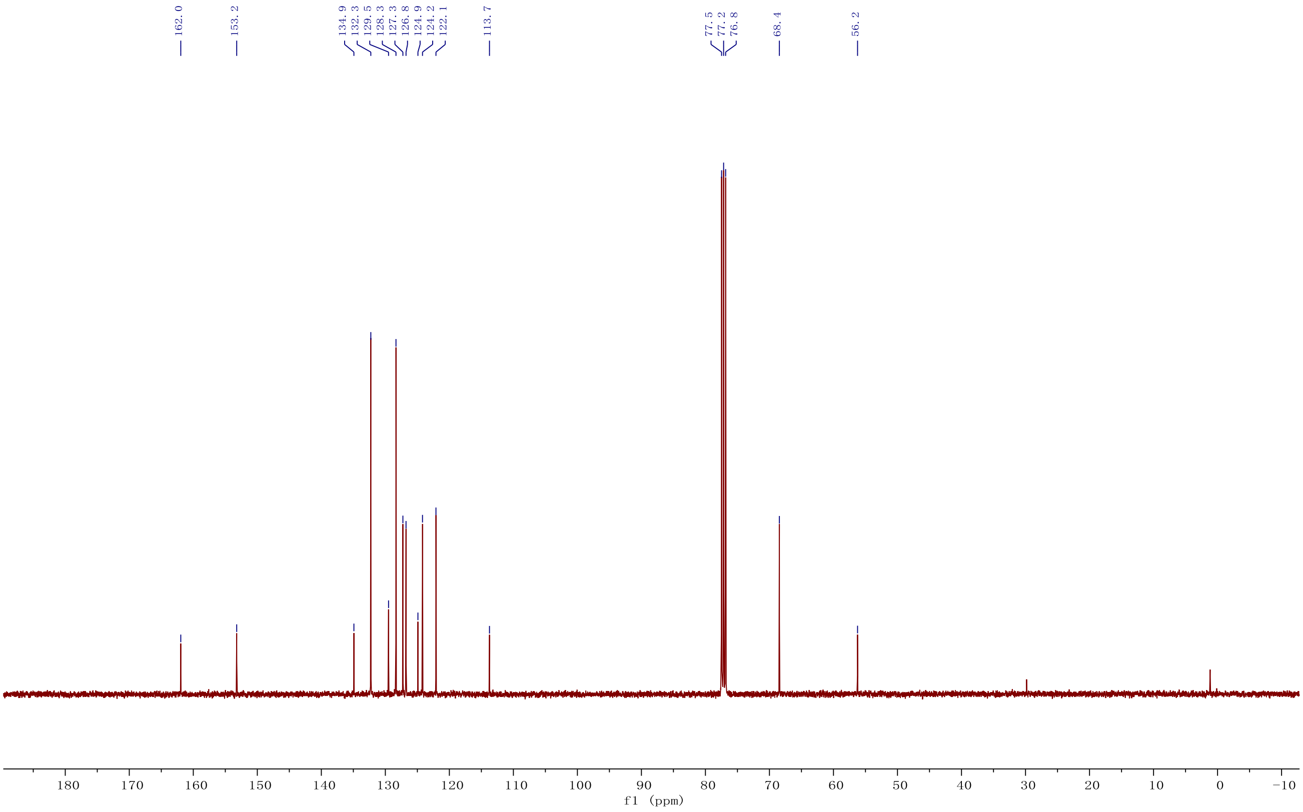

Supplementary Figure 305. ^{1}H NMR spectrum of compound **3ao** (600 MHz, $CDCl_3$)

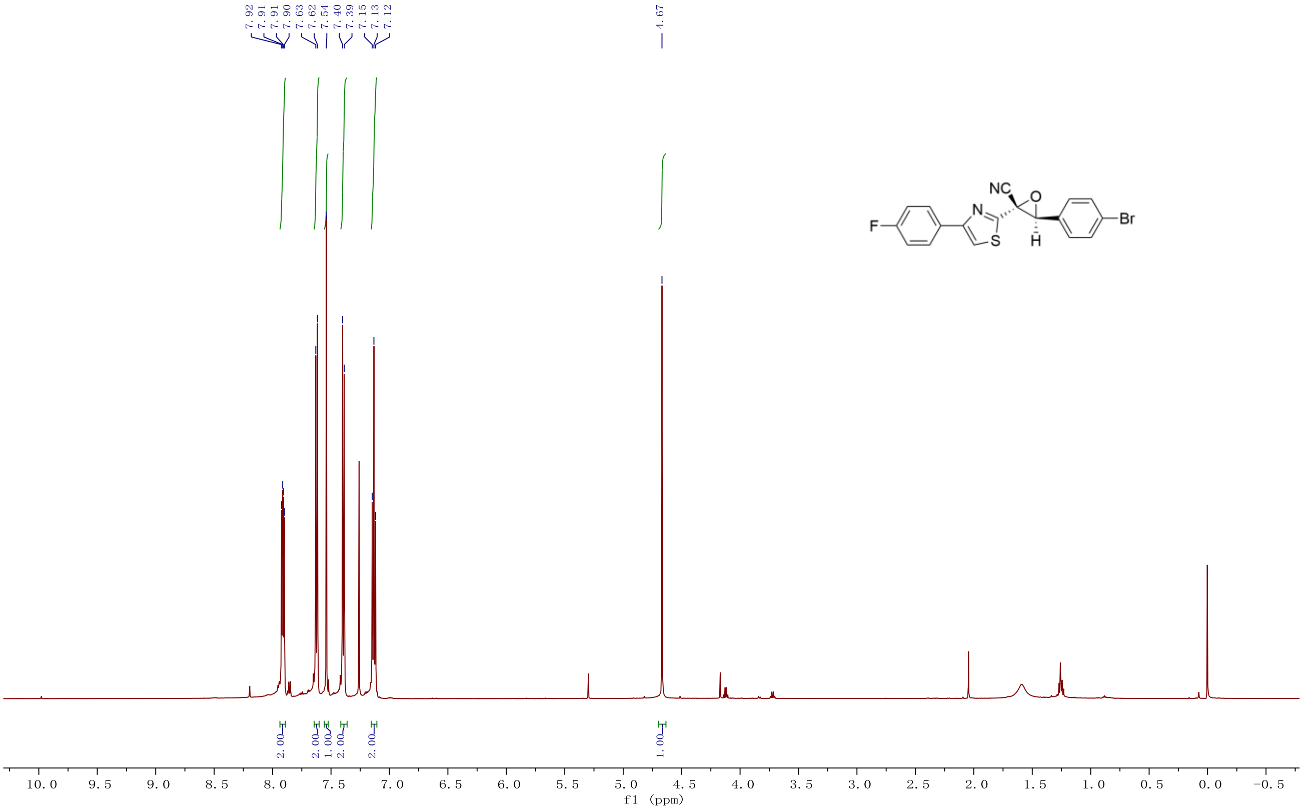

Supplementary Figure 306. ^{13}C NMR spectrum of compound **3ao** (150 MHz, $CDCl_3$)

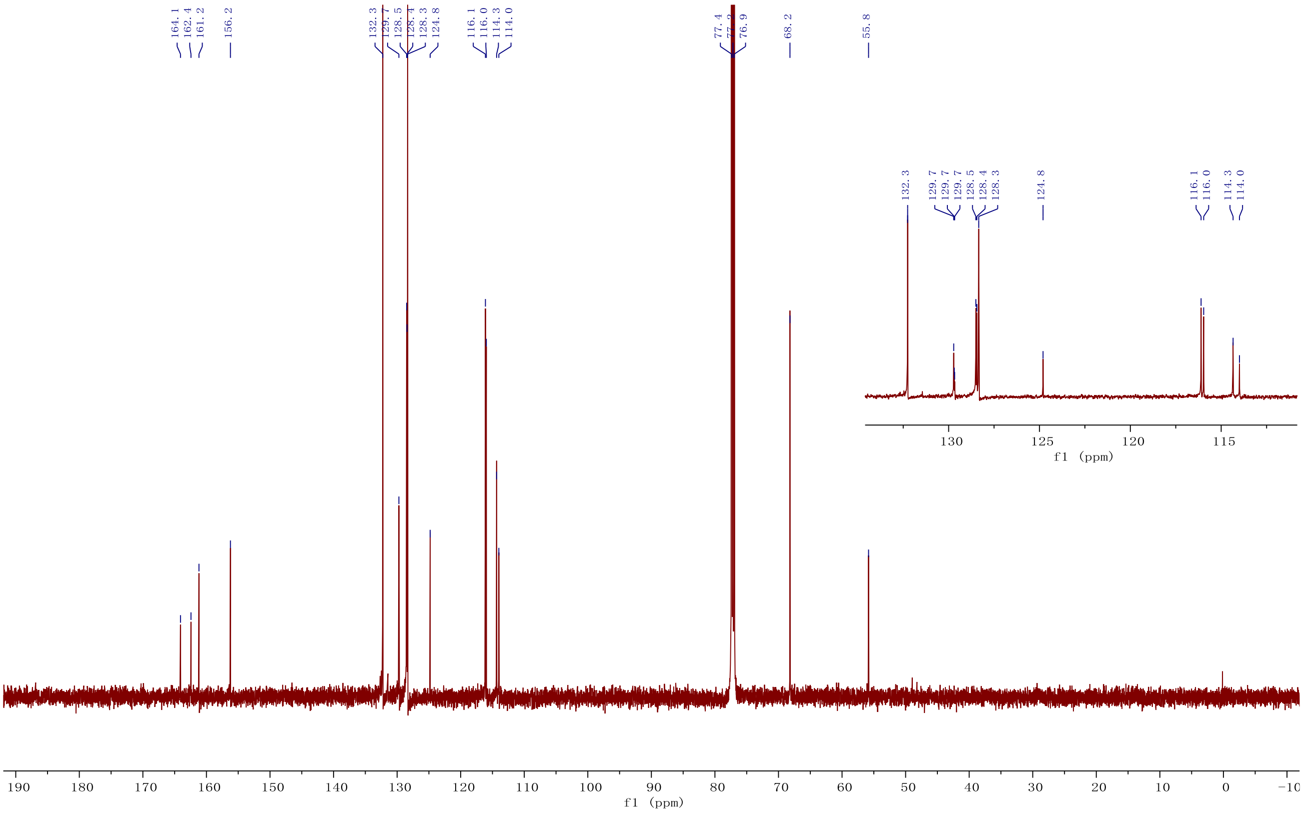

Supplementary Figure 307. ^{19}F NMR spectrum of compound **3ao** (565 MHz, $CDCl_3$)

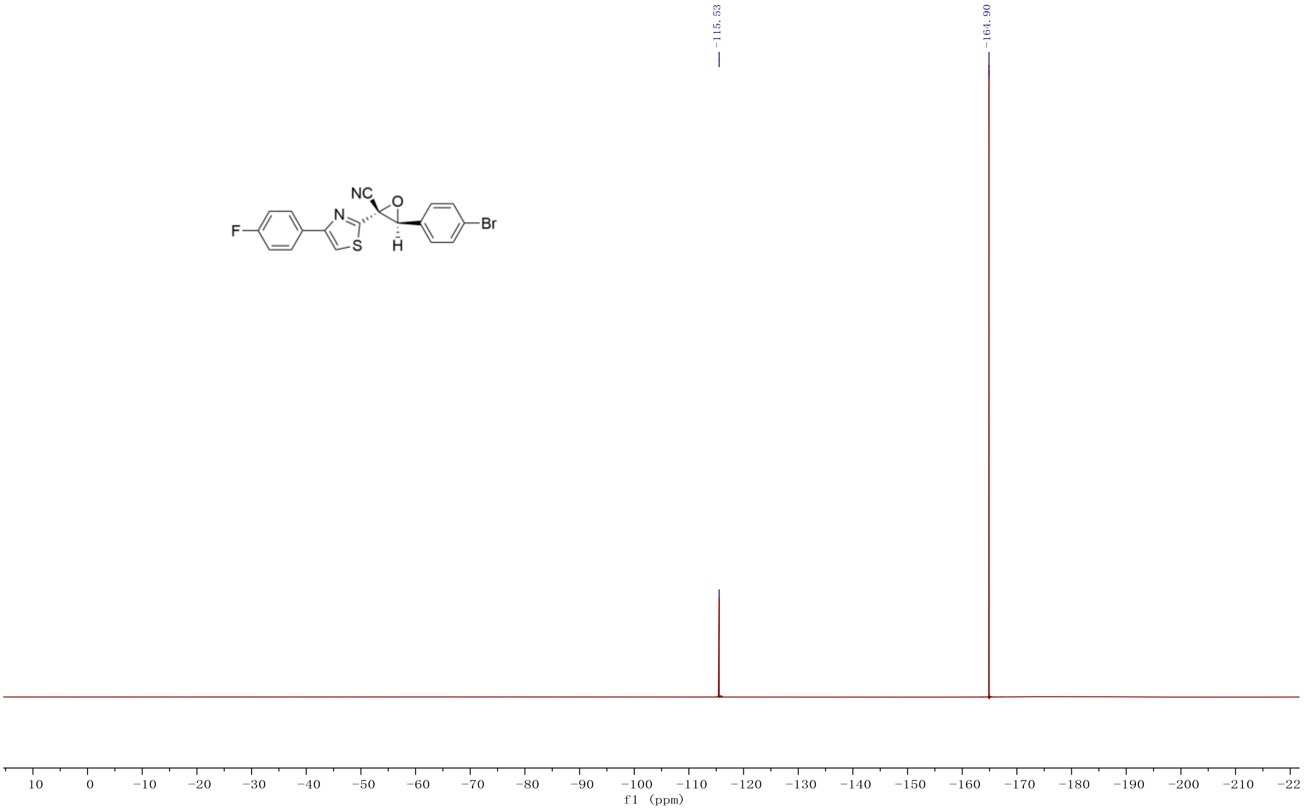

Supplementary Figure 308. ^{1}H NMR spectrum of compound **3ap** (600 MHz, $CDCl_3$)

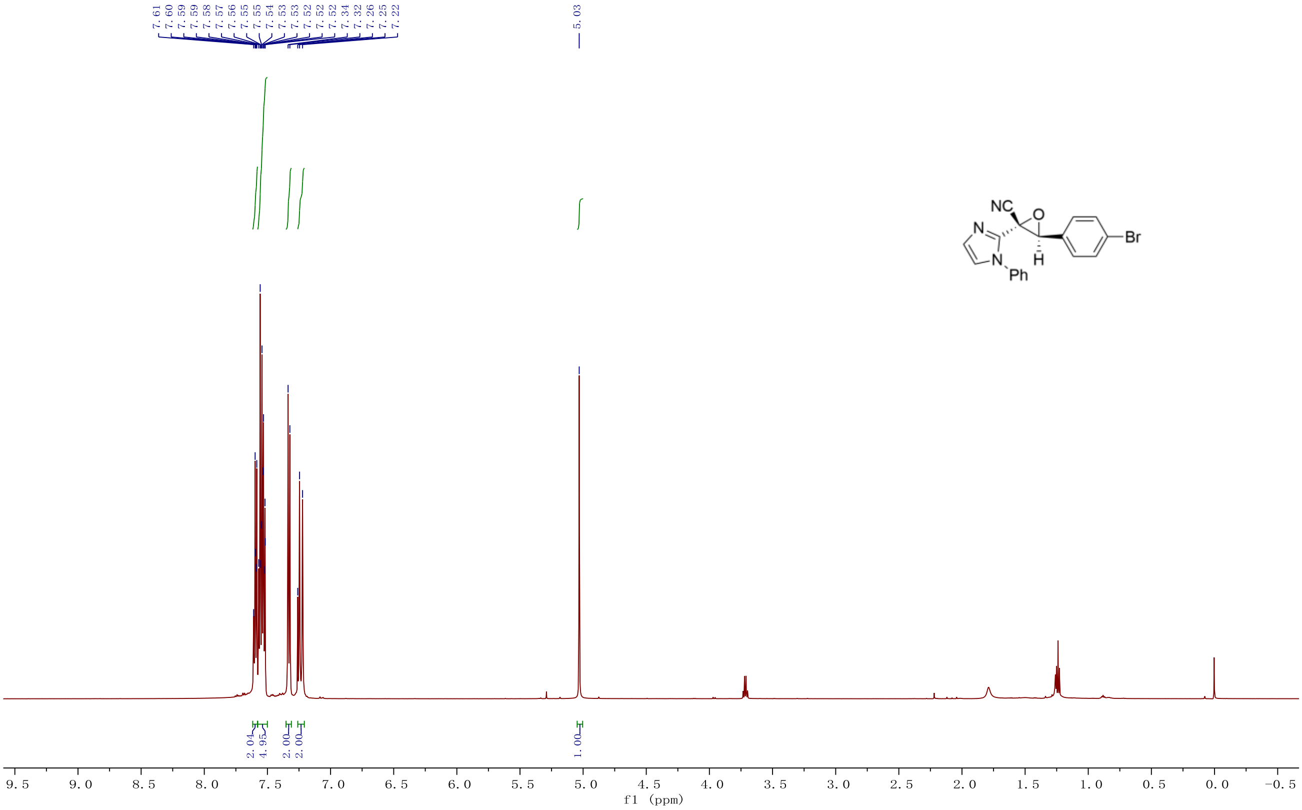

Supplementary Figure 309. ^{13}C NMR spectrum of compound **3ap** (150 MHz, $CDCl_3$)

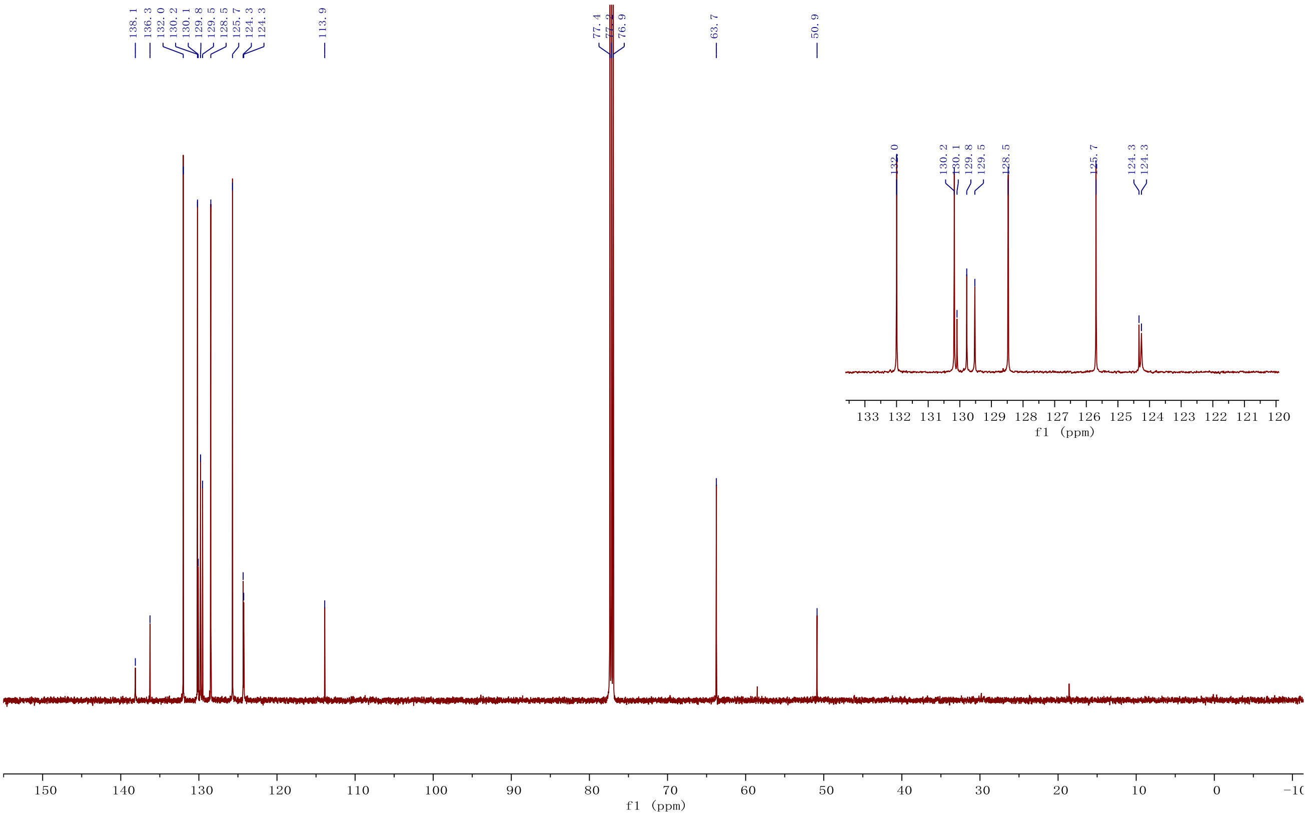

Supplementary Figure 310. ^{1}H NMR spectrum of compound **3aq** (400 MHz, $CDCl_3$)

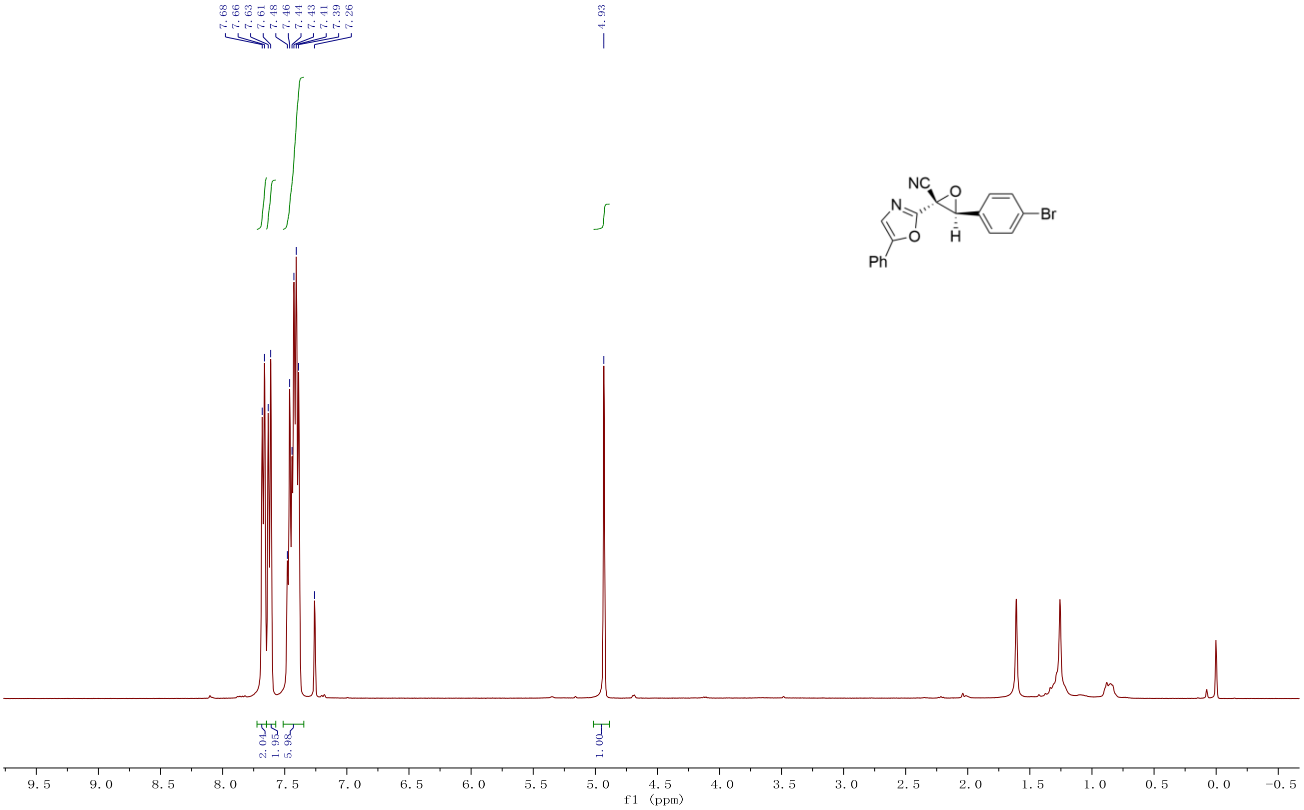

Supplementary Figure 311. ^{13}C NMR spectrum of compound **3aq** (100 MHz, $CDCl_3$)

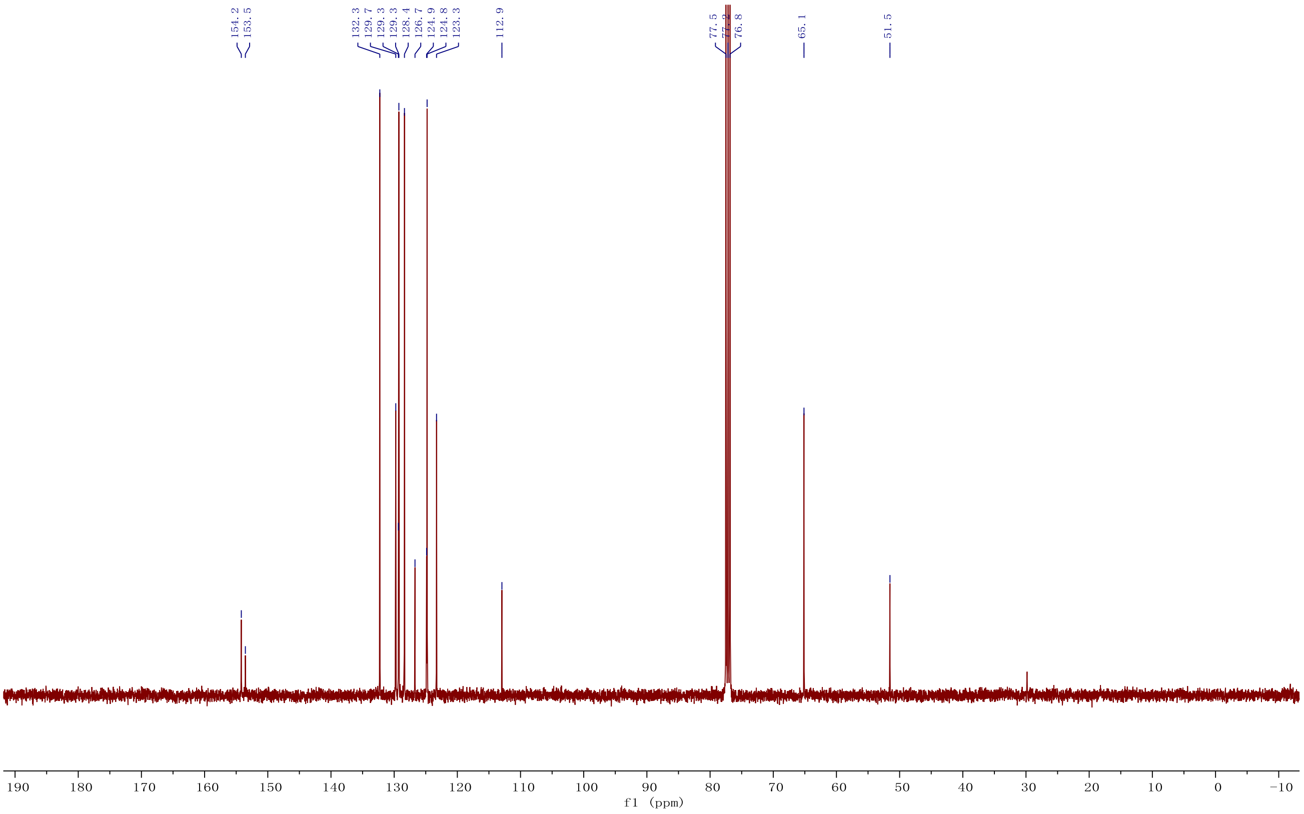

Supplementary Figure 312. ^{1}H NMR spectrum of compound **3ar** (400 MHz, $CDCl_3$)

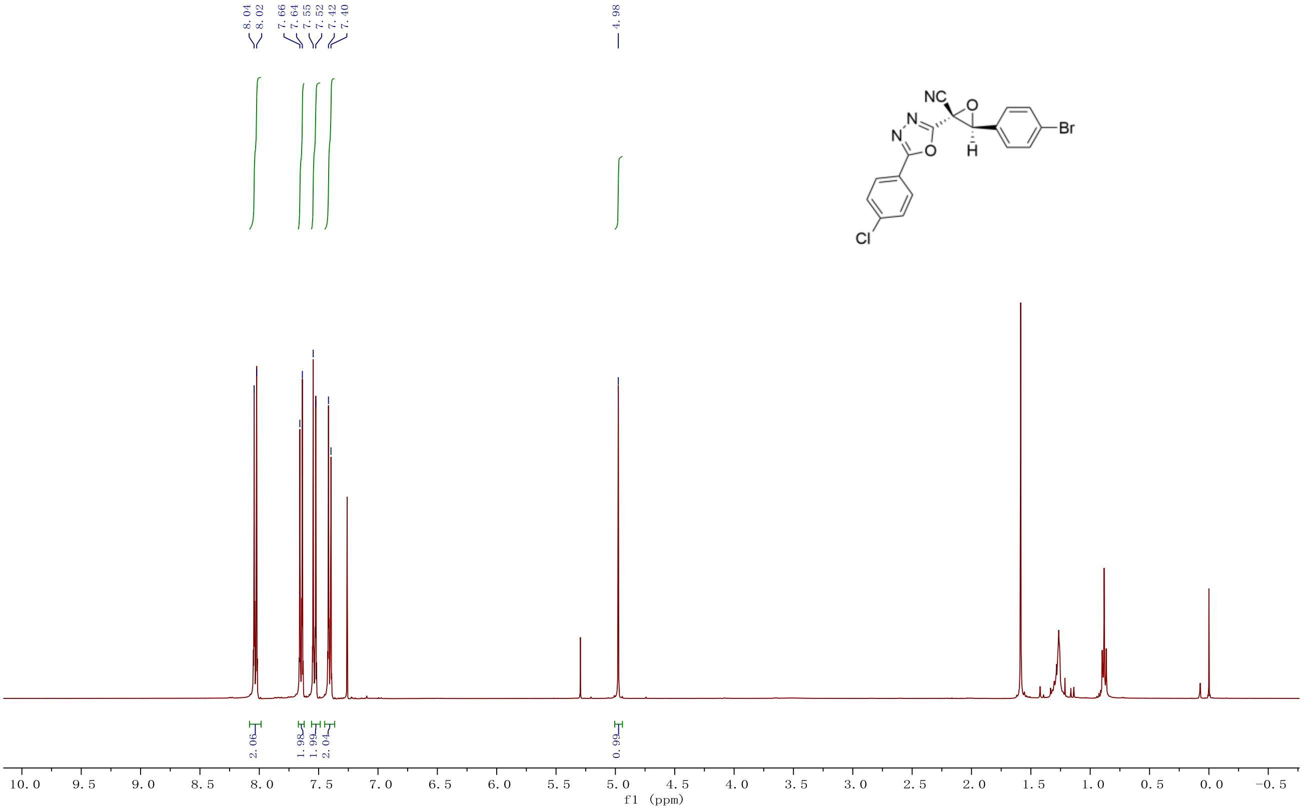

Supplementary Figure 313. ^{13}C NMR spectrum of compound **3ar** (100 MHz, $CDCl_3$)

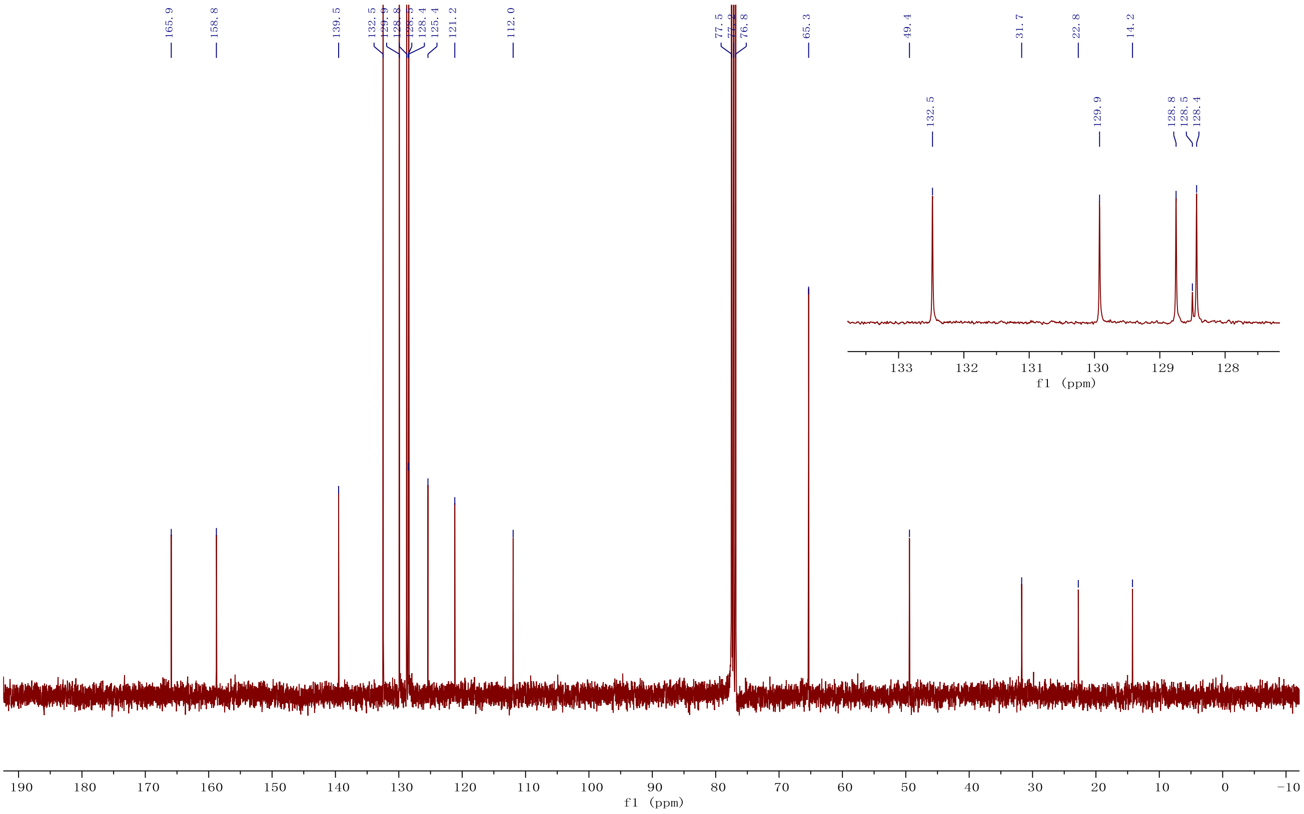

Supplementary Figure 314. ^{1}H NMR spectrum of compound **3as** (400 MHz, $CDCl_3$)

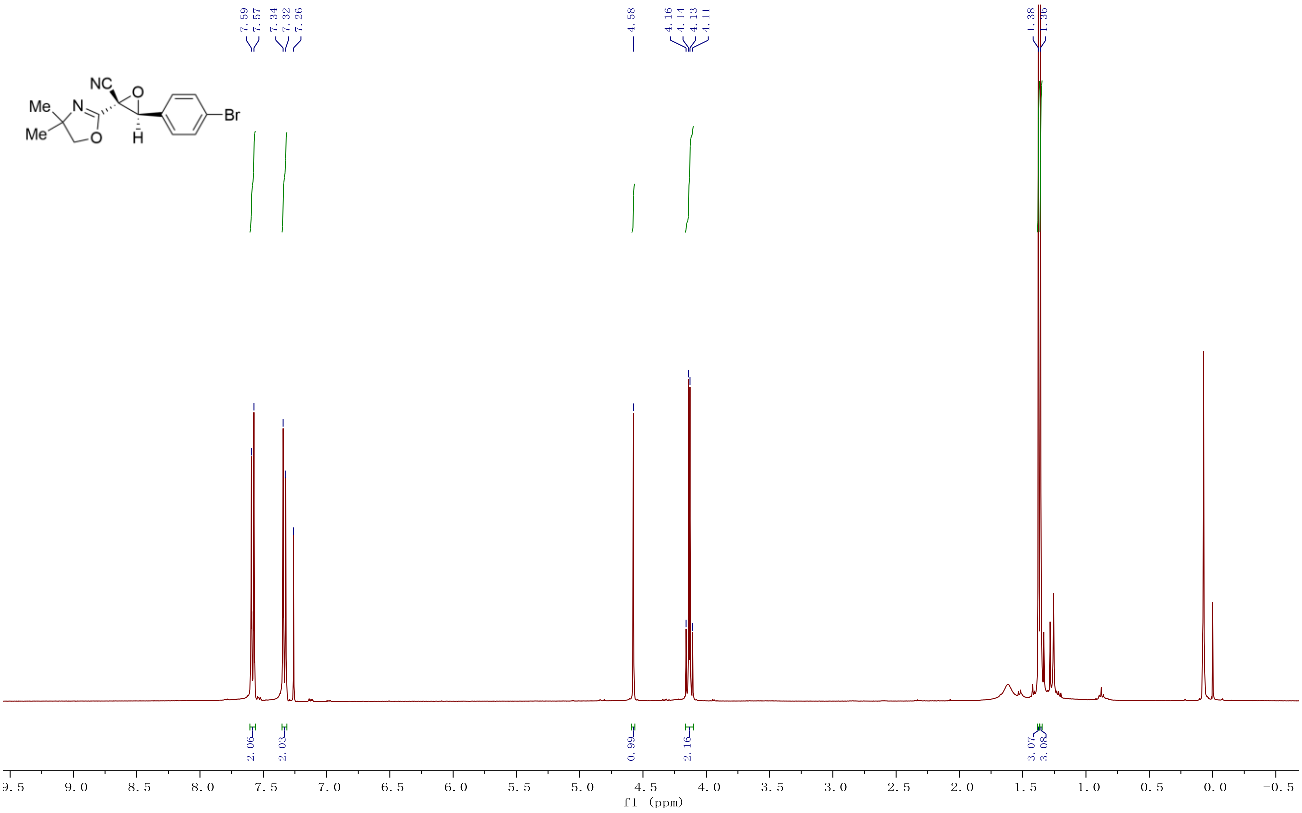

Supplementary Figure 315. ^{13}C NMR spectrum of compound **3as** (100 MHz, $CDCl_3$)

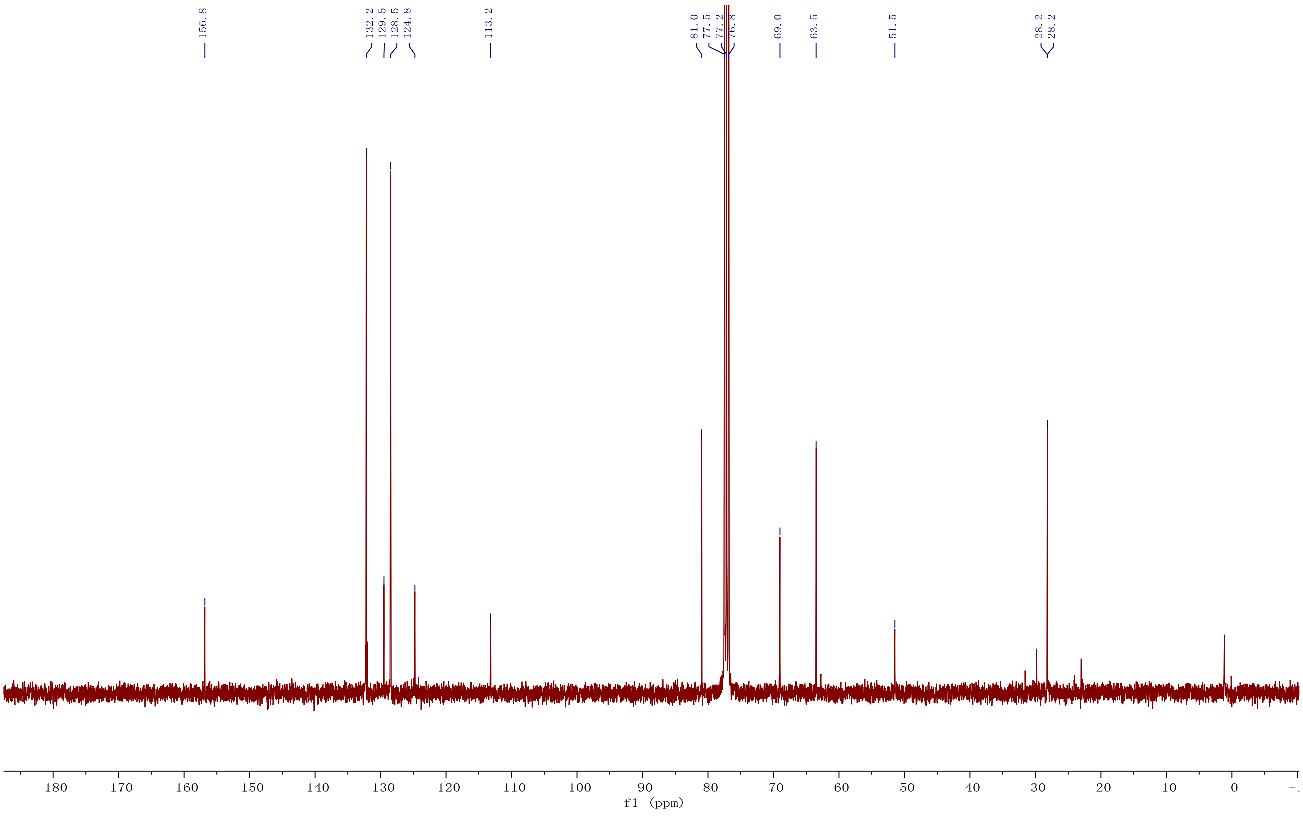

Supplementary Figure 316. ^{1}H NMR spectrum of compound **3ba** (600 MHz, $CDCl_3$)

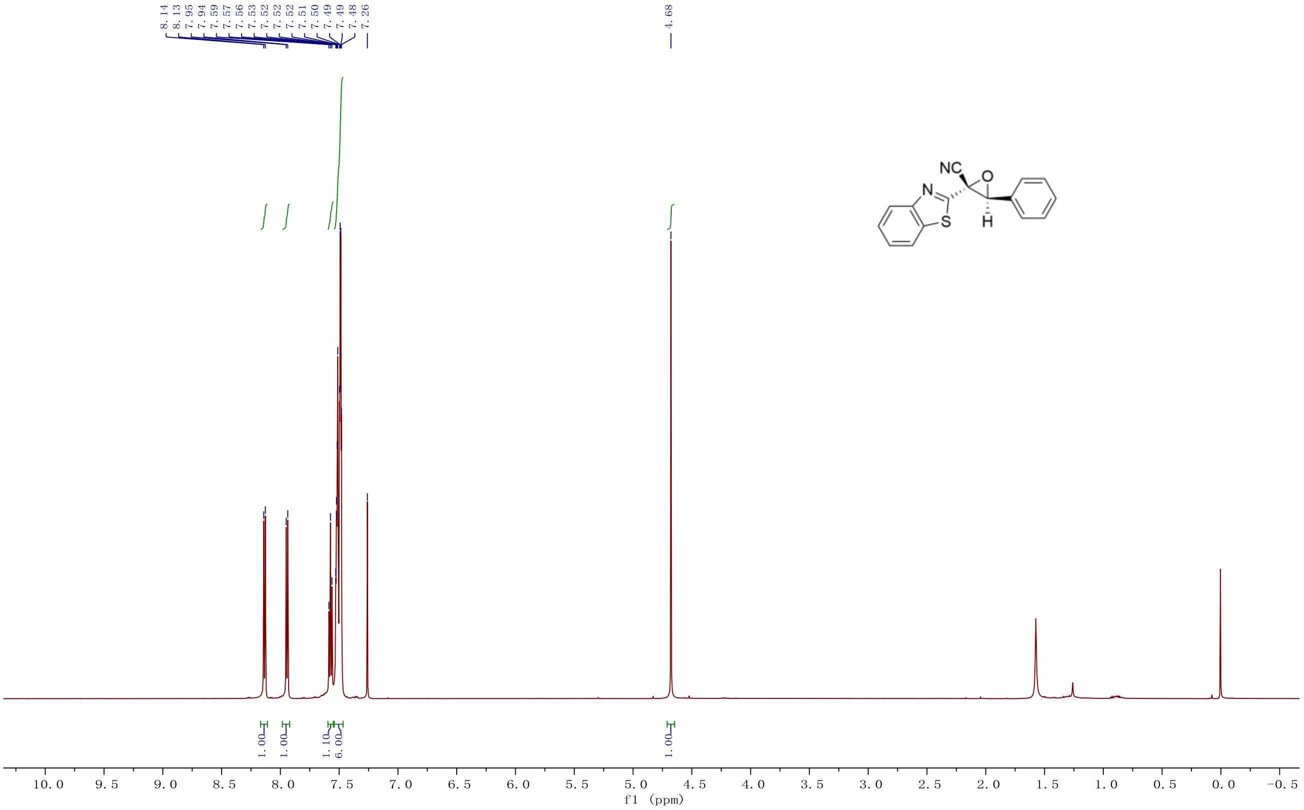

Supplementary Figure 317. ^{13}C NMR spectrum of compound **3ba** (150 MHz, $CDCl_3$)

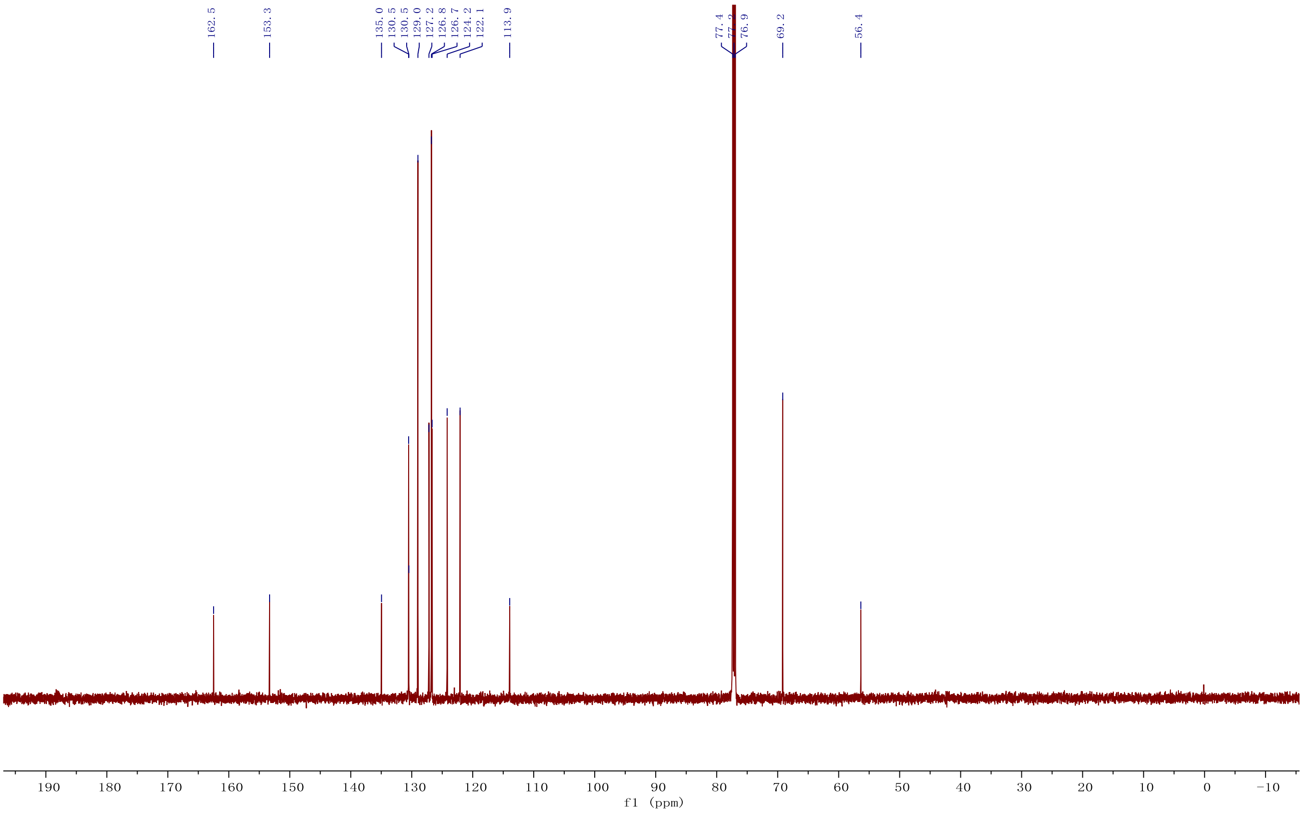

Supplementary Figure 318. ^{1}H NMR spectrum of compound **3bb** (400 MHz, $CDCl_3$)

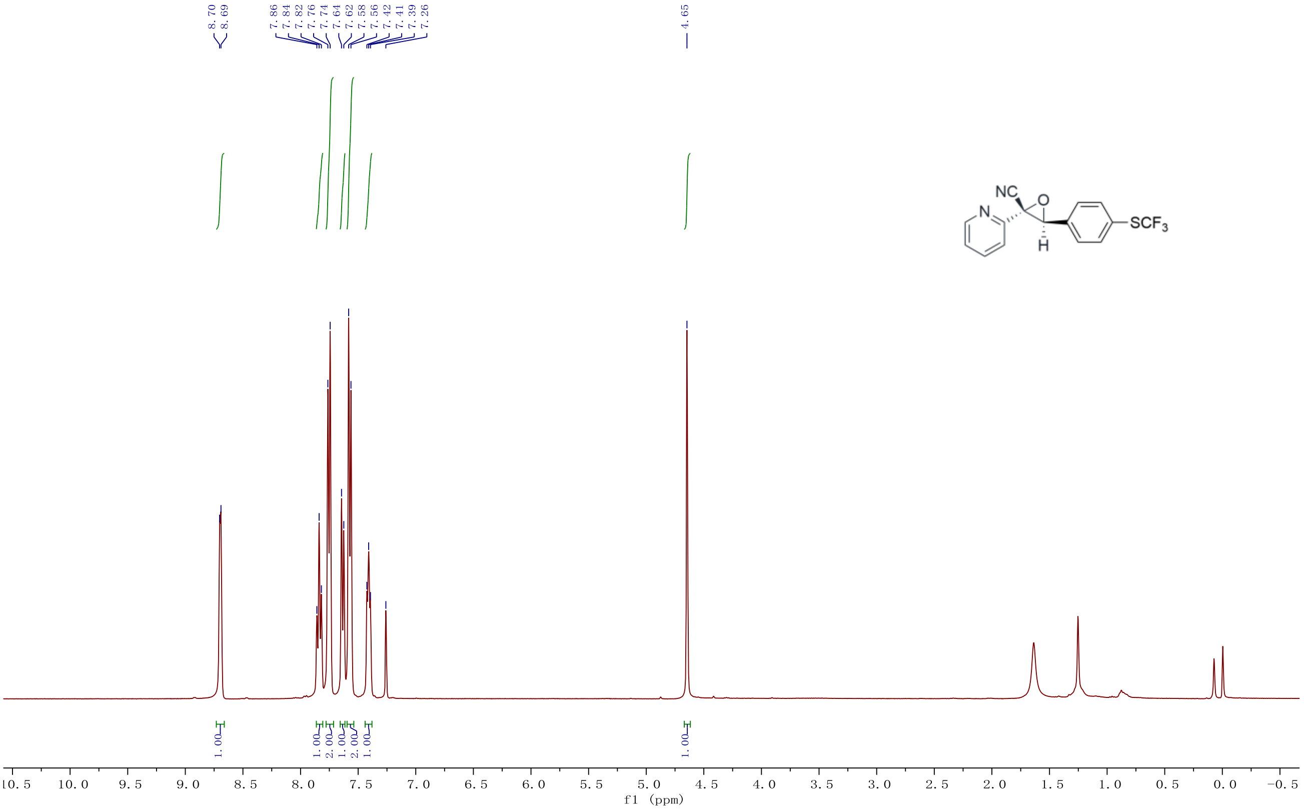

Supplementary Figure 319. ^{13}C NMR spectrum of compound **3bb** (100 MHz, $CDCl_3$)

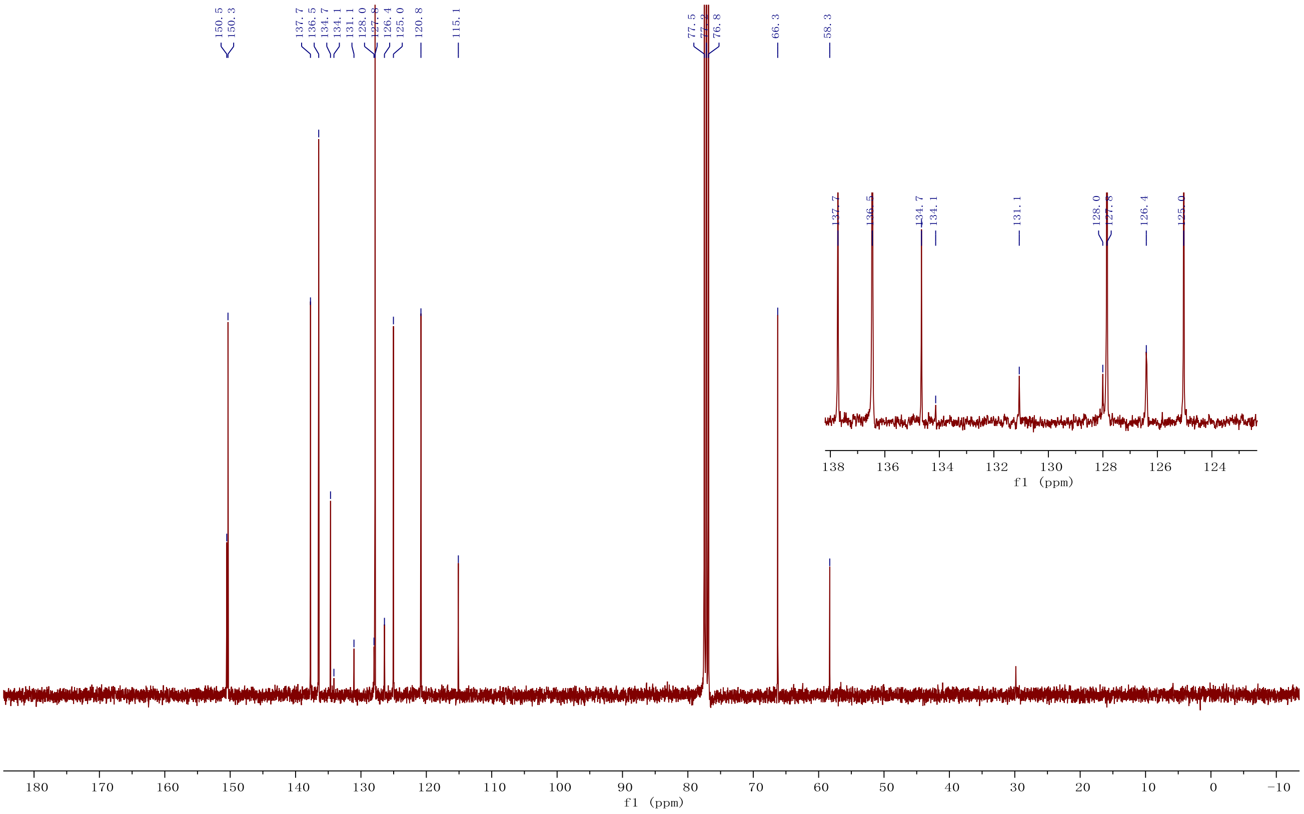

Supplementary Figure 320. ^{19}F NMR spectrum of compound **3bb** (376 MHz, $CDCl_3$)

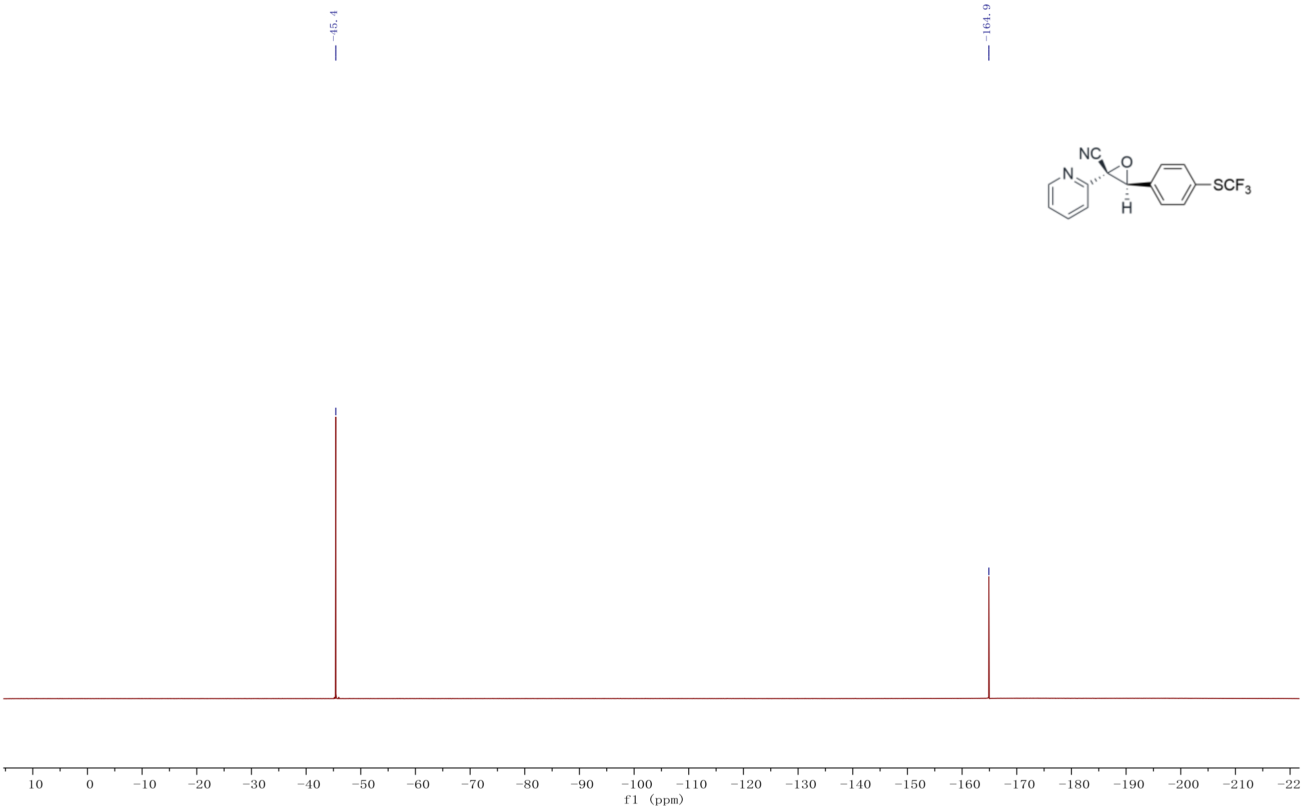

Supplementary Figure 321. ^{1}H NMR spectrum of compound **3bc** (400 MHz, $CDCl_3$)

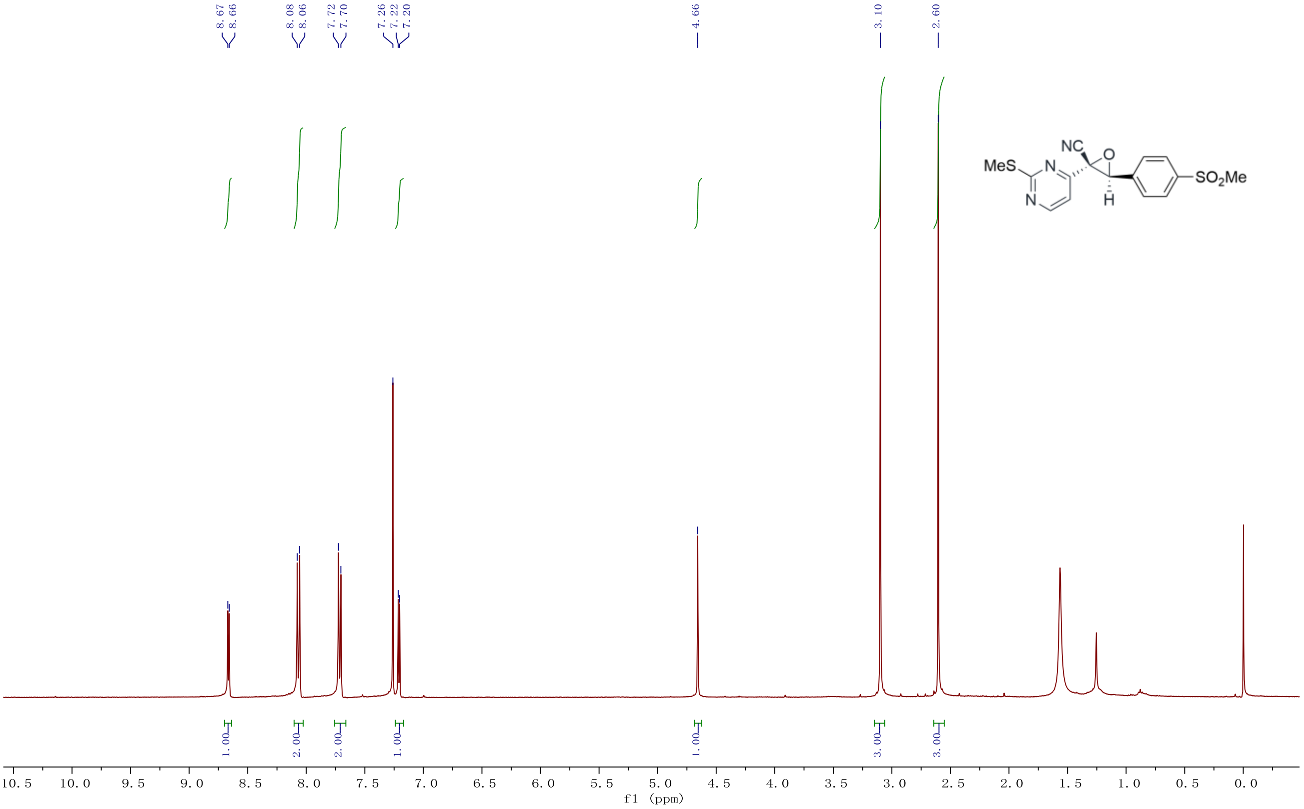

Supplementary Figure 322. ^{13}C NMR spectrum of compound **3bc** (100 MHz, $CDCl_3$)

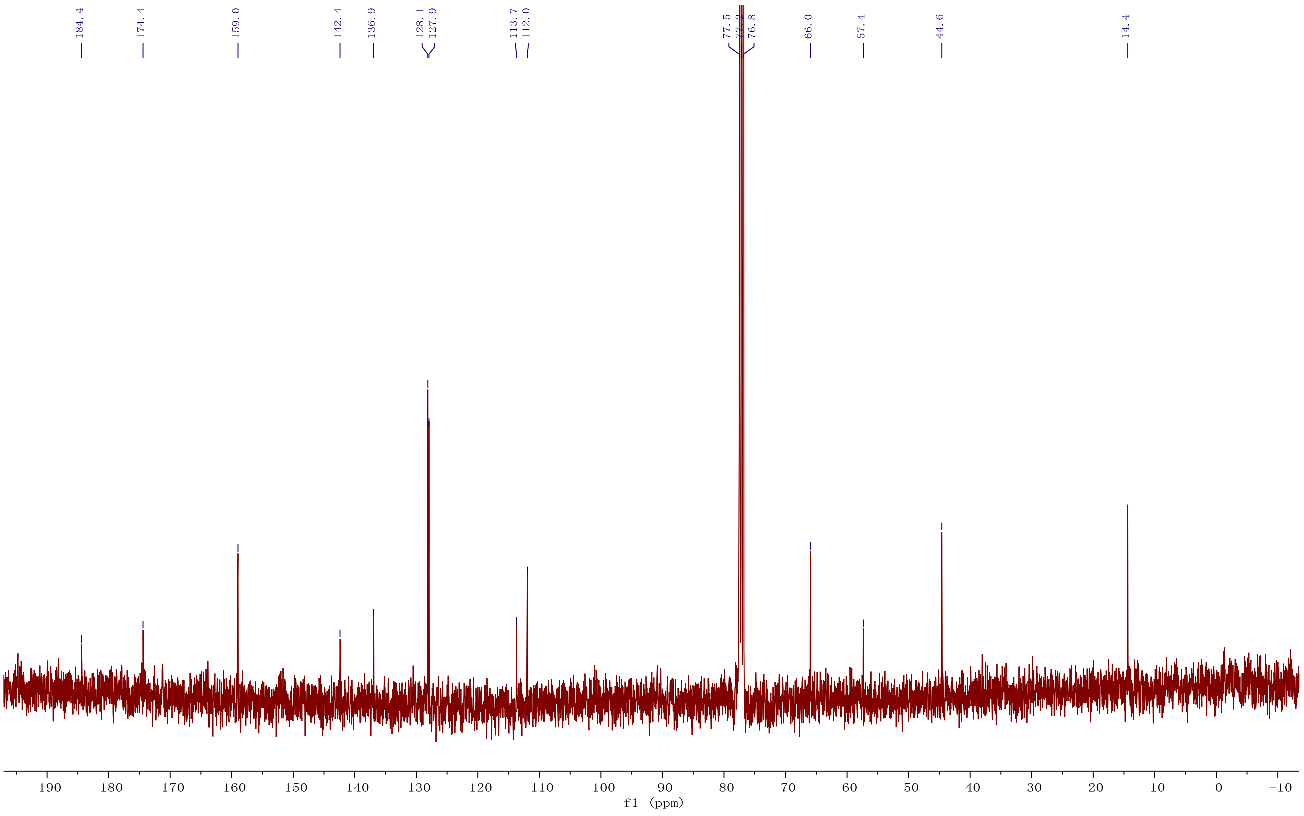

Supplementary Figure 323. ^{1}H NMR spectrum of compound **3bd** (400 MHz, DMSO-d_6)

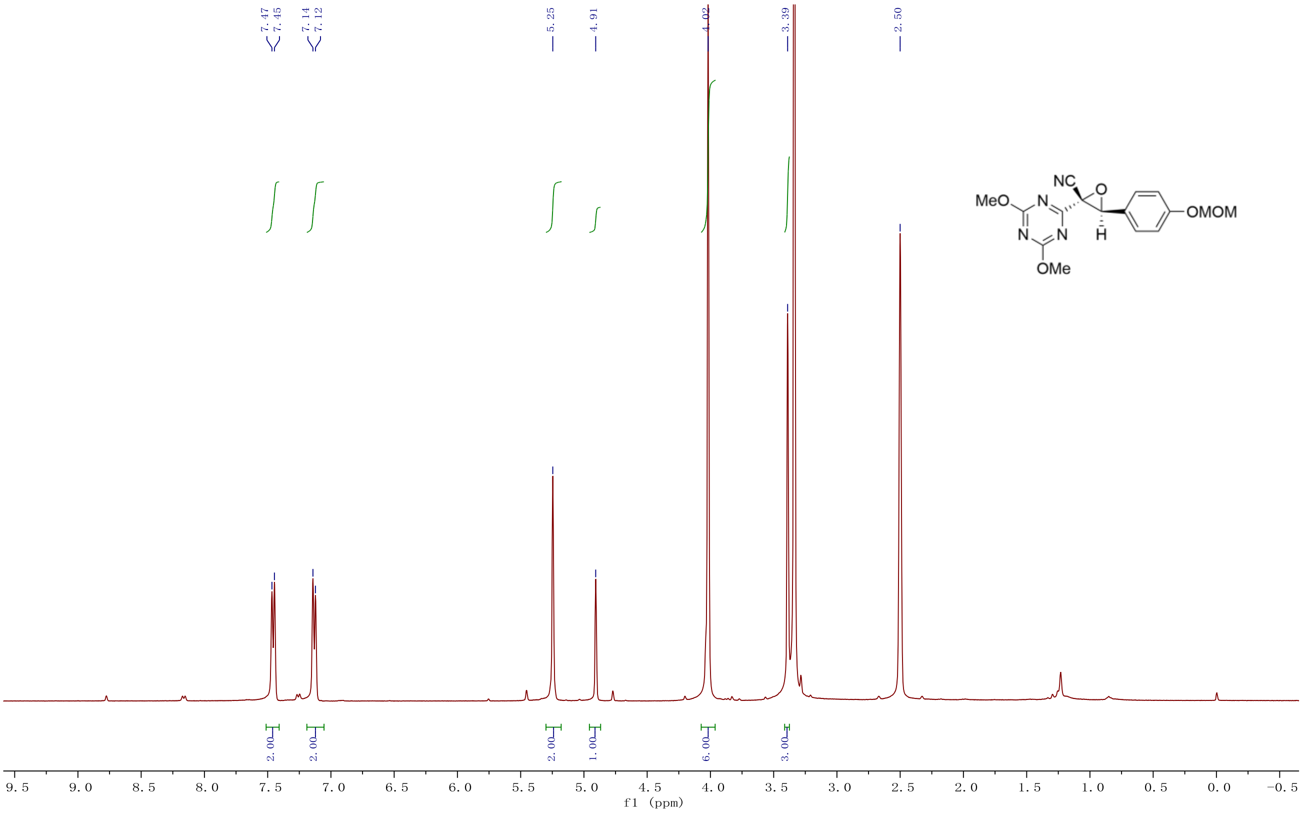

Supplementary Figure 324. ^{13}C NMR spectrum of compound **3bd** (100 MHz, DMSO-d_6)

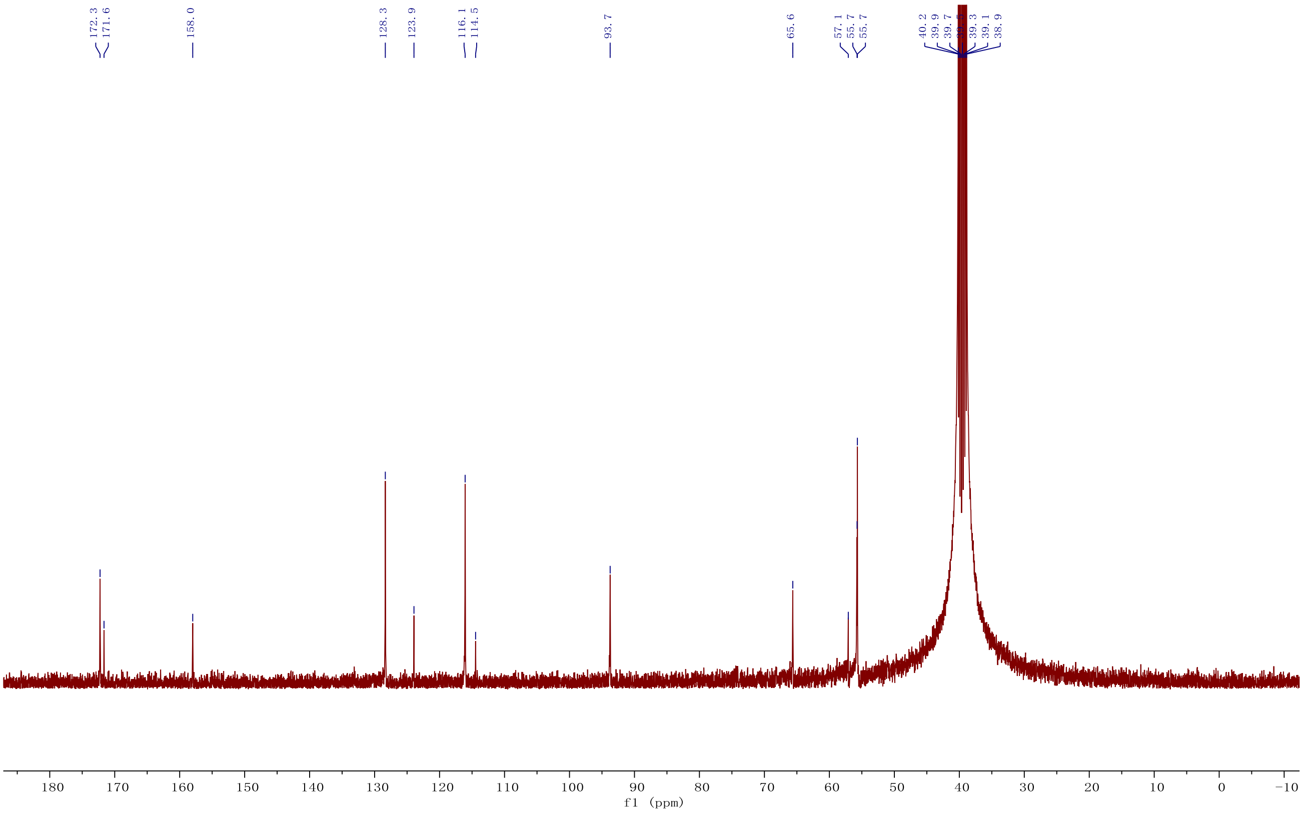

Supplementary Figure 325. ^{1}H NMR spectrum of compound **3be** (400 MHz, $CDCl_3$)

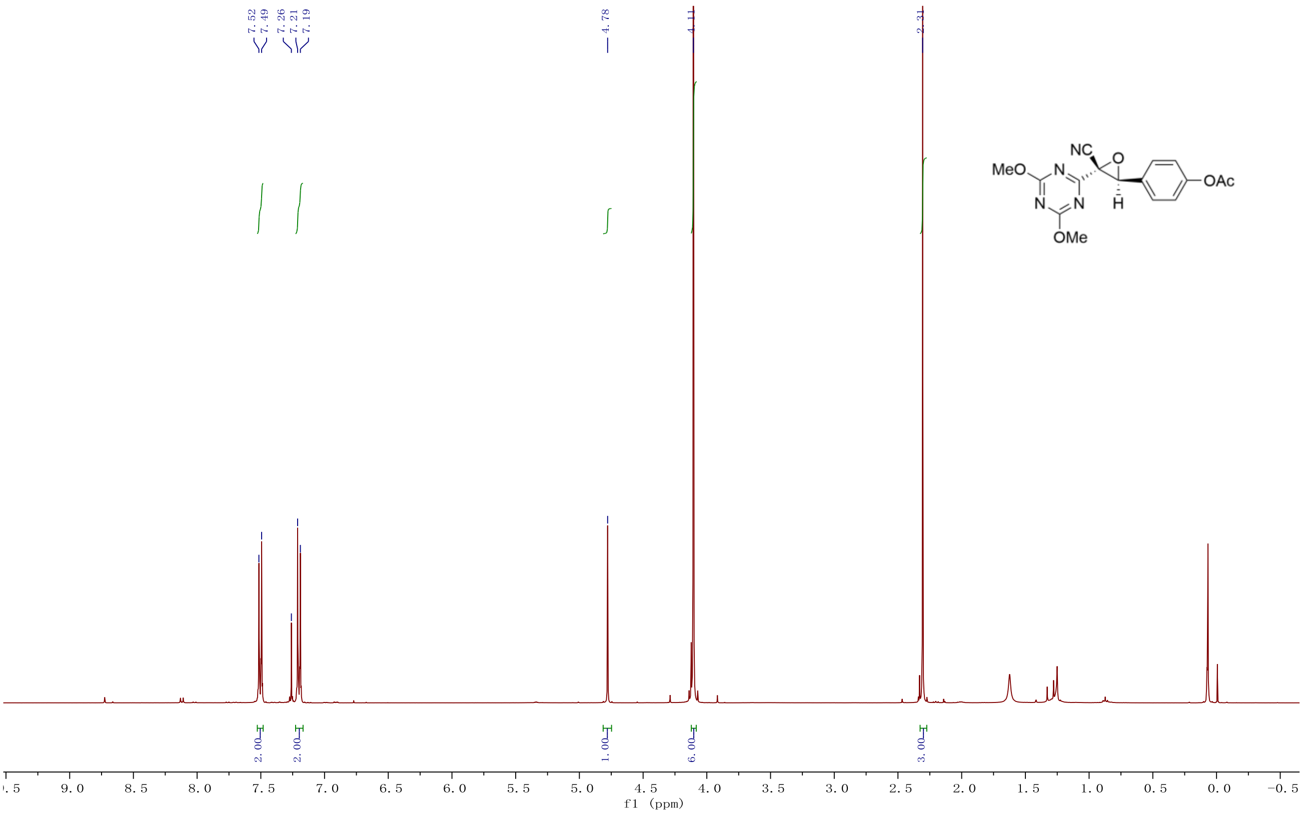

Supplementary Figure 326. ^{13}C NMR spectrum of compound **3be** (100 MHz, $CDCl_3$)

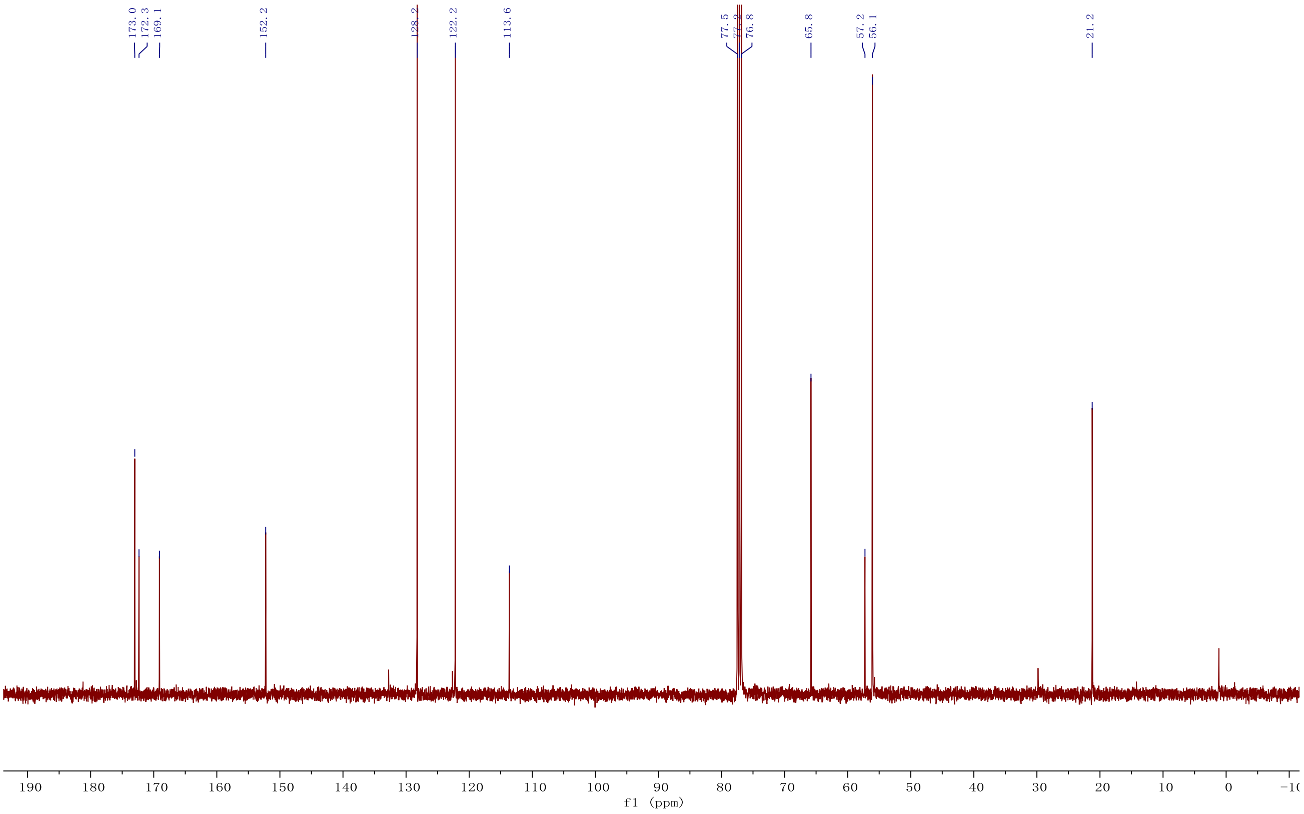

Supplementary Figure 327. ^{1}H NMR spectrum of compound **3bf** (600 MHz, $CDCl_3$)

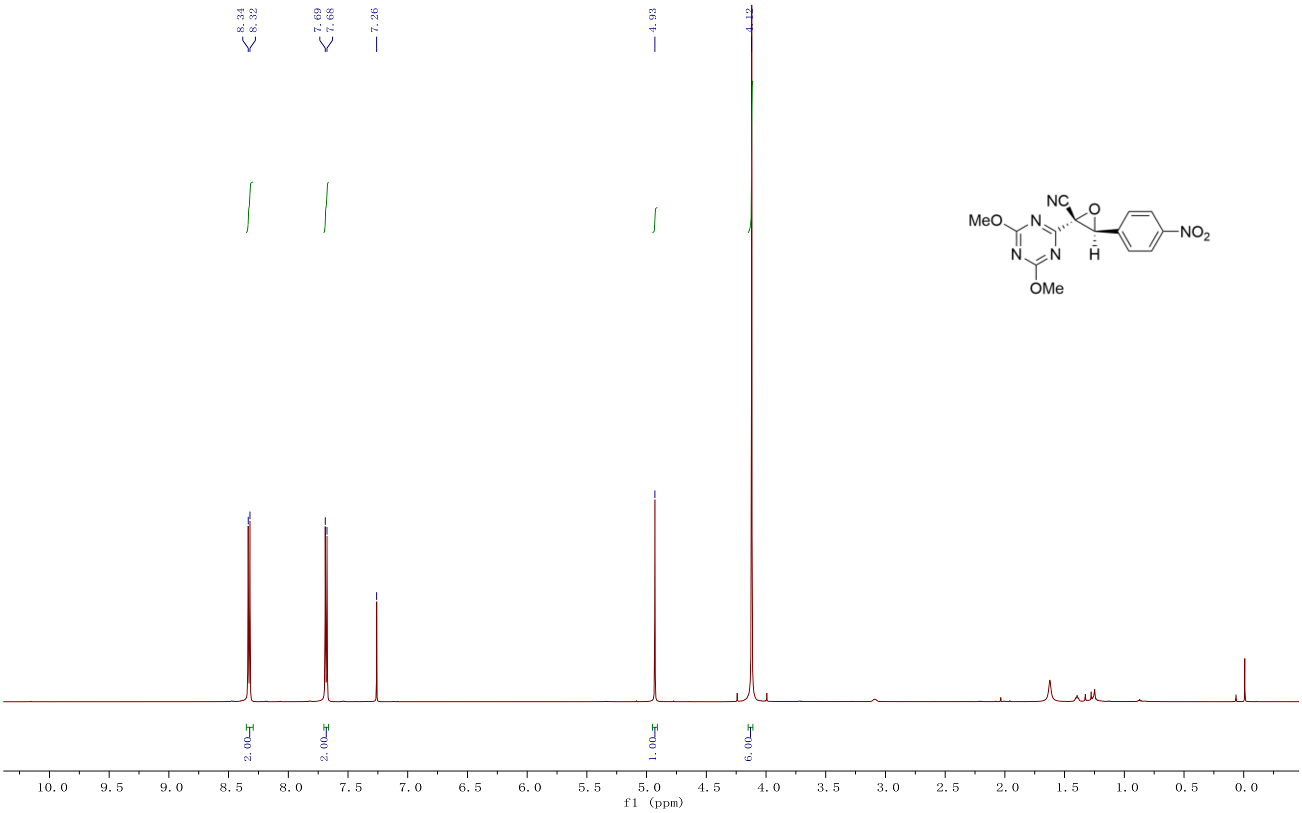

Supplementary Figure 328. ^{13}C NMR spectrum of compound **3bf** (150 MHz, $CDCl_3$)

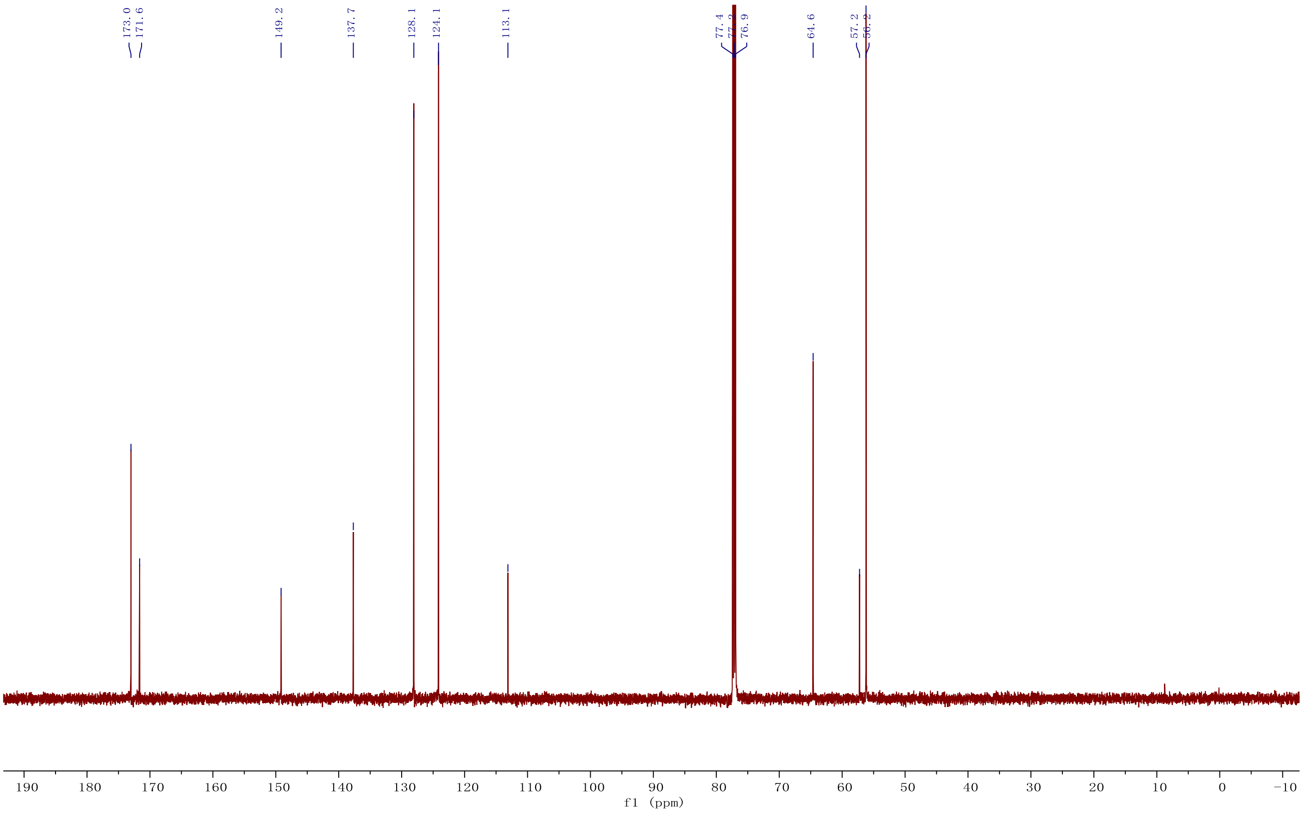

Supplementary Figure 329. ^{1}H NMR spectrum of compound **3bg** (400 MHz, $CDCl_3$)

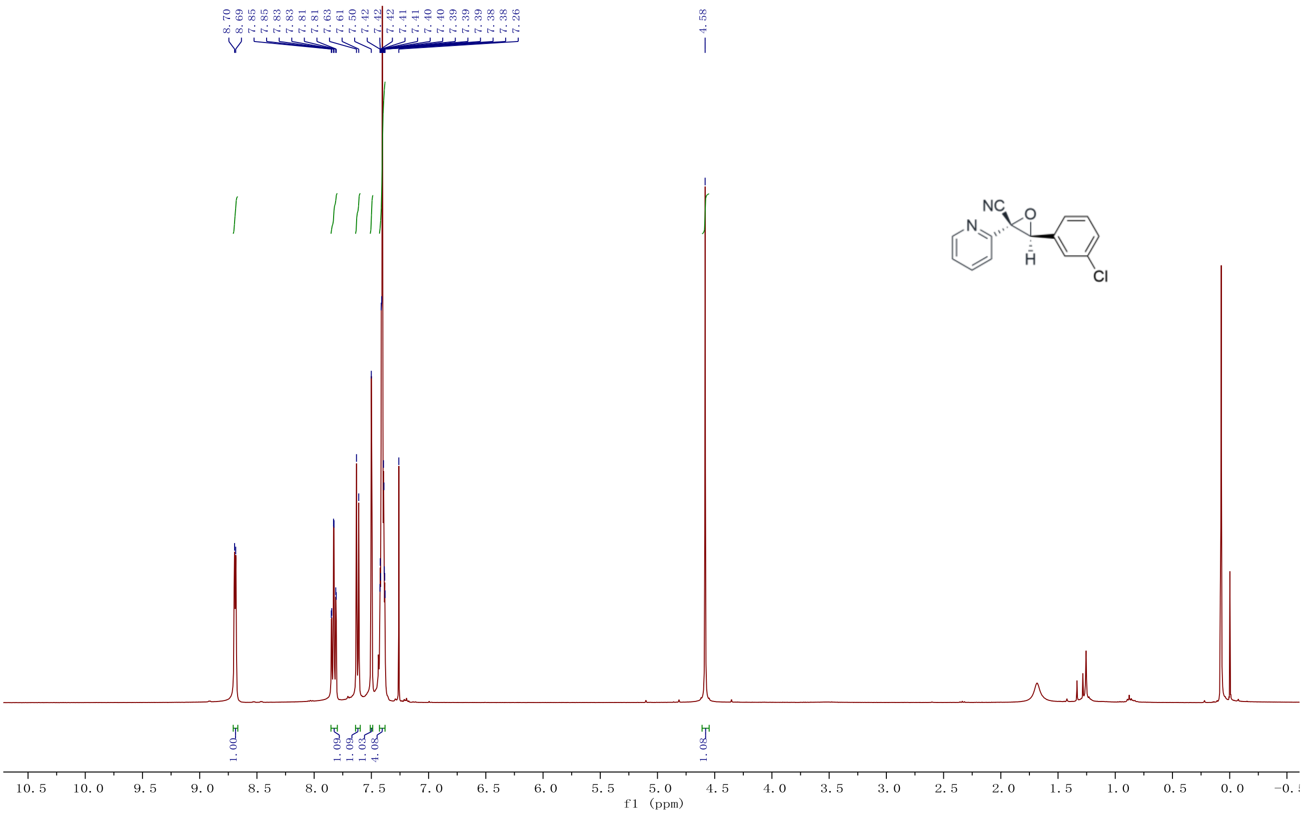

Supplementary Figure 330. ^{13}C NMR spectrum of compound **3bg** (100 MHz, $CDCl_3$)

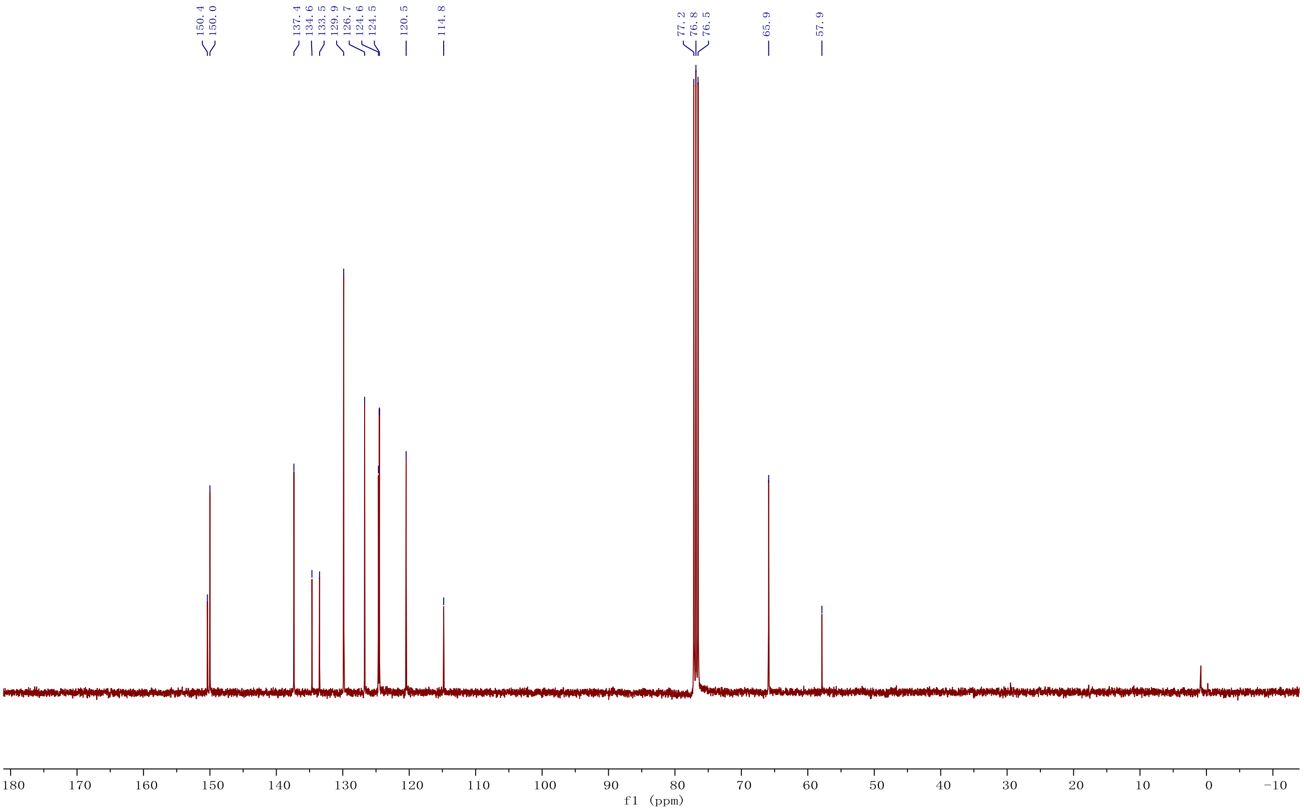

Supplementary Figure 331. ^{1}H NMR spectrum of compound **3bh** (400 MHz, $CDCl_3$)

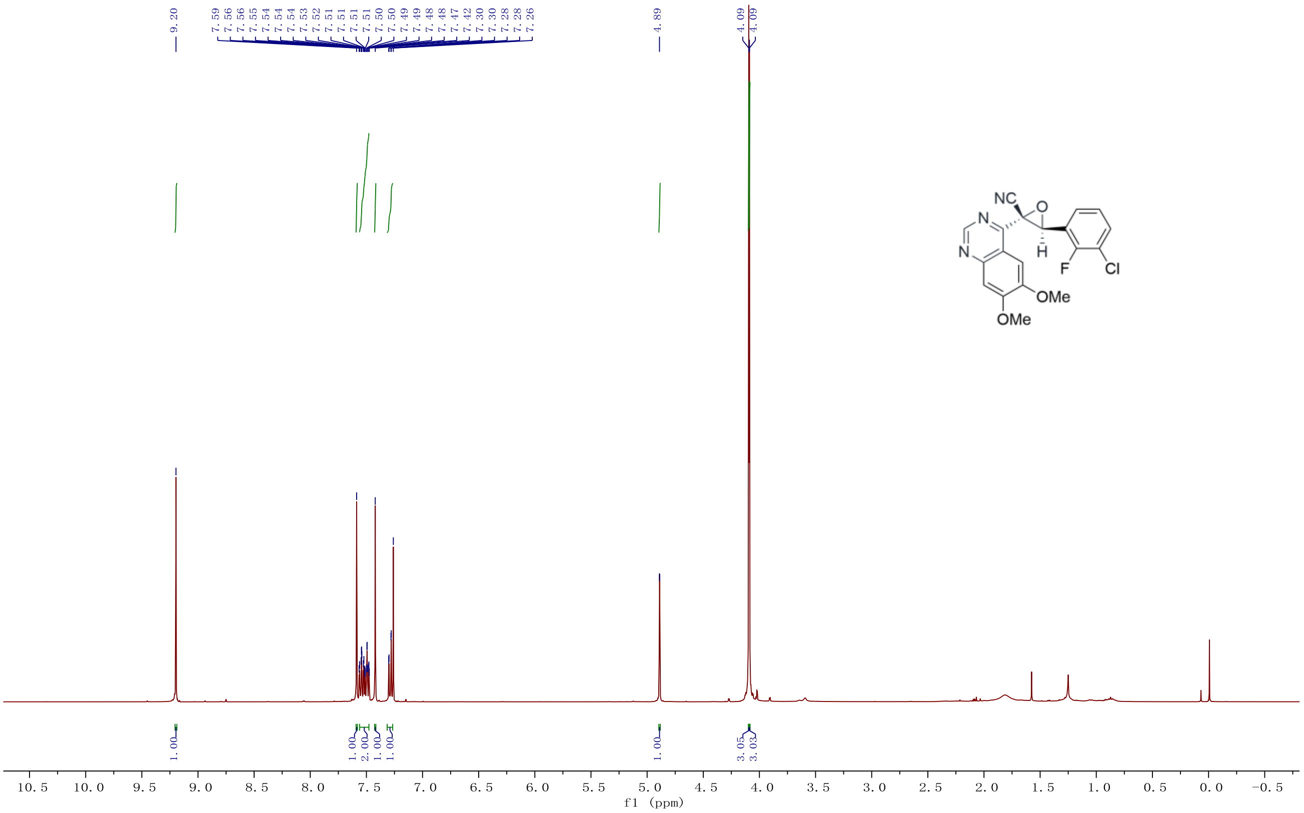

Supplementary Figure 332. ^{13}C NMR spectrum of compound **3bh** (100 MHz, $CDCl_3$)

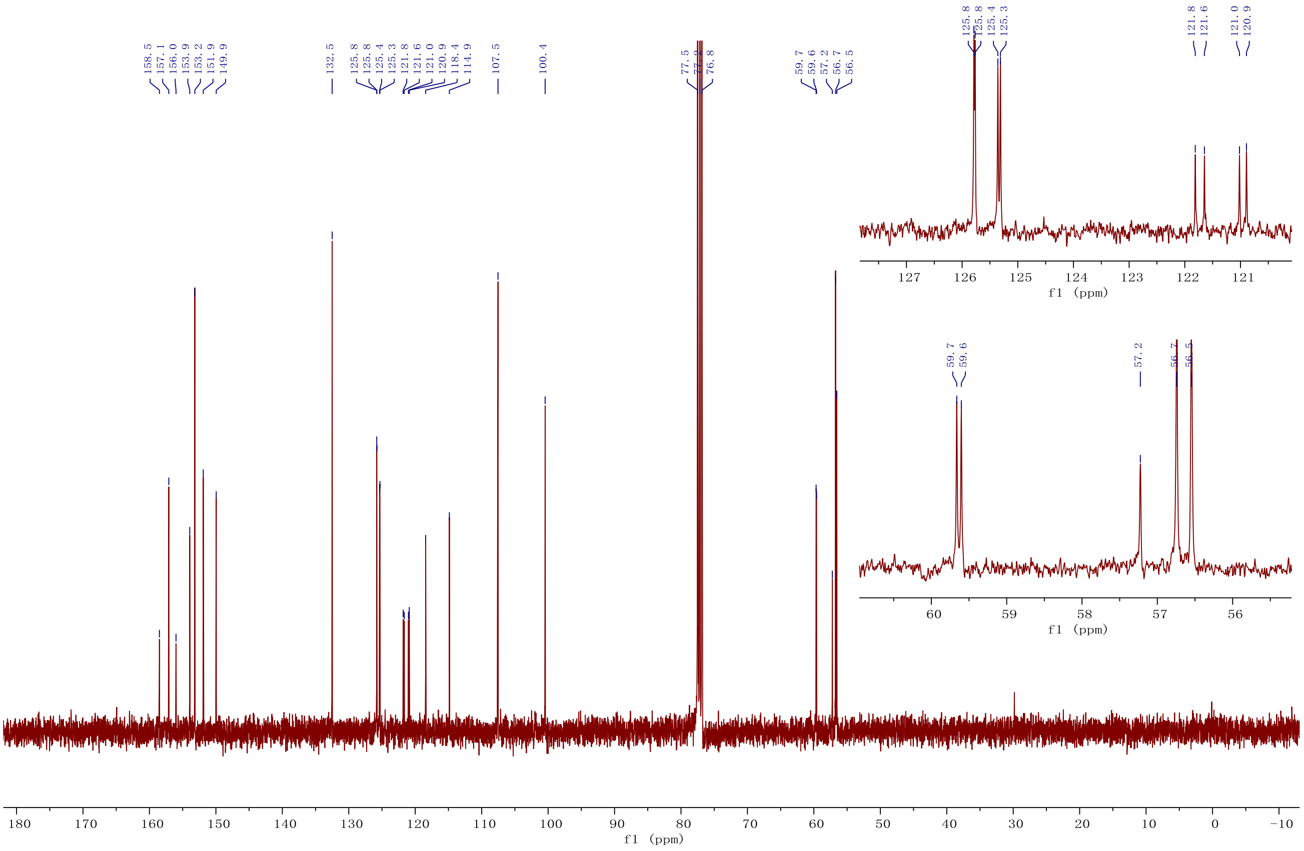

Supplementary Figure 333. ^{19}F NMR spectrum of compound **3bh** (376 MHz, $CDCl_3$)

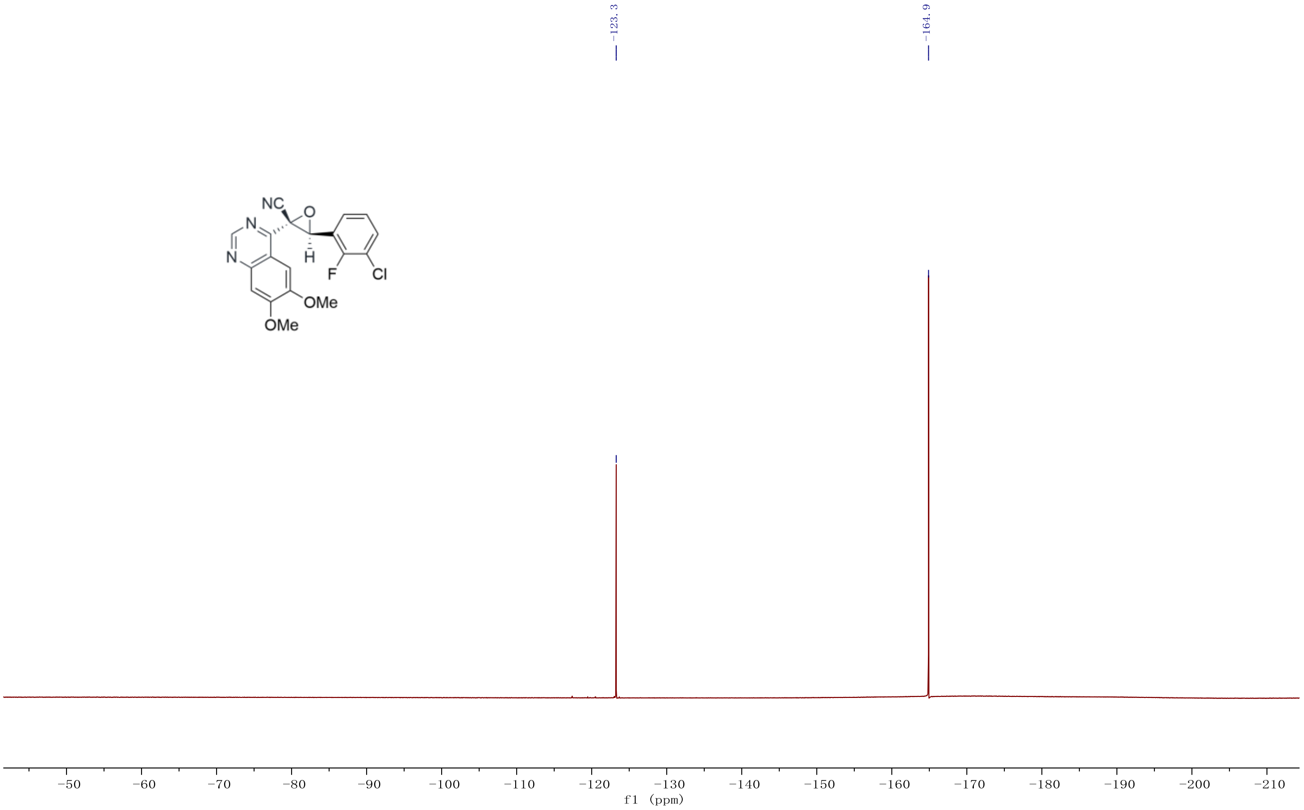

Supplementary Figure 334. ^{1}H NMR spectrum of compound **3bi** (400 MHz, $CDCl_3$)

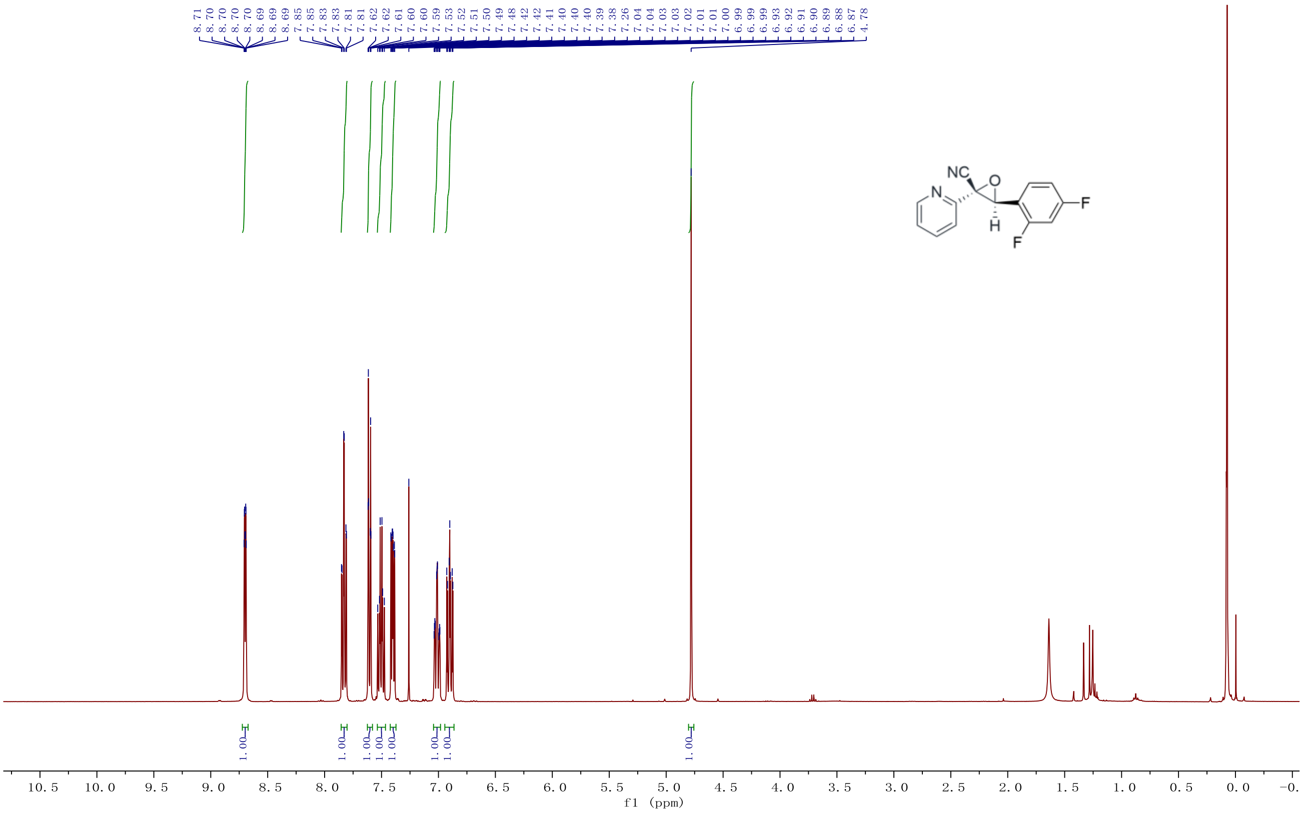

Supplementary Figure 335. ^{13}C NMR spectrum of compound **3bi** (100 MHz, $CDCl_3$)

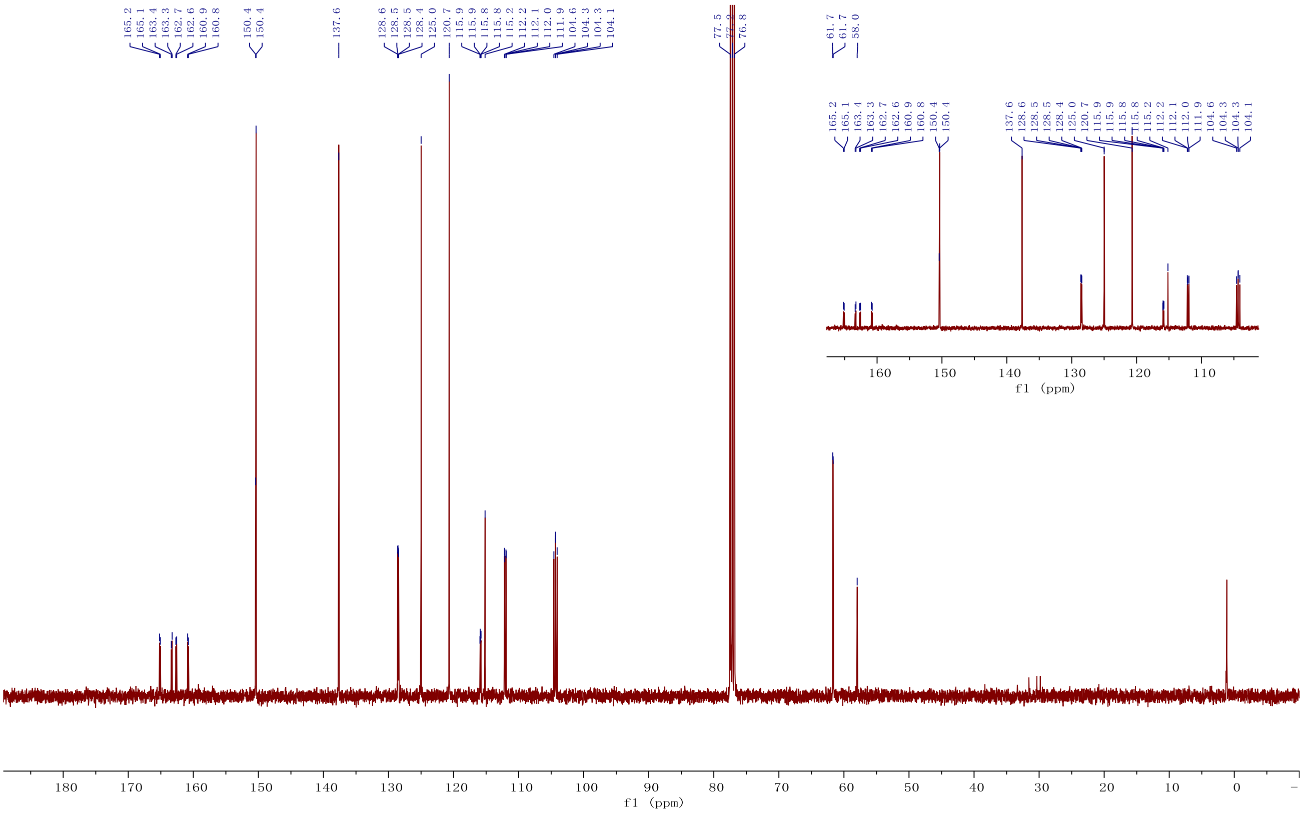

Supplementary Figure 336. ^{19}F NMR spectrum of compound **3bi** (376 MHz, $CDCl_3$)

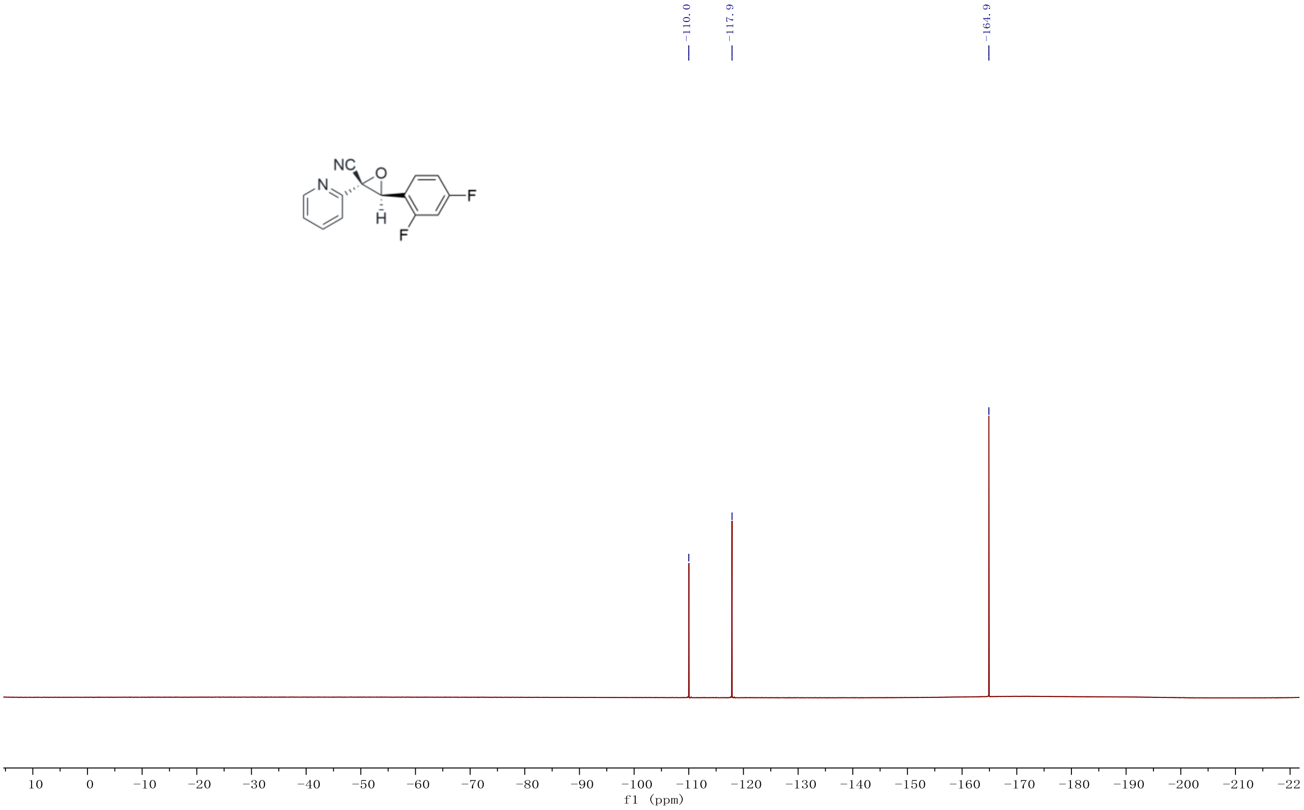

Supplementary Figure 337. ^{1}H NMR spectrum of compound **3bj** (400 MHz, $CDCl_3$)

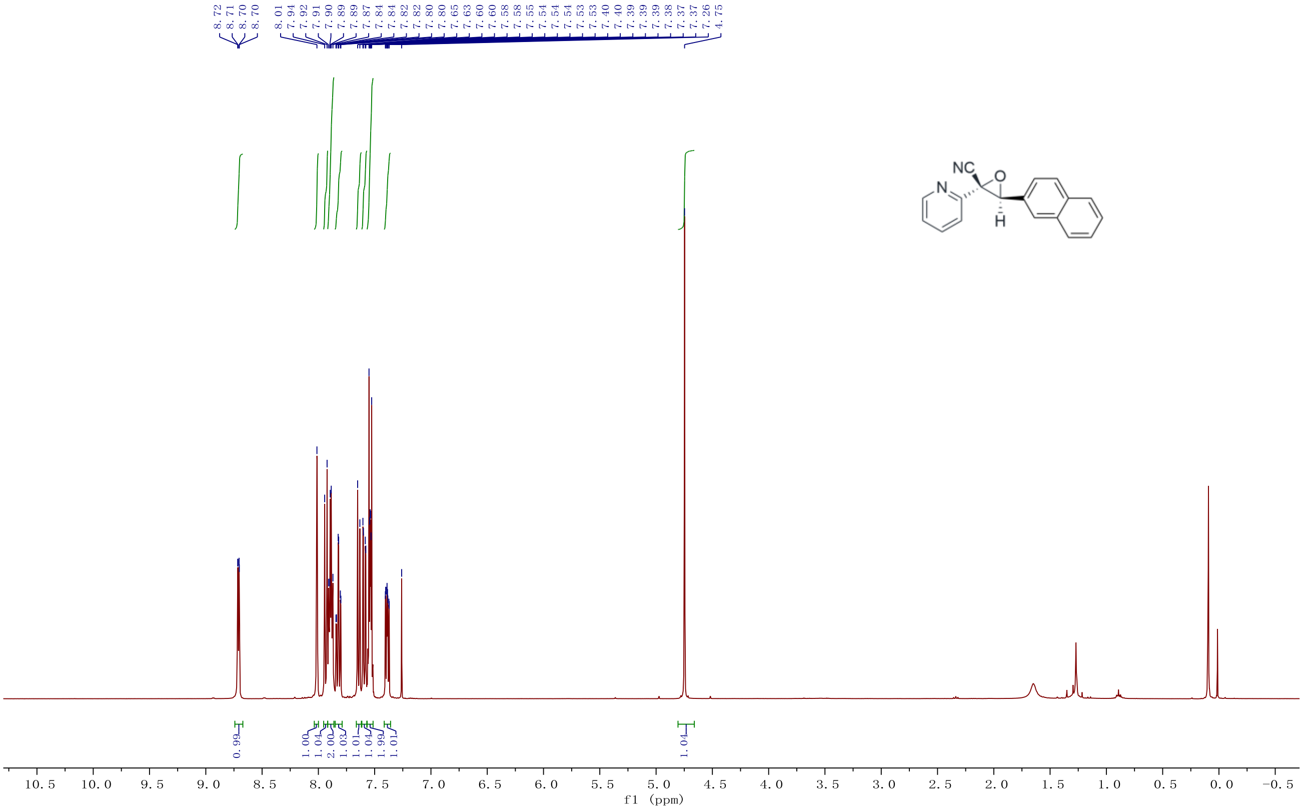

Supplementary Figure 338. ^{13}C NMR spectrum of compound **3bj** (100 MHz, $CDCl_3$)

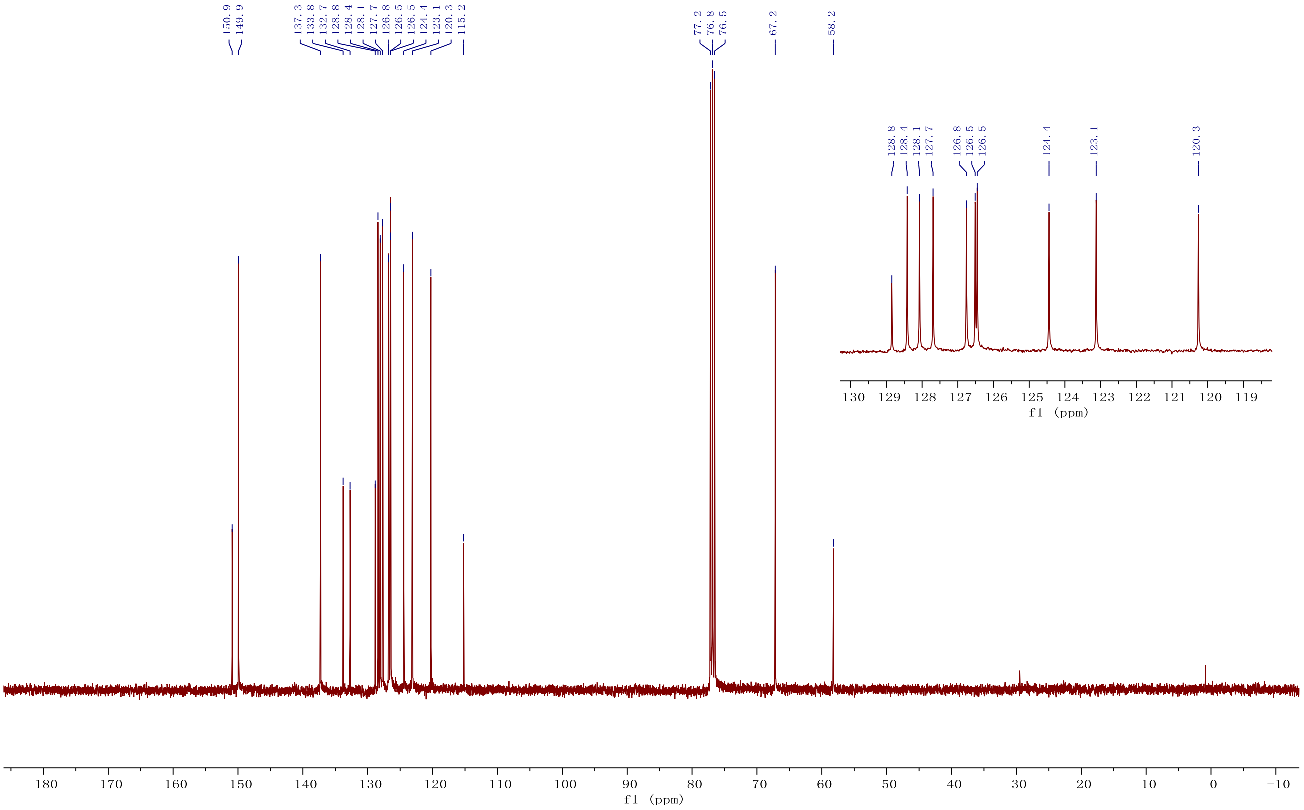

Supplementary Figure 339. ^{1}H NMR spectrum of compound **3bk** (400 MHz, $CDCl_3$)

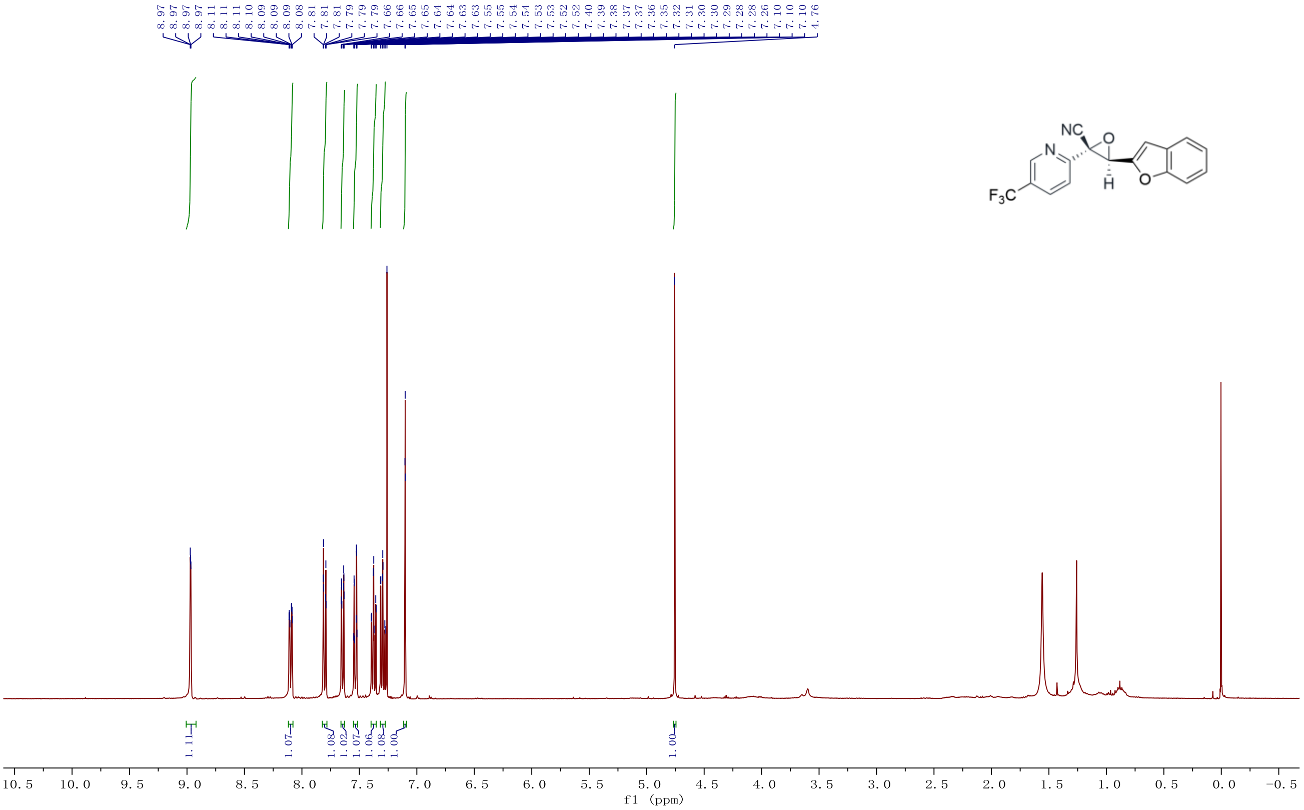

Supplementary Figure 340. ^{13}C NMR spectrum of compound **3bk** (100 MHz, $CDCl_3$)

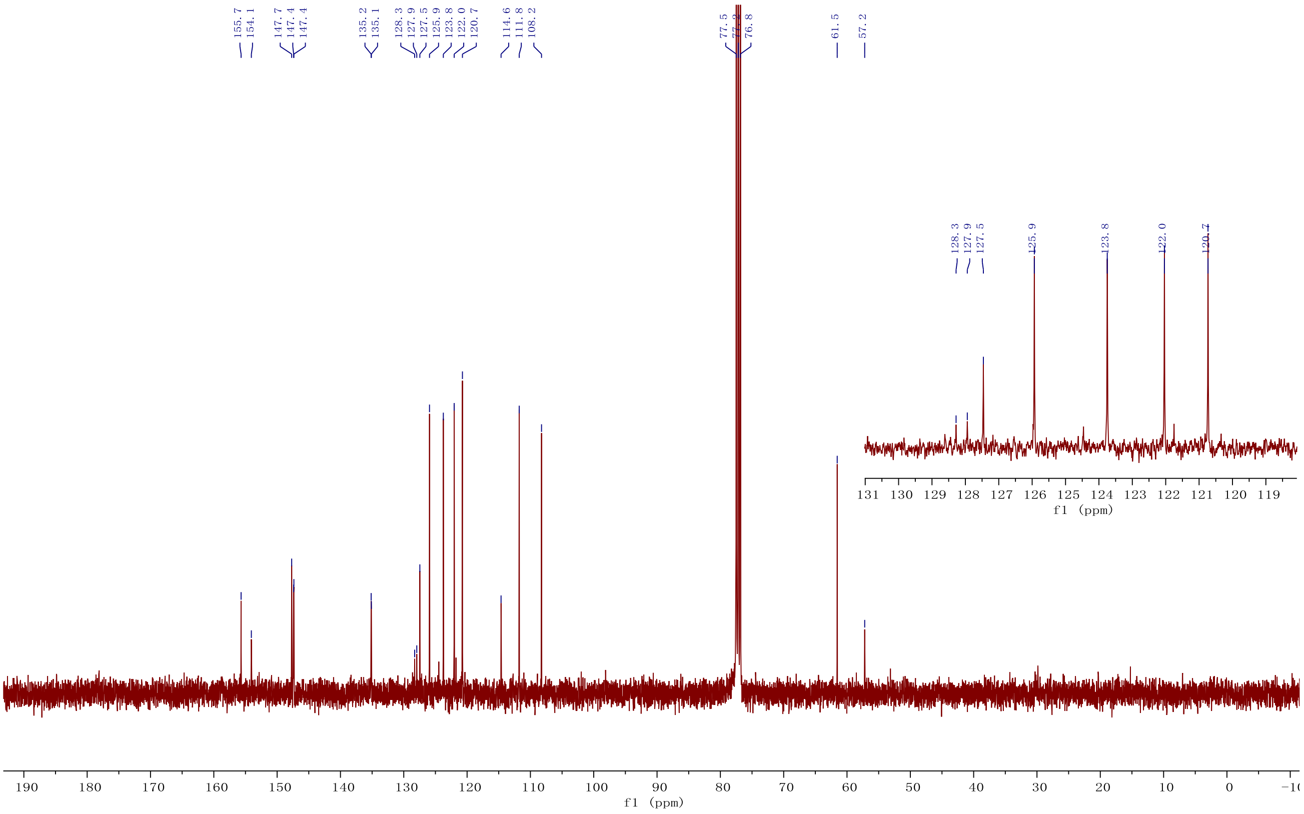

Supplementary Figure 341. ^{19}F NMR spectrum of compound **3bk** (376 MHz, $CDCl_3$)

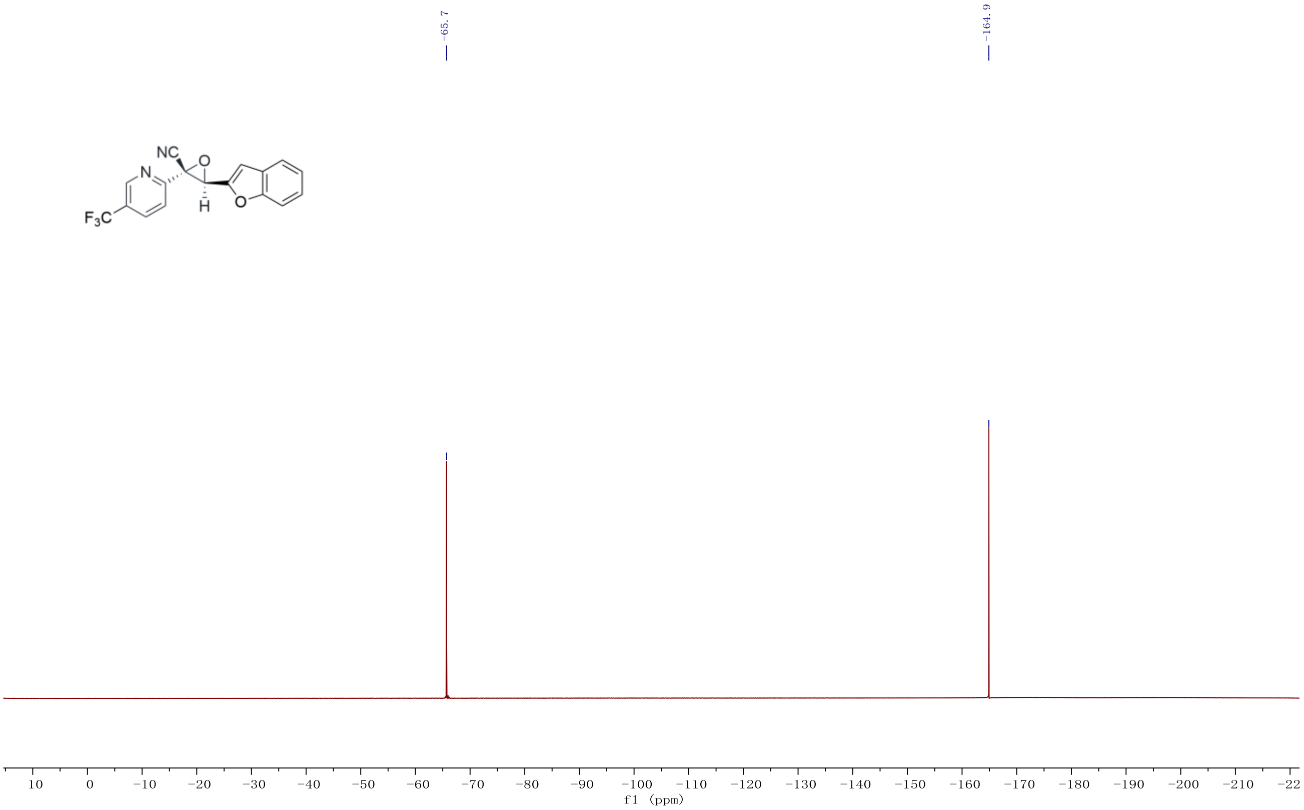

Supplementary Figure 342. ^{1}H NMR spectrum of compound **3bl** (400 MHz, $CDCl_3$)

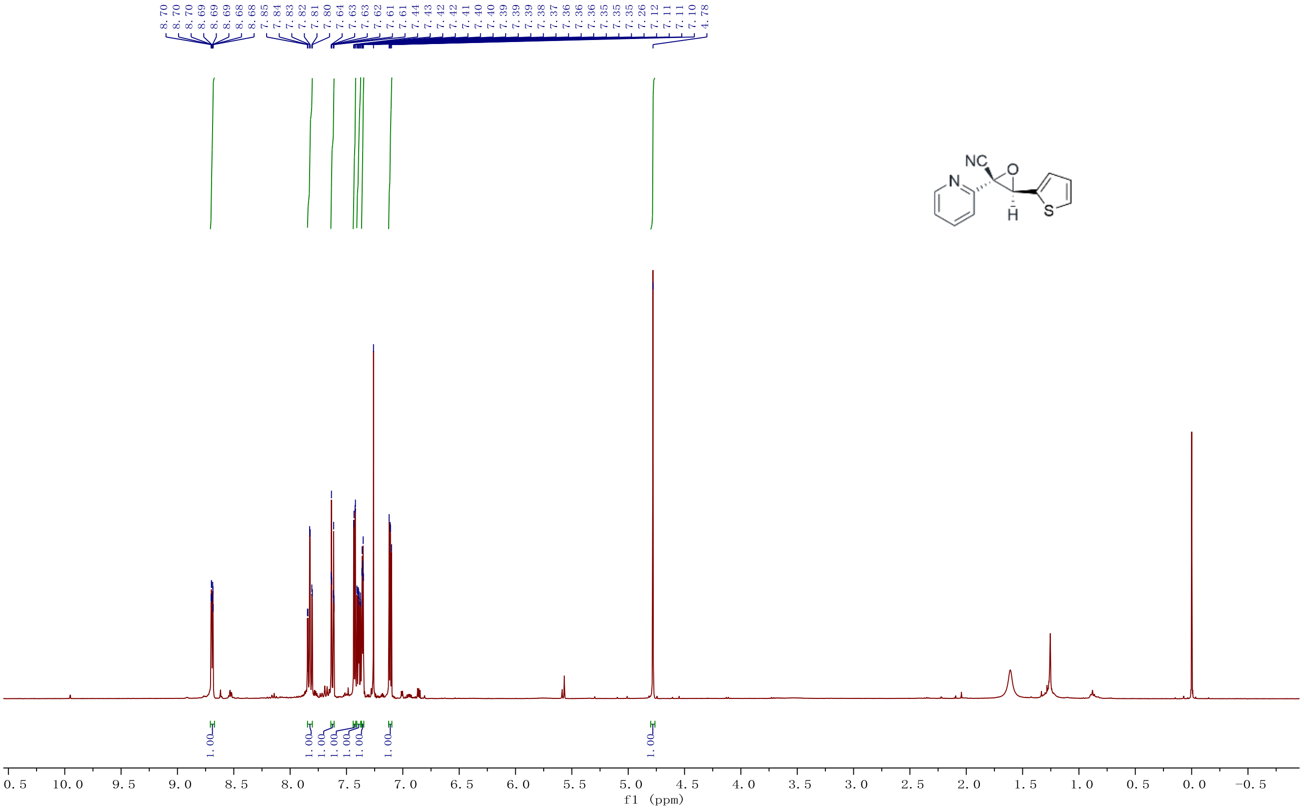

Supplementary Figure 343. ^{13}C NMR spectrum of compound **3bl** (100 MHz, $CDCl_3$)

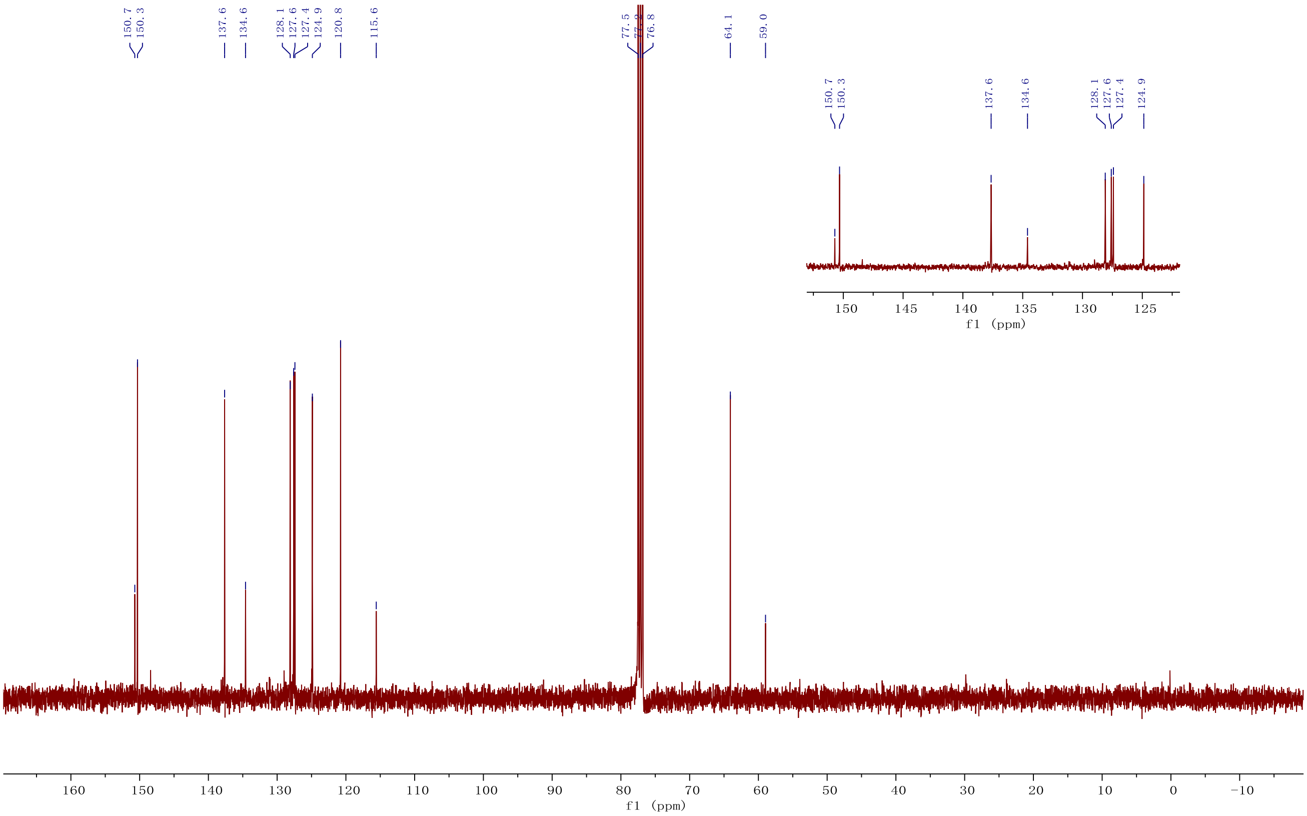

Supplementary Figure 344. ^{1}H NMR spectrum of compound **3ca** (400 MHz, $CDCl_3$)

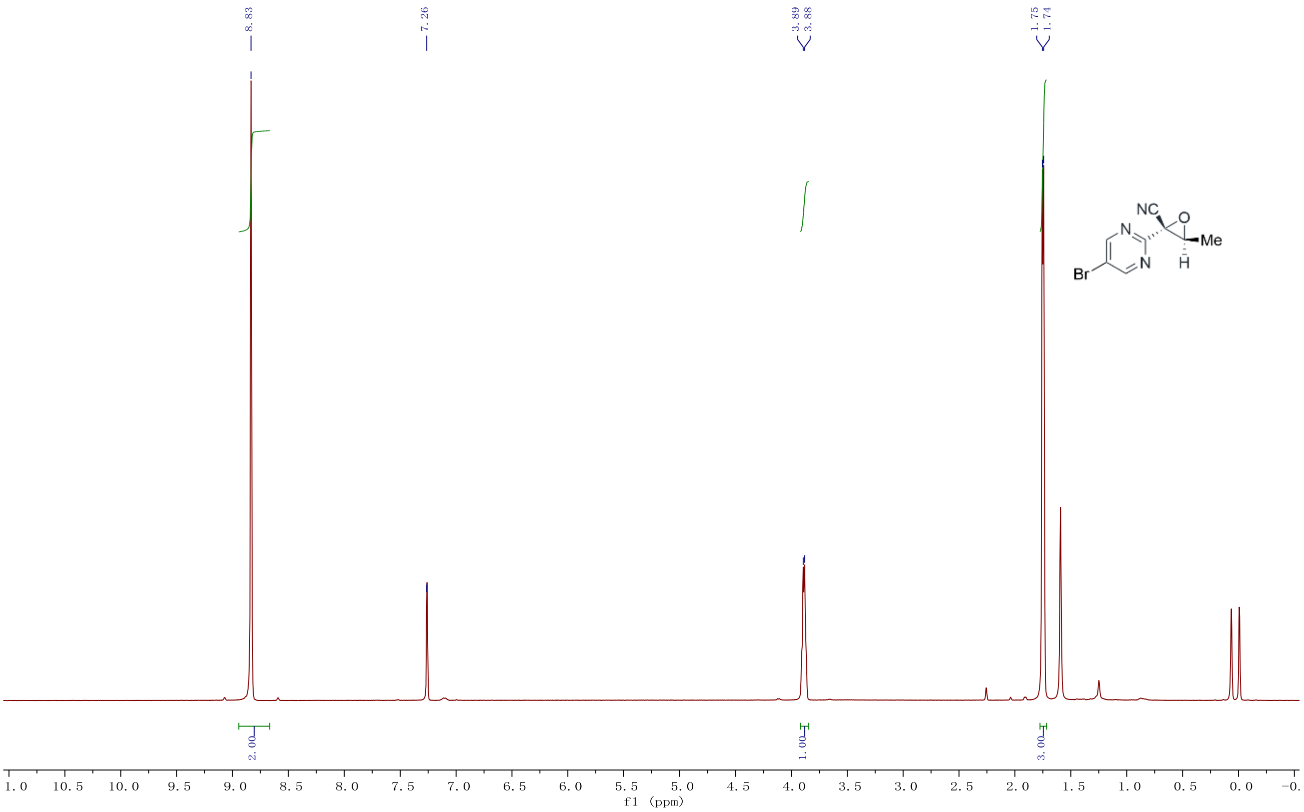

Supplementary Figure 345. ^{13}C NMR spectrum of compound **3ca** (100 MHz, $CDCl_3$)

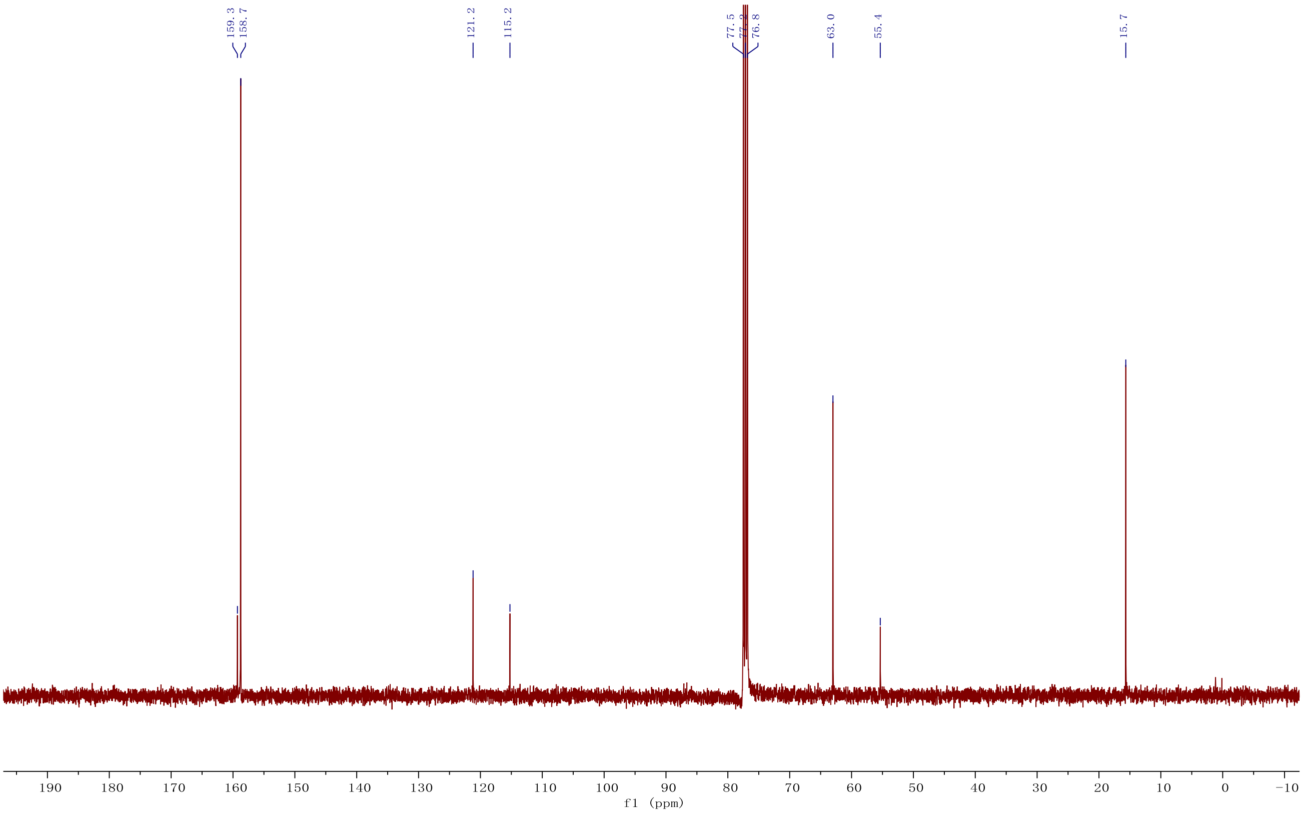

Supplementary Figure 346. ^{1}H NMR spectrum of compound **3cb** (400 MHz, $CDCl_3$)

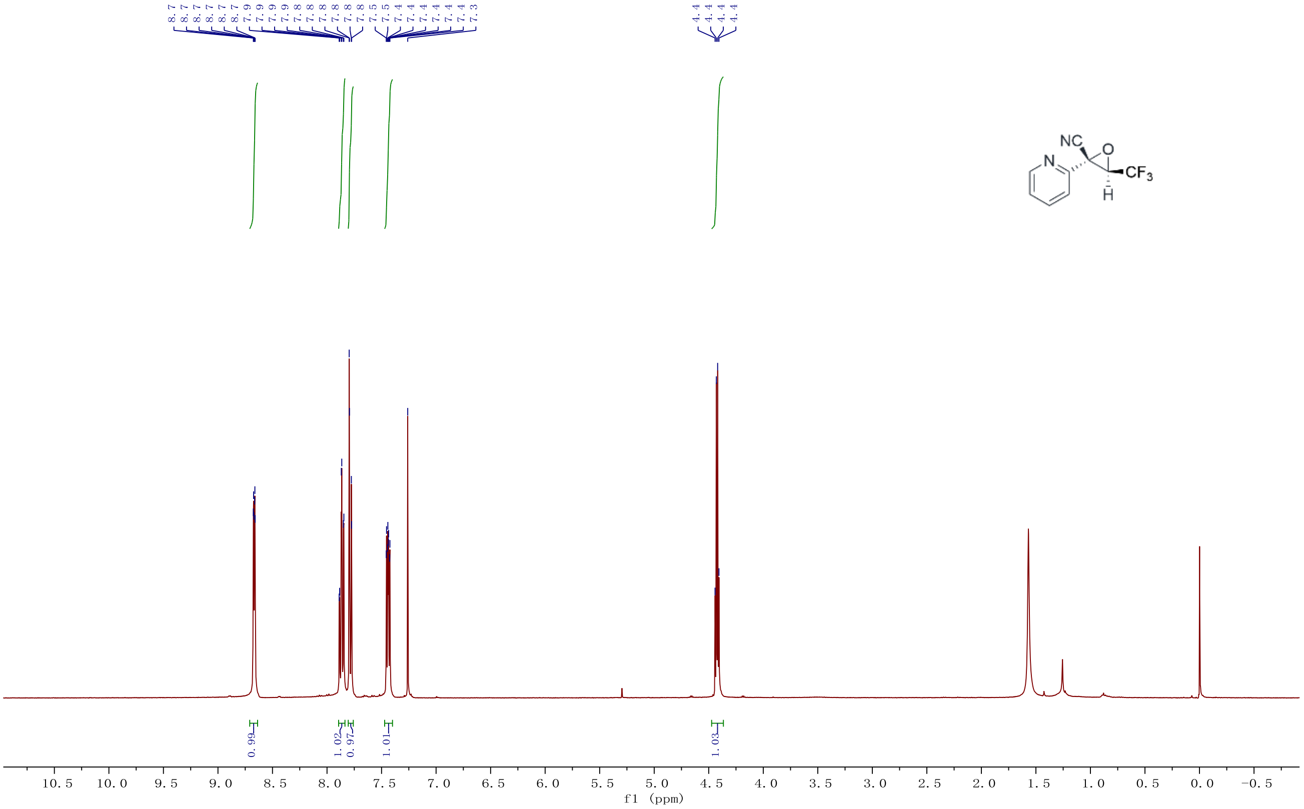

Supplementary Figure 347. ^{13}C NMR spectrum of compound **3cb** (100 MHz, $CDCl_3$)

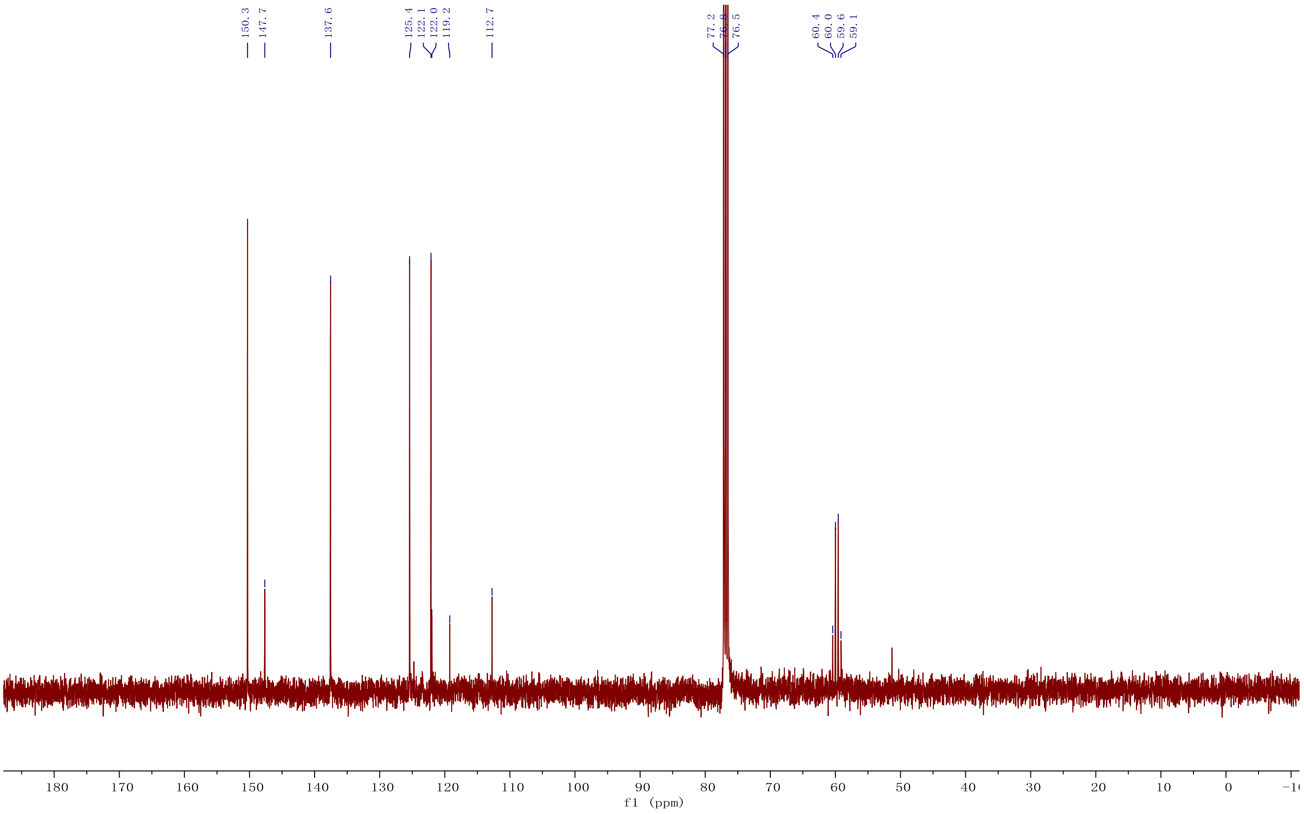

Supplementary Figure 348. ^{19}F NMR spectrum of compound **3cb** (376 MHz, $CDCl_3$)

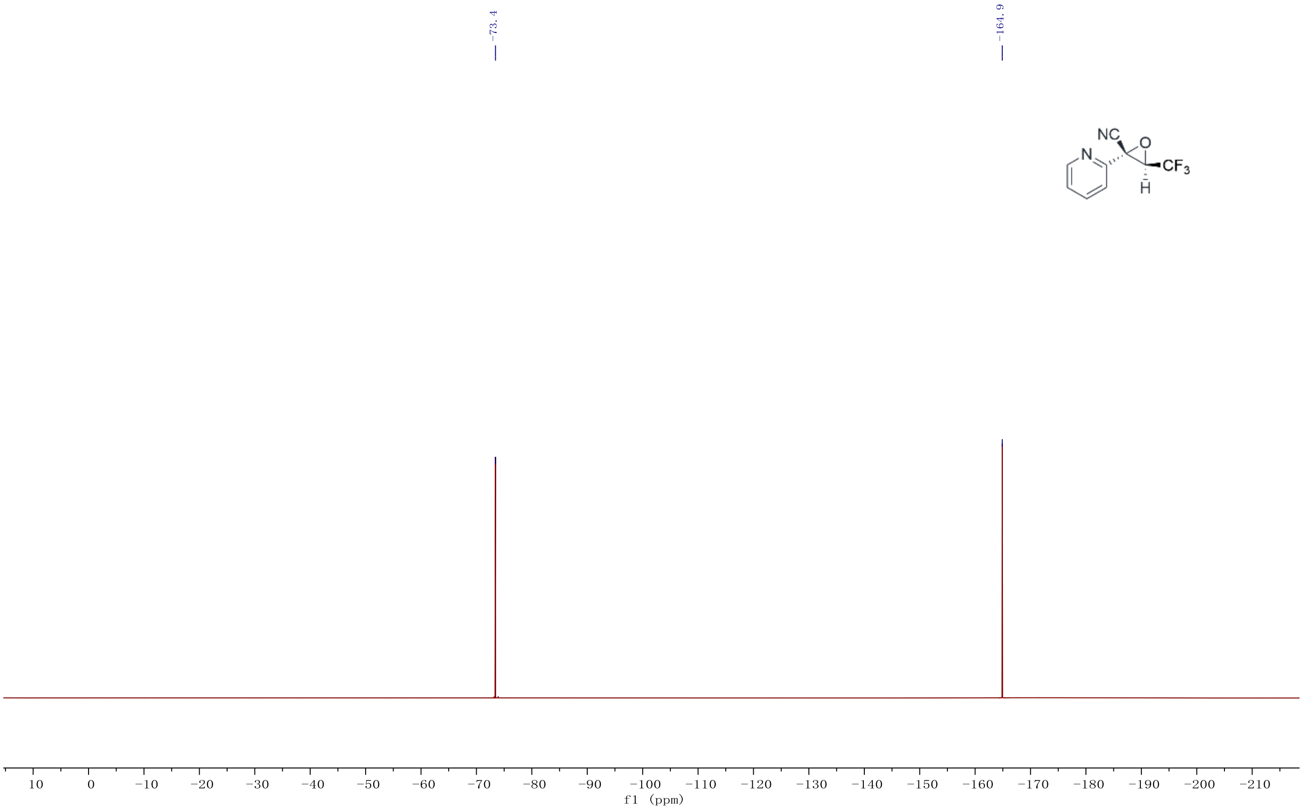

Supplementary Figure 349. ^{1}H NMR spectrum of compound **3cc** (400 MHz, $CDCl_3$)

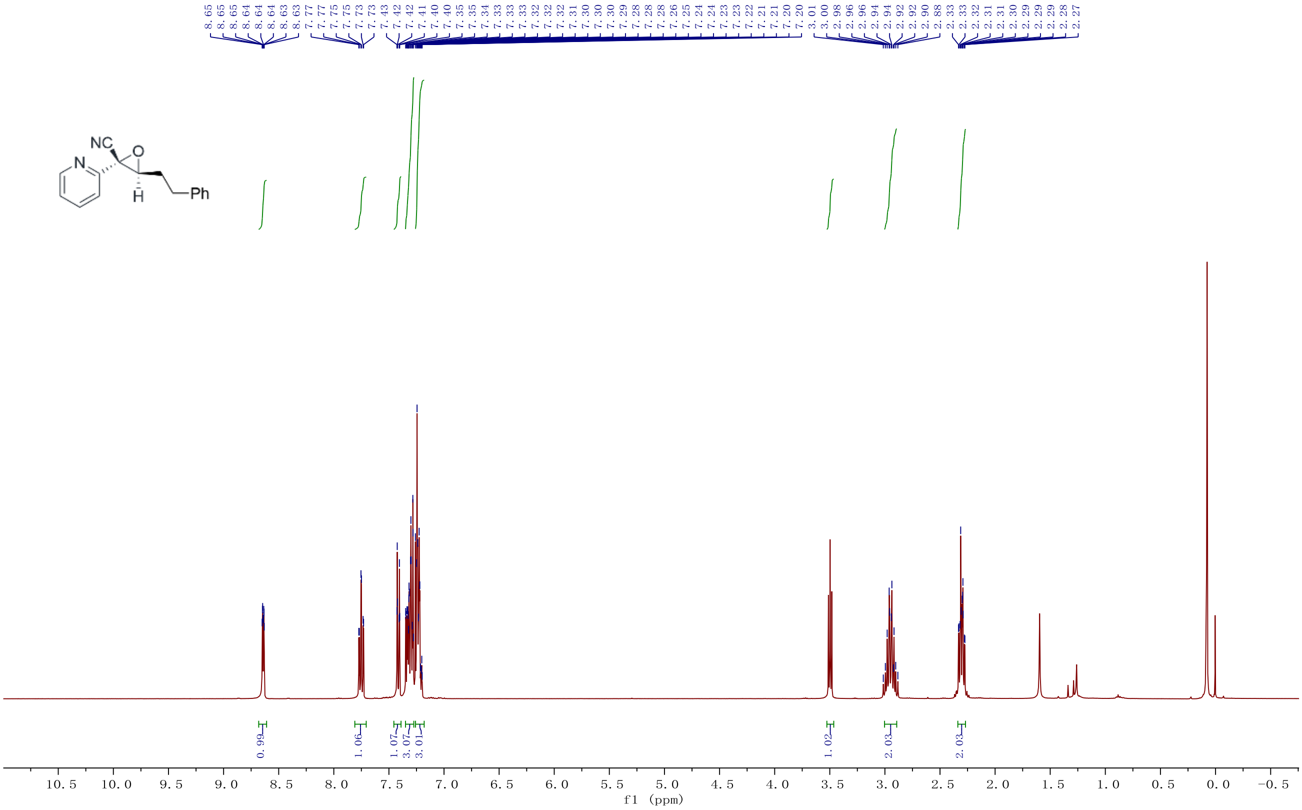

Supplementary Figure 350. ^{13}C NMR spectrum of compound **3cc** (100 MHz, $CDCl_3$)

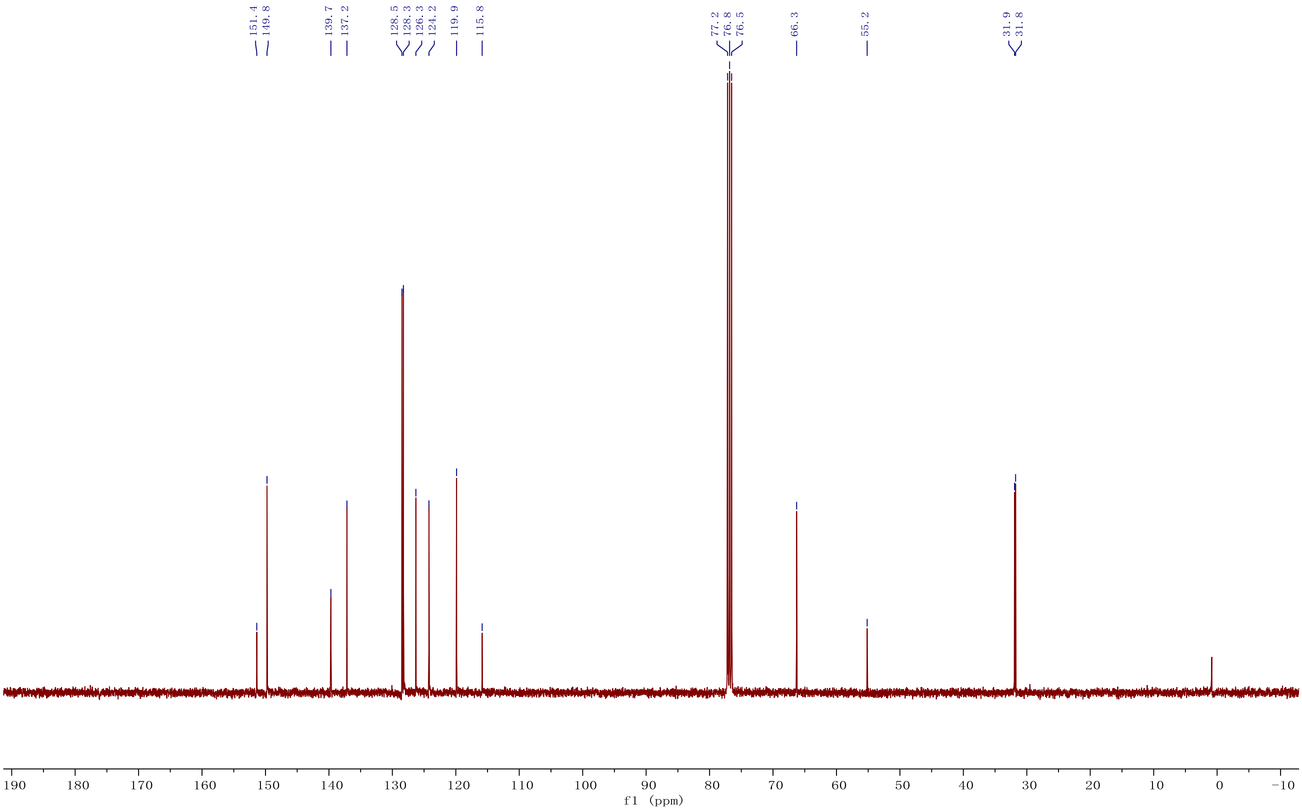

Supplementary Figure 351. ^{1}H NMR spectrum of compound **3cd** (400 MHz, $CDCl_3$)

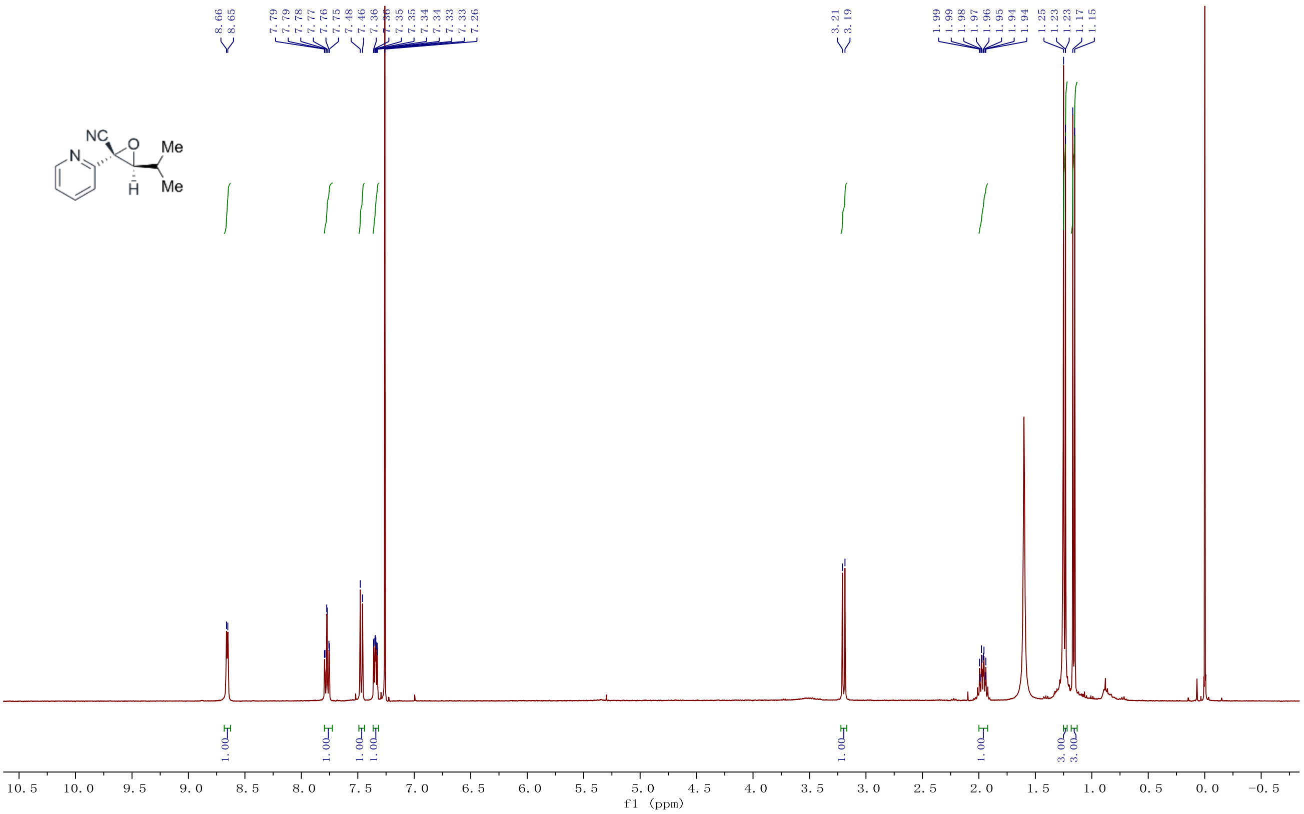

Supplementary Figure 352. ^{13}C NMR spectrum of compound **3cd** (100 MHz, $CDCl_3$)

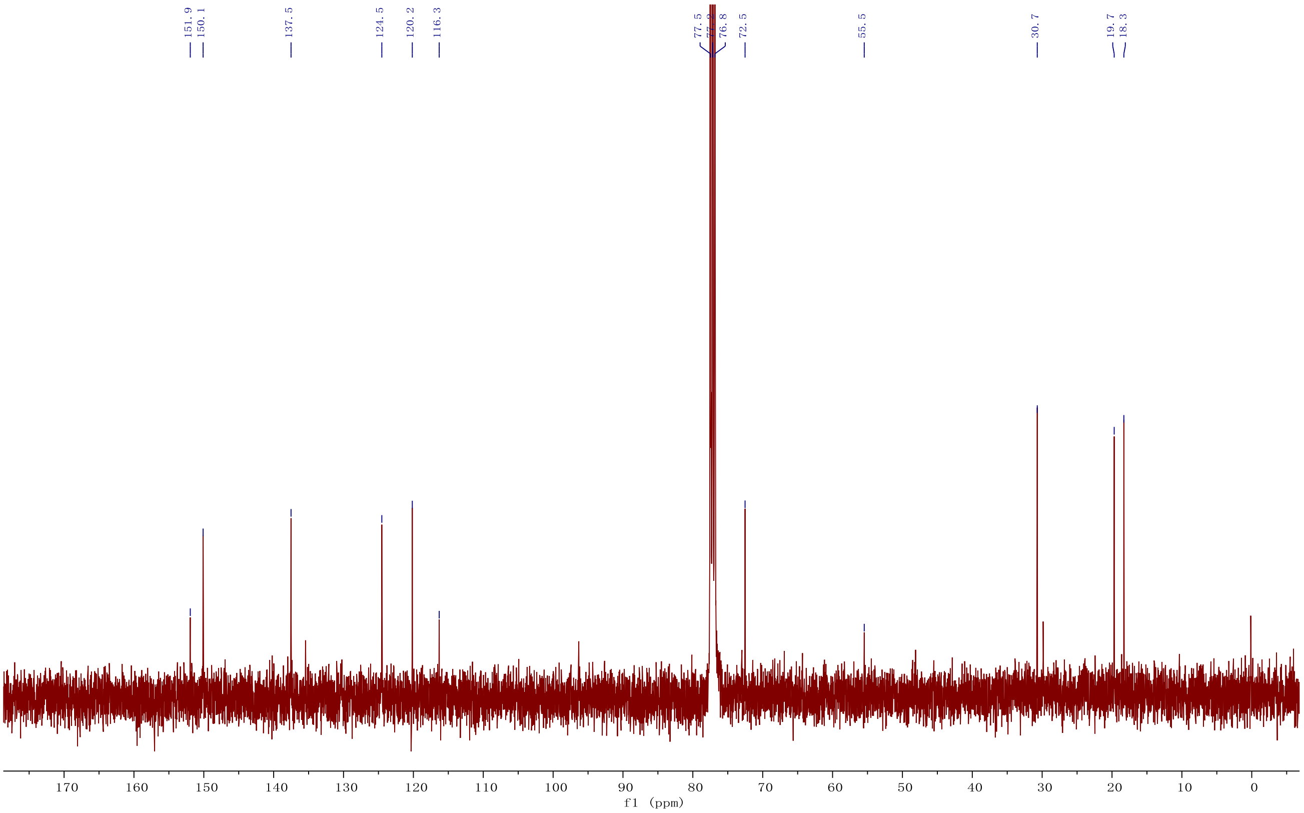

Supplementary Figure 353. ^{1}H NMR spectrum of compound **3ce** (400 MHz, $CDCl_3$)

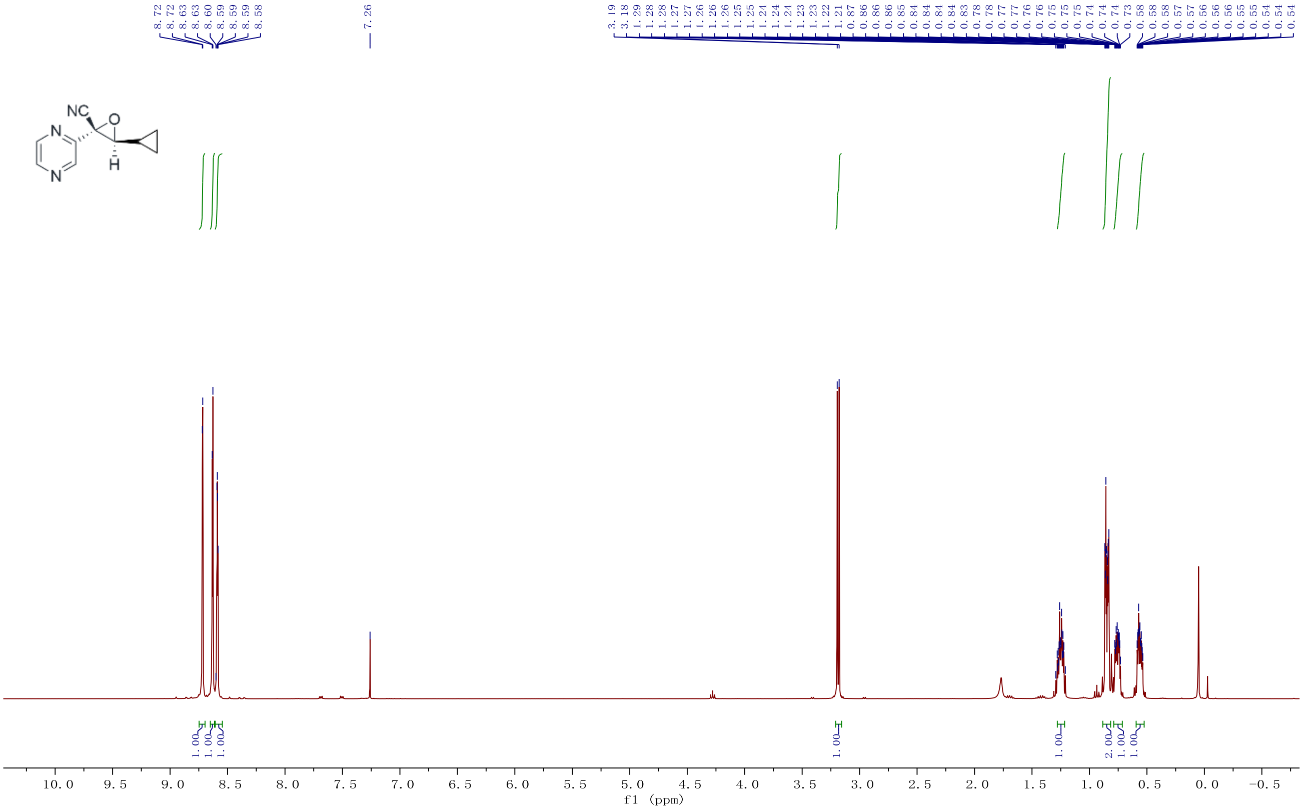

Supplementary Figure 354. ^{13}C NMR spectrum of compound **3ce** (100 MHz, $CDCl_3$)

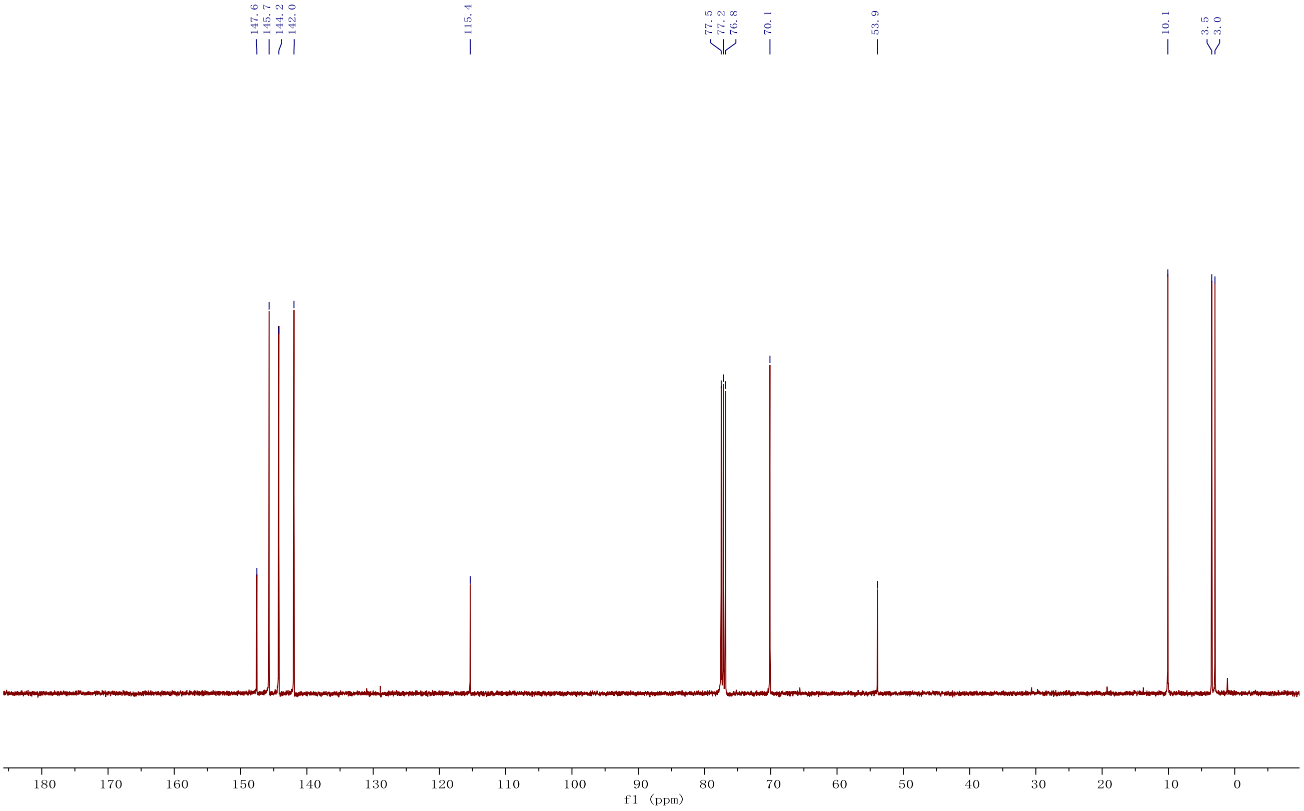

Supplementary Figure 355. ^{1}H NMR spectrum of compound **3cf** (600 MHz, $CDCl_3$)

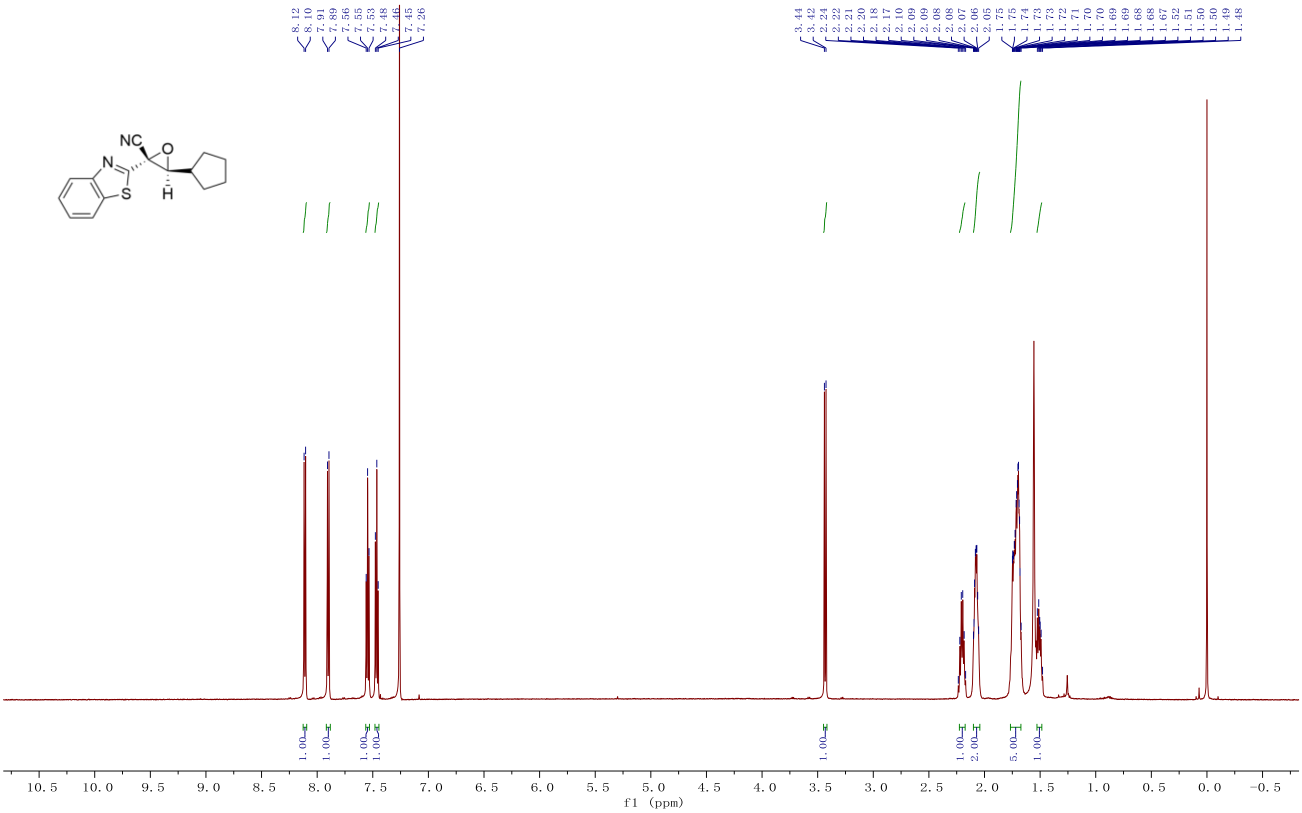

Supplementary Figure 356. ^{13}C NMR spectrum of compound **3cf** (150 MHz, $CDCl_3$)

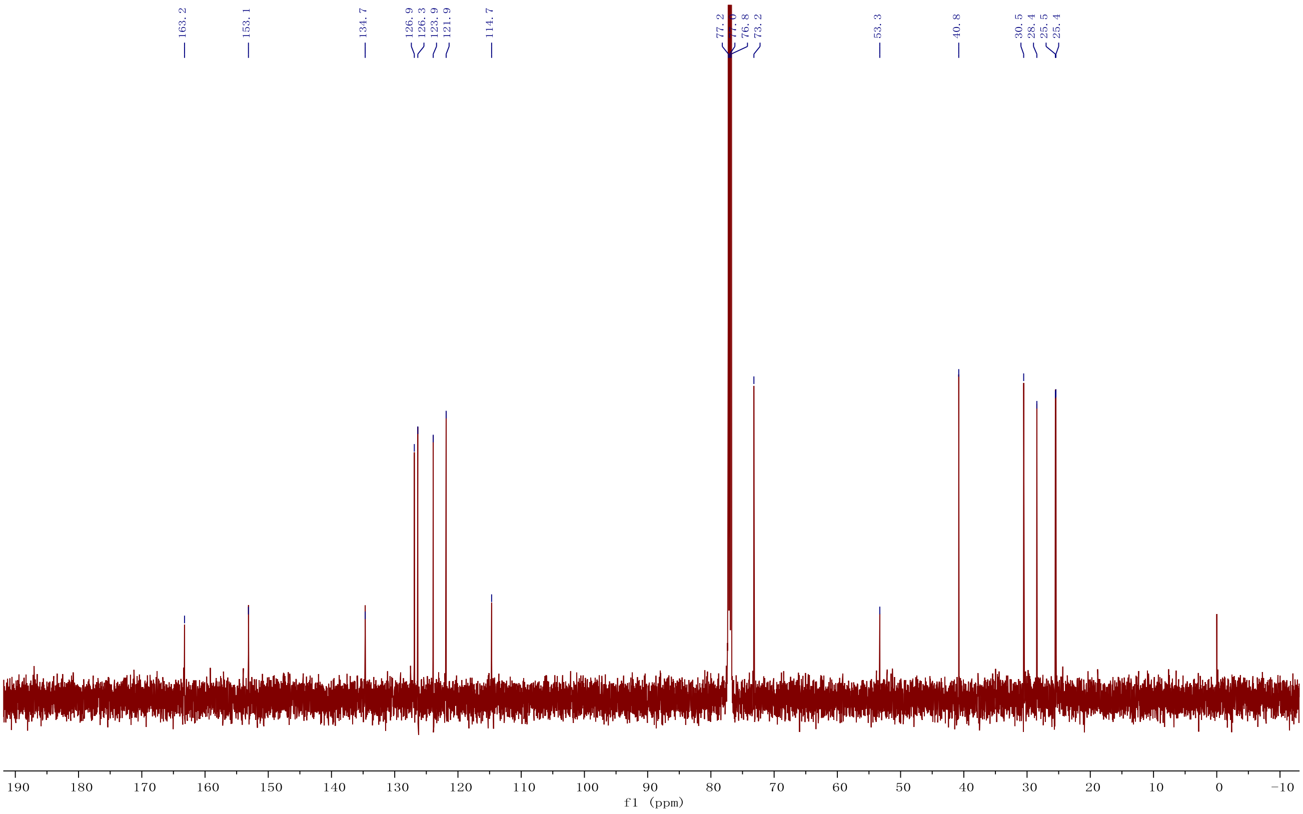

Supplementary Figure 357. ^{1}H NMR spectrum of compound **3cg** (400 MHz, $CDCl_3$)

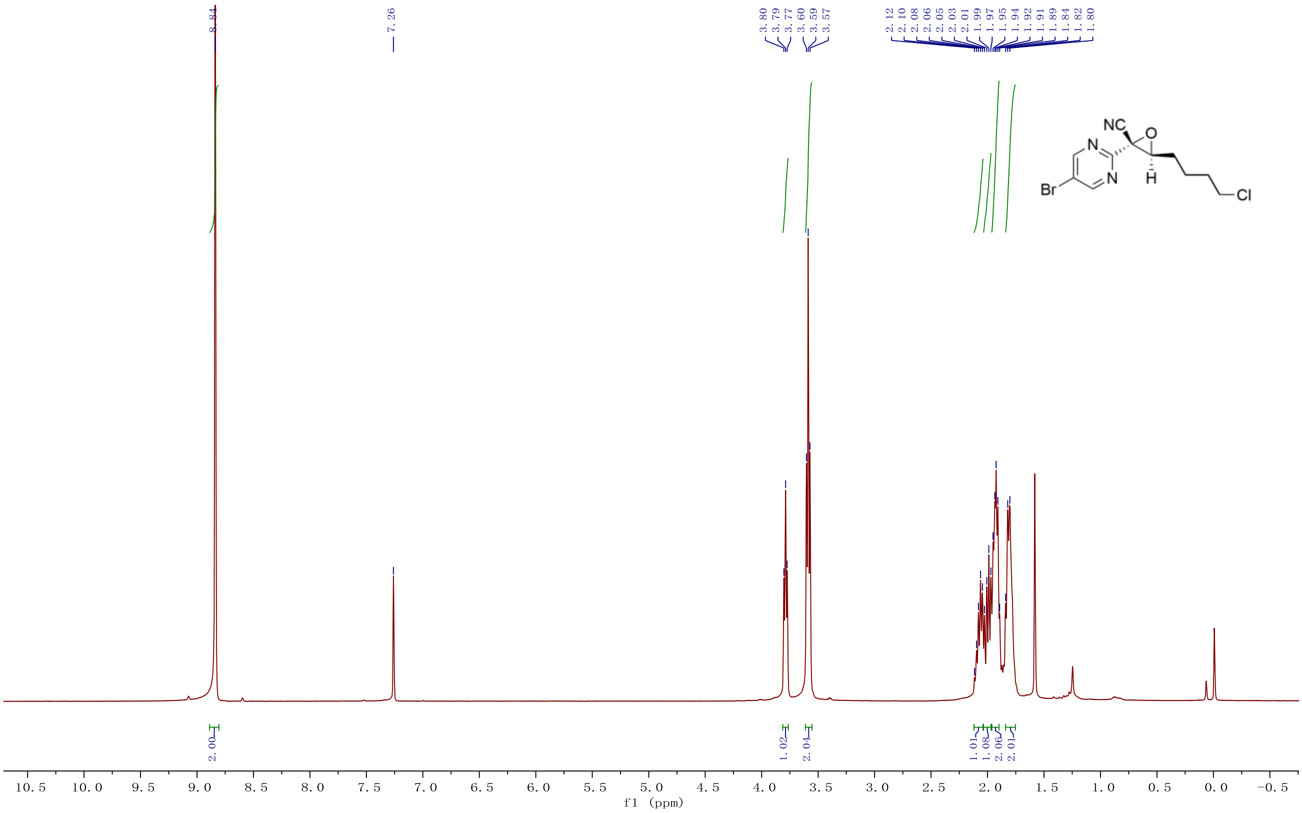

Supplementary Figure 358. ^{13}C NMR spectrum of compound **3cg** (100 MHz, $CDCl_3$)

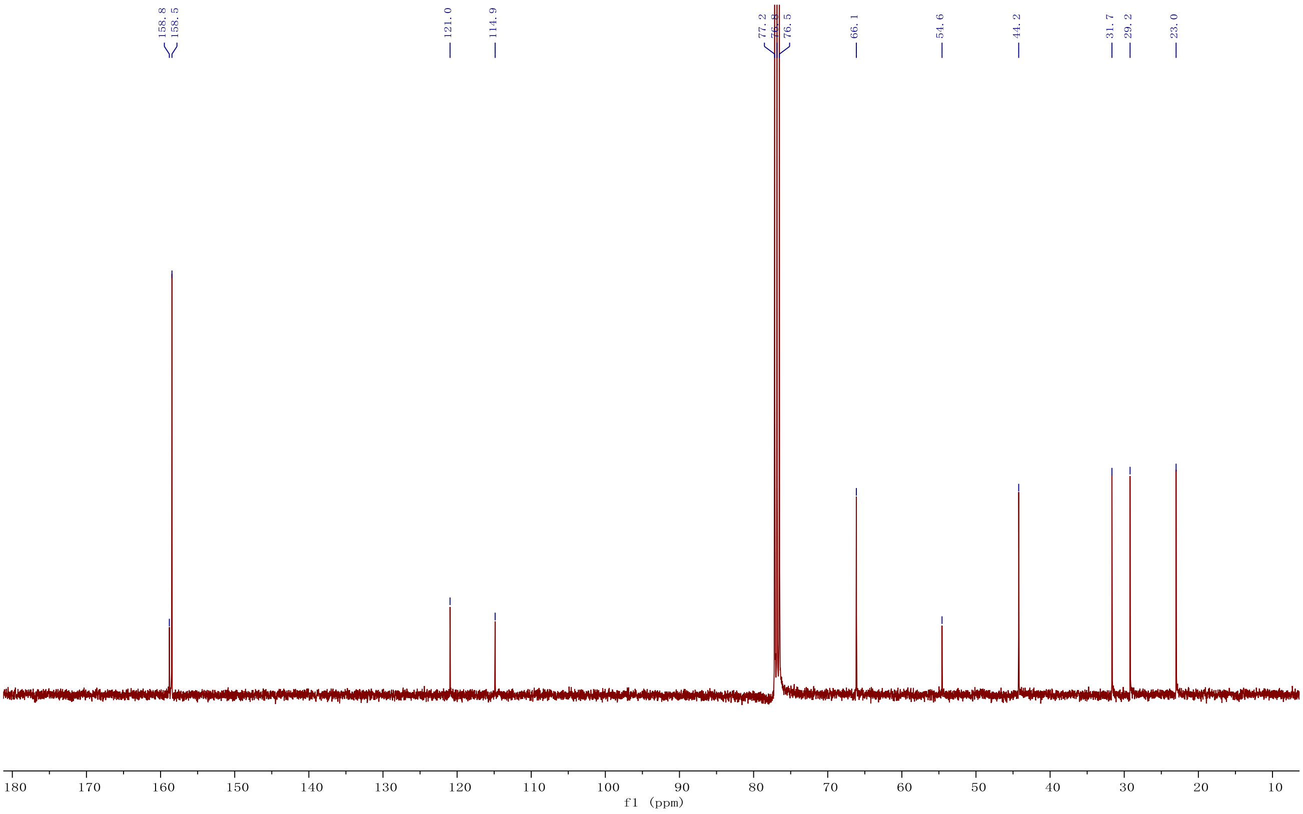

Supplementary Figure 359. ^{1}H NMR spectrum of compound **3ch** (400 MHz, $CDCl_3$)

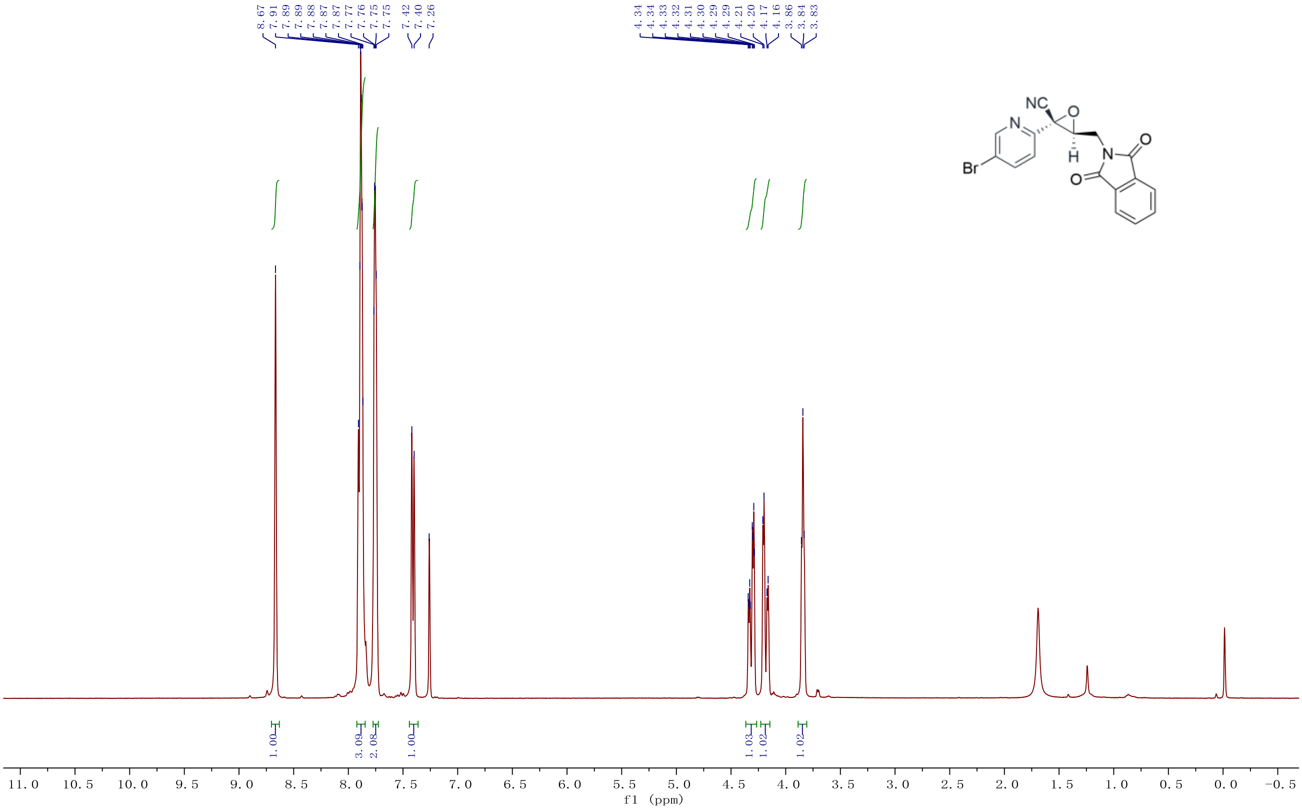

Supplementary Figure 360. ^{13}C NMR spectrum of compound **3ch** (100 MHz, $CDCl_3$)

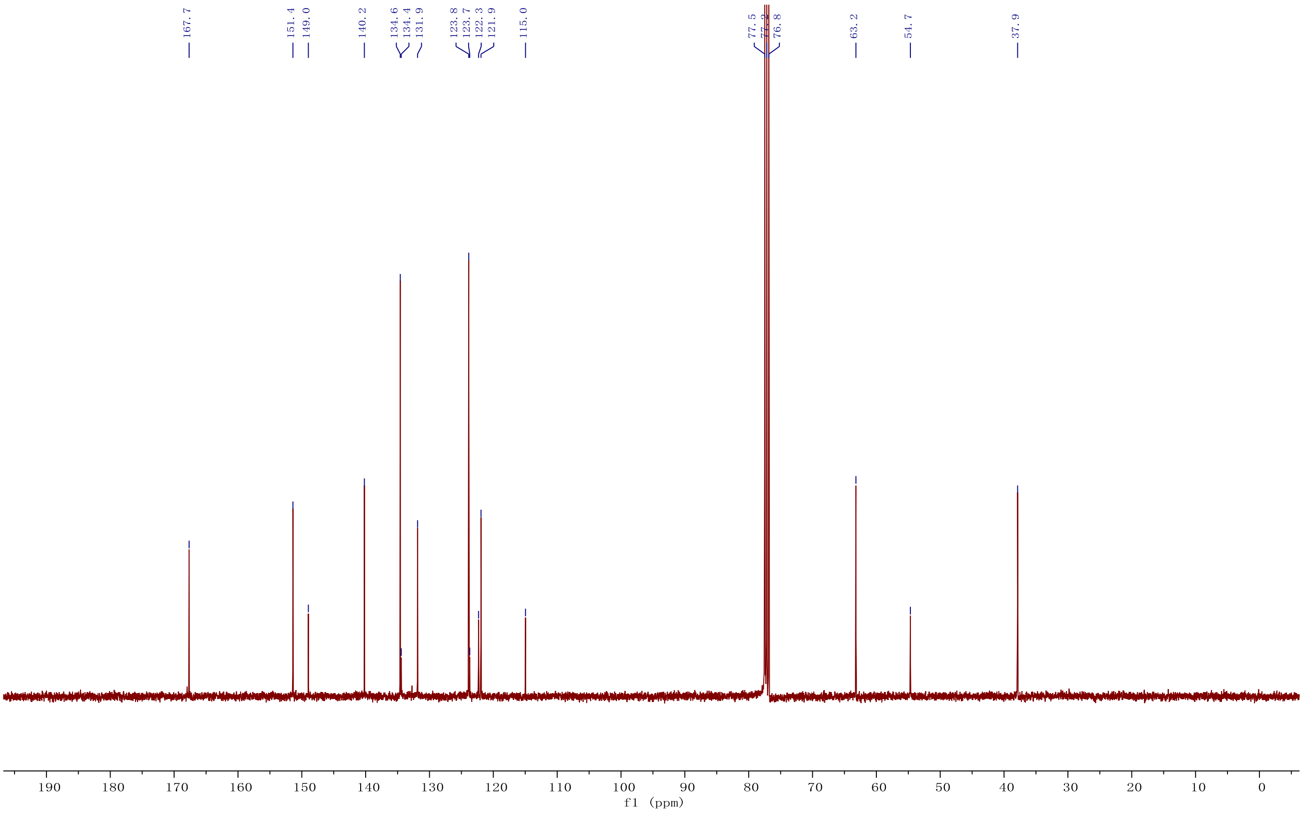

Supplementary Figure 361. ^{1}H NMR spectrum of compound **3ci** (400 MHz, $CDCl_3$)

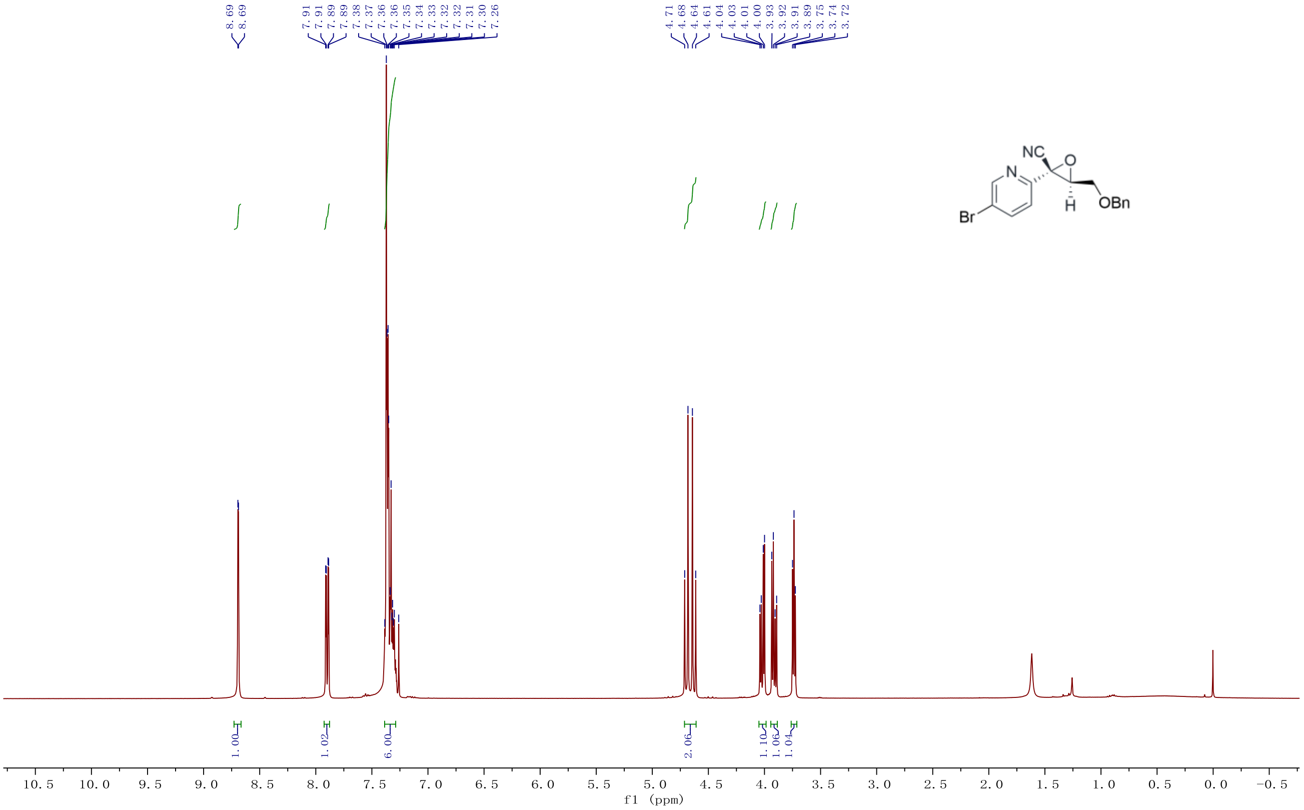

Supplementary Figure 362. ^{13}C NMR spectrum of compound **3ci** (100 MHz, $CDCl_3$)

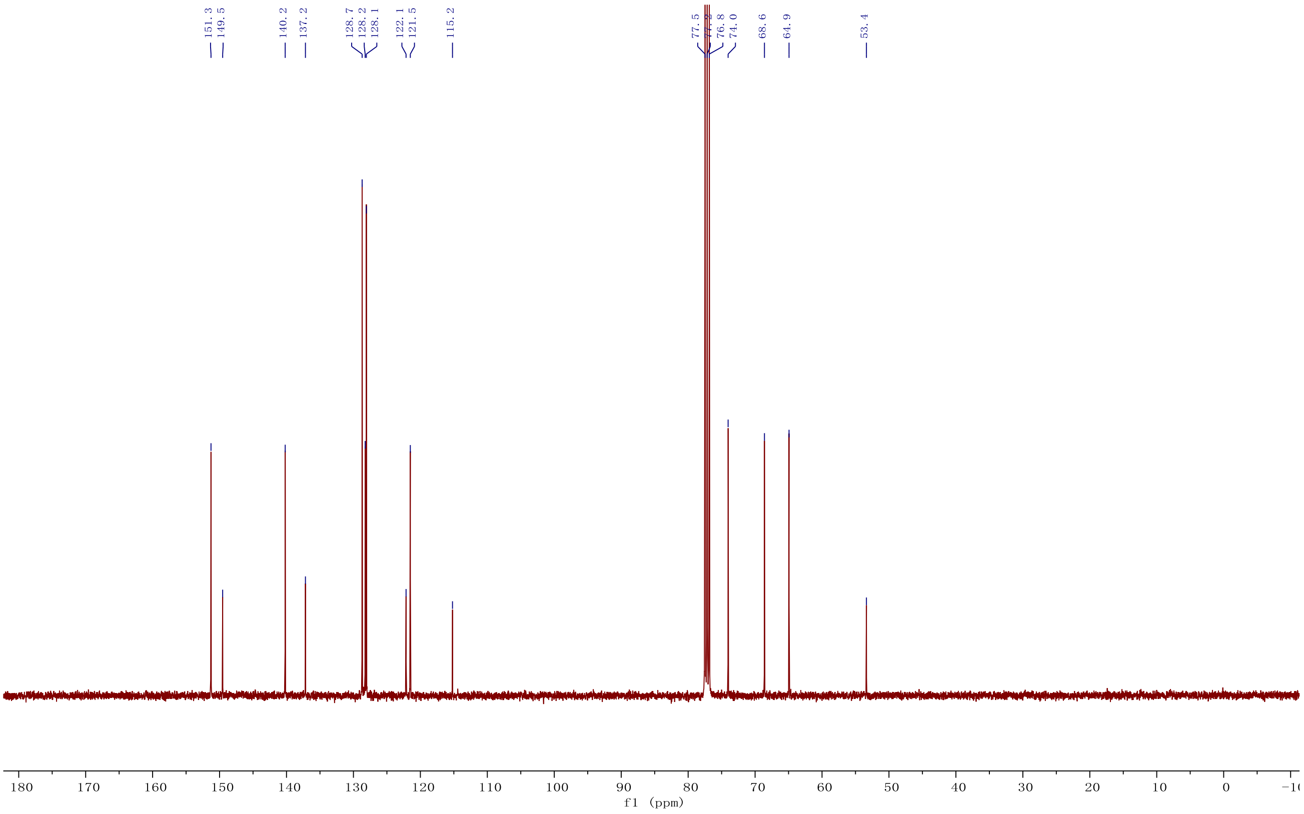

Supplementary Figure 363. ^{1}H NMR spectrum of compound **3cj** (400 MHz, $CDCl_3$)

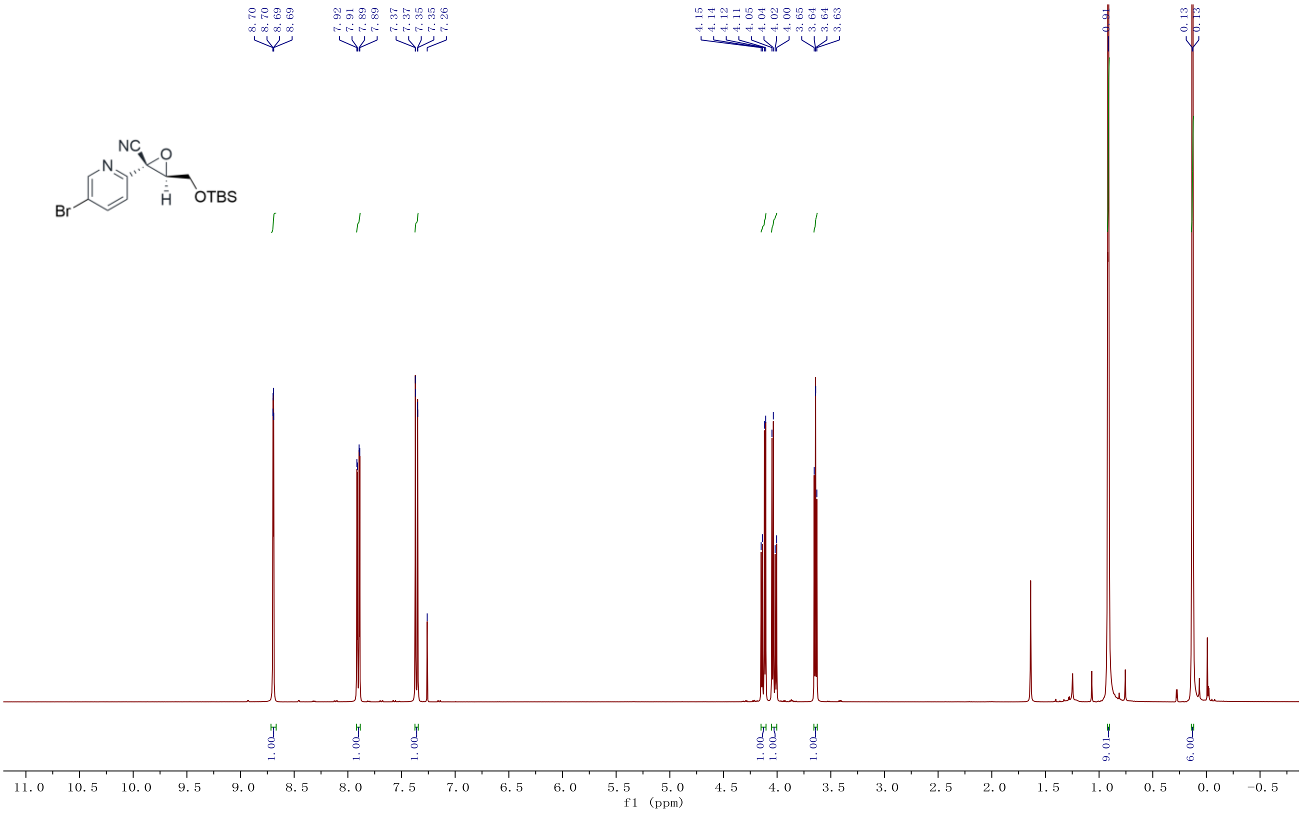

Supplementary Figure 364. ^{13}C NMR spectrum of compound **3cj** (100 MHz, $CDCl_3$)

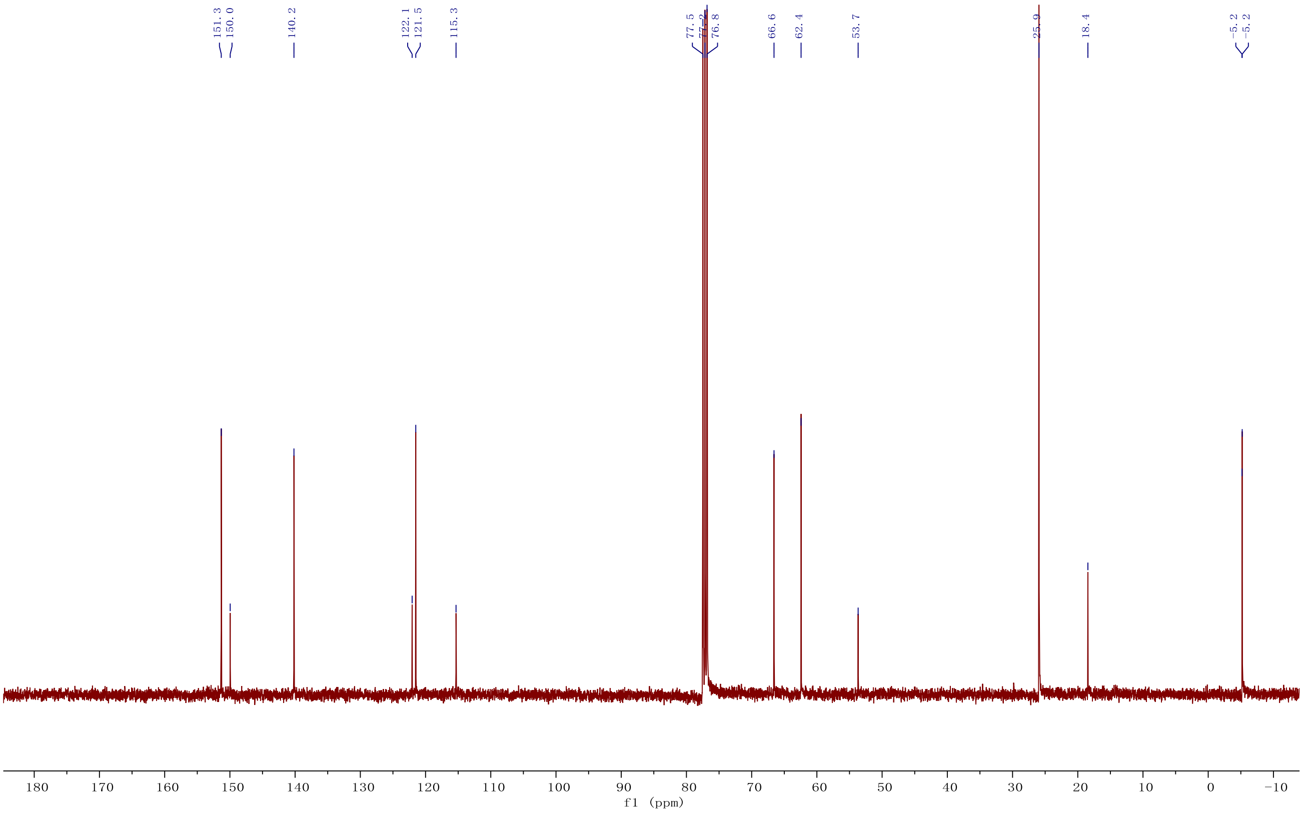

Supplementary Figure 365. ¹H NMR spectrum of compound **3ck** (400 MHz, $CDCl_3$)

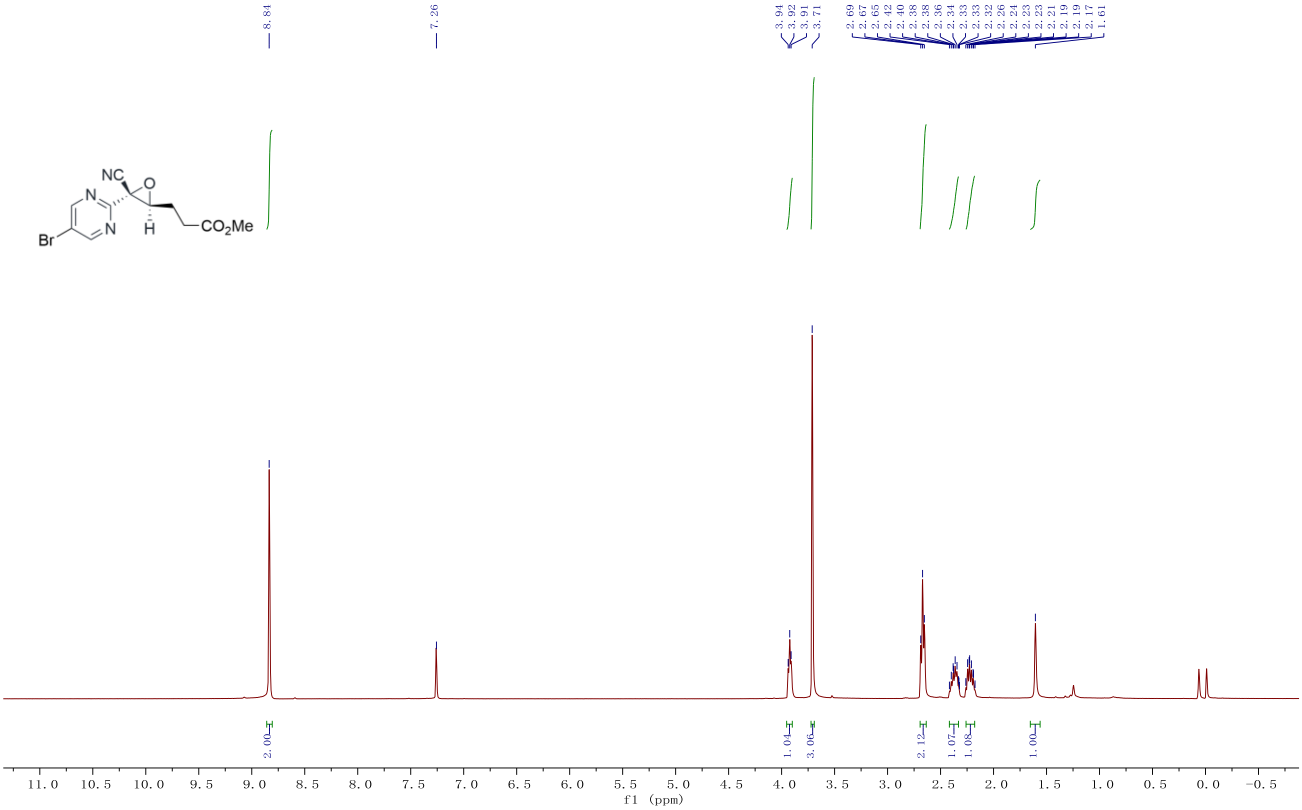

Supplementary Figure 366. ¹³C NMR spectrum of compound **3ck** (100 MHz, $CDCl_3$)

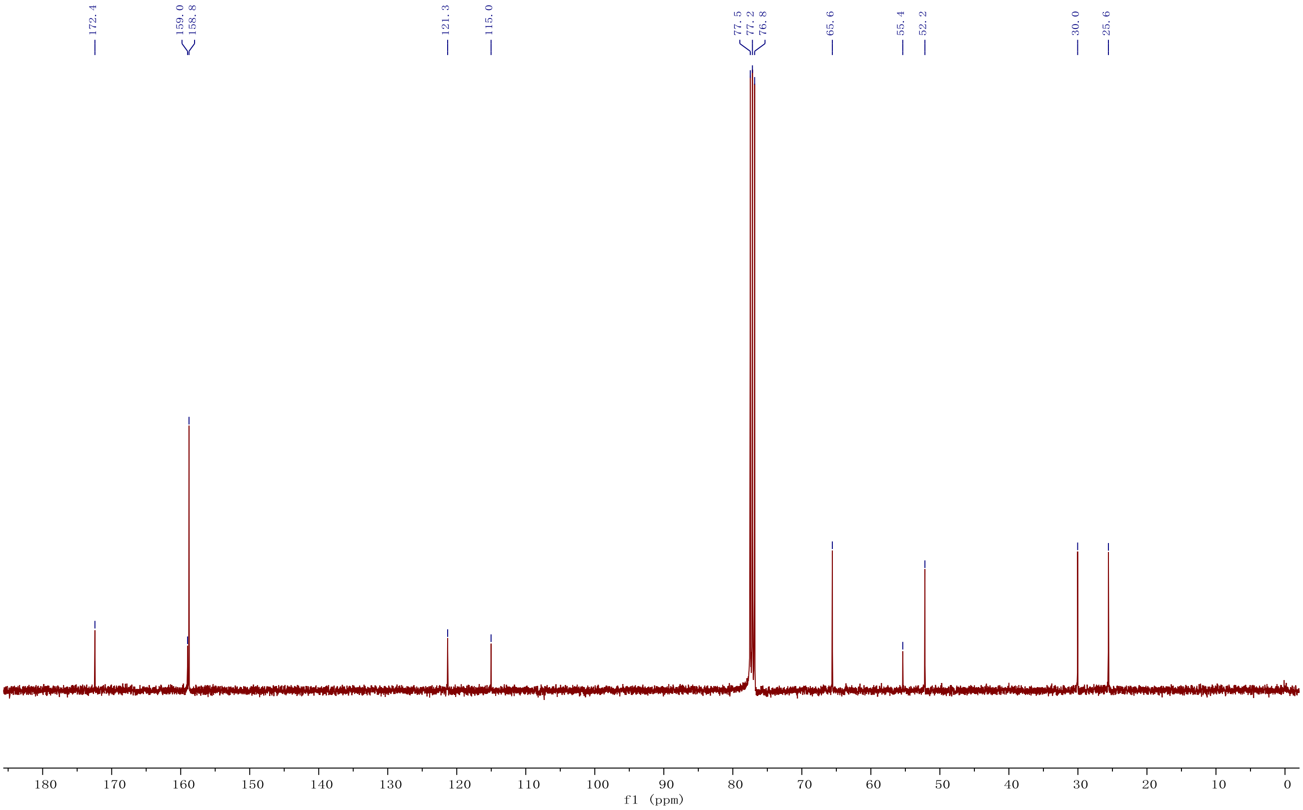

Supplementary Figure 367. ^{1}H NMR spectrum of compound **3cl** (400 MHz, $CDCl_3$)

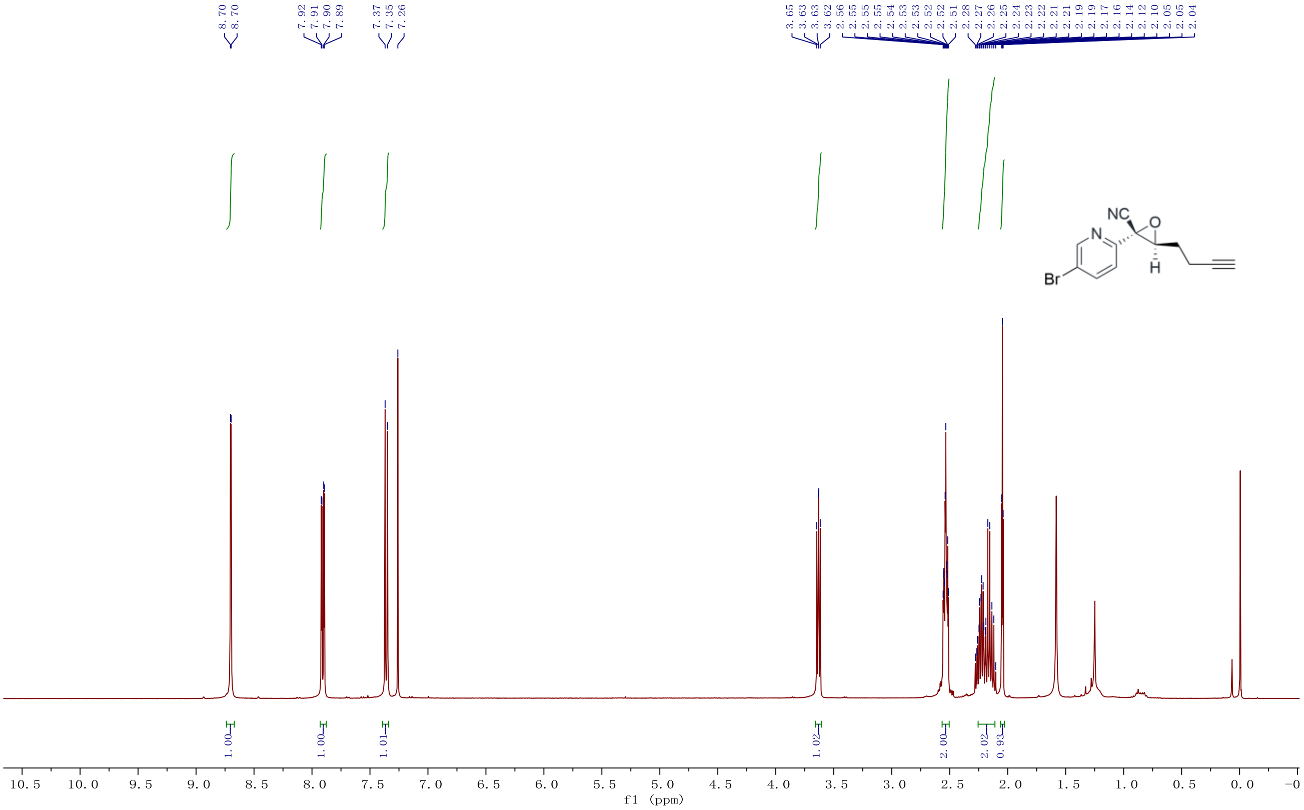

Supplementary Figure 368. ^{13}C NMR spectrum of compound **3cl** (100 MHz, $CDCl_3$)

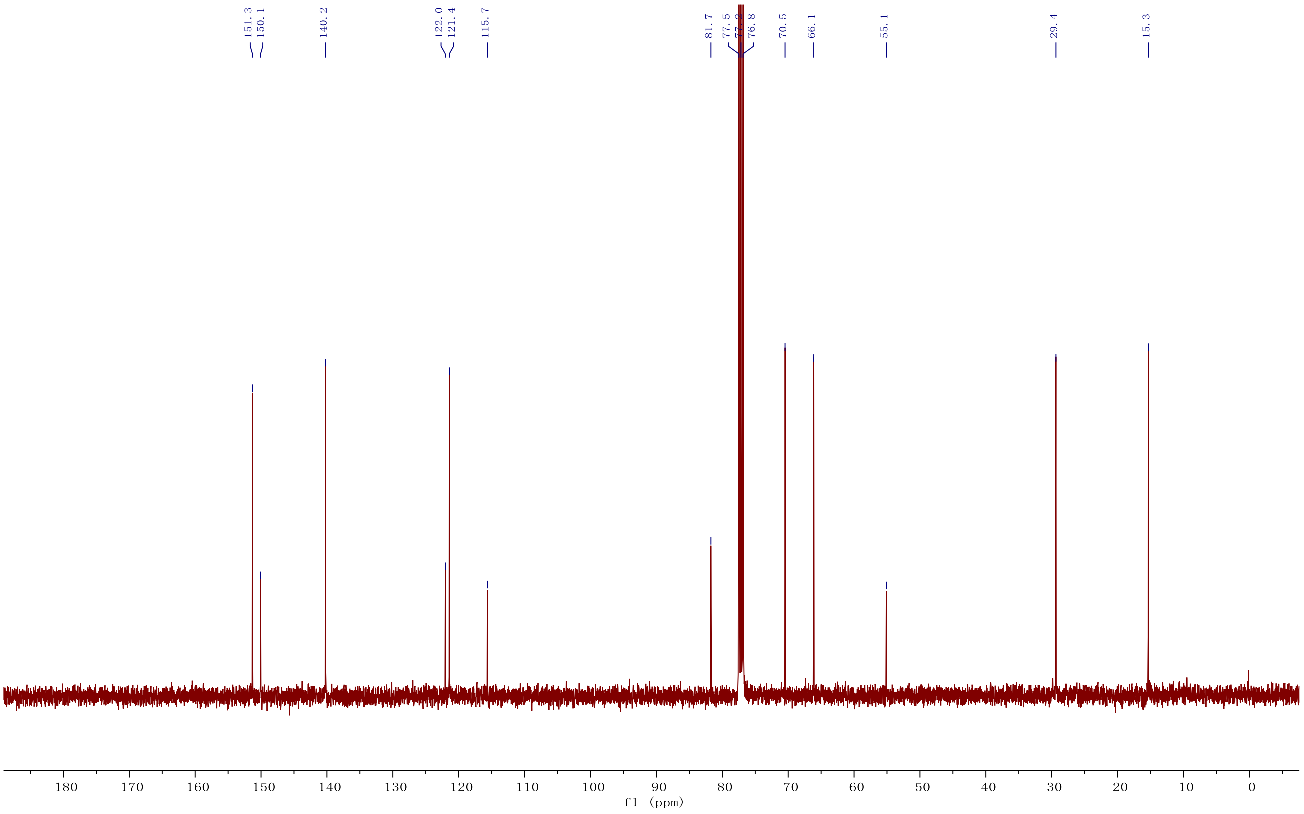

Supplementary Figure 369. ^{1}H NMR spectrum of compound **3cm** (400 MHz, DMSO-d_6)

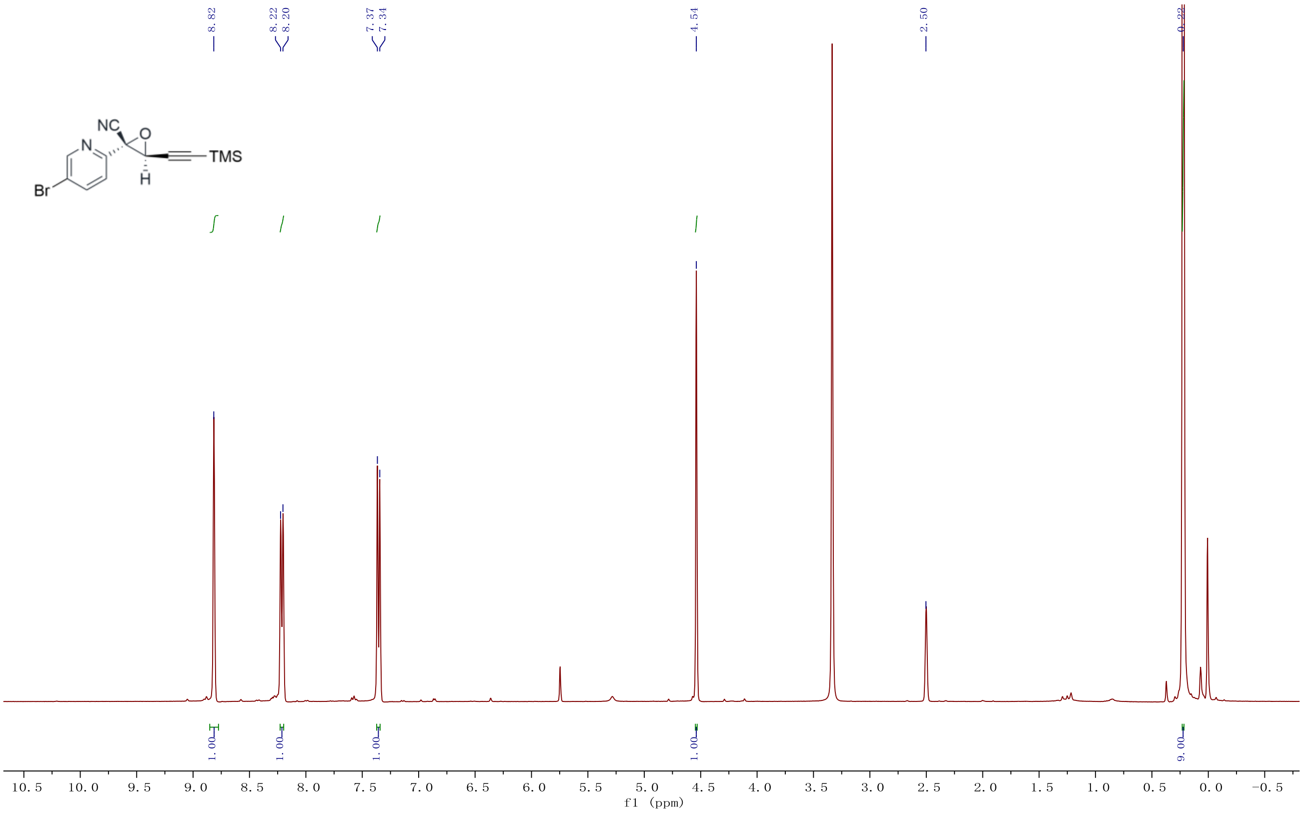

Supplementary Figure 370. ^{13}C NMR spectrum of compound **3cm** (100 MHz, DMSO-d_6)

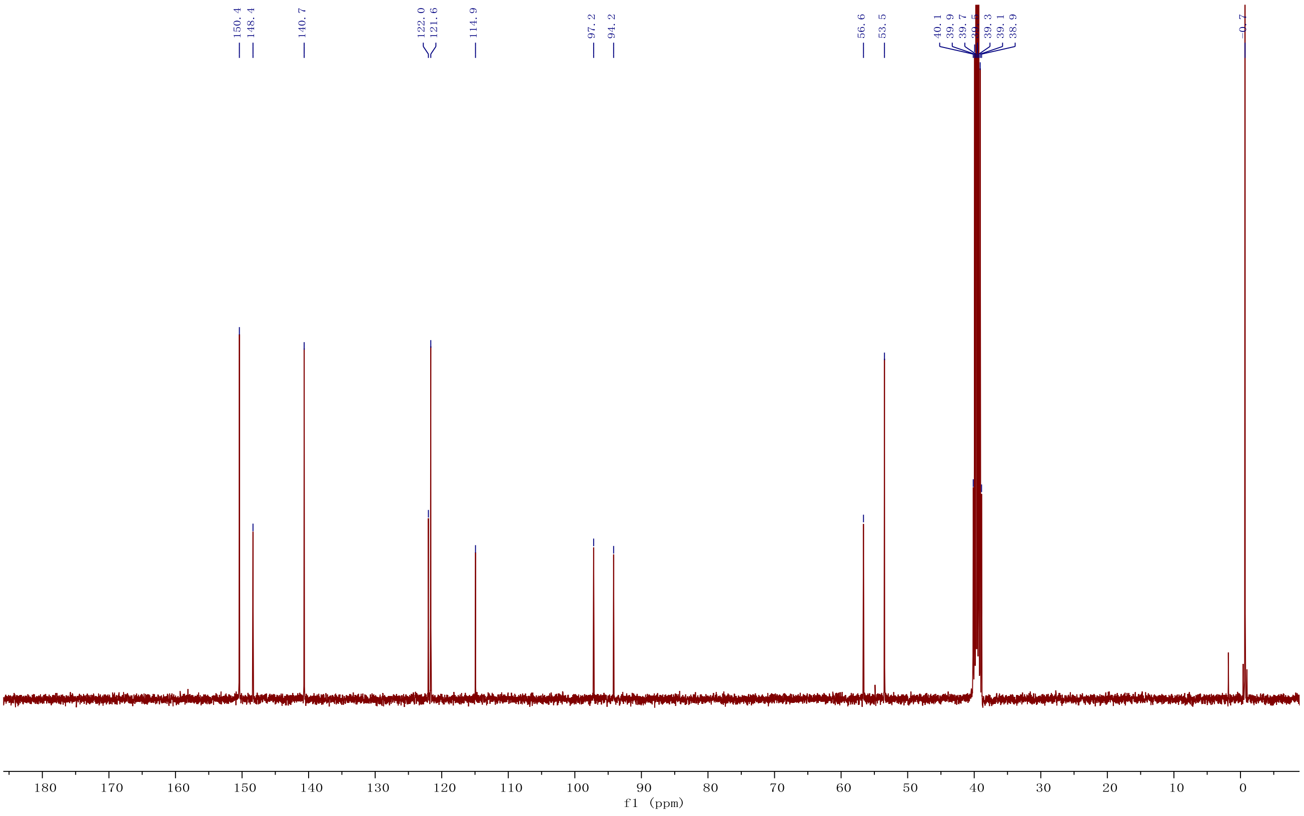

Supplementary Figure 371. ^{1}H NMR spectrum of compound **3cn** (400 MHz, $CDCl_3$)

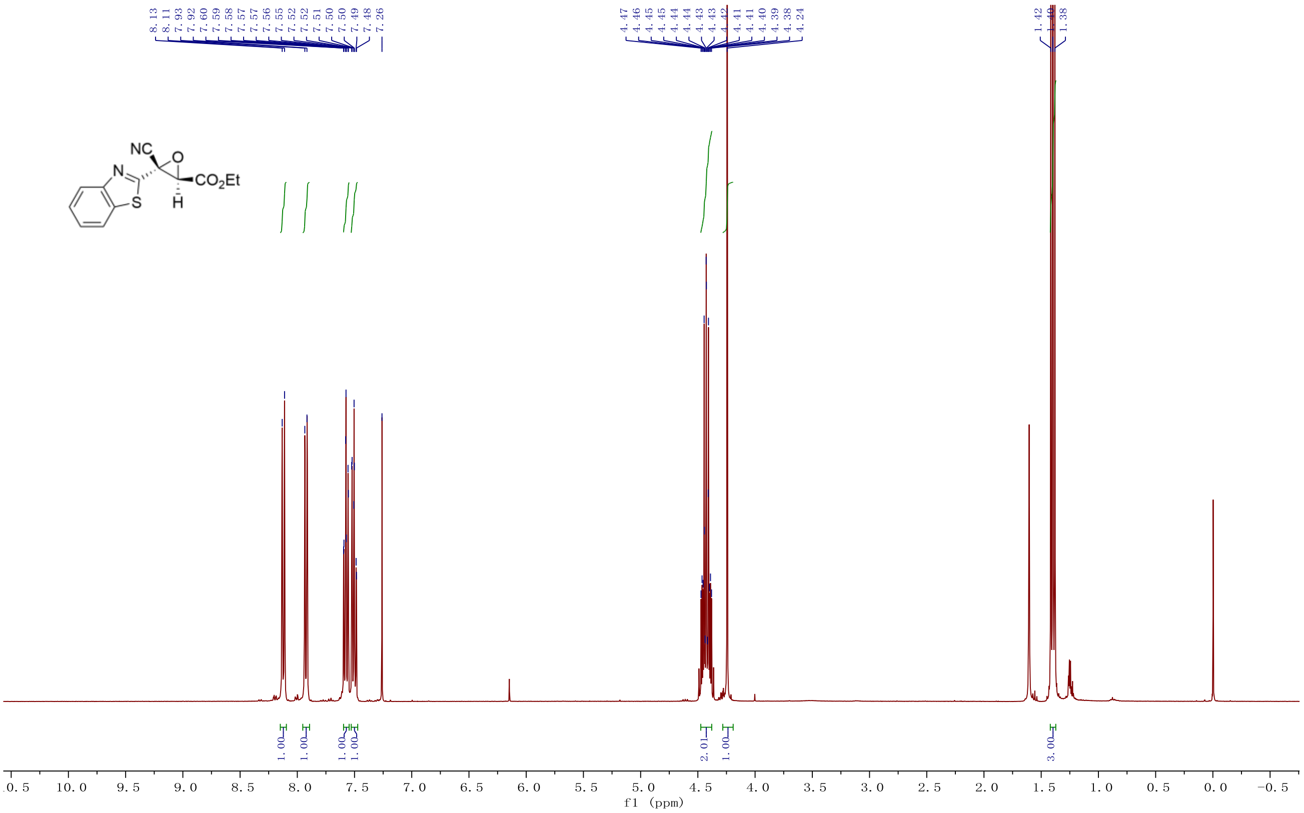

Supplementary Figure 372. ^{13}C NMR spectrum of compound **3cn** (100 MHz, $CDCl_3$)

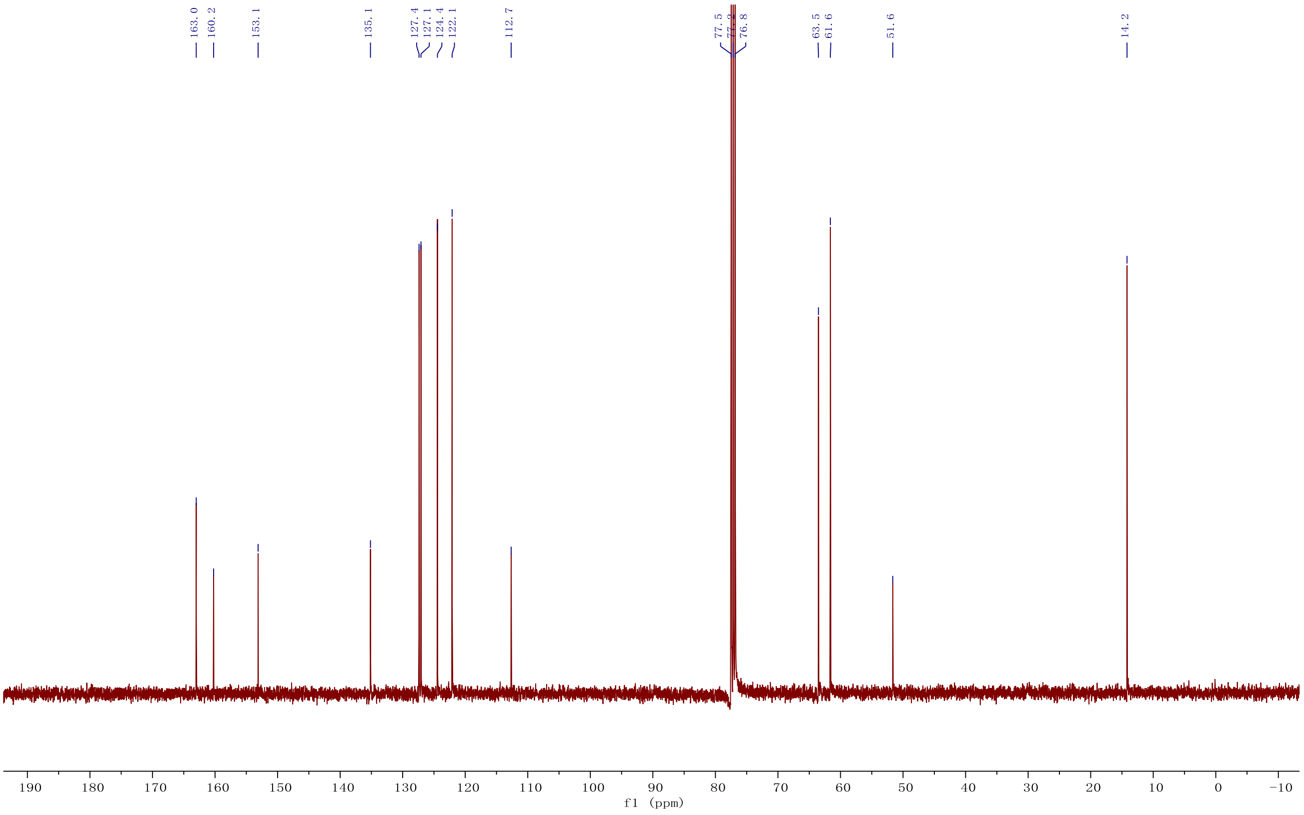

Supplementary Figure 373. ¹H NMR spectrum of compound **3co** (400 MHz, $CDCl_3$)

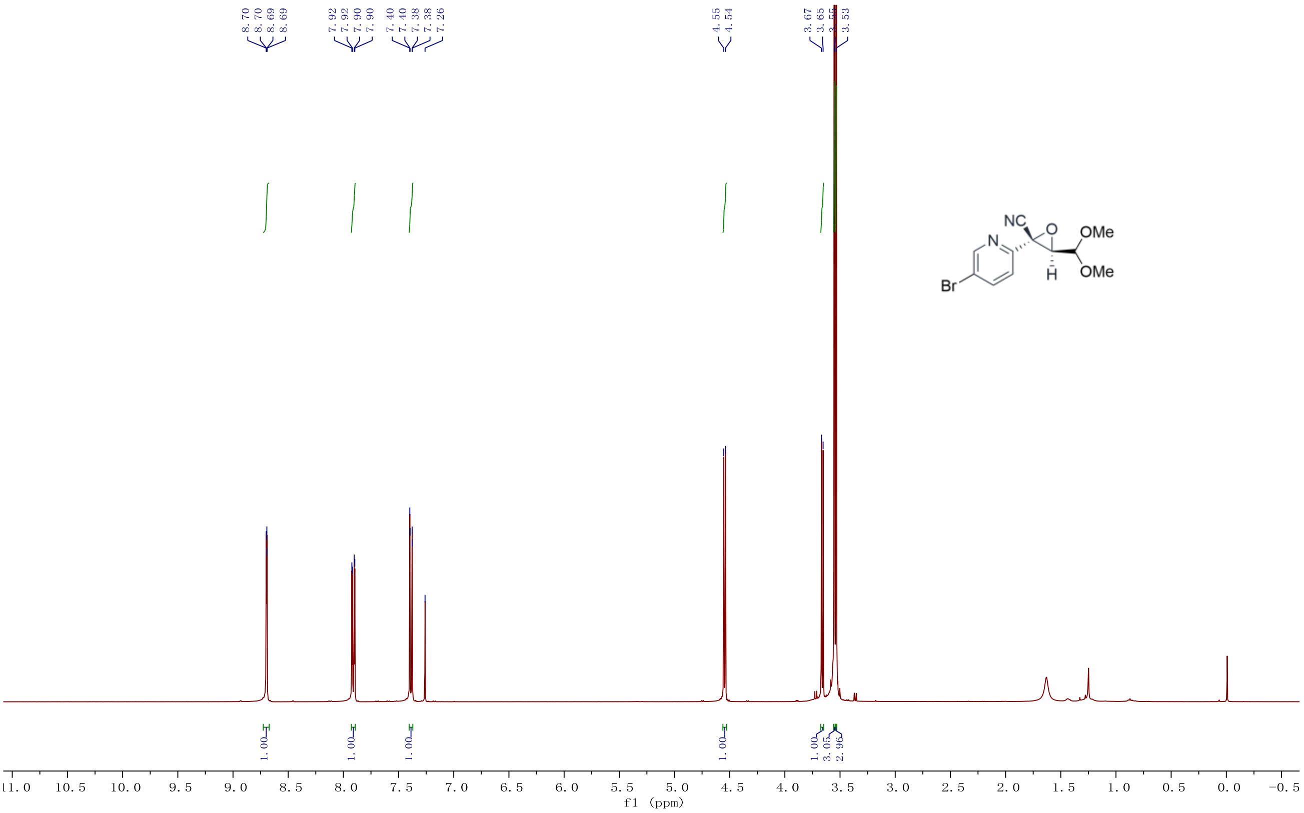

Supplementary Figure 374. ¹³C NMR spectrum of compound **3co** (100 MHz, $CDCl_3$)

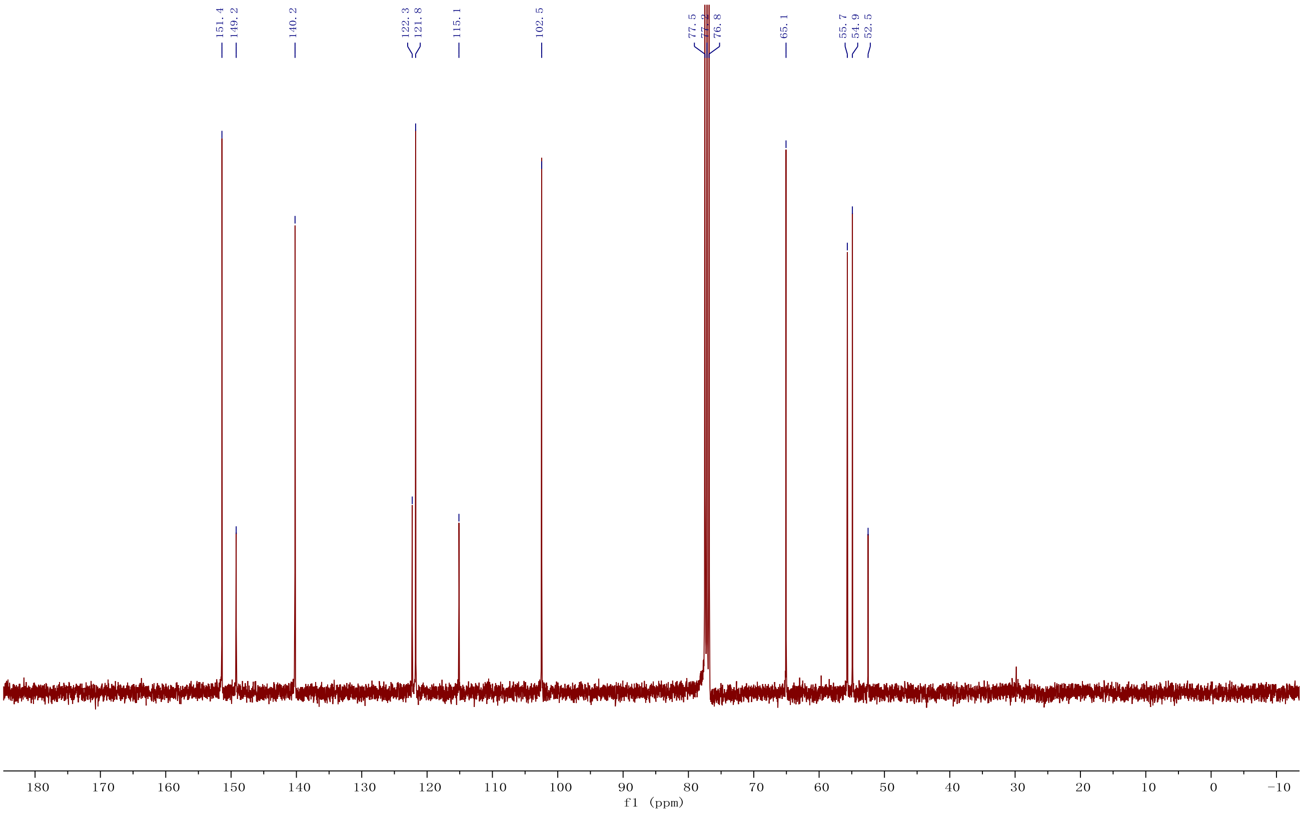

Supplementary Figure 375. ^{1}H NMR spectrum of compound **3da** (400 MHz, $CDCl_3$)

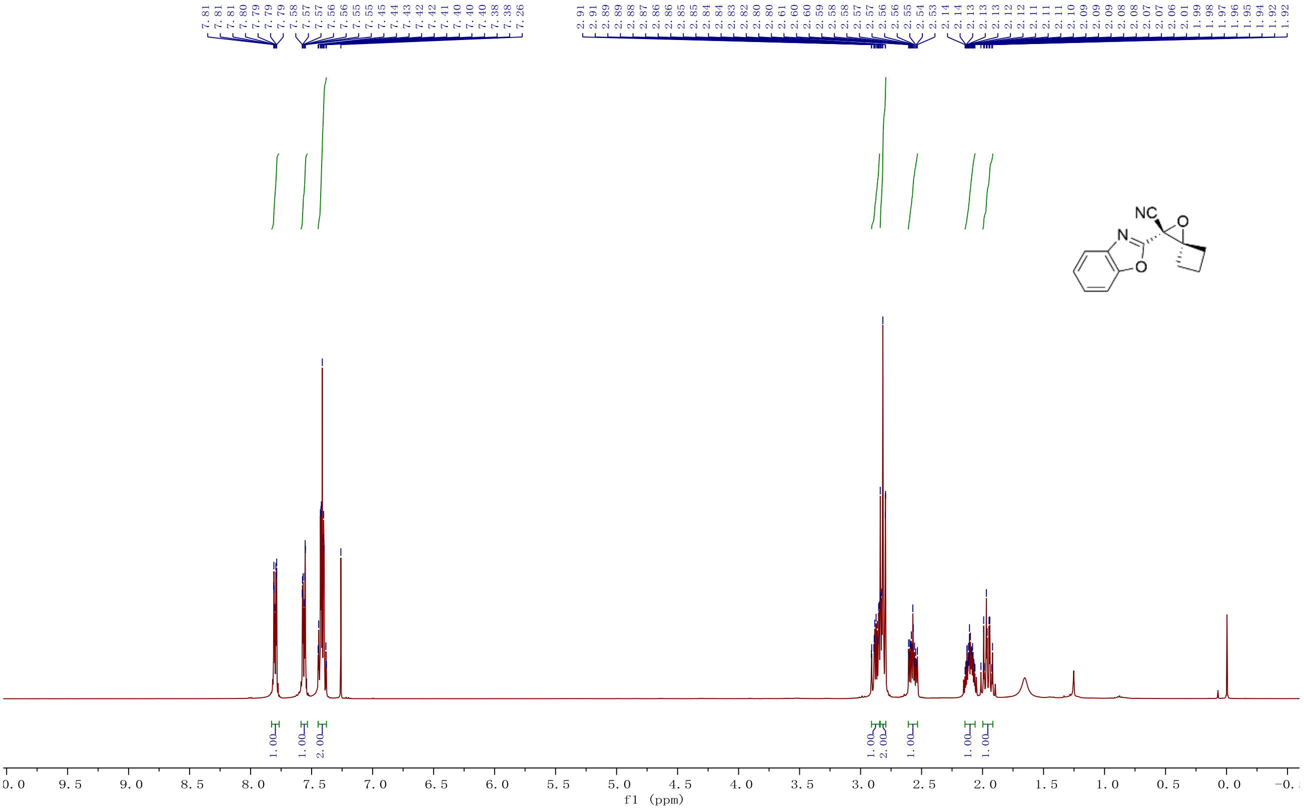

Supplementary Figure 376. ^{13}C NMR spectrum of compound **3da** (100 MHz, $CDCl_3$)

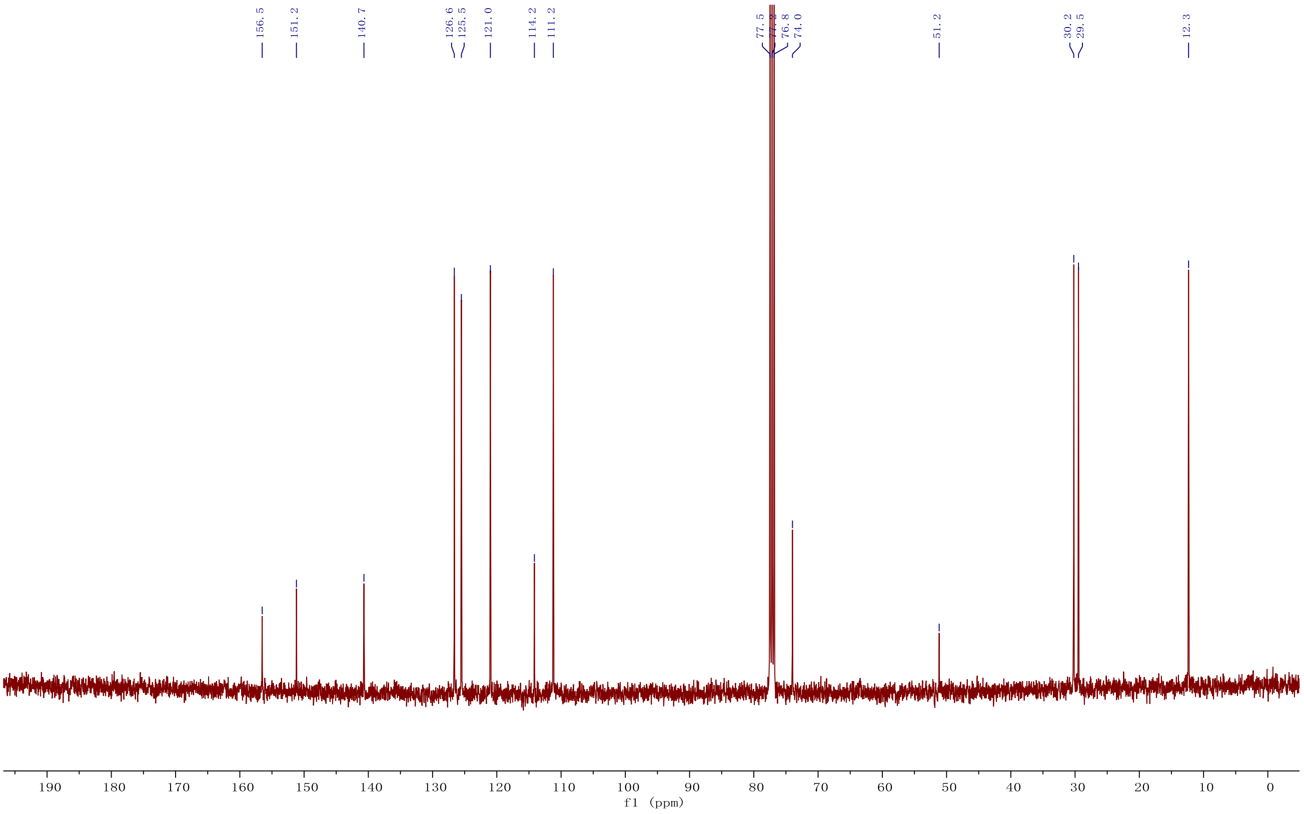

Supplementary Figure 377. ^{1}H NMR spectrum of compound **3db** (600 MHz, $CDCl_3$)

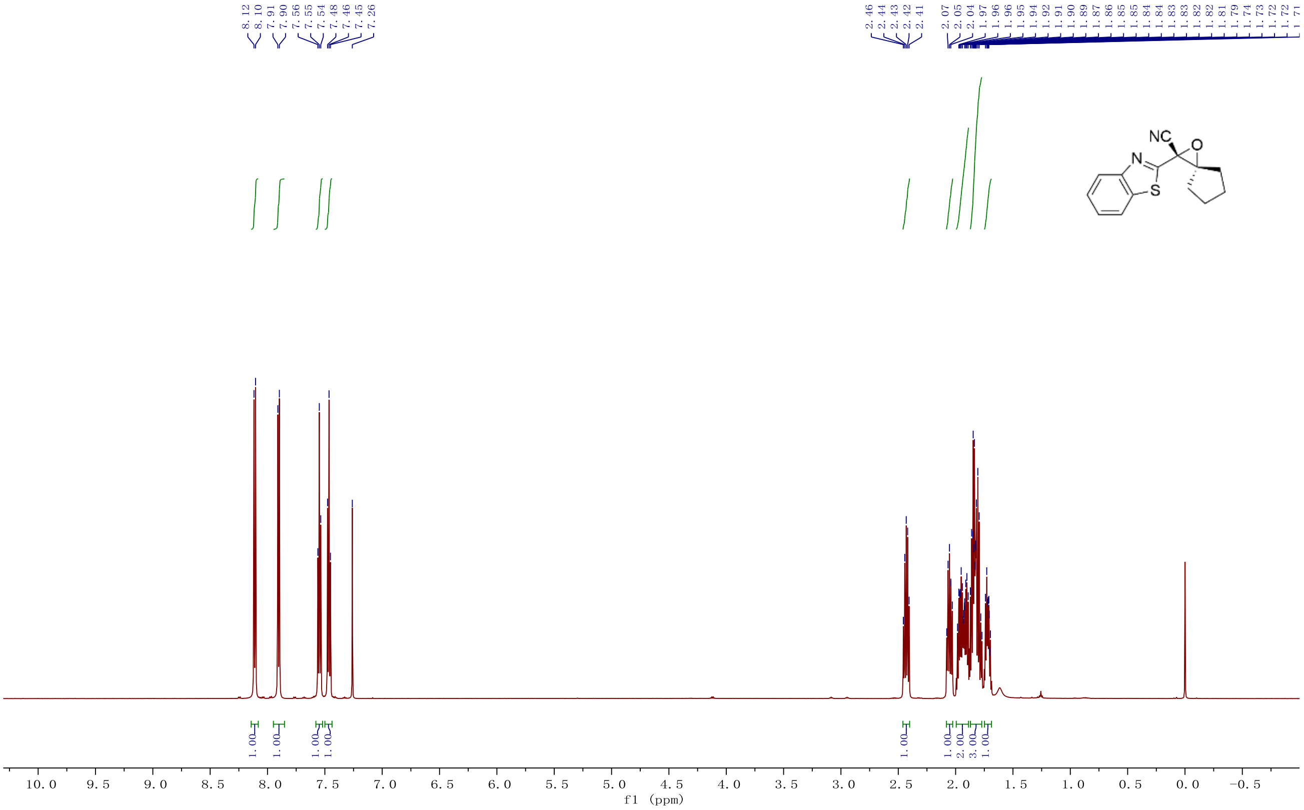

Supplementary Figure 378. ^{13}C NMR spectrum of compound **3db** (150 MHz, $CDCl_3$)

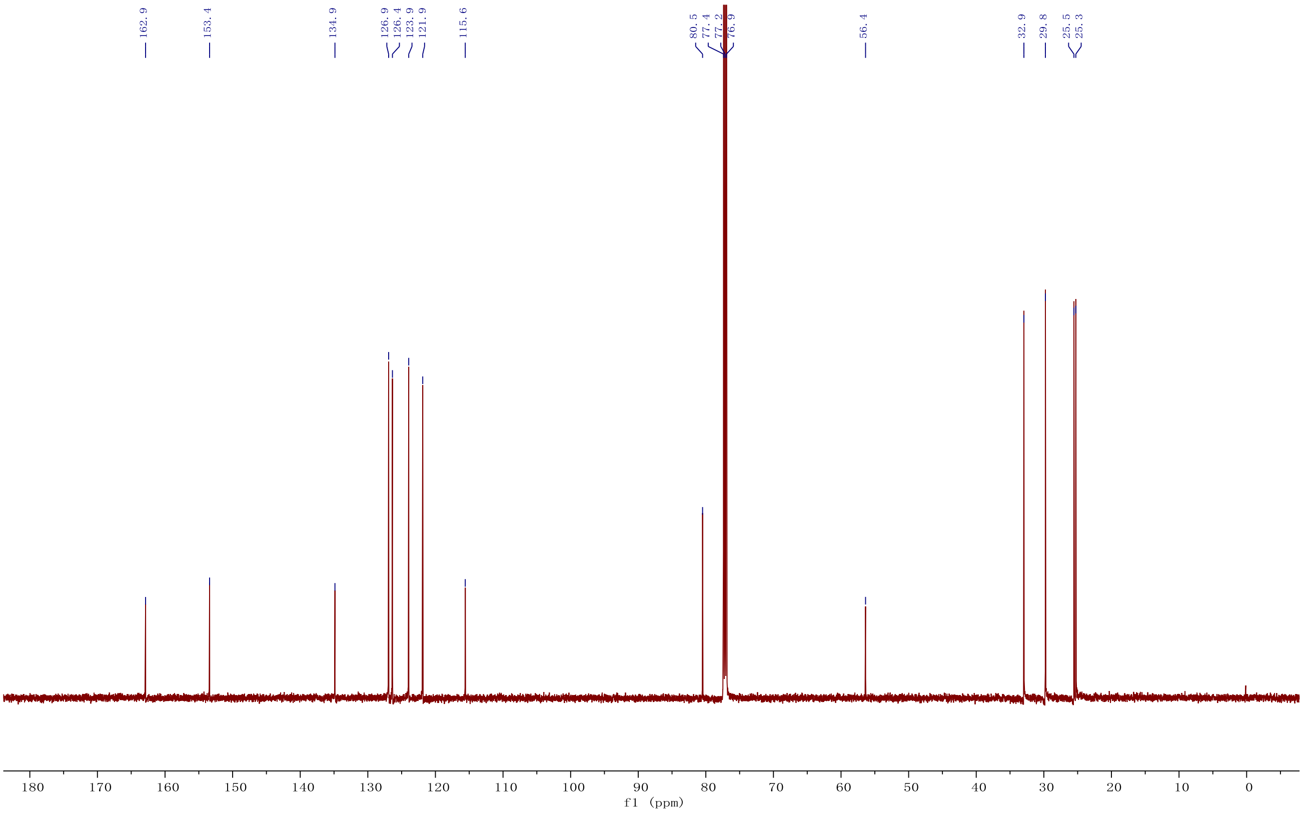

Supplementary Figure 379. ^{1}H NMR spectrum of compound **3dc** (400 MHz, $CDCl_3$)

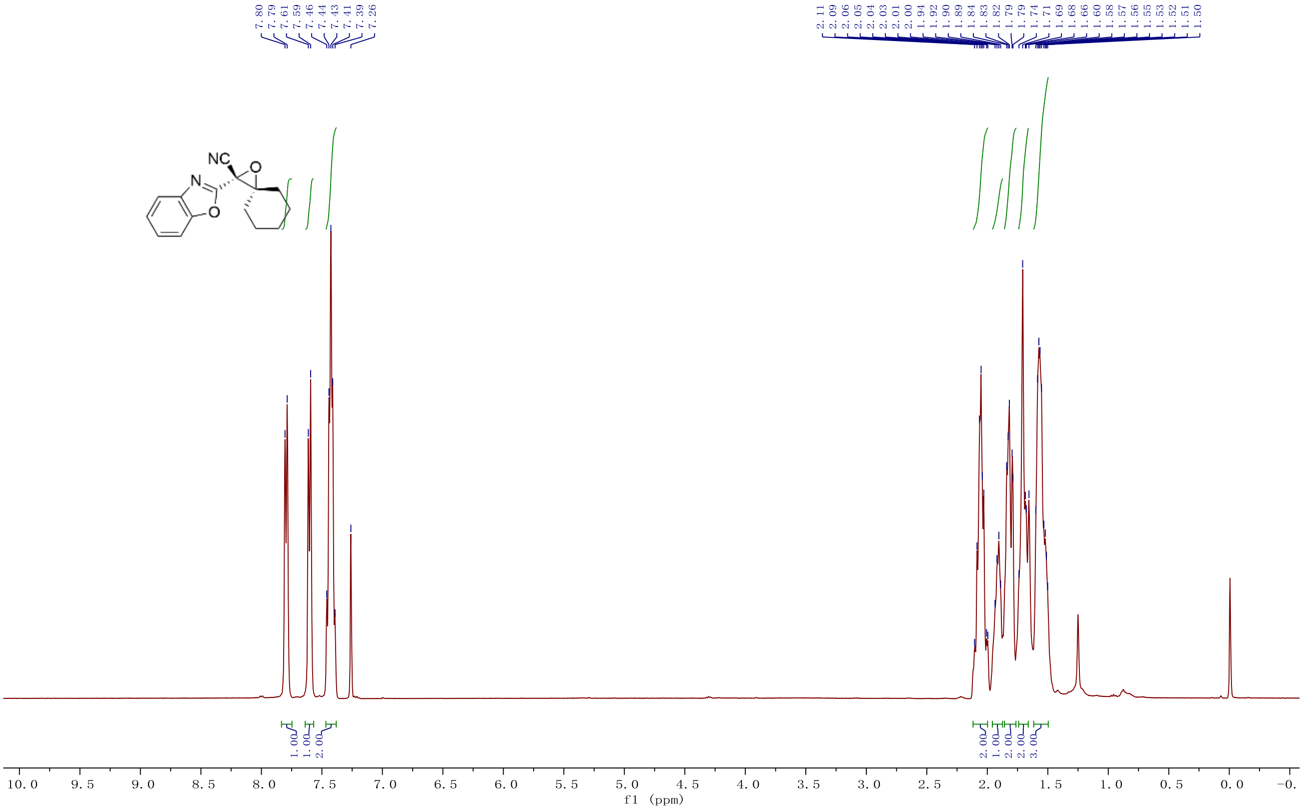

Supplementary Figure 380. ^{13}C NMR spectrum of compound **3dc** (100 MHz, $CDCl_3$)

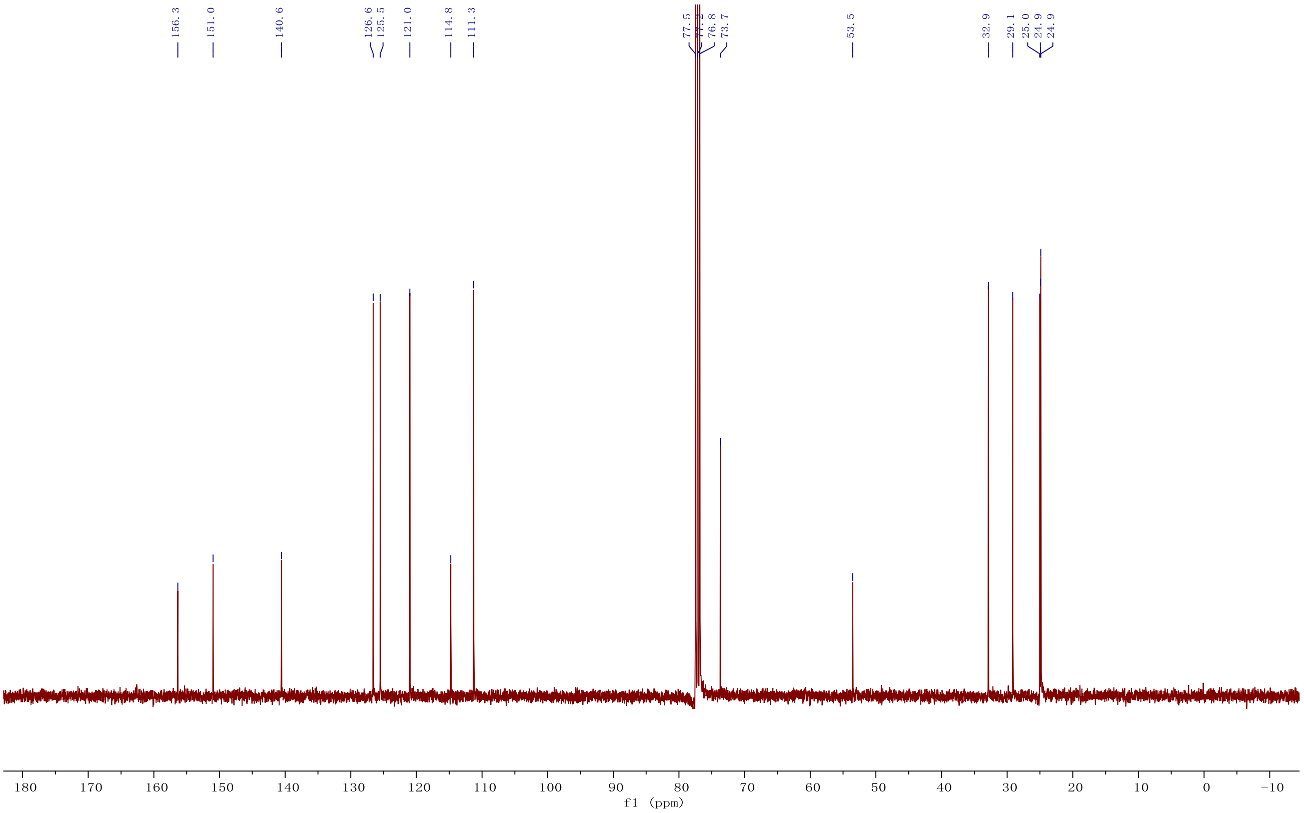

Supplementary Figure 381. ^{1}H NMR spectrum of compound **3dd** (400 MHz, $CDCl_3$)

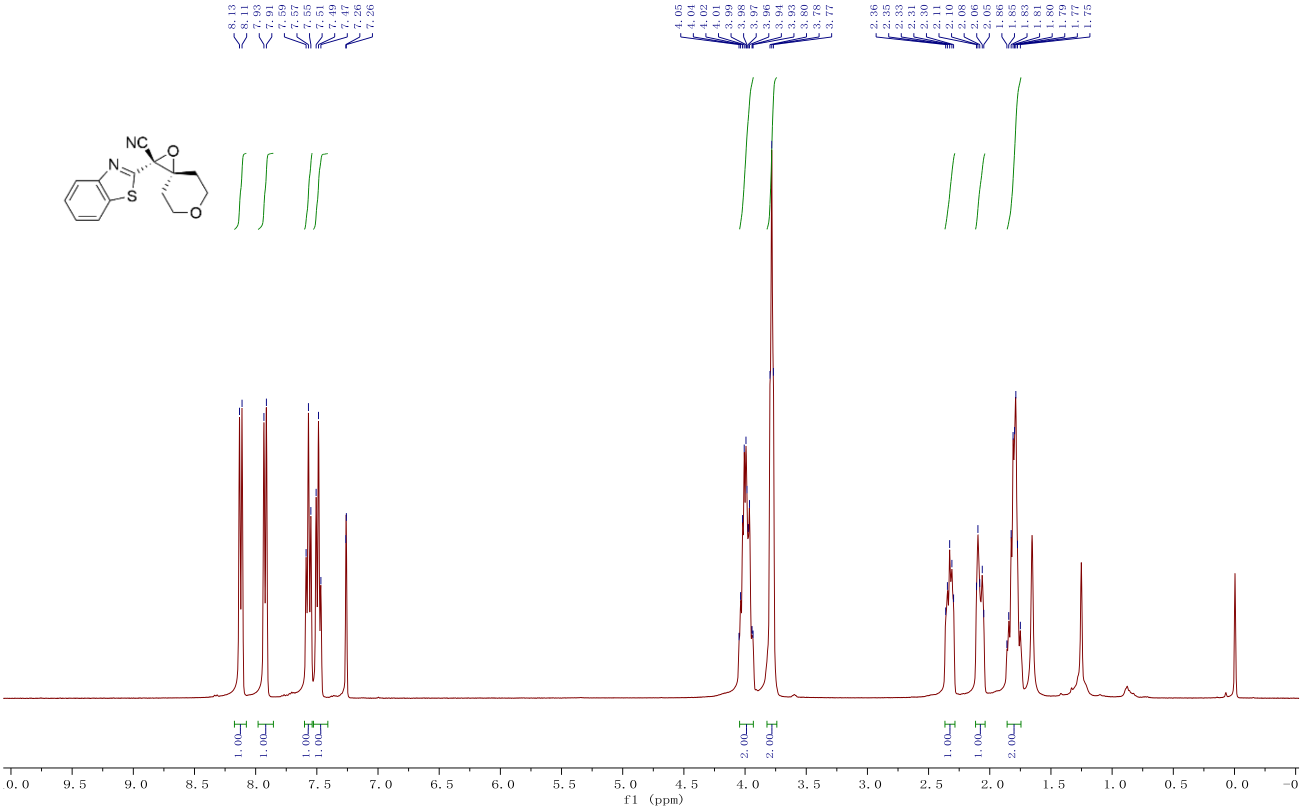

Supplementary Figure 382. ^{13}C NMR spectrum of compound **3dd** (100 MHz, $CDCl_3$)

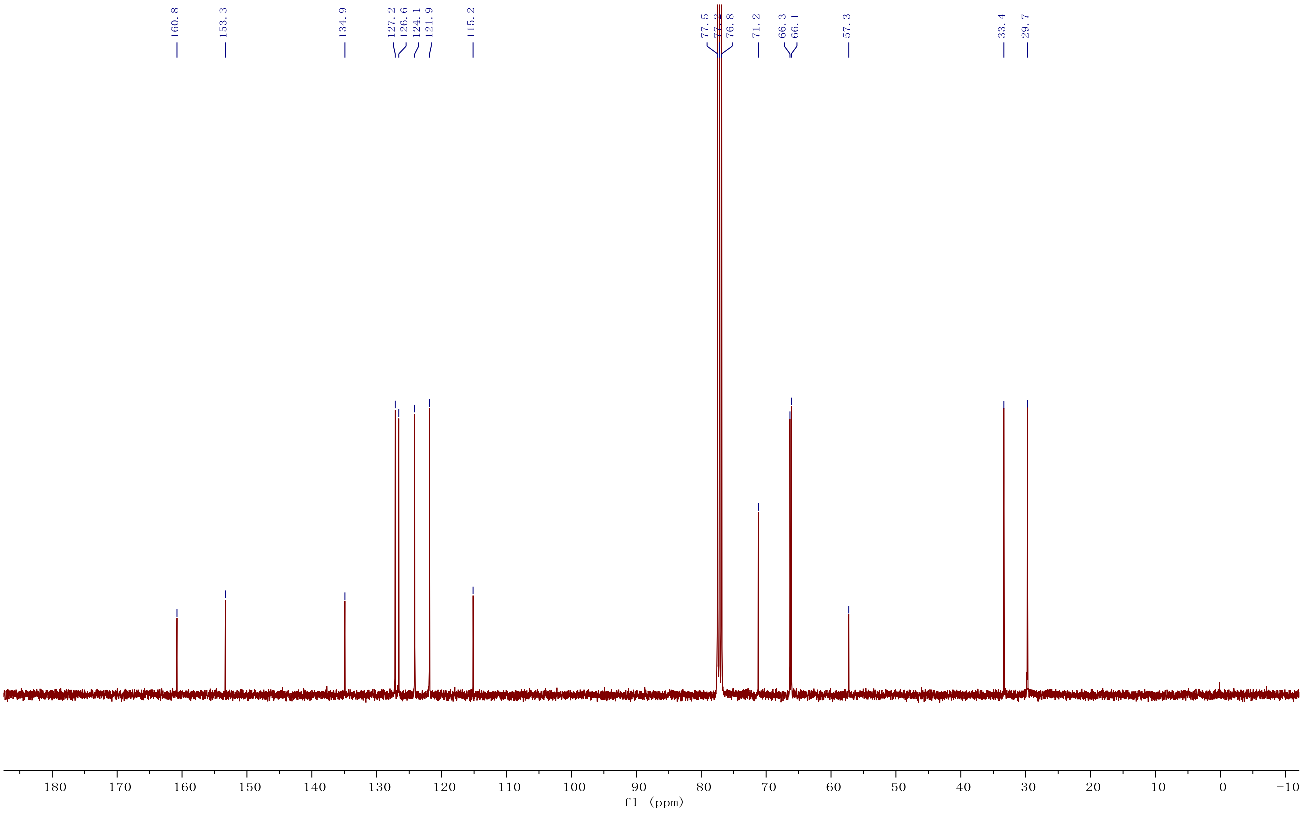

Supplementary Figure 383. ^{1}H NMR spectrum of compound **3de** (400 MHz, $CDCl_3$)

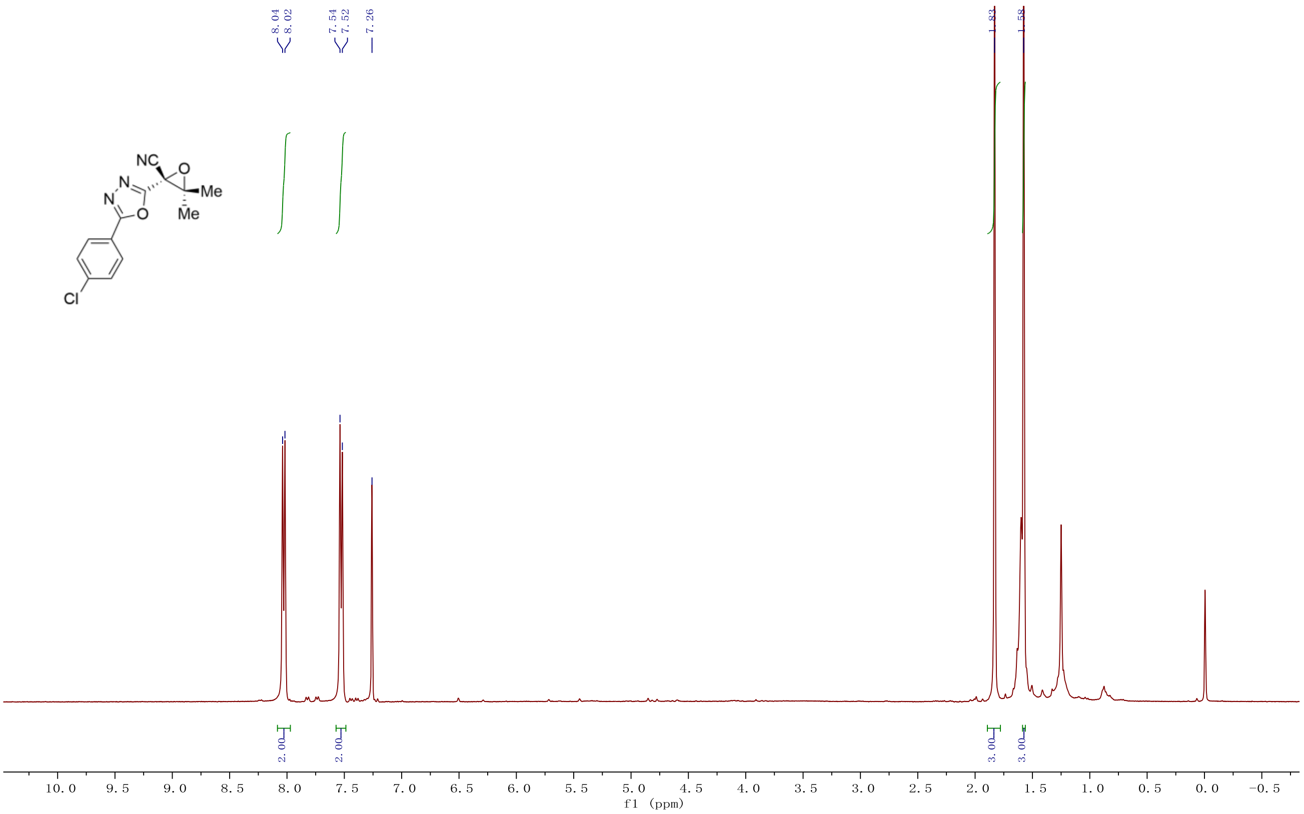

Supplementary Figure 384. ^{13}C NMR spectrum of compound **3de** (100 MHz, $CDCl_3$)

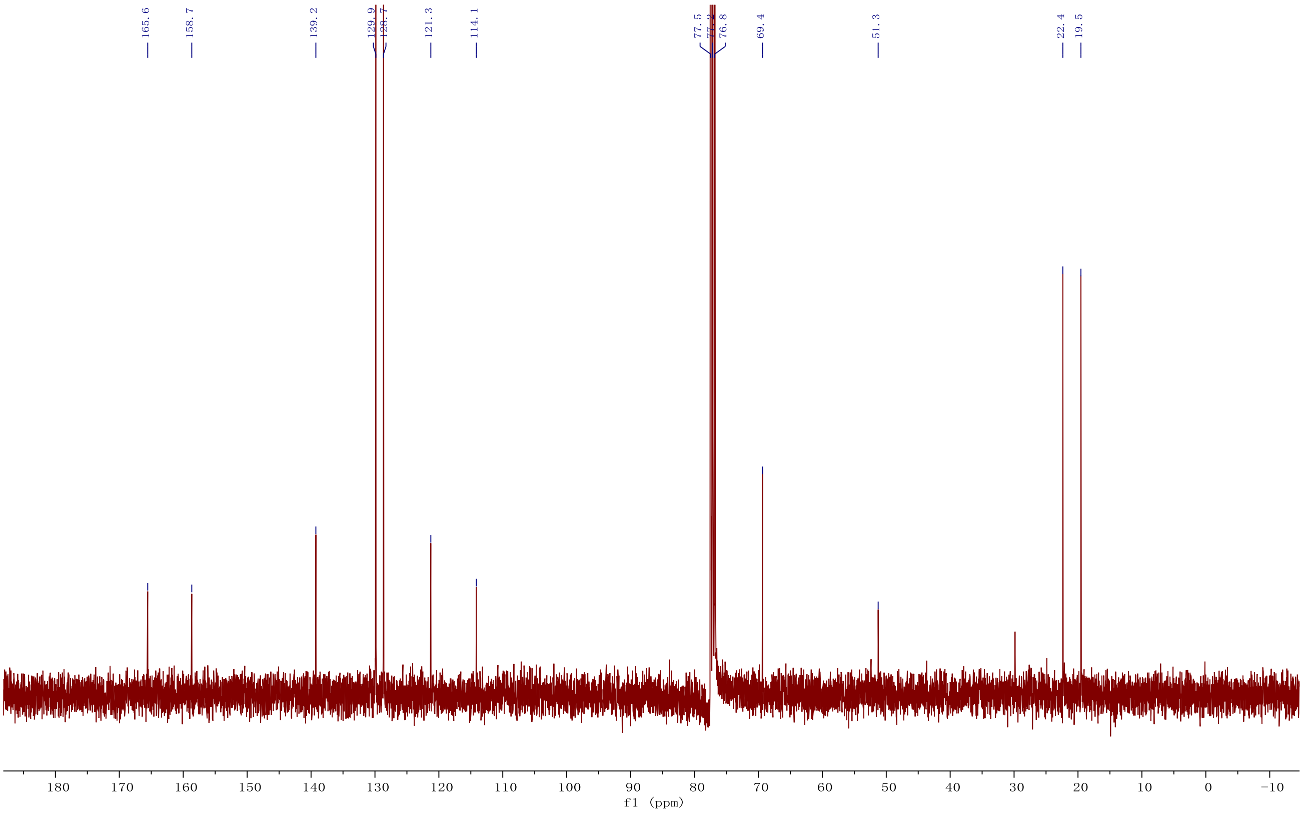

Supplementary Figure 385. ^{1}H NMR spectrum of compound **3df** (400 MHz, $CDCl_3$)

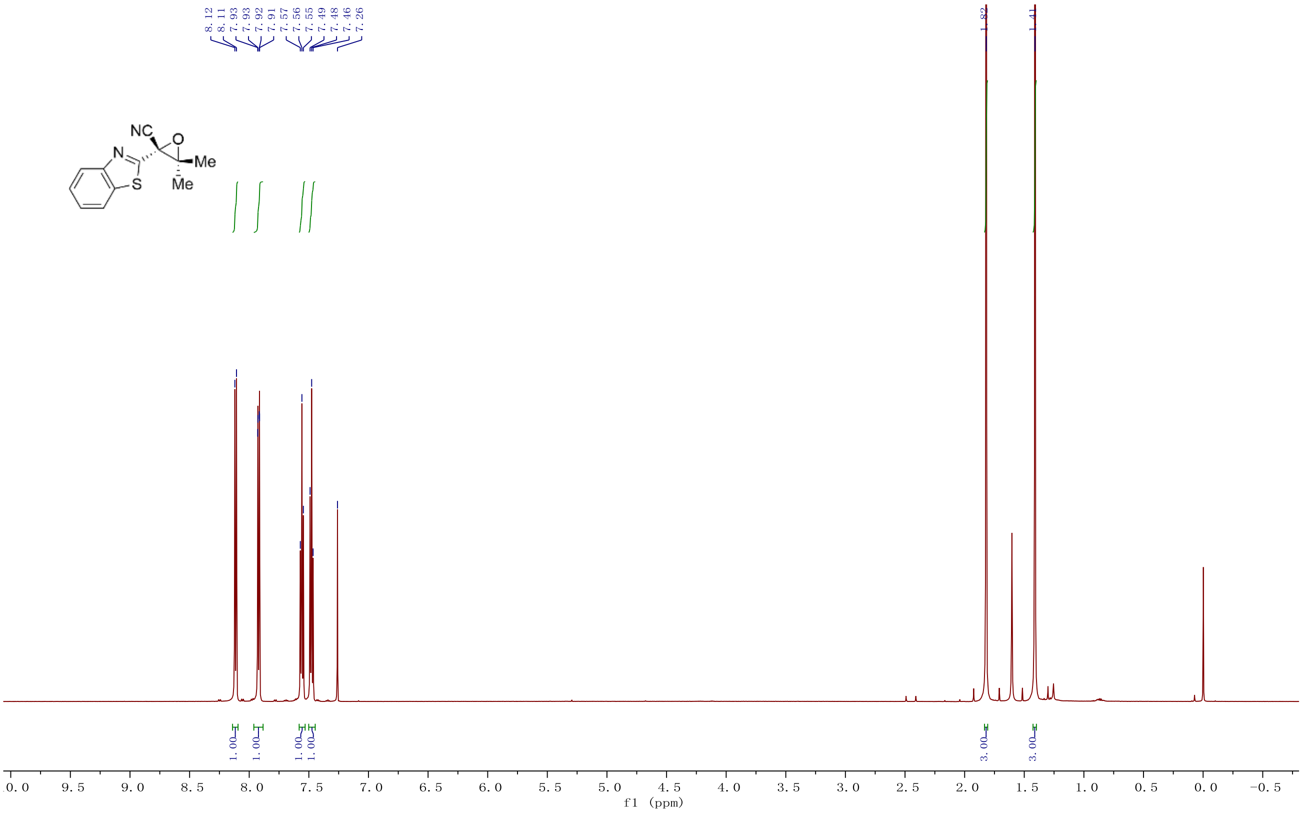

Supplementary Figure 386. ^{13}C NMR spectrum of compound **3df** (100 MHz, $CDCl_3$)

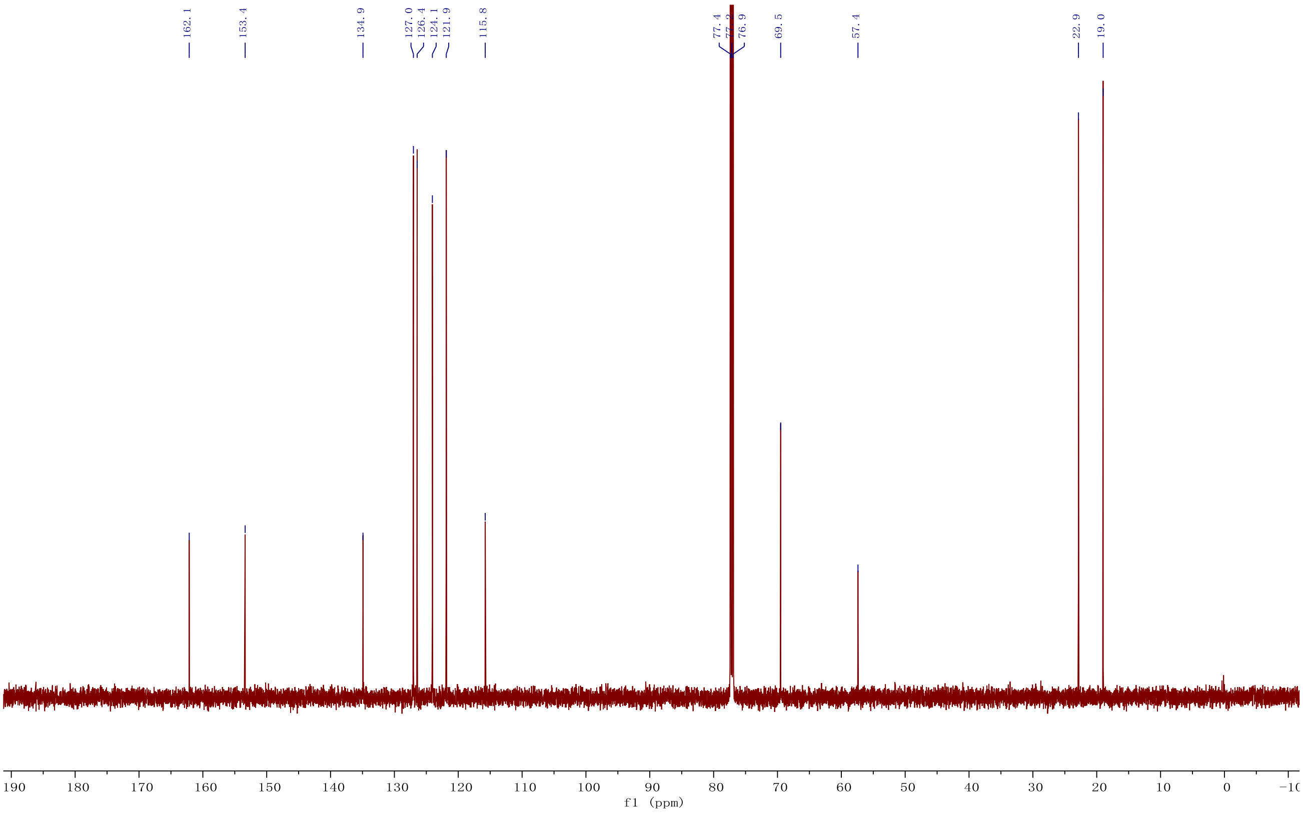

Supplementary Figure 387. ^{1}H NMR spectrum of compound *trans*-**3dg** (400 MHz, $CDCl_3$)

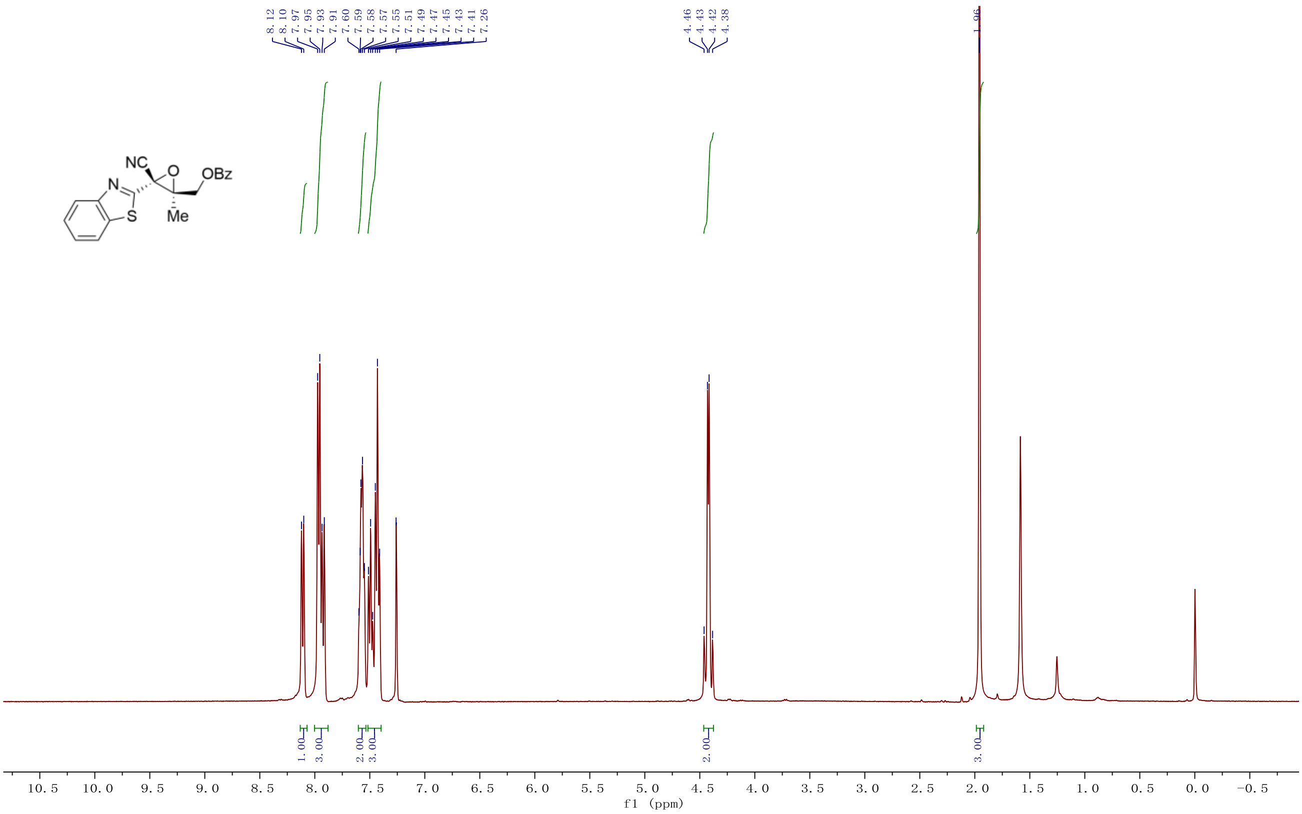

Supplementary Figure 388. ^{13}C NMR spectrum of compound *trans*-**3dg**(100 MHz, $CDCl_3$)

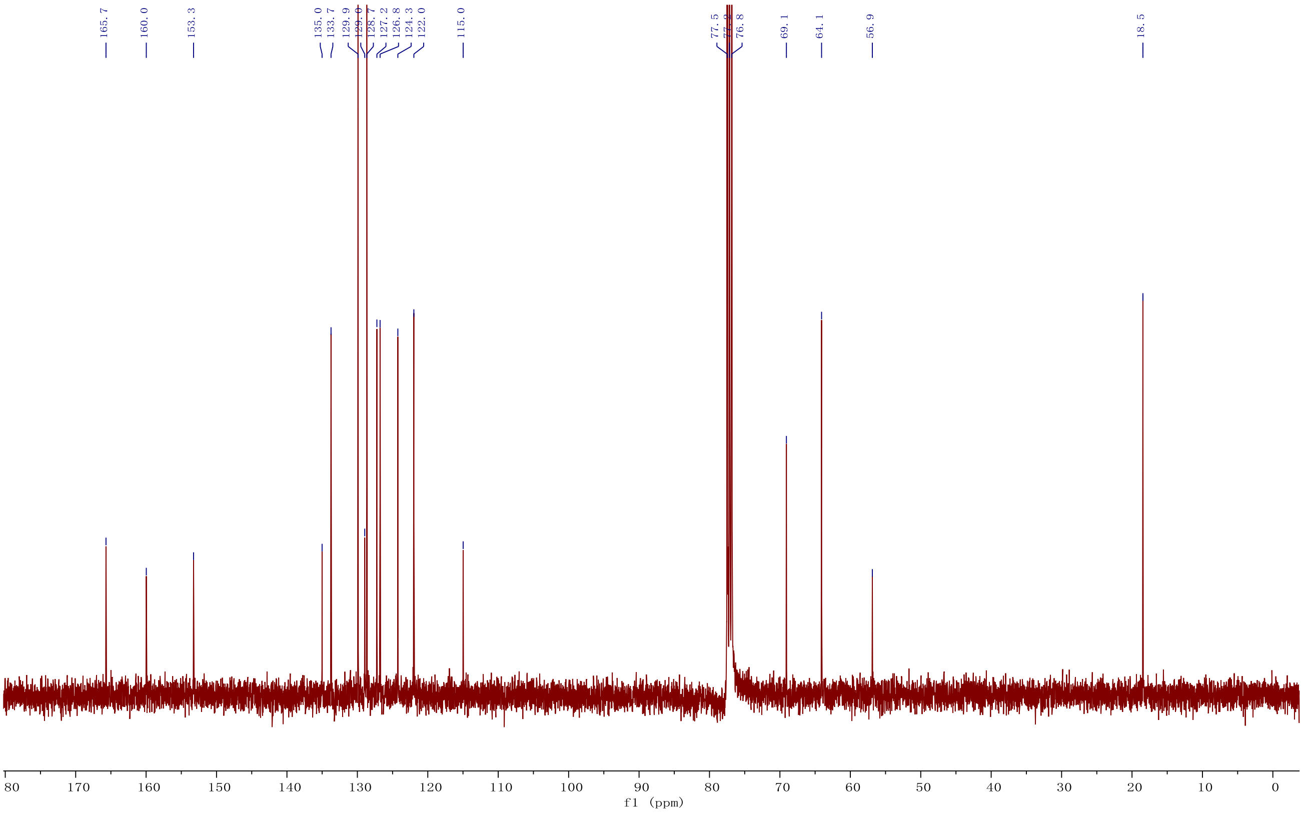

Supplementary Figure 389. 2D NMR spectrum of compound *trans*-**3dg** (600M, HSQC, $CDCl_3$)

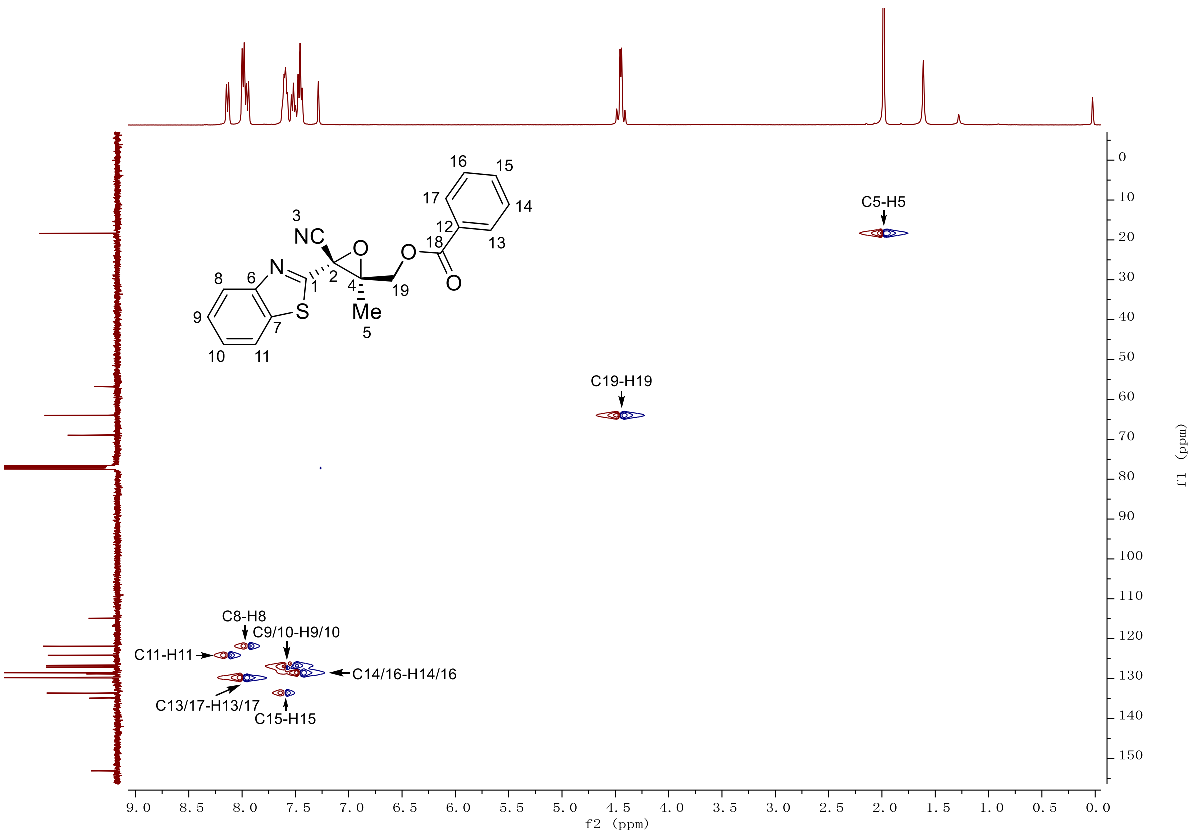

Supplementary Figure 390. 2D NMR spectrum of compound *trans*-**3dg** (600M, HMBC, $CDCl_3$)

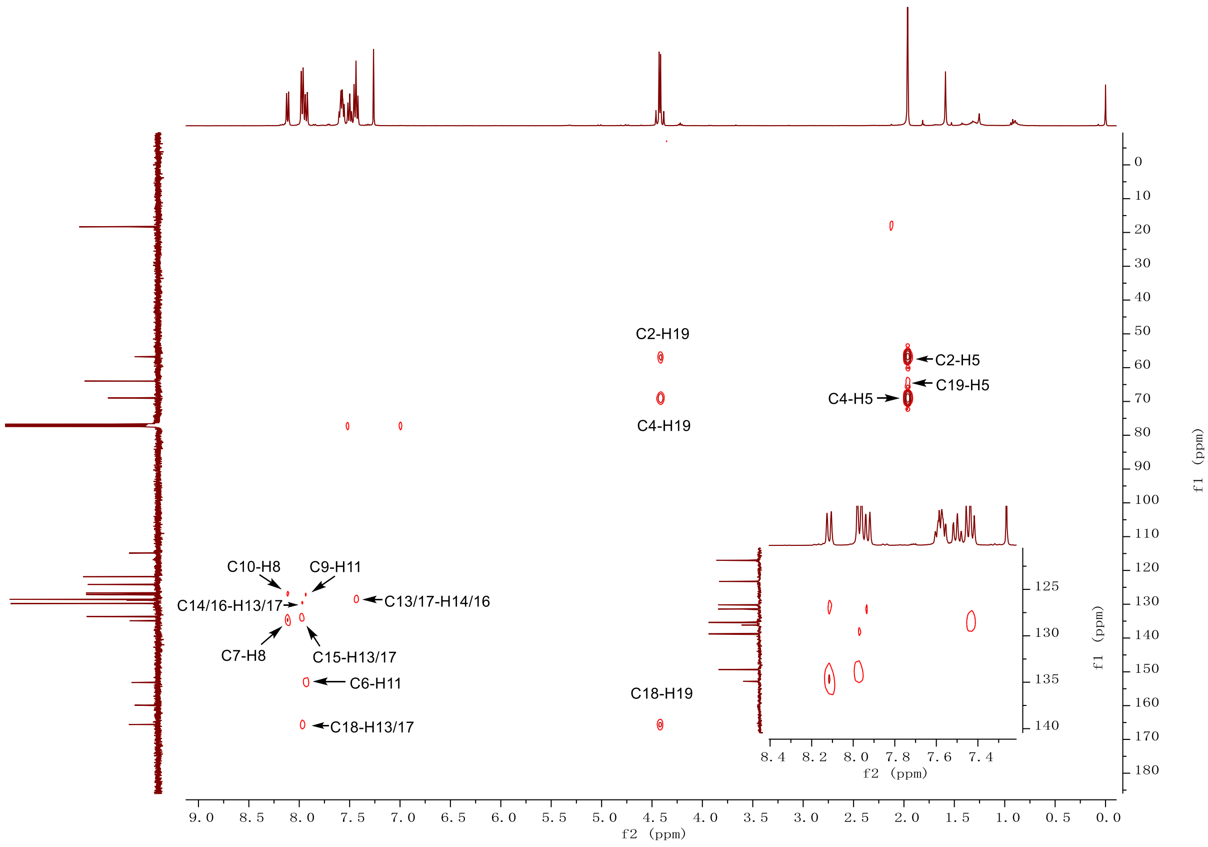

Supplementary Figure 391. ^{1}H NMR spectrum of compound *cis*-**3dg** (400 MHz, $CDCl_3$)

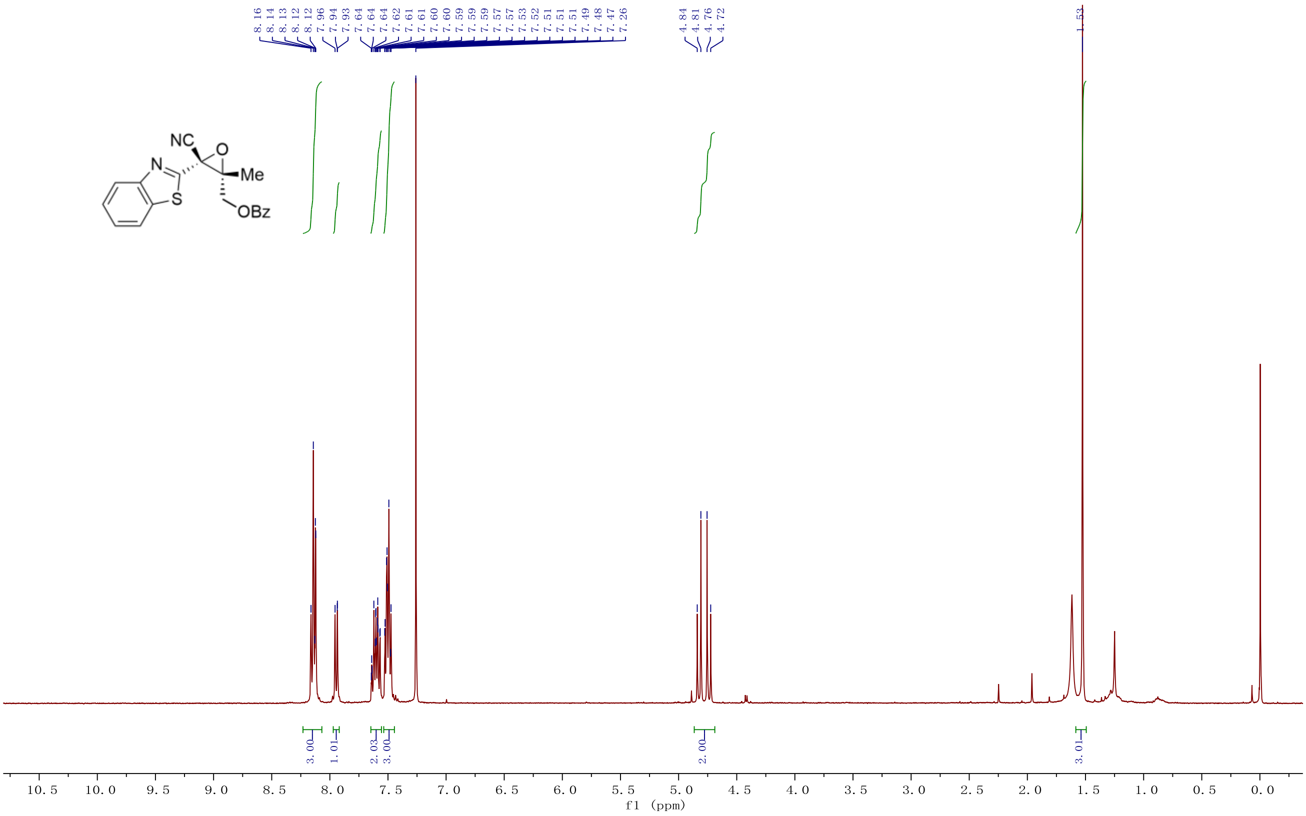

Supplementary Figure 392. ^{13}C NMR spectrum of compound *cis*-**3dg** (100 MHz, $CDCl_3$)

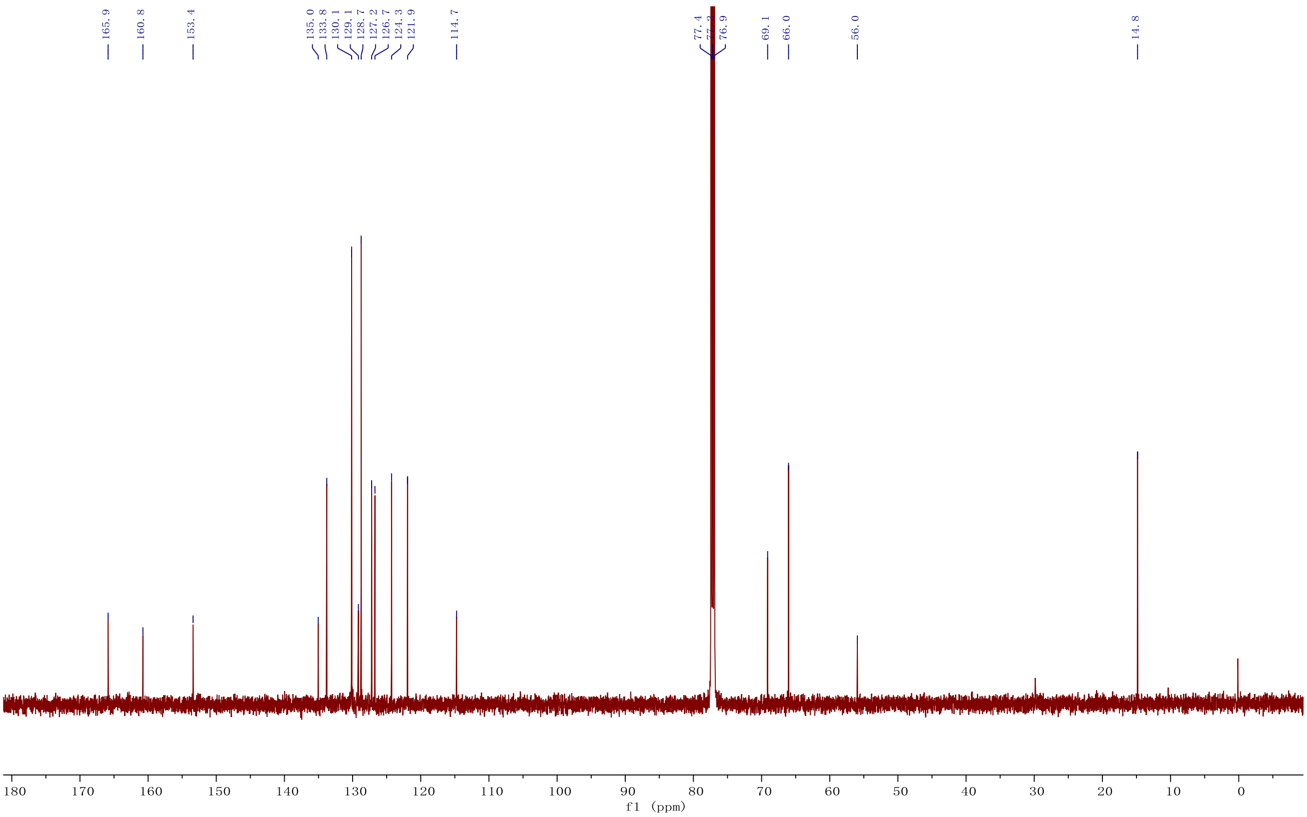

Supplementary Figure 393. ^{1}H NMR spectrum of compound **3ea** (400 MHz, $CDCl_3$)

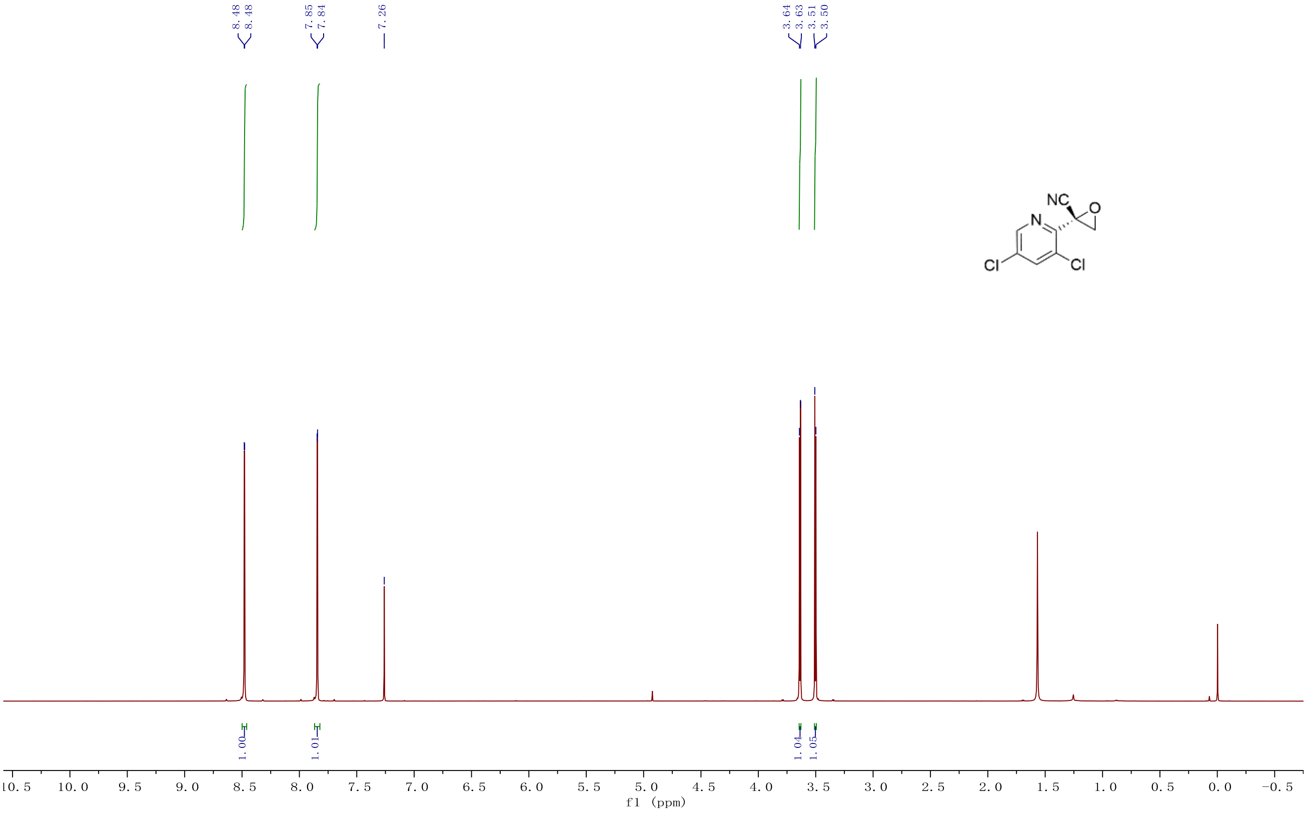

Supplementary Figure 394. ^{13}C NMR spectrum of compound **3ea** (100 MHz, $CDCl_3$)

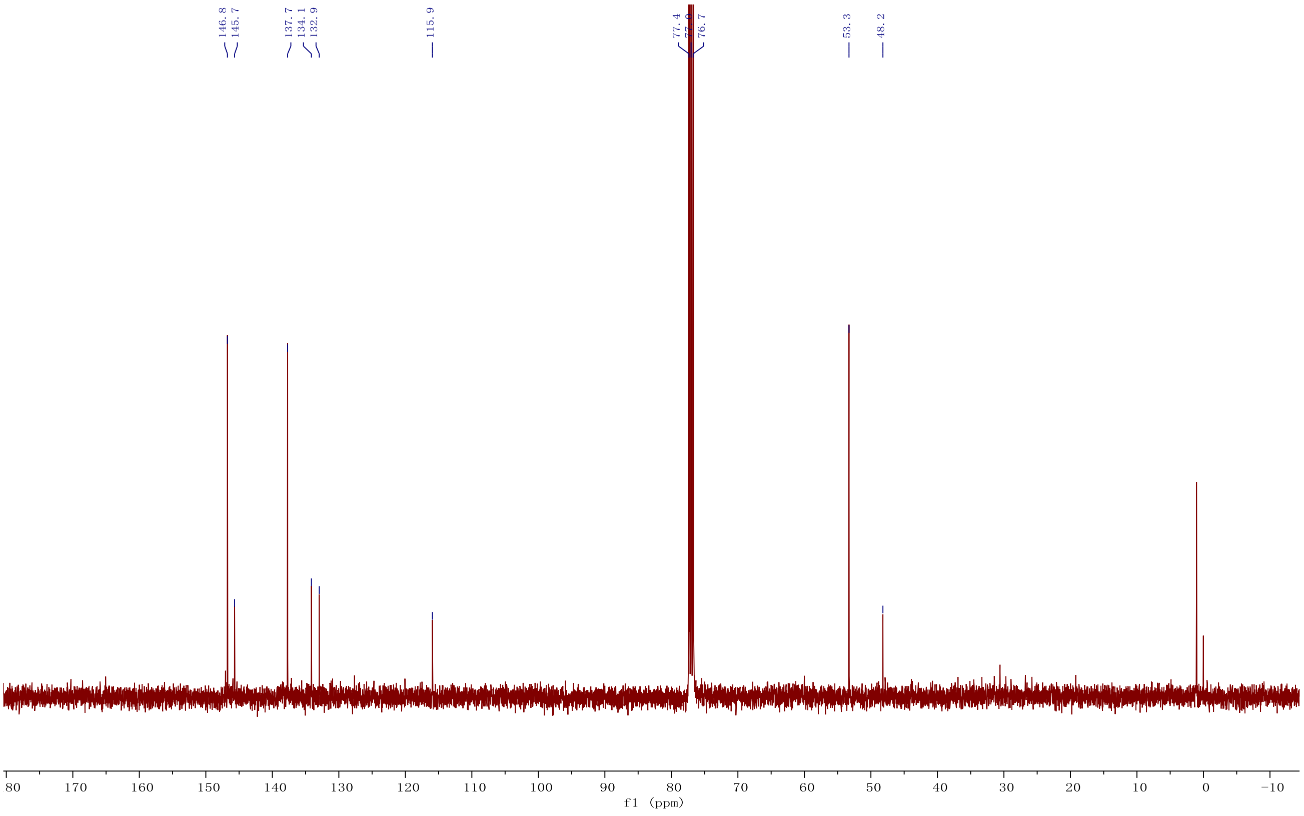

Supplementary Figure 395. ^1H NMR spectrum of compound **3eb** (600 MHz, $CDCl_3$)

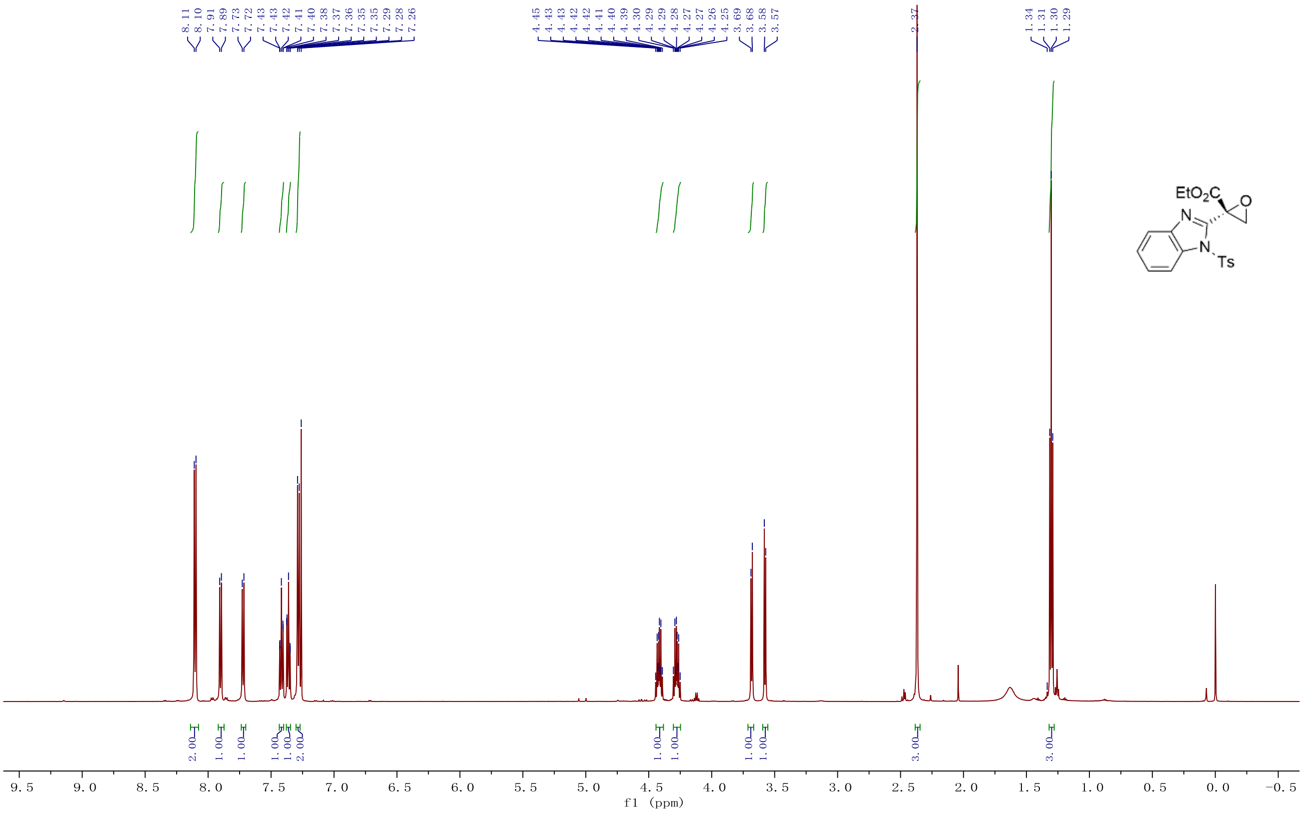

Supplementary Figure 396. ^{13}C NMR spectrum of compound **3eb** (150 MHz, $CDCl_3$)

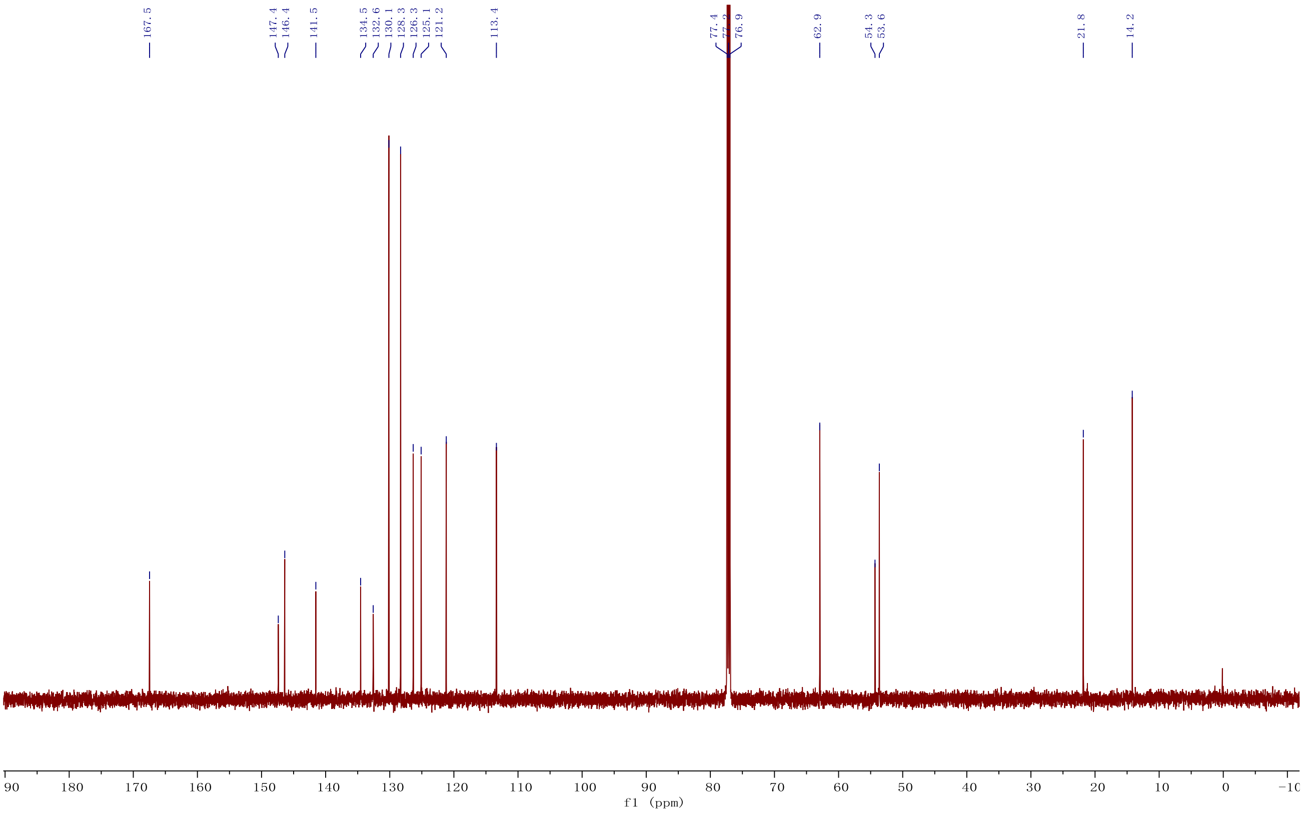

Supplementary Figure 397. ^{1}H NMR spectrum of compound **3ec** (600 MHz, $CDCl_3$)

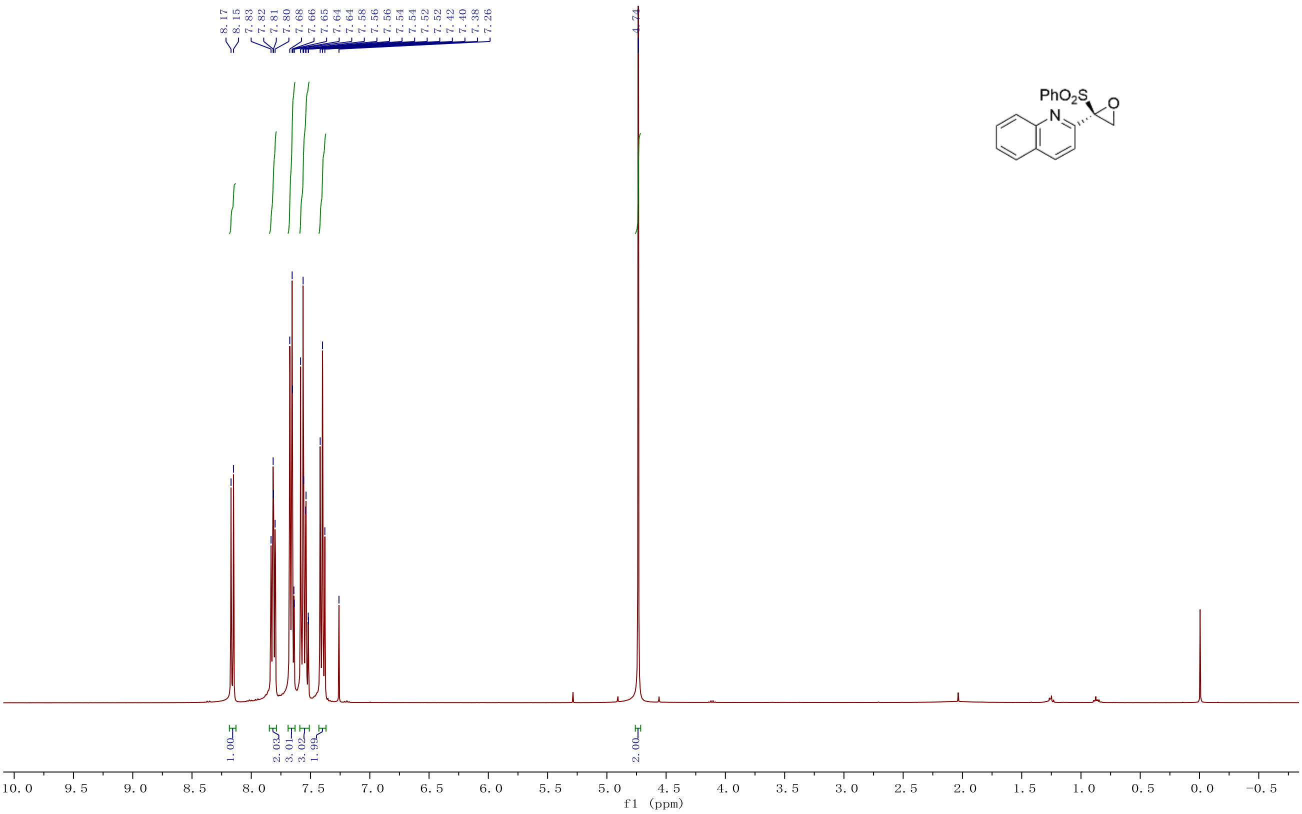

Supplementary Figure 398. ^{13}C NMR spectrum of compound **3ec** (150 MHz, $CDCl_3$)

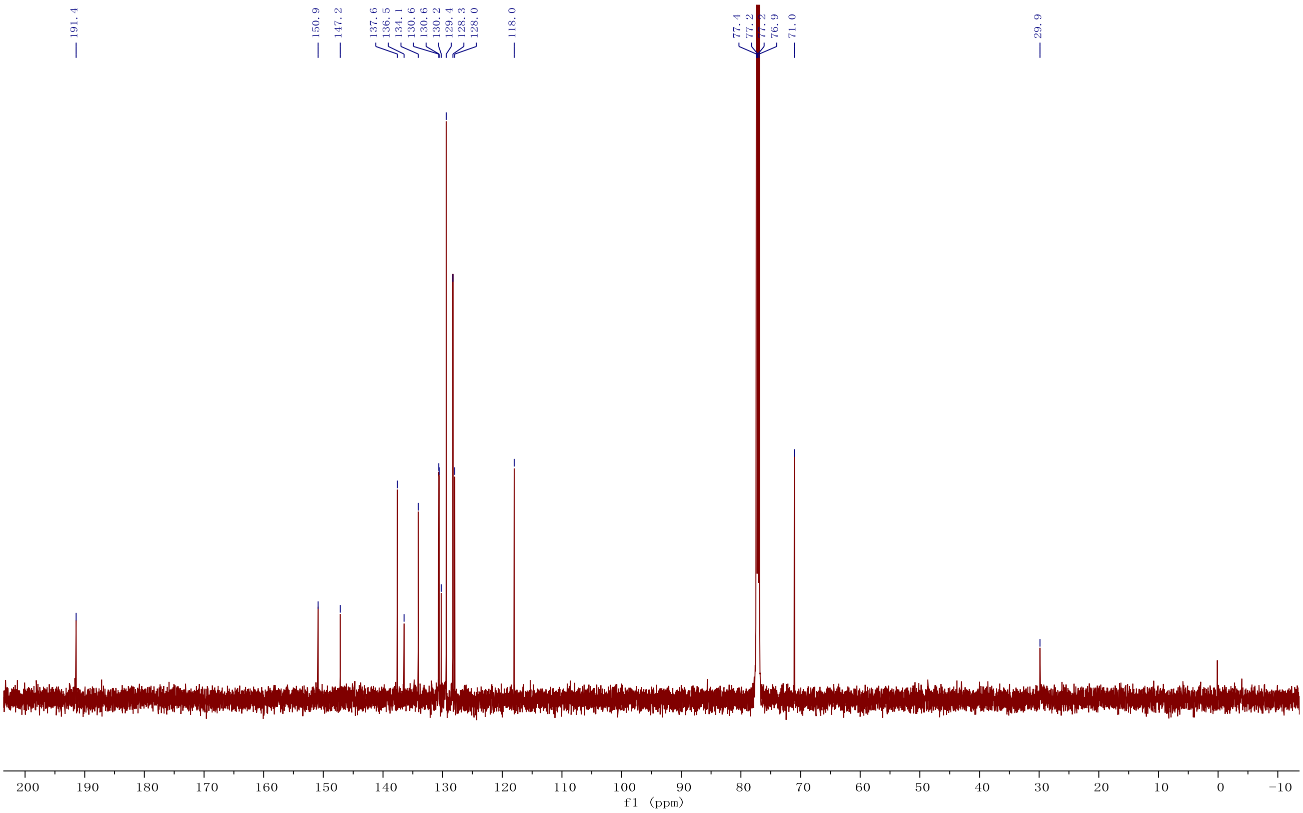

Supplementary Figure 399. ^{1}H NMR spectrum of compound **3fa** (400 MHz, $CDCl_3$)

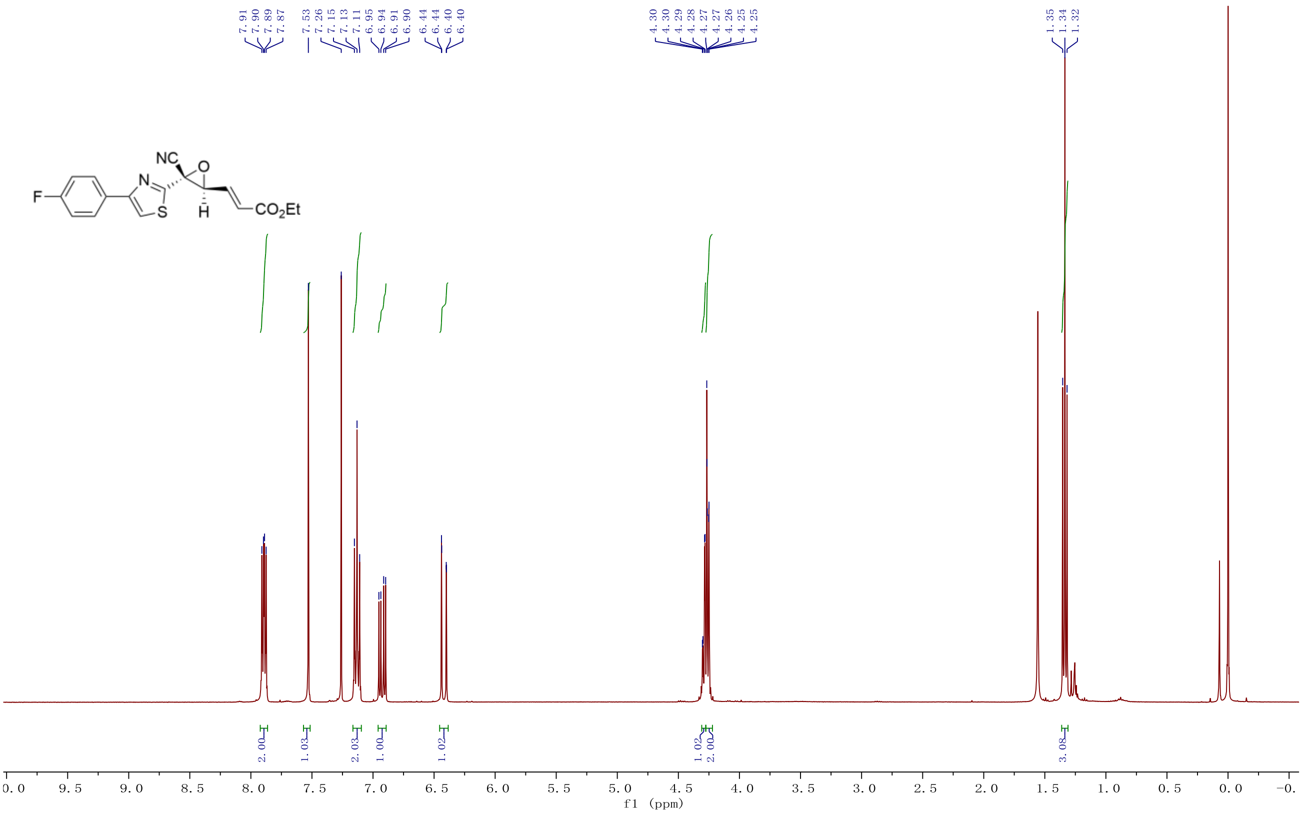

Supplementary Figure 400. ^{13}C NMR spectrum of compound **3fa** (100 MHz, $CDCl_3$)

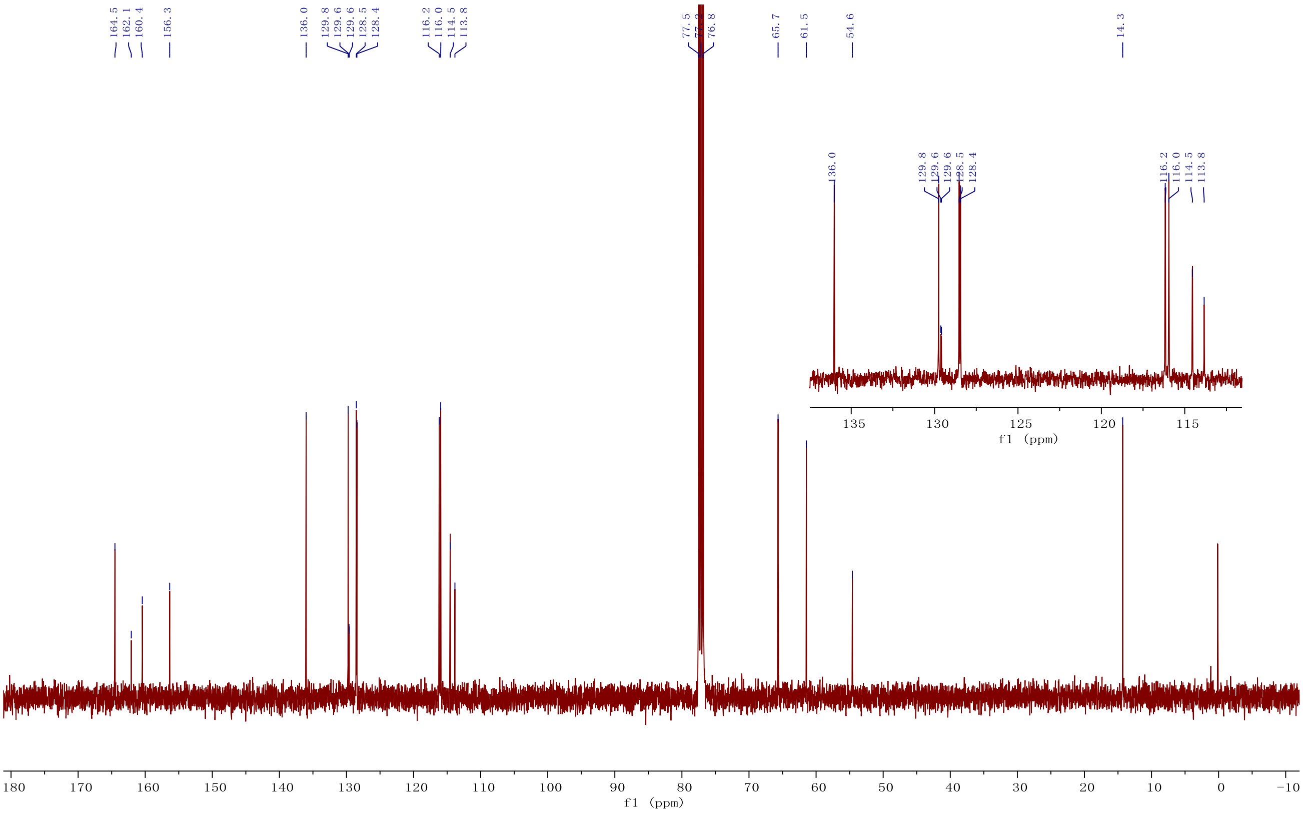

Supplementary Figure 401. ^{19}F NMR spectrum of compound **3fa** (376 MHz, $CDCl_3$)

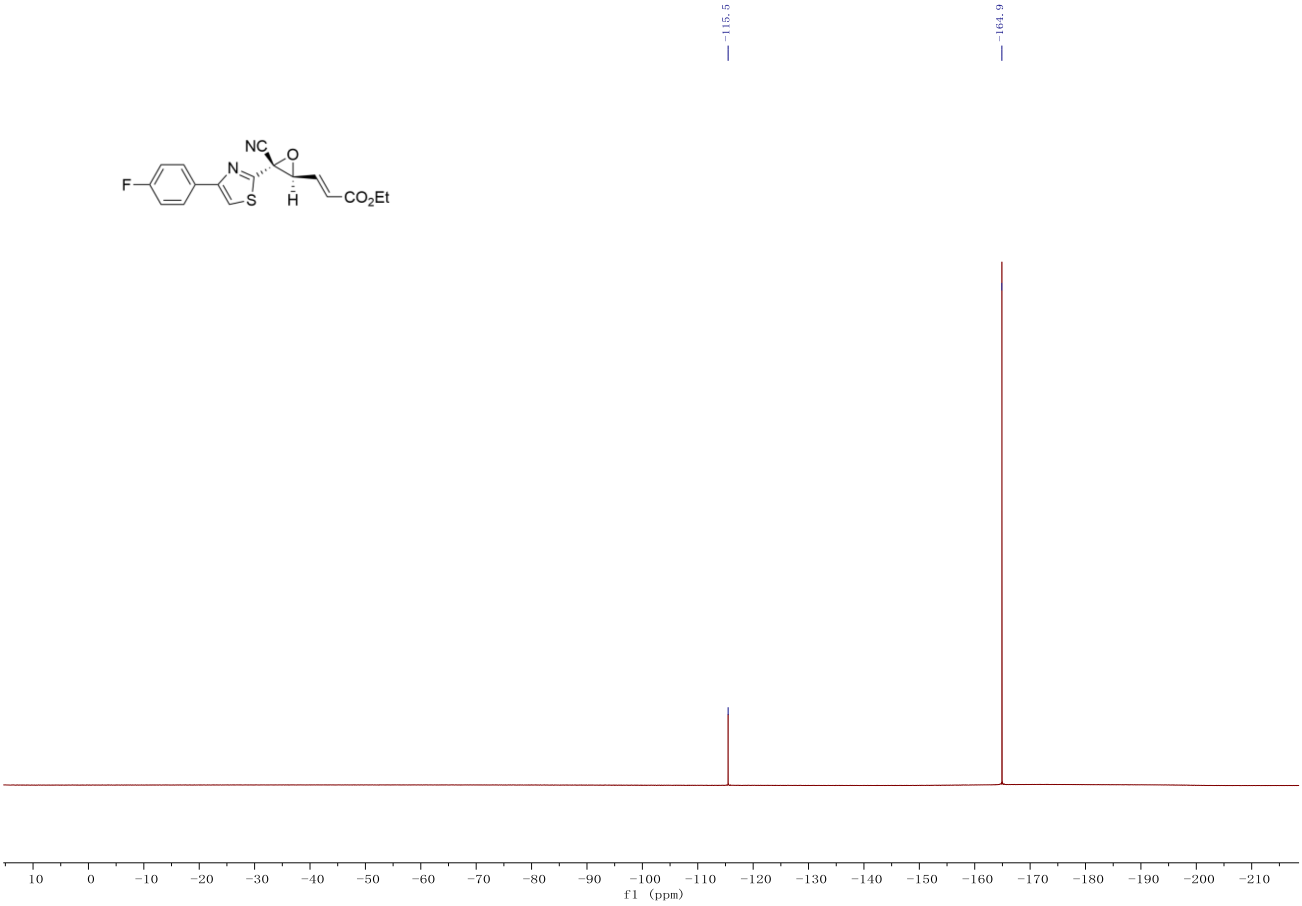

Supplementary Figure 402. ^{1}H NMR spectrum of compound **3fb** (400 MHz, $CDCl_3$)

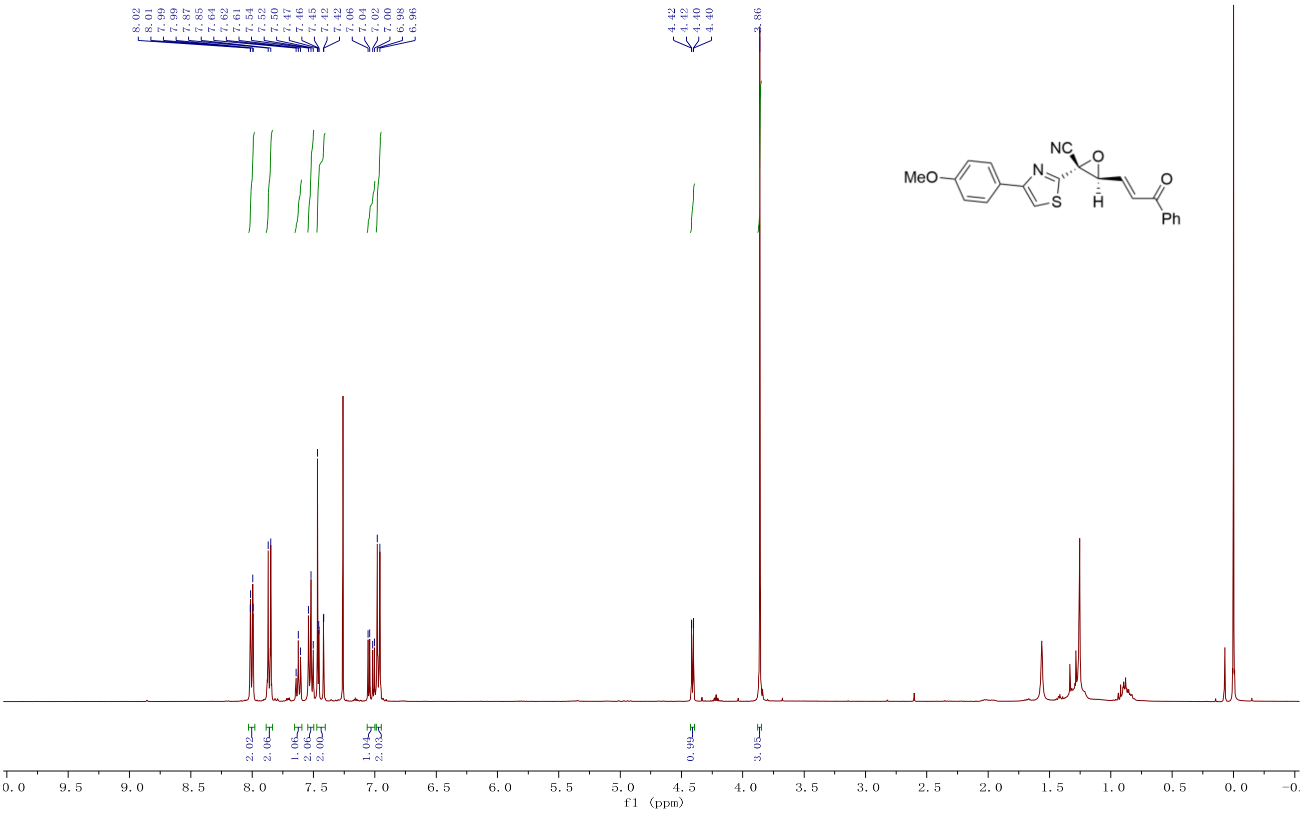

Supplementary Figure 403. ^{13}C NMR spectrum of compound **3fb** (100 MHz, $CDCl_3$)

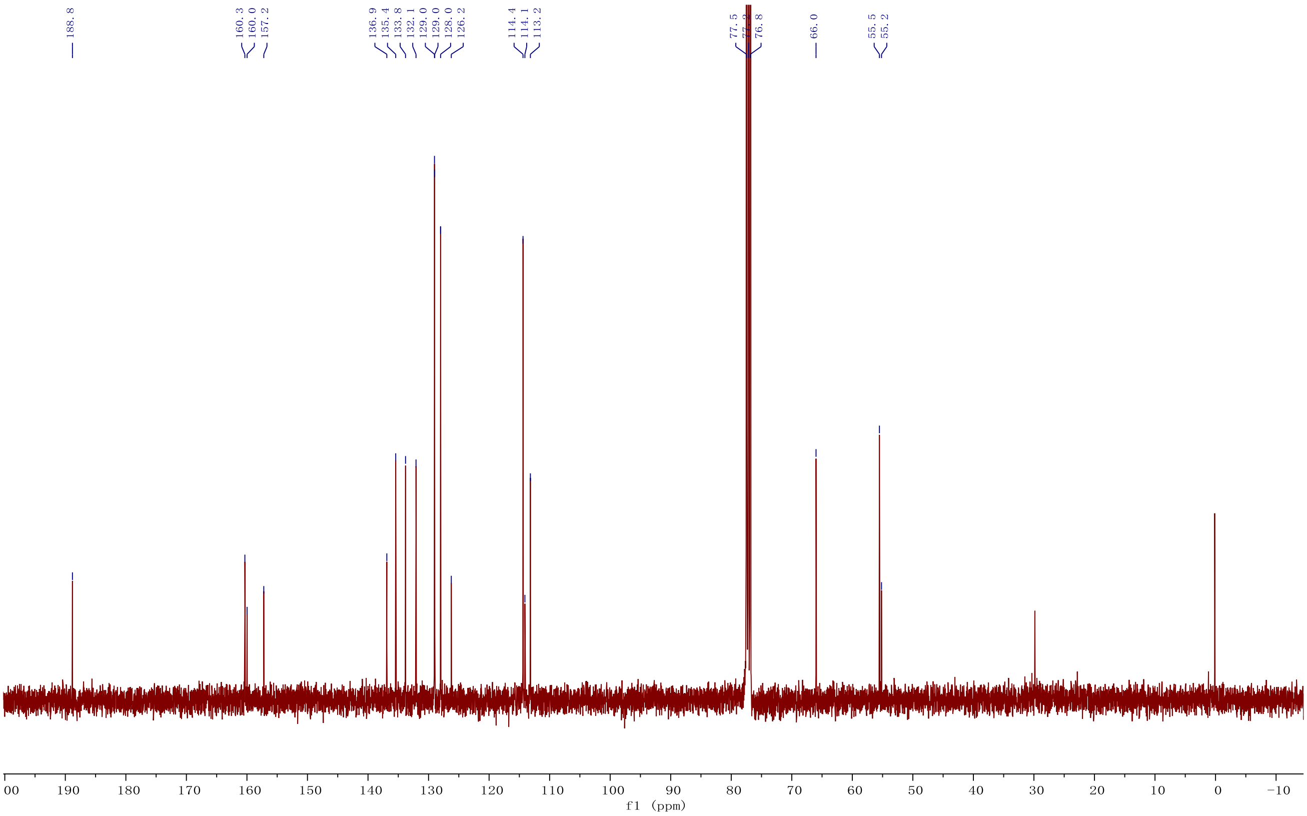

Supplementary Figure 404. ^{1}H NMR spectrum of compound **3fc** (400 MHz, $CDCl_3$)

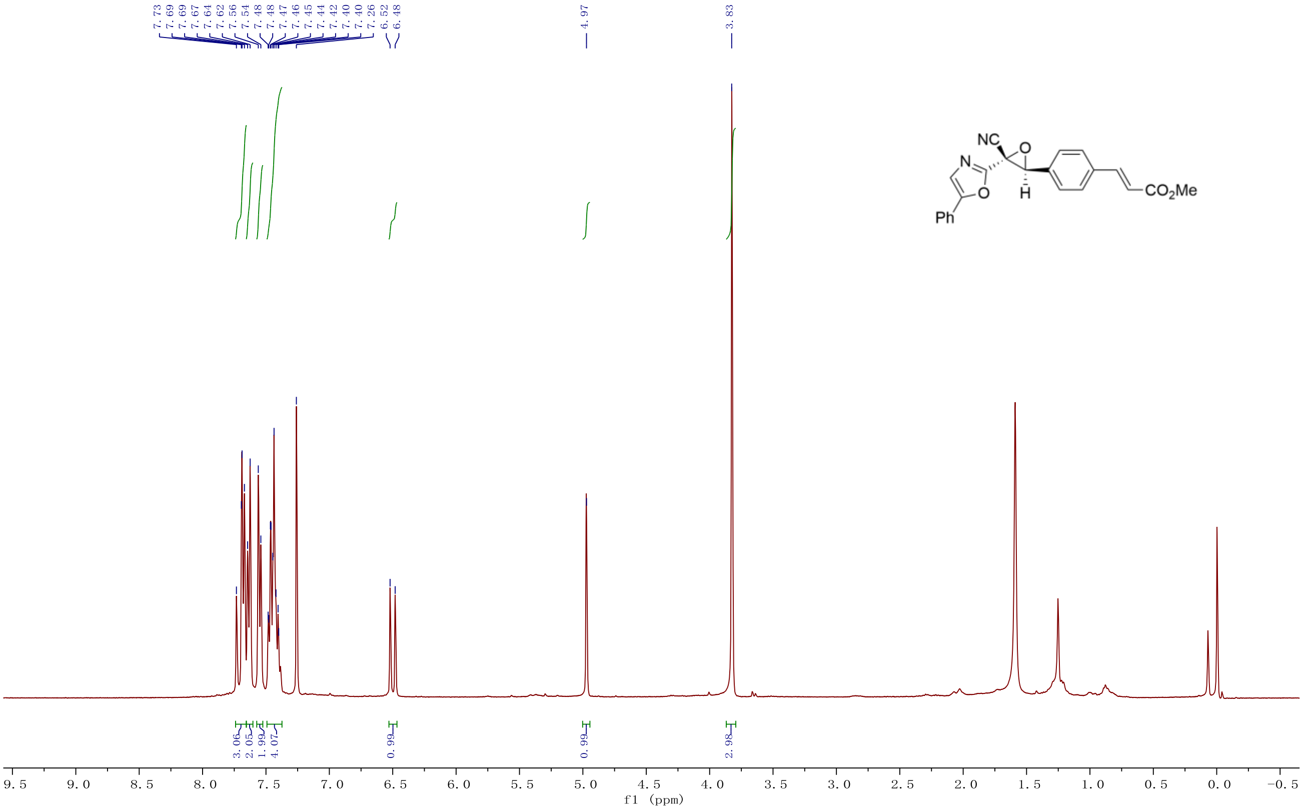

Supplementary Figure 405. ^{13}C NMR spectrum of compound **3fc** (100 MHz, $CDCl_3$)

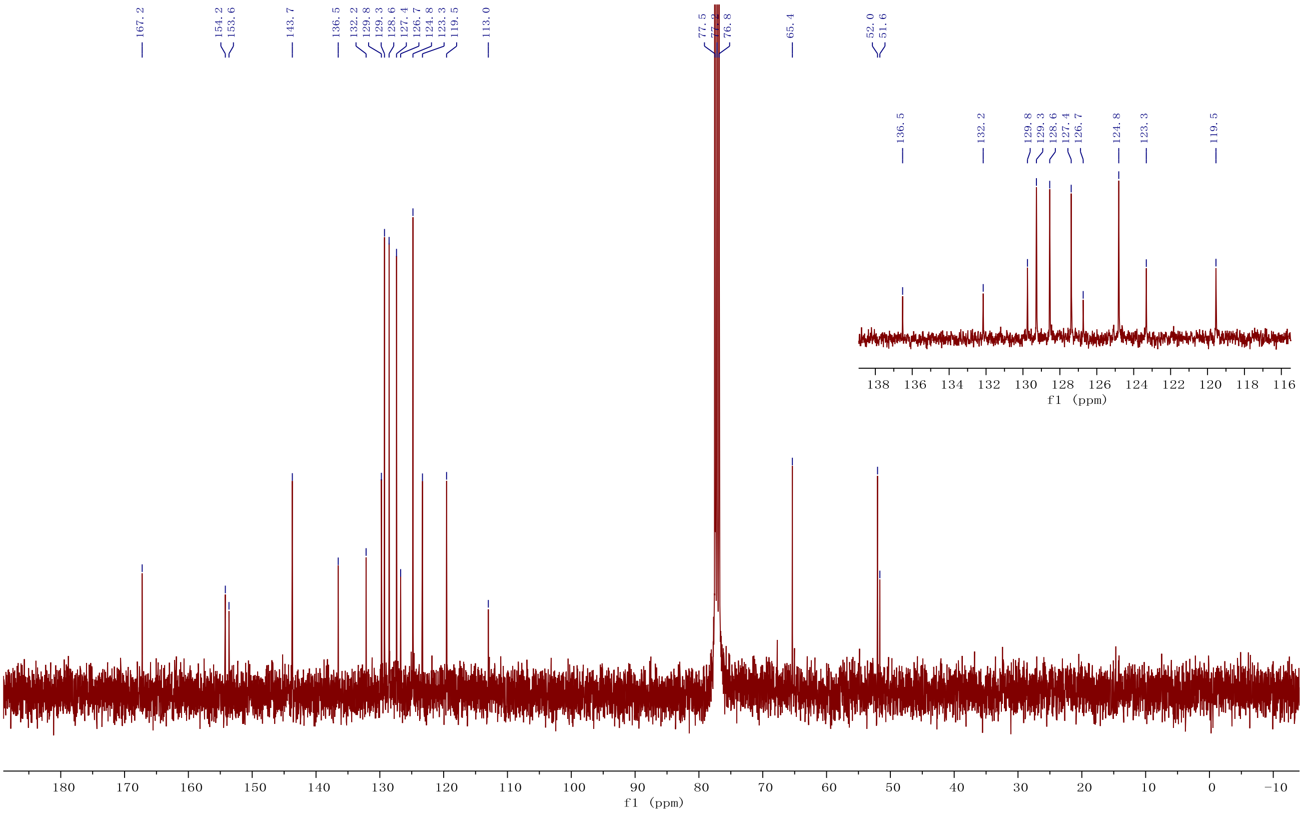

Supplementary Figure 406. ^{1}H NMR spectrum of compound **3fd** (400 MHz, $CDCl_3$)

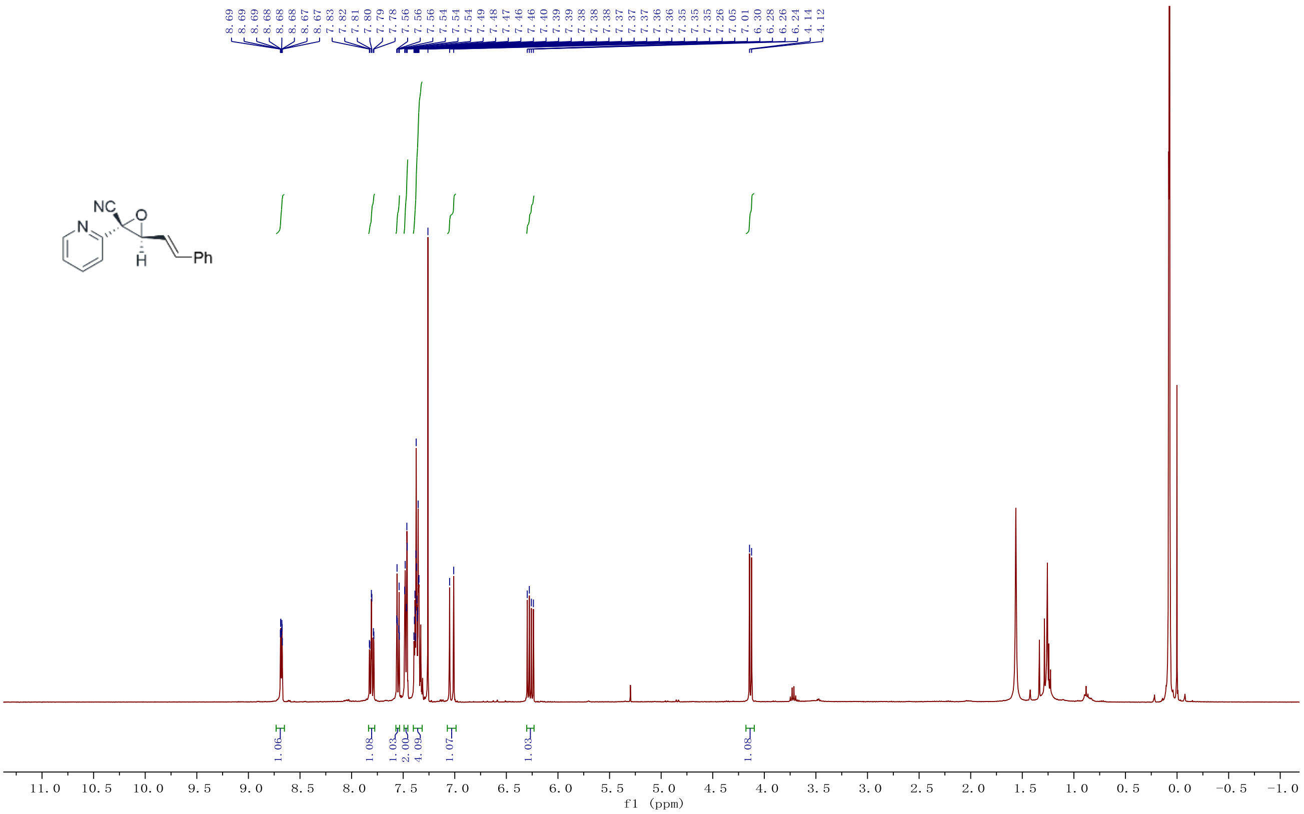

Supplementary Figure 407. ^{13}C NMR spectrum of compound **3fd** (100 MHz, $CDCl_3$)

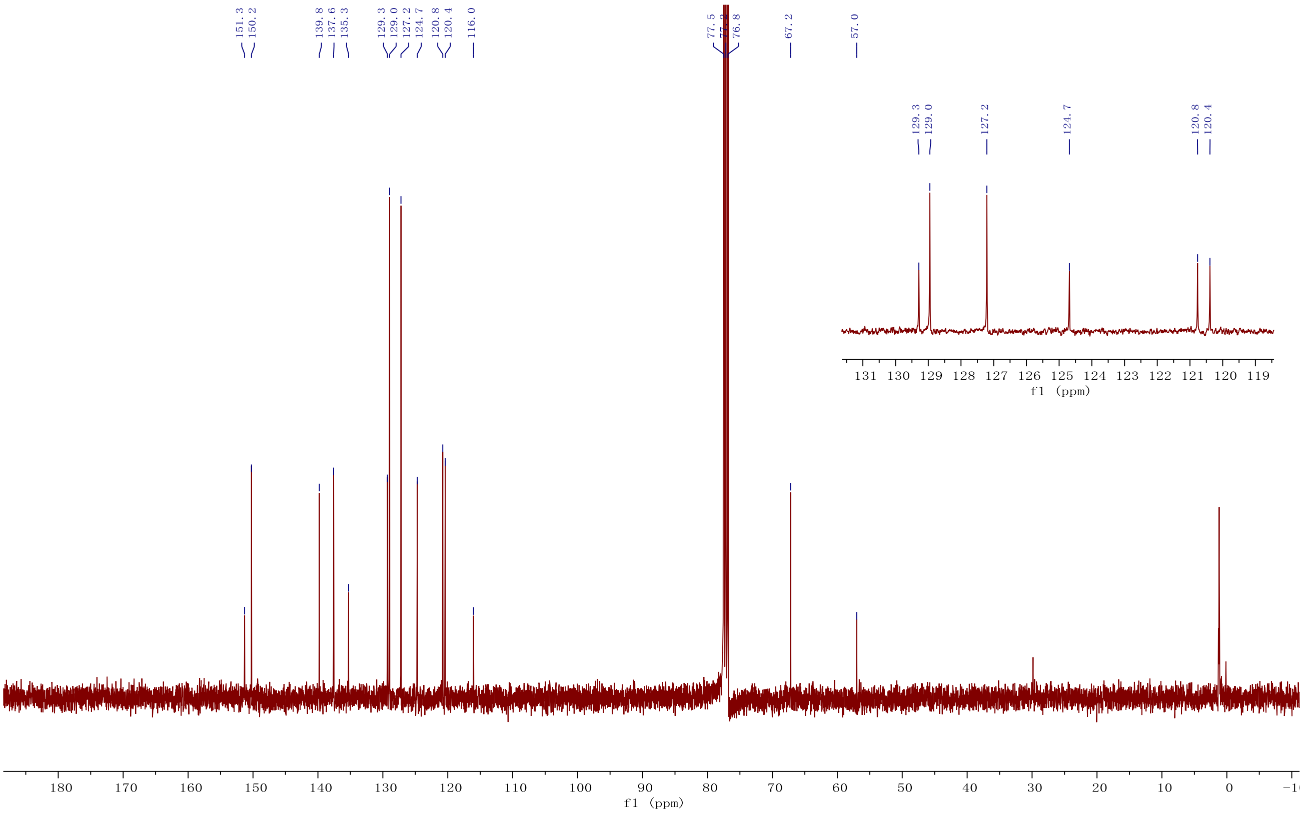

Supplementary Figure 408. ^{1}H NMR spectrum of compound **3fe** (400 MHz, DMSO-d_6)

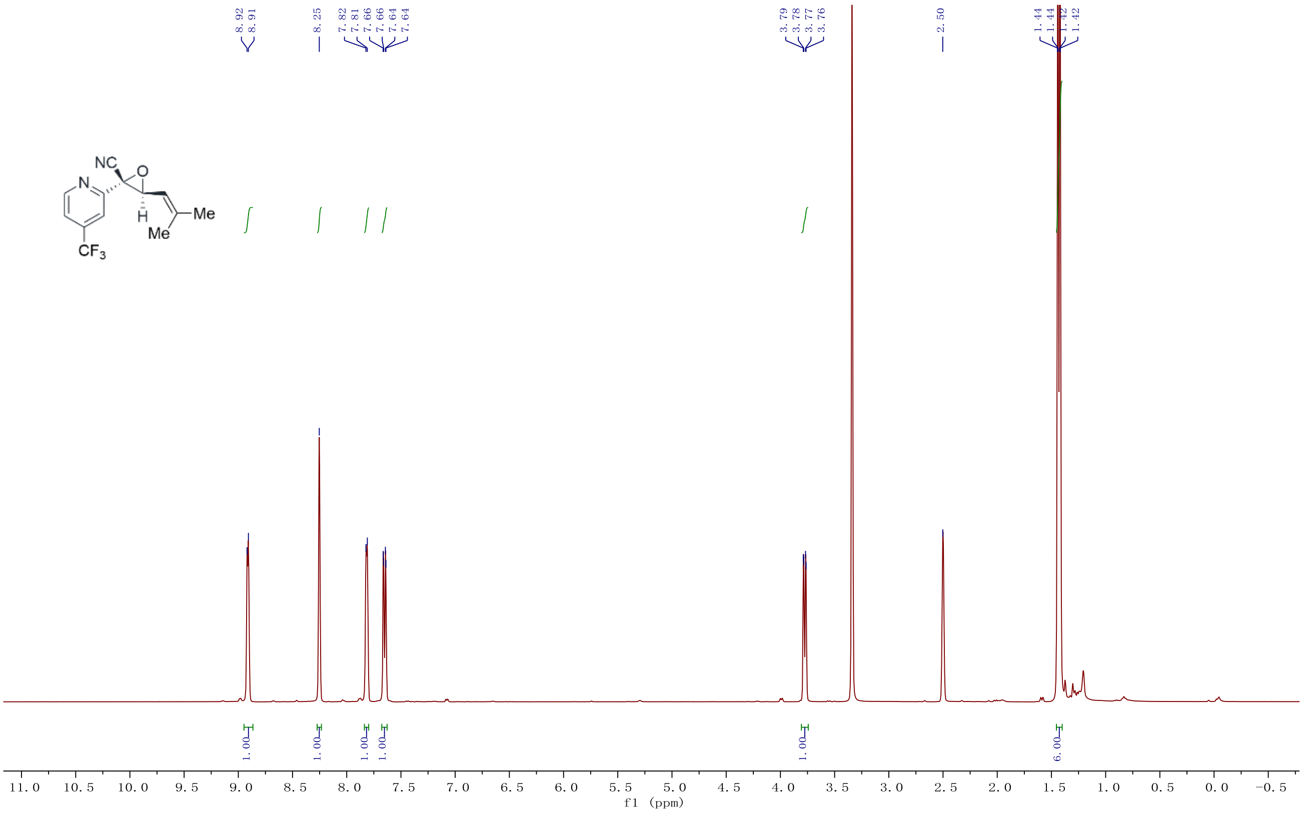

Supplementary Figure 409. ^{13}C NMR spectrum of compound **3fe** (100 MHz, DMSO-d_6)

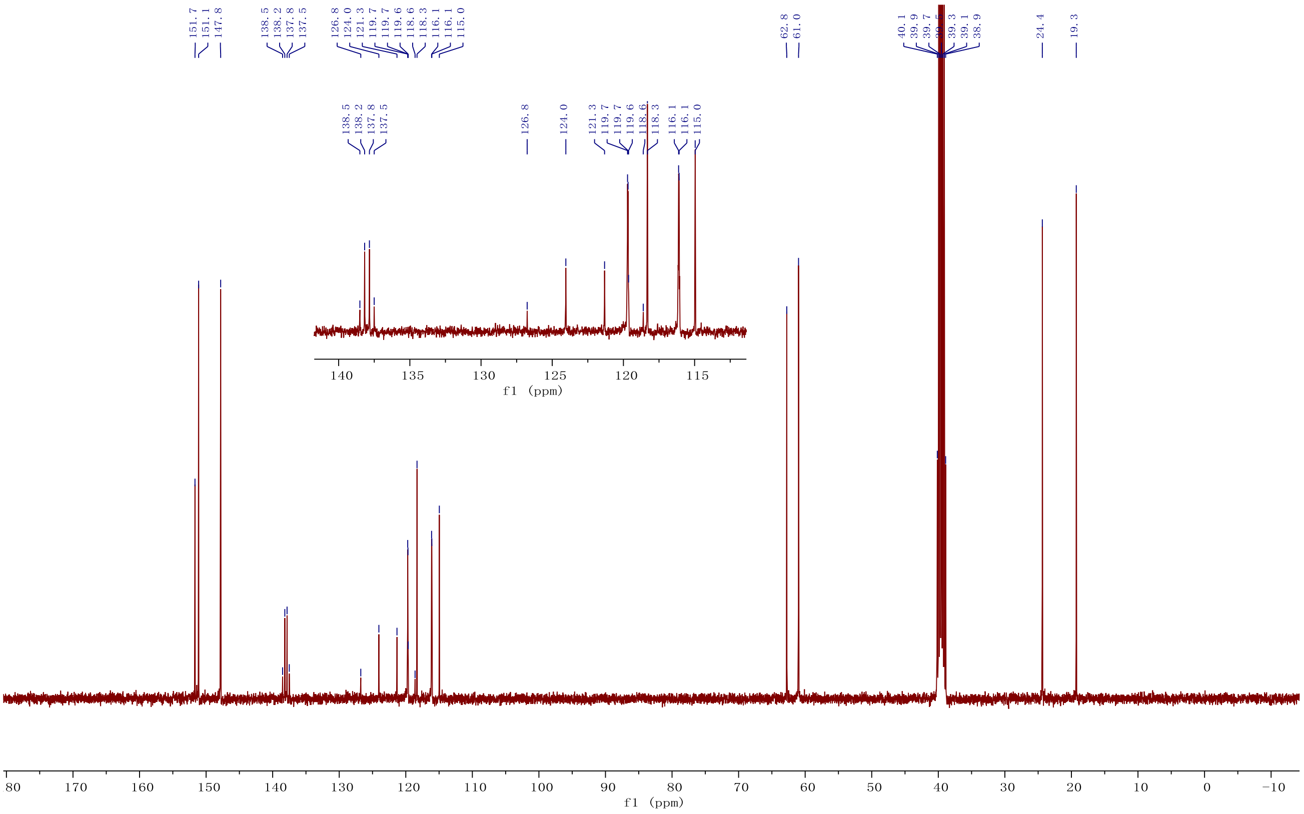

Supplementary Figure 410. ^{19}F NMR spectrum of compound **3fe** (376 MHz, DMSO-d_6)

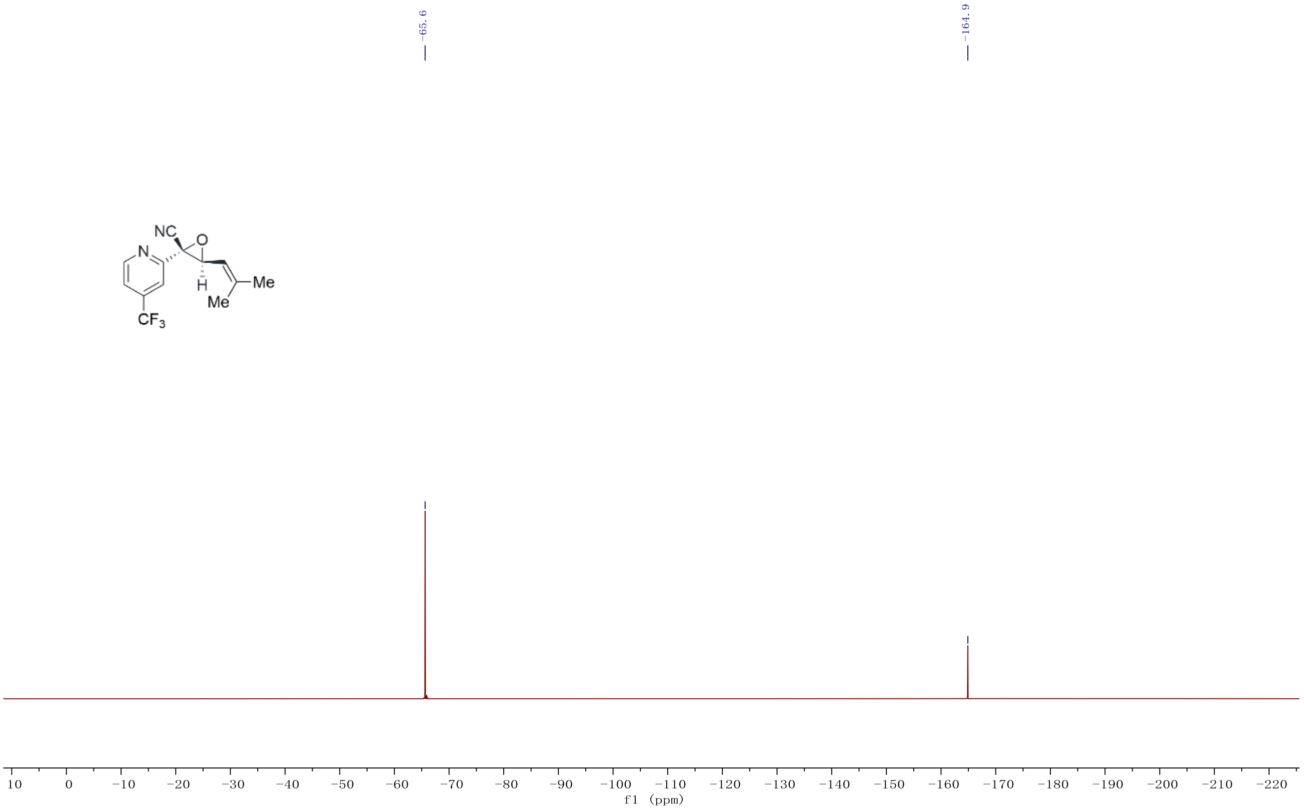

Supplementary Figure 411. ^{1}H NMR spectrum of compound **3ff** (400 MHz, $CDCl_3$)

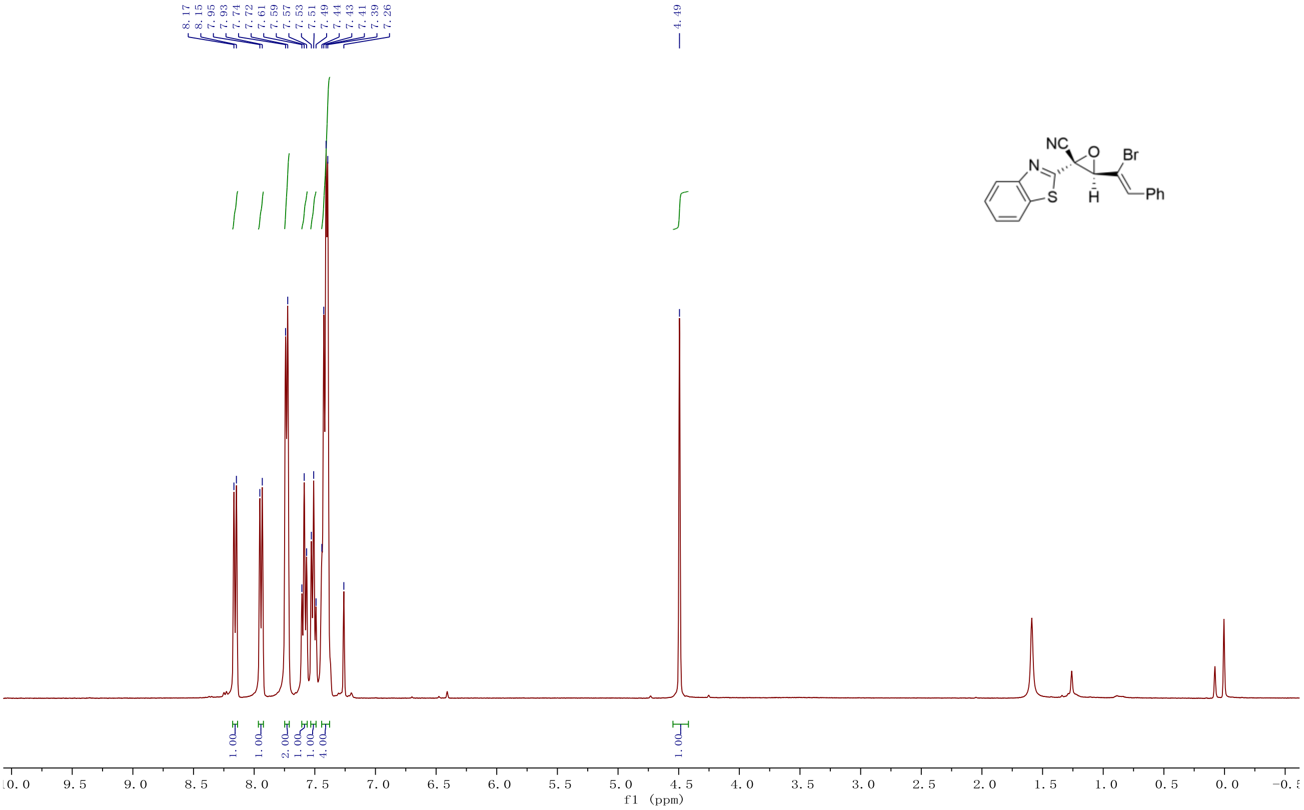

Supplementary Figure 412. ^{13}C NMR spectrum of compound **3ff** (100 MHz, $CDCl_3$)

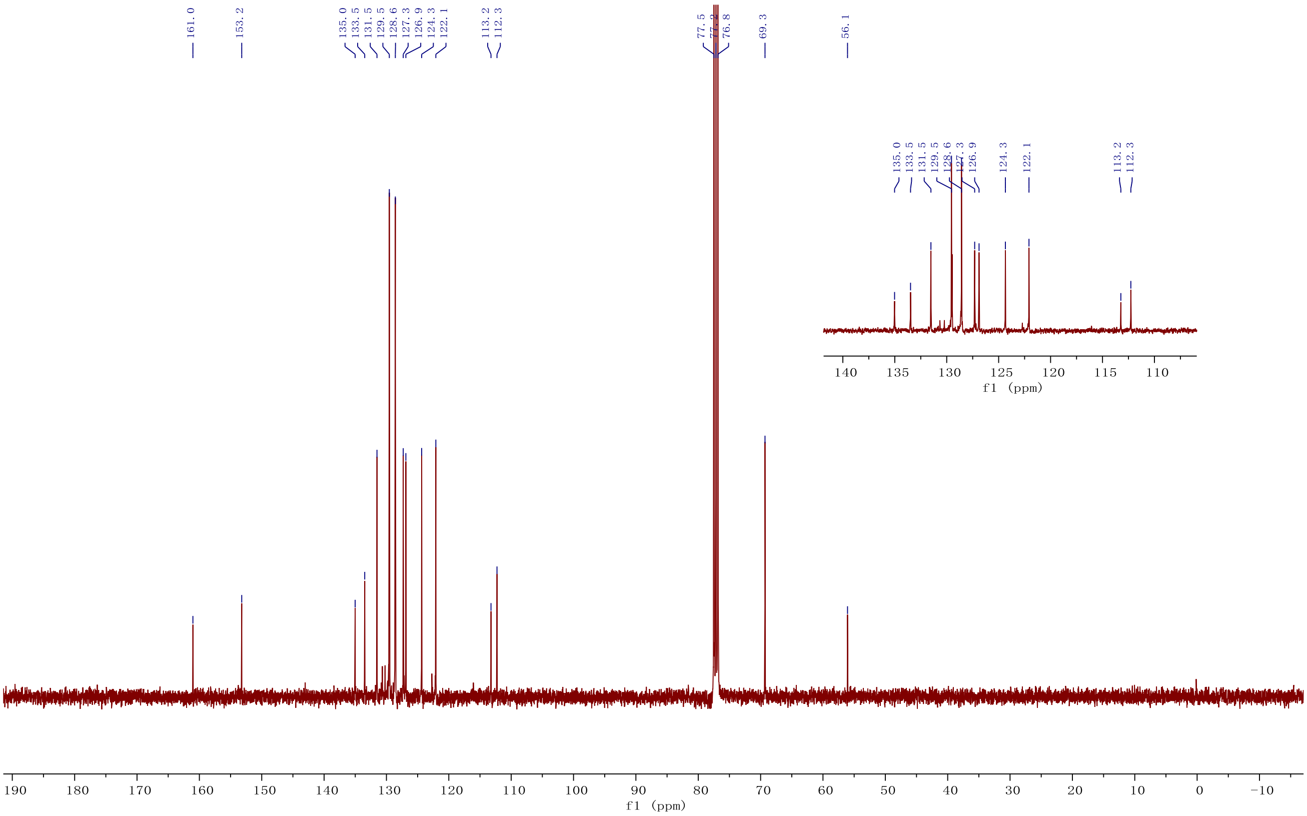

Supplementary Figure 413. ^{1}H NMR spectrum of compound **3fg** (400 MHz, $CDCl_3$)

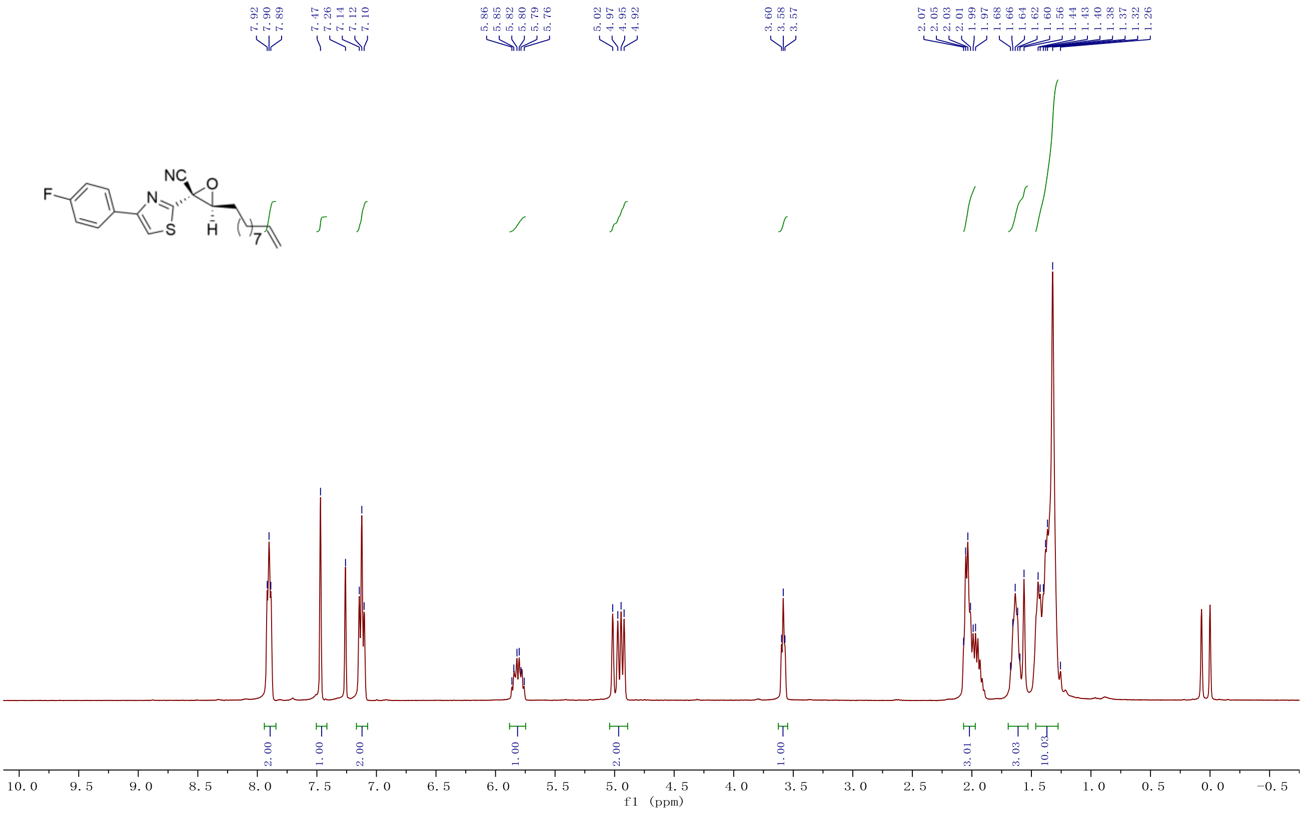

Supplementary Figure 414. ^{13}C NMR spectrum of compound **3fg** (100 MHz, $CDCl_3$)

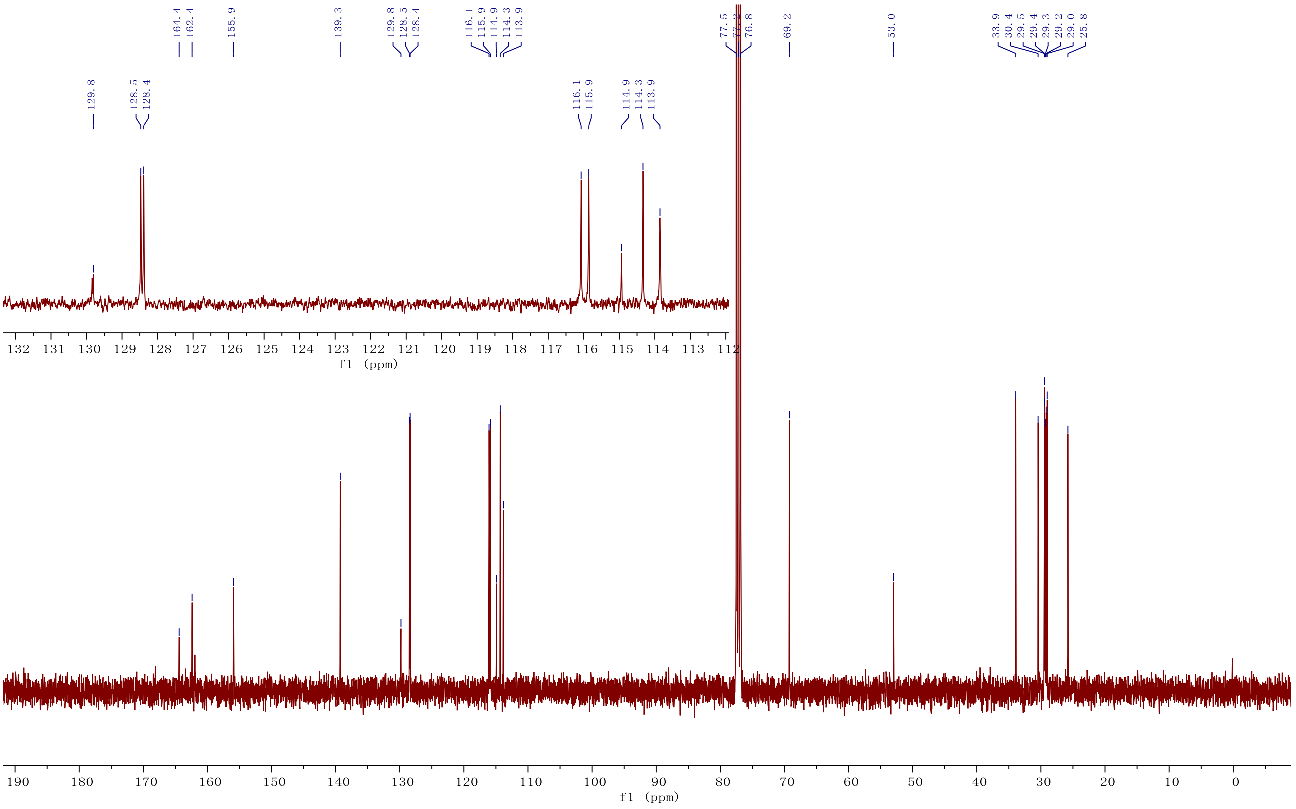

Supplementary Figure 415. ^{19}F NMR spectrum of compound **3fg** (376 MHz, $CDCl_3$)

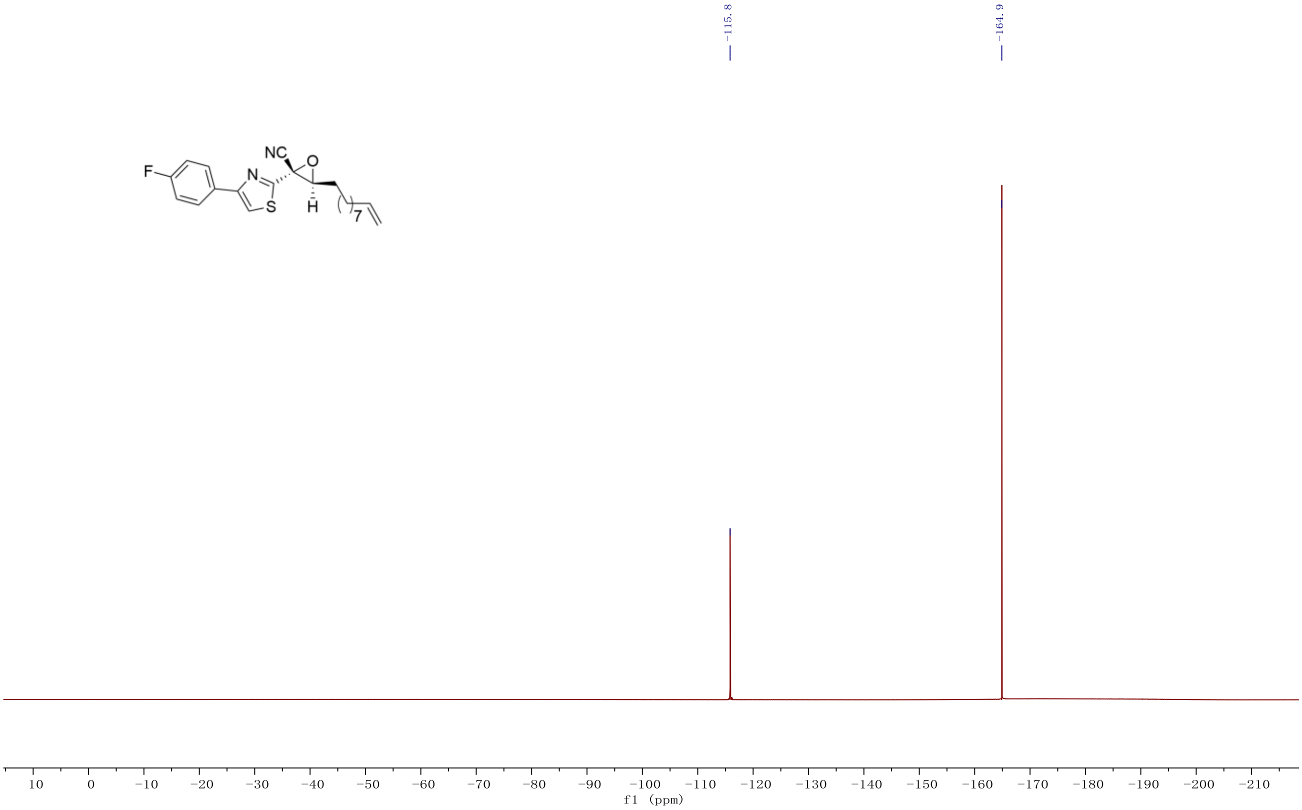

Supplementary Figure 416. ^{1}H NMR spectrum of compound **3fh** (400 MHz, $CDCl_3$)

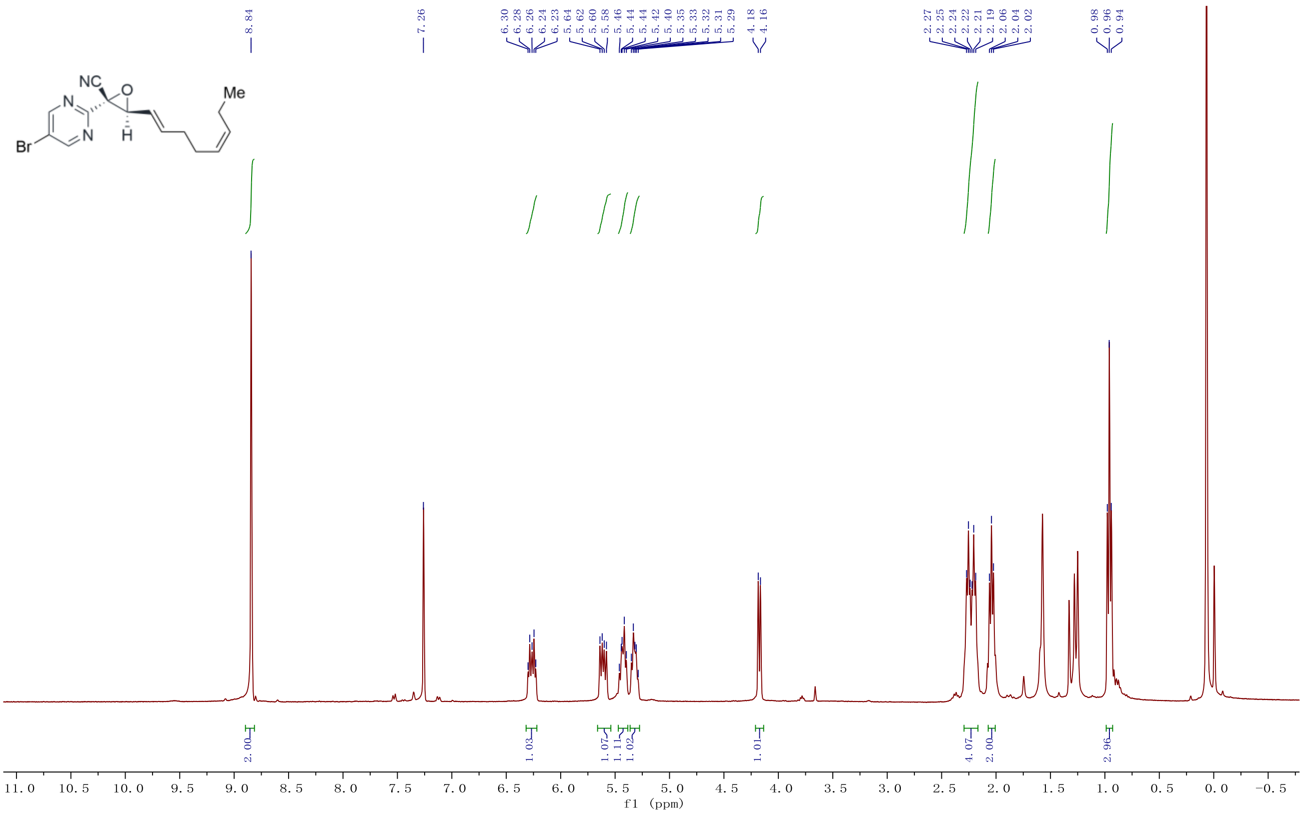

Supplementary Figure 417. ^{13}C NMR spectrum of compound **3fh** (100 MHz, $CDCl_3$)

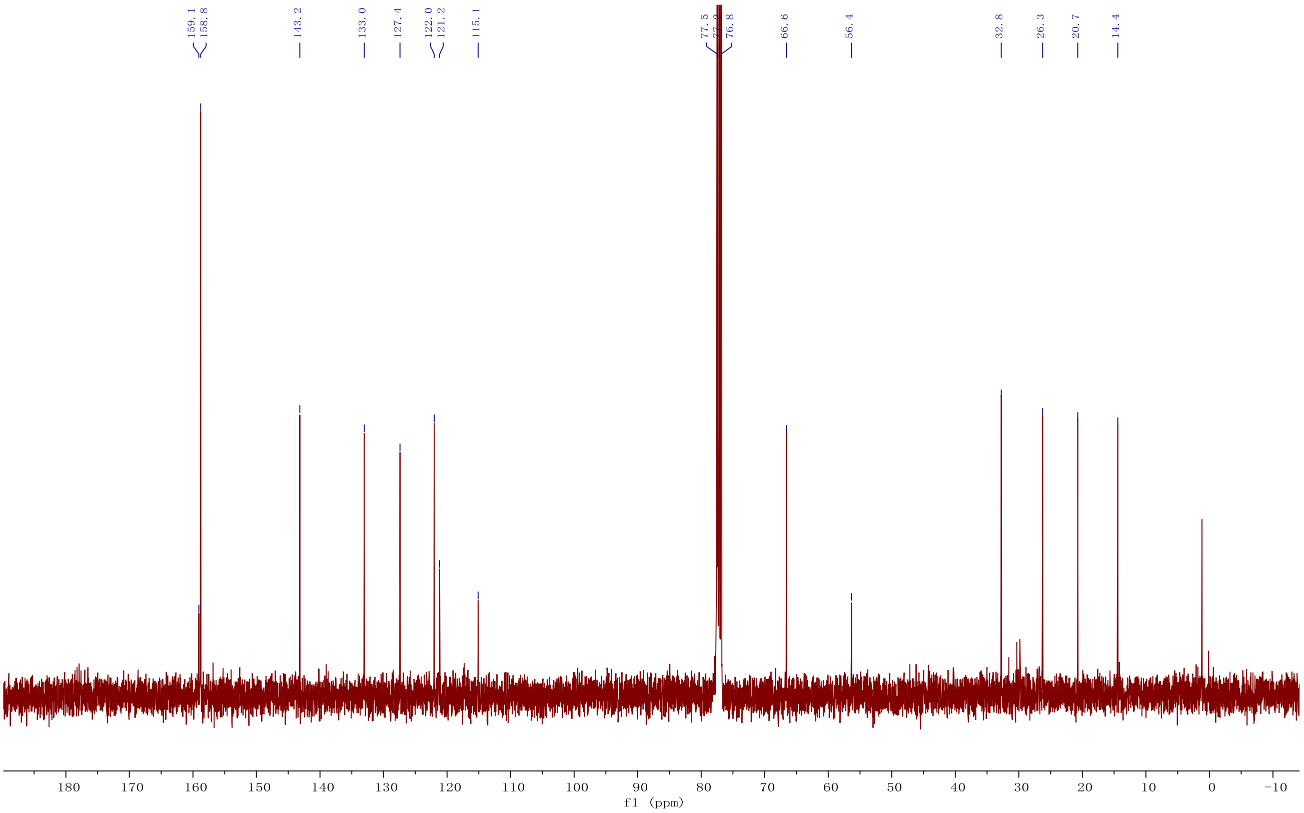

Supplementary Figure 418. ^{1}H NMR spectrum of compound **3fi** (400 MHz, $CDCl_3$)

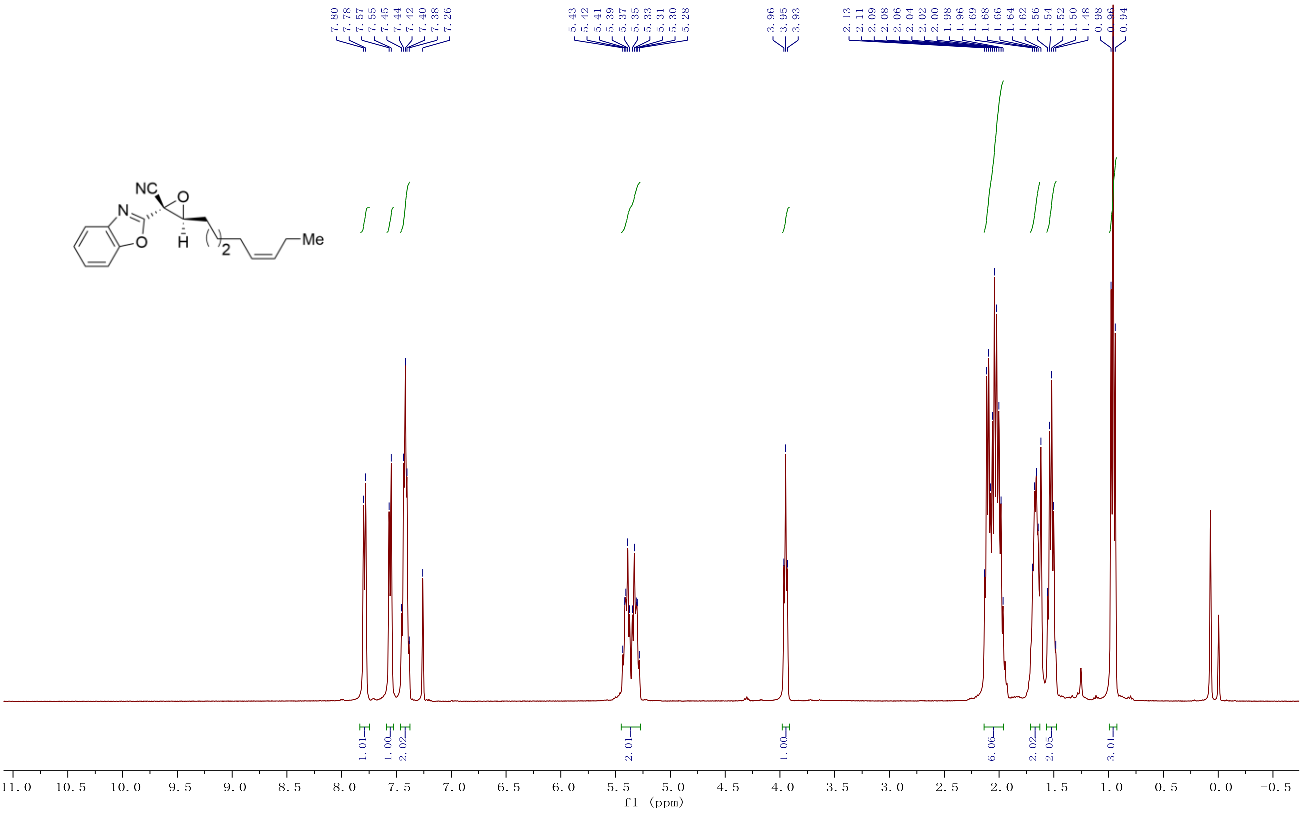

Supplementary Figure 419. ^{13}C NMR spectrum of compound **3fi** (100 MHz, $CDCl_3$)

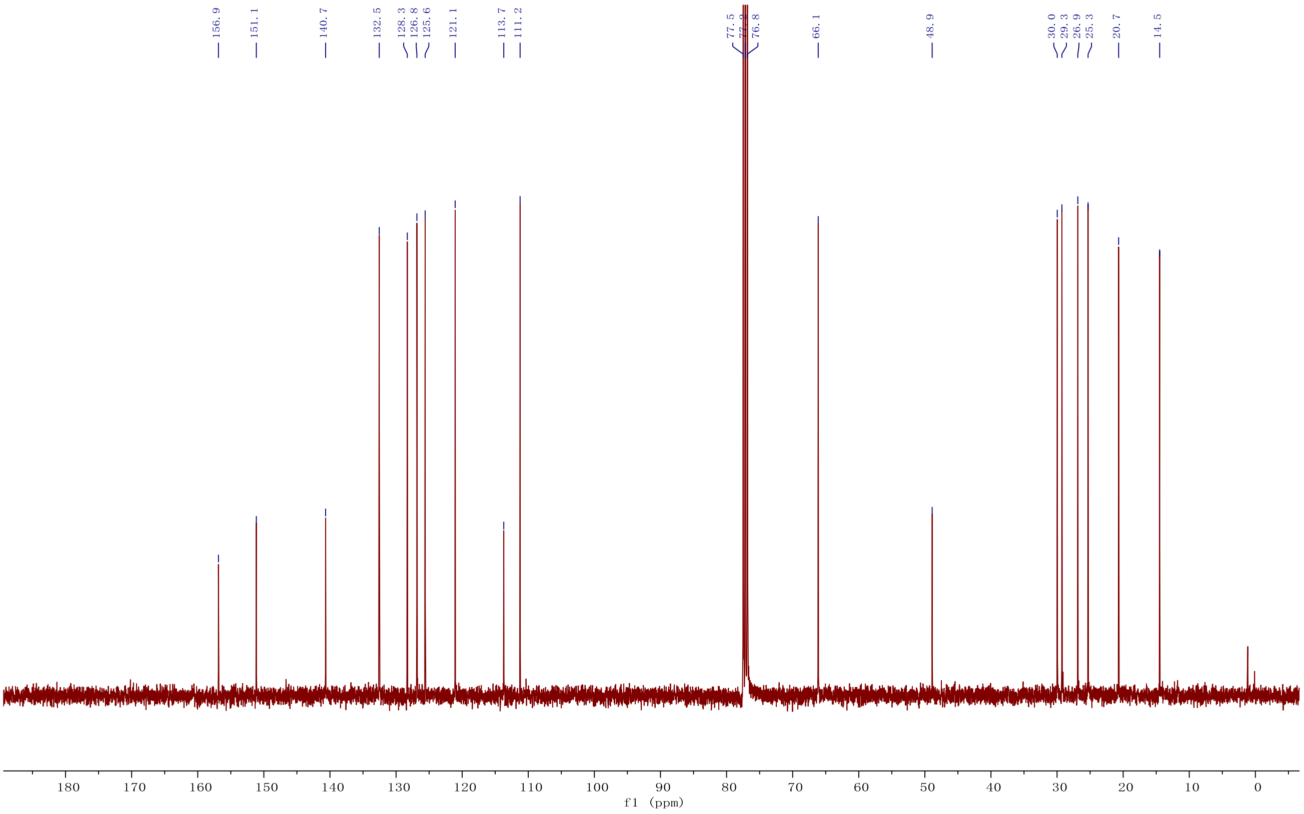

Supplementary Figure 420. ^{1}H NMR spectrum of compound **3ga** (400 MHz, $CDCl_3$)

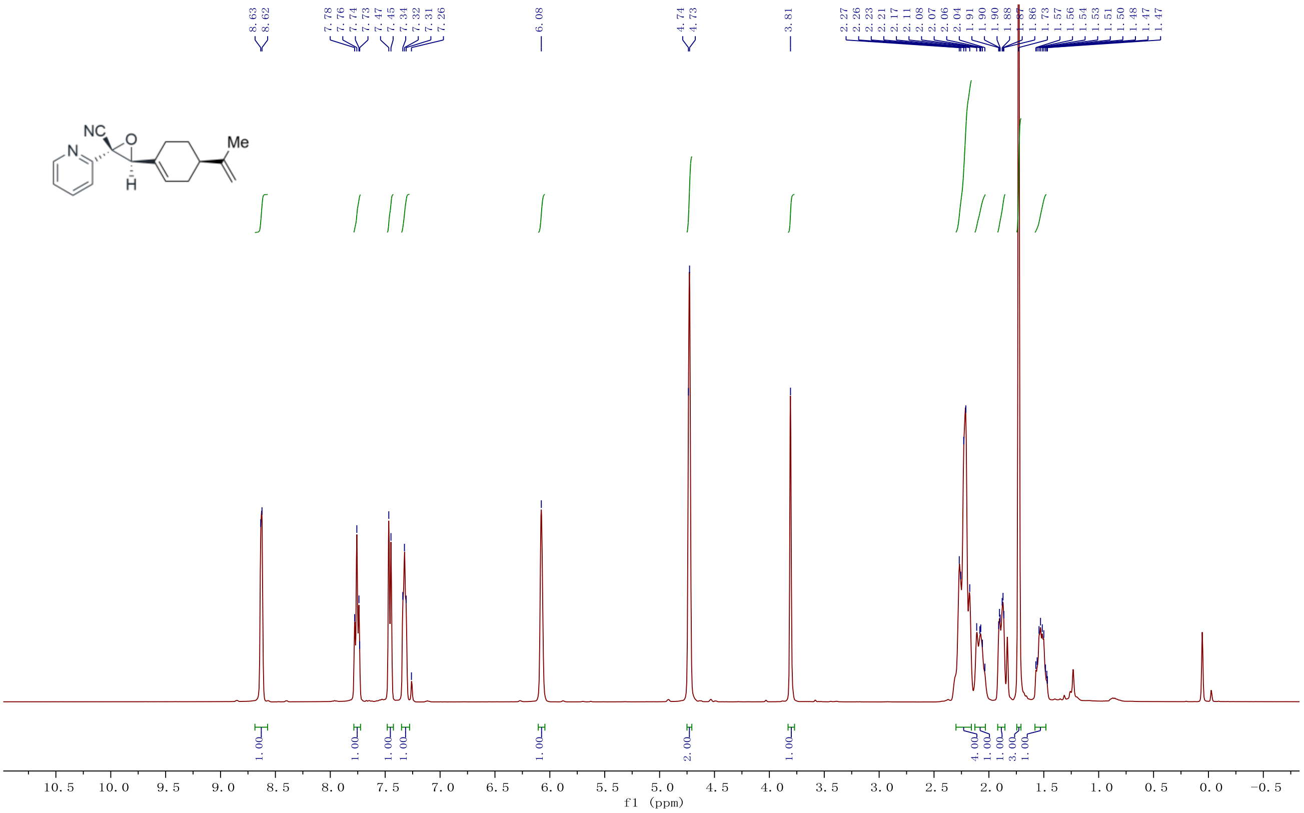

Supplementary Figure 421. ^{13}C NMR spectrum of compound **3ga** (100 MHz, $CDCl_3$)

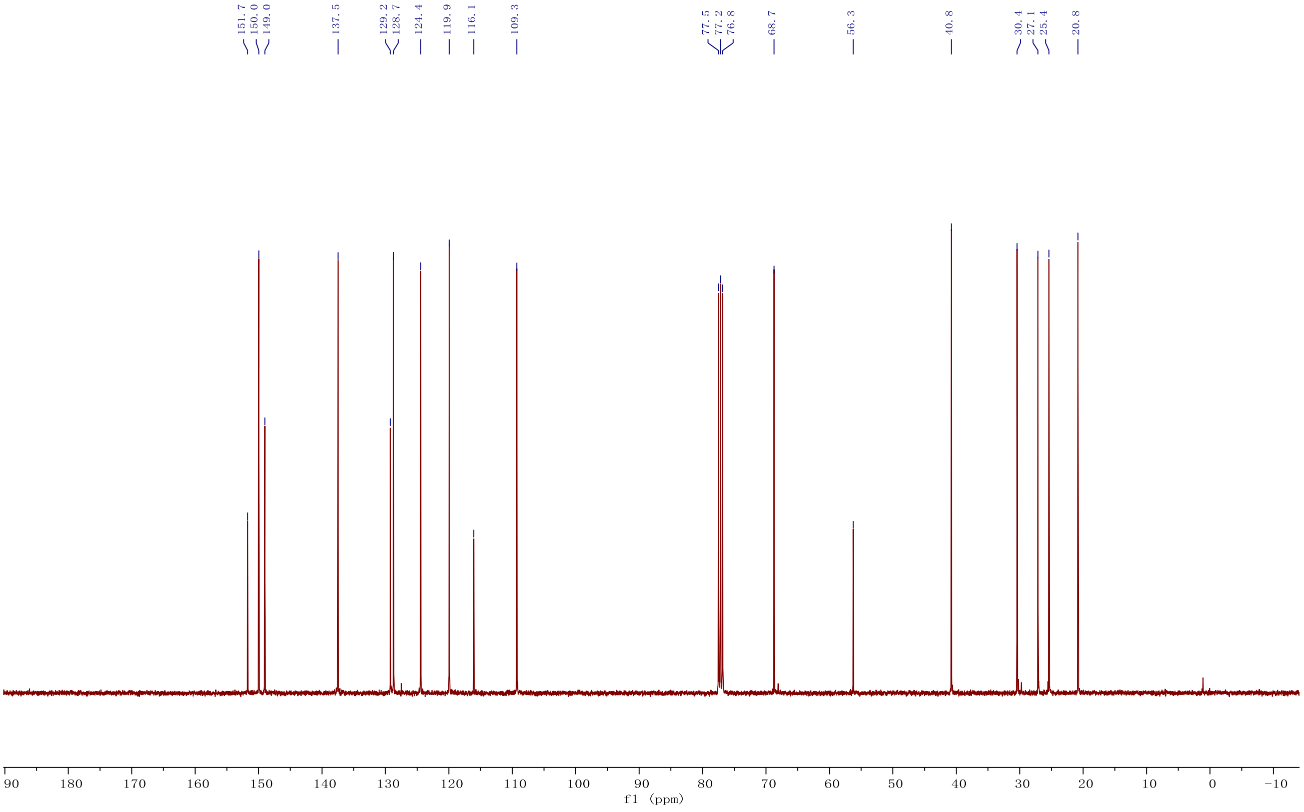

Supplementary Figure 422. ^{1}H NMR spectrum of compound **3gb** (400 MHz, $CDCl_3$)

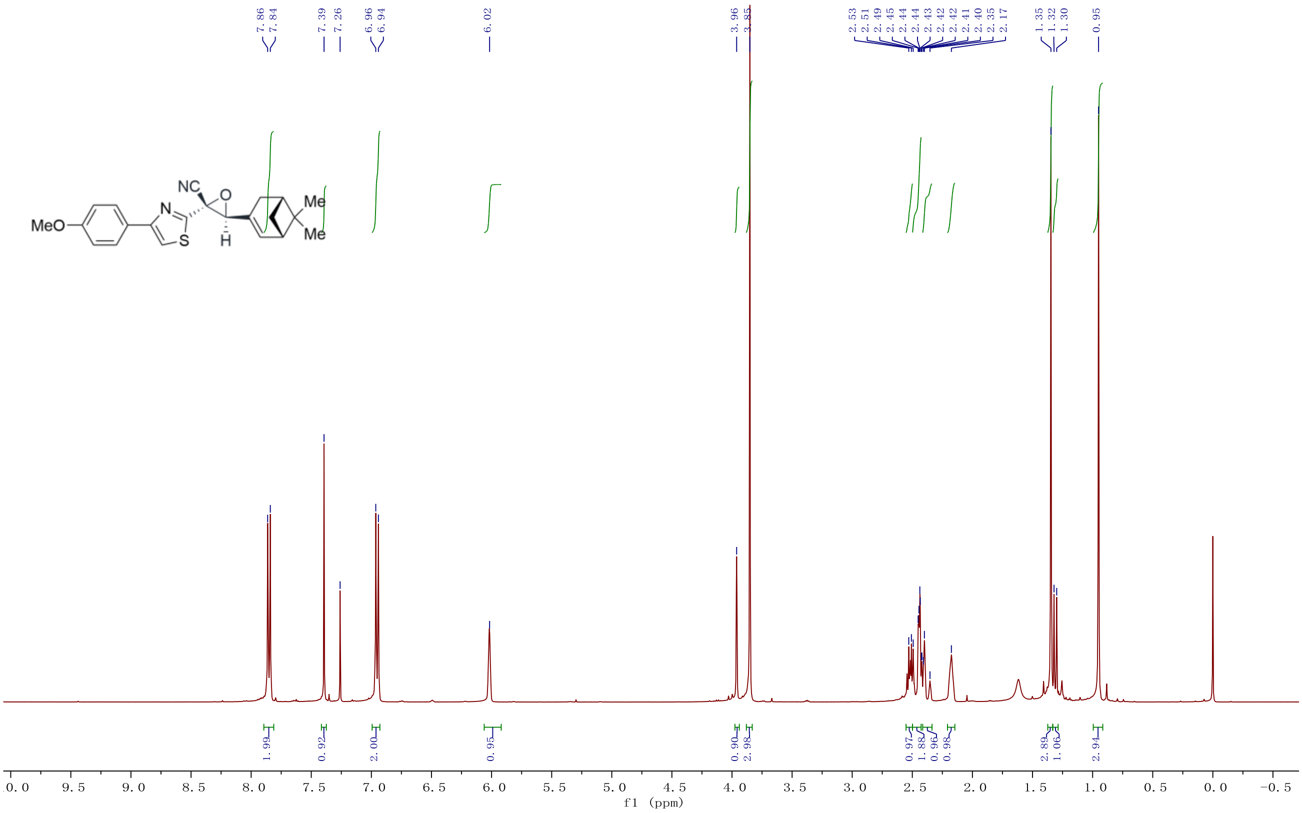

Supplementary Figure 423. ^{13}C NMR spectrum of compound **3gb** (100 MHz, $CDCl_3$)

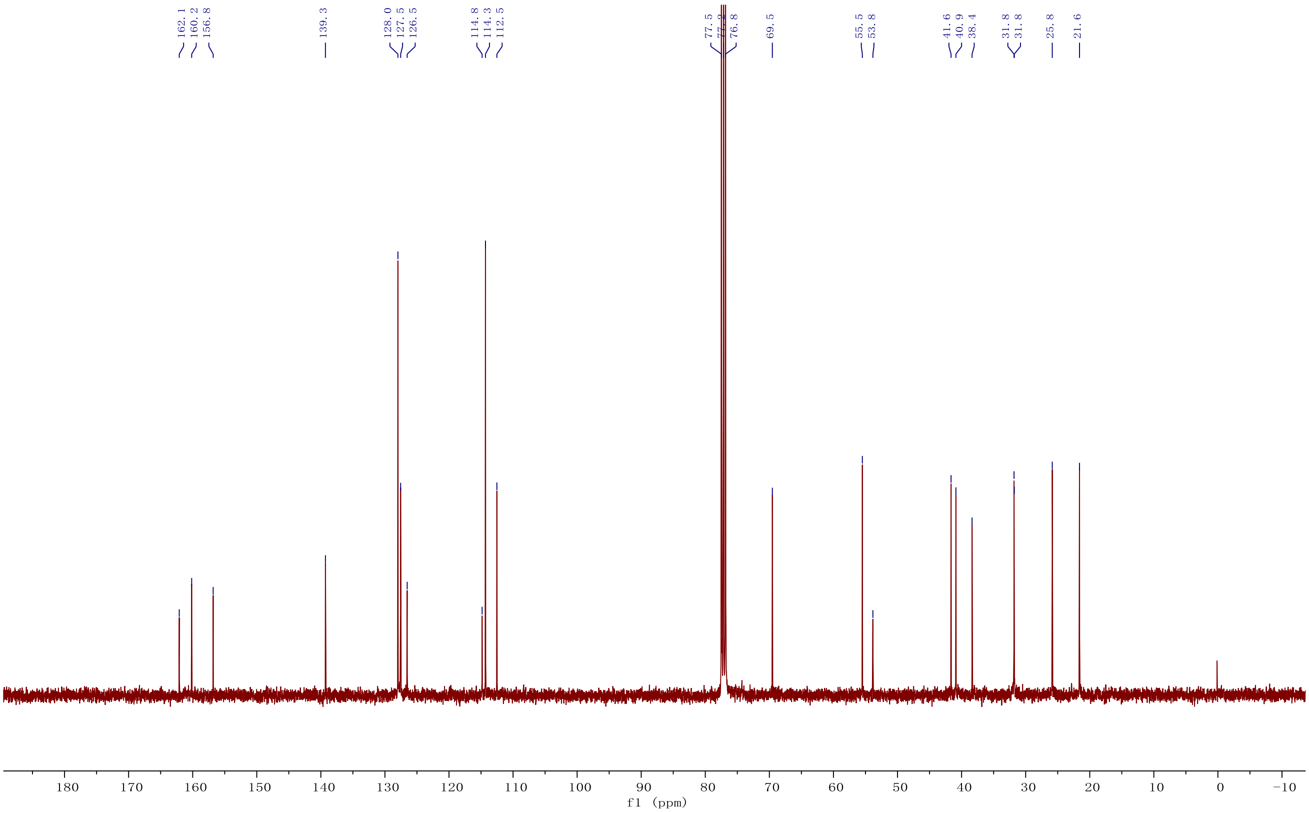

Supplementary Figure 424. ^{1}H NMR spectrum of compound **3gc** (400 MHz, $CDCl_3$)

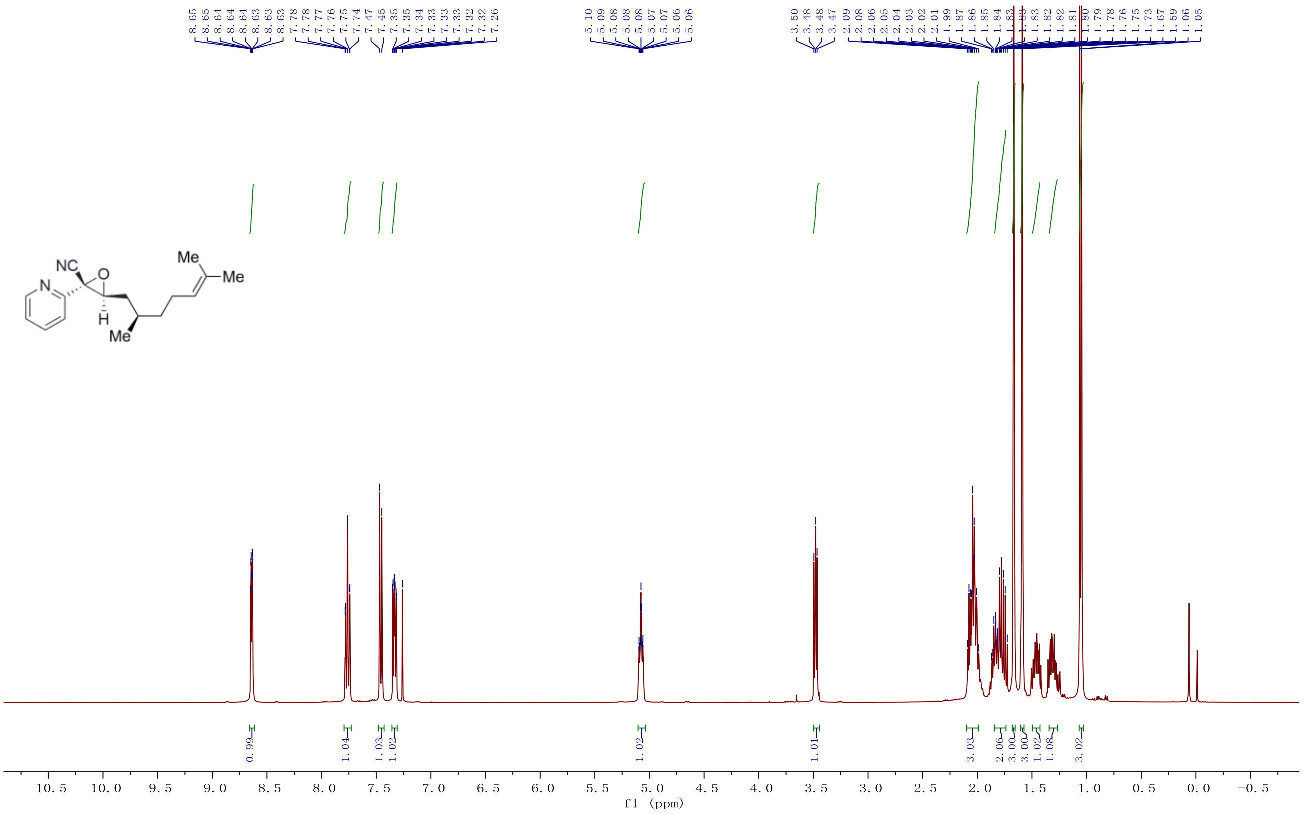

Supplementary Figure 425. ^{13}C NMR spectrum of compound **3gc** (100 MHz, $CDCl_3$)

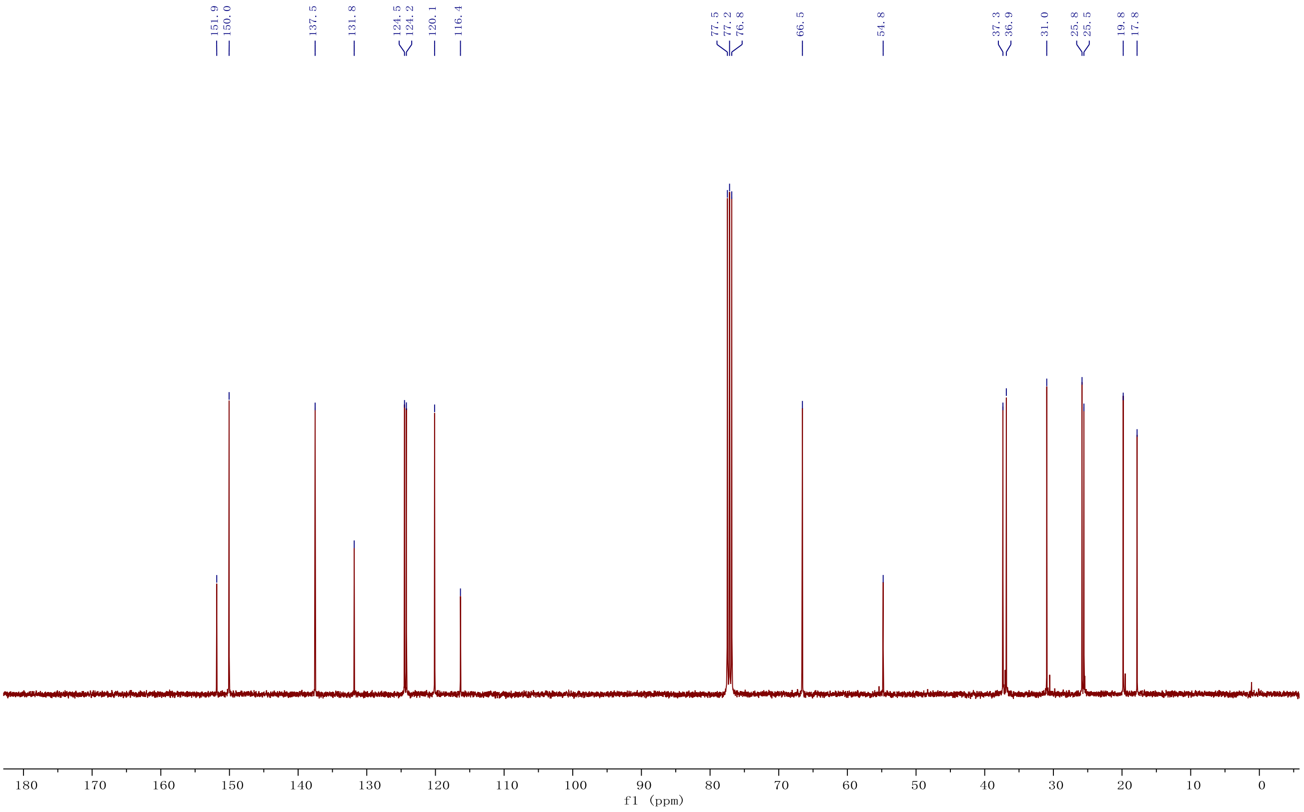

Supplementary Figure 426. ^{1}H NMR spectrum of compound **3gd** (400 MHz, $CDCl_3$)

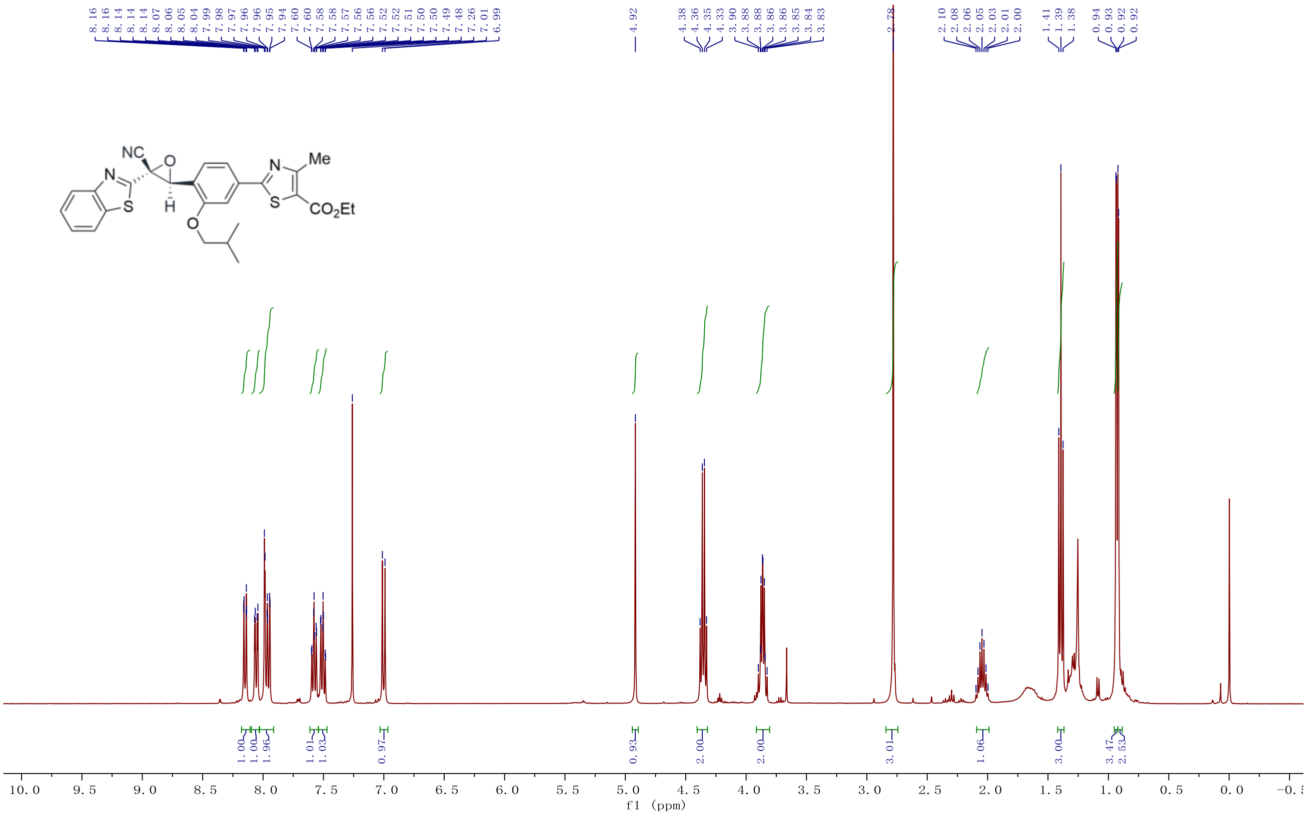

Supplementary Figure 427. ^{13}C NMR spectrum of compound **3gd** (100 MHz, $CDCl_3$)

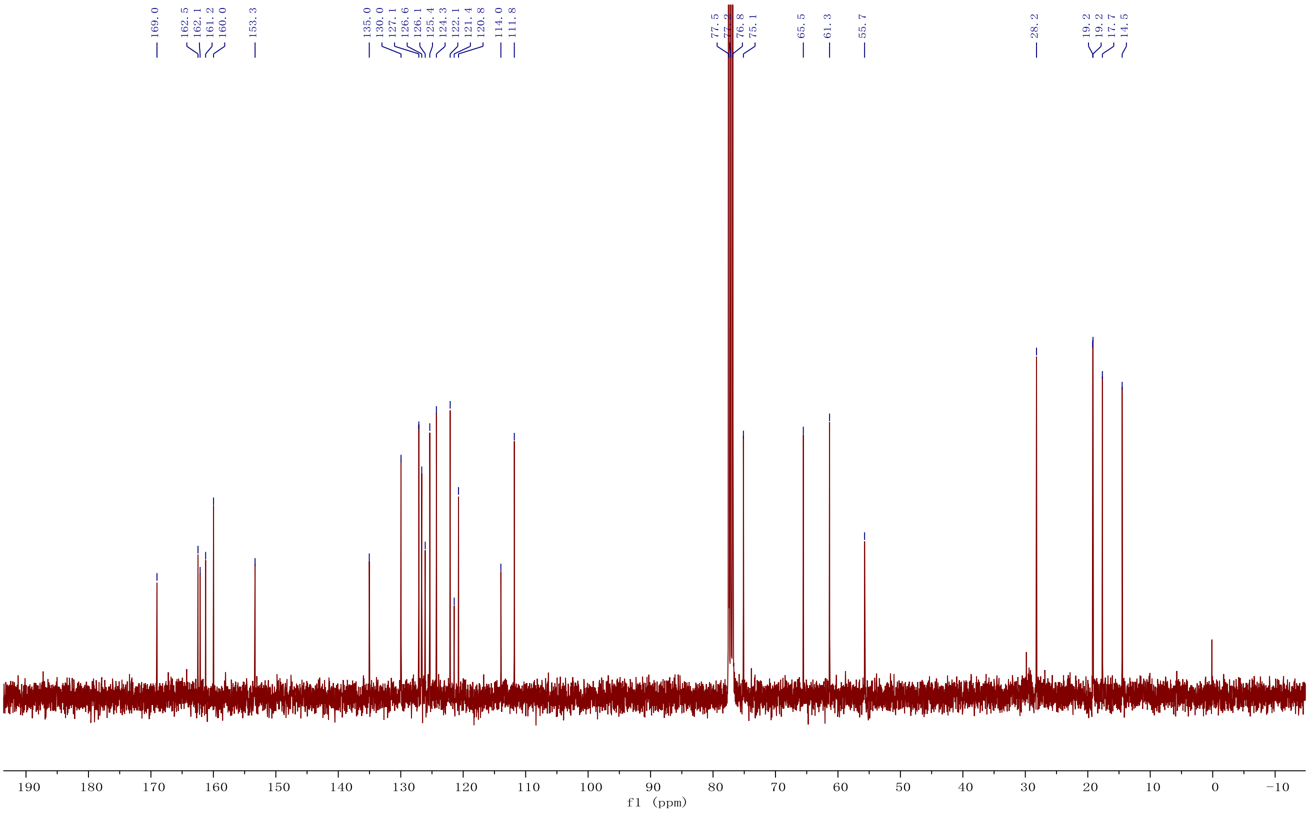

Supplementary Figure 428. ^{1}H NMR spectrum of compound **3ge** (400 MHz, $CDCl_3$)

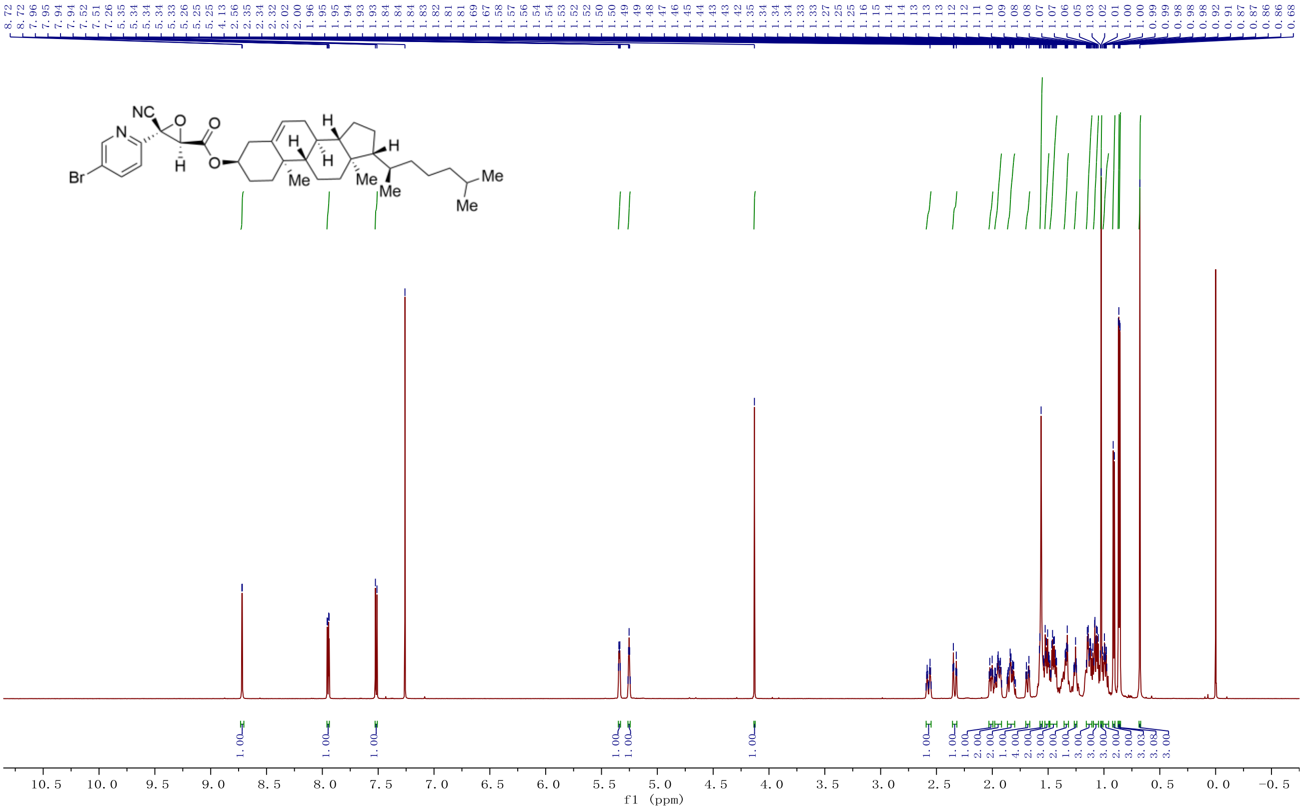

Supplementary Figure 429. ^{13}C NMR spectrum of compound **3ge** (100 MHz, $CDCl_3$)

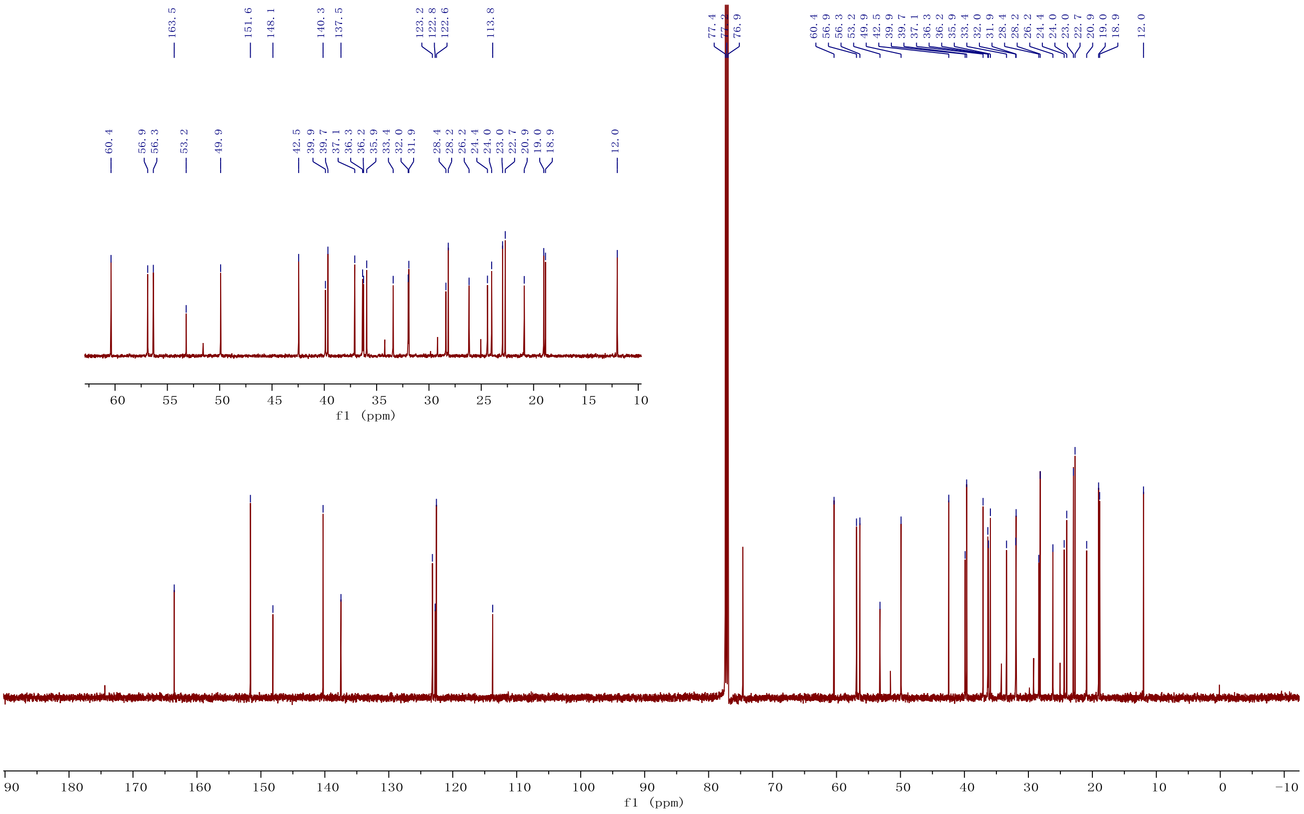

Supplementary Figure 430. ^{1}H NMR spectrum of compound **3gf** (400 MHz, $CDCl_3$)

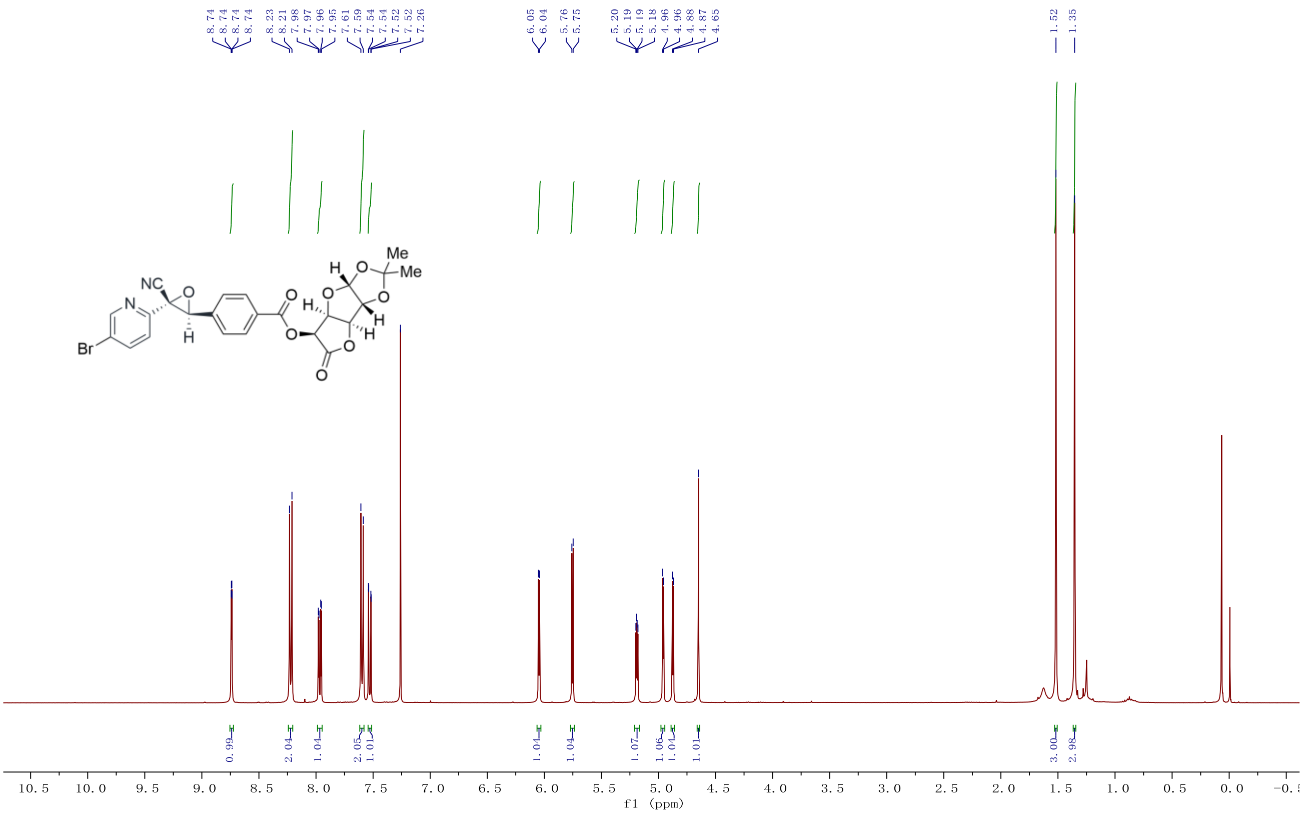

Supplementary Figure 431. ^{13}C NMR spectrum of compound **3gf** (100 MHz, $CDCl_3$)

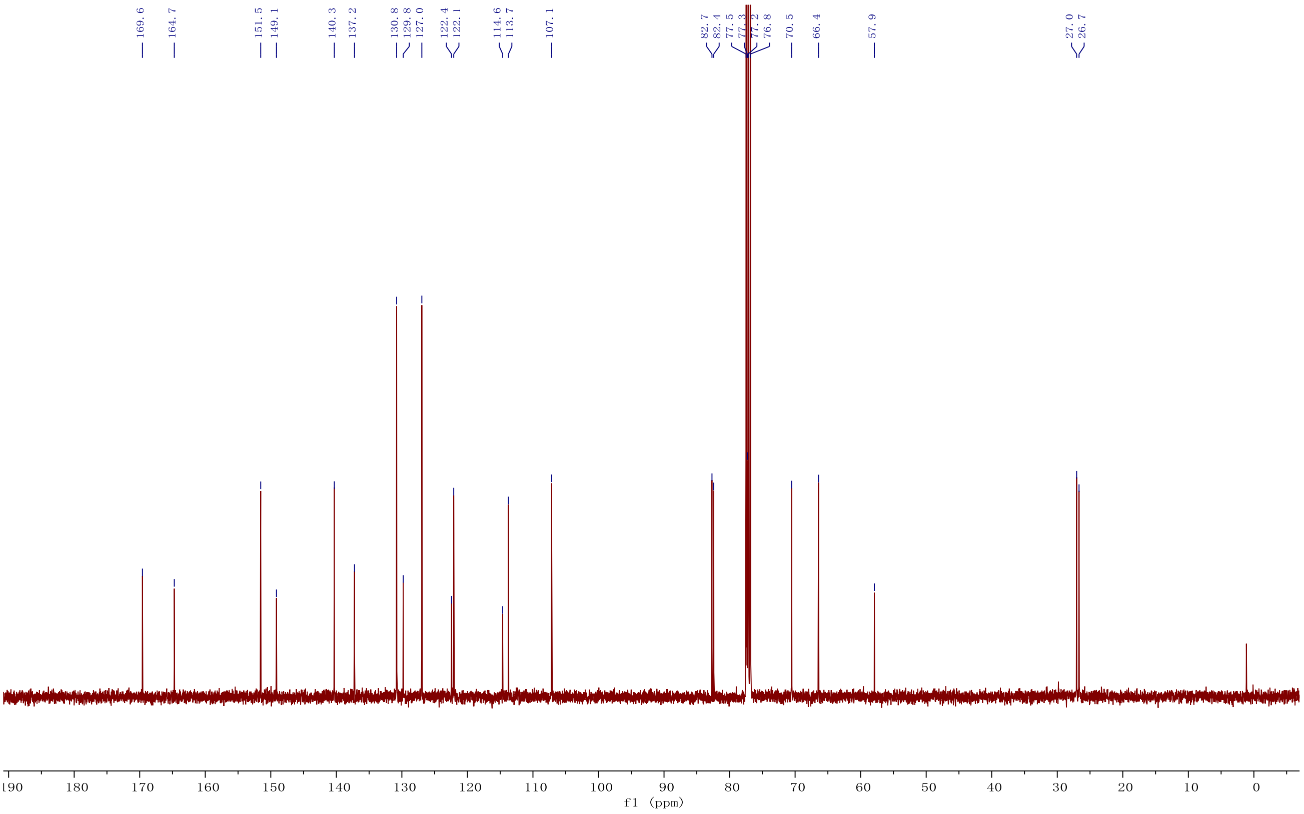

Supplementary Figure 432. ^{1}H NMR spectrum of compound **6a** (400 MHz, $CDCl_3$)

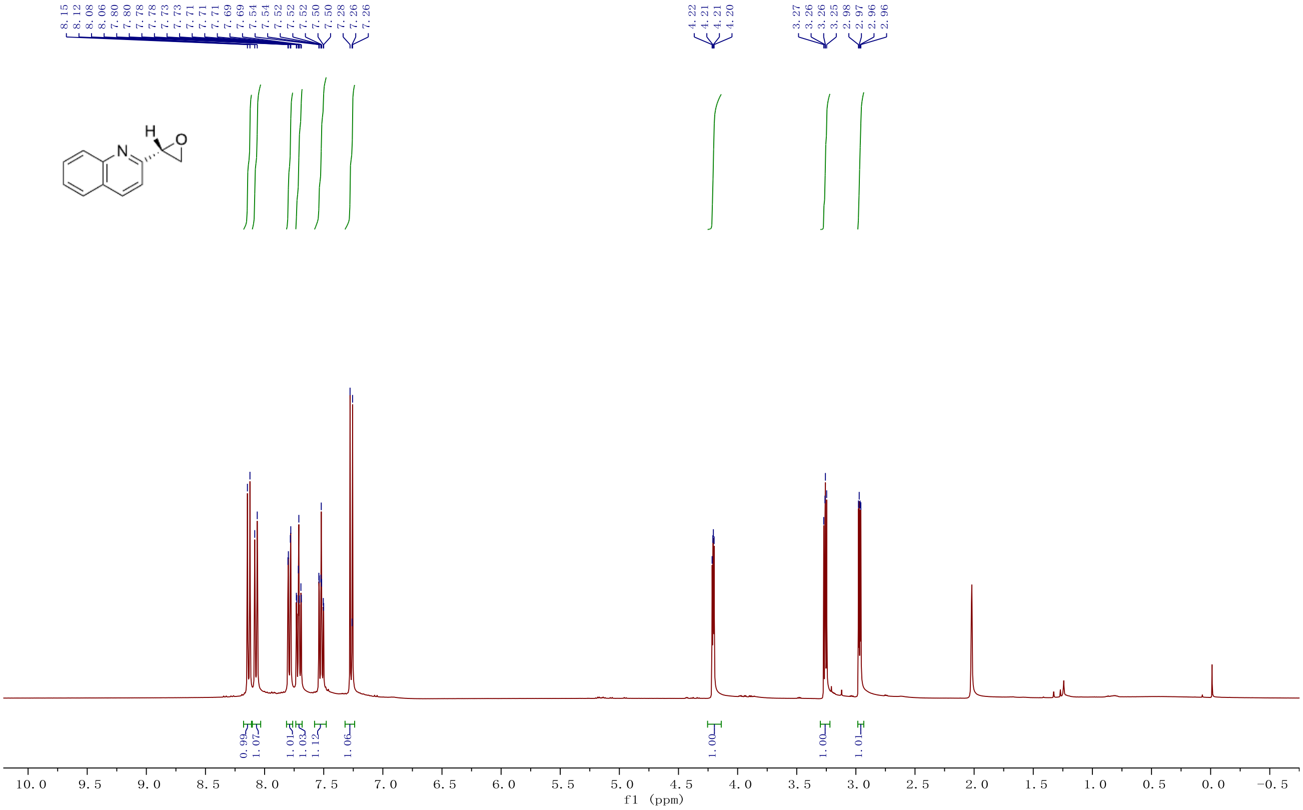

Supplementary Figure 433. ^{13}C NMR spectrum of compound **6a** (100 MHz, $CDCl_3$)

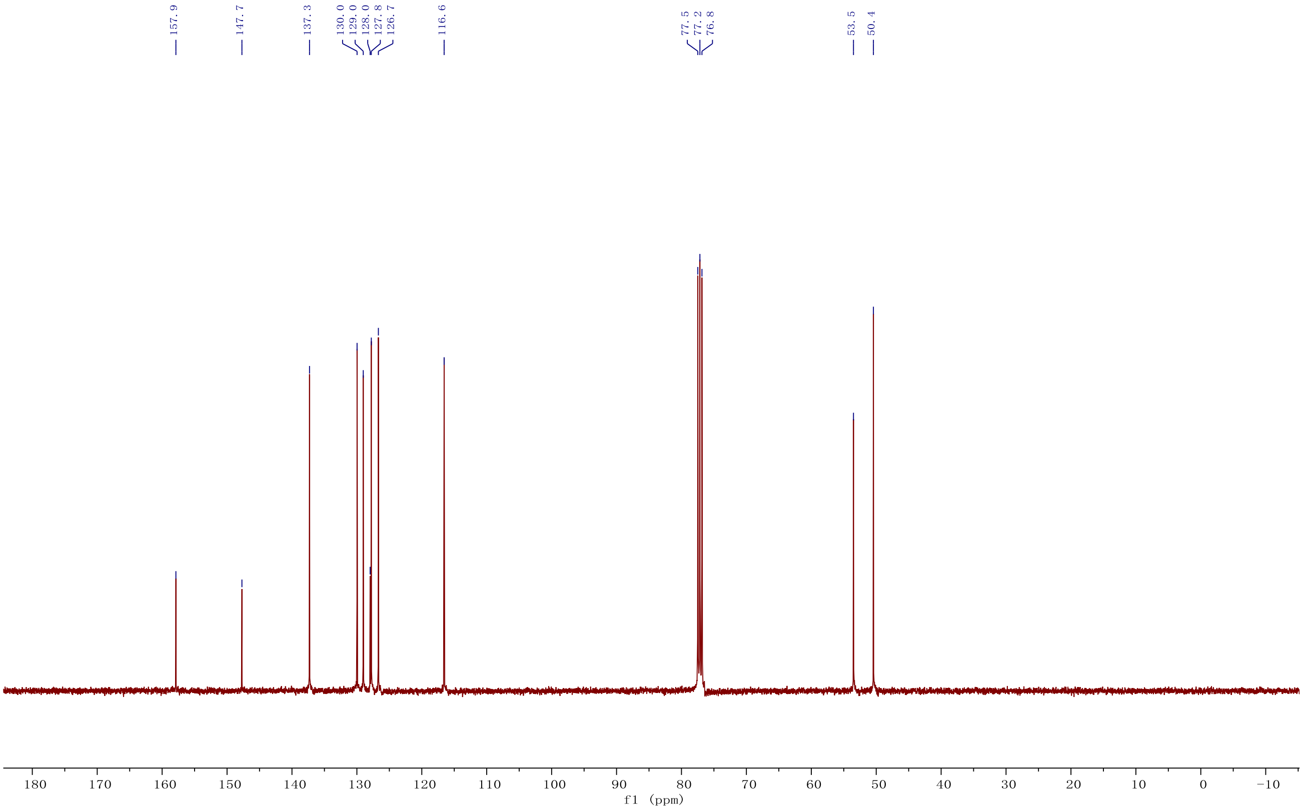

Supplementary Figure 434. ^{1}H NMR spectrum of compound **6b** (600 MHz, $CDCl_3$)

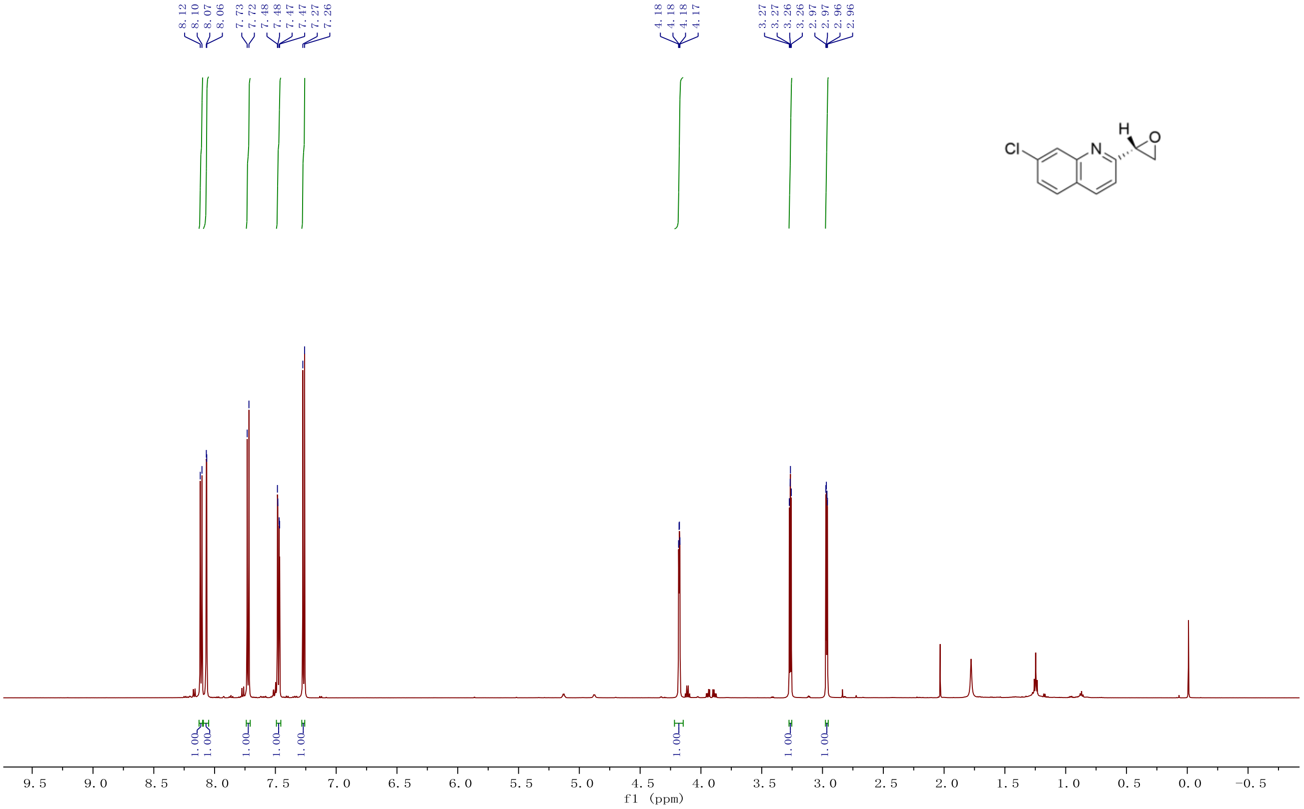

Supplementary Figure 435. ^{13}C NMR spectrum of compound **6b** (100 MHz, $CDCl_3$)

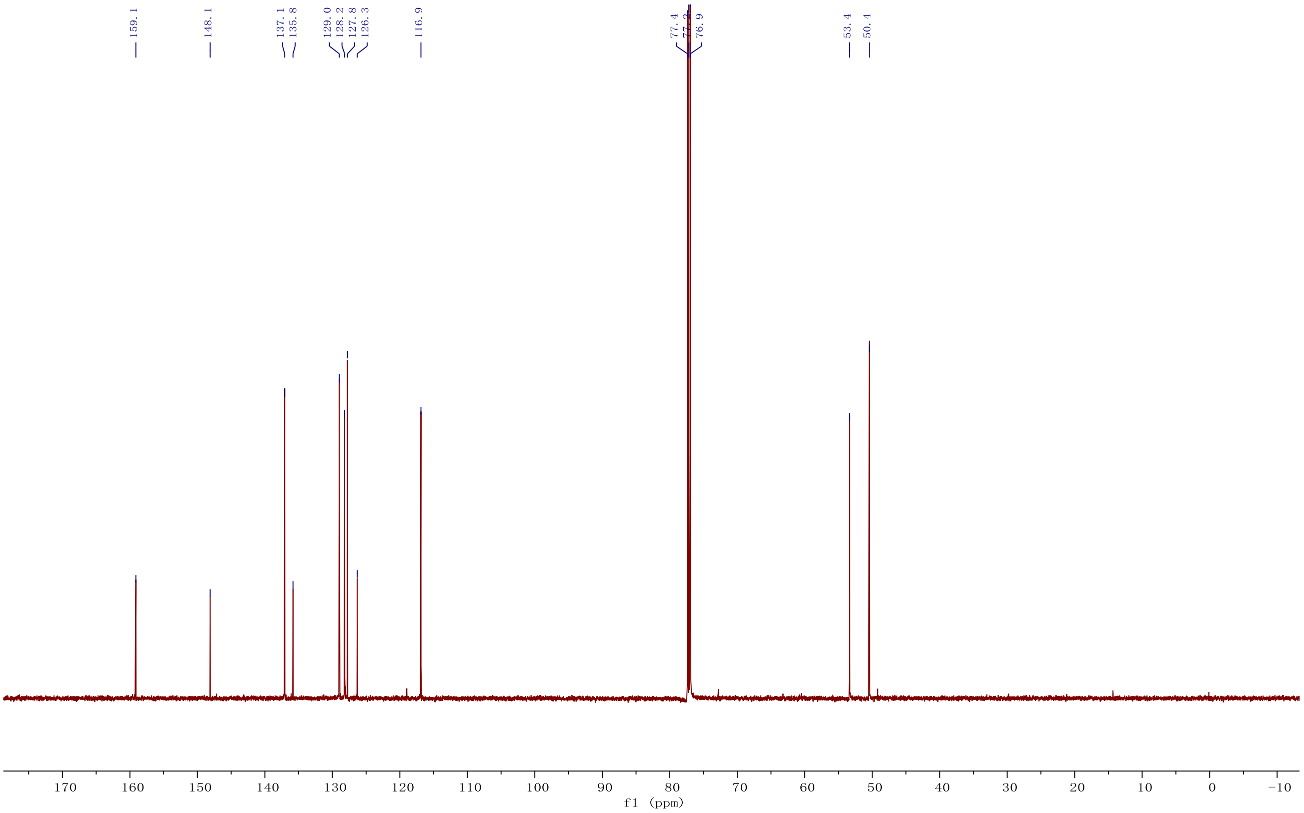

Supplementary Figure 436. ^{1}H NMR spectrum of compound **6c** (400 MHz, $CDCl_3$)

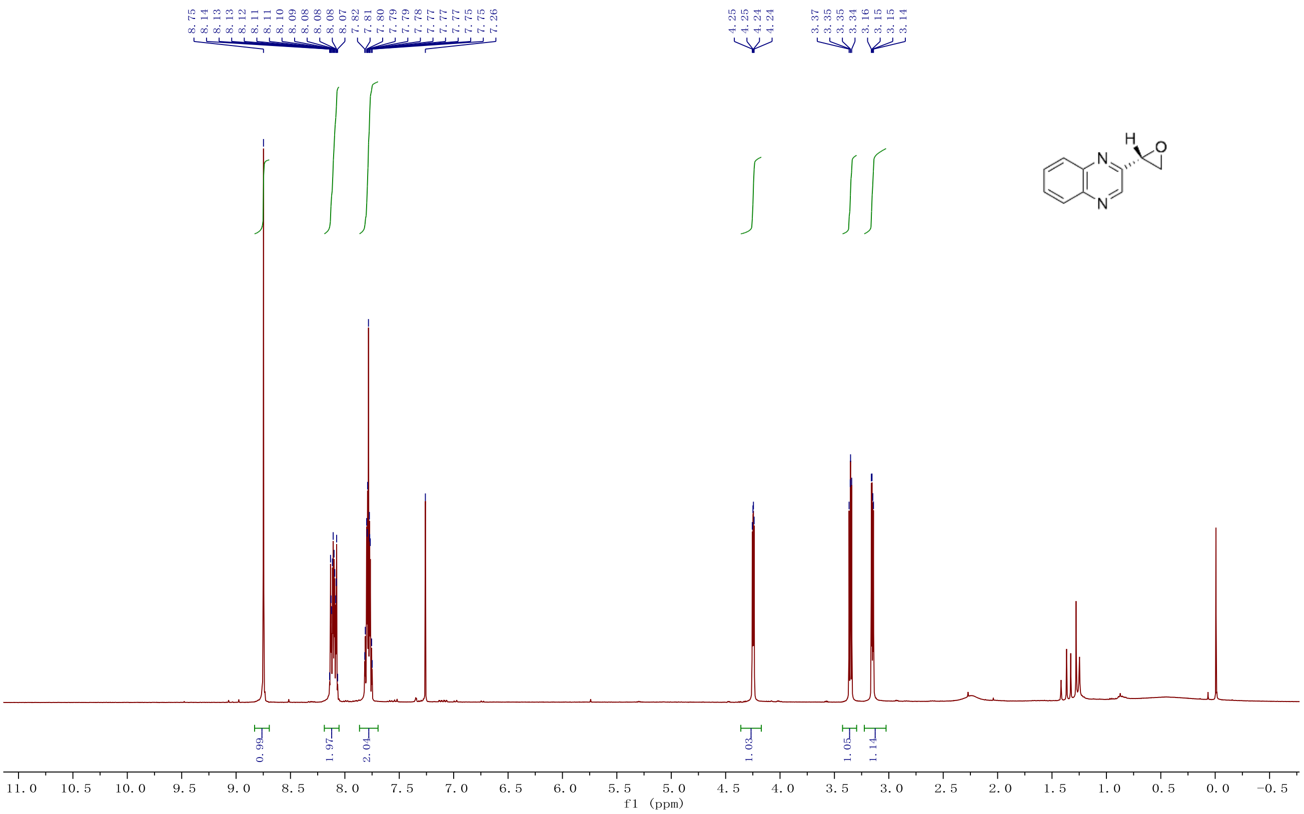

Supplementary Figure 437. ^{13}C NMR spectrum of compound **6c** (100 MHz, $CDCl_3$)

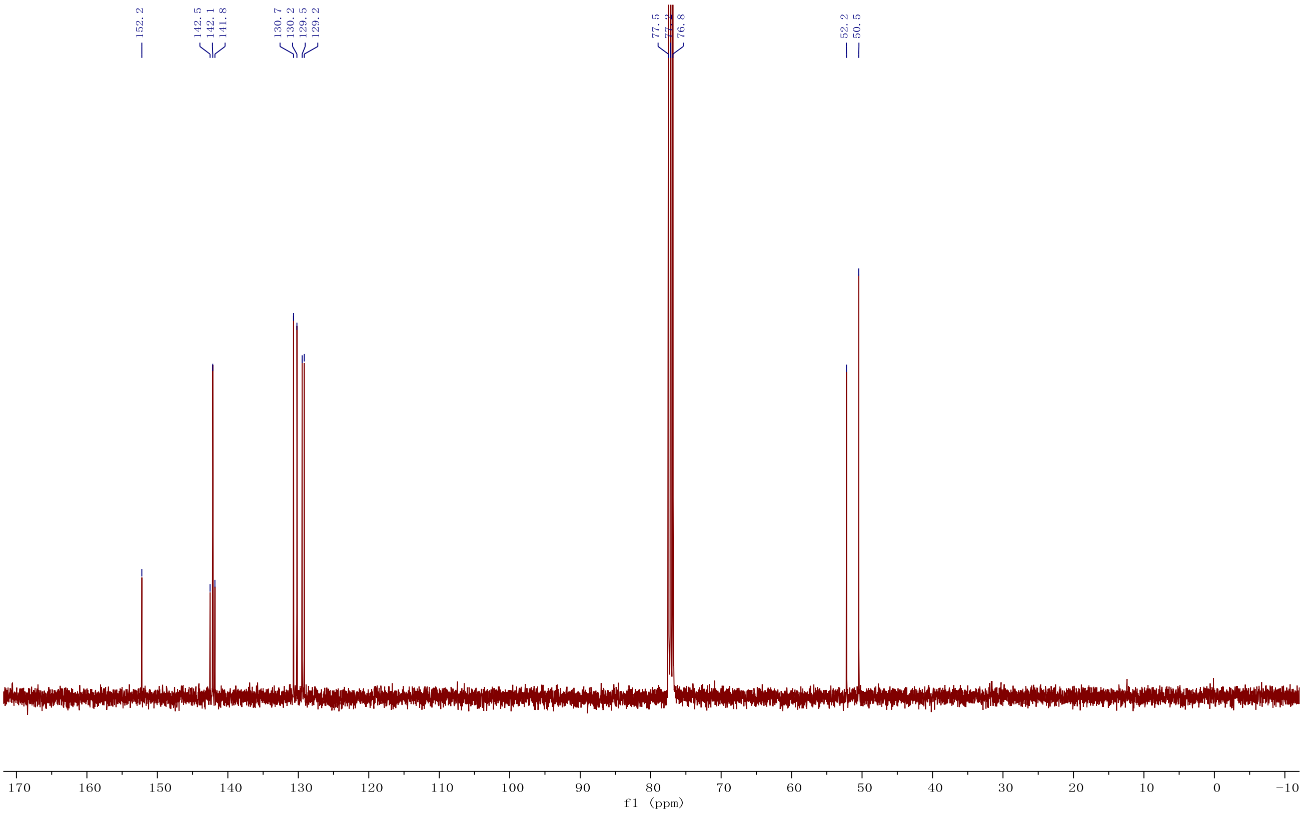

Supplementary Figure 438. ^{1}H NMR spectrum of compound **6d** (400 MHz, $CDCl_3$)

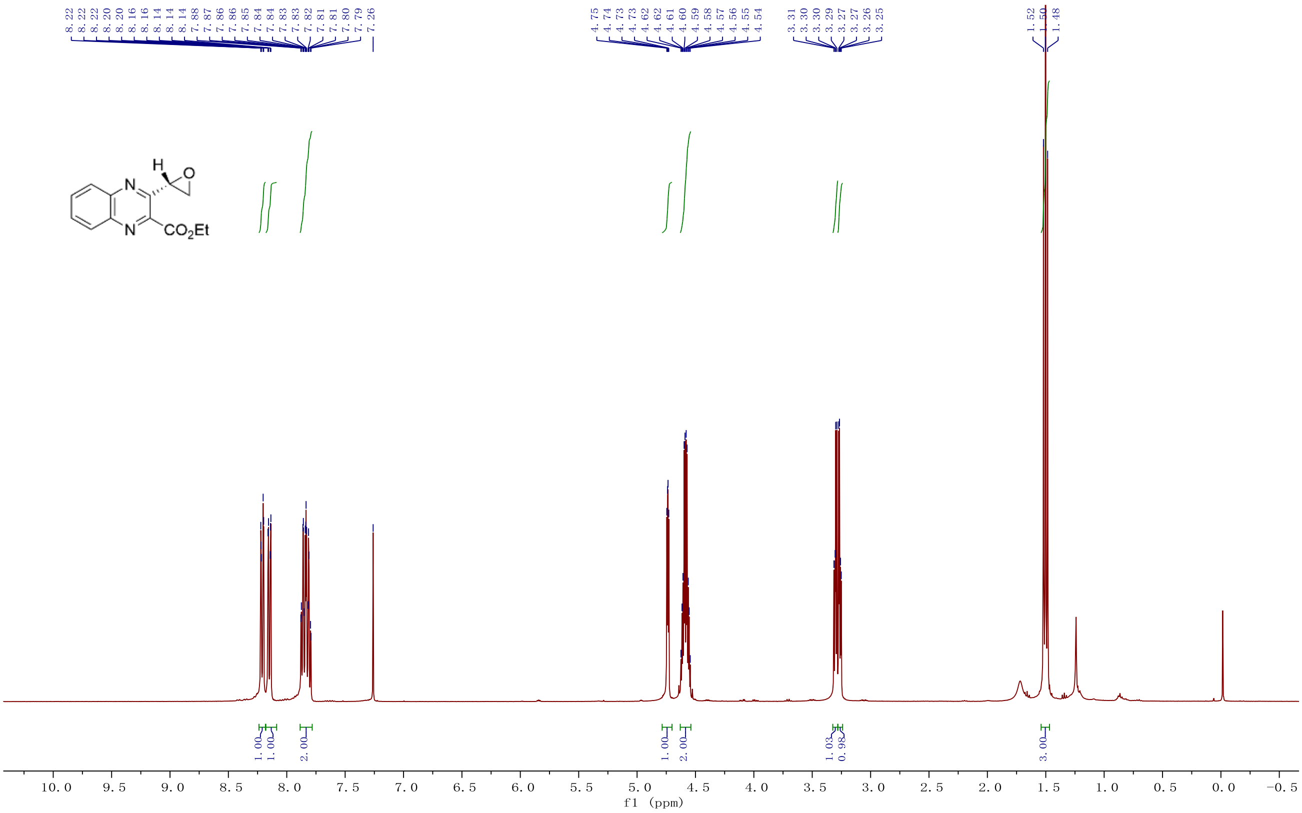

Supplementary Figure 439. ^{13}C NMR spectrum of compound **6d** (100 MHz, $CDCl_3$)

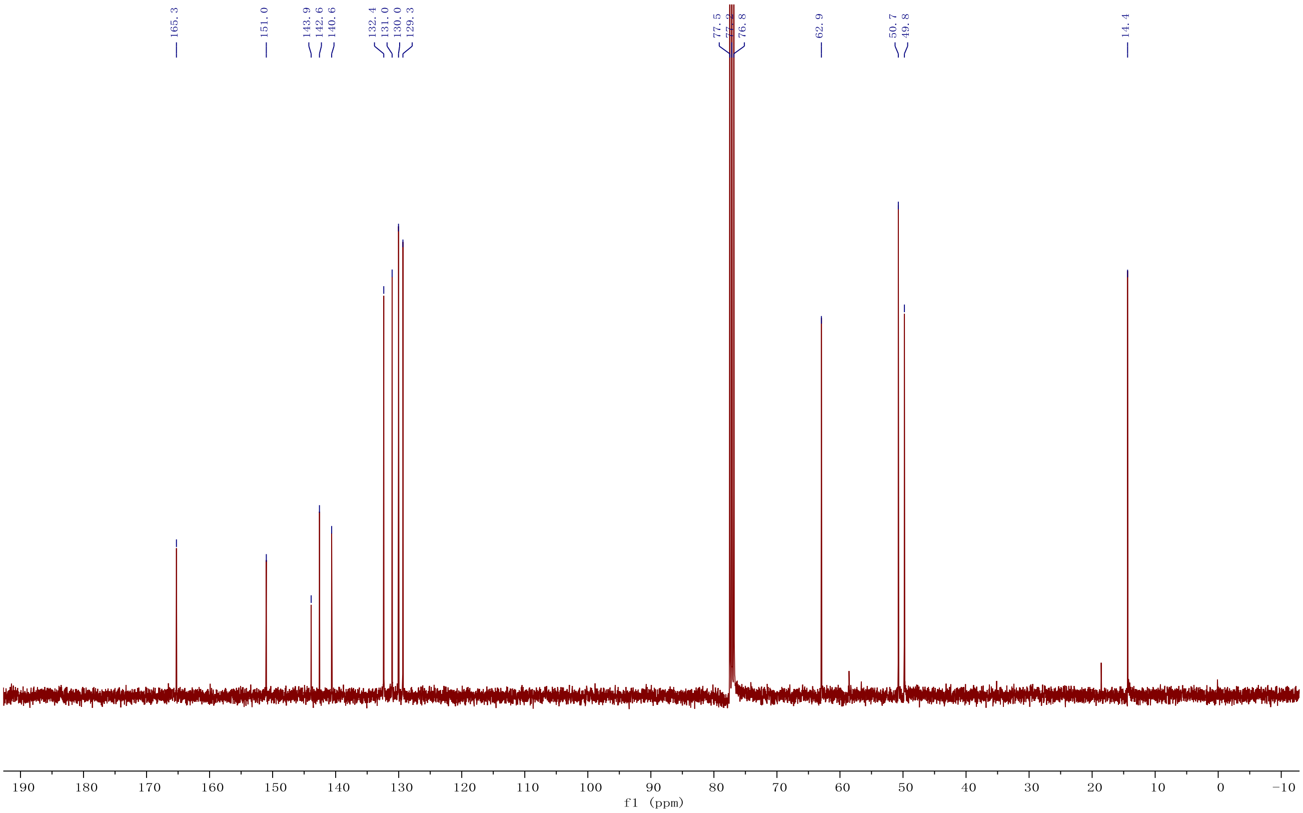

Supplementary Figure 440. ^{1}H NMR spectrum of compound **6e** (400 MHz, $CDCl_3$)

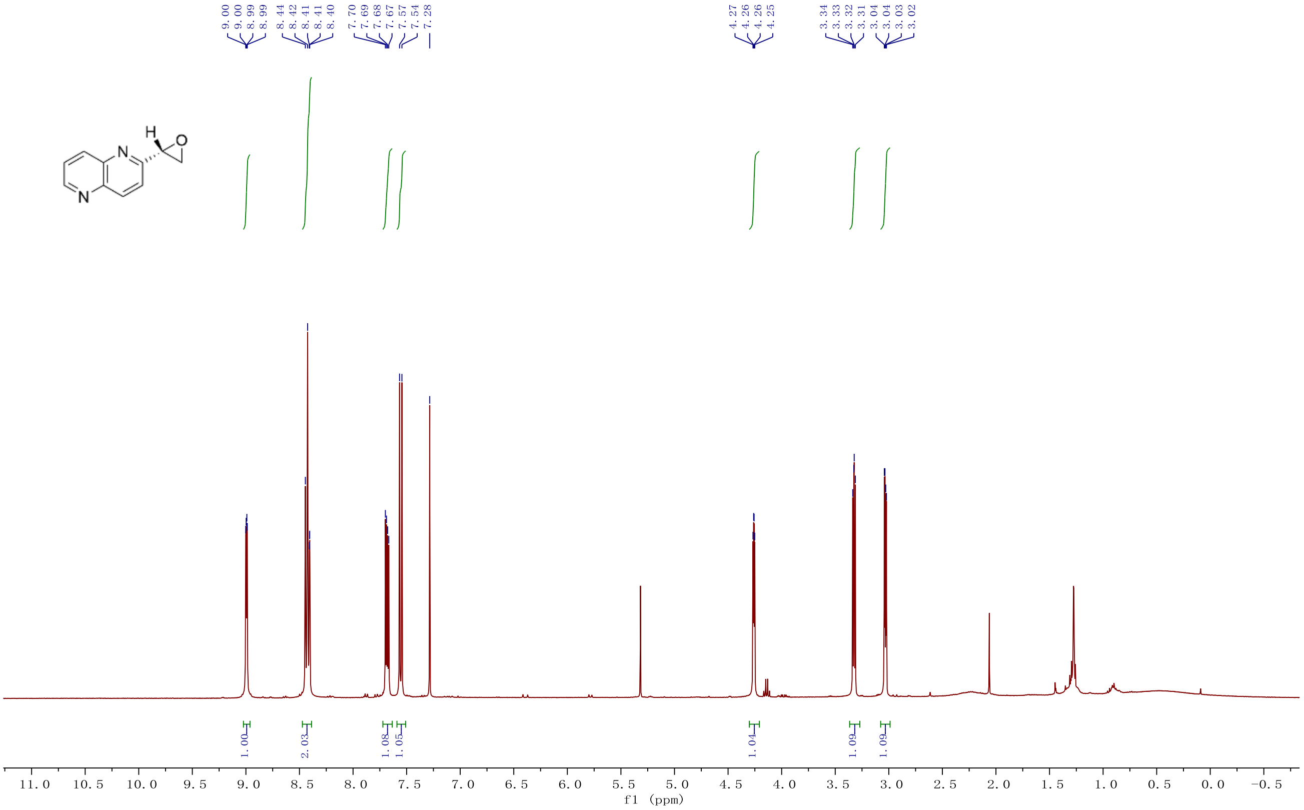

Supplementary Figure 441. ^{13}C NMR spectrum of compound **6e** (100 MHz, $CDCl_3$)

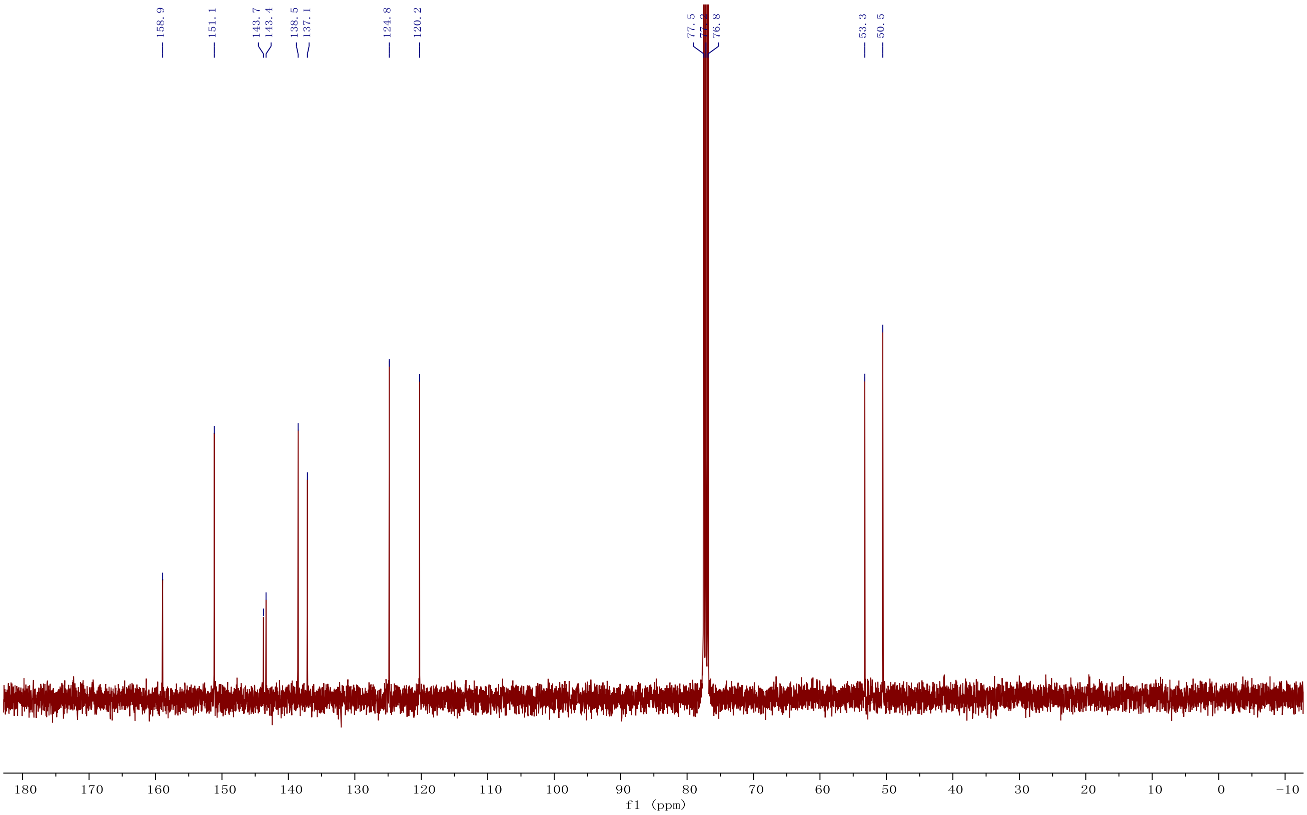

Supplementary Figure 442. ^{1}H NMR spectrum of compound **6f** (400 MHz, $CDCl_3$)

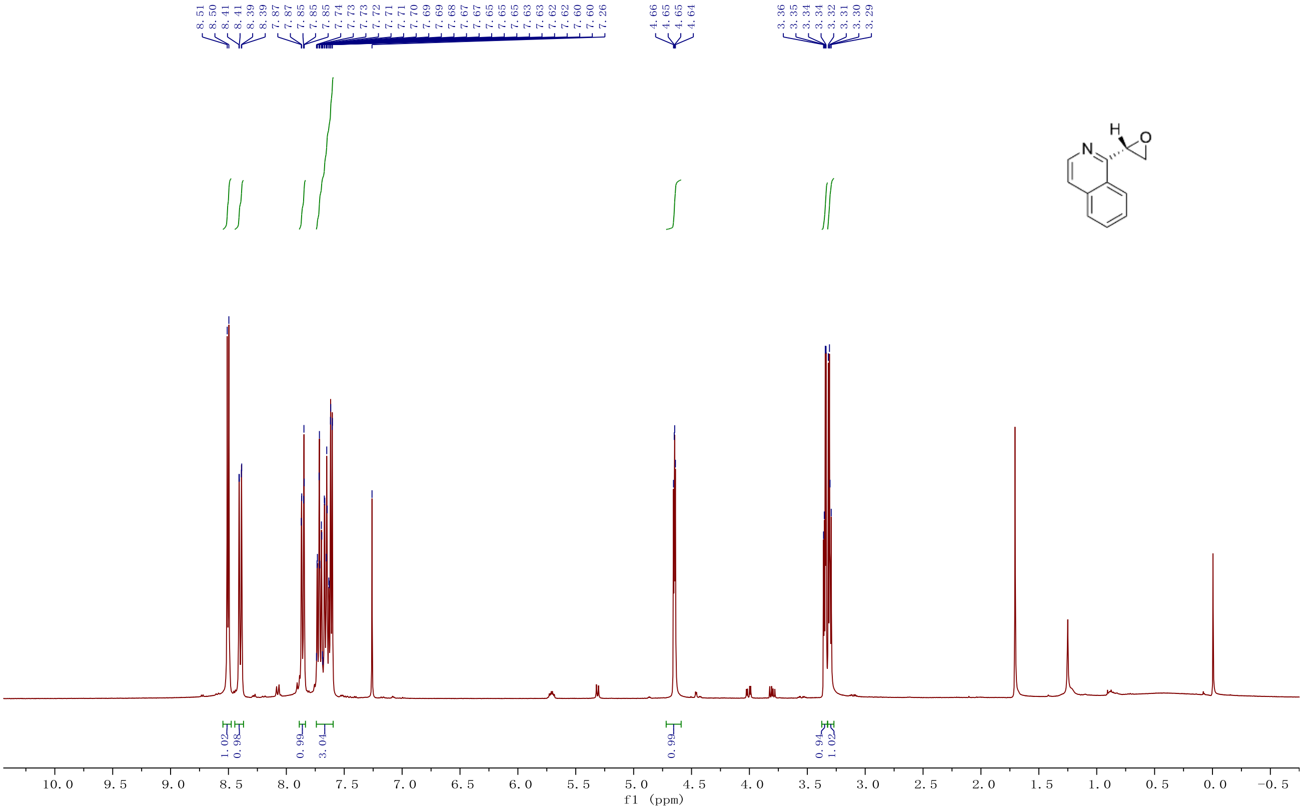

Supplementary Figure 443. ^{13}C NMR spectrum of compound **6f** (100 MHz, $CDCl_3$)

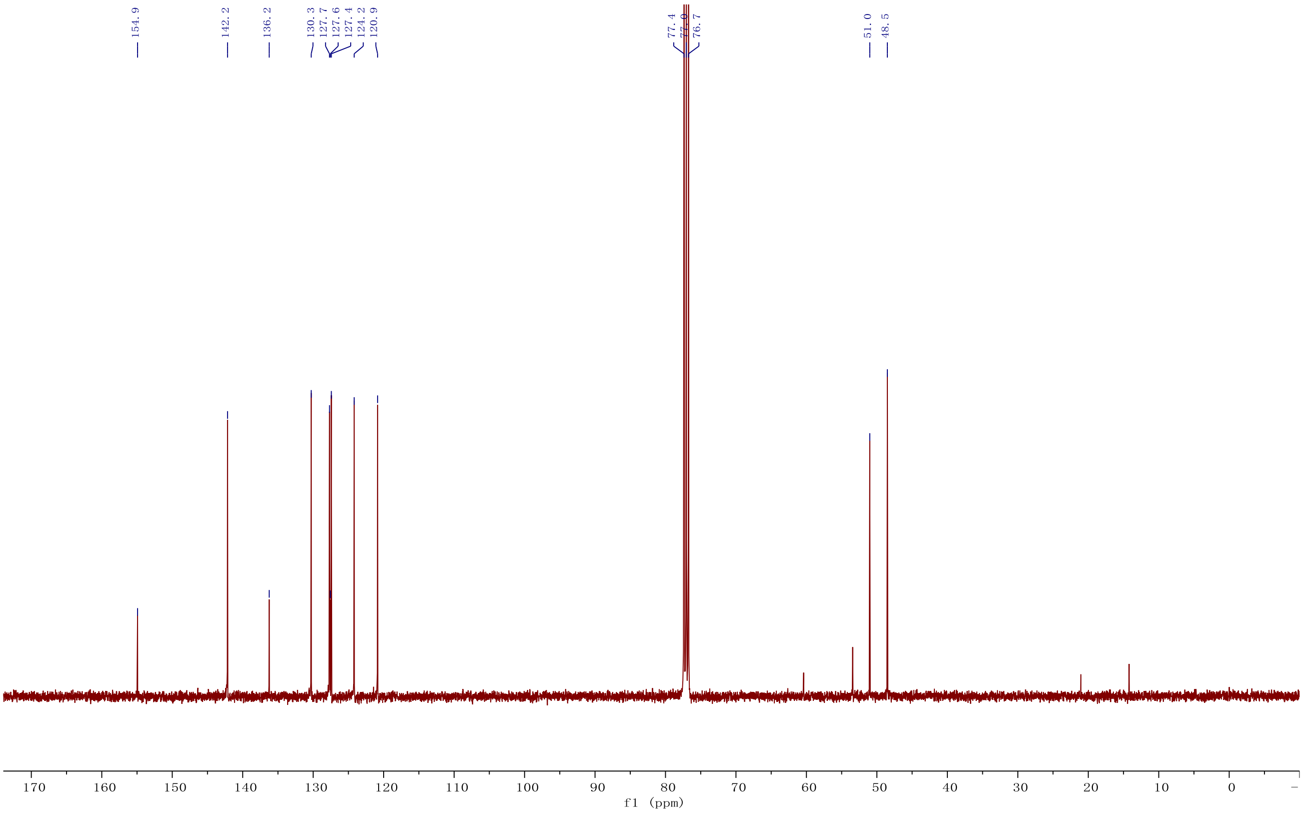

Supplementary Figure 444. ^{1}H NMR spectrum of compound **6g** (400 MHz, $CDCl_3$)

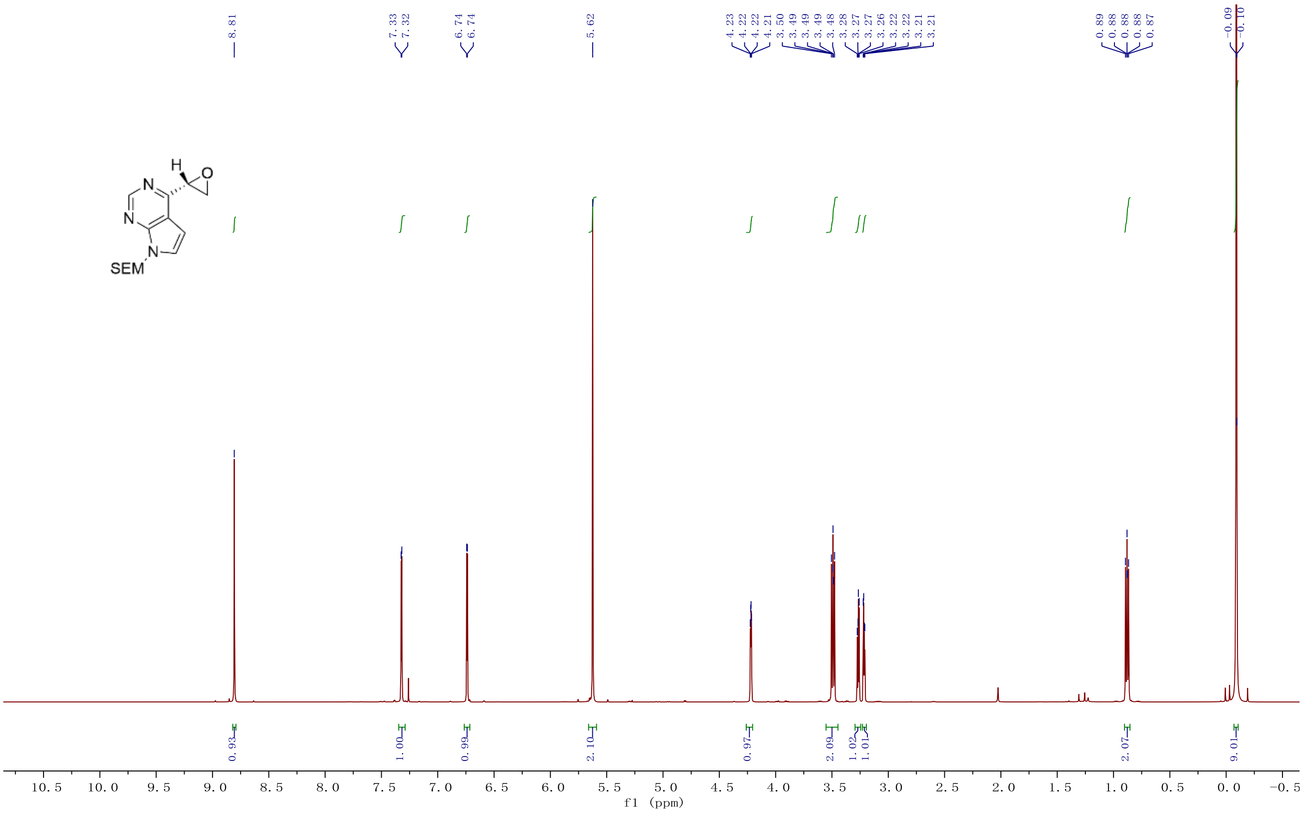

Supplementary Figure 445. ^{13}C NMR spectrum of compound **6g** (100 MHz, $CDCl_3$)

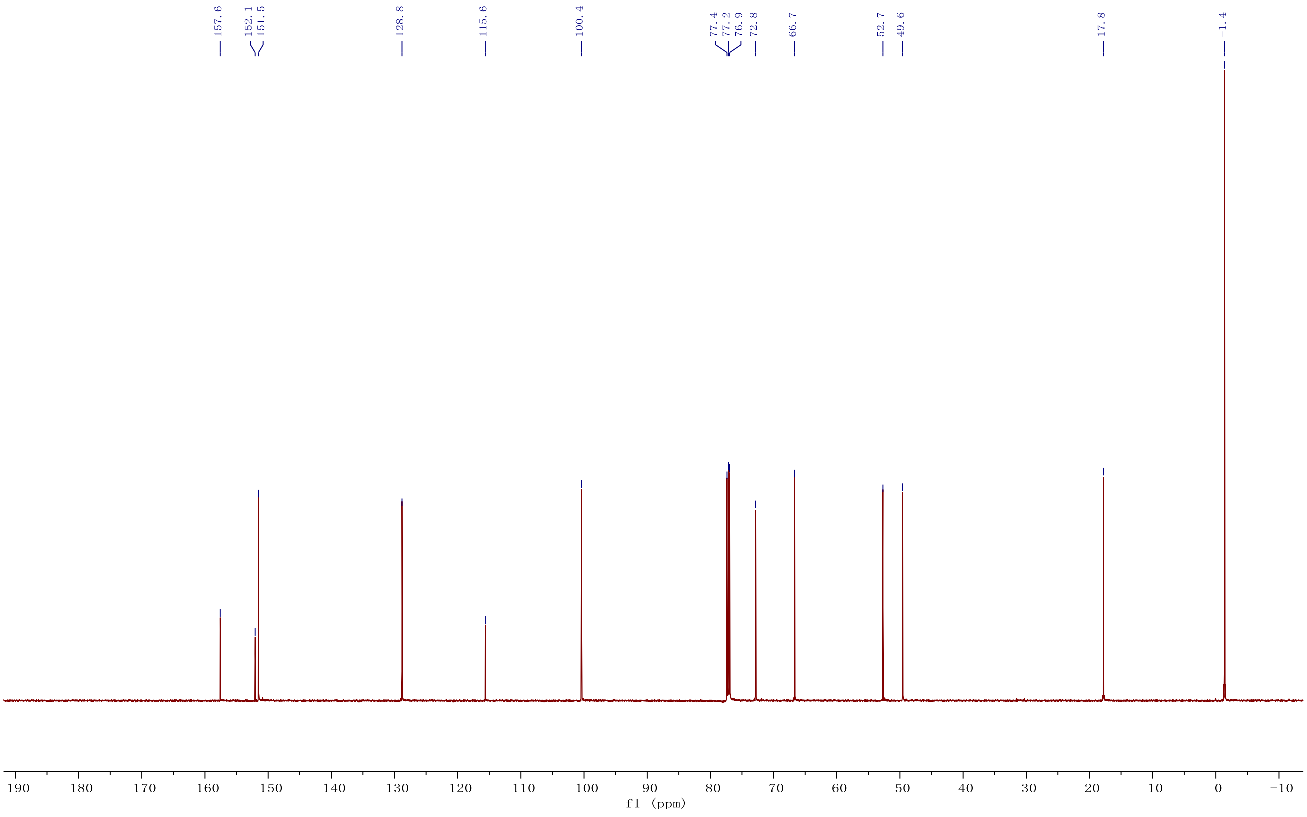

Supplementary Figure 446. ^{1}H NMR spectrum of compound **6h** (400 MHz, $CDCl_3$)

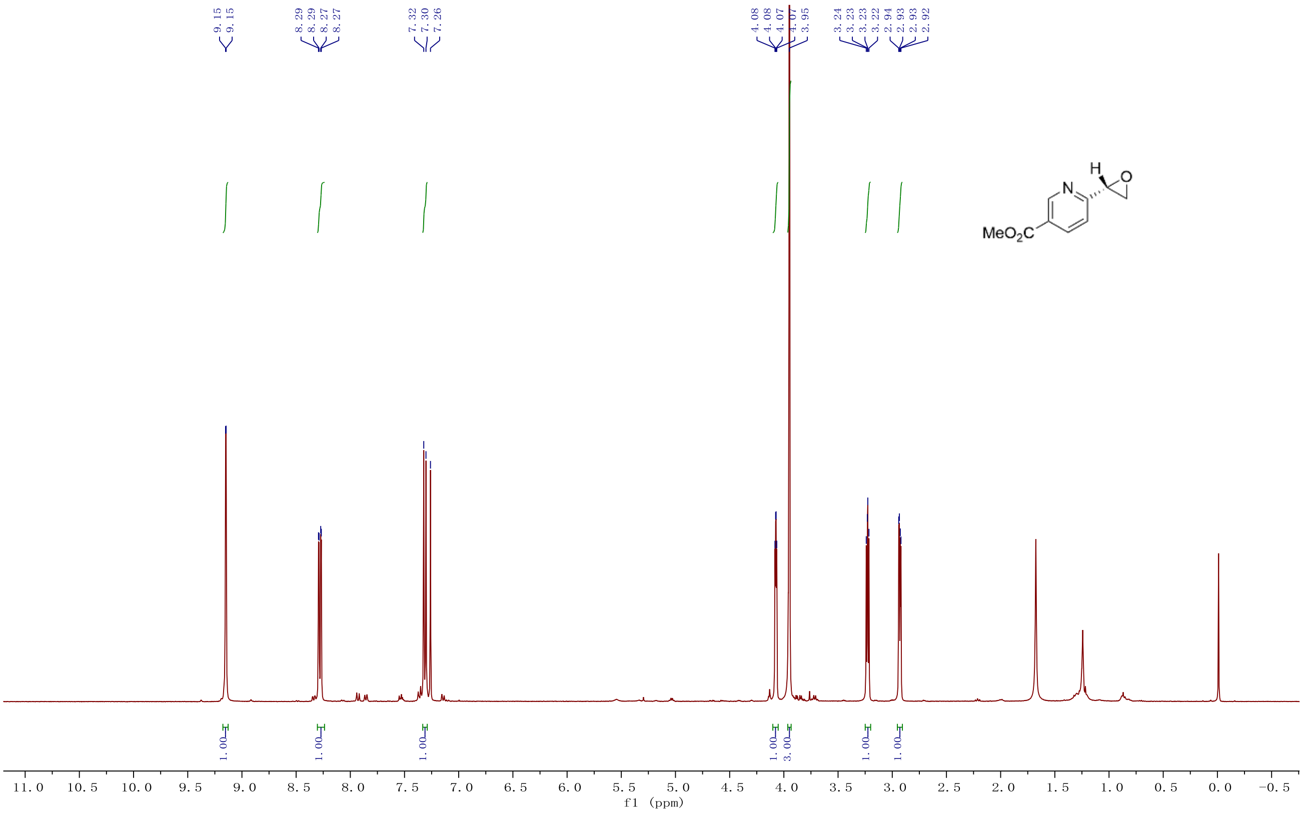

Supplementary Figure 447. ^{13}C NMR spectrum of compound **6h** (100 MHz, $CDCl_3$)

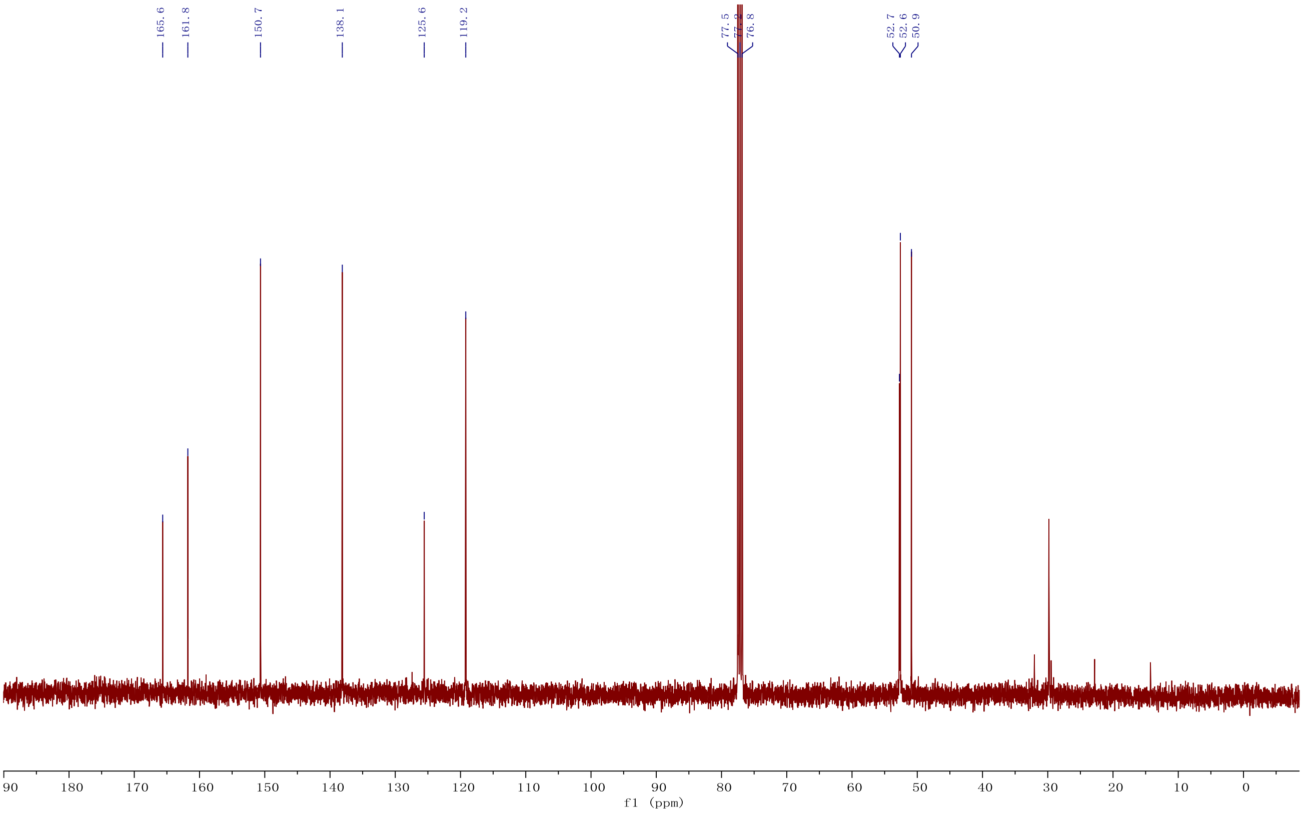

Supplementary Figure 448. ^{1}H NMR spectrum of compound **6i** (400 MHz, $CDCl_3$)

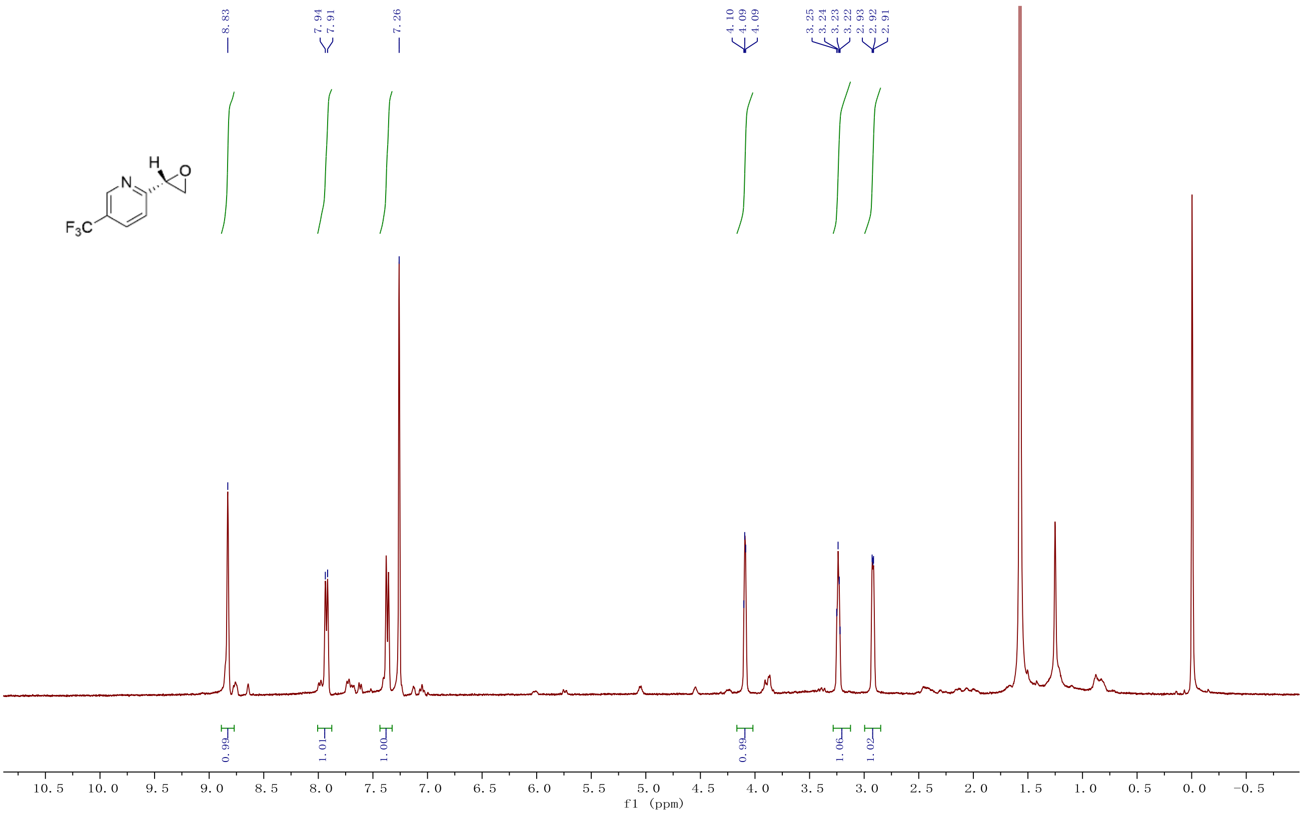

Supplementary Figure 449. ^{13}C NMR spectrum of compound **6i** (100 MHz, $CDCl_3$)

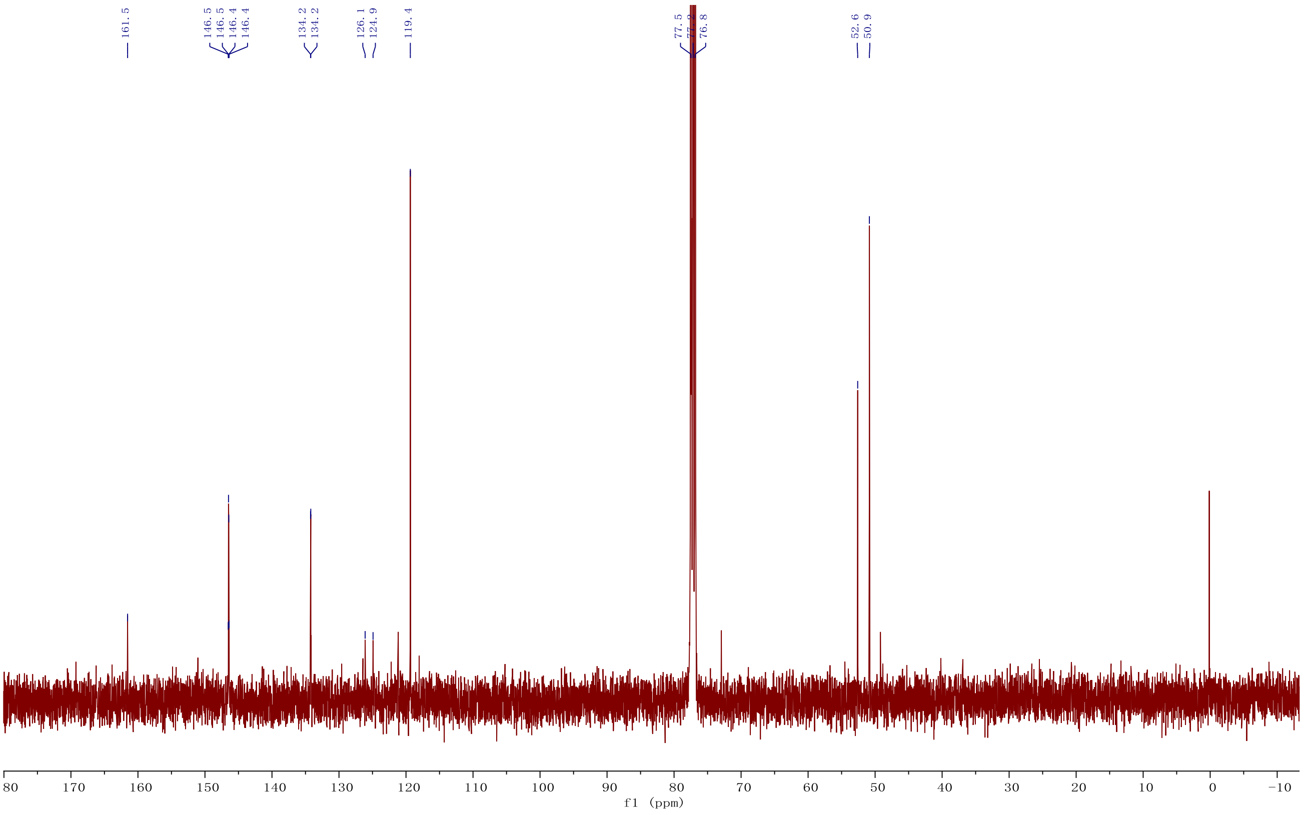

Supplementary Figure 450. ^{19}F NMR spectrum of compound **6i** (376 MHz, $CDCl_3$)

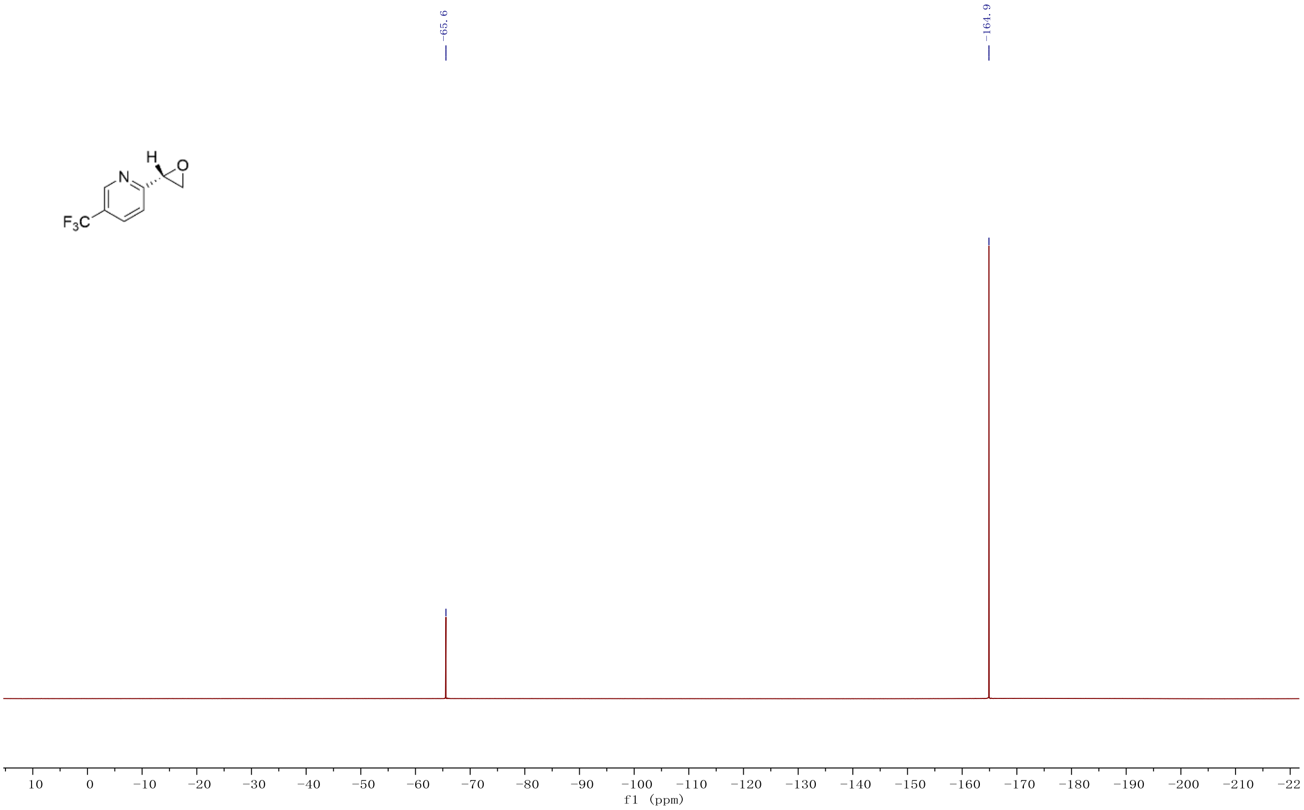

Supplementary Figure 451. ^{1}H NMR spectrum of compound **6j** (600 MHz, $CDCl_3$)

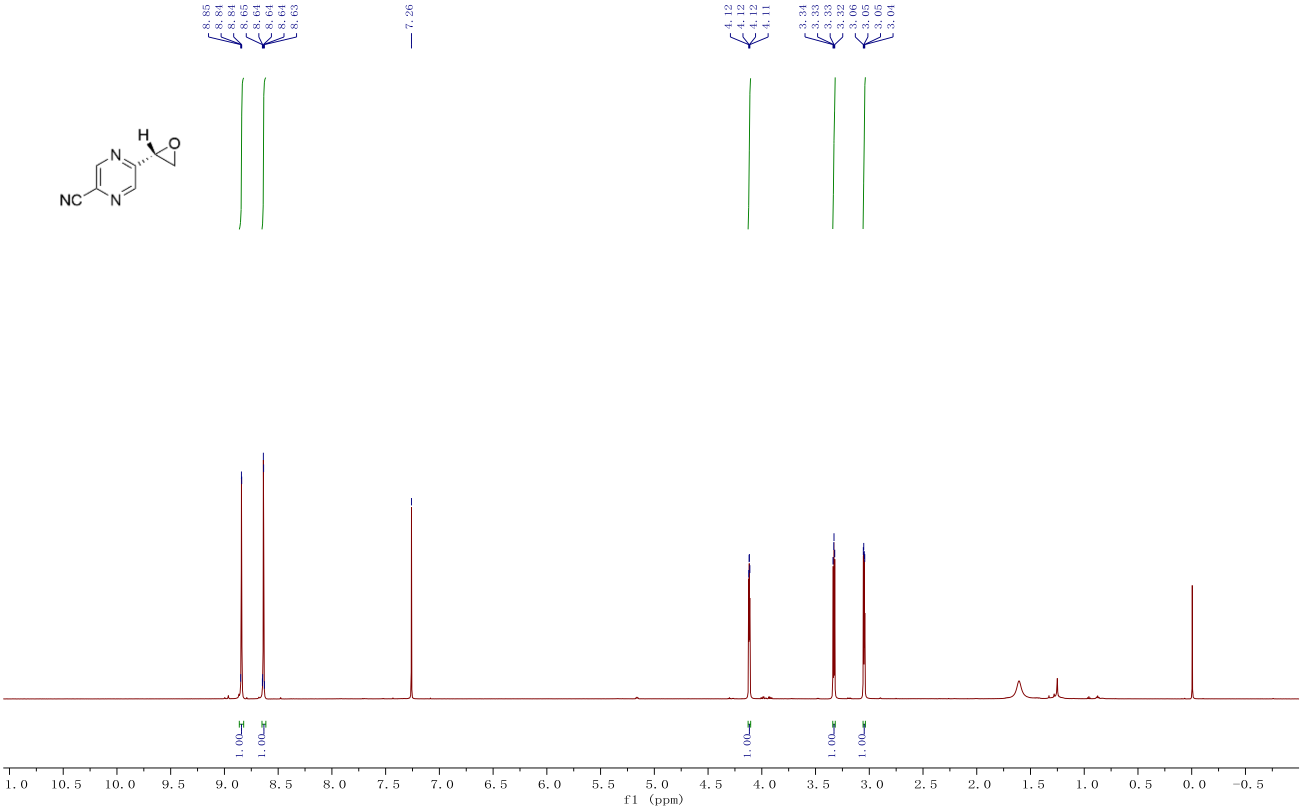

Supplementary Figure 452. ^{13}C NMR spectrum of compound **6j** (150 MHz, $CDCl_3$)

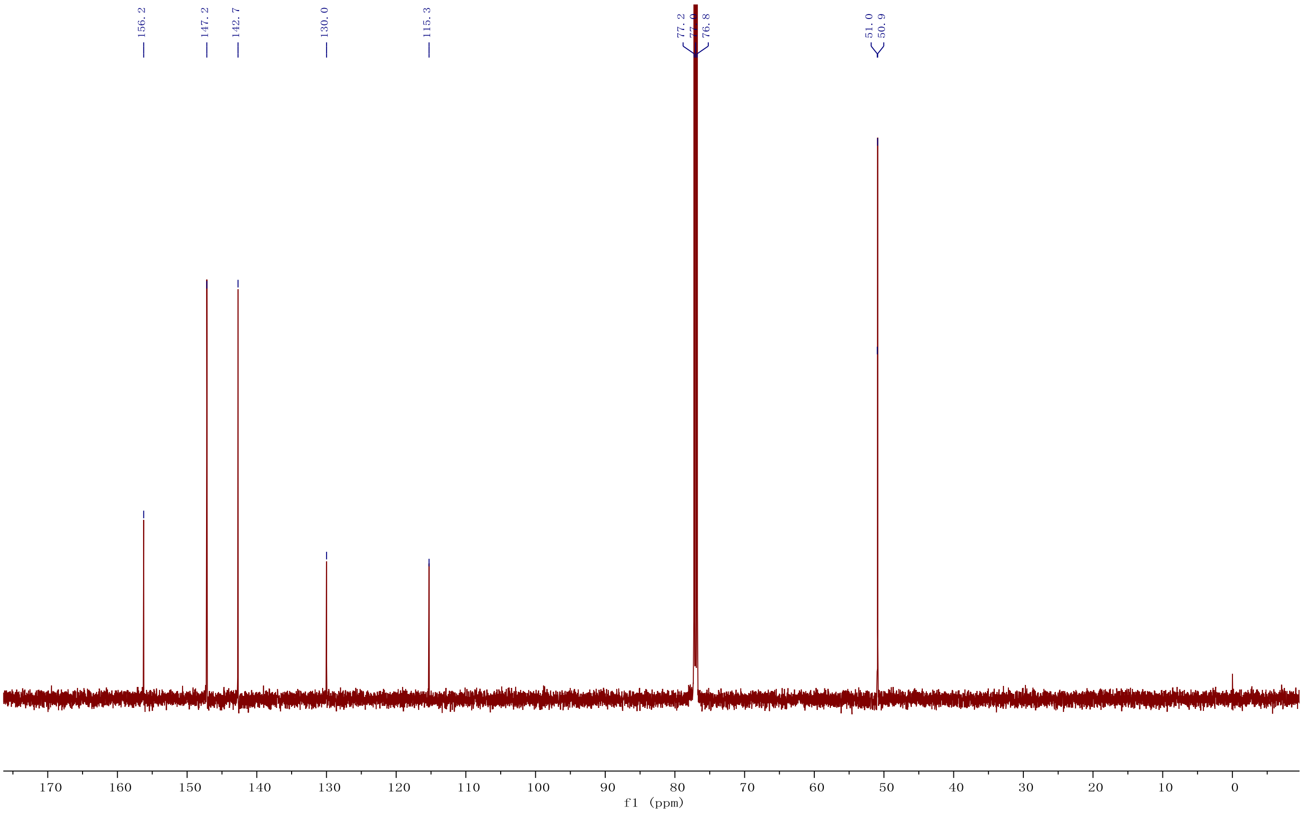

Supplementary Figure 453. ^{1}H NMR spectrum of compound **6k** (600 MHz, $CDCl_3$)

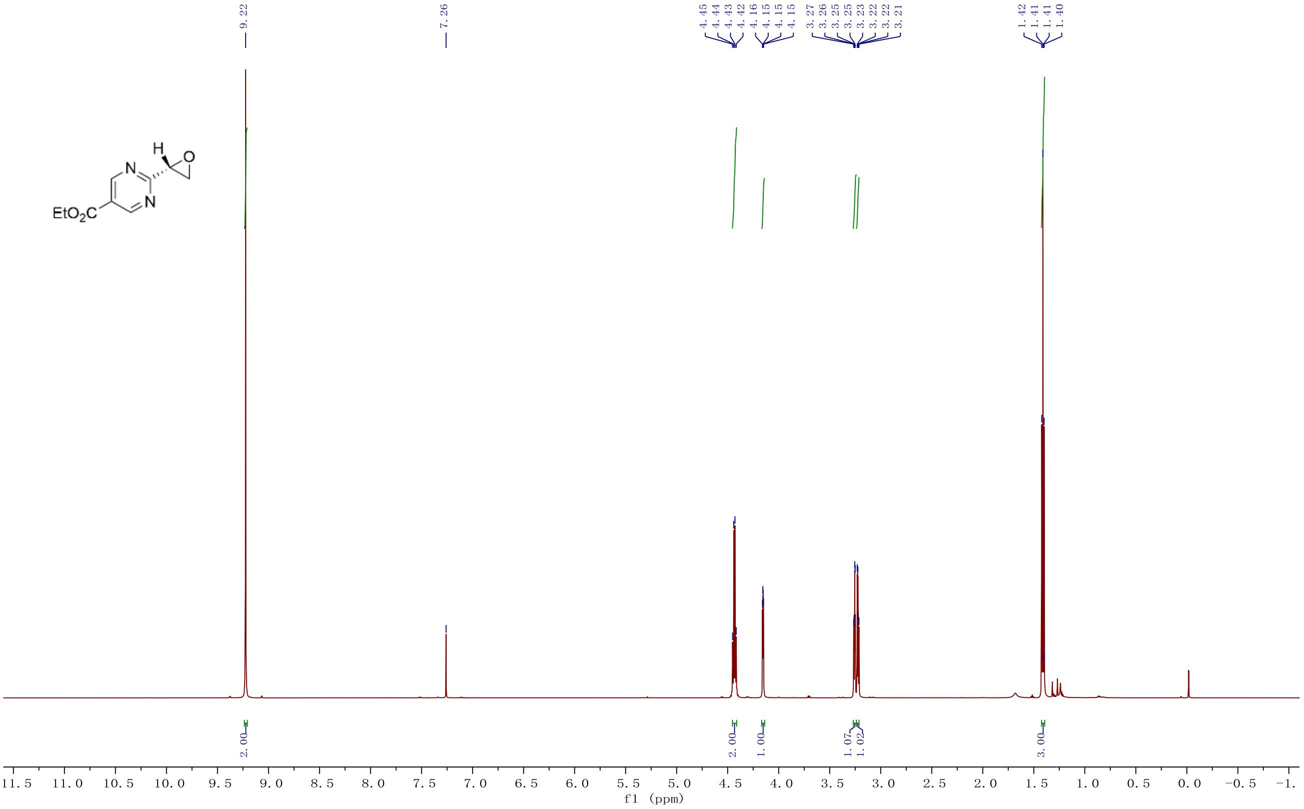

Supplementary Figure 454. ^{13}C NMR spectrum of compound**6k** (150 MHz, $CDCl_3$)

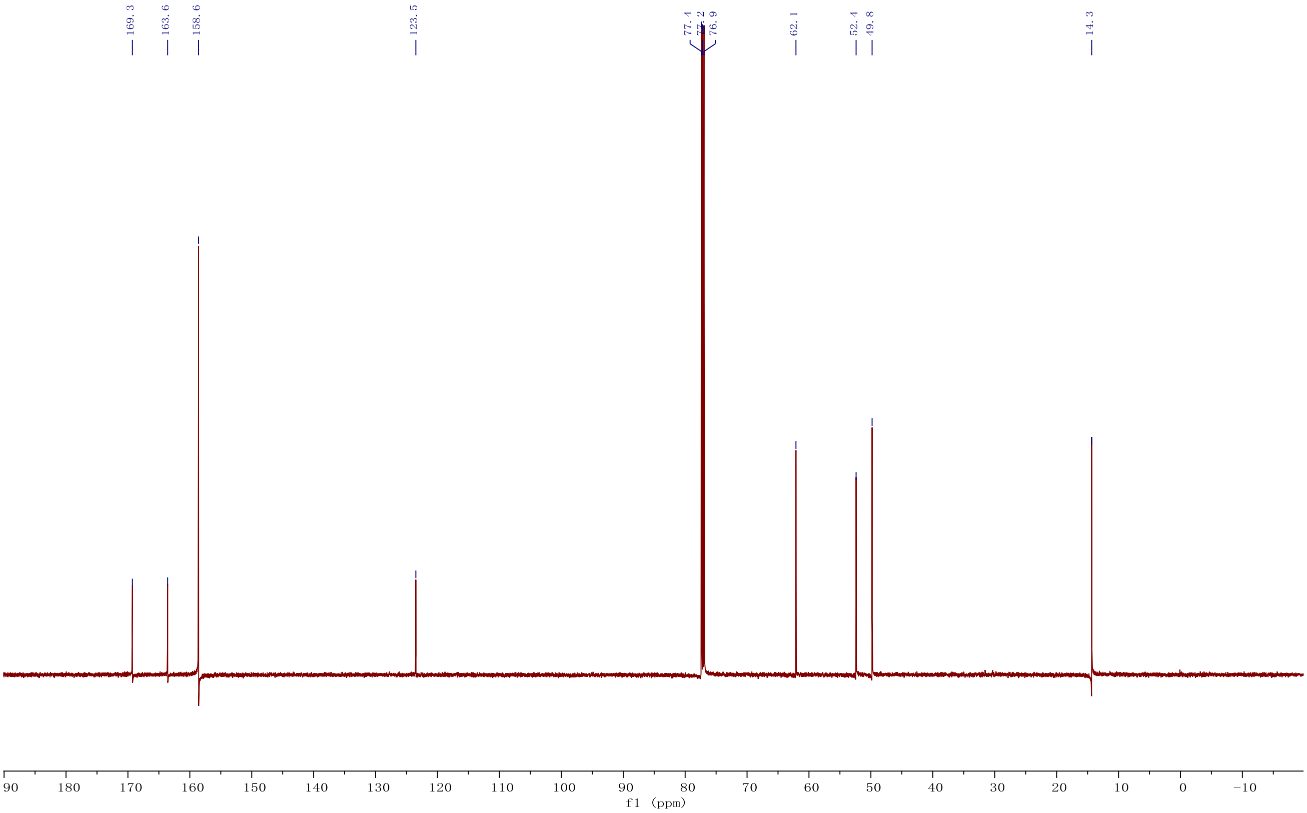

Supplementary Figure 455. ^{1}H NMR spectrum of compound **6l** (400 MHz, $CDCl_3$)

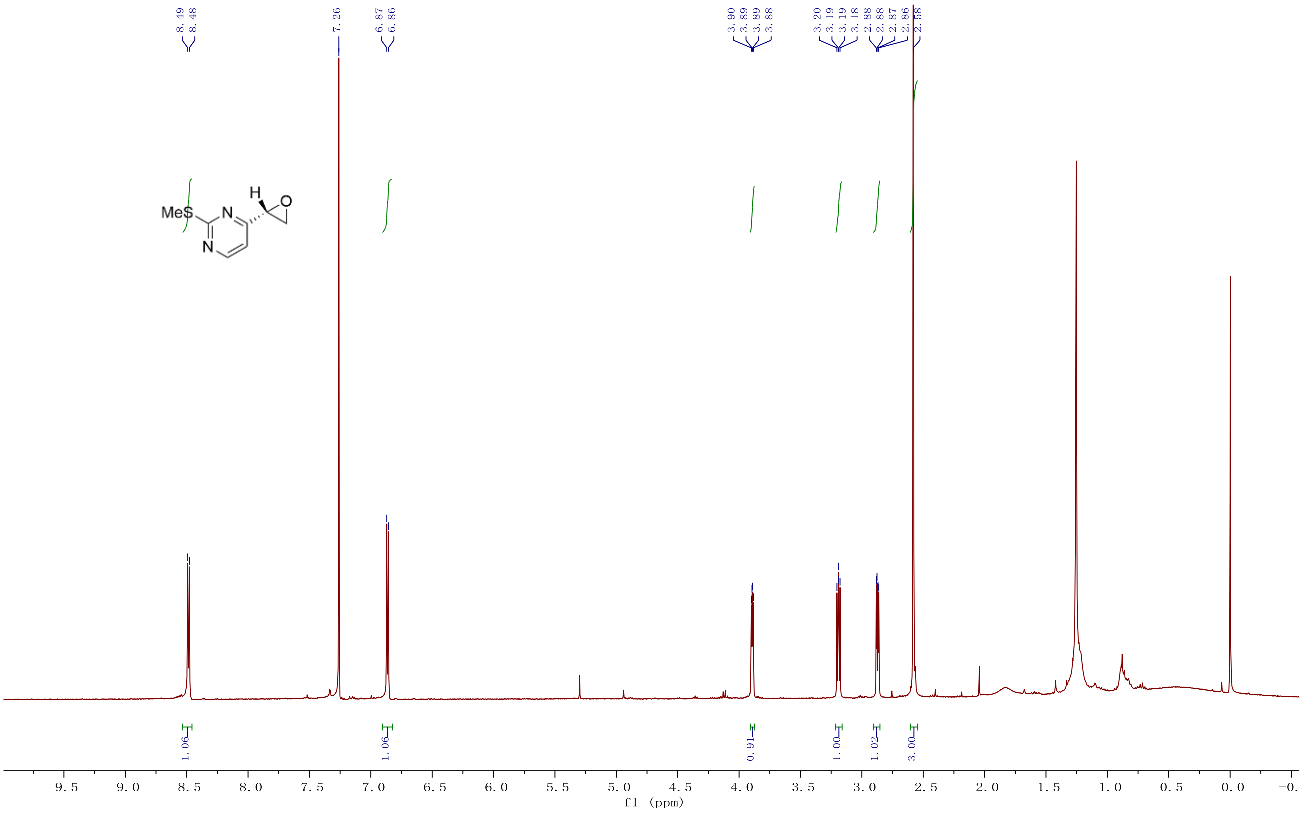

Supplementary Figure 456. ^{1}H NMR spectrum of compound **6l** (400 MHz, $CDCl_3$)

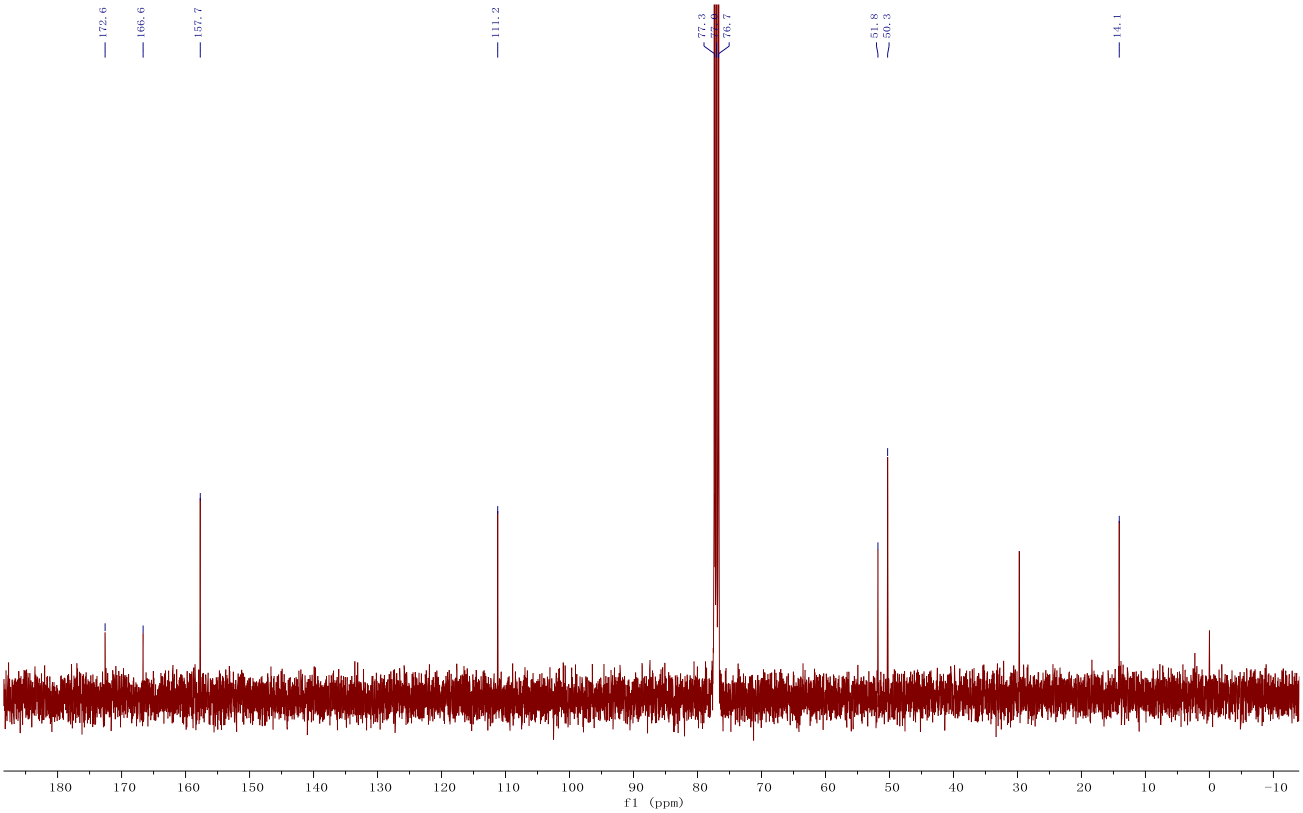

Supplementary Figure 457. ^{1}H NMR spectrum of compound **6m** (600 MHz, $CDCl_3$)

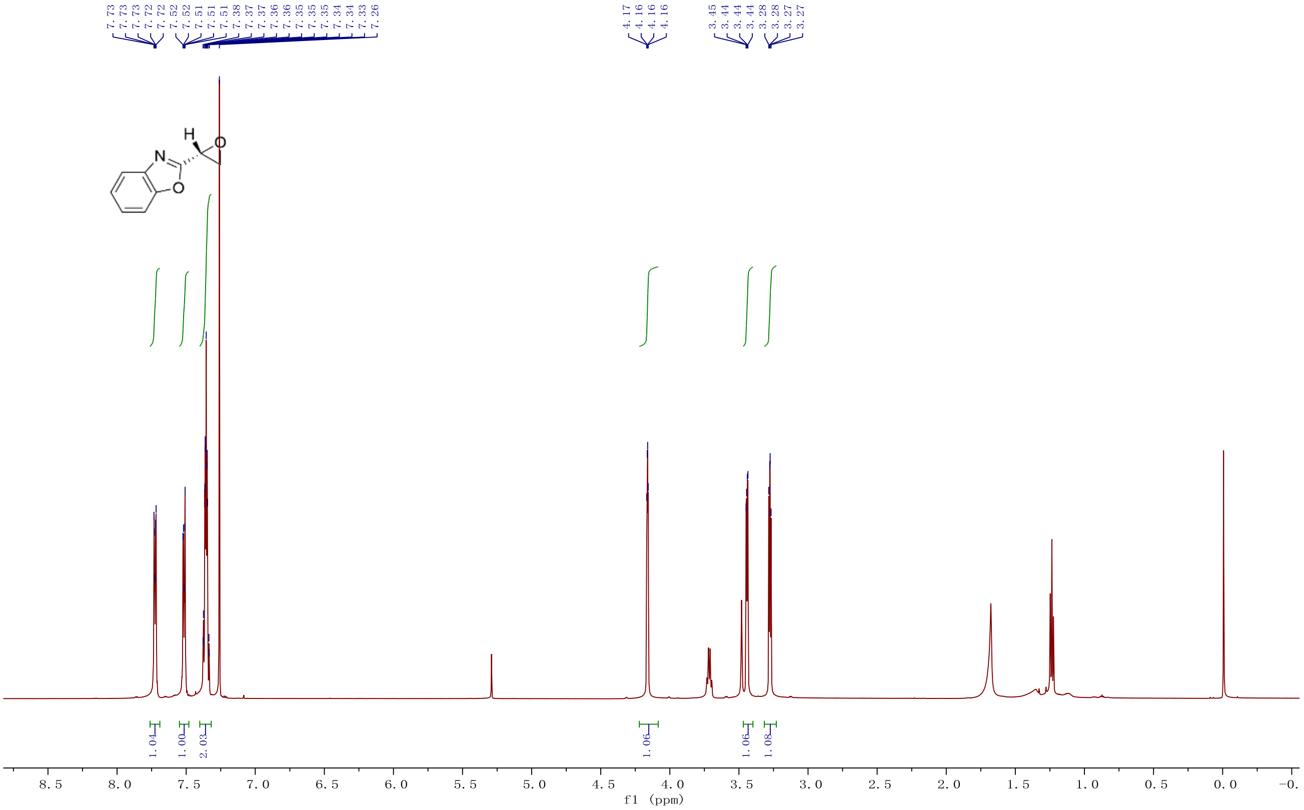

Supplementary Figure 458. ^{13}C NMR spectrum of compound **6m** (150 MHz, $CDCl_3$)

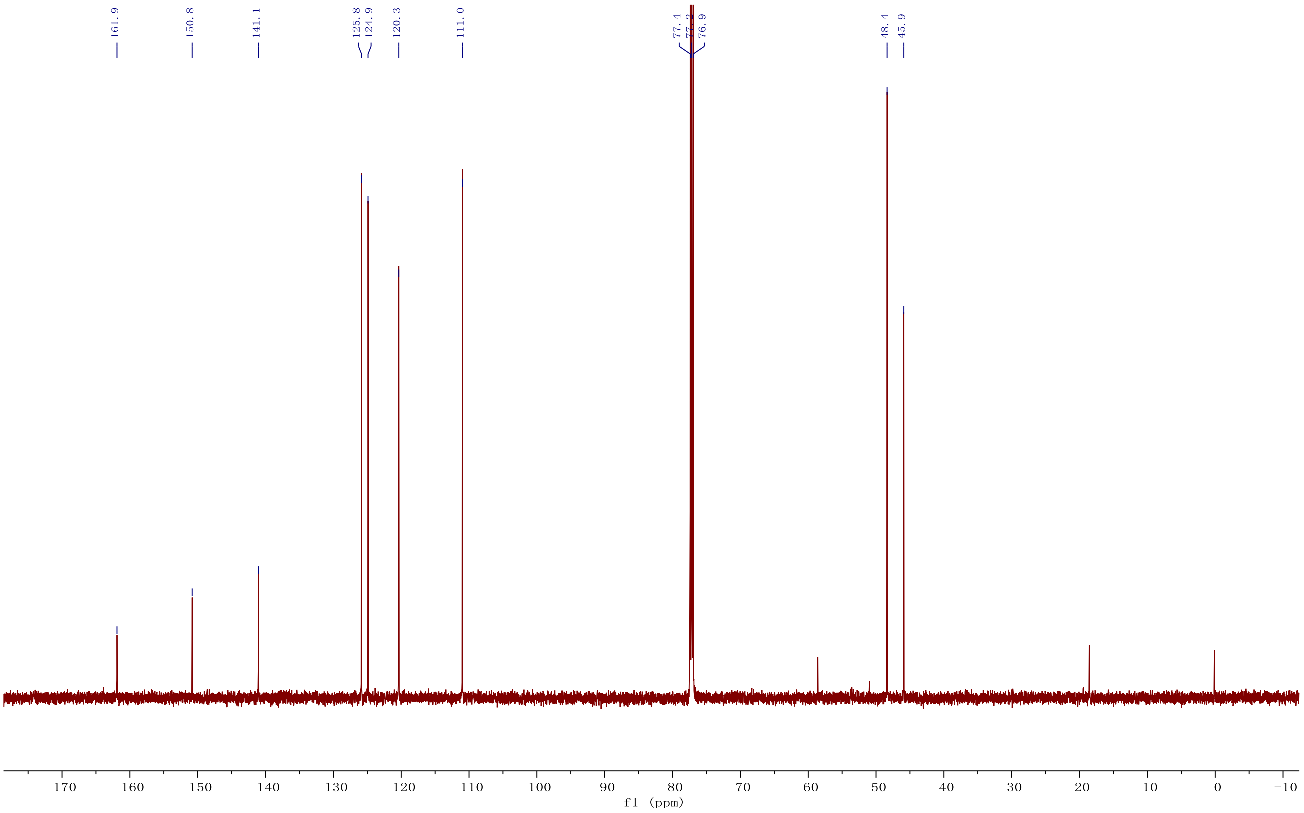

Supplementary Figure 459. ^{1}H NMR spectrum of compound **6n** (400 MHz, $CDCl_3$)

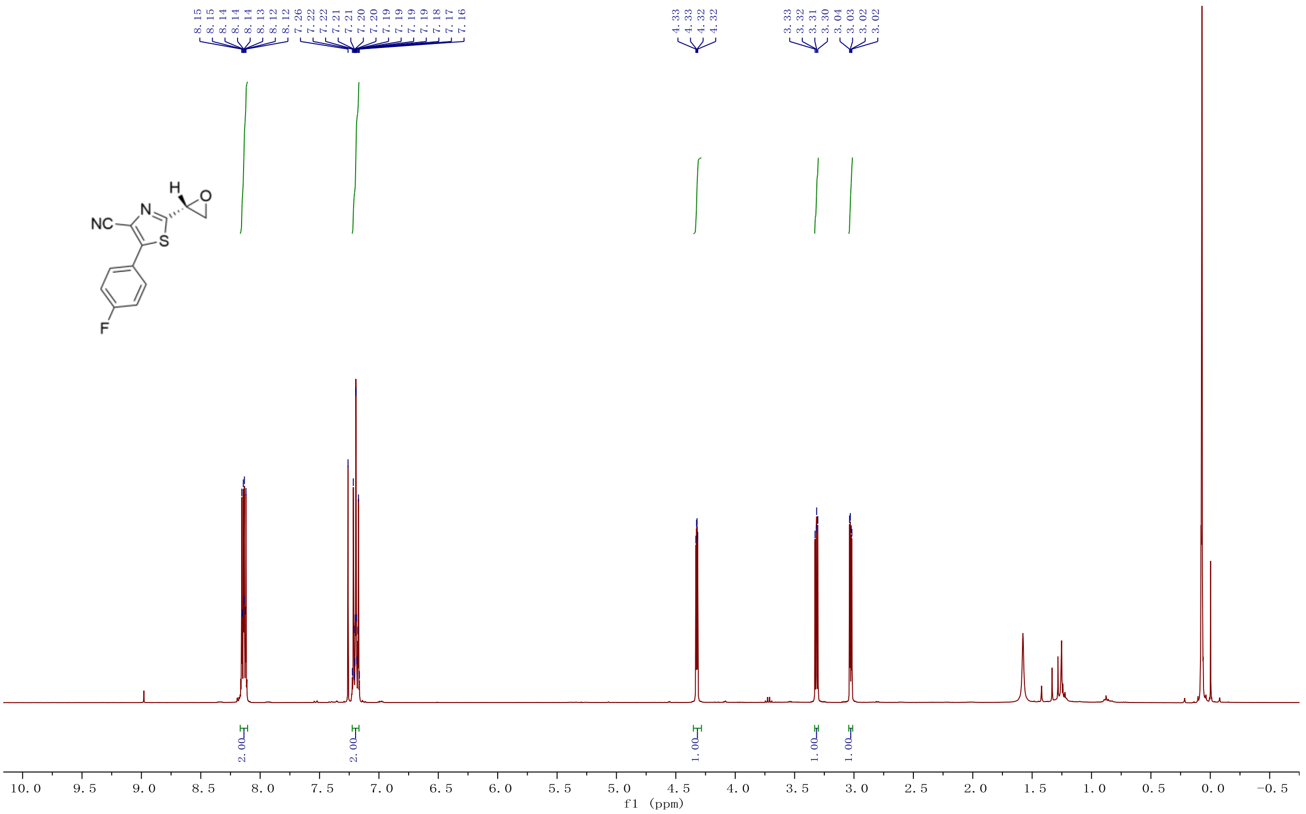

Supplementary Figure 460. ^{13}C NMR spectrum of compound **6n** (150 MHz, $CDCl_3$)

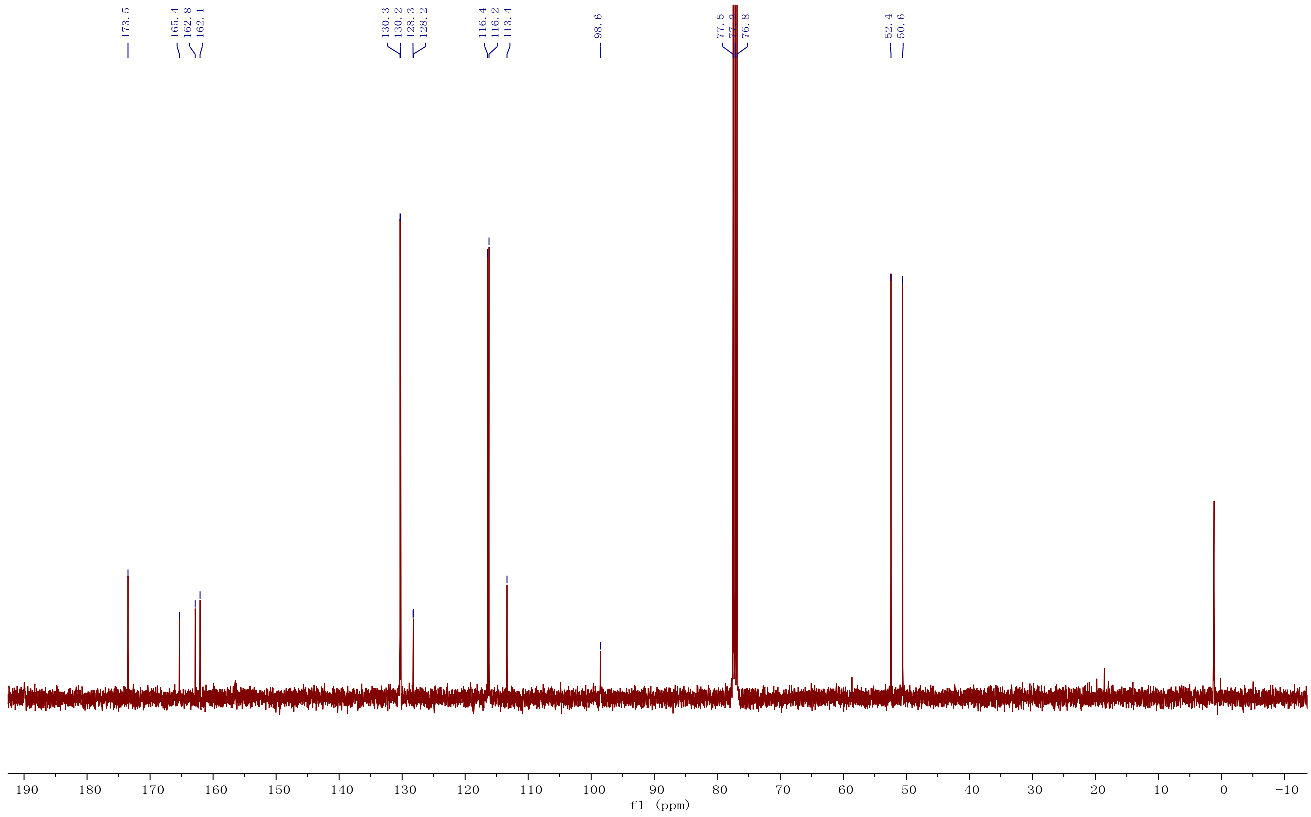

Supplementary Figure 461. ^{19}F NMR spectrum of compound **6n** (376 MHz, $CDCl_3$)

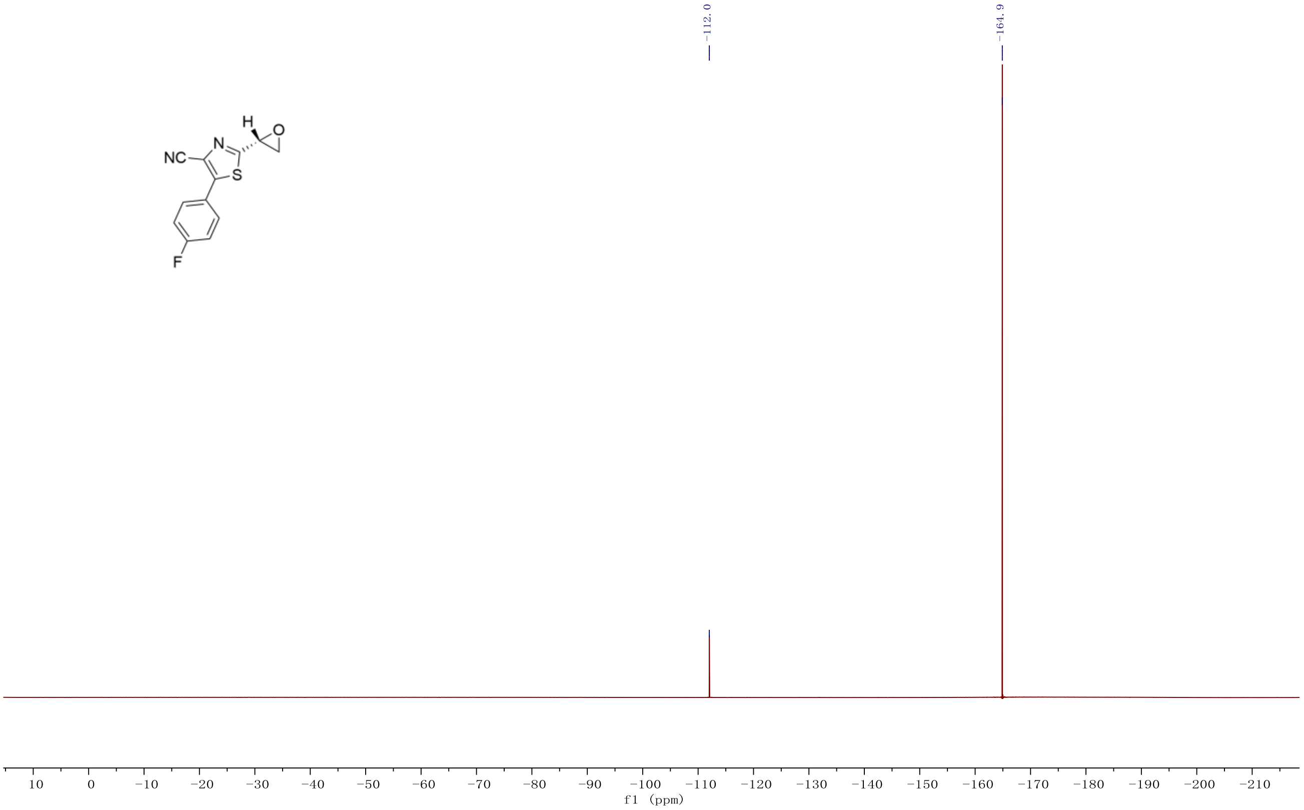

Supplementary Figure 462. ^{1}H NMR spectrum of compound **6o** (400 MHz, $CDCl_3$)

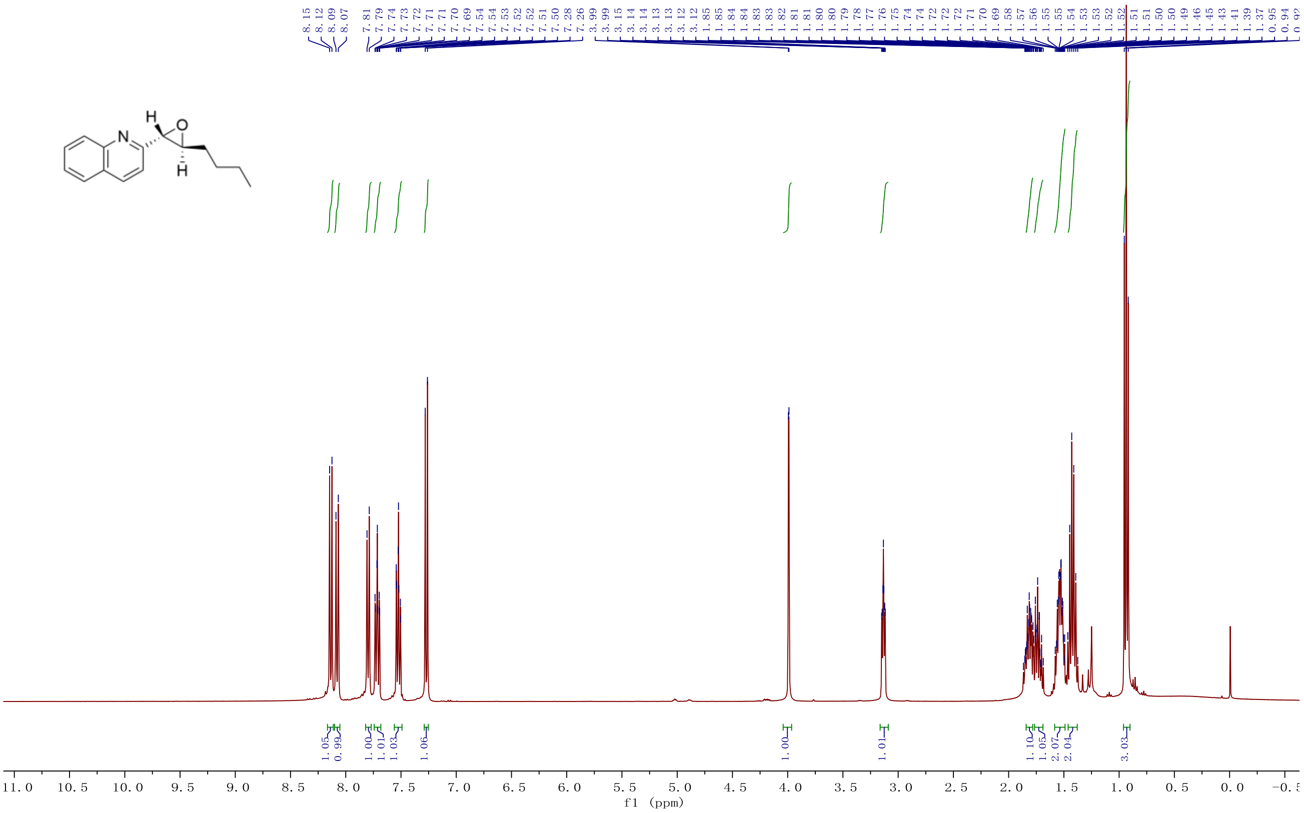

Supplementary Figure 463. ^{13}C NMR spectrum of compound **6o** (100 MHz, $CDCl_3$)

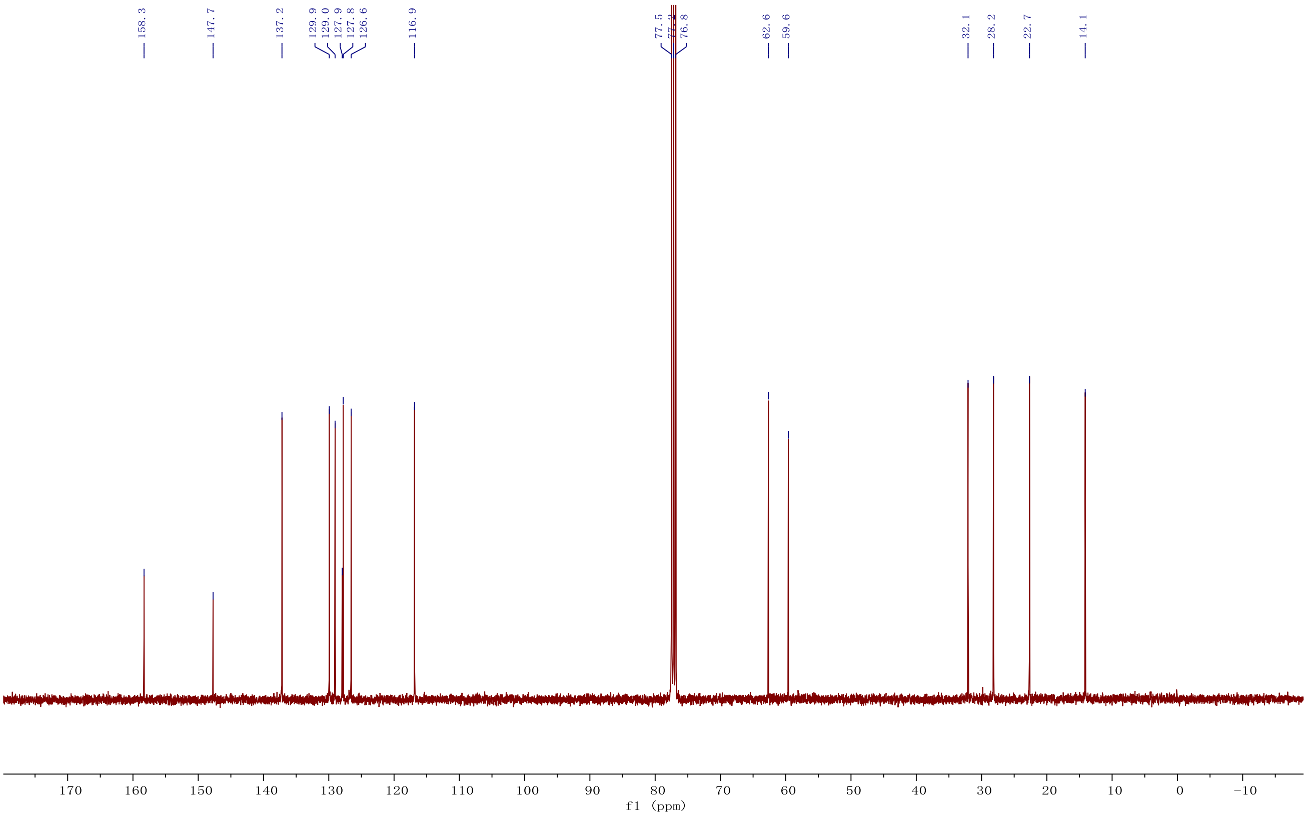

Supplementary Figure 464. ^{1}H NMR spectrum of compound **6p** (400 MHz, $CDCl_3$)

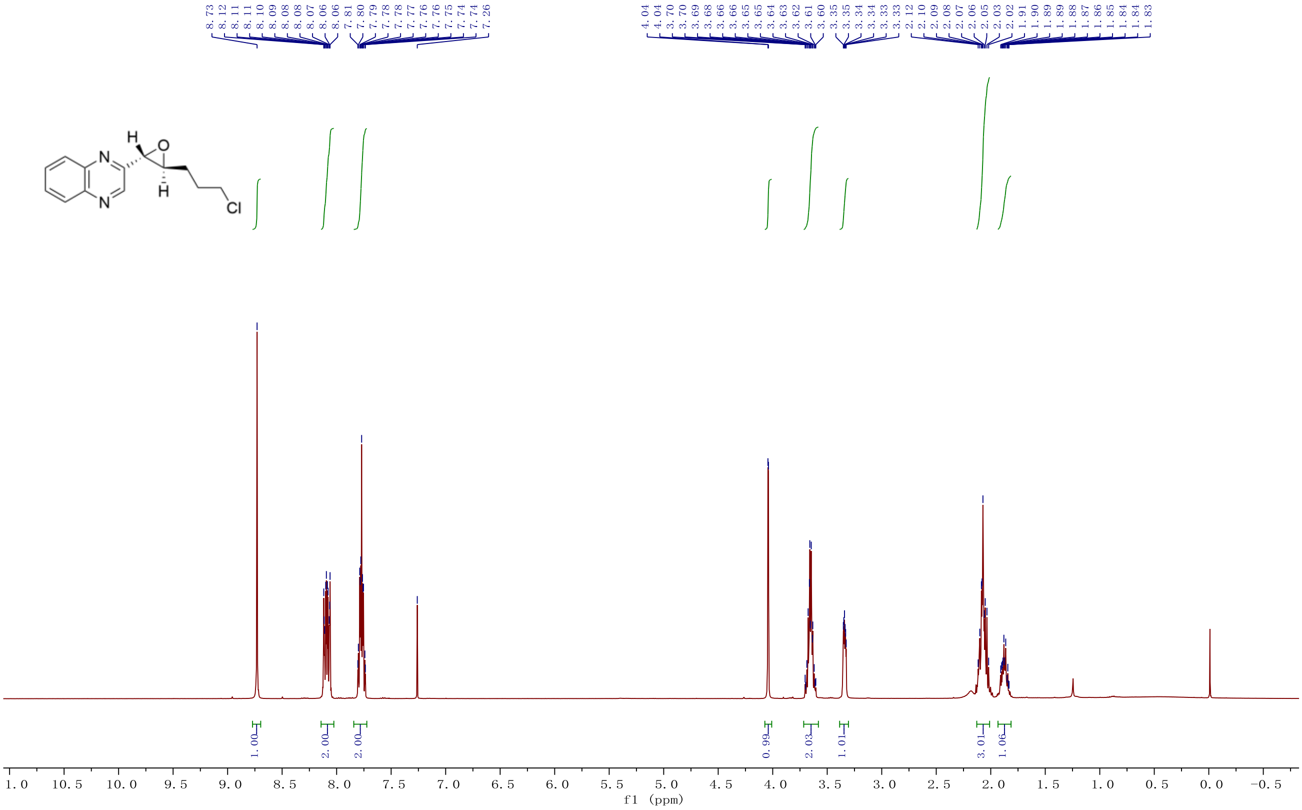

Supplementary Figure 465. ^{13}C NMR spectrum of compound **6p** (100 MHz, $CDCl_3$)

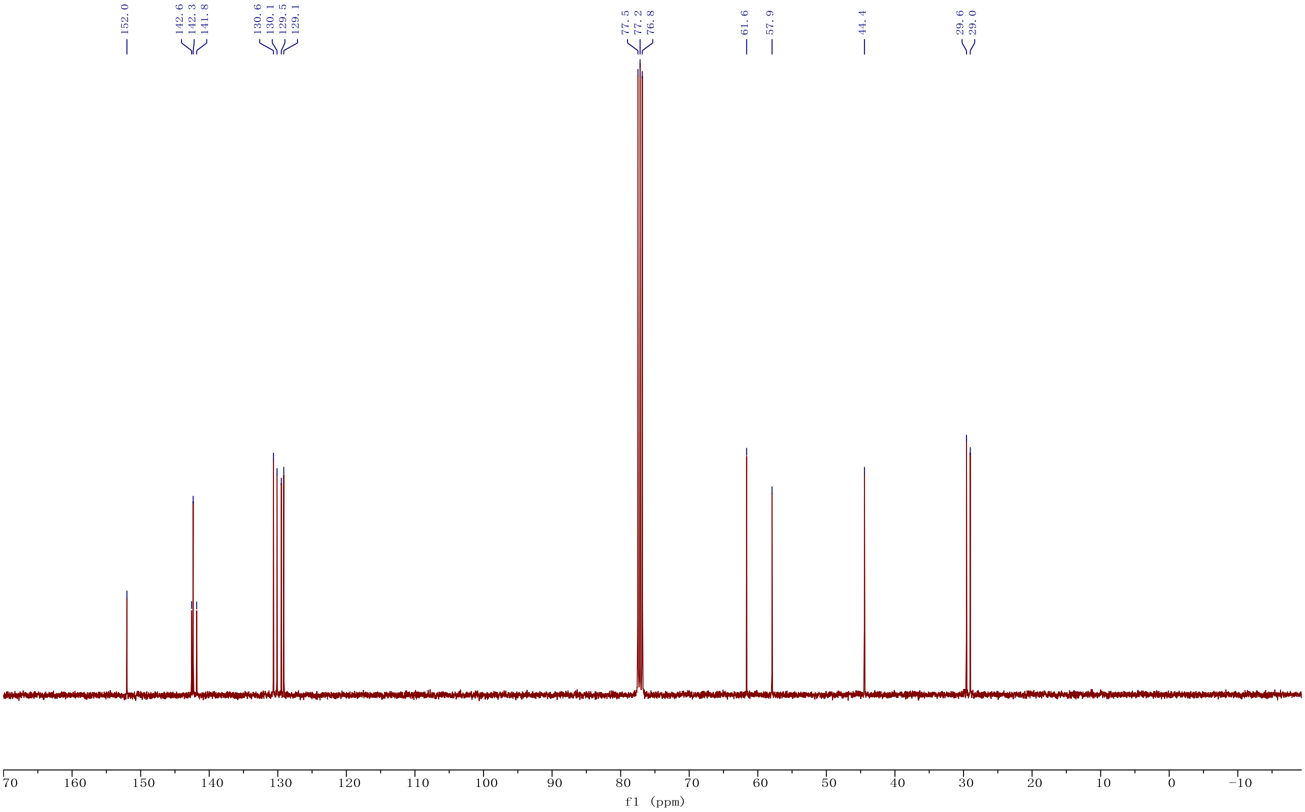

Supplementary Figure 466. ^{1}H NMR spectrum of compound **6q** (400 MHz, $CDCl_3$)

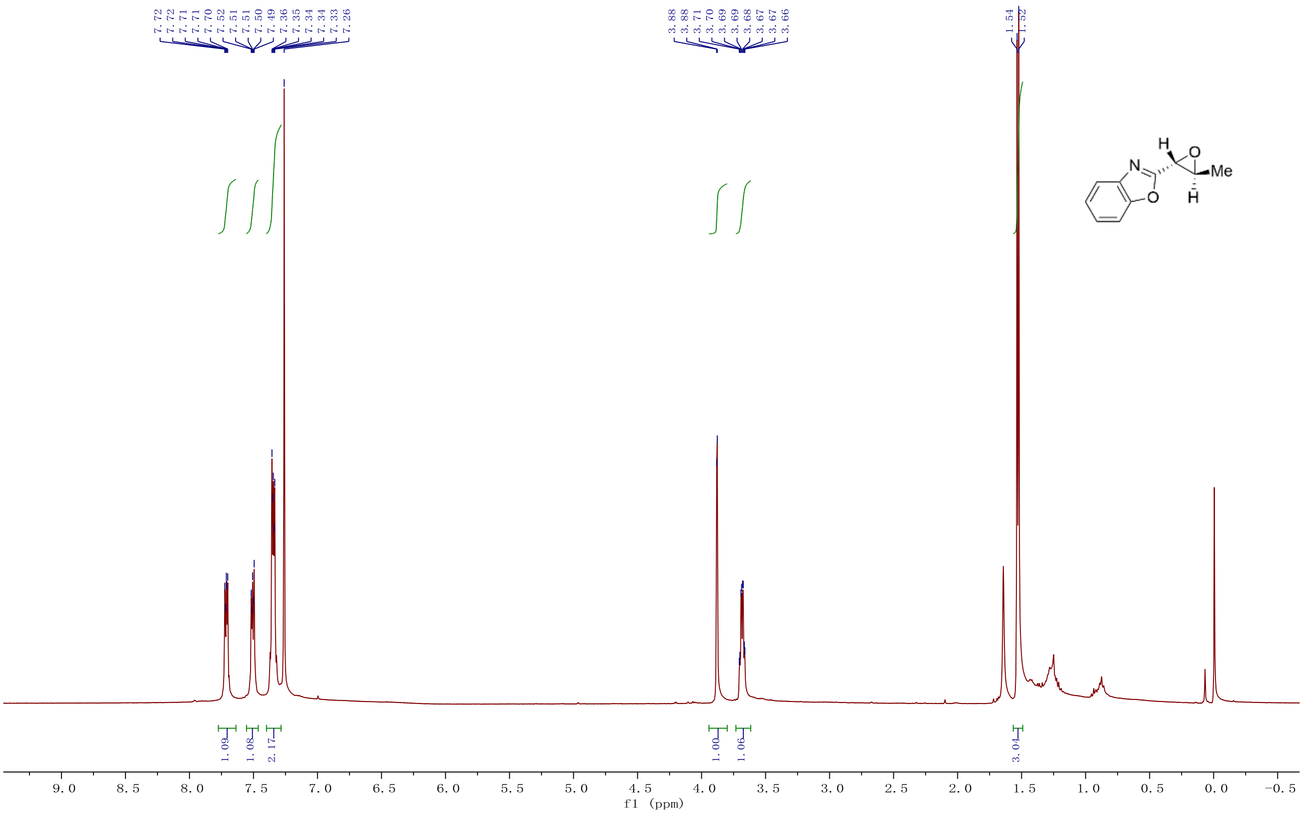

Supplementary Figure 467. ^{13}C NMR spectrum of compound **6q** (100 MHz, $CDCl_3$)

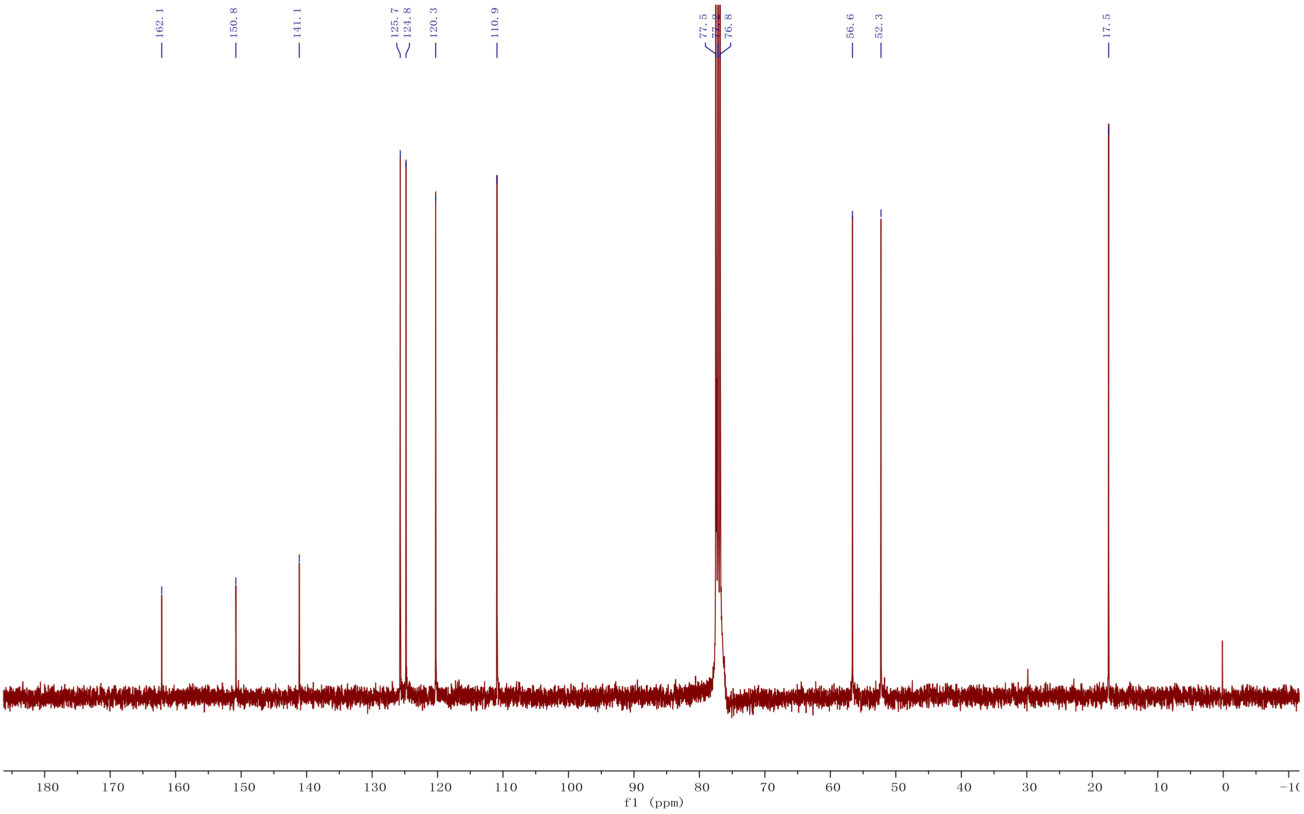

Supplementary Figure 468. ^{1}H NMR spectrum of compound **6r** (400 MHz, $CDCl_3$)

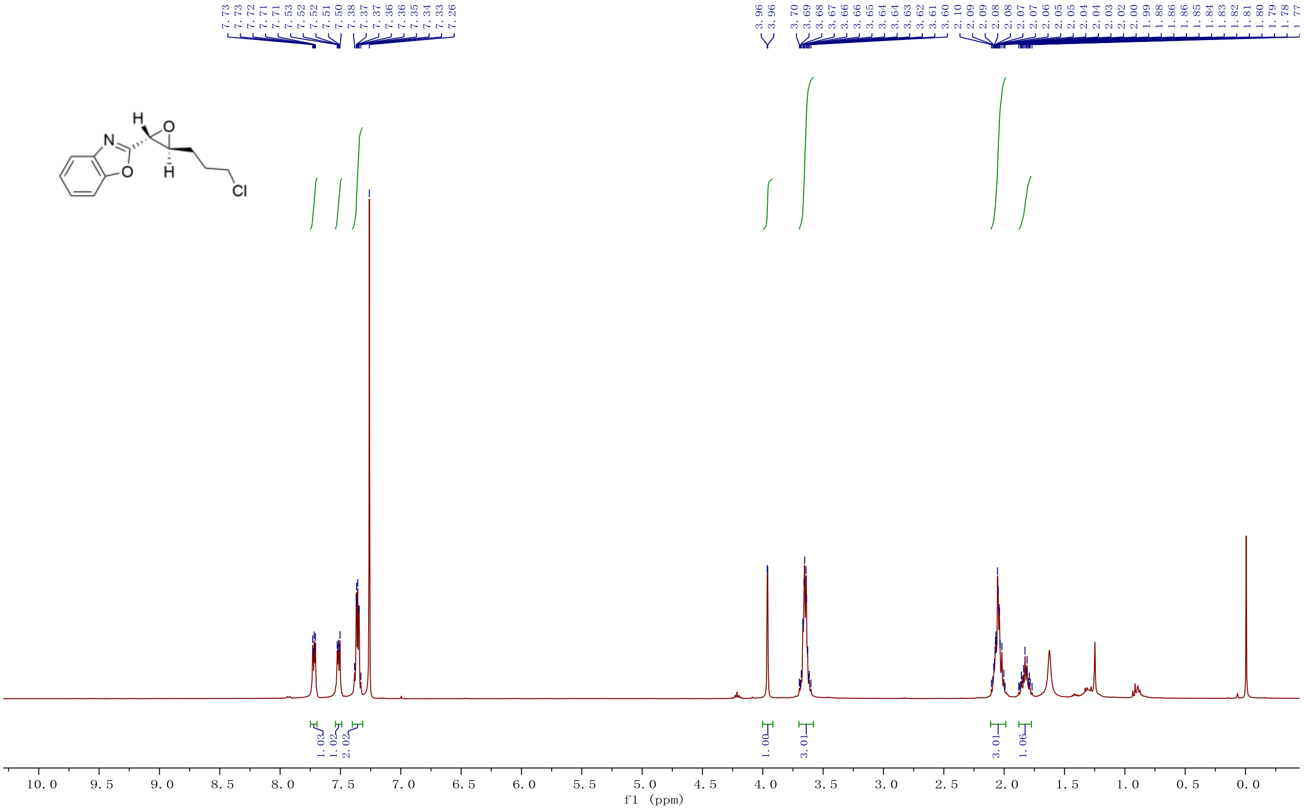

Supplementary Figure 469. ^{13}C NMR spectrum of compound **6r** (100 MHz, $CDCl_3$)

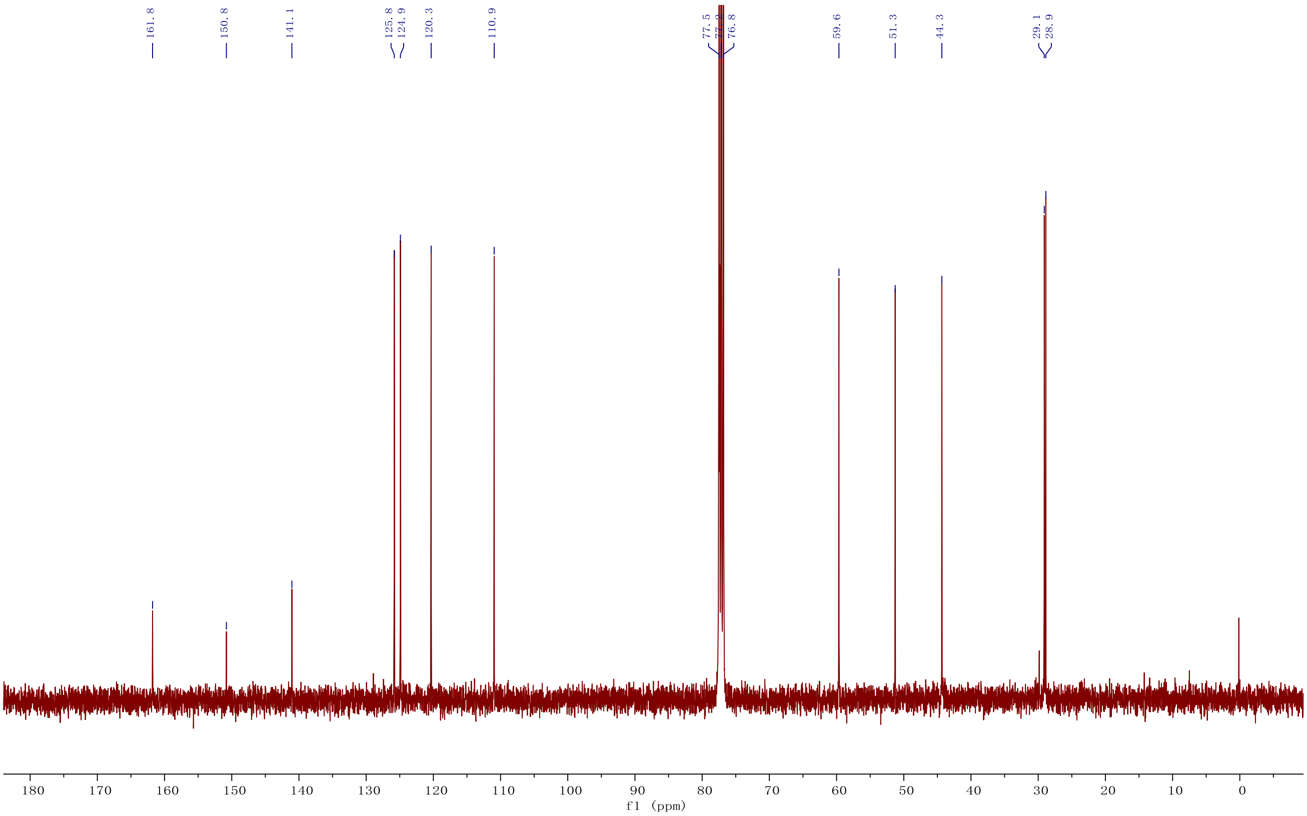

Supplementary Figure 470. ^{1}H NMR spectrum of compound **6s** (600 MHz, $CDCl_3$)

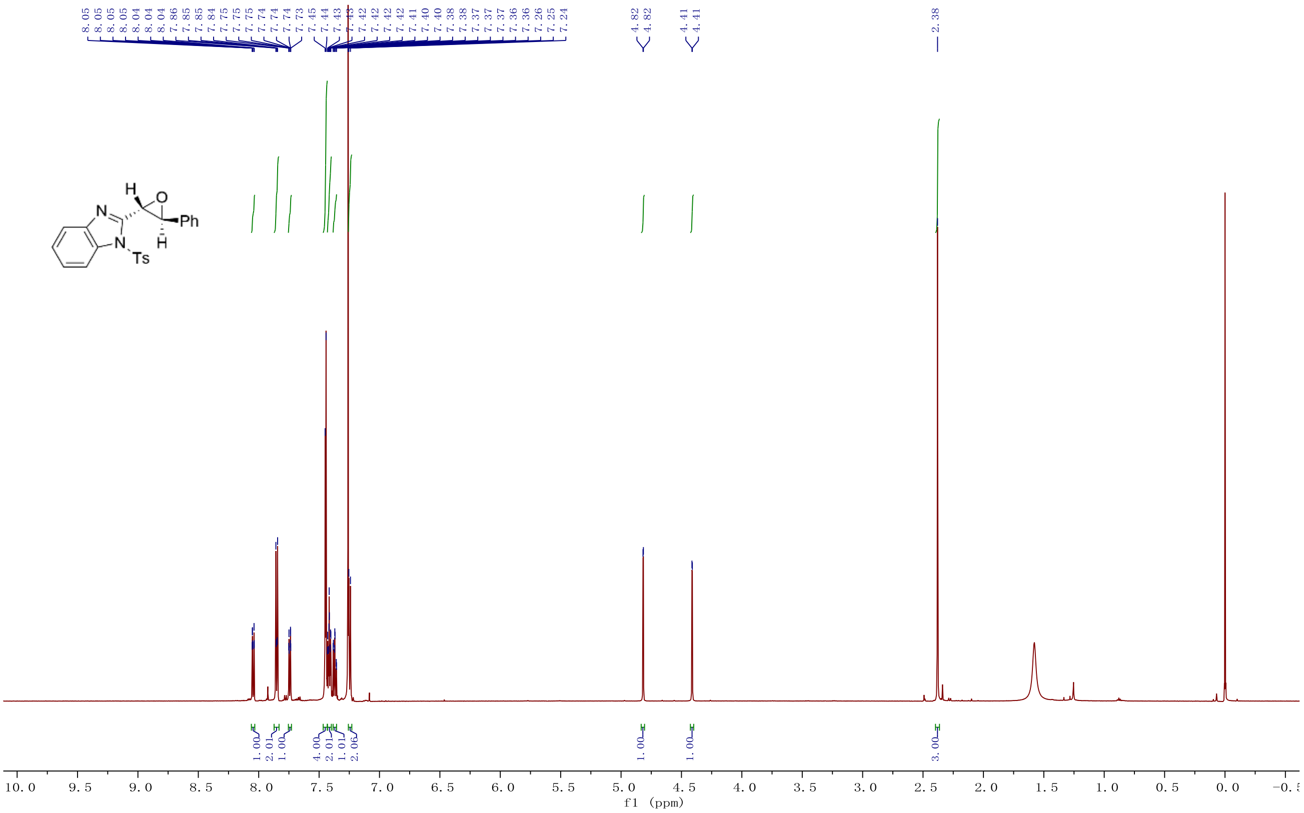

Supplementary Figure 471. ^{13}C NMR spectrum of compound **6s** (150 MHz, $CDCl_3$)

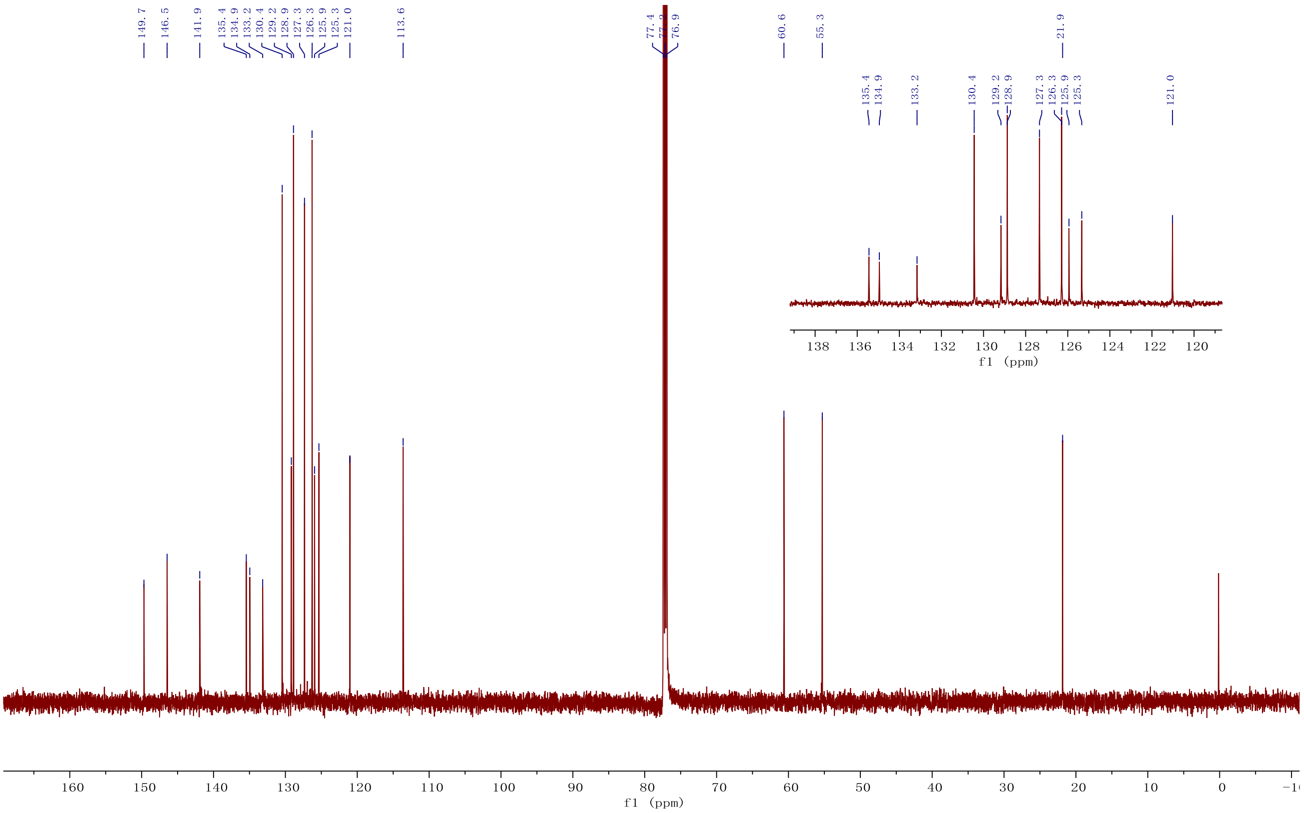

Supplementary Figure 472. ^{1}H NMR spectrum of compound **6u** (400 MHz, $CDCl_3$)

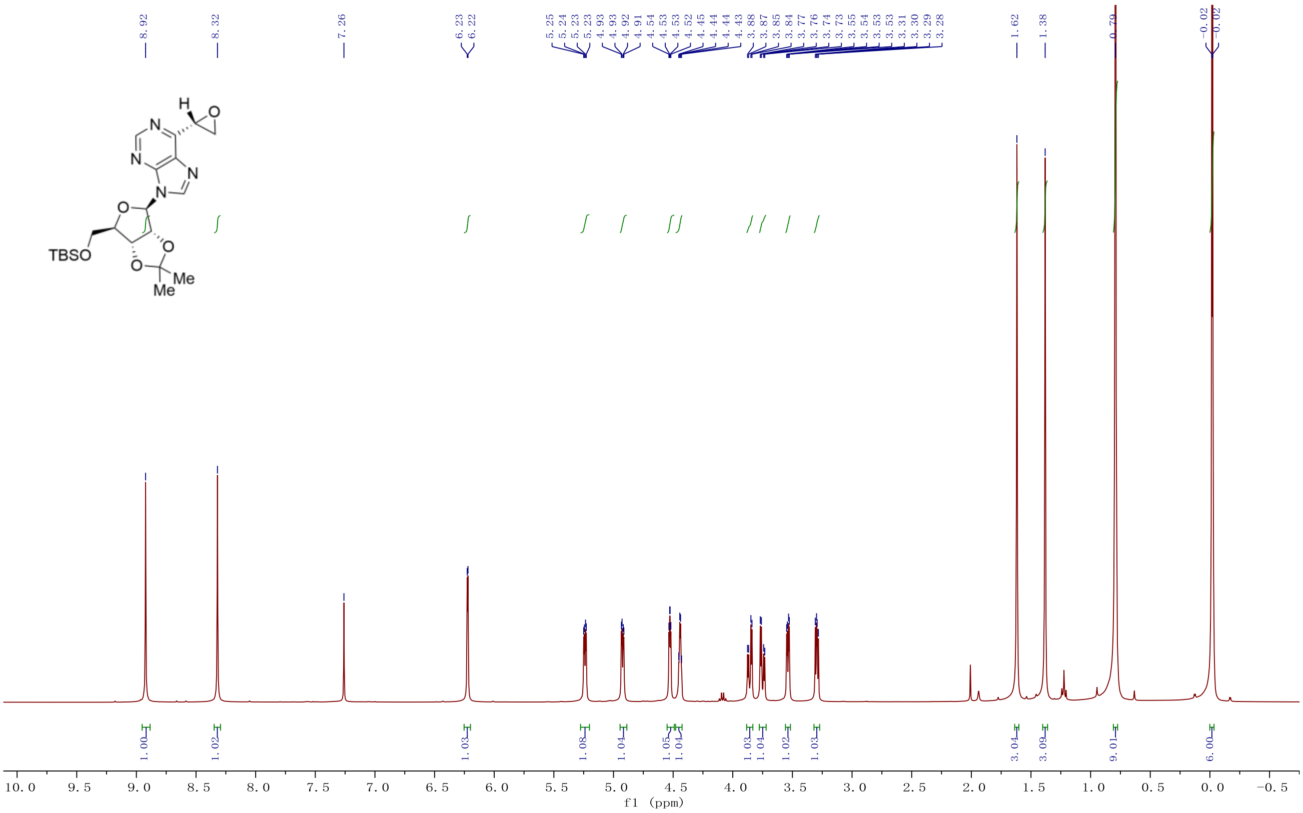

Supplementary Figure 473. ^{13}C NMR spectrum of compound **6u** (100 MHz, $CDCl_3$)

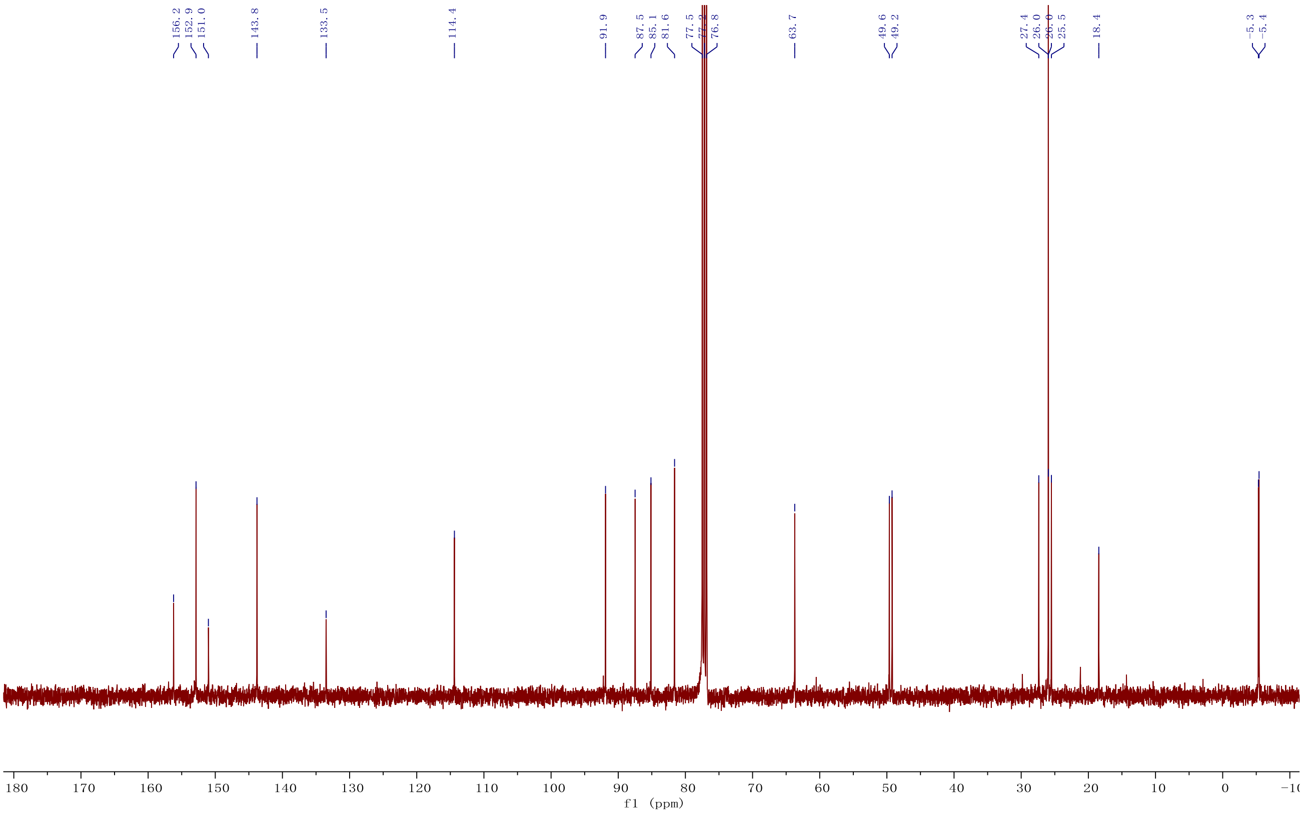

Supplementary Figure 474. ^{1}H NMR spectrum of compound **6v** (400 MHz, DMSO-d_6)

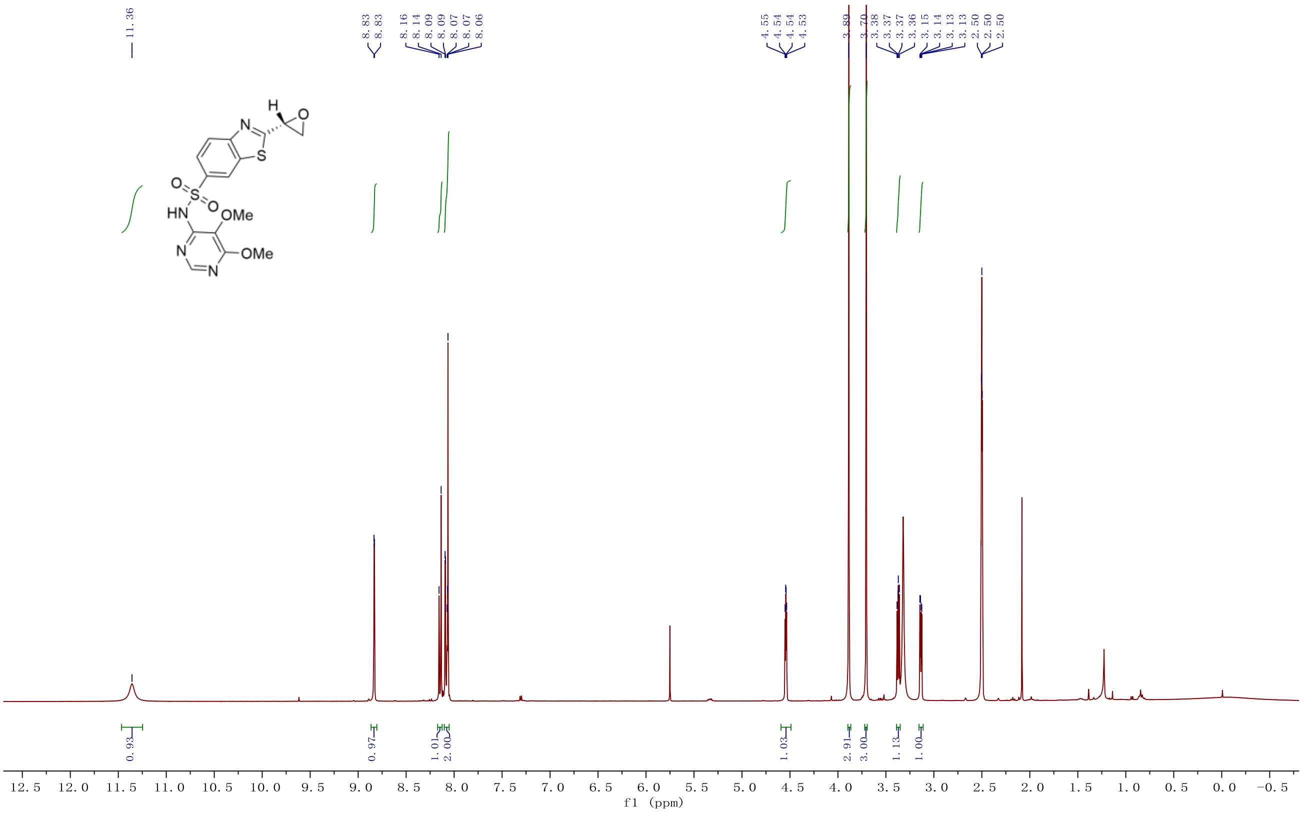

Supplementary Figure 475. ^{13}C NMR spectrum of compound **6v** (100 MHz, DMSO-d_6)

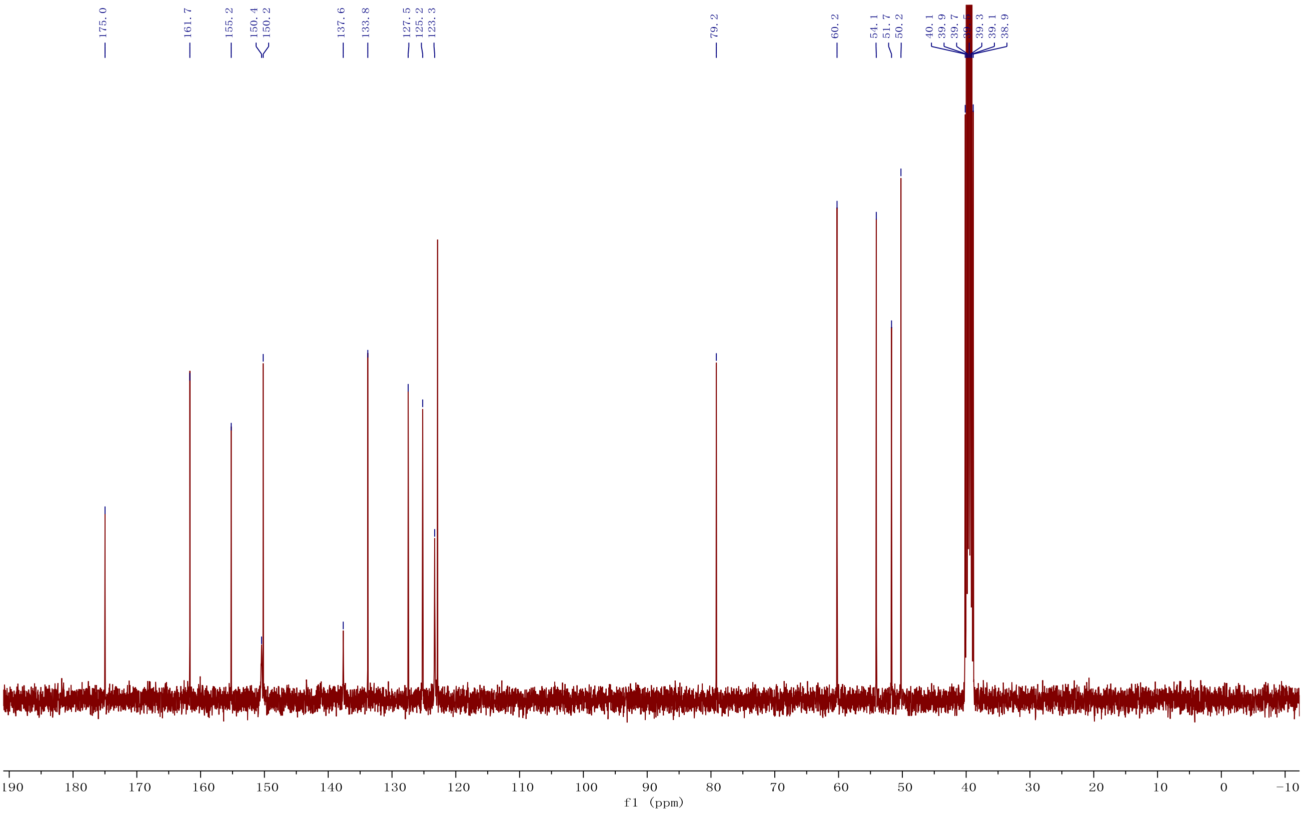

Supplementary Figure 476. ^{1}H NMR spectrum of compound **7a** (400 MHz, $CDCl_3$)

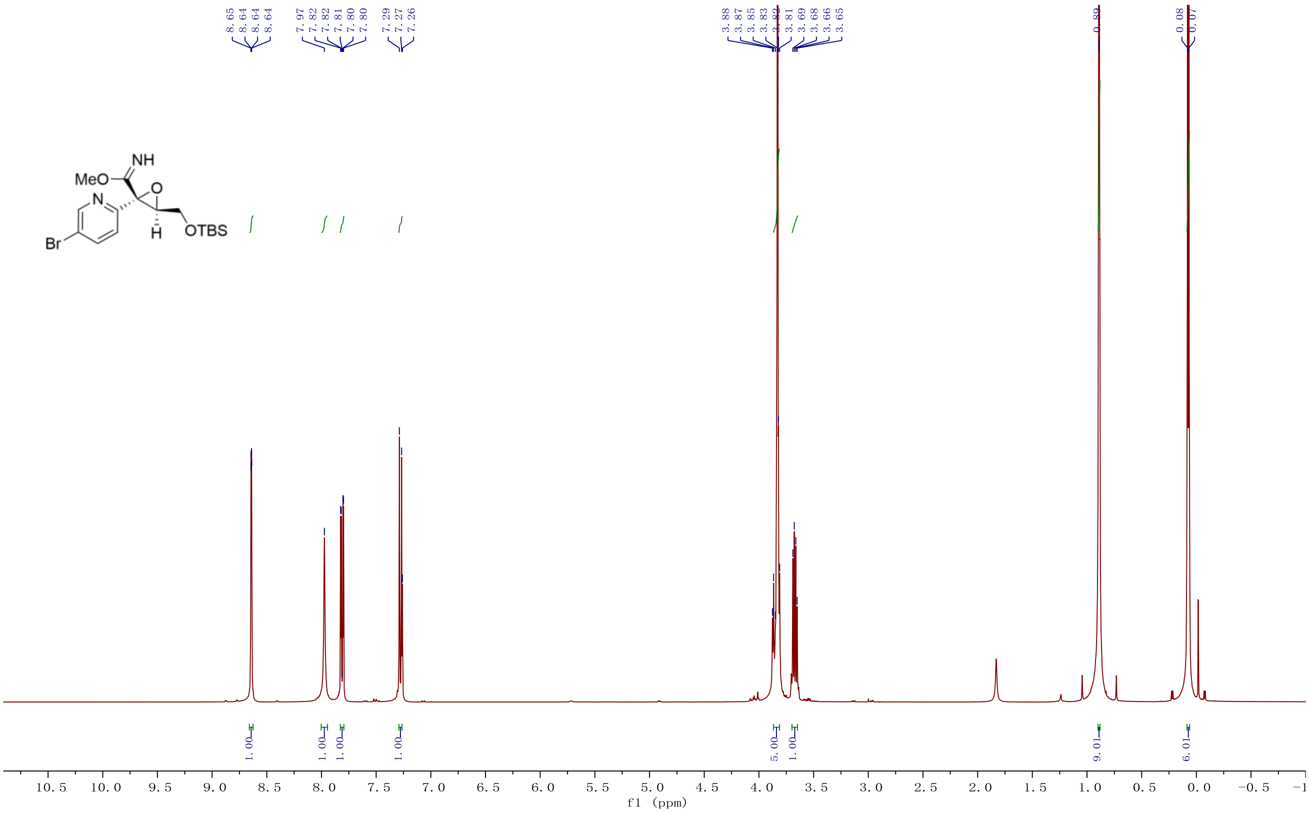

Supplementary Figure 477. ^{13}C NMR spectrum of compound **7a** (100 MHz, $CDCl_3$)

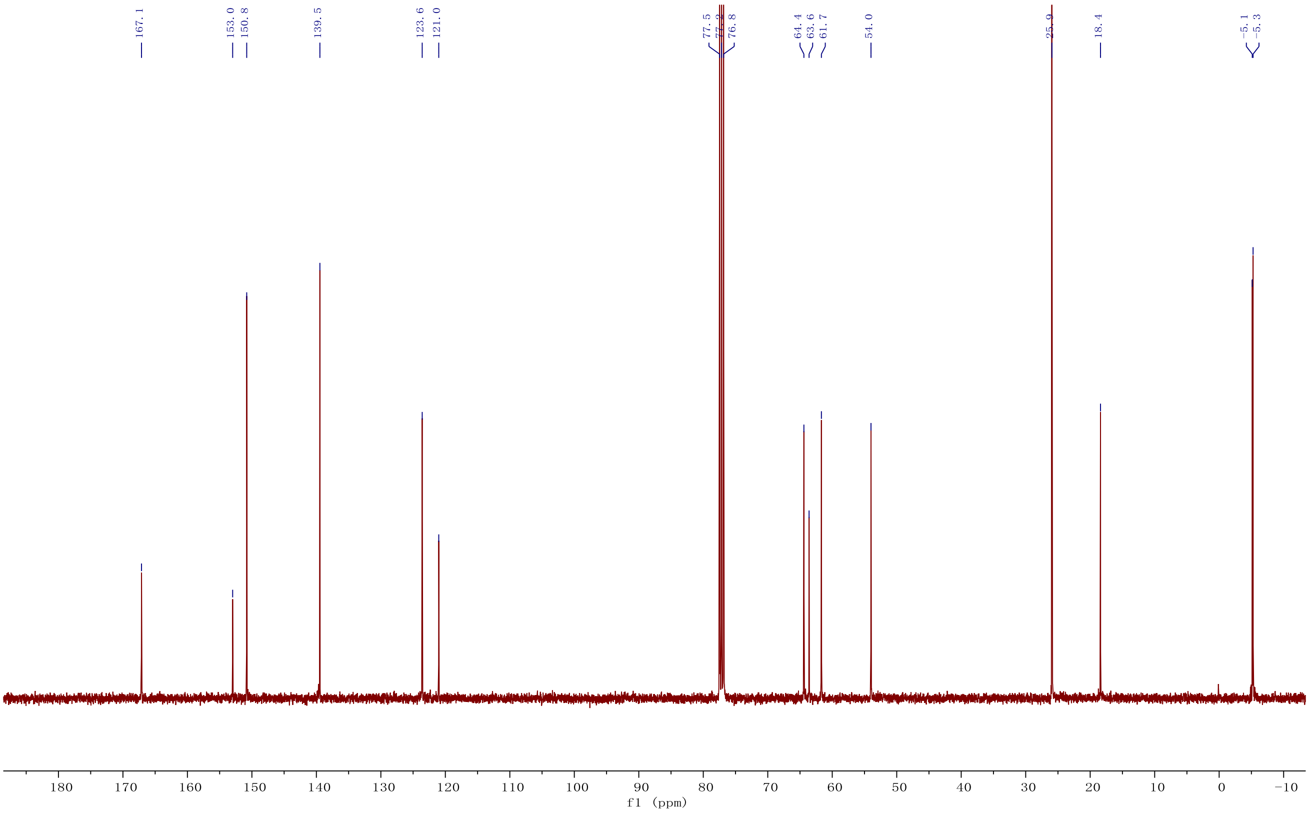

Supplementary Figure 478. ^{1}H NMR spectrum of compound **7b** (400 MHz, $CDCl_3$)

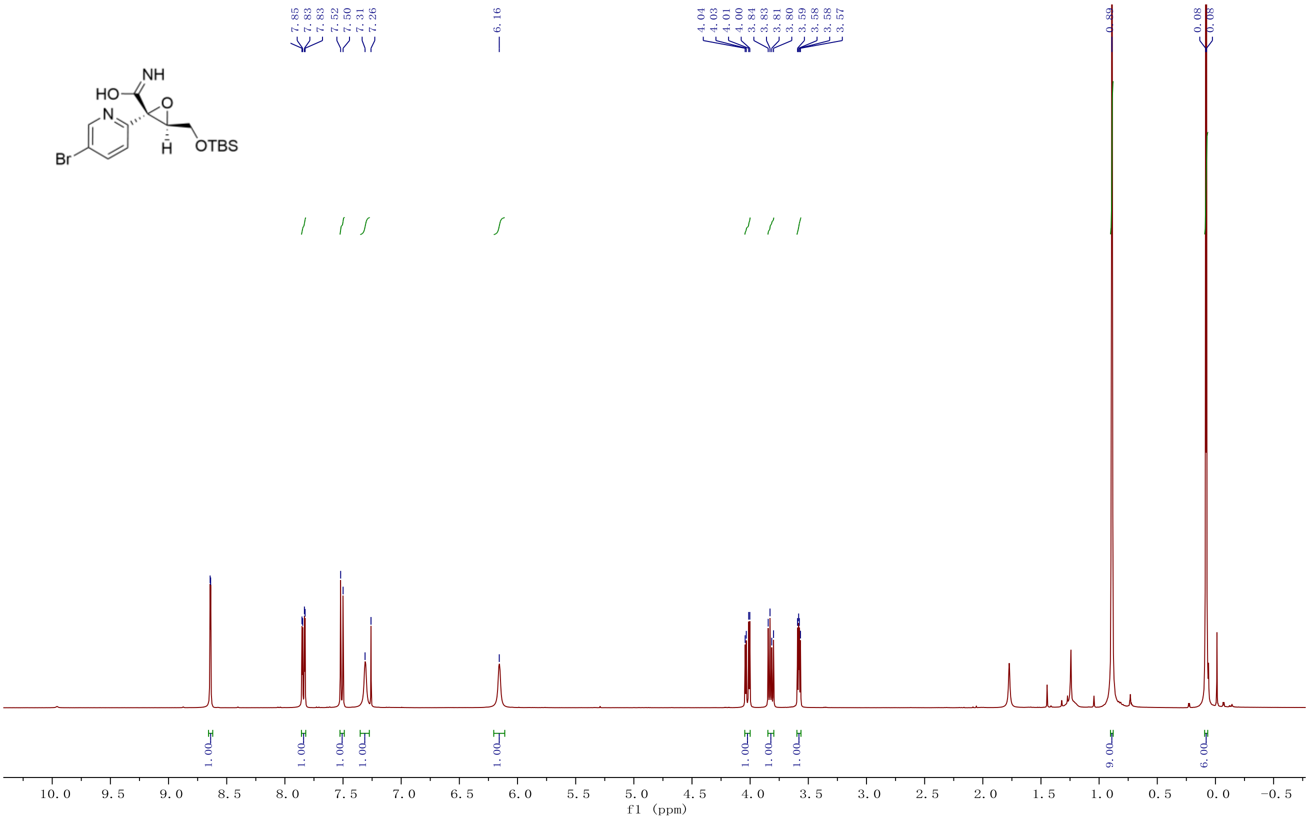

Supplementary Figure 479. ^{13}C NMR spectrum of compound **7b** (100 MHz, $CDCl_3$)

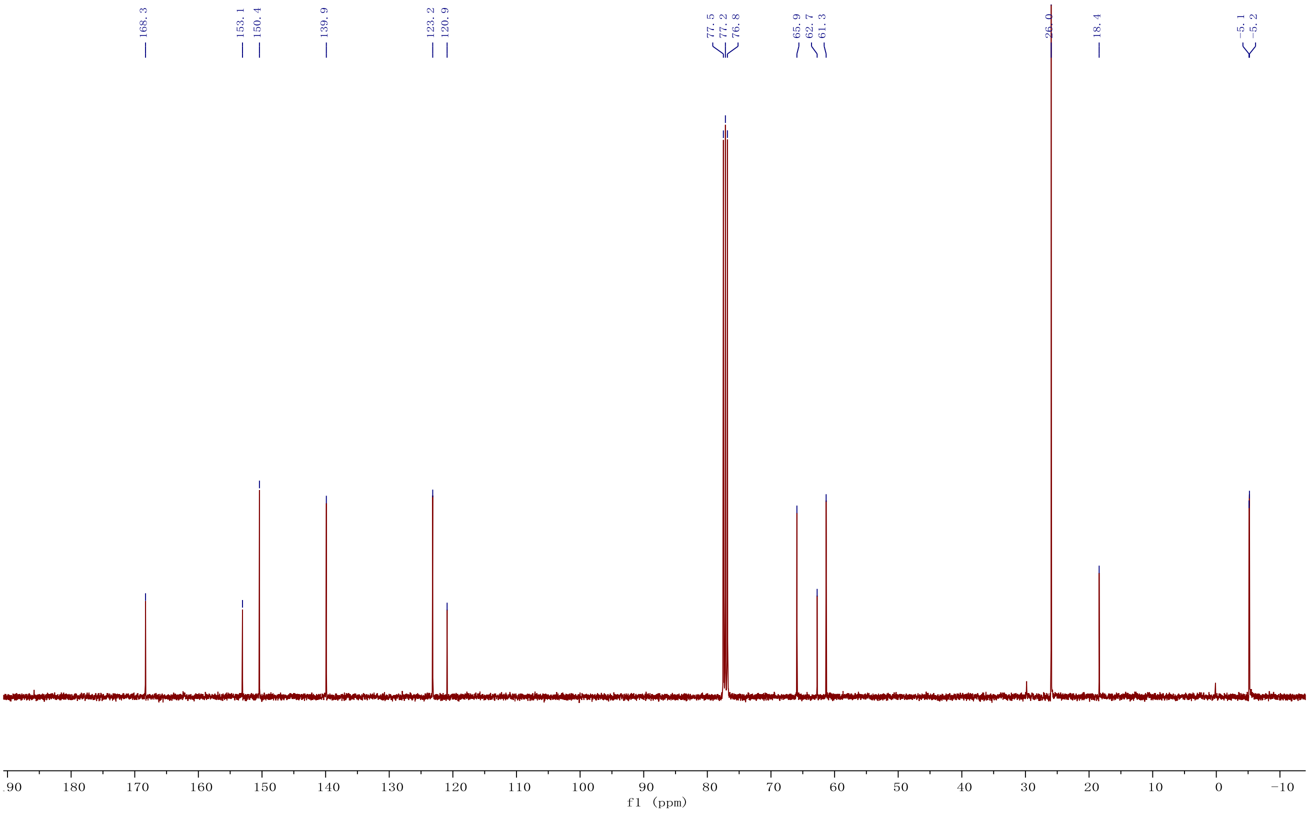

Supplementary Figure 480. ^{1}H NMR spectrum of compound **7c** (400 MHz, $CDCl_3$)

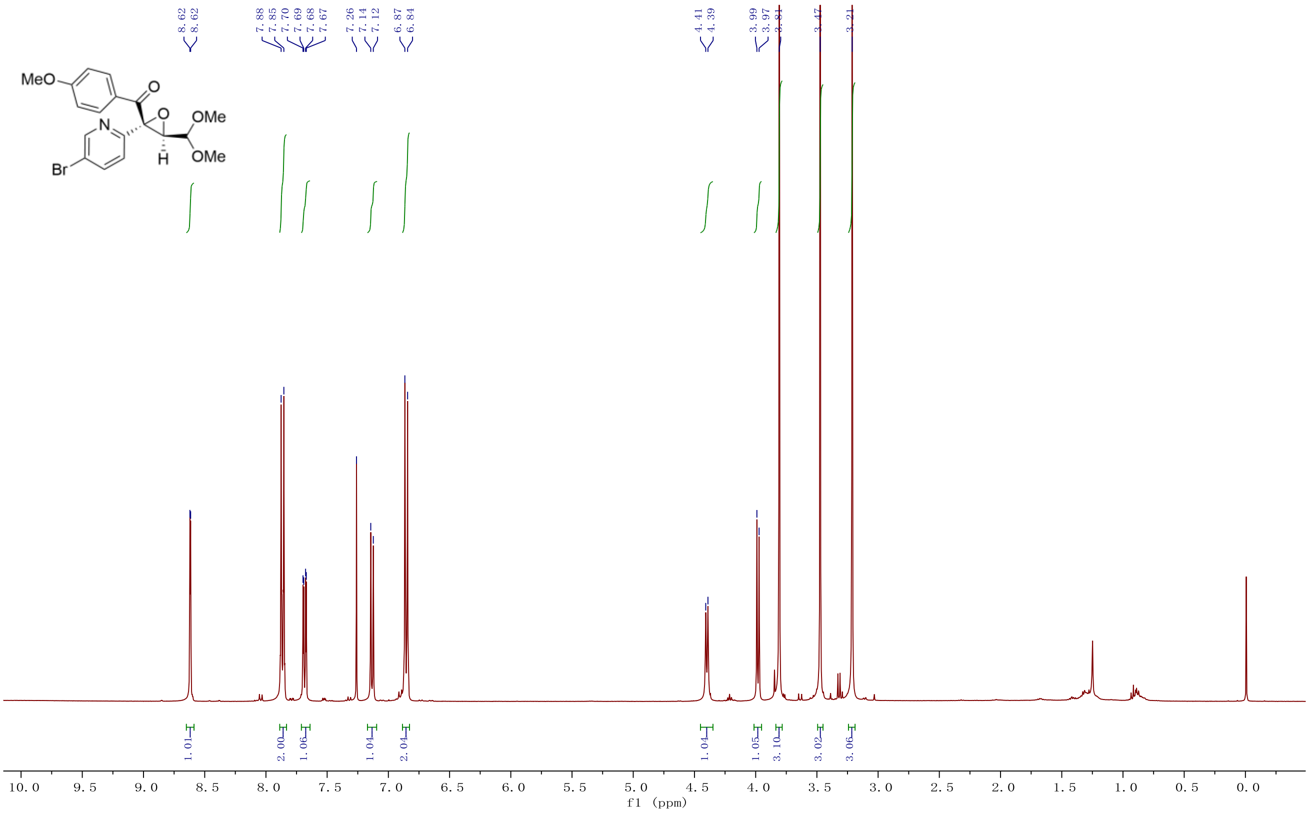

Supplementary Figure 481. ^{13}C NMR spectrum of compound **7c** (100 MHz, $CDCl_3$)

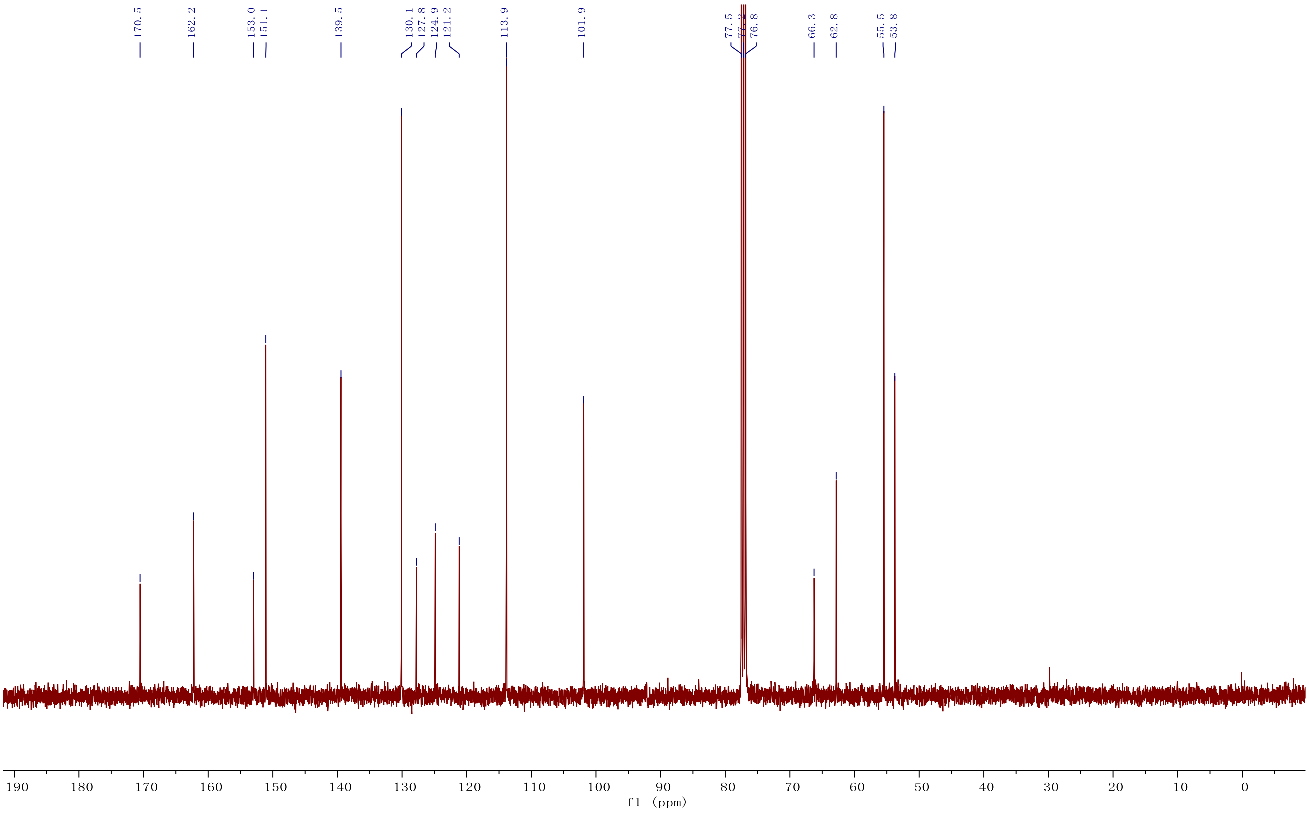

Supplementary Figure 482. ^{1}H NMR spectrum of compound **7d** (400 MHz, $CDCl_3$)

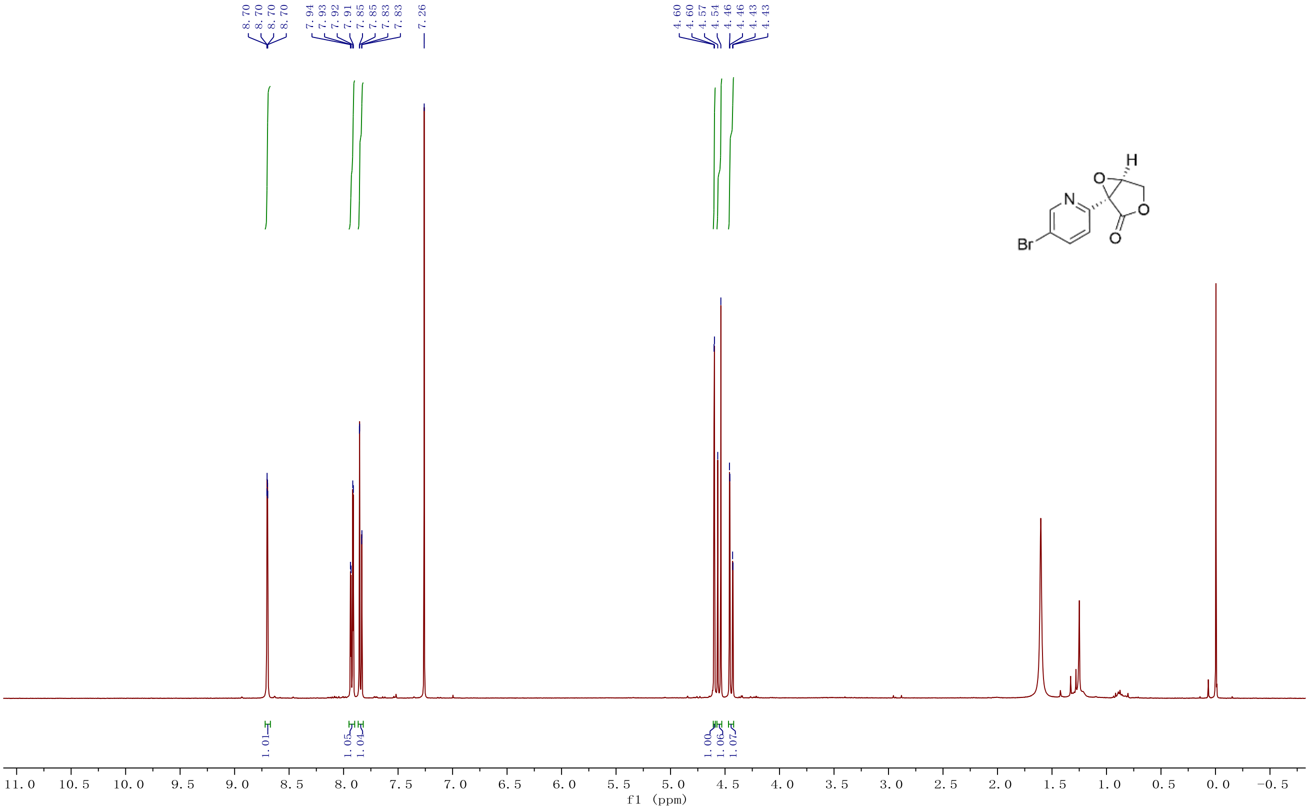

Supplementary Figure 483. ^{13}C NMR spectrum of compound **7d** (100 MHz, $CDCl_3$)

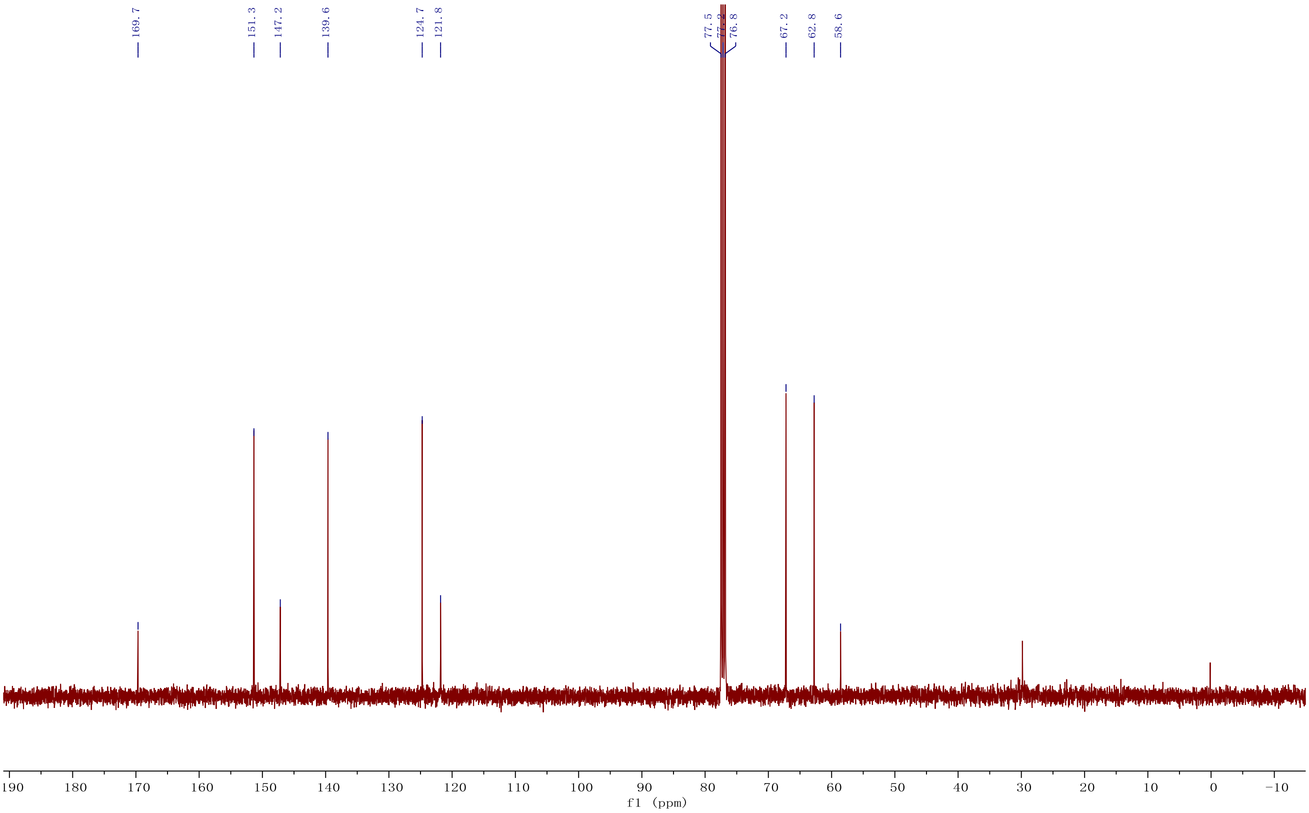

Supplementary Figure 484. ^{1}H NMR spectrum of compound **7e** (400 MHz, $CDCl_3$)

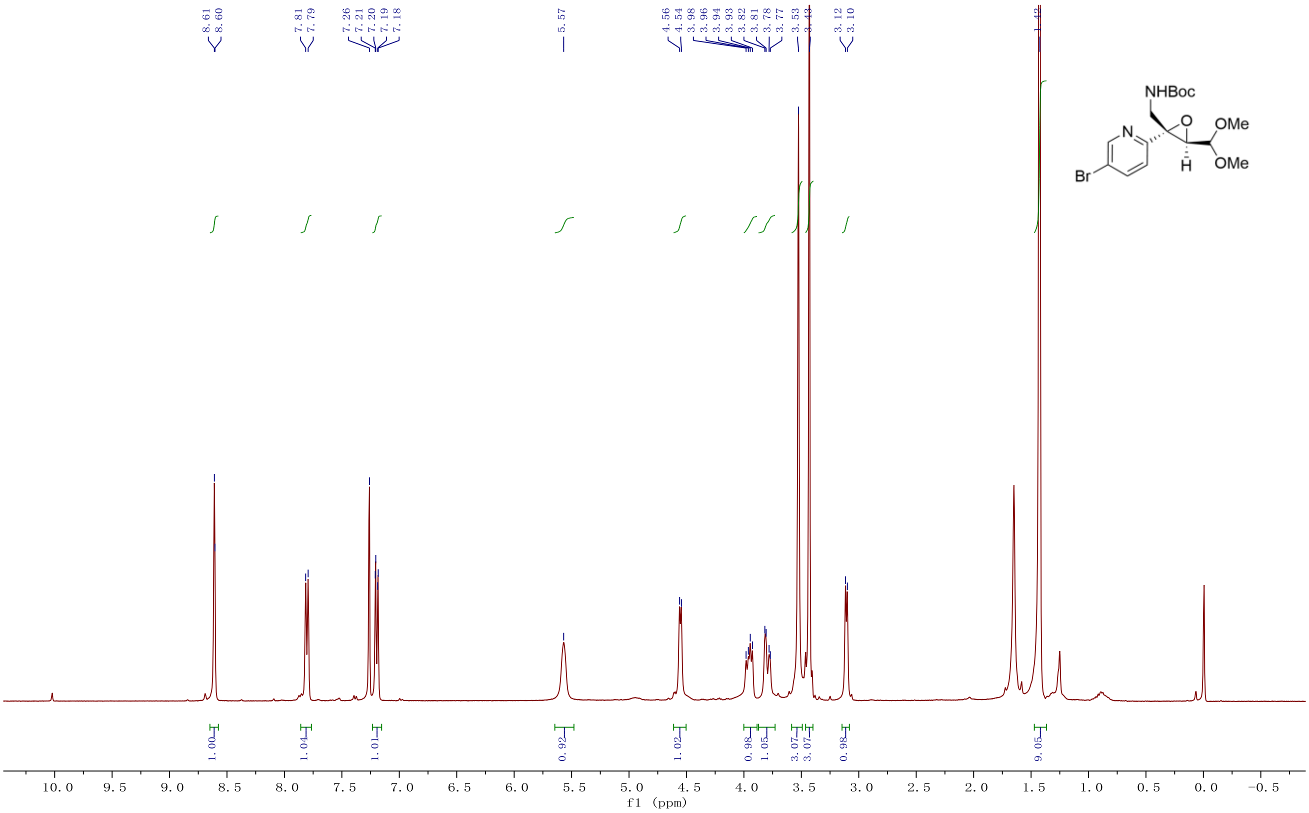

Supplementary Figure 485. ^{13}C NMR spectrum of compound **7e** (100 MHz, $CDCl_3$)

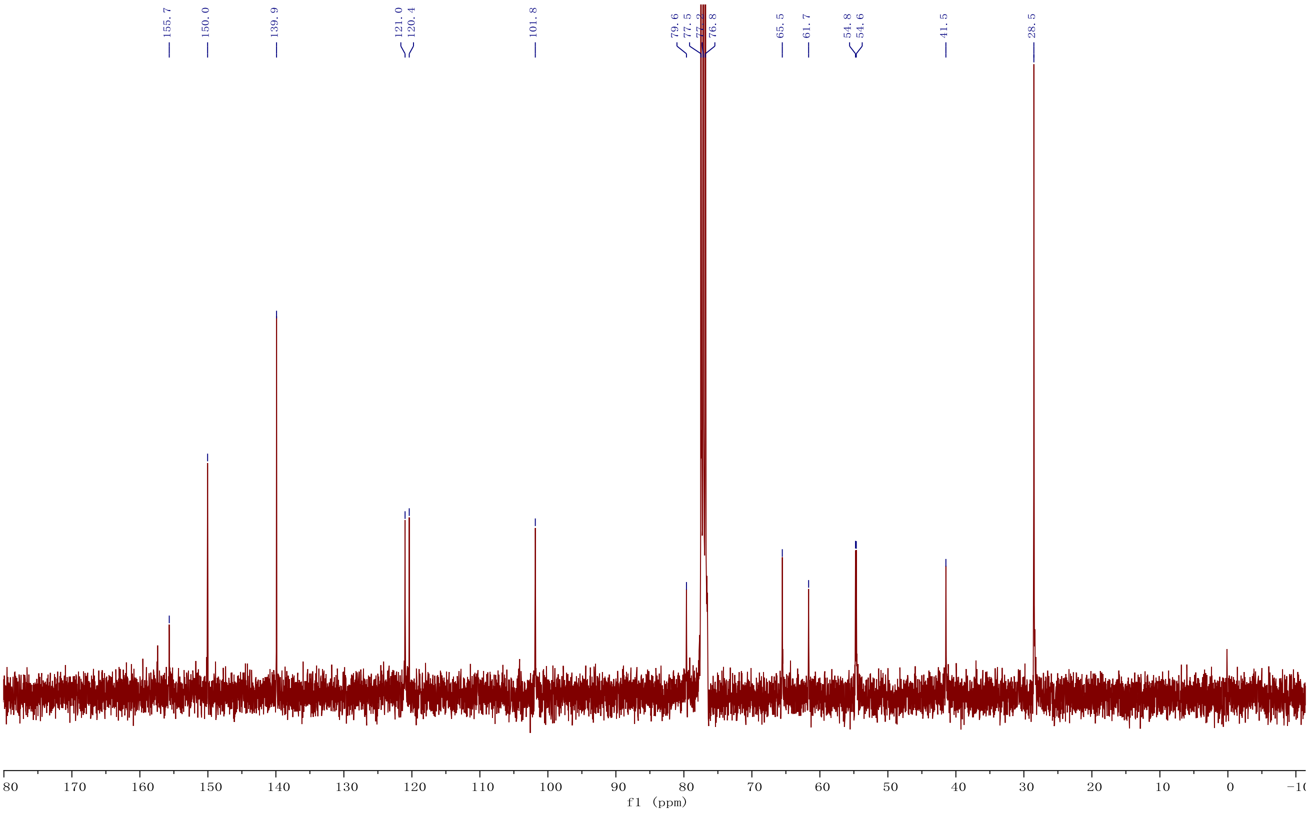

Supplementary Figure 486. ^{1}H NMR spectrum of compound **7f** (600 MHz, $CDCl_3$)

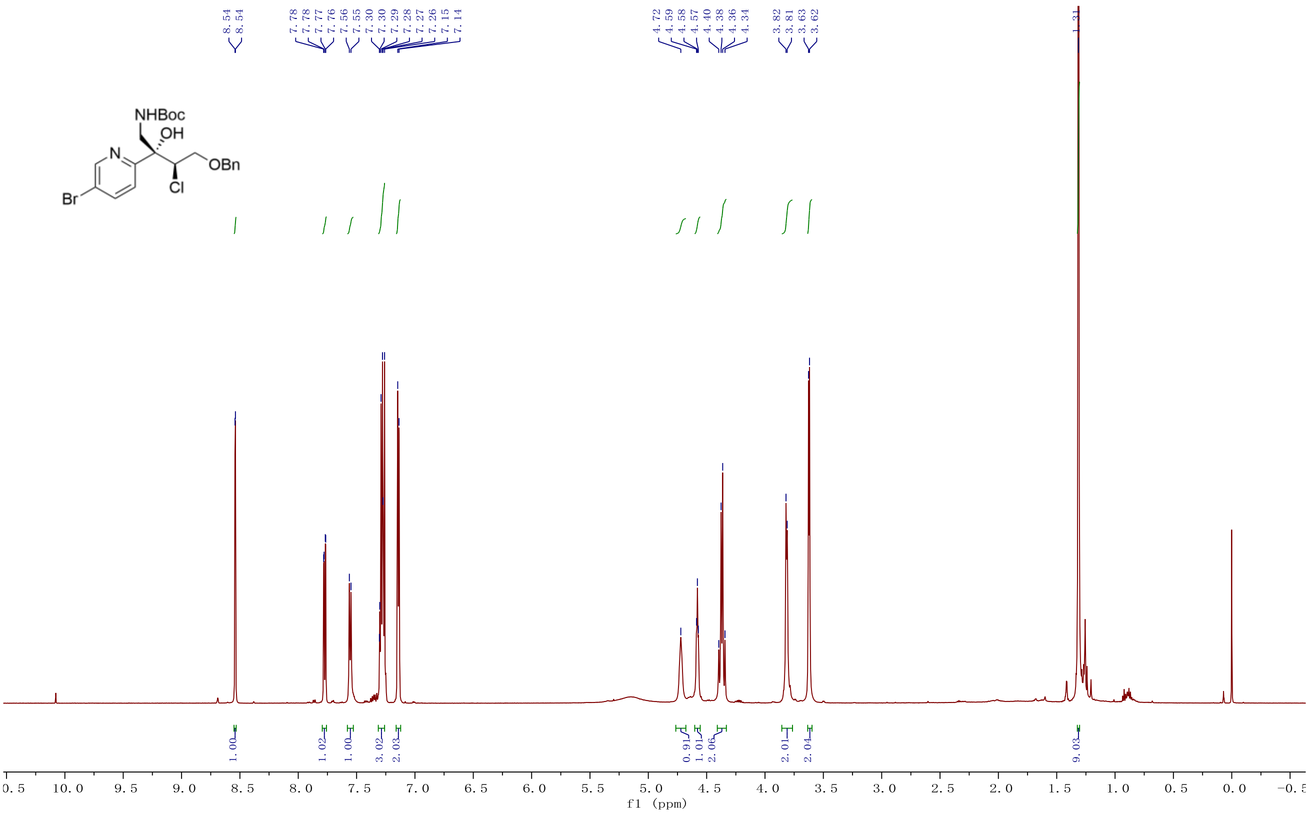

Supplementary Figure 487. ^{13}C NMR spectrum of compound **7f** (150 MHz, $CDCl_3$)

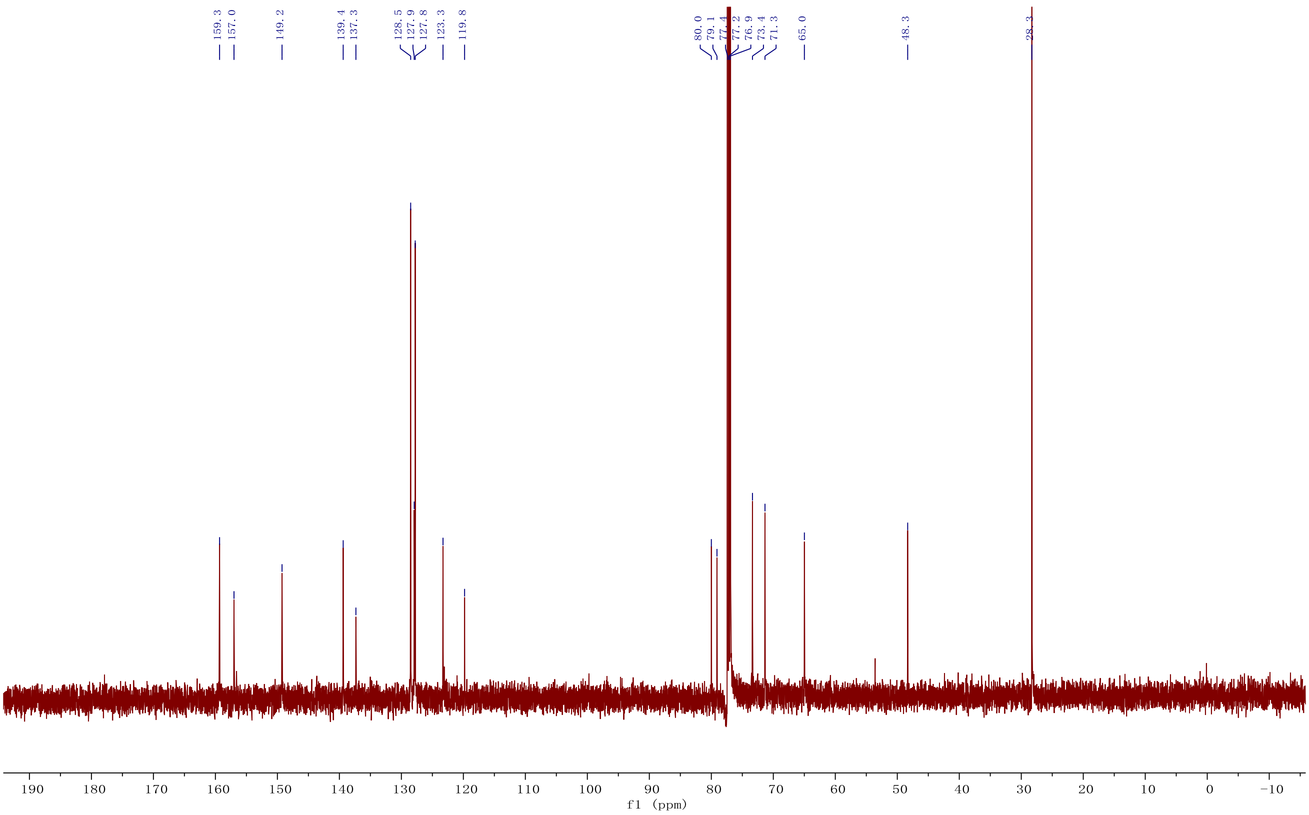

Supplementary Figure 488. ^{1}H NMR spectrum of compound **7g** (400 MHz, $CDCl_3$)

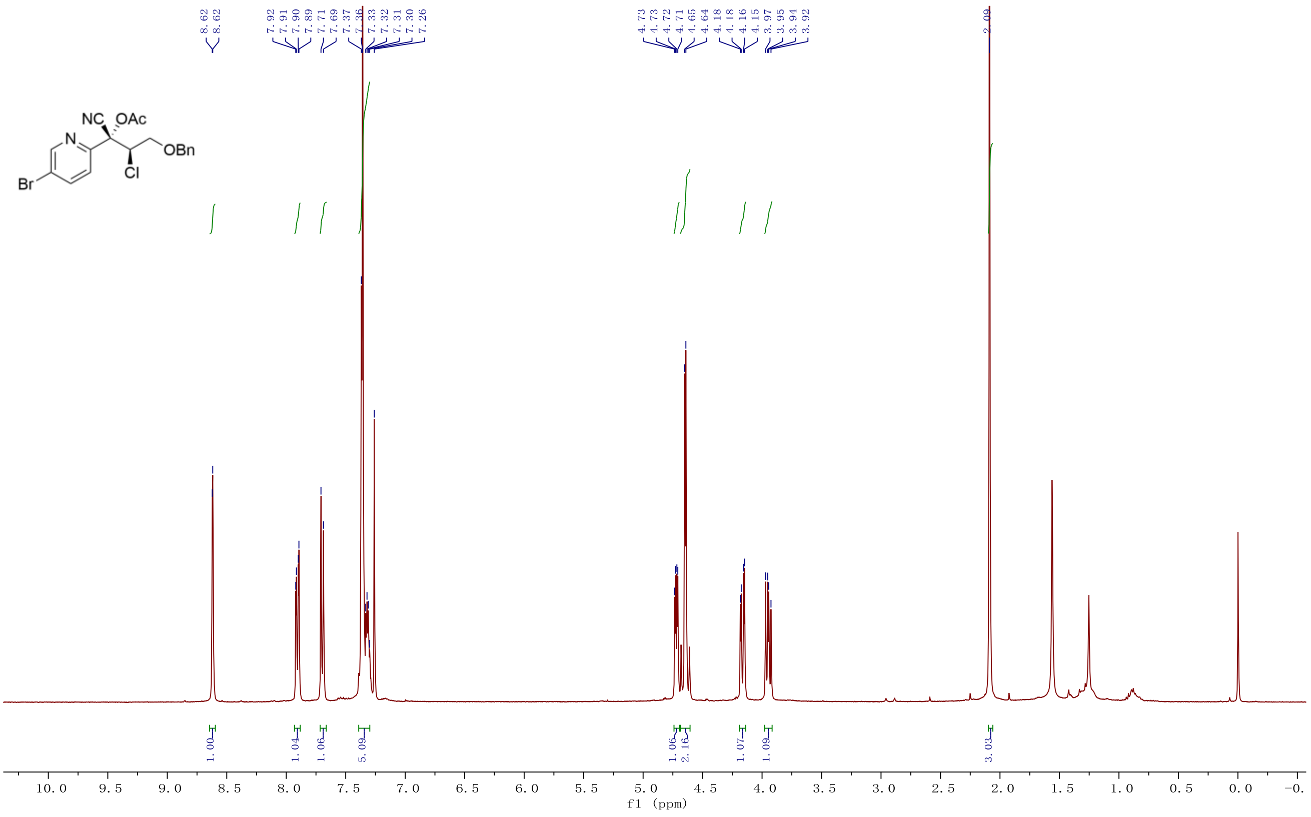

Supplementary Figure 489. ^{13}C NMR spectrum of compound **7g** (100 MHz, $CDCl_3$)

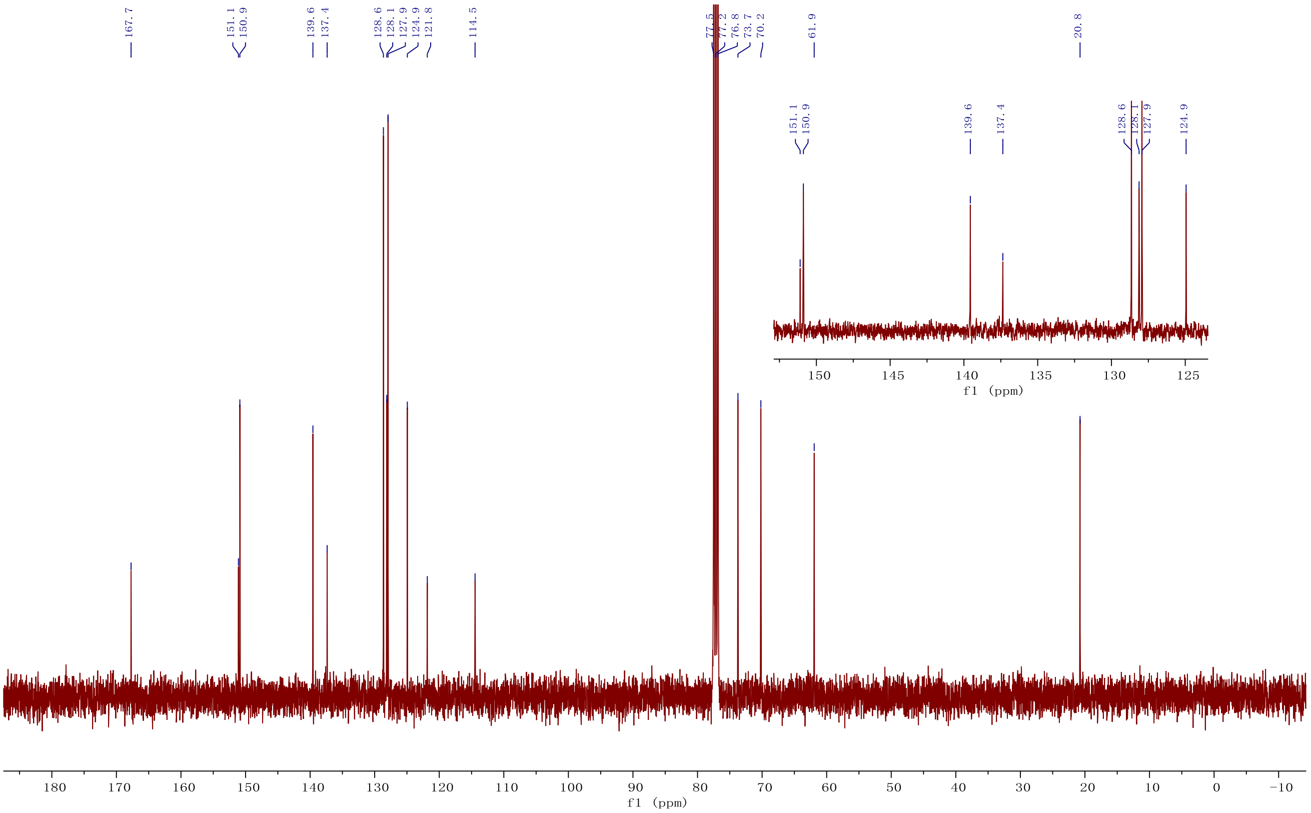

Supplementary Figure 490. ^{1}H NMR spectrum of compound **7h** (400 MHz, $CDCl_3$)

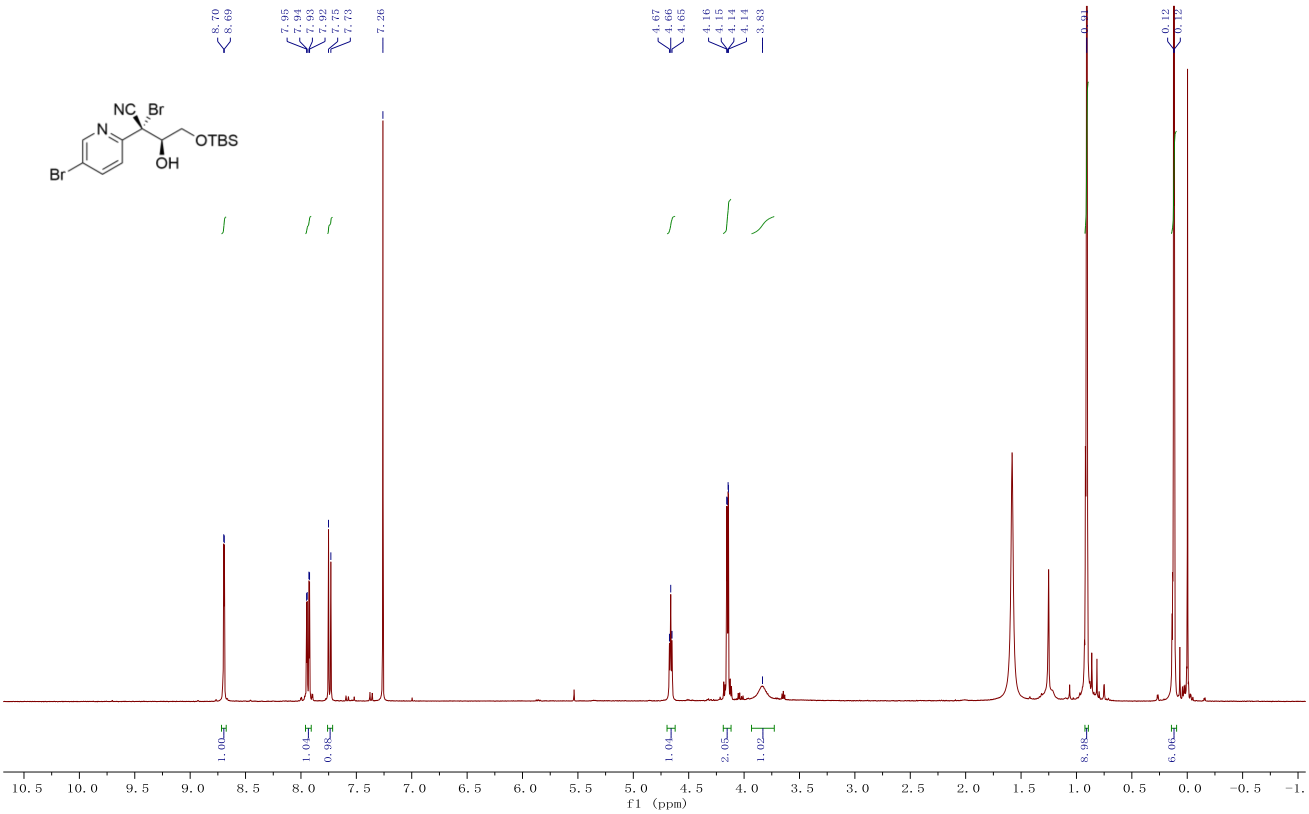

Supplementary Figure 491. ^{13}C NMR spectrum of compound **7h** (100 MHz, $CDCl_3$)

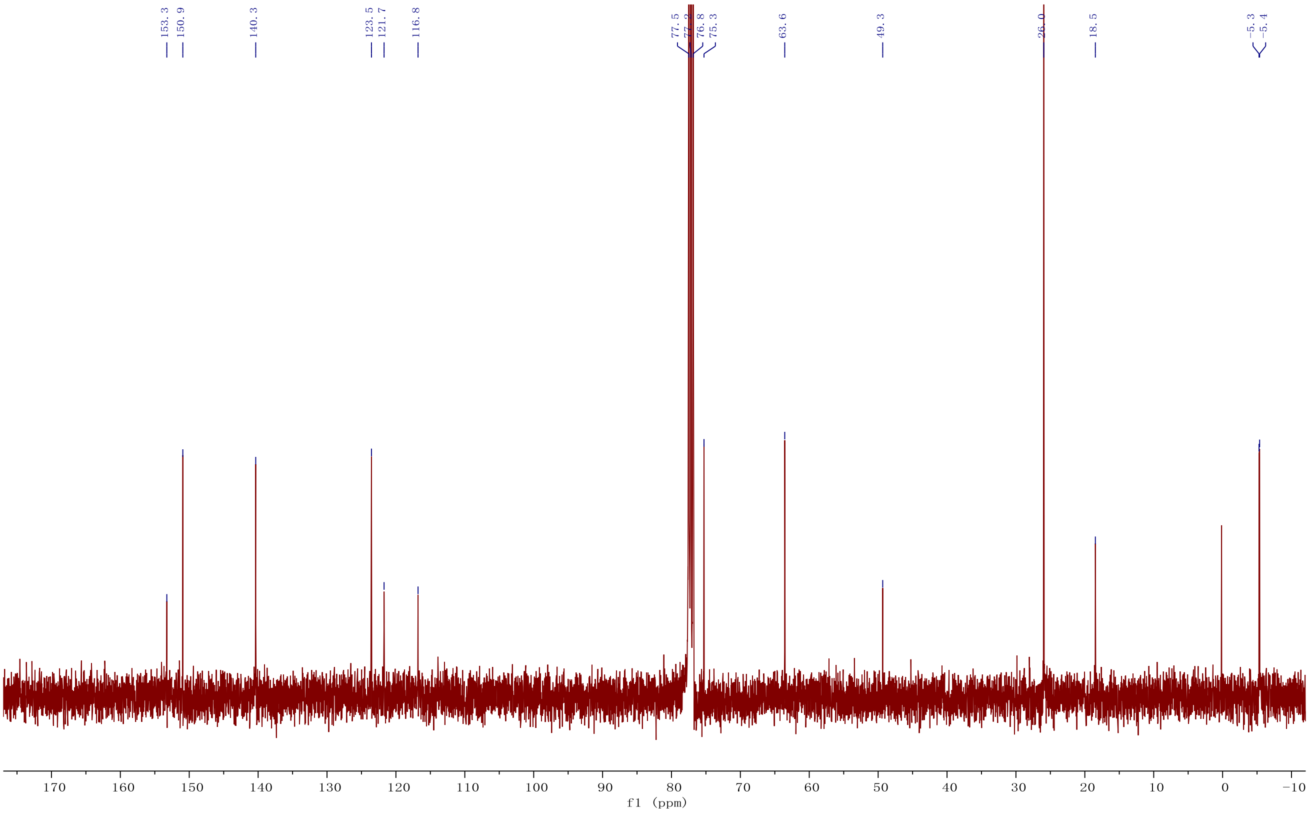

Supplementary Figure 492. ^{1}H NMR spectrum of compound **7i** (400 MHz, $CDCl_3$)

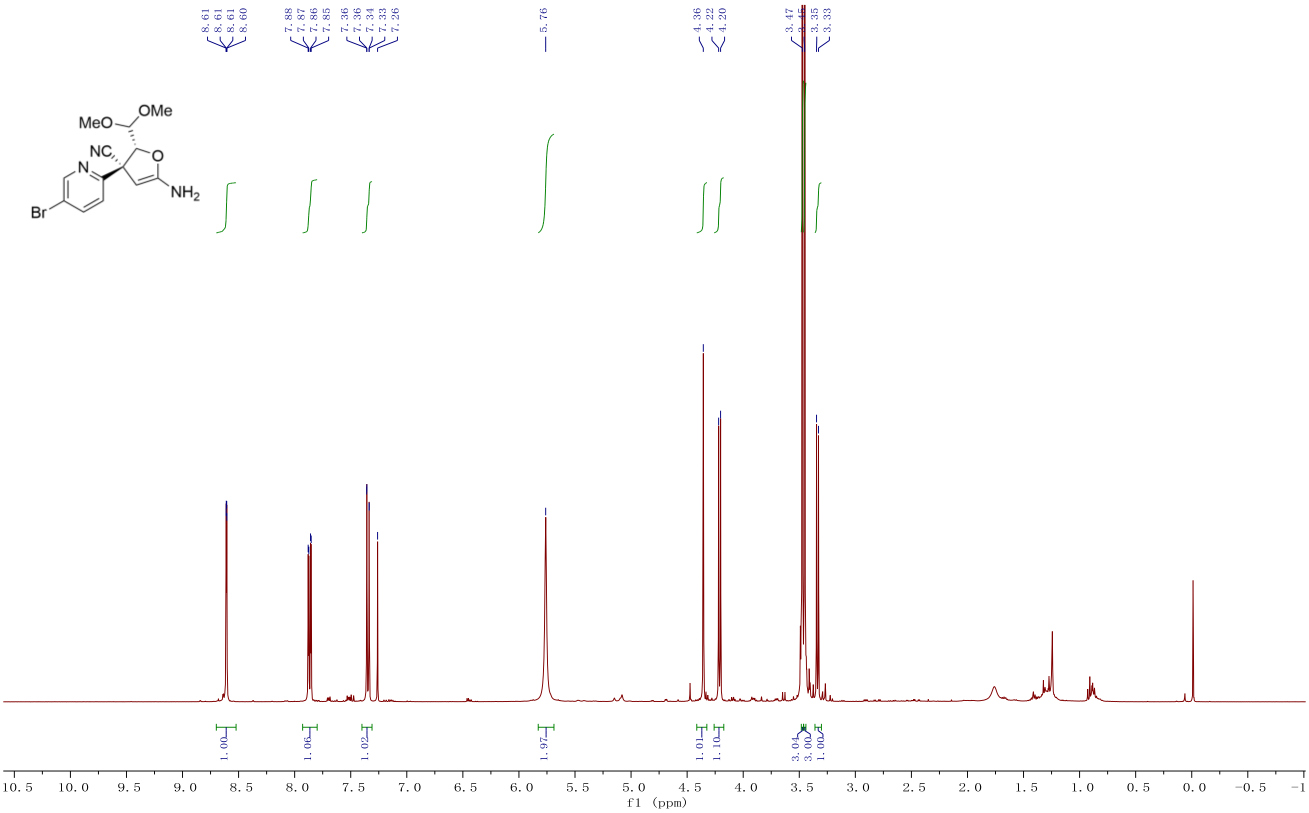

Supplementary Figure 493. ^{13}C NMR spectrum of compound **7i** (100 MHz, $CDCl_3$)

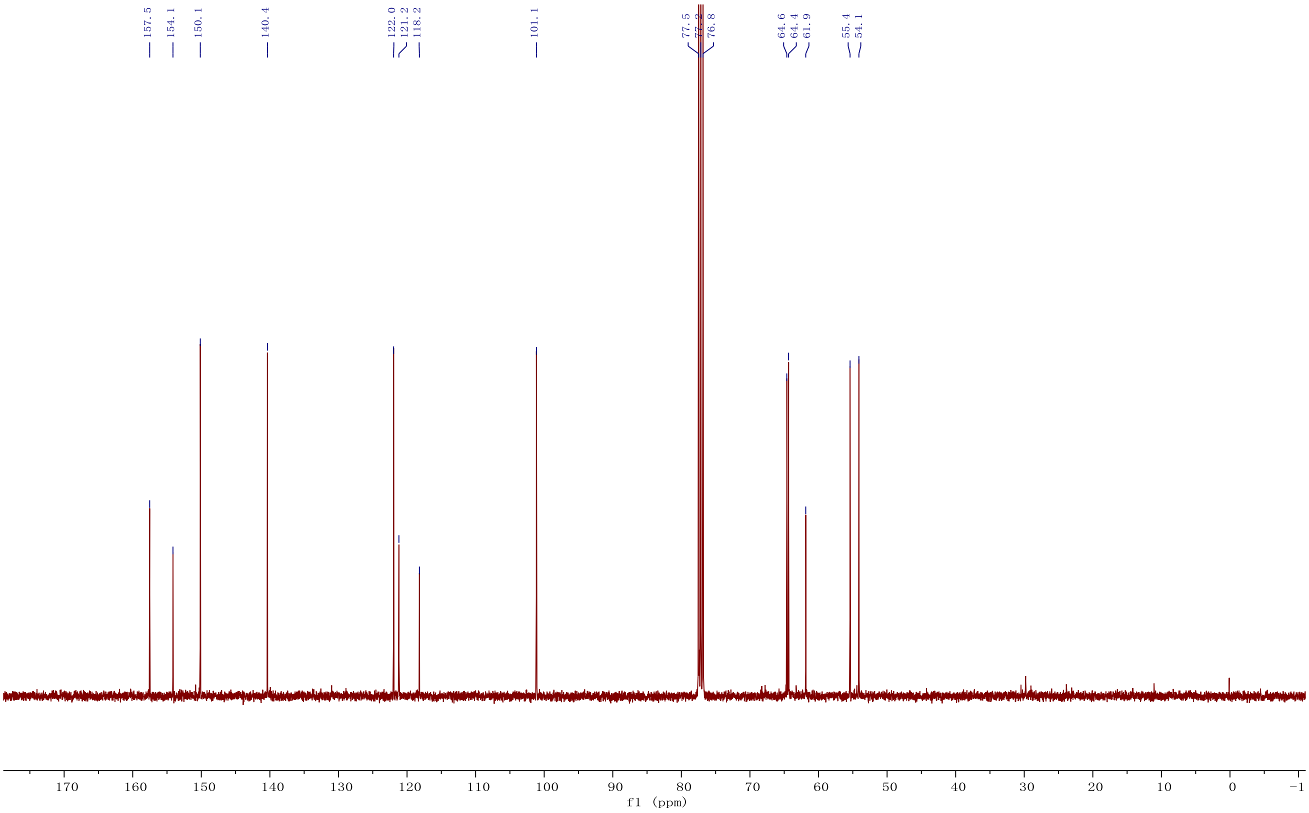

Supplementary Figure 494. 2D NMR spectrum of compound **7i** (600M, HSQC, $CDCl_3$)

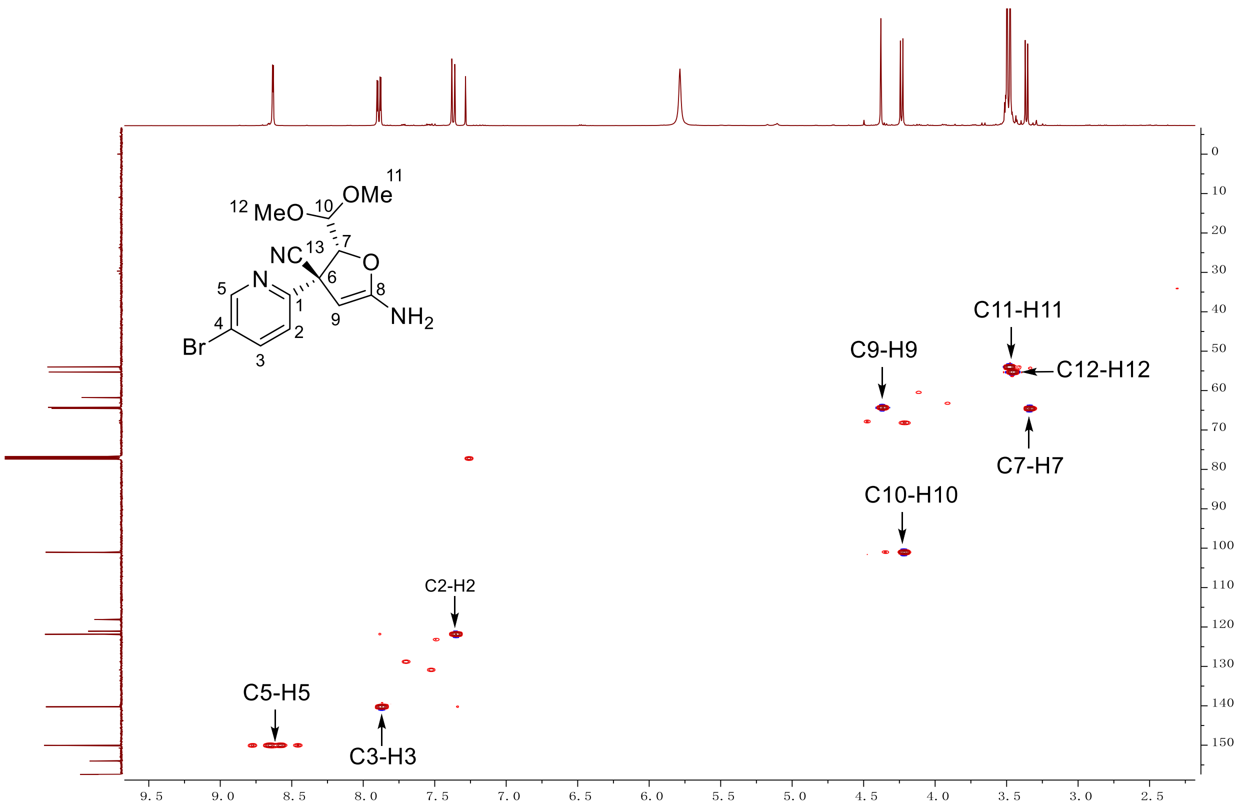

Supplementary Figure 495. 2D NMR spectrum of compound **7i** (600M, HMBC, $CDCl_3$)

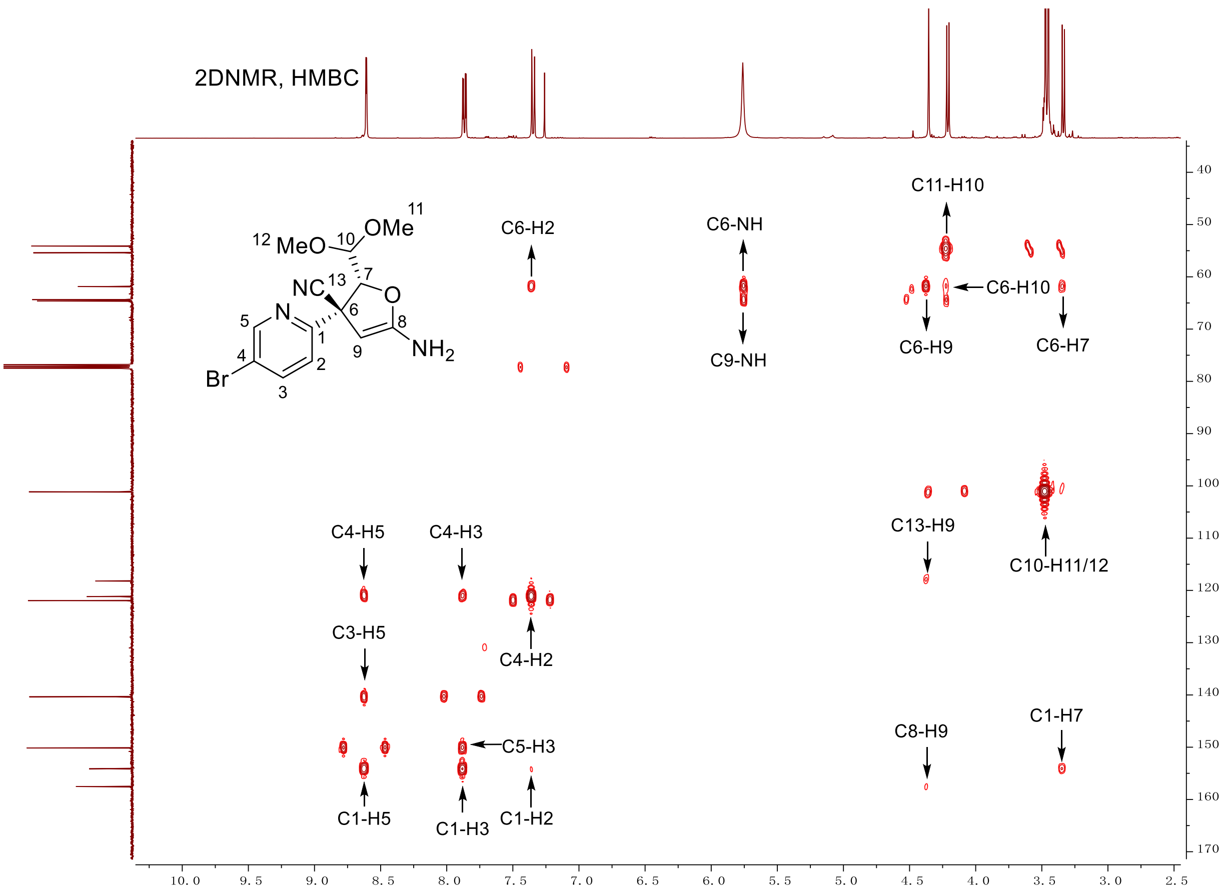

Supplementary Figure 496. ¹H NMR spectrum of compound **7j** (600 MHz, $CDCl_3$)

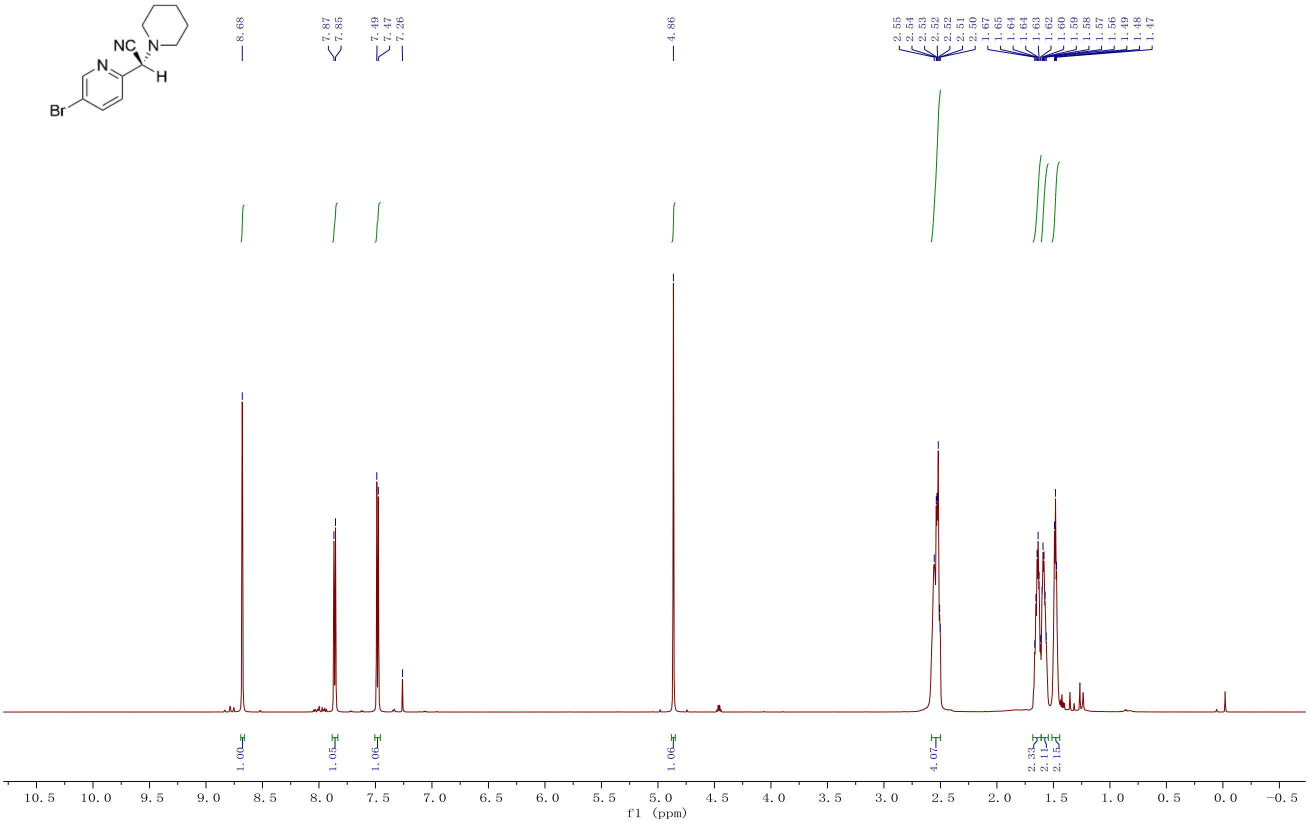

Supplementary Figure 497. ¹³C NMR spectrum of compound **7j** (150 MHz, $CDCl_3$)

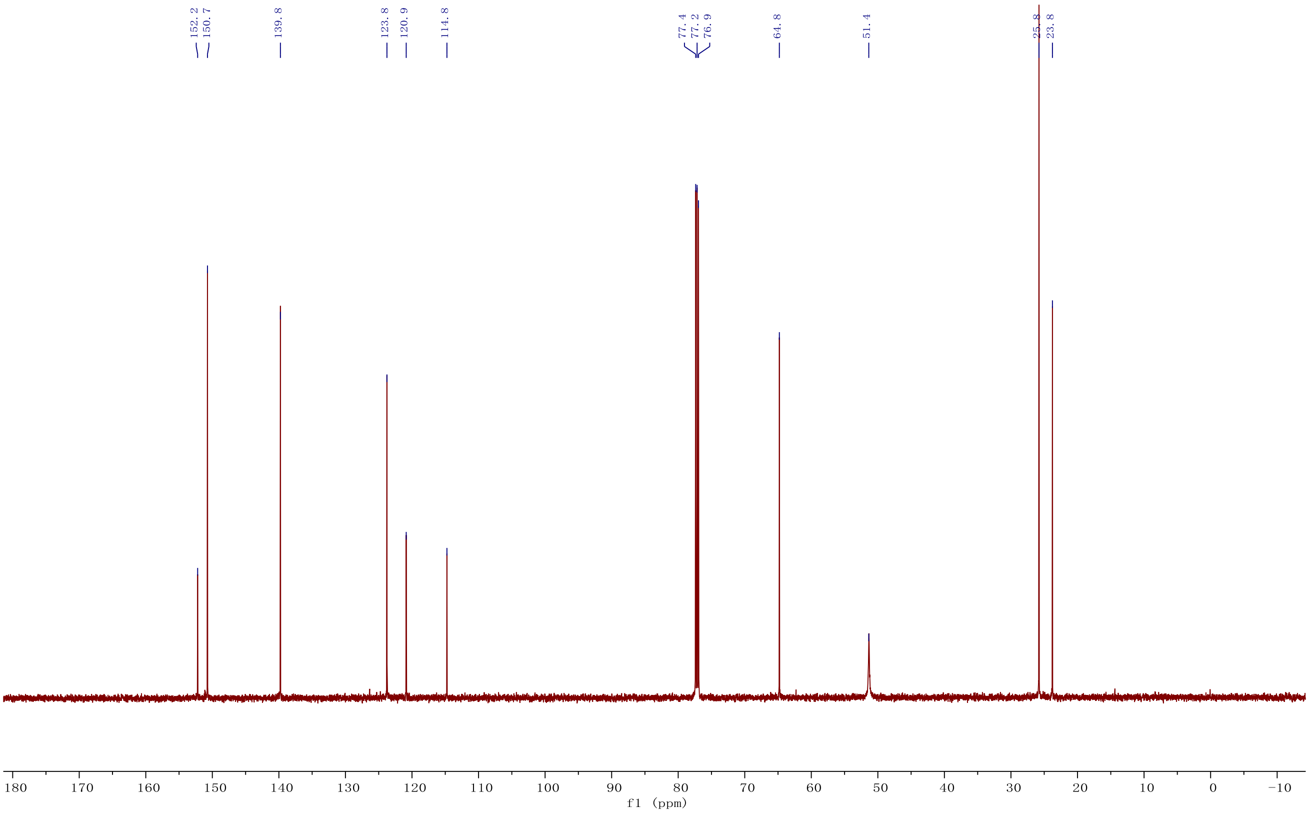

5. Supplementary References.

1. Sreekumar, V., Ilija, C. & Benjamin, L. *N*-Phosphinyl phosphoramide – a chiral Brønsted acid motif for the direct asymmetric *N*, *O*-acetalization of aldehydes. *Angew. Chem. Int. Ed.* **49**, 9749 –9752 (2010).

2. Zhang, H. *et al.* Facile synthesis of new functionalized 3,4-dihydro-2*H*-pyrroles using 2-isocyanoacetates *Tetrahedron Lett.* **61**, 151944 (2020).

3. Wang, K. *et al.* Enantioselective reaction between 2-(cyanomethyl)azaarenes and *N*-boc-amino sulfones. *Org. Lett.* **20**, 5260–5263 (2018).

4. Bernhart, C. A. *et al.* Synthesis and antiarrhythmic activity of new [(dialkylamino)alkyl]pyridylacetamides *J. Med. Chem.* **26**, 451–455 (1983).

5. Hassan, A. Y., Sarg, M. T. & Hussein, E. M. Design, Synthesis, and anticancer activity of novel benzothiazole analogues. *J. Heterocyclic Chem.* **56**, 1437–1457 (2019).

6. Ryabukhin, S. V. *et al.* Combinatorial knoevenagel reactions. *J. Comb. Chem.* **9**, 1073–1078 (2007).

7. Joshi, D. R. & Kim, I. Regioselective Synthesis of 1-cyano-3-arylindolizines: construction of pyrroles via DDQ ‐ mediated ring closure of cyclopropyl pyridines. *Adv. Synth. Catal.* **364**, 3016–3022 (2022).

8. Del Fiandra, C., Moccia, M. & Adamo, M. F. A. Enantioselective cyclopropanation of (*Z*)-3-substituted-2-(4-pyridyl)-acrylonitriles catalyzed by cinchona ammonium salts. *Org. Biomol. Chem.* **14**, 3105–3111 (2016).

9. Del Fiandra, C., Moccia, M., Cerulli, V. & Adamo, M. F. A. Catalytic asymmetric conjugate addition of isocyanoacetate to (*Z*)-3-substituted-2-(4-pyridyl)-acrylonitrile, a reactive class of michael acceptor. *Chem. Commun.* **52**, 1697–1700 (2016).

10. Sakamoto, M., Nagano, M., Suzuki, Y., Satoh, K. & Tamura, O. Inter- and intramolecular Diels-Alder reactions using a highly reactive 1-aza-1,3-butadiene, ethyl (*E*)-3-(1,3-benzothiazol-2-yl)-3-cyanopropenoate. *Tetrahedron* **52**, 733–742 (1996).

11. Kutasevich, A. V., Niktarov, A. S., Uvarova, E. S., Karnoukhova, V. A. & Mityanov, V. S. A novel approach to bis(1,3-azol-2-yl)acetonitriles and bis(1,3-azol-2-yl)methanes via the [3+2]-dipolar cycloaddition of imidazole *N*-oxides and 2-heteroaryl-3,3-dimethylacrylonitriles. *Org. Biomol. Chem.* **19**, 8988–8998 (2021).

12. Chao, X. *et al.* Catalytic Enantioselective synthesis of α-Chiral azaheteroaryl ethylamines by asymmetric protonation. *Angew. Chem. Int. Ed.* **57**, 11374–11377 (2018).

13. James, A. L., Noah, M. B. & James, H. F. Site-specific alkene hydromethylation via protonolysis of titanacyclobutanes. *Angew. Chem. Int. Ed.* **60**, 14360–14364 (2021).

14. Adhikari, A. S. & Majumdar, N. Unconventional reactivity of a grubbs catalyst: hydroalkylation overriding metathesis. *Org. Lett.* **25**, 8611–8616 (2023).

15. Xi, L. L., Wang, M. Y., Liang, Y., Zhao, Y. & Shi, Z. Z. Tunably strained metallacycles enable modular differentiation of aza-arene C–H bonds. *Nat. Commun.* **14**, 3986 (2023).

16. Liu, Z.-C., Yue, W.-J. & Yin, L. Copper(I)-catalyzed asymmetric synthesis of unnatural α-amino acid derivatives and related peptides containing γ-(aza)Aryls. *J. Org. Chem.* **87**, 399–405 (2022).

17. Chu, W.-D. *et al.* Enantioselective [3+2] cycloaddition of vinylcyclopropanes with alkenyl *N*-heteroarenes enabled by palladium catalysis. *Org. Lett.* **24**, 3965–3969 (2022).

18. McLean, L. A. *et al.* Asymmetric synthesis of heterocyclic chloroamines and aziridines by enantioselective protonation of catalytically generated enamines. *Chem. Eur. J.* **28**, e202200060 (2022).

19. Henry B. J., Harris, G. R., Smith, M. A. & Gaunt, M. J. Modular photocatalytic synthesis of α-trialkyl-α-tertiary amines. *J. Am. Chem. Soc.* **143**, 15946–15959(2021).

20. Mukherjee, P. *et al.* Novel 2,4-disubstituted pyrimidines as potent, selective, and cell-permeable inhibitors of neuronal nitric oxide synthase. *J. Med. Chem.* **58**, 1067−1088 (2015).

21. Minami, T., Isonaka, T., Okada, Y. & Ichikawa, J. Copper (I) salt-mediated arylation of phosphinyl-stabilized carbanions and synthetic application to heterocyclic compounds. *J. Org. Chem.* **58**, 7009−7015 (1993).

22. Wilson, K. L., Murray, J., Jamieson, C. & Watson, A. J. B. Cyrene as a bio-based solvent for the Suzuki–Miyaura cross-coupling. *Synlett* **29**, 650–654(2018).

23. Niu, Z., Li, L., Liu, X. & Liang, Y. Transition-metal-free alkylation/arylation of benzoxazole via Tf_2O-activated-amide. *Adv. Synth. Catal.* **361**, 5217–5222 (2019).

24. Gerelle, M., Dalencon, A. J. & Willis, M. C. Palladium-catalyzed direct functionalization of benzoxazoles with alkenyl iodides. *Tetrahedron Lett.* **53**, 1954–1957 (2012).

25. Hu, Z.-Y. *et al.* I_2-mediated intramolecular C–H amidation for the synthesis of *N*-substituted benzimidazoles. *J. Org. Chem.* **82**, 3152–3158 (2017).

26. Hepburn, H. B. & Melchiorre, P. Brønsted acid-catalysed conjugate addition of photochemically generated α-amino radicals to alkenylpyridines. *Chem. Comm.* **52**, 3520–3523 (2016).

27. Lieber, S. *et al.* (*Z*)-2-(2-Bromophenyl)-3-{[4-(1-methyl-piperazine)amino]phenyl}acrylonitrile (DG172): an orally bioavailable PPAR *β*/*δ*-selective ligand with inverse agonistic properties. *J. Med. Chem.* **55**, 2858–2868 (2012).

28. Smith, C. R. & Rajanbabu, T. V. Low pressure vinylation of aryl and vinyl halides via Heck–Mizoroki reactions using ethylene. *Tetrahedron* **66**, 1102–1110 (2010).

29. Burés, J. A simple graphical method to determine the order in catalyst. *Angew. Chem. Int. Ed*. **55**, 2028–2031 (2016).

30. Gaussian 16, Revision A.03 & Frisch, M. J. *et al.* Wallingford CT, (2016).

31. Becke, A. D. Density‐functional thermochemistry. III. The role of exact exchange. *J. Chem. Phys.* 98, 5648–5652 (1993).

32. Grimme, S. Semiempirical GGA-type density functional constructed with a long-range dispersion correction. *J. Comp. Chem.* **27**, 1787–1799 (2006).

33. Marenich, A. V., Cramer, C. J. & Truhlar, D. G. Universal solvation model based on solute electron density and on a continuum model of the solvent defined by the bulk dielectric constant and atomic surface tensions. *J. Phys. Chem. B.* **113**, 6378–6396 (2009).

34. CYLview, 1.0b, Legault, C. Y., Université de Sherbrooke, (http://www.cylview.org) (2009).

35. Lu, T. & Chen, F.-W. Multiwfn: A multifunctional wavefunction analyzer. *J. Comput. Chem*. **33**, 580–592 (2012).

36. Dolomanov, O. V., Bourhis, L. J., Gildea, R. J., Howard, J. A. K. & Puschmann, H. (IUCr) OLEX2: a complete structure solution, refinement and analysis program. *J. Appl. Cryst*. **42**, 339–341 (2009).

37. Sheldrick, G. M. (IUCr) SHELXT-Integrated space-group and crystal-structure determination. *Acta Cryst.* **A71**, 3–8 (2015).

38. Sheldrick, G. M. Crystal structure refinement with SHELXL. *Acta Cryst*. **C71**, 3–8 (2015).
